# Functional and Proteomic Profiles of CD3(+) Plasma‐Derived Small Extracellular Vesicles Differentiate Cancer Patients From Healthy Donors

**DOI:** 10.1002/eji.70273

**Published:** 2026-09-07

**Authors:** Piotr Widlak, Jie Han, Alicja Gluszko, Justyna Mika, Aneta Zebrowska, Sujan Kumar Mondal, Marta Gawin, Yana G. Najjar, Joanna Polańska, Monika Pietrowska, Theresa L. Whiteside

**Affiliations:** ^1^ 2nd Department of Radiology Medical University of Gdańsk Gdańsk Poland; ^2^ Department of Pathology University of Pittsburgh School of Medicine Pittsburgh Pennsylvania USA; ^3^ Department of Biochemistry Medical University of Warsaw Warsaw Poland; ^4^ Department of Data Science and Engineering Silesian University of Technology Gliwice Poland; ^5^ Center For Translational Research and Molecular Biology of Cancer Maria Sklodowska‐Curie National Research Institute of Oncology Gliwice Poland; ^6^ Institute of Genetics and Animal Biotechnology of the Polish Academy of Sciences Magdalenka Poland; ^7^ UPMC Hillman Cancer Center University of Pittsburgh Pittsburgh Pennsylvania USA

**Keywords:** cancer plasma, CD3(+) sEV functions, high‐resolution mass spectrometry, melanoma cell‐derived sEVs (MTEX), rewiring of CD3(+) sEV, small extracellular vesicles (sEV)

## Abstract

Small extracellular vesicles (sEV) released by T cells play a key role in immune regulation. Immune capture with anti‐CD3 antibodies was used to isolate and study T cell‐derived CD3(+)sEV from the plasma of patients with melanoma (MPs) or healthy donors (HDs). Functional responses induced in recipient target cells by CD3(+)sEV of MPs differed from responses induced by CD3(+)sEV of HDs. Approximating functions mediated by melanoma cell‐derived sEV (MTEX), CD3(+)sEV of MPs reduced metabolic activity and proliferation of T cells while promoting activity in Mel526 cell targets. Proteomics profiling confirmed functional differences between CD3(+)sEV of MPs and HDs. Of 294 sEV‐specific proteins identified in CD3(+)sEV, 226 were detected in the parent T cell proteome, confirming that the CD3(+)sEV proteome mimics that of the parent T lymphocytes. Among them were 66 differentially expressed proteins (DEPs) that differentiated vesicles from MPs and HDs. These DEPs were associated with processes linked to cancer‐related functions. DEPs upregulated in CD3(+)sEV of MPs were associated with RHO‐GTPase, cytokine, and MAPK signaling pathways. Thus, T cells of MPs were reprogrammed by MTEX to produce CD3(+)sEV that functionally resembled MTEX, partly recapitulated features of the tumor proteome, and differed from CD3(+)sEV of HDs. In cancer, the TEX‐rewired T cells produce CD3(+)sEV that potentially could serve as a liquid biopsy of patients’ T cells.

## Graphical abstract

The graphical abstract illustrates MTEX‐driven reprogramming of CD3(+) T cells in patients with melanoma.

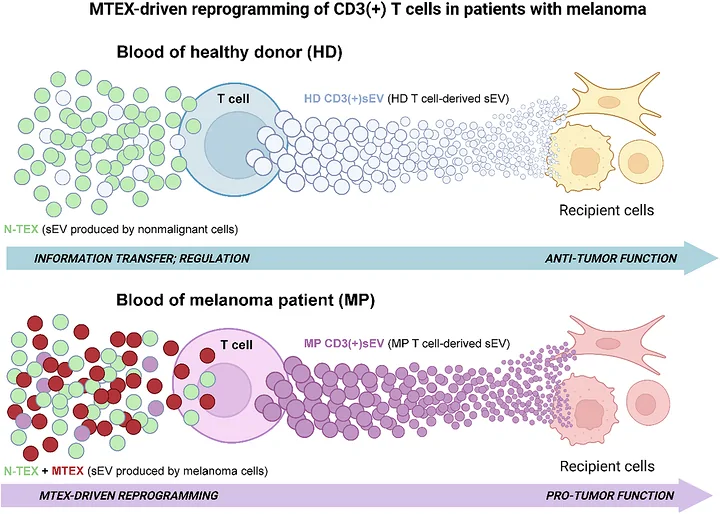

## 1 Introduction

Small extracellular vesicles (sEV), a subset of EVs sized at 30–150 nm, are derived from multivesicular bodies (MVBs) of parental cells [1]. Their molecular content reflects that of the parent cells [2, 3, 4]. sEV are produced and released by all cell types and are present in all body fluids [5, 6, 7]. The plasma of cancer patients contains a heterogeneous mix of sEV derived from tumor cells (tumor‐derived extracellular vesicles, TEX) and various tissue‐resident and circulating nonmalignant cells, including immune cells [8, 9, 10, 11, 12]. Because of this heterogeneity, it is impossible to unravel the intercellular crosstalk mediated by sEV subsets produced by malignant and nonmalignant cells without isolating and examining their molecular cargo. We have previously described the immune capture strategy for the separation of TEX from other sEV in cancer patients’ plasma [13, 14]. This strategy can be successful only when antibodies (Abs) specific for a highly overexpressed tumor‐associated antigen (TAA) are available, such as anti‐CSPG4 epitope‐specific Abs we utilized for isolation from patients’ plasma of melanoma cell‐derived sEV, called MTEX [13, 14, 15]. As an alternative approach, immune selection with Abs specific for the CD3 antigen expressed only on TCR+ cells can be used to yield fractions of CD3(+)sEV on beads and of unbound CD3(−)sEV that are produced by non‐T cells, including tumor cells [12, 16, 17, 18]. Thus, in the plasma of cancer patients, the CD3(−)sEV fraction comprises TEX and non‐TEX at various patient‐specific ratios [17]. Separation from plasma of CD3(+)sEV that are products of T lymphocytes allows for assessments of their molecular and functional characteristics in addition to enabling studies of the TEX‐enriched CD3(−)sEV fraction.

Emerging data suggest that T lymphocytes in melanoma (and other cancers) are reprogrammed by the tumor and acquire functional characteristics that differentiate them from T cells in healthy individuals [19]. The data indicate that TEX drive tumor‐mediated reprogramming of all cells in the tumor microenvironment (TME). It appears that TEX alter the functions of both immune and nonimmune cells and that TEX‐driven reprogramming has profound consequences for tumor immune escape [12, 20, 21, 22]. Specifically, we have reported on the role of glioblastoma cell‐derived TEX (GBex) in mediating reprogramming of immune cell functions in the TME and thus promoting tumor progression [23]. The mechanism of GBex‐mediated reprogramming involved simultaneous delivery of numerous suppressive signals to all types of immune cells, leading to activation of several different molecular pathways. Macrophages that avidly internalized GBex were highly susceptible in vitro and in vivo to GBex‐mediated reprogramming, which involved activation of the NF‐κB pathway and M1 to M2 polarization [24]. We also reported that TEX produced by head and neck squamous cell carcinoma (HNSCC) cells were potent inducers of angiogenesis in vitro and in vivo through functional reprogramming and phenotypic modulation of human endothelial cells [25].

Interestingly, T lymphocytes present in the TME or in the circulation of cancer patients were shown to produce CD3(+)sEV, which, like TEX, also had an immunosuppressive profile and mediated immune suppression [17]. As T cells in the tumor‐bearing hosts frequently have compromised immune competence [26, 27, 28], the examination of T cell‐derived CD3(+)sEV, which mimic parent T cells, could inform about the phenotypic and functional status of patients’ T cells. The rationale for examining CD3(+)sEV in the plasma of melanoma patients is thus justified by the available preliminary data and by the emerging view that CD3(+)sEV, reciprocating the protein profile of parent T cells, could potentially serve as a “liquid T cell biopsy”, as previously suggested [18].

Applying the immune capture strategy to the separation of CD3(+)sEV from the plasma of melanoma patients (MPs), we provide here a proof of concept that these sEV could serve as a liquid biopsy of the tumor‐reprogrammed T lymphocytes. We provide evidence for a differential proteome content and distinct functions of CD3(+)sEV in the plasma of MPs and healthy donors (HDs). Our data suggest that CD3(+)sEV may be potentially valuable for molecular, functional, and metabolic monitoring of T cells in patients with melanoma, and likely in patients with other cancers.

## 2 Materials and Methods

### 2.1 Study Material

Blood samples were obtained from 10 consenting healthy donors (HDs) and 10 melanoma patients (MPs) treated at the UPMC Hillman Cancer Center Melanoma Program Outpatient Clinic by Yana Najjar, MD. The disease status and clinicopathological information for all patients are listed in Table S1. Blood samples were collected for research under the University of Pittsburgh IRB approvals #970186 and #04‐001. The blood samples were processed to separate the plasma, which was divided into aliquots and stored at −80°C until thawed, and was then used for the sEV isolation.

### 2.2 Isolation of Circulating T Cells

Peripheral blood mononuclear cells (PBMC) were isolated from the blood using Ficoll gradient centrifugation. Next, primary human CD3^+^ T cells were isolated using the Human Pan T cell isolation kit (Miltenyi Biotec #130‐096‐535) and LS separation columns (Miltenyi Biotec #130‐042‐401). T cells were cultured in RPMI media supplemented with 10%(v/v) fetal bovine serum (FBS) and Penicillin‐Streptomycin (Life Technologies), then stimulated and expanded using TransAct Human T cell activation reagent (Miltenyi Biotec #130‐128‐758) and recombinant human IL‐2 (Peprotech #200‐02‐250UG) as previously described by us [29]. The gating strategy for flow cytometry of T cells is shown in Figure S1.

### 2.3 Isolation of sEV from Human Plasma

Vesicles were isolated from the precleared plasma by ultrafiltration followed by size exclusion chromatography (SEC) as previously described [13]. Briefly, plasma samples stored at −80°C were thawed and centrifuged at 2000×*g* for 10 min, followed by centrifugation at 10,000×*g* for 30 min at 4°C. Samples were then ultrafiltered through 0.22 µm filters (EMD Millipore, Billerica, MA). An aliquot (1 mL) of plasma was loaded onto a 10 cm‐long SEC column, and 1 mL fractions were eluted with PBS. Fraction #4, containing the majority of nonaggregated, morphologically intact sEV, was collected and used for analyses. The vesicle morphology and size range, particle numbers, and protein content of the total plasma sEV (fraction #4) were determined as previously described in detail elsewhere [13]; the characteristics of total plasma sEV (and CD3(+)sEV fraction) are presented in Figure S2. The EV protein concentration was determined by the BCA method (Pierce Biotechnology, Rockford, CA) as per the manufacturer's instructions. sEV were concentrated using Vivaspin 500 (100,000 MWCO, Sartorius, Göttingen, Germany).

### 2.4 Separation of sEV by Immunoaffinity Capture

T cell‐derived vesicles (CD3(+)sEV) were separated from non‐T cell‐derived vesicles (CD3(−)sEV) using immunoaffinity capture with anti‐CD3 mAbs, which recognize an epitope selectively expressed mainly on T cell receptor‐positive (TCR+) T cells as previously described in detail elsewhere [16, 17]. Briefly, an aliquot of total plasma sEVs (10 µg of protein) was incubated with the biotin‐labeled anti‐CD3 mAbs (2.0 µg, clone Hit3a, Biolegend #300304, San Diego, CA, USA) for 2 h at RT, and then 100 µL of streptavidin‐coated magnetic beads (ExoCap, MBL International, Woburn, MA, USA) were added, followed by overnight incubation at 4°C. This capture method results in CD3(+)sEV bound to beads and soluble CD3(−)sEV in PBS. Both these sEV fractions are used for marker detection by on‐bead flow cytometry. The CD3(+) vesicles that were captured on beads were washed twice with PBS, resuspended in 100 µL of PBS, and used for the target antigen (TCR or CD3) detection by on‐bead flow cytometry. The uncaptured CD3(−)sEV were recaptured with anti‐CD63/CD81/CD9 Abs on beads and used for confirmation of the absence of CD3 in the CD3(−)sEV fraction.

### 2.5 Functional Assays—Coincubations of sEV with Target Cells

CD3(+)sEV were immunocaptured on beads from the plasma of MPs and HDs. Although various methods (enzymatic, chemical, low pH, mild detergents) were tested for the release from beads of functionally capable immunocaptured sEV, none were successful. Consequently, all functional assays were performed with CD3(+)sEV sitting on beads. Therefore, prior to performing functional assays with target cells (including CD8+ Jurkat T cells, primary CD3^+^ T cells, or Mel526 cells), we invested much time and effort in evaluating the possible influence of beads on assay results. To this end, we first performed each assay using 1 × 10^5^ Jurkat cells coincubated with increasing concentrations of anti‐CD3Ab alone, beads alone, and beads + anti‐CD3Ab. As an example, Figure S3A shows the results of the ATP lite assay for ATP production with luminescence intensity as a readout. The results were the same for the ATP lite assay and all other functional assays we performed: neither streptavidin‐labeled beads alone, anti‐CD3Ab alone, nor beads + Ab interfered with the final assay readouts. We concluded that the presence of beads + anti‐CD3Ab did not interfere with the functional assays we performed. Nevertheless, “empty” beads (no CD3(+)sEV) coated with anti‐CD3Ab served as a negative control in each assay (see Figure S3B). As MTEX were isolated from supernatants of Mel526 cells and were soluble (not on beads), they were used to compare the results of assays run with EVs on beads, as illustrated in Figure S3B for the Annexin‐binding apoptosis assay.

To demonstrate that soluble CD3(+)sEV and CD3(+)sEV on beads were readily taken up by recipient melanoma cells, we labeled the vesicles with the PKH red dye (Sigma‐Aldrich, St. Louis, MO) and followed their uptake at 0 (control), 15, and 30 min by confocal microscopy (Figure S3C). Briefly, 3 µg sEV suspended in 500 µL PBS and 500 µL diluent C were mixed with 2 µL PKH26 and incubated for 5 min at RT in the dark. Staining was quenched with 1 mL of 1% BSA. Labeled sEV were washed ×5 using 100,000 MWCO Amicon filters and resuspended in 100 µL PBS. For uptake,1 µg of freshly labeled soluble or on‐beads sEV was co‐incubated with 1 × 10^4^ Mel526 cells in 200 µL RPMI for 0, 10, or 30 min at 37°C. Cells were fixed with 4%PFA (15 min, RT), stained with beta‐actin antibody (Santa Cruz, sc‐47778, 1:100 dilution) for 1 h at RT, and Alexa Fluor 488‐conjugated secondary antibody (Invitrogen, # A‐11001, 1:100 dilution) for 1 h at RT, washed, and counterstained with DAPI. Images were acquired using a Nikon confocal microscope equipped with a 60× objective. Negative controls included cells treated with the dye only.

Because CD3(+)sEV in MPs’ plasma were captured on beads, the amount of captured CD3(+)sEV protein could not be measured directly. Instead, this amount was calculated by subtracting the level of CD3(−)sEV protein from the total sEV protein level in plasma determined before capture (both these fractions are soluble, allowing for accurate protein measurements). All functional assays were performed in triplicate, usually at low, mid, and high protein concentrations of CD3(+)sEV on beads; only results obtained with the highest amount of sEV (approximately 8–10 µg of sEV per 10^5^ target cells) are presented in Figure 2. The results of all assays were normalized to respective controls. The significance of differences between the control and the experimental condition, as well as between CD3(+)sEV of MPs and HDs, was estimated using the two‐tailed unpaired Welch's *t*‐test. Samples of 3 CD3(+)sEV of HDs (#1,#2,#3) and 3 CD3(+)sEV of MPs (#1,#2,#3) were tested with Jurkat cells as targets, and additional 3 CD3(+)sEV of HDs(#4,#5,#6) and 3 CD3(+)sEV of MPs (#4,#5,#,6) were tested with primary CD3^+^ T cells as targets; four measurements (technical replicas) were recorded each time.

Apoptosis of T cells or Mel526 cells was measured by flow cytometry using Alexa Fluor 488 Annexin V/Dead Cell Apoptosis Kit (Invitrogen V13245) in a Cytoflex flow cytometer as previously described [16, 30]. Briefly, Jurkat cells, primary T cells, or Mel526 cells were plated in wells of a U‐bottom 96‐well plate at a concentration of 1 × 10^5^ cells/well in RPMI medium. CD3(+)sEV on beads or MTEX were then added at three increasing concentrations to the cells in 100 µL RPMI medium and co‐incubated for 6 h. As controls for CD3(+)sEV, target cells were incubated with an equal volume of beads coated with CSPG4 Ab in PBS. The percentage of apoptotic cells in experiments using sEV from different donors was calculated using GraphPad PRISM10 software.

The intracellular ATP level was measured using the Luminescent ATP Lite kit (Perkin Elmer) according to the manufacturer's recommendations. The assay is based on the mono‐oxygenation of luciferin, catalyzed by luciferase in the presence of Mg^2+^, ATP, and oxygen, resulting in a luminescent signal that is proportional to the ATP concentration; the assay measures intracellular ATP levels, enabling indirect assessment of cell viability, metabolic activity, and cell proliferation. Target cells (1 × 10^5^ Jurkat, primary T cells, or Mel526 cells/well) were plated in a U‐bottom 96‐well plate; CD3(+)sEV on beads or MTEX were added and incubated for 16 h. The other assay components were then added, and after incubation of the plate for 30 min in the dark, luminescence was determined in a plate reader. Beads coated with anti‐CD3 Abs suspended in PBS were used as a negative control in case of cells co‐incubated with CD3(+)sEV.

### 2.6 Scratch Assay for the Migration of Fibroblasts

Cultured human foreskin fibroblasts were seeded at 5 × 10^4^ cells/well using 48‐well plates and starved in serum‐free media overnight. Cells were stained with a SPY555‐actin probe (Cytoskeleton Inc., Cat.# CY‐SC202 at a dilution of 1:1000 for 1 h, followed by the addition of CD3(+)sEV or MTEX. Control wells contained fibroblasts and beads coated with anti‐CD3 Ab. After 24 h incubation at 37°C in the presence of 95% CO_2_ in air, confluent cell monolayers were wounded (scratched) by using pipette tips. Wounds were imaged using an inverted microscope at 4× magnification. Imaging was repeated at 6, 12, and 24 h after the initial scratch. Wound closure areas were analyzed using ImageJ, and results are expressed as the percent of wound closure relative to the PBS control.

### 2.7 Western Blots

Lysates of total EVs isolated from plasma (input), CD3(−)sEV fraction (immunocapture of supernatant), and CD3(+)sEV on beads were resolved by SDS‐PAGE and transferred to polyvinylidene difluoride membranes as previously described [31]. For SDS‐PAGE, each lane was loaded with 10 µg protein. Following probing with a specific primary Ab and horseradish peroxidase‐conjugated secondary Ab, the protein bands were detected by enhanced chemiluminescence (Pierce). The antibodies used for Western blot analysis of proteins from plasma sEV and Jurkat cell lysates are listed in Table S11.

### 2.8 Proteomic Analysis by LC‐MS/MS of T Cell‐Derived sEV

For the MS analysis, we routinely used sEV recovered from 1 mL of plasma (this yields approximately 1 × 10^10^ to 1 × 10^11^ sEV). Protein samples were dissolved in 100 µL of lysis buffer (0.1 M Tris‐HCl pH 8.0, 0.1 M DTT, 4% SDS), heated for 1 h at 99°C with shaking (800 rpm), and then cooled down. Samples were subsequently centrifuged at 20,000 RCF for 10 min at RT, supernatants were collected and subjected to filter‐aided sample preparation (FASP) [32] as previously described [18]. Digestion with sequencing‐grade modified trypsin (Promega), enzyme‐to‐protein ratio of 1:50 m/m, was performed at 37°C for 18 h. The obtained solution of tryptic peptides was released from the filter membrane and subsequently subjected to desalting and preconcentration on the C18 bed according to the described protocol [18]. Eluted peptides were subjected to peptide assay using the tryptophan fluorescence method [33]. Purified peptide samples were then acidified (final TFA concentration: 0.1%, v/v) and subjected to LC‐MS/MS analysis using the Dionex UltiMate 3000 RSLC nanoLC System in conjunction with the Q Exactive Plus Orbitrap mass spectrometer (Thermo Fisher Scientific, Waltham, MA, USA). Peptides from each sample (0.5 µg) were separated on a reverse‐phase Acclaim PepMap RSLC nanoViper C18 column (75 µm × 25 cm, 2 µm granulation) using an acetonitrile gradient (from 3% to 60%, in 0.1% formic acid) at 30°C and a flow rate of 300nL/min (for 200 min) [18]. The spectrometer was operated in data‐dependent MS/MS mode with survey scans acquired at a resolution of 70,000 at m/z50 in MS1 mode, and 17,500 at m/z200 in MS2 mode. Spectra were recorded via positive ion scanning mode in the range of 350–1500 m/z. Using a normalized collision energy of 28, higher‐energy collisional dissociation (HCD) was used to fragment ions.

### 2.9 Protein Identification

The protein identification was performed using a reviewed Swiss‐Prot human database (release November 30, 2018) with a precision tolerance of 10 ppm for the peptide masses and 0.02 Da for the fragment ion masses. All raw data that were obtained for each dataset were imported into Proteome Discoverer v.1.4 (Thermo Fisher Scientific) <Thermo raw files> for protein identification and quantification (the Sequest engine was used for the database searches). A protein was considered positively identified when at least two peptides per protein were found by the search engine and the peptide score reached the significance threshold of FDR = 0.01 (assessed by the Percolator algorithm). A protein was further considered as “present” when it was detected in at least one sample of a given type. The abundance of the identified proteins was estimated in Proteome Discoverer using the Precursor Ions Area detector node, which calculates the abundance of a given protein based on the average intensity of the three most intensively distinct peptides for that protein, with further normalization to the total ion current (TIC); immunoglobulins (considered a “background noise”) were removed from analysis before the TIC normalization.

### 2.10 Statistical and Bioinformatics Analyses of Proteomics Data

For proteomics data, statistical analysis was performed for all 10 individuals in each group (i.e., CD3(+)sEV of MP and HD). Due to the multiple zero counts (i.e., the protein abundance was below the measurement sensitivity threshold), the protein set was divided into two subsets. The first consisted of proteins that had a nonzero abundance in at least 12/20 samples in both groups together. In this subset, proteins with different expression between melanoma patients (MPs) and healthy donors (HDs) were detected using the *U* Mann–Whitney test and rank‐biserial correlation coefficient (RBCC) [34] for effect size estimation based on abundance level. Ranks were calculated by substituting zero abundance values with half of the lowest abundance level measured in the whole dataset. For the remaining proteins (i.e., the second subset with more than 8/20 zero counts), the differences between MPs and HDs were measured based on the detected/undetected state of the proteins in a sample. Fisher's exact test was supported by Cramer's V effect size [35]. The significance level was set to 0.05. The False Discovery Rate was calculated using the Benjamini–Hochberg correction method. Differentially expressed protein (DEP) was defined as a protein characterized by at least a large effect observed between MP and HD groups. The overrepresentation of pathways was assessed using a hypergeometric test on a list of DEPs. Functional analysis was applied to Gene Ontology and Reactome pathways. The hypothetical activity of selected pathways was estimated by calculating the Pathway Activation Score (PAS) for every sample. Selection of pathways for PAS analysis consisted of the following sequential steps: (i) filter out small‐sized pathways with a number of proteins lower than the first decile; (ii) retain pathways with the number of detected proteins equal to at least the third quartile; (iii) retain pathways with the percentage of measurements in all samples equal to at least the third quartile. For the selected pathways, PAS was calculated as the median value of the abundance level of all proteins in a given sample and a given pathway, where zero abundance measurements were substituted with half of the lowest abundance level measured in the whole dataset. Next, the significance of putative pathway activation in MP (i.e., higher abundance of the pathway components) was estimated based on a *U* Mann–Whitney test and the rank‐biserial correlation coefficient (RBCC) for effect size estimation; the significance level was set to 0.05 with the False Discovery Rate calculated using the Benjamini–Hochberg correction method. The STRING database [36] was used to estimate the overrepresentation of associated pathways and to illustrate potential relationships between selected proteins (https://string‐db.org/; last accessed on April 15, 2025).

## 3 Results

### 3.1 Circulating T Cells and T Cell‐Derived sEV in Plasma of MPs and HDs

T cells were isolated from PBMCs of MPs and HDs as described in Materials and Methods and were phenotyped to confirm the percentages of CD3^+^ T cells and ratios of CD8^+^:CD4^+^ T cells. The gating strategy for flow cytometry of T cells is shown in Figure S1.

**FIGURE 1:**
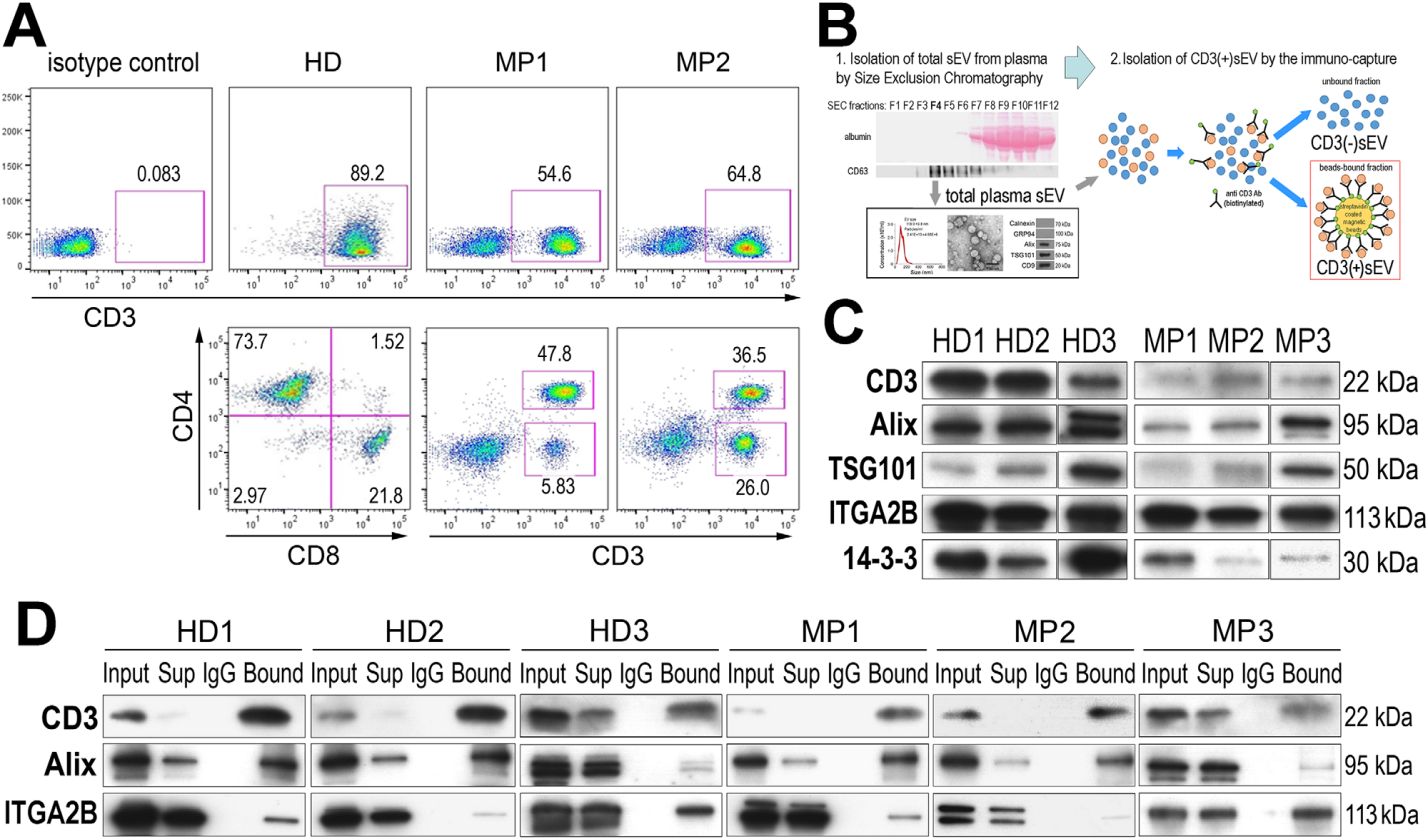
Characteristics of circulating CD3^+^ T cells and of the immunocaptured CD3(+) sEV fractions in HDs and MPs. (A) Flow cytometry of circulating CD3^+^, CD4^+^, and CD8^+^ T cells in representative HD and MP illustrates the relative paucity of circulating T cells in MPs. (B) Diagrams of total sEV isolation from plasma by SEC and their characterization by NTA, TEM, and WBs in B1, and of immune capture of CD3(+)sEV on beads in B2. (C) Western blot analysis of total sEV in plasma of HDs and MPs; note enrichment in CD3, ITGA2G, and 14‐3‐3 proteins in sEV obtained from HDs’ plasma. (D) Western blot analysis of CD3, Alix, and ITGA2B in total plasma sEV (input), sEV not bound to beads with anti‐CD3 Abs (Sup), sEV bound to beads with anti‐CD3 Abs (bound), and beads with control IgG (IgG) for the representative HDs and MPs. 10 µg of sEV were loaded in each lane.

Representative phenotyping data for T cells obtained from HDs and MPs are shown in Figure 1A. The data indicate that MPs with advanced melanoma, as indicated in Table S1, had fewer circulating CD3(+) T cells than did HDs, and either CD4^+^ or CD8^+^ T cells were variably depleted in MPs. Interestingly, this is reflected in the WBs of total plasma sEV shown in Figure 1C, where CD3 protein levels are lower for MPs relative to HDs (using 10 µg protein/lane in each case). Proteomes of the CD3(+) T cells from MPs determined by HRMS were used in our study as a reference T cell proteome (together with available literature data) as described below.

The heterogeneous populations of total sEV isolated by SEC from the plasma of HDs and MPs met the criteria attributed to sEV according to the MISEV 2021 guidelines [37]. The characteristic size (50–120 nm) and morphology of vesicles, as well as the presence of endocytic protein markers (CD9, CD63, Alix, TSG101) and the absence of cytoplasmic proteins (calnexin, Grp94) in the total plasma sEV (fraction #4) are shown in Figure S2A–C and were consistently reproduced in all sEV samples used in this study. Total plasma sEV were separated into CD3(+) and CD3(−) fractions using immune capture with anti‐CD3 Ab on beads, yielding the soluble (bead‐free) CD3(−) and the bead‐bound CD3(+) sEV fractions (Figure 1B).

Total sEV isolated from the plasma of HDs and MPs were first analyzed by Western blots to determine the presence and content of the CD3 protein relative to several sEV‐associated proteins (ALIX, TSG101) as well as signaling‐related proteins. ITGA2B, the integrin known to be present on the sEV surface and putatively related to premalignant niche formation [38], was of special interest. The data in Figure 1C compare the contents of these proteins in total sEV (10 µg protein/lane) obtained from the plasma of three representative HDs and three MPs. Importantly, in sEV of MPs, ITGA2B protein levels are highly elevated relative to CD3 protein levels.

For the immune capture of CD3(+)sEV and their separation from CD3(−)sEV, vesicles harvested from total plasma by SEC in fraction #4 were incubated with anti‐CD3 mAbs, as illustrated in Figure 1B and described in detail in Figure S2. The immune capture resulted in separation of total plasma sEV into two fractions: CD3(+) (i.e., T lymphocyte‐derived) and CD3(−) (i.e., non‐T cell‐derived) sEV. The resulting CD3(+)sEV and CD3(−)sEV fractions were monitored for CD3 positivity by on‐bead flow cytometry, as shown in Figure S2D. The sEV immunocaptured with anti‐CD3Abs on magnetic beads were from 92% to 98% CD3(+), while the CD3(−)sEV fraction contained from 1% to 9% CD3(+)sEV as determined by on‐bead flow cytometry (Figure S2D). While the CD3(+)sEV fraction largely contained sEV derived from CD3^+^ T cells, the CD3(−)sEV fraction contained a heterogeneous mix of sEV originating from various immune and/or nonimmune cells (including MTEX in the case of MPs) and was depleted of CD3(+) sEV. A complete capture of CD3(+)sEV on beads was observed in 4 out of 6 samples tested (Figure 1D). To further confirm that CD3(+)sEV from plasma effectively binds to beads under optimal capture conditions, we immunoprecipitated sEV released by Jurkat cells, which illustrated the complete binding of CD3(+)sEV to beads and their absence in the supernatant after IP (Figure S2E). Moreover, effective capture of T cell‐derived CD3(+)sEV on beads was confirmed by co‐IP with CD5, which is a cell‐surface protein found in the majority of T cells (Figure S2F). Co‐(IP) for Alix confirmed that the precipitated CD3 and CD5 were sEV‐associated (Figure S2E,F).

Due to the presence of beads used for immune capture, the numbers of CD3(+)sEV could not be counted but could be determined by subtracting the numbers (or protein levels) of soluble CD3(−)sEV from the number of total sEV recovered in fraction #4. In the individual donors, 40%–90% of total exosome protein (TEP) measured in fraction #4 was retained in the CD3(−)sEV subfraction (average = 65%) (Table S1). Hence, on average, CD3(+)sEV represented a third of total plasma sEV in MPs.

### 3.2 T Cell‐Derived CD3(+)sEV of MPs and HDs Induce Distinct Functional Effects When Co‐Incubated with T Cells and Mel526 Cells

Since CD3(+)sEV were captured on beads and could not be detached, it was necessary to show that the capture beads do not interfere with the functional assays we performed. Extensive comparisons conducted with bead‐immobilized sEV and soluble sEV indicated that beads did not interfere with sEV functions (Figure S3A,B). Importantly, we showed that the on‐bead sEV entry and accumulation in the perinuclear cytosol following 30 min co‐incubation with recipient cells were enhanced relative to those seen with soluble sEV (Figure S3C). Potentially, this was due to aggregation‐dependent endocytosis (ADE) of on‐bead CD3(+)sEV [39], although it could also be explained by the Fc‐mediated uptake.

**FIGURE 2:**
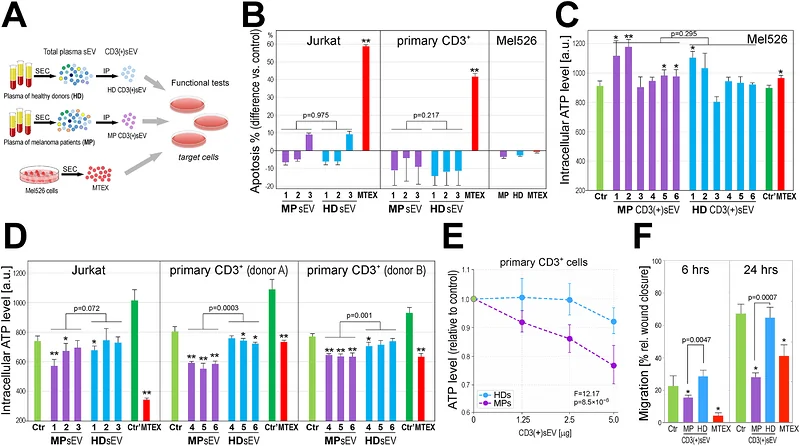
Functional responses of T cells or melanoma cells co‐incubated with CD3(+)sEV from MPs or HDs and MTEX. (A) Flowchart of functional analyses with sEV‐treated target cells. (B) Apoptosis of Jurkat cells, primary T cells, and Mel526 cells after 6 h co‐incubation with CD3(+)sEV and MTEX; relative differences against the relevant untreated controls are shown. (C) Intracellular ATP level in Mel526 cells co‐incubated with CD3(+)sEV and MTEX. (D) Intracellular ATP level in Jurkat cells and primary T cells isolated from two healthy donors (donor A and donor B), co‐incubated with CD3(+)sEV and MTEX. In panels B–D, each bar represents an independent experiment performed with CD3(+)sEV isolated from individual MPs (*n* = 3 or 6) or HDs (*n* = 3 or 6); error bars represent the standard deviation (SD) of four technical replicates. **(E)** The effect of increasing amounts of CD3(+)sEVs on intracellular ATP levels in primary CD3^+^ T cells; data from experiments using sEV isolated from 3 MPs and 3 HDs and primary T cells derived from two donors were combined. **(F)** Changes in migration of neonatal fibroblasts co‐incubated with CD3(+)sEV or MTEX in the 6 and 24 h scratch assay; error bars represent SD of four technical replicates. Anti‐CD3 Abs‐coated beads were used as negative controls in all experiments involving the co‐incubation of cells with CD3(+)sEV. Statistical significance between groups was determined using a two‐tailed unpaired Welch's *t*‐test. Significant differences between control and treatment groups are indicated by asterisks (*p < 0.05; **p < 0.001), while exact *p*‐values are shown for comparisons between CD3(+)sEV from MPs and HDs. Dose‐response curves for CD3(+)sEV derived from MPs and HDs (E) were compared using two‐way ANOVA with an interaction term.

Figure 2A illustrates the strategy for isolation of CD3(+)sEV by immunoprecipitation (IP) from plasma of MPs and HDs to be used in functional assays with various target cells: Jurkat T cells, primary human CD3^+^ T cells, or Mel526 cells. We first co‐incubated CD3(+)sEV of MPs (*n* = 3) and HDs (*n* = 3) with Jurkat CD8^+^ T cells as targets based on our previous data reporting Jurkat cell sensitivity to MTEX‐mediated apoptosis [40]. Indeed, MTEX induced high levels of apoptosis in Jurkat cells (red bar; *p* < 0.001), while CD3(+)sEV induced lower apoptosis with no significant difference between apoptosis levels induced by sEV of HDs or sEV of MPs (Figure 2B). The co‐incubation experiments were repeated using primary human CD3^+^ T cells as targets of apoptosis induced by CD3(+)sEV of HDs or MPs; the results were the same as for Jurkat cells. Similarly, MTEX induced a high level of apoptosis in primary T cells (Figure 2B). Importantly, Mel526 target cells were resistant to apoptosis by MTEX or by CD3(+)sEV of HDs and MPs (Figure 2B).

Next, we analyzed intracellular ATP levels in target cells co‐incubated for 16 h with CD3(+)sEV or MTEX. Because intracellular ATP rapidly declines in nonviable cells, ATP quantification was used as an indirect measure of cell viability, metabolic activity, and proliferation. We found that MTEX and CD3(+)sEV derived from MPs increased intracellular ATP levels in melanoma cells, with CD3(+)sEV from 4 out of 6 MPs showing statistically significant effects (*p* < 0.05). In contrast, CD3(+)sEV derived from HDs had little to no effect on ATP levels in melanoma cells (Figure 2C). These findings suggest that MTEX and CD3(+)sEV from MPs, but not from HDs, promote melanoma cell viability and proliferation. On the other hand, co‐incubation of T cells (either Jurkat cells or primary CD3^+^ T cells) with MTEX and CD3(+)sEV derived from MPs resulted in significantly reduced intracellular ATP levels, whereas CD3(+)sEV derived from HDs were markedly less effective (Figure 2D). Importantly, the differences between CD3(+)sEV from MPs and HDs were particularly pronounced in primary T cells, with highly significant effects observed in cells derived from two independent donors (*p* < 0.001). Furthermore, comparison of the dose–response curves for CD3(+)sEV derived from MPs and HDs confirmed a significant difference between the two vesicle types. A significant interaction between sEV type and dose was detected (*F* = 12.17; *p* = 8.5 × 10^−^^6^), indicating that the dose‐response relationships differed substantially between sEV from MPs and HDs (Figure 2E). Collectively, these findings indicate that MTEX and CD3(+)sEV derived from MPs, but not from HDs, reduce the viability and proliferative capacity of target T cells.

We also analyzed the effects of CD3(+)sEV and MTEX on neonatal fibroblast migration (Figure 2F). Both MTEX and CD3(+)sEV derived from MPs, but not those derived from HDs, suppressed the migration of recipient cells. The differences between CD3(+)sEV from MPs and HDs were statistically significant at both evaluated time points (*p* = 0.0047 and *p* = 0.0007 after 6 and 24 h, respectively).

In aggregate, functional in vitro studies performed with CD3(+)sEV isolated from plasma of MPs and HDs indicated differential modulation of activated T cells, neonatal fibroblasts, and melanoma cells by these vesicles. While apoptotic activity induced by CD3(+)sEV of MPs and HDs was not different, activation of T cells was significantly suppressed by CD3(+)sEV of MPs relative to CD3(+)sEV of HDs, as was the migration of neonatal fibroblasts in scratch assays. MTEX significantly increased ATP levels in autologous melanoma cells, as did CD3(+)sEV of MPs.

Overall, these functional studies indicated that responses mediated by CD3(+)sEV obtained from MPs were different from those mediated by CD3(+)sEV of HDs and tended to resemble responses induced by MTEX in T cells or in autologous melanoma target cells. Importantly, since functional tests were performed using the CD3(+)sEV bound on beads, we verified that results were not affected by the presence of beads and solely reflected activities of vesicles on beads, as described in Section 2 and in Figure S3. We have also compared the uptake and cellular fate of the internalized CD3(+)sEV on beads with the uptake of soluble CD3(+)sEV by confocal microscopy in Jurkat T cells and melanoma cells. We show that beads do not impair the uptake or processing of sEV by recipient cells.

The functional studies summarized in Figure 2 suggest that CD3(+)sEV obtained from MPs have acquired some functional attributes that are usually associated with MTEX, including pro‐tumor effects such as promotion of tumor growth and suppression of T cells or fibroblast migration. These results support our hypothesis that in MPs, T cells are reprogrammed by MTEX produced by the tumor and acquire the ability to produce and release CD3(+)sEV that differ functionally from CD3(+)sEV of HD's and recapitulate certain functional features of MTEX. To further address these hypothetical tumor‐induced differences in recipient T cells, proteomic profiles of CD3(+)sEV isolated from the plasma of MPs and HDs were compared

### 3.3 The Proteome of CD3(+)sEV Contains Proteins Characteristic of T Cells

The protein profile of captured CD3(+)sEV was assessed by HRMS in 10 HDs and 10 MPs. In total, 402 proteins encoded by unique genes were identified (after the exclusion of 94 immunoglobulin variants); 92 of the most abundant proteins were identified in each sample. This set of proteins included 108 proteins typically identified in serum/plasma specimens [41], which usually co‐purify with sEV isolated from the serum or plasma [42]. The remaining 294 proteins were considered sEV‐specific and were retained for further analyses (see list of proteins in Table S2).

In the next step, sEV proteins that could be attributed to the T‐cells were identified. As a reference, we used a set of 3281 proteins detected in the lysates of CD3^+^ T cells (after excluding immunoglobulins) isolated from the plasma of 10 MPs (the same plasma was subsequently used for immune capture of CD3(+)sEV). Additionally, we used a dataset of 6572 proteins provided by Joshi and coworkers [43], who performed an in‐depth analysis of CD3^+^/CD4^+^/CD8^−^ T cells from HDs. Both datasets generated a list of 6901 proteins present in the T cell proteome, which served as a reference for the annotation of proteins detected in the CD3(+)sEV fraction.

**FIGURE 3:**
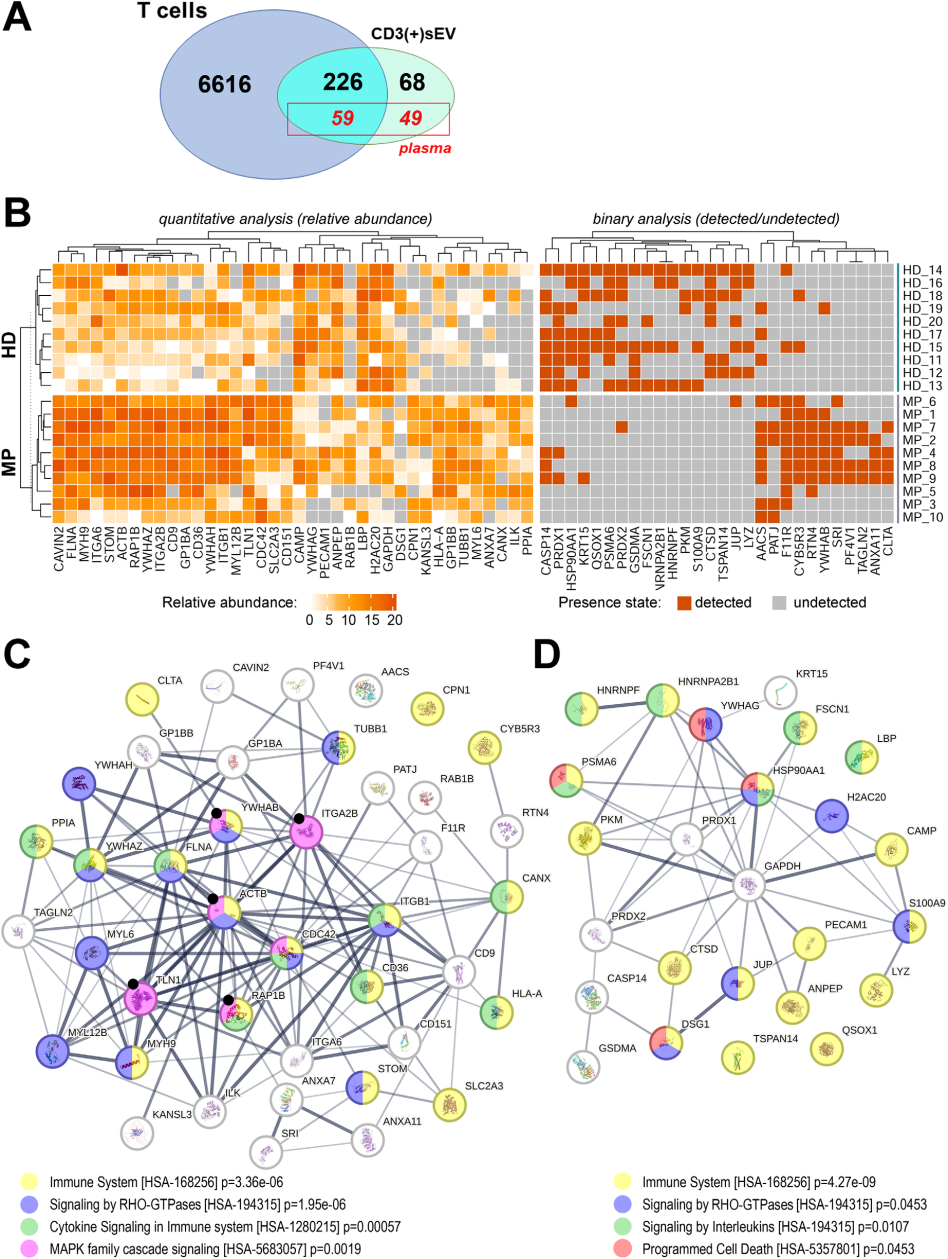
Comparisons of the CD3(+)sEV proteome of MPs and HDs. (A) The overlap between proteins detected in CD3(+) sEV and the T cell proteome (putative plasma proteins are exempted in the box). (B) T cell‐originated DEPs that discriminate CD3(+) sEV isolated from plasma of 10 MPs and 10 HDs: left part shows DEPs from continuous quantitative analysis (RBCC > 0.5; abundances are color‐coded according to ranks of all normalized signals), right part shows DEPs from discrete analysis (Cramer's *V* effect size >0.5; color‐coded is the detected/undetected status). The functional networks of T cell‐derived proteins upregulated (C) and downregulated (D) in the CD3(+)sEV from MPs; proteins that were associated with the selected biological processes are color‐coded, along with the significance of the process overrepresentation (proteins linked to BRAF‐related pathways are marked with black dots).

Out of 294 proteins identified in the CD3(+)sEV fraction that were considered sEV‐specific, 226 proteins (77%) were co‐annotated in the T cell proteome; in contrast, the remaining 68 proteins were not detected in T cells (Figure 3A; Table S2). Both subsets of proteins identified in the CD3(+)sEV fraction were associated with various biological processes/pathways. The pathway enrichment analysis revealed that putative T cell‐derived sEV proteins (i.e., proteins annotated in the T cell proteome) were primarily associated with extracellular vesicle localization (GO:0031982, 170 proteins), a large group of which was associated with the immune system (GO:0002376, 75 proteins) (Figure S4A, see also Tables S3 and S4). On the other hand, the majority of proteins detected in the CD3(+)‐sEV fraction “not” annotated in the T‐cell proteome were localized to the extracellular region (GO:0005576, 40 proteins), associated with cell adhesion (GO:0007155, 21 proteins) (Figure S4B, see also Tables S5 and S6). We concluded that the core set of proteins identified in CD3(+)sEV purified from plasma (226 proteins) represented the specific content of sEV released by T cells and could be considered as “T cell biopsy”.

### 3.4 T Cell‐Derived sEV Have Different Protein Profiles in HDs and MPs

In the core set of 226 proteins detected in CD3(+)sEV and corresponding to the T cell proteome, we searched for components that discriminated sEV of HDs from MPs. In the subset of 81 sEV proteins subjected to continuous quantitative analysis, there were 38 proteins whose levels were significantly different between the MP and HD groups (large and very large effect size; RBCC > 0.5), including 30 proteins upregulated and 8 proteins downregulated in the MP group. In the subset of 145 sEV proteins subjected to discrete binary analysis (i.e., detected vs. undetected), there were 28 proteins whose levels were significantly different between the MP and HD groups (large and very large effect size; *V* > 0.5), including 11 proteins upregulated and 17 proteins downregulated in the MP group. Collectively, there were 66 differentially expressed proteins (DEPs): 41 upregulated and 25 downregulated in the MP group (Figure 3B; Table S2).

The whole set of DEPs was subjected to the pathway enrichment analysis (Tables S7 and S8). Large subsets of DEPs were associated with over‐represented processes linked to cancer‐related functions: developmental process (GO:0032502; HSA‐1266738), regulation of response to stimulus (GO:0050896) or signal transduction (HSA‐162582), immune system (GO:0002376; HSA‐168256), cell motility (GO:0048870), and gene expression (HSA‐74160) (Figure S4C,D). In the case of MP‐upregulated DEPs, the abundant groups were associated with the immune system (18 proteins) and signal transduction (18 proteins). The latter group included 12 proteins involved in signaling by RHO‐GTPases (HSA‐194315), 9 proteins involved in cytokine signaling (HSA‐1280215), and 6 proteins involved in MAPK family cascade signaling (HSA‐5683057). Notably, this latter subset included 5 proteins (ACTB, ITGA2B, RAP1B, TLN1, YWHAB) associated with pathways linked to mutated forms of *BRAF* (Figure 3C). Among the DEPs that were downregulated in sEV of MPs, the most abundant group (17 proteins) was associated with the immune system. Furthermore, 6 proteins were involved in signaling by interleukins (HSA‐449147), 6 proteins in signaling by RHO‐GTPases (HSA‐194315), and 4 proteins in programmed cell death (HSA‐5357801) (Figure 3D).

It is worth noting that among 68 proteins detected in CD3(+)sEV not annotated in the T cell proteome were 13 DEPs, including 10 upregulated in the HD group (Table S2). However, this subset of DEPs was not associated with any overrepresented pathway related to cancer or immune processes.

### 3.5 Protein Profiles of CD3(+)sEV Putatively Reflect Differences in Pathway Activities between HDs and MPs

**FIGURE 4:**
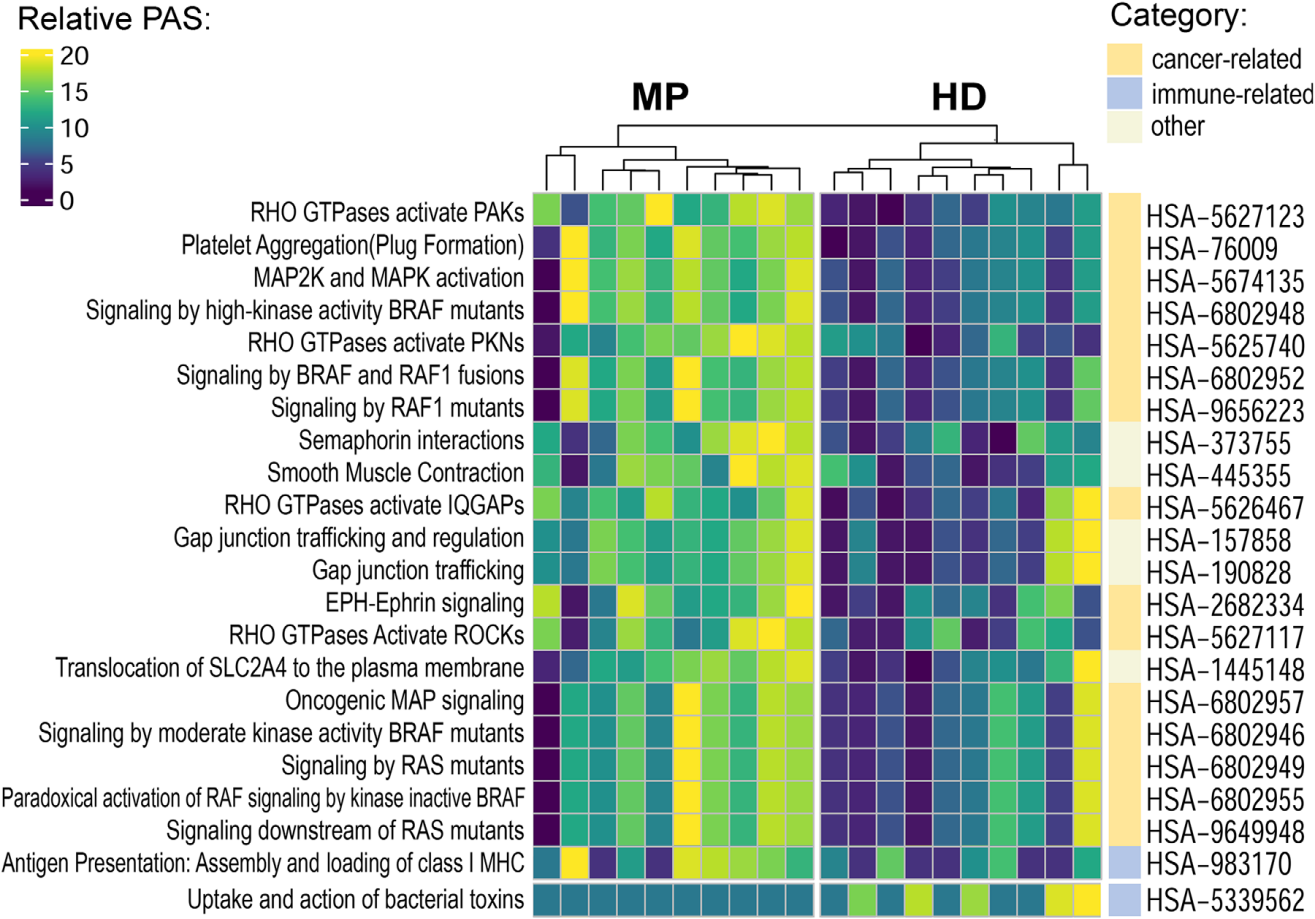
Pathways whose hypothetical activity discriminated CD3(+)sEV from MP and HD groups (RBCC > 0.5; PAS values are color‐coded according to relative value).

In addition to a classical pathway's overrepresentation analysis based on the preselected subset of DEPs, we have estimated a hypothetical activity of each pathway based on the abundance of all annotated proteins using the pathway activation score (PAS) values. The Reactome pathways were preselected using restrictive thresholds to obtain a representative set of 50 terms, whose PAS values were compared between CD3(+)sEV from MPs and HDs. As a result, 22 pathways showed significant differences (large and very large effect size) between HD and MP groups (Table S9). Among pathways relatively upregulated in the MP group (21 terms), the prevailing terms were associated with cancer‐related signaling: MAPK signaling was linked to MAPK signaling (9 terms) and RHO‐GTPases (4 terms). On the other hand, the only pathway putatively downregulated in MPs was linked to the immune response (uptake and actions of bacterial toxins) (Figure 4).

**FIGURE 5:**
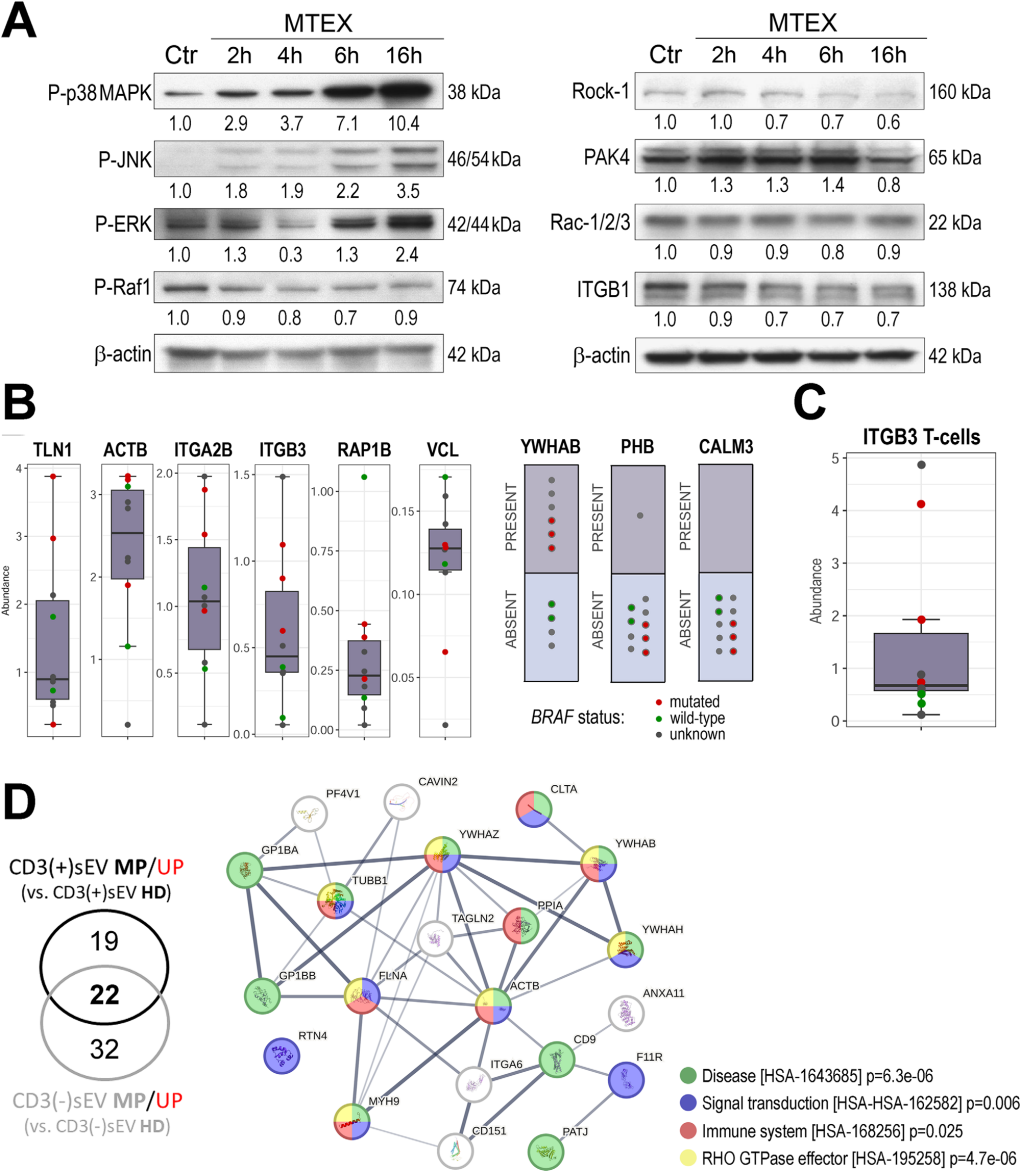
(A) Western blot analysis of proteins related to MAPK signaling (left) and RHO‐GTPase pathway (right) in Jurkat T cells co‐incubated with MTEX; relative protein levels were quantified by densitometric analysis, normalized to actin as a loading control, and expressed relative to the untreated control (quantified values are indicated below the blots. (B) Abundance of selected proteins associated with BRAF‐related pathways in CD3(+)sEV from MPs with different BRAF gene status. (C) Abundance of ITGB3 proteins in T cells from melanoma patients with different BRAF gene status. (D) Proteins upregulated in MPs’ CD3(+)sEV and CD3(−)sEV (as compared with HDs); proteins associated with the selected biological processes are color‐coded (along with the significance of the process overrepresentation).

Tumor‐derived sEV are putatively involved in the reprogramming of T cells in cancer patients, which is addressed here at the level of CD3(+)sEV from MPs. Notably, among overrepresented cancer‐related pathways associated with MTEX purified from the plasma of melanoma patients were the RHO‐GTPase pathway and RAF/MAPK signaling [13]. Hence, to confirm that cancer‐related signaling was activated in CD8+Jurkat T cells upon MTEX induction, we co‐incubated sEV derived from Mel526 cells (i.e., MTEX) with Jurkat cells for various periods and addressed the activation of different signaling pathways by Western blots. Figure 5A shows results for MTEX‐induced changes in the abundance of proteins involved in MAPK and RHO‐GTPase pathways in Jurkat T cells. As presumed, the treatment with MTEX resulted in the activation of kinases involved in MAPK signaling (phosphorylation of p38‐MAPK, ERK, and JNK) and affected levels of proteins associated with the RHO‐GTPase pathway. It is also worth noting that among the proteins upregulated in CD3(+)sEV from MPs (as compared with vesicles from HDs) were several species similarly upregulated in MTEX (relative to N‐MTEX) from the plasma of MPs [13] (illustrated in Figure S5A), which included proteins associated with the RHO‐GTPase pathway (ACTB, MYH9, TUBB1, and YWHAZ).

Interestingly, examining the mass spectrometry data, we noted that nine proteins detected in CD3(+)sEV (ACTB, CALM3, ITGA2B, ITGB3, PHB, RAP1B, TLN1, VCL, and YWHAB) were associated with the oncogenic MAPK signaling pathway (HSA‐6802957) linked to functions of *BRAF* mutants. As indicated above, the PAS analysis showed that these pathways were upregulated in sEV isolated from the MP group. As the *BRAF* status was known in 5 MP donors (2 wild‐type *BRAF*, 3 *BRAF*v600 mutations; Table S1), we were able to search for a possible link between the level of these proteins in sEV and the *BRAF* status of these few MPs. Interestingly, although the results are preliminary, we found that the abundance of ITGB3 and YWHAB was lower in CD3(+)sEV from two patients with BRAF^wt^ melanoma than in three patients with *BRAF* ^mut^ melanoma (Figure 5B). Moreover, a strong correlation (Spearman's rank correlation coefficient, 0.697) was observed between individual levels of ITGB3 in T cells and CD3(+)sEV (Figure 5C). Despite its preliminary nature, the observed putative association between melanoma *BRAF* status and the levels of proteins linked to BRAF‐related pathways in both T cells and T cell‐derived sEV further underscores the potential diagnostic relevance of CD3(+)sEV in cancer patients.

### 3.6 CD3(−)SEV from HDs and MPs Have Different Protein Profiles

While the primary objective of the analysis was to characterize T cell‐derived CD3(+)sEV, the CD3(−)sEV purified from the same cohort of HDs and MPs were also analyzed by HRMS. Proteomic and metabolomic profiles of CD3(+)sEV and CD(−)sEV from normal human plasma were comprehensively compared in our earlier study [18]. Here, we focused on the comparison of CD3(−)sEV from HDs and MPs. It could be expected that in MPs, MTEX represents a substantial fraction of CD3(−)sEV, which is absent in the corresponding fraction of plasma sEV in HDs. Indeed, proteomic profiling revealed significant differences between CD3(−)sEV of MPs and HDs. In general, 282 unique proteins were identified in CD3(−)sEV, including 67 DEPs (large and very large effect size, RBCC > 0.5 or *V* > 0.5): 54 DEPs upregulated, and 13 DEPs downregulated in vesicles from MPs compared with HDs (Table S10). The pathway enrichment analysis revealed that CD3(−)sEV proteins were primarily associated with the immune system (HSA‐168256, 93 proteins), signal transduction (HSA‐162582, 79 proteins), and hemostasis (HSA‐109582, 58 proteins), as shown in Figure S6A. Noteworthy, analysis of overrepresented pathways associated with DEPs upregulated in CD3(−)sEV from MPs revealed pathways that were associated with proteins upregulated in MTEX purified from the plasma of melanoma patients in our previous study [13], including signal transduction (22 proteins), disease (21 proteins), signaling by RHO‐GTPases (15 proteins), translocation of SLC2A4 (GLUT4) to the plasma membrane (10 proteins), Axon guidance (10 proteins), and cell cycle (7 proteins), (Figure S6B). Moreover, several proteins upregulated in CD3(−)sEV from MPs were also upregulated in MTEX from patients’ plasma (Figure S5B), including ACTB, GAPDH, LRP1, MYH9, TUBA4A, TUBB1, YWHAE, and YWHAZ, which further confirmed that CD3(−)sEV from the plasma of melanoma patients contained vesicles derived from melanoma cells. Furthermore, among proteins upregulated in CD3(−)sEV from MPs (compared with vesicles from HDs) were several species similarly upregulated in CD3(+)sEV from these patients (Figure S5C). It is worth noting that the subset of DEPs that were common in CD3(+)sEV and CD3(−)sEV represented more than half (22 of 41) of DEPs upregulated in CD3(+)sEV from MPs. This subset of DEPs common for CD3(+)sEV and CD3(−)sEV included proteins linked to cancer‐related pathways: “disease”, “immune system”, “signal transduction”, and “RHO‐GTPase effectors” (Figure 5D). This indicated that the melanoma‐related signature was observed both in CD3(+) and CD3(−) populations of plasma sEV in MPs.

## 4 Discussion

Small extracellular vesicles (sEV) are a newly emerging source of biomarkers in cancer, which can not only serve as “tumor liquid biopsy” but also as a surrogate of immune cells, reflecting phenotypic and functional attributes of their parent cells. The role of immune cells in inhibiting or promoting cancer progression is indisputable. Immunocompetence of patients with cancer, especially patients with advanced disease, is usually compromised [44]. Hence, immune cells found in situ and those present in patients’ body fluids are being used as biomarkers of immune competence and response to therapy, particularly in immunotherapy [45]. The currently promoted view that tumor‐resident T cells and circulating T cells represent distinct immune cell populations is not uniformly accepted, as cancer is a systemic, not local disease, and many studies have confirmed associations of phenotypic and functional attributes of circulating immune cells with disease status and progression [46]. This may be especially relevant to tumor‐derived EVs, which circulate freely [47], crossing all tissue and organ barriers and targeting all T cell populations. Nevertheless, a formal comparison of TEX with paired circulating immune cell‐derived EVs as biomarkers is yet to be performed. The fact is that studies of the immune cell presence and functions in the TME or body fluids are laborious, requiring tissue biopsies and/or serially sampled blood specimens [48]. In contrast, EVs can be repeatedly isolated from 1 mL of plasma in the course of disease in numbers sufficient for phenotypic and functional analyses and thus represent a more clinically promising alternative. Our earlier studies indicated that sEVs circulating in plasma can be used in place of tumor cells or immune cells as biomarkers of tumor progression/response [49], as well as of T cell competence [18]. However, since plasma contains a heterogeneous mix of EVs with distinct biogenesis and different phenotypic molecular attributes, it is unclear which EV subsets play a role as actual biomarkers. We reasoned that sEV subfractionation based on their surface attributes might provide a solution.

Here, we show that using immunocapture with Abs specific for the CD3 antigen, which is uniquely expressed on T cells and is carried by T cell‐derived sEV, it is possible to successfully separate CD3(+)sEV produced by T cells from CD3(−)sEV derived from all other cells (including tumor‐derived vesicles in cancer patients). Previously, we showed that this separation is feasible and yields highly informative data about the nature and functions of parent T cells in patients with head and neck squamous cell carcinoma HNSCC [49]. More recently, in a pilot study designed to interrogate the role of plasma CD3(+)sEV and CD3(−)sEV as predictive biomarkers of response to immunotherapy with pembrolizumab, we immunocaptured CD3(+)sEV from the plasma of patients with recurrent/metastatic HNSCC [50]. The study showed that despite small patient numbers, both immunocaptured sEV subsets provided significant data correlating the frequency and phenotype of sEV in preimmunotherapy plasma to overall survival and progression‐free survival [50]. Thus, both the CD3(+)sEV and TEX‐enriched CD3(−)sEV from the same plasma sample were found to be useful in predicting response to immunotherapy.

Studies indicate that progressing tumors induce immune suppression by interfering with antitumor functions of immune cells, which is frequently mediated by TEX [51]. As a result of interactions with TEX, T lymphocytes and other types of immune cells undergo various phenotypic and functional alterations and either lose antitumor activities [19] or acquire tumor‐promoting functions [23]. This process, broadly referred to as tumor‐induced reprogramming, is being extensively evaluated in the context of TEX [52]. While the available data suggest that TEX suppress antitumor immunity and reprogram the TME, inducing pro‐tumor effects in various immune and nonimmune cells [25, 52, 53], we observed that sEV produced by reprogrammed T cells also contribute to immune dysfunction and impact patients’ response to therapy [53]. Here, we suggest that TEX‐driven reprogramming extends to circulating T cells, involving all types of CD3+ T cells in patients with advanced melanoma, although our earlier studies indicated that subsets of activated CD8^+^ effector T cells are especially sensitive to TEX‐mediated effects [16, 40]. These data support the notion that sEV produced by circulating reprogrammed T cells in MPs contribute to immune dysfunction and thus may impact patients’ response to therapy.

Hence, it is worth emphasizing that our proteomics dataset identified several proteins known to be involved in tumor‐related immune regulation that differentiated CD3(+)sEV from MPs and HDs. Based on preliminary evidence reported in this proof‐of‐concept study, we suggest that both TEX and T cell‐derived CD3(+)sEV circulating in cancer patients’ plasma could serve as a noninvasive “liquid biopsy”. In this context, TEX recapitulate the molecular/genetic content of the tumor, and the reprogrammed circulating CD3(+)sEV report on the immune competence of the host T cells. Our preliminary data obtained with EVs of a small cohort of melanoma patients support the notion that circulating T cell‐derived sEV reflecting attributes of both circulating and tissue‐infiltrating T cells may serve as a general “T cell biopsy”. In this respect, it is important to remember that circulating sEV are likely products of all T cells, including tumor‐infiltrating T lymphocytes (TILs), that circulate freely, crossing all tissue barriers [47]. The use of CD3(+)sEV as a liquid biopsy in place of T cells is an advantage: serially collected 1 mL aliquots of plasma are adequate for sEV recovery and testing without T cell isolation, cryopreservation, and viability concerns.

The innovative separation by immune capture of T cell‐derived sEV from the plasma of melanoma patients allowed us to compare proteomic profiles of T cell‐derived vesicles from cancer patients and healthy donors. Here, we documented for the first time that proteomic profiles of T cell‐derived sEV circulating in the plasma of cancer patients and healthy individuals were distinct. Specifically, a set of proteins identified in the T cell‐derived vesicles included species involved in the function of the immune system and included an increased abundance of immunoregulatory proteins in CD3(+)sEV in MPs. In particular, this included a few immunosuppressive proteins overexpressed in most solid tumors that correlate with cancer progression and poor prognosis, including CD36 [54], CD47 [55], and CD59 [56]. Moreover, among proteins upregulated in CD3(+)sEV from MPs were CD151 and CD226. Although these proteins are known to have tumor‐suppressive functions, in certain specific contexts, they could also be tumor‐promoting [57, 58]. On the other hand, among proteins downregulated in MPs’ CD3(+)sEV were heat shock proteins, including HSP90AA1, HSPA1B, and HSPD1, which are general stimulators of immune response (e.g., via enhancing antigen presentation and chaperoning toll‐like receptors [59]. Similarly, among downregulated processes associated with proteins discriminating MPs from HDs was the response to pathogens. Hence, the concept that CD3(+)sEV from MPs reflects immunosuppressive features of cancer‐reprogrammed T cells is supported in our pilot proteome profiling. Moreover, a large subset of proteins that differentiated vesicles from patients and controls included molecules involved in other important cancer‐related functions: regulation of development, cell motility, gene expression, and signal transduction. Notably, proteins upregulated in T cell‐derived sEV from MPs were associated with the RHO‐GTPases pathway and signaling linked to MAPK family kinases (BRAF kinase in particular). Interestingly, Azoulay‐Alfaguter and Mor [60], who performed proteomic analysis of exosomes from resting vs activated T cells, report enrichment in MAPK and RAS signaling pathways in exosomes derived from activated T cells, as well as enrichment in the immune surface receptor signaling pathways and metabolic processes. Since a substantial proportion of T cells in MPs are likely to be in a “functionally activated” state, these reported results support our correlative findings. We suggest that T cells in melanoma patients are reprogrammed to produce and release sEV that carry a protein cargo partly resembling that expressed by melanoma cells [61]. The mechanisms responsible for T cell reprogramming in cancer that are driven by TEX are under intense investigation and focus on transcriptional and translational changes induced in recipient T cells by miRNA and/or enzymatic activities of TEX [16, 62]. Further assessments of these changes, including the upregulated RHO‐GTPases and MAPK/BRAF pathways in CD3(+)sEV, are necessary to confirm our preliminary data and to provide insights into the degree of T cell reprogramming induced by cancer and thereby elucidate the role of CD3(+) vesicles as biomarkers of cancer progression or response to therapy.

## 5 Data Limitations and Perspectives

The presented data have several limitations that may impact the study conclusions. In addition to a small patient cohort, several strategic issues such as the use of nonvalidated bead‐based EV isolation and the indirect EV quantification could potentially skew the results interpretation. There is also a concern that only limited immune functions were tested, with the absence of proliferation and cytokine production assays, and that direct mechanistic evidence for TEX‐induced reprogramming of T cells was lacking. While these limitations are recognized and are rationally discussed, the perspectives of the study should remain tentative until additional evidence for TEX‐driven reprogramming is obtained. Nevertheless, it is clear that separation and subtyping by immune capture of T cell‐derived CD3(+)sEV from MTEX‐enriched CD3(−)sEV provides a potential strategy for performing “liquid T cell biopsy” and “liquid tumor biopsy” in the same specimen of a cancer patient's plasma. While this study addressed the former, we are further profiling the proteome of the TEX‐enriched sEV to confirm our already reported data on the unique proteomic profile of MTEX in melanoma [13]. Performed simultaneously using a small volume (1 mL) of plasma and interpreted in the context of clinical endpoints, the strategy is expected to validate the role of these sEV subsets as interdependent biomarkers of tumor progression and response to therapy.

## Author Contributions

T.L.W. and M.P. were responsible for project design, overall supervision, data interpretation, and manuscript writing. P.W. analyzed proteomics results, wrote the manuscript, designed figures, and provided editorial supervision. M.P., M.G., and A.Z. performed and interpreted proteomics analyses. Y.G.N. provided clinical support and participated in writing the manuscript. A.G. performed functional studies of sEV. J.H. performed western blots and assisted in functional sEV studies. S.M. performed immune capture of sEV. J.P. and J.M. performed bioinformatics analyses and provided figures illustrating proteomics results.

## Conflicts of Interest

The authors declare no conflicts of interest.

## Supporting information




**Supporting File**: eji70273‐sup‐0001‐SuppMat.pdf.

## Data Availability

Data are available upon request by the authors. The Mass spectrometry proteomics data have been deposited to the ProteomeXchange Consortium via the PRIDE [63] partner repository with the temporary dataset identifier PXD052476 (https://www.ebi.ac.uk/pride/).
