# Supplementary material for: Functional and Proteomic Profiles of CD3(+) Plasma‐Derived Small Extracellular Vesicles Differentiate Cancer Patients From Healthy Donors
Source: Eur J Immunol. 2026 Sep 7;56(9):e70273. doi: 10.1002/eji.70273 (PMC13548450; doi:10.1002/eji.70273)
Supplement: Supplementary file 1 [file EJI-56-e70273-s001.pdf]

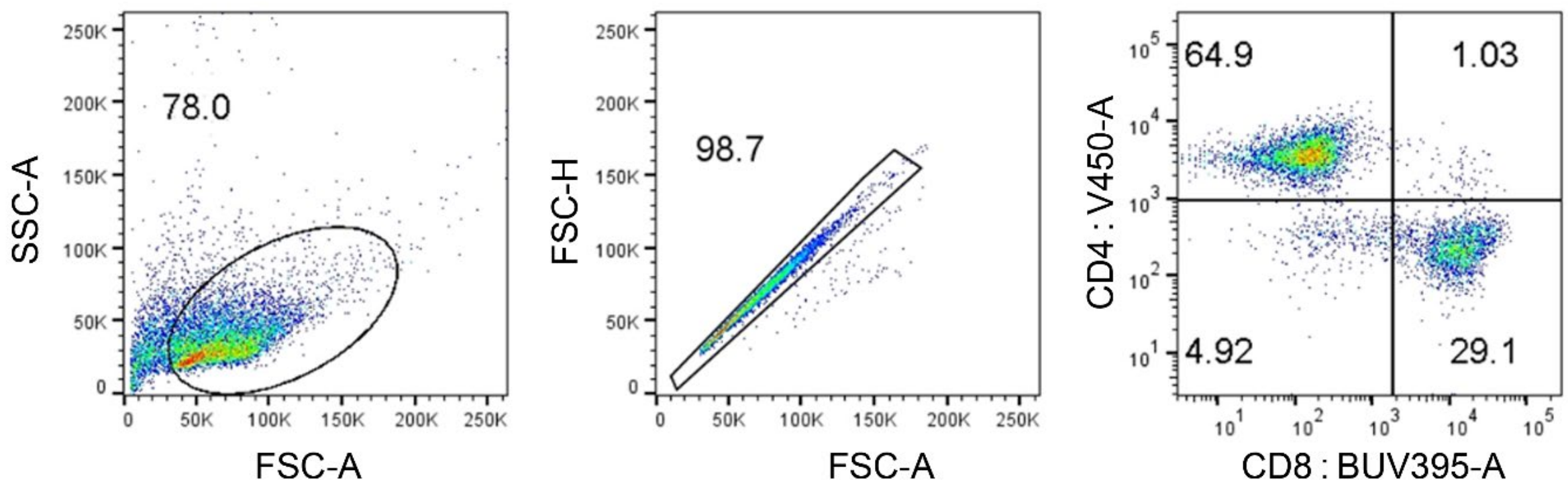

SFigure 1. Gating strategy *for flow cytometry***.** *Using SSC and FSC, we gate on live T cells. Next, doublets are eliminated by using a tight gate. Lastly, we gate on CD4⁺ and CD8⁺ T cells*

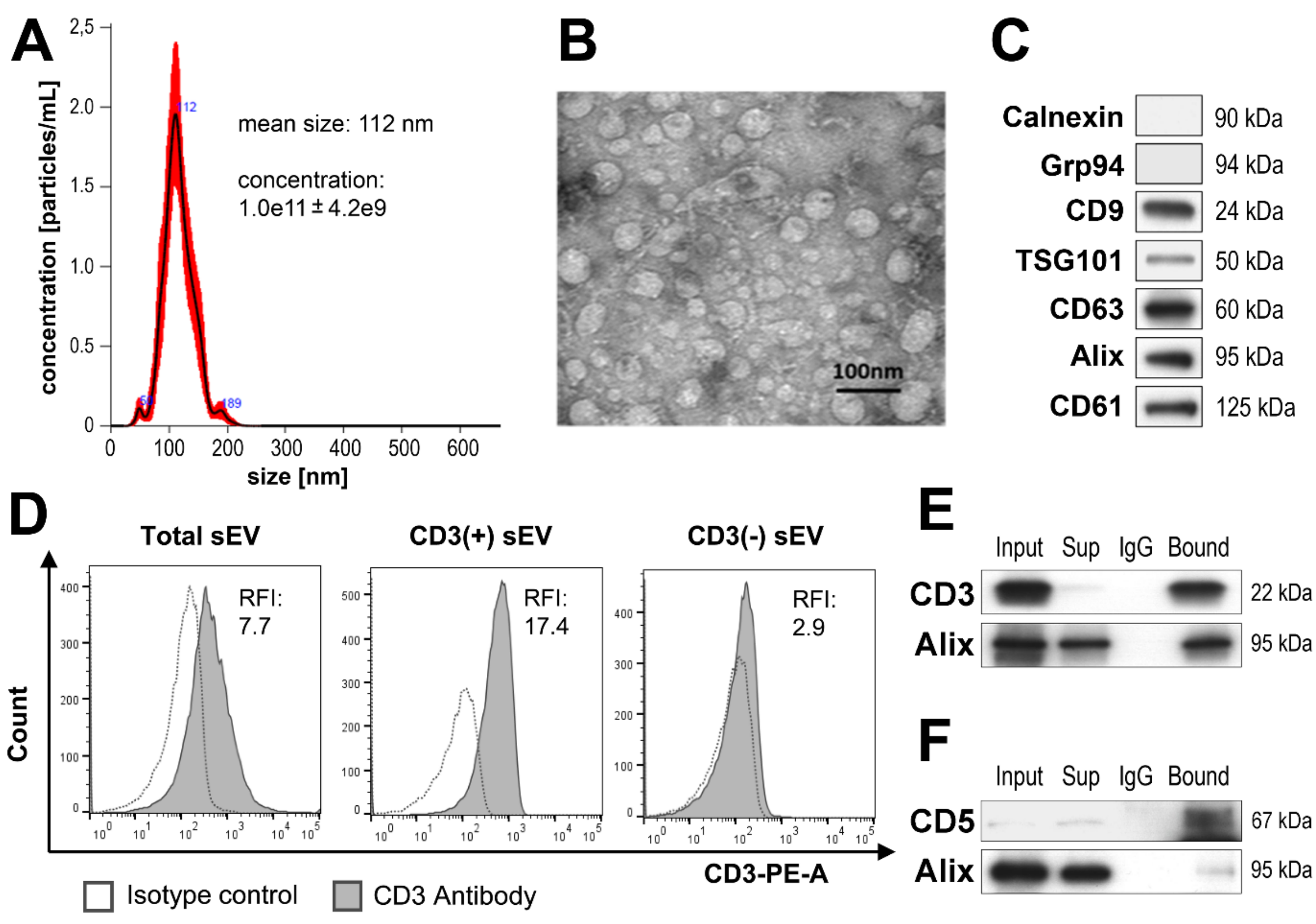

SFigure 2. ***Characteristics of sEV isolated from human plasma. Panel A*** *- Nanoparticle tracking analysis (NTA) results show the size and concentration of the total plasma sEV (SEC fraction #4).* ***Panel B*** *- transmission electron microscopy (TEM) images of the vesicles; scale bar = 100 nm.* ***Panel C*** *- Western blots of total plasma sEV: the presence of endocytic markers (Alix, TSG101, CD9, CD63), and the absence of cytoplasmic proteins (calnexin and Grp94) confirm that the analyzed vesicles are derived*

from the parental cell endosomes. ***Panel D*** *– Immune capture of CD3(+)sEV from human plasma results in the separation of CD3(+) and CD3(-) fractions. The presence of CD3 in these fractions is analyzed by on-bead flow cytometry. Relative Fluorescence Intensity (RFI) is calculated as MFI sample/MFI isotype control (the figure is representative of all sEV samples used in this study).* ***Panel E*** *– Western blot of CD3(+)sEV bound on beads after immunoprecipitation (IP) from supernatant of Jurkat T cells with anti-CD3 mAb (input). Note a near complete removal of CD3(+)sEV from the supernatant, indicating that practically all CD3(+)sEV are bound to beads. Co-IP for Alix, a marker of sEV, confirms that precipitated CD3 is EV-associated.* ***Panel F*** *– Western blot of CD3(+)sEV bound on beads after immunoprecipitation with anti-CD3 Abs from plasma of melanoma patient, illustrating co-IP of CD5 with anti-CD5Abs.*

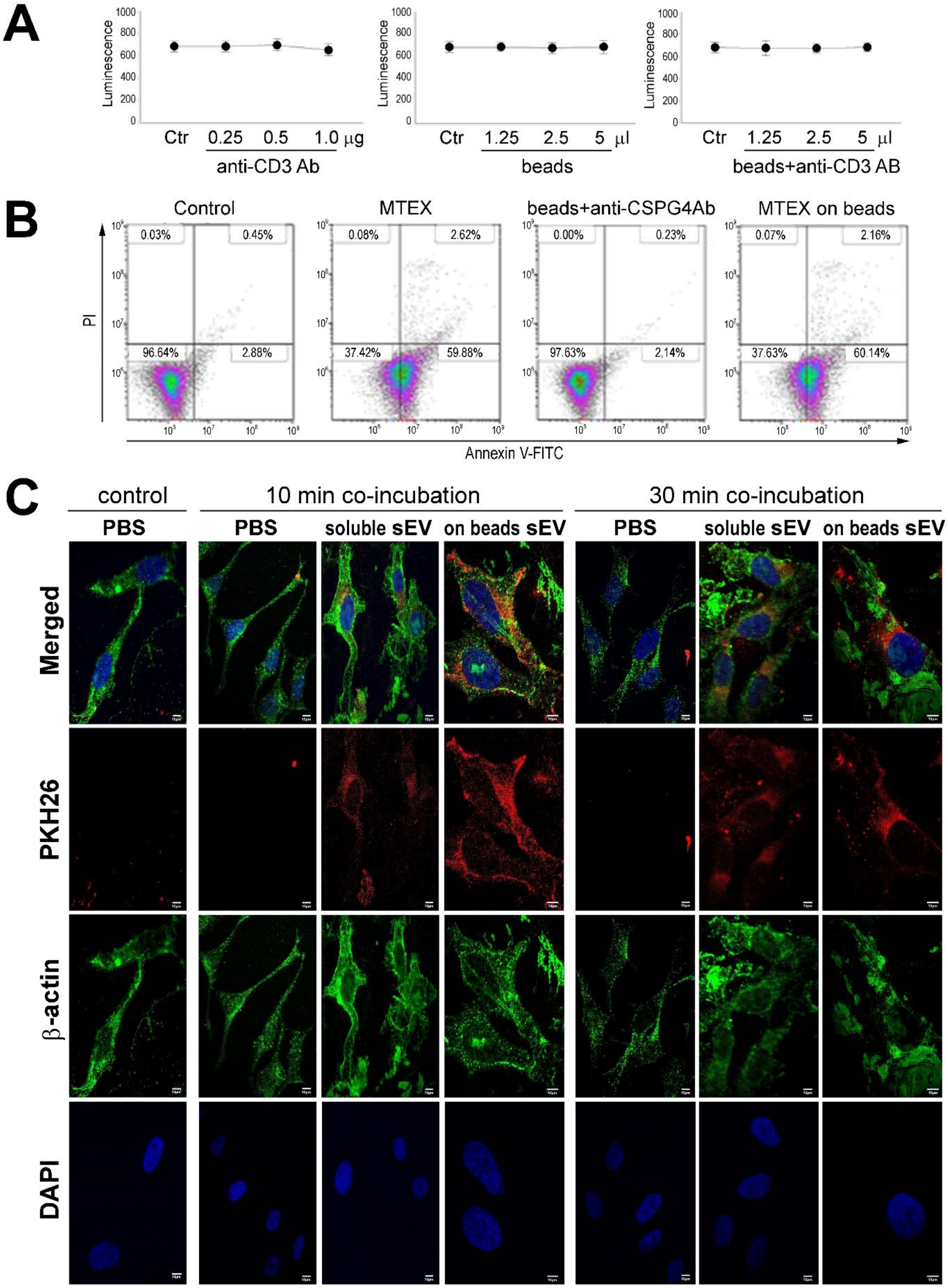

A
Luminescence
Ctr
0.25
0.5
1.0 μg
anti-CD3 Ab
1.25
2.5
5 μl
beads
beads+anti-CD3 AB
B
Control
MTEX
beads+anti-CSPG4Ab
MTEX on beads
PI
Annexin V-FITC
0.03%
0.45%
96.64%
2.88%
0.08%
2.62%
37.42%
59.88%
0.00%
0.23%
97.63%
2,14%
0.07%
2.16%
37.63%
60.14%
C
control
10 min co-incubation
30 min co-incubation
PBS
soluble sEV
on beads sEV
Merged
PKH26
β-actin
DAPI

SFigure 3. Beads used for immunocapture of sEV do not interfere with functional assays. **Panel A** - *the ATP lite assay for intracellular ATP level in Jurkat cells coincubated with increasing concentrations of anti-CD3Ab alone, beads alone, and beads with anti-CD3Ab.* ***Panel B*** *- Apoptosis of primary activated CD8+ T cells co-incubated with PBS (Control), MTEX isolated from Mel526 supernatant (no beads), beads coated with anti-CSPG4 Ab used for MTEX capture (no MTEX), MTEX isolated from Mel526 and captured on beads. Vesicles were added to T cells, and 6h co-incubation was followed by flow cytometry. Apoptosis was measured using the Annexin V-FITC Apoptosis Detection kit (Abcam).* ***Panel C*** *– Soluble CD3(+)sEV and CD3(+)sEV on beads were readily taken up by the recipient Mel526 cells. sEV were labelled with the PKH-26 red dye and their uptake into the cytosol of Mel526 cells at 0 (control), 15, and 30min was followed by confocal microscopy. After 30 min, red-labelled vesicles exhibiting more intense staining are seen in the perinuclear areas of the melanoma cells internalizing on-bead CD3(+)sEV. The scale bar = 10µm.*

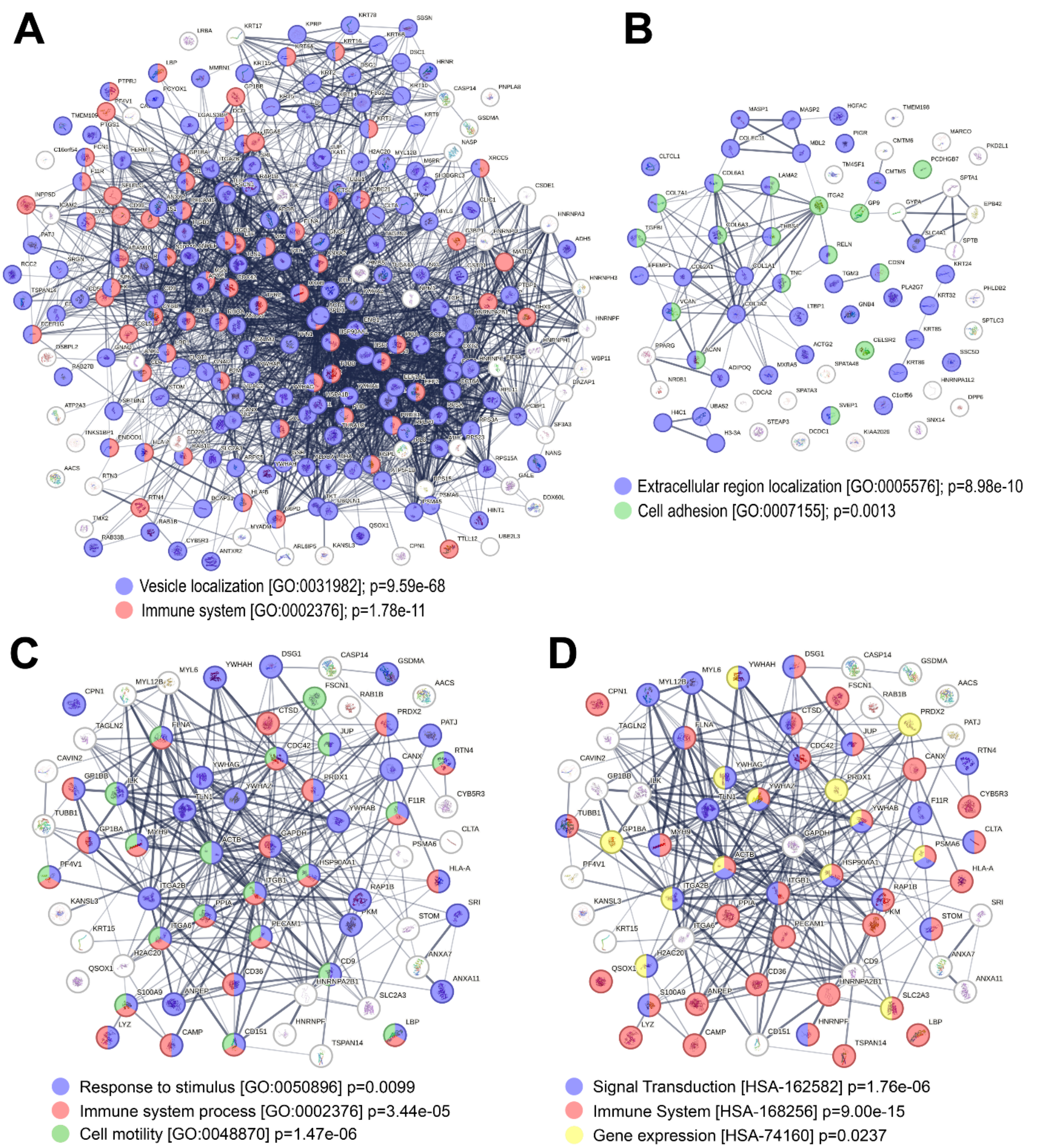

SFigure 4. Functional networks of proteins detected in the CD3(+)sEV fraction. Proteins *that were either annotated (**Panel A**) or not annotated (**Panel B**) in the T cell proteome; proteins that were associated with the selected biological processes are color-coded, along with the significance of the process overrepresentation. The functional networks of T-cell-derived DEPs that differentiate HDs and MPs: selected overrepresented pathways were annotated according to Gene Ontology terms (**Panel C**)*

*or REACTOME pathways (**Panel D**); shown are p-values of pathway's overrepresentation.*

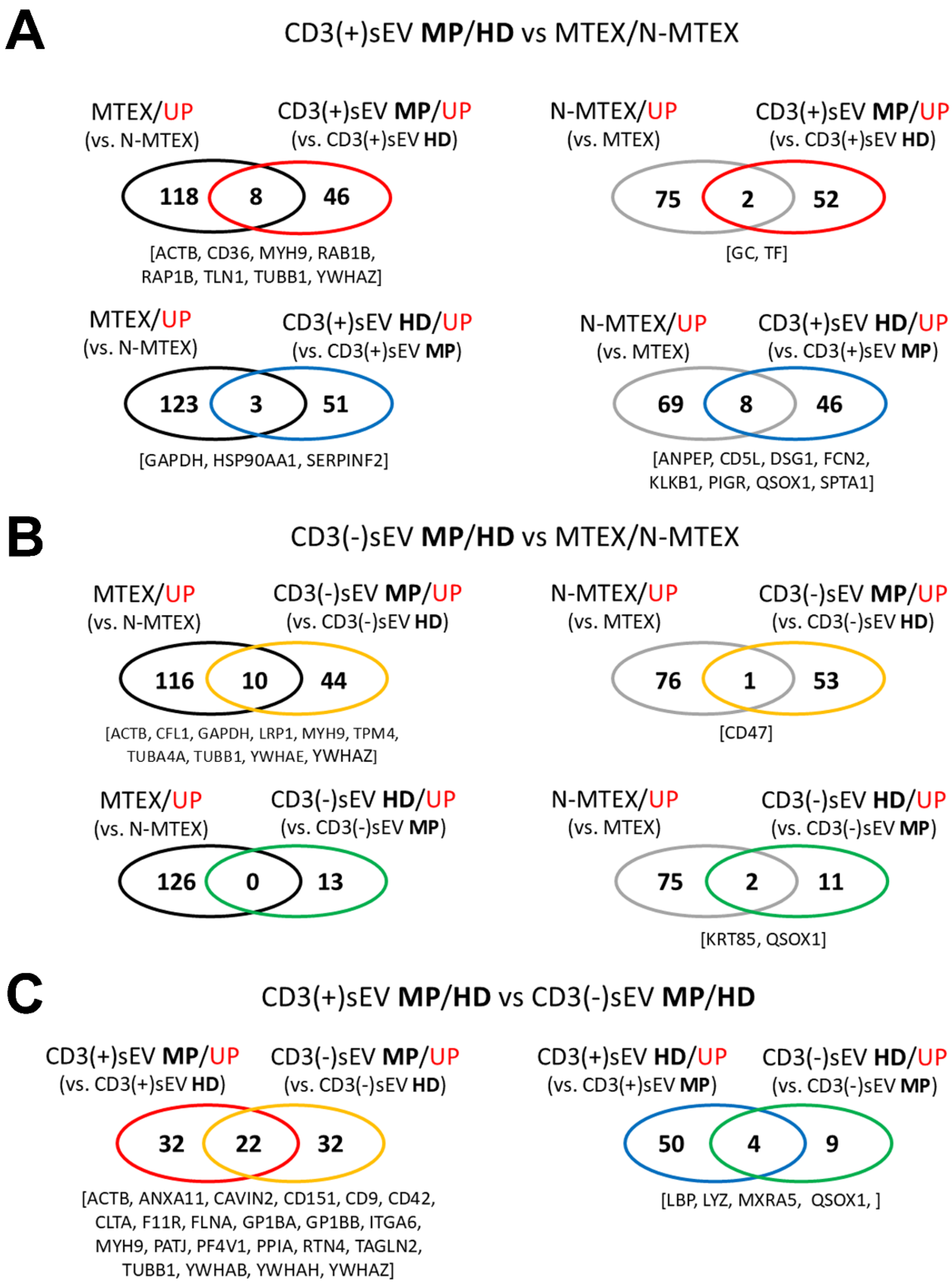

SFigure 5. *Venn diagrams showing the similarity of DEPs differentiating sEV subgroups from melanoma patients (MPs) and healthy donors (HDs). Overlap of proteins upregulated in MTEX/N-MTEX (data from Pietrowska et al. 2021[13]) and CD3(+)sEV* ***(Panel A)*** *or CD3(-)sEV* ***(Panel B)*** *from MPs and HDs. (****Panel C)*** *Overlap of proteins upregulated in CD3(+)sEV and CD3(-)sEV from MPs or HDs. All DEPs (large and very large effect size) detected in a given subset of sEV are counted, and the overlapping DEPs are listed below the diagrams.*

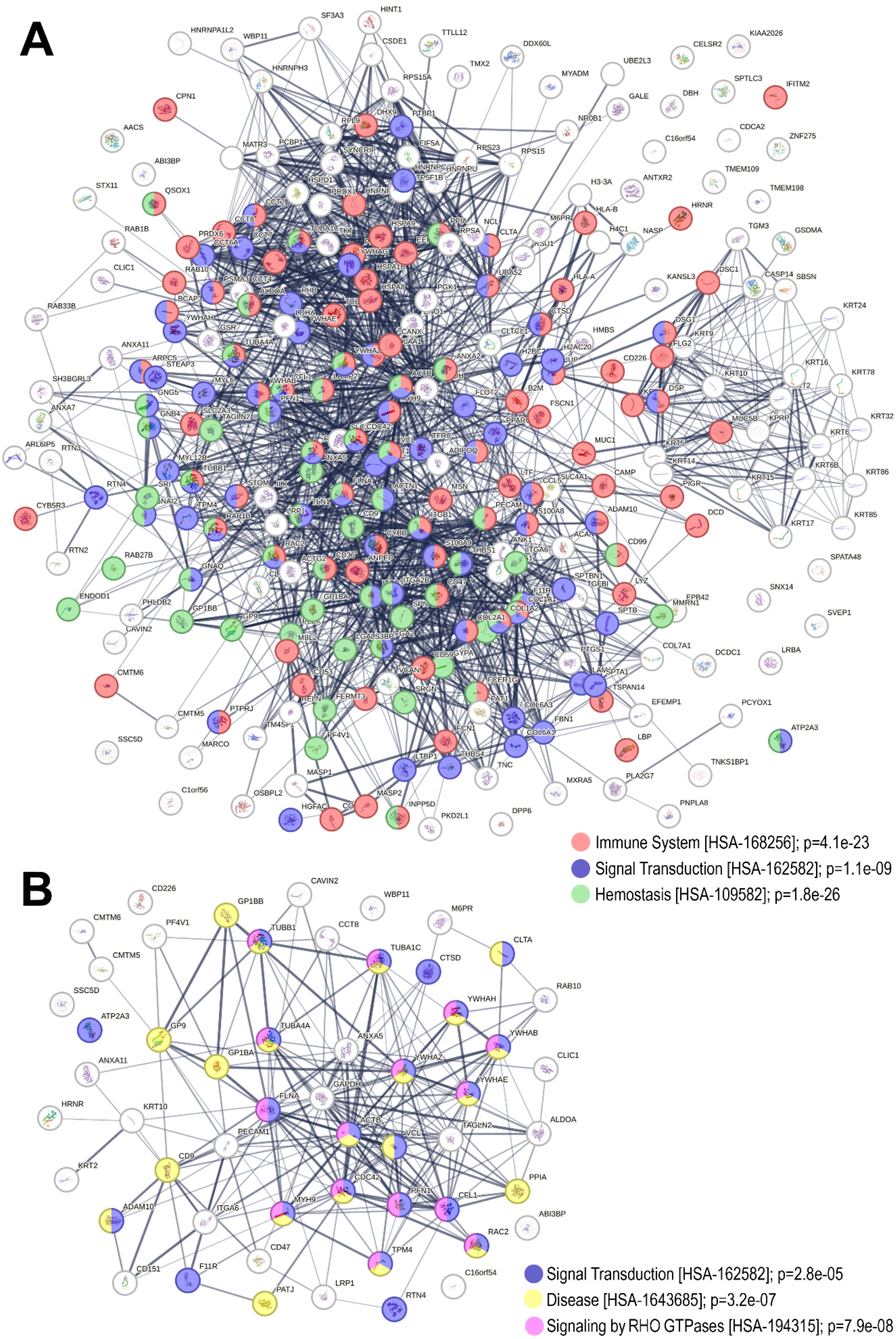
A
Immune System [HSA-168256]; p=4.1e-23
Signal Transduction [HSA-162582]; p=1.1e-09
Hemostasis [HSA-109582]; p=1.8e-26
B
Signal Transduction [HSA-162582]; p=2.8e-05
Disease [HSA-1643685]; p=3.2e-07
Signaling by RHO GTPases [HSA-194315]; p=7.9e-08

SFigure 6. Panel A *- Functional networks of proteins detected in the CD3(-)sEV fraction.* ***Panel B*** *- Functional networks of DEPs upregulated in CD3(-)sEV from MPs; proteins that were associated with the selected biological processes are color-coded, along with the significance of the process overrepresentation.*

Table 1. **Characteristics of the melanoma patiens**

Patient ID	Age at diagnosis	Age at blood draw	Gender	Clinical stage at diagnosis	Status of lymph node	Disease status at blood draw
MP_1	56	58	M	IV	negative	SD
MP_2	29	31	M	IIIC	N/A	PD
MP_3	68	71	M	IB	negative	NED
MP_4	54	73	M	IB	N/A	PD
MP_5	56	57	F	IVA	N/A	PD
MP_6	36	41	M	IV	N/A	PD
MP_7	57	63	M	NA	positive	PD
MP_8	32	33	F	NA	negative	PD
MP_9	56	57	M	III	positive	PD
MP_10	77	79	M	NA	N/A	PD

Mutation status	Total sEV (F#4) number/mL	EV size (nm) (NTA)	TEP in F#4 (µg)
BRAF codon 600 (p.K600V)	2.08E+10 ± 1.82E+09	91.7 ± 1.6	241.2
NRAS codon 61 (p.Q61K, c.181C>A); BRAF wildtype	4.19E+10 ± 2.02E+09	109.0 ± 7.8	226.8
BRAF codon 600 (p.V600E, c.1799T>A)	4.35E+10 ± 1.52E+09	87.7 ± 2.1	316.8
BRAF codon 600 (p.V600K, c.1798_1799GT>AA)	2.71E+10 ± 1.97E+09	60.5 ± 3.5	244.8
BRAF/NRAS wildtype	4.09E+10 ± 2.09E+09	81.9 ± 2.8	230.4
unknown	2.41E+10 ± 5.43+08	99.7 ± 6.6	90
unknown	1.81E+10 ± 1.22E+09	63.5 ± 3.9	162
unknown	5.72E+10 ± 3.61E+09	67.2 ± 2.8	112
unknown	1.37E+11 ± 3.43E+09	99.3 ± 12.5	202
unknown	1.99E+10 ± 1.55E+09	77.4 ± 3.2	166

Amount of protein used for immunocapture (µg)	Percentage of protein retained in CD3(-) sEV fraction
150	53
148	42
148	88
149	78
171	63
77	56
150	52
97	65
158	95
149	71

Table 2. Proteins identified in CD3(+) fraction of plasma sEV

ProteinName	Protein Description	MP_1	MP_2
**A1BG**	Alpha-1B-glycoprotein	-	28.4
**A2M**	Alpha-2-macroglobulin	19485.3	20836.4
**AACS**	Acetoacetyl-CoA synthetase	-	99.9
**ACAN**	Aggrecan core protein	-	22.5
**ACTB**	Actin, cytoplasmic 1	3218.6	1903.3
**ACTG2**	Actin, gamma-enteric smooth muscle	8.4	4.6
**ADAM10**	Disintegrin and metalloproteinase domain-containing pro	-	-
**ADH5**	Alcohol dehydrogenase class-3	-	-
**ADIPOQ**	Adiponectin	-	32.3
**AFM**	Afamin	-	1.5
**AGT**	Angiotensinogen	9.4	16.9
**AHSG**	Alpha-2-HS-glycoprotein	-	27.5
**ALB**	Albumin	2999.0	3176.4
**ALDOA**	Fructose-bisphosphate aldolase A	-	18.5
**AMBP**	Protein AMBP	2.0	1.9
**ANGPTL6**	Angiopoietin-related protein 6	-	-
**ANK1**	Ankyrin-1	-	10.8
**ANPEP**	Aminopeptidase N	36.1	6.2
**ANTXR2**	Anthrax toxin receptor 2	25.2	-
**ANXA11**	Annexin A11	-	0.4
**ANXA2**	Annexin A2	54.9	-
**ANXA5**	Annexin A5	-	-
**ANXA7**	Annexin A7	12.8	12.3
**APCS**	Serum amyloid P-component	8.1	13.0
**APOA1**	Apolipoprotein A-I	17877.9	20061.2
**APOA2**	Apolipoprotein A-II	4348.8	4618.2
**APOA4**	Apolipoprotein A-IV	425.9	1320.3
**APOA5**	Apolipoprotein A-V	18.1	24.3
**APOB**	Apolipoprotein B-100	70343.3	72950.4
**APOC1**	Apolipoprotein C-I	823.9	3841.5
**APOC2**	Apolipoprotein C-II	2007.2	4302.3
**APOC3**	Apolipoprotein C-III	1188.5	8452.1
**APOC4**	Apolipoprotein C-IV	407.3	1147.4
**APOD**	Apolipoprotein D	908.8	2304.2
**APOE**	Apolipoprotein E	14926.7	25453.1
**APOF**	Apolipoprotein F	2.0	23.2
**APOH**	Beta-2-glycoprotein 1	1.9	20.8
**APOL1**	Apolipoprotein L1	1722.5	2251.3
**APOM**	Apolipoprotein M	132.0	433.2
**ARL6IP5**	PRA1 family protein 3	-	-
**ARPC5**	Actin-related protein 2/3 complex subunit 5	-	-
**ATIC**	Bifunctional purine biosynthesis protein ATIC	-	-
**ATP2A3**	Sarcoplasmic/endoplasmic reticulum calcium ATPase 3	194.4	72.9
**ATP5F1B**	ATP synthase subunit beta, mitochondrial	-	-
**ATRN**	Attractin	-	79.0

**B2M**	Beta-2-microglobulin	-	-
**BCAP31**	B-cell receptor-associated protein 31	12.7	-
**BCHE**	Cholinesterase	-	10.5
**C16orf54**	Transmembrane protein C16orf54	16.5	-
**C1QA**	Complement C1q subcomponent subunit A	34245.0	23497.7
**C1QB**	Complement C1q subcomponent subunit B	108371.6	65445.1
**C1QC**	Complement C1q subcomponent subunit C	95482.6	61350.6
**C1R**	Complement C1r subcomponent	1343.3	5603.1
**C1RL**	Complement C1r subcomponent-like protein	-	-
**C1S**	Complement C1s subcomponent	2197.0	6574.8
**C3**	Complement C3	5098.9	3485.3
**C4A**	Complement C4-A	-	-
**C4B**	Complement C4-B	1760.8	1053.8
**C4BPA**	C4b-binding protein alpha chain	2622.4	6717.0
**C4BPB**	C4b-binding protein beta chain	115.2	255.2
**C5**	Complement C5	334.5	339.3
**C9**	Complement component C9	199.2	154.6
**CALM3**	Calmodulin-3	-	-
**CAMP**	Cathelicidin antimicrobial peptide	48.7	211.3
**CANX**	Calnexin	47.8	7.4
**CASP14**	Caspase-14	-	-
**CAVIN2**	Caveolae-associated protein 2	725.3	827.9
**CCL5**	C-C motif chemokine 5	-	-
**CCT2**	T-complex protein 1 subunit beta	-	0.6
**CCT4**	T-complex protein 1 subunit delta	-	-
**CCT6A**	T-complex protein 1 subunit zeta	-	-
**CCT7**	T-complex protein 1 subunit eta	-	-
**CCT8**	T-complex protein 1 subunit theta	-	-
**CD151**	CD151 antigen	51.0	43.1
**CD226**	CD226 antigen	-	0.7
**CD36**	Platelet glycoprotein 4	330.7	226.6
**CD47**	Leukocyte surface antigen CD47	42.7	51.1
**CD53**	Leukocyte surface antigen CD53	-	-
**CD59**	CD59 glycoprotein	-	10.7
**CD5L**	CD5 antigen-like	1081.7	807.6
**CD9**	CD9 antigen	609.7	363.5
**CD99**	CD99 antigen	-	-
**CDC42**	Cell division control protein 42 homolog	105.8	188.3
**CDCA2**	Cell division cycle-associated protein 2	12.6	27.7
**CDSN**	Corneodesmosin	-	-
**CELSR2**	Cadherin EGF LAG seven-pass G-type receptor 2	-	11.0
**CETP**	Cholesteryl ester transfer protein	-	-
**CFB**	Complement factor B	-	-
**CFH**	Complement factor H	510.8	256.2
**CFHR3**	Complement factor H-related protein 3	-	-
**CFL1**	Cofilin-1	-	8.3
**CFP**	Properdin	-	10.6
**CLIC1**	Chloride intracellular channel protein 1	-	-
**CLTA**	Clathrin light chain A	-	-
**CLTCL1**	Clathrin heavy chain 2	-	-

**CLU**	Clusterin	2740.3	3862.8
**CMTM5**	CKLF-like MARVEL transmembrane domain-containing pr	2.9	1.5
**CMTM6**	CKLF-like MARVEL transmembrane domain-containing pr	1.3	1.6
**COL1A1**	Collagen alpha-1(I) chain	95.4	91.1
**COL1A2**	Collagen alpha-2(I) chain	-	11.9
**COL2A1**	Collagen alpha-1(II) chain	50.8	-
**COL6A1**	Collagen alpha-1(VI) chain	-	8.8
**COL6A3**	Collagen alpha-3(VI) chain	-	-
**COL7A1**	Collagen alpha-1(VII) chain	-	-
**COLEC11**	Collectin-11	-	-
**CP**	Ceruloplasmin	160.9	130.4
**CPN1**	Carboxypeptidase N catalytic chain	138.1	186.5
**CPN2**	Carboxypeptidase N subunit 2	1139.9	234.3
**CSDE1**	Cold shock domain-containing protein E1	-	-
**CST6**	Cystatin-M	-	-
**CTSD**	Cathepsin D	-	-
**CYB5R3**	NADH-cytochrome b5 reductase 3	31.0	3.0
**CYBB**	Cytochrome b-245 heavy chain	-	15.0
**DAZAP1**	DAZ-associated protein 1	-	-
**DCD**	Dermcidin	173.5	6.5
**DCDC1**	Doublecortin domain-containing protein 1	-	61.0
**DDX60L**	Probable ATP-dependent RNA helicase DDX60-like	-	-
**DEFA1**	Neutrophil defensin 1	-	-
**DHX9**	ATP-dependent RNA helicase A	-	-
**DPP6**	Dipeptidyl aminopeptidase-like protein 6	-	-
**DSC1**	Desmocollin-1	140.1	-
**DSG1**	Desmoglein-1	11.9	-
**DSP**	Desmoplakin	-	-
**EEF1A1**	Elongation factor 1-alpha 1	-	-
**EEF2**	Elongation factor 2	-	-
**EFEMP1**	EGF-containing fibulin-like extracellular matrix protein 1	-	-
**EIF5A**	Eukaryotic translation initiation factor 5A-1	-	-
**ENDOD1**	Endonuclease domain-containing 1 protein	-	-
**ENO1**	Alpha-enolase	13.2	162.8
**EPB42**	Protein 4.2	-	7.9
**F10**	Coagulation factor X	4.0	-
**F11**	Coagulation factor XI	-	6.7
**F11R**	Junctional adhesion molecule A	43.9	24.7
**F13A1**	Coagulation factor XIII A chain	33.7	236.4
**F13B**	Coagulation factor XIII B chain	221.2	171.3
**F2**	Prothrombin	273.2	203.7
**F5**	Coagulation factor V	528.8	863.3
**F8**	Coagulation factor VIII	-	0.2
**FBLN1**	Fibulin-1	12.4	31.3
**FCER1G**	1 affinity immunoglobulin epsilon receptor subunit gamm	-	116.9
**FCGBP**	IgGFc-binding protein	159206.3	141135.4
**FCN1**	Ficolin-1	-	-
**FCN2**	Ficolin-2	1.9	22.1
**FCN3**	Ficolin-3	4.3	166.1
**FERMT3**	Fermitin family homolog 3	47.4	20.8

**FGA**	Fibrinogen alpha chain	62851.6	141886.6
**FGB**	Fibrinogen beta chain	24473.8	66807.4
**FGG**	Fibrinogen gamma chain	23332.3	72012.9
**FGL1**	Fibrinogen-like protein 1	-	-
**FLG2**	Filaggrin-2	-	-
**FLNA**	Filamin-A	1764.8	1262.1
**FLOT1**	Flotillin-1	-	-
**FN1**	Fibronectin	16140.9	3788.4
**FSCN1**	Fascin	-	-
**G3BP1**	Ras GTPase-activating protein-binding protein 1	-	-
**G6PD**	Glucose-6-phosphate 1-dehydrogenase	-	-
**GALE**	UDP-glucose 4-epimerase	23.3	23.2
**GAPDH**	Glyceraldehyde-3-phosphate dehydrogenase	-	37.0
**GC**	Vitamin D-binding protein	-	-
**GNAI2**	Guanine nucleotide-binding protein G(i) subunit alpha-2	20.2	9.2
**GNAQ**	Guanine nucleotide-binding protein G(q) subunit alpha	-	-
**GNB4**	Guanine nucleotide-binding protein subunit beta-4	-	-
**GNG5**	Guanine nucleotide-binding protein G(I)/G(S)/G(O) subun	6.5	-
**GP1BA**	Platelet glycoprotein Ib alpha chain	248.5	120.4
**GP1BB**	Platelet glycoprotein Ib beta chain	42.1	26.5
**GP9**	Platelet glycoprotein IX	-	37.9
**GSDMA**	Gasdermin-A	-	-
**GSN**	Gelsolin	10511.0	8770.0
**GSR**	Glutathione reductase, mitochondrial	-	-
**GSTP1**	Glutathione S-transferase P	-	-
**GYPA**	Glycophorin-A	-	-
**H2AC20**	Histone H2A type 2-C	2.8	0.9
**H2BC21**	Histone H2B type 2-E	-	16.3
**H3-3A**	Histone H3.3	-	-
**H4C1**	Histone H4	-	3.8
**HBA1**	Hemoglobin subunit alpha	597.7	1046.3
**HBB**	Hemoglobin subunit beta	107.8	277.0
**HBD**	Hemoglobin subunit delta	-	33.0
**HBG1**	Hemoglobin subunit gamma-1	-	9.8
**HGFAC**	Hepatocyte growth factor activator	-	-
**HINT1**	Histidine triad nucleotide-binding protein 1	-	-
**HLA-A**	HLA class I histocompatibility antigen, A alpha chain	40.9	29.3
**HLA-B**	HLA class I histocompatibility antigen, B alpha chain	79.8	64.8
**HMBS**	Porphobilinogen deaminase	-	-
**HNRNPA1L2**	Heterogeneous nuclear ribonucleoprotein A1-like 2	-	-
**HNRNPA2B1**	Heterogeneous nuclear ribonucleoproteins A2/B1	-	-
**HNRNPA3**	Heterogeneous nuclear ribonucleoprotein A3	-	-
**HNRNPC**	Heterogeneous nuclear ribonucleoproteins C1/C2	-	-
**HNRNPF**	Heterogeneous nuclear ribonucleoprotein F	-	-
**HNRNPH1**	Heterogeneous nuclear ribonucleoprotein H	-	-
**HNRNPH3**	Heterogeneous nuclear ribonucleoprotein H3	-	-
**HNRNPU**	Heterogeneous nuclear ribonucleoprotein U	-	-
**HP**	Haptoglobin	7900.9	6490.4
**HPR**	Haptoglobin-related protein	3515.4	5686.2
**HPX**	Hemopexin	1.1	-

**HRG**	Histidine-rich glycoprotein	43.0	101.6
**HRNR**	Hornerin	415.4	172.8
**HSP90AA1**	Heat shock protein HSP 90-alpha	-	-
**HSPA1B**	Heat shock 70 kDa protein 1B	-	-
**HSPA8**	Heat shock cognate 71 kDa protein	-	11.9
**HSPA9**	Stress-70 protein, mitochondrial	-	-
**HSPD1**	60 kDa heat shock protein, mitochondrial	-	-
**ICAM2**	Intercellular adhesion molecule 2	-	-
**ILK**	Integrin-linked protein kinase	39.5	32.5
**INPP5D**	Phosphatidylinositol 3,4,5-trisphosphate 5-phosphatase 1	-	-
**ITGA2**	Integrin alpha-2	33.7	-
**ITGA2B**	Integrin alpha-IIb	1539.2	968.2
**ITGA6**	Integrin alpha-6	114.3	36.6
**ITGB1**	Integrin beta-1	52.0	24.4
**ITGB3**	Integrin beta-3	900.4	597.5
**ITIH1**	Inter-alpha-trypsin inhibitor heavy chain H1	696.3	478.3
**ITIH2**	Inter-alpha-trypsin inhibitor heavy chain H2	1495.8	1547.4
**ITIH3**	Inter-alpha-trypsin inhibitor heavy chain H3	78.9	18.0
**ITIH4**	Inter-alpha-trypsin inhibitor heavy chain H4	292.2	685.6
**JUP**	Junction plakoglobin	-	-
**KANSL3**	KAT8 regulatory NSL complex subunit 3	2389.4	2787.7
**KIAA2026**	Uncharacterized protein KIAA2026	56.6	87.0
**KLKB1**	Plasma kallikrein	-	28.5
**KNG1**	Kininogen-1	120.5	944.9
**KPRP**	Keratinocyte proline-rich protein	-	-
**KRT1**	Keratin, type II cytoskeletal 1	41737.9	13024.7
**KRT10**	Keratin, type I cytoskeletal 10	33047.2	7965.4
**KRT14**	Keratin, type I cytoskeletal 14	1182.4	546.8
**KRT15**	Keratin, type I cytoskeletal 15	-	-
**KRT16**	Keratin, type I cytoskeletal 16	231.5	-
**KRT17**	Keratin, type I cytoskeletal 17	50.8	29.7
**KRT2**	Keratin, type II cytoskeletal 2 epidermal	22468.0	4378.6
**KRT24**	Keratin, type I cytoskeletal 24	-	-
**KRT32**	Keratin, type I cuticular Ha2	-	62.1
**KRT5**	Keratin, type II cytoskeletal 5	3530.4	955.5
**KRT6A**	Keratin, type II cytoskeletal 6A	176.4	24.8
**KRT6B**	Keratin, type II cytoskeletal 6B	-	-
**KRT78**	Keratin, type II cytoskeletal 78	-	-
**KRT85**	Keratin, type II cuticular Hb5	-	104.7
**KRT86**	Keratin, type II cuticular Hb6	-	16.0
**KRT9**	Keratin, type I cytoskeletal 9	15461.4	4975.5
**LAMA2**	Laminin subunit alpha-2	23.2	-
**LBP**	Lipopolysaccharide-binding protein	0.3	4.8
**LDHA**	L-lactate dehydrogenase A chain	-	-
**LDHB**	L-lactate dehydrogenase B chain	11.5	10.7
**LGALS3BP**	Galectin-3-binding protein	1413.7	2284.5
**LPA**	Apolipoprotein(a)	8396.4	2081.8
**LRBA**	Lipopolysaccharide-responsive and beige-like anchor pro	-	-
**LRP1**	Prolow-density lipoprotein receptor-related protein 1	-	10.5
**LTBP1**	Latent-transforming growth factor beta-binding protein 1	-	0.9

**LTF**	Lactotransferrin	-	-
**LYZ**	Lysozyme C	-	-
**M6PR**	Cation-dependent mannose-6-phosphate receptor	-	-
**MARCO**	Macrophage receptor MARCO	-	22.0
**MASP1**	Mannan-binding lectin serine protease 1	45.7	80.1
**MASP2**	Mannan-binding lectin serine protease 2	-	5.2
**MATR3**	Matrin-3	-	-
**MBL2**	Mannose-binding protein C	-	15.5
**MENT**	Protein MENT	-	160.6
**MMRN1**	Multimerin-1	146.7	107.3
**MSN**	Moesin	71.6	32.9
**MXRA5**	Matrix-remodeling-associated protein 5	-	-
**MYADM**	Myeloid-associated differentiation marker	-	-
**MYH16**	Putative uncharacterized protein MYH16	-	3.0
**MYH9**	Myosin-9	3820.8	2320.0
**MYL12B**	Myosin regulatory light chain 12B	24.6	111.4
**MYL6**	Myosin light polypeptide 6	9.1	80.8
**NANS**	Sialic acid synthase	-	-
**NASP**	Nuclear autoantigenic sperm protein	-	-
**NCL**	Nucleolin	-	-
**NPM1**	Nucleophosmin	-	-
**NR0B1**	Nuclear receptor subfamily 0 group B member 1	688.0	452.6
**ORM1**	Alpha-1-acid glycoprotein 1	5.8	6.3
**ORM2**	Alpha-1-acid glycoprotein 2	16.4	10.9
**OSBPL2**	Oxysterol-binding protein-related protein 2	-	44.1
**PATJ**	InaD-like protein	-	11.5
**PCBP1**	Poly(rC)-binding protein 1	-	-
**PCDHGB7**	Protocadherin gamma-B7	-	-
**PCYOX1**	Prenylcysteine oxidase 1	25077.4	26075.0
**PECAM1**	Platelet endothelial cell adhesion molecule	-	8.7
**PF4V1**	Platelet factor 4 variant	-	18.6
**PFN1**	Profilin-1	70.3	39.3
**PGK1**	Phosphoglycerate kinase 1	-	-
**PHB**	Prohibitin	-	-
**PHLDB2**	Pleckstrin homology-like domain family B member 2	486.9	430.2
**PIGR**	Polymeric immunoglobulin receptor	83.4	98.6
**PKD2L1**	Polycystic kidney disease 2-like 1 protein	6103.5	3497.0
**PKM**	Pyruvate kinase PKM	-	-
**PLA2G7**	Platelet-activating factor acetylhydrolase	-	0.8
**PLEK**	Pleckstrin	-	-
**PLG**	Plasminogen	-	-
**PLTP**	Phospholipid transfer protein	-	-
**PNPLA8**	Calcium-independent phospholipase A2-gamma	-	-
**PON1**	Serum paraoxonase/arylesterase 1	51.9	144.7
**PPARG**	Peroxisome proliferator-activated receptor gamma	-	-
**PPBP**	Platelet basic protein	1.6	2.1
**PPIA**	Peptidyl-prolyl cis-trans isomerase A	85.6	190.0
**PRDX1**	Peroxiredoxin-1	-	-
**PRDX2**	Peroxiredoxin-2	-	-
**PRDX6**	Peroxiredoxin-6	-	-

**PRG4**	Proteoglycan 4	1504.9	790.8
**PROS1**	Vitamin K-dependent protein S	555.6	656.4
**PRSS1**	Trypsin-1	20.7	457.3
**PSMA5**	Proteasome subunit alpha type-5	-	-
**PSMA6**	Proteasome subunit alpha type-6	-	-
**PTBP1**	Polypyrimidine tract-binding protein 1	-	-
**PTGS1**	Prostaglandin G/H synthase 1	-	-
**PTPRJ**	Receptor-type tyrosine-protein phosphatase eta	15.8	-
**PZP**	Pregnancy zone protein	29.3	100.2
**QSOX1**	Sulfhydryl oxidase 1	-	-
**RAB10**	Ras-related protein Rab-10	44.4	21.1
**RAB1B**	Ras-related protein Rab-1B	12.0	12.1
**RAB27B**	Ras-related protein Rab-27B	-	-
**RAB33B**	Ras-related protein Rab-33B	-	-
**RAC2**	Ras-related C3 botulinum toxin substrate 2	127.5	132.0
**RAP1B**	Ras-related protein Rap-1b	388.3	210.3
**RCC2**	Protein RCC2	-	-
**RELN**	Reelin	58.1	40.1
**RHOA**	Transforming protein RhoA	7.7	-
**RPL11**	60S ribosomal protein L11	-	-
**RPL8**	60S ribosomal protein L8	-	-
**RPLP0**	60S acidic ribosomal protein P0	-	-
**RPS15**	40S ribosomal protein S15	-	-
**RPS15A**	40S ribosomal protein S15a	-	-
**RPS23**	40S ribosomal protein S23	-	-
**RPS3A**	40S ribosomal protein S3a	-	-
**RPSA**	40S ribosomal protein SA	-	-
**RTN3**	Reticulon-3	21.4	6.9
**RTN4**	Reticulon-4	46.8	28.4
**S100A8**	Protein S100-A8	-	-
**S100A9**	Protein S100-A9	-	-
**SAA1**	Serum amyloid A-1 protein	152.1	146.4
**SAA4**	Serum amyloid A-4 protein	952.7	1339.6
**SACM1L**	Phosphatidylinositol-3-phosphatase SAC1	-	-
**SBSN**	Suprabasin	-	-
**SELENOP**	Selenoprotein P	43.7	45.3
**SELPLG**	P-selectin glycoprotein ligand 1	-	-
**SERPINA1**	Alpha-1-antitrypsin	3706.7	2716.8
**SERPINA3**	Alpha-1-antichymotrypsin	239.8	392.3
**SERPINA4**	Kallistatin	0.2	-
**SERPINC1**	Antithrombin-III	188.6	370.8
**SERPIND1**	Heparin cofactor 2	27.4	68.2
**SERPINF2**	Alpha-2-antiplasmin	1733.5	1047.1
**SERPING1**	Plasma protease C1 inhibitor	307.1	807.1
**SF3A3**	Splicing factor 3A subunit 3	-	-
**SH3BGRL3**	SH3 domain-binding glutamic acid-rich-like protein 3	-	-
**SHBG**	Sex hormone-binding globulin	15.7	18.6
**SIGLEC16**	Sialic acid-binding Ig-like lectin 16	2299.9	5296.3
**SLC2A1**	Solute carrier family 2, facilitated glucose transporter me	0.3	0.2
**SLC2A3**	Solute carrier family 2, facilitated glucose transporter me	61.2	43.7

**SLC4A1**	Band 3 anion transport protein	102.8	97.5
**SNX14**	Sorting nexin-14	-	128.9
**SOD1**	Superoxide dismutase [Cu-Zn]	-	-
**SPATA3**	Spermatogenesis-associated protein 3	84.3	52.5
**SPATA48**	Spermatogenesis-associated protein 48	29.7	432.2
**SPN**	Leukosialin	-	-
**SPTA1**	Spectrin alpha chain, erythrocytic 1	-	-
**SPTB**	Spectrin beta chain, erythrocytic	60.8	71.0
**SPTBN1**	Spectrin beta chain, non-erythrocytic 1	-	-
**SPTLC3**	Serine palmitoyltransferase 3	5955.2	12507.0
**SRGN**	Serglycin	2.1	-
**SRI**	Sorcin	-	30.3
**SSC5D**	Soluble scavenger receptor cysteine-rich domain-contain	23.4	738.5
**STEAP3**	Metalloreductase STEAP3	-	-
**STOM**	Stomatin	183.8	129.7
**SVEP1**	Sushi, von Willebrand factor type A, EGF and pentraxin d	-	130.4
**SYNCRIP**	Heterogeneous nuclear ribonucleoprotein Q	-	-
**TAGLN2**	Transgelin-2	-	7.9
**TCP1**	T-complex protein 1 subunit alpha	-	-
**TF**	Serotransferrin	3746.6	1053.5
**TFRC**	Transferrin receptor protein 1	51.2	36.5
**TGFBI**	Transforming growth factor-beta-induced protein ig-h3	15.7	26.8
**TGM3**	Protein-glutamine gamma-glutamyltransferase E	-	24.2
**THBS1**	Thrombospondin-1	471.1	294.5
**THBS4**	Thrombospondin-4	-	168.9
**TKT**	Transketolase	-	-
**TLN1**	Talin-1	3878.5	2967.5
**TM4SF1**	Transmembrane 4 L6 family member 1	-	-
**TMEM109**	Transmembrane protein 109	0.8	-
**TMEM198**	Transmembrane protein 198	-	-
**TMX2**	Thioredoxin-related transmembrane protein 2	0.8	-
**TNC**	Tenascin	280.4	249.5
**TNKS1BP1**	182 kDa tankyrase-1-binding protein	586.7	364.5
**TPM4**	Tropomyosin alpha-4 chain	-	-
**TSPAN14**	Tetraspanin-14	-	-
**TTLL12**	Tubulin--tyrosine ligase-like protein 12	-	-
**TTR**	Transthyretin	15.4	1.7
**TUBA1C**	Tubulin alpha-1C chain	2.7	24.3
**TUBA4A**	Tubulin alpha-4A chain	21.7	8.4
**TUBB**	Tubulin beta chain	-	-
**TUBB1**	Tubulin beta-1 chain	41.0	22.9
**UBA52**	Ubiquitin-60S ribosomal protein L40	-	-
**UBE2L3**	Ubiquitin-conjugating enzyme E2 L3	-	-
**UBQLN1**	Ubiquilin-1	-	-
**VCAN**	Versican core protein	-	-
**VCL**	Vinculin	129.4	127.7
**VDAC2**	Voltage-dependent anion-selective channel protein 2	-	-
**VIM**	Vimentin	-	-
**VTN**	Vitronectin	631.1	611.4
**VWF**	von Willebrand factor	8300.6	2812.8

**WBP11**	WW domain-binding protein 11	158.7	18.7
**XRCC5**	X-ray repair cross-complementing protein 5	-	-
**YWHAB**	14-3-3 protein beta/alpha	28.8	19.4
**YWHAE**	14-3-3 protein epsilon	29.1	-
**YWHAG**	14-3-3 protein gamma	27.7	21.8
**YWHAH**	14-3-3 protein eta	213.5	85.5
**YWHAZ**	14-3-3 protein zeta/delta	1625.9	1048.7

MP_3	MP_4	MP_5	MP_6	MP_7	MP_8	MP_9	MP_10
-	-	-	9.6	-	-	-	-
17405.3	7376.3	1277.0	15169.2	3303.9	4986.6	3162.5	3117.3
70.0	28.4	-	13.2	173.8	212.3	45.1	494.0
-	-	-	-	1.7	-	-	13.3
1159.4	3181.1	3099.8	2835.1	2197.7	2226.1	2909.9	211.8
3.5	-	-	-	3.4	-	23.6	-
-	-	-	-	7.4	11.3	-	-
-	-	-	-	-	-	-	-
18.1	-	32.2	-	-	15.3	-	-
-	-	-	1.2	-	1.8	-	-
28.2	4.3	-	116.0	37.3	10.8	54.1	28.2
22.2	7.4	3.5	-	3.8	-	3.0	1.7
3122.6	1007.1	1471.6	43074.3	1393.0	4612.4	1428.8	1607.1
29.2	0.2	1.9	42.8	10.6	13.3	19.6	8.5
1.0	8.1	181.3	4.9	3.3	19.5	44.8	4.4
-	-	-	-	22.1	7.5	6.6	15.5
-	-	-	9.9	17.4	3.0	4.9	-
15.1	49.4	-	49.5	3.9	16.5	28.4	-
-	-	5.5	-	12.4	10.9	8.0	4.2
-	1.6	-	-	-	5.4	1.3	-
10.7	-	-	16.3	-	-	14.6	6.5
-	-	12.5	16.7	23.8	2.6	7.4	-
25.0	13.4	55.4	205.1	20.3	42.3	50.2	-
7.0	-	2.0	41.4	31.1	11.4	6.0	8.4
24884.5	2737.7	7246.7	10116.8	17580.2	36756.8	15764.2	19318.4
4347.6	13593.0	1839.9	4379.2	1459.6	9113.5	11808.2	3780.1
298.0	759.9	19.2	542.5	110.4	2129.0	1599.5	272.3
10.0	-	-	33.4	4.3	-	19.3	5.2
53053.9	42554.6	379981.2	130375.2	360745.5	88187.7	48516.8	125451.5
384.8	109.2	48.0	707.5	214.6	645.0	1505.1	327.3
6723.3	493.1	8168.1	8924.4	1252.9	2141.9	3577.6	3041.7
1860.8	220.9	4688.5	1629.9	499.4	2246.1	3661.8	1658.0
1089.0	135.4	-	103.4	326.3	807.8	1238.2	582.3
430.9	402.1	5.2	1094.0	951.8	1099.0	949.9	1300.2
11304.6	2238.4	14699.4	21644.8	8008.2	17085.6	13305.7	10857.2
27.5	6.9	4.0	51.8	7.1	21.8	9.8	13.5
-	-	-	57.8	14.8	13.1	18.8	-
4873.4	5271.2	4427.9	4835.3	3316.8	11671.6	9587.3	3320.0
427.4	94.7	98.4	131.4	188.8	478.4	541.0	230.1
-	-	-	-	0.1	-	-	-
-	13.5	-	-	32.8	13.3	8.2	8.6
-	-	-	-	-	-	-	-
469.8	270.6	69.1	313.5	242.9	186.6	293.3	20.3
-	-	-	-	-	-	-	-
-	58.0	-	-	17.9	40.4	58.2	-

-	30.3	2.2	-	9.7	30.5	34.4	-
-	12.2	-	-	19.5	11.3	5.8	-
3.1	61.7	2.8	53.8	4.3	45.9	27.2	2.7
-	-	13.8	10.9	-	-	-	-
23267.8	33421.6	2896.4	2757.5	2057.1	7093.5	9988.0	2820.4
60735.5	89126.8	39690.0	27137.0	7014.9	24870.3	29119.4	10360.6
55779.7	92491.9	75728.8	16901.1	5157.3	16348.6	14658.7	7381.8
4965.9	4448.7	6039.8	8024.9	1698.7	5007.3	3360.0	2277.6
-	-	-	-	-	-	-	-
4404.6	5057.6	3513.8	5623.1	4762.8	9601.7	5920.7	5186.3
2737.8	820.7	2558.8	4445.1	4074.4	2832.4	2133.2	2971.7
-	-	-	-	2.3	-	-	1.4
653.3	1070.4	5671.3	1683.5	1311.4	1056.1	979.0	1132.7
1370.0	458.6	1036.7	5496.3	3025.3	6767.3	1168.1	1869.7
100.7	88.8	-	173.9	133.9	349.3	110.2	75.0
179.3	-	19.4	335.3	60.6	12.5	5.1	84.6
632.1	22.0	58.7	780.1	114.6	67.4	0.5	199.5
-	-	-	-	-	-	-	-
135.6	344.2	8.4	73.4	69.1	3494.7	2956.9	69.0
-	44.4	361.9	48.3	18.2	26.6	-	3.9
-	19.0	-	-	-	12.5	18.4	-
539.4	417.1	376.7	537.9	542.7	1070.9	611.1	77.9
-	-	-	-	-	-	-	-
0.4	0.1	-	4.2	14.6	-	-	0.4
-	-	-	-	-	-	-	-
-	-	-	-	-	-	-	-
-	-	-	-	-	-	-	-
0.3	-	-	-	-	-	-	-
37.4	54.5	29.6	64.8	42.0	20.7	14.3	24.5
-	2.3	48.6	-	1.8	0.6	-	-
79.4	266.0	624.0	175.2	408.7	260.4	356.0	26.8
5.4	92.4	3.4	20.6	37.7	12.3	2.7	-
-	-	2.3	-	-	-	-	-
-	16.2	-	36.0	6.1	16.8	5.1	-
2557.8	676.1	495.8	420.7	397.9	1310.2	997.9	488.7
88.3	555.9	-	180.3	402.2	670.4	514.9	44.2
-	-	-	25.2	-	-	-	-
275.3	35.9	17.4	214.9	182.7	25.6	4.9	304.6
34.2	-	-	39.3	39.4	48.7	52.0	112.3
-	-	-	-	-	3.0	6.9	1.8
9.6	8.5	-	-	183.7	12.8	2.3	189.1
-	-	1.9	15.5	6.2	-	2.3	-
-	-	2.1	-	-	15.5	8.4	-
673.5	407.7	63.6	378.2	87.6	195.7	121.1	129.8
-	17.6	2.0	33.8	11.1	11.3	22.0	5.5
-	12.5	-	10.0	-	5.6	8.3	5.1
5.3	-	-	15.6	5.3	-	-	9.3
-	6.7	-	-	-	5.9	12.8	-
-	11.3	-	-	3.8	19.2	24.7	-
-	-	-	-	17.7	-	-	2.5

2045.7	749.9	4149.2	4858.2	1912.0	2775.7	4039.5	2015.5
-	-	161.0	1.4	15.9	-	-	-
1.8	10.4	-	2.0	-	3.1	10.8	-
240.2	34.9	389.1	149.4	49.3	33.1	26.0	99.0
15.3	8.4	1.8	-	19.3	-	2.2	19.8
-	-	-	-	-	-	-	-
-	-	-	-	19.2	15.4	1.2	13.4
-	13.6	-	10.1	27.3	9.9	-	14.9
-	-	-	-	-	10.5	-	-
-	-	-	-	0.9	-	-	0.4
92.8	283.0	8.7	34.1	43.3	99.9	253.2	32.4
184.4	10.5	-	186.9	30.1	6.7	10.1	47.6
384.9	41637.0	2.5	735.4	75.8	16892.6	29666.9	110.2
-	-	-	-	-	-	-	-
-	-	-	-	-	-	-	-
-	-	-	-	-	-	-	-
-	8.2	-	5.3	67.3	13.4	15.1	-
-	-	-	-	-	-	-	-
-	-	-	-	-	-	-	-
56.2	-	-	83.9	-	-	44.4	53.9
40.5	27.8	-	-	69.5	78.7	85.3	207.5
-	-	-	-	55.0	-	-	10.2
-	-	-	-	-	-	-	-
-	-	-	-	-	-	-	-
-	-	-	-	2.5	-	-	3.6
345.4	22.1	-	7.6	55.4	10.4	36.1	826.3
-	-	27.8	-	2.2	7.8	3.5	-
-	-	-	-	-	9.2	20.2	-
-	-	-	-	-	-	-	-
-	-	-	-	-	-	-	-
-	-	-	-	-	-	-	-
-	-	-	-	0.3	-	-	0.1
-	11.3	-	-	-	-	6.6	-
9.1	0.8	-	47.1	1.9	1.4	2.8	1.4
-	-	-	-	-	-	-	-
-	-	4.0	-	-	3.0	4.4	-
-	-	-	4.6	19.9	4.6	-	3.2
1.6	99.2	131.1	26.9	37.1	12.2	2.0	-
152.3	154.4	5.7	59.0	70.2	26.2	30.7	17.9
407.3	125.3	-	9.7	84.7	341.6	208.9	115.7
148.5	678.0	28.9	175.8	214.4	460.6	101.2	201.1
174.8	183.7	259.9	1752.3	1024.1	407.5	164.5	767.3
-	-	46.2	-	4.0	-	-	1.1
13.4	24.3	46.1	4.1	41.8	33.8	8.7	20.1
17.6	118.2	1.6	95.3	89.9	44.2	78.5	4.6
26442.1	85551.0	59959.6	37008.0	4140.0	39757.4	33153.7	9911.4
-	-	-	58.4	-	14.0	11.7	-
16.5	8.3	-	11.4	1.1	2.1	7.7	2.0
195.9	5.2	30.2	274.9	48.9	68.3	145.9	86.0
18.8	26.7	-	43.5	122.0	31.3	35.8	10.4

201939.7	52664.5	93438.9	189527.5	176311.7	149208.5	141624.8	301647.2
91940.2	21296.8	37820.3	66047.4	71121.5	69145.1	52395.4	115197.8
94478.8	22112.8	41461.2	50730.8	90607.0	69817.1	48982.2	120712.5
-	-	-	-	-	4.3	1.6	-
-	-	-	-	-	-	12.0	-
925.2	2137.0	2061.7	1232.4	2379.3	1364.5	878.9	300.2
-	-	-	-	1.3	-	-	-
5545.2	6029.9	1732.2	18313.6	22156.2	6073.6	2326.4	27657.7
-	-	-	-	-	-	-	-
-	-	-	-	-	-	-	-
-	-	-	-	0.2	-	-	-
20.7	6.7	-	98.8	9.7	41.9	11.5	8.3
-	13.1	15.3	79.9	47.2	23.5	53.1	12.5
-	7.2	18.7	89.0	35.6	9.9	7.4	6.2
-	26.7	-	6.0	27.8	13.3	6.4	-
-	17.7	-	-	25.0	16.0	12.7	-
-	55.6	-	-	-	43.2	36.7	-
-	13.5	-	11.9	-	34.0	23.2	-
103.5	293.2	154.3	129.1	336.4	151.5	192.5	24.9
12.3	108.7	542.4	4.2	55.7	67.3	63.3	3.4
-	271.9	-	-	123.2	83.6	59.7	-
-	-	-	-	-	-	-	-
8385.7	139.1	201.1	4015.3	2257.4	356.1	236.3	3809.7
-	-	-	-	-	-	-	-
-	-	-	-	-	-	-	-
-	-	-	-	-	-	-	-
0.4	7.8	-	6.8	1.6	3.4	4.4	0.3
18.7	3.4	139.1	66.8	11.6	-	2.1	-
-	-	13.4	4.2	-	-	-	-
3.5	18.2	5.4	-	2.8	13.2	25.3	5.0
1997.0	918.9	72.6	1775.4	1061.6	1173.9	1245.7	1284.8
342.9	560.3	5.3	1004.0	390.7	126.7	339.1	72.0
-	-	-	61.9	5.3	0.5	8.4	-
-	-	-	-	-	-	7.4	-
-	-	-	-	-	-	4.9	1.7
-	1.9	-	-	-	-	-	-
24.1	39.9	214.8	79.4	124.0	88.7	142.0	1.2
63.1	168.9	47.4	-	94.2	66.5	83.2	14.4
-	0.7	-	-	-	1.7	-	0.6
-	-	-	-	-	-	-	-
-	-	-	-	-	-	-	-
-	-	-	-	-	-	-	-
-	-	-	-	-	-	-	-
-	-	-	-	-	-	-	-
-	-	-	-	3.3	-	3.5	-
-	-	-	-	-	-	-	-
-	-	-	-	-	-	-	-
7506.7	2227.3	7666.6	10683.4	2106.9	4314.2	2343.6	2669.5
10348.2	2972.4	1945.1	7643.8	5906.0	6814.2	3187.7	10972.5
5.2	8.2	-	32.2	1.0	10.2	0.7	3.2

1.3	51.4	124.6	62.7	177.3	348.5	86.0	51.7
388.8	179.1	-	100.5	68.7	236.7	379.5	200.0
-	-	-	1.9	-	-	-	-
-	-	-	-	-	-	-	-
5.0	-	-	-	2.8	-	13.3	-
-	-	7.3	-	2.0	-	-	-
-	-	-	4.9	-	-	-	0.3
-	-	1.7	4.6	-	-	16.9	-
27.3	0.5	99.9	41.3	33.6	10.5	20.4	5.9
4.6	-	-	-	-	-	-	-
9.3	-	-	10.6	-	-	-	-
531.4	1876.1	1142.7	580.3	1975.0	1069.1	1005.2	114.3
8.9	65.3	34.7	38.1	60.9	43.3	43.4	1.8
4.7	127.9	7.1	33.5	88.4	84.9	55.7	12.5
387.6	1095.7	94.4	363.0	1487.5	510.9	356.7	54.1
190.4	52.8	199.7	1525.0	764.7	272.8	297.0	662.4
766.8	587.9	345.6	2569.1	1153.4	2214.1	728.8	1452.3
15.4	-	70.8	112.2	31.9	2.7	8.7	17.6
67.9	492.0	448.9	698.1	709.0	1121.6	1087.9	600.0
-	-	-	16.5	-	-	-	-
1938.5	-	6.3	161.6	212.1	6.1	3.5	347.5
47.3	55.9	-	39.7	440.1	135.9	57.6	151.5
17.8	2948.7	7.5	11.6	8.3	1255.9	1373.2	12.4
495.3	106.8	698.7	262.3	865.7	383.0	200.3	510.1
-	-	-	-	-	9.7	23.1	-
37847.4	20425.2	11351.7	35715.2	4220.1	15030.8	25831.9	16609.5
23214.7	10400.8	5190.8	20139.4	1466.7	8418.0	19205.0	10979.0
1339.8	793.3	148.3	1675.9	64.4	1527.4	1801.6	278.9
-	-	-	-	-	-	48.3	-
323.3	56.4	-	89.6	-	29.6	57.6	-
49.9	-	-	48.8	-	-	-	-
10846.3	5372.3	3992.5	9620.0	666.0	4975.6	10838.9	5270.5
-	-	-	-	-	-	-	-
-	-	-	-	-	-	-	-
2807.9	967.8	120.8	2571.8	197.5	1053.8	2296.4	689.2
508.6	104.1	1.2	72.6	19.4	42.0	92.2	49.3
-	-	-	-	-	9.6	21.4	-
-	10.0	-	-	-	-	40.5	-
36.4	-	-	-	-	48.8	117.2	6.6
-	-	-	-	-	-	-	-
15185.7	9790.6	2061.7	18184.9	1581.4	6954.0	14892.4	6582.1
8.4	-	-	23.9	-	-	-	5.2
3.9	2.4	-	11.5	5.0	3.6	2.9	17.7
-	-	-	-	-	8.5	-	-
-	-	3.2	-	-	13.5	6.6	-
2457.5	154.1	1339.0	3023.0	1103.0	3443.7	1546.5	890.6
803.4	686.5	33368.8	20299.5	13988.7	5932.6	401.0	7733.6
-	-	-	-	-	-	-	-
-	-	10.4	145.9	2.4	3.4	11.9	12.5
-	-	-	17.4	7.4	1.7	-	2.0

-	3.4	-	-	-	-	-	-
-	-	-	-	-	-	-	-
16.3	-	-	-	-	8.4	-	-
-	-	-	-	14.4	7.3	9.9	3.6
44.6	104.1	122.6	35.5	113.2	138.9	52.3	50.6
24.7	5.6	-	2.5	9.2	8.5	9.1	9.9
-	-	-	-	-	-	-	-
14.7	9.2	-	52.8	14.2	90.2	33.6	9.5
-	-	0.9	-	42.2	4.8	8.8	123.0
44.4	99.3	60.2	147.2	265.2	66.7	46.8	44.8
35.6	9.5	9.8	24.1	34.1	52.4	32.1	16.5
-	14.6	-	-	-	25.8	17.7	-
-	-	-	-	-	1.7	-	-
0.2	2.2	-	2.7	1.0	1.0	-	0.3
2017.4	1834.3	1439.0	1546.1	4886.5	3918.0	1135.6	531.2
10.7	135.2	-	402.4	119.2	369.1	238.5	45.4
14.6	16.2	17.5	29.2	203.7	230.7	194.5	43.9
-	-	-	-	-	-	-	-
-	-	-	-	-	-	-	-
-	-	-	-	-	-	-	-
-	-	-	-	-	-	-	-
857.0	309.0	232.7	389.3	304.5	849.6	580.6	213.6
-	-	-	-	-	-	-	-
7.4	23.0	-	-	37.0	65.6	16.6	4.4
-	-	-	-	49.6	-	-	-
7.9	-	-	6.8	4.1	-	-	1.9
-	-	-	-	-	-	-	-
-	-	492.0	-	-	-	-	-
34274.1	30.6	54.7	85.8	3627.7	75.0	40.9	5254.8
-	83.3	6.8	-	27.4	54.1	23.7	-
-	-	-	-	15.5	15.0	18.6	-
49.9	-	-	7.4	61.6	30.5	17.5	3.6
-	-	-	-	-	-	-	-
-	-	-	-	0.1	-	-	-
598.6	126.8	-	187.7	454.1	1554.2	771.6	472.7
86.3	98.1	12.6	555.9	45.6	28.6	54.5	16.7
2186.4	1749.5	3696.4	4441.7	251.4	3621.9	1918.2	529.2
-	-	-	-	-	-	-	-
-	-	-	-	22.8	-	-	9.7
-	-	-	-	11.3	-	-	-
-	-	-	-	-	-	-	-
-	-	-	29.2	0.2	-	-	-
11.8	-	-	-	2.3	20.7	-	3.0
227.9	151.9	238.8	284.5	374.7	336.4	283.8	234.9
-	-	-	-	-	-	-	-
3.4	7.8	-	-	10.9	13.6	16.2	-
102.7	66.3	244.9	144.4	94.7	38.7	130.1	99.8
-	12.0	-	-	-	-	13.9	-
-	-	-	-	0.2	-	-	-
-	-	-	-	-	-	-	-

701.1	365.7	1119.1	2660.0	2224.7	678.1	737.2	1507.0
175.0	97.8	-	368.0	424.3	1096.3	100.0	198.8
162.9	544.9	-	3.7	261.7	303.0	95.9	309.8
-	-	-	-	-	-	-	-
-	-	-	-	-	-	-	-
-	-	-	-	-	-	-	-
-	-	-	-	12.5	6.9	-	-
-	11.7	-	3.0	7.4	3.6	24.2	-
27.9	30.5	21.0	118.7	268.9	0.3	31.3	14.0
-	-	-	-	-	-	-	-
-	33.8	-	-	63.8	25.6	35.7	-
17.6	61.1	-	-	3.8	42.1	18.4	0.2
-	-	-	-	-	0.1	-	-
-	-	-	-	-	6.3	5.4	-
19.3	24.6	-	53.1	24.1	17.6	31.5	13.9
135.0	443.2	1060.5	91.9	245.1	326.3	182.3	20.5
-	-	-	-	-	-	-	-
23.8	20.5	-	131.3	271.8	37.2	-	63.0
-	-	-	-	5.3	-	-	-
1.4	-	-	-	-	-	-	-
-	-	-	-	-	-	-	-
-	-	-	-	-	-	-	-
-	-	-	-	-	-	-	-
-	-	-	-	-	0.6	-	-
-	-	-	-	-	-	-	-
-	-	-	-	-	-	-	-
-	-	-	-	-	-	-	-
-	41.5	-	6.4	62.4	32.8	30.5	6.0
-	66.0	3.2	-	100.9	44.1	15.0	-
-	17.7	-	-	-	9.0	6.5	-
-	-	-	-	-	-	-	-
76.4	82.9	33.7	479.9	74.2	59.3	7.1	139.4
2757.0	457.7	2597.3	7199.1	2204.1	1331.0	4009.6	1585.9
8.6	-	-	-	-	-	-	-
-	-	-	-	-	-	20.4	-
25.4	-	-	27.1	37.3	28.9	40.0	-
-	-	-	-	6.5	-	-	1.6
2637.3	501.4	914.1	5180.5	2273.0	1794.4	889.1	1279.9
201.1	-	-	249.7	279.0	9.6	8.0	216.9
0.1	-	-	0.5	-	-	-	-
343.2	293.8	10.3	553.2	129.2	299.1	286.9	198.5
62.5	199.9	41.0	95.0	38.7	58.6	20.4	61.6
78.6	294089.8	224.3	1404.8	206.2	181438.7	283138.7	147.1
498.9	960.3	2792.6	1091.9	470.8	1047.9	1599.0	939.3
-	-	-	-	-	-	-	-
-	-	-	-	-	-	17.8	-
8.9	-	3.5	-	3.3	-	-	-
8007.7	2182.0	840.6	1278.6	14332.8	7279.6	6317.5	12042.0
5.8	0.2	-	31.1	0.5	8.9	4.8	-
4.8	34.0	1.3	64.9	200.4	12.2	4.7	12.4

128.3	88.0	6.0	264.6	38.0	34.2	124.3	31.0
40.5	-	-	171.7	9.0	-	-	12.0
-	-	-	-	-	-	-	-
35.7	49.8	-	22.1	2.0	-	10.4	-
277.5	345.6	6.4	25.5	40.5	-	125.6	76.6
-	7.7	-	-	-	4.5	6.8	-
-	-	8.2	23.6	-	41.2	-	-
61.4	-	15.7	137.8	41.4	-	-	80.5
-	-	-	-	-	-	-	-
16316.6	3598.0	16805.7	33429.6	17564.5	15366.9	17351.5	28526.4
-	10.0	-	-	-	2.9	3.0	-
-	36.6	-	29.5	12.6	20.7	16.5	-
111.6	611.4	1.4	303.8	78.5	500.7	141.9	32.4
-	-	-	-	-	-	-	1.8
106.9	651.4	253.4	292.3	496.9	210.2	682.1	34.3
-	11.9	59.2	379.5	231.2	5.8	-	67.5
-	1.8	-	-	-	-	-	0.4
-	-	-	-	5.5	6.7	11.5	-
-	-	-	-	5.7	-	-	-
904.7	79.3	26.1	3714.6	545.3	67.2	112.1	205.9
4.4	20.7	-	9.8	6.2	12.2	28.6	-
-	-	2.4	11.4	5.0	5.5	24.2	6.1
-	-	4.2	32.8	6.1	4.8	-	9.8
211.7	774.5	158.3	837.8	1297.8	565.6	132.7	166.3
5.7	6.8	7.4	11.2	-	9.1	-	-
-	-	-	-	-	-	-	-
732.9	233.5	1816.8	2130.8	929.4	865.6	521.8	561.3
-	-	-	-	7.4	4.3	-	-
-	9.9	-	-	-	-	2.7	-
-	-	-	18.0	-	-	-	-
1.3	-	-	-	-	-	-	-
283.2	71.7	19.4	325.0	572.0	768.9	137.4	309.3
297.6	805.7	-	245.8	-	128.4	58.8	58.1
-	-	-	-	-	13.4	14.2	-
-	-	-	-	-	-	-	-
4.3	-	-	-	6.2	4.1	-	4.2
-	8.9	-	2.5	0.2	0.5	-	-
39.3	5.2	177.2	136.8	313.3	40.7	11.7	97.1
5.0	34.5	-	2.2	24.9	45.3	22.6	2.9
-	-	-	-	-	-	0.5	-
32.5	20.9	1.2	11.9	165.7	58.8	34.2	8.6
-	-	-	1.2	-	-	-	-
-	-	-	-	-	-	-	-
-	-	-	-	-	-	-	-
-	-	4.7	-	3.5	13.3	-	2.0
118.2	65.4	170.6	127.4	142.4	113.4	159.1	21.5
-	-	-	-	-	-	-	-
-	1.8	-	-	-	-	0.1	-
650.6	588.5	-	1266.9	495.8	714.6	866.0	368.3
1370.5	970.6	5577.5	13059.4	16417.0	4583.3	1044.5	4058.8

54.7	43.2	-	191.2	209.4	48.6	14.9	234.9
-	-	-	-	-	-	-	-
-	38.2	-	-	28.6	31.4	10.4	-
15.2	11.9	-	22.3	39.9	9.2	10.3	-
-	129.0	175.6	37.9	32.7	290.3	302.6	-
48.2	158.1	18.4	137.6	145.9	101.4	104.8	72.1
638.7	1562.8	3655.7	827.4	1209.9	1017.1	1025.7	150.8

HD_1	HD_2	HD_3	HD_4	HD_5	HD_6	HD_7	HD_8
-	-	24.2	44.8	11.1	119.6	-	-
7796.1	3717.9	4605.5	5661.4	3086.4	8210.7	3740.6	5499.4
49.0	-	-	-	-	-	18.7	-
-	-	-	-	-	-	-	3.7
326.6	103.0	841.7	3406.7	552.1	124.1	443.1	1176.6
3.0	4.5	2.7	10.6	0.6	-	0.6	104.8
-	-	-	-	-	-	-	-
-	-	12.9	67.8	-	-	-	-
-	-	-	-	18.3	-	8.4	-
-	-	6.5	35.8	12.6	4.4	0.3	-
15.0	2.7	31.0	6.6	17.9	-	9.2	41.8
10.0	-	9.0	-	11.9	-	-	1.2
3053.8	969.2	2148.3	7457.0	2677.3	2893.7	4308.7	2033.7
14.3	0.1	298.5	1685.6	45.9	313.7	46.8	21.4
21.6	3.7	15.2	7.0	3.6	4.5	26.2	27.2
-	-	-	-	22.8	29.6	13.9	80.7
3.0	-	2.9	-	-	-	-	43.8
42.2	87.6	1.4	79.9	50.7	288.0	34.2	26.6
72.8	-	-	27.2	-	-	9.7	43.5
-	-	-	-	-	-	-	-
5.8	92.2	-	20.6	31.0	13.0	-	-
-	-	-	11.1	-	53.8	-	-
10.0	-	-	29.5	-	-	19.2	67.6
78.9	-	-	50.8	-	17.5	1.7	23.4
26237.9	2745.1	30319.7	5881.9	22852.3	14054.1	28672.5	27991.6
19320.3	15191.5	22109.7	33114.7	7047.1	47963.3	16432.3	13375.8
2776.5	448.8	4606.3	2197.7	4402.3	7217.7	3059.8	1640.7
-	15.8	19.8	32.4	-	-	16.2	-
30228.3	15638.2	26282.5	68430.3	126228.9	59690.0	82261.1	294910.0
1812.0	1160.0	2502.4	1255.5	1016.4	674.1	908.1	323.7
8762.0	2496.3	3659.5	3912.4	1837.0	935.1	1218.8	358.8
5980.2	2963.1	4556.2	1369.1	1344.0	184.2	1507.2	548.1
686.8	215.7	445.9	24.2	316.3	54.5	274.3	497.9
708.1	482.7	525.3	873.9	599.2	772.5	889.4	472.6
18655.2	15351.8	12769.7	16779.9	14053.6	4863.9	11601.8	14505.5
23.6	10.0	14.7	2.6	27.1	-	15.7	6.7
13.7	-	-	37.5	22.7	-	-	11.8
6028.0	2484.1	5158.6	3891.8	11668.9	15612.6	6085.4	3691.6
542.0	116.4	223.9	507.4	186.3	392.4	356.1	556.5
-	-	-	-	-	-	-	-
-	-	26.3	-	9.5	-	12.4	-
-	-	15.6	79.4	-	-	-	-
20.8	36.3	215.3	328.3	408.1	59.6	22.4	43.1
-	-	18.2	83.1	-	-	-	-
43.0	216.7	149.7	363.8	-	383.0	87.5	199.4

-	-	-	-	-	27.4	7.4	96.3
-	5.8	-	-	-	-	-	-
4.0	11.6	44.4	25.1	20.7	23.4	18.5	3.3
-	-	-	-	-	-	-	1181.4
10328.2	10822.9	14336.9	2202.9	2430.3	526.7	6313.4	22895.4
36949.2	39758.8	53450.2	22664.1	13504.7	36181.6	27863.2	44524.4
26967.5	23819.2	42747.1	9343.3	10047.1	4770.9	17370.8	25827.7
5499.7	499.8	3087.3	2893.8	3771.4	10233.0	5224.7	8413.9
-	-	-	21.2	-	-	-	29.3
6171.8	553.3	4234.0	3728.6	7243.4	14371.3	9764.6	10447.7
1748.2	627.7	1748.2	4332.0	2974.1	1024.5	1853.6	8700.6
-	-	-	-	-	-	-	-
1252.4	710.0	1942.3	3955.1	2128.6	4867.2	3490.0	4783.3
6683.7	821.0	1820.1	4809.0	2598.4	959.0	2528.6	1861.0
226.6	45.1	62.5	223.3	75.3	150.0	131.7	162.6
3.8	-	4.0	87.3	36.1	-	5.4	139.8
71.3	112.1	544.1	1794.9	379.9	107.0	1111.9	1016.8
-	-	23.5	80.9	0.2	-	-	-
4363.2	2766.4	2494.1	7240.0	4356.3	3861.3	2784.0	2281.3
0.4	1.7	1.4	34.8	-	-	-	-
14.3	100.5	7.4	24.1	93.4	-	8.2	24.3
34.9	43.5	7.2	150.6	36.3	117.9	71.0	20.9
-	-	-	-	-	-	-	0.2
-	-	125.4	626.3	1.3	-	0.3	0.8
-	-	13.0	163.2	-	-	-	-
-	-	37.1	194.5	0.2	-	-	-
-	-	43.1	198.4	0.4	-	-	-
-	-	60.3	286.8	0.3	-	0.2	-
-	-	-	14.9	22.1	8.7	-	32.4
-	-	-	5.6	-	-	-	-
11.3	-	9.0	172.3	37.7	-	69.0	41.5
4.4	-	5.6	40.8	3.3	18.5	10.5	-
-	-	-	10.5	-	19.8	-	-
5.0	-	-	-	-	-	6.3	-
1655.6	479.3	3170.1	1262.2	2305.4	2397.7	1864.0	1446.4
26.9	4.6	35.1	87.7	2.8	-	59.4	61.7
-	-	-	-	-	-	-	-
13.1	-	34.7	42.3	3.8	-	23.5	93.8
54.5	-	12.1	13.7	29.6	-	52.3	25.8
6.6	96.3	4.5	3.4	14.4	44.3	2.9	-
2.9	2.8	12.7	-	0.5	-	0.4	2.1
19.5	20.0	69.1	158.5	68.6	50.5	367.4	456.6
5.9	-	-	6.8	0.6	-	-	11.1
84.6	473.3	71.6	308.7	141.3	243.8	131.3	376.0
-	-	-	63.5	-	15.3	-	-
14.8	6.8	21.9	4.1	-	4.6	-	-
-	36.3	-	11.4	19.7	-	-	62.7
3.9	-	4.0	7.4	-	-	3.8	-
-	-	-	-	-	-	-	-
-	-	-	-	-	-	-	-

5881.4	1528.2	8773.9	5239.8	4238.8	5185.3	8231.8	6878.3
-	1.2	-	-	-	-	-	12.9
0.4	0.7	5.1	-	-	-	0.4	-
47.9	27.1	75.7	137.1	66.3	88.9	40.7	4.1
-	-	2.8	33.4	1.8	-	-	6.5
18.7	-	-	-	-	-	-	-
-	-	-	16.8	8.4	-	19.9	101.6
-	-	-	-	8.7	-	19.0	48.1
-	10.9	-	-	3.9	7.8	-	51.9
21.4	13.2	31.7	-	-	-	9.0	-
313.4	458.6	350.4	144.5	39.5	165.2	215.3	38.7
17.5	-	7.8	-	-	-	-	4.9
2.8	7.5	9.7	6.0	-	9.3	2.8	-
-	-	13.9	66.8	-	-	-	-
-	87.6	-	-	-	-	-	-
32.5	92.8	-	15.7	-	16.3	-	16.3
-	-	-	-	0.5	-	-	0.9
-	-	-	-	-	-	0.8	7.2
-	-	15.2	53.5	-	-	-	-
18.3	54.9	42.1	37.3	51.4	70.3	21.7	11.3
62.3	-	38.2	6.8	56.0	-	106.1	341.6
-	-	-	-	-	-	-	-
-	-	-	10.0	-	-	-	-
-	-	13.3	84.9	0.2	-	-	-
-	-	-	-	-	-	-	-
12.7	300.2	10.8	10.3	56.8	-	25.4	13.0
19.2	158.4	18.7	9.6	19.0	-	7.7	20.9
15.9	90.5	-	-	70.6	-	16.4	17.9
-	-	174.4	1293.2	0.5	-	-	-
-	-	21.3	74.1	-	-	-	-
-	-	4.8	9.5	-	-	-	-
-	-	16.8	86.5	-	-	-	-
-	-	-	-	-	-	-	-
3.3	2.4	741.8	3842.0	24.1	98.8	1.7	24.0
-	22.2	-	10.3	-	-	-	7.8
2.3	-	48.0	33.1	16.6	65.2	-	-
4.3	16.1	-	12.6	-	55.2	8.6	22.9
-	-	-	90.7	6.7	-	-	-
11.1	12.3	44.8	38.4	44.4	228.1	0.1	18.1
53.7	9.3	81.4	4.7	53.5	-	54.9	37.4
124.6	234.7	301.9	601.9	22.7	853.4	122.9	86.9
163.7	102.7	97.5	507.5	302.3	840.1	397.5	431.8
-	-	-	-	-	-	-	-
41.2	4.8	0.7	156.2	79.7	-	134.6	522.6
53.4	89.6	-	68.9	-	169.6	68.1	33.1
62453.2	57371.2	108491.5	180003.3	64225.7	366365.5	81518.5	55305.8
11.8	-	-	-	12.1	-	10.0	2.9
-	0.3	-	-	0.4	-	-	6.8
180.0	14.5	52.1	14.8	90.8	12.9	79.8	56.6
13.2	70.4	26.4	100.6	63.6	202.2	19.9	20.5

123954.5	20522.7	60254.8	40978.3	122042.0	28182.1	107721.5	74865.8
53592.4	7463.7	26181.9	14186.8	65379.4	10082.9	48882.0	30523.4
57916.4	7145.0	26989.8	13456.9	45231.1	8739.9	47394.4	31502.8
2.4	-	-	-	8.1	-	1.2	-
-	17.9	-	-	52.3	-	19.6	-
125.7	103.8	189.1	712.0	455.9	1233.7	218.9	423.5
-	-	-	-	-	-	-	-
3213.3	2481.5	1840.8	12785.8	3223.0	8167.8	4854.1	3900.2
-	-	44.3	189.3	1.6	-	-	-
-	-	9.0	52.1	-	-	-	-
-	-	15.5	67.5	-	-	-	-
29.7	7.6	14.6	15.1	5.9	41.1	15.5	-
24.2	141.0	851.9	3806.0	81.4	109.8	16.7	138.3
-	-	-	3.2	-	-	-	-
-	-	-	3.4	-	-	3.4	-
-	-	9.2	-	-	-	-	-
4.6	-	-	53.0	-	-	12.8	59.5
-	-	633.1	1594.0	-	894.8	-	-
19.5	17.1	3.3	26.1	12.0	6.8	48.2	-
8.8	-	-	14.2	4.2	-	1.6	-
-	-	-	4.2	-	-	3.0	-
3.8	96.5	11.9	15.0	36.9	-	-	-
370.7	314.7	316.9	2854.6	691.6	189.7	1142.1	4154.4
-	-	13.0	63.0	-	-	-	-
-	-	31.1	104.8	-	-	-	-
-	2.7	-	-	-	-	-	-
0.3	136.3	13.6	87.9	9.7	20.1	5.5	2469.9
-	267.7	17.7	137.0	16.3	44.4	14.0	3529.2
-	104.3	-	25.9	29.7	35.6	8.9	862.4
-	381.1	7.0	209.2	51.9	-	19.6	1908.7
1257.6	2293.3	732.4	3620.0	701.9	127.9	963.0	2050.2
331.8	1074.8	311.1	1881.5	104.2	88.9	637.6	1178.0
11.9	15.2	-	19.3	8.0	-	7.6	-
-	-	-	8.9	-	-	-	-
-	-	-	-	-	-	-	-
-	-	55.2	266.9	-	-	-	-
-	-	-	17.5	17.0	41.2	16.0	20.5
10.2	10.2	2.1	161.0	17.7	197.4	28.6	118.2
-	-	17.4	71.6	-	-	-	-
-	-	121.4	445.3	2.1	7.9	-	129.5
-	-	91.5	424.4	0.5	13.3	-	-
-	-	19.5	82.2	-	-	-	-
-	-	19.0	82.6	-	-	-	-
-	-	41.1	695.7	8.6	37.5	-	-
-	-	29.1	301.4	4.6	13.2	-	-
-	3.9	21.0	276.7	-	-	-	-
-	-	40.2	313.2	-	-	-	-
4702.7	2932.9	8112.8	10441.3	5743.9	21250.9	4738.4	3900.6
5097.6	517.2	8069.6	2474.4	10978.9	10134.8	5050.9	4028.3
5.1	-	4.5	16.4	0.5	7.6	2.2	9.5

427.5	64.5	166.0	800.5	229.8	266.8	339.6	300.6
136.5	206.9	227.4	439.3	62.7	-	521.1	883.1
1.2	-	57.4	1393.2	4.2	4.3	0.8	-
-	-	76.8	400.7	0.5	-	-	-
-	82.1	66.9	211.3	-	58.1	22.8	-
-	-	-	-	-	-	-	-
0.1	-	98.7	703.1	1.1	6.0	-	-
-	-	-	-	-	17.5	-	-
-	-	1.4	9.9	-	-	-	33.0
-	-	-	-	-	162.4	-	-
-	-	-	2.0	1.2	-	-	-
69.0	48.4	67.1	333.8	58.9	94.2	176.7	696.0
6.6	5.7	9.3	31.1	30.9	31.7	10.7	22.1
11.0	4.0	14.7	19.8	1.4	11.0	5.4	3.9
34.4	477.6	48.8	344.1	47.3	1959.1	180.6	209.8
332.7	108.2	220.3	707.6	5263.1	445.7	357.7	2150.1
970.4	124.8	402.2	3405.2	2117.6	1406.6	1269.8	8663.2
4.2	0.3	6.6	5.1	20.4	39.0	17.3	299.1
506.0	893.8	46.1	405.0	52.6	97.4	451.7	340.5
-	161.2	-	12.7	23.5	20.6	-	19.9
-	7.4	-	8.3	6.5	-	-	-
31.6	10.1	26.4	19.6	68.6	-	98.5	313.6
2537.5	1914.7	2709.2	2547.1	983.9	6424.4	1470.1	6.5
495.7	7.5	227.2	165.6	424.2	965.9	530.3	559.4
-	34.5	-	-	78.6	-	24.9	31.9
21315.0	68211.6	13690.1	32828.3	41494.3	13342.9	21389.7	25291.9
10222.9	110596.1	9243.0	20233.0	49784.7	5739.5	15794.9	15522.8
945.7	2121.3	646.0	898.4	1021.1	344.1	415.7	308.2
34.7	596.6	-	51.5	32.8	22.2	31.3	71.6
97.5	10.7	63.8	67.1	73.8	10.5	9.8	-
57.6	97.7	-	19.7	30.5	-	19.9	9.1
4587.7	72477.7	4115.0	9153.0	27822.4	4889.1	6605.3	10961.1
-	-	-	100.5	-	70.1	228.6	76.1
45.3	18.6	56.9	34.8	36.5	41.2	-	14.8
1887.8	7617.1	1403.2	2405.6	3642.0	728.0	1206.5	1127.0
196.3	24.5	96.9	77.0	82.5	58.1	18.5	28.1
11.6	-	-	-	10.4	-	-	-
7.5	143.2	-	21.1	134.9	-	9.7	32.5
-	-	69.1	-	-	-	-	-
-	-	-	-	-	-	-	-
9501.3	15503.2	6167.2	16336.7	12064.3	7288.6	7250.2	10260.0
-	-	-	-	-	-	-	-
25.3	33.3	126.9	14.3	6.2	50.0	60.3	745.8
-	-	-	-	-	-	-	-
19.7	-	17.1	103.0	0.8	-	8.6	0.2
5237.4	202.8	341.0	2283.4	1608.6	390.8	1571.6	2553.2
117.2	644.0	1190.4	692.6	238.8	1170.2	7150.1	52742.6
-	-	-	67.0	-	-	-	-
-	-	9.0	-	-	-	4.6	8.2
-	-	-	51.4	-	-	20.6	9.0

-	7.3	3.0	39.6	4.6	41.6	-	-
-	51.3	-	90.3	103.4	89.9	-	-
7.3	-	-	10.2	1.9	13.0	7.9	5.9
7.5	-	-	-	-	-	13.5	-
65.1	10.1	56.7	11.0	74.8	10.3	92.3	154.7
23.3	7.8	8.5	4.0	-	20.6	14.4	22.7
-	-	0.3	-	-	-	-	-
8.0	32.4	-	95.2	-	83.3	68.5	9.1
7.7	-	-	6.2	-	7.4	10.1	-
86.4	127.1	124.2	165.4	63.9	217.5	101.5	281.0
37.5	20.1	24.7	130.5	114.8	155.4	76.9	157.8
50.3	6.7	18.5	17.5	22.8	-	43.5	-
-	-	-	-	-	-	-	-
0.2	1.8	4.5	-	0.3	-	0.3	3.2
345.0	477.8	719.4	1016.5	593.6	1659.9	726.3	815.3
17.5	23.4	19.2	-	17.4	-	11.1	2.2
-	-	-	8.3	-	-	-	2.1
-	-	18.3	68.8	-	-	-	-
-	-	50.1	263.2	-	2.9	-	-
-	-	27.7	120.5	-	-	-	-
-	-	51.4	240.4	-	-	-	-
1007.1	160.8	1681.3	1045.1	1869.4	3776.8	1140.5	1191.6
-	-	-	-	-	-	-	-
29.3	3.4	58.8	-	10.7	-	18.2	17.5
11.7	46.3	73.3	13.7	-	-	-	-
-	-	-	-	-	-	-	-
-	-	7.3	46.2	-	-	-	-
-	-	-	-	-	-	-	-
12640.6	12932.2	19309.9	2135.3	1116.0	199.1	7976.1	91.0
29.0	67.3	23.4	278.7	50.0	61.2	77.3	39.4
-	-	-	-	-	-	-	-
0.1	0.3	44.4	366.3	1.1	6.0	-	5.5
-	-	19.0	85.3	-	-	-	-
-	-	10.7	71.3	-	-	-	-
1589.8	143.7	1843.1	520.9	2800.5	1202.9	2177.5	2435.2
2400.0	2652.8	3198.4	3425.8	1612.5	5143.0	2359.4	105.6
2974.8	2214.7	4715.1	5187.8	3479.7	23601.4	3282.9	1308.0
-	-	16.1	67.8	-	-	-	2.5
-	-	-	-	-	-	-	1.9
-	6.1	9.0	-	-	-	-	-
-	-	-	31.6	-	-	-	-
-	-	-	9.9	-	-	17.4	-
17.7	5.5	-	6.4	-	11.6	67.5	-
364.3	69.7	143.5	386.9	408.2	1231.8	421.4	900.5
-	-	-	-	-	-	-	48.7
-	-	-	-	-	-	-	-
-	-	-	6.9	-	2.5	-	-
3.8	28.9	15.8	75.3	12.3	-	3.0	-
-	-	30.5	154.1	0.3	4.5	-	1.3
-	-	17.9	59.7	-	-	-	-

731.9	373.7	496.6	2465.5	1151.8	1532.5	1213.7	1459.4
911.3	59.0	219.4	207.0	132.0	49.7	482.0	455.4
172.1	322.3	475.9	7.2	4.3	2.8	21.6	-
-	-	21.6	109.1	-	-	-	-
0.3	-	30.0	140.7	0.9	2.8	1.7	1.9
-	-	25.0	183.4	1.6	-	-	-
-	-	-	-	-	-	-	-
11.9	-	-	15.2	12.4	-	5.8	5.9
125.3	-	43.7	422.9	6.5	112.0	157.6	268.3
-	-	-	6.8	10.7	-	30.5	62.1
-	28.3	-	10.2	-	-	7.9	26.0
-	-	4.0	-	-	-	6.7	4.6
-	-	-	-	-	-	-	-
7.2	-	-	162.8	-	-	-	-
21.1	39.8	-	61.5	2.9	-	4.2	-
42.9	23.6	43.4	140.6	25.7	61.5	65.2	153.4
-	-	19.4	84.0	1.3	-	-	-
63.6	8.0	86.0	69.4	21.4	109.8	-	19.6
-	-	-	-	-	-	-	-
0.2	-	24.6	126.7	-	11.8	-	-
-	-	17.3	81.8	-	-	-	-
-	-	22.6	98.2	-	-	-	-
-	-	26.6	116.5	-	-	-	-
-	-	19.3	82.6	-	-	-	-
-	-	23.1	175.8	-	-	-	-
-	-	42.3	199.3	0.6	-	-	-
-	-	17.8	88.9	-	-	-	-
7.4	21.2	-	51.2	11.7	26.0	5.6	20.3
-	-	-	-	-	-	-	-
6.2	4.3	9.1	-	-	-	1.8	-
-	-	30.6	51.7	69.3	-	-	1.2
24.5	7.5	11.7	33.3	20.6	13.7	14.9	21.0
1443.2	1084.7	663.3	1980.3	1822.0	733.2	972.7	2246.4
-	-	-	-	-	-	-	-
6.0	869.9	-	-	-	-	6.5	-
-	-	-	32.7	31.3	120.4	-	74.4
-	-	-	-	36.2	49.2	-	-
1663.8	193.7	708.8	724.0	472.0	731.5	1370.1	641.7
-	-	-	2.5	11.2	-	7.9	64.3
-	-	-	-	0.2	-	0.2	24.6
268.2	69.3	380.4	205.4	129.9	171.6	238.1	293.5
118.1	145.2	414.0	794.8	229.3	169.3	804.8	1248.0
257646.9	361633.6	323767.1	247633.7	156270.4	112748.2	247448.8	2009.7
747.9	200.9	1336.8	317.1	1221.9	2433.3	1320.3	912.8
-	-	11.1	84.9	-	-	-	-
-	-	-	-	-	-	-	-
5.9	1.0	85.9	93.0	8.7	268.9	35.9	800.4
5564.9	773.4	2824.0	675.5	3207.4	-	4379.3	6449.6
-	11.7	1.7	6.4	-	14.7	7.5	2.7
9.3	-	-	24.5	10.0	-	10.7	12.4

43.7	209.1	94.8	321.1	30.9	-	141.7	130.8
-	-	-	-	-	-	-	-
-	-	14.3	71.0	-	-	-	-
39.2	61.6	-	14.3	-	12.2	21.7	16.4
172.5	322.3	285.2	26.5	-	24.2	-	579.7
-	-	-	-	-	-	-	10.2
-	18.8	65.3	39.2	-	43.2	20.0	101.9
-	31.4	-	63.1	0.3	97.6	0.5	50.0
-	-	-	27.7	-	36.8	-	-
14057.5	2302.7	6412.8	9103.5	19970.8	9069.0	16725.2	16581.1
6.2	2.8	3.2	-	1.7	-	3.3	2.8
-	-	-	-	-	-	-	-
284.9	58.8	368.1	609.4	229.9	199.4	258.4	75.3
-	-	-	-	-	-	-	-
65.3	40.3	6.2	140.6	47.4	19.0	97.4	305.9
-	-	-	-	-	28.5	-	13.8
-	0.7	17.9	107.2	-	-	-	-
-	-	-	-	-	-	-	-
-	-	28.3	110.1	-	-	-	-
28.6	79.5	33.9	321.3	42.0	31.5	23.8	124.5
20.6	87.2	-	5.3	18.2	8.5	17.7	24.3
-	21.6	-	28.6	-	45.3	7.6	-
15.1	391.6	18.9	81.6	116.7	78.7	42.5	24.8
192.6	63.6	228.2	1134.1	477.1	437.7	988.1	1373.1
6.1	-	-	41.5	10.1	-	23.2	-
-	-	48.8	404.0	-	-	-	-
995.3	108.7	340.6	996.7	345.8	903.2	413.1	550.1
-	-	-	-	-	-	-	13.4
-	-	-	2.0	-	-	-	-
-	7.1	-	-	-	-	-	-
-	-	-	-	-	-	-	-
80.0	10.6	56.3	127.3	67.0	33.0	145.2	236.4
203.0	80.0	168.2	26.4	85.3	-	143.0	-
-	-	-	-	-	-	-	-
0.5	0.9	-	2.3	-	-	-	0.6
-	267.4	-	-	-	-	-	200.1
5.0	0.1	-	3.4	-	-	-	-
-	0.9	308.7	1424.4	1.5	80.7	0.1	11.8
-	-	86.3	373.5	1.5	-	4.6	-
-	-	52.4	260.7	-	-	-	-
3.7	-	-	53.1	4.6	-	-	0.7
-	9.1	-	7.8	10.1	-	-	-
-	-	18.9	103.0	-	-	-	-
-	-	12.1	65.6	-	-	-	-
-	-	-	28.8	-	6.1	3.3	169.2
64.3	133.4	100.6	443.4	112.2	150.8	97.4	216.7
-	-	-	56.5	-	-	-	-
-	-	20.1	92.6	-	-	-	-
465.4	219.6	786.6	1072.5	599.9	153.4	795.8	1358.2
1298.3	373.6	1979.8	4343.9	1657.5	1383.5	4034.4	10314.4

27.1	5.4	6.8	143.6	42.3	13.9	32.8	4.4
-	-	28.0	121.4	2.1	-	-	-
-	-	-	-	-	-	-	-
-	-	-	-	6.4	-	23.8	19.5
133.1	93.6	98.2	390.3	956.6	178.2	327.2	-
22.4	17.1	10.0	11.0	15.1	14.6	17.5	34.8
60.1	15.1	28.0	268.8	70.2	32.5	156.2	556.5

HD_9	HD_10	MP.number of observations	HD.number of observations	Analysis type	p-value (U-test)	FDR (U-test)
-	24.3	2	5	D	NA	NA
7478.0	6504.6	10	10	C	0.7959	0.8706
16.9	-	8	3	D	NA	NA
-	-	3	1	D	NA	NA
1884.9	773.9	10	10	C	0.0185	0.0600
-	3.2	5	8	C	0.6710	0.8007
-	-	2	0	D	NA	NA
-	-	0	2	D	NA	NA
-	-	4	2	D	NA	NA
-	-	3	5	D	NA	NA
30.5	5.8	9	9	C	0.3401	0.5809
8.1	-	7	5	C	0.7551	0.8674
931.2	951.9	10	10	C	0.6305	0.7902
-	22.0	9	9	C	0.0820	0.2050
5.0	16.5	10	10	C	0.3527	0.5868
18.3	43.3	4	6	D	NA	NA
7.4	3.8	5	5	D	NA	NA
56.0	53.0	8	10	C	0.0140	0.0539
-	-	6	4	D	NA	NA
-	-	4	0	D	NA	NA
-	9.3	5	6	D	NA	NA
-	27.1	5	3	D	NA	NA
8.9	-	9	5	C	0.0464	0.1309
-	-	9	5	C	0.2398	0.4820
9223.8	10770.0	10	10	C	0.7959	0.8706
14133.1	11588.6	10	10	C	0.0003	0.0090
1547.4	2066.7	10	10	C	0.0011	0.0169
-	22.3	7	5	C	0.5303	0.7154
56880.7	43980.7	10	10	C	0.1431	0.2971
95.4	222.6	10	10	C	0.3527	0.5868
908.1	429.4	10	10	C	0.1431	0.3369
767.8	581.5	10	10	C	0.4359	0.6350
440.4	101.8	9	10	C	0.0789	0.2379
267.2	227.3	10	10	C	0.0753	0.2343
6422.0	7700.0	10	10	C	0.6305	0.8007
6.4	6.8	10	9	C	0.7197	0.8545
-	-	6	4	D	NA	NA
7443.9	2994.6	10	10	C	0.3930	0.6181
141.7	205.9	10	10	C	0.4359	0.6350
-	-	1	0	D	NA	NA
7.3	-	5	4	D	NA	NA
-	-	0	2	D	NA	NA
144.7	48.6	10	10	C	0.1903	0.3548
-	-	0	2	D	NA	NA
85.6	298.6	5	9	C	0.0070	0.0500

-	-	5	3	D	NA	NA
-	-	5	1	D	NA	NA
130.5	30.2	9	10	C	0.6038	0.7730
-	-	3	1	D	NA	NA
16895.9	9490.7	10	10	C	0.5288	0.7154
50104.9	32501.6	10	10	C	0.8534	0.8952
38724.8	25295.9	10	10	C	0.4359	0.6350
5521.3	6609.5	10	10	C	0.4813	0.6682
-	-	0	2	D	NA	NA
5121.3	6572.8	10	10	C	0.3150	0.5584
1519.0	1382.4	10	10	C	0.1431	0.3369
-	-	2	0	D	NA	NA
757.2	1442.0	10	10	C	0.1655	0.3758
1920.1	2279.8	10	10	C	0.7394	0.8545
81.7	124.3	9	10	C	0.6038	0.7730
6.8	10.4	9	8	C	0.0592	0.1938
175.1	594.8	10	10	C	0.1051	0.2849
-	-	0	3	D	NA	NA
1105.2	1465.0	10	10	C	0.0029	0.0198
-	-	8	4	C	0.0139	0.0539
9.1	-	3	8	D	NA	NA
56.2	32.4	10	10	C	0.00004	0.0024
-	-	0	1	D	NA	NA
-	0.4	6	6	C	0.6675	0.8007
-	-	0	2	D	NA	NA
-	-	0	3	D	NA	NA
-	-	0	3	D	NA	NA
0.2	-	1	5	D	NA	NA
12.3	-	10	5	C	0.0012	0.0121
-	0.6	5	2	D	NA	NA
184.9	12.1	10	8	C	0.0017	0.0156
9.8	9.5	9	8	C	0.2406	0.4085
-	-	1	2	D	NA	NA
-	-	6	2	D	NA	NA
1141.9	1780.0	10	10	C	0.0147	0.0788
165.8	16.7	9	9	C	0.0081	0.0409
-	-	1	0	D	NA	NA
31.1	8.7	10	8	C	0.0172	0.0592
9.2	20.9	8	8	C	0.3429	0.5312
-	-	3	7	D	NA	NA
27.3	1.7	7	8	C	0.3811	0.5665
51.8	27.2	4	10	C	0.0020	0.0237
-	9.2	3	5	D	NA	NA
303.8	210.3	10	10	C	0.7959	0.8706
-	-	7	2	D	NA	NA
38.5	41.8	6	7	C	0.4631	0.6616
-	48.0	5	5	D	NA	NA
8.6	-	3	5	D	NA	NA
-	-	4	0	D	NA	NA
-	-	2	0	D	NA	NA

2295.8	4487.7	10	10	C	0.0115	0.0693
-	-	5	2	D	NA	NA
3.1	-	7	5	C	0.1843	0.3513
90.7	48.6	10	10	C	0.2176	0.3799
-	4.6	7	5	C	0.2742	0.4436
-	-	1	1	D	NA	NA
13.4	24.0	5	6	D	NA	NA
3.9	16.8	5	5	D	NA	NA
5.9	-	1	5	D	NA	NA
10.4	-	2	5	D	NA	NA
381.7	325.6	10	10	C	0.0288	0.1264
18.5	-	9	4	C	0.0061	0.0336
4.7	2.5	10	8	C	0.0021	0.0237
-	-	0	2	D	NA	NA
-	-	0	1	D	NA	NA
6.0	24.8	0	7	D	NA	NA
-	-	7	2	D	NA	NA
-	2.4	1	3	D	NA	NA
-	-	0	2	D	NA	NA
15.8	71.6	6	10	C	0.5189	0.7136
25.5	86.9	7	8	C	0.9090	0.9287
-	-	2	0	D	NA	NA
-	-	0	1	D	NA	NA
-	-	0	3	D	NA	NA
-	-	2	0	D	NA	NA
8.8	-	8	8	C	0.4710	0.6642
53.3	-	5	8	C	0.0489	0.1345
-	21.6	2	6	D	NA	NA
-	-	0	3	D	NA	NA
-	-	0	2	D	NA	NA
-	-	0	2	D	NA	NA
-	-	2	2	D	NA	NA
-	-	2	0	D	NA	NA
0.6	5.2	9	10	C	0.2176	0.3799
-	-	1	3	D	NA	NA
-	3.8	4	6	D	NA	NA
27.5	63.8	5	8	C	0.0653	0.2099
-	-	9	2	D	NA	NA
-	32.4	10	9	C	0.3154	0.5584
50.6	15.3	9	9	C	0.0019	0.0237
686.3	293.2	10	10	C	0.6305	0.7902
228.4	141.3	10	10	C	0.1051	0.2849
-	-	4	0	D	NA	NA
11.0	37.9	10	9	C	0.2775	0.5150
84.0	106.5	9	8	C	1.0000	1.0000
87633.5	64765.2	10	10	C	0.0524	0.1814
-	-	3	4	D	NA	NA
-	-	9	3	C	0.1000	0.2849
48.5	43.7	10	10	C	0.4359	0.6350
88.0	31.3	9	10	C	0.3527	0.5314

45214.0	66359.5	10	10	C	0.0052	0.0401
21708.7	26767.5	10	10	C	0.0147	0.0788
22709.9	26267.0	10	10	C	0.0147	0.0788
2.6	1.2	2	5	D	NA	NA
-	-	1	3	D	NA	NA
805.1	292.3	10	10	C	0.0007	0.0080
-	-	1	0	D	NA	NA
4696.9	5252.4	10	10	C	0.1655	0.3758
-	0.3	0	4	D	NA	NA
-	-	0	2	D	NA	NA
-	-	1	2	D	NA	NA
10.4	15.1	9	9	C	0.6500	0.8007
0.3	14.9	8	10	C	0.0451	0.1305
-	-	7	1	D	NA	NA
9.5	-	7	3	D	NA	NA
11.8	-	4	2	D	NA	NA
39.5	25.5	3	6	D	NA	NA
-	-	5	3	D	NA	NA
142.2	61.4	10	9	C	0.0005	0.0067
11.4	14.5	10	6	C	0.0035	0.0226
21.9	12.8	5	4	D	NA	NA
-	-	0	5	D	NA	NA
582.4	519.7	10	10	C	0.4359	0.6350
-	-	0	2	D	NA	NA
-	-	0	2	D	NA	NA
-	-	0	1	D	NA	NA
2.9	3.6	9	10	C	0.0147	0.0539
13.5	4.6	7	9	C	0.1720	0.3379
5.6	7.3	2	8	D	NA	NA
-	41.9	8	7	C	0.1588	0.3235
447.6	684.7	10	10	C	0.9118	0.9311
209.7	163.5	10	10	C	0.4359	0.6350
6.2	-	5	6	D	NA	NA
-	-	2	1	D	NA	NA
-	-	2	0	D	NA	NA
0.5	-	1	3	D	NA	NA
18.7	-	10	6	C	0.0027	0.0198
94.0	57.7	9	10	C	0.7959	0.8669
6.1	-	3	3	D	NA	NA
-	-	0	5	D	NA	NA
-	-	0	4	D	NA	NA
-	-	0	2	D	NA	NA
-	-	0	2	D	NA	NA
-	-	0	4	D	NA	NA
-	-	2	4	D	NA	NA
-	-	0	3	D	NA	NA
-	-	0	2	D	NA	NA
4027.6	4385.6	10	10	C	0.4813	0.6682
3471.2	3400.1	10	10	C	0.7394	0.8545
6.2	4.5	8	9	C	0.8884	0.9218

162.5	283.8	10	10	C	0.0052	0.0401
152.5	182.3	9	9	C	0.7623	0.8644
1.7	-	1	7	D	NA	NA
-	-	0	3	D	NA	NA
-	126.0	4	6	D	NA	NA
-	-	2	0	D	NA	NA
-	0.3	2	6	D	NA	NA
-	-	3	1	D	NA	NA
12.5	-	10	4	C	0.0041	0.0249
-	-	1	1	D	NA	NA
-	-	3	2	D	NA	NA
670.3	94.3	10	10	C	0.0007	0.0080
15.0	55.0	10	10	C	0.0433	0.1286
29.9	19.7	10	10	C	0.0147	0.0539
407.2	102.5	10	10	C	0.1051	0.2360
293.8	267.6	10	10	C	0.7959	0.8706
660.0	599.0	10	10	C	0.8534	0.8952
0.9	-	9	9	C	0.2224	0.4567
1211.3	769.2	10	10	C	0.3930	0.6181
-	31.0	1	6	D	NA	NA
-	8.1	9	4	C	0.0185	0.0600
95.2	28.4	9	9	C	0.2567	0.4278
1886.1	1229.6	9	10	C	0.0535	0.1814
480.8	67.2	10	10	C	0.7394	0.8545
-	22.1	2	5	D	NA	NA
15225.1	11774.7	10	10	C	0.6842	0.8007
6900.2	10142.7	10	10	C	0.6842	0.8007
367.1	1296.0	10	10	C	0.8534	0.8941
-	-	1	7	D	NA	NA
6.9	321.1	6	9	C	0.6753	0.8007
16.4	96.7	4	8	C	0.1975	0.3562
4084.7	4844.2	10	10	C	0.7959	0.8669
-	206.9	0	5	D	NA	NA
14.6	19.3	1	9	D	NA	NA
789.3	2140.5	10	10	C	0.3527	0.5314
35.5	589.9	10	10	C	0.9118	0.9287
-	87.7	2	3	D	NA	NA
-	-	2	6	D	NA	NA
-	-	5	1	D	NA	NA
-	-	1	0	D	NA	NA
8027.7	6265.8	10	10	C	0.6842	0.8007
-	-	4	0	D	NA	NA
87.4	46.1	9	10	C	0.0001	0.0027
-	-	1	0	D	NA	NA
-	-	5	6	D	NA	NA
1238.3	4465.4	10	10	C	0.9118	0.9287
79.2	4935.7	10	10	C	0.1051	0.2849
-	-	0	1	D	NA	NA
7.0	11.0	7	5	C	0.2414	0.4085
-	4.6	5	4	D	NA	NA

-	-	1	5	D	NA	NA
-	-	0	4	D	NA	NA
-	-	2	6	D	NA	NA
-	-	5	2	D	NA	NA
156.4	77.5	10	10	C	0.7959	0.8669
10.6	23.4	8	9	C	0.1852	0.3513
-	-	0	1	D	NA	NA
4.2	18.8	8	8	C	1.0000	1.0000
-	-	6	4	D	NA	NA
157.2	68.7	10	10	C	0.1230	0.2706
26.3	30.1	10	10	C	0.0892	0.2180
27.0	10.2	3	8	D	NA	NA
-	-	1	0	D	NA	NA
2.7	0.9	7	8	C	0.6753	0.8007
544.8	582.2	10	10	C	0.0021	0.0177
42.4	40.5	9	8	C	0.0171	0.0592
2.3	0.6	10	4	C	0.0001	0.0027
-	-	0	2	D	NA	NA
-	-	0	3	D	NA	NA
-	-	0	2	D	NA	NA
-	-	0	2	D	NA	NA
468.5	924.2	10	10	C	0.0052	0.0301
-	-	2	0	D	NA	NA
9.6	10.6	8	8	C	0.7984	0.8706
167.1	10.5	2	6	D	NA	NA
-	-	5	0	D	NA	NA
-	-	0	2	D	NA	NA
-	-	1	0	D	NA	NA
20353.3	11607.0	10	10	C	0.3930	0.5765
36.9	20.2	6	10	C	0.0207	0.0649
-	-	4	0	D	NA	NA
48.7	0.4	8	9	C	0.4267	0.6175
-	-	0	2	D	NA	NA
-	-	1	2	D	NA	NA
478.5	1151.6	9	10	C	0.0115	0.0486
3525.8	3640.1	10	10	C	0.00002	0.0024
1375.9	2058.5	10	10	C	0.6842	0.8007
0.5	-	0	4	D	NA	NA
-	-	3	1	D	NA	NA
-	-	1	2	D	NA	NA
-	-	0	1	D	NA	NA
-	-	2	2	D	NA	NA
-	-	4	5	D	NA	NA
262.3	254.6	10	10	C	0.0892	0.2609
-	-	0	1	D	NA	NA
4.3	6.3	7	2	D	NA	NA
-	-	10	2	C	0.0001	0.0027
7.4	8.0	2	8	D	NA	NA
-	4.2	1	6	D	NA	NA
-	-	0	2	D	NA	NA

931.0	1079.3	10	10	C	1.0000	1.0000
407.2	387.4	9	10	C	0.7197	0.8545
994.5	6.6	9	9	C	0.5457	0.7257
-	-	0	2	D	NA	NA
-	1.9	0	8	D	NA	NA
-	-	0	3	D	NA	NA
-	-	2	0	D	NA	NA
23.1	11.6	6	7	C	0.5624	0.7638
47.6	29.5	10	9	C	0.1128	0.2981
-	-	0	4	D	NA	NA
15.1	-	6	5	D	NA	NA
11.0	-	8	4	C	0.0317	0.0970
-	-	1	0	D	NA	NA
-	-	2	2	D	NA	NA
13.9	86.3	9	7	C	0.1971	0.3562
159.1	71.4	10	10	C	0.0089	0.0409
-	-	0	3	D	NA	NA
21.3	16.4	8	9	C	0.7333	0.8472
-	-	2	0	D	NA	NA
-	1.7	1	5	D	NA	NA
-	-	0	2	D	NA	NA
-	-	0	2	D	NA	NA
-	-	0	2	D	NA	NA
-	-	1	2	D	NA	NA
-	-	0	2	D	NA	NA
-	-	0	3	D	NA	NA
-	-	0	2	D	NA	NA
15.6	-	8	8	C	0.6764	0.8007
-	-	7	0	D	NA	NA
6.2	-	3	5	D	NA	NA
-	-	0	4	D	NA	NA
61.4	-	10	9	C	0.0041	0.0363
1369.7	987.3	10	10	C	0.1903	0.4174
-	-	1	0	D	NA	NA
-	-	1	3	D	NA	NA
-	-	7	4	D	NA	NA
-	27.2	2	3	D	NA	NA
490.9	649.9	10	10	C	0.0039	0.0357
-	4.1	8	5	C	0.0186	0.0923
-	-	3	3	D	NA	NA
106.7	228.8	10	10	C	0.3150	0.5584
383.5	302.0	10	10	C	0.0001	0.0049
406835.3	441921.5	10	10	C	0.0068	0.0500
533.2	802.9	10	10	C	0.8534	0.8952
-	-	0	2	D	NA	NA
-	-	1	0	D	NA	NA
18.5	5.4	5	10	C	0.2544	0.4960
2271.0	4874.6	10	9	C	0.1431	0.2971
11.1	5.9	8	8	C	0.2712	0.4436
10.0	7.8	10	7	C	0.0636	0.1628

77.4	81.9	10		9	C		0.7394	0.8472
-	-	5		0	D	NA	NA	
-	-	0		2	D	NA	NA	
44.7	116.8	7		8	C		0.6753	0.8007
119.7	312.1	9		8	C		0.8499	0.8941
6.4	6.9	3		3	D	NA	NA	
20.7	21.1	3		8	D	NA	NA	
-	-	7		6	C		0.3343	0.5312
-	-	0		2	D	NA	NA	
5562.9	11830.0	10		10	C		0.1431	0.2971
12.8	3.8	4		8	C		0.0931	0.2180
-	-	6		0	D	NA	NA	
309.6	556.2	10		10	C		0.4813	0.6701
-	-	1		0	D	NA	NA	
72.0	84.5	10		10	C		0.0089	0.0409
5.2	-	7		3	D	NA	NA	
1.8	-	2		4	D	NA	NA	
-	-	4		0	D	NA	NA	
-	-	1		2	D	NA	NA	
202.1	90.2	10		10	C		0.0433	0.1643
10.8	11.6	8		9	C		0.8499	0.8941
-	12.3	8		5	C		0.7869	0.8669
-	20.6	6		9	C		0.0086	0.0409
942.3	173.4	10		10	C		0.5787	0.7764
5.0	-	6		5	D	NA	NA	
-	-	0		2	D	NA	NA	
297.2	371.0	10		10	C		0.0524	0.1402
-	-	2		1	D	NA	NA	
-	-	3		1	D	NA	NA	
-	14.4	1		2	D	NA	NA	
-	2.1	2		1	D	NA	NA	
107.4	101.1	10		10	C		0.0115	0.0486
277.2	94.1	8		8	C		0.3429	0.5312
-	-	2		0	D	NA	NA	
-	-	0		4	D	NA	NA	
-	-	4		2	D	NA	NA	
11.6	-	6		4	D	NA	NA	
0.3	18.6	10		9	C		0.1655	0.3310
-	0.6	9		5	C		0.0919	0.2180
-	-	1		2	D	NA	NA	
0.4	-	10		5	C		0.0026	0.0198
2.1	-	1		4	D	NA	NA	
-	-	0		2	D	NA	NA	
-	-	0		2	D	NA	NA	
-	17.5	4		5	D	NA	NA	
52.9	219.8	10		10	C		0.8534	0.8941
-	-	0		1	D	NA	NA	
1.3	-	2		3	D	NA	NA	
231.3	799.3	9		10	C		0.8421	0.8952
1818.3	1966.7	10		10	C		0.2475	0.4873

35.3	57.7	9	10	C	0.1051	0.2360
-	-	0	3	D	NA	NA
8.9	-	6	1	D	NA	NA
-	-	7	3	D	NA	NA
190.9	367.1	8	9	C	0.0535	0.1402
76.0	47.2	10	10	C	0.0003	0.0051
389.7	122.1	10	10	C	0.0001	0.0027

Effect size (RBCC)	p-value (Fisher test)	FDR (Fisher test)	Effect size (Crammer V)	Size of effect	change in MP vs. HD	**ProteinName**
NA	0.3498	1.0000	0.315	medium	DOWN	**A1BG**
-0.080	NA	NA	NA	no effect	UP	**A2M**
NA	0.0698	0.6823	0.503	large	UP	**AACS**
NA	0.5820	1.0000	0.250	small	UP	**ACAN**
-0.620	NA	NA	NA	large	UP	**ACTB**
0.120	NA	NA	NA	small	DOWN	**ACTG2**
NA	0.4737	1.0000	0.333	medium	UP	**ADAM10**
NA	0.4737	1.0000	0.333	medium	DOWN	**ADH5**
NA	0.6285	1.0000	0.218	small	UP	**ADIPOQ**
NA	0.6499	1.0000	0.204	small	DOWN	**AFM**
-0.040	NA	NA	NA	no effect	UP	**AGT**
0.700	NA	NA	NA	very large	DOWN	**AHSG**
-0.140	NA	NA	NA	small	UP	**ALB**
0.470	NA	NA	NA	medium	DOWN	**ALDOA**
0.260	NA	NA	NA	small	DOWN	**AMBP**
NA	0.6563	1.0000	0.200	small	DOWN	**ANGPTL6**
NA	1.0000	1.0000	0.000	no effect	NA	**ANK1**
0.660	NA	NA	NA	large	DOWN	**ANPEP**
NA	0.6563	1.0000	0.200	small	UP	**ANTXR2**
NA	0.0867	0.6823	0.500	large	UP	**ANXA11**
NA	1.0000	1.0000	0.101	small	DOWN	**ANXA2**
NA	0.6499	1.0000	0.204	small	UP	**ANXA5**
-0.530	NA	NA	NA	large	UP	**ANXA7**
0.740	NA	NA	NA	very large	DOWN	**APCS**
0.080	NA	NA	NA	no effect	DOWN	**APOA1**
0.880	NA	NA	NA	very large	DOWN	**APOA2**
0.820	NA	NA	NA	very large	DOWN	**APOA4**
0.740	NA	NA	NA	very large	DOWN	**APOA5**
-0.400	NA	NA	NA	medium	UP	**APOB**
0.260	NA	NA	NA	small	DOWN	**APOC1**
-0.400	NA	NA	NA	medium	UP	**APOC2**
-0.220	NA	NA	NA	small	UP	**APOC3**
-0.340	NA	NA	NA	medium	UP	**APOC4**
-0.480	NA	NA	NA	medium	UP	**APOD**
-0.140	NA	NA	NA	small	UP	**APOE**
0.000	NA	NA	NA	no effect	UP	**APOF**
NA	0.6563	1.0000	0.200	small	UP	**APOH**
0.240	NA	NA	NA	small	DOWN	**APOL1**
0.220	NA	NA	NA	small	DOWN	**APOM**
NA	1.0000	1.0000	0.229	small	UP	**ARL6IP5**
NA	1.0000	1.0000	0.101	small	UP	**ARPC5**
NA	0.4737	1.0000	0.333	medium	DOWN	**ATIC**
-0.360	NA	NA	NA	medium	UP	**ATP2A3**
NA	0.4737	1.0000	0.333	medium	DOWN	**ATP5F1B**
0.940	NA	NA	NA	very large	DOWN	**ATRN**

NA		0.6499	1.0000	0.204	small	UP	**B2M**
NA		0.1409	0.9354	0.436	medium	UP	**BCAP31**
	0.240	NA	NA	NA	small	DOWN	**BCHE**
NA		0.5820	1.0000	0.250	small	UP	**C16orf54**
	-0.180	NA	NA	NA	small	UP	**C1QA**
	-0.060	NA	NA	NA	no effect	UP	**C1QB**
	-0.220	NA	NA	NA	small	UP	**C1QC**
	0.200	NA	NA	NA	small	DOWN	**C1R**
NA		0.4737	1.0000	0.333	medium	DOWN	**C1RL**
	0.280	NA	NA	NA	small	DOWN	**C1S**
	-0.400	NA	NA	NA	medium	UP	**C3**
NA		0.4737	1.0000	0.333	medium	UP	**C4A**
	0.380	NA	NA	NA	medium	DOWN	**C4B**
	-0.100	NA	NA	NA	small	UP	**C4BPA**
	-0.040	NA	NA	NA	no effect	UP	**C4BPB**
	-0.120	NA	NA	NA	small	UP	**C5**
	0.440	NA	NA	NA	medium	DOWN	**C9**
NA		0.2105	0.9832	0.420	medium	DOWN	**CALM3**
	0.760	NA	NA	NA	very large	DOWN	**CAMP**
	-0.640	NA	NA	NA	large	UP	**CANX**
NA		0.0698	0.6823	0.503	large	DOWN	**CASP14**
	-0.960	NA	NA	NA	very large	UP	**CAVIN2**
NA		1.0000	1.0000	0.229	small	DOWN	**CCL5**
	0.120	NA	NA	NA	small	DOWN	**CCT2**
NA		0.4737	1.0000	0.333	medium	DOWN	**CCT4**
NA		0.2105	0.9832	0.420	medium	DOWN	**CCT6A**
NA		0.2105	0.9832	0.420	medium	DOWN	**CCT7**
NA		0.1409	0.9354	0.436	medium	DOWN	**CCT8**
	-0.860	NA	NA	NA	very large	UP	**CD151**
NA		0.3498	1.0000	0.315	medium	UP	**CD226**
	-0.840	NA	NA	NA	very large	UP	**CD36**
	-0.320	NA	NA	NA	medium	UP	**CD47**
NA		1.0000	1.0000	0.140	small	DOWN	**CD53**
NA		0.1698	0.9748	0.408	medium	UP	**CD59**
	0.640	NA	NA	NA	large	DOWN	**CD5L**
	-0.710	NA	NA	NA	very large	UP	**CD9**
NA		1.0000	1.0000	0.229	small	UP	**CD99**
	-0.640	NA	NA	NA	large	UP	**CDC42**
	-0.260	NA	NA	NA	small	UP	**CDCA2**
NA		0.1789	0.9748	0.400	medium	DOWN	**CDSN**
	-0.240	NA	NA	NA	small	UP	**CELSR2**
	1.000	NA	NA	NA	very large	DOWN	**CETP**
NA		0.6499	1.0000	0.204	small	DOWN	**CFB**
	-0.080	NA	NA	NA	no effect	UP	**CFH**
NA		0.0698	0.6823	0.503	large	UP	**CFHR3**
	0.200	NA	NA	NA	small	DOWN	**CFL1**
NA		1.0000	1.0000	0.000	no effect	NA	**CFP**
NA		0.6499	1.0000	0.204	small	DOWN	**CLIC1**
NA		0.0867	0.6823	0.500	large	UP	**CLTA**
NA		0.4737	1.0000	0.333	medium	UP	**CLTCL1**

0.660	NA	NA	NA	large	DOWN	**CLU**
NA	0.3498	1.0000	0.315	medium	UP	**CMTM5**
-0.350	NA	NA	NA	medium	UP	**CMTM6**
-0.340	NA	NA	NA	medium	UP	**COL1A1**
-0.290	NA	NA	NA	small	UP	**COL1A2**
NA	1.0000	1.0000	0.000	no effect	NA	**COL2A1**
NA	1.0000	1.0000	0.101	small	DOWN	**COL6A1**
NA	1.0000	1.0000	0.000	no effect	NA	**COL6A3**
NA	0.1409	0.9354	0.436	medium	DOWN	**COL7A1**
NA	0.3498	1.0000	0.315	medium	DOWN	**COLEC11**
0.580	NA	NA	NA	large	DOWN	**CP**
-0.720	NA	NA	NA	very large	UP	**CPN1**
-0.460	NA	NA	NA	medium	UP	**CPN2**
NA	0.4737	1.0000	0.333	medium	DOWN	**CSDE1**
NA	1.0000	1.0000	0.229	small	DOWN	**CST6**
NA	0.0031	0.2632	0.734	very large	DOWN	**CTSD**
NA	0.0698	0.6823	0.503	large	UP	**CYB5R3**
NA	0.5820	1.0000	0.250	small	DOWN	**CYBB**
NA	0.4737	1.0000	0.333	medium	DOWN	**DAZAP1**
0.180	NA	NA	NA	small	DOWN	**DCD**
0.040	NA	NA	NA	no effect	DOWN	**DCDC1**
NA	0.4737	1.0000	0.333	medium	UP	**DDX60L**
NA	1.0000	1.0000	0.229	small	DOWN	**DEFA1**
NA	0.2105	0.9832	0.420	medium	DOWN	**DHX9**
NA	0.4737	1.0000	0.333	medium	UP	**DPP6**
-0.200	NA	NA	NA	small	UP	**DSC1**
0.520	NA	NA	NA	large	DOWN	**DSG1**
NA	0.1698	0.9748	0.408	medium	DOWN	**DSP**
NA	0.2105	0.9832	0.420	medium	DOWN	**EEF1A1**
NA	0.4737	1.0000	0.333	medium	DOWN	**EEF2**
NA	0.4737	1.0000	0.333	medium	DOWN	**EFEMP1**
NA	1.0000	1.0000	0.000	no effect	NA	**EIF5A**
NA	0.4737	1.0000	0.333	medium	UP	**ENDOD1**
0.340	NA	NA	NA	medium	DOWN	**ENO1**
NA	0.5820	1.0000	0.250	small	DOWN	**EPB42**
NA	0.6563	1.0000	0.200	small	DOWN	**F10**
0.860	NA	NA	NA	very large	DOWN	**F11**
NA	0.0055	0.3880	0.704	very large	UP	**F11R**
-0.160	NA	NA	NA	small	UP	**F13A1**
-0.480	NA	NA	NA	medium	UP	**F13B**
0.140	NA	NA	NA	small	DOWN	**F2**
-0.440	NA	NA	NA	medium	UP	**F5**
NA	0.0867	0.6823	0.500	large	UP	**F8**
0.380	NA	NA	NA	medium	DOWN	**FBLN1**
0.000	NA	NA	NA	no effect	UP	**FCER1G**
0.520	NA	NA	NA	large	DOWN	**FCGBP**
NA	1.0000	1.0000	0.105	small	DOWN	**FCN1**
0.540	NA	NA	NA	large	DOWN	**FCN2**
-0.220	NA	NA	NA	small	UP	**FCN3**
0.260	NA	NA	NA	small	DOWN	**FERMT3**

-0.720	NA	NA	NA		very large	UP	**FGA**
-0.640	NA	NA	NA		large	UP	**FGB**
-0.640	NA	NA	NA		large	UP	**FGG**
NA		0.3498	1.0000	0.315	medium	DOWN	**FGL1**
NA		0.5820	1.0000	0.250	small	DOWN	**FLG2**
-0.840	NA	NA	NA		very large	UP	**FLNA**
NA		1.0000	1.0000	0.229	small	UP	**FLOT1**
-0.380	NA	NA	NA		medium	UP	**FN1**
NA		0.0867	0.6823	0.500	large	DOWN	**FSCN1**
NA		0.4737	1.0000	0.333	medium	DOWN	**G3BP1**
NA		1.0000	1.0000	0.140	small	DOWN	**G6PD**
-0.130	NA	NA	NA		small	UP	**GALE**
0.540	NA	NA	NA		large	DOWN	**GAPDH**
NA		0.0198	0.5757	0.612	large	UP	**GC**
NA		0.1789	0.9748	0.400	medium	UP	**GNAI2**
NA		0.6285	1.0000	0.218	small	UP	**GNAQ**
NA		0.3698	1.0000	0.302	medium	DOWN	**GNB4**
NA		0.6499	1.0000	0.204	small	UP	**GNG5**
-0.860	NA	NA	NA		very large	UP	**GP1BA**
-0.780	NA	NA	NA		very large	UP	**GP1BB**
NA		1.0000	1.0000	0.101	small	UP	**GP9**
NA		0.0325	0.5757	0.577	large	DOWN	**GSDMA**
-0.220	NA	NA	NA		small	UP	**GSN**
NA		0.4737	1.0000	0.333	medium	DOWN	**GSR**
NA		0.4737	1.0000	0.333	medium	DOWN	**GSTP1**
NA		1.0000	1.0000	0.229	small	DOWN	**GYPA**
0.640	NA	NA	NA		large	DOWN	**H2AC20**
0.370	NA	NA	NA		medium	DOWN	**H2BC21**
NA		0.0230	0.5757	0.600	large	DOWN	**H3-3A**
0.380	NA	NA	NA		medium	DOWN	**H4C1**
-0.040	NA	NA	NA		no effect	UP	**HBA1**
0.220	NA	NA	NA		small	DOWN	**HBB**
NA		1.0000	1.0000	0.101	small	DOWN	**HBD**
NA		1.0000	1.0000	0.140	small	UP	**HBG1**
NA		0.4737	1.0000	0.333	medium	UP	**HGFAC**
NA		0.5820	1.0000	0.250	small	DOWN	**HINT1**
-0.800	NA	NA	NA		very large	UP	**HLA-A**
-0.080	NA	NA	NA		no effect	UP	**HLA-B**
NA		1.0000	1.0000	0.000	no effect	NA	**HMBS**
NA		0.0325	0.5757	0.577	large	DOWN	**HNRNPA1L2**
NA		0.0867	0.6823	0.500	large	DOWN	**HNRNPA2B1**
NA		0.4737	1.0000	0.333	medium	DOWN	**HNRNPA3**
NA		0.4737	1.0000	0.333	medium	DOWN	**HNRNPC**
NA		0.0867	0.6823	0.500	large	DOWN	**HNRNPF**
NA		0.6285	1.0000	0.218	small	DOWN	**HNRNPH1**
NA		0.2105	0.9832	0.420	medium	DOWN	**HNRNPH3**
NA		0.4737	1.0000	0.333	medium	DOWN	**HNRNPU**
0.200	NA	NA	NA		small	DOWN	**HP**
-0.100	NA	NA	NA		small	UP	**HPR**
0.320	NA	NA	NA		medium	DOWN	**HPX**

0.720	NA	NA	NA	very large	DOWN	**HRG**
0.090	NA	NA	NA	no effect	DOWN	**HRNR**
NA	0.0198	0.5757	0.612	large	DOWN	**HSP90AA1**
NA	0.2105	0.9832	0.420	medium	DOWN	**HSPA1B**
NA	0.6563	1.0000	0.200	small	DOWN	**HSPA8**
NA	0.4737	1.0000	0.333	medium	UP	**HSPA9**
NA	0.1698	0.9748	0.408	medium	DOWN	**HSPD1**
NA	0.5820	1.0000	0.250	small	UP	**ICAM2**
-0.760	NA	NA	NA	very large	UP	**ILK**
NA	1.0000	1.0000	0.000	no effect	NA	**INPP5D**
NA	1.0000	1.0000	0.116	small	UP	**ITGA2**
-0.840	NA	NA	NA	very large	UP	**ITGA2B**
-0.540	NA	NA	NA	large	UP	**ITGA6**
-0.640	NA	NA	NA	large	UP	**ITGB1**
-0.440	NA	NA	NA	medium	UP	**ITGB3**
0.080	NA	NA	NA	no effect	DOWN	**ITIH1**
-0.060	NA	NA	NA	no effect	UP	**ITIH2**
-0.100	NA	NA	NA	small	UP	**ITIH3**
-0.240	NA	NA	NA	small	UP	**ITIH4**
NA	0.0573	0.6823	0.524	large	DOWN	**JUP**
-0.620	NA	NA	NA	large	UP	**KANSL3**
-0.310	NA	NA	NA	medium	UP	**KIAA2026**
0.580	NA	NA	NA	large	DOWN	**KLKB1**
-0.100	NA	NA	NA	small	UP	**KNG1**
NA	0.3498	1.0000	0.315	medium	DOWN	**KPRP**
0.120	NA	NA	NA	small	DOWN	**KRT1**
0.120	NA	NA	NA	small	DOWN	**KRT10**
-0.060	NA	NA	NA	no effect	UP	**KRT14**
NA	0.0198	0.5757	0.612	large	DOWN	**KRT15**
0.120	NA	NA	NA	small	DOWN	**KRT16**
0.340	NA	NA	NA	medium	DOWN	**KRT17**
0.080	NA	NA	NA	no effect	DOWN	**KRT2**
NA	0.0325	0.5757	0.577	large	DOWN	**KRT24**
NA	0.0011	0.1549	0.800	very large	DOWN	**KRT32**
0.260	NA	NA	NA	small	DOWN	**KRT5**
0.040	NA	NA	NA	no effect	DOWN	**KRT6A**
NA	1.0000	1.0000	0.116	small	DOWN	**KRT6B**
NA	0.1698	0.9748	0.408	medium	DOWN	**KRT78**
NA	0.1409	0.9354	0.436	medium	UP	**KRT85**
NA	1.0000	1.0000	0.229	small	UP	**KRT86**
0.120	NA	NA	NA	small	DOWN	**KRT9**
NA	0.0867	0.6823	0.500	large	UP	**LAMA2**
0.940	NA	NA	NA	very large	DOWN	**LBP**
NA	1.0000	1.0000	0.229	small	UP	**LDHA**
NA	1.0000	1.0000	0.101	small	DOWN	**LDHB**
0.040	NA	NA	NA	no effect	DOWN	**LGALS3BP**
-0.440	NA	NA	NA	medium	UP	**LPA**
NA	1.0000	1.0000	0.229	small	DOWN	**LRBA**
-0.310	NA	NA	NA	medium	UP	**LRP1**
NA	1.0000	1.0000	0.101	small	UP	**LTBP1**

NA	0.1409	0.9354	0.436	medium	DOWN	**LTF**
NA	0.0867	0.6823	0.500	large	DOWN	**LYZ**
NA	0.1698	0.9748	0.408	medium	DOWN	**M6PR**
NA	0.3498	1.0000	0.315	medium	UP	**MARCO**
-0.080	NA	NA	NA	no effect	UP	**MASP1**
0.360	NA	NA	NA	medium	DOWN	**MASP2**
NA	1.0000	1.0000	0.229	small	DOWN	**MATR3**
0.000	NA	NA	NA	no effect	UP	**MBL2**
NA	0.6563	1.0000	0.200	small	UP	**MENT**
0.420	NA	NA	NA	medium	DOWN	**MMRN1**
0.460	NA	NA	NA	medium	DOWN	**MSN**
NA	0.0698	0.6823	0.503	large	DOWN	**MXRA5**
NA	1.0000	1.0000	0.229	small	UP	**MYADM**
0.120	NA	NA	NA	small	DOWN	**MYH16**
-0.780	NA	NA	NA	very large	UP	**MYH9**
-0.640	NA	NA	NA	large	UP	**MYL12B**
-1.000	NA	NA	NA	very large	UP	**MYL6**
NA	0.4737	1.0000	0.333	medium	DOWN	**NANS**
NA	0.2105	0.9832	0.420	medium	DOWN	**NASP**
NA	0.4737	1.0000	0.333	medium	DOWN	**NCL**
NA	0.4737	1.0000	0.333	medium	DOWN	**NPM1**
0.720	NA	NA	NA	very large	DOWN	**NR0B1**
NA	0.4737	1.0000	0.333	medium	UP	**ORM1**
0.300	NA	NA	NA	medium	DOWN	**ORM2**
NA	0.1698	0.9748	0.408	medium	DOWN	**OSBPL2**
NA	0.0325	0.5757	0.577	large	UP	**PATJ**
NA	0.4737	1.0000	0.333	medium	DOWN	**PCBP1**
NA	1.0000	1.0000	0.229	small	UP	**PCDHGB7**
0.240	NA	NA	NA	small	DOWN	**PCYOX1**
0.620	NA	NA	NA	large	DOWN	**PECAM1**
NA	0.0867	0.6823	0.500	large	UP	**PF4V1**
-0.220	NA	NA	NA	small	UP	**PFN1**
NA	0.4737	1.0000	0.333	medium	DOWN	**PGK1**
NA	1.0000	1.0000	0.140	small	DOWN	**PHB**
0.660	NA	NA	NA	large	DOWN	**PHLDB2**
0.980	NA	NA	NA	very large	DOWN	**PIGR**
0.120	NA	NA	NA	small	DOWN	**PKD2L1**
NA	0.0867	0.6823	0.500	large	DOWN	**PKM**
NA	0.5820	1.0000	0.250	small	UP	**PLA2G7**
NA	1.0000	1.0000	0.140	small	DOWN	**PLEK**
NA	1.0000	1.0000	0.229	small	DOWN	**PLG**
NA	1.0000	1.0000	0.000	no effect	NA	**PLTP**
NA	1.0000	1.0000	0.101	small	DOWN	**PNPLA8**
0.460	NA	NA	NA	medium	DOWN	**PON1**
NA	1.0000	1.0000	0.229	small	DOWN	**PPARG**
NA	0.0698	0.6823	0.503	large	UP	**PPBP**
-1.000	NA	NA	NA	very large	UP	**PPIA**
NA	0.0230	0.5757	0.600	large	DOWN	**PRDX1**
NA	0.0573	0.6823	0.524	large	DOWN	**PRDX2**
NA	0.4737	1.0000	0.333	medium	DOWN	**PRDX6**

0.000	NA	NA	NA	no effect	UP	**PRG4**
0.000	NA	NA	NA	no effect	UP	**PROS1**
0.040	NA	NA	NA	no effect	DOWN	**PRSS1**
NA	0.4737	1.0000	0.333	medium	DOWN	**PSMA5**
NA	0.0007	0.1518	0.817	very large	DOWN	**PSMA6**
NA	0.2105	0.9832	0.420	medium	DOWN	**PTBP1**
NA	0.4737	1.0000	0.333	medium	UP	**PTGS1**
0.160	NA	NA	NA	small	DOWN	**PTPRJ**
0.500	NA	NA	NA	large	DOWN	**PZP**
NA	0.0867	0.6823	0.500	large	DOWN	**QSOX1**
NA	1.0000	1.0000	0.101	small	UP	**RAB10**
-0.560	NA	NA	NA	large	UP	**RAB1B**
NA	1.0000	1.0000	0.229	small	UP	**RAB27B**
NA	1.0000	1.0000	0.000	no effect	NA	**RAB33B**
-0.350	NA	NA	NA	medium	UP	**RAC2**
-0.680	NA	NA	NA	large	UP	**RAP1B**
NA	0.2105	0.9832	0.420	medium	DOWN	**RCC2**
-0.100	NA	NA	NA	small	UP	**RELN**
NA	0.4737	1.0000	0.333	medium	UP	**RHOA**
NA	0.1409	0.9354	0.436	medium	DOWN	**RPL11**
NA	0.4737	1.0000	0.333	medium	DOWN	**RPL8**
NA	0.4737	1.0000	0.333	medium	DOWN	**RPLP0**
NA	0.4737	1.0000	0.333	medium	DOWN	**RPS15**
NA	1.0000	1.0000	0.140	small	DOWN	**RPS15A**
NA	0.4737	1.0000	0.333	medium	DOWN	**RPS23**
NA	0.2105	0.9832	0.420	medium	DOWN	**RPS3A**
NA	0.4737	1.0000	0.333	medium	DOWN	**RPSA**
-0.120	NA	NA	NA	small	UP	**RTN3**
NA	0.0031	0.2632	0.734	very large	UP	**RTN4**
NA	0.6499	1.0000	0.204	small	DOWN	**S100A8**
NA	0.0867	0.6823	0.500	large	DOWN	**S100A9**
-0.580	NA	NA	NA	large	UP	**SAA1**
-0.360	NA	NA	NA	medium	UP	**SAA4**
NA	1.0000	1.0000	0.229	small	UP	**SACM1L**
NA	0.5820	1.0000	0.250	small	DOWN	**SBSN**
NA	0.3698	1.0000	0.302	medium	UP	**SELENOP**
NA	1.0000	1.0000	0.116	small	DOWN	**SELPLG**
-0.740	NA	NA	NA	very large	UP	**SERPINA1**
0.280	NA	NA	NA	small	DOWN	**SERPINA3**
NA	1.0000	1.0000	0.000	no effect	NA	**SERPINA4**
-0.280	NA	NA	NA	small	UP	**SERPINC1**
0.940	NA	NA	NA	very large	DOWN	**SERPIND1**
0.700	NA	NA	NA	very large	DOWN	**SERPINF2**
-0.060	NA	NA	NA	no effect	UP	**SERPING1**
NA	0.4737	1.0000	0.333	medium	DOWN	**SF3A3**
NA	1.0000	1.0000	0.229	small	UP	**SH3BGRL3**
0.700	NA	NA	NA	very large	DOWN	**SHBG**
-0.400	NA	NA	NA	medium	UP	**SIGLEC16**
0.300	NA	NA	NA	medium	DOWN	**SLC2A1**
-0.500	NA	NA	NA	large	UP	**SLC2A3**

0.100	NA	NA	NA	small	DOWN	**SLC4A1**
NA	0.0325	0.5757	0.577	large	UP	**SNX14**
NA	0.4737	1.0000	0.333	medium	DOWN	**SOD1**
0.120	NA	NA	NA	small	DOWN	**SPATA3**
0.060	NA	NA	NA	no effect	DOWN	**SPATA48**
NA	1.0000	1.0000	0.000	no effect	NA	**SPN**
NA	0.0698	0.6823	0.503	large	DOWN	**SPTA1**
-0.260	NA	NA	NA	small	UP	**SPTB**
NA	0.4737	1.0000	0.333	medium	DOWN	**SPTBN1**
-0.400	NA	NA	NA	medium	UP	**SPTLC3**
0.440	NA	NA	NA	medium	DOWN	**SRGN**
NA	0.0108	0.4605	0.655	large	UP	**SRI**
0.200	NA	NA	NA	small	DOWN	**SSC5D**
NA	1.0000	1.0000	0.229	small	UP	**STEAP3**
-0.680	NA	NA	NA	large	UP	**STOM**
NA	0.1789	0.9748	0.400	medium	UP	**SVEP1**
NA	0.6285	1.0000	0.218	small	DOWN	**SYNCRIP**
NA	0.0867	0.6823	0.500	large	UP	**TAGLN2**
NA	1.0000	1.0000	0.140	small	DOWN	**TCP1**
-0.540	NA	NA	NA	large	UP	**TF**
0.060	NA	NA	NA	no effect	DOWN	**TFRC**
-0.080	NA	NA	NA	no effect	UP	**TGFBI**
0.700	NA	NA	NA	very large	DOWN	**TGM3**
0.160	NA	NA	NA	small	DOWN	**THBS1**
NA	1.0000	1.0000	0.101	small	UP	**THBS4**
NA	0.4737	1.0000	0.333	medium	DOWN	**TKT**
-0.520	NA	NA	NA	large	UP	**TLN1**
NA	1.0000	1.0000	0.140	small	UP	**TM4SF1**
NA	0.5820	1.0000	0.250	small	UP	**TMEM109**
NA	1.0000	1.0000	0.140	small	DOWN	**TMEM198**
NA	1.0000	1.0000	0.140	small	UP	**TMX2**
-0.660	NA	NA	NA	large	UP	**TNC**
-0.260	NA	NA	NA	small	UP	**TNKS1BP1**
NA	0.4737	1.0000	0.333	medium	UP	**TPM4**
NA	0.0867	0.6823	0.500	large	DOWN	**TSPAN14**
NA	0.6285	1.0000	0.218	small	UP	**TTLL12**
NA	0.6563	1.0000	0.200	small	UP	**TTR**
-0.380	NA	NA	NA	medium	UP	**TUBA1C**
-0.450	NA	NA	NA	medium	UP	**TUBA4A**
NA	1.0000	1.0000	0.140	small	DOWN	**TUBB**
-0.800	NA	NA	NA	very large	UP	**TUBB1**
NA	0.3034	1.0000	0.346	medium	DOWN	**UBA52**
NA	0.4737	1.0000	0.333	medium	DOWN	**UBE2L3**
NA	0.4737	1.0000	0.333	medium	DOWN	**UBQLN1**
NA	1.0000	1.0000	0.101	small	DOWN	**VCAN**
0.060	NA	NA	NA	no effect	DOWN	**VCL**
NA	1.0000	1.0000	0.229	small	DOWN	**VDAC2**
NA	1.0000	1.0000	0.116	small	DOWN	**VIM**
0.040	NA	NA	NA	no effect	DOWN	**VTN**
-0.320	NA	NA	NA	medium	UP	**VWF**

-0.440	NA	NA	NA	medium	UP	**WBP11**
NA	0.2105	0.9832	0.420	medium	DOWN	**XRCC5**
NA	0.0573	0.6823	0.524	large	UP	**YWHAB**
NA	0.1789	0.9748	0.400	medium	UP	**YWHAE**
0.520	NA	NA	NA	large	DOWN	**YWHAG**
-0.880	NA	NA	NA	very large	UP	**YWHAH**
-0.920	NA	NA	NA	very large	UP	**YWHAZ**

EntrezID	T-cell-specific protein	putative serum protein (0- no; 1- yes)
1	1	1
2	1	1
65985	1	0
176	NA	0
60	1	0
72	NA	0
102	1	0
128	1	0
9370	NA	0
173	1	1
183	1	1
197	1	1
213	1	1
226	1	0
259	1	1
83854	NA	1
286	1	0
290	1	0
118429	1	0
311	1	0
302	1	0
308	1	0
310	1	0
325	1	1
335	1	1
336	1	1
337	1	1
116519	NA	1
338	1	1
341	1	1
344	NA	1
345	1	1
346	NA	1
347	1	1
348	1	1
319	NA	1
350	1	1
8542	NA	1
55937	NA	1
10550	1	0
10092	1	0
471	1	0
489	1	0
506	1	0
8455	NA	1

567	1	0
10134	1	0
590	NA	1
283897	1	0
712	NA	1
713	NA	1
714	NA	1
715	1	1
51279	NA	1
716	1	1
718	1	1
720	NA	1
721	NA	1
722	NA	1
725	NA	1
727	1	1
735	1	1
808	1	0
820	1	0
821	1	0
23581	1	0
8436	1	0
6352	1	0
10576	1	0
10575	1	0
908	1	0
10574	1	0
10694	1	0
977	1	0
10666	1	0
948	1	0
961	1	0
963	1	0
966	1	0
922	NA	1
928	1	0
4267	1	0
998	1	0
157313	NA	0
1041	NA	0
1952	NA	0
1071	NA	1
629	1	1
3075	1	1
10878	NA	1
1072	1	0
5199	NA	1
1192	1	0
1211	1	0
8218	NA	0

1191	1	1
116173	NA	0
54918	NA	0
1277	NA	0
1278	NA	0
1280	NA	0
1291	NA	0
1293	NA	0
1294	NA	0
78989	NA	0
1356	1	1
1369	1	0
1370	NA	1
7812	1	0
1474	NA	1
1509	1	0
1727	1	0
1536	1	0
26528	1	0
117159	1	0
341019	NA	0
91351	1	0
1667	NA	1
1660	1	0
1804	NA	0
1823	1	0
1828	1	0
1832	1	0
1915	1	0
1938	1	0
2202	NA	0
1984	1	0
23052	1	0
2023	1	0
2038	NA	0
2159	NA	1
2160	NA	1
50848	1	0
2162	1	1
2165	NA	1
2147	1	1
2153	1	1
2157	NA	1
2192	NA	1
2207	1	0
8857	NA	1
2219	1	0
2220	NA	1
8547	NA	1
83706	1	0

2243	1	1
2244	1	1
2266	1	1
2267	NA	1
388698	1	0
2316	1	0
10211	1	0
2335	1	1
6624	1	0
10146	1	0
2539	1	0
2582	1	0
2597	1	0
2638	1	1
2771	1	0
2776	1	0
59345	NA	0
2787	1	0
2811	1	0
2812	1	0
2815	NA	0
284110	1	0
2934	1	1
2936	1	0
2950	1	0
2993	NA	0
8338	1	0
8349	1	0
3020	NA	0
8359	NA	0
3039	NA	1
3043	1	1
3045	NA	1
3047	NA	1
3083	NA	0
3094	1	0
3105	1	0
3106	1	0
3145	1	0
144983	NA	0
3181	1	0
220988	1	0
3183	1	0
3185	1	0
3187	1	0
3189	1	0
3192	1	0
3240	1	1
3250	NA	1
3263	1	1

3273	1	1
388697	1	0
3320	1	0
3304	1	0
3312	1	0
3313	1	0
3329	1	0
3384	1	0
3611	1	0
3635	1	0
3673	NA	0
3674	1	0
3655	1	0
3688	1	0
3690	1	0
3697	1	1
3698	1	1
3699	1	1
3700	1	1
3728	1	0
55683	1	0
158358	NA	0
3818	1	1
3827	1	1
448834	1	0
3848	1	0
3858	1	0
3861	1	0
3866	1	0
3868	1	0
3872	1	0
3849	1	0
192666	NA	0
3882	NA	0
3852	1	0
3853	1	0
3854	1	0
196374	1	0
3891	NA	0
3892	NA	0
3857	1	0
3908	NA	0
3929	1	0
3939	1	0
3945	1	0
3959	1	0
4018	NA	1
987	1	0
4035	1	0
4052	NA	0

4057	1	0
4069	1	0
4074	1	0
8685	NA	0
5648	NA	0
10747	NA	0
9782	1	0
4153	NA	0
54964	NA	0
22915	1	0
4478	1	0
25878	NA	0
91663	1	0
84176	1	0
4627	1	0
103910	1	0
4637	1	0
54187	1	0
4678	1	0
4691	1	0
4869	1	0
190	NA	0
5004	NA	1
5005	NA	1
9885	1	0
10207	1	0
5093	1	0
56099	NA	0
51449	1	0
5175	1	0
5197	1	0
5216	1	0
5230	1	0
5245	1	0
90102	NA	0
5284	NA	0
9033	NA	0
5315	1	0
7941	NA	0
5341	1	0
5340	1	1
5360	NA	1
50640	1	0
5444	1	1
5468	NA	0
5473	1	1
5478	1	0
5052	1	0
7001	1	0
9588	1	0

10216	NA	1
5627	NA	1
5644	NA	1
5686	1	0
5687	1	0
5725	1	0
5742	1	0
5795	1	0
5858	NA	1
5768	1	0
10890	1	0
81876	1	0
5874	1	0
83452	1	0
5880	1	0
5908	1	0
55920	1	0
5649	NA	0
387	1	0
6135	1	0
6132	1	0
6175	1	0
6209	1	0
6210	1	0
6228	1	0
6189	1	0
3921	1	0
10313	1	0
57142	1	0
6279	1	0
6280	1	0
6288	NA	1
6291	1	1
22908	1	0
374897	1	0
6414	NA	1
6404	1	0
5265	1	1
12	1	1
5267	1	1
462	1	1
3053	1	1
5345	1	1
710	1	1
10946	1	0
83442	1	0
6462	NA	1
400709	NA	0
6513	1	0
6515	1	0

6521	NA	0
57231	NA	0
6647	1	0
130560	NA	0
1E+08	NA	0
6693	1	0
6708	NA	0
6710	NA	0
6711	1	0
55304	NA	0
5552	1	0
6717	1	0
284297	NA	0
55240	NA	0
2040	1	0
79987	NA	0
10492	1	0
8407	1	0
6950	1	0
7018	1	1
7037	1	0
7045	NA	0
7053	NA	0
7057	1	0
7060	NA	0
7086	1	0
7094	1	0
4071	NA	0
79073	1	0
130612	NA	0
51075	1	0
3371	NA	0
85456	1	0
7171	1	0
81619	1	0
23170	1	0
7276	1	1
84790	1	0
7277	1	0
203068	1	0
81027	1	0
7311	NA	0
7332	1	0
29979	1	0
1462	NA	0
7414	1	0
7417	1	0
7431	1	0
7448	1	1
7450	NA	1

51729	1	0
7520	1	0
7529	1	0
7531	1	0
7532	1	0
7533	1	0
7534	1	0

Table 3. Gene Ontology terms associated with T-cell-originated proteins identified in CD3(+)sEV

GO ID	Gene Ontology term	# all protei	# detected	p.val
GO:0070062	extracellular exosome	2210	146	1.09E-85
GO:1903561	extracellular vesicle	2307	146	4.68E-83
GO:0043230	extracellular organelle	2308	146	4.98E-83
GO:0065010	extracellular membrane-bounded organelle	2308	146	4.98E-83
GO:0031982	vesicle	4146	168	4.77E-70
GO:0005615	extracellular space	3652	153	1.26E-62
GO:0005576	extracellular region	4648	159	1.38E-53
GO:0030141	secretory granule	872	69	2.41E-40
GO:0099503	secretory vesicle	1041	71	2.50E-37
GO:0030055	cell-substrate junction	432	45	5.01E-31
GO:0005925	focal adhesion	422	44	2.37E-30
GO:0031410	cytoplasmic vesicle	2479	93	5.55E-29
GO:0097708	intracellular vesicle	2482	93	6.09E-29
GO:0050839	cell adhesion molecule binding	558	47	2.85E-28
GO:0005737	cytoplasm	12069	201	1.76E-24
GO:0005829	cytosol	5418	131	3.31E-24
GO:0034774	secretory granule lumen	322	34	1.06E-23
GO:0060205	cytoplasmic vesicle lumen	325	34	1.44E-23
GO:0031983	vesicle lumen	327	34	1.76E-23
GO:0070161	anchoring junction	1330	61	1.64E-22
GO:0045296	cadherin binding	333	32	4.37E-21
GO:0005515	protein binding	14027	211	5.69E-21
GO:0065008	regulation of biological quality	3773	102	7.56E-21
GO:0030054	cell junction	2112	73	6.06E-20
GO:0016020	membrane	9649	172	7.95E-20
GO:0005200	structural constituent of cytoskeleton	107	20	2.50E-19
GO:0007596	blood coagulation	225	26	2.64E-19
GO:0050817	coagulation	230	26	4.62E-19
GO:0007599	hemostasis	231	26	5.15E-19
GO:0034109	homotypic cell-cell adhesion	95	19	5.22E-19
GO:0005856	cytoskeleton	2374	76	6.92E-19
GO:0044877	protein-containing complex binding	1299	55	1.23E-18
GO:0003723	RNA binding	1971	68	2.01E-18
GO:0012505	endomembrane system	4715	111	2.58E-18
GO:0050878	regulation of body fluid levels	390	31	4.72E-18
GO:0005198	structural molecule activity	819	43	6.39E-18
GO:0101002	ficolin-1-rich granule	185	23	6.50E-18
GO:0042060	wound healing	442	32	2.03E-17
GO:0070820	tertiary granule	164	21	1.02E-16
GO:0042470	melanosome	110	18	1.97E-16
GO:0048770	pigment granule	110	18	1.97E-16
GO:0044419	biological process involved in interspecies interaction be	1726	60	2.69E-16
GO:0030168	platelet activation	133	19	3.87E-16

GO:0007155	cell adhesion	1539	56	4.86E-16
GO:0043227	membrane-bounded organelle	13883	203	6.43E-16
GO:0030667	secretory granule membrane	313	26	9.51E-16
GO:0070527	platelet aggregation	71	15	1.22E-15
GO:0071944	cell periphery	6372	127	1.48E-15
GO:0098609	cell-cell adhesion	957	43	1.76E-15
GO:0001533	cornified envelope	59	14	1.93E-15
GO:0099512	supramolecular fiber	1029	44	4.63E-15
GO:0043226	organelle	14728	208	4.90E-15
GO:0009611	response to wounding	577	33	6.13E-15
GO:0099081	supramolecular polymer	1038	44	6.32E-15
GO:0005488	binding	16619	220	7.27E-15
GO:0099080	supramolecular complex	1390	51	9.78E-15
GO:0042581	specific granule	160	19	1.27E-14
GO:0006996	organelle organization	3556	87	1.50E-14
GO:0032879	regulation of localization	2129	64	2.16E-14
GO:0098552	side of membrane	685	35	2.48E-14
GO:1904813	ficolin-1-rich granule lumen	124	17	2.92E-14
GO:0019899	enzyme binding	2086	63	2.99E-14
GO:0005886	plasma membrane	5891	118	3.54E-14
GO:0051179	localization	5370	111	4.95E-14
GO:0006950	response to stress	4044	93	4.96E-14
GO:0022607	cellular component assembly	3011	77	1.11E-13
GO:0051641	cellular localization	3351	82	1.34E-13
GO:0001775	cell activation	1153	44	2.50E-13
GO:0005911	cell-cell junction	505	29	2.81E-13
GO:0002376	immune system process	2892	74	4.11E-13
GO:0044403	biological process involved in symbiotic interaction	307	23	4.18E-13
GO:0140662	ATP-dependent protein folding chaperone	41	11	4.81E-13
GO:0045109	intermediate filament organization	69	13	4.91E-13
GO:0008104	protein localization	2469	67	5.71E-13
GO:0070727	cellular macromolecule localization	2479	67	6.90E-13
GO:0005622	intracellular anatomical structure	15464	210	7.54E-13
GO:0045104	intermediate filament cytoskeleton organization	89	14	8.30E-13
GO:0045103	intermediate filament-based process	90	14	9.74E-13
GO:0033036	macromolecule localization	2946	74	1.05E-12
GO:0009986	cell surface	962	39	1.11E-12
GO:0048518	positive regulation of biological process	6421	121	1.27E-12
GO:0051234	establishment of localization	4734	99	1.91E-12
GO:0050900	leukocyte migration	398	25	1.95E-12
GO:0010628	positive regulation of gene expression	1177	43	2.10E-12
GO:0016043	cellular component organization	6395	120	2.44E-12
GO:0098588	bounding membrane of organelle	2122	60	2.56E-12
GO:0051128	regulation of cellular component organization	2369	64	2.82E-12
GO:0044085	cellular component biogenesis	3273	78	2.91E-12
GO:0032502	developmental process	6527	121	4.34E-12
GO:0071840	cellular component organization or biogenesis	6601	121	1.00E-11

GO:0007010	cytoskeleton organization	1500	48	1.01E-11
GO:0006810	transport	4578	95	1.23E-11
GO:0048522	positive regulation of cellular process	5749	110	1.56E-11
GO:0005178	integrin binding	156	16	1.61E-11
GO:0097435	supramolecular fiber organization	815	34	1.74E-11
GO:0048856	anatomical structure development	5933	112	2.07E-11
GO:0007229	integrin-mediated signaling pathway	113	14	2.37E-11
GO:0050821	protein stabilization	214	18	2.42E-11
GO:1903829	positive regulation of protein localization	451	25	2.95E-11
GO:0032880	regulation of protein localization	842	34	4.20E-11
GO:0030855	epithelial cell differentiation	715	31	5.76E-11
GO:0043229	intracellular organelle	13770	193	6.94E-11
GO:0017111	ribonucleoside triphosphate phosphatase activity	677	30	7.15E-11
GO:0060341	regulation of cellular localization	955	36	7.20E-11
GO:0010833	telomere maintenance via telomere lengthening	81	12	7.68E-11
GO:0009607	response to biotic stimulus	1593	48	8.15E-11
GO:0051082	unfolded protein binding	124	14	8.46E-11
GO:0019900	kinase binding	781	32	1.16E-10
GO:0010033	response to organic substance	3109	72	1.16E-10
GO:0043232	intracellular non-membrane-bounded organelle	5700	107	1.35E-10
GO:0043228	non-membrane-bounded organelle	5701	107	1.37E-10
GO:0099513	polymeric cytoskeletal fiber	789	32	1.50E-10
GO:0030659	cytoplasmic vesicle membrane	1190	40	1.74E-10
GO:0044183	protein folding chaperone	68	11	1.80E-10
GO:0007004	telomere maintenance via telomerase	69	11	2.12E-10
GO:0009605	response to external stimulus	2886	68	2.28E-10
GO:0012506	vesicle membrane	1209	40	2.77E-10
GO:1904851	positive regulation of establishment of protein localizati	10	6	3.18E-10
GO:0043168	anion binding	2388	60	3.25E-10
GO:0070887	cellular response to chemical stimulus	3116	71	3.47E-10
GO:0051707	response to other organism	1548	46	3.47E-10
GO:0043207	response to external biotic stimulus	1551	46	3.70E-10
GO:0045111	intermediate filament cytoskeleton	253	18	3.81E-10
GO:0006518	peptide metabolic process	966	35	3.96E-10
GO:0006278	RNA-templated DNA biosynthetic process	75	11	5.39E-10
GO:0016462	pyrophosphatase activity	737	30	5.48E-10
GO:0016817	hydrolase activity, acting on acid anhydrides	738	30	5.65E-10
GO:0016818	hydrolase activity, acting on acid anhydrides, in phospho	738	30	5.65E-10
GO:0051247	positive regulation of protein metabolic process	1516	45	5.84E-10
GO:0030154	cell differentiation	4302	87	6.58E-10
GO:0019901	protein kinase binding	697	29	6.61E-10
GO:0072562	blood microparticle	145	14	6.92E-10
GO:0070203	regulation of establishment of protein localization to tel	11	6	6.94E-10
GO:1904869	regulation of protein localization to Cajal body	11	6	6.94E-10
GO:1904871	positive regulation of protein localization to Cajal body	11	6	6.94E-10
GO:0005832	chaperonin-containing T-complex	11	6	6.94E-10
GO:0060429	epithelium development	1204	39	8.81E-10

GO:0048869	cellular developmental process	4329	87	9.12E-10
GO:0016477	cell migration	1595	46	9.14E-10
GO:0030155	regulation of cell adhesion	803	31	9.90E-10
GO:1901265	nucleoside phosphate binding	2141	55	1.01E-09
GO:1904356	regulation of telomere maintenance via telomere length	61	10	1.08E-09
GO:0051050	positive regulation of transport	904	33	1.11E-09
GO:0051246	regulation of protein metabolic process	2668	63	1.26E-09
GO:0070202	regulation of establishment of protein localization to chr	12	6	1.38E-09
GO:1903405	protein localization to nuclear body	12	6	1.38E-09
GO:1904816	positive regulation of protein localization to chromosom	12	6	1.38E-09
GO:1904867	protein localization to Cajal body	12	6	1.38E-09
GO:0032501	multicellular organismal process	7674	128	1.38E-09
GO:0016032	viral process	421	22	1.46E-09
GO:0036094	small molecule binding	2482	60	1.48E-09
GO:0005102	signaling receptor binding	1565	45	1.59E-09
GO:0050896	response to stimulus	9216	145	1.66E-09
GO:0005882	intermediate filament	215	16	1.94E-09
GO:0042802	identical protein binding	1997	52	2.08E-09
GO:0044093	positive regulation of molecular function	1587	45	2.46E-09
GO:0001931	uropod	13	6	2.53E-09
GO:0031254	cell trailing edge	13	6	2.53E-09
GO:1900182	positive regulation of protein localization to nucleus	87	11	2.75E-09
GO:0000166	nucleotide binding	2140	54	2.85E-09
GO:0031647	regulation of protein stability	325	19	3.27E-09
GO:0010604	positive regulation of macromolecule metabolic process	3570	75	3.69E-09
GO:1904814	regulation of protein localization to chromosome, telom	14	6	4.39E-09
GO:1990173	protein localization to nucleoplasm	14	6	4.39E-09
GO:0031091	platelet alpha granule	91	11	4.47E-09
GO:0010543	regulation of platelet activation	52	9	4.55E-09
GO:0008037	cell recognition	230	16	5.13E-09
GO:1904358	positive regulation of telomere maintenance via telome	37	8	5.19E-09
GO:0051702	biological process involved in interaction with symbiont	116	12	5.35E-09
GO:0032210	regulation of telomere maintenance via telomerase	53	9	5.43E-09
GO:0009893	positive regulation of metabolic process	3897	79	5.93E-09
GO:0045727	positive regulation of translation	143	13	5.98E-09
GO:0098542	defense response to other organism	1183	37	6.51E-09
GO:0007163	establishment or maintenance of cell polarity	234	16	6.57E-09
GO:0006952	defense response	1820	48	6.76E-09
GO:1904874	positive regulation of telomerase RNA localization to Caj	15	6	7.25E-09
GO:0015629	actin cytoskeleton	502	23	7.42E-09
GO:0097159	organic cyclic compound binding	6330	110	7.52E-09
GO:1901363	heterocyclic compound binding	6259	109	8.38E-09
GO:0045216	cell-cell junction organization	208	15	9.65E-09
GO:0019904	protein domain specific binding	693	27	1.08E-08
GO:0003674	molecular_function	18432	223	1.09E-08
GO:0048870	cell motility	1791	47	1.18E-08
GO:0045184	establishment of protein localization	1671	45	1.19E-08

GO:0045095	keratin filament	101	11	1.36E-08
GO:0016192	vesicle-mediated transport	1621	44	1.42E-08
GO:0032991	protein-containing complex	6153	107	1.46E-08
GO:0034330	cell junction organization	705	27	1.55E-08
GO:0070200	establishment of protein localization to telomere	17	6	1.76E-08
GO:0033365	protein localization to organelle	917	31	2.23E-08
GO:0033043	regulation of organelle organization	1186	36	2.31E-08
GO:0006457	protein folding	223	15	2.46E-08
GO:0002181	cytoplasmic translation	161	13	2.51E-08
GO:1904872	regulation of telomerase RNA localization to Cajal body	18	6	2.62E-08
GO:0031424	keratinization	84	10	2.66E-08
GO:0070198	protein localization to chromosome, telomeric region	30	7	2.75E-08
GO:1900180	regulation of protein localization to nucleus	135	12	3.00E-08
GO:0007159	leukocyte cell-cell adhesion	414	20	3.12E-08
GO:0051049	regulation of transport	1791	46	3.36E-08
GO:0090670	RNA localization to Cajal body	19	6	3.79E-08
GO:0090671	telomerase RNA localization to Cajal body	19	6	3.79E-08
GO:0090672	telomerase RNA localization	19	6	3.79E-08
GO:0090685	RNA localization to nucleus	19	6	3.79E-08
GO:0051130	positive regulation of cellular component organization	1050	33	4.26E-08
GO:0034250	positive regulation of cellular amide metabolic process	170	13	4.79E-08
GO:1904951	positive regulation of establishment of protein localizati	310	17	5.76E-08
GO:0035579	specific granule membrane	91	10	5.78E-08
GO:0048584	positive regulation of response to stimulus	2272	53	5.93E-08
GO:0019538	protein metabolic process	5494	97	6.07E-08
GO:0044087	regulation of cellular component biogenesis	959	31	6.13E-08
GO:0005938	cell cortex	312	17	6.32E-08
GO:0035036	sperm-egg recognition	50	8	6.39E-08
GO:0031090	organelle membrane	3667	73	6.63E-08
GO:0032212	positive regulation of telomere maintenance via telome	34	7	6.99E-08
GO:0009988	cell-cell recognition	71	9	7.69E-08
GO:0061077	chaperone-mediated protein folding	71	9	7.69E-08
GO:0048583	regulation of response to stimulus	4131	79	8.31E-08
GO:0008219	cell death	2166	51	8.45E-08
GO:0051239	regulation of multicellular organismal process	2907	62	8.69E-08
GO:0019058	viral life cycle	319	17	8.71E-08
GO:2000278	regulation of DNA biosynthetic process	121	11	9.06E-08
GO:0070821	tertiary granule membrane	73	9	9.84E-08
GO:0043231	intracellular membrane-bounded organelle	12663	175	1.11E-07
GO:0045121	membrane raft	326	17	1.19E-07
GO:0098857	membrane microdomain	327	17	1.24E-07
GO:0000723	telomere maintenance	154	12	1.29E-07
GO:0030280	structural constituent of skin epidermis	37	7	1.30E-07
GO:0000377	RNA splicing, via transesterification reactions with bulge	329	17	1.35E-07
GO:0000398	mRNA splicing, via spliceosome	329	17	1.35E-07
GO:0031589	cell-substrate adhesion	369	18	1.36E-07
GO:1904724	tertiary granule lumen	55	8	1.38E-07

GO:0005773	vacuole	838	28	1.45E-07
GO:0042221	response to chemical	4494	83	1.54E-07
GO:0032204	regulation of telomere maintenance	101	10	1.57E-07
GO:0000375	RNA splicing, via transesterification reactions	333	17	1.61E-07
GO:0010810	regulation of cell-substrate adhesion	222	14	1.62E-07
GO:0006955	immune response	1955	47	1.68E-07
GO:0042592	homeostatic process	1705	43	1.72E-07
GO:0002682	regulation of immune system process	1525	40	1.82E-07
GO:0065003	protein-containing complex assembly	1650	42	1.92E-07
GO:0031625	ubiquitin protein ligase binding	299	16	2.00E-07
GO:0031252	cell leading edge	421	19	2.02E-07
GO:0070937	CRD-mediated mRNA stability complex	6	4	2.03E-07
GO:1902533	positive regulation of intracellular signal transduction	1013	31	2.06E-07
GO:0043933	protein-containing complex organization	1845	45	2.18E-07
GO:0043603	cellular amide metabolic process	1242	35	2.26E-07
GO:0043588	skin development	302	16	2.28E-07
GO:1901998	toxin transport	40	7	2.29E-07
GO:0051716	cellular response to stimulus	7653	121	2.36E-07
GO:0030139	endocytic vesicle	343	17	2.45E-07
GO:0006897	endocytosis	657	24	2.46E-07
GO:0043085	positive regulation of catalytic activity	1191	34	2.55E-07
GO:0140694	non-membrane-bounded organelle assembly	386	18	2.64E-07
GO:0030334	regulation of cell migration	1025	31	2.67E-07
GO:0007339	binding of sperm to zona pellucida	41	7	2.74E-07
GO:0034329	cell junction assembly	430	19	2.79E-07
GO:0009888	tissue development	1990	47	2.83E-07
GO:0030016	myofibril	233	14	2.92E-07
GO:0097367	carbohydrate derivative binding	2255	51	2.98E-07
GO:0048513	animal organ development	3591	70	3.24E-07
GO:0101031	chaperone complex	42	7	3.26E-07
GO:0012501	programmed cell death	2000	47	3.28E-07
GO:0045087	innate immune response	927	29	3.37E-07
GO:0070201	regulation of establishment of protein localization	526	21	3.46E-07
GO:0035639	purine ribonucleoside triphosphate binding	1813	44	3.52E-07
GO:0035580	specific granule lumen	62	8	3.60E-07
GO:0045335	phagocytic vesicle	139	11	3.73E-07
GO:0007160	cell-matrix adhesion	238	14	3.78E-07
GO:0051701	biological process involved in interaction with host	203	13	3.79E-07
GO:0030216	keratinocyte differentiation	170	12	3.79E-07
GO:0001894	tissue homeostasis	275	15	3.80E-07
GO:0001817	regulation of cytokine production	826	27	3.80E-07
GO:0042026	protein refolding	27	6	3.84E-07
GO:0070199	establishment of protein localization to chromosome	27	6	3.84E-07
GO:0023026	MHC class II protein complex binding	27	6	3.84E-07
GO:0006909	phagocytosis	314	16	3.86E-07
GO:0045785	positive regulation of cell adhesion	484	20	3.88E-07
GO:2000767	positive regulation of cytoplasmic translation	15	5	4.05E-07

GO:0001819	positive regulation of cytokine production	486	20	4.14E-07
GO:0050865	regulation of cell activation	727	25	4.20E-07
GO:0008092	cytoskeletal protein binding	993	30	4.34E-07
GO:0021762	substantia nigra development	44	7	4.54E-07
GO:0044389	ubiquitin-like protein ligase binding	318	16	4.57E-07
GO:0043292	contractile fiber	242	14	4.63E-07
GO:0001816	cytokine production	839	27	5.15E-07
GO:0008544	epidermis development	362	17	5.21E-07
GO:1990748	cellular detoxification	115	10	5.34E-07
GO:0032970	regulation of actin filament-based process	406	18	5.53E-07
GO:0003727	single-stranded RNA binding	89	9	5.54E-07
GO:0009987	cellular process	17266	213	5.64E-07
GO:0060627	regulation of vesicle-mediated transport	544	21	5.98E-07
GO:0051649	establishment of localization in cell	2108	48	6.01E-07
GO:0032206	positive regulation of telomere maintenance	67	8	6.62E-07
GO:0000323	lytic vacuole	746	25	6.75E-07
GO:0005764	lysosome	746	25	6.75E-07
GO:0030017	sarcomere	214	13	6.92E-07
GO:0071310	cellular response to organic substance	2464	53	7.92E-07
GO:0051240	positive regulation of multicellular organismal process	1553	39	7.98E-07
GO:0031092	platelet alpha granule membrane	17	5	8.20E-07
GO:0009897	external side of plasma membrane	462	19	8.25E-07
GO:0060249	anatomical structure homeostasis	333	16	8.42E-07
GO:0006915	apoptotic process	1941	45	9.00E-07
GO:0033993	response to lipid	918	28	9.05E-07
GO:0007043	cell-cell junction assembly	152	11	9.14E-07
GO:2000145	regulation of cell motility	1086	31	9.20E-07
GO:0002199	zona pellucida receptor complex	8	4	9.31E-07
GO:0032200	telomere organization	185	12	9.37E-07
GO:0003013	circulatory system process	609	22	9.66E-07
GO:0005525	GTP binding	379	17	9.82E-07
GO:0050776	regulation of immune response	922	28	9.86E-07
GO:0097237	cellular response to toxic substance	123	10	9.97E-07
GO:0051092	positive regulation of NF-kappaB transcription factor act	154	11	1.04E-06
GO:1990904	ribonucleoprotein complex	1152	32	1.08E-06
GO:0010941	regulation of cell death	1698	41	1.09E-06
GO:0005766	primary lysosome	155	11	1.11E-06
GO:0042582	azurophil granule	155	11	1.11E-06
GO:1902531	regulation of intracellular signal transduction	1766	42	1.17E-06
GO:0006412	translation	770	25	1.20E-06
GO:0017076	purine nucleotide binding	1897	44	1.21E-06
GO:0072678	T cell migration	73	8	1.29E-06
GO:0097530	granulocyte migration	158	11	1.34E-06
GO:0009653	anatomical structure morphogenesis	2790	57	1.42E-06
GO:0045123	cellular extravasation	74	8	1.43E-06
GO:2000573	positive regulation of DNA biosynthetic process	74	8	1.43E-06
GO:0034504	protein localization to nucleus	306	15	1.46E-06

GO:0098869	cellular oxidant detoxification	100	9	1.49E-06
GO:0050818	regulation of coagulation	75	8	1.59E-06
GO:0005681	spliceosomal complex	195	12	1.63E-06
GO:0002685	regulation of leukocyte migration	231	13	1.63E-06
GO:1990266	neutrophil migration	130	10	1.66E-06
GO:0071345	cellular response to cytokine stimulus	838	26	1.68E-06
GO:1901564	organonitrogen compound metabolic process	6535	105	1.70E-06
GO:0071702	organic substance transport	2466	52	1.89E-06
GO:0019220	regulation of phosphate metabolic process	1423	36	1.91E-06
GO:0071897	DNA biosynthetic process	198	12	1.92E-06
GO:0001618	virus receptor activity	77	8	1.94E-06
GO:0051174	regulation of phosphorus metabolic process	1424	36	1.95E-06
GO:0009913	epidermal cell differentiation	235	13	1.98E-06
GO:0051493	regulation of cytoskeleton organization	539	20	2.05E-06
GO:0032101	regulation of response to external stimulus	1014	29	2.06E-06
GO:0040012	regulation of locomotion	1130	31	2.11E-06
GO:0019001	guanyl nucleotide binding	401	17	2.12E-06
GO:0032561	guanyl ribonucleotide binding	401	17	2.12E-06
GO:0140272	exogenous protein binding	78	8	2.15E-06
GO:0035088	establishment or maintenance of apical/basal cell polari	55	7	2.17E-06
GO:0061245	establishment or maintenance of bipolar cell polarity	55	7	2.17E-06
GO:0005080	protein kinase C binding	55	7	2.17E-06
GO:0080134	regulation of response to stress	1431	36	2.18E-06
GO:0098590	plasma membrane region	1250	33	2.19E-06
GO:0006793	phosphorus metabolic process	2832	57	2.29E-06
GO:0072594	establishment of protein localization to organelle	449	18	2.32E-06
GO:0023023	MHC protein complex binding	36	6	2.33E-06
GO:0043043	peptide biosynthetic process	801	25	2.43E-06
GO:0032555	purine ribonucleotide binding	1883	43	2.46E-06
GO:1901700	response to oxygen-containing compound	1691	40	2.52E-06
GO:0150105	protein localization to cell-cell junction	21	5	2.60E-06
GO:0001895	retina homeostasis	80	8	2.61E-06
GO:1901698	response to nitrogen compound	1142	31	2.62E-06
GO:0065009	regulation of molecular function	3131	61	2.62E-06
GO:0042981	regulation of apoptotic process	1506	37	2.68E-06
GO:0002684	positive regulation of immune system process	971	28	2.69E-06
GO:0040011	locomotion	1445	36	2.72E-06
GO:0097529	myeloid leukocyte migration	242	13	2.73E-06
GO:0010572	positive regulation of platelet activation	10	4	2.75E-06
GO:0071705	nitrogen compound transport	2025	45	2.83E-06
GO:0071674	mononuclear cell migration	206	12	2.89E-06
GO:0042995	cell projection	2368	50	3.03E-06
GO:0032553	ribonucleotide binding	1900	43	3.10E-06
GO:0035089	establishment of apical/basal cell polarity	22	5	3.34E-06
GO:0046907	intracellular transport	1587	38	3.53E-06
GO:0098797	plasma membrane protein complex	713	23	3.66E-06
GO:0014070	response to organic cyclic compound	931	27	3.70E-06

GO:0030029	actin filament-based process	821	25	3.75E-06
GO:0006796	phosphate-containing compound metabolic process	2806	56	3.77E-06
GO:2001233	regulation of apoptotic signaling pathway	374	16	3.79E-06
GO:0003924	GTPase activity	331	15	3.83E-06
GO:0005775	vacuolar lumen	176	11	3.84E-06
GO:0034097	response to cytokine	933	27	3.85E-06
GO:0048731	system development	4454	78	4.01E-06
GO:0051129	negative regulation of cellular component organization	717	23	4.02E-06
GO:0016209	antioxidant activity	85	8	4.13E-06
GO:0051173	positive regulation of nitrogen compound metabolic pro	3175	61	4.15E-06
GO:0070934	CRD-mediated mRNA stabilization	11	4	4.28E-06
GO:0001726	ruffle	178	11	4.28E-06
GO:0030335	positive regulation of cell migration	566	20	4.28E-06
GO:0048523	negative regulation of cellular process	4934	84	4.31E-06
GO:0034113	heterotypic cell-cell adhesion	61	7	4.42E-06
GO:0005912	adherens junction	179	11	4.52E-06
GO:0043067	regulation of programmed cell death	1541	37	4.54E-06
GO:0043484	regulation of RNA splicing	180	11	4.77E-06
GO:0003925	G protein activity	41	6	5.14E-06
GO:0016787	hydrolase activity	2344	49	5.17E-06
GO:0006898	receptor-mediated endocytosis	257	13	5.28E-06
GO:0061162	establishment of monopolar cell polarity	24	5	5.29E-06
GO:0002687	positive regulation of leukocyte migration	148	10	5.34E-06
GO:0097193	intrinsic apoptotic signaling pathway	298	14	5.37E-06
GO:0044000	movement in host	183	11	5.59E-06
GO:0046983	protein dimerization activity	1070	29	5.87E-06
GO:0030010	establishment of cell polarity	150	10	6.02E-06
GO:0098631	cell adhesion mediator activity	64	7	6.12E-06
GO:0071013	catalytic step 2 spliceosome	90	8	6.35E-06
GO:1900152	negative regulation of nuclear-transcribed mRNA catabo	12	4	6.36E-06
GO:0015031	protein transport	1564	37	6.37E-06
GO:0061339	establishment or maintenance of monopolar cell polarit	25	5	6.56E-06
GO:0016887	ATP hydrolysis activity	347	15	6.77E-06
GO:0048857	neural nucleus development	65	7	6.79E-06
GO:0035578	azurophil granule lumen	91	8	6.89E-06
GO:0048871	multicellular organismal homeostasis	536	19	7.18E-06
GO:0051248	negative regulation of protein metabolic process	1141	30	7.25E-06
GO:0031623	receptor internalization	121	9	7.29E-06
GO:0005844	polysome	66	7	7.53E-06
GO:0051338	regulation of transferase activity	911	26	7.55E-06
GO:0098754	detoxification	154	10	7.60E-06
GO:0001906	cell killing	189	11	7.60E-06
GO:0031399	regulation of protein modification process	1579	37	7.89E-06
GO:1903311	regulation of mRNA metabolic process	309	14	8.12E-06
GO:2000147	positive regulation of cell motility	592	20	8.32E-06
GO:1903312	negative regulation of mRNA metabolic process	94	8	8.78E-06
GO:0030097	hemopoiesis	920	26	8.98E-06

GO:0022604	regulation of cell morphogenesis	312	14	9.05E-06
GO:0070486	leukocyte aggregation	13	4	9.11E-06
GO:0060255	regulation of macromolecule metabolic process	6762	105	9.40E-06
GO:0001952	regulation of cell-matrix adhesion	125	9	9.50E-06
GO:0019730	antimicrobial humoral response	125	9	9.50E-06
GO:0007044	cell-substrate junction assembly	95	8	9.50E-06
GO:0044409	entry into host	158	10	9.53E-06
GO:0030666	endocytic vesicle membrane	194	11	9.74E-06
GO:0097190	apoptotic signaling pathway	600	20	1.01E-05
GO:0044788	modulation by host of viral process	46	6	1.02E-05
GO:0110165	cellular anatomical entity	19469	225	1.03E-05
GO:0002831	regulation of response to biotic stimulus	361	15	1.08E-05
GO:0140657	ATP-dependent activity	552	19	1.09E-05
GO:0032587	ruffle membrane	97	8	1.11E-05
GO:0030193	regulation of blood coagulation	70	7	1.12E-05
GO:0044325	transmembrane transporter binding	128	9	1.15E-05
GO:0051972	regulation of telomerase activity	47	6	1.16E-05
GO:0008187	poly-pyrimidine tract binding	28	5	1.18E-05
GO:0040017	positive regulation of locomotion	607	20	1.20E-05
GO:0043254	regulation of protein-containing complex assembly	410	16	1.20E-05
GO:0032388	positive regulation of intracellular transport	199	11	1.24E-05
GO:0050794	regulation of cellular process	11335	155	1.25E-05
GO:0090666	scaRNA localization to Cajal body	5	3	1.26E-05
GO:1990779	glycoprotein Ib-IX-V complex	5	3	1.26E-05
GO:0010638	positive regulation of organelle organization	508	18	1.27E-05
GO:0071621	granulocyte chemotaxis	130	9	1.30E-05
GO:0061028	establishment of endothelial barrier	48	6	1.32E-05
GO:1900046	regulation of hemostasis	72	7	1.35E-05
GO:0044291	cell-cell contact zone	72	7	1.35E-05
GO:0009966	regulation of signal transduction	3075	58	1.37E-05
GO:2000765	regulation of cytoplasmic translation	29	5	1.41E-05
GO:0006403	RNA localization	202	11	1.42E-05
GO:0007275	multicellular organism development	4922	82	1.44E-05
GO:0009967	positive regulation of signal transduction	1559	36	1.47E-05
GO:0048519	negative regulation of biological process	5903	94	1.49E-05
GO:0150115	cell-substrate junction organization	101	8	1.49E-05
GO:0023056	positive regulation of signaling	1757	39	1.52E-05
GO:0098562	cytoplasmic side of membrane	204	11	1.56E-05
GO:0050793	regulation of developmental process	2584	51	1.59E-05
GO:0006801	superoxide metabolic process	74	7	1.62E-05
GO:0008380	RNA splicing	469	17	1.66E-05
GO:0014704	intercalated disc	50	6	1.67E-05
GO:0031974	membrane-enclosed lumen	6090	96	1.73E-05
GO:0043233	organelle lumen	6090	96	1.73E-05
GO:0070013	intracellular organelle lumen	6090	96	1.73E-05
GO:0009617	response to bacterium	786	23	1.77E-05
GO:0023051	regulation of signaling	3477	63	1.84E-05

GO:0043277	apoptotic cell clearance	51	6	1.88E-05
GO:0045197	establishment or maintenance of epithelial cell apical/ba	51	6	1.88E-05
GO:0003725	double-stranded RNA binding	76	7	1.93E-05
GO:0048534	hematopoietic or lymphoid organ development	962	26	1.95E-05
GO:0019222	regulation of metabolic process	7297	110	1.98E-05
GO:0022626	cytosolic ribosome	105	8	1.98E-05
GO:0061041	regulation of wound healing	137	9	1.99E-05
GO:1903034	regulation of response to wounding	172	10	2.00E-05
GO:2001242	regulation of intrinsic apoptotic signaling pathway	172	10	2.00E-05
GO:0002520	immune system development	1024	27	2.06E-05
GO:0120025	plasma membrane bounded cell projection	2258	46	2.09E-05
GO:0009898	cytoplasmic side of plasma membrane	173	10	2.10E-05
GO:0030593	neutrophil chemotaxis	106	8	2.13E-05
GO:2001252	positive regulation of chromosome organization	106	8	2.13E-05
GO:1990778	protein localization to cell periphery	337	14	2.14E-05
GO:0072359	circulatory system development	1209	30	2.20E-05
GO:0050684	regulation of mRNA processing	139	9	2.23E-05
GO:0062023	collagen-containing extracellular matrix	433	16	2.34E-05
GO:1903313	positive regulation of mRNA metabolic process	140	9	2.36E-05
GO:0008283	cell population proliferation	2062	43	2.37E-05
GO:0031325	positive regulation of cellular metabolic process	3133	58	2.40E-05
GO:0030863	cortical cytoskeleton	108	8	2.44E-05
GO:0050789	regulation of biological process	12322	164	2.45E-05
GO:0031256	leading edge membrane	177	10	2.56E-05
GO:1901701	cellular response to oxygen-containing compound	1220	30	2.60E-05
GO:0048024	regulation of mRNA splicing, via spliceosome	109	8	2.60E-05
GO:0050819	negative regulation of coagulation	54	6	2.62E-05
GO:0016071	mRNA metabolic process	751	22	2.67E-05
GO:0022407	regulation of cell-cell adhesion	490	17	2.90E-05
GO:0002252	immune effector process	700	21	2.91E-05
GO:0061844	antimicrobial humoral immune response mediated by ar	81	7	2.93E-05
GO:0017014	protein nitrosylation	17	4	2.93E-05
GO:0018119	peptidyl-cysteine S-nitrosylation	17	4	2.93E-05
GO:0034446	substrate adhesion-dependent cell spreading	111	8	2.97E-05
GO:0009056	catabolic process	2579	50	3.17E-05
GO:0051973	positive regulation of telomerase activity	34	5	3.17E-05
GO:0003676	nucleic acid binding	4309	73	3.18E-05
GO:0043604	amide biosynthetic process	932	25	3.22E-05
GO:0065007	biological regulation	13067	171	3.22E-05
GO:0001508	action potential	146	9	3.29E-05
GO:0010647	positive regulation of cell communication	1753	38	3.35E-05
GO:0010243	response to organonitrogen compound	1054	27	3.40E-05
GO:0032640	tumor necrosis factor production	184	10	3.57E-05
GO:0032680	regulation of tumor necrosis factor production	184	10	3.57E-05
GO:1901699	cellular response to nitrogen compound	711	21	3.64E-05
GO:0035633	maintenance of blood-brain barrier	35	5	3.67E-05
GO:2000406	positive regulation of T cell migration	35	5	3.67E-05

GO:0098576	lumenal side of membrane	35	5	3.67E-05
GO:0034248	regulation of cellular amide metabolic process	551	18	3.70E-05
GO:0051851	modulation by host of symbiont process	84	7	3.71E-05
GO:0051091	positive regulation of DNA-binding transcription factor a	266	12	3.78E-05
GO:0034502	protein localization to chromosome	115	8	3.84E-05
GO:0030135	coated vesicle	310	13	3.84E-05
GO:2000377	regulation of reactive oxygen species metabolic process	149	9	3.86E-05
GO:0035556	intracellular signal transduction	2673	51	3.96E-05
GO:0042175	nuclear outer membrane-endoplasmic reticulum membr	1186	29	3.98E-05
GO:0050778	positive regulation of immune response	607	19	4.01E-05
GO:0044092	negative regulation of molecular function	1187	29	4.04E-05
GO:0019725	cellular homeostasis	773	22	4.12E-05
GO:0008289	lipid binding	831	23	4.20E-05
GO:0018149	peptide cross-linking	36	5	4.22E-05
GO:0008150	biological_process	18903	221	4.25E-05
GO:0046718	viral entry into host cell	151	9	4.29E-05
GO:0008379	thioredoxin peroxidase activity	7	3	4.33E-05
GO:0140824	thioredoxin-dependent peroxiredoxin activity	7	3	4.33E-05
GO:0045321	leukocyte activation	1009	26	4.35E-05
GO:0002237	response to molecule of bacterial origin	360	14	4.41E-05
GO:0006397	mRNA processing	507	17	4.43E-05
GO:0071706	tumor necrosis factor superfamily cytokine production	189	10	4.48E-05
GO:1903555	regulation of tumor necrosis factor superfamily cytokine	189	10	4.48E-05
GO:0002544	chronic inflammatory response	19	4	4.69E-05
GO:0016310	phosphorylation	1853	39	4.98E-05
GO:0042325	regulation of phosphorylation	1264	30	5.01E-05
GO:0033627	cell adhesion mediated by integrin	88	7	5.01E-05
GO:0005840	ribosome	232	11	5.10E-05
GO:0031334	positive regulation of protein-containing complex assem	192	10	5.11E-05
GO:0032956	regulation of actin cytoskeleton organization	365	14	5.12E-05
GO:0048471	perinuclear region of cytoplasm	729	21	5.22E-05
GO:0042255	ribosome assembly	61	6	5.29E-05
GO:1900024	regulation of substrate adhesion-dependent cell spreadi	61	6	5.29E-05
GO:0101003	ficolin-1-rich granule membrane	61	6	5.29E-05
GO:0001786	phosphatidylserine binding	61	6	5.29E-05
GO:0030036	actin cytoskeleton organization	731	21	5.43E-05
GO:0072593	reactive oxygen species metabolic process	235	11	5.72E-05
GO:0050867	positive regulation of cell activation	467	16	5.79E-05
GO:0044794	positive regulation by host of viral process	20	4	5.81E-05
GO:0055065	metal ion homeostasis	519	17	5.92E-05
GO:0071675	regulation of mononuclear cell migration	123	8	6.20E-05
GO:0030901	midbrain development	91	7	6.21E-05
GO:0006605	protein targeting	325	13	6.22E-05
GO:0010646	regulation of cell communication	3464	61	6.23E-05
GO:0032481	positive regulation of type I interferon production	63	6	6.36E-05
GO:2001244	positive regulation of intrinsic apoptotic signaling pathw	63	6	6.36E-05
GO:0072676	lymphocyte migration	124	8	6.56E-05

GO:0097581	lamellipodium organization	92	7	6.66E-05
GO:0007338	single fertilization	160	9	6.73E-05
GO:0051920	peroxiredoxin activity	8	3	6.88E-05
GO:0045088	regulation of innate immune response	240	11	6.91E-05
GO:0010811	positive regulation of cell-substrate adhesion	125	8	6.95E-05
GO:0043280	positive regulation of cysteine-type endopeptidase activ	125	8	6.95E-05
GO:0051893	regulation of focal adhesion assembly	64	6	6.95E-05
GO:0090109	regulation of cell-substrate junction assembly	64	6	6.95E-05
GO:0005783	endoplasmic reticulum	2020	41	7.00E-05
GO:0098751	bone cell development	40	5	7.11E-05
GO:0005789	endoplasmic reticulum membrane	1163	28	7.14E-05
GO:0071407	cellular response to organic cyclic compound	581	18	7.32E-05
GO:0072659	protein localization to plasma membrane	285	12	7.35E-05
GO:0016363	nuclear matrix	126	8	7.35E-05
GO:0042803	protein homodimerization activity	691	20	7.42E-05
GO:0098827	endoplasmic reticulum subcompartment	1166	28	7.46E-05
GO:0001885	endothelial cell development	65	6	7.59E-05
GO:0050790	regulation of catalytic activity	2380	46	7.65E-05
GO:0006458	'de novo' protein folding	41	5	8.02E-05
GO:0022603	regulation of anatomical structure morphogenesis	987	25	8.10E-05
GO:0030027	lamellipodium	203	10	8.15E-05
GO:0097110	scaffold protein binding	66	6	8.28E-05
GO:0042742	defense response to bacterium	382	14	8.33E-05
GO:0071404	cellular response to low-density lipoprotein particle stim	22	4	8.63E-05
GO:0051604	protein maturation	336	13	8.71E-05
GO:0045807	positive regulation of endocytosis	96	7	8.74E-05
GO:0007009	plasma membrane organization	166	9	8.94E-05
GO:0006979	response to oxidative stress	434	15	8.96E-05
GO:0031093	platelet alpha granule lumen	67	6	9.01E-05
GO:2000403	positive regulation of lymphocyte migration	42	5	9.03E-05
GO:0006417	regulation of translation	486	16	9.22E-05
GO:0036464	cytoplasmic ribonucleoprotein granule	248	11	9.26E-05
GO:0009719	response to endogenous stimulus	1637	35	9.41E-05
GO:0032496	response to lipopolysaccharide	339	13	9.52E-05
GO:0009636	response to toxic substance	250	11	9.95E-05
GO:1902414	protein localization to cell junction	98	7	9.96E-05
GO:0003779	actin binding	439	15	1.02E-04
GO:0007597	blood coagulation, intrinsic pathway	9	3	1.02E-04
GO:0044829	positive regulation by host of viral genome replication	9	3	1.02E-04
GO:0005916	fascia adherens	9	3	1.02E-04
GO:0042612	MHC class I protein complex	9	3	1.02E-04
GO:0019430	removal of superoxide radicals	23	4	1.04E-04
GO:0031430	M band	23	4	1.04E-04
GO:0150116	regulation of cell-substrate junction organization	69	6	1.06E-04
GO:2000401	regulation of lymphocyte migration	69	6	1.06E-04
GO:0033044	regulation of chromosome organization	252	11	1.07E-04
GO:0005509	calcium ion binding	710	20	1.07E-04

GO:0043167	ion binding	6021	92	1.08E-04
GO:0031253	cell projection membrane	344	13	1.10E-04
GO:0055080	cation homeostasis	601	18	1.12E-04
GO:1900026	positive regulation of substrate adhesion-dependent cel	44	5	1.13E-04
GO:0062197	cellular response to chemical stress	345	13	1.13E-04
GO:0035606	peptidyl-cysteine S-trans-nitrosylation	2	2	1.18E-04
GO:0070488	neutrophil aggregation	2	2	1.18E-04
GO:0061337	cardiac conduction	101	7	1.20E-04
GO:0044089	positive regulation of cellular component biogenesis	498	16	1.22E-04
GO:0051054	positive regulation of DNA metabolic process	301	12	1.23E-04
GO:0030859	polarized epithelial cell differentiation	24	4	1.23E-04
GO:0062208	positive regulation of pattern recognition receptor signa	45	5	1.26E-04
GO:0022627	cytosolic small ribosomal subunit	45	5	1.26E-04
GO:0001525	angiogenesis	607	18	1.27E-04
GO:0005903	brush border	102	7	1.28E-04
GO:0071396	cellular response to lipid	608	18	1.29E-04
GO:0000902	cell morphogenesis	1018	25	1.31E-04
GO:0000904	cell morphogenesis involved in differentiation	724	20	1.39E-04
GO:0015911	long-chain fatty acid import across plasma membrane	10	3	1.45E-04
GO:0060368	regulation of Fc receptor mediated stimulatory signaling	10	3	1.45E-04
GO:0050815	phosphoserine residue binding	10	3	1.45E-04
GO:0002699	positive regulation of immune effector process	261	11	1.45E-04
GO:0003015	heart process	261	11	1.45E-04
GO:0071450	cellular response to oxygen radical	25	4	1.45E-04
GO:0071451	cellular response to superoxide	25	4	1.45E-04
GO:0030057	desmosome	25	4	1.45E-04
GO:0030032	lamellipodium assembly	73	6	1.46E-04
GO:0071677	positive regulation of mononuclear cell migration	73	6	1.46E-04
GO:0007015	actin filament organization	454	15	1.47E-04
GO:0051090	regulation of DNA-binding transcription factor activity	454	15	1.47E-04
GO:0098771	inorganic ion homeostasis	616	18	1.52E-04
GO:0032760	positive regulation of tumor necrosis factor production	105	7	1.54E-04
GO:0050766	positive regulation of phagocytosis	74	6	1.57E-04
GO:0006954	inflammatory response	848	22	1.58E-04
GO:0035770	ribonucleoprotein granule	265	11	1.66E-04
GO:0071222	cellular response to lipopolysaccharide	222	10	1.70E-04
GO:0033622	integrin activation	26	4	1.70E-04
GO:0048026	positive regulation of mRNA splicing, via spliceosome	26	4	1.70E-04
GO:1900151	regulation of nuclear-transcribed mRNA catabolic proce:	26	4	1.70E-04
GO:0031984	organelle subcompartment	1486	32	1.71E-04
GO:0062207	regulation of pattern recognition receptor signaling path	107	7	1.73E-04
GO:0010562	positive regulation of phosphorus metabolic process	914	23	1.74E-04
GO:0043066	negative regulation of apoptotic process	914	23	1.74E-04
GO:0045937	positive regulation of phosphate metabolic process	914	23	1.74E-04
GO:2001056	positive regulation of cysteine-type endopeptidase activ	143	8	1.78E-04
GO:0006090	pyruvate metabolic process	108	7	1.83E-04
GO:0019882	antigen processing and presentation	108	7	1.83E-04

GO:0032231	regulation of actin filament bundle assembly	108	7	1.83E-04
GO:0050801	ion homeostasis	626	18	1.85E-04
GO:0032103	positive regulation of response to external stimulus	464	15	1.86E-04
GO:2000404	regulation of T cell migration	49	5	1.90E-04
GO:0060548	negative regulation of cell death	1043	25	1.91E-04
GO:1903557	positive regulation of tumor necrosis factor superfamily	109	7	1.94E-04
GO:0031012	extracellular matrix	573	17	1.94E-04
GO:0045089	positive regulation of innate immune response	145	8	1.95E-04
GO:0034399	nuclear periphery	145	8	1.95E-04
GO:0031400	negative regulation of protein modification process	519	16	1.95E-04
GO:0034121	regulation of toll-like receptor signaling pathway	77	6	1.96E-04
GO:0015935	small ribosomal subunit	77	6	1.96E-04
GO:0030670	phagocytic vesicle membrane	77	6	1.96E-04
GO:0051651	maintenance of location in cell	226	10	1.96E-04
GO:0071803	positive regulation of podosome assembly	11	3	1.98E-04
GO:0031639	plasminogen activation	27	4	1.98E-04
GO:0042730	fibrinolysis	27	4	1.98E-04
GO:0061436	establishment of skin barrier	27	4	1.98E-04
GO:0030312	external encapsulating structure	574	17	1.98E-04
GO:0007165	signal transduction	6120	92	2.03E-04
GO:0002009	morphogenesis of an epithelium	521	16	2.04E-04
GO:0007520	myoblast fusion	50	5	2.09E-04
GO:0018198	peptidyl-cysteine modification	50	5	2.09E-04
GO:0030195	negative regulation of blood coagulation	50	5	2.09E-04
GO:0061045	negative regulation of wound healing	78	6	2.10E-04
GO:0048729	tissue morphogenesis	633	18	2.12E-04
GO:0051171	regulation of nitrogen compound metabolic process	5792	88	2.13E-04
GO:0006959	humoral immune response	320	12	2.17E-04
GO:0008015	blood circulation	524	16	2.18E-04
GO:0006886	intracellular protein transport	868	22	2.19E-04
GO:0002221	pattern recognition receptor signaling pathway	187	9	2.20E-04
GO:0006875	cellular metal ion homeostasis	420	14	2.23E-04
GO:0032432	actin filament bundle	79	6	2.25E-04
GO:0002833	positive regulation of response to biotic stimulus	188	9	2.29E-04
GO:0044391	ribosomal subunit	188	9	2.29E-04
GO:0000303	response to superoxide	28	4	2.29E-04
GO:0022409	positive regulation of cell-cell adhesion	322	12	2.30E-04
GO:0034332	adherens junction organization	51	5	2.30E-04
GO:1900047	negative regulation of hemostasis	51	5	2.30E-04
GO:0072503	cellular divalent inorganic cation homeostasis	323	12	2.36E-04
GO:0001932	regulation of protein phosphorylation	1121	26	2.37E-04
GO:0045862	positive regulation of proteolysis	372	13	2.37E-04
GO:0006811	ion transport	1581	33	2.38E-04
GO:0010959	regulation of metal ion transport	423	14	2.40E-04
GO:0070925	organelle assembly	935	23	2.41E-04
GO:0030864	cortical actin cytoskeleton	80	6	2.41E-04
GO:0048878	chemical homeostasis	1060	25	2.44E-04

GO:0043069	negative regulation of programmed cell death	938	23	2.52E-04
GO:0098636	protein complex involved in cell adhesion	52	5	2.52E-04
GO:0072657	protein localization to membrane	586	17	2.53E-04
GO:0001944	vasculature development	818	21	2.57E-04
GO:0048306	calcium-dependent protein binding	81	6	2.58E-04
GO:0071219	cellular response to molecule of bacterial origin	234	10	2.60E-04
GO:0048514	blood vessel morphogenesis	701	19	2.60E-04
GO:0008152	metabolic process	12433	161	2.61E-04
GO:0035325	Toll-like receptor binding	12	3	2.62E-04
GO:0086080	protein binding involved in heterotypic cell-cell adhesior	12	3	2.62E-04
GO:0000305	response to oxygen radical	29	4	2.64E-04
GO:0010592	positive regulation of lamellipodium assembly	29	4	2.64E-04
GO:0034123	positive regulation of toll-like receptor signaling pathwa	29	4	2.64E-04
GO:0051873	killing by host of symbiont cells	29	4	2.64E-04
GO:0071556	integral component of lumenal side of endoplasmic retic	29	4	2.64E-04
GO:0098553	lumenal side of endoplasmic reticulum membrane	29	4	2.64E-04
GO:2000116	regulation of cysteine-type endopeptidase activity	235	10	2.69E-04
GO:0098796	membrane protein complex	1328	29	2.81E-04
GO:0002697	regulation of immune effector process	379	13	2.84E-04
GO:0006096	glycolytic process	83	6	2.95E-04
GO:0061515	myeloid cell development	83	6	2.95E-04
GO:0010256	endomembrane system organization	594	17	2.96E-04
GO:0098632	cell-cell adhesion mediator activity	54	5	3.02E-04
GO:0002474	antigen processing and presentation of peptide antigen	30	4	3.02E-04
GO:0002102	podosome	30	4	3.02E-04
GO:0006757	ATP generation from ADP	84	6	3.15E-04
GO:0050830	defense response to Gram-positive bacterium	118	7	3.16E-04
GO:0030595	leukocyte chemotaxis	240	10	3.18E-04
GO:0010761	fibroblast migration	55	5	3.29E-04
GO:0042743	hydrogen peroxide metabolic process	55	5	3.29E-04
GO:0004601	peroxidase activity	55	5	3.29E-04
GO:0045202	synapse	1341	29	3.30E-04
GO:0090316	positive regulation of intracellular protein transport	157	8	3.35E-04
GO:0048041	focal adhesion assembly	85	6	3.36E-04
GO:0030662	coated vesicle membrane	198	9	3.36E-04
GO:0032386	regulation of intracellular transport	336	12	3.38E-04
GO:0034599	cellular response to oxidative stress	288	11	3.39E-04
GO:0009205	purine ribonucleoside triphosphate metabolic process	242	10	3.40E-04
GO:0033561	regulation of water loss via skin	31	4	3.44E-04
GO:0008305	integrin complex	31	4	3.44E-04
GO:0042788	polysomal ribosome	31	4	3.44E-04
GO:0080090	regulation of primary metabolic process	5954	89	3.46E-04
GO:0043269	regulation of ion transport	718	19	3.50E-04
GO:0150106	regulation of protein localization to cell-cell junction	3	2	3.53E-04
GO:0016049	cell growth	493	15	3.55E-04
GO:0006816	calcium ion transport	440	14	3.57E-04
GO:0045936	negative regulation of phosphate metabolic process	440	14	3.57E-04

GO:0042110	T cell activation	548	16	3.58E-04
GO:0050832	defense response to fungus	56	5	3.58E-04
GO:0050840	extracellular matrix binding	56	5	3.58E-04
GO:0051336	regulation of hydrolase activity	1025	24	3.62E-04
GO:0010563	negative regulation of phosphorus metabolic process	441	14	3.66E-04
GO:0009566	fertilization	201	9	3.75E-04
GO:0010232	vascular transport	87	6	3.80E-04
GO:0150104	transport across blood-brain barrier	87	6	3.80E-04
GO:0051017	actin filament bundle assembly	160	8	3.81E-04
GO:0001649	osteoblast differentiation	246	10	3.86E-04
GO:0006749	glutathione metabolic process	57	5	3.89E-04
GO:0061756	leukocyte adhesion to vascular endothelial cell	57	5	3.89E-04
GO:0016684	oxidoreductase activity, acting on peroxide as acceptor	57	5	3.89E-04
GO:0001568	blood vessel development	784	20	3.92E-04
GO:0051235	maintenance of location	342	12	3.96E-04
GO:0009144	purine nucleoside triphosphate metabolic process	247	10	3.99E-04
GO:0030496	midbody	203	9	4.03E-04
GO:0030003	cellular cation homeostasis	499	15	4.03E-04
GO:0051222	positive regulation of protein transport	294	11	4.04E-04
GO:0002224	toll-like receptor signaling pathway	123	7	4.06E-04
GO:1901888	regulation of cell junction assembly	204	9	4.18E-04
GO:0009199	ribonucleoside triphosphate metabolic process	249	10	4.25E-04
GO:0071801	regulation of podosome assembly	14	3	4.26E-04
GO:0031323	regulation of cellular metabolic process	5737	86	4.27E-04
GO:0010942	positive regulation of cell death	614	17	4.32E-04
GO:0043281	regulation of cysteine-type endopeptidase activity invol	205	9	4.33E-04
GO:0002764	immune response-regulating signaling pathway	503	15	4.38E-04
GO:0060047	heart contraction	250	10	4.39E-04
GO:0002431	Fc receptor mediated stimulatory signaling pathway	33	4	4.39E-04
GO:0050901	leukocyte tethering or rolling	33	4	4.39E-04
GO:0061572	actin filament bundle organization	164	8	4.49E-04
GO:0016667	oxidoreductase activity, acting on a sulfur group of dono	59	5	4.57E-04
GO:0044248	cellular catabolic process	2134	40	4.59E-04
GO:0005887	integral component of plasma membrane	1713	34	4.76E-04
GO:0055094	response to lipoprotein particle	34	4	4.94E-04
GO:0090322	regulation of superoxide metabolic process	34	4	4.94E-04
GO:0048646	anatomical structure formation involved in morphogene	1242	27	4.94E-04
GO:0030100	regulation of endocytosis	209	9	4.98E-04
GO:0031347	regulation of defense response	680	18	4.99E-04
GO:0006936	muscle contraction	351	12	5.00E-04
GO:0046031	ADP metabolic process	92	6	5.14E-04
GO:0005634	nucleus	8188	114	5.18E-04
GO:0048255	mRNA stabilization	61	5	5.34E-04
GO:0086065	cell communication involved in cardiac conduction	61	5	5.34E-04
GO:0098900	regulation of action potential	61	5	5.34E-04
GO:0031226	intrinsic component of plasma membrane	1794	35	5.34E-04
GO:0050685	positive regulation of mRNA processing	35	4	5.53E-04

GO:0030099	myeloid cell differentiation	407	13	5.58E-04
GO:0072507	divalent inorganic cation homeostasis	356	12	5.66E-04
GO:0019079	viral genome replication	130	7	5.67E-04
GO:0005774	vacuolar membrane	461	14	5.67E-04
GO:0051094	positive regulation of developmental process	1387	29	5.69E-04
GO:0035637	multicellular organismal signaling	170	8	5.70E-04
GO:0006873	cellular ion homeostasis	516	15	5.70E-04
GO:0060284	regulation of cell development	516	15	5.70E-04
GO:0032233	positive regulation of actin filament bundle assembly	62	5	5.75E-04
GO:0046824	positive regulation of nucleocytoplasmic transport	62	5	5.75E-04
GO:0035304	regulation of protein dephosphorylation	94	6	5.76E-04
GO:1903035	negative regulation of response to wounding	94	6	5.76E-04
GO:0072341	modified amino acid binding	94	6	5.76E-04
GO:0031349	positive regulation of defense response	307	11	5.79E-04
GO:2000112	regulation of cellular macromolecule biosynthetic proce	574	16	5.91E-04
GO:0071216	cellular response to biotic stimulus	261	10	6.13E-04
GO:0019897	extrinsic component of plasma membrane	172	8	6.15E-04
GO:0002504	antigen processing and presentation of peptide or polys	36	4	6.16E-04
GO:0031672	A band	36	4	6.16E-04
GO:0031663	lipopolysaccharide-mediated signaling pathway	63	5	6.20E-04
GO:0032615	interleukin-12 production	63	5	6.20E-04
GO:0032655	regulation of interleukin-12 production	63	5	6.20E-04
GO:0006735	NADH regeneration	16	3	6.45E-04
GO:0034112	positive regulation of homotypic cell-cell adhesion	16	3	6.45E-04
GO:0061621	canonical glycolysis	16	3	6.45E-04
GO:0061684	chaperone-mediated autophagy	16	3	6.45E-04
GO:0061718	glucose catabolic process to pyruvate	16	3	6.45E-04
GO:0035303	regulation of dephosphorylation	133	7	6.50E-04
GO:0046034	ATP metabolic process	217	9	6.52E-04
GO:2000026	regulation of multicellular organismal development	1467	30	6.53E-04
GO:0006937	regulation of muscle contraction	174	8	6.64E-04
GO:0010950	positive regulation of endopeptidase activity	174	8	6.64E-04
GO:0000768	syncytium formation by plasma membrane fusion	64	5	6.66E-04
GO:0048002	antigen processing and presentation of peptide antigen	64	5	6.66E-04
GO:0140253	cell-cell fusion	64	5	6.66E-04
GO:0061024	membrane organization	881	21	6.79E-04
GO:0034110	regulation of homotypic cell-cell adhesion	37	4	6.85E-04
GO:0044319	wound healing, spreading of cells	37	4	6.85E-04
GO:0051084	'de novo' post-translational protein folding	37	4	6.85E-04
GO:0071402	cellular response to lipoprotein particle stimulus	37	4	6.85E-04
GO:0090505	epiboly involved in wound healing	37	4	6.85E-04
GO:1902745	positive regulation of lamellipodium organization	37	4	6.85E-04
GO:0001530	lipopolysaccharide binding	37	4	6.85E-04
GO:0043488	regulation of mRNA stability	175	8	6.89E-04
GO:0010041	response to iron(III) ion	4	2	7.00E-04
GO:0071787	endoplasmic reticulum tubular network formation	4	2	7.00E-04
GO:0036457	keratohyalin granule	4	2	7.00E-04

GO:0097452	GAIT complex	4	2	7.00E-04
GO:0033677	DNA/RNA helicase activity	4	2	7.00E-04
GO:0035662	Toll-like receptor 4 binding	4	2	7.00E-04
GO:0051022	Rho GDP-dissociation inhibitor binding	4	2	7.00E-04
GO:0070051	fibrinogen binding	4	2	7.00E-04
GO:0070053	thrombospondin receptor activity	4	2	7.00E-04
GO:2001235	positive regulation of apoptotic signaling pathway	135	7	7.10E-04
GO:0005765	lysosomal membrane	418	13	7.14E-04
GO:0098852	lytic vacuole membrane	418	13	7.14E-04
GO:0003735	structural constituent of ribosome	176	8	7.15E-04
GO:0005575	cellular_component	19869	225	7.23E-04
GO:0009141	nucleoside triphosphate metabolic process	267	10	7.30E-04
GO:0007154	cell communication	6682	96	7.45E-04
GO:0019693	ribose phosphate metabolic process	475	14	7.59E-04
GO:0090504	epiboly	38	4	7.59E-04
GO:0051347	positive regulation of transferase activity	588	16	7.64E-04
GO:0042327	positive regulation of phosphorylation	828	20	7.75E-04
GO:0006887	exocytosis	369	12	7.75E-04
GO:0010763	positive regulation of fibroblast migration	17	3	7.77E-04
GO:0019885	antigen processing and presentation of endogenous pep	17	3	7.77E-04
GO:0033631	cell-cell adhesion mediated by integrin	17	3	7.77E-04
GO:0044539	long-chain fatty acid import into cell	17	3	7.77E-04
GO:0045198	establishment of epithelial cell apical/basal polarity	17	3	7.77E-04
GO:0072378	blood coagulation, fibrin clot formation	17	3	7.77E-04
GO:0090136	epithelial cell-cell adhesion	17	3	7.77E-04
GO:0051345	positive regulation of hydrolase activity	589	16	7.78E-04
GO:0043065	positive regulation of apoptotic process	532	15	7.78E-04
GO:1901575	organic substance catabolic process	2120	39	7.84E-04
GO:0006165	nucleoside diphosphate phosphorylation	100	6	7.99E-04
GO:0010769	regulation of cell morphogenesis involved in differentiat	100	6	7.99E-04
GO:0009620	response to fungus	67	5	8.22E-04
GO:0009595	detection of biotic stimulus	39	4	8.39E-04
GO:0045730	respiratory burst	39	4	8.39E-04
GO:0033157	regulation of intracellular protein transport	225	9	8.43E-04
GO:0003158	endothelium development	139	7	8.43E-04
GO:0023052	signaling	6622	95	8.59E-04
GO:0000288	nuclear-transcribed mRNA catabolic process, deadenylat	68	5	8.79E-04
GO:0006949	syncytium formation	68	5	8.79E-04
GO:0002521	leukocyte differentiation	596	16	8.81E-04
GO:0046939	nucleotide phosphorylation	102	6	8.86E-04
GO:0005811	lipid droplet	102	6	8.86E-04
GO:0098978	glutamatergic synapse	324	11	9.01E-04
GO:0071417	cellular response to organonitrogen compound	656	17	9.01E-04
GO:0048585	negative regulation of response to stimulus	1706	33	9.04E-04
GO:0051052	regulation of DNA metabolic process	540	15	9.05E-04
GO:0050863	regulation of T cell activation	376	12	9.11E-04
GO:0015630	microtubule cytoskeleton	1362	28	9.22E-04

GO:0002478	antigen processing and presentation of exogenous pepti	40	4	9.24E-04
GO:0010762	regulation of fibroblast migration	40	4	9.24E-04
GO:0033120	positive regulation of RNA splicing	40	4	9.24E-04
GO:0010755	regulation of plasminogen activation	18	3	9.25E-04
GO:0019081	viral translation	18	3	9.25E-04
GO:0051580	regulation of neurotransmitter uptake	18	3	9.25E-04
GO:0051988	regulation of attachment of spindle microtubules to kine	18	3	9.25E-04
GO:0061620	glycolytic process through glucose-6-phosphate	18	3	9.25E-04
GO:0071786	endoplasmic reticulum tubular network organization	18	3	9.25E-04
GO:1902001	fatty acid transmembrane transport	18	3	9.25E-04
GO:0034185	apolipoprotein binding	18	3	9.25E-04
GO:0050764	regulation of phagocytosis	103	6	9.33E-04
GO:0016323	basolateral plasma membrane	229	9	9.55E-04
GO:0009135	purine nucleoside diphosphate metabolic process	104	6	9.81E-04
GO:0009179	purine ribonucleoside diphosphate metabolic process	104	6	9.81E-04
GO:0110053	regulation of actin filament organization	278	10	9.93E-04
GO:0055076	transition metal ion homeostasis	143	7	9.96E-04
GO:0043489	RNA stabilization	70	5	1.00E-03
GO:1902373	negative regulation of mRNA catabolic process	70	5	1.00E-03
GO:0001725	stress fiber	70	5	1.00E-03
GO:0097517	contractile actin filament bundle	70	5	1.00E-03
GO:0046649	lymphocyte activation	846	20	1.01E-03
GO:0045454	cell redox homeostasis	41	4	1.02E-03
GO:0006468	protein phosphorylation	1578	31	1.02E-03
GO:0043068	positive regulation of programmed cell death	547	15	1.03E-03
GO:0032479	regulation of type I interferon production	105	6	1.03E-03
GO:0032606	type I interferon production	105	6	1.03E-03
GO:0043549	regulation of kinase activity	786	19	1.04E-03
GO:0032102	negative regulation of response to external stimulus	436	13	1.05E-03
GO:0043487	regulation of RNA stability	187	8	1.06E-03
GO:0001667	ameboidal-type cell migration	492	14	1.06E-03
GO:0048468	cell development	2155	39	1.07E-03
GO:2000379	positive regulation of reactive oxygen species metabolic	71	5	1.07E-03
GO:2001234	negative regulation of apoptotic signaling pathway	233	9	1.08E-03
GO:0000028	ribosomal small subunit assembly	19	3	1.09E-03
GO:0002483	antigen processing and presentation of endogenous pep	19	3	1.09E-03
GO:0002523	leukocyte migration involved in inflammatory response	19	3	1.09E-03
GO:0032930	positive regulation of superoxide anion generation	19	3	1.09E-03
GO:0061615	glycolytic process through fructose-6-phosphate	19	3	1.09E-03
GO:0071800	podosome assembly	19	3	1.09E-03
GO:0099563	modification of synaptic structure	19	3	1.09E-03
GO:0140354	lipid import into cell	19	3	1.09E-03
GO:0003746	translation elongation factor activity	19	3	1.09E-03
GO:1902903	regulation of supramolecular fiber organization	384	12	1.09E-03
GO:0051276	chromosome organization	609	16	1.10E-03
GO:0010591	regulation of lamellipodium assembly	42	4	1.11E-03
GO:0014002	astrocyte development	42	4	1.11E-03

GO:0019320	hexose catabolic process	42	4	1.11E-03
GO:0010468	regulation of gene expression	5401	80	1.13E-03
GO:0060369	positive regulation of Fc receptor mediated stimulatory	5	2	1.16E-03
GO:0071593	lymphocyte aggregation	5	2	1.16E-03
GO:0098708	glucose import across plasma membrane	5	2	1.16E-03
GO:1905449	regulation of Fc-gamma receptor signaling pathway invo	5	2	1.16E-03
GO:1905912	regulation of calcium ion export across plasma membrar	5	2	1.16E-03
GO:1905913	negative regulation of calcium ion export across plasma	5	2	1.16E-03
GO:2000371	regulation of DNA topoisomerase (ATP-hydrolyzing) acti	5	2	1.16E-03
GO:2000373	positive regulation of DNA topoisomerase (ATP-hydroly	5	2	1.16E-03
GO:0106002	mCRD-mediated mRNA stability complex	5	2	1.16E-03
GO:0004459	L-lactate dehydrogenase activity	5	2	1.16E-03
GO:0019960	C-X3-C chemokine binding	5	2	1.16E-03
GO:0038132	neuregulin binding	5	2	1.16E-03
GO:0051021	GDP-dissociation inhibitor binding	5	2	1.16E-03
GO:0086083	cell adhesive protein binding involved in bundle of His ce	5	2	1.16E-03
GO:0140545	ATP-dependent protein disaggregase activity	5	2	1.16E-03
GO:0006874	cellular calcium ion homeostasis	284	10	1.17E-03
GO:0061013	regulation of mRNA catabolic process	190	8	1.17E-03
GO:0008284	positive regulation of cell population proliferation	986	22	1.20E-03
GO:0043086	negative regulation of catalytic activity	796	19	1.20E-03
GO:0016529	sarcoplasmic reticulum	73	5	1.21E-03
GO:0002274	myeloid leukocyte activation	237	9	1.21E-03
GO:0016491	oxidoreductase activity	735	18	1.21E-03
GO:0002347	response to tumor cell	43	4	1.22E-03
GO:0042554	superoxide anion generation	43	4	1.22E-03
GO:0044238	primary metabolic process	10825	141	1.24E-03
GO:0046822	regulation of nucleocytoplasmic transport	109	6	1.25E-03
GO:0051223	regulation of protein transport	501	14	1.26E-03
GO:0044237	cellular metabolic process	10271	135	1.27E-03
GO:0046931	pore complex assembly	20	3	1.27E-03
GO:0072376	protein activation cascade	20	3	1.27E-03
GO:0034762	regulation of transmembrane transport	618	16	1.28E-03
GO:0042267	natural killer cell mediated cytotoxicity	74	5	1.29E-03
GO:0048545	response to steroid hormone	339	11	1.30E-03
GO:0051668	localization within membrane	679	17	1.31E-03
GO:0071704	organic substance metabolic process	11974	153	1.31E-03
GO:1901020	negative regulation of calcium ion transmembrane trans	44	4	1.33E-03
GO:0015036	disulfide oxidoreductase activity	44	4	1.33E-03
GO:0007417	central nervous system development	1060	23	1.34E-03
GO:0010952	positive regulation of peptidase activity	194	8	1.34E-03
GO:0006091	generation of precursor metabolites and energy	505	14	1.36E-03
GO:0031532	actin cytoskeleton reorganization	111	6	1.37E-03
GO:0044297	cell body	565	15	1.42E-03
GO:0008631	intrinsic apoptotic signaling pathway in response to oxid	45	4	1.44E-03
GO:0002253	activation of immune response	397	12	1.45E-03
GO:0044233	mitochondria-associated endoplasmic reticulum membr	21	3	1.47E-03

GO:0097038	perinuclear endoplasmic reticulum	21	3	1.47E-03
GO:0035613	RNA stem-loop binding	21	3	1.47E-03
GO:0070588	calcium ion transmembrane transport	345	11	1.49E-03
GO:0030162	regulation of proteolysis	750	18	1.52E-03
GO:0008360	regulation of cell shape	154	7	1.53E-03
GO:0003012	muscle system process	455	13	1.54E-03
GO:0002228	natural killer cell mediated immunity	77	5	1.54E-03
GO:1903036	positive regulation of response to wounding	77	5	1.54E-03
GO:0001934	positive regulation of protein phosphorylation	751	18	1.54E-03
GO:0031640	killing of cells of another organism	46	4	1.57E-03
GO:0046365	monosaccharide catabolic process	46	4	1.57E-03
GO:0002824	positive regulation of adaptive immune response based	114	6	1.58E-03
GO:0009185	ribonucleoside diphosphate metabolic process	114	6	1.58E-03
GO:0035239	tube morphogenesis	943	21	1.58E-03
GO:0016052	carbohydrate catabolic process	155	7	1.59E-03
GO:0070661	leukocyte proliferation	348	11	1.60E-03
GO:0043393	regulation of protein binding	200	8	1.63E-03
GO:0055117	regulation of cardiac muscle contraction	78	5	1.63E-03
GO:0042641	actomyosin	78	5	1.63E-03
GO:0042127	regulation of cell population proliferation	1769	33	1.65E-03
GO:0005996	monosaccharide metabolic process	248	9	1.66E-03
GO:0006402	mRNA catabolic process	248	9	1.66E-03
GO:0052547	regulation of peptidase activity	459	13	1.66E-03
GO:0032271	regulation of protein polymerization	201	8	1.68E-03
GO:0032928	regulation of superoxide anion generation	22	3	1.69E-03
GO:0035855	megakaryocyte development	22	3	1.69E-03
GO:0051220	cytoplasmic sequestering of protein	22	3	1.69E-03
GO:0090665	glycoprotein complex	22	3	1.69E-03
GO:0042288	MHC class I protein binding	22	3	1.69E-03
GO:0055106	ubiquitin-protein transferase regulator activity	22	3	1.69E-03
GO:0045646	regulation of erythrocyte differentiation	47	4	1.70E-03
GO:0043209	myelin sheath	47	4	1.70E-03
GO:0042270	protection from natural killer cell mediated cytotoxicity	6	2	1.72E-03
GO:0071896	protein localization to adherens junction	6	2	1.72E-03
GO:0086073	bundle of His cell-Purkinje myocyte adhesion involved ir	6	2	1.72E-03
GO:0098704	carbohydrate import across plasma membrane	6	2	1.72E-03
GO:0140271	hexose import across plasma membrane	6	2	1.72E-03
GO:1905684	regulation of plasma membrane repair	6	2	1.72E-03
GO:0004457	lactate dehydrogenase activity	6	2	1.72E-03
GO:0050543	icosatetraenoic acid binding	6	2	1.72E-03
GO:0050544	arachidonic acid binding	6	2	1.72E-03
GO:0055131	C3HC4-type RING finger domain binding	6	2	1.72E-03
GO:1990254	keratin filament binding	6	2	1.72E-03
GO:0086001	cardiac muscle cell action potential	79	5	1.73E-03
GO:0034765	regulation of ion transmembrane transport	520	14	1.78E-03
GO:0021782	glial cell development	117	6	1.80E-03
GO:0031401	positive regulation of protein modification process	1019	22	1.81E-03

GO:0098862	cluster of actin-based cell projections	159	7	1.84E-03
GO:0044232	organelle membrane contact site	48	4	1.84E-03
GO:0098657	import into cell	252	9	1.85E-03
GO:0071346	cellular response to interferon-gamma	118	6	1.88E-03
GO:0005874	microtubule	466	13	1.90E-03
GO:0044827	modulation by host of viral genome replication	23	3	1.93E-03
GO:0048025	negative regulation of mRNA splicing, via spliceosome	23	3	1.93E-03
GO:0051131	chaperone-mediated protein complex assembly	23	3	1.93E-03
GO:0071359	cellular response to dsRNA	23	3	1.93E-03
GO:1900120	regulation of receptor binding	23	3	1.93E-03
GO:0034612	response to tumor necrosis factor	254	9	1.95E-03
GO:0009925	basal plasma membrane	254	9	1.95E-03
GO:0002708	positive regulation of lymphocyte mediated immunity	119	6	1.96E-03
GO:0046651	lymphocyte proliferation	305	10	1.98E-03
GO:0019884	antigen processing and presentation of exogenous antig	49	4	1.99E-03
GO:0005504	fatty acid binding	49	4	1.99E-03
GO:1902115	regulation of organelle assembly	207	8	2.02E-03
GO:0010770	positive regulation of cell morphogenesis involved in dif	82	5	2.04E-03
GO:1902369	negative regulation of RNA catabolic process	82	5	2.04E-03
GO:0002821	positive regulation of adaptive immune response	120	6	2.05E-03
GO:0034645	cellular macromolecule biosynthetic process	1231	25	2.06E-03
GO:0090257	regulation of muscle system process	256	9	2.06E-03
GO:0045446	endothelial cell differentiation	121	6	2.13E-03
GO:0097225	sperm midpiece	50	4	2.14E-03
GO:0055074	calcium ion homeostasis	309	10	2.17E-03
GO:0005543	phospholipid binding	474	13	2.20E-03
GO:0031396	regulation of protein ubiquitination	210	8	2.20E-03
GO:0043170	macromolecule metabolic process	10395	135	2.27E-03
GO:0030136	clathrin-coated vesicle	211	8	2.27E-03
GO:0072655	establishment of protein localization to mitochondrion	123	6	2.32E-03
GO:0032943	mononuclear cell proliferation	312	10	2.33E-03
GO:0042098	T cell proliferation	212	8	2.34E-03
GO:0006807	nitrogen compound metabolic process	10310	134	2.35E-03
GO:0030001	metal ion transport	909	20	2.35E-03
GO:0010631	epithelial cell migration	366	11	2.36E-03
GO:0035425	autocrine signaling	7	2	2.40E-03
GO:0038027	apolipoprotein A-I-mediated signaling pathway	7	2	2.40E-03
GO:0044806	G-quadruplex DNA unwinding	7	2	2.40E-03
GO:0086042	cardiac muscle cell-cardiac muscle cell adhesion	7	2	2.40E-03
GO:1903071	positive regulation of ER-associated ubiquitin-dependen	7	2	2.40E-03
GO:0030235	nitric-oxide synthase regulator activity	7	2	2.40E-03
GO:0050542	icosanoid binding	7	2	2.40E-03
GO:0001558	regulation of cell growth	422	12	2.40E-03
GO:0002064	epithelial cell development	213	8	2.41E-03
GO:0017157	regulation of exocytosis	213	8	2.41E-03
GO:0051051	negative regulation of transport	479	13	2.41E-03
GO:0009117	nucleotide metabolic process	597	15	2.41E-03

GO:0051209	release of sequestered calcium ion into cytosol	124	6	2.41E-03
GO:0003697	single-stranded DNA binding	124	6	2.41E-03
GO:0006007	glucose catabolic process	25	3	2.46E-03
GO:0031579	membrane raft organization	25	3	2.46E-03
GO:0050686	negative regulation of mRNA processing	25	3	2.46E-03
GO:0098581	detection of external biotic stimulus	25	3	2.46E-03
GO:0042611	MHC protein complex	25	3	2.46E-03
GO:0008266	poly(U) RNA binding	25	3	2.46E-03
GO:1903522	regulation of blood circulation	263	9	2.47E-03
GO:0006968	cellular defense response	52	4	2.47E-03
GO:0008016	regulation of heart contraction	214	8	2.48E-03
GO:0071495	cellular response to endogenous stimulus	1387	27	2.49E-03
GO:0032092	positive regulation of protein binding	86	5	2.51E-03
GO:0051283	negative regulation of sequestering of calcium ion	125	6	2.51E-03
GO:0090132	epithelium migration	369	11	2.52E-03
GO:0002262	myeloid cell homeostasis	169	7	2.59E-03
GO:0050660	flavin adenine dinucleotide binding	87	5	2.64E-03
GO:0071622	regulation of granulocyte chemotaxis	53	4	2.65E-03
GO:0051098	regulation of binding	372	11	2.68E-03
GO:0052548	regulation of endopeptidase activity	428	12	2.69E-03
GO:0051282	regulation of sequestering of calcium ion	127	6	2.72E-03
GO:0006753	nucleoside phosphate metabolic process	605	15	2.73E-03
GO:0060326	cell chemotaxis	319	10	2.73E-03
GO:0001503	ossification	429	12	2.75E-03
GO:0010954	positive regulation of protein processing	26	3	2.76E-03
GO:0019883	antigen processing and presentation of endogenous anti	26	3	2.76E-03
GO:0051238	sequestering of metal ion	26	3	2.76E-03
GO:0016460	myosin II complex	26	3	2.76E-03
GO:0048027	mRNA 5'-UTR binding	26	3	2.76E-03
GO:0055072	iron ion homeostasis	88	5	2.77E-03
GO:0090130	tissue migration	374	11	2.79E-03
GO:0051924	regulation of calcium ion transport	268	9	2.80E-03
GO:0005635	nuclear envelope	488	13	2.83E-03
GO:0051225	spindle assembly	128	6	2.83E-03
GO:0070585	protein localization to mitochondrion	128	6	2.83E-03
GO:0045744	negative regulation of G protein-coupled receptor signal	54	4	2.84E-03
GO:1902743	regulation of lamellipodium organization	54	4	2.84E-03
GO:0032154	cleavage furrow	54	4	2.84E-03
GO:0005544	calcium-dependent phospholipid binding	54	4	2.84E-03
GO:0042391	regulation of membrane potential	431	12	2.85E-03
GO:0051015	actin filament binding	219	8	2.85E-03
GO:0003824	catalytic activity	5488	79	2.89E-03
GO:0002694	regulation of leukocyte activation	670	16	2.89E-03
GO:0033554	cellular response to stress	1979	35	2.89E-03
GO:0009725	response to hormone	861	19	2.91E-03
GO:0016528	sarcoplasm	89	5	2.91E-03
GO:0003018	vascular process in circulatory system	270	9	2.94E-03

GO:1903037	regulation of leukocyte cell-cell adhesion	377	11	2.97E-03
GO:0045859	regulation of protein kinase activity	672	16	2.97E-03
GO:0055086	nucleobase-containing small molecule metabolic proces	673	16	3.02E-03
GO:0051262	protein tetramerization	90	5	3.06E-03
GO:2000177	regulation of neural precursor cell proliferation	90	5	3.06E-03
GO:0009894	regulation of catabolic process	997	21	3.07E-03
GO:0002418	immune response to tumor cell	27	3	3.08E-03
GO:0045178	basal part of cell	272	9	3.09E-03
GO:0055082	cellular chemical homeostasis	675	16	3.11E-03
GO:0051208	sequestering of calcium ion	131	6	3.17E-03
GO:0000056	ribosomal small subunit export from nucleus	8	2	3.17E-03
GO:0002036	regulation of L-glutamate import across plasma membra	8	2	3.17E-03
GO:0002486	antigen processing and presentation of endogenous pep	8	2	3.17E-03
GO:0002536	respiratory burst involved in inflammatory response	8	2	3.17E-03
GO:0010756	positive regulation of plasminogen activation	8	2	3.17E-03
GO:0010911	regulation of isomerase activity	8	2	3.17E-03
GO:0010912	positive regulation of isomerase activity	8	2	3.17E-03
GO:0032119	sequestering of zinc ion	8	2	3.17E-03
GO:0035696	monocyte extravasation	8	2	3.17E-03
GO:0045955	negative regulation of calcium ion-dependent exocytosis	8	2	3.17E-03
GO:0051683	establishment of Golgi localization	8	2	3.17E-03
GO:0070837	dehydroascorbic acid transport	8	2	3.17E-03
GO:0098885	modification of postsynaptic actin cytoskeleton	8	2	3.17E-03
GO:0150094	amyloid-beta clearance by cellular catabolic process	8	2	3.17E-03
GO:1900122	positive regulation of receptor binding	8	2	3.17E-03
GO:1902255	positive regulation of intrinsic apoptotic signaling pathw	8	2	3.17E-03
GO:0019773	proteasome core complex, alpha-subunit complex	8	2	3.17E-03
GO:0042824	MHC class I peptide loading complex	8	2	3.17E-03
GO:1990712	HFE-transferrin receptor complex	8	2	3.17E-03
GO:0033300	dehydroascorbic acid transmembrane transporter activi	8	2	3.17E-03
GO:0034046	poly(G) binding	8	2	3.17E-03
GO:0046977	TAP binding	8	2	3.17E-03
GO:1990948	ubiquitin ligase inhibitor activity	8	2	3.17E-03
GO:0001954	positive regulation of cell-matrix adhesion	56	4	3.24E-03
GO:1903170	negative regulation of calcium ion transmembrane trans	56	4	3.24E-03
GO:0016459	myosin complex	56	4	3.24E-03
GO:0032635	interleukin-6 production	176	7	3.25E-03
GO:0032675	regulation of interleukin-6 production	176	7	3.25E-03
GO:0007166	cell surface receptor signaling pathway	2907	47	3.27E-03
GO:0009132	nucleoside diphosphate metabolic process	132	6	3.29E-03
GO:1903039	positive regulation of leukocyte cell-cell adhesion	275	9	3.32E-03
GO:0051099	positive regulation of binding	177	7	3.35E-03
GO:0051348	negative regulation of transferase activity	276	9	3.40E-03
GO:0006913	nucleocytoplasmic transport	329	10	3.41E-03
GO:0051169	nuclear transport	329	10	3.41E-03
GO:0002227	innate immune response in mucosa	28	3	3.43E-03
GO:0030194	positive regulation of blood coagulation	28	3	3.43E-03

GO:0031664	regulation of lipopolysaccharide-mediated signaling path	28	3	3.43E-03
GO:0033119	negative regulation of RNA splicing	28	3	3.43E-03
GO:0034063	stress granule assembly	28	3	3.43E-03
GO:1900048	positive regulation of hemostasis	28	3	3.43E-03
GO:1903319	positive regulation of protein maturation	28	3	3.43E-03
GO:0043236	laminin binding	28	3	3.43E-03
GO:0045185	maintenance of protein location	93	5	3.52E-03
GO:0005902	microvillus	93	5	3.52E-03
GO:0010035	response to inorganic substance	561	14	3.54E-03
GO:0001909	leukocyte mediated cytotoxicity	134	6	3.55E-03
GO:0030048	actin filament-based movement	134	6	3.55E-03
GO:0009628	response to abiotic stimulus	1148	23	3.66E-03
GO:0006575	cellular modified amino acid metabolic process	180	7	3.67E-03
GO:0045309	protein phosphorylated amino acid binding	58	4	3.68E-03
GO:0051492	regulation of stress fiber assembly	94	5	3.69E-03
GO:0006941	striated muscle contraction	181	7	3.79E-03
GO:0006508	proteolysis	1791	32	3.79E-03
GO:1902175	regulation of oxidative stress-induced intrinsic apoptotic	29	3	3.79E-03
GO:0051787	misfolded protein binding	29	3	3.79E-03
GO:0099024	plasma membrane invagination	136	6	3.81E-03
GO:0042383	sarcolemma	136	6	3.81E-03
GO:0009150	purine ribonucleotide metabolic process	447	12	3.82E-03
GO:0051258	protein polymerization	281	9	3.83E-03
GO:0014909	smooth muscle cell migration	95	5	3.86E-03
GO:1900407	regulation of cellular response to oxidative stress	95	5	3.86E-03
GO:0046903	secretion	951	20	3.91E-03
GO:0001912	positive regulation of leukocyte mediated cytotoxicity	59	4	3.92E-03
GO:0042306	regulation of protein import into nucleus	59	4	3.92E-03
GO:0034101	erythrocyte homeostasis	137	6	3.95E-03
GO:0002683	negative regulation of immune system process	449	12	3.96E-03
GO:0002696	positive regulation of leukocyte activation	450	12	4.03E-03
GO:0001738	morphogenesis of a polarized epithelium	96	5	4.03E-03
GO:0002484	antigen processing and presentation of endogenous pep	9	2	4.05E-03
GO:0019852	L-ascorbic acid metabolic process	9	2	4.05E-03
GO:0034139	regulation of toll-like receptor 3 signaling pathway	9	2	4.05E-03
GO:0042590	antigen processing and presentation of exogenous pepti	9	2	4.05E-03
GO:1901334	lactone metabolic process	9	2	4.05E-03
GO:1903069	regulation of ER-associated ubiquitin-dependent protein	9	2	4.05E-03
GO:0014731	spectrin-associated cytoskeleton	9	2	4.05E-03
GO:0042584	chromaffin granule membrane	9	2	4.05E-03
GO:0047499	calcium-independent phospholipase A2 activity	9	2	4.05E-03
GO:0055105	ubiquitin-protein transferase inhibitor activity	9	2	4.05E-03
GO:0002011	morphogenesis of an epithelial sheet	60	4	4.16E-03
GO:0002711	positive regulation of T cell mediated immunity	60	4	4.16E-03
GO:0019886	antigen processing and presentation of exogenous pepti	30	3	4.18E-03
GO:0043032	positive regulation of macrophage activation	30	3	4.18E-03
GO:0050820	positive regulation of coagulation	30	3	4.18E-03

GO:0090330	regulation of platelet aggregation	30	3	4.18E-03
GO:1901798	positive regulation of signal transduction by p53 class m	30	3	4.18E-03
GO:0070717	poly-purine tract binding	30	3	4.18E-03
GO:0071813	lipoprotein particle binding	30	3	4.18E-03
GO:0071814	protein-lipid complex binding	30	3	4.18E-03
GO:0034249	negative regulation of cellular amide metabolic process	285	9	4.19E-03
GO:0002690	positive regulation of leukocyte chemotaxis	97	5	4.22E-03
GO:0006942	regulation of striated muscle contraction	97	5	4.22E-03
GO:0055038	recycling endosome membrane	97	5	4.22E-03
GO:0140535	intracellular protein-containing complex	892	19	4.26E-03
GO:0022618	ribonucleoprotein complex assembly	234	8	4.26E-03
GO:0071356	cellular response to tumor necrosis factor	234	8	4.26E-03
GO:0046982	protein heterodimerization activity	341	10	4.38E-03
GO:0002705	positive regulation of leukocyte mediated immunity	140	6	4.39E-03
GO:0007266	Rho protein signal transduction	140	6	4.39E-03
GO:0034341	response to interferon-gamma	140	6	4.39E-03
GO:0060048	cardiac muscle contraction	140	6	4.39E-03
GO:0030865	cortical cytoskeleton organization	61	4	4.42E-03
GO:0043005	neuron projection	1378	26	4.51E-03
GO:0005768	endosome	1031	21	4.51E-03
GO:0000910	cytokinesis	187	7	4.52E-03
GO:0045766	positive regulation of angiogenesis	187	7	4.52E-03
GO:1904018	positive regulation of vasculature development	187	7	4.52E-03
GO:0006986	response to unfolded protein	141	6	4.55E-03
GO:0051241	negative regulation of multicellular organismal process	1169	23	4.56E-03
GO:0001664	G protein-coupled receptor binding	289	9	4.59E-03
GO:0001916	positive regulation of T cell mediated cytotoxicity	31	3	4.59E-03
GO:0010039	response to iron ion	31	3	4.59E-03
GO:0033688	regulation of osteoblast proliferation	31	3	4.59E-03
GO:0098901	regulation of cardiac muscle cell action potential	31	3	4.59E-03
GO:0032839	dendrite cytoplasm	31	3	4.59E-03
GO:0001968	fibronectin binding	31	3	4.59E-03
GO:2001237	negative regulation of extrinsic apoptotic signaling pathw	99	5	4.60E-03
GO:0031234	extrinsic component of cytoplasmic side of plasma mem	99	5	4.60E-03
GO:0022617	extracellular matrix disassembly	62	4	4.68E-03
GO:0090303	positive regulation of wound healing	62	4	4.68E-03
GO:1903409	reactive oxygen species biosynthetic process	62	4	4.68E-03
GO:0120035	regulation of plasma membrane bounded cell projection	642	15	4.73E-03
GO:0030424	axon	642	15	4.73E-03
GO:0019080	viral gene expression	100	5	4.80E-03
GO:0009057	macromolecule catabolic process	1385	26	4.81E-03
GO:0010324	membrane invagination	143	6	4.87E-03
GO:0031674	I band	143	6	4.87E-03
GO:0019731	antibacterial humoral response	63	4	4.96E-03
GO:0012507	ER to Golgi transport vesicle membrane	63	4	4.96E-03
GO:0051085	chaperone cofactor-dependent protein refolding	32	3	5.02E-03
GO:0001765	membrane raft assembly	10	2	5.03E-03

GO:0002934	desmosome organization	10	2	5.03E-03
GO:0070391	response to lipoteichoic acid	10	2	5.03E-03
GO:0071223	cellular response to lipoteichoic acid	10	2	5.03E-03
GO:1990034	calcium ion export across plasma membrane	10	2	5.03E-03
GO:0097197	tetraspanin-enriched microdomain	10	2	5.03E-03
GO:0030957	Tat protein binding	10	2	5.03E-03
GO:0032050	clathrin heavy chain binding	10	2	5.03E-03
GO:0045294	alpha-catenin binding	10	2	5.03E-03
GO:0050786	RAGE receptor binding	10	2	5.03E-03
GO:1990247	N6-methyladenosine-containing RNA binding	10	2	5.03E-03
GO:0071826	ribonucleoprotein complex subunit organization	242	8	5.20E-03
GO:0031341	regulation of cell killing	102	5	5.22E-03
GO:0002218	activation of innate immune response	64	4	5.24E-03
GO:0035306	positive regulation of dephosphorylation	64	4	5.24E-03
GO:0048524	positive regulation of viral process	64	4	5.24E-03
GO:0050891	multicellular organismal water homeostasis	64	4	5.24E-03
GO:0009259	ribonucleotide metabolic process	466	12	5.29E-03
GO:0048144	fibroblast proliferation	103	5	5.43E-03
GO:0061157	mRNA destabilization	103	5	5.43E-03
GO:0038094	Fc-gamma receptor signaling pathway	33	3	5.48E-03
GO:0005524	ATP binding	1472	27	5.54E-03
GO:0007265	Ras protein signal transduction	353	10	5.56E-03
GO:0006626	protein targeting to mitochondrion	104	5	5.66E-03
GO:0070252	actin-mediated cell contraction	104	5	5.66E-03
GO:0110020	regulation of actomyosin structure organization	104	5	5.66E-03
GO:1902882	regulation of response to oxidative stress	104	5	5.66E-03
GO:0007051	spindle organization	195	7	5.67E-03
GO:0006401	RNA catabolic process	299	9	5.71E-03
GO:0030554	adenyl nucleotide binding	1548	28	5.72E-03
GO:0019207	kinase regulator activity	246	8	5.73E-03
GO:1901342	regulation of vasculature development	355	10	5.78E-03
GO:0010467	gene expression	7096	96	5.83E-03
GO:0031343	positive regulation of cell killing	66	4	5.85E-03
GO:0034605	cellular response to heat	66	4	5.85E-03
GO:0048488	synaptic vesicle endocytosis	66	4	5.85E-03
GO:0140238	presynaptic endocytosis	66	4	5.85E-03
GO:0031330	negative regulation of cellular catabolic process	247	8	5.87E-03
GO:0051588	regulation of neurotransmitter transport	105	5	5.89E-03
GO:0051087	chaperone binding	105	5	5.89E-03
GO:0061351	neural precursor cell proliferation	149	6	5.93E-03
GO:1903131	mononuclear cell differentiation	473	12	5.94E-03
GO:0002495	antigen processing and presentation of peptide antigen	34	3	5.97E-03
GO:0035308	negative regulation of protein dephosphorylation	34	3	5.97E-03
GO:0086004	regulation of cardiac muscle cell contraction	34	3	5.97E-03
GO:0090162	establishment of epithelial cell polarity	34	3	5.97E-03
GO:1902624	positive regulation of neutrophil migration	34	3	5.97E-03
GO:0005834	heterotrimeric G-protein complex	34	3	5.97E-03

GO:0031344	regulation of cell projection organization	659	15	5.98E-03
GO:0003729	mRNA binding	596	14	5.98E-03
GO:0016311	dephosphorylation	357	10	6.00E-03
GO:0033625	positive regulation of integrin activation	11	2	6.10E-03
GO:0033632	regulation of cell-cell adhesion mediated by integrin	11	2	6.10E-03
GO:0051987	positive regulation of attachment of spindle microtubule	11	2	6.10E-03
GO:0060315	negative regulation of ryanodine-sensitive calcium-relea	11	2	6.10E-03
GO:0075522	IRES-dependent viral translational initiation	11	2	6.10E-03
GO:1901731	positive regulation of platelet aggregation	11	2	6.10E-03
GO:1901844	regulation of cell communication by electrical coupling i	11	2	6.10E-03
GO:0005536	glucose binding	11	2	6.10E-03
GO:0016670	oxidoreductase activity, acting on a sulfur group of dono	11	2	6.10E-03
GO:0047134	protein-disulfide reductase (NAD(P)) activity	11	2	6.10E-03
GO:0055037	recycling endosome	198	7	6.15E-03
GO:0015909	long-chain fatty acid transport	67	4	6.17E-03
GO:0031638	zymogen activation	67	4	6.17E-03
GO:0070613	regulation of protein processing	67	4	6.17E-03
GO:0019843	rRNA binding	67	4	6.17E-03
GO:0006163	purine nucleotide metabolic process	476	12	6.23E-03
GO:0016485	protein processing	250	8	6.29E-03
GO:0002027	regulation of heart rate	107	5	6.37E-03
GO:0050779	RNA destabilization	107	5	6.37E-03
GO:0061014	positive regulation of mRNA catabolic process	107	5	6.37E-03
GO:0050870	positive regulation of T cell activation	251	8	6.44E-03
GO:0042176	regulation of protein catabolic process	361	10	6.47E-03
GO:0070528	protein kinase C signaling	35	3	6.47E-03
GO:0097718	disordered domain specific binding	35	3	6.47E-03
GO:0008154	actin polymerization or depolymerization	200	7	6.48E-03
GO:1903320	regulation of protein modification by small protein conju	252	8	6.59E-03
GO:0014812	muscle cell migration	108	5	6.63E-03
GO:0030175	filopodium	108	5	6.63E-03
GO:0045595	regulation of cell differentiation	1639	29	6.66E-03
GO:0097305	response to alcohol	253	8	6.75E-03
GO:0030104	water homeostasis	69	4	6.84E-03
GO:0051093	negative regulation of developmental process	1002	20	6.87E-03
GO:0007162	negative regulation of cell adhesion	308	9	6.88E-03
GO:0030038	contractile actin filament bundle assembly	109	5	6.88E-03
GO:0043149	stress fiber assembly	109	5	6.88E-03
GO:0034763	negative regulation of transmembrane transport	154	6	6.94E-03
GO:0033687	osteoblast proliferation	36	3	7.01E-03
GO:0016235	aggresome	36	3	7.01E-03
GO:0051233	spindle midzone	36	3	7.01E-03
GO:0044260	cellular macromolecule metabolic process	3266	50	7.04E-03
GO:0034641	cellular nitrogen compound metabolic process	7053	95	7.09E-03
GO:0000045	autophagosome assembly	110	5	7.15E-03
GO:0042063	gliogenesis	310	9	7.17E-03
GO:0031032	actomyosin structure organization	204	7	7.20E-03

GO:0002449	lymphocyte mediated immunity	367	10	7.23E-03
GO:0032535	regulation of cellular component size	367	10	7.23E-03
GO:0001867	complement activation, lectin pathway	12	2	7.27E-03
GO:0002424	T cell mediated immune response to tumor cell	12	2	7.27E-03
GO:0034145	positive regulation of toll-like receptor 4 signaling pathw	12	2	7.27E-03
GO:0070493	thrombin-activated receptor signaling pathway	12	2	7.27E-03
GO:0098911	regulation of ventricular cardiac muscle cell action poter	12	2	7.27E-03
GO:0099010	modification of postsynaptic structure	12	2	7.27E-03
GO:1904667	negative regulation of ubiquitin protein ligase activity	12	2	7.27E-03
GO:0008035	high-density lipoprotein particle binding	12	2	7.27E-03
GO:0046790	virion binding	12	2	7.27E-03
GO:0055056	D-glucose transmembrane transporter activity	12	2	7.27E-03
GO:0140776	protein-containing complex destabilizing activity	12	2	7.27E-03
GO:0005819	spindle	426	11	7.31E-03
GO:1901654	response to ketone	205	7	7.38E-03
GO:0120034	positive regulation of plasma membrane bounded cell p	111	5	7.42E-03
GO:0005905	clathrin-coated pit	71	4	7.56E-03
GO:0006734	NADH metabolic process	37	3	7.56E-03
GO:0042162	telomeric DNA binding	37	3	7.56E-03
GO:0035295	tube development	1154	22	7.85E-03
GO:0032787	monocarboxylic acid metabolic process	616	14	7.90E-03
GO:1903317	regulation of protein maturation	72	4	7.94E-03
GO:0032153	cell division site	72	4	7.94E-03
GO:0030705	cytoskeleton-dependent intracellular transport	208	7	7.97E-03
GO:0044853	plasma membrane raft	113	5	7.99E-03
GO:0006914	autophagy	554	13	8.02E-03
GO:0061919	process utilizing autophagic mechanism	554	13	8.02E-03
GO:0008064	regulation of actin polymerization or depolymerization	159	6	8.06E-03
GO:0043021	ribonucleoprotein complex binding	159	6	8.06E-03
GO:0045920	negative regulation of exocytosis	38	3	8.15E-03
GO:0098794	postsynapse	619	14	8.22E-03
GO:0009408	response to heat	114	5	8.28E-03
GO:0043462	regulation of ATP-dependent activity	73	4	8.33E-03
GO:0019003	GDP binding	73	4	8.33E-03
GO:0032940	secretion by cell	817	17	8.40E-03
GO:1901566	organonitrogen compound biosynthetic process	1817	31	8.41E-03
GO:0006935	chemotaxis	621	14	8.45E-03
GO:0022008	neurogenesis	1669	29	8.48E-03
GO:0043410	positive regulation of MAPK cascade	496	12	8.51E-03
GO:0010642	negative regulation of platelet-derived growth factor rec	13	2	8.53E-03
GO:0033690	positive regulation of osteoblast proliferation	13	2	8.53E-03
GO:0034333	adherens junction assembly	13	2	8.53E-03
GO:0051280	negative regulation of release of sequestered calcium io	13	2	8.53E-03
GO:0072683	T cell extravasation	13	2	8.53E-03
GO:1900121	negative regulation of receptor binding	13	2	8.53E-03
GO:0042583	chromaffin granule	13	2	8.53E-03
GO:0004859	phospholipase inhibitor activity	13	2	8.53E-03

GO:0016668	oxidoreductase activity, acting on a sulfur group of donc	13	2	8.53E-03
GO:0019215	intermediate filament binding	13	2	8.53E-03
GO:0031994	insulin-like growth factor I binding	13	2	8.53E-03
GO:0045295	gamma-catenin binding	13	2	8.53E-03
GO:0050998	nitric-oxide synthase binding	13	2	8.53E-03
GO:0098802	plasma membrane signaling receptor complex	319	9	8.56E-03
GO:0034766	negative regulation of ion transmembrane transport	115	5	8.58E-03
GO:0009612	response to mechanical stimulus	211	7	8.59E-03
GO:0042330	taxis	623	14	8.67E-03
GO:0005813	centrosome	623	14	8.67E-03
GO:0051926	negative regulation of calcium ion transport	74	4	8.73E-03
GO:0090307	mitotic spindle assembly	74	4	8.73E-03
GO:0017022	myosin binding	74	4	8.73E-03
GO:0002385	mucosal immune response	39	3	8.76E-03
GO:0032728	positive regulation of interferon-beta production	39	3	8.76E-03
GO:0015035	protein-disulfide reductase activity	39	3	8.76E-03
GO:0071949	FAD binding	39	3	8.76E-03
GO:0040008	regulation of growth	624	14	8.79E-03
GO:0030832	regulation of actin filament length	162	6	8.80E-03
GO:0050792	regulation of viral process	162	6	8.80E-03
GO:0002832	negative regulation of response to biotic stimulus	116	5	8.89E-03
GO:0046916	cellular transition metal ion homeostasis	116	5	8.89E-03
GO:0045229	external encapsulating structure organization	321	9	8.90E-03
GO:0140352	export from cell	890	18	8.93E-03
GO:0035966	response to topologically incorrect protein	163	6	9.05E-03
GO:0021987	cerebral cortex development	117	5	9.21E-03
GO:1905037	autophagosome organization	117	5	9.21E-03
GO:0042326	negative regulation of phosphorylation	381	10	9.27E-03
GO:0030176	integral component of endoplasmic reticulum membran	164	6	9.32E-03
GO:0043408	regulation of MAPK cascade	694	15	9.39E-03
GO:0032506	cytokinetic process	40	3	9.39E-03
GO:0042307	positive regulation of protein import into nucleus	40	3	9.39E-03
GO:1903115	regulation of actin filament-based movement	40	3	9.39E-03
GO:1905360	GTPase complex	40	3	9.39E-03
GO:0032559	adenyl ribonucleotide binding	1536	27	9.53E-03
GO:0019637	organophosphate metabolic process	1034	20	9.54E-03
GO:0006809	nitric oxide biosynthetic process	76	4	9.57E-03
GO:0086003	cardiac muscle cell contraction	76	4	9.57E-03
GO:0031960	response to corticosteroid	165	6	9.58E-03
GO:0051168	nuclear export	165	6	9.58E-03
GO:0036211	protein modification process	3645	54	9.63E-03
GO:0009968	negative regulation of signal transduction	1319	24	9.65E-03
GO:0031665	negative regulation of lipopolysaccharide-mediated sign	14	2	9.88E-03
GO:0051645	Golgi localization	14	2	9.88E-03
GO:0072672	neutrophil extravasation	14	2	9.88E-03
GO:0098760	response to interleukin-7	14	2	9.88E-03
GO:0098761	cellular response to interleukin-7	14	2	9.88E-03

GO:1901841	regulation of high voltage-gated calcium channel activity	14	2	9.88E-03
GO:1904294	positive regulation of ERAD pathway	14	2	9.88E-03
GO:1990023	mitotic spindle midzone	14	2	9.88E-03
GO:0005161	platelet-derived growth factor receptor binding	14	2	9.88E-03
GO:0044548	S100 protein binding	14	2	9.88E-03
GO:0098847	sequence-specific single stranded DNA binding	14	2	9.88E-03
GO:0072521	purine-containing compound metabolic process	507	12	1.00E-02
GO:0006919	activation of cysteine-type endopeptidase activity involv	77	4	1.00E-02
GO:0008088	axo-dendritic transport	77	4	1.00E-02
GO:0043297	apical junction assembly	77	4	1.00E-02
GO:0001914	regulation of T cell mediated cytotoxicity	41	3	1.01E-02
GO:0002691	regulation of cellular extravasation	41	3	1.01E-02
GO:0042119	neutrophil activation	41	3	1.01E-02
GO:0045124	regulation of bone resorption	41	3	1.01E-02
GO:0033017	sarcoplasmic reticulum membrane	41	3	1.01E-02
GO:0030307	positive regulation of cell growth	167	6	1.01E-02
GO:0030426	growth cone	167	6	1.01E-02
GO:0043491	protein kinase B signaling	218	7	1.02E-02
GO:0019887	protein kinase regulator activity	218	7	1.02E-02
GO:0051897	positive regulation of protein kinase B signaling	120	5	1.02E-02
GO:0007420	brain development	768	16	1.03E-02
GO:0031967	organelle envelope	1255	23	1.04E-02
GO:0031975	envelope	1255	23	1.04E-02
GO:0051249	regulation of lymphocyte activation	573	13	1.04E-02
GO:0042274	ribosomal small subunit biogenesis	78	4	1.05E-02
GO:0042277	peptide binding	330	9	1.05E-02
GO:0051301	cell division	638	14	1.05E-02
GO:0086091	regulation of heart rate by cardiac conduction	42	3	1.07E-02
GO:0035254	glutamate receptor binding	42	3	1.07E-02
GO:0042287	MHC protein binding	42	3	1.07E-02
GO:0001897	cytolysis by symbiont of host cells	1	1	1.09E-02
GO:0001898	regulation of cytolysis by symbiont of host cells	1	1	1.09E-02
GO:0001899	negative regulation of cytolysis by symbiont of host cells	1	1	1.09E-02
GO:0001907	killing by symbiont of host cells	1	1	1.09E-02
GO:0001998	angiotensin-mediated vasoconstriction involved in regul	1	1	1.09E-02
GO:0006055	CMP-N-acetylneuraminate biosynthetic process	1	1	1.09E-02
GO:0006713	glucocorticoid catabolic process	1	1	1.09E-02
GO:0008039	synaptic target recognition	1	1	1.09E-02
GO:0010920	negative regulation of inositol phosphate biosynthetic p	1	1	1.09E-02
GO:0010924	regulation of inositol-polyphosphate 5-phosphatase acti	1	1	1.09E-02
GO:0010925	positive regulation of inositol-polyphosphate 5-phospha	1	1	1.09E-02
GO:0018160	peptidyl-pyrromethane cofactor linkage	1	1	1.09E-02
GO:0021556	central nervous system formation	1	1	1.09E-02
GO:0030845	phospholipase C-inhibiting G protein-coupled receptor s	1	1	1.09E-02
GO:0032980	keratinocyte activation	1	1	1.09E-02
GO:0033367	protein localization to mast cell secretory granule	1	1	1.09E-02
GO:0033368	protease localization to mast cell secretory granule	1	1	1.09E-02

GO:0033370	maintenance of protein location in mast cell secretory g	1	1	1.09E-02
GO:0033371	T cell secretory granule organization	1	1	1.09E-02
GO:0033373	maintenance of protease location in mast cell secretory	1	1	1.09E-02
GO:0033374	protein localization to T cell secretory granule	1	1	1.09E-02
GO:0033375	protease localization to T cell secretory granule	1	1	1.09E-02
GO:0033377	maintenance of protein location in T cell secretory granu	1	1	1.09E-02
GO:0033379	maintenance of protease location in T cell secretory gra	1	1	1.09E-02
GO:0033380	granzyme B localization to T cell secretory granule	1	1	1.09E-02
GO:0033382	maintenance of granzyme B location in T cell secretory g	1	1	1.09E-02
GO:0035732	nitric oxide storage	1	1	1.09E-02
GO:0039695	DNA-templated viral transcription	1	1	1.09E-02
GO:0042000	translocation of peptides or proteins into host	1	1	1.09E-02
GO:0044053	translocation of peptides or proteins into host cell cytop	1	1	1.09E-02
GO:0045107	intermediate filament polymerization	1	1	1.09E-02
GO:0045919	positive regulation of cytolysis	1	1	1.09E-02
GO:0048626	myoblast fate specification	1	1	1.09E-02
GO:0051086	chaperone mediated protein folding independent of cof	1	1	1.09E-02
GO:0051089	constitutive protein ectodomain proteolysis	1	1	1.09E-02
GO:0051623	positive regulation of norepinephrine uptake	1	1	1.09E-02
GO:0060305	regulation of cell diameter	1	1	1.09E-02
GO:0060735	regulation of eIF2 alpha phosphorylation by dsRNA	1	1	1.09E-02
GO:0071283	cellular response to iron(III) ion	1	1	1.09E-02
GO:0072356	chromosome passenger complex localization to kinetoch	1	1	1.09E-02
GO:0072749	cellular response to cytochalasin B	1	1	1.09E-02
GO:0150024	oxidised low-density lipoprotein particle clearance	1	1	1.09E-02
GO:0150103	reactive gliosis	1	1	1.09E-02
GO:0150107	positive regulation of protein localization to cell-cell jun	1	1	1.09E-02
GO:1900022	regulation of D-erythro-sphingosine kinase activity	1	1	1.09E-02
GO:1900729	regulation of adenylate cyclase-inhibiting opioid recepto	1	1	1.09E-02
GO:1900731	positive regulation of adenylate cyclase-inhibiting opioid	1	1	1.09E-02
GO:1901328	response to cytochalasin B	1	1	1.09E-02
GO:1901340	negative regulation of store-operated calcium channel a	1	1	1.09E-02
GO:1901585	regulation of acid-sensing ion channel activity	1	1	1.09E-02
GO:1902629	regulation of mRNA stability involved in cellular respons	1	1	1.09E-02
GO:1903434	negative regulation of constitutive secretory pathway	1	1	1.09E-02
GO:1903673	mitotic cleavage furrow formation	1	1	1.09E-02
GO:1903918	regulation of actin filament severing	1	1	1.09E-02
GO:1903919	negative regulation of actin filament severing	1	1	1.09E-02
GO:1904702	regulation of protein localization to adherens junction	1	1	1.09E-02
GO:1904764	chaperone-mediated autophagy translocation complex	1	1	1.09E-02
GO:1905578	regulation of ERBB3 signaling pathway	1	1	1.09E-02
GO:1905580	positive regulation of ERBB3 signaling pathway	1	1	1.09E-02
GO:1905597	positive regulation of low-density lipoprotein particle re	1	1	1.09E-02
GO:1905698	positive regulation of polysome binding	1	1	1.09E-02
GO:1990280	RNA localization to chromatin	1	1	1.09E-02
GO:1990832	slow axonal transport	1	1	1.09E-02
GO:2000308	negative regulation of tumor necrosis factor (ligand) sup	1	1	1.09E-02

GO:2000476	positive regulation of opioid receptor signaling pathway	1	1	1.09E-02
GO:0034677	integrin alpha7-beta1 complex	1	1	1.09E-02
GO:0071665	gamma-catenin-TCF7L2 complex	1	1	1.09E-02
GO:0098577	inactive sex chromosome	1	1	1.09E-02
GO:1990972	multimerin complex	1	1	1.09E-02
GO:0003937	IMP cyclohydrolase activity	1	1	1.09E-02
GO:0003974	UDP-N-acetylglucosamine 4-epimerase activity	1	1	1.09E-02
GO:0003978	UDP-glucose 4-epimerase activity	1	1	1.09E-02
GO:0004362	glutathione-disulfide reductase (NADPH) activity	1	1	1.09E-02
GO:0004418	hydroxymethylbilane synthase activity	1	1	1.09E-02
GO:0004643	phosphoribosylaminoimidazolecarboxamide formyltrans	1	1	1.09E-02
GO:0016964	alpha-2 macroglobulin receptor activity	1	1	1.09E-02
GO:0017130	poly(C) RNA binding	1	1	1.09E-02
GO:0018467	formaldehyde dehydrogenase activity	1	1	1.09E-02
GO:0019767	IgE receptor activity	1	1	1.09E-02
GO:0030729	acetoacetate-CoA ligase activity	1	1	1.09E-02
GO:0033679	3'-5' DNA/RNA helicase activity	1	1	1.09E-02
GO:0035605	peptidyl-cysteine S-nitrosylase activity	1	1	1.09E-02
GO:0035730	S-nitrosoglutathione binding	1	1	1.09E-02
GO:0035731	dinitrosyl-iron complex binding	1	1	1.09E-02
GO:0046817	chemokine receptor antagonist activity	1	1	1.09E-02
GO:0047444	N-acylneuraminate-9-phosphate synthase activity	1	1	1.09E-02
GO:0050462	N-acetylneuraminate synthase activity	1	1	1.09E-02
GO:0070892	lipoteichoic acid immune receptor activity	1	1	1.09E-02
GO:0102149	farnesylcysteine lyase activity	1	1	1.09E-02
GO:0106321	S-(hydroxymethyl)glutathione dehydrogenase NADP act	1	1	1.09E-02
GO:0106322	S-(hydroxymethyl)glutathione dehydrogenase NAD activ	1	1	1.09E-02
GO:0150025	oxidised low-density lipoprotein particle receptor activit	1	1	1.09E-02
GO:1990833	clathrin-uncoating ATPase activity	1	1	1.09E-02
GO:0009895	negative regulation of catabolic process	332	9	1.09E-02
GO:0002720	positive regulation of cytokine production involved in im	79	4	1.09E-02
GO:0036465	synaptic vesicle recycling	79	4	1.09E-02
GO:0000226	microtubule cytoskeleton organization	642	14	1.11E-02
GO:0030658	transport vesicle membrane	222	7	1.12E-02
GO:0000054	ribosomal subunit export from nucleus	15	2	1.13E-02
GO:0002679	respiratory burst involved in defense response	15	2	1.13E-02
GO:0002836	positive regulation of response to tumor cell	15	2	1.13E-02
GO:0002839	positive regulation of immune response to tumor cell	15	2	1.13E-02
GO:0010649	regulation of cell communication by electrical coupling	15	2	1.13E-02
GO:0033750	ribosome localization	15	2	1.13E-02
GO:0051284	positive regulation of sequestering of calcium ion	15	2	1.13E-02
GO:0051290	protein heterotetramerization	15	2	1.13E-02
GO:0071474	cellular hyperosmotic response	15	2	1.13E-02
GO:0086069	bundle of His cell to Purkinje myocyte communication	15	2	1.13E-02
GO:0005041	low-density lipoprotein particle receptor activity	15	2	1.13E-02
GO:0036041	long-chain fatty acid binding	15	2	1.13E-02
GO:0098858	actin-based cell projection	223	7	1.14E-02

GO:0005791	rough endoplasmic reticulum	80	4	1.14E-02
GO:0002251	organ or tissue specific immune response	43	3	1.15E-02
GO:0008608	attachment of spindle microtubules to kinetochore	43	3	1.15E-02
GO:0033574	response to testosterone	43	3	1.15E-02
GO:0150034	distal axon	278	8	1.15E-02
GO:1904062	regulation of cation transmembrane transport	394	10	1.15E-02
GO:1905475	regulation of protein localization to membrane	172	6	1.16E-02
GO:0031227	intrinsic component of endoplasmic reticulum membrar	172	6	1.16E-02
GO:0031072	heat shock protein binding	124	5	1.16E-02
GO:0032272	negative regulation of protein polymerization	81	4	1.19E-02
GO:0072332	intrinsic apoptotic signaling pathway by p53 class media	81	4	1.19E-02
GO:0033293	monocarboxylic acid binding	81	4	1.19E-02
GO:0072331	signal transduction by p53 class mediator	173	6	1.19E-02
GO:0030427	site of polarized growth	173	6	1.19E-02
GO:0000956	nuclear-transcribed mRNA catabolic process	125	5	1.20E-02
GO:1904375	regulation of protein localization to cell periphery	125	5	1.20E-02
GO:1990204	oxidoreductase complex	125	5	1.20E-02
GO:0009058	biosynthetic process	5962	81	1.22E-02
GO:0001933	negative regulation of protein phosphorylation	338	9	1.22E-02
GO:0001504	neurotransmitter uptake	44	3	1.22E-02
GO:1905521	regulation of macrophage migration	44	3	1.22E-02
GO:0040007	growth	921	18	1.24E-02
GO:0046209	nitric oxide metabolic process	82	4	1.24E-02
GO:0048708	astrocyte differentiation	82	4	1.24E-02
GO:1902117	positive regulation of organelle assembly	82	4	1.24E-02
GO:0005901	caveola	82	4	1.24E-02
GO:1905369	endopeptidase complex	82	4	1.24E-02
GO:0038024	cargo receptor activity	82	4	1.24E-02
GO:0010001	glial cell differentiation	227	7	1.25E-02
GO:0045596	negative regulation of cell differentiation	718	15	1.25E-02
GO:0048660	regulation of smooth muscle cell proliferation	175	6	1.25E-02
GO:0010648	negative regulation of cell communication	1424	25	1.26E-02
GO:0002688	regulation of leukocyte chemotaxis	127	5	1.28E-02
GO:0006911	phagocytosis, engulfment	127	5	1.28E-02
GO:0044271	cellular nitrogen compound biosynthetic process	4865	68	1.28E-02
GO:0010715	regulation of extracellular matrix disassembly	16	2	1.28E-02
GO:0010820	positive regulation of T cell chemotaxis	16	2	1.28E-02
GO:0051014	actin filament severing	16	2	1.28E-02
GO:1901741	positive regulation of myoblast fusion	16	2	1.28E-02
GO:1902931	negative regulation of alcohol biosynthetic process	16	2	1.28E-02
GO:0003688	DNA replication origin binding	16	2	1.28E-02
GO:0005159	insulin-like growth factor receptor binding	16	2	1.28E-02
GO:0031489	myosin V binding	16	2	1.28E-02
GO:1902905	positive regulation of supramolecular fiber organization	176	6	1.29E-02
GO:0007017	microtubule-based process	925	18	1.29E-02
GO:0050848	regulation of calcium-mediated signaling	83	4	1.29E-02
GO:2001057	reactive nitrogen species metabolic process	83	4	1.29E-02

GO:0055085	transmembrane transport	1575	27	1.29E-02
GO:0032781	positive regulation of ATP-dependent activity	45	3	1.30E-02
GO:0001772	immunological synapse	45	3	1.30E-02
GO:0009295	nucleoid	45	3	1.30E-02
GO:0042645	mitochondrial nucleoid	45	3	1.30E-02
GO:0019318	hexose metabolic process	229	7	1.31E-02
GO:0002443	leukocyte mediated immunity	463	11	1.31E-02
GO:0044249	cellular biosynthetic process	5807	79	1.31E-02
GO:0031328	positive regulation of cellular biosynthetic process	2032	33	1.32E-02
GO:0043534	blood vessel endothelial cell migration	177	6	1.32E-02
GO:0030218	erythrocyte differentiation	128	5	1.32E-02
GO:0045471	response to ethanol	128	5	1.32E-02
GO:1902494	catalytic complex	1653	28	1.33E-02
GO:0023057	negative regulation of signaling	1430	25	1.33E-02
GO:0031324	negative regulation of cellular metabolic process	2345	37	1.35E-02
GO:0031397	negative regulation of protein ubiquitination	84	4	1.35E-02
GO:0000165	MAPK cascade	792	16	1.35E-02
GO:0031503	protein-containing complex localization	178	6	1.36E-02
GO:0014902	myotube differentiation	129	5	1.36E-02
GO:0044057	regulation of system process	594	13	1.37E-02
GO:0002706	regulation of lymphocyte mediated immunity	179	6	1.39E-02
GO:0048659	smooth muscle cell proliferation	179	6	1.39E-02
GO:0005546	phosphatidylinositol-4,5-bisphosphate binding	85	4	1.40E-02
GO:0030018	Z disc	130	5	1.41E-02
GO:0006812	cation transport	1219	22	1.42E-02
GO:0008134	transcription factor binding	597	13	1.43E-02
GO:0031329	regulation of cellular catabolic process	798	16	1.44E-02
GO:0033218	amide binding	408	10	1.44E-02
GO:0006089	lactate metabolic process	17	2	1.45E-02
GO:0008228	opsonization	17	2	1.45E-02
GO:0010819	regulation of T cell chemotaxis	17	2	1.45E-02
GO:0016045	detection of bacterium	17	2	1.45E-02
GO:0033623	regulation of integrin activation	17	2	1.45E-02
GO:0051895	negative regulation of focal adhesion assembly	17	2	1.45E-02
GO:0098712	L-glutamate import across plasma membrane	17	2	1.45E-02
GO:0150118	negative regulation of cell-substrate junction organizatio	17	2	1.45E-02
GO:1901550	regulation of endothelial cell development	17	2	1.45E-02
GO:1903140	regulation of establishment of endothelial barrier	17	2	1.45E-02
GO:1903729	regulation of plasma membrane organization	17	2	1.45E-02
GO:0043020	NADPH oxidase complex	17	2	1.45E-02
GO:0005324	long-chain fatty acid transporter activity	17	2	1.45E-02
GO:0030169	low-density lipoprotein particle binding	17	2	1.45E-02
GO:0055102	lipase inhibitor activity	17	2	1.45E-02
GO:0140693	molecular condensate scaffold activity	17	2	1.45E-02
GO:0036230	granulocyte activation	47	3	1.46E-02
GO:0042269	regulation of natural killer cell mediated cytotoxicity	47	3	1.46E-02
GO:1902622	regulation of neutrophil migration	47	3	1.46E-02

GO:0005044	scavenger receptor activity	47	3	1.46E-02
GO:0045765	regulation of angiogenesis	349	9	1.47E-02
GO:0022898	regulation of transmembrane transporter activity	291	8	1.48E-02
GO:0030665	clathrin-coated vesicle membrane	132	5	1.49E-02
GO:0002822	regulation of adaptive immune response based on soma	182	6	1.50E-02
GO:0097553	calcium ion transmembrane import into cytosol	182	6	1.50E-02
GO:0000139	Golgi membrane	667	14	1.50E-02
GO:0001910	regulation of leukocyte mediated cytotoxicity	87	4	1.51E-02
GO:0014910	regulation of smooth muscle cell migration	87	4	1.51E-02
GO:0008361	regulation of cell size	183	6	1.53E-02
GO:0002702	positive regulation of production of molecular mediator	133	5	1.54E-02
GO:0035305	negative regulation of dephosphorylation	48	3	1.54E-02
GO:0046850	regulation of bone remodeling	48	3	1.54E-02
GO:1903706	regulation of hemopoiesis	413	10	1.56E-02
GO:0050727	regulation of inflammatory response	414	10	1.58E-02
GO:0005769	early endosome	414	10	1.58E-02
GO:0000278	mitotic cell cycle	946	18	1.59E-02
GO:0010008	endosome membrane	541	12	1.60E-02
GO:0002693	positive regulation of cellular extravasation	18	2	1.61E-02
GO:0010958	regulation of amino acid import across plasma membrar	18	2	1.61E-02
GO:0034138	toll-like receptor 3 signaling pathway	18	2	1.61E-02
GO:0036035	osteoclast development	18	2	1.61E-02
GO:0045780	positive regulation of bone resorption	18	2	1.61E-02
GO:0050665	hydrogen peroxide biosynthetic process	18	2	1.61E-02
GO:0060263	regulation of respiratory burst	18	2	1.61E-02
GO:0071360	cellular response to exogenous dsRNA	18	2	1.61E-02
GO:0086103	G protein-coupled receptor signaling pathway involved i	18	2	1.61E-02
GO:1903789	regulation of amino acid transmembrane transport	18	2	1.61E-02
GO:0003756	protein disulfide isomerase activity	18	2	1.61E-02
GO:0016864	intramolecular oxidoreductase activity, transposing S-S b	18	2	1.61E-02
GO:0030228	lipoprotein particle receptor activity	18	2	1.61E-02
GO:0098641	cadherin binding involved in cell-cell adhesion	18	2	1.61E-02
GO:0001666	response to hypoxia	296	8	1.63E-02
GO:0048872	homeostasis of number of cells	296	8	1.63E-02
GO:0033628	regulation of cell adhesion mediated by integrin	49	3	1.63E-02
GO:0035307	positive regulation of protein dephosphorylation	49	3	1.63E-02
GO:0045933	positive regulation of muscle contraction	49	3	1.63E-02
GO:0002709	regulation of T cell mediated immunity	89	4	1.63E-02
GO:0032413	negative regulation of ion transmembrane transporter a	89	4	1.63E-02
GO:0050829	defense response to Gram-negative bacterium	89	4	1.63E-02
GO:0033673	negative regulation of kinase activity	240	7	1.65E-02
GO:0009266	response to temperature stimulus	186	6	1.65E-02
GO:0043235	receptor complex	544	12	1.66E-02
GO:0002703	regulation of leukocyte mediated immunity	241	7	1.68E-02
GO:0034220	ion transmembrane transport	1167	21	1.69E-02
GO:0060191	regulation of lipase activity	90	4	1.70E-02
GO:0045334	clathrin-coated endocytic vesicle	90	4	1.70E-02

GO:0120111	neuron projection cytoplasm	90	4	1.70E-02
GO:0030098	lymphocyte differentiation	419	10	1.71E-02
GO:0045055	regulated exocytosis	242	7	1.72E-02
GO:0060322	head development	815	16	1.72E-02
GO:0006692	prostanoid metabolic process	50	3	1.72E-02
GO:0006693	prostaglandin metabolic process	50	3	1.72E-02
GO:0010557	positive regulation of macromolecule biosynthetic proce	1918	31	1.72E-02
GO:1903169	regulation of calcium ion transmembrane transport	188	6	1.73E-02
GO:0045860	positive regulation of protein kinase activity	420	10	1.73E-02
GO:0022411	cellular component disassembly	483	11	1.73E-02
GO:0009891	positive regulation of biosynthetic process	2074	33	1.74E-02
GO:1901576	organic substance biosynthetic process	5877	79	1.75E-02
GO:0048525	negative regulation of viral process	91	4	1.76E-02
GO:0010038	response to metal ion	360	9	1.77E-02
GO:0006839	mitochondrial transport	189	6	1.77E-02
GO:0043123	positive regulation of I-kappaB kinase/NF-kappaB signali	189	6	1.77E-02
GO:0006098	pentose-phosphate shunt	19	2	1.79E-02
GO:0007213	G protein-coupled acetylcholine receptor signaling pathv	19	2	1.79E-02
GO:0010759	positive regulation of macrophage chemotaxis	19	2	1.79E-02
GO:0043652	engulfment of apoptotic cell	19	2	1.79E-02
GO:0045953	negative regulation of natural killer cell mediated cytoto	19	2	1.79E-02
GO:0005520	insulin-like growth factor binding	19	2	1.79E-02
GO:0030133	transport vesicle	423	10	1.81E-02
GO:0001913	T cell mediated cytotoxicity	51	3	1.81E-02
GO:0002715	regulation of natural killer cell mediated immunity	51	3	1.81E-02
GO:0061980	regulatory RNA binding	51	3	1.81E-02
GO:0032273	positive regulation of protein polymerization	92	4	1.82E-02
GO:0051219	phosphoprotein binding	92	4	1.82E-02
GO:0008277	regulation of G protein-coupled receptor signaling pathv	139	5	1.83E-02
GO:0002020	protease binding	139	5	1.83E-02
GO:0051020	GTPase binding	303	8	1.84E-02
GO:0030833	regulation of actin filament polymerization	140	5	1.88E-02
GO:0051496	positive regulation of stress fiber assembly	52	3	1.91E-02
GO:0061462	protein localization to lysosome	52	3	1.91E-02
GO:0031175	neuron projection development	967	18	1.93E-02
GO:0034655	nucleobase-containing compound catabolic process	428	10	1.94E-02
GO:0051046	regulation of secretion	623	13	1.96E-02
GO:0002716	negative regulation of natural killer cell mediated immu	20	2	1.98E-02
GO:0002837	regulation of immune response to tumor cell	20	2	1.98E-02
GO:0010310	regulation of hydrogen peroxide metabolic process	20	2	1.98E-02
GO:0030011	maintenance of cell polarity	20	2	1.98E-02
GO:0032211	negative regulation of telomere maintenance via telome	20	2	1.98E-02
GO:0042953	lipoprotein transport	20	2	1.98E-02
GO:0051546	keratinocyte migration	20	2	1.98E-02
GO:0098543	detection of other organism	20	2	1.98E-02
GO:1904292	regulation of ERAD pathway	20	2	1.98E-02
GO:0005839	proteasome core complex	20	2	1.98E-02

GO:0031362	anchored component of external side of plasma membr	20	2	1.98E-02
GO:0010720	positive regulation of cell development	307	8	1.98E-02
GO:0010977	negative regulation of neuron projection development	142	5	1.98E-02
GO:0051384	response to glucocorticoid	142	5	1.98E-02
GO:0031345	negative regulation of cell projection organization	194	6	1.99E-02
GO:0051172	negative regulation of nitrogen compound metabolic pr	2490	38	2.00E-02
GO:0050661	NADP binding	53	3	2.01E-02
GO:0030134	COPII-coated ER to Golgi transport vesicle	95	4	2.03E-02
GO:0051495	positive regulation of cytoskeleton organization	195	6	2.03E-02
GO:0005815	microtubule organizing center	832	16	2.04E-02
GO:0036293	response to decreased oxygen levels	309	8	2.05E-02
GO:0007399	nervous system development	2495	38	2.06E-02
GO:0050808	synapse organization	432	10	2.06E-02
GO:0033674	positive regulation of kinase activity	496	11	2.06E-02
GO:0050921	positive regulation of chemotaxis	144	5	2.09E-02
GO:1903321	negative regulation of protein modification by small pro	96	4	2.10E-02
GO:0051293	establishment of spindle localization	54	3	2.11E-02
GO:0086002	cardiac muscle cell action potential involved in contracti	54	3	2.11E-02
GO:0048667	cell morphogenesis involved in neuron differentiation	564	12	2.14E-02
GO:0045177	apical part of cell	435	10	2.15E-02
GO:1901653	cellular response to peptide	373	9	2.16E-02
GO:0001971	negative regulation of activation of membrane attack co	2	1	2.17E-02
GO:0002037	negative regulation of L-glutamate import across plasma	2	1	2.17E-02
GO:0002481	antigen processing and presentation of exogenous prote	2	1	2.17E-02
GO:0002485	antigen processing and presentation of endogenous pep	2	1	2.17E-02
GO:0003064	regulation of heart rate by hormone	2	1	2.17E-02
GO:0006407	rRNA export from nucleus	2	1	2.17E-02
GO:0010731	protein glutathionylation	2	1	2.17E-02
GO:0010732	regulation of protein glutathionylation	2	1	2.17E-02
GO:0010734	negative regulation of protein glutathionylation	2	1	2.17E-02
GO:0015920	lipopolysaccharide transport	2	1	2.17E-02
GO:0019322	pentose biosynthetic process	2	1	2.17E-02
GO:0030328	prenylcysteine catabolic process	2	1	2.17E-02
GO:0030329	prenylcysteine metabolic process	2	1	2.17E-02
GO:0032796	uropod organization	2	1	2.17E-02
GO:0032804	negative regulation of low-density lipoprotein particle re	2	1	2.17E-02
GO:0033364	mast cell secretory granule organization	2	1	2.17E-02
GO:0034514	mitochondrial unfolded protein response	2	1	2.17E-02
GO:0035683	memory T cell extravasation	2	1	2.17E-02
GO:0038004	epidermal growth factor receptor ligand maturation	2	1	2.17E-02
GO:0042710	biofilm formation	2	1	2.17E-02
GO:0044010	single-species biofilm formation	2	1	2.17E-02
GO:0044407	single-species biofilm formation in or on host organism	2	1	2.17E-02
GO:0045659	negative regulation of neutrophil differentiation	2	1	2.17E-02
GO:0046166	glyceraldehyde-3-phosphate biosynthetic process	2	1	2.17E-02
GO:0046381	CMP-N-acetylneuraminate metabolic process	2	1	2.17E-02
GO:0050902	leukocyte adhesive activation	2	1	2.17E-02

GO:0051621	regulation of norepinephrine uptake	2	1	2.17E-02
GO:0051943	positive regulation of amino acid uptake involved in syn	2	1	2.17E-02
GO:0051951	positive regulation of glutamate uptake involved in tran	2	1	2.17E-02
GO:0060265	positive regulation of respiratory burst involved in infla	2	1	2.17E-02
GO:0060471	cortical granule exocytosis	2	1	2.17E-02
GO:0061944	negative regulation of protein K48-linked ubiquitination	2	1	2.17E-02
GO:0062029	positive regulation of stress granule assembly	2	1	2.17E-02
GO:0070489	T cell aggregation	2	1	2.17E-02
GO:0070560	protein secretion by platelet	2	1	2.17E-02
GO:0072564	blood microparticle formation	2	1	2.17E-02
GO:0097332	response to antipsychotic drug	2	1	2.17E-02
GO:0097498	endothelial tube lumen extension	2	1	2.17E-02
GO:0098630	aggregation of unicellular organisms	2	1	2.17E-02
GO:1900159	positive regulation of bone mineralization involved in bo	2	1	2.17E-02
GO:1900190	regulation of single-species biofilm formation	2	1	2.17E-02
GO:1900191	negative regulation of single-species biofilm formation	2	1	2.17E-02
GO:1900228	regulation of single-species biofilm formation in or on ho	2	1	2.17E-02
GO:1900229	negative regulation of single-species biofilm formation i	2	1	2.17E-02
GO:1900756	protein processing in phagocytic vesicle	2	1	2.17E-02
GO:1902766	skeletal muscle satellite cell migration	2	1	2.17E-02
GO:1902889	protein localization to spindle microtubule	2	1	2.17E-02
GO:1903433	regulation of constitutive secretory pathway	2	1	2.17E-02
GO:1903515	calcium ion transport from cytosol to endoplasmic reticu	2	1	2.17E-02
GO:1903921	regulation of protein processing in phagocytic vesicle	2	1	2.17E-02
GO:1903923	positive regulation of protein processing in phagocytic v	2	1	2.17E-02
GO:1904298	regulation of transcytosis	2	1	2.17E-02
GO:1904300	positive regulation of transcytosis	2	1	2.17E-02
GO:1904401	cellular response to Thyroid stimulating hormone	2	1	2.17E-02
GO:1904432	regulation of ferrous iron binding	2	1	2.17E-02
GO:1904434	positive regulation of ferrous iron binding	2	1	2.17E-02
GO:1904435	regulation of transferrin receptor binding	2	1	2.17E-02
GO:1904437	positive regulation of transferrin receptor binding	2	1	2.17E-02
GO:1904695	positive regulation of vascular associated smooth muscl	2	1	2.17E-02
GO:1905000	regulation of membrane repolarization during atrial card	2	1	2.17E-02
GO:1905450	negative regulation of Fc-gamma receptor signaling path	2	1	2.17E-02
GO:1905552	positive regulation of protein localization to endoplasmi	2	1	2.17E-02
GO:1905602	positive regulation of receptor-mediated endocytosis inv	2	1	2.17E-02
GO:1905663	positive regulation of telomerase RNA reverse transcript	2	1	2.17E-02
GO:1905696	regulation of polysome binding	2	1	2.17E-02
GO:1990145	maintenance of translational fidelity	2	1	2.17E-02
GO:2000156	regulation of retrograde vesicle-mediated transport, Gol	2	1	2.17E-02
GO:2000332	regulation of blood microparticle formation	2	1	2.17E-02
GO:2000334	positive regulation of blood microparticle formation	2	1	2.17E-02
GO:2000536	negative regulation of entry of bacterium into host cell	2	1	2.17E-02
GO:2000567	regulation of memory T cell activation	2	1	2.17E-02
GO:2000568	positive regulation of memory T cell activation	2	1	2.17E-02
GO:0000322	storage vacuole	2	1	2.17E-02

GO:0005754	mitochondrial proton-transporting ATP synthase, catalyt	2	1	2.17E-02
GO:0034665	integrin alpha1-beta1 complex	2	1	2.17E-02
GO:0034667	integrin alpha3-beta1 complex	2	1	2.17E-02
GO:0034674	integrin alpha5-beta1 complex	2	1	2.17E-02
GO:0034678	integrin alpha8-beta1 complex	2	1	2.17E-02
GO:0034680	integrin alpha10-beta1 complex	2	1	2.17E-02
GO:0034681	integrin alpha11-beta1 complex	2	1	2.17E-02
GO:0043564	Ku70:Ku80 complex	2	1	2.17E-02
GO:0045267	proton-transporting ATP synthase, catalytic core	2	1	2.17E-02
GO:0071133	alpha9-beta1 integrin-ADAM8 complex	2	1	2.17E-02
GO:0097057	TRAF2-GSTP1 complex	2	1	2.17E-02
GO:0098575	lumenal side of lysosomal membrane	2	1	2.17E-02
GO:0099631	postsynaptic endocytic zone cytoplasmic component	2	1	2.17E-02
GO:1990665	AnxA2-p11 complex	2	1	2.17E-02
GO:1990667	PCSK9-AnxA2 complex	2	1	2.17E-02
GO:0001069	regulatory region RNA binding	2	1	2.17E-02
GO:0001097	TFIIH-class transcription factor complex binding	2	1	2.17E-02
GO:0001735	prenylcysteine oxidase activity	2	1	2.17E-02
GO:0002135	CTP binding	2	1	2.17E-02
GO:0004345	glucose-6-phosphate dehydrogenase activity	2	1	2.17E-02
GO:0004365	glyceraldehyde-3-phosphate dehydrogenase (NAD+) (ph	2	1	2.17E-02
GO:0004618	phosphoglycerate kinase activity	2	1	2.17E-02
GO:0004666	prostaglandin-endoperoxide synthase activity	2	1	2.17E-02
GO:0004743	pyruvate kinase activity	2	1	2.17E-02
GO:0004998	transferrin receptor activity	2	1	2.17E-02
GO:0005055	laminin receptor activity	2	1	2.17E-02
GO:0008781	N-acylneuraminate cytidylyltransferase activity	2	1	2.17E-02
GO:0030899	calcium-dependent ATPase activity	2	1	2.17E-02
GO:0031729	CCR4 chemokine receptor binding	2	1	2.17E-02
GO:0032427	GBD domain binding	2	1	2.17E-02
GO:0032564	dATP binding	2	1	2.17E-02
GO:0043891	glyceraldehyde-3-phosphate dehydrogenase (NAD(P)+) (	2	1	2.17E-02
GO:0045142	triplex DNA binding	2	1	2.17E-02
GO:0051903	S-(hydroxymethyl)glutathione dehydrogenase activity	2	1	2.17E-02
GO:0062061	TAP complex binding	2	1	2.17E-02
GO:0070052	collagen V binding	2	1	2.17E-02
GO:1990889	H4K20me3 modified histone binding	2	1	2.17E-02
GO:0002834	regulation of response to tumor cell	21	2	2.17E-02
GO:0043651	linoleic acid metabolic process	21	2	2.17E-02
GO:0044872	lipoprotein localization	21	2	2.17E-02
GO:0140131	positive regulation of lymphocyte chemotaxis	21	2	2.17E-02
GO:1901739	regulation of myoblast fusion	21	2	2.17E-02
GO:0042827	platelet dense granule	21	2	2.17E-02
GO:0002819	regulation of adaptive immune response	198	6	2.17E-02
GO:1903530	regulation of secretion by cell	566	12	2.19E-02
GO:0031333	negative regulation of protein-containing complex asser	146	5	2.21E-02
GO:0006414	translational elongation	55	3	2.21E-02

GO:0038093	Fc receptor signaling pathway	55	3	2.21E-02
GO:2000179	positive regulation of neural precursor cell proliferation	55	3	2.21E-02
GO:0043122	regulation of I-kappaB kinase/NF-kappaB signaling	255	7	2.21E-02
GO:0120032	regulation of plasma membrane bounded cell projection	199	6	2.22E-02
GO:0019932	second-messenger-mediated signaling	314	8	2.23E-02
GO:0002460	adaptive immune response based on somatic recombina	375	9	2.23E-02
GO:0048661	positive regulation of smooth muscle cell proliferation	98	4	2.24E-02
GO:0005794	Golgi apparatus	1652	27	2.26E-02
GO:0019898	extrinsic component of membrane	316	8	2.30E-02
GO:0060491	regulation of cell projection assembly	201	6	2.32E-02
GO:0032392	DNA geometric change	99	4	2.32E-02
GO:0032755	positive regulation of interleukin-6 production	99	4	2.32E-02
GO:0043331	response to dsRNA	56	3	2.32E-02
GO:0051438	regulation of ubiquitin-protein transferase activity	56	3	2.32E-02
GO:0017148	negative regulation of translation	258	7	2.34E-02
GO:0098739	import across plasma membrane	202	6	2.37E-02
GO:0006740	NADPH regeneration	22	2	2.37E-02
GO:0007398	ectoderm development	22	2	2.37E-02
GO:0032891	negative regulation of organic acid transport	22	2	2.37E-02
GO:0033630	positive regulation of cell adhesion mediated by integrin	22	2	2.37E-02
GO:0035584	calcium-mediated signaling using intracellular calcium sc	22	2	2.37E-02
GO:0051444	negative regulation of ubiquitin-protein transferase acti	22	2	2.37E-02
GO:0005697	telomerase holoenzyme complex	22	2	2.37E-02
GO:0031258	lamellipodium membrane	22	2	2.37E-02
GO:0004602	glutathione peroxidase activity	22	2	2.37E-02
GO:0031683	G-protein beta/gamma-subunit complex binding	22	2	2.37E-02
GO:0035255	ionotropic glutamate receptor binding	22	2	2.37E-02
GO:0070034	telomerase RNA binding	22	2	2.37E-02
GO:0030198	extracellular matrix organization	318	8	2.38E-02
GO:0019903	protein phosphatase binding	149	5	2.38E-02
GO:0030316	osteoclast differentiation	100	4	2.39E-02
GO:0034620	cellular response to unfolded protein	100	4	2.39E-02
GO:0036473	cell death in response to oxidative stress	100	4	2.39E-02
GO:1990830	cellular response to leukemia inhibitory factor	100	4	2.39E-02
GO:0043062	extracellular structure organization	319	8	2.42E-02
GO:0032608	interferon-beta production	57	3	2.43E-02
GO:0032648	regulation of interferon-beta production	57	3	2.43E-02
GO:1903053	regulation of extracellular matrix organization	57	3	2.43E-02
GO:0036002	pre-mRNA binding	57	3	2.43E-02
GO:0090066	regulation of anatomical structure size	509	11	2.44E-02
GO:1901652	response to peptide	509	11	2.44E-02
GO:0015908	fatty acid transport	101	4	2.47E-02
GO:1905477	positive regulation of protein localization to membrane	101	4	2.47E-02
GO:1990823	response to leukemia inhibitory factor	101	4	2.47E-02
GO:0006470	protein dephosphorylation	261	7	2.47E-02
GO:0043296	apical junction complex	151	5	2.51E-02
GO:0006695	cholesterol biosynthetic process	58	3	2.54E-02

GO:0006826	iron ion transport	58	3	2.54E-02
GO:0031529	ruffle organization	58	3	2.54E-02
GO:0048008	platelet-derived growth factor receptor signaling pathwa	58	3	2.54E-02
GO:1902653	secondary alcohol biosynthetic process	58	3	2.54E-02
GO:1901019	regulation of calcium ion transmembrane transporter ac	102	4	2.55E-02
GO:0002363	alpha-beta T cell lineage commitment	23	2	2.58E-02
GO:0032727	positive regulation of interferon-alpha production	23	2	2.58E-02
GO:1901673	regulation of mitotic spindle assembly	23	2	2.58E-02
GO:0044295	axonal growth cone	23	2	2.58E-02
GO:0071682	endocytic vesicle lumen	23	2	2.58E-02
GO:0008093	cytoskeletal anchor activity	23	2	2.58E-02
GO:0017134	fibroblast growth factor binding	23	2	2.58E-02
GO:1903076	regulation of protein localization to plasma membrane	103	4	2.63E-02
GO:0019752	carboxylic acid metabolic process	930	17	2.64E-02
GO:0098660	inorganic ion transmembrane transport	930	17	2.64E-02
GO:0002090	regulation of receptor internalization	59	3	2.66E-02
GO:0030042	actin filament depolymerization	59	3	2.66E-02
GO:0043903	regulation of biological process involved in symbiotic int	59	3	2.66E-02
GO:0051289	protein homotetramerization	59	3	2.66E-02
GO:0051653	spindle localization	59	3	2.66E-02
GO:0071709	membrane assembly	59	3	2.66E-02
GO:1903078	positive regulation of protein localization to plasma mer	59	3	2.66E-02
GO:0000502	proteasome complex	59	3	2.66E-02
GO:0022625	cytosolic large ribosomal subunit	59	3	2.66E-02
GO:0016765	transferase activity, transferring alkyl or aryl (other than	59	3	2.66E-02
GO:0070063	RNA polymerase binding	59	3	2.66E-02
GO:0019722	calcium-mediated signaling	208	6	2.68E-02
GO:1901361	organic cyclic compound catabolic process	517	11	2.69E-02
GO:0050729	positive regulation of inflammatory response	154	5	2.70E-02
GO:0032409	regulation of transporter activity	326	8	2.70E-02
GO:0045732	positive regulation of protein catabolic process	209	6	2.74E-02
GO:0071383	cellular response to steroid hormone stimulus	209	6	2.74E-02
GO:0098793	presynapse	519	11	2.76E-02
GO:1905039	carboxylic acid transmembrane transport	155	5	2.77E-02
GO:2001236	regulation of extrinsic apoptotic signaling pathway	155	5	2.77E-02
GO:1905517	macrophage migration	60	3	2.78E-02
GO:0010640	regulation of platelet-derived growth factor receptor sig	24	2	2.79E-02
GO:0060143	positive regulation of syncytium formation by plasma m	24	2	2.79E-02
GO:1904666	regulation of ubiquitin protein ligase activity	24	2	2.79E-02
GO:0031233	intrinsic component of external side of plasma membrar	24	2	2.79E-02
GO:0071782	endoplasmic reticulum tubular network	24	2	2.79E-02
GO:0005355	glucose transmembrane transporter activity	24	2	2.79E-02
GO:0008143	poly(A) binding	24	2	2.79E-02
GO:0015149	hexose transmembrane transporter activity	24	2	2.79E-02
GO:1901796	regulation of signal transduction by p53 class mediator	105	4	2.80E-02
GO:1903825	organic acid transmembrane transport	156	5	2.83E-02
GO:0016853	isomerase activity	156	5	2.83E-02

GO:0044281	small molecule metabolic process	1841	29	2.84E-02
GO:0080135	regulation of cellular response to stress	726	14	2.85E-02
GO:0070663	regulation of leukocyte proliferation	269	7	2.85E-02
GO:0043412	macromolecule modification	3869	54	2.87E-02
GO:0071103	DNA conformation change	106	4	2.89E-02
GO:0047485	protein N-terminus binding	106	4	2.89E-02
GO:1901681	sulfur compound binding	270	7	2.90E-02
GO:0099003	vesicle-mediated transport in synapse	212	6	2.91E-02
GO:0030163	protein catabolic process	1014	18	2.92E-02
GO:0098772	molecular function regulator activity	2003	31	2.94E-02
GO:0007033	vacuole organization	213	6	2.96E-02
GO:0004857	enzyme inhibitor activity	395	9	2.98E-02
GO:0005975	carbohydrate metabolic process	593	12	2.99E-02
GO:0001911	negative regulation of leukocyte mediated cytotoxicity	25	2	3.01E-02
GO:0019835	cytolysis	25	2	3.01E-02
GO:0034114	regulation of heterotypic cell-cell adhesion	25	2	3.01E-02
GO:0045932	negative regulation of muscle contraction	25	2	3.01E-02
GO:0046716	muscle cell cellular homeostasis	25	2	3.01E-02
GO:0051984	positive regulation of chromosome segregation	25	2	3.01E-02
GO:0086064	cell communication by electrical coupling involved in car	25	2	3.01E-02
GO:0090023	positive regulation of neutrophil chemotaxis	25	2	3.01E-02
GO:1904996	positive regulation of leukocyte adhesion to vascular en	25	2	3.01E-02
GO:0050431	transforming growth factor beta binding	25	2	3.01E-02
GO:0043030	regulation of macrophage activation	62	3	3.02E-02
GO:0045428	regulation of nitric oxide biosynthetic process	62	3	3.02E-02
GO:0032870	cellular response to hormone stimulus	595	12	3.06E-02
GO:0009896	positive regulation of catabolic process	528	11	3.07E-02
GO:0016064	immunoglobulin mediated immune response	215	6	3.08E-02
GO:0006469	negative regulation of protein kinase activity	216	6	3.14E-02
GO:0030030	cell projection organization	1548	25	3.14E-02
GO:0032757	positive regulation of interleukin-8 production	63	3	3.15E-02
GO:0044091	membrane biogenesis	63	3	3.15E-02
GO:0045453	bone resorption	63	3	3.15E-02
GO:2001258	negative regulation of cation channel activity	63	3	3.15E-02
GO:0033209	tumor necrosis factor-mediated signaling pathway	109	4	3.15E-02
GO:1904063	negative regulation of cation transmembrane transport	109	4	3.15E-02
GO:1902936	phosphatidylinositol bisphosphate binding	109	4	3.15E-02
GO:0051251	positive regulation of lymphocyte activation	400	9	3.19E-02
GO:0070482	response to oxygen levels	337	8	3.20E-02
GO:0001905	activation of membrane attack complex	3	1	3.24E-02
GO:0001969	regulation of activation of membrane attack complex	3	1	3.24E-02
GO:0002581	negative regulation of antigen processing and presentat	3	1	3.24E-02
GO:0010751	negative regulation of nitric oxide mediated signal trans	3	1	3.24E-02
GO:0015912	short-chain fatty acid transport	3	1	3.24E-02
GO:0021551	central nervous system morphogenesis	3	1	3.24E-02
GO:0021769	orbitofrontal cortex development	3	1	3.24E-02
GO:0021943	formation of radial glial scaffolds	3	1	3.24E-02

GO:0030327	prenylated protein catabolic process	3	1	3.24E-02
GO:0031635	adenylate cyclase-inhibiting opioid receptor signaling pa	3	1	3.24E-02
GO:0032226	positive regulation of synaptic transmission, dopaminerg	3	1	3.24E-02
GO:0033227	dsRNA transport	3	1	3.24E-02
GO:0033484	nitric oxide homeostasis	3	1	3.24E-02
GO:0034140	negative regulation of toll-like receptor 3 signaling pathw	3	1	3.24E-02
GO:0034462	small-subunit processome assembly	3	1	3.24E-02
GO:0043163	cell envelope organization	3	1	3.24E-02
GO:0043335	protein unfolding	3	1	3.24E-02
GO:0043366	beta selection	3	1	3.24E-02
GO:0045041	protein import into mitochondrial intermembrane space	3	1	3.24E-02
GO:0045105	intermediate filament polymerization or depolymerizati	3	1	3.24E-02
GO:0045196	establishment or maintenance of neuroblast polarity	3	1	3.24E-02
GO:0045200	establishment of neuroblast polarity	3	1	3.24E-02
GO:0045658	regulation of neutrophil differentiation	3	1	3.24E-02
GO:0046294	formaldehyde catabolic process	3	1	3.24E-02
GO:0050434	positive regulation of viral transcription	3	1	3.24E-02
GO:0055095	lipoprotein particle mediated signaling	3	1	3.24E-02
GO:0055096	low-density lipoprotein particle mediated signaling	3	1	3.24E-02
GO:0061738	late endosomal microautophagy	3	1	3.24E-02
GO:0061740	protein targeting to lysosome involved in chaperone-me	3	1	3.24E-02
GO:0070543	response to linoleic acid	3	1	3.24E-02
GO:0070966	nuclear-transcribed mRNA catabolic process, no-go deca	3	1	3.24E-02
GO:0072535	tumor necrosis factor (ligand) superfamily member 11 p	3	1	3.24E-02
GO:0086101	endothelin receptor signaling pathway involved in heart	3	1	3.24E-02
GO:0097051	establishment of protein localization to endoplasmic ret	3	1	3.24E-02
GO:1902309	negative regulation of peptidyl-serine dephosphorylatio	3	1	3.24E-02
GO:1903614	negative regulation of protein tyrosine phosphatase acti	3	1	3.24E-02
GO:1904400	response to Thyroid stimulating hormone	3	1	3.24E-02
GO:1904430	negative regulation of t-circle formation	3	1	3.24E-02
GO:1904588	cellular response to glycoprotein	3	1	3.24E-02
GO:1904589	regulation of protein import	3	1	3.24E-02
GO:1904669	ATP export	3	1	3.24E-02
GO:1904845	cellular response to L-glutamine	3	1	3.24E-02
GO:1904879	positive regulation of calcium ion transmembrane trans	3	1	3.24E-02
GO:1904971	regulation of viral translation	3	1	3.24E-02
GO:1904973	positive regulation of viral translation	3	1	3.24E-02
GO:1905451	positive regulation of Fc-gamma receptor signaling path	3	1	3.24E-02
GO:1905550	regulation of protein localization to endoplasmic reticul	3	1	3.24E-02
GO:1905581	positive regulation of low-density lipoprotein particle cl	3	1	3.24E-02
GO:1905661	regulation of telomerase RNA reverse transcriptase acti	3	1	3.24E-02
GO:1905686	positive regulation of plasma membrane repair	3	1	3.24E-02
GO:1990428	miRNA transport	3	1	3.24E-02
GO:2000307	regulation of tumor necrosis factor (ligand) superfamily	3	1	3.24E-02
GO:2000474	regulation of opioid receptor signaling pathway	3	1	3.24E-02
GO:2000645	negative regulation of receptor catabolic process	3	1	3.24E-02
GO:2000978	negative regulation of forebrain neuron differentiation	3	1	3.24E-02

GO:0031523	Myb complex	3	1	3.24E-02
GO:0031905	early endosome lumen	3	1	3.24E-02
GO:0032998	Fc-epsilon receptor I complex	3	1	3.24E-02
GO:0033001	Fc-gamma receptor III complex	3	1	3.24E-02
GO:0034666	integrin alpha2-beta1 complex	3	1	3.24E-02
GO:0034668	integrin alpha4-beta1 complex	3	1	3.24E-02
GO:0034683	integrin alphav-beta3 complex	3	1	3.24E-02
GO:0035866	alphav-beta3 integrin-PKCalpha complex	3	1	3.24E-02
GO:0035868	alphav-beta3 integrin-HMGB1 complex	3	1	3.24E-02
GO:0042629	mast cell granule	3	1	3.24E-02
GO:0044393	microspike	3	1	3.24E-02
GO:0070418	DNA-dependent protein kinase complex	3	1	3.24E-02
GO:0071062	alphav-beta3 integrin-vitronectin complex	3	1	3.24E-02
GO:0072563	endothelial microparticle	3	1	3.24E-02
GO:0097513	myosin II filament	3	1	3.24E-02
GO:1903958	nitric-oxide synthase complex	3	1	3.24E-02
GO:0002134	UTP binding	3	1	3.24E-02
GO:0004332	fructose-bisphosphate aldolase activity	3	1	3.24E-02
GO:0004802	transketolase activity	3	1	3.24E-02
GO:0008426	protein kinase C inhibitor activity	3	1	3.24E-02
GO:0015636	short-chain fatty acid transmembrane transporter activi	3	1	3.24E-02
GO:0016774	phosphotransferase activity, carboxyl group as acceptor	3	1	3.24E-02
GO:0016971	flavin-linked sulfhydryl oxidase activity	3	1	3.24E-02
GO:0017098	sulfonylurea receptor binding	3	1	3.24E-02
GO:0030226	apolipoprotein receptor activity	3	1	3.24E-02
GO:0031800	type 3 metabotropic glutamate receptor binding	3	1	3.24E-02
GO:0031997	N-terminal myristoylation domain binding	3	1	3.24E-02
GO:0032558	adenyl deoxyribonucleotide binding	3	1	3.24E-02
GO:0034988	Fc-gamma receptor I complex binding	3	1	3.24E-02
GO:0042610	CD8 receptor binding	3	1	3.24E-02
GO:0045027	DNA end binding	3	1	3.24E-02
GO:1902945	metalloendopeptidase activity involved in amyloid precu	3	1	3.24E-02
GO:1990518	single-stranded 3'-5' DNA helicase activity	3	1	3.24E-02
GO:0000027	ribosomal large subunit assembly	26	2	3.24E-02
GO:0002433	immune response-regulating cell surface receptor signal	26	2	3.24E-02
GO:0002726	positive regulation of T cell cytokine production	26	2	3.24E-02
GO:0035821	modulation of process of another organism	26	2	3.24E-02
GO:0038096	Fc-gamma receptor signaling pathway involved in phago	26	2	3.24E-02
GO:0043931	ossification involved in bone maturation	26	2	3.24E-02
GO:0060314	regulation of ryanodine-sensitive calcium-release chann	26	2	3.24E-02
GO:0086013	membrane repolarization during cardiac muscle cell acti	26	2	3.24E-02
GO:1903306	negative regulation of regulated secretory pathway	26	2	3.24E-02
GO:1903901	negative regulation of viral life cycle	26	2	3.24E-02
GO:1905523	positive regulation of macrophage migration	26	2	3.24E-02
GO:0046930	pore complex	26	2	3.24E-02
GO:0002573	myeloid leukocyte differentiation	218	6	3.27E-02
GO:0019724	B cell mediated immunity	218	6	3.27E-02

GO:0046700	heterocycle catabolic process	468	10	3.32E-02
GO:1902850	microtubule cytoskeleton organization involved in mitos	163	5	3.33E-02
GO:0043436	oxoacid metabolic process	957	17	3.34E-02
GO:0016126	sterol biosynthetic process	65	3	3.41E-02
GO:0080164	regulation of nitric oxide metabolic process	65	3	3.41E-02
GO:0048259	regulation of receptor-mediated endocytosis	112	4	3.43E-02
GO:0031954	positive regulation of protein autophosphorylation	27	2	3.47E-02
GO:0034143	regulation of toll-like receptor 4 signaling pathway	27	2	3.47E-02
GO:0051291	protein heterooligomerization	27	2	3.47E-02
GO:0070269	pyroptosis	27	2	3.47E-02
GO:1901623	regulation of lymphocyte chemotaxis	27	2	3.47E-02
GO:1904357	negative regulation of telomere maintenance via telome	27	2	3.47E-02
GO:2000737	negative regulation of stem cell differentiation	27	2	3.47E-02
GO:0015145	monosaccharide transmembrane transporter activity	27	2	3.47E-02
GO:0038187	pattern recognition receptor activity	27	2	3.47E-02
GO:0051494	negative regulation of cytoskeleton organization	165	5	3.48E-02
GO:0032412	regulation of ion transmembrane transporter activity	281	7	3.49E-02
GO:0043542	endothelial cell migration	281	7	3.49E-02
GO:0002456	T cell mediated immunity	113	4	3.53E-02
GO:0006082	organic acid metabolic process	964	17	3.54E-02
GO:0006879	cellular iron ion homeostasis	66	3	3.54E-02
GO:0010517	regulation of phospholipase activity	66	3	3.54E-02
GO:0010812	negative regulation of cell-substrate adhesion	66	3	3.54E-02
GO:0017156	calcium-ion regulated exocytosis	66	3	3.54E-02
GO:0030219	megakaryocyte differentiation	66	3	3.54E-02
GO:0030837	negative regulation of actin filament polymerization	66	3	3.54E-02
GO:0008235	metalloexopeptidase activity	66	3	3.54E-02
GO:0048666	neuron development	1112	19	3.56E-02
GO:0099173	postsynapse organization	166	5	3.56E-02
GO:0044270	cellular nitrogen compound catabolic process	474	10	3.57E-02
GO:0009615	response to virus	409	9	3.60E-02
GO:2000113	negative regulation of cellular macromolecule biosynthe	283	7	3.61E-02
GO:0002526	acute inflammatory response	114	4	3.63E-02
GO:1903707	negative regulation of hemopoiesis	114	4	3.63E-02
GO:0043271	negative regulation of ion transport	167	5	3.64E-02
GO:0140297	DNA-binding transcription factor binding	476	10	3.66E-02
GO:1904377	positive regulation of protein localization to cell periphe	67	3	3.68E-02
GO:0006972	hyperosmotic response	28	2	3.71E-02
GO:0009048	dosage compensation by inactivation of X chromosome	28	2	3.71E-02
GO:0010758	regulation of macrophage chemotaxis	28	2	3.71E-02
GO:0010818	T cell chemotaxis	28	2	3.71E-02
GO:0031342	negative regulation of cell killing	28	2	3.71E-02
GO:0051156	glucose 6-phosphate metabolic process	28	2	3.71E-02
GO:0071624	positive regulation of granulocyte chemotaxis	28	2	3.71E-02
GO:1903959	regulation of anion transmembrane transport	28	2	3.71E-02
GO:0031528	microvillus membrane	28	2	3.71E-02
GO:0043495	protein-membrane adaptor activity	28	2	3.71E-02

GO:0010970	transport along microtubule	168	5	3.72E-02
GO:0032993	protein-DNA complex	225	6	3.72E-02
GO:0032410	negative regulation of transporter activity	115	4	3.73E-02
GO:0015934	large ribosomal subunit	115	4	3.73E-02
GO:0007568	aging	169	5	3.80E-02
GO:1902904	negative regulation of supramolecular fiber organization	169	5	3.80E-02
GO:0042987	amyloid precursor protein catabolic process	68	3	3.82E-02
GO:0060193	positive regulation of lipase activity	68	3	3.82E-02
GO:0030041	actin filament polymerization	170	5	3.88E-02
GO:0045935	positive regulation of nucleobase-containing compound	2052	31	3.89E-02
GO:0007249	I-kappaB kinase/NF-kappaB signaling	288	7	3.90E-02
GO:0061061	muscle structure development	688	13	3.91E-02
GO:0006622	protein targeting to lysosome	29	2	3.96E-02
GO:0007263	nitric oxide mediated signal transduction	29	2	3.96E-02
GO:0010800	positive regulation of peptidyl-threonine phosphorylatio	29	2	3.96E-02
GO:0015813	L-glutamate transmembrane transport	29	2	3.96E-02
GO:0022011	myelination in peripheral nervous system	29	2	3.96E-02
GO:0032292	peripheral nervous system axon ensheathment	29	2	3.96E-02
GO:0032367	intracellular cholesterol transport	29	2	3.96E-02
GO:0032607	interferon-alpha production	29	2	3.96E-02
GO:0032647	regulation of interferon-alpha production	29	2	3.96E-02
GO:0051938	L-glutamate import	29	2	3.96E-02
GO:0060074	synapse maturation	29	2	3.96E-02
GO:0070977	bone maturation	29	2	3.96E-02
GO:0072012	glomerulus vasculature development	29	2	3.96E-02
GO:0095500	acetylcholine receptor signaling pathway	29	2	3.96E-02
GO:1902430	negative regulation of amyloid-beta formation	29	2	3.96E-02
GO:0051119	sugar transmembrane transporter activity	29	2	3.96E-02
GO:0034394	protein localization to cell surface	69	3	3.96E-02
GO:0043270	positive regulation of ion transport	289	7	3.96E-02
GO:0031406	carboxylic acid binding	171	5	3.97E-02
GO:0031346	positive regulation of cell projection organization	352	8	3.98E-02
GO:0030425	dendrite	621	12	4.03E-02
GO:0120036	plasma membrane bounded cell projection organization	1509	24	4.03E-02
GO:0006259	DNA metabolic process	1054	18	4.03E-02
GO:0002718	regulation of cytokine production involved in immune re	118	4	4.04E-02
GO:0051261	protein depolymerization	118	4	4.04E-02
GO:1905368	peptidase complex	118	4	4.04E-02
GO:0031490	chromatin DNA binding	118	4	4.04E-02
GO:0051881	regulation of mitochondrial membrane potential	70	3	4.11E-02
GO:0004860	protein kinase inhibitor activity	70	3	4.11E-02
GO:0097447	dendritic tree	623	12	4.11E-02
GO:0098662	inorganic cation transmembrane transport	837	15	4.14E-02
GO:0031300	intrinsic component of organelle membrane	420	9	4.14E-02
GO:0032609	interferon-gamma production	119	4	4.14E-02
GO:0032649	regulation of interferon-gamma production	119	4	4.14E-02
GO:1990782	protein tyrosine kinase binding	119	4	4.14E-02

GO:0010632	regulation of epithelial cell migration	292	7	4.15E-02
GO:0001778	plasma membrane repair	30	2	4.22E-02
GO:0001975	response to amphetamine	30	2	4.22E-02
GO:0002360	T cell lineage commitment	30	2	4.22E-02
GO:0007549	dosage compensation	30	2	4.22E-02
GO:0009651	response to salt stress	30	2	4.22E-02
GO:0042744	hydrogen peroxide catabolic process	30	2	4.22E-02
GO:0060142	regulation of syncytium formation by plasma membrane	30	2	4.22E-02
GO:0071480	cellular response to gamma radiation	30	2	4.22E-02
GO:0005640	nuclear outer membrane	30	2	4.22E-02
GO:0051146	striated muscle cell differentiation	293	7	4.22E-02
GO:0021543	pallium development	174	5	4.22E-02
GO:0048812	neuron projection morphogenesis	626	12	4.24E-02
GO:0002367	cytokine production involved in immune response	120	4	4.25E-02
GO:0007006	mitochondrial membrane organization	120	4	4.25E-02
GO:0035967	cellular response to topologically incorrect protein	120	4	4.25E-02
GO:0018193	peptidyl-amino acid modification	1287	21	4.25E-02
GO:0019439	aromatic compound catabolic process	489	10	4.26E-02
GO:0022613	ribonucleoprotein complex biogenesis	489	10	4.26E-02
GO:0060348	bone development	233	6	4.29E-02
GO:0002038	positive regulation of L-glutamate import across plasma	4	1	4.29E-02
GO:0002159	desmosome assembly	4	1	4.29E-02
GO:0002265	astrocyte activation involved in immune response	4	1	4.29E-02
GO:0002296	T-helper 1 cell lineage commitment	4	1	4.29E-02
GO:0002477	antigen processing and presentation of exogenous pepti	4	1	4.29E-02
GO:0002605	negative regulation of dendritic cell antigen processing a	4	1	4.29E-02
GO:0006933	negative regulation of cell adhesion involved in substrate	4	1	4.29E-02
GO:0007161	calcium-independent cell-matrix adhesion	4	1	4.29E-02
GO:0009051	pentose-phosphate shunt, oxidative branch	4	1	4.29E-02
GO:0010710	regulation of collagen catabolic process	4	1	4.29E-02
GO:0010748	negative regulation of long-chain fatty acid import acros	4	1	4.29E-02
GO:0010825	positive regulation of centrosome duplication	4	1	4.29E-02
GO:0030035	microspike assembly	4	1	4.29E-02
GO:0030043	actin filament fragmentation	4	1	4.29E-02
GO:0032382	positive regulation of intracellular sterol transport	4	1	4.29E-02
GO:0032385	positive regulation of intracellular cholesterol transport	4	1	4.29E-02
GO:0033366	protein localization to secretory granule	4	1	4.29E-02
GO:0033499	galactose catabolic process via UDP-galactose	4	1	4.29E-02
GO:0034465	response to carbon monoxide	4	1	4.29E-02
GO:0035709	memory T cell activation	4	1	4.29E-02
GO:0038156	interleukin-3-mediated signaling pathway	4	1	4.29E-02
GO:0038189	neuropilin signaling pathway	4	1	4.29E-02
GO:0042268	regulation of cytolysis	4	1	4.29E-02
GO:0045901	positive regulation of translational elongation	4	1	4.29E-02
GO:0045905	positive regulation of translational termination	4	1	4.29E-02
GO:0046292	formaldehyde metabolic process	4	1	4.29E-02
GO:0046338	phosphatidylethanolamine catabolic process	4	1	4.29E-02

GO:0046452	dihydrofolate metabolic process	4	1	4.29E-02
GO:0046967	cytosol to endoplasmic reticulum transport	4	1	4.29E-02
GO:0051611	regulation of serotonin uptake	4	1	4.29E-02
GO:0051771	negative regulation of nitric-oxide synthase biosynthetic	4	1	4.29E-02
GO:0051941	regulation of amino acid uptake involved in synaptic tra	4	1	4.29E-02
GO:0051946	regulation of glutamate uptake involved in transmission	4	1	4.29E-02
GO:0060699	regulation of endoribonuclease activity	4	1	4.29E-02
GO:0060912	cardiac cell fate specification	4	1	4.29E-02
GO:0070668	positive regulation of mast cell proliferation	4	1	4.29E-02
GO:0071211	protein targeting to vacuole involved in autophagy	4	1	4.29E-02
GO:0071603	endothelial cell-cell adhesion	4	1	4.29E-02
GO:0072011	glomerular endothelium development	4	1	4.29E-02
GO:0072513	positive regulation of secondary heart field cardioblast p	4	1	4.29E-02
GO:0090038	negative regulation of protein kinase C signaling	4	1	4.29E-02
GO:0090168	Golgi reassembly	4	1	4.29E-02
GO:0098795	global gene silencing by mRNA cleavage	4	1	4.29E-02
GO:0140192	regulation of adenylate cyclase-activating adrenergic rec	4	1	4.29E-02
GO:0140199	negative regulation of adenylate cyclase-activating adre	4	1	4.29E-02
GO:0140213	negative regulation of long-chain fatty acid import into c	4	1	4.29E-02
GO:0150111	regulation of transepithelial transport	4	1	4.29E-02
GO:1901685	glutathione derivative metabolic process	4	1	4.29E-02
GO:1901687	glutathione derivative biosynthetic process	4	1	4.29E-02
GO:1902308	regulation of peptidyl-serine dephosphorylation	4	1	4.29E-02
GO:1902396	protein localization to bicellular tight junction	4	1	4.29E-02
GO:1904154	positive regulation of retrograde protein transport, ER to	4	1	4.29E-02
GO:1904844	response to L-glutamine	4	1	4.29E-02
GO:1905167	positive regulation of lysosomal protein catabolic proces	4	1	4.29E-02
GO:1905274	regulation of modification of postsynaptic actin cytoskel	4	1	4.29E-02
GO:1905595	regulation of low-density lipoprotein particle receptor b	4	1	4.29E-02
GO:1990809	endoplasmic reticulum tubular network membrane orga	4	1	4.29E-02
GO:2000435	negative regulation of protein neddylation	4	1	4.29E-02
GO:2000439	positive regulation of monocyte extravasation	4	1	4.29E-02
GO:2000566	positive regulation of CD8-positive, alpha-beta T cell pro	4	1	4.29E-02
GO:2000587	negative regulation of platelet-derived growth factor rec	4	1	4.29E-02
GO:0000015	phosphopyruvate hydratase complex	4	1	4.29E-02
GO:0005726	perichromatin fibrils	4	1	4.29E-02
GO:0005826	actomyosin contractile ring	4	1	4.29E-02
GO:0005853	eukaryotic translation elongation factor 1 complex	4	1	4.29E-02
GO:0005862	muscle thin filament tropomyosin	4	1	4.29E-02
GO:0035867	alphav-beta3 integrin-IGF-1-IGF1R complex	4	1	4.29E-02
GO:0071664	catenin-TCF7L2 complex	4	1	4.29E-02
GO:0098871	postsynaptic actin cytoskeleton	4	1	4.29E-02
GO:0004634	phosphopyruvate hydratase activity	4	1	4.29E-02
GO:0004784	superoxide dismutase activity	4	1	4.29E-02
GO:0005354	galactose transmembrane transporter activity	4	1	4.29E-02
GO:0010856	adenylate cyclase activator activity	4	1	4.29E-02
GO:0016721	oxidoreductase activity, acting on superoxide radicals as	4	1	4.29E-02

GO:0032554	purine deoxyribonucleotide binding	4	1	4.29E-02
GO:0032557	pyrimidine ribonucleotide binding	4	1	4.29E-02
GO:0034191	apolipoprotein A-I receptor binding	4	1	4.29E-02
GO:0034458	3'-5' RNA helicase activity	4	1	4.29E-02
GO:0043530	adenosine 5'-monophosphoramidase activity	4	1	4.29E-02
GO:0043532	angiostatin binding	4	1	4.29E-02
GO:0043812	phosphatidylinositol-4-phosphate phosphatase activity	4	1	4.29E-02
GO:0044547	DNA topoisomerase binding	4	1	4.29E-02
GO:0070026	nitric oxide binding	4	1	4.29E-02
GO:0106006	cytoskeletal protein-membrane anchor activity	4	1	4.29E-02
GO:1904399	heparan sulfate binding	4	1	4.29E-02
GO:1905172	RISC complex binding	4	1	4.29E-02
GO:0045597	positive regulation of cell differentiation	915	16	4.32E-02
GO:0030301	cholesterol transport	121	4	4.36E-02
GO:0009306	protein secretion	359	8	4.38E-02
GO:0010634	positive regulation of epithelial cell migration	176	5	4.40E-02
GO:0060090	molecular adaptor activity	492	10	4.41E-02
GO:0030669	clathrin-coated endocytic vesicle membrane	72	3	4.41E-02
GO:0003678	DNA helicase activity	72	3	4.41E-02
GO:0048029	monosaccharide binding	72	3	4.41E-02
GO:0030217	T cell differentiation	296	7	4.41E-02
GO:0035592	establishment of protein localization to extracellular reg	360	8	4.44E-02
GO:0031968	organelle outer membrane	235	6	4.44E-02
GO:0002698	negative regulation of immune effector process	122	4	4.47E-02
GO:0048675	axon extension	122	4	4.47E-02
GO:0010880	regulation of release of sequestered calcium ion into cyt	31	2	4.47E-02
GO:0051491	positive regulation of filopodium assembly	31	2	4.47E-02
GO:0060390	regulation of SMAD protein signal transduction	31	2	4.47E-02
GO:0061437	renal system vasculature development	31	2	4.47E-02
GO:0061440	kidney vasculature development	31	2	4.47E-02
GO:0086011	membrane repolarization during action potential	31	2	4.47E-02
GO:1900027	regulation of ruffle assembly	31	2	4.47E-02
GO:1902751	positive regulation of cell cycle G2/M phase transition	31	2	4.47E-02
GO:1905145	cellular response to acetylcholine	31	2	4.47E-02
GO:0051059	NF-kappaB binding	31	2	4.47E-02
GO:0002263	cell activation involved in immune response	297	7	4.48E-02
GO:0019362	pyridine nucleotide metabolic process	73	3	4.56E-02
GO:0045665	negative regulation of neuron differentiation	73	3	4.56E-02
GO:0046496	nicotinamide nucleotide metabolic process	73	3	4.56E-02
GO:0071260	cellular response to mechanical stimulus	73	3	4.56E-02
GO:0043022	ribosome binding	73	3	4.56E-02
GO:0036477	somatodendritic compartment	849	15	4.58E-02
GO:0006413	translational initiation	123	4	4.58E-02
GO:0019867	outer membrane	237	6	4.59E-02
GO:0048699	generation of neurons	1453	23	4.62E-02
GO:0060759	regulation of response to cytokine stimulus	179	5	4.67E-02
GO:0002381	immunoglobulin production involved in immunoglobulin	74	3	4.71E-02

GO:0019210	kinase inhibitor activity	74	3	4.71E-02
GO:0001516	prostaglandin biosynthetic process	32	2	4.74E-02
GO:0010644	cell communication by electrical coupling	32	2	4.74E-02
GO:0010743	regulation of macrophage derived foam cell differentiati	32	2	4.74E-02
GO:0014044	Schwann cell development	32	2	4.74E-02
GO:0032366	intracellular sterol transport	32	2	4.74E-02
GO:0045070	positive regulation of viral genome replication	32	2	4.74E-02
GO:0045671	negative regulation of osteoclast differentiation	32	2	4.74E-02
GO:0046457	prostanoid biosynthetic process	32	2	4.74E-02
GO:0046856	phosphatidylinositol dephosphorylation	32	2	4.74E-02
GO:1901889	negative regulation of cell junction assembly	32	2	4.74E-02
GO:1903955	positive regulation of protein targeting to mitochondrio	32	2	4.74E-02
GO:0090482	vitamin transmembrane transporter activity	32	2	4.74E-02
GO:0050670	regulation of lymphocyte proliferation	239	6	4.75E-02
GO:0030182	neuron differentiation	1381	22	4.78E-02
GO:0009410	response to xenobiotic stimulus	432	9	4.80E-02
GO:0010639	negative regulation of organelle organization	366	8	4.80E-02
GO:0006869	lipid transport	433	9	4.86E-02
GO:0051640	organelle localization	570	11	4.87E-02
GO:0032890	regulation of organic acid transport	75	3	4.87E-02
GO:0045669	positive regulation of osteoblast differentiation	75	3	4.87E-02
GO:0071479	cellular response to ionizing radiation	75	3	4.87E-02
GO:0120192	tight junction assembly	75	3	4.87E-02
GO:0010608	post-transcriptional regulation of gene expression	1080	18	4.90E-02
GO:0046390	ribose phosphate biosynthetic process	241	6	4.91E-02
GO:0071692	protein localization to extracellular region	368	8	4.93E-02
GO:0016324	apical plasma membrane	368	8	4.93E-02
GO:0016616	oxidoreductase activity, acting on the CH-OH group of d	126	4	4.93E-02
GO:0042129	regulation of T cell proliferation	182	5	4.95E-02
GO:0120039	plasma membrane bounded cell projection morphogene	642	12	4.96E-02
GO:0051047	positive regulation of secretion	304	7	4.96E-02
GO:0032984	protein-containing complex disassembly	242	6	4.99E-02
GO:0043171	peptide catabolic process	33	2	5.01E-02
GO:0045684	positive regulation of epidermis development	33	2	5.01E-02
GO:0048799	animal organ maturation	33	2	5.01E-02
GO:0051497	negative regulation of stress fiber assembly	33	2	5.01E-02
GO:0090169	regulation of spindle assembly	33	2	5.01E-02
GO:1902253	regulation of intrinsic apoptotic signaling pathway by p5	33	2	5.01E-02
GO:1902992	negative regulation of amyloid precursor protein catabo	33	2	5.01E-02
GO:0019956	chemokine binding	33	2	5.01E-02
GO:0042273	ribosomal large subunit biogenesis	76	3	5.03E-02
GO:0016234	inclusion body	76	3	5.03E-02
GO:0031965	nuclear membrane	305	7	5.04E-02
GO:0072686	mitotic spindle	183	5	5.04E-02
GO:0032944	regulation of mononuclear cell proliferation	243	6	5.07E-02
GO:0051656	establishment of organelle localization	437	9	5.09E-02
GO:0007264	small GTPase mediated signal transduction	506	10	5.14E-02

GO:0042826	histone deacetylase binding	128	4	5.17E-02
GO:0048858	cell projection morphogenesis	647	12	5.20E-02
GO:0032729	positive regulation of interferon-gamma production	77	3	5.20E-02
GO:0045824	negative regulation of innate immune response	77	3	5.20E-02
GO:0003724	RNA helicase activity	77	3	5.20E-02
GO:0010629	negative regulation of gene expression	1551	24	5.23E-02
GO:1905952	regulation of lipid localization	185	5	5.24E-02
GO:0006820	anion transport	508	10	5.26E-02
GO:0051346	negative regulation of hydrolase activity	373	8	5.26E-02
GO:0034383	low-density lipoprotein particle clearance	34	2	5.29E-02
GO:0036336	dendritic cell migration	34	2	5.29E-02
GO:0045648	positive regulation of erythrocyte differentiation	34	2	5.29E-02
GO:0046475	glycerophospholipid catabolic process	34	2	5.29E-02
GO:0048333	mesodermal cell differentiation	34	2	5.29E-02
GO:0090022	regulation of neutrophil chemotaxis	34	2	5.29E-02
GO:1905144	response to acetylcholine	34	2	5.29E-02
GO:0043531	ADP binding	34	2	5.29E-02
GO:0052866	phosphatidylinositol phosphate phosphatase activity	34	2	5.29E-02
GO:0051928	positive regulation of calcium ion transport	129	4	5.29E-02
GO:0002034	maintenance of blood vessel diameter homeostasis by r	5	1	5.33E-02
GO:0002419	T cell mediated cytotoxicity directed against tumor cell t	5	1	5.33E-02
GO:0002580	regulation of antigen processing and presentation of pe	5	1	5.33E-02
GO:0003072	renal control of peripheral vascular resistance involved i	5	1	5.33E-02
GO:0010286	heat acclimation	5	1	5.33E-02
GO:0014905	myoblast fusion involved in skeletal muscle regeneratio	5	1	5.33E-02
GO:0015757	galactose transmembrane transport	5	1	5.33E-02
GO:0019388	galactose catabolic process	5	1	5.33E-02
GO:0019732	antifungal humoral response	5	1	5.33E-02
GO:0022614	membrane to membrane docking	5	1	5.33E-02
GO:0032379	positive regulation of intracellular lipid transport	5	1	5.33E-02
GO:0035385	Roundabout signaling pathway	5	1	5.33E-02
GO:0038129	ERBB3 signaling pathway	5	1	5.33E-02
GO:0044860	protein localization to plasma membrane raft	5	1	5.33E-02
GO:0045113	regulation of integrin biosynthetic process	5	1	5.33E-02
GO:0046833	positive regulation of RNA export from nucleus	5	1	5.33E-02
GO:0050748	negative regulation of lipoprotein metabolic process	5	1	5.33E-02
GO:0051029	rRNA transport	5	1	5.33E-02
GO:0051295	establishment of meiotic spindle localization	5	1	5.33E-02
GO:0051343	positive regulation of cyclic-nucleotide phosphodiestera	5	1	5.33E-02
GO:0060501	positive regulation of epithelial cell proliferation involve	5	1	5.33E-02
GO:0061945	regulation of protein K48-linked ubiquitination	5	1	5.33E-02
GO:0062028	regulation of stress granule assembly	5	1	5.33E-02
GO:0070370	cellular heat acclimation	5	1	5.33E-02
GO:0070434	positive regulation of nucleotide-binding oligomerizatio	5	1	5.33E-02
GO:0071681	cellular response to indole-3-methanol	5	1	5.33E-02
GO:0071724	response to diacyl bacterial lipopeptide	5	1	5.33E-02
GO:0071726	cellular response to diacyl bacterial lipopeptide	5	1	5.33E-02

GO:0090156	cellular sphingolipid homeostasis	5	1	5.33E-02
GO:0098961	dendritic transport of ribonucleoprotein complex	5	1	5.33E-02
GO:0098963	dendritic transport of messenger ribonucleoprotein com	5	1	5.33E-02
GO:0099159	regulation of modification of postsynaptic structure	5	1	5.33E-02
GO:1902037	negative regulation of hematopoietic stem cell different	5	1	5.33E-02
GO:1904016	response to Thyroglobulin triiodothyronine	5	1	5.33E-02
GO:1905599	positive regulation of low-density lipoprotein receptor a	5	1	5.33E-02
GO:2000172	regulation of branching morphogenesis of a nerve	5	1	5.33E-02
GO:2000437	regulation of monocyte extravasation	5	1	5.33E-02
GO:2000503	positive regulation of natural killer cell chemotaxis	5	1	5.33E-02
GO:2000977	regulation of forebrain neuron differentiation	5	1	5.33E-02
GO:2001205	negative regulation of osteoclast development	5	1	5.33E-02
GO:0005642	annulate lamellae	5	1	5.33E-02
GO:0032997	Fc receptor complex	5	1	5.33E-02
GO:0046696	lipopolysaccharide receptor complex	5	1	5.33E-02
GO:0097013	phagocytic vesicle lumen	5	1	5.33E-02
GO:0097226	sperm mitochondrial sheath	5	1	5.33E-02
GO:0097433	dense body	5	1	5.33E-02
GO:0098826	endoplasmic reticulum tubular network membrane	5	1	5.33E-02
GO:0098843	postsynaptic endocytic zone	5	1	5.33E-02
GO:0001648	proteinase-activated receptor activity	5	1	5.33E-02
GO:0015038	glutathione disulfide oxidoreductase activity	5	1	5.33E-02
GO:0015057	thrombin-activated receptor activity	5	1	5.33E-02
GO:0015288	porin activity	5	1	5.33E-02
GO:0016314	phosphatidylinositol-3,4,5-trisphosphate 3-phosphatase	5	1	5.33E-02
GO:0016662	oxidoreductase activity, acting on other nitrogenous cor	5	1	5.33E-02
GO:0016744	transketolase or transaldolase activity	5	1	5.33E-02
GO:0019238	cyclohydrolase activity	5	1	5.33E-02
GO:0019863	IgE binding	5	1	5.33E-02
GO:0030298	receptor signaling protein tyrosine kinase activator activ	5	1	5.33E-02
GO:0031726	CCR1 chemokine receptor binding	5	1	5.33E-02
GO:0032552	deoxyribonucleotide binding	5	1	5.33E-02
GO:0034186	apolipoprotein A-I binding	5	1	5.33E-02
GO:0050421	nitrite reductase (NO-forming) activity	5	1	5.33E-02
GO:0051575	5'-deoxyribose-5-phosphate lyase activity	5	1	5.33E-02
GO:0070891	lipoteichoic acid binding	5	1	5.33E-02
GO:0071987	WD40-repeat domain binding	5	1	5.33E-02
GO:0098639	collagen binding involved in cell-matrix adhesion	5	1	5.33E-02
GO:0098973	structural constituent of postsynaptic actin cytoskeletor	5	1	5.33E-02
GO:1901480	oleate transmembrane transporter activity	5	1	5.33E-02
GO:0034767	positive regulation of ion transmembrane transport	186	5	5.34E-02
GO:0043537	negative regulation of blood vessel endothelial cell migr	78	3	5.36E-02
GO:0070972	protein localization to endoplasmic reticulum	78	3	5.36E-02
GO:0072665	protein localization to vacuole	78	3	5.36E-02
GO:1903533	regulation of protein targeting	78	3	5.36E-02
GO:0042254	ribosome biogenesis	310	7	5.40E-02
GO:0033002	muscle cell proliferation	247	6	5.41E-02

GO:0002700	regulation of production of molecular mediator of immu	187	5	5.44E-02
GO:0007193	adenylate cyclase-inhibiting G protein-coupled receptor	79	3	5.53E-02
GO:0072524	pyridine-containing compound metabolic process	79	3	5.53E-02
GO:1903556	negative regulation of tumor necrosis factor superfamily	79	3	5.53E-02
GO:0008186	ATP-dependent activity, acting on RNA	79	3	5.53E-02
GO:0006006	glucose metabolic process	188	5	5.54E-02
GO:0010721	negative regulation of cell development	188	5	5.54E-02
GO:0030308	negative regulation of cell growth	188	5	5.54E-02
GO:0002429	immune response-activating cell surface receptor signali	312	7	5.56E-02
GO:0002757	immune response-activating signal transduction	312	7	5.56E-02
GO:0014808	release of sequestered calcium ion into cytosol by sarco	35	2	5.57E-02
GO:0032205	negative regulation of telomere maintenance	35	2	5.57E-02
GO:0048730	epidermis morphogenesis	35	2	5.57E-02
GO:0060218	hematopoietic stem cell differentiation	35	2	5.57E-02
GO:0090075	relaxation of muscle	35	2	5.57E-02
GO:1901099	negative regulation of signal transduction in absence of	35	2	5.57E-02
GO:1905898	positive regulation of response to endoplasmic reticulur	35	2	5.57E-02
GO:2001240	negative regulation of extrinsic apoptotic signaling pathv	35	2	5.57E-02
GO:0007565	female pregnancy	189	5	5.64E-02
GO:0051896	regulation of protein kinase B signaling	189	5	5.64E-02
GO:0010975	regulation of neuron projection development	446	9	5.64E-02
GO:0019838	growth factor binding	132	4	5.67E-02
GO:1903201	regulation of oxidative stress-induced cell death	80	3	5.70E-02
GO:0033116	endoplasmic reticulum-Golgi intermediate compartmen	80	3	5.70E-02
GO:0050777	negative regulation of immune response	190	5	5.75E-02
GO:0070372	regulation of ERK1 and ERK2 cascade	315	7	5.79E-02
GO:0002244	hematopoietic progenitor cell differentiation	133	4	5.79E-02
GO:0007052	mitotic spindle organization	133	4	5.79E-02
GO:0005793	endoplasmic reticulum-Golgi intermediate compartmen	133	4	5.79E-02
GO:0044265	cellular macromolecule catabolic process	1029	17	5.85E-02
GO:0008217	regulation of blood pressure	191	5	5.85E-02
GO:0010922	positive regulation of phosphatase activity	36	2	5.85E-02
GO:0019934	cGMP-mediated signaling	36	2	5.85E-02
GO:0030224	monocyte differentiation	36	2	5.85E-02
GO:0032232	negative regulation of actin filament bundle assembly	36	2	5.85E-02
GO:0034260	negative regulation of GTPase activity	36	2	5.85E-02
GO:0043243	positive regulation of protein-containing complex disass	36	2	5.85E-02
GO:0045777	positive regulation of blood pressure	36	2	5.85E-02
GO:0048261	negative regulation of receptor-mediated endocytosis	36	2	5.85E-02
GO:0086005	ventricular cardiac muscle cell action potential	36	2	5.85E-02
GO:0098810	neurotransmitter reuptake	36	2	5.85E-02
GO:1903514	release of sequestered calcium ion into cytosol by endo	36	2	5.85E-02
GO:1903749	positive regulation of establishment of protein localizati	36	2	5.85E-02
GO:0000993	RNA polymerase II complex binding	36	2	5.85E-02
GO:0004623	phospholipase A2 activity	36	2	5.85E-02
GO:0043394	proteoglycan binding	36	2	5.85E-02
GO:0000380	alternative mRNA splicing, via spliceosome	81	3	5.88E-02

GO:0120193	tight junction organization	81	3	5.88E-02
GO:0016236	macroautophagy	317	7	5.95E-02
GO:1902105	regulation of leukocyte differentiation	317	7	5.95E-02
GO:0099504	synaptic vesicle cycle	192	5	5.96E-02
GO:0006446	regulation of translational initiation	82	3	6.05E-02
GO:0051279	regulation of release of sequestered calcium ion into cyt	82	3	6.05E-02
GO:0060395	SMAD protein signal transduction	82	3	6.05E-02
GO:0015918	sterol transport	135	4	6.05E-02
GO:0050731	positive regulation of peptidyl-tyrosine phosphorylation	193	5	6.06E-02
GO:0051385	response to mineralocorticoid	37	2	6.14E-02
GO:0071276	cellular response to cadmium ion	37	2	6.14E-02
GO:0090151	establishment of protein localization to mitochondrial m	37	2	6.14E-02
GO:2000279	negative regulation of DNA biosynthetic process	37	2	6.14E-02
GO:0030140	trans-Golgi network transport vesicle	37	2	6.14E-02
GO:0000146	microfilament motor activity	37	2	6.14E-02
GO:0035198	miRNA binding	37	2	6.14E-02
GO:0042605	peptide antigen binding	37	2	6.14E-02
GO:0032990	cell part morphogenesis	666	12	6.18E-02
GO:0019902	phosphatase binding	195	5	6.28E-02
GO:0009892	negative regulation of metabolic process	3554	48	6.31E-02
GO:0007049	cell cycle	1823	27	6.35E-02
GO:0000389	mRNA 3'-splice site recognition	6	1	6.37E-02
GO:0002842	positive regulation of T cell mediated immune response	6	1	6.37E-02
GO:0002860	positive regulation of natural killer cell mediated cytoto	6	1	6.37E-02
GO:0003100	regulation of systemic arterial blood pressure by endoth	6	1	6.37E-02
GO:0006189	'de novo' IMP biosynthetic process	6	1	6.37E-02
GO:0006880	intracellular sequestering of iron ion	6	1	6.37E-02
GO:0007195	adenylate cyclase-inhibiting dopamine receptor signaling	6	1	6.37E-02
GO:0009052	pentose-phosphate shunt, non-oxidative branch	6	1	6.37E-02
GO:0010430	fatty acid omega-oxidation	6	1	6.37E-02
GO:0010746	regulation of long-chain fatty acid import across plasma	6	1	6.37E-02
GO:0010754	negative regulation of cGMP-mediated signaling	6	1	6.37E-02
GO:0019682	glyceraldehyde-3-phosphate metabolic process	6	1	6.37E-02
GO:0030913	paranodal junction assembly	6	1	6.37E-02
GO:0031584	activation of phospholipase D activity	6	1	6.37E-02
GO:0032241	positive regulation of nucleobase-containing compound	6	1	6.37E-02
GO:0032534	regulation of microvillus assembly	6	1	6.37E-02
GO:0032799	low-density lipoprotein receptor particle metabolic proc	6	1	6.37E-02
GO:0032802	low-density lipoprotein particle receptor catabolic proce	6	1	6.37E-02
GO:0032803	regulation of low-density lipoprotein particle receptor c	6	1	6.37E-02
GO:0033601	positive regulation of mammary gland epithelial cell pro	6	1	6.37E-02
GO:0033634	positive regulation of cell-cell adhesion mediated by inte	6	1	6.37E-02
GO:0034165	positive regulation of toll-like receptor 9 signaling pathw	6	1	6.37E-02
GO:0035634	response to stilbenoid	6	1	6.37E-02
GO:0045112	integrin biosynthetic process	6	1	6.37E-02
GO:0046951	ketone body biosynthetic process	6	1	6.37E-02
GO:0048625	myoblast fate commitment	6	1	6.37E-02

GO:0051582	positive regulation of neurotransmitter uptake	6	1	6.37E-02
GO:0051935	glutamate reuptake	6	1	6.37E-02
GO:0055059	asymmetric neuroblast division	6	1	6.37E-02
GO:0055118	negative regulation of cardiac muscle contraction	6	1	6.37E-02
GO:0070426	positive regulation of nucleotide-binding oligomerizatio	6	1	6.37E-02
GO:0071224	cellular response to peptidoglycan	6	1	6.37E-02
GO:0071349	cellular response to interleukin-12	6	1	6.37E-02
GO:0071638	negative regulation of monocyte chemotactic protein-1	6	1	6.37E-02
GO:0071680	response to indole-3-methanol	6	1	6.37E-02
GO:0071878	negative regulation of adenylate cyclase-activating adre	6	1	6.37E-02
GO:0097411	hypoxia-inducible factor-1alpha signaling pathway	6	1	6.37E-02
GO:0150003	regulation of spontaneous synaptic transmission	6	1	6.37E-02
GO:1901552	positive regulation of endothelial cell development	6	1	6.37E-02
GO:1901842	negative regulation of high voltage-gated calcium chann	6	1	6.37E-02
GO:1903142	positive regulation of establishment of endothelial barri	6	1	6.37E-02
GO:1903297	regulation of hypoxia-induced intrinsic apoptotic signali	6	1	6.37E-02
GO:1903298	negative regulation of hypoxia-induced intrinsic apoptot	6	1	6.37E-02
GO:1903613	regulation of protein tyrosine phosphatase activity	6	1	6.37E-02
GO:1904352	positive regulation of protein catabolic process in the va	6	1	6.37E-02
GO:1904751	positive regulation of protein localization to nucleolus	6	1	6.37E-02
GO:1905323	telomerase holoenzyme complex assembly	6	1	6.37E-02
GO:1905600	regulation of receptor-mediated endocytosis involved in	6	1	6.37E-02
GO:1990379	lipid transport across blood-brain barrier	6	1	6.37E-02
GO:2000301	negative regulation of synaptic vesicle exocytosis	6	1	6.37E-02
GO:0000275	mitochondrial proton-transporting ATP synthase comple	6	1	6.37E-02
GO:0031088	platelet dense granule membrane	6	1	6.37E-02
GO:0035748	myelin sheath abaxonal region	6	1	6.37E-02
GO:0035749	myelin sheath adaxonal region	6	1	6.37E-02
GO:0060473	cortical granule	6	1	6.37E-02
GO:0071204	histone pre-mRNA 3'end processing complex	6	1	6.37E-02
GO:0071439	clathrin complex	6	1	6.37E-02
GO:0097165	nuclear stress granule	6	1	6.37E-02
GO:0098835	presynaptic endocytic zone membrane	6	1	6.37E-02
GO:0016972	thiol oxidase activity	6	1	6.37E-02
GO:0019834	phospholipase A2 inhibitor activity	6	1	6.37E-02
GO:0030911	TPR domain binding	6	1	6.37E-02
GO:0034190	apolipoprotein receptor binding	6	1	6.37E-02
GO:0052659	inositol-1,3,4,5-tetrakisphosphate 5-phosphatase activit	6	1	6.37E-02
GO:0070061	fructose binding	6	1	6.37E-02
GO:0070180	large ribosomal subunit rRNA binding	6	1	6.37E-02
GO:0071532	ankyrin repeat binding	6	1	6.37E-02
GO:0098809	nitrite reductase activity	6	1	6.37E-02
GO:1905538	polysome binding	6	1	6.37E-02
GO:0045664	regulation of neuron differentiation	196	5	6.39E-02
GO:0003007	heart morphogenesis	258	6	6.40E-02
GO:0010921	regulation of phosphatase activity	84	3	6.41E-02
GO:0008135	translation factor activity, RNA binding	84	3	6.41E-02

GO:0009889	regulation of biosynthetic process	4239	56	6.43E-02
GO:0002369	T cell cytokine production	38	2	6.44E-02
GO:0002724	regulation of T cell cytokine production	38	2	6.44E-02
GO:0010742	macrophage derived foam cell differentiation	38	2	6.44E-02
GO:0014037	Schwann cell differentiation	38	2	6.44E-02
GO:0060306	regulation of membrane repolarization	38	2	6.44E-02
GO:0098926	postsynaptic signal transduction	38	2	6.44E-02
GO:0016614	oxidoreductase activity, acting on CH-OH group of dono	138	4	6.46E-02
GO:0031301	integral component of organelle membrane	390	8	6.47E-02
GO:2001257	regulation of cation channel activity	197	5	6.50E-02
GO:0050852	T cell receptor signaling pathway	139	4	6.59E-02
GO:0000281	mitotic cytokinesis	85	3	6.60E-02
GO:0034103	regulation of tissue remodeling	85	3	6.60E-02
GO:0034976	response to endoplasmic reticulum stress	261	6	6.69E-02
GO:0045927	positive regulation of growth	261	6	6.69E-02
GO:0048813	dendrite morphogenesis	140	4	6.73E-02
GO:0099106	ion channel regulator activity	140	4	6.73E-02
GO:0003016	respiratory system process	39	2	6.74E-02
GO:0032373	positive regulation of sterol transport	39	2	6.74E-02
GO:0032376	positive regulation of cholesterol transport	39	2	6.74E-02
GO:0032570	response to progesterone	39	2	6.74E-02
GO:0090077	foam cell differentiation	39	2	6.74E-02
GO:0097242	amyloid-beta clearance	39	2	6.74E-02
GO:0016620	oxidoreductase activity, acting on the aldehyde or oxo g	39	2	6.74E-02
GO:0006970	response to osmotic stress	86	3	6.78E-02
GO:0032434	regulation of proteasomal ubiquitin-dependent protein	141	4	6.87E-02
GO:0010556	regulation of macromolecule biosynthetic process	4002	53	6.90E-02
GO:0007507	heart development	607	11	6.96E-02
GO:0030433	ubiquitin-dependent ERAD pathway	87	3	6.97E-02
GO:0045069	regulation of viral genome replication	87	3	6.97E-02
GO:0048145	regulation of fibroblast proliferation	87	3	6.97E-02
GO:1903900	regulation of viral life cycle	142	4	7.01E-02
GO:0005777	peroxisome	142	4	7.01E-02
GO:0042579	microbody	142	4	7.01E-02
GO:0001953	negative regulation of cell-matrix adhesion	40	2	7.05E-02
GO:0006882	cellular zinc ion homeostasis	40	2	7.05E-02
GO:0030866	cortical actin cytoskeleton organization	40	2	7.05E-02
GO:0043001	Golgi to plasma membrane protein transport	40	2	7.05E-02
GO:0048246	macrophage chemotaxis	40	2	7.05E-02
GO:0099622	cardiac muscle cell membrane repolarization	40	2	7.05E-02
GO:1904994	regulation of leukocyte adhesion to vascular endothelial	40	2	7.05E-02
GO:1990000	amyloid fibril formation	40	2	7.05E-02
GO:0031492	nucleosomal DNA binding	40	2	7.05E-02
GO:0046165	alcohol biosynthetic process	143	4	7.16E-02
GO:0035023	regulation of Rho protein signal transduction	88	3	7.16E-02
GO:0050730	regulation of peptidyl-tyrosine phosphorylation	266	6	7.18E-02
GO:1901565	organonitrogen compound catabolic process	1371	21	7.27E-02

GO:0019955	cytokine binding	144	4	7.30E-02
GO:0000302	response to reactive oxygen species	204	5	7.31E-02
GO:0001960	negative regulation of cytokine-mediated signaling path	89	3	7.35E-02
GO:0046849	bone remodeling	89	3	7.35E-02
GO:0005798	Golgi-associated vesicle	89	3	7.35E-02
GO:0045171	intercellular bridge	89	3	7.35E-02
GO:0014912	negative regulation of smooth muscle cell migration	41	2	7.36E-02
GO:0017158	regulation of calcium ion-dependent exocytosis	41	2	7.36E-02
GO:0046688	response to copper ion	41	2	7.36E-02
GO:0050850	positive regulation of calcium-mediated signaling	41	2	7.36E-02
GO:0051281	positive regulation of release of sequestered calcium ior	41	2	7.36E-02
GO:1902562	H4 histone acetyltransferase complex	41	2	7.36E-02
GO:0050997	quaternary ammonium group binding	41	2	7.36E-02
GO:0140513	nuclear protein-containing complex	1295	20	7.37E-02
GO:0000055	ribosomal large subunit export from nucleus	7	1	7.39E-02
GO:0002428	antigen processing and presentation of peptide antigen	7	1	7.39E-02
GO:0002752	cell surface pattern recognition receptor signaling pathw	7	1	7.39E-02
GO:0002857	positive regulation of natural killer cell mediated immun	7	1	7.39E-02
GO:0003062	regulation of heart rate by chemical signal	7	1	7.39E-02
GO:0007182	common-partner SMAD protein phosphorylation	7	1	7.39E-02
GO:0010536	positive regulation of activation of Janus kinase activity	7	1	7.39E-02
GO:0010668	ectodermal cell differentiation	7	1	7.39E-02
GO:0010815	bradykinin catabolic process	7	1	7.39E-02
GO:0021861	forebrain radial glial cell differentiation	7	1	7.39E-02
GO:0030046	parallel actin filament bundle assembly	7	1	7.39E-02
GO:0031055	chromatin remodeling at centromere	7	1	7.39E-02
GO:0032741	positive regulation of interleukin-18 production	7	1	7.39E-02
GO:0032914	positive regulation of transforming growth factor beta1	7	1	7.39E-02
GO:0033299	secretion of lysosomal enzymes	7	1	7.39E-02
GO:0033864	positive regulation of NAD(P)H oxidase activity	7	1	7.39E-02
GO:0034080	CENP-A containing chromatin assembly	7	1	7.39E-02
GO:0034141	positive regulation of toll-like receptor 3 signaling pathw	7	1	7.39E-02
GO:0036089	cleavage furrow formation	7	1	7.39E-02
GO:0043654	recognition of apoptotic cell	7	1	7.39E-02
GO:0044387	negative regulation of protein kinase activity by regulati	7	1	7.39E-02
GO:0044854	plasma membrane raft assembly	7	1	7.39E-02
GO:0045054	constitutive secretory pathway	7	1	7.39E-02
GO:0045110	intermediate filament bundle assembly	7	1	7.39E-02
GO:0048549	positive regulation of pinocytosis	7	1	7.39E-02
GO:0050904	diapedesis	7	1	7.39E-02
GO:0060264	regulation of respiratory burst involved in inflammatory	7	1	7.39E-02
GO:0060467	negative regulation of fertilization	7	1	7.39E-02
GO:0060468	prevention of polyspermy	7	1	7.39E-02
GO:0070100	negative regulation of chemokine-mediated signaling pa	7	1	7.39E-02
GO:0070671	response to interleukin-12	7	1	7.39E-02
GO:0071316	cellular response to nicotine	7	1	7.39E-02
GO:0071475	cellular hyperosmotic salinity response	7	1	7.39E-02

GO:0071895	odontoblast differentiation	7	1	7.39E-02
GO:0072126	positive regulation of glomerular mesangial cell prolifera	7	1	7.39E-02
GO:0097292	XMP metabolic process	7	1	7.39E-02
GO:0097293	XMP biosynthetic process	7	1	7.39E-02
GO:0097294	'de novo' XMP biosynthetic process	7	1	7.39E-02
GO:0097577	sequestering of iron ion	7	1	7.39E-02
GO:0098914	membrane repolarization during atrial cardiac muscle ce	7	1	7.39E-02
GO:0140507	granzyme-mediated programmed cell death signaling pa	7	1	7.39E-02
GO:0150093	amyloid-beta clearance by transcytosis	7	1	7.39E-02
GO:1900157	regulation of bone mineralization involved in bone matu	7	1	7.39E-02
GO:1901979	regulation of inward rectifier potassium channel activity	7	1	7.39E-02
GO:1902177	positive regulation of oxidative stress-induced intrinsic a	7	1	7.39E-02
GO:1903608	protein localization to cytoplasmic stress granule	7	1	7.39E-02
GO:1904429	regulation of t-circle formation	7	1	7.39E-02
GO:1905031	regulation of membrane repolarization during cardiac m	7	1	7.39E-02
GO:1905598	negative regulation of low-density lipoprotein receptor a	7	1	7.39E-02
GO:2000192	negative regulation of fatty acid transport	7	1	7.39E-02
GO:2000391	positive regulation of neutrophil extravasation	7	1	7.39E-02
GO:2000535	regulation of entry of bacterium into host cell	7	1	7.39E-02
GO:2000794	regulation of epithelial cell proliferation involved in lung	7	1	7.39E-02
GO:2001027	negative regulation of endothelial cell chemotaxis	7	1	7.39E-02
GO:0001652	granular component	7	1	7.39E-02
GO:0045261	proton-transporting ATP synthase complex, catalytic cor	7	1	7.39E-02
GO:0098684	photoreceptor ribbon synapse	7	1	7.39E-02
GO:0098690	glycinergic synapse	7	1	7.39E-02
GO:0098833	presynaptic endocytic zone	7	1	7.39E-02
GO:0004024	alcohol dehydrogenase activity, zinc-dependent	7	1	7.39E-02
GO:0004128	cytochrome-b5 reductase activity, acting on NAD(P)H	7	1	7.39E-02
GO:0004445	inositol-polyphosphate 5-phosphatase activity	7	1	7.39E-02
GO:0016929	deSUMOylase activity	7	1	7.39E-02
GO:0019103	pyrimidine nucleotide binding	7	1	7.39E-02
GO:0019763	immunoglobulin receptor activity	7	1	7.39E-02
GO:0019828	aspartic-type endopeptidase inhibitor activity	7	1	7.39E-02
GO:0030274	LIM domain binding	7	1	7.39E-02
GO:0030346	protein phosphatase 2B binding	7	1	7.39E-02
GO:0031730	CCR5 chemokine receptor binding	7	1	7.39E-02
GO:0034485	phosphatidylinositol-3,4,5-trisphosphate 5-phosphatase	7	1	7.39E-02
GO:0042731	PH domain binding	7	1	7.39E-02
GO:0052658	inositol-1,4,5-trisphosphate 5-phosphatase activity	7	1	7.39E-02
GO:0098634	cell-matrix adhesion mediator activity	7	1	7.39E-02
GO:0007005	mitochondrion organization	542	10	7.40E-02
GO:1903305	regulation of regulated secretory pathway	145	4	7.45E-02
GO:0016247	channel regulator activity	145	4	7.45E-02
GO:0032868	response to insulin	269	6	7.49E-02
GO:0071214	cellular response to abiotic stimulus	335	7	7.50E-02
GO:0104004	cellular response to environmental stimulus	335	7	7.50E-02
GO:0006405	RNA export from nucleus	90	3	7.55E-02

GO:0009059	macromolecule biosynthetic process	4806	62	7.65E-02
GO:0002714	positive regulation of B cell mediated immunity	42	2	7.67E-02
GO:0002891	positive regulation of immunoglobulin mediated immun	42	2	7.67E-02
GO:0006623	protein targeting to vacuole	42	2	7.67E-02
GO:0032735	positive regulation of interleukin-12 production	42	2	7.67E-02
GO:0034142	toll-like receptor 4 signaling pathway	42	2	7.67E-02
GO:0045022	early endosome to late endosome transport	42	2	7.67E-02
GO:0045429	positive regulation of nitric oxide biosynthetic process	42	2	7.67E-02
GO:0050691	regulation of defense response to virus by host	42	2	7.67E-02
GO:0050892	intestinal absorption	42	2	7.67E-02
GO:0055069	zinc ion homeostasis	42	2	7.67E-02
GO:0070296	sarcoplasmic reticulum calcium ion transport	42	2	7.67E-02
GO:0071354	cellular response to interleukin-6	42	2	7.67E-02
GO:0090051	negative regulation of cell migration involved in sproutir	42	2	7.67E-02
GO:2000249	regulation of actin cytoskeleton reorganization	42	2	7.67E-02
GO:0008307	structural constituent of muscle	42	2	7.67E-02
GO:0032869	cellular response to insulin stimulus	208	5	7.80E-02
GO:0031348	negative regulation of defense response	272	6	7.81E-02
GO:0032989	cellular component morphogenesis	768	13	7.84E-02
GO:0044703	multi-organism reproductive process	209	5	7.92E-02
GO:0050807	regulation of synapse organization	209	5	7.92E-02
GO:0099111	microtubule-based transport	209	5	7.92E-02
GO:0070371	ERK1 and ERK2 cascade	340	7	7.96E-02
GO:0006356	regulation of transcription by RNA polymerase I	43	2	7.99E-02
GO:0006739	NADP metabolic process	43	2	7.99E-02
GO:0031952	regulation of protein autophosphorylation	43	2	7.99E-02
GO:0060236	regulation of mitotic spindle organization	43	2	7.99E-02
GO:0071364	cellular response to epidermal growth factor stimulus	43	2	7.99E-02
GO:0015144	carbohydrate transmembrane transporter activity	43	2	7.99E-02
GO:0043175	RNA polymerase core enzyme binding	43	2	7.99E-02
GO:0048156	tau protein binding	43	2	7.99E-02
GO:0042692	muscle cell differentiation	409	8	8.01E-02
GO:0045619	regulation of lymphocyte differentiation	210	5	8.04E-02
GO:0002768	immune response-regulating cell surface receptor signal	341	7	8.06E-02
GO:0030246	carbohydrate binding	275	6	8.13E-02
GO:0007029	endoplasmic reticulum organization	93	3	8.15E-02
GO:0022406	membrane docking	93	3	8.15E-02
GO:0032508	DNA duplex unwinding	93	3	8.15E-02
GO:1904427	positive regulation of calcium ion transmembrane trans	93	3	8.15E-02
GO:0099572	postsynaptic specialization	342	7	8.16E-02
GO:0034614	cellular response to reactive oxygen species	150	4	8.20E-02
GO:0043535	regulation of blood vessel endothelial cell migration	150	4	8.20E-02
GO:0036126	sperm flagellum	150	4	8.20E-02
GO:1902532	negative regulation of intracellular signal transduction	554	10	8.28E-02
GO:0006836	neurotransmitter transport	212	5	8.30E-02
GO:0006509	membrane protein ectodomain proteolysis	44	2	8.31E-02
GO:0014075	response to amine	44	2	8.31E-02

GO:0014911	positive regulation of smooth muscle cell migration	44	2	8.31E-02
GO:0050856	regulation of T cell receptor signaling pathway	44	2	8.31E-02
GO:1903214	regulation of protein targeting to mitochondrion	44	2	8.31E-02
GO:1904407	positive regulation of nitric oxide metabolic process	44	2	8.31E-02
GO:0010876	lipid localization	483	9	8.33E-02
GO:0002042	cell migration involved in sprouting angiogenesis	94	3	8.35E-02
GO:0002793	positive regulation of peptide secretion	94	3	8.35E-02
GO:0032436	positive regulation of proteasomal ubiquitin-dependent	94	3	8.35E-02
GO:0060761	negative regulation of response to cytokine stimulus	94	3	8.35E-02
GO:0071456	cellular response to hypoxia	151	4	8.36E-02
GO:0002232	leukocyte chemotaxis involved in inflammatory respons	8	1	8.40E-02
GO:0002249	lymphocyte anergy	8	1	8.40E-02
GO:0002667	regulation of T cell anergy	8	1	8.40E-02
GO:0002729	positive regulation of natural killer cell cytokine product	8	1	8.40E-02
GO:0002858	regulation of natural killer cell mediated cytotoxicity dir	8	1	8.40E-02
GO:0002870	T cell anergy	8	1	8.40E-02
GO:0002911	regulation of lymphocyte anergy	8	1	8.40E-02
GO:0003223	ventricular compact myocardium morphogenesis	8	1	8.40E-02
GO:0003360	brainstem development	8	1	8.40E-02
GO:0007021	tubulin complex assembly	8	1	8.40E-02
GO:0008354	germ cell migration	8	1	8.40E-02
GO:0010637	negative regulation of mitochondrial fusion	8	1	8.40E-02
GO:0010757	negative regulation of plasminogen activation	8	1	8.40E-02
GO:0010886	positive regulation of cholesterol storage	8	1	8.40E-02
GO:0016479	negative regulation of transcription by RNA polymerase	8	1	8.40E-02
GO:0017038	protein import	8	1	8.40E-02
GO:0021932	hindbrain radial glia guided cell migration	8	1	8.40E-02
GO:0030853	negative regulation of granulocyte differentiation	8	1	8.40E-02
GO:0032071	regulation of endodeoxyribonuclease activity	8	1	8.40E-02
GO:0032225	regulation of synaptic transmission, dopaminergic	8	1	8.40E-02
GO:0032471	negative regulation of endoplasmic reticulum calcium io	8	1	8.40E-02
GO:0034196	acylglycerol transport	8	1	8.40E-02
GO:0034197	triglyceride transport	8	1	8.40E-02
GO:0034756	regulation of iron ion transport	8	1	8.40E-02
GO:0035726	common myeloid progenitor cell proliferation	8	1	8.40E-02
GO:0035740	CD8-positive, alpha-beta T cell proliferation	8	1	8.40E-02
GO:0044208	'de novo' AMP biosynthetic process	8	1	8.40E-02
GO:0045541	negative regulation of cholesterol biosynthetic process	8	1	8.40E-02
GO:0045647	negative regulation of erythrocyte differentiation	8	1	8.40E-02
GO:0045656	negative regulation of monocyte differentiation	8	1	8.40E-02
GO:0045988	negative regulation of striated muscle contraction	8	1	8.40E-02
GO:0046654	tetrahydrofolate biosynthetic process	8	1	8.40E-02
GO:0051620	norepinephrine uptake	8	1	8.40E-02
GO:0051838	cytolysis by host of symbiont cells	8	1	8.40E-02
GO:0060087	relaxation of vascular associated smooth muscle	8	1	8.40E-02
GO:0060316	positive regulation of ryanodine-sensitive calcium-releas	8	1	8.40E-02
GO:0060372	regulation of atrial cardiac muscle cell membrane repola	8	1	8.40E-02

GO:0060753	regulation of mast cell chemotaxis	8	1	8.40E-02
GO:0070432	regulation of nucleotide-binding oligomerization domair	8	1	8.40E-02
GO:0070662	mast cell proliferation	8	1	8.40E-02
GO:0070666	regulation of mast cell proliferation	8	1	8.40E-02
GO:0070973	protein localization to endoplasmic reticulum exit site	8	1	8.40E-02
GO:0071447	cellular response to hydroperoxide	8	1	8.40E-02
GO:0072318	clathrin coat disassembly	8	1	8.40E-02
GO:0090063	positive regulation of microtubule nucleation	8	1	8.40E-02
GO:0090091	positive regulation of extracellular matrix disassembly	8	1	8.40E-02
GO:0090118	receptor-mediated endocytosis involved in cholesterol t	8	1	8.40E-02
GO:0090206	negative regulation of cholesterol metabolic process	8	1	8.40E-02
GO:0099640	axo-dendritic protein transport	8	1	8.40E-02
GO:0099641	anterograde axonal protein transport	8	1	8.40E-02
GO:0106119	negative regulation of sterol biosynthetic process	8	1	8.40E-02
GO:0140212	regulation of long-chain fatty acid import into cell	8	1	8.40E-02
GO:1900223	positive regulation of amyloid-beta clearance	8	1	8.40E-02
GO:1901339	regulation of store-operated calcium channel activity	8	1	8.40E-02
GO:1902423	regulation of attachment of mitotic spindle microtubule	8	1	8.40E-02
GO:1902425	positive regulation of attachment of mitotic spindle mic	8	1	8.40E-02
GO:1902732	positive regulation of chondrocyte proliferation	8	1	8.40E-02
GO:1902949	positive regulation of tau-protein kinase activity	8	1	8.40E-02
GO:1902965	regulation of protein localization to early endosome	8	1	8.40E-02
GO:1902966	positive regulation of protein localization to early endos	8	1	8.40E-02
GO:1903361	protein localization to basolateral plasma membrane	8	1	8.40E-02
GO:1903659	regulation of complement-dependent cytotoxicity	8	1	8.40E-02
GO:1904714	regulation of chaperone-mediated autophagy	8	1	8.40E-02
GO:1990314	cellular response to insulin-like growth factor stimulus	8	1	8.40E-02
GO:2000389	regulation of neutrophil extravasation	8	1	8.40E-02
GO:2000501	regulation of natural killer cell chemotaxis	8	1	8.40E-02
GO:2000564	regulation of CD8-positive, alpha-beta T cell proliferatio	8	1	8.40E-02
GO:2000586	regulation of platelet-derived growth factor receptor-be	8	1	8.40E-02
GO:2000643	positive regulation of early endosome to late endosome	8	1	8.40E-02
GO:0005577	fibrinogen complex	8	1	8.40E-02
GO:0005915	zonula adherens	8	1	8.40E-02
GO:0016461	unconventional myosin complex	8	1	8.40E-02
GO:0031232	extrinsic component of external side of plasma membra	8	1	8.40E-02
GO:0061200	clathrin-sculpted gamma-aminobutyric acid transport ve	8	1	8.40E-02
GO:0061202	clathrin-sculpted gamma-aminobutyric acid transport ve	8	1	8.40E-02
GO:0070578	RISC-loading complex	8	1	8.40E-02
GO:0030881	beta-2-microglobulin binding	8	1	8.40E-02
GO:0030976	thiamine pyrophosphate binding	8	1	8.40E-02
GO:0047184	1-acylglycerophosphocholine O-acyltransferase activity	8	1	8.40E-02
GO:0052743	inositol tetrakisphosphate phosphatase activity	8	1	8.40E-02
GO:0061676	importin-alpha family protein binding	8	1	8.40E-02
GO:0070573	metallodipeptidase activity	8	1	8.40E-02
GO:0099122	RNA polymerase II C-terminal domain binding	8	1	8.40E-02
GO:1905394	retromer complex binding	8	1	8.40E-02

GO:0045637	regulation of myeloid cell differentiation	214	5	8.56E-02
GO:0032465	regulation of cytokinesis	95	3	8.56E-02
GO:0042982	amyloid precursor protein metabolic process	95	3	8.56E-02
GO:0045638	negative regulation of myeloid cell differentiation	95	3	8.56E-02
GO:0001825	blastocyst formation	45	2	8.64E-02
GO:0006904	vesicle docking involved in exocytosis	45	2	8.64E-02
GO:0010712	regulation of collagen metabolic process	45	2	8.64E-02
GO:0010799	regulation of peptidyl-threonine phosphorylation	45	2	8.64E-02
GO:0033003	regulation of mast cell activation	45	2	8.64E-02
GO:0046839	phospholipid dephosphorylation	45	2	8.64E-02
GO:0051955	regulation of amino acid transport	45	2	8.64E-02
GO:0070741	response to interleukin-6	45	2	8.64E-02
GO:0098927	vesicle-mediated transport between endosomal compar	45	2	8.64E-02
GO:1904646	cellular response to amyloid-beta	45	2	8.64E-02
GO:0005246	calcium channel regulator activity	45	2	8.64E-02
GO:0030674	protein-macromolecule adaptor activity	347	7	8.65E-02
GO:0050803	regulation of synapse structure or activity	215	5	8.69E-02
GO:0002275	myeloid cell activation involved in immune response	96	3	8.76E-02
GO:0032091	negative regulation of protein binding	96	3	8.76E-02
GO:0070664	negative regulation of leukocyte proliferation	96	3	8.76E-02
GO:0061564	axon development	490	9	8.91E-02
GO:0021795	cerebral cortex cell migration	46	2	8.97E-02
GO:0032467	positive regulation of cytokinesis	46	2	8.97E-02
GO:0097178	ruffle assembly	46	2	8.97E-02
GO:2001239	regulation of extrinsic apoptotic signaling pathway in ab	46	2	8.97E-02
GO:0030544	Hsp70 protein binding	46	2	8.97E-02
GO:0010596	negative regulation of endothelial cell migration	97	3	8.98E-02
GO:0032368	regulation of lipid transport	155	4	8.99E-02
GO:0070507	regulation of microtubule cytoskeleton organization	155	4	8.99E-02
GO:0004386	helicase activity	155	4	8.99E-02
GO:0098984	neuron to neuron synapse	351	7	9.05E-02
GO:0044706	multi-multicellular organism process	218	5	9.08E-02
GO:0043434	response to peptide hormone	421	8	9.09E-02
GO:0030234	enzyme regulator activity	1253	19	9.16E-02
GO:0060537	muscle tissue development	422	8	9.19E-02
GO:1990868	response to chemokine	98	3	9.19E-02
GO:1990869	cellular response to chemokine	98	3	9.19E-02
GO:0043202	lysosomal lumen	98	3	9.19E-02
GO:0008238	exopeptidase activity	98	3	9.19E-02
GO:0001505	regulation of neurotransmitter levels	219	5	9.22E-02
GO:0032365	intracellular lipid transport	47	2	9.30E-02
GO:0032369	negative regulation of lipid transport	47	2	9.30E-02
GO:0045601	regulation of endothelial cell differentiation	47	2	9.30E-02
GO:0046677	response to antibiotic	47	2	9.30E-02
GO:0050919	negative chemotaxis	47	2	9.30E-02
GO:0051180	vitamin transport	47	2	9.30E-02
GO:0070849	response to epidermal growth factor	47	2	9.30E-02

GO:0071470	cellular response to osmotic stress	47	2	9.30E-02
GO:0072583	clathrin-dependent endocytosis	47	2	9.30E-02
GO:0090224	regulation of spindle organization	47	2	9.30E-02
GO:0016903	oxidoreductase activity, acting on the aldehyde or oxo g	47	2	9.30E-02
GO:0031326	regulation of cellular biosynthetic process	4175	54	9.35E-02
GO:0002370	natural killer cell cytokine production	9	1	9.40E-02
GO:0002420	natural killer cell mediated cytotoxicity directed against	9	1	9.40E-02
GO:0002576	platelet degranulation	9	1	9.40E-02
GO:0002578	negative regulation of antigen processing and presentat	9	1	9.40E-02
GO:0002727	regulation of natural killer cell cytokine production	9	1	9.40E-02
GO:0002840	regulation of T cell mediated immune response to tumo	9	1	9.40E-02
GO:0002855	regulation of natural killer cell mediated immune respor	9	1	9.40E-02
GO:0006012	galactose metabolic process	9	1	9.40E-02
GO:0006069	ethanol oxidation	9	1	9.40E-02
GO:0006782	protoporphyrinogen IX biosynthetic process	9	1	9.40E-02
GO:0010501	RNA secondary structure unwinding	9	1	9.40E-02
GO:0010533	regulation of activation of Janus kinase activity	9	1	9.40E-02
GO:0014908	myotube differentiation involved in skeletal muscle rege	9	1	9.40E-02
GO:0016926	protein desumoylation	9	1	9.40E-02
GO:0019371	cyclooxygenase pathway	9	1	9.40E-02
GO:0019674	NAD metabolic process	9	1	9.40E-02
GO:0031340	positive regulation of vesicle fusion	9	1	9.40E-02
GO:0032287	peripheral nervous system myelin maintenance	9	1	9.40E-02
GO:0035635	entry of bacterium into host cell	9	1	9.40E-02
GO:0035973	aggrephagy	9	1	9.40E-02
GO:0044417	translocation of molecules into host	9	1	9.40E-02
GO:0045792	negative regulation of cell size	9	1	9.40E-02
GO:0046950	cellular ketone body metabolic process	9	1	9.40E-02
GO:0050774	negative regulation of dendrite morphogenesis	9	1	9.40E-02
GO:0051121	hepoxilin metabolic process	9	1	9.40E-02
GO:0051122	hepoxilin biosynthetic process	9	1	9.40E-02
GO:0051292	nuclear pore complex assembly	9	1	9.40E-02
GO:0060052	neurofilament cytoskeleton organization	9	1	9.40E-02
GO:0060242	contact inhibition	9	1	9.40E-02
GO:0060267	positive regulation of respiratory burst	9	1	9.40E-02
GO:0060392	negative regulation of SMAD protein signal transduction	9	1	9.40E-02
GO:0060700	regulation of ribonuclease activity	9	1	9.40E-02
GO:0070268	cornification	9	1	9.40E-02
GO:0070339	response to bacterial lipopeptide	9	1	9.40E-02
GO:0070508	cholesterol import	9	1	9.40E-02
GO:0070922	RISC complex assembly	9	1	9.40E-02
GO:0071220	cellular response to bacterial lipoprotein	9	1	9.40E-02
GO:0071221	cellular response to bacterial lipopeptide	9	1	9.40E-02
GO:0071225	cellular response to muramyl dipeptide	9	1	9.40E-02
GO:0071281	cellular response to iron ion	9	1	9.40E-02
GO:0072319	vesicle uncoating	9	1	9.40E-02
GO:0086023	adenylate cyclase-activating adrenergic receptor signalir	9	1	9.40E-02

GO:0090324	negative regulation of oxidative phosphorylation	9	1	9.40E-02
GO:0097064	ncRNA export from nucleus	9	1	9.40E-02
GO:0098696	regulation of neurotransmitter receptor localization to p	9	1	9.40E-02
GO:0110095	cellular detoxification of aldehyde	9	1	9.40E-02
GO:1900038	negative regulation of cellular response to hypoxia	9	1	9.40E-02
GO:1901724	positive regulation of cell proliferation involved in kidne	9	1	9.40E-02
GO:1903028	positive regulation of opsonization	9	1	9.40E-02
GO:1903044	protein localization to membrane raft	9	1	9.40E-02
GO:1903960	negative regulation of anion transmembrane transport	9	1	9.40E-02
GO:1904627	response to phorbol 13-acetate 12-myristate	9	1	9.40E-02
GO:1904628	cellular response to phorbol 13-acetate 12-myristate	9	1	9.40E-02
GO:1905244	regulation of modification of synaptic structure	9	1	9.40E-02
GO:1990144	intrinsic apoptotic signaling pathway in response to hyp	9	1	9.40E-02
GO:2001187	positive regulation of CD8-positive, alpha-beta T cell act	9	1	9.40E-02
GO:0005885	Arp2/3 protein complex	9	1	9.40E-02
GO:0008091	spectrin	9	1	9.40E-02
GO:0016600	flotillin complex	9	1	9.40E-02
GO:0031095	platelet dense tubular network membrane	9	1	9.40E-02
GO:0032437	cuticular plate	9	1	9.40E-02
GO:0032593	insulin-responsive compartment	9	1	9.40E-02
GO:0044294	dendritic growth cone	9	1	9.40E-02
GO:0070419	nonhomologous end joining complex	9	1	9.40E-02
GO:0099523	presynaptic cytosol	9	1	9.40E-02
GO:0099571	postsynaptic cytoskeleton	9	1	9.40E-02
GO:0003691	double-stranded telomeric DNA binding	9	1	9.40E-02
GO:0004022	alcohol dehydrogenase (NAD+) activity	9	1	9.40E-02
GO:0005388	P-type calcium transporter activity	9	1	9.40E-02
GO:0008432	JUN kinase binding	9	1	9.40E-02
GO:0043184	vascular endothelial growth factor receptor 2 binding	9	1	9.40E-02
GO:0097200	cysteine-type endopeptidase activity involved in executi	9	1	9.40E-02
GO:0140311	protein sequestering activity	9	1	9.40E-02
GO:1901379	regulation of potassium ion transmembrane transport	99	3	9.40E-02
GO:0019221	cytokine-mediated signaling pathway	496	9	9.43E-02
GO:0098655	cation transmembrane transport	948	15	9.59E-02
GO:0002532	production of molecular mediator involved in inflammat	100	3	9.62E-02
GO:0097306	cellular response to alcohol	100	3	9.62E-02
GO:0006953	acute-phase response	48	2	9.64E-02
GO:0008038	neuron recognition	48	2	9.64E-02
GO:0048538	thymus development	48	2	9.64E-02
GO:1902003	regulation of amyloid-beta formation	48	2	9.64E-02
GO:0015026	coreceptor activity	48	2	9.64E-02
GO:0035051	cardiocyte differentiation	159	4	9.65E-02
GO:0036294	cellular response to decreased oxygen levels	159	4	9.65E-02
GO:0015931	nucleobase-containing compound transport	223	5	9.77E-02
GO:0070374	positive regulation of ERK1 and ERK2 cascade	223	5	9.77E-02
GO:2001243	negative regulation of intrinsic apoptotic signaling pathv	101	3	9.84E-02
GO:0031331	positive regulation of cellular catabolic process	429	8	9.86E-02

GO:0097191	extrinsic apoptotic signaling pathway	224	5	9.90E-02
GO:0010605	negative regulation of macromolecule metabolic proces	3336	44	9.93E-02
GO:0008089	anterograde axonal transport	49	2	9.98E-02
GO:0043330	response to exogenous dsRNA	49	2	9.98E-02
GO:1901385	regulation of voltage-gated calcium channel activity	49	2	9.98E-02
GO:1903426	regulation of reactive oxygen species biosynthetic proce	49	2	9.98E-02
GO:2000378	negative regulation of reactive oxygen species metaboli	49	2	9.98E-02
GO:0008009	chemokine activity	49	2	9.98E-02
GO:0015850	organic hydroxy compound transport	292	6	1.01E-01
GO:0002366	leukocyte activation involved in immune response	293	6	1.02E-01
GO:0003008	system process	2333	32	1.02E-01
GO:0006910	phagocytosis, recognition	103	3	1.03E-01
GO:0032677	regulation of interleukin-8 production	103	3	1.03E-01
GO:0044283	small molecule biosynthetic process	579	10	1.03E-01
GO:0089718	amino acid import across plasma membrane	50	2	1.03E-01
GO:1903747	regulation of establishment of protein localization to mi	50	2	1.03E-01
GO:0050657	nucleic acid transport	163	4	1.03E-01
GO:0050658	RNA transport	163	4	1.03E-01
GO:0002423	natural killer cell mediated immune response to tumor c	10	1	1.04E-01
GO:0002676	regulation of chronic inflammatory response	10	1	1.04E-01
GO:0003056	regulation of vascular associated smooth muscle contra	10	1	1.04E-01
GO:0003253	cardiac neural crest cell migration involved in outflow tr	10	1	1.04E-01
GO:0003266	regulation of secondary heart field cardioblast prolifera	10	1	1.04E-01
GO:0007343	egg activation	10	1	1.04E-01
GO:0009396	folic acid-containing compound biosynthetic process	10	1	1.04E-01
GO:0010998	regulation of translational initiation by eIF2 alpha phosp	10	1	1.04E-01
GO:0030223	neutrophil differentiation	10	1	1.04E-01
GO:0030388	fructose 1,6-bisphosphate metabolic process	10	1	1.04E-01
GO:0032070	regulation of deoxyribonuclease activity	10	1	1.04E-01
GO:0032380	regulation of intracellular sterol transport	10	1	1.04E-01
GO:0032383	regulation of intracellular cholesterol transport	10	1	1.04E-01
GO:0033483	gas homeostasis	10	1	1.04E-01
GO:0033572	transferrin transport	10	1	1.04E-01
GO:0035747	natural killer cell chemotaxis	10	1	1.04E-01
GO:0035878	nail development	10	1	1.04E-01
GO:0048664	neuron fate determination	10	1	1.04E-01
GO:0051342	regulation of cyclic-nucleotide phosphodiesterase activit	10	1	1.04E-01
GO:0051409	response to nitrosative stress	10	1	1.04E-01
GO:0051933	amino acid neurotransmitter reuptake	10	1	1.04E-01
GO:0060245	detection of cell density	10	1	1.04E-01
GO:0060452	positive regulation of cardiac muscle contraction	10	1	1.04E-01
GO:0060502	epithelial cell proliferation involved in lung morphogene	10	1	1.04E-01
GO:0070099	regulation of chemokine-mediated signaling pathway	10	1	1.04E-01
GO:0090557	establishment of endothelial intestinal barrier	10	1	1.04E-01
GO:0098903	regulation of membrane repolarization during action po	10	1	1.04E-01
GO:0098974	postsynaptic actin cytoskeleton organization	10	1	1.04E-01
GO:1901838	positive regulation of transcription of nucleolar large rR	10	1	1.04E-01

GO:1902224	ketone body metabolic process	10	1	1.04E-01
GO:1902946	protein localization to early endosome	10	1	1.04E-01
GO:1904587	response to glycoprotein	10	1	1.04E-01
GO:1904749	regulation of protein localization to nucleolus	10	1	1.04E-01
GO:1905165	regulation of lysosomal protein catabolic process	10	1	1.04E-01
GO:1905383	protein localization to presynapse	10	1	1.04E-01
GO:2000121	regulation of removal of superoxide radicals	10	1	1.04E-01
GO:0001939	female pronucleus	10	1	1.04E-01
GO:0005641	nuclear envelope lumen	10	1	1.04E-01
GO:0043220	Schmidt-Lanterman incisure	10	1	1.04E-01
GO:0070938	contractile ring	10	1	1.04E-01
GO:0140092	bBAF complex	10	1	1.04E-01
GO:0140268	endoplasmic reticulum-plasma membrane contact site	10	1	1.04E-01
GO:0003796	lysozyme activity	10	1	1.04E-01
GO:0004439	phosphatidylinositol-4,5-bisphosphate 5-phosphatase ac	10	1	1.04E-01
GO:0008097	5S rRNA binding	10	1	1.04E-01
GO:0008429	phosphatidylethanolamine binding	10	1	1.04E-01
GO:0008526	phosphatidylinositol transfer activity	10	1	1.04E-01
GO:0015245	fatty acid transmembrane transporter activity	10	1	1.04E-01
GO:0015643	toxic substance binding	10	1	1.04E-01
GO:0016175	superoxide-generating NAD(P)H oxidase activity	10	1	1.04E-01
GO:0016653	oxidoreductase activity, acting on NAD(P)H, heme prote	10	1	1.04E-01
GO:0016742	hydroxymethyl-, formyl- and related transferase activity	10	1	1.04E-01
GO:0016832	aldehyde-lyase activity	10	1	1.04E-01
GO:0018455	alcohol dehydrogenase [NAD(P)+] activity	10	1	1.04E-01
GO:0034594	phosphatidylinositol trisphosphate phosphatase activity	10	1	1.04E-01
GO:0034596	phosphatidylinositol phosphate 4-phosphatase activity	10	1	1.04E-01
GO:0042608	T cell receptor binding	10	1	1.04E-01
GO:0070097	delta-catenin binding	10	1	1.04E-01
GO:0070567	cytidylyltransferase activity	10	1	1.04E-01
GO:0071723	lipopeptide binding	10	1	1.04E-01
GO:0097027	ubiquitin-protein transferase activator activity	10	1	1.04E-01
GO:0098505	G-rich strand telomeric DNA binding	10	1	1.04E-01
GO:0051100	negative regulation of binding	164	4	1.05E-01
GO:0000041	transition metal ion transport	104	3	1.05E-01
GO:0032637	interleukin-8 production	104	3	1.05E-01
GO:0042100	B cell proliferation	104	3	1.05E-01
GO:0048588	developmental cell growth	229	5	1.06E-01
GO:0019083	viral transcription	51	2	1.07E-01
GO:0032873	negative regulation of stress-activated MAPK cascade	51	2	1.07E-01
GO:0043124	negative regulation of I-kappaB kinase/NF-kappaB signal	51	2	1.07E-01
GO:0044088	regulation of vacuole organization	51	2	1.07E-01
GO:0045058	T cell selection	51	2	1.07E-01
GO:0051489	regulation of filopodium assembly	51	2	1.07E-01
GO:0070303	negative regulation of stress-activated protein kinase sig	51	2	1.07E-01
GO:0015485	cholesterol binding	51	2	1.07E-01
GO:0016860	intramolecular oxidoreductase activity	51	2	1.07E-01

GO:0006606	protein import into nucleus	165	4	1.07E-01
GO:0007030	Golgi organization	165	4	1.07E-01
GO:0097729	9+2 motile cilium	165	4	1.07E-01
GO:0007409	axonogenesis	438	8	1.08E-01
GO:0010594	regulation of endothelial cell migration	230	5	1.08E-01
GO:0050920	regulation of chemotaxis	230	5	1.08E-01
GO:0048193	Golgi vesicle transport	298	6	1.08E-01
GO:0051236	establishment of RNA localization	166	4	1.08E-01
GO:0030336	negative regulation of cell migration	368	7	1.09E-01
GO:0006754	ATP biosynthetic process	106	3	1.10E-01
GO:0042102	positive regulation of T cell proliferation	106	3	1.10E-01
GO:0002762	negative regulation of myeloid leukocyte differentiation	52	2	1.10E-01
GO:0006636	unsaturated fatty acid biosynthetic process	52	2	1.10E-01
GO:0021872	forebrain generation of neurons	52	2	1.10E-01
GO:0086009	membrane repolarization	52	2	1.10E-01
GO:1902930	regulation of alcohol biosynthetic process	52	2	1.10E-01
GO:0001959	regulation of cytokine-mediated signaling pathway	167	4	1.10E-01
GO:0070665	positive regulation of leukocyte proliferation	167	4	1.10E-01
GO:0019953	sexual reproduction	896	14	1.13E-01
GO:0015711	organic anion transport	372	7	1.14E-01
GO:0002604	regulation of dendritic cell antigen processing and prese	11	1	1.14E-01
GO:0006188	IMP biosynthetic process	11	1	1.14E-01
GO:0006449	regulation of translational termination	11	1	1.14E-01
GO:0010917	negative regulation of mitochondrial membrane potenti	11	1	1.14E-01
GO:0010968	regulation of microtubule nucleation	11	1	1.14E-01
GO:0010986	positive regulation of lipoprotein particle clearance	11	1	1.14E-01
GO:0032377	regulation of intracellular lipid transport	11	1	1.14E-01
GO:0032429	regulation of phospholipase A2 activity	11	1	1.14E-01
GO:0032493	response to bacterial lipoprotein	11	1	1.14E-01
GO:0033860	regulation of NAD(P)H oxidase activity	11	1	1.14E-01
GO:0034115	negative regulation of heterotypic cell-cell adhesion	11	1	1.14E-01
GO:0034163	regulation of toll-like receptor 9 signaling pathway	11	1	1.14E-01
GO:0034975	protein folding in endoplasmic reticulum	11	1	1.14E-01
GO:0035090	maintenance of apical/basal cell polarity	11	1	1.14E-01
GO:0038003	G protein-coupled opioid receptor signaling pathway	11	1	1.14E-01
GO:0042118	endothelial cell activation	11	1	1.14E-01
GO:0044557	relaxation of smooth muscle	11	1	1.14E-01
GO:0044857	plasma membrane raft organization	11	1	1.14E-01
GO:0045199	maintenance of epithelial cell apical/basal polarity	11	1	1.14E-01
GO:0045793	positive regulation of cell size	11	1	1.14E-01
GO:0045837	negative regulation of membrane potential	11	1	1.14E-01
GO:0046501	protoporphyrinogen IX metabolic process	11	1	1.14E-01
GO:0048755	branching morphogenesis of a nerve	11	1	1.14E-01
GO:0051561	positive regulation of mitochondrial calcium ion concent	11	1	1.14E-01
GO:0051610	serotonin uptake	11	1	1.14E-01
GO:0060020	Bergmann glial cell differentiation	11	1	1.14E-01
GO:0070587	regulation of cell-cell adhesion involved in gastrulation	11	1	1.14E-01

GO:0071394	cellular response to testosterone stimulus	11	1	1.14E-01
GO:0072124	regulation of glomerular mesangial cell proliferation	11	1	1.14E-01
GO:0086100	endothelin receptor signaling pathway	11	1	1.14E-01
GO:0090084	negative regulation of inclusion body assembly	11	1	1.14E-01
GO:0097278	complement-dependent cytotoxicity	11	1	1.14E-01
GO:0099624	atrial cardiac muscle cell membrane repolarization	11	1	1.14E-01
GO:1900095	regulation of dosage compensation by inactivation of X c	11	1	1.14E-01
GO:1901856	negative regulation of cellular respiration	11	1	1.14E-01
GO:1902915	negative regulation of protein polyubiquitination	11	1	1.14E-01
GO:1903027	regulation of opsonization	11	1	1.14E-01
GO:1905668	positive regulation of protein localization to endosome	11	1	1.14E-01
GO:2000049	positive regulation of cell-cell adhesion mediated by cad	11	1	1.14E-01
GO:2000109	regulation of macrophage apoptotic process	11	1	1.14E-01
GO:2000644	regulation of receptor catabolic process	11	1	1.14E-01
GO:2000672	negative regulation of motor neuron apoptotic process	11	1	1.14E-01
GO:2001204	regulation of osteoclast development	11	1	1.14E-01
GO:0017119	Golgi transport complex	11	1	1.14E-01
GO:0031094	platelet dense tubular network	11	1	1.14E-01
GO:0042589	zymogen granule membrane	11	1	1.14E-01
GO:0044545	NSL complex	11	1	1.14E-01
GO:0097470	ribbon synapse	11	1	1.14E-01
GO:0098691	dopaminergic synapse	11	1	1.14E-01
GO:1905370	serine-type endopeptidase complex	11	1	1.14E-01
GO:0000774	adenyl-nucleotide exchange factor activity	11	1	1.14E-01
GO:0002162	dystroglycan binding	11	1	1.14E-01
GO:0016661	oxidoreductase activity, acting on other nitrogenous cor	11	1	1.14E-01
GO:0016857	racemase and epimerase activity, acting on carbohydrat	11	1	1.14E-01
GO:0019864	IgG binding	11	1	1.14E-01
GO:0031996	thioesterase binding	11	1	1.14E-01
GO:0034511	U3 snoRNA binding	11	1	1.14E-01
GO:0035256	G protein-coupled glutamate receptor binding	11	1	1.14E-01
GO:0047429	nucleoside triphosphate diphosphatase activity	11	1	1.14E-01
GO:0097371	MDM2/MDM4 family protein binding	11	1	1.14E-01
GO:0102545	phosphatidyl phospholipase B activity	11	1	1.14E-01
GO:0002204	somatic recombination of immunoglobulin genes involve	53	2	1.14E-01
GO:0002208	somatic diversification of immunoglobulins involved in i	53	2	1.14E-01
GO:0009409	response to cold	53	2	1.14E-01
GO:0030838	positive regulation of actin filament polymerization	53	2	1.14E-01
GO:0034381	plasma lipoprotein particle clearance	53	2	1.14E-01
GO:0045190	isotype switching	53	2	1.14E-01
GO:0048146	positive regulation of fibroblast proliferation	53	2	1.14E-01
GO:0048260	positive regulation of receptor-mediated endocytosis	53	2	1.14E-01
GO:0052372	modulation by symbiont of entry into host	53	2	1.14E-01
GO:0060071	Wnt signaling pathway, planar cell polarity pathway	53	2	1.14E-01
GO:0017069	snRNA binding	53	2	1.14E-01
GO:0019829	ATPase-coupled cation transmembrane transporter acti	53	2	1.14E-01
GO:0051287	NAD binding	53	2	1.14E-01

GO:0140296	general transcription initiation factor binding	53	2	1.14E-01
GO:0051170	import into nucleus	169	4	1.14E-01
GO:0033138	positive regulation of peptidyl-serine phosphorylation	108	3	1.14E-01
GO:1902106	negative regulation of leukocyte differentiation	108	3	1.14E-01
GO:0034451	centriolar satellite	108	3	1.14E-01
GO:0004620	phospholipase activity	108	3	1.14E-01
GO:0090079	translation regulator activity, nucleic acid binding	108	3	1.14E-01
GO:0007156	homophilic cell adhesion via plasma membrane adhesio	170	4	1.16E-01
GO:2000058	regulation of ubiquitin-dependent protein catabolic pro	170	4	1.16E-01
GO:0042116	macrophage activation	109	3	1.17E-01
GO:0009395	phospholipid catabolic process	54	2	1.17E-01
GO:0010883	regulation of lipid storage	54	2	1.17E-01
GO:0030834	regulation of actin filament depolymerization	54	2	1.17E-01
GO:0015459	potassium channel regulator activity	54	2	1.17E-01
GO:0043539	protein serine/threonine kinase activator activity	54	2	1.17E-01
GO:1904064	positive regulation of cation transmembrane transport	171	4	1.17E-01
GO:0016358	dendrite development	237	5	1.18E-01
GO:0034764	positive regulation of transmembrane transport	237	5	1.18E-01
GO:0019219	regulation of nucleobase-containing compound metabol	4076	52	1.19E-01
GO:0071375	cellular response to peptide hormone stimulus	306	6	1.19E-01
GO:2000060	positive regulation of ubiquitin-dependent protein catab	110	3	1.19E-01
GO:1990138	neuron projection extension	172	4	1.19E-01
GO:0003730	mRNA 3'-UTR binding	238	5	1.20E-01
GO:0010518	positive regulation of phospholipase activity	55	2	1.21E-01
GO:0071320	cellular response to cAMP	55	2	1.21E-01
GO:0071385	cellular response to glucocorticoid stimulus	55	2	1.21E-01
GO:1902991	regulation of amyloid precursor protein catabolic proces	55	2	1.21E-01
GO:1904645	response to amyloid-beta	55	2	1.21E-01
GO:0001676	long-chain fatty acid metabolic process	111	3	1.21E-01
GO:0042177	negative regulation of protein catabolic process	111	3	1.21E-01
GO:0071347	cellular response to interleukin-1	111	3	1.21E-01
GO:1901222	regulation of NIK/NF-kappaB signaling	111	3	1.21E-01
GO:0018108	peptidyl-tyrosine phosphorylation	380	7	1.23E-01
GO:0001921	positive regulation of receptor recycling	12	1	1.23E-01
GO:0002551	mast cell chemotaxis	12	1	1.23E-01
GO:0002674	negative regulation of acute inflammatory response	12	1	1.23E-01
GO:0003263	cardioblast proliferation	12	1	1.23E-01
GO:0003264	regulation of cardioblast proliferation	12	1	1.23E-01
GO:0010459	negative regulation of heart rate	12	1	1.23E-01
GO:0010635	regulation of mitochondrial fusion	12	1	1.23E-01
GO:0010749	regulation of nitric oxide mediated signal transduction	12	1	1.23E-01
GO:0010826	negative regulation of centrosome duplication	12	1	1.23E-01
GO:0019321	pentose metabolic process	12	1	1.23E-01
GO:0032490	detection of molecule of bacterial origin	12	1	1.23E-01
GO:0032908	regulation of transforming growth factor beta1 producti	12	1	1.23E-01
GO:0035815	positive regulation of renal sodium excretion	12	1	1.23E-01
GO:0043589	skin morphogenesis	12	1	1.23E-01

GO:0046606	negative regulation of centrosome cycle	12	1	1.23E-01
GO:0046929	negative regulation of neurotransmitter secretion	12	1	1.23E-01
GO:0048548	regulation of pinocytosis	12	1	1.23E-01
GO:0051639	actin filament network formation	12	1	1.23E-01
GO:0051673	membrane disruption in another organism	12	1	1.23E-01
GO:0051798	positive regulation of hair follicle development	12	1	1.23E-01
GO:0060379	cardiac muscle cell myoblast differentiation	12	1	1.23E-01
GO:0060394	negative regulation of pathway-restricted SMAD protein	12	1	1.23E-01
GO:0061309	cardiac neural crest cell development involved in outflov	12	1	1.23E-01
GO:0061635	regulation of protein complex stability	12	1	1.23E-01
GO:0070586	cell-cell adhesion involved in gastrulation	12	1	1.23E-01
GO:0071472	cellular response to salt stress	12	1	1.23E-01
GO:0072110	glomerular mesangial cell proliferation	12	1	1.23E-01
GO:0090331	negative regulation of platelet aggregation	12	1	1.23E-01
GO:0099149	regulation of postsynaptic neurotransmitter receptor int	12	1	1.23E-01
GO:0099188	postsynaptic cytoskeleton organization	12	1	1.23E-01
GO:0106072	negative regulation of adenylate cyclase-activating G pro	12	1	1.23E-01
GO:0140052	cellular response to oxidised low-density lipoprotein par	12	1	1.23E-01
GO:1900227	positive regulation of NLRP3 inflammasome complex ass	12	1	1.23E-01
GO:1903238	positive regulation of leukocyte tethering or rolling	12	1	1.23E-01
GO:1904350	regulation of protein catabolic process in the vacuole	12	1	1.23E-01
GO:1905666	regulation of protein localization to endosome	12	1	1.23E-01
GO:0000782	telomere cap complex	12	1	1.23E-01
GO:0000783	nuclear telomere cap complex	12	1	1.23E-01
GO:0000974	Prp19 complex	12	1	1.23E-01
GO:0005797	Golgi medial cisterna	12	1	1.23E-01
GO:0005833	hemoglobin complex	12	1	1.23E-01
GO:0060198	clathrin-sculpted vesicle	12	1	1.23E-01
GO:0098993	anchored component of synaptic vesicle membrane	12	1	1.23E-01
GO:0008179	adenylate cyclase binding	12	1	1.23E-01
GO:0017166	vinculin binding	12	1	1.23E-01
GO:0030955	potassium ion binding	12	1	1.23E-01
GO:0035197	siRNA binding	12	1	1.23E-01
GO:0043047	single-stranded telomeric DNA binding	12	1	1.23E-01
GO:0046030	inositol trisphosphate phosphatase activity	12	1	1.23E-01
GO:0106222	lncRNA binding	12	1	1.23E-01
GO:0036503	ERAD pathway	112	3	1.24E-01
GO:0048738	cardiac muscle tissue development	241	5	1.24E-01
GO:0002707	negative regulation of lymphocyte mediated immunity	56	2	1.25E-01
GO:0034205	amyloid-beta formation	56	2	1.25E-01
GO:0045761	regulation of adenylate cyclase activity	56	2	1.25E-01
GO:0045806	negative regulation of endocytosis	56	2	1.25E-01
GO:0071453	cellular response to oxygen levels	175	4	1.25E-01
GO:0043197	dendritic spine	175	4	1.25E-01
GO:0018212	peptidyl-tyrosine modification	382	7	1.25E-01
GO:0010633	negative regulation of epithelial cell migration	113	3	1.26E-01
GO:0061640	cytoskeleton-dependent cytokinesis	113	3	1.26E-01

GO:1905954	positive regulation of lipid localization	113	3	1.26E-01
GO:0030900	forebrain development	383	7	1.27E-01
GO:2000146	negative regulation of cell motility	383	7	1.27E-01
GO:0044309	neuron spine	176	4	1.27E-01
GO:0005788	endoplasmic reticulum lumen	312	6	1.27E-01
GO:0008347	glial cell migration	57	2	1.28E-01
GO:0010332	response to gamma radiation	57	2	1.28E-01
GO:0045071	negative regulation of viral genome replication	57	2	1.28E-01
GO:0090175	regulation of establishment of planar polarity	57	2	1.28E-01
GO:0031526	brush border membrane	57	2	1.28E-01
GO:0043266	regulation of potassium ion transport	114	3	1.29E-01
GO:0005884	actin filament	114	3	1.29E-01
GO:0030672	synaptic vesicle membrane	114	3	1.29E-01
GO:0099501	exocytic vesicle membrane	114	3	1.29E-01
GO:0006694	steroid biosynthetic process	178	4	1.31E-01
GO:0044815	DNA packaging complex	178	4	1.31E-01
GO:1901617	organic hydroxy compound biosynthetic process	245	5	1.31E-01
GO:0033559	unsaturated fatty acid metabolic process	115	3	1.31E-01
GO:0042303	molting cycle	115	3	1.31E-01
GO:0042633	hair cycle	115	3	1.31E-01
GO:0030295	protein kinase activator activity	115	3	1.31E-01
GO:0098791	Golgi apparatus subcompartment	387	7	1.32E-01
GO:0010803	regulation of tumor necrosis factor-mediated signaling p	58	2	1.32E-01
GO:0019369	arachidonic acid metabolic process	58	2	1.32E-01
GO:0033619	membrane protein proteolysis	58	2	1.32E-01
GO:0060997	dendritic spine morphogenesis	58	2	1.32E-01
GO:0035577	azurophil granule membrane	58	2	1.32E-01
GO:0031098	stress-activated protein kinase signaling cascade	246	5	1.32E-01
GO:0045580	regulation of T cell differentiation	179	4	1.32E-01
GO:0048771	tissue remodeling	179	4	1.32E-01
GO:0001973	G protein-coupled adenosine receptor signaling pathway	13	1	1.33E-01
GO:0002357	defense response to tumor cell	13	1	1.33E-01
GO:0002664	regulation of T cell tolerance induction	13	1	1.33E-01
GO:0003084	positive regulation of systemic arterial blood pressure	13	1	1.33E-01
GO:0003334	keratinocyte development	13	1	1.33E-01
GO:0005513	detection of calcium ion	13	1	1.33E-01
GO:0007008	outer mitochondrial membrane organization	13	1	1.33E-01
GO:0010745	negative regulation of macrophage derived foam cell dif	13	1	1.33E-01
GO:0010771	negative regulation of cell morphogenesis involved in di	13	1	1.33E-01
GO:0030836	positive regulation of actin filament depolymerization	13	1	1.33E-01
GO:0031272	regulation of pseudopodium assembly	13	1	1.33E-01
GO:0031274	positive regulation of pseudopodium assembly	13	1	1.33E-01
GO:0032494	response to peptidoglycan	13	1	1.33E-01
GO:0032530	regulation of microvillus organization	13	1	1.33E-01
GO:0032621	interleukin-18 production	13	1	1.33E-01
GO:0032661	regulation of interleukin-18 production	13	1	1.33E-01
GO:0035588	G protein-coupled purinergic receptor signaling pathway	13	1	1.33E-01

GO:0035810	positive regulation of urine volume	13	1	1.33E-01
GO:0035865	cellular response to potassium ion	13	1	1.33E-01
GO:0042921	glucocorticoid receptor signaling pathway	13	1	1.33E-01
GO:0045040	protein insertion into mitochondrial outer membrane	13	1	1.33E-01
GO:0045916	negative regulation of complement activation	13	1	1.33E-01
GO:0046831	regulation of RNA export from nucleus	13	1	1.33E-01
GO:0048680	positive regulation of axon regeneration	13	1	1.33E-01
GO:0051775	response to redox state	13	1	1.33E-01
GO:0051918	negative regulation of fibrinolysis	13	1	1.33E-01
GO:0060100	positive regulation of phagocytosis, engulfment	13	1	1.33E-01
GO:0061052	negative regulation of cell growth involved in cardiac mu	13	1	1.33E-01
GO:0070424	regulation of nucleotide-binding oligomerization domair	13	1	1.33E-01
GO:0071481	cellular response to X-ray	13	1	1.33E-01
GO:0090235	regulation of metaphase plate congression	13	1	1.33E-01
GO:0098840	protein transport along microtubule	13	1	1.33E-01
GO:0098935	dendritic transport	13	1	1.33E-01
GO:0099118	microtubule-based protein transport	13	1	1.33E-01
GO:1900025	negative regulation of substrate adhesion-dependent ce	13	1	1.33E-01
GO:1900242	regulation of synaptic vesicle endocytosis	13	1	1.33E-01
GO:1903265	positive regulation of tumor necrosis factor-mediated si	13	1	1.33E-01
GO:1903651	positive regulation of cytoplasmic transport	13	1	1.33E-01
GO:1903961	positive regulation of anion transmembrane transport	13	1	1.33E-01
GO:1905155	positive regulation of membrane invagination	13	1	1.33E-01
GO:2001046	positive regulation of integrin-mediated signaling pathw	13	1	1.33E-01
GO:2001198	regulation of dendritic cell differentiation	13	1	1.33E-01
GO:0001401	SAM complex	13	1	1.33E-01
GO:0043218	compact myelin	13	1	1.33E-01
GO:0044194	cytolytic granule	13	1	1.33E-01
GO:0044292	dendrite terminus	13	1	1.33E-01
GO:0099524	postsynaptic cytosol	13	1	1.33E-01
GO:0140275	MIB complex	13	1	1.33E-01
GO:0140445	chromosome, telomeric repeat region	13	1	1.33E-01
GO:1905286	serine-type peptidase complex	13	1	1.33E-01
GO:0031432	titin binding	13	1	1.33E-01
GO:0034595	phosphatidylinositol phosphate 5-phosphatase activity	13	1	1.33E-01
GO:0035259	nuclear glucocorticoid receptor binding	13	1	1.33E-01
GO:0043560	insulin receptor substrate binding	13	1	1.33E-01
GO:0060590	ATPase regulator activity	13	1	1.33E-01
GO:0097157	pre-mRNA intronic binding	13	1	1.33E-01
GO:0099186	structural constituent of postsynapse	13	1	1.33E-01
GO:0001824	blastocyst development	116	3	1.33E-01
GO:0098586	cellular response to virus	116	3	1.33E-01
GO:0051259	protein complex oligomerization	247	5	1.34E-01
GO:1901981	phosphatidylinositol phosphate binding	180	4	1.34E-01
GO:0048863	stem cell differentiation	248	5	1.35E-01
GO:0006611	protein export from nucleus	59	2	1.36E-01
GO:0007566	embryo implantation	59	2	1.36E-01

GO:0043666	regulation of phosphoprotein phosphatase activity	59	2	1.36E-01
GO:0072666	establishment of protein localization to vacuole	59	2	1.36E-01
GO:0009206	purine ribonucleoside triphosphate biosynthetic process	117	3	1.36E-01
GO:0045926	negative regulation of growth	249	5	1.37E-01
GO:0002791	regulation of peptide secretion	182	4	1.38E-01
GO:0009145	purine nucleoside triphosphate biosynthetic process	118	3	1.38E-01
GO:1901800	positive regulation of proteasomal protein catabolic pro	118	3	1.38E-01
GO:0006893	Golgi to plasma membrane transport	60	2	1.39E-01
GO:0022029	telencephalon cell migration	60	2	1.39E-01
GO:0043407	negative regulation of MAP kinase activity	60	2	1.39E-01
GO:0046686	response to cadmium ion	60	2	1.39E-01
GO:0051353	positive regulation of oxidoreductase activity	60	2	1.39E-01
GO:1903670	regulation of sprouting angiogenesis	60	2	1.39E-01
GO:2000300	regulation of synaptic vesicle exocytosis	60	2	1.39E-01
GO:2000649	regulation of sodium ion transmembrane transporter ac	60	2	1.39E-01
GO:0014069	postsynaptic density	321	6	1.40E-01
GO:0005739	mitochondrion	1660	23	1.40E-01
GO:0031901	early endosome membrane	183	4	1.40E-01
GO:0031398	positive regulation of protein ubiquitination	119	3	1.41E-01
GO:0071900	regulation of protein serine/threonine kinase activity	395	7	1.42E-01
GO:0032886	regulation of microtubule-based process	252	5	1.42E-01
GO:0090087	regulation of peptide transport	184	4	1.42E-01
GO:0002475	antigen processing and presentation via MHC class Ib	14	1	1.42E-01
GO:0006000	fructose metabolic process	14	1	1.42E-01
GO:0006167	AMP biosynthetic process	14	1	1.42E-01
GO:0006177	GMP biosynthetic process	14	1	1.42E-01
GO:0006999	nuclear pore organization	14	1	1.42E-01
GO:0010269	response to selenium ion	14	1	1.42E-01
GO:0010752	regulation of cGMP-mediated signaling	14	1	1.42E-01
GO:0030049	muscle filament sliding	14	1	1.42E-01
GO:0031958	corticosteroid receptor signaling pathway	14	1	1.42E-01
GO:0032486	Rap protein signal transduction	14	1	1.42E-01
GO:0034111	negative regulation of homotypic cell-cell adhesion	14	1	1.42E-01
GO:0035630	bone mineralization involved in bone maturation	14	1	1.42E-01
GO:0035791	platelet-derived growth factor receptor-beta signaling p	14	1	1.42E-01
GO:0042117	monocyte activation	14	1	1.42E-01
GO:0042538	hyperosmotic salinity response	14	1	1.42E-01
GO:0043558	regulation of translational initiation in response to stress	14	1	1.42E-01
GO:0043922	negative regulation by host of viral transcription	14	1	1.42E-01
GO:0044793	negative regulation by host of viral process	14	1	1.42E-01
GO:0046185	aldehyde catabolic process	14	1	1.42E-01
GO:0051764	actin crosslink formation	14	1	1.42E-01
GO:0055057	neuroblast division	14	1	1.42E-01
GO:0060019	radial glial cell differentiation	14	1	1.42E-01
GO:0070307	lens fiber cell development	14	1	1.42E-01
GO:0070572	positive regulation of neuron projection regeneration	14	1	1.42E-01
GO:0071361	cellular response to ethanol	14	1	1.42E-01

GO:0071888	macrophage apoptotic process	14	1	1.42E-01
GO:0090128	regulation of synapse maturation	14	1	1.42E-01
GO:0090158	endoplasmic reticulum membrane organization	14	1	1.42E-01
GO:0098814	spontaneous synaptic transmission	14	1	1.42E-01
GO:1901077	regulation of relaxation of muscle	14	1	1.42E-01
GO:1901533	negative regulation of hematopoietic progenitor cell diff	14	1	1.42E-01
GO:1902043	positive regulation of extrinsic apoptotic signaling pathw	14	1	1.42E-01
GO:1902947	regulation of tau-protein kinase activity	14	1	1.42E-01
GO:1904044	response to aldosterone	14	1	1.42E-01
GO:1904152	regulation of retrograde protein transport, ER to cytosol	14	1	1.42E-01
GO:1905049	negative regulation of metallopeptidase activity	14	1	1.42E-01
GO:2001171	positive regulation of ATP biosynthetic process	14	1	1.42E-01
GO:0016586	RSC-type complex	14	1	1.42E-01
GO:0030014	CCR4-NOT complex	14	1	1.42E-01
GO:0030132	clathrin coat of coated pit	14	1	1.42E-01
GO:0030673	axolemma	14	1	1.42E-01
GO:0031089	platelet dense granule lumen	14	1	1.42E-01
GO:0031616	spindle pole centrosome	14	1	1.42E-01
GO:0035060	brahma complex	14	1	1.42E-01
GO:0042588	zymogen granule	14	1	1.42E-01
GO:0045120	pronucleus	14	1	1.42E-01
GO:0071564	npBAF complex	14	1	1.42E-01
GO:0097524	sperm plasma membrane	14	1	1.42E-01
GO:0098574	cytoplasmic side of lysosomal membrane	14	1	1.42E-01
GO:0098637	protein complex involved in cell-matrix adhesion	14	1	1.42E-01
GO:0099059	integral component of presynaptic active zone membrar	14	1	1.42E-01
GO:0140288	GBAF complex	14	1	1.42E-01
GO:1990498	mitotic spindle microtubule	14	1	1.42E-01
GO:0005172	vascular endothelial growth factor receptor binding	14	1	1.42E-01
GO:0016004	phospholipase activator activity	14	1	1.42E-01
GO:0032036	myosin heavy chain binding	14	1	1.42E-01
GO:0043023	ribosomal large subunit binding	14	1	1.42E-01
GO:0043138	3'-5' DNA helicase activity	14	1	1.42E-01
GO:0061783	peptidoglycan muralytic activity	14	1	1.42E-01
GO:0106019	phosphatidylinositol-4,5-bisphosphate phosphatase acti	14	1	1.42E-01
GO:0140313	molecular sequestering activity	14	1	1.42E-01
GO:1990825	sequence-specific mRNA binding	14	1	1.42E-01
GO:0016447	somatic recombination of immunoglobulin gene segmer	61	2	1.43E-01
GO:0046456	icosanoid biosynthetic process	61	2	1.43E-01
GO:0060135	maternal process involved in female pregnancy	61	2	1.43E-01
GO:0001098	basal transcription machinery binding	61	2	1.43E-01
GO:0001099	basal RNA polymerase II transcription machinery binding	61	2	1.43E-01
GO:0032934	sterol binding	61	2	1.43E-01
GO:0022402	cell cycle process	1256	18	1.44E-01
GO:0015849	organic acid transport	324	6	1.44E-01
GO:0002040	sprouting angiogenesis	185	4	1.44E-01
GO:0140014	mitotic nuclear division	325	6	1.46E-01

GO:0008094	ATP-dependent activity, acting on DNA	121	3	1.46E-01
GO:0019209	kinase activator activity	121	3	1.46E-01
GO:0000381	regulation of alternative mRNA splicing, via spliceosome	62	2	1.47E-01
GO:0006360	transcription by RNA polymerase I	62	2	1.47E-01
GO:0006767	water-soluble vitamin metabolic process	62	2	1.47E-01
GO:0061951	establishment of protein localization to plasma membra	62	2	1.47E-01
GO:0046658	anchored component of plasma membrane	62	2	1.47E-01
GO:1904115	axon cytoplasm	62	2	1.47E-01
GO:0021537	telencephalon development	255	5	1.47E-01
GO:0032279	asymmetric synapse	327	6	1.48E-01
GO:0009055	electron transfer activity	122	3	1.49E-01
GO:0014706	striated muscle tissue development	257	5	1.50E-01
GO:0021885	forebrain cell migration	63	2	1.50E-01
GO:0070542	response to fatty acid	63	2	1.50E-01
GO:0099072	regulation of postsynaptic membrane neurotransmitter	63	2	1.50E-01
GO:1990841	promoter-specific chromatin binding	63	2	1.50E-01
GO:0009201	ribonucleoside triphosphate biosynthetic process	123	3	1.51E-01
GO:0000212	meiotic spindle organization	15	1	1.52E-01
GO:0001325	formation of extrachromosomal circular DNA	15	1	1.52E-01
GO:0001768	establishment of T cell polarity	15	1	1.52E-01
GO:0001771	immunological synapse formation	15	1	1.52E-01
GO:0002468	dendritic cell antigen processing and presentation	15	1	1.52E-01
GO:0002517	T cell tolerance induction	15	1	1.52E-01
GO:0032488	Cdc42 protein signal transduction	15	1	1.52E-01
GO:0032905	transforming growth factor beta1 production	15	1	1.52E-01
GO:0033599	regulation of mammary gland epithelial cell proliferatior	15	1	1.52E-01
GO:0034116	positive regulation of heterotypic cell-cell adhesion	15	1	1.52E-01
GO:0042308	negative regulation of protein import into nucleus	15	1	1.52E-01
GO:0042559	pteridine-containing compound biosynthetic process	15	1	1.52E-01
GO:0042976	activation of Janus kinase activity	15	1	1.52E-01
GO:0042994	cytoplasmic sequestering of transcription factor	15	1	1.52E-01
GO:0045176	apical protein localization	15	1	1.52E-01
GO:0045579	positive regulation of B cell differentiation	15	1	1.52E-01
GO:0045989	positive regulation of striated muscle contraction	15	1	1.52E-01
GO:0048291	isotype switching to IgG isotypes	15	1	1.52E-01
GO:0048308	organelle inheritance	15	1	1.52E-01
GO:0048313	Golgi inheritance	15	1	1.52E-01
GO:0051589	negative regulation of neurotransmitter transport	15	1	1.52E-01
GO:0051956	negative regulation of amino acid transport	15	1	1.52E-01
GO:0060099	regulation of phagocytosis, engulfment	15	1	1.52E-01
GO:0061307	cardiac neural crest cell differentiation involved in heart	15	1	1.52E-01
GO:0061308	cardiac neural crest cell development involved in heart d	15	1	1.52E-01
GO:0070234	positive regulation of T cell apoptotic process	15	1	1.52E-01
GO:0070262	peptidyl-serine dephosphorylation	15	1	1.52E-01
GO:0070863	positive regulation of protein exit from endoplasmic reti	15	1	1.52E-01
GO:0090656	t-circle formation	15	1	1.52E-01
GO:0090737	telomere maintenance via telomere trimming	15	1	1.52E-01

GO:0097531	mast cell migration	15	1	1.52E-01
GO:0106070	regulation of adenylate cyclase-activating G protein-cou	15	1	1.52E-01
GO:1900029	positive regulation of ruffle assembly	15	1	1.52E-01
GO:1901722	regulation of cell proliferation involved in kidney develo	15	1	1.52E-01
GO:1901836	regulation of transcription of nucleolar large rRNA by RN	15	1	1.52E-01
GO:1905153	regulation of membrane invagination	15	1	1.52E-01
GO:2000671	regulation of motor neuron apoptotic process	15	1	1.52E-01
GO:0004029	aldehyde dehydrogenase (NAD+) activity	15	1	1.52E-01
GO:0032794	GTPase activating protein binding	15	1	1.52E-01
GO:0097153	cysteine-type endopeptidase activity involved in apopto	15	1	1.52E-01
GO:0071478	cellular response to radiation	189	4	1.52E-01
GO:0055007	cardiac muscle cell differentiation	124	3	1.54E-01
GO:0002712	regulation of B cell mediated immunity	64	2	1.54E-01
GO:0002889	regulation of immunoglobulin mediated immune respon	64	2	1.54E-01
GO:0050918	positive chemotaxis	64	2	1.54E-01
GO:0071384	cellular response to corticosteroid stimulus	64	2	1.54E-01
GO:0098930	axonal transport	64	2	1.54E-01
GO:0016328	lateral plasma membrane	64	2	1.54E-01
GO:1903047	mitotic cell cycle process	789	12	1.55E-01
GO:0090068	positive regulation of cell cycle process	260	5	1.56E-01
GO:1901135	carbohydrate derivative metabolic process	1111	16	1.57E-01
GO:0002440	production of molecular mediator of immune response	333	6	1.58E-01
GO:0006940	regulation of smooth muscle contraction	65	2	1.58E-01
GO:0015800	acidic amino acid transport	65	2	1.58E-01
GO:0032507	maintenance of protein location in cell	65	2	1.58E-01
GO:0032613	interleukin-10 production	65	2	1.58E-01
GO:0032653	regulation of interleukin-10 production	65	2	1.58E-01
GO:0048247	lymphocyte chemotaxis	65	2	1.58E-01
GO:0050435	amyloid-beta metabolic process	65	2	1.58E-01
GO:0060760	positive regulation of response to cytokine stimulus	65	2	1.58E-01
GO:1905953	negative regulation of lipid localization	65	2	1.58E-01
GO:0006690	icosanoid metabolic process	126	3	1.59E-01
GO:0043244	regulation of protein-containing complex disassembly	126	3	1.59E-01
GO:0010466	negative regulation of peptidase activity	263	5	1.61E-01
GO:0001767	establishment of lymphocyte polarity	16	1	1.61E-01
GO:0002399	MHC class II protein complex assembly	16	1	1.61E-01
GO:0002503	peptide antigen assembly with MHC class II protein com	16	1	1.61E-01
GO:0010919	regulation of inositol phosphate biosynthetic process	16	1	1.61E-01
GO:0021535	cell migration in hindbrain	16	1	1.61E-01
GO:0023035	CD40 signaling pathway	16	1	1.61E-01
GO:0030033	microvillus assembly	16	1	1.61E-01
GO:0030852	regulation of granulocyte differentiation	16	1	1.61E-01
GO:0031053	primary miRNA processing	16	1	1.61E-01
GO:0031269	pseudopodium assembly	16	1	1.61E-01
GO:0032239	regulation of nucleobase-containing compound transpo	16	1	1.61E-01
GO:0033275	actin-myosin filament sliding	16	1	1.61E-01
GO:0034638	phosphatidylcholine catabolic process	16	1	1.61E-01

GO:0042159	lipoprotein catabolic process	16	1	1.61E-01
GO:0043249	erythrocyte maturation	16	1	1.61E-01
GO:0043486	histone exchange	16	1	1.61E-01
GO:0043508	negative regulation of JUN kinase activity	16	1	1.61E-01
GO:0043981	histone H4-K5 acetylation	16	1	1.61E-01
GO:0043982	histone H4-K8 acetylation	16	1	1.61E-01
GO:0044406	adhesion of symbiont to host	16	1	1.61E-01
GO:0045779	negative regulation of bone resorption	16	1	1.61E-01
GO:0045986	negative regulation of smooth muscle contraction	16	1	1.61E-01
GO:0046184	aldehyde biosynthetic process	16	1	1.61E-01
GO:0048266	behavioral response to pain	16	1	1.61E-01
GO:0048268	clathrin coat assembly	16	1	1.61E-01
GO:0050862	positive regulation of T cell receptor signaling pathway	16	1	1.61E-01
GO:0060088	auditory receptor cell stereocilium organization	16	1	1.61E-01
GO:0060911	cardiac cell fate commitment	16	1	1.61E-01
GO:0070431	nucleotide-binding oligomerization domain containing 2	16	1	1.61E-01
GO:0090336	positive regulation of brown fat cell differentiation	16	1	1.61E-01
GO:0099633	protein localization to postsynaptic specialization memb	16	1	1.61E-01
GO:0099645	neurotransmitter receptor localization to postsynaptic s	16	1	1.61E-01
GO:0001674	female germ cell nucleus	16	1	1.61E-01
GO:0005744	TIM23 mitochondrial import inner membrane transloca	16	1	1.61E-01
GO:0043034	costamere	16	1	1.61E-01
GO:0071565	nBAF complex	16	1	1.61E-01
GO:0099522	cytosolic region	16	1	1.61E-01
GO:0010851	cyclase regulator activity	16	1	1.61E-01
GO:0017070	U6 snRNA binding	16	1	1.61E-01
GO:0050664	oxidoreductase activity, acting on NAD(P)H, oxygen as a	16	1	1.61E-01
GO:0072542	protein phosphatase activator activity	16	1	1.61E-01
GO:0045682	regulation of epidermis development	66	2	1.62E-01
GO:0048278	vesicle docking	66	2	1.62E-01
GO:0050854	regulation of antigen receptor-mediated signaling pathw	66	2	1.62E-01
GO:0043073	germ cell nucleus	66	2	1.62E-01
GO:0061136	regulation of proteasomal protein catabolic process	194	4	1.63E-01
GO:0046683	response to organophosphorus	128	3	1.64E-01
GO:0032872	regulation of stress-activated MAPK cascade	195	4	1.65E-01
GO:0120031	plasma membrane bounded cell projection assembly	564	9	1.65E-01
GO:0006790	sulfur compound metabolic process	338	6	1.65E-01
GO:0007405	neuroblast proliferation	67	2	1.66E-01
GO:0030888	regulation of B cell proliferation	67	2	1.66E-01
GO:0045576	mast cell activation	67	2	1.66E-01
GO:1901224	positive regulation of NIK/NF-kappaB signaling	67	2	1.66E-01
GO:0098656	anion transmembrane transport	266	5	1.66E-01
GO:0007041	lysosomal transport	129	3	1.67E-01
GO:0050714	positive regulation of protein secretion	129	3	1.67E-01
GO:0045621	positive regulation of lymphocyte differentiation	130	3	1.69E-01
GO:0016298	lipase activity	130	3	1.69E-01
GO:0002704	negative regulation of leukocyte mediated immunity	68	2	1.70E-01

GO:0032024	positive regulation of insulin secretion	68	2	1.70E-01
GO:0038034	signal transduction in absence of ligand	68	2	1.70E-01
GO:0045670	regulation of osteoclast differentiation	68	2	1.70E-01
GO:0046503	glycerolipid catabolic process	68	2	1.70E-01
GO:0097192	extrinsic apoptotic signaling pathway in absence of ligan	68	2	1.70E-01
GO:0031594	neuromuscular junction	68	2	1.70E-01
GO:0002091	negative regulation of receptor internalization	17	1	1.70E-01
GO:0002921	negative regulation of humoral immune response	17	1	1.70E-01
GO:0015867	ATP transport	17	1	1.70E-01
GO:0030949	positive regulation of vascular endothelial growth factor	17	1	1.70E-01
GO:0031268	pseudopodium organization	17	1	1.70E-01
GO:0032048	cardiolipin metabolic process	17	1	1.70E-01
GO:0033194	response to hydroperoxide	17	1	1.70E-01
GO:0034497	protein localization to phagophore assembly site	17	1	1.70E-01
GO:0051412	response to corticosterone	17	1	1.70E-01
GO:0072109	glomerular mesangium development	17	1	1.70E-01
GO:0080154	regulation of fertilization	17	1	1.70E-01
GO:0090083	regulation of inclusion body assembly	17	1	1.70E-01
GO:0110096	cellular response to aldehyde	17	1	1.70E-01
GO:1900037	regulation of cellular response to hypoxia	17	1	1.70E-01
GO:1901163	regulation of trophoblast cell migration	17	1	1.70E-01
GO:1902036	regulation of hematopoietic stem cell differentiation	17	1	1.70E-01
GO:1902514	regulation of calcium ion transmembrane transport via h	17	1	1.70E-01
GO:1903236	regulation of leukocyte tethering or rolling	17	1	1.70E-01
GO:1903358	regulation of Golgi organization	17	1	1.70E-01
GO:0042613	MHC class II protein complex	17	1	1.70E-01
GO:0061827	sperm head	17	1	1.70E-01
GO:0098945	intrinsic component of presynaptic active zone membra	17	1	1.70E-01
GO:0004030	aldehyde dehydrogenase [NAD(P)+] activity	17	1	1.70E-01
GO:0008308	voltage-gated anion channel activity	17	1	1.70E-01
GO:0008656	cysteine-type endopeptidase activator activity involved	17	1	1.70E-01
GO:0008970	phospholipase A1 activity	17	1	1.70E-01
GO:0016805	dipeptidase activity	17	1	1.70E-01
GO:0016854	racemase and epimerase activity	17	1	1.70E-01
GO:0031420	alkali metal ion binding	17	1	1.70E-01
GO:0043024	ribosomal small subunit binding	17	1	1.70E-01
GO:0046933	proton-transporting ATP synthase activity, rotational me	17	1	1.70E-01
GO:0060229	lipase activator activity	17	1	1.70E-01
GO:0070064	proline-rich region binding	17	1	1.70E-01
GO:0070302	regulation of stress-activated protein kinase signaling ca	198	4	1.71E-01
GO:0010595	positive regulation of endothelial cell migration	131	3	1.72E-01
GO:0090150	establishment of protein localization to membrane	270	5	1.73E-01
GO:0032835	glomerulus development	69	2	1.74E-01
GO:0035418	protein localization to synapse	69	2	1.74E-01
GO:0046847	filopodium assembly	69	2	1.74E-01
GO:0051148	negative regulation of muscle cell differentiation	69	2	1.74E-01
GO:0070830	bicellular tight junction assembly	69	2	1.74E-01

GO:1902305	regulation of sodium ion transmembrane transport	69	2	1.74E-01
GO:0005518	collagen binding	69	2	1.74E-01
GO:0030276	clathrin binding	69	2	1.74E-01
GO:0040013	negative regulation of locomotion	419	7	1.75E-01
GO:0006956	complement activation	132	3	1.75E-01
GO:0051983	regulation of chromosome segregation	132	3	1.75E-01
GO:0070160	tight junction	132	3	1.75E-01
GO:0043025	neuronal cell body	497	8	1.77E-01
GO:0005244	voltage-gated ion channel activity	201	4	1.77E-01
GO:0022832	voltage-gated channel activity	201	4	1.77E-01
GO:0006081	cellular aldehyde metabolic process	70	2	1.77E-01
GO:0016445	somatic diversification of immunoglobulins	70	2	1.77E-01
GO:1901016	regulation of potassium ion transmembrane transporter	70	2	1.77E-01
GO:0031491	nucleosome binding	70	2	1.77E-01
GO:0000786	nucleosome	133	3	1.78E-01
GO:0030031	cell projection assembly	575	9	1.78E-01
GO:0002093	auditory receptor cell morphogenesis	18	1	1.79E-01
GO:0002295	T-helper cell lineage commitment	18	1	1.79E-01
GO:0003159	morphogenesis of an endothelium	18	1	1.79E-01
GO:0006415	translational termination	18	1	1.79E-01
GO:0008356	asymmetric cell division	18	1	1.79E-01
GO:0034501	protein localization to kinetochore	18	1	1.79E-01
GO:0035234	ectopic germ cell programmed cell death	18	1	1.79E-01
GO:0035864	response to potassium ion	18	1	1.79E-01
GO:0043116	negative regulation of vascular permeability	18	1	1.79E-01
GO:0043217	myelin maintenance	18	1	1.79E-01
GO:0046040	IMP metabolic process	18	1	1.79E-01
GO:0046653	tetrahydrofolate metabolic process	18	1	1.79E-01
GO:0046827	positive regulation of protein export from nucleus	18	1	1.79E-01
GO:0046851	negative regulation of bone remodeling	18	1	1.79E-01
GO:0050746	regulation of lipoprotein metabolic process	18	1	1.79E-01
GO:0051917	regulation of fibrinolysis	18	1	1.79E-01
GO:0061154	endothelial tube morphogenesis	18	1	1.79E-01
GO:0061450	trophoblast cell migration	18	1	1.79E-01
GO:0070230	positive regulation of lymphocyte apoptotic process	18	1	1.79E-01
GO:0071318	cellular response to ATP	18	1	1.79E-01
GO:0090493	catecholamine uptake	18	1	1.79E-01
GO:1902570	protein localization to nucleolus	18	1	1.79E-01
GO:1902683	regulation of receptor localization to synapse	18	1	1.79E-01
GO:1903083	protein localization to condensed chromosome	18	1	1.79E-01
GO:1905146	lysosomal protein catabolic process	18	1	1.79E-01
GO:2000641	regulation of early endosome to late endosome transpo	18	1	1.79E-01
GO:2000811	negative regulation of anoikis	18	1	1.79E-01
GO:0030130	clathrin coat of trans-Golgi network vesicle	18	1	1.79E-01
GO:0031143	pseudopodium	18	1	1.79E-01
GO:0031527	filopodium membrane	18	1	1.79E-01
GO:0044305	calyx of Held	18	1	1.79E-01

GO:0004438	phosphatidylinositol-3-phosphatase activity	18	1	1.79E-01
GO:0043395	heparan sulfate proteoglycan binding	18	1	1.79E-01
GO:0045236	CXCR chemokine receptor binding	18	1	1.79E-01
GO:0052745	inositol phosphate phosphatase activity	18	1	1.79E-01
GO:0022408	negative regulation of cell-cell adhesion	202	4	1.79E-01
GO:0006888	endoplasmic reticulum to Golgi vesicle-mediated transp	134	3	1.80E-01
GO:0009142	nucleoside triphosphate biosynthetic process	134	3	1.80E-01
GO:0007517	muscle organ development	348	6	1.81E-01
GO:0002562	somatic diversification of immune receptors via germlin	71	2	1.81E-01
GO:0016444	somatic cell DNA recombination	71	2	1.81E-01
GO:0042379	chemokine receptor binding	71	2	1.81E-01
GO:0043621	protein self-association	71	2	1.81E-01
GO:0035567	non-canonical Wnt signaling pathway	72	2	1.85E-01
GO:0045911	positive regulation of DNA recombination	72	2	1.85E-01
GO:0046626	regulation of insulin receptor signaling pathway	72	2	1.85E-01
GO:1900076	regulation of cellular response to insulin stimulus	72	2	1.85E-01
GO:0098685	Schaffer collateral - CA1 synapse	72	2	1.85E-01
GO:1903052	positive regulation of proteolysis involved in protein cat	136	3	1.86E-01
GO:0035091	phosphatidylinositol binding	277	5	1.86E-01
GO:0045861	negative regulation of proteolysis	352	6	1.88E-01
GO:1903532	positive regulation of secretion by cell	278	5	1.88E-01
GO:0002281	macrophage activation involved in immune response	19	1	1.88E-01
GO:0002283	neutrophil activation involved in immune response	19	1	1.88E-01
GO:0006837	serotonin transport	19	1	1.88E-01
GO:0009226	nucleotide-sugar biosynthetic process	19	1	1.88E-01
GO:0010544	negative regulation of platelet activation	19	1	1.88E-01
GO:0010744	positive regulation of macrophage derived foam cell diff	19	1	1.88E-01
GO:0010801	negative regulation of peptidyl-threonine phosphorylati	19	1	1.88E-01
GO:0010885	regulation of cholesterol storage	19	1	1.88E-01
GO:0030299	intestinal cholesterol absorption	19	1	1.88E-01
GO:0030889	negative regulation of B cell proliferation	19	1	1.88E-01
GO:0032026	response to magnesium ion	19	1	1.88E-01
GO:0032695	negative regulation of interleukin-12 production	19	1	1.88E-01
GO:0034162	toll-like receptor 9 signaling pathway	19	1	1.88E-01
GO:0034244	negative regulation of transcription elongation by RNA p	19	1	1.88E-01
GO:0035372	protein localization to microtubule	19	1	1.88E-01
GO:0035813	regulation of renal sodium excretion	19	1	1.88E-01
GO:0046782	regulation of viral transcription	19	1	1.88E-01
GO:0048245	eosinophil chemotaxis	19	1	1.88E-01
GO:0051797	regulation of hair follicle development	19	1	1.88E-01
GO:0061577	calcium ion transmembrane transport via high voltage-g	19	1	1.88E-01
GO:0086014	atrial cardiac muscle cell action potential	19	1	1.88E-01
GO:0086026	atrial cardiac muscle cell to AV node cell signaling	19	1	1.88E-01
GO:0086066	atrial cardiac muscle cell to AV node cell communication	19	1	1.88E-01
GO:0090036	regulation of protein kinase C signaling	19	1	1.88E-01
GO:0098884	postsynaptic neurotransmitter receptor internalization	19	1	1.88E-01
GO:0140239	postsynaptic endocytosis	19	1	1.88E-01

GO:1901881	positive regulation of protein depolymerization	19	1	1.88E-01
GO:1902176	negative regulation of oxidative stress-induced intrinsic	19	1	1.88E-01
GO:2000136	regulation of cell proliferation involved in heart morpho	19	1	1.88E-01
GO:2000251	positive regulation of actin cytoskeleton reorganization	19	1	1.88E-01
GO:2000434	regulation of protein neddylation	19	1	1.88E-01
GO:2000774	positive regulation of cellular senescence	19	1	1.88E-01
GO:2001185	regulation of CD8-positive, alpha-beta T cell activation	19	1	1.88E-01
GO:0033178	proton-transporting two-sector ATPase complex, catalyt	19	1	1.88E-01
GO:0034045	phagophore assembly site membrane	19	1	1.88E-01
GO:0034506	chromosome, centromeric core domain	19	1	1.88E-01
GO:0030506	ankyrin binding	19	1	1.88E-01
GO:0052744	phosphatidylinositol monophosphate phosphatase activ	19	1	1.88E-01
GO:0098918	structural constituent of synapse	19	1	1.88E-01
GO:1903322	positive regulation of protein modification by small prot	137	3	1.88E-01
GO:0046635	positive regulation of alpha-beta T cell activation	73	2	1.89E-01
GO:0050805	negative regulation of synaptic transmission	73	2	1.89E-01
GO:0150076	neuroinflammatory response	73	2	1.89E-01
GO:0002285	lymphocyte activation involved in immune response	207	4	1.90E-01
GO:0005741	mitochondrial outer membrane	207	4	1.90E-01
GO:0005654	nucleoplasm	4155	51	1.91E-01
GO:0008021	synaptic vesicle	208	4	1.93E-01
GO:0001707	mesoderm formation	74	2	1.93E-01
GO:0001736	establishment of planar polarity	74	2	1.93E-01
GO:0007164	establishment of tissue polarity	74	2	1.93E-01
GO:0007585	respiratory gaseous exchange by respiratory system	74	2	1.93E-01
GO:0043967	histone H4 acetylation	74	2	1.93E-01
GO:0050688	regulation of defense response to virus	74	2	1.93E-01
GO:0008203	cholesterol metabolic process	139	3	1.94E-01
GO:0045165	cell fate commitment	282	5	1.95E-01
GO:0098742	cell-cell adhesion via plasma-membrane adhesion molec	282	5	1.95E-01
GO:1901362	organic cyclic compound biosynthetic process	4253	52	1.97E-01
GO:0002396	MHC protein complex assembly	20	1	1.97E-01
GO:0002501	peptide antigen assembly with MHC protein complex	20	1	1.97E-01
GO:0002577	regulation of antigen processing and presentation	20	1	1.97E-01
GO:0002643	regulation of tolerance induction	20	1	1.97E-01
GO:0006448	regulation of translational elongation	20	1	1.97E-01
GO:0007603	phototransduction, visible light	20	1	1.97E-01
GO:0008340	determination of adult lifespan	20	1	1.97E-01
GO:0009168	purine ribonucleoside monophosphate biosynthetic pro	20	1	1.97E-01
GO:0030220	platelet formation	20	1	1.97E-01
GO:0032495	response to muramyl dipeptide	20	1	1.97E-01
GO:0045655	regulation of monocyte differentiation	20	1	1.97E-01
GO:0048643	positive regulation of skeletal muscle tissue developmer	20	1	1.97E-01
GO:0060339	negative regulation of type I interferon-mediated signali	20	1	1.97E-01
GO:0061323	cell proliferation involved in heart morphogenesis	20	1	1.97E-01
GO:0097320	plasma membrane tubulation	20	1	1.97E-01
GO:1900221	regulation of amyloid-beta clearance	20	1	1.97E-01

GO:1900225	regulation of NLRP3 inflammasome complex assembly	20	1	1.97E-01
GO:1902473	regulation of protein localization to synapse	20	1	1.97E-01
GO:1903421	regulation of synaptic vesicle recycling	20	1	1.97E-01
GO:1903427	negative regulation of reactive oxygen species biosynthe	20	1	1.97E-01
GO:2000678	negative regulation of transcription regulatory region DI	20	1	1.97E-01
GO:0012510	trans-Golgi network transport vesicle membrane	20	1	1.97E-01
GO:0034361	very-low-density lipoprotein particle	20	1	1.97E-01
GO:0034385	triglyceride-rich plasma lipoprotein particle	20	1	1.97E-01
GO:0036020	endolysosome membrane	20	1	1.97E-01
GO:0015662	P-type ion transporter activity	20	1	1.97E-01
GO:0016018	cyclosporin A binding	20	1	1.97E-01
GO:0016505	peptidase activator activity involved in apoptotic proces	20	1	1.97E-01
GO:0097001	ceramide binding	20	1	1.97E-01
GO:0043536	positive regulation of blood vessel endothelial cell migra	75	2	1.97E-01
GO:0046323	glucose import	75	2	1.97E-01
GO:2000242	negative regulation of reproductive process	75	2	1.97E-01
GO:0048786	presynaptic active zone	75	2	1.97E-01
GO:0043547	positive regulation of GTPase activity	283	5	1.97E-01
GO:0031981	nuclear lumen	4964	60	1.99E-01
GO:0045667	regulation of osteoblast differentiation	141	3	2.00E-01
GO:0070555	response to interleukin-1	141	3	2.00E-01
GO:0015807	L-amino acid transport	76	2	2.01E-01
GO:0048332	mesoderm morphogenesis	76	2	2.01E-01
GO:0051339	regulation of lyase activity	76	2	2.01E-01
GO:0099568	cytoplasmic region	285	5	2.01E-01
GO:0016051	carbohydrate biosynthetic process	212	4	2.01E-01
GO:0014074	response to purine-containing compound	142	3	2.02E-01
GO:0038061	NIK/NF-kappaB signaling	142	3	2.02E-01
GO:0042552	myelination	142	3	2.02E-01
GO:0045787	positive regulation of cell cycle	362	6	2.04E-01
GO:0031279	regulation of cyclase activity	77	2	2.05E-01
GO:0032720	negative regulation of tumor necrosis factor production	77	2	2.05E-01
GO:0090559	regulation of membrane permeability	77	2	2.05E-01
GO:0010212	response to ionizing radiation	143	3	2.05E-01
GO:0033135	regulation of peptidyl-serine phosphorylation	143	3	2.05E-01
GO:0006067	ethanol metabolic process	21	1	2.06E-01
GO:0007194	negative regulation of adenylate cyclase activity	21	1	2.06E-01
GO:0007413	axonal fasciculation	21	1	2.06E-01
GO:0008090	retrograde axonal transport	21	1	2.06E-01
GO:0010002	cardioblast differentiation	21	1	2.06E-01
GO:0010878	cholesterol storage	21	1	2.06E-01
GO:0030502	negative regulation of bone mineralization	21	1	2.06E-01
GO:0031338	regulation of vesicle fusion	21	1	2.06E-01
GO:0032482	Rab protein signal transduction	21	1	2.06E-01
GO:0033005	positive regulation of mast cell activation	21	1	2.06E-01
GO:0035812	renal sodium excretion	21	1	2.06E-01
GO:0042790	nucleolar large rRNA transcription by RNA polymerase I	21	1	2.06E-01

GO:0043373	CD4-positive, alpha-beta T cell lineage commitment	21	1	2.06E-01
GO:0043555	regulation of translation in response to stress	21	1	2.06E-01
GO:0043984	histone H4-K16 acetylation	21	1	2.06E-01
GO:0044090	positive regulation of vacuole organization	21	1	2.06E-01
GO:0045056	transcytosis	21	1	2.06E-01
GO:0045063	T-helper 1 cell differentiation	21	1	2.06E-01
GO:0050849	negative regulation of calcium-mediated signaling	21	1	2.06E-01
GO:0051315	attachment of mitotic spindle microtubules to kinetocho	21	1	2.06E-01
GO:0060973	cell migration involved in heart development	21	1	2.06E-01
GO:0061050	regulation of cell growth involved in cardiac muscle cell	21	1	2.06E-01
GO:0071605	monocyte chemotactic protein-1 production	21	1	2.06E-01
GO:0071636	positive regulation of transforming growth factor beta p	21	1	2.06E-01
GO:0071637	regulation of monocyte chemotactic protein-1 productio	21	1	2.06E-01
GO:0090026	positive regulation of monocyte chemotaxis	21	1	2.06E-01
GO:0097049	motor neuron apoptotic process	21	1	2.06E-01
GO:0106030	neuron projection fasciculation	21	1	2.06E-01
GO:2000047	regulation of cell-cell adhesion mediated by cadherin	21	1	2.06E-01
GO:0005753	mitochondrial proton-transporting ATP synthase comple	21	1	2.06E-01
GO:0005865	striated muscle thin filament	21	1	2.06E-01
GO:0016327	apicolateral plasma membrane	21	1	2.06E-01
GO:0031681	G-protein beta-subunit binding	21	1	2.06E-01
GO:0007272	ensheathment of neurons	144	3	2.08E-01
GO:0008366	axon ensheathment	144	3	2.08E-01
GO:0090049	regulation of cell migration involved in sprouting angiog	78	2	2.09E-01
GO:0140115	export across plasma membrane	78	2	2.09E-01
GO:1901264	carbohydrate derivative transport	78	2	2.09E-01
GO:1902475	L-alpha-amino acid transmembrane transport	78	2	2.09E-01
GO:0000792	heterochromatin	78	2	2.09E-01
GO:0008233	peptidase activity	601	9	2.11E-01
GO:0002292	T cell differentiation involved in immune response	79	2	2.13E-01
GO:0050671	positive regulation of lymphocyte proliferation	146	3	2.14E-01
GO:0050768	negative regulation of neurogenesis	146	3	2.14E-01
GO:0001919	regulation of receptor recycling	22	1	2.14E-01
GO:0006907	pinocytosis	22	1	2.14E-01
GO:0007039	protein catabolic process in the vacuole	22	1	2.14E-01
GO:0007342	fusion of sperm to egg plasma membrane involved in sir	22	1	2.14E-01
GO:0009127	purine nucleoside monophosphate biosynthetic process	22	1	2.14E-01
GO:0010042	response to manganese ion	22	1	2.14E-01
GO:0010881	regulation of cardiac muscle contraction by regulation o	22	1	2.14E-01
GO:0010888	negative regulation of lipid storage	22	1	2.14E-01
GO:0010988	regulation of low-density lipoprotein particle clearance	22	1	2.14E-01
GO:0015874	norepinephrine transport	22	1	2.14E-01
GO:0030449	regulation of complement activation	22	1	2.14E-01
GO:0032069	regulation of nuclease activity	22	1	2.14E-01
GO:0032288	myelin assembly	22	1	2.14E-01
GO:0032516	positive regulation of phosphoprotein phosphatase activ	22	1	2.14E-01
GO:0032780	negative regulation of ATP-dependent activity	22	1	2.14E-01

GO:0032785	negative regulation of DNA-templated transcription, elo	22	1	2.14E-01
GO:0034104	negative regulation of tissue remodeling	22	1	2.14E-01
GO:0035313	wound healing, spreading of epidermal cells	22	1	2.14E-01
GO:0035809	regulation of urine volume	22	1	2.14E-01
GO:0036344	platelet morphogenesis	22	1	2.14E-01
GO:0044062	regulation of excretion	22	1	2.14E-01
GO:0044546	NLRP3 inflammasome complex assembly	22	1	2.14E-01
GO:0046597	negative regulation of viral entry into host cell	22	1	2.14E-01
GO:0046628	positive regulation of insulin receptor signaling pathway	22	1	2.14E-01
GO:0050765	negative regulation of phagocytosis	22	1	2.14E-01
GO:0051000	positive regulation of nitric-oxide synthase activity	22	1	2.14E-01
GO:0051767	nitric-oxide synthase biosynthetic process	22	1	2.14E-01
GO:0051769	regulation of nitric-oxide synthase biosynthetic process	22	1	2.14E-01
GO:0060117	auditory receptor cell development	22	1	2.14E-01
GO:0070886	positive regulation of calcineurin-NFAT signaling cascade	22	1	2.14E-01
GO:0072111	cell proliferation involved in kidney development	22	1	2.14E-01
GO:0090042	tubulin deacetylation	22	1	2.14E-01
GO:0098743	cell aggregation	22	1	2.14E-01
GO:0106058	positive regulation of calcineurin-mediated signaling	22	1	2.14E-01
GO:1903209	positive regulation of oxidative stress-induced cell death	22	1	2.14E-01
GO:1903428	positive regulation of reactive oxygen species biosynthe	22	1	2.14E-01
GO:2000810	regulation of bicellular tight junction assembly	22	1	2.14E-01
GO:2001044	regulation of integrin-mediated signaling pathway	22	1	2.14E-01
GO:0000242	pericentriolar material	22	1	2.14E-01
GO:0005742	mitochondrial outer membrane translocase complex	22	1	2.14E-01
GO:0045259	proton-transporting ATP synthase complex	22	1	2.14E-01
GO:1990391	DNA repair complex	22	1	2.14E-01
GO:0030742	GTP-dependent protein binding	22	1	2.14E-01
GO:0033691	sialic acid binding	22	1	2.14E-01
GO:0070182	DNA polymerase binding	22	1	2.14E-01
GO:0120020	cholesterol transfer activity	22	1	2.14E-01
GO:0070997	neuron death	368	6	2.15E-01
GO:0046483	heterocycle metabolic process	6336	75	2.15E-01
GO:0072330	monocarboxylic acid biosynthetic process	218	4	2.15E-01
GO:0051592	response to calcium ion	147	3	2.16E-01
GO:0003151	outflow tract morphogenesis	80	2	2.17E-01
GO:1901184	regulation of ERBB signaling pathway	80	2	2.17E-01
GO:0016835	carbon-oxygen lyase activity	80	2	2.17E-01
GO:0050804	modulation of chemical synaptic transmission	448	7	2.18E-01
GO:0032147	activation of protein kinase activity	148	3	2.19E-01
GO:0009152	purine ribonucleotide biosynthetic process	220	4	2.20E-01
GO:0001818	negative regulation of cytokine production	371	6	2.20E-01
GO:0099177	regulation of trans-synaptic signaling	449	7	2.20E-01
GO:0090407	organophosphate biosynthetic process	608	9	2.21E-01
GO:0002200	somatic diversification of immune receptors	81	2	2.21E-01
GO:0007422	peripheral nervous system development	81	2	2.21E-01
GO:0032371	regulation of sterol transport	81	2	2.21E-01

GO:0032374	regulation of cholesterol transport	81	2	2.21E-01
GO:0097061	dendritic spine organization	81	2	2.21E-01
GO:0043161	proteasome-mediated ubiquitin-dependent protein cata	450	7	2.22E-01
GO:0050866	negative regulation of cell activation	221	4	2.22E-01
GO:0032946	positive regulation of mononuclear cell proliferation	149	3	2.22E-01
GO:0050796	regulation of insulin secretion	149	3	2.22E-01
GO:0051053	negative regulation of DNA metabolic process	149	3	2.22E-01
GO:1902652	secondary alcohol metabolic process	149	3	2.22E-01
GO:0002082	regulation of oxidative phosphorylation	23	1	2.23E-01
GO:0006353	DNA-templated transcription termination	23	1	2.23E-01
GO:0035024	negative regulation of Rho protein signal transduction	23	1	2.23E-01
GO:0040019	positive regulation of embryonic development	23	1	2.23E-01
GO:0043968	histone H2A acetylation	23	1	2.23E-01
GO:0044241	lipid digestion	23	1	2.23E-01
GO:0045540	regulation of cholesterol biosynthetic process	23	1	2.23E-01
GO:0046599	regulation of centriole replication	23	1	2.23E-01
GO:0046823	negative regulation of nucleocytoplasmic transport	23	1	2.23E-01
GO:0048143	astrocyte activation	23	1	2.23E-01
GO:0051457	maintenance of protein location in nucleus	23	1	2.23E-01
GO:0072677	eosinophil migration	23	1	2.23E-01
GO:0090335	regulation of brown fat cell differentiation	23	1	2.23E-01
GO:0098856	intestinal lipid absorption	23	1	2.23E-01
GO:0106118	regulation of sterol biosynthetic process	23	1	2.23E-01
GO:0140747	regulation of ncRNA transcription	23	1	2.23E-01
GO:2000178	negative regulation of neural precursor cell proliferation	23	1	2.23E-01
GO:2001169	regulation of ATP biosynthetic process	23	1	2.23E-01
GO:0098799	outer mitochondrial membrane protein complex	23	1	2.23E-01
GO:0005212	structural constituent of eye lens	23	1	2.23E-01
GO:0017116	single-stranded DNA helicase activity	23	1	2.23E-01
GO:0019211	phosphatase activator activity	23	1	2.23E-01
GO:0120015	sterol transfer activity	23	1	2.23E-01
GO:0009314	response to radiation	452	7	2.25E-01
GO:1905897	regulation of response to endoplasmic reticulum stress	82	2	2.25E-01
GO:2000736	regulation of stem cell differentiation	82	2	2.25E-01
GO:0030165	PDZ domain binding	82	2	2.25E-01
GO:0015631	tubulin binding	375	6	2.27E-01
GO:1901360	organic cyclic compound metabolic process	6632	78	2.27E-01
GO:0051961	negative regulation of nervous system development	151	3	2.28E-01
GO:1903531	negative regulation of secretion by cell	151	3	2.28E-01
GO:0043087	regulation of GTPase activity	376	6	2.28E-01
GO:0030500	regulation of bone mineralization	83	2	2.29E-01
GO:0055013	cardiac muscle cell development	83	2	2.29E-01
GO:0061912	selective autophagy	83	2	2.29E-01
GO:0140029	exocytic process	83	2	2.29E-01
GO:0050767	regulation of neurogenesis	377	6	2.30E-01
GO:0030183	B cell differentiation	152	3	2.31E-01
GO:0072006	nephron development	152	3	2.31E-01

GO:0016055	Wnt signaling pathway	456	7	2.31E-01
GO:0002755	MyD88-dependent toll-like receptor signaling pathway	24	1	2.31E-01
GO:0006925	inflammatory cell apoptotic process	24	1	2.31E-01
GO:0007097	nuclear migration	24	1	2.31E-01
GO:0010623	programmed cell death involved in cell development	24	1	2.31E-01
GO:0031280	negative regulation of cyclase activity	24	1	2.31E-01
GO:0032528	microvillus organization	24	1	2.31E-01
GO:0035988	chondrocyte proliferation	24	1	2.31E-01
GO:0036010	protein localization to endosome	24	1	2.31E-01
GO:0036120	cellular response to platelet-derived growth factor stimu	24	1	2.31E-01
GO:0038095	Fc-epsilon receptor signaling pathway	24	1	2.31E-01
GO:0040037	negative regulation of fibroblast growth factor receptor	24	1	2.31E-01
GO:0043369	CD4-positive or CD8-positive, alpha-beta T cell lineage c	24	1	2.31E-01
GO:0051590	positive regulation of neurotransmitter transport	24	1	2.31E-01
GO:0060352	cell adhesion molecule production	24	1	2.31E-01
GO:0060907	positive regulation of macrophage cytokine production	24	1	2.31E-01
GO:0070233	negative regulation of T cell apoptotic process	24	1	2.31E-01
GO:0070841	inclusion body assembly	24	1	2.31E-01
GO:0070935	3'-UTR-mediated mRNA stabilization	24	1	2.31E-01
GO:0140632	inflammasome complex assembly	24	1	2.31E-01
GO:1901386	negative regulation of voltage-gated calcium channel ac	24	1	2.31E-01
GO:1901863	positive regulation of muscle tissue development	24	1	2.31E-01
GO:1902410	mitotic cytokinetic process	24	1	2.31E-01
GO:1903241	U2-type prespliceosome assembly	24	1	2.31E-01
GO:1903792	negative regulation of anion transport	24	1	2.31E-01
GO:2000353	positive regulation of endothelial cell apoptotic process	24	1	2.31E-01
GO:2000637	positive regulation of miRNA-mediated gene silencing	24	1	2.31E-01
GO:2001026	regulation of endothelial cell chemotaxis	24	1	2.31E-01
GO:0032982	myosin filament	24	1	2.31E-01
GO:0004622	lysophospholipase activity	24	1	2.31E-01
GO:0015252	proton channel activity	24	1	2.31E-01
GO:0016702	oxidoreductase activity, acting on single donors with inc	24	1	2.31E-01
GO:0019865	immunoglobulin binding	24	1	2.31E-01
GO:0042625	ATPase-coupled ion transmembrane transporter activity	24	1	2.31E-01
GO:0044769	ATPase activity, coupled to transmembrane movement	24	1	2.31E-01
GO:0046961	proton-transporting ATPase activity, rotational mechani	24	1	2.31E-01
GO:0140358	P-type transmembrane transporter activity	24	1	2.31E-01
GO:0097060	synaptic membrane	378	6	2.32E-01
GO:0015844	monoamine transport	84	2	2.33E-01
GO:0032418	lysosome localization	84	2	2.33E-01
GO:0097006	regulation of plasma lipoprotein particle levels	84	2	2.33E-01
GO:0140056	organelle localization by membrane tethering	84	2	2.33E-01
GO:1990849	vacuolar localization	84	2	2.33E-01
GO:0010494	cytoplasmic stress granule	84	2	2.33E-01
GO:0070382	exocytic vesicle	226	4	2.34E-01
GO:0005795	Golgi stack	153	3	2.34E-01
GO:0198738	cell-cell signaling by wnt	458	7	2.34E-01

GO:1903050	regulation of proteolysis involved in protein catabolic pr	227	4	2.36E-01
GO:0008509	anion transmembrane transporter activity	303	5	2.36E-01
GO:0007584	response to nutrient	154	3	2.36E-01
GO:0016125	sterol metabolic process	154	3	2.36E-01
GO:0043204	perikaryon	154	3	2.36E-01
GO:0001540	amyloid-beta binding	85	2	2.37E-01
GO:0021700	developmental maturation	304	5	2.38E-01
GO:0001501	skeletal system development	541	8	2.39E-01
GO:0099699	integral component of synaptic membrane	155	3	2.39E-01
GO:0003071	renal system process involved in regulation of systemic a	25	1	2.40E-01
GO:0003081	regulation of systemic arterial blood pressure by renin-a	25	1	2.40E-01
GO:0008211	glucocorticoid metabolic process	25	1	2.40E-01
GO:0021801	cerebral cortex radial glia-guided migration	25	1	2.40E-01
GO:0022030	telencephalon glial cell migration	25	1	2.40E-01
GO:0032469	endoplasmic reticulum calcium ion homeostasis	25	1	2.40E-01
GO:0046033	AMP metabolic process	25	1	2.40E-01
GO:0046039	GTP metabolic process	25	1	2.40E-01
GO:0046641	positive regulation of alpha-beta T cell proliferation	25	1	2.40E-01
GO:0050860	negative regulation of T cell receptor signaling pathway	25	1	2.40E-01
GO:0051560	mitochondrial calcium ion homeostasis	25	1	2.40E-01
GO:0060148	positive regulation of post-transcriptional gene silencing	25	1	2.40E-01
GO:0070423	nucleotide-binding oligomerization domain containing si	25	1	2.40E-01
GO:1900078	positive regulation of cellular response to insulin stimulu	25	1	2.40E-01
GO:1900370	positive regulation of post-transcriptional gene silencing	25	1	2.40E-01
GO:2000114	regulation of establishment of cell polarity	25	1	2.40E-01
GO:2000209	regulation of anoikis	25	1	2.40E-01
GO:0036379	myofilament	25	1	2.40E-01
GO:0044322	endoplasmic reticulum quality control compartment	25	1	2.40E-01
GO:0001091	RNA polymerase II general transcription initiation factor	25	1	2.40E-01
GO:0016701	oxidoreductase activity, acting on single donors with inc	25	1	2.40E-01
GO:0016769	transferase activity, transferring nitrogenous groups	25	1	2.40E-01
GO:0120014	phospholipid transfer activity	25	1	2.40E-01
GO:0071902	positive regulation of protein serine/threonine kinase ac	229	4	2.41E-01
GO:0019935	cyclic-nucleotide-mediated signaling	86	2	2.41E-01
GO:0033077	T cell differentiation in thymus	86	2	2.41E-01
GO:0045921	positive regulation of exocytosis	86	2	2.41E-01
GO:0034728	nucleosome organization	156	3	2.42E-01
GO:0002790	peptide secretion	230	4	2.43E-01
GO:0006396	RNA processing	1460	19	2.44E-01
GO:0010976	positive regulation of neuron projection development	157	3	2.45E-01
GO:0044839	cell cycle G2/M phase transition	157	3	2.45E-01
GO:0001523	retinoid metabolic process	87	2	2.45E-01
GO:0019915	lipid storage	87	2	2.45E-01
GO:0050672	negative regulation of lymphocyte proliferation	87	2	2.45E-01
GO:0099175	regulation of postsynapse organization	87	2	2.45E-01
GO:0005876	spindle microtubule	87	2	2.45E-01
GO:0008013	beta-catenin binding	87	2	2.45E-01

GO:0016651	oxidoreductase activity, acting on NAD(P)H	87	2	2.45E-01
GO:0008285	negative regulation of cell population proliferation	790	11	2.45E-01
GO:0061134	peptidase regulator activity	232	4	2.48E-01
GO:0006706	steroid catabolic process	26	1	2.48E-01
GO:0006929	substrate-dependent cell migration	26	1	2.48E-01
GO:0010882	regulation of cardiac muscle contraction by calcium ion s	26	1	2.48E-01
GO:0030810	positive regulation of nucleotide biosynthetic process	26	1	2.48E-01
GO:0032515	negative regulation of phosphoprotein phosphatase acti	26	1	2.48E-01
GO:0033598	mammary gland epithelial cell proliferation	26	1	2.48E-01
GO:0035872	nucleotide-binding domain, leucine rich repeat containir	26	1	2.48E-01
GO:0036119	response to platelet-derived growth factor	26	1	2.48E-01
GO:0045026	plasma membrane fusion	26	1	2.48E-01
GO:0045822	negative regulation of heart contraction	26	1	2.48E-01
GO:0050857	positive regulation of antigen receptor-mediated signali	26	1	2.48E-01
GO:0051894	positive regulation of focal adhesion assembly	26	1	2.48E-01
GO:0051957	positive regulation of amino acid transport	26	1	2.48E-01
GO:0072010	glomerular epithelium development	26	1	2.48E-01
GO:0099590	neurotransmitter receptor internalization	26	1	2.48E-01
GO:1900373	positive regulation of purine nucleotide biosynthetic pro	26	1	2.48E-01
GO:1903020	positive regulation of glycoprotein metabolic process	26	1	2.48E-01
GO:1903588	negative regulation of blood vessel endothelial cell proli	26	1	2.48E-01
GO:1904753	negative regulation of vascular associated smooth musc	26	1	2.48E-01
GO:1905048	regulation of metallopeptidase activity	26	1	2.48E-01
GO:0031045	dense core granule	26	1	2.48E-01
GO:0001848	complement binding	26	1	2.48E-01
GO:0004190	aspartic-type endopeptidase activity	26	1	2.48E-01
GO:0004364	glutathione transferase activity	26	1	2.48E-01
GO:0004435	phosphatidylinositol phospholipase C activity	26	1	2.48E-01
GO:0017025	TBP-class protein binding	26	1	2.48E-01
GO:0030507	spectrin binding	26	1	2.48E-01
GO:0034593	phosphatidylinositol bisphosphate phosphatase activity	26	1	2.48E-01
GO:0070006	metalloaminopeptidase activity	26	1	2.48E-01
GO:0140597	protein carrier chaperone	26	1	2.48E-01
GO:0005740	mitochondrial envelope	793	11	2.49E-01
GO:0002312	B cell activation involved in immune response	88	2	2.49E-01
GO:0032370	positive regulation of lipid transport	88	2	2.49E-01
GO:0032945	negative regulation of mononuclear cell proliferation	88	2	2.49E-01
GO:1901879	regulation of protein depolymerization	88	2	2.49E-01
GO:0060560	developmental growth involved in morphogenesis	233	4	2.50E-01
GO:0009260	ribonucleotide biosynthetic process	234	4	2.52E-01
GO:0006650	glycerophospholipid metabolic process	311	5	2.53E-01
GO:0048678	response to axon injury	89	2	2.53E-01
GO:0016241	regulation of macroautophagy	160	3	2.54E-01
GO:0120254	olefinic compound metabolic process	160	3	2.54E-01
GO:0001963	synaptic transmission, dopaminergic	27	1	2.56E-01
GO:0002407	dendritic cell chemotaxis	27	1	2.56E-01
GO:0006760	folic acid-containing compound metabolic process	27	1	2.56E-01

GO:0010875	positive regulation of cholesterol efflux	27	1	2.56E-01
GO:0010884	positive regulation of lipid storage	27	1	2.56E-01
GO:0014829	vascular associated smooth muscle contraction	27	1	2.56E-01
GO:0032682	negative regulation of chemokine production	27	1	2.56E-01
GO:0032753	positive regulation of interleukin-4 production	27	1	2.56E-01
GO:0032967	positive regulation of collagen biosynthetic process	27	1	2.56E-01
GO:0033081	regulation of T cell differentiation in thymus	27	1	2.56E-01
GO:0035774	positive regulation of insulin secretion involved in cellula	27	1	2.56E-01
GO:0036037	CD8-positive, alpha-beta T cell activation	27	1	2.56E-01
GO:0042059	negative regulation of epidermal growth factor receptor	27	1	2.56E-01
GO:0045116	protein neddylation	27	1	2.56E-01
GO:0045745	positive regulation of G protein-coupled receptor signali	27	1	2.56E-01
GO:0045954	positive regulation of natural killer cell mediated cytotox	27	1	2.56E-01
GO:0046037	GMP metabolic process	27	1	2.56E-01
GO:0048641	regulation of skeletal muscle tissue development	27	1	2.56E-01
GO:0051350	negative regulation of lyase activity	27	1	2.56E-01
GO:0051503	adenine nucleotide transport	27	1	2.56E-01
GO:0051571	positive regulation of histone H3-K4 methylation	27	1	2.56E-01
GO:0051953	negative regulation of amine transport	27	1	2.56E-01
GO:0070861	regulation of protein exit from endoplasmic reticulum	27	1	2.56E-01
GO:0071880	adenylate cyclase-activating adrenergic receptor signalir	27	1	2.56E-01
GO:1903055	positive regulation of extracellular matrix organization	27	1	2.56E-01
GO:1903523	negative regulation of blood circulation	27	1	2.56E-01
GO:0035267	NuA4 histone acetyltransferase complex	27	1	2.56E-01
GO:0043189	H4/H2A histone acetyltransferase complex	27	1	2.56E-01
GO:0009678	pyrophosphate hydrolysis-driven proton transmembrane	27	1	2.56E-01
GO:0016405	CoA-ligase activity	27	1	2.56E-01
GO:0070001	aspartic-type peptidase activity	27	1	2.56E-01
GO:0046434	organophosphate catabolic process	161	3	2.57E-01
GO:0005319	lipid transporter activity	161	3	2.57E-01
GO:0016101	diterpenoid metabolic process	90	2	2.57E-01
GO:0046364	monosaccharide biosynthetic process	90	2	2.57E-01
GO:0051591	response to cAMP	90	2	2.57E-01
GO:0055006	cardiac cell development	90	2	2.57E-01
GO:0070098	chemokine-mediated signaling pathway	90	2	2.57E-01
GO:0043178	alcohol binding	90	2	2.57E-01
GO:0001701	in utero embryonic development	393	6	2.59E-01
GO:0005539	glycosaminoglycan binding	237	4	2.59E-01
GO:0007034	vacuolar transport	162	3	2.60E-01
GO:0046488	phosphatidylinositol metabolic process	162	3	2.60E-01
GO:1901615	organic hydroxy compound metabolic process	555	8	2.61E-01
GO:0031667	response to nutrient levels	474	7	2.61E-01
GO:0046928	regulation of neurotransmitter secretion	91	2	2.61E-01
GO:0106027	neuron projection organization	91	2	2.61E-01
GO:2000117	negative regulation of cysteine-type endopeptidase acti	91	2	2.61E-01
GO:0000123	histone acetyltransferase complex	91	2	2.61E-01
GO:0005253	anion channel activity	91	2	2.61E-01

GO:0006139	nucleobase-containing compound metabolic process	6165	72	2.64E-01
GO:0051252	regulation of RNA metabolic process	3754	45	2.64E-01
GO:0051403	stress-activated MAPK cascade	239	4	2.64E-01
GO:0002052	positive regulation of neuroblast proliferation	28	1	2.64E-01
GO:0007190	activation of adenylate cyclase activity	28	1	2.64E-01
GO:0007214	gamma-aminobutyric acid signaling pathway	28	1	2.64E-01
GO:0010714	positive regulation of collagen metabolic process	28	1	2.64E-01
GO:0010894	negative regulation of steroid biosynthetic process	28	1	2.64E-01
GO:0010971	positive regulation of G2/M transition of mitotic cell cyc	28	1	2.64E-01
GO:0015868	purine ribonucleotide transport	28	1	2.64E-01
GO:0032801	receptor catabolic process	28	1	2.64E-01
GO:0032878	regulation of establishment or maintenance of cell polar	28	1	2.64E-01
GO:0032958	inositol phosphate biosynthetic process	28	1	2.64E-01
GO:0042634	regulation of hair cycle	28	1	2.64E-01
GO:0044331	cell-cell adhesion mediated by cadherin	28	1	2.64E-01
GO:0045830	positive regulation of isotype switching	28	1	2.64E-01
GO:0046337	phosphatidylethanolamine metabolic process	28	1	2.64E-01
GO:0051647	nucleus localization	28	1	2.64E-01
GO:0090314	positive regulation of protein targeting to membrane	28	1	2.64E-01
GO:1903649	regulation of cytoplasmic transport	28	1	2.64E-01
GO:1903671	negative regulation of sprouting angiogenesis	28	1	2.64E-01
GO:1903672	positive regulation of sprouting angiogenesis	28	1	2.64E-01
GO:1903779	regulation of cardiac conduction	28	1	2.64E-01
GO:2000819	regulation of nucleotide-excision repair	28	1	2.64E-01
GO:0032585	multivesicular body membrane	28	1	2.64E-01
GO:0051019	mitogen-activated protein kinase binding	28	1	2.64E-01
GO:0070273	phosphatidylinositol-4-phosphate binding	28	1	2.64E-01
GO:0002028	regulation of sodium ion transport	92	2	2.65E-01
GO:0060996	dendritic spine development	92	2	2.65E-01
GO:0090277	positive regulation of peptide hormone secretion	92	2	2.65E-01
GO:0006633	fatty acid biosynthetic process	164	3	2.66E-01
GO:0007519	skeletal muscle tissue development	164	3	2.66E-01
GO:0006835	dicarboxylic acid transport	93	2	2.69E-01
GO:0044070	regulation of anion transport	93	2	2.69E-01
GO:0051781	positive regulation of cell division	93	2	2.69E-01
GO:1904705	regulation of vascular associated smooth muscle cell pro	93	2	2.69E-01
GO:2000106	regulation of leukocyte apoptotic process	93	2	2.69E-01
GO:0006164	purine nucleotide biosynthetic process	242	4	2.71E-01
GO:0051250	negative regulation of lymphocyte activation	166	3	2.72E-01
GO:0099240	intrinsic component of synaptic membrane	166	3	2.72E-01
GO:0006631	fatty acid metabolic process	400	6	2.72E-01
GO:0008053	mitochondrial fusion	29	1	2.73E-01
GO:0010804	negative regulation of tumor necrosis factor-mediated s	29	1	2.73E-01
GO:0010955	negative regulation of protein processing	29	1	2.73E-01
GO:0015865	purine nucleotide transport	29	1	2.73E-01
GO:0019076	viral release from host cell	29	1	2.73E-01
GO:0030970	retrograde protein transport, ER to cytosol	29	1	2.73E-01

GO:0035891	exit from host cell	29	1	2.73E-01
GO:0042759	long-chain fatty acid biosynthetic process	29	1	2.73E-01
GO:0048265	response to pain	29	1	2.73E-01
GO:0090025	regulation of monocyte chemotaxis	29	1	2.73E-01
GO:0097254	renal tubular secretion	29	1	2.73E-01
GO:0099623	regulation of cardiac muscle cell membrane repolarizati	29	1	2.73E-01
GO:1903318	negative regulation of protein maturation	29	1	2.73E-01
GO:1903513	endoplasmic reticulum to cytosol transport	29	1	2.73E-01
GO:0005686	U2 snRNP	29	1	2.73E-01
GO:0036019	endolysosome	29	1	2.73E-01
GO:0048787	presynaptic active zone membrane	29	1	2.73E-01
GO:0003785	actin monomer binding	29	1	2.73E-01
GO:0004181	metallocarboxypeptidase activity	29	1	2.73E-01
GO:0004629	phospholipase C activity	29	1	2.73E-01
GO:0043325	phosphatidylinositol-3,4-bisphosphate binding	29	1	2.73E-01
GO:0046625	sphingolipid binding	29	1	2.73E-01
GO:0070325	lipoprotein particle receptor binding	29	1	2.73E-01
GO:0022414	reproductive process	1491	19	2.73E-01
GO:0031970	organelle envelope lumen	94	2	2.73E-01
GO:0031514	motile cilium	244	4	2.76E-01
GO:0008202	steroid metabolic process	323	5	2.77E-01
GO:1990874	vascular associated smooth muscle cell proliferation	95	2	2.77E-01
GO:0031225	anchored component of membrane	169	3	2.80E-01
GO:0007202	activation of phospholipase C activity	30	1	2.80E-01
GO:0008045	motor neuron axon guidance	30	1	2.80E-01
GO:0016226	iron-sulfur cluster assembly	30	1	2.80E-01
GO:0031163	metallo-sulfur cluster assembly	30	1	2.80E-01
GO:0034067	protein localization to Golgi apparatus	30	1	2.80E-01
GO:0039694	viral RNA genome replication	30	1	2.80E-01
GO:0070168	negative regulation of biomineral tissue development	30	1	2.80E-01
GO:0070979	protein K11-linked ubiquitination	30	1	2.80E-01
GO:1902914	regulation of protein polyubiquitination	30	1	2.80E-01
GO:2000108	positive regulation of leukocyte apoptotic process	30	1	2.80E-01
GO:0005790	smooth endoplasmic reticulum	30	1	2.80E-01
GO:0016514	SWI/SNF complex	30	1	2.80E-01
GO:0030125	clathrin vesicle coat	30	1	2.80E-01
GO:0030296	protein tyrosine kinase activator activity	30	1	2.80E-01
GO:0043548	phosphatidylinositol 3-kinase binding	30	1	2.80E-01
GO:0007267	cell-cell signaling	1672	21	2.81E-01
GO:0032838	plasma membrane bounded cell projection cytoplasm	246	4	2.81E-01
GO:0000003	reproduction	1500	19	2.81E-01
GO:0061097	regulation of protein tyrosine kinase activity	96	2	2.82E-01
GO:0008610	lipid biosynthetic process	735	10	2.82E-01
GO:0006629	lipid metabolic process	1415	18	2.82E-01
GO:0008593	regulation of Notch signaling pathway	97	2	2.86E-01
GO:2001251	negative regulation of chromosome organization	97	2	2.86E-01
GO:0000932	P-body	97	2	2.86E-01

GO:0001750	photoreceptor outer segment	97	2	2.86E-01
GO:0007188	adenylate cyclase-modulating G protein-coupled recepto	248	4	2.86E-01
GO:0003823	antigen binding	171	3	2.86E-01
GO:0008201	heparin binding	171	3	2.86E-01
GO:0000413	protein peptidyl-prolyl isomerization	31	1	2.88E-01
GO:0002220	innate immune response activating cell surface receptor	31	1	2.88E-01
GO:0002230	positive regulation of defense response to virus by host	31	1	2.88E-01
GO:0002507	tolerance induction	31	1	2.88E-01
GO:0003298	physiological muscle hypertrophy	31	1	2.88E-01
GO:0003301	physiological cardiac muscle hypertrophy	31	1	2.88E-01
GO:0006783	heme biosynthetic process	31	1	2.88E-01
GO:0031069	hair follicle morphogenesis	31	1	2.88E-01
GO:0032148	activation of protein kinase B activity	31	1	2.88E-01
GO:0035767	endothelial cell chemotaxis	31	1	2.88E-01
GO:0045939	negative regulation of steroid metabolic process	31	1	2.88E-01
GO:0045948	positive regulation of translational initiation	31	1	2.88E-01
GO:0045987	positive regulation of smooth muscle contraction	31	1	2.88E-01
GO:0048679	regulation of axon regeneration	31	1	2.88E-01
GO:0055022	negative regulation of cardiac muscle tissue growth	31	1	2.88E-01
GO:0060055	angiogenesis involved in wound healing	31	1	2.88E-01
GO:0061049	cell growth involved in cardiac muscle cell development	31	1	2.88E-01
GO:0061117	negative regulation of heart growth	31	1	2.88E-01
GO:0110150	negative regulation of biomineralization	31	1	2.88E-01
GO:0150117	positive regulation of cell-substrate junction organizatio	31	1	2.88E-01
GO:1901185	negative regulation of ERBB signaling pathway	31	1	2.88E-01
GO:0000803	sex chromosome	31	1	2.88E-01
GO:0016342	catenin complex	31	1	2.88E-01
GO:0016878	acid-thiol ligase activity	31	1	2.88E-01
GO:0022829	wide pore channel activity	31	1	2.88E-01
GO:0031210	phosphatidylcholine binding	31	1	2.88E-01
GO:0042910	xenobiotic transmembrane transporter activity	31	1	2.88E-01
GO:0000922	spindle pole	172	3	2.89E-01
GO:0032642	regulation of chemokine production	98	2	2.90E-01
GO:0051952	regulation of amine transport	98	2	2.90E-01
GO:0042626	ATPase-coupled transmembrane transporter activity	98	2	2.90E-01
GO:0072522	purine-containing compound biosynthetic process	251	4	2.93E-01
GO:0032602	chemokine production	99	2	2.94E-01
GO:0005604	basement membrane	99	2	2.94E-01
GO:0030527	structural constituent of chromatin	99	2	2.94E-01
GO:0031966	mitochondrial membrane	744	10	2.95E-01
GO:0046631	alpha-beta T cell activation	174	3	2.95E-01
GO:0010951	negative regulation of endopeptidase activity	252	4	2.96E-01
GO:0050851	antigen receptor-mediated signaling pathway	252	4	2.96E-01
GO:0002717	positive regulation of natural killer cell mediated immun	32	1	2.96E-01
GO:0002758	innate immune response-activating signal transduction	32	1	2.96E-01
GO:0006335	DNA replication-dependent chromatin assembly	32	1	2.96E-01
GO:0006884	cell volume homeostasis	32	1	2.96E-01

GO:0010165	response to X-ray	32	1	2.96E-01
GO:0019082	viral protein processing	32	1	2.96E-01
GO:0031116	positive regulation of microtubule polymerization	32	1	2.96E-01
GO:0032770	positive regulation of monooxygenase activity	32	1	2.96E-01
GO:0033198	response to ATP	32	1	2.96E-01
GO:0034389	lipid droplet organization	32	1	2.96E-01
GO:0034508	centromere complex assembly	32	1	2.96E-01
GO:0043304	regulation of mast cell degranulation	32	1	2.96E-01
GO:0043552	positive regulation of phosphatidylinositol 3-kinase activ	32	1	2.96E-01
GO:0046825	regulation of protein export from nucleus	32	1	2.96E-01
GO:0050482	arachidonic acid secretion	32	1	2.96E-01
GO:0051443	positive regulation of ubiquitin-protein transferase activ	32	1	2.96E-01
GO:1903715	regulation of aerobic respiration	32	1	2.96E-01
GO:1903963	arachidonate transport	32	1	2.96E-01
GO:1990776	response to angiotensin	32	1	2.96E-01
GO:0005921	gap junction	32	1	2.96E-01
GO:0030515	snoRNA binding	32	1	2.96E-01
GO:0048019	receptor antagonist activity	32	1	2.96E-01
GO:0050699	WW domain binding	32	1	2.96E-01
GO:0051048	negative regulation of secretion	175	3	2.98E-01
GO:0015833	peptide transport	254	4	3.01E-01
GO:0022900	electron transport chain	176	3	3.01E-01
GO:0060538	skeletal muscle organ development	176	3	3.01E-01
GO:0006721	terpenoid metabolic process	101	2	3.02E-01
GO:1902476	chloride transmembrane transport	101	2	3.02E-01
GO:0031248	protein acetyltransferase complex	101	2	3.02E-01
GO:0032588	trans-Golgi network membrane	101	2	3.02E-01
GO:1902493	acetyltransferase complex	101	2	3.02E-01
GO:0005496	steroid binding	101	2	3.02E-01
GO:0051402	neuron apoptotic process	255	4	3.03E-01
GO:0000132	establishment of mitotic spindle orientation	33	1	3.04E-01
GO:0001881	receptor recycling	33	1	3.04E-01
GO:0030511	positive regulation of transforming growth factor beta re	33	1	3.04E-01
GO:0030947	regulation of vascular endothelial growth factor recepto	33	1	3.04E-01
GO:0033032	regulation of myeloid cell apoptotic process	33	1	3.04E-01
GO:0036475	neuron death in response to oxidative stress	33	1	3.04E-01
GO:0042219	cellular modified amino acid catabolic process	33	1	3.04E-01
GO:0045577	regulation of B cell differentiation	33	1	3.04E-01
GO:0045907	positive regulation of vasoconstriction	33	1	3.04E-01
GO:0045943	positive regulation of transcription by RNA polymerase I	33	1	3.04E-01
GO:0048147	negative regulation of fibroblast proliferation	33	1	3.04E-01
GO:0050858	negative regulation of antigen receptor-mediated signal	33	1	3.04E-01
GO:0051204	protein insertion into mitochondrial membrane	33	1	3.04E-01
GO:0060122	inner ear receptor cell stereocilium organization	33	1	3.04E-01
GO:0061842	microtubule organizing center localization	33	1	3.04E-01
GO:0070633	transepithelial transport	33	1	3.04E-01
GO:0071353	cellular response to interleukin-4	33	1	3.04E-01

GO:0071875	adrenergic receptor signaling pathway	33	1	3.04E-01
GO:0098801	regulation of renal system process	33	1	3.04E-01
GO:1903846	positive regulation of cellular response to transforming g	33	1	3.04E-01
GO:2000191	regulation of fatty acid transport	33	1	3.04E-01
GO:0070971	endoplasmic reticulum exit site	33	1	3.04E-01
GO:0030291	protein serine/threonine kinase inhibitor activity	33	1	3.04E-01
GO:0001764	neuron migration	177	3	3.04E-01
GO:0030111	regulation of Wnt signaling pathway	336	5	3.05E-01
GO:0018130	heterocycle biosynthetic process	4086	48	3.06E-01
GO:0050708	regulation of protein secretion	257	4	3.08E-01
GO:0045639	positive regulation of myeloid cell differentiation	103	2	3.10E-01
GO:0046620	regulation of organ growth	103	2	3.10E-01
GO:0050773	regulation of dendrite development	103	2	3.10E-01
GO:0070167	regulation of biomineral tissue development	103	2	3.10E-01
GO:0090276	regulation of peptide hormone secretion	179	3	3.10E-01
GO:0000289	nuclear-transcribed mRNA poly(A) tail shortening	34	1	3.11E-01
GO:0006891	intra-Golgi vesicle-mediated transport	34	1	3.11E-01
GO:0009156	ribonucleoside monophosphate biosynthetic process	34	1	3.11E-01
GO:0009649	entrainment of circadian clock	34	1	3.11E-01
GO:0010984	regulation of lipoprotein particle clearance	34	1	3.11E-01
GO:0021799	cerebral cortex radially oriented cell migration	34	1	3.11E-01
GO:0032743	positive regulation of interleukin-2 production	34	1	3.11E-01
GO:0033006	regulation of mast cell activation involved in immune res	34	1	3.11E-01
GO:0035025	positive regulation of Rho protein signal transduction	34	1	3.11E-01
GO:0035335	peptidyl-tyrosine dephosphorylation	34	1	3.11E-01
GO:0035590	purinergic nucleotide receptor signaling pathway	34	1	3.11E-01
GO:0042558	pteridine-containing compound metabolic process	34	1	3.11E-01
GO:0060317	cardiac epithelial to mesenchymal transition	34	1	3.11E-01
GO:0060563	neuroepithelial cell differentiation	34	1	3.11E-01
GO:0061081	positive regulation of myeloid leukocyte cytokine produ	34	1	3.11E-01
GO:0070570	regulation of neuron projection regeneration	34	1	3.11E-01
GO:0090313	regulation of protein targeting to membrane	34	1	3.11E-01
GO:1900181	negative regulation of protein localization to nucleus	34	1	3.11E-01
GO:0098686	hippocampal mossy fiber to CA3 synapse	34	1	3.11E-01
GO:1902555	endoribonuclease complex	34	1	3.11E-01
GO:0009991	response to extracellular stimulus	504	7	3.12E-01
GO:0004866	endopeptidase inhibitor activity	180	3	3.13E-01
GO:0003333	amino acid transmembrane transport	104	2	3.14E-01
GO:0006892	post-Golgi vesicle-mediated transport	104	2	3.14E-01
GO:0019438	aromatic compound biosynthetic process	4100	48	3.15E-01
GO:0060828	regulation of canonical Wnt signaling pathway	260	4	3.15E-01
GO:0043409	negative regulation of MAPK cascade	181	3	3.16E-01
GO:0034654	nucleobase-containing compound biosynthetic process	4013	47	3.16E-01
GO:0110149	regulation of biomineralization	105	2	3.18E-01
GO:1901655	cellular response to ketone	105	2	3.18E-01
GO:0001893	maternal placenta development	35	1	3.19E-01
GO:0006779	porphyrin-containing compound biosynthetic process	35	1	3.19E-01

GO:0016242	negative regulation of macroautophagy	35	1	3.19E-01
GO:0032633	interleukin-4 production	35	1	3.19E-01
GO:0032673	regulation of interleukin-4 production	35	1	3.19E-01
GO:0033014	tetrapyrrole biosynthetic process	35	1	3.19E-01
GO:0043276	anoikis	35	1	3.19E-01
GO:0045762	positive regulation of adenylate cyclase activity	35	1	3.19E-01
GO:0060914	heart formation	35	1	3.19E-01
GO:0071398	cellular response to fatty acid	35	1	3.19E-01
GO:0071425	hematopoietic stem cell proliferation	35	1	3.19E-01
GO:1903539	protein localization to postsynaptic membrane	35	1	3.19E-01
GO:0031904	endosome lumen	35	1	3.19E-01
GO:0005802	trans-Golgi network	262	4	3.20E-01
GO:0015837	amine transport	106	2	3.22E-01
GO:0022600	digestive system process	106	2	3.22E-01
GO:1901890	positive regulation of cell junction assembly	106	2	3.22E-01
GO:0090734	site of DNA damage	106	2	3.22E-01
GO:0030073	insulin secretion	183	3	3.22E-01
GO:0071466	cellular response to xenobiotic stimulus	183	3	3.22E-01
GO:0016604	nuclear body	849	11	3.22E-01
GO:0050728	negative regulation of inflammatory response	184	3	3.25E-01
GO:1902107	positive regulation of leukocyte differentiation	184	3	3.25E-01
GO:1903708	positive regulation of hemopoiesis	184	3	3.25E-01
GO:0008237	metallopeptidase activity	184	3	3.25E-01
GO:0032963	collagen metabolic process	107	2	3.26E-01
GO:0033045	regulation of sister chromatid segregation	107	2	3.26E-01
GO:0000423	mitophagy	36	1	3.26E-01
GO:0006699	bile acid biosynthetic process	36	1	3.26E-01
GO:0010614	negative regulation of cardiac muscle hypertrophy	36	1	3.26E-01
GO:0016486	peptide hormone processing	36	1	3.26E-01
GO:0019068	virion assembly	36	1	3.26E-01
GO:0031112	positive regulation of microtubule polymerization or dep	36	1	3.26E-01
GO:0035909	aorta morphogenesis	36	1	3.26E-01
GO:0044743	protein transmembrane import into intracellular organe	36	1	3.26E-01
GO:0046471	phosphatidylglycerol metabolic process	36	1	3.26E-01
GO:0050869	negative regulation of B cell activation	36	1	3.26E-01
GO:0062237	protein localization to postsynapse	36	1	3.26E-01
GO:0070306	lens fiber cell differentiation	36	1	3.26E-01
GO:0070670	response to interleukin-4	36	1	3.26E-01
GO:0071711	basement membrane organization	36	1	3.26E-01
GO:0086010	membrane depolarization during action potential	36	1	3.26E-01
GO:0090218	positive regulation of lipid kinase activity	36	1	3.26E-01
GO:0097345	mitochondrial outer membrane permeabilization	36	1	3.26E-01
GO:1903580	positive regulation of ATP metabolic process	36	1	3.26E-01
GO:0034358	plasma lipoprotein particle	36	1	3.26E-01
GO:1990777	lipoprotein particle	36	1	3.26E-01
GO:0015248	sterol transporter activity	36	1	3.26E-01
GO:0016814	hydrolase activity, acting on carbon-nitrogen (but not pe	36	1	3.26E-01

GO:0042056	chemoattractant activity	36	1	3.26E-01
GO:0061631	ubiquitin conjugating enzyme activity	36	1	3.26E-01
GO:0008234	cysteine-type peptidase activity	185	3	3.28E-01
GO:0008637	apoptotic mitochondrial changes	108	2	3.30E-01
GO:0051341	regulation of oxidoreductase activity	108	2	3.30E-01
GO:0062014	negative regulation of small molecule metabolic process	108	2	3.30E-01
GO:0090263	positive regulation of canonical Wnt signaling pathway	108	2	3.30E-01
GO:0090398	cellular senescence	108	2	3.30E-01
GO:0015698	inorganic anion transport	186	3	3.31E-01
GO:0043405	regulation of MAP kinase activity	186	3	3.31E-01
GO:0003161	cardiac conduction system development	37	1	3.34E-01
GO:0007588	excretion	37	1	3.34E-01
GO:0009225	nucleotide-sugar metabolic process	37	1	3.34E-01
GO:0009303	rRNA transcription	37	1	3.34E-01
GO:0010923	negative regulation of phosphatase activity	37	1	3.34E-01
GO:0010934	macrophage cytokine production	37	1	3.34E-01
GO:0010935	regulation of macrophage cytokine production	37	1	3.34E-01
GO:0021955	central nervous system neuron axonogenesis	37	1	3.34E-01
GO:0032435	negative regulation of proteasomal ubiquitin-dependent	37	1	3.34E-01
GO:0033144	negative regulation of intracellular steroid hormone rec	37	1	3.34E-01
GO:0070229	negative regulation of lymphocyte apoptotic process	37	1	3.34E-01
GO:0086019	cell-cell signaling involved in cardiac conduction	37	1	3.34E-01
GO:0000407	phagophore assembly site	37	1	3.34E-01
GO:0030414	peptidase inhibitor activity	187	3	3.34E-01
GO:0042113	B cell activation	350	5	3.35E-01
GO:0061629	RNA polymerase II-specific DNA-binding transcription fa	350	5	3.35E-01
GO:0016072	rRNA metabolic process	268	4	3.35E-01
GO:0006766	vitamin metabolic process	110	2	3.38E-01
GO:1902749	regulation of cell cycle G2/M phase transition	110	2	3.38E-01
GO:0006725	cellular aromatic compound metabolic process	6392	73	3.39E-01
GO:0019941	modification-dependent protein catabolic process	690	9	3.40E-01
GO:0006862	nucleotide transport	38	1	3.41E-01
GO:0007020	microtubule nucleation	38	1	3.41E-01
GO:0014741	negative regulation of muscle hypertrophy	38	1	3.41E-01
GO:0030851	granulocyte differentiation	38	1	3.41E-01
GO:0032094	response to food	38	1	3.41E-01
GO:0040001	establishment of mitotic spindle localization	38	1	3.41E-01
GO:0040036	regulation of fibroblast growth factor receptor signaling	38	1	3.41E-01
GO:0043368	positive T cell selection	38	1	3.41E-01
GO:0043516	regulation of DNA damage response, signal transduction	38	1	3.41E-01
GO:0045191	regulation of isotype switching	38	1	3.41E-01
GO:0048821	erythrocyte development	38	1	3.41E-01
GO:0140448	signaling receptor ligand precursor processing	38	1	3.41E-01
GO:0097386	glial cell projection	38	1	3.41E-01
GO:1905348	endonuclease complex	38	1	3.41E-01
GO:0003755	peptidyl-prolyl cis-trans isomerase activity	38	1	3.41E-01
GO:0010498	proteasomal protein catabolic process	521	7	3.42E-01

GO:0016079	synaptic vesicle exocytosis	111	2	3.42E-01
GO:0018107	peptidyl-threonine phosphorylation	111	2	3.42E-01
GO:0021761	limbic system development	111	2	3.42E-01
GO:0032611	interleukin-1 beta production	111	2	3.42E-01
GO:0032651	regulation of interleukin-1 beta production	111	2	3.42E-01
GO:0003774	cytoskeletal motor activity	111	2	3.42E-01
GO:0008017	microtubule binding	271	4	3.42E-01
GO:0043433	negative regulation of DNA-binding transcription factor a	190	3	3.43E-01
GO:0051260	protein homooligomerization	190	3	3.43E-01
GO:0051302	regulation of cell division	190	3	3.43E-01
GO:0008514	organic anion transmembrane transporter activity	190	3	3.43E-01
GO:0031267	small GTPase binding	272	4	3.45E-01
GO:0045445	myoblast differentiation	112	2	3.46E-01
GO:0055001	muscle cell development	191	3	3.46E-01
GO:0001990	regulation of systemic arterial blood pressure by hormo	39	1	3.48E-01
GO:0030279	negative regulation of ossification	39	1	3.48E-01
GO:0033028	myeloid cell apoptotic process	39	1	3.48E-01
GO:0043403	skeletal muscle tissue regeneration	39	1	3.48E-01
GO:0046329	negative regulation of JNK cascade	39	1	3.48E-01
GO:0046621	negative regulation of organ growth	39	1	3.48E-01
GO:0051569	regulation of histone H3-K4 methylation	39	1	3.48E-01
GO:0070316	regulation of G0 to G1 transition	39	1	3.48E-01
GO:0071634	regulation of transforming growth factor beta productio	39	1	3.48E-01
GO:0090181	regulation of cholesterol metabolic process	39	1	3.48E-01
GO:1905168	positive regulation of double-strand break repair via hor	39	1	3.48E-01
GO:0032040	small-subunit processome	39	1	3.48E-01
GO:0032994	protein-lipid complex	39	1	3.48E-01
GO:0099738	cell cortex region	39	1	3.48E-01
GO:0004177	aminopeptidase activity	39	1	3.48E-01
GO:0031099	regeneration	192	3	3.49E-01
GO:0006939	smooth muscle contraction	113	2	3.50E-01
GO:1904659	glucose transmembrane transport	113	2	3.50E-01
GO:0046578	regulation of Ras protein signal transduction	193	3	3.52E-01
GO:0044344	cellular response to fibroblast growth factor stimulus	114	2	3.54E-01
GO:0009749	response to glucose	194	3	3.55E-01
GO:0061135	endopeptidase regulator activity	194	3	3.55E-01
GO:0007007	inner mitochondrial membrane organization	40	1	3.55E-01
GO:0007212	dopamine receptor signaling pathway	40	1	3.55E-01
GO:0010863	positive regulation of phospholipase C activity	40	1	3.55E-01
GO:0032965	regulation of collagen biosynthetic process	40	1	3.55E-01
GO:0045740	positive regulation of DNA replication	40	1	3.55E-01
GO:0045823	positive regulation of heart contraction	40	1	3.55E-01
GO:0046627	negative regulation of insulin receptor signaling pathway	40	1	3.55E-01
GO:0051154	negative regulation of striated muscle cell differentiatior	40	1	3.55E-01
GO:0051294	establishment of spindle orientation	40	1	3.55E-01
GO:1901186	positive regulation of ERBB signaling pathway	40	1	3.55E-01
GO:1901532	regulation of hematopoietic progenitor cell differentiati	40	1	3.55E-01

GO:1902692	regulation of neuroblast proliferation	40	1	3.55E-01
GO:0017080	sodium channel regulator activity	40	1	3.55E-01
GO:0043028	cysteine-type endopeptidase regulator activity involved	40	1	3.55E-01
GO:0061650	ubiquitin-like protein conjugating enzyme activity	40	1	3.55E-01
GO:0046634	regulation of alpha-beta T cell activation	115	2	3.57E-01
GO:0048640	negative regulation of developmental growth	115	2	3.57E-01
GO:0005776	autophagosome	115	2	3.57E-01
GO:0043679	axon terminus	115	2	3.57E-01
GO:0043632	modification-dependent macromolecule catabolic proce	702	9	3.58E-01
GO:0007369	gastrulation	196	3	3.61E-01
GO:0008645	hexose transmembrane transport	116	2	3.61E-01
GO:0030518	intracellular steroid hormone receptor signaling pathwa	116	2	3.61E-01
GO:0072009	nephron epithelium development	116	2	3.61E-01
GO:0030513	positive regulation of BMP signaling pathway	41	1	3.62E-01
GO:0032733	positive regulation of interleukin-10 production	41	1	3.62E-01
GO:0034122	negative regulation of toll-like receptor signaling pathwa	41	1	3.62E-01
GO:0045023	G0 to G1 transition	41	1	3.62E-01
GO:0045292	mRNA cis splicing, via spliceosome	41	1	3.62E-01
GO:0051693	actin filament capping	41	1	3.62E-01
GO:0060338	regulation of type I interferon-mediated signaling pathw	41	1	3.62E-01
GO:0070232	regulation of T cell apoptotic process	41	1	3.62E-01
GO:0070884	regulation of calcineurin-NFAT signaling cascade	41	1	3.62E-01
GO:0072595	maintenance of protein localization in organelle	41	1	3.62E-01
GO:1901031	regulation of response to reactive oxygen species	41	1	3.62E-01
GO:1903524	positive regulation of blood circulation	41	1	3.62E-01
GO:0016859	cis-trans isomerase activity	41	1	3.62E-01
GO:0016877	ligase activity, forming carbon-sulfur bonds	41	1	3.62E-01
GO:0045582	positive regulation of T cell differentiation	117	2	3.65E-01
GO:0046632	alpha-beta T cell differentiation	117	2	3.65E-01
GO:0071887	leukocyte apoptotic process	117	2	3.65E-01
GO:0002695	negative regulation of leukocyte activation	198	3	3.67E-01
GO:0071248	cellular response to metal ion	198	3	3.67E-01
GO:0003713	transcription coactivator activity	281	4	3.67E-01
GO:0051726	regulation of cell cycle	1145	14	3.68E-01
GO:0002286	T cell activation involved in immune response	118	2	3.69E-01
GO:0006821	chloride transport	118	2	3.69E-01
GO:0043200	response to amino acid	118	2	3.69E-01
GO:0007099	centriole replication	42	1	3.69E-01
GO:0009167	purine ribonucleoside monophosphate metabolic proces	42	1	3.69E-01
GO:0043029	T cell homeostasis	42	1	3.69E-01
GO:0050999	regulation of nitric-oxide synthase activity	42	1	3.69E-01
GO:0055090	acylglycerol homeostasis	42	1	3.69E-01
GO:0070328	triglyceride homeostasis	42	1	3.69E-01
GO:0071604	transforming growth factor beta production	42	1	3.69E-01
GO:0106056	regulation of calcineurin-mediated signaling	42	1	3.69E-01
GO:1900077	negative regulation of cellular response to insulin stimul	42	1	3.69E-01
GO:1900274	regulation of phospholipase C activity	42	1	3.69E-01

GO:1902110	positive regulation of mitochondrial membrane permea	42	1	3.69E-01
GO:0098563	intrinsic component of synaptic vesicle membrane	42	1	3.69E-01
GO:0009746	response to hexose	199	3	3.70E-01
GO:0016829	lyase activity	199	3	3.70E-01
GO:0006066	alcohol metabolic process	367	5	3.71E-01
GO:0005516	calmodulin binding	200	3	3.73E-01
GO:0010927	cellular component assembly involved in morphogenesis	119	2	3.73E-01
GO:0015718	monocarboxylic acid transport	119	2	3.73E-01
GO:0015749	monosaccharide transmembrane transport	119	2	3.73E-01
GO:0007595	lactation	43	1	3.76E-01
GO:0009124	nucleoside monophosphate biosynthetic process	43	1	3.76E-01
GO:0009154	purine ribonucleotide catabolic process	43	1	3.76E-01
GO:0016339	calcium-dependent cell-cell adhesion via plasma membr	43	1	3.76E-01
GO:0021879	forebrain neuron differentiation	43	1	3.76E-01
GO:0032691	negative regulation of interleukin-1 beta production	43	1	3.76E-01
GO:0042491	inner ear auditory receptor cell differentiation	43	1	3.76E-01
GO:0042789	mRNA transcription by RNA polymerase II	43	1	3.76E-01
GO:0045494	photoreceptor cell maintenance	43	1	3.76E-01
GO:0051954	positive regulation of amine transport	43	1	3.76E-01
GO:0060428	lung epithelium development	43	1	3.76E-01
GO:0071459	protein localization to chromosome, centromeric region	43	1	3.76E-01
GO:0097009	energy homeostasis	43	1	3.76E-01
GO:1900371	regulation of purine nucleotide biosynthetic process	43	1	3.76E-01
GO:1902275	regulation of chromatin organization	43	1	3.76E-01
GO:1904037	positive regulation of epithelial cell apoptotic process	43	1	3.76E-01
GO:0030547	signaling receptor inhibitor activity	43	1	3.76E-01
GO:0018210	peptidyl-threonine modification	120	2	3.77E-01
GO:0071774	response to fibroblast growth factor	120	2	3.77E-01
GO:0031985	Golgi cisterna	120	2	3.77E-01
GO:0004197	cysteine-type endopeptidase activity	120	2	3.77E-01
GO:0051960	regulation of nervous system development	456	6	3.79E-01
GO:0006334	nucleosome assembly	121	2	3.81E-01
GO:0006720	isoprenoid metabolic process	121	2	3.81E-01
GO:0051101	regulation of DNA binding	121	2	3.81E-01
GO:0090100	positive regulation of transmembrane receptor protein s	121	2	3.81E-01
GO:0001709	cell fate determination	44	1	3.83E-01
GO:0003009	skeletal muscle contraction	44	1	3.83E-01
GO:0009126	purine nucleoside monophosphate metabolic process	44	1	3.83E-01
GO:0016601	Rac protein signal transduction	44	1	3.83E-01
GO:0030808	regulation of nucleotide biosynthetic process	44	1	3.83E-01
GO:0042771	intrinsic apoptotic signaling pathway in response to DNA	44	1	3.83E-01
GO:0045214	sarcomere organization	44	1	3.83E-01
GO:0045663	positive regulation of myoblast differentiation	44	1	3.83E-01
GO:0046640	regulation of alpha-beta T cell proliferation	44	1	3.83E-01
GO:0060119	inner ear receptor cell development	44	1	3.83E-01
GO:0061001	regulation of dendritic spine morphogenesis	44	1	3.83E-01
GO:0071526	semaphorin-plexin signaling pathway	44	1	3.83E-01

GO:0090317	negative regulation of intracellular protein transport	44	1	3.83E-01
GO:1902686	mitochondrial outer membrane permeabilization involve	44	1	3.83E-01
GO:1904706	negative regulation of vascular associated smooth musc	44	1	3.83E-01
GO:0030118	clathrin coat	44	1	3.83E-01
GO:0001046	core promoter sequence-specific DNA binding	44	1	3.83E-01
GO:0001222	transcription corepressor binding	44	1	3.83E-01
GO:0004180	carboxypeptidase activity	44	1	3.83E-01
GO:0006338	chromatin remodeling	373	5	3.84E-01
GO:0005770	late endosome	288	4	3.84E-01
GO:0002761	regulation of myeloid leukocyte differentiation	122	2	3.85E-01
GO:0008286	insulin receptor signaling pathway	122	2	3.85E-01
GO:0030278	regulation of ossification	122	2	3.85E-01
GO:0071901	negative regulation of protein serine/threonine kinase a	122	2	3.85E-01
GO:1901989	positive regulation of cell cycle phase transition	122	2	3.85E-01
GO:0097733	photoreceptor cell cilium	122	2	3.85E-01
GO:0030282	bone mineralization	123	2	3.89E-01
GO:0046887	positive regulation of hormone secretion	123	2	3.89E-01
GO:0046942	carboxylic acid transport	290	4	3.89E-01
GO:0030501	positive regulation of bone mineralization	45	1	3.90E-01
GO:0030517	negative regulation of axon extension	45	1	3.90E-01
GO:0030574	collagen catabolic process	45	1	3.90E-01
GO:0030835	negative regulation of actin filament depolymerization	45	1	3.90E-01
GO:0030890	positive regulation of B cell proliferation	45	1	3.90E-01
GO:0046427	positive regulation of receptor signaling pathway via JAK	45	1	3.90E-01
GO:0060964	regulation of miRNA-mediated gene silencing	45	1	3.90E-01
GO:1901021	positive regulation of calcium ion transmembrane trans	45	1	3.90E-01
GO:1901381	positive regulation of potassium ion transmembrane tra	45	1	3.90E-01
GO:2000785	regulation of autophagosome assembly	45	1	3.90E-01
GO:0016504	peptidase activator activity	45	1	3.90E-01
GO:2000241	regulation of reproductive process	206	3	3.90E-01
GO:0072089	stem cell proliferation	124	2	3.93E-01
GO:0034284	response to monosaccharide	207	3	3.93E-01
GO:0003014	renal system process	125	2	3.96E-01
GO:0004519	endonuclease activity	125	2	3.96E-01
GO:0017124	SH3 domain binding	125	2	3.96E-01
GO:0002920	regulation of humoral immune response	46	1	3.96E-01
GO:0010677	negative regulation of cellular carbohydrate metabolic p	46	1	3.96E-01
GO:0033173	calcineurin-NFAT signaling cascade	46	1	3.96E-01
GO:0042088	T-helper 1 type immune response	46	1	3.96E-01
GO:0042168	heme metabolic process	46	1	3.96E-01
GO:0042398	cellular modified amino acid biosynthetic process	46	1	3.96E-01
GO:0046596	regulation of viral entry into host cell	46	1	3.96E-01
GO:0055010	ventricular cardiac muscle tissue morphogenesis	46	1	3.96E-01
GO:0085029	extracellular matrix assembly	46	1	3.96E-01
GO:0098534	centriole assembly	46	1	3.96E-01
GO:1901861	regulation of muscle tissue development	46	1	3.96E-01
GO:1904752	regulation of vascular associated smooth muscle cell mi	46	1	3.96E-01

GO:2000273	positive regulation of signaling receptor activity	46	1	3.96E-01
GO:0001784	phosphotyrosine residue binding	46	1	3.96E-01
GO:0019894	kinesin binding	46	1	3.96E-01
GO:0005929	cilium	728	9	3.98E-01
GO:0003231	cardiac ventricle development	126	2	4.00E-01
GO:0038127	ERBB signaling pathway	126	2	4.00E-01
GO:0098661	inorganic anion transmembrane transport	126	2	4.00E-01
GO:0008542	visual learning	47	1	4.03E-01
GO:0010824	regulation of centrosome duplication	47	1	4.03E-01
GO:0030521	androgen receptor signaling pathway	47	1	4.03E-01
GO:0034314	Arp2/3 complex-mediated actin nucleation	47	1	4.03E-01
GO:0035794	positive regulation of mitochondrial membrane permea	47	1	4.03E-01
GO:0046633	alpha-beta T cell proliferation	47	1	4.03E-01
GO:0061383	trabecula morphogenesis	47	1	4.03E-01
GO:0097028	dendritic cell differentiation	47	1	4.03E-01
GO:1900368	regulation of post-transcriptional gene silencing by RNA	47	1	4.03E-01
GO:1902459	positive regulation of stem cell population maintenance	47	1	4.03E-01
GO:0043195	terminal bouton	47	1	4.03E-01
GO:0048020	CCR chemokine receptor binding	47	1	4.03E-01
GO:0001704	formation of primary germ layer	127	2	4.04E-01
GO:0005923	bicellular tight junction	127	2	4.04E-01
GO:0140534	endoplasmic reticulum protein-containing complex	127	2	4.04E-01
GO:0042886	amide transport	296	4	4.04E-01
GO:0045739	positive regulation of DNA repair	128	2	4.08E-01
GO:0050868	negative regulation of T cell activation	128	2	4.08E-01
GO:0016810	hydrolase activity, acting on carbon-nitrogen (but not pe	128	2	4.08E-01
GO:0001974	blood vessel remodeling	48	1	4.10E-01
GO:0002673	regulation of acute inflammatory response	48	1	4.10E-01
GO:0006195	purine nucleotide catabolic process	48	1	4.10E-01
GO:0009299	mRNA transcription	48	1	4.10E-01
GO:0032309	icosanoid secretion	48	1	4.10E-01
GO:0032527	protein exit from endoplasmic reticulum	48	1	4.10E-01
GO:0032892	positive regulation of organic acid transport	48	1	4.10E-01
GO:0042220	response to cocaine	48	1	4.10E-01
GO:0045581	negative regulation of T cell differentiation	48	1	4.10E-01
GO:0045747	positive regulation of Notch signaling pathway	48	1	4.10E-01
GO:0045981	positive regulation of nucleotide metabolic process	48	1	4.10E-01
GO:0060147	regulation of post-transcriptional gene silencing	48	1	4.10E-01
GO:0071985	multivesicular body sorting pathway	48	1	4.10E-01
GO:1900544	positive regulation of purine nucleotide metabolic proce	48	1	4.10E-01
GO:1903587	regulation of blood vessel endothelial cell proliferation i	48	1	4.10E-01
GO:1904738	vascular associated smooth muscle cell migration	48	1	4.10E-01
GO:0044282	small molecule catabolic process	385	5	4.10E-01
GO:0030258	lipid modification	213	3	4.11E-01
GO:2000027	regulation of animal organ morphogenesis	129	2	4.12E-01
GO:0032612	interleukin-1 production	130	2	4.15E-01
GO:0032652	regulation of interleukin-1 production	130	2	4.15E-01

GO:0051028	mRNA transport	130	2	4.15E-01
GO:0003044	regulation of systemic arterial blood pressure mediated	49	1	4.16E-01
GO:0007602	phototransduction	49	1	4.16E-01
GO:0008206	bile acid metabolic process	49	1	4.16E-01
GO:0009261	ribonucleotide catabolic process	49	1	4.16E-01
GO:0018208	peptidyl-proline modification	49	1	4.16E-01
GO:0031281	positive regulation of cyclase activity	49	1	4.16E-01
GO:0035094	response to nicotine	49	1	4.16E-01
GO:0042311	vasodilation	49	1	4.16E-01
GO:0043114	regulation of vascular permeability	49	1	4.16E-01
GO:0043300	regulation of leukocyte degranulation	49	1	4.16E-01
GO:0043457	regulation of cellular respiration	49	1	4.16E-01
GO:0061178	regulation of insulin secretion involved in cellular respor	49	1	4.16E-01
GO:1902108	regulation of mitochondrial membrane permeability inv	49	1	4.16E-01
GO:1904894	positive regulation of receptor signaling pathway via STA	49	1	4.16E-01
GO:0098687	chromosomal region	388	5	4.17E-01
GO:0006644	phospholipid metabolic process	389	5	4.19E-01
GO:0044306	neuron projection terminus	131	2	4.19E-01
GO:0008047	enzyme activator activity	565	7	4.20E-01
GO:0007178	transmembrane receptor protein serine/threonine kinas	390	5	4.21E-01
GO:0006376	mRNA splice site selection	50	1	4.22E-01
GO:0032964	collagen biosynthetic process	50	1	4.22E-01
GO:0051349	positive regulation of lyase activity	50	1	4.22E-01
GO:0060966	regulation of gene silencing by RNA	50	1	4.22E-01
GO:0061082	myeloid leukocyte cytokine production	50	1	4.22E-01
GO:0072599	establishment of protein localization to endoplasmic ret	50	1	4.22E-01
GO:0090102	cochlea development	50	1	4.22E-01
GO:0120009	intermembrane lipid transfer	50	1	4.22E-01
GO:1901570	fatty acid derivative biosynthetic process	50	1	4.22E-01
GO:1902041	regulation of extrinsic apoptotic signaling pathway via de	50	1	4.22E-01
GO:1904707	positive regulation of vascular associated smooth muscl	50	1	4.22E-01
GO:2000059	negative regulation of ubiquitin-dependent protein cata	50	1	4.22E-01
GO:2001238	positive regulation of extrinsic apoptotic signaling pathw	50	1	4.22E-01
GO:0016469	proton-transporting two-sector ATPase complex	50	1	4.22E-01
GO:0071005	U2-type precatalytic spliceosome	50	1	4.22E-01
GO:0120013	lipid transfer activity	50	1	4.22E-01
GO:0034219	carbohydrate transmembrane transport	132	2	4.23E-01
GO:0002250	adaptive immune response	744	9	4.23E-01
GO:0009165	nucleotide biosynthetic process	304	4	4.24E-01
GO:0051607	defense response to virus	304	4	4.24E-01
GO:0043523	regulation of neuron apoptotic process	218	3	4.25E-01
GO:0140546	defense response to symbiont	305	4	4.26E-01
GO:0000018	regulation of DNA recombination	133	2	4.27E-01
GO:0001101	response to acid chemical	133	2	4.27E-01
GO:0001669	acrosomal vesicle	133	2	4.27E-01
GO:0000280	nuclear division	481	6	4.28E-01
GO:0050673	epithelial cell proliferation	481	6	4.28E-01

GO:0002377	immunoglobulin production	219	3	4.28E-01
GO:1901293	nucleoside phosphate biosynthetic process	306	4	4.29E-01
GO:0030225	macrophage differentiation	51	1	4.29E-01
GO:0031062	positive regulation of histone methylation	51	1	4.29E-01
GO:0032692	negative regulation of interleukin-1 production	51	1	4.29E-01
GO:0035315	hair cell differentiation	51	1	4.29E-01
GO:0046580	negative regulation of Ras protein signal transduction	51	1	4.29E-01
GO:0050873	brown fat cell differentiation	51	1	4.29E-01
GO:0050982	detection of mechanical stimulus	51	1	4.29E-01
GO:0060425	lung morphogenesis	51	1	4.29E-01
GO:0060688	regulation of morphogenesis of a branching structure	51	1	4.29E-01
GO:0097720	calcineurin-mediated signaling	51	1	4.29E-01
GO:1901799	negative regulation of proteasomal protein catabolic pro	51	1	4.29E-01
GO:1903793	positive regulation of anion transport	51	1	4.29E-01
GO:0001673	male germ cell nucleus	51	1	4.29E-01
GO:0016830	carbon-carbon lyase activity	51	1	4.29E-01
GO:0006661	phosphatidylinositol biosynthetic process	134	2	4.30E-01
GO:0097731	9+0 non-motile cilium	134	2	4.30E-01
GO:0005759	mitochondrial matrix	483	6	4.31E-01
GO:0015085	calcium ion transmembrane transporter activity	135	2	4.34E-01
GO:0007215	glutamate receptor signaling pathway	52	1	4.35E-01
GO:0010043	response to zinc ion	52	1	4.35E-01
GO:0010823	negative regulation of mitochondrion organization	52	1	4.35E-01
GO:0043268	positive regulation of potassium ion transport	52	1	4.35E-01
GO:0043303	mast cell degranulation	52	1	4.35E-01
GO:0043392	negative regulation of DNA binding	52	1	4.35E-01
GO:0045912	negative regulation of carbohydrate metabolic process	52	1	4.35E-01
GO:0051480	regulation of cytosolic calcium ion concentration	52	1	4.35E-01
GO:1905710	positive regulation of membrane permeability	52	1	4.35E-01
GO:0030315	T-tubule	52	1	4.35E-01
GO:0034707	chloride channel complex	52	1	4.35E-01
GO:0001822	kidney development	309	4	4.36E-01
GO:0007498	mesoderm development	136	2	4.38E-01
GO:0043401	steroid hormone mediated signaling pathway	136	2	4.38E-01
GO:0060070	canonical Wnt signaling pathway	310	4	4.38E-01
GO:0044770	cell cycle phase transition	576	7	4.39E-01
GO:0071805	potassium ion transmembrane transport	223	3	4.40E-01
GO:0046486	glycerolipid metabolic process	399	5	4.40E-01
GO:0002639	positive regulation of immunoglobulin production	53	1	4.41E-01
GO:0006778	porphyrin-containing compound metabolic process	53	1	4.41E-01
GO:0006890	retrograde vesicle-mediated transport, Golgi to endopla	53	1	4.41E-01
GO:0007632	visual behavior	53	1	4.41E-01
GO:0009584	detection of visible light	53	1	4.41E-01
GO:0010874	regulation of cholesterol efflux	53	1	4.41E-01
GO:0046638	positive regulation of alpha-beta T cell differentiation	53	1	4.41E-01
GO:0065002	intracellular protein transmembrane transport	53	1	4.41E-01
GO:2000677	regulation of transcription regulatory region DNA bindin	53	1	4.41E-01

GO:0071011	precatalytic spliceosome	53	1	4.41E-01
GO:0009581	detection of external stimulus	137	2	4.42E-01
GO:0043177	organic acid binding	137	2	4.42E-01
GO:0050679	positive regulation of epithelial cell proliferation	224	3	4.43E-01
GO:0030879	mammary gland development	138	2	4.45E-01
GO:0030072	peptide hormone secretion	225	3	4.45E-01
GO:0000287	magnesium ion binding	225	3	4.45E-01
GO:0018105	peptidyl-serine phosphorylation	313	4	4.46E-01
GO:0002279	mast cell activation involved in immune response	54	1	4.47E-01
GO:0031113	regulation of microtubule polymerization	54	1	4.47E-01
GO:0042149	cellular response to glucose starvation	54	1	4.47E-01
GO:0045778	positive regulation of ossification	54	1	4.47E-01
GO:0046605	regulation of centrosome cycle	54	1	4.47E-01
GO:0051703	biological process involved in intraspecies interaction be	54	1	4.47E-01
GO:0061900	glial cell activation	54	1	4.47E-01
GO:0072523	purine-containing compound catabolic process	54	1	4.47E-01
GO:0072698	protein localization to microtubule cytoskeleton	54	1	4.47E-01
GO:0008374	O-acyltransferase activity	54	1	4.47E-01
GO:0031593	polyubiquitin modification-dependent protein binding	54	1	4.47E-01
GO:0007586	digestion	139	2	4.49E-01
GO:0020037	heme binding	139	2	4.49E-01
GO:1905114	cell surface receptor signaling pathway involved in cell-c	582	7	4.50E-01
GO:0009582	detection of abiotic stimulus	140	2	4.53E-01
GO:0042157	lipoprotein metabolic process	140	2	4.53E-01
GO:0070851	growth factor receptor binding	140	2	4.53E-01
GO:0015267	channel activity	494	6	4.53E-01
GO:0002043	blood vessel endothelial cell proliferation involved in spr	55	1	4.53E-01
GO:0002448	mast cell mediated immunity	55	1	4.53E-01
GO:0003229	ventricular cardiac muscle tissue development	55	1	4.53E-01
GO:0010659	cardiac muscle cell apoptotic process	55	1	4.53E-01
GO:0031103	axon regeneration	55	1	4.53E-01
GO:0043525	positive regulation of neuron apoptotic process	55	1	4.53E-01
GO:0043616	keratinocyte proliferation	55	1	4.53E-01
GO:0043647	inositol phosphate metabolic process	55	1	4.53E-01
GO:0055078	sodium ion homeostasis	55	1	4.53E-01
GO:0070169	positive regulation of biomineral tissue development	55	1	4.53E-01
GO:1903202	negative regulation of oxidative stress-induced cell deat	55	1	4.53E-01
GO:2000107	negative regulation of leukocyte apoptotic process	55	1	4.53E-01
GO:2000648	positive regulation of stem cell proliferation	55	1	4.53E-01
GO:2000772	regulation of cellular senescence	55	1	4.53E-01
GO:0022803	passive transmembrane transporter activity	495	6	4.55E-01
GO:0015075	ion transmembrane transporter activity	855	10	4.56E-01
GO:0006364	rRNA processing	229	3	4.57E-01
GO:0071241	cellular response to inorganic substance	229	3	4.57E-01
GO:0072001	renal system development	318	4	4.58E-01
GO:0009116	nucleoside metabolic process	56	1	4.59E-01
GO:0015695	organic cation transport	56	1	4.59E-01

GO:0033047	regulation of mitotic sister chromatid segregation	56	1	4.59E-01
GO:0042908	xenobiotic transport	56	1	4.59E-01
GO:0043551	regulation of phosphatidylinositol 3-kinase activity	56	1	4.59E-01
GO:0071715	icosanoid transport	56	1	4.59E-01
GO:0097120	receptor localization to synapse	56	1	4.59E-01
GO:0110151	positive regulation of biomineralization	56	1	4.59E-01
GO:0004869	cysteine-type endopeptidase inhibitor activity	56	1	4.59E-01
GO:0000086	G2/M transition of mitotic cell cycle	142	2	4.60E-01
GO:0006511	ubiquitin-dependent protein catabolic process	680	8	4.64E-01
GO:0098813	nuclear chromosome segregation	321	4	4.65E-01
GO:0007080	mitotic metaphase plate congression	57	1	4.65E-01
GO:0010658	striated muscle cell apoptotic process	57	1	4.65E-01
GO:0032768	regulation of monooxygenase activity	57	1	4.65E-01
GO:0035196	miRNA processing	57	1	4.65E-01
GO:0043113	receptor clustering	57	1	4.65E-01
GO:0043388	positive regulation of DNA binding	57	1	4.65E-01
GO:0046148	pigment biosynthetic process	57	1	4.65E-01
GO:0050879	multicellular organismal movement	57	1	4.65E-01
GO:0050881	musculoskeletal movement	57	1	4.65E-01
GO:0070231	T cell apoptotic process	57	1	4.65E-01
GO:0030120	vesicle coat	57	1	4.65E-01
GO:0030660	Golgi-associated vesicle membrane	57	1	4.65E-01
GO:0030177	positive regulation of Wnt signaling pathway	144	2	4.67E-01
GO:0008406	gonad development	233	3	4.68E-01
GO:0009743	response to carbohydrate	234	3	4.71E-01
GO:0046883	regulation of hormone secretion	234	3	4.71E-01
GO:0001541	ovarian follicle development	58	1	4.71E-01
GO:0001961	positive regulation of cytokine-mediated signaling pathv	58	1	4.71E-01
GO:0044380	protein localization to cytoskeleton	58	1	4.71E-01
GO:0045620	negative regulation of lymphocyte differentiation	58	1	4.71E-01
GO:0048010	vascular endothelial growth factor receptor signaling pa	58	1	4.71E-01
GO:0048016	inositol phosphate-mediated signaling	58	1	4.71E-01
GO:0051058	negative regulation of small GTPase mediated signal tra	58	1	4.71E-01
GO:0051898	negative regulation of protein kinase B signaling	58	1	4.71E-01
GO:0050678	regulation of epithelial cell proliferation	414	5	4.72E-01
GO:0007018	microtubule-based movement	415	5	4.74E-01
GO:0072073	kidney epithelium development	146	2	4.74E-01
GO:1901214	regulation of neuron death	325	4	4.74E-01
GO:0046394	carboxylic acid biosynthetic process	326	4	4.77E-01
GO:0001755	neural crest cell migration	59	1	4.77E-01
GO:0009161	ribonucleoside monophosphate metabolic process	59	1	4.77E-01
GO:0033363	secretory granule organization	59	1	4.77E-01
GO:0035773	insulin secretion involved in cellular response to glucose	59	1	4.77E-01
GO:0045010	actin nucleation	59	1	4.77E-01
GO:0061098	positive regulation of protein tyrosine kinase activity	59	1	4.77E-01
GO:0097352	autophagosome maturation	59	1	4.77E-01
GO:0006119	oxidative phosphorylation	147	2	4.78E-01

GO:0006865	amino acid transport	147	2	4.78E-01
GO:0008584	male gonad development	147	2	4.78E-01
GO:0035296	regulation of tube diameter	147	2	4.78E-01
GO:0097746	blood vessel diameter maintenance	147	2	4.78E-01
GO:1903038	negative regulation of leukocyte cell-cell adhesion	147	2	4.78E-01
GO:0015748	organophosphate ester transport	148	2	4.81E-01
GO:0035150	regulation of tube size	148	2	4.81E-01
GO:0046546	development of primary male sexual characteristics	148	2	4.81E-01
GO:0042734	presynaptic membrane	148	2	4.81E-01
GO:0016053	organic acid biosynthetic process	328	4	4.81E-01
GO:0071496	cellular response to external stimulus	328	4	4.81E-01
GO:0045137	development of primary sexual characteristics	238	3	4.82E-01
GO:0001658	branching involved in ureteric bud morphogenesis	60	1	4.82E-01
GO:0002753	cytoplasmic pattern recognition receptor signaling pathv	60	1	4.82E-01
GO:0002886	regulation of myeloid leukocyte mediated immunity	60	1	4.82E-01
GO:0055008	cardiac muscle tissue morphogenesis	60	1	4.82E-01
GO:2000351	regulation of endothelial cell apoptotic process	60	1	4.82E-01
GO:0031305	integral component of mitochondrial inner membrane	60	1	4.82E-01
GO:0032589	neuron projection membrane	60	1	4.82E-01
GO:0008028	monocarboxylic acid transmembrane transporter activit	60	1	4.82E-01
GO:0016607	nuclear speck	419	5	4.82E-01
GO:0003712	transcription coregulator activity	510	6	4.83E-01
GO:0009887	animal organ morphogenesis	1056	12	4.84E-01
GO:0016525	negative regulation of angiogenesis	149	2	4.85E-01
GO:0046906	tetrapyrrole binding	149	2	4.85E-01
GO:0043112	receptor metabolic process	61	1	4.88E-01
GO:0060113	inner ear receptor cell differentiation	61	1	4.88E-01
GO:0070918	small regulatory ncRNA processing	61	1	4.88E-01
GO:0090497	mesenchymal cell migration	61	1	4.88E-01
GO:0031304	intrinsic component of mitochondrial inner membrane	61	1	4.88E-01
GO:0005507	copper ion binding	61	1	4.88E-01
GO:0001890	placenta development	150	2	4.88E-01
GO:0031023	microtubule organizing center organization	150	2	4.88E-01
GO:0098876	vesicle-mediated transport to the plasma membrane	150	2	4.88E-01
GO:0140097	catalytic activity, acting on DNA	241	3	4.90E-01
GO:0006310	DNA recombination	332	4	4.91E-01
GO:0018209	peptidyl-serine modification	332	4	4.91E-01
GO:0051603	proteolysis involved in protein catabolic process	788	9	4.91E-01
GO:0007269	neurotransmitter secretion	151	2	4.92E-01
GO:0099643	signal release from synapse	151	2	4.92E-01
GO:2000181	negative regulation of blood vessel morphogenesis	151	2	4.92E-01
GO:0009890	negative regulation of biosynthetic process	1702	19	4.92E-01
GO:0065004	protein-DNA complex assembly	242	3	4.93E-01
GO:0031102	neuron projection regeneration	62	1	4.94E-01
GO:0031122	cytoplasmic microtubule organization	62	1	4.94E-01
GO:0032387	negative regulation of intracellular transport	62	1	4.94E-01
GO:0035904	aorta development	62	1	4.94E-01

GO:0043506	regulation of JUN kinase activity	62	1	4.94E-01
GO:0046902	regulation of mitochondrial membrane permeability	62	1	4.94E-01
GO:0060393	regulation of pathway-restricted SMAD protein phospho	62	1	4.94E-01
GO:0070228	regulation of lymphocyte apoptotic process	62	1	4.94E-01
GO:1903018	regulation of glycoprotein metabolic process	62	1	4.94E-01
GO:0099023	vesicle tethering complex	62	1	4.94E-01
GO:1901343	negative regulation of vasculature development	152	2	4.95E-01
GO:0052689	carboxylic ester hydrolase activity	152	2	4.95E-01
GO:0007346	regulation of mitotic cell cycle	517	6	4.96E-01
GO:0004175	endopeptidase activity	426	5	4.97E-01
GO:0030902	hindbrain development	153	2	4.99E-01
GO:0048284	organelle fusion	153	2	4.99E-01
GO:0032623	interleukin-2 production	63	1	4.99E-01
GO:0032663	regulation of interleukin-2 production	63	1	4.99E-01
GO:0032731	positive regulation of interleukin-1 beta production	63	1	4.99E-01
GO:0046173	polyol biosynthetic process	63	1	4.99E-01
GO:0046579	positive regulation of Ras protein signal transduction	63	1	4.99E-01
GO:0051055	negative regulation of lipid biosynthetic process	63	1	4.99E-01
GO:0070059	intrinsic apoptotic signaling pathway in response to endo	63	1	4.99E-01
GO:0098781	ncRNA transcription	63	1	4.99E-01
GO:0005637	nuclear inner membrane	63	1	4.99E-01
GO:0005771	multivesicular body	63	1	4.99E-01
GO:0044304	main axon	63	1	4.99E-01
GO:0043524	negative regulation of neuron apoptotic process	154	2	5.02E-01
GO:0019229	regulation of vasoconstriction	64	1	5.05E-01
GO:0030199	collagen fibril organization	64	1	5.05E-01
GO:0071806	protein transmembrane transport	64	1	5.05E-01
GO:0005778	peroxisomal membrane	64	1	5.05E-01
GO:0030173	integral component of Golgi membrane	64	1	5.05E-01
GO:0031903	microbody membrane	64	1	5.05E-01
GO:0005548	phospholipid transporter activity	64	1	5.05E-01
GO:0031224	intrinsic component of membrane	5564	61	5.05E-01
GO:0035148	tube formation	155	2	5.06E-01
GO:0050871	positive regulation of B cell activation	155	2	5.06E-01
GO:0031902	late endosome membrane	155	2	5.06E-01
GO:0006813	potassium ion transport	247	3	5.07E-01
GO:0008643	carbohydrate transport	156	2	5.09E-01
GO:0003170	heart valve development	65	1	5.10E-01
GO:0031100	animal organ regeneration	65	1	5.10E-01
GO:0033013	tetrapyrrole metabolic process	65	1	5.10E-01
GO:0040014	regulation of multicellular organism growth	65	1	5.10E-01
GO:0050771	negative regulation of axonogenesis	65	1	5.10E-01
GO:0016836	hydro-lyase activity	65	1	5.10E-01
GO:0022836	gated channel activity	341	4	5.12E-01
GO:0007189	adenylate cyclase-activating G protein-coupled receptor	157	2	5.12E-01
GO:0005814	centriole	157	2	5.12E-01
GO:0000082	G1/S transition of mitotic cell cycle	250	3	5.15E-01

GO:0071695	anatomical structure maturation	250	3	5.15E-01
GO:0000775	chromosome, centromeric region	250	3	5.15E-01
GO:0042490	mechanoreceptor differentiation	66	1	5.16E-01
GO:0048814	regulation of dendrite morphogenesis	66	1	5.16E-01
GO:0060389	pathway-restricted SMAD protein phosphorylation	66	1	5.16E-01
GO:0060675	ureteric bud morphogenesis	66	1	5.16E-01
GO:0072577	endothelial cell apoptotic process	66	1	5.16E-01
GO:1903051	negative regulation of proteolysis involved in protein cat	66	1	5.16E-01
GO:0098800	inner mitochondrial membrane protein complex	158	2	5.16E-01
GO:0016021	integral component of membrane	5397	59	5.16E-01
GO:0048285	organelle fission	528	6	5.17E-01
GO:0010506	regulation of autophagy	344	4	5.19E-01
GO:0015103	inorganic anion transmembrane transporter activity	159	2	5.19E-01
GO:0048762	mesenchymal cell differentiation	252	3	5.20E-01
GO:0006303	double-strand break repair via nonhomologous end join	67	1	5.21E-01
GO:0006406	mRNA export from nucleus	67	1	5.21E-01
GO:0042531	positive regulation of tyrosine phosphorylation of STAT p	67	1	5.21E-01
GO:0042776	proton motive force-driven mitochondrial ATP synthesis	67	1	5.21E-01
GO:0051205	protein insertion into membrane	67	1	5.21E-01
GO:0072171	mesonephric tubule morphogenesis	67	1	5.21E-01
GO:0002039	p53 binding	67	1	5.21E-01
GO:0051147	regulation of muscle cell differentiation	160	2	5.23E-01
GO:1990845	adaptive thermogenesis	160	2	5.23E-01
GO:0006325	chromatin organization	624	7	5.23E-01
GO:0009583	detection of light stimulus	68	1	5.26E-01
GO:0030239	myofibril assembly	68	1	5.26E-01
GO:0042093	T-helper cell differentiation	68	1	5.26E-01
GO:0043550	regulation of lipid kinase activity	68	1	5.26E-01
GO:0048663	neuron fate commitment	68	1	5.26E-01
GO:0050909	sensory perception of taste	68	1	5.26E-01
GO:0070936	protein K48-linked ubiquitination	68	1	5.26E-01
GO:0048732	gland development	441	5	5.28E-01
GO:0002260	lymphocyte homeostasis	69	1	5.31E-01
GO:0010611	regulation of cardiac muscle hypertrophy	69	1	5.31E-01
GO:0048645	animal organ formation	69	1	5.31E-01
GO:0050922	negative regulation of chemotaxis	69	1	5.31E-01
GO:0055002	striated muscle cell development	69	1	5.31E-01
GO:0055021	regulation of cardiac muscle tissue growth	69	1	5.31E-01
GO:0061180	mammary gland epithelium development	69	1	5.31E-01
GO:0031228	intrinsic component of Golgi membrane	69	1	5.31E-01
GO:0034704	calcium channel complex	69	1	5.31E-01
GO:0040029	epigenetic regulation of gene expression	163	2	5.33E-01
GO:0090304	nucleic acid metabolic process	5606	61	5.33E-01
GO:0030856	regulation of epithelial cell differentiation	164	2	5.36E-01
GO:0055067	monovalent inorganic cation homeostasis	164	2	5.36E-01
GO:0000776	kinetochore	164	2	5.36E-01
GO:0042393	histone binding	258	3	5.36E-01

GO:0002294	CD4-positive, alpha-beta T cell differentiation involved i	70	1	5.36E-01
GO:0014823	response to activity	70	1	5.36E-01
GO:0032722	positive regulation of chemokine production	70	1	5.36E-01
GO:0042130	negative regulation of T cell proliferation	70	1	5.36E-01
GO:0043627	response to estrogen	70	1	5.36E-01
GO:0016811	hydrolase activity, acting on carbon-nitrogen (but not pe	70	1	5.36E-01
GO:0016874	ligase activity	165	2	5.39E-01
GO:0002287	alpha-beta T cell activation involved in immune respons	71	1	5.41E-01
GO:0002293	alpha-beta T cell differentiation involved in immune res	71	1	5.41E-01
GO:0002548	monocyte chemotaxis	71	1	5.41E-01
GO:0003208	cardiac ventricle morphogenesis	71	1	5.41E-01
GO:0032715	negative regulation of interleukin-6 production	71	1	5.41E-01
GO:0032922	circadian regulation of gene expression	71	1	5.41E-01
GO:0033344	cholesterol efflux	71	1	5.41E-01
GO:0051057	positive regulation of small GTPase mediated signal tran	71	1	5.41E-01
GO:0051310	metaphase plate congression	71	1	5.41E-01
GO:2000036	regulation of stem cell population maintenance	71	1	5.41E-01
GO:0001917	photoreceptor inner segment	71	1	5.41E-01
GO:0003684	damaged DNA binding	71	1	5.41E-01
GO:2001022	positive regulation of response to DNA damage stimulus	166	2	5.42E-01
GO:0051254	positive regulation of RNA metabolic process	1843	20	5.43E-01
GO:0016567	protein ubiquitination	824	9	5.46E-01
GO:0003205	cardiac chamber development	167	2	5.46E-01
GO:0010569	regulation of double-strand break repair via homologou	72	1	5.46E-01
GO:0014743	regulation of muscle hypertrophy	72	1	5.46E-01
GO:0033143	regulation of intracellular steroid hormone receptor sig	72	1	5.46E-01
GO:0045600	positive regulation of fat cell differentiation	72	1	5.46E-01
GO:1901880	negative regulation of protein depolymerization	72	1	5.46E-01
GO:0098982	GABA-ergic synapse	72	1	5.46E-01
GO:0000781	chromosome, telomeric region	168	2	5.49E-01
GO:0097730	non-motile cilium	168	2	5.49E-01
GO:0044255	cellular lipid metabolic process	1013	11	5.49E-01
GO:0008333	endosome to lysosome transport	73	1	5.51E-01
GO:0042246	tissue regeneration	73	1	5.51E-01
GO:0046637	regulation of alpha-beta T cell differentiation	73	1	5.51E-01
GO:0051937	catecholamine transport	73	1	5.51E-01
GO:0060415	muscle tissue morphogenesis	73	1	5.51E-01
GO:1903578	regulation of ATP metabolic process	73	1	5.51E-01
GO:0099056	integral component of presynaptic membrane	73	1	5.51E-01
GO:0000049	tRNA binding	73	1	5.51E-01
GO:0001837	epithelial to mesenchymal transition	169	2	5.52E-01
GO:0015399	primary active transmembrane transporter activity	169	2	5.52E-01
GO:0010564	regulation of cell cycle process	735	8	5.52E-01
GO:0007169	transmembrane receptor protein tyrosine kinase signali	642	7	5.53E-01
GO:0001655	urogenital system development	360	4	5.54E-01
GO:0003281	ventricular septum development	74	1	5.56E-01
GO:0030330	DNA damage response, signal transduction by p53 class	74	1	5.56E-01

GO:0032732	positive regulation of interleukin-1 production	74	1	5.56E-01
GO:0042058	regulation of epidermal growth factor receptor signaling	74	1	5.56E-01
GO:0048662	negative regulation of smooth muscle cell proliferation	74	1	5.56E-01
GO:0051298	centrosome duplication	74	1	5.56E-01
GO:0030522	intracellular receptor signaling pathway	266	3	5.57E-01
GO:0016482	cytosolic transport	171	2	5.58E-01
GO:0046661	male sex differentiation	171	2	5.58E-01
GO:0045661	regulation of myoblast differentiation	75	1	5.61E-01
GO:0050810	regulation of steroid biosynthetic process	75	1	5.61E-01
GO:0051568	histone H3-K4 methylation	75	1	5.61E-01
GO:0060337	type I interferon signaling pathway	75	1	5.61E-01
GO:0072078	nephron tubule morphogenesis	75	1	5.61E-01
GO:0140030	modification-dependent protein binding	172	2	5.62E-01
GO:0045211	postsynaptic membrane	269	3	5.64E-01
GO:0000725	recombinational repair	173	2	5.65E-01
GO:0005201	extracellular matrix structural constituent	173	2	5.65E-01
GO:0042578	phosphoric ester hydrolase activity	365	4	5.65E-01
GO:0001937	negative regulation of endothelial cell proliferation	76	1	5.66E-01
GO:0002637	regulation of immunoglobulin production	76	1	5.66E-01
GO:0009166	nucleotide catabolic process	76	1	5.66E-01
GO:0015986	proton motive force-driven ATP synthesis	76	1	5.66E-01
GO:0019226	transmission of nerve impulse	76	1	5.66E-01
GO:0060420	regulation of heart growth	76	1	5.66E-01
GO:0030684	preribosome	76	1	5.66E-01
GO:0021953	central nervous system neuron differentiation	174	2	5.68E-01
GO:0030178	negative regulation of Wnt signaling pathway	174	2	5.68E-01
GO:0031214	biomineral tissue development	174	2	5.68E-01
GO:0055088	lipid homeostasis	174	2	5.68E-01
GO:0000779	condensed chromosome, centromeric region	174	2	5.68E-01
GO:0048589	developmental growth	652	7	5.70E-01
GO:0009123	nucleoside monophosphate metabolic process	77	1	5.71E-01
GO:0046470	phosphatidylcholine metabolic process	77	1	5.71E-01
GO:0071357	cellular response to type I interferon	77	1	5.71E-01
GO:0072088	nephron epithelium morphogenesis	77	1	5.71E-01
GO:0035861	site of double-strand break	77	1	5.71E-01
GO:0030971	receptor tyrosine kinase binding	77	1	5.71E-01
GO:0009267	cellular response to starvation	175	2	5.71E-01
GO:0050680	negative regulation of epithelial cell proliferation	176	2	5.74E-01
GO:0007611	learning or memory	273	3	5.74E-01
GO:0005126	cytokine receptor binding	273	3	5.74E-01
GO:0043009	chordate embryonic development	655	7	5.75E-01
GO:0043154	negative regulation of cysteine-type endopeptidase acti	78	1	5.75E-01
GO:0070373	negative regulation of ERK1 and ERK2 cascade	78	1	5.75E-01
GO:1901568	fatty acid derivative metabolic process	78	1	5.75E-01
GO:0005254	chloride channel activity	78	1	5.75E-01
GO:0061025	membrane fusion	177	2	5.77E-01
GO:0009790	embryo development	1130	12	5.79E-01

GO:0043299	leukocyte degranulation	79	1	5.80E-01
GO:0048844	artery morphogenesis	79	1	5.80E-01
GO:0061333	renal tubule morphogenesis	79	1	5.80E-01
GO:0008076	voltage-gated potassium channel complex	79	1	5.80E-01
GO:0034987	immunoglobulin receptor binding	79	1	5.80E-01
GO:0016791	phosphatase activity	276	3	5.82E-01
GO:0016788	hydrolase activity, acting on ester bonds	755	8	5.83E-01
GO:0071824	protein-DNA complex subunit organization	277	3	5.84E-01
GO:0023061	signal release	470	5	5.84E-01
GO:0014068	positive regulation of phosphatidylinositol 3-kinase signa	80	1	5.85E-01
GO:0042440	pigment metabolic process	80	1	5.85E-01
GO:0048644	muscle organ morphogenesis	80	1	5.85E-01
GO:0070227	lymphocyte apoptotic process	80	1	5.85E-01
GO:0071230	cellular response to amino acid stimulus	80	1	5.85E-01
GO:0072028	nephron morphogenesis	80	1	5.85E-01
GO:2001259	positive regulation of cation channel activity	80	1	5.85E-01
GO:0098889	intrinsic component of presynaptic membrane	80	1	5.85E-01
GO:0035064	methylated histone binding	80	1	5.85E-01
GO:0140034	methylation-dependent protein binding	80	1	5.85E-01
GO:0099536	synaptic signaling	757	8	5.86E-01
GO:0016573	histone acetylation	180	2	5.86E-01
GO:0035265	organ growth	180	2	5.86E-01
GO:0031060	regulation of histone methylation	81	1	5.89E-01
GO:0033555	multicellular organismal response to stress	81	1	5.89E-01
GO:1901657	glycosyl compound metabolic process	81	1	5.89E-01
GO:0000118	histone deacetylase complex	81	1	5.89E-01
GO:0015030	Cajal body	81	1	5.89E-01
GO:0110148	biomineralization	181	2	5.89E-01
GO:0008022	protein C-terminus binding	181	2	5.89E-01
GO:0044772	mitotic cell cycle phase transition	473	5	5.90E-01
GO:0044843	cell cycle G1/S phase transition	280	3	5.91E-01
GO:0021766	hippocampus development	82	1	5.94E-01
GO:0042509	regulation of tyrosine phosphorylation of STAT protein	82	1	5.94E-01
GO:0043242	negative regulation of protein-containing complex disas	82	1	5.94E-01
GO:0051303	establishment of chromosome localization	82	1	5.94E-01
GO:0001936	regulation of endothelial cell proliferation	183	2	5.95E-01
GO:0007219	Notch signaling pathway	183	2	5.95E-01
GO:1901137	carbohydrate derivative biosynthetic process	669	7	5.97E-01
GO:0006094	gluconeogenesis	83	1	5.98E-01
GO:0006900	vesicle budding from membrane	83	1	5.98E-01
GO:0014032	neural crest cell development	83	1	5.98E-01
GO:0034340	response to type I interferon	83	1	5.98E-01
GO:0042310	vasoconstriction	83	1	5.98E-01
GO:0140104	molecular carrier activity	83	1	5.98E-01
GO:0035725	sodium ion transmembrane transport	184	2	5.98E-01
GO:0007059	chromosome segregation	382	4	6.01E-01
GO:0006289	nucleotide-excision repair	84	1	6.03E-01

GO:0021675	nerve development	84	1	6.03E-01
GO:0035050	embryonic heart tube development	84	1	6.03E-01
GO:0051899	membrane depolarization	84	1	6.03E-01
GO:0097194	execution phase of apoptosis	84	1	6.03E-01
GO:0005758	mitochondrial intermembrane space	84	1	6.03E-01
GO:0051606	detection of stimulus	673	7	6.03E-01
GO:0018393	internal peptidyl-lysine acetylation	186	2	6.04E-01
GO:0008306	associative learning	85	1	6.07E-01
GO:0008625	extrinsic apoptotic signaling pathway via death domain r	85	1	6.07E-01
GO:0021954	central nervous system neuron development	85	1	6.07E-01
GO:0046785	microtubule polymerization	85	1	6.07E-01
GO:0051453	regulation of intracellular pH	85	1	6.07E-01
GO:1901292	nucleoside phosphate catabolic process	85	1	6.07E-01
GO:0051117	ATPase binding	85	1	6.07E-01
GO:0030324	lung development	187	2	6.07E-01
GO:0009792	embryo development ending in birth or egg hatching	677	7	6.10E-01
GO:0006475	internal protein amino acid acetylation	188	2	6.10E-01
GO:0007548	sex differentiation	288	3	6.10E-01
GO:0002088	lens development in camera-type eye	86	1	6.11E-01
GO:0007260	tyrosine phosphorylation of STAT protein	86	1	6.11E-01
GO:0019319	hexose biosynthetic process	86	1	6.11E-01
GO:0034243	regulation of transcription elongation by RNA polymeras	86	1	6.11E-01
GO:0005581	collagen trimer	86	1	6.11E-01
GO:0090092	regulation of transmembrane receptor protein serine/th	290	3	6.15E-01
GO:0008543	fibroblast growth factor receptor signaling pathway	87	1	6.15E-01
GO:0010507	negative regulation of autophagy	87	1	6.15E-01
GO:0031110	regulation of microtubule polymerization or depolymeri	87	1	6.15E-01
GO:0046879	hormone secretion	291	3	6.17E-01
GO:0030323	respiratory tube development	191	2	6.19E-01
GO:0003714	transcription corepressor activity	191	2	6.19E-01
GO:0043367	CD4-positive, alpha-beta T cell differentiation	88	1	6.20E-01
GO:0072384	organelle transport along microtubule	88	1	6.20E-01
GO:2000781	positive regulation of double-strand break repair	88	1	6.20E-01
GO:0008081	phosphoric diester hydrolase activity	88	1	6.20E-01
GO:0009755	hormone-mediated signaling pathway	192	2	6.22E-01
GO:0007589	body fluid secretion	89	1	6.24E-01
GO:0034644	cellular response to UV	89	1	6.24E-01
GO:0048864	stem cell development	89	1	6.24E-01
GO:0050000	chromosome localization	89	1	6.24E-01
GO:0071229	cellular response to acid chemical	89	1	6.24E-01
GO:0030117	membrane coat	89	1	6.24E-01
GO:0034705	potassium channel complex	89	1	6.24E-01
GO:0048475	coated membrane	89	1	6.24E-01
GO:0048839	inner ear development	193	2	6.24E-01
GO:0009060	aerobic respiration	194	2	6.27E-01
GO:0030641	regulation of cellular pH	90	1	6.28E-01
GO:0045995	regulation of embryonic development	90	1	6.28E-01

GO:0070603	SWI/SNF superfamily-type complex	90	1	6.28E-01
GO:0001942	hair follicle development	91	1	6.32E-01
GO:1901216	positive regulation of neuron death	91	1	6.32E-01
GO:1903351	cellular response to dopamine	91	1	6.32E-01
GO:1990542	mitochondrial transmembrane transport	91	1	6.32E-01
GO:0018394	peptidyl-lysine acetylation	197	2	6.35E-01
GO:0001843	neural tube closure	92	1	6.36E-01
GO:0010657	muscle cell apoptotic process	92	1	6.36E-01
GO:0030071	regulation of mitotic metaphase/anaphase transition	92	1	6.36E-01
GO:0072091	regulation of stem cell proliferation	92	1	6.36E-01
GO:1903350	response to dopamine	92	1	6.36E-01
GO:0005684	U2-type spliceosomal complex	92	1	6.36E-01
GO:0032580	Golgi cisterna membrane	92	1	6.36E-01
GO:0032592	integral component of mitochondrial membrane	92	1	6.36E-01
GO:0006302	double-strand break repair	300	3	6.38E-01
GO:0009914	hormone transport	300	3	6.38E-01
GO:0000422	autophagy of mitochondrion	93	1	6.40E-01
GO:0030516	regulation of axon extension	93	1	6.40E-01
GO:0045666	positive regulation of neuron differentiation	93	1	6.40E-01
GO:0050886	endocrine process	93	1	6.40E-01
GO:0055017	cardiac muscle tissue growth	93	1	6.40E-01
GO:0060606	tube closure	93	1	6.40E-01
GO:0061726	mitochondrion disassembly	93	1	6.40E-01
GO:0051213	dioxygenase activity	93	1	6.40E-01
GO:0001935	endothelial cell proliferation	199	2	6.41E-01
GO:0007186	G protein-coupled receptor signaling pathway	1278	13	6.42E-01
GO:0048511	rhythmic process	302	3	6.42E-01
GO:0022404	molting cycle process	94	1	6.44E-01
GO:0022405	hair cycle process	94	1	6.44E-01
GO:1900542	regulation of purine nucleotide metabolic process	94	1	6.44E-01
GO:0098573	intrinsic component of mitochondrial membrane	94	1	6.44E-01
GO:1904949	ATPase complex	94	1	6.44E-01
GO:0004553	hydrolase activity, hydrolyzing O-glycosyl compounds	94	1	6.44E-01
GO:0000245	spliceosomal complex assembly	95	1	6.48E-01
GO:0007091	metaphase/anaphase transition of mitotic cell cycle	95	1	6.48E-01
GO:0032088	negative regulation of NF-kappaB transcription factor ac	95	1	6.48E-01
GO:0043648	dicarboxylic acid metabolic process	95	1	6.48E-01
GO:0098773	skin epidermis development	95	1	6.48E-01
GO:1902099	regulation of metaphase/anaphase transition of cell cycl	95	1	6.48E-01
GO:0097525	spliceosomal snRNP complex	95	1	6.48E-01
GO:0032446	protein modification by small protein conjugation	898	9	6.49E-01
GO:0051056	regulation of small GTPase mediated signal transduction	305	3	6.49E-01
GO:0048608	reproductive structure development	306	3	6.51E-01
GO:0001763	morphogenesis of a branching structure	203	2	6.52E-01
GO:0003073	regulation of systemic arterial blood pressure	96	1	6.52E-01
GO:0014033	neural crest cell differentiation	96	1	6.52E-01
GO:0051153	regulation of striated muscle cell differentiation	96	1	6.52E-01

GO:0072080	nephron tubule development	96	1	6.52E-01
GO:0019888	protein phosphatase regulator activity	96	1	6.52E-01
GO:0000070	mitotic sister chromatid segregation	204	2	6.54E-01
GO:0050864	regulation of B cell activation	204	2	6.54E-01
GO:0046914	transition metal ion binding	1097	11	6.55E-01
GO:0001657	ureteric bud development	97	1	6.56E-01
GO:0043502	regulation of muscle adaptation	97	1	6.56E-01
GO:0060993	kidney morphogenesis	97	1	6.56E-01
GO:0007204	positive regulation of cytosolic calcium ion concentratio	205	2	6.57E-01
GO:0010965	regulation of mitotic sister chromatid separation	98	1	6.59E-01
GO:0014020	primary neural tube formation	98	1	6.59E-01
GO:0044784	metaphase/anaphase transition of cell cycle	98	1	6.59E-01
GO:0060349	bone morphogenesis	98	1	6.59E-01
GO:0072163	mesonephric epithelium development	98	1	6.59E-01
GO:0072164	mesonephric tubule development	98	1	6.59E-01
GO:0120162	positive regulation of cold-induced thermogenesis	98	1	6.59E-01
GO:0004725	protein tyrosine phosphatase activity	98	1	6.59E-01
GO:0004867	serine-type endopeptidase inhibitor activity	98	1	6.59E-01
GO:0061458	reproductive system development	310	3	6.60E-01
GO:0048167	regulation of synaptic plasticity	207	2	6.62E-01
GO:0003300	cardiac muscle hypertrophy	99	1	6.63E-01
GO:0006140	regulation of nucleotide metabolic process	99	1	6.63E-01
GO:0006885	regulation of pH	99	1	6.63E-01
GO:0010389	regulation of G2/M transition of mitotic cell cycle	99	1	6.63E-01
GO:0030101	natural killer cell activation	99	1	6.63E-01
GO:0043279	response to alkaloid	99	1	6.63E-01
GO:0061326	renal tubule development	99	1	6.63E-01
GO:2001020	regulation of response to DNA damage stimulus	312	3	6.64E-01
GO:0004518	nuclease activity	208	2	6.65E-01
GO:0060485	mesenchyme development	313	3	6.66E-01
GO:0001776	leukocyte homeostasis	100	1	6.67E-01
GO:1901992	positive regulation of mitotic cell cycle phase transition	100	1	6.67E-01
GO:0005643	nuclear pore	100	1	6.67E-01
GO:0007167	enzyme-linked receptor protein signaling pathway	1010	10	6.67E-01
GO:0050890	cognition	314	3	6.68E-01
GO:0007623	circadian rhythm	210	2	6.70E-01
GO:0021549	cerebellum development	101	1	6.70E-01
GO:0051306	mitotic sister chromatid separation	101	1	6.70E-01
GO:0042594	response to starvation	211	2	6.72E-01
GO:0060541	respiratory system development	211	2	6.72E-01
GO:0001823	mesonephros development	102	1	6.74E-01
GO:0008630	intrinsic apoptotic signaling pathway in response to DNA	102	1	6.74E-01
GO:0014897	striated muscle hypertrophy	102	1	6.74E-01
GO:0019218	regulation of steroid metabolic process	102	1	6.74E-01
GO:0031058	positive regulation of histone modification	102	1	6.74E-01
GO:0060419	heart growth	102	1	6.74E-01
GO:0005881	cytoplasmic microtubule	102	1	6.74E-01

GO:0006282	regulation of DNA repair	212	2	6.75E-01
GO:0042472	inner ear morphogenesis	103	1	6.78E-01
GO:0043255	regulation of carbohydrate biosynthetic process	103	1	6.78E-01
GO:0008270	zinc ion binding	823	8	6.79E-01
GO:0046474	glycerophospholipid biosynthetic process	214	2	6.80E-01
GO:0014896	muscle hypertrophy	104	1	6.81E-01
GO:0015914	phospholipid transport	104	1	6.81E-01
GO:0032784	regulation of DNA-templated transcription elongation	104	1	6.81E-01
GO:0046425	regulation of receptor signaling pathway via JAK-STAT	104	1	6.81E-01
GO:0010558	negative regulation of macromolecule biosynthetic proc	1604	16	6.81E-01
GO:0009416	response to light stimulus	321	3	6.83E-01
GO:0007268	chemical synaptic transmission	727	7	6.83E-01
GO:0098916	anterograde trans-synaptic signaling	727	7	6.83E-01
GO:0008585	female gonad development	105	1	6.85E-01
GO:0071868	cellular response to monoamine stimulus	105	1	6.85E-01
GO:0071870	cellular response to catecholamine stimulus	105	1	6.85E-01
GO:0016605	PML body	105	1	6.85E-01
GO:0030532	small nuclear ribonucleoprotein complex	105	1	6.85E-01
GO:0001841	neural tube formation	106	1	6.88E-01
GO:0005796	Golgi lumen	106	1	6.88E-01
GO:0051253	negative regulation of RNA metabolic process	1418	14	6.88E-01
GO:1901215	negative regulation of neuron death	218	2	6.90E-01
GO:0002444	myeloid leukocyte mediated immunity	107	1	6.92E-01
GO:0003279	cardiac septum development	107	1	6.92E-01
GO:0060840	artery development	107	1	6.92E-01
GO:0015108	chloride transmembrane transporter activity	107	1	6.92E-01
GO:0099537	trans-synaptic signaling	734	7	6.93E-01
GO:0032526	response to retinoic acid	108	1	6.95E-01
GO:0034308	primary alcohol metabolic process	108	1	6.95E-01
GO:0061387	regulation of extent of cell growth	108	1	6.95E-01
GO:0071867	response to monoamine	108	1	6.95E-01
GO:0071869	response to catecholamine	108	1	6.95E-01
GO:0043583	ear development	221	2	6.97E-01
GO:0001708	cell fate specification	109	1	6.98E-01
GO:0006368	transcription elongation by RNA polymerase II promoter	109	1	6.98E-01
GO:0046545	development of primary female sexual characteristics	109	1	6.98E-01
GO:0001221	transcription coregulator binding	109	1	6.98E-01
GO:0044242	cellular lipid catabolic process	222	2	6.99E-01
GO:0070647	protein modification by small protein conjugation or ren	1039	10	7.01E-01
GO:0007173	epidermal growth factor receptor signaling pathway	110	1	7.02E-01
GO:0019395	fatty acid oxidation	110	1	7.02E-01
GO:0045833	negative regulation of lipid metabolic process	110	1	7.02E-01
GO:1904035	regulation of epithelial cell apoptotic process	110	1	7.02E-01
GO:0038023	signaling receptor activity	1531	15	7.04E-01
GO:0060089	molecular transducer activity	1531	15	7.04E-01
GO:0048638	regulation of developmental growth	332	3	7.05E-01
GO:0014066	regulation of phosphatidylinositol 3-kinase signaling	111	1	7.05E-01

GO:0022037	metencephalon development	111	1	7.05E-01
GO:0030510	regulation of BMP signaling pathway	111	1	7.05E-01
GO:1905818	regulation of chromosome separation	111	1	7.05E-01
GO:0004222	metalloendopeptidase activity	112	1	7.08E-01
GO:0034470	ncRNA processing	439	4	7.08E-01
GO:0046777	protein autophosphorylation	226	2	7.09E-01
GO:0060562	epithelial tube morphogenesis	335	3	7.10E-01
GO:0048705	skeletal system morphogenesis	227	2	7.11E-01
GO:0016042	lipid catabolic process	336	3	7.12E-01
GO:0030004	cellular monovalent inorganic cation homeostasis	114	1	7.14E-01
GO:0019208	phosphatase regulator activity	114	1	7.14E-01
GO:0090287	regulation of cellular response to growth factor stimulus	338	3	7.16E-01
GO:0001938	positive regulation of endothelial cell proliferation	115	1	7.18E-01
GO:0032414	positive regulation of ion transmembrane transporter ac	115	1	7.18E-01
GO:0035710	CD4-positive, alpha-beta T cell activation	115	1	7.18E-01
GO:1904892	regulation of receptor signaling pathway via STAT	115	1	7.18E-01
GO:0099634	postsynaptic specialization membrane	115	1	7.18E-01
GO:0006473	protein acetylation	230	2	7.18E-01
GO:0062012	regulation of small molecule metabolic process	339	3	7.18E-01
GO:0005216	ion channel activity	446	4	7.20E-01
GO:0031669	cellular response to nutrient levels	231	2	7.20E-01
GO:0050769	positive regulation of neurogenesis	231	2	7.20E-01
GO:0002065	columnar/cuboidal epithelial cell differentiation	116	1	7.21E-01
GO:0034440	lipid oxidation	116	1	7.21E-01
GO:0120114	Sm-like protein family complex	116	1	7.21E-01
GO:0000079	regulation of cyclin-dependent protein serine/threonine	117	1	7.24E-01
GO:0043406	positive regulation of MAP kinase activity	117	1	7.24E-01
GO:0006805	xenobiotic metabolic process	118	1	7.27E-01
GO:0007088	regulation of mitotic nuclear division	118	1	7.27E-01
GO:0019751	polyol metabolic process	118	1	7.27E-01
GO:0099055	integral component of postsynaptic membrane	118	1	7.27E-01
GO:0006906	vesicle fusion	119	1	7.30E-01
GO:0005262	calcium channel activity	119	1	7.30E-01
GO:0043169	cation binding	4337	44	7.30E-01
GO:0046872	metal ion binding	4246	43	7.32E-01
GO:0042752	regulation of circadian rhythm	120	1	7.33E-01
GO:0090288	negative regulation of cellular response to growth factor	120	1	7.33E-01
GO:1904029	regulation of cyclin-dependent protein kinase activity	120	1	7.33E-01
GO:0005125	cytokine activity	237	2	7.33E-01
GO:0019866	organelle inner membrane	560	5	7.34E-01
GO:0090174	organelle membrane fusion	121	1	7.36E-01
GO:0000819	sister chromatid segregation	239	2	7.37E-01
GO:0006476	protein deacetylation	122	1	7.39E-01
GO:0043500	muscle adaptation	122	1	7.39E-01
GO:1902554	serine/threonine protein kinase complex	122	1	7.39E-01
GO:0045333	cellular respiration	240	2	7.39E-01
GO:0022612	gland morphogenesis	123	1	7.41E-01

GO:0071482	cellular response to light stimulus	123	1	7.41E-01
GO:0090630	activation of GTPase activity	123	1	7.41E-01
GO:0098936	intrinsic component of postsynaptic membrane	123	1	7.41E-01
GO:0006354	DNA-templated transcription elongation	241	2	7.41E-01
GO:0031327	negative regulation of cellular biosynthetic process	1673	16	7.44E-01
GO:0031109	microtubule polymerization or depolymerization	124	1	7.44E-01
GO:0042471	ear morphogenesis	124	1	7.44E-01
GO:0046660	female sex differentiation	124	1	7.44E-01
GO:0003206	cardiac chamber morphogenesis	126	1	7.50E-01
GO:0032411	positive regulation of transporter activity	126	1	7.50E-01
GO:0016779	nucleotidyltransferase activity	126	1	7.50E-01
GO:0000209	protein polyubiquitination	246	2	7.52E-01
GO:0001838	embryonic epithelial tube formation	127	1	7.53E-01
GO:0019783	ubiquitin-like protein peptidase activity	127	1	7.53E-01
GO:0000228	nuclear chromosome	249	2	7.58E-01
GO:0045931	positive regulation of mitotic cell cycle	129	1	7.58E-01
GO:0051224	negative regulation of protein transport	129	1	7.58E-01
GO:0032874	positive regulation of stress-activated MAPK cascade	130	1	7.61E-01
GO:0034968	histone lysine methylation	130	1	7.61E-01
GO:0042542	response to hydrogen peroxide	130	1	7.61E-01
GO:0006355	regulation of DNA-templated transcription	3443	34	7.63E-01
GO:1903506	regulation of nucleic acid-templated transcription	3445	34	7.64E-01
GO:0090305	nucleic acid phosphodiester bond hydrolysis	253	2	7.65E-01
GO:0070304	positive regulation of stress-activated protein kinase sig	132	1	7.66E-01
GO:0016798	hydrolase activity, acting on glycosyl bonds	132	1	7.66E-01
GO:0045017	glycerolipid biosynthetic process	254	2	7.67E-01
GO:0035601	protein deacylation	133	1	7.68E-01
GO:1904950	negative regulation of establishment of protein localizat	133	1	7.68E-01
GO:0006814	sodium ion transport	255	2	7.69E-01
GO:2001141	regulation of RNA biosynthetic process	3454	34	7.70E-01
GO:0006612	protein targeting to membrane	134	1	7.71E-01
GO:0043467	regulation of generation of precursor metabolites and e	134	1	7.71E-01
GO:0007098	centrosome cycle	135	1	7.73E-01
GO:0042476	odontogenesis	135	1	7.73E-01
GO:0051304	chromosome separation	135	1	7.73E-01
GO:2000779	regulation of double-strand break repair	135	1	7.73E-01
GO:0006281	DNA repair	591	5	7.76E-01
GO:0006275	regulation of DNA replication	136	1	7.76E-01
GO:0071333	cellular response to glucose stimulus	136	1	7.76E-01
GO:1902911	protein kinase complex	136	1	7.76E-01
GO:0015078	proton transmembrane transporter activity	136	1	7.76E-01
GO:0008654	phospholipid biosynthetic process	259	2	7.77E-01
GO:0098732	macromolecule deacylation	137	1	7.78E-01
GO:1904019	epithelial cell apoptotic process	137	1	7.78E-01
GO:0071331	cellular response to hexose stimulus	138	1	7.81E-01
GO:0004713	protein tyrosine kinase activity	138	1	7.81E-01
GO:0031668	cellular response to extracellular stimulus	262	2	7.82E-01

GO:0022857	transmembrane transporter activity	1118	10	7.83E-01
GO:0003682	chromatin binding	597	5	7.83E-01
GO:0006997	nucleus organization	139	1	7.83E-01
GO:0072175	epithelial tube formation	139	1	7.83E-01
GO:1903008	organelle disassembly	139	1	7.83E-01
GO:0016922	nuclear receptor binding	139	1	7.83E-01
GO:0071326	cellular response to monosaccharide stimulus	140	1	7.86E-01
GO:0005215	transporter activity	1225	11	7.87E-01
GO:0046328	regulation of JNK cascade	141	1	7.88E-01
GO:0090090	negative regulation of canonical Wnt signaling pathway	141	1	7.88E-01
GO:0140098	catalytic activity, acting on RNA	384	3	7.92E-01
GO:0071363	cellular response to growth factor stimulus	712	6	7.92E-01
GO:0018022	peptidyl-lysine methylation	144	1	7.95E-01
GO:0005743	mitochondrial inner membrane	498	4	7.95E-01
GO:0014065	phosphatidylinositol 3-kinase signaling	145	1	7.97E-01
GO:0051783	regulation of nuclear division	145	1	7.97E-01
GO:0043543	protein acylation	272	2	7.99E-01
GO:0000793	condensed chromosome	273	2	8.01E-01
GO:0017015	regulation of transforming growth factor beta receptor s	147	1	8.02E-01
GO:0035264	multicellular organism growth	147	1	8.02E-01
GO:0045598	regulation of fat cell differentiation	147	1	8.02E-01
GO:0106106	cold-induced thermogenesis	147	1	8.02E-01
GO:0120161	regulation of cold-induced thermogenesis	147	1	8.02E-01
GO:0008528	G protein-coupled peptide receptor activity	147	1	8.02E-01
GO:0048598	embryonic morphogenesis	614	5	8.03E-01
GO:0044272	sulfur compound biosynthetic process	148	1	8.04E-01
GO:0140375	immune receptor activity	148	1	8.04E-01
GO:0045934	negative regulation of nucleobase-containing compound	1550	14	8.04E-01
GO:0009411	response to UV	149	1	8.06E-01
GO:0019842	vitamin binding	149	1	8.06E-01
GO:0016070	RNA metabolic process	5053	50	8.07E-01
GO:0007612	learning	150	1	8.08E-01
GO:0010821	regulation of mitochondrion organization	150	1	8.08E-01
GO:1903844	regulation of cellular response to transforming growth fa	150	1	8.08E-01
GO:0022853	active ion transmembrane transporter activity	278	2	8.09E-01
GO:0140096	catalytic activity, acting on a protein	2257	21	8.10E-01
GO:0051962	positive regulation of nervous system development	279	2	8.11E-01
GO:0062013	positive regulation of small molecule metabolic process	152	1	8.12E-01
GO:0071322	cellular response to carbohydrate stimulus	152	1	8.12E-01
GO:0016331	morphogenesis of embryonic epithelium	153	1	8.14E-01
GO:0001653	peptide receptor activity	153	1	8.14E-01
GO:0005506	iron ion binding	153	1	8.14E-01
GO:0005730	nucleolus	1463	13	8.15E-01
GO:0045834	positive regulation of lipid metabolic process	154	1	8.16E-01
GO:0050905	neuromuscular process	154	1	8.16E-01
GO:0010675	regulation of cellular carbohydrate metabolic process	155	1	8.18E-01
GO:0010817	regulation of hormone levels	517	4	8.18E-01

GO:0007610	behavior	629	5	8.19E-01
GO:0050770	regulation of axonogenesis	156	1	8.20E-01
GO:0006260	DNA replication	286	2	8.21E-01
GO:0048754	branching morphogenesis of an epithelial tube	157	1	8.22E-01
GO:0070848	response to growth factor	742	6	8.23E-01
GO:0045182	translation regulator activity	407	3	8.24E-01
GO:0001678	cellular glucose homeostasis	158	1	8.24E-01
GO:0090101	negative regulation of transmembrane receptor protein	158	1	8.24E-01
GO:0034660	ncRNA metabolic process	634	5	8.25E-01
GO:0006351	DNA-templated transcription	3557	34	8.25E-01
GO:0097659	nucleic acid-templated transcription	3559	34	8.26E-01
GO:0007605	sensory perception of sound	159	1	8.26E-01
GO:1902600	proton transmembrane transport	160	1	8.28E-01
GO:0018205	peptidyl-lysine modification	414	3	8.32E-01
GO:0032774	RNA biosynthetic process	3576	34	8.34E-01
GO:0098798	mitochondrial protein-containing complex	295	2	8.35E-01
GO:0021915	neural tube development	164	1	8.36E-01
GO:0034702	ion channel complex	296	2	8.36E-01
GO:0046943	carboxylic acid transmembrane transporter activity	165	1	8.37E-01
GO:0005342	organic acid transmembrane transporter activity	166	1	8.39E-01
GO:0016571	histone methylation	167	1	8.41E-01
GO:0030509	BMP signaling pathway	167	1	8.41E-01
GO:0000724	double-strand break repair via homologous recombinati	169	1	8.44E-01
GO:0050806	positive regulation of synaptic transmission	170	1	8.46E-01
GO:0007254	JNK cascade	172	1	8.49E-01
GO:0007259	receptor signaling pathway via JAK-STAT	172	1	8.49E-01
GO:0004252	serine-type endopeptidase activity	174	1	8.53E-01
GO:0010469	regulation of signaling receptor activity	175	1	8.54E-01
GO:0031056	regulation of histone modification	175	1	8.54E-01
GO:0070646	protein modification by small protein removal	176	1	8.56E-01
GO:1901136	carbohydrate derivative catabolic process	176	1	8.56E-01
GO:0019827	stem cell population maintenance	177	1	8.58E-01
GO:0004721	phosphoprotein phosphatase activity	177	1	8.58E-01
GO:0001659	temperature homeostasis	178	1	8.59E-01
GO:0048015	phosphatidylinositol-mediated signaling	178	1	8.59E-01
GO:0048736	appendage development	178	1	8.59E-01
GO:0060173	limb development	178	1	8.59E-01
GO:0071772	response to BMP	178	1	8.59E-01
GO:0071773	cellular response to BMP stimulus	178	1	8.59E-01
GO:0016705	oxidoreductase activity, acting on paired donors, with in	179	1	8.61E-01
GO:0050954	sensory perception of mechanical stimulus	180	1	8.62E-01
GO:0042770	signal transduction in response to DNA damage	181	1	8.64E-01
GO:0098727	maintenance of cell number	181	1	8.64E-01
GO:0046890	regulation of lipid biosynthetic process	182	1	8.65E-01
GO:0048017	inositol lipid-mediated signaling	182	1	8.65E-01
GO:0097696	receptor signaling pathway via STAT	183	1	8.67E-01
GO:2000045	regulation of G1/S transition of mitotic cell cycle	185	1	8.70E-01

GO:0006109	regulation of carbohydrate metabolic process	186	1	8.71E-01
GO:0048469	cell maturation	187	1	8.72E-01
GO:0045944	positive regulation of transcription by RNA polymerase I	1237	10	8.73E-01
GO:0061138	morphogenesis of a branching epithelium	188	1	8.74E-01
GO:1901987	regulation of cell cycle phase transition	453	3	8.74E-01
GO:0008236	serine-type peptidase activity	192	1	8.79E-01
GO:0032504	multicellular organism reproduction	922	7	8.80E-01
GO:0015980	energy derivation by oxidation of organic compounds	333	2	8.81E-01
GO:0017171	serine hydrolase activity	196	1	8.85E-01
GO:0007626	locomotory behavior	197	1	8.86E-01
GO:0140640	catalytic activity, acting on a nucleic acid	592	4	8.90E-01
GO:0045893	positive regulation of DNA-templated transcription	1692	14	8.90E-01
GO:1903508	positive regulation of nucleic acid-templated transcriptio	1692	14	8.90E-01
GO:0007283	spermatogenesis	593	4	8.91E-01
GO:1903828	negative regulation of protein localization	203	1	8.93E-01
GO:1902680	positive regulation of RNA biosynthetic process	1698	14	8.93E-01
GO:0005261	cation channel activity	346	2	8.94E-01
GO:0019216	regulation of lipid metabolic process	347	2	8.95E-01
GO:0006479	protein methylation	208	1	8.99E-01
GO:0008213	protein alkylation	208	1	8.99E-01
GO:0016050	vesicle organization	353	2	9.00E-01
GO:1901990	regulation of mitotic cell cycle phase transition	354	2	9.01E-01
GO:1903046	meiotic cell cycle process	212	1	9.03E-01
GO:1902806	regulation of cell cycle G1/S phase transition	213	1	9.04E-01
GO:0048232	male gamete generation	613	4	9.05E-01
GO:0007179	transforming growth factor beta receptor signaling path	216	1	9.07E-01
GO:0015318	inorganic molecular entity transmembrane transporter a	743	5	9.11E-01
GO:0016747	acyltransferase activity, transferring groups other than a	221	1	9.12E-01
GO:0034703	cation channel complex	222	1	9.13E-01
GO:0005085	guanyl-nucleotide exchange factor activity	224	1	9.15E-01
GO:0016741	transferase activity, transferring one-carbon groups	225	1	9.16E-01
GO:1902495	transmembrane transporter complex	379	2	9.21E-01
GO:0006974	cellular response to DNA damage stimulus	879	6	9.21E-01
GO:0048609	multicellular organismal reproductive process	882	6	9.23E-01
GO:0007276	gamete generation	765	5	9.23E-01
GO:0007411	axon guidance	236	1	9.26E-01
GO:0097485	neuron projection guidance	237	1	9.27E-01
GO:0042445	hormone metabolic process	241	1	9.30E-01
GO:0045444	fat cell differentiation	244	1	9.32E-01
GO:0070085	glycosylation	244	1	9.32E-01
GO:0016197	endosomal transport	247	1	9.34E-01
GO:0042593	glucose homeostasis	248	1	9.35E-01
GO:0016746	acyltransferase activity	248	1	9.35E-01
GO:0033500	carbohydrate homeostasis	249	1	9.36E-01
GO:0090575	RNA polymerase II transcription regulator complex	249	1	9.36E-01
GO:1990351	transporter complex	405	2	9.37E-01
GO:0022804	active transmembrane transporter activity	417	2	9.44E-01

GO:0004672	protein kinase activity	565	3	9.48E-01
GO:0071560	cellular response to transforming growth factor beta sti	271	1	9.50E-01
GO:0022412	cellular process involved in reproduction in multicellular	430	2	9.50E-01
GO:0090596	sensory organ morphogenesis	272	1	9.50E-01
GO:0007423	sensory organ development	573	3	9.51E-01
GO:0061448	connective tissue development	274	1	9.51E-01
GO:0005096	GTPase activator activity	275	1	9.52E-01
GO:0046873	metal ion transmembrane transporter activity	436	2	9.53E-01
GO:0003006	developmental process involved in reproduction	959	6	9.53E-01
GO:0071559	response to transforming growth factor beta	277	1	9.53E-01
GO:0005694	chromosome	1880	14	9.55E-01
GO:0004842	ubiquitin-protein transferase activity	441	2	9.55E-01
GO:0051321	meiotic cell cycle	285	1	9.57E-01
GO:0048568	embryonic organ development	449	2	9.58E-01
GO:0016301	kinase activity	728	4	9.59E-01
GO:0048562	embryonic organ morphogenesis	294	1	9.61E-01
GO:0061695	transferase complex, transferring phosphorus-containin	295	1	9.62E-01
GO:0044262	cellular carbohydrate metabolic process	296	1	9.62E-01
GO:0022890	inorganic cation transmembrane transporter activity	607	3	9.63E-01
GO:0019787	ubiquitin-like protein transferase activity	465	2	9.64E-01
GO:0016772	transferase activity, transferring phosphorus-containing	876	5	9.65E-01
GO:0016570	histone modification	474	2	9.67E-01
GO:0000151	ubiquitin ligase complex	309	1	9.67E-01
GO:0010948	negative regulation of cell cycle process	318	1	9.70E-01
GO:0030695	GTPase regulator activity	488	2	9.71E-01
GO:0060589	nucleoside-triphosphatase regulator activity	488	2	9.71E-01
GO:0061630	ubiquitin protein ligase activity	322	1	9.72E-01
GO:0001227	DNA-binding transcription repressor activity, RNA polym	323	1	9.72E-01
GO:0048018	receptor ligand activity	491	2	9.72E-01
GO:0001217	DNA-binding transcription repressor activity	327	1	9.73E-01
GO:0030546	signaling receptor activator activity	498	2	9.74E-01
GO:0061659	ubiquitin-like protein ligase activity	334	1	9.75E-01
GO:0045892	negative regulation of DNA-templated transcription	1302	8	9.76E-01
GO:0008324	cation transmembrane transporter activity	656	3	9.76E-01
GO:1903507	negative regulation of nucleic acid-templated transcripti	1304	8	9.76E-01
GO:1902679	negative regulation of RNA biosynthetic process	1306	8	9.76E-01
GO:0000785	chromatin	1306	8	9.76E-01
GO:0043010	camera-type eye development	340	1	9.77E-01
GO:0009593	detection of chemical stimulus	512	2	9.77E-01
GO:0043414	macromolecule methylation	348	1	9.79E-01
GO:0016773	phosphotransferase activity, alcohol group as acceptor	672	3	9.79E-01
GO:0000122	negative regulation of transcription by RNA polymerase	956	5	9.80E-01
GO:0060271	cilium assembly	362	1	9.82E-01
GO:0007606	sensory perception of chemical stimulus	537	2	9.82E-01
GO:0106310	protein serine kinase activity	363	1	9.82E-01
GO:1990234	transferase complex	839	4	9.83E-01
GO:0030545	signaling receptor regulator activity	546	2	9.83E-01

GO:0003677	DNA binding	2522	18	9.84E-01
GO:0001654	eye development	388	1	9.86E-01
GO:0032259	methylation	390	1	9.87E-01
GO:0044782	cilium organization	391	1	9.87E-01
GO:0150063	visual system development	392	1	9.87E-01
GO:0009100	glycoprotein metabolic process	395	1	9.87E-01
GO:0048880	sensory system development	398	1	9.88E-01
GO:0045786	negative regulation of cell cycle	405	1	9.89E-01
GO:0016442	RISC complex	413	1	9.90E-01
GO:0031332	RNAi effector complex	413	1	9.90E-01
GO:0004888	transmembrane signaling receptor activity	1309	7	9.90E-01
GO:0004674	protein serine/threonine kinase activity	430	1	9.91E-01
GO:0007608	sensory perception of smell	457	1	9.94E-01
GO:0006357	regulation of transcription by RNA polymerase II	2581	17	9.94E-01
GO:0043565	sequence-specific DNA binding	1689	9	9.96E-01
GO:0031047	gene silencing by RNA	690	2	9.96E-01
GO:0005667	transcription regulator complex	509	1	9.96E-01
GO:0006366	transcription by RNA polymerase II	2677	17	9.97E-01
GO:0016740	transferase activity	2242	13	9.97E-01
GO:0007600	sensory perception	1006	3	9.99E-01
GO:0035195	miRNA-mediated gene silencing	626	1	9.99E-01
GO:0035194	post-transcriptional gene silencing by RNA	634	1	9.99E-01
GO:0016441	post-transcriptional gene silencing	639	1	9.99E-01
GO:0050877	nervous system process	1541	6	9.99E-01
GO:1990837	sequence-specific double-stranded DNA binding	1587	6	1.00E+00
GO:0000976	transcription cis-regulatory region binding	1529	5	1.00E+00
GO:0001067	transcription regulatory region nucleic acid binding	1531	5	1.00E+00
GO:0003690	double-stranded DNA binding	1686	6	1.00E+00
GO:0004930	G protein-coupled receptor activity	868	1	1.00E+00
GO:0140110	transcription regulator activity	1939	7	1.00E+00
GO:0000978	RNA polymerase II cis-regulatory region sequence-specif	1213	2	1.00E+00
GO:0000977	RNA polymerase II transcription regulatory region seque	1431	3	1.00E+00
GO:0000987	cis-regulatory region sequence-specific DNA binding	1234	2	1.00E+00
GO:0000981	DNA-binding transcription factor activity, RNA polymera	1382	2	1.00E+00
GO:0003700	DNA-binding transcription factor activity	1430	2	1.00E+00

FDR	OR	ProteinName
6.32E-82	16.30	ACTB, ADAM10, ADH5, ALDOA, ANPEP, ANXA11, ANXA2, ANXA5, ANXA7, ARPC5, ATIC, A
2.72E-79	15.49	ACTB, ADAM10, ADH5, ALDOA, ANPEP, ANXA11, ANXA2, ANXA5, ANXA7, ARPC5, ATIC, A
2.89E-79	15.48	ACTB, ADAM10, ADH5, ALDOA, ANPEP, ANXA11, ANXA2, ANXA5, ANXA7, ARPC5, ATIC, A
2.89E-79	15.48	ACTB, ADAM10, ADH5, ALDOA, ANPEP, ANXA11, ANXA2, ANXA5, ANXA7, ARPC5, ATIC, A
2.77E-66	12.03	ACTB, ADAM10, ADH5, ALDOA, ANPEP, ANTXR2, ANXA11, ANXA2, ANXA5, ANXA7, ARPC
7.32E-59	10.18	ACTB, ADAM10, ADH5, ALDOA, ANPEP, ANXA11, ANXA2, ANXA5, ANXA7, ARPC5, ATIC, A
8.00E-50	8.47	ACTB, ADAM10, ADH5, ALDOA, ANPEP, ANTXR2, ANXA11, ANXA2, ANXA5, ANXA7, ARPC
1.40E-36	10.78	ADAM10, ALDOA, ANPEP, ANXA11, ANXA2, ANXA7, ARPC5, B2M, CAMP, CCT2, CCT8, CD
1.45E-33	9.22	ADAM10, ALDOA, ANPEP, ANXA11, ANXA2, ANXA7, ARPC5, B2M, CALM3, CAMP, CCT2, C
2.91E-27	12.92	ACTB, ADAM10, ANXA5, ARPC5, B2M, CD151, CD59, CD9, CD99, CDC42, CFL1, FERMT3, F
1.38E-26	12.87	ACTB, ADAM10, ANXA5, ARPC5, B2M, CD151, CD59, CD9, CD99, CDC42, CFL1, FLNA, FLO
3.22E-25	5.31	ADAM10, ALDOA, ANPEP, ANTXR2, ANXA11, ANXA2, ANXA7, ARPC5, B2M, BCAP31, CALI
3.54E-25	5.30	ADAM10, ALDOA, ANPEP, ANTXR2, ANXA11, ANXA2, ANXA7, ARPC5, B2M, BCAP31, CALI
1.65E-24	10.27	ADAM10, ALDOA, ANXA2, ANXA7, ATIC, CCT8, CD151, CD226, CD47, CD9, CLIC1, DSP, EE
1.02E-20	5.85	AACS, ACTB, ADAM10, ADH5, ALDOA, ANK1, ANPEP, ANTXR2, ANXA11, ANXA2, ANXA5, /
1.92E-20	3.97	AACS, ACTB, ADH5, ALDOA, ANK1, ANXA11, ANXA2, ANXA5, ARPC5, ATIC, B2M, BCAP31,
6.16E-20	12.43	ALDOA, ANXA2, ARPC5, B2M, CAMP, CCT2, CCT8, CTSD, CYB5R3, EEF1A1, EEF2, FCN1, FE
8.36E-20	12.30	ALDOA, ANXA2, ARPC5, B2M, CAMP, CCT2, CCT8, CTSD, CYB5R3, EEF1A1, EEF2, FCN1, FE
1.02E-19	12.21	ALDOA, ANXA2, ARPC5, B2M, CAMP, CCT2, CCT8, CTSD, CYB5R3, EEF1A1, EEF2, FCN1, FE
9.50E-19	5.60	ACTB, ADAM10, ANXA2, ANXA5, ARPC5, B2M, CD151, CD53, CD59, CD9, CD99, CDC42, C
2.53E-17	11.07	ALDOA, ANXA2, ATIC, CCT8, CLIC1, EEF2, ENO1, F11R, FLNA, FSCN1, HSPA8, ITGA6, ITGB1
3.29E-17	6.81	AACS, ACTB, ADAM10, ADH5, ALDOA, ANK1, ANTXR2, ANXA11, ANXA2, ANXA5, ANXA7, /
4.38E-17	3.77	AACS, ACTB, ADAM10, ADH5, ALDOA, ANPEP, ANXA2, ANXA5, ARL6IP5, ARPC5, ATP2A3,
3.51E-16	4.32	ACTB, ADAM10, ANXA2, ANXA5, ARPC5, B2M, CALM3, CANX, CD151, CD53, CD59, CD9, C
4.60E-16	3.71	ACTB, ADAM10, ALDOA, ANK1, ANPEP, ANTXR2, ANXA11, ANXA2, ANXA5, ANXA7, ARL6I
1.45E-15	22.78	ACTB, ANK1, ARPC5, DSP, KRT14, KRT15, KRT16, KRT2, KRT5, KRT6A, KRT6B, KRT9, MSN,
1.53E-15	13.26	ACTB, ANXA2, ANXA5, CD36, CD59, CD9, CLIC1, F11R, FCER1G, FERMT3, FLNA, GNAQ, GI
2.67E-15	12.94	ACTB, ANXA2, ANXA5, CD36, CD59, CD9, CLIC1, F11R, FCER1G, FERMT3, FLNA, GNAQ, GI
2.98E-15	12.87	ACTB, ANXA2, ANXA5, CD36, CD59, CD9, CLIC1, F11R, FCER1G, FERMT3, FLNA, GNAQ, GI
3.02E-15	24.67	ACTB, CCL5, CD9, CD99, CLIC1, DSP, F11R, FERMT3, FLNA, GP1BA, ILK, ITGB3, JUP, MMRI
4.00E-15	4.01	ACTB, ALDOA, ANK1, ANXA11, ARL6IP5, ARPC5, CALM3, CASP14, CCT2, CCT4, CCT6A, CC
7.09E-15	4.98	ACTB, ADAM10, ANK1, ANXA11, ANXA7, ARPC5, B2M, BCAP31, CD151, CD226, CD36, CD
1.16E-14	4.21	ALDOA, ANXA11, ANXA2, ANXA7, CANX, CCT4, CCT6A, CSDE1, DAZAP1, DCD, DDX60L, DI
1.49E-14	3.33	ADAM10, ALDOA, ANK1, ANPEP, ANTXR2, ANXA11, ANXA2, ANXA7, ARL6IP5, ARPC5, ATI
2.73E-14	8.92	ACTB, ANXA2, ANXA5, CD36, CD59, CD9, CLIC1, F11R, FCER1G, FERMT3, FLG2, FLNA, GN/
3.69E-14	5.97	ACTB, ANK1, ARPC5, CLTA, DSP, FLG2, H2AC20, H2BC21, JUP, KRT1, KRT10, KRT14, KRT1
3.75E-14	14.23	ALDOA, ARPC5, CCT8, CTSD, DSC1, DSG1, DSP, EEF1A1, EEF2, FCER1G, FCN1, GSTP1, HSP
1.17E-13	8.08	ACTB, ANXA2, ANXA5, CD151, CD36, CD59, CD9, CLIC1, DSP, F11R, FCER1G, FERMT3, FLN
5.89E-13	14.58	ADAM10, ALDOA, B2M, CAMP, CD47, CD53, CD59, CTSD, CYBB, DSC1, DSG1, DSP, FCER1
1.14E-12	19.20	ANXA11, ANXA2, CANX, CCT4, CTSD, FLOT1, HSP90AA1, HSPA8, ITGB1, ITGB3, PRDX1, RA
1.14E-12	19.20	ANXA11, ANXA2, CANX, CCT4, CTSD, FLOT1, HSP90AA1, HSPA8, ITGB1, ITGB3, PRDX1, RA
1.55E-12	4.09	ADH5, ANPEP, B2M, CAMP, CCL5, CD226, CD36, CD47, CDC42, CFL1, CYBB, DCD, DDX60L
2.23E-12	16.42	ACTB, CD9, CLIC1, F11R, FCER1G, FERMT3, FLNA, GNAQ, GP1BA, GP1BB, ILK, ITGB3, MM

2.80E-12	4.22	ACTB, ADAM10, ANXA2, ATP5F1B, B2M, CCL5, CD151, CD226, CD36, CD47, CD9, CD99, C
3.71E-12	4.40	ACTB, ADAM10, ADH5, ALDOA, ANK1, ANPEP, ANTXR2, ANXA11, ANXA2, ANXA5, ANXA7
5.48E-12	9.16	ADAM10, ANPEP, ANXA7, CD36, CD47, CD53, CD59, CD9, CYBB, DSC1, DSG1, DSP, FCER1
7.05E-12	25.96	ACTB, CD9, CLIC1, F11R, FERMT3, FLNA, GP1BA, ILK, ITGB3, MMRN1, MYH9, PLEK, PPIA,
8.51E-12	2.93	ACTB, ADAM10, ANK1, ANPEP, ANTXR2, ANXA11, ANXA2, ANXA5, ANXA7, ARL6IP5, ATIC
1.01E-11	5.04	ACTB, ANXA2, B2M, CCL5, CD47, CD9, CD99, CLIC1, DSC1, DSG1, DSP, F11R, FERMT3, FLN
1.11E-11	30.02	ANXA2, CASP14, DSC1, DSG1, DSP, FLG2, HRNR, JUP, KRT1, KRT10, KRT16, KRT17, KRT2,
2.67E-11	4.79	ACTB, ALDOA, ANK1, CALM3, CASP14, CCT2, CCT4, CCT6A, CCT7, CCT8, DSP, ENO1, FLNA
2.82E-11	4.76	ACTB, ADAM10, ADH5, ALDOA, ANK1, ANPEP, ANTXR2, ANXA11, ANXA2, ANXA5, ANXA7
3.53E-11	6.27	ACTB, ANXA2, ANXA5, CD151, CD36, CD59, CD9, CLIC1, DSP, F11R, FCER1G, FERMT3, FLN
3.64E-11	4.74	ACTB, ALDOA, ANK1, CALM3, CASP14, CCT2, CCT4, CCT6A, CCT7, CCT8, DSP, ENO1, FLNA
4.18E-11	9.17	AACS, ACTB, ADAM10, ADH5, ALDOA, ANK1, ANPEP, ANTXR2, ANXA11, ANXA2, ANXA5, A
5.63E-11	4.17	ACTB, ALDOA, ANK1, CALM3, CASP14, CCT2, CCT4, CCT6A, CCT7, CCT8, CDC42, CSDE1, D
7.31E-11	13.25	ADAM10, ANXA11, B2M, CAMP, CD36, CD47, CD53, CD59, CTSD, CYBB, JUP, LTF, LYZ, PT
8.65E-11	3.07	ACTB, ALDOA, ANK1, ANXA2, ARPC5, BCAP31, CALM3, CCT2, CCT4, CCT6A, CCT7, CCT8, C
1.24E-10	3.53	AACS, ACTB, ADAM10, ANXA2, ARL6IP5, B2M, BCAP31, CALM3, CCL5, CCT2, CCT4, CCT6A
1.42E-10	5.60	ANK1, ANPEP, ANXA5, B2M, BCAP31, CANX, CD226, CD36, CD59, CD9, DSG1, EEF1A1, FC
1.68E-10	15.50	ALDOA, ARPC5, CCT8, CTSD, EEF1A1, EEF2, FCN1, GSTP1, HSP90AA1, HSPA1B, HSPA8, JU
1.72E-10	3.53	ACTB, ADAM10, ANK1, ANXA2, CALM3, CAVIN2, CCT2, CD226, CDC42, CLTA, DHX9, DSP,
2.03E-10	2.79	ACTB, ADAM10, ANK1, ANPEP, ANTXR2, ANXA2, ANXA5, ARL6IP5, ATIC, ATP5F1B, B2M, I
2.85E-10	2.80	AACS, ACTB, ADAM10, ANK1, ANTXR2, ANXA11, ANXA2, ARL6IP5, ARPC5, ATP2A3, ATP5I
2.85E-10	2.93	ACTB, ADH5, ANXA2, ANXA5, ARL6IP5, ATP2A3, B2M, BCAP31, CAMP, CANX, CCL5, CD15
6.35E-10	3.09	ACTB, ADAM10, ALDOA, ANXA2, ARPC5, B2M, CCT2, CCT8, CD36, CD47, CD9, CDC42, CLT
7.67E-10	3.00	AACS, ACTB, ADAM10, ANK1, ANXA2, ARL6IP5, ATP2A3, BCAP31, CALM3, CANX, CCL5, C
1.43E-09	4.23	ACTB, ADAM10, B2M, CAMP, CCL5, CD151, CD226, CD47, CD9, CLIC1, F11R, FCER1G, FEF
1.61E-09	6.19	ACTB, ADAM10, ANXA2, CD53, CDC42, DSC1, DSG1, DSP, F11R, FLNA, FLOT1, FSCN1, ITG
2.36E-09	3.06	ACTB, ADAM10, ANXA2, B2M, CAMP, CCL5, CD151, CD226, CD36, CD47, CD59, CD9, CD9
2.40E-09	8.07	ANPEP, CAMP, CCL5, CDC42, CFL1, DCD, EEF1A1, F11R, FCN1, GAPDH, HSPA1B, HSPA8, H
2.76E-09	34.91	CCT2, CCT4, CCT6A, CCT7, CCT8, HSP90AA1, HSPA1B, HSPA8, HSPA9, HSPD1, TCP1
2.82E-09	22.28	DSP, KRT1, KRT14, KRT15, KRT16, KRT17, KRT2, KRT5, KRT6A, KRT6B, KRT78, KRT9, VIM
3.28E-09	3.18	AACS, ACTB, ADAM10, ANK1, ARL6IP5, BCAP31, CALM3, CANX, CCL5, CCT2, CCT4, CCT6A
3.96E-09	3.16	AACS, ACTB, ADAM10, ANK1, ARL6IP5, BCAP31, CALM3, CANX, CCL5, CCT2, CCT4, CCT6A
4.32E-09	4.52	AACS, ACTB, ADAM10, ADH5, ALDOA, ANK1, ANPEP, ANTXR2, ANXA11, ANXA2, ANXA5, A
4.76E-09	17.99	DSP, KRT1, KRT14, KRT15, KRT16, KRT17, KRT2, KRT5, KRT6A, KRT6B, KRT78, KRT9, SOD1
5.58E-09	17.75	DSP, KRT1, KRT14, KRT15, KRT16, KRT17, KRT2, KRT5, KRT6A, KRT6B, KRT78, KRT9, SOD1
5.99E-09	2.99	AACS, ACTB, ADAM10, ANK1, ANXA2, ARL6IP5, BCAP31, CALM3, CANX, CCL5, CCT2, CCT4
6.38E-09	4.42	ADAM10, ANPEP, ANTXR2, ANXA2, ANXA5, ATP5F1B, B2M, CD151, CD226, CD36, CD47,
7.27E-09	2.60	AACS, ACTB, ADAM10, ANXA2, ARL6IP5, ATP2A3, ATP5F1B, B2M, BCAP31, CALM3, CAMI
1.09E-08	2.67	AACS, ACTB, ADAM10, ANK1, ANTXR2, ANXA11, ANXA2, ARL6IP5, ATP2A3, ATP5F1B, B2N
1.12E-08	6.71	ADAM10, CCL5, CD47, CD9, CD99, CDC42, F11R, FCER1G, GP1BA, ITGA6, ITGB1, ITGB3, L
1.20E-08	4.01	ANXA2, B2M, CCL5, CD226, CD36, CSDE1, CYBB, DAZAP1, DHX9, EEF2, EIF5A, ENO1, FCEI
1.40E-08	2.57	ACTB, ADAM10, ALDOA, ANK1, ANXA2, ARPC5, B2M, BCAP31, CALM3, CAVIN2, CCT2, CC
1.47E-08	3.23	ADAM10, ANPEP, ANTXR2, ANXA2, ANXA7, ATP2A3, B2M, BCAP31, CALM3, CD36, CD47,
1.61E-08	3.12	ACTB, ADAM10, ANXA2, ARPC5, B2M, CALM3, CCT2, CCT4, CCT6A, CCT7, CCT8, CD151, C
1.67E-08	2.85	ACTB, ADAM10, ALDOA, ANXA2, ARPC5, B2M, CCT2, CCT8, CD36, CD47, CD9, CDC42, CLT
2.48E-08	2.54	ACTB, ADAM10, ALDOA, ANPEP, ANXA2, ANXA7, ARPC5, ATIC, ATP5F1B, B2M, BCAP31, C
5.74E-08	2.49	ACTB, ADAM10, ALDOA, ANK1, ANXA2, ARPC5, B2M, BCAP31, CALM3, CAVIN2, CCT2, CC

5.76E-08	3.54	ACTB, ALDOA, ANK1, ARPC5, CD47, CDC42, CFL1, DSP, F11R, FLNA, FSCN1, GAPDH, HNRN
7.03E-08	2.59	AACS, ACTB, ADAM10, ANK1, ANTXR2, ANXA11, ANXA2, ARL6IP5, ATP2A3, ATP5F1B, B2M
8.92E-08	2.50	AACS, ACTB, ADAM10, ANXA2, ARL6IP5, ATP5F1B, B2M, BCAP31, CALM3, CAMP, CCL5, C
9.22E-08	11.08	ADAM10, ANXA7, CD151, CD226, CD9, F11R, FERMT3, ICAM2, ITGA2B, ITGA6, ITGB1, ITG
9.96E-08	4.47	ALDOA, ANXA2, ARPC5, B2M, CD36, CD47, CDC42, CFL1, DSP, F11R, FLNA, FSCN1, HSPA1
1.19E-07	2.48	ACTB, ADAM10, ALDOA, ANPEP, ANXA2, ANXA7, ARPC5, ATIC, ATP5F1B, B2M, CALM3, C
1.35E-07	13.61	ADAM10, CD47, CDC42, FERMT3, FLNA, ILK, ITGA2B, ITGA6, ITGB1, ITGB3, MYH9, PLEK, I
1.38E-07	8.97	CCT2, CCT4, CCT6A, CCT7, CCT8, DSG1, FLNA, FLOT1, GAPDH, GNAQ, HSP90AA1, HSPA1B
1.68E-07	5.86	AACS, BCAP31, CCT2, CCT4, CCT6A, CCT7, CCT8, FLNA, HSP90AA1, ITGB1, JUP, LRP1, MSN
2.40E-07	4.32	AACS, ACTB, ADAM10, BCAP31, CCL5, CCT2, CCT4, CCT6A, CCT7, CCT8, CD36, CTSD, FLNA
3.29E-07	4.61	ANXA7, CASP14, CDC42, DSP, F11R, HNRNPH3, HRNR, KRT1, KRT10, KRT14, KRT15, KRT1
3.96E-07	2.98	ACTB, ADAM10, ADH5, ALDOA, ANK1, ANPEP, ANTXR2, ANXA11, ANXA2, ANXA7, ARL6IP
4.08E-07	4.70	ATP2A3, CCT2, CCT4, CCT6A, CCT7, CCT8, CDC42, DDX60L, DHX9, EEF1A1, EEF2, G3BP1, (
4.11E-07	4.04	AACS, ACTB, ADAM10, ANXA2, BCAP31, CCL5, CCT2, CCT4, CCT6A, CCT7, CCT8, CD36, CT
4.39E-07	16.61	CCT2, CCT4, CCT6A, CCT7, CCT8, HNRNPA2B1, HNRNPC, HNRNPU, HSP90AA1, TCP1, TNK
4.65E-07	3.31	ADH5, B2M, CAMP, CCL5, CD226, CD36, CD47, CDC42, CFL1, CYBB, DCD, DDX60L, DHX9,
4.82E-07	12.24	CANX, CCT2, CCT4, CCT6A, CCT7, CCT8, HSP90AA1, HSPA1B, HSPA8, HSPA9, HSPD1, NPM
6.61E-07	4.35	ACTB, ADAM10, CALM3, CAVIN2, CD226, CDC42, DSP, EEF1A1, EEF2, FLNA, GSTP1, HINT
6.62E-07	2.69	ACTB, ADAM10, ADH5, ANXA5, ARL6IP5, ATIC, ATP5F1B, B2M, BCAP31, CALM3, CAMP, C
7.72E-07	2.40	ACTB, ALDOA, ANK1, ANXA11, ANXA2, ARL6IP5, ARPC5, ATP5F1B, BCAP31, CALM3, CANX
7.81E-07	2.40	ACTB, ALDOA, ANK1, ANXA11, ANXA2, ARL6IP5, ARPC5, ATP5F1B, BCAP31, CALM3, CANX
8.55E-07	4.30	ACTB, CALM3, CASP14, CCT2, CCT4, CCT6A, CCT7, CCT8, DSP, HNRNPU, JUP, KRT1, KRT10
9.89E-07	3.62	ADAM10, ANPEP, ANTXR2, ANXA2, ANXA7, B2M, CALM3, CD36, CD47, CD53, CD59, CD9,
1.03E-06	18.35	CCT2, CCT4, CCT6A, CCT7, CCT8, HSP90AA1, HSPA1B, HSPA8, HSPA9, HSPD1, TCP1
1.21E-06	18.03	CCT2, CCT4, CCT6A, CCT7, CCT8, HNRNPC, HNRNPU, HSP90AA1, TCP1, TNKS1BP1, XRCC5
1.30E-06	2.70	ADAM10, ADH5, ANXA2, B2M, CAMP, CCL5, CD226, CD36, CD47, CD9, CDC42, CFL1, CYB
1.58E-06	3.56	ADAM10, ANPEP, ANTXR2, ANXA2, ANXA7, B2M, CALM3, CD36, CD47, CD53, CD59, CD9,
1.81E-06	139.76	CCT2, CCT4, CCT6A, CCT7, CCT8, TCP1
1.85E-06	2.82	AACS, ACTB, ADH5, ANXA2, ATP2A3, ATP5F1B, CCT2, CCT4, CCT6A, CCT7, CCT8, CDC42, (
1.97E-06	2.63	ACTB, ADAM10, ADH5, ARL6IP5, ATIC, ATP5F1B, B2M, CAMP, CCL5, CD36, CD47, CDC42,
1.98E-06	3.23	ADH5, B2M, CAMP, CCL5, CD226, CD36, CD47, CDC42, CFL1, CYBB, DCD, DDX60L, DHX9,
2.11E-06	3.23	ADH5, B2M, CAMP, CCL5, CD226, CD36, CD47, CDC42, CFL1, CYBB, DCD, DDX60L, DHX9,
2.17E-06	7.46	CASP14, CCT8, DSP, JUP, KRT1, KRT10, KRT14, KRT15, KRT16, KRT17, KRT2, KRT5, KRT6A,
2.25E-06	3.85	ADAM10, ANPEP, ARL6IP5, CCL5, CPN1, CSDE1, DHX9, EEF1A1, EEF2, EIF5A, G6PD, GAPD
3.06E-06	16.34	CCT2, CCT4, CCT6A, CCT7, CCT8, HNRNPC, HNRNPU, HSP90AA1, TCP1, TNKS1BP1, XRCC5
3.11E-06	4.29	ATP2A3, CCT2, CCT4, CCT6A, CCT7, CCT8, CDC42, DDX60L, DHX9, EEF1A1, EEF2, G3BP1, (
3.22E-06	4.28	ATP2A3, CCT2, CCT4, CCT6A, CCT7, CCT8, CDC42, DDX60L, DHX9, EEF1A1, EEF2, G3BP1, (
3.22E-06	4.28	ATP2A3, CCT2, CCT4, CCT6A, CCT7, CCT8, CDC42, DDX60L, DHX9, EEF1A1, EEF2, G3BP1, (
3.32E-06	3.22	ANXA2, ARL6IP5, ATP2A3, BCAP31, CALM3, CAMP, CCL5, CD36, CSDE1, CTSD, DHX9, EEF
3.74E-06	2.42	ACTB, ANPEP, ANXA2, ANXA7, ATP5F1B, B2M, CASP14, CD36, CD53, CD9, CDC42, CFL1, (
3.75E-06	4.37	ACTB, ADAM10, CALM3, CAVIN2, CD226, CDC42, DSP, EEF1A1, EEF2, FLNA, GSTP1, HINT
3.93E-06	10.27	ACTB, ANXA5, CLIC1, HSPA1B, HSPA8, ITGA2B, KRT1, LGALS3BP, MSN, PFN1, SLC2A1, STO
3.94E-06	111.80	CCT2, CCT4, CCT6A, CCT7, CCT8, TCP1
3.94E-06	111.80	CCT2, CCT4, CCT6A, CCT7, CCT8, TCP1
3.94E-06	111.80	CCT2, CCT4, CCT6A, CCT7, CCT8, TCP1
3.94E-06	111.80	CCT2, CCT4, CCT6A, CCT7, CCT8, TCP1
5.00E-06	3.46	ACTB, ANXA7, CASP14, CD151, CDC42, DSP, F11R, FLG2, FLNA, HNRNPH3, HRNR, ILK, ITG

5.17E-06	2.40	ACTB, ANPEP, ANXA2, ANXA7, ATP5F1B, B2M, CASP14, CD36, CD53, CD9, CDC42, CFL1, C
5.18E-06	3.13	ADAM10, ARPC5, ATP5F1B, CCL5, CD151, CD47, CD9, CD99, CDC42, F11R, FCER1G, FERM
5.62E-06	4.06	ACTB, ADAM10, ATP5F1B, B2M, CCL5, CD36, CD47, CD9, CDC42, F11R, FERMT3, FLNA, FI
5.74E-06	2.84	AACS, ACTB, ATP2A3, ATP5F1B, CCT2, CCT4, CCT6A, CCT7, CCT8, CDC42, CYB5R3, CYBB, I
6.12E-06	18.56	CCT2, CCT4, CCT6A, CCT7, CCT8, HNRNPA2B1, HNRNPC, HNRNPU, TCP1, XRCC5
6.28E-06	3.85	AACS, ACTB, ANXA2, B2M, BCAP31, CALM3, CCL5, CD151, CD36, CD47, CDC42, DHX9, FC
7.16E-06	2.66	ACTB, ANXA2, ARL6IP5, ATP2A3, BCAP31, CALM3, CAMP, CCL5, CD36, CSDE1, CTSD, DHX
7.80E-06	93.16	CCT2, CCT4, CCT6A, CCT7, CCT8, TCP1
7.80E-06	93.16	CCT2, CCT4, CCT6A, CCT7, CCT8, TCP1
7.80E-06	93.16	CCT2, CCT4, CCT6A, CCT7, CCT8, TCP1
7.80E-06	93.16	CCT2, CCT4, CCT6A, CCT7, CCT8, TCP1
7.82E-06	2.24	ACTB, ADAM10, ADH5, ALDOA, ANPEP, ANXA2, ANXA5, ARL6IP5, ARPC5, ATIC, ATP2A3, /
8.26E-06	5.43	ANPEP, CANX, CCL5, CFL1, CSDE1, DHX9, EEF1A1, F11R, FCN1, HSPA1B, HSPA8, ITGB1, IT
8.35E-06	2.70	AACS, ACTB, ALDOA, ATP2A3, ATP5F1B, CCT2, CCT4, CCT6A, CCT7, CCT8, CDC42, CYB5R3
9.01E-06	3.10	ADAM10, ANXA7, ATP5F1B, BCAP31, CALM3, CANX, CCL5, CD151, CD226, CD36, CD9, CD
9.39E-06	2.26	ACTB, ADAM10, ADH5, ANK1, ANPEP, ANXA11, ANXA2, ANXA5, ARL6IP5, ATIC, ATP2A3, /
1.10E-05	7.77	CASP14, DSP, JUP, KRT1, KRT10, KRT14, KRT15, KRT16, KRT17, KRT2, KRT5, KRT6A, KRT6I
1.17E-05	2.85	ACTB, ADAM10, ADH5, ALDOA, ANXA2, ATIC, B2M, CCL5, CD226, CD53, CDC42, ENO1, F1
1.39E-05	3.06	ANXA2, ARL6IP5, ATP2A3, B2M, BCAP31, CALM3, CCL5, CCT2, CCT4, CD36, CDC42, CTSD,
1.43E-05	79.85	FLOT1, ICAM2, MSN, MYH9, SELPLG, SPN
1.43E-05	79.85	FLOT1, ICAM2, MSN, MYH9, SELPLG, SPN
1.55E-05	13.75	CCT2, CCT4, CCT6A, CCT7, CCT8, FLNA, HSP90AA1, JUP, NPM1, TCP1, TFRC
1.61E-05	2.77	AACS, ACTB, ATP2A3, ATP5F1B, CCT2, CCT4, CCT6A, CCT7, CCT8, CDC42, CYB5R3, CYBB, I
1.85E-05	6.06	CCT2, CCT4, CCT6A, CCT7, CCT8, DSG1, FLNA, FLOT1, GAPDH, GNAQ, HSP90AA1, HSPA1E
2.09E-05	2.42	ACTB, ANXA2, ARL6IP5, ATP2A3, B2M, BCAP31, CALM3, CAMP, CCL5, CCT2, CCT4, CCT6A
2.48E-05	69.87	CCT2, CCT4, CCT6A, CCT7, CCT8, TCP1
2.48E-05	69.87	CCT2, CCT4, CCT6A, CCT7, CCT8, TCP1
2.52E-05	13.06	ALDOA, CD36, CD9, FERMT3, ITGA2B, ITGB3, MMRN1, PECAM1, QSOX1, SRGN, THBS1
2.57E-05	19.73	CD9, F11R, FCER1G, FLNA, GNAQ, GP1BA, GP1BB, MMRN1, PLEK
2.90E-05	7.22	ALDOA, CCT2, CCT4, CCT7, CCT8, CD226, CD36, CD9, FCN1, LBP, MSN, PECAM1, RTN4, TC
2.93E-05	25.91	CCT2, CCT4, CCT6A, CCT7, CCT8, HNRNPA2B1, TCP1, XRCC5
3.02E-05	11.00	CAMP, CCL5, CDC42, CFL1, DCD, EEF1A1, GAPDH, HSPA8, HSPD1, KRT6A, LTF, STOM
3.06E-05	19.28	CCT2, CCT4, CCT6A, CCT7, CCT8, HNRNPC, HNRNPU, TCP1, XRCC5
3.35E-05	2.35	ACTB, ANXA2, ARL6IP5, ATP2A3, B2M, BCAP31, CALM3, CAMP, CCL5, CCT2, CCT4, CCT6A
3.37E-05	9.56	CCL5, CSDE1, DHX9, EEF2, EIF5A, HNRNPU, KRT17, NPM1, PKM, RHOA, SYNCRIP, THBS1,
3.67E-05	3.31	CAMP, CCL5, CD226, CD36, CD47, CDC42, CYBB, DCD, DDX60L, DHX9, ENDOD1, FCER1G,
3.71E-05	7.09	ACTB, ANK1, ARPC5, CDC42, FSCN1, HSP90AA1, ILK, ITGB1, MSN, MYH9, PATJ, RAB10, RA
3.81E-05	2.85	CAMP, CCL5, CD226, CD36, CD47, CDC42, CYBB, DCD, DDX60L, DHX9, ENDOD1, F11R, FC
4.09E-05	62.10	CCT2, CCT4, CCT6A, CCT7, CCT8, TCP1
4.18E-05	4.74	ACTB, ALDOA, ARPC5, CFL1, DHX9, EEF1A1, FERMT3, FLNA, FLOT1, FSCN1, HNRNPC, ILK,
4.24E-05	2.18	AACS, ACTB, ALDOA, ANXA11, ANXA2, ANXA7, ATP2A3, ATP5F1B, CANX, CCT2, CCT4, CCT
4.72E-05	2.17	AACS, ACTB, ALDOA, ANXA11, ANXA2, ANXA7, ATP2A3, ATP5F1B, CANX, CCT2, CCT4, CCT
5.44E-05	7.48	ACTB, ADAM10, CD9, CDC42, DSG1, DSP, F11R, FLNA, FSCN1, JUP, PATJ, PECAM1, RHOA,
6.11E-05	4.04	ADAM10, CALM3, CCT6A, CDC42, F11R, GAPDH, GNG5, HSP90AA1, HSPA1B, HSPA8, INPI
6.14E-05	9.36	AACS, ACTB, ADAM10, ADH5, ALDOA, ANK1, ANPEP, ANTXR2, ANXA11, ANXA2, ANXA5, /
6.64E-05	2.82	ACTB, ADAM10, ARPC5, ATP5F1B, CCL5, CD151, CD47, CD9, CD99, CDC42, F11R, FCER1G
6.72E-05	2.89	AACS, ARL6IP5, BCAP31, CALM3, CANX, CCL5, CCT2, CCT4, CCT6A, CCT7, CCT8, CD36, CLT

7.68E-05	11.60	CASP14, KRT1, KRT10, KRT14, KRT17, KRT2, KRT5, KRT6A, KRT6B, KRT78, KRT9
8.01E-05	2.90	ACTB, ANK1, ANXA11, ANXA2, B2M, BCAP31, CALM3, CANX, CCL5, CD151, CD36, CD47, C
8.21E-05	2.15	ACTB, ADAM10, ANXA2, ARPC5, ATP5F1B, B2M, CALM3, CANX, CCT2, CCT4, CCT6A, CCT7
8.73E-05	3.97	ACTB, ADAM10, CD9, CDC42, CFL1, DSG1, DSP, F11R, FLNA, FLOT1, FSCN1, HSPA8, ITGA6
9.90E-05	50.80	CCT2, CCT4, CCT6A, CCT7, CCT8, TCP1
1.25E-04	3.52	ADAM10, BCAP31, CALM3, CCT2, CCT4, CCT6A, CCT7, CCT8, CD36, DHX9, FLNA, HNRNPU
1.30E-04	3.19	ACTB, ANXA2, ARPC5, CCT2, CCT4, CCT6A, CCT7, CCT8, CD47, CDC42, F11R, FLNA, FSCN1
1.38E-04	6.94	B2M, CANX, CCT2, CCT4, CCT6A, CCT7, CCT8, HSP90AA1, HSPA1B, HSPA8, HSPA9, HSPD1
1.41E-04	8.39	CSDE1, DHX9, HNRNPU, PKM, RPL11, RPL8, RPLP0, RPS15, RPS15A, RPS23, RPS3A, RPSA,
1.47E-04	46.57	CCT2, CCT4, CCT6A, CCT7, CCT8, TCP1
1.49E-04	12.78	CASP14, HRNR, KRT1, KRT16, KRT17, KRT2, KRT5, KRT6A, KRT6B, KRT78
1.54E-04	28.46	CCT2, CCT4, CCT6A, CCT7, CCT8, TCP1, XRCC5
1.68E-04	9.29	CCT2, CCT4, CCT6A, CCT7, CCT8, CD36, FLNA, HSP90AA1, JUP, NPM1, TCP1, TFRC
1.75E-04	4.95	ACTB, B2M, CCL5, CD47, F11R, FERMT3, GP1BA, HLA-A, HSPD1, ITGB1, MSN, PECAM1, PI
1.88E-04	2.75	AACS, ACTB, ANXA2, ARL6IP5, B2M, BCAP31, CALM3, CCL5, CD151, CD36, CD47, CDC42,
2.13E-04	42.98	CCT2, CCT4, CCT6A, CCT7, CCT8, TCP1
2.13E-04	42.98	CCT2, CCT4, CCT6A, CCT7, CCT8, TCP1
2.13E-04	42.98	CCT2, CCT4, CCT6A, CCT7, CCT8, TCP1
2.13E-04	42.98	CCT2, CCT4, CCT6A, CCT7, CCT8, TCP1
2.39E-04	3.28	ANXA2, B2M, CCT2, CCT4, CCT6A, CCT7, CCT8, CD151, CD36, CD47, CD53, CDC42, EIF5A,
2.69E-04	7.91	CCL5, CSDE1, DHX9, EEF2, EIF5A, HNRNPU, KRT17, NPM1, PKM, RHOA, SYNCRIP, THBS1,
3.23E-04	5.61	AACS, BCAP31, CCT2, CCT4, CCT6A, CCT7, CCT8, FLNA, HSP90AA1, JUP, PPIA, RAC2, SRI, S
3.24E-04	11.67	ADAM10, CD36, CD47, CD53, CD59, CYBB, PTPRJ, SLC2A3, STOM, TSPAN14
3.33E-04	2.52	ACTB, ADAM10, ARL6IP5, B2M, BCAP31, CCL5, CD226, CD36, CD47, CD59, CDC42, DHX9,
3.40E-04	2.10	ACTB, ADAM10, ADH5, ANPEP, ANXA2, ARL6IP5, ATP2A3, B2M, BCAP31, CALM3, CAMP,
3.44E-04	3.35	ARPC5, CD36, CD47, CDC42, F11R, FLNA, FLOT1, FSCN1, G3BP1, HNRNPU, HSP90AA1, HS
3.54E-04	5.57	ACTB, CLTA, EEF1A1, ENO1, FLNA, FLOT1, FSCN1, MYADM, MYH9, MYL12B, NCL, PFN1, R
3.58E-04	17.88	ALDOA, CCT2, CCT4, CCT7, CCT8, CD9, TCP1, VDAC2
3.72E-04	2.24	ADAM10, ANPEP, ANTXR2, ANXA2, ANXA7, ARL6IP5, ATP2A3, ATP5F1B, B2M, BCAP31, C
3.91E-04	24.24	CCT2, CCT4, CCT6A, CCT7, CCT8, TCP1, XRCC5
4.31E-04	13.67	ALDOA, CCT2, CCT4, CCT7, CCT8, CD9, MSN, TCP1, VDAC2
4.31E-04	13.67	CCT2, CCT4, CCT6A, CCT7, CCT8, HSPA1B, HSPA8, HSPA9, TCP1
4.65E-04	2.18	ACTB, ADAM10, ANXA2, ARL6IP5, B2M, BCAP31, CALM3, CCL5, CD226, CD36, CD47, CD5
4.73E-04	2.53	ACTB, ANXA5, ARL6IP5, ATP2A3, BCAP31, CASP14, CCL5, CD36, CFL1, CTSD, DHX9, EIF5A,
4.86E-04	2.35	ACTB, ADAM10, ANXA2, ANXA5, ATP2A3, ATP5F1B, B2M, CALM3, CAMP, CD226, CD36, C
4.87E-04	5.44	ANPEP, CCL5, EEF1A1, F11R, FCN1, HSPA1B, HSPA8, ITGB1, ITGB3, LTF, PCBP1, PPIA, RAB
5.06E-04	9.48	CCT2, CCT4, CCT6A, CCT7, CCT8, HNRNPA2B1, HNRNPC, HNRNPU, HSP90AA1, TCP1, XRC
5.50E-04	13.24	ADAM10, CD47, CD53, CD59, CYBB, FCER1G, SLC2A3, STOM, TSPAN14
6.22E-04	2.20	ACTB, ADAM10, ADH5, ALDOA, ANK1, ANPEP, ANTXR2, ANXA11, ANXA2, ANXA7, ARL6IP
6.64E-04	5.32	ANXA2, CAVIN2, CD226, CD36, CTSD, FLOT1, GNAI2, INPP5D, ITGB1, LDHB, MYADM, PEC
6.94E-04	5.30	ANXA2, CAVIN2, CD226, CD36, CTSD, FLOT1, GNAI2, INPP5D, ITGB1, LDHB, MYADM, PEC
7.23E-04	8.04	CCT2, CCT4, CCT6A, CCT7, CCT8, HNRNPA2B1, HNRNPC, HNRNPU, HSP90AA1, TCP1, TNK
7.27E-04	21.81	KRT1, KRT10, KRT2, KRT5, KRT6A, KRT6B, KRT78
7.57E-04	5.26	DAZAP1, DHX9, HNRNPA2B1, HNRNPA3, HNRNPC, HNRNPF, HNRNPH1, HNRNPH3, HNRN
7.57E-04	5.26	DAZAP1, DHX9, HNRNPA2B1, HNRNPA3, HNRNPC, HNRNPF, HNRNPH1, HNRNPH3, HNRN
7.58E-04	4.97	CD36, CDC42, FERMT3, FLNA, ILK, ITGA2B, ITGA6, ITGB1, ITGB3, JUP, MYADM, PTPRJ, RA
7.72E-04	15.97	ALDOA, B2M, CAMP, CTSD, FLG2, LTF, LYZ, QSOX1

8.10E-04	3.44	ANPEP, ANXA11, ANXA2, CCT2, CCT8, CDC42, CLTA, CTSD, CYB5R3, EEF1A1, FLOT1, GNA(
8.61E-04	2.12	ACTB, ADAM10, ADH5, ANXA11, ANXA5, ARL6IP5, ATIC, ATP5F1B, B2M, BCAP31, CALM3
8.77E-04	10.38	CCT2, CCT4, CCT6A, CCT7, CCT8, HNRNPA2B1, HNRNPC, HNRNPU, TCP1, XRCC5
8.98E-04	5.20	DAZAP1, DHX9, HNRNPA2B1, HNRNPA3, HNRNPC, HNRNPF, HNRNPH1, HNRNPH3, HNRN
9.03E-04	6.44	CD36, CDC42, FLNA, ILK, ITGB3, JUP, MYADM, PTPRJ, RAC2, RCC2, RHOA, THBS1, TLN1, V
9.40E-04	2.56	B2M, CAMP, CCL5, CD226, CD36, CD47, CD59, CDC42, CYBB, DHX9, ENDOD1, FCER1G, F(
9.60E-04	2.66	ACTB, ALDOA, ATP2A3, ATP5F1B, B2M, BCAP31, CALM3, CCL5, CD36, F11R, FLG2, FLNA,
1.01E-03	2.75	ACTB, ADAM10, B2M, CCL5, CD226, CD36, CD47, CD59, CD9, CD99, DHX9, FCER1G, FCN1
1.07E-03	2.68	ADAM10, ALDOA, ARPC5, B2M, CCT2, CCT8, CD36, CDC42, CLTA, DHX9, FERMT3, FSCN1,
1.11E-03	5.44	CCT2, HSP90AA1, HSPA1B, HSPA8, HSPA9, HSPD1, PRDX6, RPL11, RTN4, TCP1, TUBB, UB
1.12E-03	4.59	ACTB, ARPC5, CDC42, CFL1, EEF1A1, FLOT1, FSCN1, ILK, ITGB1, ITGB3, MYADM, MYH9, P
1.13E-03	184.68	CSDE1, DHX9, HNRNPU, SYNCRIP
1.15E-03	3.16	ARL6IP5, BCAP31, CCL5, CD36, CDC42, EIF5A, F11R, FLNA, GAPDH, GNAI2, HINT1, HSP90.
1.22E-03	2.58	ADAM10, ALDOA, ARPC5, B2M, CCT2, CCT8, CD36, CDC42, CFL1, CLTA, DHX9, EIF5A, FER
1.26E-03	2.93	ADAM10, ANPEP, ARL6IP5, CCL5, CPN1, CSDE1, DHX9, EEF1A1, EEF2, EIF5A, G6PD, GAPD
1.27E-03	5.39	CASP14, DSP, FLG2, HRNR, ITGA6, JUP, KRT1, KRT10, KRT16, KRT17, KRT2, KRT5, KRT6A,
1.28E-03	19.83	ADAM10, ANTXR2, CCT2, CCT4, CCT7, CCT8, TCP1
1.31E-03	1.98	ACTB, ADAM10, ADH5, ANK1, ANPEP, ANXA5, ARL6IP5, ATIC, ATP2A3, ATP5F1B, B2M, B(
1.36E-03	5.03	ANXA11, B2M, CD36, CD9, CDC42, CLTA, CYBB, HLA-A, HLA-B, HSP90AA1, LRP1, LTF, M6I
1.37E-03	3.73	ACTB, ANXA2, B2M, CALM3, CANX, CD151, CD36, CD9, CDC42, CLTA, FCER1G, FCN1, FLO
1.42E-03	2.96	ARL6IP5, ATP2A3, BCAP31, CALM3, CCL5, CCT2, CCT4, CDC42, CTSD, DHX9, F11R, GNAI2,
1.47E-03	4.73	CSDE1, FLNA, FSCN1, G3BP1, HNRNPU, HSPA1B, ITGB1, MSN, NPM1, RHOA, RPL11, RPLP
1.48E-03	3.12	ADAM10, ATP5F1B, CCL5, CD151, CD47, CD9, CD99, FERMT3, FLNA, GNAI2, GP1BA, ITGA
1.52E-03	19.24	ALDOA, CCT2, CCT4, CCT7, CCT8, TCP1, VDAC2
1.55E-03	4.49	ACTB, CD9, CDC42, DSG1, F11R, FLOT1, FSCN1, ITGA6, ITGB3, JUP, PATJ, PECAM1, PTPRJ,
1.58E-03	2.51	ACTB, ANXA7, CASP14, CD151, CDC42, DSP, F11R, FLG2, FLNA, FLOT1, G6PD, HNRNPH3,
1.62E-03	6.12	ALDOA, ANK1, CALM3, ENO1, FLNA, ILK, JUP, MYL12B, PSMA6, SLC2A1, SPTBN1, SRI, TPN
1.66E-03	2.42	AACS, ACTB, ATP2A3, ATP5F1B, CAMP, CCT2, CCT4, CCT6A, CCT7, CCT8, CDC42, DDX60L,
1.80E-03	2.16	ACTB, ADAM10, ANXA2, ARPC5, ATIC, B2M, CALM3, CASP14, CDC42, CSDE1, DAZAP1, DS
1.81E-03	18.69	CCT2, CCT4, CCT6A, CCT7, CCT8, HSPA8, TCP1
1.82E-03	2.49	ACTB, ANXA5, ARL6IP5, ATP2A3, BCAP31, CASP14, CCL5, CFL1, CTSD, DHX9, EIF5A, ENO1
1.87E-03	3.21	CAMP, CCL5, CD226, CD47, CDC42, CYBB, DHX9, ENDOD1, FCER1G, FCN1, G3BP1, GAPDI
1.92E-03	4.06	AACS, BCAP31, CCL5, CCT2, CCT4, CCT6A, CCT7, CCT8, CD36, CTSD, FLNA, HSP90AA1, HSI
1.95E-03	2.56	AACS, ACTB, ATP2A3, ATP5F1B, CCT2, CCT4, CCT6A, CCT7, CCT8, CDC42, DDX60L, DHX9,
1.99E-03	13.90	B2M, CAMP, CTSD, JUP, LTF, LYZ, QSOX1, VCL
2.07E-03	8.14	ANXA11, B2M, CD36, CDC42, CYBB, HLA-A, HLA-B, LTF, RAB10, RAC2, VIM
2.10E-03	5.98	CD36, FERMT3, ILK, ITGA2B, ITGA6, ITGB1, ITGB3, JUP, PTPRJ, RCC2, RHOA, THBS1, TLN1,
2.10E-03	6.52	ANPEP, F11R, FCN1, HSPA1B, ITGB1, ITGB3, KRT6A, LTF, PPIA, RPSA, SELPLG, TCP1, TFRC
2.10E-03	7.22	CASP14, DSP, HRNR, KRT1, KRT10, KRT16, KRT17, KRT2, KRT5, KRT6A, KRT6B, KRT78
2.11E-03	5.53	ACTB, B2M, F11R, INPP5D, ITGB1, ITGB3, KRT1, LTF, LYZ, PECAM1, PRDX1, SLC2A1, SOD1
2.11E-03	3.35	B2M, CD226, CD36, CD47, CYBB, DHX9, F11R, FCER1G, FCN1, FLOT1, G3BP1, GAPDH, GS
2.13E-03	26.60	B2M, HSP90AA1, HSPA1B, HSPA8, HSPA9, HSPD1
2.13E-03	26.60	CCT2, CCT4, CCT6A, CCT7, CCT8, TCP1
2.13E-03	26.60	ANXA11, B2M, HSP90AA1, HSPA8, PKM, YWHAE
2.14E-03	5.17	ANXA11, CD36, CD47, CDC42, FCER1G, FCN1, ITGB1, ITGB3, LBP, LRP1, MYH9, PECAM1, I
2.15E-03	4.19	ACTB, B2M, CCL5, CD36, CD47, CDC42, F11R, FLNA, FLOT1, GP1BA, HLA-A, HSPD1, ILK, IT
2.24E-03	46.36	CSDE1, DHX9, HNRNPU, PKM, SYNCRIP

2.29E-03	4.17	B2M, CD226, CD36, CYBB, DHX9, FCER1G, FCN1, FLOT1, G3BP1, GAPDH, HLA-A, HSP90A/
2.32E-03	3.51	ACTB, B2M, CCL5, CD226, CD47, CD9, F11R, FCER1G, FLNA, GNAQ, GP1BA, GP1BB, HLA-/
2.40E-03	3.11	ACTB, ALDOA, ANK1, ANXA2, ARPC5, CALM3, CFL1, FLNA, FSCN1, GAPDH, HNRNPU, HSP9
2.51E-03	17.68	ACTB, CALM3, CDC42, G6PD, RHOA, YWHAE, YWHAH
2.53E-03	5.10	CCT2, HSP90AA1, HSPA1B, HSPA8, HSPA9, HSPD1, PRDX6, RPL11, RTN4, TCP1, TUBB, UB
2.56E-03	5.87	ALDOA, ANK1, CALM3, ENO1, FLNA, ILK, JUP, MYL12B, PSMA6, SLC2A1, SPTBN1, SRI, TPN
2.85E-03	3.29	B2M, CD226, CD36, CD47, CYBB, DHX9, F11R, FCER1G, FCN1, FLOT1, G3BP1, GAPDH, GS
2.88E-03	4.75	CASP14, DSP, FLG2, HRNR, KRT1, KRT10, KRT14, KRT15, KRT16, KRT17, KRT2, KRT5, KRT6
2.95E-03	8.99	ADH5, CD36, GSR, GSTP1, PRDX1, PRDX2, PRDX6, PTGS1, S100A9, SOD1
3.05E-03	4.49	ARPC5, CD47, CDC42, DSP, F11R, FLNA, FSCN1, ITGB3, JUP, LRP1, MYADM, MYH9, PFN1,
3.06E-03	10.59	DAZAP1, DDX60L, DHX9, HNRNPC, HNRNPF, HNRNPH1, HNRNPU, PTBP1, SYNCRIP
3.11E-03	3.31	AACS, ACTB, ADAM10, ADH5, ALDOA, ANK1, ANPEP, ANXA11, ANXA2, ANXA5, ANXA7, A
3.30E-03	3.91	ANXA2, B2M, CALM3, CD151, CD36, CD47, CDC42, FCER1G, FCN1, FLOT1, GNAI2, ITGB3,
3.32E-03	2.41	ACTB, ANK1, ANXA2, ATP2A3, BCAP31, CALM3, CANX, CD36, CDC42, CFL1, CLTA, DHX9, F
3.65E-03	12.72	CCT2, CCT4, CCT6A, CCT7, CCT8, HNRNPA2B1, TCP1, XRCC5
3.72E-03	3.41	ANPEP, ANXA11, ANXA2, CCT2, CCT8, CLTA, CTSD, CYB5R3, EEF1A1, FLOT1, GNAQ, HRNR
3.72E-03	3.41	ANPEP, ANXA11, ANXA2, CCT2, CCT8, CLTA, CTSD, CYB5R3, EEF1A1, FLOT1, GNAQ, HRNR
3.82E-03	6.16	ALDOA, ANK1, CALM3, ENO1, FLNA, ILK, JUP, MYL12B, PSMA6, SLC2A1, SPTBN1, SRI, TPN
4.37E-03	2.30	ACTB, ARL6IP5, ATIC, ATP5F1B, CAMP, CCL5, CD36, CD47, CDC42, CYBB, DHX9, EEF1A1, E
4.40E-03	2.62	ACTB, ANXA2, B2M, CAMP, CD226, CD36, CFL1, CYBB, DHX9, ENO1, FCER1G, FCN1, FLOT
4.52E-03	38.63	CD36, CD9, ITGA2B, ITGB3, PECAM1
4.55E-03	4.16	ANPEP, ANXA5, B2M, CD226, CD36, CD59, CD9, FCER1G, FCN1, FLOT1, GP1BA, GSR, ITG/
4.64E-03	4.85	ACTB, ALDOA, B2M, F11R, INPP5D, ITGB1, ITGB3, KRT1, LTF, LYZ, PECAM1, PRDX1, SLC2/
4.96E-03	2.44	ACTB, ANXA5, ARL6IP5, ATP2A3, BCAP31, CASP14, CCL5, CFL1, CTSD, EIF5A, ENO1, FLNA,
4.99E-03	3.12	ADH5, CALM3, CAMP, CCL5, CD36, CPN1, CYBB, DSG1, GSTP1, HNRNPU, HSPA1B, HSPA8,
5.03E-03	7.39	ACTB, CD9, DSG1, F11R, FSCN1, JUP, PATJ, PECAM1, RHOA, TLN1, VCL
5.07E-03	2.93	ADAM10, ATP5F1B, CCL5, CD151, CD47, CD9, CD99, FERMT3, FLNA, GNAI2, GP1BA, ITGA
5.13E-03	92.33	CCT2, CCT4, CCT8, TCP1
5.16E-03	6.59	CCT2, CCT4, CCT6A, CCT7, CCT8, HNRNPA2B1, HNRNPC, HNRNPU, HSP90AA1, TCP1, TNK
5.32E-03	3.66	ADH5, ANPEP, ATP2A3, CALM3, CD36, CDC42, CYB5R3, DSP, FLNA, GNAI2, HSP90AA1, JU
5.41E-03	4.53	CDC42, EEF1A1, EEF2, GNAI2, GNAQ, HSP90AA1, RAB10, RAB1B, RAB27B, RAB33B, RAC2
5.43E-03	3.10	B2M, CCL5, CD226, CD36, CD47, CD59, DHX9, FCER1G, FCN1, FLOT1, HLA-A, HLA-B, HSP9
5.48E-03	8.35	ADH5, CD36, GSR, GSTP1, PRDX1, PRDX2, PRDX6, PTGS1, S100A9, SOD1
5.72E-03	7.28	CD36, DHX9, FLOT1, HSPA1B, LTF, NPM1, PPIA, PSMA6, S100A8, S100A9, TFRC
5.96E-03	2.85	ACTB, DAZAP1, DHX9, EEF2, G3BP1, GAPDH, HNRNPA2B1, HNRNPA3, HNRNPC, HNRNPF,
6.00E-03	2.52	ACTB, ANXA5, ARL6IP5, BCAP31, CCL5, CD36, CFL1, CTSD, EIF5A, ENO1, FLNA, G6PD, GN/
6.10E-03	7.23	ANXA11, ANXA2, CCT2, CCT8, CYB5R3, HRNR, LYZ, PRDX6, RAP1B, STOM, TUBB
6.10E-03	7.23	ANXA11, ANXA2, CCT2, CCT8, CYB5R3, HRNR, LYZ, PRDX6, RAP1B, STOM, TUBB
6.42E-03	2.49	ARL6IP5, BCAP31, CALM3, CCL5, CD36, CDC42, EIF5A, ENO1, F11R, FLNA, FLOT1, GAPDH,
6.59E-03	3.30	CCL5, CSDE1, DHX9, EEF1A1, EEF2, EIF5A, GAPDH, HNRNPU, KRT17, NCL, NPM1, PKM, R
6.64E-03	2.43	AACS, ACTB, ATP2A3, ATP5F1B, CCT2, CCT4, CCT6A, CCT7, CCT8, CDC42, DDX60L, DHX9,
7.08E-03	11.54	ADAM10, CCL5, CD99, F11R, ITGB3, MSN, RHOA, SPN
7.36E-03	7.08	CCL5, CD99, FCER1G, LBP, PECAM1, PF4V1, PPIA, RAC2, S100A8, S100A9, THBS1
7.82E-03	2.19	ACTB, ALDOA, ANPEP, ANXA2, ATP5F1B, CAMP, CD151, CD36, CD53, CD9, CDC42, CFL1, (
7.86E-03	11.36	CD47, CD99, F11R, GP1BA, ITGB1, PECAM1, SELPLG, SPN
7.86E-03	11.36	CCT2, CCT4, CCT6A, CCT7, CCT8, HSP90AA1, TCP1, XRCC5
8.00E-03	4.94	CCT2, CCT4, CCT6A, CCT7, CCT8, CD36, FLNA, HNRNPU, HSP90AA1, JUP, NPM1, RPL11, T

8.20E-03	9.30	CD36, GSR, GSTP1, PRDX1, PRDX2, PRDX6, PTGS1, S100A9, SOD1
8.72E-03	11.19	ANXA2, ANXA5, CD36, CD9, GP1BA, KRT1, PRDX2, THBS1
8.95E-03	6.23	HNRNPA2B1, HNRNPA3, HNRNPC, HNRNPF, HNRNPH1, HNRNPH3, HNRNPU, HSPA8, NCL
8.96E-03	5.68	ADAM10, CCL5, CD47, CD9, CD99, GP1BA, ITGB3, LBP, MSN, RAC2, RHOA, SPN, THBS1
9.08E-03	7.86	CCL5, CD99, FCER1G, LBP, PECAM1, PF4V1, PPIA, RAC2, S100A8, S100A9
9.20E-03	3.15	ATIC, ATP5F1B, CAMP, CCL5, CD47, CDC42, DHX9, EIF5A, FCER1G, GAPDH, GSTP1, HNRN
9.33E-03	1.90	ACTB, ADAM10, ADH5, ALDOA, ANPEP, ANXA2, ARL6IP5, ATIC, ATP2A3, ATP5F1B, B2M, I
1.03E-02	2.24	AACS, ACTB, ANXA2, ARL6IP5, BCAP31, CANX, CCL5, CD36, CD47, CLTA, DHX9, FLNA, FLO
1.05E-02	2.61	ACTB, CALM3, CAMP, CCL5, CD36, CDC42, EEF1A1, ENO1, FLOT1, GNAI2, GNAQ, GSTP1, I
1.05E-02	6.12	CCT2, CCT4, CCT6A, CCT7, CCT8, HNRNPA2B1, HNRNPC, HNRNPU, HSP90AA1, TCP1, TNK
1.07E-02	10.87	ANPEP, F11R, HSPA1B, ITGB1, ITGB3, RPSA, SELPLG, TFRC
1.07E-02	2.61	ACTB, CALM3, CAMP, CCL5, CD36, CDC42, EEF1A1, ENO1, FLOT1, GNAI2, GNAQ, GSTP1, I
1.08E-02	5.58	CASP14, DSP, HRNR, KRT1, KRT10, KRT16, KRT17, KRT2, KRT5, KRT6A, KRT6B, KRT78, SOI
1.12E-02	3.74	ARPC5, CD47, CDC42, F11R, FLNA, FSCN1, HNRNPU, HSPA1B, ITGB3, LRP1, MYADM, NPM
1.13E-02	2.92	ADAM10, ANXA2, CCL5, CD226, CD36, CD47, CD9, DHX9, FCN1, FLNA, GP1BA, GSTP1, HL
1.15E-02	2.81	ADAM10, ATP5F1B, CCL5, CD151, CD47, CD9, CD99, FERMT3, FLNA, GNAI2, GP1BA, ITGA
1.16E-02	4.26	CDC42, EEF1A1, EEF2, GNAI2, GNAQ, HSP90AA1, RAB10, RAB1B, RAB27B, RAB33B, RAC2
1.16E-02	4.26	CDC42, EEF1A1, EEF2, GNAI2, GNAQ, HSP90AA1, RAB10, RAB1B, RAB27B, RAB33B, RAC2
1.17E-02	10.71	ANPEP, F11R, HSPA1B, ITGB1, ITGB3, RPSA, SELPLG, TFRC
1.19E-02	13.62	ANK1, CDC42, FSCN1, ILK, MSN, PATJ, RHOA
1.19E-02	13.62	ANK1, CDC42, FSCN1, ILK, MSN, PATJ, RHOA
1.19E-02	13.62	CAVIN2, DSP, FLNA, HINT1, ITGB3, PLEK, YWHAG
1.19E-02	2.60	ACTB, ANXA2, ARL6IP5, BCAP31, CCL5, CD226, CD36, CD47, CD9, DHX9, ENO1, FCN1, FLN
1.20E-02	2.71	ADAM10, ANK1, ANXA2, CANX, CAVIN2, CD36, CFL1, CLTA, DSG1, DSP, EEF1A1, FLOT1, F
1.25E-02	2.15	ACTB, ADAM10, ALDOA, ATIC, ATP5F1B, CALM3, CAMP, CCL5, CD36, CDC42, EEF1A1, EN
1.27E-02	4.03	BCAP31, CALM3, CCT2, CCT4, CCT6A, CCT7, CCT8, CD36, FLNA, HSP90AA1, HSPA8, HSPD1
1.27E-02	18.61	ANXA11, B2M, HSP90AA1, HSPA8, PKM, YWHAE
1.33E-02	3.16	CCL5, CSDE1, DHX9, EEF1A1, EEF2, EIF5A, GAPDH, HNRNPU, KRT17, NCL, NPM1, PKM, RH
1.34E-02	2.38	AACS, ACTB, ATP2A3, ATP5F1B, CCT2, CCT4, CCT6A, CCT7, CCT8, CDC42, DDX60L, DHX9,
1.37E-02	2.46	ACTB, ADH5, CALM3, CAMP, CCL5, CD36, CYBB, DSG1, FLNA, G6PD, GNAI2, GNAQ, GSTP1
1.42E-02	28.97	ACTB, DSP, F11R, PECAM1, VCL
1.42E-02	10.41	ACTB, B2M, KRT1, LTF, LYZ, PRDX1, SLC2A1, SOD1
1.43E-02	2.77	ACTB, BCAP31, CALM3, CAMP, CANX, CD36, CD9, CYBB, DHX9, FCER1G, FLNA, FLOT1, GN
1.43E-02	2.10	ACTB, ANXA2, ANXA5, ARL6IP5, ATP2A3, B2M, BCAP31, CALM3, CCL5, CCT2, CCT4, CD36
1.46E-02	2.54	ACTB, ANXA5, ARL6IP5, BCAP31, CCL5, CFL1, CTSD, EIF5A, ENO1, FLNA, G6PD, GNAI2, GS
1.47E-02	2.93	ACTB, ADAM10, B2M, CCL5, CD226, CD36, CD47, CD59, CD99, DHX9, FCER1G, FCN1, HLA
1.48E-02	2.57	ADAM10, ATP5F1B, CCL5, CD151, CD47, CD9, CD99, FCER1G, FERMT3, FLNA, GNAI2, GP1
1.49E-02	5.40	CCL5, CD47, CD9, CD99, FCER1G, LBP, PECAM1, PF4V1, PPIA, RAC2, S100A8, S100A9, THI
1.50E-02	61.55	FLNA, GP1BA, GP1BB, PLEK
1.54E-02	2.33	AACS, ACTB, ARL6IP5, BCAP31, CANX, CCL5, CD36, CD47, CLTA, DHX9, FLNA, FLOT1, HNR
1.58E-02	5.87	ADAM10, CCL5, CD47, CD99, CDC42, F11R, ITGB3, MSN, PECAM1, RHOA, SPN, THBS1
1.65E-02	2.23	ACTB, ADAM10, ANK1, ARPC5, CALM3, CAMP, CANX, CCT4, CCT8, CD36, CDC42, CFL1, CY
1.69E-02	2.36	AACS, ACTB, ATP2A3, ATP5F1B, CCT2, CCT4, CCT6A, CCT7, CCT8, CDC42, DDX60L, DHX9,
1.82E-02	27.26	CDC42, FSCN1, MSN, PATJ, RHOA
1.92E-02	2.47	ANK1, ANXA2, ATP2A3, BCAP31, CD36, CDC42, CLTA, DHX9, FLNA, HNRNPA2B1, HNRNPU
1.99E-02	3.25	ANXA2, B2M, CALM3, CLTA, CYBB, FCER1G, FLNA, FLOT1, GNAI2, GNAQ, GNG5, GP1BA, (
2.02E-02	2.94	ACTB, ARL6IP5, CALM3, CCL5, CD36, CPN1, CYBB, DHX9, DSG1, FLNA, FLOT1, G6PD, HNR

2.04E-02	3.08	ACTB, ALDOA, ARPC5, CD47, CDC42, CFL1, DSP, F11R, FLNA, FSCN1, ITGB1, ITGB3, JUP, L
2.05E-02	2.13	ACTB, ADAM10, ALDOA, ATIC, ATP5F1B, CALM3, CAMP, CCL5, CD36, CDC42, EEF1A1, EN
2.06E-02	4.29	BCAP31, EIF5A, ENO1, GNAI2, GSTP1, HSPA1B, ITGA6, PPIA, PRDX2, RPL11, S100A8, S100
2.08E-02	4.54	CDC42, EEF1A1, EEF2, GNAI2, GNAQ, GNG5, RAB10, RAB1B, RAB27B, RAB33B, RAC2, RAP
2.09E-02	6.31	ANXA2, CCT2, CCT8, CTSD, CYB5R3, HRNR, HSP90AA1, HSPA8, LYZ, PRDX6, TUBB
2.09E-02	2.93	ADAM10, ATIC, ATP5F1B, CAMP, CCL5, CD47, CDC42, DHX9, EIF5A, FCER1G, GAPDH, GST
2.18E-02	1.94	ACTB, ADAM10, ANPEP, ANXA2, ARPC5, ATIC, ATP5F1B, B2M, CALM3, CAMP, CD9, CDC4
2.18E-02	3.23	ANXA2, B2M, CDC42, F11R, FLNA, HNRNPC, HNRNPU, HSPA1B, HSPA8, ITGB3, MYADM, I
2.24E-02	9.73	GSR, GSTP1, PRDX1, PRDX2, PRDX6, PTGS1, S100A9, SOD1
2.25E-02	2.06	ACTB, ANXA2, ARL6IP5, ATP2A3, BCAP31, CALM3, CAMP, CCL5, CCT2, CCT4, CCT6A, CCT
2.32E-02	52.75	CSDE1, DHX9, HNRNPU, SYNCRIP
2.33E-02	6.23	CFL1, EEF1A1, FSCN1, ITGB1, ITGB3, MYADM, MYH9, PLEK, PTPRJ, RHOA, TLN1
2.33E-02	3.55	ADAM10, ATP5F1B, CCL5, CD151, CD47, CD99, FERMT3, GNAI2, ITGA6, ITGB1, ITGB3, LB
2.34E-02	1.91	ACTB, ADAM10, ANXA2, ANXA5, ARL6IP5, ATP5F1B, B2M, CALM3, CCL5, CD36, CD47, CD
2.40E-02	12.10	CD47, DSP, FLOT1, ITGB1, ITGB3, JUP, MYADM
2.45E-02	6.19	ACTB, ADAM10, ANXA2, FLOT1, JUP, MSN, MYH9, RAB10, TLN1, TNKS1BP1, VCL
2.47E-02	2.47	ACTB, ANXA5, ARL6IP5, BCAP31, CCL5, CFL1, CTSD, EIF5A, ENO1, FLNA, G6PD, GNAI2, GS
2.59E-02	6.16	DAZAP1, HNRNPA2B1, HNRNPF, HNRNPH1, HNRNPH3, HNRNPU, HSPA8, NCL, NPM1, PR
2.79E-02	15.95	CDC42, RAB10, RAB1B, RAB27B, RAP1B, RHOA
2.80E-02	2.20	ADAM10, ANPEP, ATIC, ATP2A3, CASP14, CCL5, CCT2, CCT4, CCT6A, CCT7, CCT8, CDC42,
2.86E-02	5.07	ANXA2, B2M, CANX, CD36, CD9, CLTA, FCER1G, FLOT1, ITGB1, ITGB3, LRP1, M6PR, TFRC
2.87E-02	24.39	CDC42, FSCN1, MSN, PATJ, RHOA
2.89E-02	6.83	ADAM10, CCL5, CD47, CD99, ITGB3, LBP, RAC2, RHOA, SPN, THBS1
2.91E-02	4.70	ARL6IP5, ATP2A3, BCAP31, EIF5A, ENO1, HINT1, PPIA, RPL11, S100A8, S100A9, SOD1, TM
3.03E-02	6.05	ANPEP, F11R, FCN1, HSPA1B, ITGB1, ITGB3, KRT6A, PPIA, RPSA, SELPLG, TFRC
3.18E-02	2.75	ADAM10, ATIC, B2M, CCL5, CYBB, ENO1, F11R, FCER1G, FLNA, G6PD, GALE, H2AC20, H2E
3.26E-02	6.73	CDC42, FSCN1, HSP90AA1, ITGB1, MSN, MYH9, PATJ, RAB10, RAP1B, RHOA
3.31E-02	11.46	ANXA2, CD47, DSP, ITGB1, JUP, RAB10, RPSA
3.43E-02	9.14	HNRNPA2B1, HNRNPA3, HNRNPC, HNRNPF, HNRNPH1, HNRNPU, SF3A3, SYNCRIP
3.44E-02	46.16	CSDE1, DHX9, HNRNPU, SYNCRIP
3.44E-02	2.43	AACS, ARL6IP5, BCAP31, CANX, CCL5, CD36, CLTA, FLNA, HSP90AA1, HSPA8, HSPA9, HSP
3.55E-02	23.17	CDC42, FSCN1, MSN, PATJ, RHOA
3.66E-02	4.32	ATP2A3, CCT2, CCT4, CCT6A, CCT7, CCT8, DDX60L, DHX9, G3BP1, HSP90AA1, HSPA1B, HS
3.67E-02	11.27	ACTB, CALM3, CDC42, G6PD, RHOA, YWHAE, YWHAH
3.73E-02	9.03	ANXA2, CCT2, CCT8, CYB5R3, HRNR, LYZ, PRDX6, TUBB
3.88E-02	3.55	ACTB, B2M, CD36, F11R, FLG2, HRNR, INPP5D, ITGB1, ITGB3, KRT1, KRT16, LTF, LYZ, PEC
3.92E-02	2.67	ANXA2, CALM3, CSDE1, DHX9, FLNA, G6PD, GAPDH, GNAI2, GNAQ, GSTP1, HNRNPU, HSP
3.94E-02	7.55	ANXA2, CD36, CD9, FCER1G, FLOT1, ITGB1, ITGB3, LRP1, TFRC
4.07E-02	11.08	DHX9, EEF2, PSMA6, RPL11, RPL8, RPS23, VIM
4.08E-02	2.88	ACTB, CALM3, CCL5, CCT2, CCT4, CDC42, EEF1A1, GNAQ, GSTP1, HNRNPA2B1, HNRNPU,
4.10E-02	6.55	ADH5, CD36, GSR, GSTP1, PRDX1, PRDX2, PRDX6, PTGS1, S100A9, SOD1
4.10E-02	5.84	B2M, CD226, CD59, DCD, GAPDH, HLA-A, HLA-B, LTF, LYZ, PRDX1, TUBB
4.26E-02	2.41	ACTB, CALM3, CAMP, CCL5, CD36, FLOT1, G6PD, GNAI2, GNAQ, GSTP1, HSP90AA1, HSPA
4.38E-02	4.52	CSDE1, DAZAP1, DHX9, HNRNPA2B1, HNRNPC, HNRNPU, HSPA8, NCL, NPM1, PRDX6, PT
4.49E-02	3.38	ADAM10, ATP5F1B, CCL5, CD151, CD47, CD99, FERMT3, GNAI2, ITGA6, ITGB1, ITGB3, LB
4.74E-02	8.71	CSDE1, DHX9, HNRNPA2B1, HNRNPC, HNRNPU, NPM1, PTBP1, SYNCRIP
4.84E-02	2.85	ACTB, ANXA2, B2M, CDC42, EEF2, FCER1G, FLNA, G6PD, GP1BA, GP1BB, HLA-B, HSPA1B,

4.88E-02	4.48	ALDOA, CDC42, CFL1, F11R, FLNA, ILK, ITGB3, MSN, MYADM, MYH9, MYL12B, RAC2, RCC
4.91E-02	41.03	MSN, RAC2, S100A8, S100A9
5.07E-02	1.80	ACTB, ADAM10, ANXA2, ANXA7, ARL6IP5, ATP2A3, B2M, BCAP31, CALM3, CAMP, CCL5,
5.12E-02	7.29	CD36, ITGB3, JUP, PTPRJ, RCC2, RHOA, THBS1, TLN1, VCL
5.12E-02	7.29	CAMP, GAPDH, H2BC21, HLA-A, KRT6A, LTF, LYZ, PF4V1, S100A9
5.12E-02	8.61	ITGA6, ITGB3, PTPRJ, RCC2, RHOA, THBS1, TLN1, VCL
5.13E-02	6.37	ANPEP, F11R, FCN1, HSPA1B, ITGB1, ITGB3, KRT6A, RPSA, SELPLG, TFRC
5.24E-02	5.68	B2M, CD36, CD9, CYBB, HLA-A, HLA-B, LRP1, M6PR, RAB10, RAC2, TFRC
5.45E-02	3.33	ARL6IP5, ATP2A3, BCAP31, EIF5A, ENO1, GNAI2, GSTP1, HINT1, HSPA1B, ITGA6, PPIA, PR
5.51E-02	13.95	CDC42, CFL1, EEF1A1, HSPA8, LTF, STOM
5.55E-02	14.71	AACS, ACTB, ADAM10, ADH5, ALDOA, ANK1, ANPEP, ANTXR2, ANXA11, ANXA2, ANXA5,
5.83E-02	4.14	CCL5, CD226, CD36, DHX9, FCN1, HLA-A, HLA-B, HSP90AA1, HSPD1, LBP, LTF, MATR3, PR
5.85E-02	3.44	ATP2A3, ATP5F1B, CCT2, CCT4, CCT6A, CCT7, CCT8, DDX60L, DHX9, G3BP1, HSP90AA1,
5.96E-02	8.42	CFL1, EEF1A1, ITGB1, ITGB3, PLEK, PTPRJ, RHOA, TLN1
6.01E-02	10.37	ANXA2, CD36, CD9, GP1BA, KRT1, PRDX2, THBS1
6.19E-02	7.10	ANK1, ATP2A3, CALM3, FLNA, HSP90AA1, SRI, YWHAE, YWHAH, YWHAZ
6.25E-02	13.61	CCT2, CCT4, HNRNPA2B1, HSP90AA1, TCP1, XRCC5
6.35E-02	20.14	DAZAP1, HNRNPC, HNRNPH1, HNRNPU, PTBP1
6.44E-02	3.29	ADAM10, ATP5F1B, CCL5, CD151, CD47, CD99, FERMT3, GNAI2, ITGA6, ITGB1, ITGB3, LB
6.46E-02	3.89	ARPC5, CD36, CDC42, FSCN1, HSP90AA1, HSPA1B, HSPA8, MSN, MYADM, PFN1, PLEK, R
6.65E-02	5.53	ANXA2, BCAP31, DHX9, FLNA, HSP90AA1, JUP, MSN, RAC2, STOM, UBE2L3, YWHAE
6.73E-02	1.82	AACS, ACTB, ADAM10, ANK1, ANPEP, ANXA2, ANXA5, ARL6IP5, ARPC5, ATP2A3, ATP5F1
6.76E-02	137.89	CCT2, CCT4, TCP1
6.76E-02	137.89	FLNA, GP1BA, GP1BB
6.80E-02	3.53	ANXA2, CCT2, CCT4, CCT6A, CCT7, CCT8, CD47, CDC42, FSCN1, G3BP1, HNRNPA2B1, HSP
7.00E-02	6.99	CCL5, FCER1G, LBP, PF4V1, PPIA, RAC2, S100A8, S100A9, THBS1
7.06E-02	13.29	F11R, MSN, MYADM, PECAM1, RAP1B, VCL
7.23E-02	10.05	ANXA2, CD36, CD9, GP1BA, KRT1, PRDX2, THBS1
7.23E-02	10.05	DSP, FLOT1, ITGB1, JUP, SLC2A1, VCL, YWHAH
7.37E-02	2.00	ADAM10, ARL6IP5, BCAP31, CALM3, CCL5, CD226, CD36, CD47, CDC42, EIF5A, ENO1, F1
7.58E-02	19.30	CSDE1, DHX9, HNRNPU, PKM, SYNCRIP
7.64E-02	5.44	CCT2, CCT4, CCT6A, CCT7, CCT8, DHX9, FLOT1, HNRNPA2B1, HNRNPU, NPM1, TCP1
7.69E-02	1.84	ACTB, ADAM10, ANPEP, ANXA2, ARPC5, ATIC, ATP5F1B, B2M, CALM3, CAMP, CD9, CDC4
7.86E-02	2.36	ARL6IP5, BCAP31, CCL5, CD226, CD36, CDC42, EIF5A, F11R, FLNA, FLOT1, GAPDH, GNAI2
7.97E-02	1.80	ACTB, ADAM10, ANXA2, ANXA5, ANXA7, ARL6IP5, ATP5F1B, B2M, CALM3, CCL5, CD36,
8.00E-02	8.05	ITGA6, ITGB3, PTPRJ, RCC2, RHOA, THBS1, TLN1, VCL
8.14E-02	2.28	AACS, ANXA2, ARL6IP5, BCAP31, CCL5, CD226, CD36, CDC42, EIF5A, F11R, FLNA, FLOT1,
8.36E-02	5.38	ANK1, DSG1, EEF1A1, G6PD, GNAI2, GNAQ, GNG5, JUP, MYH9, OSBPL2, RHOA
8.50E-02	2.07	ACTB, ADAM10, ALDOA, ANXA2, B2M, CAMP, CD36, CD53, CDC42, CFL1, CLIC1, CYBB, F1
8.65E-02	9.75	CD36, CYBB, GNAI2, GSTP1, PRDX1, PRDX2, SOD1
8.89E-02	3.61	DAZAP1, DHX9, HNRNPA2B1, HNRNPA3, HNRNPC, HNRNPF, HNRNPH1, HNRNPH3, HNRN
8.96E-02	12.68	DSP, ITGB1, JUP, SLC2A1, VCL, YWHAH
9.28E-02	1.79	ACTB, ADAM10, ALDOA, ANXA11, ANXA2, ARPC5, ATP5F1B, B2M, CAMP, CANX, CASP14,
9.28E-02	1.79	ACTB, ADAM10, ALDOA, ANXA11, ANXA2, ARPC5, ATP5F1B, B2M, CAMP, CANX, CASP14,
9.28E-02	1.79	ACTB, ADAM10, ALDOA, ANXA11, ANXA2, ARPC5, ATP5F1B, B2M, CAMP, CANX, CASP14,
9.46E-02	2.93	ADH5, B2M, CAMP, CCL5, CD36, DCD, FCER1G, GSDMA, GSTP1, H2BC21, HLA-A, HLA-B,
9.85E-02	1.93	AACS, ADAM10, ANXA2, ARL6IP5, ATP2A3, BCAP31, CALM3, CCL5, CD226, CD36, CD47,

1.00E-01	12.40	CD36, FCN1, ITGB3, LRP1, RAC2, THBS1
1.00E-01	12.40	ANK1, CDC42, ILK, MSN, PATJ, RHOA
1.03E-01	9.47	DDX60L, DHX9, HNRNPU, HSPD1, MSN, TFRC, VIM
1.04E-01	2.72	ACTB, ANXA2, B2M, CDC42, EEF2, FCER1G, FLNA, G6PD, GP1BA, GP1BB, HLA-B, HSPA1B,
1.06E-01	1.76	ACTB, ADAM10, ANXA2, ANXA7, ARL6IP5, ATP2A3, B2M, BCAP31, CALM3, CAMP, CCL5, (
1.06E-01	7.72	RPL11, RPL8, RPLP0, RPS15, RPS15A, RPS23, RPS3A, RPSA
1.06E-01	6.60	ANXA2, CD36, CD9, GP1BA, ITGB1, KRT1, MMRN1, PRDX2, THBS1
1.07E-01	5.81	ANXA2, CD36, CD9, FLNA, GP1BA, ITGB1, KRT1, MMRN1, PRDX2, THBS1
1.07E-01	5.81	BCAP31, EIF5A, ENO1, PPIA, RPL11, S100A8, S100A9, SOD1, UBQLN1, VDAC2
1.10E-01	2.65	ACTB, ANXA2, B2M, CDC42, EEF2, FCER1G, FLNA, G6PD, GP1BA, GP1BB, HLA-B, HSPA1B,
1.12E-01	2.11	ACTB, ADAM10, ANK1, ARPC5, CALM3, CANX, CCT8, CD36, CDC42, CFL1, CYBB, EEF1A1, I
1.12E-01	5.78	ANK1, DSG1, G6PD, GNAI2, GNAQ, GNG5, JUP, MYH9, OSBPL2, RHOA
1.13E-01	7.64	CCL5, FCER1G, LBP, PF4V1, PPIA, RAC2, S100A8, S100A9
1.13E-01	7.64	CCT2, CCT4, CCT6A, CCT7, CCT8, HNRNPA2B1, TCP1, XRCC5
1.14E-01	4.13	ACTB, ADAM10, ANK1, ARL6IP5, F11R, FLNA, FLOT1, ITGB1, JUP, LRP1, MYADM, RAB10, !
1.17E-01	2.51	ADAM10, ANPEP, ANXA2, ATP5F1B, CAMP, CDC42, CYBB, DSP, FLNA, G6PD, GNG5, HNRI
1.19E-01	6.50	DAZAP1, DHX9, HNRNPA2B1, HNRNPU, HSPA8, NCL, NPM1, PRDX6, PTBP1
1.25E-01	3.67	ANXA11, ANXA2, ANXA5, ANXA7, CD151, CTSD, FCN1, HRNR, HSP90AA1, KRT1, LGALS3B
1.26E-01	6.45	CSDE1, DAZAP1, DHX9, HNRNPU, HSPA8, NCL, PRDX6, SYNCRIP, TNKS1BP1
1.26E-01	2.15	ACTB, ADAM10, B2M, CAMP, CCL5, CD151, CD47, CD9, CDC42, DAZAP1, DHX9, GNAI2, G
1.28E-01	1.96	ACTB, ANXA2, B2M, BCAP31, CALM3, CAMP, CCL5, CCT2, CCT4, CCT6A, CCT7, CCT8, CD3(
1.30E-01	7.49	ACTB, EEF1A1, FLOT1, MYADM, MYH9, SLC2A1, SPTBN1, TPM4
1.30E-01	1.82	AACS, ACTB, ADAM10, ALDOA, ANK1, ANPEP, ANXA2, ANXA5, ANXA7, ARL6IP5, ARPC5, /
1.36E-01	5.64	CDC42, CFL1, EEF1A1, ITGB1, ITGB3, PLEK, PTPRJ, RHOA, SPTBN1, TLN1
1.39E-01	2.48	ACTB, ADH5, CAMP, CCL5, CD36, CYBB, FLNA, GNAI2, GNAQ, GSTP1, ITGB3, JUP, LBP, LRI
1.39E-01	7.41	DAZAP1, HNRNPA2B1, HNRNPU, HSPA8, NCL, NPM1, PRDX6, PTBP1
1.40E-01	11.62	ANXA2, ANXA5, CD9, GP1BA, KRT1, THBS1
1.42E-01	2.93	ANXA2, CSDE1, DAZAP1, DHX9, HNRNPA2B1, HNRNPA3, HNRNPC, HNRNPF, HNRNPH1, H
1.54E-01	3.44	ACTB, B2M, CCL5, CD47, CD9, F11R, FERMT3, FLOT1, GP1BA, HLA-A, HSPD1, MMRN1, M
1.54E-01	2.99	B2M, CD226, CD36, CD47, CD59, FCER1G, FCN1, HLA-A, HLA-B, HSPD1, INPP5D, KRT1, LB
1.56E-01	8.82	CAMP, GAPDH, H2BC21, KRT6A, LTF, PF4V1, S100A9
1.56E-01	28.40	ADH5, GAPDH, S100A8, S100A9
1.56E-01	28.40	ADH5, GAPDH, S100A8, S100A9
1.58E-01	7.27	CDC42, FERMT3, FLNA, ILK, ITGB3, MYADM, RCC2, RHOA
1.68E-01	2.02	ADH5, ALDOA, ANPEP, ANXA2, ANXA7, BCAP31, CANX, CD36, CPN1, CSDE1, CTSD, DHX9,
1.68E-01	15.97	CCT2, CCT4, HSP90AA1, TCP1, XRCC5
1.69E-01	1.83	ACTB, ALDOA, ANXA11, ANXA2, ANXA7, CANX, CCT4, CCT6A, CSDE1, DAZAP1, DCD, DDX(
1.71E-01	2.69	CCL5, CSDE1, DHX9, EEF1A1, EEF2, EIF5A, GAPDH, HNRNPU, KRT17, NCL, NPM1, PKM, RI
1.71E-01	1.83	AACS, ACTB, ADAM10, ADH5, ALDOA, ANK1, ANPEP, ANXA2, ANXA5, ANXA7, ARL6IP5, AI
1.75E-01	6.17	CALM3, CD36, DSP, FLNA, GNAQ, JUP, SRI, YWHAE, YWHAH
1.78E-01	2.21	AACS, ARL6IP5, BCAP31, CCL5, CD226, CD36, CDC42, EIF5A, F11R, FLNA, FLOT1, GAPDH,
1.80E-01	2.57	ACTB, BCAP31, CALM3, CAMP, CANX, CD36, CD9, CYBB, FCER1G, FLNA, GNAI2, HSP90AA
1.89E-01	5.41	CD36, CD47, CYBB, DHX9, GSTP1, LBP, LTF, PTPRJ, SPN, THBS1
1.89E-01	5.41	CD36, CD47, CYBB, DHX9, GSTP1, LBP, LTF, PTPRJ, SPN, THBS1
1.93E-01	2.94	ACTB, CAMP, CD36, CYBB, DHX9, FLNA, FLOT1, GNAI2, GNAQ, ITGB3, JUP, LRP1, NCL, NP
1.94E-01	15.44	ACTB, F11R, ITGB1, PECAM1, VCL
1.94E-01	15.44	ADAM10, CCL5, ITGB3, RHOA, SPN

1.94E-01	15.44	BCAP31, CANX, HLA-A, HLA-B, HSPA8
1.96E-01	3.24	CCL5, CSDE1, DHX9, EEF2, EIF5A, GAPDH, HNRNPU, KRT17, NCL, NPM1, PKM, RHOA, RTN
1.96E-01	8.48	CCL5, CDC42, CFL1, EEF1A1, HSPA8, LTF, STOM
2.00E-01	4.47	CD36, DHX9, FLOT1, HSPA1B, JUP, LTF, NPM1, PPIA, PSMA6, S100A8, S100A9, TFRC
2.03E-01	6.99	CCT2, CCT4, CCT6A, CCT7, CCT8, RCC2, TCP1, XRCC5
2.03E-01	4.15	ADAM10, B2M, BCAP31, CD59, CD9, CLTA, HLA-A, HLA-B, HSPA8, HSPD1, M6PR, RAB27B
2.05E-01	6.03	CD36, G6PD, GNAI2, GSTP1, PRDX2, RAC2, RHOA, SOD1, THBS1
2.09E-01	1.99	ARL6IP5, ATP2A3, BCAP31, CALM3, CCL5, CD36, CDC42, CFL1, EIF5A, ENO1, F11R, FLNA,
2.10E-01	2.46	ANTXR2, ANXA7, ARL6IP5, ATP2A3, B2M, BCAP31, CANX, CD59, CDC42, CYB5R3, CYBB, E
2.12E-01	3.11	B2M, CCL5, CD226, CD47, CD59, DHX9, FCER1G, FCN1, HLA-A, HLA-B, HSP90AA1, HSPD1,
2.14E-01	2.46	ACTB, ANXA2, ANXA5, ATP2A3, B2M, CALM3, F11R, FLNA, GAPDH, GNAI2, GNAQ, GSTP1
2.18E-01	2.84	ALDOA, ATP2A3, ATP5F1B, BCAP31, CALM3, CCL5, FLNA, GP1BA, GP1BB, GSR, ITGB3, NP
2.22E-01	2.76	ADH5, ANXA11, ANXA2, ANXA5, ANXA7, CAMP, CAVIN2, CD36, FERMT3, GSDMA, GSTP1,
2.23E-01	14.94	DSP, KRT1, KRT10, KRT2, THBS1
2.25E-01	4.31	AACS, ACTB, ADAM10, ADH5, ALDOA, ANK1, ANPEP, ANTXR2, ANXA11, ANXA2, ANXA5, /
2.27E-01	5.95	ANPEP, F11R, FCN1, HSPA1B, ITGB1, ITGB3, RPSA, SELPLG, TFRC
2.29E-01	68.94	PRDX1, PRDX2, PRDX6
2.29E-01	68.94	PRDX1, PRDX2, PRDX6
2.30E-01	2.58	ACTB, ADAM10, B2M, CAMP, CCL5, CD151, CD226, CD47, FCER1G, HLA-A, HSPD1, INPP5
2.33E-01	3.85	ADH5, B2M, CAMP, CCL5, CD36, GSTP1, LBP, LTF, PF4V1, PRDX2, RHOA, S100A8, S100A9
2.34E-01	3.32	DAZAP1, DHX9, HNRNPA2B1, HNRNPA3, HNRNPC, HNRNPF, HNRNPH1, HNRNPH3, HNRN
2.36E-01	5.26	CD36, CD47, CYBB, DHX9, GSTP1, LBP, LTF, PTPRJ, SPN, THBS1
2.36E-01	5.26	CD36, CD47, CYBB, DHX9, GSTP1, LBP, LTF, PTPRJ, SPN, THBS1
2.47E-01	24.61	CCL5, S100A8, S100A9, THBS1
2.63E-01	2.15	ACTB, ADAM10, ALDOA, CALM3, CAMP, CCL5, CD36, CDC42, EEF1A1, ENO1, FLOT1, GAP
2.64E-01	2.39	ACTB, CALM3, CAMP, CCL5, CD36, CDC42, EEF1A1, FLOT1, GNAQ, GSTP1, HNRNPU, HSP9
2.64E-01	8.06	CCL5, FERMT3, ITGA2B, ITGA6, ITGB1, ITGB3, MMRN1
2.69E-01	4.70	CANX, DHX9, NPM1, RPL11, RPL8, RPLP0, RPS15, RPS15A, RPS23, RPS3A, RPSA
2.70E-01	5.17	CD36, FSCN1, HSP90AA1, HSPA1B, MSN, PFN1, PLEK, RAP1B, RHOA, TFRC
2.70E-01	3.79	ARPC5, CD47, CDC42, F11R, FLNA, FSCN1, ITGB3, LRP1, MYADM, PFN1, PLEK, RAC2, RHO
2.75E-01	2.86	ADAM10, BCAP31, CLIC1, CYBB, GAPDH, GSDMA, HRNR, HSP90AA1, HSPA1B, HSPA8, ITG
2.79E-01	10.14	NPM1, RPL11, RPLP0, RPS15, RPSA, XRCC5
2.79E-01	10.14	CDC42, FLNA, ILK, ITGB3, MYADM, RCC2
2.79E-01	10.14	DSC1, DSG1, DSP, FCER1G, RHOA, SLC2A3
2.79E-01	10.14	ANXA2, CAVIN2, GSDMA, HSPA8, THBS1, TLN1
2.86E-01	2.86	ACTB, ALDOA, ARPC5, CD47, CDC42, CFL1, F11R, FLNA, FSCN1, ITGB1, ITGB3, LRP1, MYAI
3.01E-01	4.63	CD36, CYBB, G6PD, GNAI2, GSTP1, PRDX1, PRDX2, RAC2, RHOA, SOD1, THBS1
3.05E-01	3.39	ACTB, B2M, CCL5, CD226, CD47, FLNA, GP1BA, GP1BB, HLA-A, HSPD1, INPP5D, LBP, PLEK
3.06E-01	23.07	CFL1, EEF1A1, HSPA8, STOM
3.11E-01	3.24	ATP2A3, B2M, BCAP31, CALM3, CCL5, FLNA, GNAI2, GP1BA, GP1BB, ITGB3, LTF, S100A8,
3.26E-01	6.51	ADAM10, CCL5, CD47, ITGB3, MSN, RHOA, SPN, THBS1
3.27E-01	7.77	ACTB, CALM3, CDC42, G6PD, RHOA, YWHAE, YWHAH
3.27E-01	3.95	BCAP31, HSP90AA1, HSPA8, HSPD1, M6PR, RAC2, RPL11, STOM, UBE2L3, YWHAB, YWHA
3.27E-01	1.86	AACS, ADAM10, ARL6IP5, BCAP31, CALM3, CCL5, CD226, CD36, CD47, CDC42, EIF5A, ENO
3.34E-01	9.78	DHX9, FLOT1, G3BP1, GAPDH, HSP90AA1, HSPD1
3.34E-01	9.78	BCAP31, EIF5A, RPL11, S100A8, S100A9, SOD1
3.45E-01	6.45	ADAM10, CCL5, CD99, F11R, ITGB3, MSN, RHOA, SPN

3.50E-01	7.68	ARPC5, CDC42, FSCN1, HSP90AA1, ITGB1, RAC2, VCL
3.54E-01	5.59	ALDOA, CCT2, CCT4, CCT7, CCT8, CD9, MYH9, TCP1, VDAC2
3.61E-01	55.15	PRDX1, PRDX2, PRDX6
3.63E-01	4.53	CCL5, CD226, DHX9, FCN1, HLA-A, HLA-B, HSP90AA1, LBP, MATR3, TTLL12, XRCC5
3.65E-01	6.39	CD36, CDC42, FLNA, ILK, ITGB3, JUP, MYADM, PTPRJ
3.65E-01	6.39	ARL6IP5, ATP2A3, BCAP31, CTSD, HSPD1, RHOA, S100A8, S100A9
3.65E-01	9.61	PTPRJ, RCC2, RHOA, THBS1, TLN1, VCL
3.65E-01	9.61	PTPRJ, RCC2, RHOA, THBS1, TLN1, VCL
3.67E-01	2.07	ADAM10, ANK1, ANTXR2, ANXA7, ARL6IP5, ATP2A3, B2M, BCAP31, CANX, CD59, CDC42,
3.73E-01	13.23	ANXA2, FLNA, GP1BA, GP1BB, LTF
3.74E-01	2.41	ANTXR2, ANXA7, ARL6IP5, ATP2A3, B2M, BCAP31, CANX, CD59, CDC42, CYB5R3, CYBB, E
3.83E-01	3.06	ACTB, ARL6IP5, CCL5, DHX9, FLNA, FLOT1, HNRNPU, HSPA1B, HSPA8, JUP, MSN, NPM1, F
3.85E-01	4.16	ACTB, ANK1, ARL6IP5, F11R, FLNA, FLOT1, ITGB1, JUP, LRP1, RAB10, SPTBN1, TSPAN14
3.85E-01	6.34	ACTB, ANXA2, CFL1, HNRNPA2B1, HNRNPU, MATR3, PSMA6, VIM
3.88E-01	2.87	ADAM10, ATIC, B2M, CCL5, ENO1, F11R, FCER1G, FLNA, G6PD, GALE, HSP90AA1, JUP, M'
3.91E-01	2.41	ANTXR2, ANXA7, ARL6IP5, ATP2A3, B2M, BCAP31, CANX, CD59, CDC42, CYB5R3, CYBB, E
3.98E-01	9.45	F11R, MSN, MYADM, PECAM1, RAP1B, VCL
4.01E-01	1.99	ACTB, ANXA2, ANXA5, ARL6IP5, ATP2A3, BCAP31, CALM3, CCL5, CCT2, CCT4, CDC42, CTS
4.20E-01	12.86	CCT2, HSPA1B, HSPA8, HSPA9, HSPD1
4.24E-01	2.53	ALDOA, CAMP, CDC42, CFL1, CYBB, F11R, FLNA, ILK, ITGB1, ITGB3, JUP, KRT1, MSN, MYA
4.27E-01	4.87	ACTB, ARPC5, CFL1, FLOT1, FSCN1, ILK, ITGB1, ITGB3, RAC2, RHOA
4.33E-01	9.29	DSP, HSP90AA1, KRT15, KRT5, VIM, YWHAE
4.35E-01	3.61	CAMP, CD36, DCD, FCER1G, GSDMA, H2BC21, HLA-A, KRT6A, LBP, LTF, LYZ, S100A8, S10(
4.51E-01	20.50	CD36, CD9, FCER1G, ITGB1
4.55E-01	3.81	ADAM10, ANXA2, CPN1, ENO1, FLNA, GP1BA, GP1BB, HSPD1, MYH9, PGK1, SRGN, THBS:
4.57E-01	7.33	ANXA2, B2M, CD151, CD36, CDC42, FLOT1, LRP1
4.67E-01	5.37	ANXA2, CAVIN2, CD9, FLOT1, MYH9, OSBPL2, S100A9, SOD1, SPTBN1
4.68E-01	3.41	ARL6IP5, CD36, G6PD, GSR, GSTP1, HSPA1B, KRT1, PNPLA8, PPIA, PRDX1, PRDX2, PRDX6,
4.71E-01	9.14	ALDOA, FERMT3, MMRN1, QSOX1, SRGN, THBS1
4.71E-01	12.51	ADAM10, CCL5, ITGB3, RHOA, SPN
4.82E-01	3.25	CCL5, CSDE1, DHX9, EEF2, EIF5A, GAPDH, HNRNPU, KRT17, NCL, NPM1, PKM, RHOA, SYN
4.84E-01	4.37	ACTB, CDC42, CSDE1, DHX9, G3BP1, HNRNPU, NCL, PCBP1, PSMA6, RPLP0, TUBB
4.91E-01	2.16	ACTB, CALM3, CAMP, CCL5, CD36, CD9, CPN1, CYBB, DSG1, EEF1A1, FCER1G, FLNA, GNA
4.97E-01	3.78	ADH5, CAMP, CCL5, CD36, GSTP1, LBP, LTF, PF4V1, PRDX2, RHOA, S100A8, S100A9, VIM
5.19E-01	4.34	ADH5, CCL5, CD36, GSR, GSTP1, PRDX1, PRDX2, PRDX6, PTGS1, S100A9, SOD1
5.20E-01	7.17	ACTB, ADAM10, DSP, F11R, PECAM1, RAB27B, VCL
5.31E-01	3.37	ALDOA, ARPC5, CFL1, FLNA, FSCN1, HNRNPU, ITGB1, MSN, MYH9, PFN1, SPTBN1, TLN1, ⁻
5.34E-01	45.96	FLNA, GP1BA, GP1BB
5.34E-01	45.96	EEF1A1, HSPA8, STOM
5.34E-01	45.96	DSP, JUP, VCL
5.34E-01	45.96	B2M, HLA-A, HLA-B
5.40E-01	19.42	CD36, PRDX1, PRDX2, SOD1
5.40E-01	19.42	ALDOA, ANK1, ENO1, SPTBN1
5.54E-01	8.85	PTPRJ, RCC2, RHOA, THBS1, TLN1, VCL
5.54E-01	8.85	ADAM10, CCL5, ITGB3, MSN, RHOA, SPN
5.56E-01	4.30	ACTB, CCT2, CCT4, CCT6A, CCT7, CCT8, HNRNPA2B1, HNRNPC, HNRNPU, TCP1, XRCC5
5.57E-01	2.79	ANXA11, ANXA2, ANXA5, ANXA7, CALM3, CANX, DSC1, DSG1, FLG2, HRNR, LRP1, MMRN

5.61E-01	1.69	AACS, ACTB, ADAM10, ADH5, ANPEP, ANTXR2, ANXA11, ANXA2, ANXA5, ANXA7, ATP2A3
5.73E-01	3.72	CD36, CFL1, EEF1A1, FSCN1, HSP90AA1, ITGB1, ITGB3, MSN, PLEK, PTPRJ, RHOA, SPTBN1
5.83E-01	2.96	ATP2A3, ATP5F1B, B2M, BCAP31, CALM3, CCL5, FLNA, GNAI2, GP1BA, GP1BB, ITGB3, LTF
5.89E-01	11.87	CDC42, FLNA, ILK, ITGB3, MYADM
5.90E-01	3.71	ARL6IP5, CD36, G6PD, GSR, HSPA1B, PNPLA8, PPIA, PRDX1, PRDX2, SLC2A1, SOD1, UBQL
6.15E-01	Inf	GAPDH, S100A9
6.15E-01	Inf	S100A8, S100A9
6.26E-01	6.94	ATP2A3, CALM3, DSP, FLNA, JUP, SRI, YWHAE
6.35E-01	3.16	CD36, CD47, CDC42, FLOT1, FSCN1, G3BP1, HSP90AA1, HSPA1B, MSN, PFN1, PLEK, PTPR
6.39E-01	3.92	ACTB, CCT2, CCT4, CCT6A, CCT7, CCT8, DHX9, HNRNPA2B1, HSP90AA1, TCP1, TFRC, XRC(
6.40E-01	18.45	CDC42, MSN, RAB10, RHOA
6.56E-01	11.57	FLOT1, HSPA1B, LBP, LTF, RTN4
6.56E-01	11.57	RPS15, RPS15A, RPS23, RPS3A, RPSA
6.58E-01	2.93	ANPEP, ANXA2, ATP5F1B, CAMP, CYBB, FLNA, ITGA2B, ITGB1, ITGB3, JUP, KRT1, MYH9, N
6.65E-01	6.87	CD36, CLIC1, FLNA, HSP90AA1, MYH9, MYL12B, MYL6
6.71E-01	2.92	CAMP, CCL5, CD36, GSTP1, HNRNPU, HSPA1B, HSPA8, LBP, LTF, MSN, PF4V1, PRDX2, RH
6.82E-01	2.44	ACTB, ALDOA, CDC42, CFL1, F11R, FERMT3, FLNA, FLOT1, GP1BA, HSP90AA1, ILK, ITGB1,
7.21E-01	2.73	ACTB, CDC42, CFL1, FERMT3, FLNA, FLOT1, HSP90AA1, ILK, ITGB1, ITGB3, MYADM, MYH
7.52E-01	39.39	CD36, SLC2A1, THBS1
7.52E-01	39.39	CD226, CD47, PTPRJ
7.52E-01	39.39	YWHAB, YWHAE, YWHAZ
7.53E-01	4.14	B2M, CD226, CD36, FCN1, HLA-A, HLA-B, HSPD1, LBP, PTPRJ, RAC2, TFRC
7.53E-01	4.14	ATP2A3, CALM3, CDC42, DSP, FLNA, GNAI2, HSP90AA1, JUP, SOD1, SRI, YWHAE
7.54E-01	17.57	CD36, PRDX1, PRDX2, SOD1
7.54E-01	17.57	CD36, PRDX1, PRDX2, SOD1
7.54E-01	17.57	DSC1, DSG1, DSP, JUP
7.54E-01	8.32	CDC42, FSCN1, HSP90AA1, ITGB1, RAC2, VCL
7.54E-01	8.32	ADAM10, CCL5, CD47, ITGB3, RHOA, SPN
7.61E-01	3.25	ALDOA, ARPC5, CD47, CDC42, CFL1, F11R, FLNA, FSCN1, MYADM, PFN1, PLEK, RAC2, RH(
7.61E-01	3.25	CD36, DHX9, FLNA, FLOT1, HSPA1B, JUP, LTF, NPM1, PPIA, PRDX2, PSMA6, S100A8, S10(
7.87E-01	2.88	ATP2A3, ATP5F1B, B2M, BCAP31, CALM3, CCL5, FLNA, GNAI2, GP1BA, GP1BB, ITGB3, LTF
7.95E-01	6.65	CD36, CYBB, DHX9, LBP, PTPRJ, SPN, THBS1
8.12E-01	8.20	CD36, CD47, FCER1G, FCN1, PTPRJ, SOD1
8.16E-01	2.57	CCL5, CD36, CD47, CYBB, DHX9, F11R, GSTP1, ITGB1, KRT1, KRT16, LBP, LRP1, LYZ, PF4V1
8.58E-01	4.08	ACTB, CDC42, CSDE1, DHX9, G3BP1, HNRNPU, NCL, PCBP1, PSMA6, RPLP0, TUBB
8.79E-01	4.43	CAMP, CCL5, CD36, GSTP1, LBP, LTF, PF4V1, PRDX2, RHOA, VIM
8.81E-01	16.77	FERMT3, PLEK, RAP1B, TLN1
8.81E-01	16.77	DAZAP1, HSPA8, NCL, PRDX6
8.81E-01	16.77	CSDE1, DHX9, HNRNPU, SYNCRIP
8.82E-01	2.16	ANTXR2, ANXA7, ARL6IP5, ATP2A3, B2M, BCAP31, CANX, CD59, CDC42, CLTA, CYB5R3, C
8.93E-01	6.52	CD36, FLOT1, HSPA1B, LBP, LTF, RTN4, UBQLN1
8.98E-01	2.49	CALM3, CAMP, CCL5, CD36, CDC42, ENO1, FLOT1, HSP90AA1, ILK, ITGA6, ITGB3, LTF, NP
8.98E-01	2.49	ANXA5, CCL5, CFL1, ENO1, FLNA, GNAI2, GSTP1, HSPA1B, HSPA9, HSPD1, ITGA6, ITGB1, L
8.98E-01	2.49	CALM3, CAMP, CCL5, CD36, CDC42, ENO1, FLOT1, HSP90AA1, ILK, ITGA6, ITGB3, LTF, NP
9.17E-01	5.54	ARL6IP5, ATP2A3, BCAP31, CTSD, HSPD1, RHOA, S100A8, S100A9
9.45E-01	6.46	ALDOA, ENO1, GAPDH, LDHA, LDHB, PGK1, PKM
9.45E-01	6.46	B2M, CTSD, FCER1G, HLA-A, HLA-B, RAB10, THBS1

9.45E-01	6.46	CD47, CDC42, F11R, FLNA, PFN1, PLEK, RHOA
9.56E-01	2.83	ATP2A3, ATP5F1B, B2M, BCAP31, CALM3, CCL5, FLNA, GNAI2, GP1BA, GP1BB, ITGB3, LTI
9.60E-01	3.17	ADAM10, CCL5, CD226, CD47, DHX9, FCN1, FLNA, HSP90AA1, LBP, MATR3, RAC2, S100A
9.80E-01	10.52	ADAM10, CCL5, ITGB3, RHOA, SPN
9.84E-01	2.38	ANXA5, CCL5, CFL1, ENO1, FLNA, GNAI2, GSTP1, HSPA1B, HSPA9, HSPD1, ITGA6, ITGB1, I
9.99E-01	6.39	CD36, CYBB, DHX9, LBP, PTPRJ, SPN, THBS1
1.00E+00	2.92	ANXA11, ANXA2, ANXA5, ANXA7, CD151, CTSD, FCN1, GP1BA, HRNR, HSP90AA1, KRT1, L
1.00E+00	5.46	CCL5, CD226, DHX9, FCN1, HSP90AA1, LBP, MATR3, XRCC5
1.00E+00	5.46	ACTB, ANXA2, CFL1, HNRNPA2B1, HNRNPU, MATR3, PSMA6, VIM
1.00E+00	3.03	CALM3, G6PD, GNAI2, GNAQ, GSTP1, HSPA1B, LRP1, MYADM, NPM1, PPIA, PTPRJ, RPL11
1.00E+00	7.85	CD36, FLOT1, LBP, LTF, RTN4, UBQLN1
1.00E+00	7.85	NPM1, RPS15, RPS15A, RPS23, RPS3A, RPSA
1.00E+00	7.85	B2M, CYBB, HLA-A, HLA-B, RAB10, RAC2
1.00E+00	4.35	CALM3, FLNA, GP1BA, GP1BB, HNRNPU, ITGB3, S100A8, S100A9, SRGN, SRI
1.00E+00	34.46	FSCN1, MSN, RHOA
1.00E+00	16.04	ANXA2, ENO1, PGK1, THBS1
1.00E+00	16.04	ANXA2, GP1BA, KRT1, THBS1
1.00E+00	16.04	FLG2, HRNR, KRT1, KRT16
1.00E+00	2.91	ANXA11, ANXA2, ANXA5, ANXA7, CD151, CTSD, FCN1, GP1BA, HRNR, HSP90AA1, KRT1, L
1.00E+00	1.65	ADAM10, ANK1, ANPEP, ANXA5, ARL6IP5, ATP2A3, BCAP31, CALM3, CCL5, CD226, CD36,
1.00E+00	3.02	ACTB, CD151, CDC42, FLG2, FLNA, ILK, ITGB1, KRT10, KRT16, KRT17, KRT6A, MSN, PFN1,
1.00E+00	10.29	CD53, CD9, FLOT1, ITGB1, MYH9
1.00E+00	10.29	ADH5, GAPDH, HMBS, S100A8, S100A9
1.00E+00	10.29	ANXA2, CD9, GP1BA, KRT1, THBS1
1.00E+00	7.74	ANXA2, CD9, GP1BA, KRT1, MMRN1, THBS1
1.00E+00	2.80	ACTB, CD151, CDC42, DSP, FLG2, FLNA, ILK, ITGB1, ITGB3, KRT10, KRT16, KRT17, KRT6A,
1.00E+00	1.65	ACTB, ANXA2, ARL6IP5, ATP2A3, BCAP31, CALM3, CAMP, CCL5, CCT2, CCT4, CCT6A, CCT
1.00E+00	3.68	CAMP, CD59, FCN1, GAPDH, H2BC21, HLA-A, KRT1, KRT6A, LTF, LYZ, PF4V1, S100A9
1.00E+00	3.00	ADH5, ANPEP, ATP2A3, CALM3, CDC42, CYB5R3, DSP, FLNA, HSP90AA1, JUP, PTGS1, PTP
1.00E+00	2.51	BCAP31, CD36, CLTA, FLNA, HSP90AA1, HSPA8, HSPA9, HSPD1, JUP, M6PR, NPM1, RAB1I
1.00E+00	4.74	CD36, FCN1, FLOT1, HSPA1B, HSPD1, LBP, LTF, RTN4, UBQLN1
1.00E+00	3.27	ATP2A3, BCAP31, CALM3, CCL5, FLNA, GP1BA, GP1BB, ITGB3, S100A8, S100A9, SOD1, SR
1.00E+00	7.63	FLNA, FSCN1, ILK, MYH9, MYL12B, TPM4
1.00E+00	4.71	CCL5, CD226, DHX9, FCN1, HSP90AA1, HSPD1, LBP, MATR3, XRCC5
1.00E+00	4.71	NPM1, RPL11, RPL8, RPLP0, RPS15, RPS15A, RPS23, RPS3A, RPSA
1.00E+00	15.37	CD36, PRDX1, PRDX2, SOD1
1.00E+00	3.65	ACTB, B2M, CCL5, CD47, F11R, FLOT1, GP1BA, HLA-A, HSPD1, MMRN1, RHOA, TFRC
1.00E+00	10.06	ACTB, ADAM10, CDC42, DSP, VCL
1.00E+00	10.06	ANXA2, CD9, GP1BA, KRT1, THBS1
1.00E+00	3.64	ATP2A3, BCAP31, CALM3, CCL5, FLNA, GP1BA, GP1BB, ITGB3, S100A8, S100A9, SRI, YWH
1.00E+00	2.30	ACTB, CALM3, CAMP, CCL5, CD36, FLOT1, GNAQ, GSTP1, HSP90AA1, ILK, ITGB3, LRP1, LT
1.00E+00	3.42	ANXA2, ARL6IP5, ATP2A3, BCAP31, CTSD, ENO1, HSPA1B, HSPD1, MYH9, RHOA, S100A8,
1.00E+00	2.09	ACTB, ANXA2, ARL6IP5, ATP2A3, ATP5F1B, B2M, CALM3, CCL5, CD36, CD47, CLIC1, CYBB
1.00E+00	3.24	B2M, CALM3, CCL5, FLNA, G6PD, GNAI2, ITGB1, ITGB3, RHOA, SRI, STOM, UBQLN1, YWH
1.00E+00	2.43	CSDE1, CTSD, FLNA, FSCN1, G3BP1, HNRNPU, HSPA1B, ITGB1, LRBA, MSN, NPM1, RAB1B
1.00E+00	7.53	EEF1A1, FLOT1, MYADM, MYH9, SLC2A1, SPTBN1
1.00E+00	2.34	ATP2A3, ATP5F1B, B2M, BCAP31, CALM3, CCL5, FLG2, FLNA, GNAI2, GP1BA, GP1BB, GST

1.00E+00	2.43	ANXA5, CCL5, CFL1, ENO1, FLNA, GNAI2, GSTP1, HSPA1B, HSPA9, HSPD1, ITGA6, ITGB1, L
1.00E+00	9.85	ITGA2B, ITGA6, ITGB1, ITGB3, MMRN1
1.00E+00	2.85	ACTB, ADAM10, ANK1, ARL6IP5, CALM3, F11R, FLNA, FLOT1, HSP90AA1, ITGB1, JUP, LRP
1.00E+00	2.53	ADAM10, ANPEP, ANXA2, ATP5F1B, CAMP, CYBB, FLNA, ITGA2B, ITGB1, ITGB3, JUP, KRT1
1.00E+00	7.43	ANXA11, ANXA2, ANXA7, CALM3, S100A8, S100A9
1.00E+00	4.19	CAMP, CCL5, CD36, GSTP1, LBP, LTF, PF4V1, PRDX2, RHOA, VIM
1.00E+00	2.67	ANPEP, ANXA2, ATP5F1B, CAMP, CYBB, FLNA, ITGA2B, ITGB1, ITGB3, JUP, KRT1, LRP1, M
1.00E+00	1.66	AACS, ACTB, ADAM10, ADH5, ALDOA, ANPEP, ANXA2, ANXA7, ARL6IP5, ATIC, ATP2A3, A
1.00E+00	30.63	CD36, S100A8, S100A9
1.00E+00	30.63	CD47, DSP, JUP
1.00E+00	14.76	CD36, PRDX1, PRDX2, SOD1
1.00E+00	14.76	CDC42, FSCN1, HSP90AA1, RAC2
1.00E+00	14.76	FLOT1, LBP, LTF, RTN4
1.00E+00	14.76	CAMP, DCD, GAPDH, KRT6A
1.00E+00	14.76	BCAP31, CANX, HLA-A, HLA-B
1.00E+00	14.76	BCAP31, CANX, HLA-A, HLA-B
1.00E+00	4.17	ARL6IP5, ATP2A3, BCAP31, CTSD, HSPD1, LTF, RHOA, S100A8, S100A9, THBS1
1.00E+00	2.18	ADAM10, ANXA2, ATP5F1B, B2M, CALM3, CLIC1, CLTA, CYBB, FCER1G, FLNA, FLOT1, GN
1.00E+00	3.36	B2M, CD226, CD36, CD47, CD59, FCN1, HLA-A, HLA-B, HSPD1, LBP, PTPRJ, RAC2, TFRC
1.00E+00	7.23	ALDOA, ENO1, GAPDH, LDHA, PGK1, PKM
1.00E+00	7.23	ANXA2, FLNA, G6PD, GP1BA, GP1BB, LTF
1.00E+00	2.81	ANXA2, CAVIN2, CD9, CDC42, FLOT1, MYH9, OSBPL2, RAB10, RAB1B, RAB33B, RTN3, RTN
1.00E+00	9.44	ANXA2, CD47, DSP, JUP, RAB10
1.00E+00	14.19	B2M, FCER1G, HLA-A, HLA-B
1.00E+00	14.19	FERMT3, FSCN1, TPM4, VCL
1.00E+00	7.14	ALDOA, ENO1, GAPDH, LDHA, PGK1, PKM
1.00E+00	5.87	CAMP, CD36, H2BC21, HLA-A, KRT6A, LBP, LYZ
1.00E+00	4.08	ADAM10, CCL5, FCER1G, LBP, PF4V1, PPIA, RAC2, S100A8, S100A9, THBS1
1.00E+00	9.25	ILK, ITGB1, ITGB3, RCC2, THBS1
1.00E+00	9.25	CYBB, PRDX1, PRDX2, RAC2, SOD1
1.00E+00	9.25	GSTP1, PRDX1, PRDX2, PRDX6, PTGS1
1.00E+00	2.15	ACTB, ADAM10, CALM3, CANX, CDC42, CLTA, EEF2, FLOT1, GNAI2, GNAQ, HSPA8, ITGB1,
1.00E+00	5.01	BCAP31, FLNA, HSP90AA1, JUP, RAC2, STOM, UBE2L3, YWHAE
1.00E+00	7.05	PTPRJ, RCC2, RHOA, THBS1, TLN1, VCL
1.00E+00	4.46	B2M, CD59, CD9, CLTA, HLA-A, HLA-B, HSPA8, M6PR, TFRC
1.00E+00	3.49	ANXA2, BCAP31, CD36, DHX9, FLNA, HSP90AA1, JUP, MSN, RAC2, STOM, UBE2L3, YWHA
1.00E+00	3.74	ARL6IP5, CD36, G6PD, GSR, HSPA1B, PNPLA8, PPIA, PRDX1, PRDX2, SOD1, UBQLN1
1.00E+00	4.04	ALDOA, ATP5F1B, ENO1, GAPDH, HSPA1B, HSPA8, LDHA, PGK1, PKM, RHOA
1.00E+00	13.66	FLG2, HRNR, KRT1, KRT16
1.00E+00	13.66	ITGA2B, ITGA6, ITGB1, ITGB3
1.00E+00	13.66	DHX9, RPL11, RPL8, RPS23
1.00E+00	1.62	ACTB, ANXA2, ARL6IP5, ATP2A3, BCAP31, CALM3, CAMP, CCL5, CCT2, CCT4, CCT6A, CCT
1.00E+00	2.60	ACTB, ARL6IP5, B2M, CALM3, CCL5, CLIC1, CYBB, FLNA, G6PD, GNAI2, ITGB1, ITGB3, RHC
1.00E+00	183.04	PECAM1, VCL
1.00E+00	2.98	ADAM10, CDC42, ENO1, G6PD, HSP90AA1, HSPA1B, ITGB1, KRT17, NPM1, PTPRJ, RHOA,
1.00E+00	3.11	ANXA2, ATP2A3, CALM3, CCL5, FLNA, G6PD, GNAI2, GP1BA, GP1BB, ITGB3, RHOA, SRI, U
1.00E+00	3.11	CALM3, GNAI2, GNAQ, GSTP1, HNRNPU, LRP1, MYADM, NPM1, PLEK, PPIA, PTPRJ, YWH

1.00E+00	2.86	ACTB, B2M, CCL5, CD151, CD47, FCER1G, HLA-A, HSPD1, MSN, MYH9, PRDX2, RAC2, RHO
1.00E+00	9.07	DCD, GAPDH, LTF, S100A8, S100A9
1.00E+00	9.07	ITGA2B, ITGB1, ITGB3, RPSA, THBS1
1.00E+00	2.31	ANXA2, ARL6IP5, ATP2A3, BCAP31, CALM3, CCL5, CTSD, F11R, GAPDH, GNAI2, GNAQ, HS
1.00E+00	3.10	CALM3, GNAI2, GNAQ, GSTP1, HNRNPU, LRP1, MYADM, NPM1, PLEK, PPIA, PTPRJ, YWHA
1.00E+00	4.39	ALDOA, CCT2, CCT4, CCT7, CCT8, CD9, MYH9, TCP1, VDAC2
1.00E+00	6.88	ATP2A3, CD36, LRP1, SLC2A1, SLC2A3, TFRC
1.00E+00	6.88	ATP2A3, CD36, LRP1, SLC2A1, SLC2A3, TFRC
1.00E+00	4.91	CD47, CDC42, F11R, FLNA, FSCN1, PFN1, PLEK, RHOA
1.00E+00	3.98	ATP5F1B, CLIC1, DHX9, HNRNPC, HNRNPU, ILK, LTF, RPS15, SYNCRIP, TPM4
1.00E+00	8.90	ARL6IP5, G6PD, GSR, GSTP1, SOD1
1.00E+00	8.90	GP1BA, ITGB1, RHOA, SELPLG, SPN
1.00E+00	8.90	GSTP1, PRDX1, PRDX2, PRDX6, PTGS1
1.00E+00	2.51	ANPEP, ANXA2, ATP5F1B, CAMP, CYBB, FLNA, ITGA2B, ITGB1, ITGB3, JUP, KRT1, LRP1, M
1.00E+00	3.43	CALM3, CD36, FLNA, GP1BA, GP1BB, HNRNPU, ITGB3, S100A8, S100A9, SRGN, SRI, YWHA
1.00E+00	3.96	ALDOA, ATP5F1B, ENO1, GAPDH, HSPA1B, HSPA8, LDHA, PGK1, PKM, RHOA
1.00E+00	4.34	ANXA11, ANXA2, CDC42, GNAI2, HNRNPU, RCC2, RHOA, SLC2A1, TTLL12
1.00E+00	2.94	ATP2A3, ATP5F1B, BCAP31, CALM3, CCL5, FLNA, GP1BA, GP1BB, ITGB3, S100A8, S100A9
1.00E+00	3.66	AACS, BCAP31, FLNA, HSP90AA1, JUP, PPIA, RAC2, SRI, STOM, UBE2L3, YWHAE
1.00E+00	5.62	CD36, FLOT1, HSPD1, LBP, LTF, RTN4, UBQLN1
1.00E+00	4.32	F11R, FLOT1, PTPRJ, RAP1B, RCC2, RHOA, THBS1, TLN1, VCL
1.00E+00	3.93	ALDOA, ATP5F1B, ENO1, GAPDH, HSPA1B, HSPA8, LDHA, PGK1, PKM, RHOA
1.00E+00	25.06	FSCN1, MSN, RHOA
1.00E+00	1.61	ACTB, ANXA2, B2M, BCAP31, CALM3, CAMP, CCL5, CCT2, CCT4, CCT6A, CCT7, CCT8, CD3
1.00E+00	2.71	ARL6IP5, BCAP31, CCL5, CD36, CTSD, EIF5A, HSPD1, INPP5D, ITGA6, ITGB1, LRP1, RHOA,
1.00E+00	4.30	ARL6IP5, ATP2A3, BCAP31, CTSD, HSPD1, RHOA, S100A8, S100A9, THBS1
1.00E+00	2.92	CD226, CD36, CD47, FCER1G, FCN1, FLOT1, HLA-A, HSPA1B, HSPD1, INPP5D, LBP, LTF, PT
1.00E+00	3.91	ATP2A3, CALM3, CDC42, DSP, FLNA, HSP90AA1, JUP, SOD1, SRI, YWHAE
1.00E+00	12.72	CD226, CD47, FCER1G, PTPRJ
1.00E+00	12.72	GP1BA, ITGB1, SELPLG, SPN
1.00E+00	4.79	CD47, CDC42, F11R, FLNA, FSCN1, PFN1, PLEK, RHOA
1.00E+00	8.57	GSR, PCYOX1, PGK1, QSOX1, TMX2
1.00E+00	1.89	ADH5, ANPEP, ANXA2, ANXA7, BCAP31, CANX, CD36, CPN1, CSDE1, CTSD, DHX9, EEF1A1
1.00E+00	1.99	BCAP31, CALM3, CANX, CD151, CD226, CD36, CD47, CD53, CD9, CD99, CYBB, FCER1G, FL
1.00E+00	12.30	CD36, CD9, FCER1G, ITGB1
1.00E+00	12.30	CD36, GNAI2, GSTP1, SOD1
1.00E+00	2.15	ANPEP, ANXA2, ATP5F1B, CAMP, CD36, CD53, CD9, CYBB, FLNA, FLOT1, GNG5, ITGA2B, I
1.00E+00	4.21	ANXA2, B2M, CALM3, CD151, CD36, CDC42, FLOT1, ITGB3, LRP1
1.00E+00	2.59	CCL5, CD226, CD47, DHX9, FCN1, GSTP1, HLA-A, HLA-B, HSP90AA1, KRT1, LBP, MATR3, P
1.00E+00	3.34	ALDOA, CALM3, DSP, ENO1, FLNA, HSP90AA1, JUP, MYL6, RHOA, SOD1, SRI, TPM4
1.00E+00	6.47	ALDOA, ENO1, GAPDH, LDHA, PGK1, PKM
1.00E+00	1.57	ACTB, ADAM10, ALDOA, ANXA11, ANXA2, ANXA7, ARPC5, ATP2A3, ATP5F1B, CALM3, CA
1.00E+00	8.26	CSDE1, DHX9, HNRNPC, HNRNPU, SYNCRIP
1.00E+00	8.26	CALM3, DSP, FLNA, JUP, SRI
1.00E+00	8.26	CALM3, CD36, DSP, FLNA, JUP
1.00E+00	1.95	BCAP31, CALM3, CANX, CD151, CD226, CD36, CD47, CD53, CD59, CD9, CD99, CYBB, FCEI
1.00E+00	11.90	DAZAP1, HSPA8, NCL, PRDX6

1.00E+00	3.11	ANXA2, B2M, CDC42, FLNA, G6PD, GP1BA, GP1BB, HSPA1B, HSPA9, INPP5D, LTF, MYH9,
1.00E+00	3.29	ATP2A3, BCAP31, CALM3, CCL5, FLNA, GP1BA, GP1BB, ITGB3, S100A8, S100A9, SRI, YWH
1.00E+00	5.30	CCL5, EEF1A1, HSPA8, LTF, PCBP1, PPIA, STOM
1.00E+00	2.96	ANPEP, ANXA2, CLTA, CTSD, EEF1A1, FLOT1, GNAQ, HSPA8, LRBA, LRP1, M6PR, RAP1B, S
1.00E+00	2.08	ACTB, CAMP, CD36, CD53, CDC42, CFL1, CLIC1, CYBB, F11R, FLNA, FLOT1, HNRNPU, HSPA
1.00E+00	4.61	ATP2A3, CALM3, DSP, FLNA, JUP, SOD1, SRI, YWHAE
1.00E+00	2.84	ATP2A3, ATP5F1B, BCAP31, CALM3, CCL5, FLNA, GP1BA, GP1BB, ITGB3, S100A8, S100A9
1.00E+00	2.84	ANXA2, B2M, CDC42, F11R, FLNA, ILK, ITGB1, ITGB3, LTF, MYADM, RCC2, RTN4, VCL, XRC
1.00E+00	8.12	CD47, CDC42, PFN1, PLEK, RHOA
1.00E+00	8.12	DHX9, FLNA, HSP90AA1, JUP, YWHAE
1.00E+00	6.33	CALM3, GNAI2, PPIA, PTBP1, YWHAB, YWHAE
1.00E+00	6.33	ANXA2, CD9, GP1BA, KRT1, MMRN1, THBS1
1.00E+00	6.33	ANXA2, CAVIN2, GSDMA, HSPA8, THBS1, TLN1
1.00E+00	3.49	CCL5, CD226, CD47, DHX9, FCN1, HSP90AA1, LBP, MATR3, S100A8, S100A9, XRCC5
1.00E+00	2.72	CCL5, CSDE1, DHX9, EEF2, EIF5A, GAPDH, HNRNPU, KRT17, NCL, NPM1, PKM, RHOA, SYN
1.00E+00	3.74	CAMP, CCL5, CD36, GSTP1, LBP, LTF, PF4V1, PRDX2, RHOA, VIM
1.00E+00	4.55	ANXA2, FCN1, GNAI2, GNAQ, GNG5, JUP, OSBPL2, RHOA
1.00E+00	11.53	B2M, CTSD, FCER1G, THBS1
1.00E+00	11.53	ALDOA, ANK1, ENO1, SPTBN1
1.00E+00	7.97	CCL5, CD36, LBP, LTF, PRDX2
1.00E+00	7.97	CD36, CD47, HLA-B, HSPD1, THBS1
1.00E+00	7.97	CD36, CD47, HLA-B, HSPD1, THBS1
1.00E+00	21.20	ENO1, PGK1, PKM
1.00E+00	21.20	CCL5, F11R, MMRN1
1.00E+00	21.20	ENO1, PGK1, PKM
1.00E+00	21.20	EEF1A1, HSP90AA1, HSPA8
1.00E+00	21.20	ENO1, PGK1, PKM
1.00E+00	5.17	CALM3, GNAI2, PLEK, PPIA, PTBP1, YWHAB, YWHAE
1.00E+00	4.05	ALDOA, ATP5F1B, ENO1, GAPDH, HSPA1B, HSPA8, LDHA, PGK1, PKM
1.00E+00	2.03	ACTB, ADAM10, ANXA2, B2M, CAMP, CFL1, CYBB, F11R, G6PD, HLA-B, HSPA1B, HSPA9, II
1.00E+00	4.50	CALM3, DSP, ENO1, HSP90AA1, JUP, RHOA, SOD1, SRI
1.00E+00	4.50	ARL6IP5, ATP2A3, BCAP31, CTSD, HSPD1, RHOA, S100A8, S100A9
1.00E+00	7.84	CD53, CD9, FLOT1, ITGB1, MYH9
1.00E+00	7.84	B2M, CTSD, FCER1G, HLA-A, HLA-B
1.00E+00	7.84	CD53, CD9, FLOT1, ITGB1, MYH9
1.00E+00	2.34	ANXA2, CALM3, CAVIN2, CD36, CD9, CDC42, FLOT1, HSP90AA1, HSPA8, HSPA9, MYADM,
1.00E+00	11.18	CCL5, CD9, F11R, MMRN1
1.00E+00	11.18	CD151, FLNA, ITGB1, RHOA
1.00E+00	11.18	CCT2, HSPA1B, HSPA8, HSPA9
1.00E+00	11.18	CD36, CD9, FCER1G, ITGB1
1.00E+00	11.18	CD151, FLNA, ITGB1, RHOA
1.00E+00	11.18	CDC42, FSCN1, HSP90AA1, RAC2
1.00E+00	11.18	CAMP, HSPD1, LBP, LTF
1.00E+00	4.47	CSDE1, DHX9, HNRNPC, HNRNPU, NPM1, SYNCRIP, TNKS1BP1, VIM
1.00E+00	91.52	B2M, G6PD
1.00E+00	91.52	RTN3, RTN4
1.00E+00	91.52	FLG2, HRNR

1.00E+00	91.52	GAPDH, SYNCRIP
1.00E+00	91.52	DHX9, G3BP1
1.00E+00	91.52	S100A8, S100A9
1.00E+00	91.52	HSP90AA1, RHOA
1.00E+00	91.52	ITGB3, THBS1
1.00E+00	91.52	CD36, CD47
1.00E+00	5.09	BCAP31, EIF5A, RPL11, S100A8, S100A9, SOD1, THBS1
1.00E+00	3.03	ANPEP, ANXA2, CLTA, CTSD, EEF1A1, FLOT1, GNAQ, HSPA8, LRBA, LRP1, M6PR, RAP1B,
1.00E+00	3.03	ANPEP, ANXA2, CLTA, CTSD, EEF1A1, FLOT1, GNAQ, HSPA8, LRBA, LRP1, M6PR, RAP1B,
1.00E+00	4.44	RPL11, RPL8, RPLP0, RPS15, RPS15A, RPS23, RPS3A, RPSA
1.00E+00	9.83	AACS, ACTB, ADAM10, ADH5, ALDOA, ANK1, ANPEP, ANTXR2, ANXA11, ANXA2, ANXA5,
1.00E+00	3.65	ALDOA, ATP5F1B, ENO1, GAPDH, HSPA1B, HSPA8, LDHA, PGK1, PKM, RHOA
1.00E+00	1.56	AACS, ADAM10, ANK1, ANPEP, ANXA5, ARL6IP5, ATP2A3, BCAP31, CALM3, CCL5, CD226,
1.00E+00	2.87	ALDOA, ATIC, ATP5F1B, ENO1, G6PD, GAPDH, HINT1, HSPA1B, HSPA8, LDHA, PGK1, PKM
1.00E+00	10.85	CD151, FLNA, ITGB1, RHOA
1.00E+00	2.65	CALM3, CCL5, CCT2, CCT4, CDC42, HNRNPA2B1, HSP90AA1, ITGB3, LTF, NPM1, PPIA,
1.00E+00	2.37	CALM3, CAMP, CCL5, CD36, CDC42, FLOT1, HSP90AA1, ILK, ITGA6, ITGB3, LTF, NPM1,
1.00E+00	3.16	ANK1, ANXA2, CALM3, CCL5, GNAI2, MYH9, PLEK, RAB10, RAB27B, RAB33B, RAC2,
1.00E+00	19.69	ITGB1, ITGB3, THBS1
1.00E+00	19.69	B2M, HLA-A, HLA-B
1.00E+00	19.69	CCL5, FERMT3, ITGB1
1.00E+00	19.69	CD36, SLC2A1, THBS1
1.00E+00	19.69	CDC42, MSN, RHOA
1.00E+00	19.69	FLNA, GP1BA, GP1BB
1.00E+00	19.69	DSP, JUP, VCL
1.00E+00	2.65	ARL6IP5, ATP2A3, BCAP31, CALM3, CCL5, CTSD, F11R, GNAQ, HSPD1, ITGA6, ITGB1,
1.00E+00	2.75	ARL6IP5, BCAP31, CCL5, CTSD, EIF5A, HSPD1, INPP5D, ITGA6, ITGB1, RHOA, RPL11,
1.00E+00	1.85	ADH5, ALDOA, ANPEP, ANXA2, BCAP31, CANX, CPN1, CSDE1, CTSD, DHX9, EEF1A1, ENO1
1.00E+00	5.92	ALDOA, ENO1, GAPDH, LDHA, PGK1, PKM
1.00E+00	5.92	CDC42, FLNA, ILK, ITGB3, MYADM, RCC2
1.00E+00	7.46	DCD, GAPDH, LTF, S100A8, S100A9
1.00E+00	10.54	HLA-A, HLA-B, LBP, PTPRJ
1.00E+00	10.54	CYBB, LBP, PRDX2, RAC2
1.00E+00	3.90	BCAP31, CD36, FLNA, HSP90AA1, JUP, RAC2, STOM, UBE2L3, YWHAE
1.00E+00	4.93	F11R, MSN, MYADM, PECAM1, RAP1B, RHOA, VCL
1.00E+00	1.55	AACS, ADAM10, ANK1, ANPEP, ANXA2, ANXA5, ARL6IP5, ATP2A3, BCAP31, CALM3, CCL5
1.00E+00	7.34	CSDE1, DHX9, HNRNPU, SYNCRIP, TNKS1BP1
1.00E+00	7.34	CD53, CD9, FLOT1, ITGB1, MYH9
1.00E+00	2.62	ACTB, ANXA2, B2M, CDC42, FCER1G, HLA-B, INPP5D, ITGB1, LTF, MYH9, PRDX2, PTPRJ,
1.00E+00	5.80	ALDOA, ENO1, GAPDH, LDHA, PGK1, PKM
1.00E+00	5.80	ANXA2, BCAP31, CYB5R3, GAPDH, OSBPL2, RAP1B
1.00E+00	3.30	ACTB, ADAM10, CANX, FLOT1, HSPA8, ITGB1, ITGB3, PFN1, RHOA, SPTBN1, YWHAZ
1.00E+00	2.53	ACTB, CAMP, CD36, CYBB, FLNA, GNAI2, ITGB3, JUP, LRP1, NCL, PKM, RAB10, RAP1B,
1.00E+00	1.92	ANXA2, CCL5, CD47, CD59, CD9, ENO1, G3BP1, GNAI2, GP1BA, GSTP1, HLA-A, HLA-B,
1.00E+00	2.71	ACTB, CCT2, CCT4, CCT6A, CCT7, CCT8, DHX9, HNRNPA2B1, HNRNPC, HNRNPU,
1.00E+00	3.10	ACTB, B2M, CCL5, CD47, HLA-A, HSPD1, PRDX2, RAC2, RHOA, SOD1, SPN, TFRC
1.00E+00	2.03	ANXA11, CALM3, CCT2, CCT4, CCT6A, CCT7, CCT8, CDC42, CLTA, DHX9, FLOT1, G6PD,

1.00E+00	10.24	B2M, CTSD, FCER1G, HLA-A
1.00E+00	10.24	ITGB1, ITGB3, RCC2, THBS1
1.00E+00	10.24	DAZAP1, HSPA8, NCL, PRDX6
1.00E+00	18.37	ANXA2, ENO1, THBS1
1.00E+00	18.37	CSDE1, DHX9, PTBP1
1.00E+00	18.37	FLOT1, ITGB1, ITGB3
1.00E+00	18.37	CDC42, HNRNPU, RCC2
1.00E+00	18.37	ENO1, PGK1, PKM
1.00E+00	18.37	RAB10, RTN3, RTN4
1.00E+00	18.37	CD36, SLC2A1, THBS1
1.00E+00	18.37	CANX, HSPD1, LRP1
1.00E+00	5.74	CD36, CD47, FCER1G, FCN1, PTPRJ, SOD1
1.00E+00	3.82	ANK1, ANXA2, DSP, FLOT1, HSP90AA1, LRP1, MSN, SLC2A1, TFRC
1.00E+00	5.68	ALDOA, ENO1, GAPDH, LDHA, PGK1, PKM
1.00E+00	5.68	ALDOA, ENO1, GAPDH, LDHA, PGK1, PKM
1.00E+00	3.50	ARPC5, CD47, CDC42, F11R, FLNA, MYADM, PFN1, PLEK, RHOA, SPTBN1
1.00E+00	4.79	B2M, LTF, S100A8, S100A9, SOD1, SRI, TFRC
1.00E+00	7.11	CSDE1, DHX9, HNRNPC, HNRNPU, SYNCRIP
1.00E+00	7.11	CSDE1, DHX9, HNRNPC, HNRNPU, SYNCRIP
1.00E+00	7.11	FSCN1, ILK, MYH9, MYL12B, TPM4
1.00E+00	7.11	FSCN1, ILK, MYH9, MYL12B, TPM4
1.00E+00	2.31	ACTB, B2M, CCL5, CD151, CD47, FCER1G, HLA-A, HSPD1, INPP5D, ITGB1, MSN, MYH9, PR
1.00E+00	9.97	GSR, PRDX1, PRDX2, PRDX6
1.00E+00	1.95	ACTB, ADAM10, CALM3, CAMP, CCL5, CD36, CDC42, FLOT1, GNAQ, GSTP1, HSP90AA1, IL
1.00E+00	2.67	ARL6IP5, BCAP31, CCL5, CTSD, EIF5A, HSPD1, INPP5D, ITGA6, ITGB1, RHOA, RPL11, S100
1.00E+00	5.62	DHX9, FLOT1, G3BP1, GAPDH, HSP90AA1, HSPD1
1.00E+00	5.62	DHX9, FLOT1, G3BP1, GAPDH, HSP90AA1, HSPD1
1.00E+00	2.36	ACTB, CALM3, CCL5, CDC42, EEF1A1, GNAQ, GSTP1, HNRNPU, HSP90AA1, ITGB3, LTF, NP
1.00E+00	2.90	ANXA2, CD9, GP1BA, GSTP1, HLA-A, HLA-B, KRT1, LTF, MMRN1, PRDX2, SOD1, THBS1, TT
1.00E+00	4.17	CSDE1, DHX9, HNRNPC, HNRNPU, NPM1, SYNCRIP, TNKS1BP1, VIM
1.00E+00	2.77	ATP5F1B, CDC42, ILK, ITGB1, ITGB3, JUP, KRT16, KRT2, MYH9, PFN1, RCC2, RHOA, RTN4,
1.00E+00	1.81	ACTB, ANXA2, B2M, CDC42, CFL1, F11R, FERMT3, FLNA, FLOT1, G6PD, GP1BA, GP1BB, HI
1.00E+00	7.01	CD36, GNAI2, GSTP1, SOD1, THBS1
1.00E+00	3.75	ENO1, GNAI2, GSTP1, HSPA1B, ITGA6, PPIA, PRDX2, THBS1, VDAC2
1.00E+00	17.22	RPS15, RPSA, XRCC5
1.00E+00	17.22	B2M, HLA-A, HLA-B
1.00E+00	17.22	LBP, S100A8, S100A9
1.00E+00	17.22	GNAI2, GSTP1, SOD1
1.00E+00	17.22	ENO1, PGK1, PKM
1.00E+00	17.22	FSCN1, MSN, RHOA
1.00E+00	17.22	CDC42, PFN1, RHOA
1.00E+00	17.22	CD36, SLC2A1, THBS1
1.00E+00	17.22	EEF1A1, EEF2, EIF5A
1.00E+00	3.03	ARPC5, CD47, CDC42, F11R, FLNA, HSPA1B, HSPA8, MYADM, PFN1, PLEK, RHOA, SPTBN1
1.00E+00	2.56	ACTB, CCT2, CCT4, CCT6A, CCT7, CCT8, DHX9, G3BP1, HNRNPA2B1, HNRNPC, HNRNPU, H
1.00E+00	9.70	CDC42, FSCN1, HSP90AA1, RAC2
1.00E+00	9.70	LRP1, S100A8, S100A9, VIM

1.00E+00	9.70	ENO1, GALE, PGK1, PKM
1.00E+00	1.56	ACTB, ADAM10, ANXA2, ANXA7, B2M, CCL5, CD226, CD36, CD47, CSDE1, CYBB, DAZAP1,
1.00E+00	61.01	CD226, PTPRJ
1.00E+00	61.01	MSN, RAC2
1.00E+00	61.01	SLC2A1, SLC2A3
1.00E+00	61.01	CD47, PTPRJ
1.00E+00	61.01	CALM3, YWHAE
1.00E+00	61.01	CALM3, YWHAE
1.00E+00	61.01	DHX9, HNRNPU
1.00E+00	61.01	DHX9, HNRNPU
1.00E+00	61.01	CSDE1, SYNCRIP
1.00E+00	61.01	LDHA, LDHB
1.00E+00	61.01	ITGB1, ITGB3
1.00E+00	61.01	ITGA6, ITGB3
1.00E+00	61.01	HSP90AA1, RHOA
1.00E+00	61.01	DSP, JUP
1.00E+00	61.01	HSPA1B, HSPA8
1.00E+00	3.42	ATP2A3, BCAP31, CALM3, CCL5, FLNA, GP1BA, GP1BB, ITGB3, SRI, YWHAE
1.00E+00	4.10	CSDE1, DHX9, HNRNPC, HNRNPU, NPM1, SYNCRIP, TNKS1BP1, VIM
1.00E+00	2.19	ACTB, ADAM10, CAMP, CCL5, CD47, CDC42, DHX9, GNAI2, GNG5, HLA-A, HNRNPU, ILK, I
1.00E+00	2.33	ANXA2, ANXA5, F11R, GAPDH, GNAI2, GNAQ, GSTP1, HNRNPU, LRP1, LTF, NPM1, PPIA, F
1.00E+00	6.80	ANK1, ATP2A3, SRI, THBS1, TMEM109
1.00E+00	3.69	ADAM10, CAMP, CCL5, CD226, FCER1G, HSPD1, LBP, RAC2, THBS1
1.00E+00	2.39	ADH5, CYB5R3, CYBB, G6PD, GAPDH, GSR, GSTP1, LDHA, LDHB, PCYOX1, PGK1, PRDX1, P
1.00E+00	9.45	CD226, HLA-A, HSPD1, PRDX2
1.00E+00	9.45	CYBB, GNAI2, GSTP1, SOD1
1.00E+00	1.52	AACS, ACTB, ADAM10, ADH5, ALDOA, ANPEP, ANXA2, ARL6IP5, ATIC, ATP2A3, ATP5F1B,
1.00E+00	5.40	CD36, DHX9, FLNA, HSP90AA1, JUP, YWHAE
1.00E+00	2.71	AACS, BCAP31, CCL5, CD36, FLNA, HSP90AA1, HSPA8, JUP, PPIA, RAC2, SRI, STOM, UBE2I
1.00E+00	1.52	AACS, ACTB, ADAM10, ADH5, ALDOA, ANPEP, ANXA2, ANXA7, ARL6IP5, ATIC, ATP5F1B, E
1.00E+00	16.21	ADAM10, CCT8, RTN4
1.00E+00	16.21	FLNA, GP1BA, GP1BB
1.00E+00	2.52	ACTB, ARL6IP5, CALM3, CLIC1, CYBB, FLNA, G6PD, ITGB1, ITGB3, SRI, STOM, THBS1, TME
1.00E+00	6.70	CD226, HLA-A, HLA-B, PRDX1, TUBB
1.00E+00	3.15	CALM3, CPN1, CYBB, DSG1, HNRNPU, HSPA1B, HSPA8, RHOA, THBS1, UBE2L3, YWHAH
1.00E+00	2.44	ACTB, ADAM10, ANK1, ARL6IP5, CALM3, F11R, FLNA, FLOT1, HSP90AA1, ITGB1, JUP, LRP
1.00E+00	1.54	AACS, ACTB, ADAM10, ADH5, ALDOA, ANPEP, ANXA2, ANXA7, ARL6IP5, ATIC, ATP2A3, A
1.00E+00	9.22	CALM3, SRI, UBQLN1, YWHAE
1.00E+00	9.22	GSR, PGK1, QSOX1, TMX2
1.00E+00	2.13	ACTB, ARPC5, ATIC, B2M, CALM3, CDC42, G6PD, GSTP1, HSP90AA1, ITGB1, LRP1, RHOA,
1.00E+00	4.01	ARL6IP5, ATP2A3, BCAP31, CTSD, HSPD1, RHOA, S100A8, S100A9
1.00E+00	2.69	AACS, ADH5, ALDOA, ATP5F1B, CYBB, ENO1, G6PD, GAPDH, GSR, LDHA, PGK1, PKM, RHC
1.00E+00	5.30	CDC42, F11R, FLNA, MYH9, PLEK, RHOA
1.00E+00	2.58	CANX, CCT2, CCT4, CCT7, CCT8, CDC42, CYBB, FLNA, G3BP1, GNAI2, HSP90AA1, SLC2A3,
1.00E+00	8.99	ARL6IP5, PPIA, SOD1, UBQLN1
1.00E+00	2.93	CD226, CD47, CD59, FCER1G, FCN1, HLA-A, HSP90AA1, INPP5D, KRT1, MATR3, PTPRJ, XR
1.00E+00	15.31	BCAP31, CANX, TMX2

1.00E+00	15.31	ADAM10, BCAP31, CYBB
1.00E+00	15.31	CSDE1, DAZAP1, DHX9
1.00E+00	3.09	ANXA2, ATP2A3, CALM3, FLNA, G6PD, GP1BA, GP1BB, ITGB3, SRI, UBQLN1, YWHAE
1.00E+00	2.34	ANXA2, ARL6IP5, ATP2A3, BCAP31, CTSD, ENO1, GAPDH, HSPA1B, HSPD1, LRP1, LTF, MY
1.00E+00	4.43	ALDOA, F11R, MSN, MYH9, MYL12B, RAC2, RHOA
1.00E+00	2.77	ALDOA, CALM3, DSP, ENO1, FLNA, G6PD, HSP90AA1, JUP, MYL6, RHOA, SOD1, SRI, TPM4
1.00E+00	6.42	CD226, HLA-A, HLA-B, PRDX1, TUBB
1.00E+00	6.42	CD36, FLNA, ITGB1, PRDX2, THBS1
1.00E+00	2.33	CALM3, CAMP, CCL5, CD36, FLOT1, HSP90AA1, ILK, ITGB3, LTF, NPM1, PECAM1, PPIA, PT
1.00E+00	8.78	DCD, GAPDH, LTF, LYZ
1.00E+00	8.78	ENO1, GALE, PGK1, PKM
1.00E+00	5.15	B2M, CD226, HLA-A, HLA-B, HSPD1, TFRC
1.00E+00	5.15	ALDOA, ENO1, GAPDH, LDHA, PGK1, PKM
1.00E+00	2.18	ANPEP, ANXA2, ATP5F1B, CAMP, CYBB, FLNA, ILK, ITGA2B, ITGB1, ITGB3, JUP, KRT1, LRP1
1.00E+00	4.40	ALDOA, ENO1, GALE, GAPDH, LDHA, PGK1, PKM
1.00E+00	3.06	CCL5, CD151, GSTP1, HLA-A, HSPD1, INPP5D, MSN, PRDX2, RAC2, SPN, TFRC
1.00E+00	3.88	ACTB, ANXA2, ATP2A3, B2M, CDC42, FLOT1, LRP1, RPL11
1.00E+00	6.33	CALM3, DSP, HSP90AA1, JUP, SRI
1.00E+00	6.33	FSCN1, ILK, MYH9, MYL12B, TPM4
1.00E+00	1.85	ACTB, ADAM10, B2M, CAMP, CCL5, CD47, CD9, CDC42, DHX9, GNAI2, GNG5, GSTP1, HLA
1.00E+00	3.52	ALDOA, ENO1, G6PD, GALE, GAPDH, PGK1, PKM, SLC2A1, SLC2A3
1.00E+00	3.52	CSDE1, DHX9, HNRNPC, HNRNPU, HSPA1B, NPM1, SYNCRIP, TNKS1BP1, VIM
1.00E+00	2.74	ANXA2, ARL6IP5, ATP2A3, BCAP31, CTSD, GAPDH, HSPD1, LRP1, LTF, RHOA, S100A8, S10
1.00E+00	3.86	ARPC5, HSP90AA1, HSPA1B, MYADM, PFN1, RHOA, SPTBN1, VDAC2
1.00E+00	14.50	GNAI2, GSTP1, SOD1
1.00E+00	14.50	FLNA, GP1BA, GP1BB
1.00E+00	14.50	FLNA, SRI, YWHAB
1.00E+00	14.50	FLNA, GP1BA, GP1BB
1.00E+00	14.50	ATP5F1B, BCAP31, TUBB
1.00E+00	14.50	RPL11, RPS15, UBE2L3
1.00E+00	8.57	B2M, HSPA1B, HSPA9, INPP5D
1.00E+00	8.57	ANXA2, CALM3, HSP90AA1, ITGB1
1.00E+00	45.75	HLA-A, HLA-B
1.00E+00	45.75	ACTB, VCL
1.00E+00	45.75	DSP, JUP
1.00E+00	45.75	SLC2A1, SLC2A3
1.00E+00	45.75	SLC2A1, SLC2A3
1.00E+00	45.75	ANXA2, MYH9
1.00E+00	45.75	LDHA, LDHB
1.00E+00	45.75	S100A8, S100A9
1.00E+00	45.75	S100A8, S100A9
1.00E+00	45.75	HSPA1B, HSPA8
1.00E+00	45.75	KRT14, VIM
1.00E+00	6.25	CALM3, DSP, FLNA, JUP, YWHAE
1.00E+00	2.61	ARL6IP5, CALM3, CLIC1, CYBB, FLNA, G6PD, ITGB1, ITGB3, SRI, STOM, TMEM109, UBQLN
1.00E+00	5.01	ILK, LRP1, S100A8, S100A9, SOD1, VIM
1.00E+00	2.11	CALM3, CAMP, CCL5, CD36, FLOT1, HSP90AA1, ILK, ITGB3, KANSL3, LTF, NPM1, PECAM1,

1.00E+00	4.28	CD36, CLIC1, FLNA, HSP90AA1, MYH9, MYL12B, MYL6
1.00E+00	8.38	BCAP31, CANX, SACM1L, TMX2
1.00E+00	3.46	ACTB, ARL6IP5, CD36, FLOT1, ITGB1, ITGB3, SLC2A1, SLC2A3, THBS1
1.00E+00	4.97	CCL5, CD47, CDC42, GAPDH, SYNCRIP, VIM
1.00E+00	2.70	CALM3, CCT2, CCT4, CCT6A, CCT7, CCT8, HNRNPU, RCC2, TCP1, TUBA1C, TUBA4A, TUBB,
1.00E+00	13.78	EEF1A1, HSPA8, STOM
1.00E+00	13.78	HNRNPA2B1, NPM1, PTBP1
1.00E+00	13.78	CCT2, HSP90AA1, HSPD1
1.00E+00	13.78	DHX9, FLOT1, NPM1
1.00E+00	13.78	ANXA2, ATP2A3, B2M
1.00E+00	3.43	ADAM10, CAMP, CCL5, DHX9, EIF5A, GSTP1, HSPA1B, ILK, THBS1
1.00E+00	3.43	ANK1, ANXA2, DSP, FLOT1, HSP90AA1, LRP1, MSN, SLC2A1, TFRC
1.00E+00	4.92	B2M, CD226, HLA-A, HLA-B, HSPD1, TFRC
1.00E+00	3.17	CCL5, CD151, HLA-A, HSPD1, INPP5D, MSN, PRDX2, RAC2, SPN, TFRC
1.00E+00	8.19	B2M, CTSD, FCER1G, HLA-A
1.00E+00	8.19	ADH5, GSTP1, S100A8, S100A9
1.00E+00	3.74	FSCN1, G3BP1, HNRNPU, HSPA1B, MSN, NPM1, RAB1B, RHOA
1.00E+00	6.00	CDC42, FLNA, ILK, ITGB3, MYADM
1.00E+00	6.00	CSDE1, DHX9, HNRNPC, HNRNPU, SYNCRIP
1.00E+00	4.88	B2M, CD226, HLA-A, HLA-B, HSPD1, TFRC
1.00E+00	1.99	CCL5, CSDE1, DHX9, EEF1A1, EEF2, EIF5A, GAPDH, HNRNPU, KRT17, NCL, NPM1, PKM, RH
1.00E+00	3.40	CALM3, DSP, ENO1, G6PD, HSP90AA1, JUP, RHOA, SOD1, SRI
1.00E+00	4.83	F11R, MSN, MYADM, PECAM1, RAP1B, VCL
1.00E+00	8.01	CALM3, HSP90AA1, HSPD1, VDAC2
1.00E+00	3.13	ATP2A3, BCAP31, CALM3, CCL5, FLNA, GP1BA, GP1BB, ITGB3, SRI, YWHAE
1.00E+00	2.65	ANXA11, ANXA2, ANXA5, ANXA7, CAVIN2, GSDMA, HSPA8, OSBPL2, PFN1, SPTBN1, THBS
1.00E+00	3.69	HSP90AA1, HSPA1B, NPM1, PPIA, RPL11, RPS15, UBE2L3, UBQLN1
1.00E+00	1.48	ACTB, ADAM10, ADH5, ANPEP, ANXA2, ANXA7, ARL6IP5, ATP2A3, B2M, BCAP31, CALM3,
1.00E+00	3.67	ADAM10, BCAP31, CD9, CLTA, HSPA8, M6PR, RAB27B, TFRC
1.00E+00	4.75	BCAP31, CALM3, HSP90AA1, HSPD1, RAC2, UBE2L3
1.00E+00	3.10	CCL5, CD151, HLA-A, HSPD1, INPP5D, MSN, PRDX2, RAC2, SPN, TFRC
1.00E+00	3.65	CCL5, CD151, HLA-A, MSN, PRDX2, RAC2, SPN, TFRC
1.00E+00	1.48	ACTB, ADAM10, ADH5, ALDOA, ANPEP, ANXA2, ARL6IP5, ATIC, ATP2A3, ATP5F1B, B2M, I
1.00E+00	2.14	ANXA2, ATP2A3, B2M, CALM3, CCL5, FLNA, G6PD, GNAI2, GP1BA, GP1BB, ITGB1, ITGB3,
1.00E+00	2.90	ATP5F1B, ITGB1, ITGB3, JUP, KRT16, KRT2, MYH9, PFN1, RHOA, RTN4, THBS1
1.00E+00	36.60	S100A8, S100A9
1.00E+00	36.60	ITGB3, RHOA
1.00E+00	36.60	DHX9, HNRNPA2B1
1.00E+00	36.60	DSP, JUP
1.00E+00	36.60	BCAP31, UBQLN1
1.00E+00	36.60	CALM3, HSP90AA1
1.00E+00	36.60	S100A8, S100A9
1.00E+00	2.75	ADAM10, CDC42, ENO1, G6PD, HSPA1B, KRT17, NPM1, PTPRJ, RHOA, RTN4, S100A8, S10
1.00E+00	3.63	F11R, KRT2, MSN, MYADM, PECAM1, RAP1B, VCL, VIM
1.00E+00	3.63	ANXA2, CALM3, GNAI2, RAB10, RAB27B, RAB33B, RAC2, RAP1B
1.00E+00	2.62	ANXA2, ARL6IP5, CALM3, CD36, CD47, GNAI2, ITGB3, RAB33B, RAP1B, SRI, THBS1, UBQL
1.00E+00	2.43	ALDOA, ATIC, ATP5F1B, ENO1, G6PD, GAPDH, HINT1, HSPA1B, HSPA8, LDHA, LDHB, PGK

1.00E+00	4.71	CALM3, FLNA, GP1BA, GP1BB, ITGB3, SRI
1.00E+00	4.71	DHX9, HNRNPA2B1, HNRNPU, HSPD1, PCBP1, WBP11
1.00E+00	12.52	ENO1, PGK1, PKM
1.00E+00	12.52	ANXA2, FLOT1, MYADM
1.00E+00	12.52	HNRNPA2B1, NPM1, PTBP1
1.00E+00	12.52	HLA-A, HLA-B, LBP
1.00E+00	12.52	B2M, HLA-A, HLA-B
1.00E+00	12.52	DAZAP1, HNRNPC, HNRNPH1
1.00E+00	3.31	ATP2A3, CALM3, DSP, FLNA, HSP90AA1, JUP, RHOA, SRI, YWHAE
1.00E+00	7.68	ITGB1, LBP, LGALS3BP, SPN
1.00E+00	3.62	ATP2A3, CALM3, DSP, FLNA, HSP90AA1, JUP, SRI, YWHAE
1.00E+00	1.91	ACTB, CAMP, CCL5, CD36, CYBB, EEF1A1, FLNA, GNAI2, HNRNPU, HSPA1B, HSPA8, ILK, IT
1.00E+00	5.70	ANXA2, B2M, FLOT1, LRP1, RPL11
1.00E+00	4.67	CALM3, FLNA, GP1BA, GP1BB, ITGB3, SRI
1.00E+00	2.88	ATP5F1B, ITGB1, ITGB3, JUP, KRT16, KRT2, MYH9, PFN1, RHOA, RTN4, THBS1
1.00E+00	4.01	B2M, G6PD, HSPA1B, HSPA9, INPP5D, PRDX1, SOD1
1.00E+00	5.63	CYB5R3, CYBB, GSR, PCYOX1, QSOX1
1.00E+00	7.52	CCL5, LBP, RAC2, THBS1
1.00E+00	2.85	ACTB, ANXA2, ATP2A3, B2M, CALM3, CDC42, DHX9, FLOT1, LRP1, RPL11, SRI
1.00E+00	2.71	ANXA2, ARL6IP5, ATP2A3, BCAP31, CTSD, GAPDH, HSPD1, LTF, RHOA, S100A8, S100A9, T
1.00E+00	4.59	CALM3, FLNA, GP1BA, GP1BB, ITGB3, SRI
1.00E+00	2.40	ALDOA, ATIC, ATP5F1B, ENO1, G6PD, GAPDH, HINT1, HSPA1B, HSPA8, LDHA, LDHB, PGK
1.00E+00	3.03	ADAM10, CCL5, FCER1G, LBP, PF4V1, PPIA, RAC2, S100A8, S100A9, THBS1
1.00E+00	2.70	ATP5F1B, CLIC1, DHX9, HNRNPC, HNRNPU, ILK, LTF, RHOA, RPS15, SRGN, SYNCRIP, TPM
1.00E+00	11.98	ANXA2, ENO1, MYH9
1.00E+00	11.98	B2M, HLA-A, HLA-B
1.00E+00	11.98	S100A8, S100A9, SRI
1.00E+00	11.98	MYH9, MYL12B, MYL6
1.00E+00	11.98	NCL, RPS3A, SYNCRIP
1.00E+00	5.57	B2M, LTF, SOD1, SRI, TFRC
1.00E+00	2.84	ATP5F1B, ITGB1, ITGB3, JUP, KRT16, KRT2, MYH9, PFN1, RHOA, RTN4, THBS1
1.00E+00	3.24	CALM3, CCL5, G6PD, GNAI2, ITGB3, RHOA, SRI, UBQLN1, YWHAE
1.00E+00	2.57	ANXA11, ATP2A3, CLIC1, CYBB, EIF5A, ENO1, GAPDH, GNAQ, MATR3, RAC2, RTN4, TMEN
1.00E+00	4.56	FLNA, HNRNPU, HSPA1B, RHOA, TUBB, TUBB1
1.00E+00	4.56	BCAP31, CALM3, HSP90AA1, HSPD1, RAC2, UBE2L3
1.00E+00	7.37	CCL5, GNAI2, PLEK, YWHAB
1.00E+00	7.37	CDC42, FSCN1, HSP90AA1, RAC2
1.00E+00	7.37	ICAM2, ITGB1, MYH9, RHOA
1.00E+00	7.37	ANXA11, ANXA2, ANXA5, ANXA7
1.00E+00	2.69	ARL6IP5, CALM3, CD36, CLIC1, DSP, FLNA, GNAQ, JUP, SOD1, SRI, YWHAE, YWHAH
1.00E+00	3.53	ARPC5, CFL1, FLNA, FSCN1, MYH9, SPTBN1, TLN1, TPM4
1.00E+00	1.50	AACS, ADAM10, ADH5, ALDOA, ANPEP, ATIC, ATP2A3, ATP5F1B, CASP14, CCL5, CCT2, CC
1.00E+00	2.31	ACTB, B2M, CCL5, CD226, CD47, HLA-A, HSPD1, INPP5D, LBP, PRDX2, RAC2, RHOA, SOD1
1.00E+00	1.75	ACTB, ARL6IP5, ATP2A3, B2M, BCAP31, CANX, CD36, CYBB, DHX9, ENO1, FLNA, FLOT1, G
1.00E+00	2.14	CALM3, CPN1, CYBB, DSG1, GNAI2, HNRNPU, HSP90AA1, HSPA1B, HSPA8, ITGB3, NCL, PI
1.00E+00	5.50	ANK1, ATP2A3, SRI, THBS1, TMEM109
1.00E+00	3.22	ATP2A3, CD36, LRP1, PTPRJ, RHOA, SLC2A1, SLC2A3, SOD1, TFRC

1.00E+00	2.81	ACTB, B2M, CCL5, CD47, GP1BA, HLA-A, HSPD1, PRDX2, RHOA, SPN, TFRC
1.00E+00	2.30	ACTB, CALM3, CCL5, GNAQ, GSTP1, HSP90AA1, ITGB3, LTF, NPM1, PPIA, PTPRJ, RHOA, S(
1.00E+00	2.30	ALDOA, ATIC, ATP5F1B, ENO1, G6PD, GAPDH, HINT1, HSPA1B, HSPA8, LDHA, LDHB, NAN
1.00E+00	5.43	ALDOA, B2M, KRT1, KRT10, OSBPL2
1.00E+00	5.43	GNAI2, GNG5, ILK, ITGB1, RHOA
1.00E+00	2.05	ANXA2, BCAP31, CSDE1, DHX9, EEF1A1, FLNA, GAPDH, HNRNPC, HNRNPU, HSP90AA1, H
1.00E+00	11.48	CD226, HLA-A, HSPD1
1.00E+00	3.19	ANK1, ANXA2, DSP, FLOT1, HSP90AA1, LRP1, MSN, SLC2A1, TFRC
1.00E+00	2.29	ATP2A3, ATP5F1B, BCAP31, CALM3, CCL5, FLNA, GP1BA, GP1BB, ITGB3, RTN4, S100A8, S
1.00E+00	4.45	CALM3, FLNA, GP1BA, GP1BB, ITGB3, SRI
1.00E+00	30.50	NPM1, RPS15
1.00E+00	30.50	ARL6IP5, ITGB1
1.00E+00	30.50	HLA-A, HLA-B
1.00E+00	30.50	LBP, PRDX2
1.00E+00	30.50	ANXA2, ENO1
1.00E+00	30.50	DHX9, HNRNPU
1.00E+00	30.50	DHX9, HNRNPU
1.00E+00	30.50	S100A8, S100A9
1.00E+00	30.50	CD47, PECAM1
1.00E+00	30.50	GNAI2, RAP1B
1.00E+00	30.50	CDC42, YWHAZ
1.00E+00	30.50	SLC2A1, SLC2A3
1.00E+00	30.50	PFN1, RHOA
1.00E+00	30.50	CD36, LRP1
1.00E+00	30.50	ANXA2, B2M
1.00E+00	30.50	EIF5A, RPL11
1.00E+00	30.50	PSMA5, PSMA6
1.00E+00	30.50	B2M, HLA-A
1.00E+00	30.50	B2M, TFRC
1.00E+00	30.50	SLC2A1, SLC2A3
1.00E+00	30.50	DAZAP1, HNRNPU
1.00E+00	30.50	HLA-A, HLA-B
1.00E+00	30.50	RPL11, RPS15
1.00E+00	7.09	CD36, ITGB3, JUP, PTPRJ
1.00E+00	7.09	CALM3, SRI, UBQLN1, YWHAE
1.00E+00	7.09	MYH16, MYH9, MYL12B, MYL6
1.00E+00	3.85	CD36, CD47, DHX9, HLA-B, HSPD1, INPP5D, LBP
1.00E+00	3.85	CD36, CD47, DHX9, HLA-B, HSPD1, INPP5D, LBP
1.00E+00	1.62	ADAM10, CCL5, CD226, CD36, CD47, CD59, CDC42, EIF5A, FCER1G, FCN1, FERMT3, FLNA
1.00E+00	4.41	ALDOA, ENO1, GAPDH, LDHA, PGK1, PKM
1.00E+00	3.16	ACTB, B2M, CCL5, CD47, GP1BA, HLA-A, HSPD1, RHOA, TFRC
1.00E+00	3.82	ANXA2, B2M, CALM3, DHX9, FLOT1, LRP1, RPL11
1.00E+00	3.14	GNAQ, GSTP1, HNRNPU, NPM1, PPIA, PTPRJ, RPL11, RPS15, YWHAG
1.00E+00	2.93	CD36, DHX9, FLNA, HNRNPA2B1, HSP90AA1, HSPA9, JUP, NPM1, RPS15, YWHAE
1.00E+00	2.93	CD36, DHX9, FLNA, HNRNPA2B1, HSP90AA1, HSPA9, JUP, NPM1, RPS15, YWHAE
1.00E+00	11.02	CAMP, H2BC21, LTF
1.00E+00	11.02	CD36, PRDX2, THBS1

1.00E+00	11.02	CD36, LTF, PRDX2
1.00E+00	11.02	HNRNPA2B1, NPM1, PTBP1
1.00E+00	11.02	CSDE1, G3BP1, RPS23
1.00E+00	11.02	CD36, PRDX2, THBS1
1.00E+00	11.02	ANXA2, ENO1, MYH9
1.00E+00	11.02	ITGB1, RPSA, THBS1
1.00E+00	5.25	FLNA, HNRNPU, SRGN, SRI, YWHAB
1.00E+00	5.25	FSCN1, ICAM2, ITGB3, MSN, SPN
1.00E+00	2.41	ANXA11, ATIC, B2M, CALM3, CD36, CYBB, G6PD, PRDX1, PRDX2, RPLP0, S100A8, SOD1, T
1.00E+00	4.34	B2M, CD226, HLA-A, HLA-B, PRDX1, TUBB
1.00E+00	4.34	DSP, FLNA, JUP, MYH9, MYL6, SRI
1.00E+00	1.95	CYBB, ENO1, F11R, GNAQ, HSP90AA1, HSPA1B, HSPD1, ITGB1, ITGB3, JUP, KRT5, NPM1,
1.00E+00	3.76	ARL6IP5, ATIC, G6PD, GSR, GSTP1, PCYOX1, SOD1
1.00E+00	6.82	PFN1, YWHAB, YWHAE, YWHAZ
1.00E+00	5.19	CD47, CDC42, F11R, PFN1, RHOA
1.00E+00	3.73	ALDOA, CALM3, DSP, FLNA, HSP90AA1, JUP, SRI
1.00E+00	1.76	ADAM10, ANPEP, ANXA2, ARL6IP5, ATP2A3, BCAP31, CANX, CASP14, CPN1, CTSD, DCD, I
1.00E+00	10.59	PPIA, SOD1, UBQLN1
1.00E+00	10.59	HSPA1B, HSPA8, HSPA9
1.00E+00	4.27	CD36, CDC42, MYH9, RAC2, RHOA, THBS1
1.00E+00	4.27	ANXA2, FLOT1, ITGB1, SLC2A1, SRI, VCL
1.00E+00	2.59	ALDOA, ATIC, ATP5F1B, ENO1, GAPDH, HINT1, HSPA1B, HSPA8, LDHA, PGK1, PKM, RHOA
1.00E+00	3.08	ARPC5, HSP90AA1, HSPA1B, KRT5, MYADM, PFN1, RHOA, SPTBN1, VDAC2
1.00E+00	5.13	ARPC5, CCL5, ITGB3, LRP1, RHOA
1.00E+00	5.13	CD36, PNPLA8, PPIA, SOD1, UBQLN1
1.00E+00	2.04	AACS, ANK1, ANXA2, CALM3, CANX, CCL5, GNAI2, M6PR, MYH9, PLEK, PNPLA8, PPIA, RA
1.00E+00	6.70	B2M, CD226, HLA-A, HLA-B
1.00E+00	6.70	CD36, FLNA, HSP90AA1, JUP
1.00E+00	4.24	B2M, G6PD, HSPA1B, HSPA9, INPP5D, PRDX1
1.00E+00	2.57	CD47, CD59, HLA-A, HLA-B, HSPA9, INPP5D, LTF, PRDX2, PTPRJ, SPN, THBS1, TTLL12
1.00E+00	2.57	ACTB, B2M, CCL5, CD226, CD47, HLA-A, HSPD1, INPP5D, LBP, RHOA, TFRC, THBS1
1.00E+00	5.07	ACTB, CDC42, MSN, RAB10, RHOA
1.00E+00	26.14	HLA-A, HLA-B
1.00E+00	26.14	SLC2A1, SLC2A3
1.00E+00	26.14	FLOT1, UBQLN1
1.00E+00	26.14	FCER1G, HLA-A
1.00E+00	26.14	SLC2A1, SLC2A3
1.00E+00	26.14	BCAP31, UBQLN1
1.00E+00	26.14	ANK1, SPTBN1
1.00E+00	26.14	ANXA7, SRI
1.00E+00	26.14	PNPLA8, PRDX6
1.00E+00	26.14	RPL11, RPS15
1.00E+00	6.58	CD151, FLNA, ITGB1, RHOA
1.00E+00	6.58	B2M, HLA-A, HLA-B, HSPD1
1.00E+00	10.20	B2M, CTSD, FCER1G
1.00E+00	10.20	HSPD1, LBP, THBS1
1.00E+00	10.20	CD36, PRDX2, THBS1

1.00E+00	10.20	CD9, F11R, MMRN1
1.00E+00	10.20	EIF5A, RPL11, RPS15
1.00E+00	10.20	DAZAP1, HNRNPU, SYNCRIP
1.00E+00	10.20	CD36, HSPD1, THBS1
1.00E+00	10.20	CD36, HSPD1, THBS1
1.00E+00	3.04	CSDE1, DHX9, GAPDH, HNRNPU, NCL, RTN3, RTN4, SYNCRIP, TNKS1BP1
1.00E+00	5.02	ADAM10, CCL5, LBP, RAC2, THBS1
1.00E+00	5.02	CALM3, DSP, HSP90AA1, JUP, SRI
1.00E+00	5.02	B2M, HLA-A, HLA-B, RAB10, TFRC
1.00E+00	2.06	ACTB, CCT2, CCT4, CCT6A, CCT7, CCT8, CSDE1, DHX9, HNRNPU, HSPA8, KANSL3, PSMA5,
1.00E+00	3.29	DHX9, HSP90AA1, RPL11, RPLP0, RPS15, RPSA, SF3A3, XRCC5
1.00E+00	3.29	CAMP, CCL5, DHX9, EIF5A, GSTP1, HSPA1B, ILK, THBS1
1.00E+00	2.82	CYBB, H2AC20, H2BC21, ITGB1, KRT1, KRT10, SRI, TPM4, YWHAE, YWHAH
1.00E+00	4.15	B2M, CD226, HLA-A, HLA-B, HSPD1, TFRC
1.00E+00	4.15	CDC42, CFL1, F11R, FLOT1, ITGB1, RHOA
1.00E+00	4.15	CCL5, CD47, CDC42, GAPDH, SYNCRIP, VIM
1.00E+00	4.15	CALM3, DSP, FLNA, HSP90AA1, JUP, SRI
1.00E+00	6.46	PLEK, RAC2, RHOA, TLN1
1.00E+00	1.84	ACTB, ADAM10, ANK1, ARPC5, CALM3, CANX, CDC42, CFL1, CYBB, FLNA, FSCN1, GNAI2, (
1.00E+00	1.98	ANTXR2, ANXA2, ARPC5, B2M, CLTA, CTSD, FLOT1, HLA-A, HLA-B, HSPA8, HSPD1, ITGB1,
1.00E+00	3.61	ANXA11, CALM3, CDC42, CFL1, MYH9, RHOA, SPTBN1
1.00E+00	3.61	CAMP, CYBB, ITGB1, ITGB3, JUP, PKM, THBS1
1.00E+00	3.61	CAMP, CYBB, ITGB1, ITGB3, JUP, PKM, THBS1
1.00E+00	4.11	HSP90AA1, HSPA1B, HSPA8, HSPA9, HSPD1, THBS1
1.00E+00	1.91	ANXA2, ANXA5, B2M, CD9, G6PD, GNAI2, GP1BA, GSTP1, HSPA9, INPP5D, JUP, KRT1, LBF
1.00E+00	3.00	CALM3, CCL5, FCN1, FLNA, GNAI2, GNAQ, HSPA1B, HSPA8, PF4V1
1.00E+00	9.84	B2M, HLA-A, HLA-B
1.00E+00	9.84	B2M, G6PD, TFRC
1.00E+00	9.84	ITGB3, LTF, RHOA
1.00E+00	9.84	CALM3, DSP, JUP
1.00E+00	9.84	CANX, HNRNPU, SOD1
1.00E+00	9.84	ITGB1, ITGB3, THBS1
1.00E+00	4.91	GSTP1, HSPA1B, ITGA6, PRDX2, THBS1
1.00E+00	4.91	GNAI2, GNAQ, GNG5, OSBPL2, RHOA
1.00E+00	6.35	ADAM10, FLOT1, FSCN1, LRP1
1.00E+00	6.35	CD36, ITGB1, PRDX2, THBS1
1.00E+00	6.35	CD36, CYBB, RHOA, SOD1
1.00E+00	2.25	B2M, CDC42, CFL1, FLNA, FSCN1, HSP90AA1, ITGA6, PFN1, RAC2, RCC2, RHOA, RTN4, S1(
1.00E+00	2.25	ACTB, ADAM10, ARPC5, CALM3, CANX, CFL1, FLNA, FSCN1, HSP90AA1, HSPA8, RAB27B, I
1.00E+00	4.86	CANX, CCL5, CSDE1, DHX9, PTBP1
1.00E+00	1.83	ANXA2, BCAP31, CANX, CPN1, CSDE1, CTSD, DHX9, EEF1A1, FLNA, HNRNPC, HNRNPU, HS
1.00E+00	4.05	CD36, CDC42, MYH9, RAC2, RHOA, THBS1
1.00E+00	4.05	ALDOA, FLNA, JUP, MYL12B, SLC2A1, SRI
1.00E+00	6.24	CAMP, H2BC21, HLA-A, LTF
1.00E+00	6.24	B2M, CD59, HLA-A, HLA-B
1.00E+00	9.50	HSPA1B, HSPA8, HSPA9
1.00E+00	22.87	ANXA2, FLOT1

1.00E+00	22.87	DSP, JUP
1.00E+00	22.87	CD36, LBP
1.00E+00	22.87	CD36, LBP
1.00E+00	22.87	CALM3, YWHAE
1.00E+00	22.87	ADAM10, TSPAN14
1.00E+00	22.87	ACTB, NPM1
1.00E+00	22.87	CLTA, LRP1
1.00E+00	22.87	JUP, VCL
1.00E+00	22.87	S100A8, S100A9
1.00E+00	22.87	HNRNPA2B1, HNRNPC
1.00E+00	3.18	DHX9, HSP90AA1, RPL11, RPLP0, RPS15, RPSA, SF3A3, XRCC5
1.00E+00	4.76	B2M, CD226, CD59, HLA-A, HLA-B
1.00E+00	6.14	FCN1, HSP90AA1, MATR3, XRCC5
1.00E+00	6.14	CALM3, PLEK, PPIA, PTBP1
1.00E+00	6.14	CCL5, DHX9, PPIA, STOM
1.00E+00	6.14	FLG2, HRNR, KRT1, KRT16
1.00E+00	2.48	ALDOA, ATIC, ATP5F1B, ENO1, GAPDH, HINT1, HSPA1B, HSPA8, LDHA, PGK1, PKM, RHOA
1.00E+00	4.71	DAZAP1, DHX9, GSTP1, ITGB3, PRDX1
1.00E+00	4.71	CSDE1, DHX9, HNRNPU, SYNCRIP, TNKS1BP1
1.00E+00	9.18	CD47, FCER1G, PTPRJ
1.00E+00	1.79	AACS, ACTB, ATP2A3, ATP5F1B, CCT2, CCT4, CCT6A, CCT7, CCT8, DDX60L, DHX9, G3BP1,
1.00E+00	2.72	CDC42, CFL1, F11R, FLOT1, G3BP1, ITGB1, RAB33B, RAC2, RAP1B, RHOA
1.00E+00	4.66	BCAP31, HSP90AA1, HSPD1, RAC2, UBE2L3
1.00E+00	4.66	DSP, FLNA, JUP, MYL6, SRI
1.00E+00	4.66	CD47, CDC42, F11R, PFN1, RHOA
1.00E+00	4.66	CD36, PNPLA8, PPIA, SOD1, UBQLN1
1.00E+00	3.45	FLNA, HNRNPU, HSPA1B, MYH9, RHOA, TUBB, TUBB1
1.00E+00	2.89	CSDE1, DHX9, HNRNPC, HNRNPU, HSPA1B, NPM1, SYNCRIP, TNKS1BP1, VIM
1.00E+00	1.77	AACS, ACTB, ATP2A3, ATP5F1B, CCT2, CCT4, CCT6A, CCT7, CCT8, DDX60L, DHX9, G3BP1,
1.00E+00	3.12	CALM3, CCL5, GSTP1, LTF, NPM1, RAC2, YWHAB, YWHAG
1.00E+00	2.70	ADAM10, CAMP, CYBB, ITGB1, ITGB3, JUP, KRT1, PGK1, PKM, THBS1
1.00E+00	1.42	ACTB, ADAM10, ANXA2, ANXA7, B2M, CCL5, CD226, CD36, CD47, CPN1, CSDE1, CYBB, D
1.00E+00	5.94	B2M, CD226, HLA-A, HLA-B
1.00E+00	5.94	HSP90AA1, HSPA1B, THBS1, YWHAE
1.00E+00	5.94	ACTB, CALM3, CANX, RAB27B
1.00E+00	5.94	ACTB, CALM3, CANX, RAB27B
1.00E+00	3.11	ANXA2, CSDE1, DHX9, HNRNPC, HNRNPU, QSOX1, RPL11, SYNCRIP
1.00E+00	4.62	CALM3, FLOT1, ITGB1, ITGB3, RAP1B
1.00E+00	4.62	HLA-B, HSPA8, HSPD1, SOD1, TFRC
1.00E+00	3.88	GNAI2, GNG5, ILK, ITGB1, RAB10, RHOA
1.00E+00	2.44	ACTB, B2M, FCER1G, HLA-B, INPP5D, ITGB1, MYH9, PRDX2, PTPRJ, RHOA, SOD1, SPN
1.00E+00	8.88	B2M, CTSD, FCER1G
1.00E+00	8.88	GNAI2, YWHAB, YWHAE
1.00E+00	8.88	DSP, JUP, SRI
1.00E+00	8.88	CDC42, MSN, RHOA
1.00E+00	8.88	CD99, LBP, RAC2
1.00E+00	8.88	GNAI2, GNAQ, GNG5

1.00E+00	2.19	B2M, CDC42, CFL1, FLNA, FSCN1, HSP90AA1, ITGA6, PFN1, RAC2, RCC2, RHOA, RTN4, S1(
1.00E+00	2.26	DAZAP1, DHX9, G3BP1, HNRNPA2B1, HNRNPA3, HNRNPC, HNRNPU, HSP90AA1, NCL, PC
1.00E+00	2.69	CALM3, GNAI2, INPP5D, PLEK, PPIA, PTBP1, PTPRJ, SACM1L, YWHAB, YWHAE
1.00E+00	20.33	PLEK, RAP1B
1.00E+00	20.33	CCL5, FERMT3
1.00E+00	20.33	HNRNPU, RCC2
1.00E+00	20.33	CALM3, SRI
1.00E+00	20.33	CSDE1, PTBP1
1.00E+00	20.33	F11R, MMRN1
1.00E+00	20.33	CALM3, SRI
1.00E+00	20.33	G6PD, SLC2A3
1.00E+00	20.33	PCYOX1, QSOX1
1.00E+00	20.33	GSR, PGK1
1.00E+00	3.40	B2M, HLA-A, HLA-B, ITGB1, LTF, RAB10, TFRC
1.00E+00	5.85	CD36, PNPLA8, SLC2A1, THBS1
1.00E+00	5.85	ANXA2, ENO1, PGK1, THBS1
1.00E+00	5.85	ANXA2, ENO1, MYH9, THBS1
1.00E+00	5.85	NPM1, RPL11, RPL8, RPLP0
1.00E+00	2.42	ALDOA, ATIC, ATP5F1B, ENO1, GAPDH, HINT1, HSPA1B, HSPA8, LDHA, PGK1, PKM, RHOA
1.00E+00	3.07	ADAM10, ANXA2, CPN1, ENO1, MYH9, PGK1, SRGN, THBS1
1.00E+00	4.52	CALM3, DSP, JUP, SRI, YWHAE
1.00E+00	4.52	CSDE1, DHX9, HNRNPU, SYNCRIP, TNKS1BP1
1.00E+00	4.52	CSDE1, DHX9, HNRNPU, SYNCRIP, TNKS1BP1
1.00E+00	3.06	ACTB, B2M, CCL5, CD47, HLA-A, HSPD1, RHOA, TFRC
1.00E+00	2.66	ANXA2, BCAP31, EEF1A1, FLNA, HSP90AA1, HSPA1B, LRP1, MSN, RPL11, UBQLN1
1.00E+00	8.61	FLOT1, MYADM, PLEK
1.00E+00	8.61	CALM3, GAPDH, HSP90AA1
1.00E+00	3.36	ARPC5, CFL1, MYADM, PFN1, PLEK, RHOA, SPTBN1
1.00E+00	3.05	HSP90AA1, HSPA1B, NPM1, PPIA, RPL11, RPS15, UBE2L3, UBQLN1
1.00E+00	4.48	ARPC5, CCL5, ITGB3, LRP1, RHOA
1.00E+00	4.48	CDC42, FSCN1, ITGB1, ITGB3, MSN
1.00E+00	1.73	ACTB, ANXA2, B2M, CD36, CDC42, CLIC1, F11R, FLNA, G6PD, HLA-B, HNRNPU, HSPA1B, H
1.00E+00	3.03	CALM3, CYBB, G6PD, JUP, RHOA, RPLP0, S100A8, SOD1
1.00E+00	5.67	FLG2, HRNR, KRT1, KRT16
1.00E+00	1.93	ACTB, B2M, G6PD, HNRNPU, HSPA9, INPP5D, ITGB1, ITGB3, LTF, PGK1, PRDX2, PTBP1, R(
1.00E+00	2.80	ADAM10, ATP5F1B, CD9, MYADM, PRDX2, RCC2, RHOA, SPN, THBS1
1.00E+00	4.44	CD47, CDC42, F11R, PFN1, RHOA
1.00E+00	4.44	CD47, CDC42, F11R, PFN1, RHOA
1.00E+00	3.75	ARL6IP5, CALM3, SRI, THBS1, UBQLN1, YWHAE
1.00E+00	8.34	ITGB3, LTF, RHOA
1.00E+00	8.34	EEF2, HSPA1B, UBQLN1
1.00E+00	8.34	CDC42, HNRNPU, RCC2
1.00E+00	1.53	ACTB, ANXA2, BCAP31, CANX, CCL5, CCT2, CCT4, CCT6A, CCT7, CCT8, CSDE1, DHX9, EEF1
1.00E+00	1.41	ACTB, ADAM10, ALDOA, ANPEP, ANXA2, ARL6IP5, ATIC, ATP5F1B, CCL5, CCT2, CCT4, CCT
1.00E+00	4.39	CTSD, LRBA, RAB1B, RAB33B, UBQLN1
1.00E+00	2.78	CD9, ILK, LRP1, RHOA, RTN4, S100A8, S100A9, SOD1, VIM
1.00E+00	3.29	CD47, CDC42, F11R, ITGB1, MYH9, PFN1, RHOA

1.00E+00	2.61	B2M, CD226, FCER1G, HLA-A, HLA-B, HSPD1, INPP5D, PRDX1, TFRC, TUBB
1.00E+00	2.61	ARPC5, HSP90AA1, MSN, MYADM, NPM1, PFN1, PLEK, RHOA, RTN4, SPTBN1
1.00E+00	18.30	FCN1, KRT1
1.00E+00	18.30	HLA-A, HSPD1
1.00E+00	18.30	LBP, LTF
1.00E+00	18.30	GP1BA, PLEK
1.00E+00	18.30	DSP, JUP
1.00E+00	18.30	PFN1, RHOA
1.00E+00	18.30	RPL11, RPS15
1.00E+00	18.30	CD36, HSPD1
1.00E+00	18.30	ANXA2, PPIA
1.00E+00	18.30	SLC2A1, SLC2A3
1.00E+00	18.30	HSPA1B, HSPA8
1.00E+00	2.48	ANXA11, CALM3, CDC42, CLTA, HNRNPU, MYH9, NPM1, RCC2, TTLL12, TUBB, TUBB1
1.00E+00	3.28	CALM3, CYBB, DSG1, MSN, NASP, RPLP0, THBS1
1.00E+00	4.35	CDC42, FSCN1, HSP90AA1, PFN1, RAC2
1.00E+00	5.50	CLTA, HSPD1, LRP1, TFRC
1.00E+00	8.10	ENO1, PGK1, PKM
1.00E+00	8.10	HNRNPA2B1, NCL, XRCC5
1.00E+00	1.85	ANPEP, ANXA2, ATP5F1B, CAMP, CDC42, CYBB, FLNA, ILK, ITGA2B, ITGB1, ITGB3, JUP, KR
1.00E+00	2.18	AACS, ADH5, ALDOA, CD36, ENO1, GAPDH, GSTP1, LDHA, LDHB, OSBPL2, PGK1, PKM, PN
1.00E+00	5.41	ANXA2, ENO1, MYH9, THBS1
1.00E+00	5.41	ICAM2, ITGB1, MYH9, RHOA
1.00E+00	3.23	CDC42, HNRNPU, HSPA8, RAB27B, SOD1, TUBA1C, TUBB
1.00E+00	4.27	CAVIN2, CD36, FLOT1, SELPLG, SLC2A1
1.00E+00	2.25	ANXA7, CTSD, EEF1A1, GAPDH, HSP90AA1, HSPA8, LRBA, QSOX1, RAB1B, RAB33B, S100A
1.00E+00	2.25	ANXA7, CTSD, EEF1A1, GAPDH, HSP90AA1, HSPA8, LRBA, QSOX1, RAB1B, RAB33B, S100A
1.00E+00	3.63	ARPC5, MYADM, PFN1, PLEK, RHOA, SPTBN1
1.00E+00	3.63	DHX9, EEF2, EIF5A, HNRNPU, NPM1, RPSA
1.00E+00	7.87	GNAI2, RAB33B, RAP1B
1.00E+00	2.17	ACTB, ADAM10, CANX, CDC42, CLTA, HSPA8, ITGB1, ITGB3, RHOA, RPL8, RPLP0, RTN3, R
1.00E+00	4.23	HSP90AA1, HSPA1B, SOD1, THBS1, YWHAE
1.00E+00	5.34	DHX9, HNRNPU, LTF, PFN1
1.00E+00	5.34	RAB10, RAB27B, RAP1B, RHOA
1.00E+00	2.00	AACS, ANK1, ANXA2, CALM3, CANX, CCL5, GNAI2, M6PR, MYH9, PLEK, PPIA, RAB10, RAB
1.00E+00	1.67	ALDOA, ATIC, ATP5F1B, CCL5, CSDE1, DHX9, EEF1A1, EEF2, EIF5A, ENO1, GAPDH, GSTP1,
1.00E+00	2.16	ADAM10, CCL5, FCER1G, ITGB3, LBP, PF4V1, PPIA, PTPRJ, RAC2, RHOA, S100A8, S100A9,
1.00E+00	1.69	ACTB, ANXA2, B2M, CD9, CDC42, CFL1, FLNA, FLOT1, HSP90AA1, ILK, ITGA6, ITGB1, LRP1
1.00E+00	2.32	ARL6IP5, CCL5, CD36, CDC42, GNAI2, ITGB3, PPIA, PRDX2, PTPRJ, RAP1B, SOD1, THBS1
1.00E+00	16.63	LRP1, PTPRJ
1.00E+00	16.63	ITGB3, LTF
1.00E+00	16.63	ACTB, VCL
1.00E+00	16.63	CALM3, SRI
1.00E+00	16.63	CD99, F11R
1.00E+00	16.63	ATP2A3, B2M
1.00E+00	16.63	ANXA7, SRI
1.00E+00	16.63	ANXA2, ANXA5

1.00E+00	16.63	GSR, PGK1
1.00E+00	16.63	KRT14, VIM
1.00E+00	16.63	ITGA6, ITGB3
1.00E+00	16.63	DSG1, PTPRJ
1.00E+00	16.63	ACTB, CALM3
1.00E+00	2.70	B2M, FLNA, GP1BA, GP1BB, ITGA2B, ITGA6, ITGB1, ITGB3, TFRC
1.00E+00	4.19	ARL6IP5, CALM3, SRI, UBQLN1, YWHAE
1.00E+00	3.18	F11R, ITGB3, JUP, KRT5, RHOA, SLC2A1, THBS1
1.00E+00	2.16	ADAM10, CCL5, FCER1G, ITGB3, LBP, PF4V1, PPIA, PTPRJ, RAC2, RHOA, S100A8, S100A9,
1.00E+00	2.16	CALM3, CCT4, CCT8, CDC42, DHX9, FLOT1, G6PD, GNAI2, HNRNPU, HSPA1B, MYH16, NPI
1.00E+00	5.26	CALM3, SRI, UBQLN1, YWHAE
1.00E+00	5.26	FLNA, HNRNPU, HSPA1B, RHOA
1.00E+00	5.26	MYL12B, RAB10, RAB27B, RHOA
1.00E+00	7.65	CAMP, H2BC21, LTF
1.00E+00	7.65	DHX9, FLOT1, HSP90AA1
1.00E+00	7.65	GSR, PGK1, QSOX1
1.00E+00	7.65	CYB5R3, PCYOX1, QSOX1
1.00E+00	2.15	ADAM10, CDC42, ENO1, G6PD, HSPA1B, KRT17, NPM1, PTPRJ, RHOA, RTN4, S100A8, S10
1.00E+00	3.56	ARPC5, MYADM, PFN1, PLEK, RHOA, SPTBN1
1.00E+00	3.56	CCL5, DHX9, FCN1, LTF, PPIA, STOM
1.00E+00	4.16	HLA-A, HLA-B, LTF, PRDX2, TTLL12
1.00E+00	4.16	S100A8, S100A9, SOD1, SRI, TFRC
1.00E+00	2.68	ADAM10, ANXA2, FLOT1, FSCN1, HRNR, ITGB1, ITGB3, LRP1, QSOX1
1.00E+00	1.95	AACS, ANK1, ANXA2, CALM3, CANX, CCL5, GNAI2, M6PR, MYH9, PLEK, PPIA, RAB10, RAB
1.00E+00	3.53	HSP90AA1, HSPA1B, HSPA8, HSPA9, HSPD1, THBS1
1.00E+00	4.12	ATIC, RHOA, RTN4, SLC2A1, YWHAE
1.00E+00	4.12	CTSD, LRBA, RAB1B, RAB33B, UBQLN1
1.00E+00	2.51	CALM3, GNAQ, GSTP1, HNRNPU, LRP1, MYADM, NPM1, PPIA, PTPRJ, YWHAG
1.00E+00	3.51	B2M, BCAP31, CANX, HLA-A, HLA-B, RTN4
1.00E+00	2.08	ARL6IP5, CCL5, CD36, CDC42, GNAI2, GSTP1, ITGB3, PPIA, PRDX1, PRDX2, PTPRJ, RAP1B,
1.00E+00	7.44	ANXA11, MYH9, RHOA
1.00E+00	7.44	FLNA, HSP90AA1, JUP
1.00E+00	7.44	DSP, JUP, SRI
1.00E+00	7.44	GNAI2, GNAQ, GNG5
1.00E+00	1.71	AACS, ACTB, ATP2A3, ATP5F1B, CCT2, CCT4, CCT6A, CCT7, CCT8, DDX60L, DHX9, G3BP1,
1.00E+00	1.87	ALDOA, ATIC, ATP5F1B, ENO1, G6PD, GAPDH, HINT1, HSPA1B, HSPA8, INPP5D, LDHA, LD
1.00E+00	5.11	CD36, CD47, CYB5R3, HSP90AA1
1.00E+00	5.11	DSP, FLNA, JUP, SRI
1.00E+00	3.49	CALM3, CPN1, CYBB, HNRNPU, RHOA, UBE2L3
1.00E+00	3.49	DHX9, HNRNPA2B1, HSPA9, NPM1, RPS15, YWHAE
1.00E+00	1.48	ACTB, ADAM10, ADH5, CALM3, CAMP, CCL5, CD36, CDC42, DSP, FLNA, FLOT1, G6PD, GA
1.00E+00	1.76	CCL5, CD47, ENO1, G3BP1, GNAI2, GSTP1, HSPA1B, INPP5D, ITGA6, ITGB1, LRP1, LTF, MY
1.00E+00	15.25	LTF, PRDX2
1.00E+00	15.25	CDC42, YWHAZ
1.00E+00	15.25	CD99, PECAM1
1.00E+00	15.25	ATIC, ATP5F1B
1.00E+00	15.25	ATIC, ATP5F1B

1.00E+00	15.25	CALM3, SRI
1.00E+00	15.25	BCAP31, UBQLN1
1.00E+00	15.25	HNRNPU, RCC2
1.00E+00	15.25	ITGB3, PTPRJ
1.00E+00	15.25	ANXA11, ANXA2
1.00E+00	15.25	HNRNPA2B1, PCBP1
1.00E+00	2.27	ALDOA, ATIC, ATP5F1B, ENO1, GAPDH, HINT1, HSPA1B, HSPA8, LDHA, PGK1, PKM, RHOA
1.00E+00	5.04	ATP2A3, HSPD1, S100A8, S100A9
1.00E+00	5.04	HNRNPU, HSPA8, RAB27B, SOD1
1.00E+00	5.04	F11R, PECAM1, RHOA, VCL
1.00E+00	7.24	B2M, HLA-A, HLA-B
1.00E+00	7.24	CD47, CD99, GP1BA
1.00E+00	7.24	CAMP, CCL5, FCER1G
1.00E+00	7.24	INPP5D, ITGB3, TFRC
1.00E+00	7.24	ATP2A3, SRI, TMEM109
1.00E+00	3.45	ADAM10, CDC42, KRT17, RHOA, S100A8, S100A9
1.00E+00	3.45	ARPC5, CALM3, CFL1, FLNA, FSCN1, HSP90AA1
1.00E+00	3.07	CCL5, HSP90AA1, ILK, ITGB1, PTPRJ, RTN4, THBS1
1.00E+00	3.07	CALM3, CCL5, LTF, NPM1, RAC2, YWHAB, YWHAG
1.00E+00	4.01	HSP90AA1, ITGB1, PTPRJ, RTN4, THBS1
1.00E+00	2.00	ACTB, ARPC5, ATIC, B2M, CALM3, CDC42, G6PD, ITGB1, RHOA, RTN3, RTN4, SLC2A1, TM
1.00E+00	1.77	ANXA11, ATP2A3, ATP5F1B, CALM3, CLIC1, CYB5R3, CYBB, EIF5A, ENO1, GAPDH, GNAQ,
1.00E+00	1.77	ANXA11, ATP2A3, ATP5F1B, CALM3, CLIC1, CYB5R3, CYBB, EIF5A, ENO1, GAPDH, GNAQ,
1.00E+00	2.17	ACTB, B2M, CCL5, CD47, HLA-A, HSPD1, INPP5D, PRDX2, RAC2, RHOA, SOD1, SPN, TFRC
1.00E+00	4.97	NPM1, RPS15, RPSA, XRCC5
1.00E+00	2.61	ANPEP, CD36, CLTA, HLA-A, HLA-B, LBP, LRP1, PPIA, RPLP0
1.00E+00	2.10	ANXA11, CALM3, CDC42, CFL1, CLTA, GNAI2, HNRNPU, MYH9, RAB10, RCC2, RHOA, SPTE
1.00E+00	7.06	DSP, JUP, YWHAE
1.00E+00	7.06	CALM3, CANX, FLOT1
1.00E+00	7.06	ATP5F1B, BCAP31, TUBB
1.00E+00	Inf	KRT6A
1.00E+00	Inf	KRT6A
1.00E+00	Inf	KRT6A
1.00E+00	Inf	KRT6A
1.00E+00	Inf	RHOA
1.00E+00	Inf	NANS
1.00E+00	Inf	YWHAH
1.00E+00	Inf	YWHAZ
1.00E+00	Inf	PLEK
1.00E+00	Inf	PLEK
1.00E+00	Inf	PLEK
1.00E+00	Inf	HMBS
1.00E+00	Inf	SPTBN1
1.00E+00	Inf	PLEK
1.00E+00	Inf	KRT2
1.00E+00	Inf	SRGN
1.00E+00	Inf	SRGN

1.00E+00	Inf	SRGN
1.00E+00	Inf	SRGN
1.00E+00	Inf	SRGN
1.00E+00	Inf	SRGN
1.00E+00	Inf	SRGN
1.00E+00	Inf	SRGN
1.00E+00	Inf	SRGN
1.00E+00	Inf	SRGN
1.00E+00	Inf	SRGN
1.00E+00	Inf	GSTP1
1.00E+00	Inf	DHX9
1.00E+00	Inf	TCP1
1.00E+00	Inf	TCP1
1.00E+00	Inf	KRT5
1.00E+00	Inf	LBP
1.00E+00	Inf	ITGB1
1.00E+00	Inf	CCT2
1.00E+00	Inf	ADAM10
1.00E+00	Inf	ACTB
1.00E+00	Inf	PLEK
1.00E+00	Inf	NPM1
1.00E+00	Inf	B2M
1.00E+00	Inf	RCC2
1.00E+00	Inf	ACTB
1.00E+00	Inf	CD36
1.00E+00	Inf	ITGB1
1.00E+00	Inf	PECAM1
1.00E+00	Inf	EEF1A1
1.00E+00	Inf	ITGB3
1.00E+00	Inf	ITGB3
1.00E+00	Inf	ACTB
1.00E+00	Inf	UBQLN1
1.00E+00	Inf	STOM
1.00E+00	Inf	NPM1
1.00E+00	Inf	RAB33B
1.00E+00	Inf	RHOA
1.00E+00	Inf	MYH9
1.00E+00	Inf	MYH9
1.00E+00	Inf	VCL
1.00E+00	Inf	HSPA8
1.00E+00	Inf	RTN4
1.00E+00	Inf	RTN4
1.00E+00	Inf	ANXA2
1.00E+00	Inf	DHX9
1.00E+00	Inf	HNRNPU
1.00E+00	Inf	HSPA8
1.00E+00	Inf	LTF

1.00E+00	Inf	ITGB3
1.00E+00	Inf	ITGB1
1.00E+00	Inf	JUP
1.00E+00	Inf	HNRNPU
1.00E+00	Inf	MMRN1
1.00E+00	Inf	ATIC
1.00E+00	Inf	GALE
1.00E+00	Inf	GALE
1.00E+00	Inf	GSR
1.00E+00	Inf	HMBS
1.00E+00	Inf	ATIC
1.00E+00	Inf	LRP1
1.00E+00	Inf	HNRNPU
1.00E+00	Inf	ADH5
1.00E+00	Inf	FCER1G
1.00E+00	Inf	AACS
1.00E+00	Inf	DHX9
1.00E+00	Inf	GAPDH
1.00E+00	Inf	GSTP1
1.00E+00	Inf	GSTP1
1.00E+00	Inf	CCL5
1.00E+00	Inf	NANS
1.00E+00	Inf	NANS
1.00E+00	Inf	CD36
1.00E+00	Inf	PCYOX1
1.00E+00	Inf	ADH5
1.00E+00	Inf	ADH5
1.00E+00	Inf	CD36
1.00E+00	Inf	HSPA8
1.00E+00	2.59	ANXA2, CSDE1, DHX9, FLNA, HNRNPC, HNRNPU, QSOX1, RPL11, SYNCRIP
1.00E+00	4.91	B2M, CD226, CD36, HLA-A
1.00E+00	4.91	ACTB, CALM3, CANX, RAB27B
1.00E+00	2.09	CFL1, FLNA, GAPDH, HNRNPU, HSPA1B, ITGB1, MYH9, NPM1, PATJ, RHOA, TUBA1C, TUB
1.00E+00	3.02	B2M, CALM3, CD59, CLTA, HLA-A, HLA-B, RAB27B
1.00E+00	14.07	NPM1, RPS15
1.00E+00	14.07	LBP, PRDX2
1.00E+00	14.07	CD226, HSPD1
1.00E+00	14.07	CD226, HSPD1
1.00E+00	14.07	CALM3, SRI
1.00E+00	14.07	NPM1, RPS15
1.00E+00	14.07	CALM3, SRI
1.00E+00	14.07	KRT1, KRT10
1.00E+00	14.07	SLC2A1, XRCC5
1.00E+00	14.07	DSP, JUP
1.00E+00	14.07	CD36, LRP1
1.00E+00	14.07	S100A8, S100A9
1.00E+00	3.00	CDC42, FSCN1, ICAM2, ITGB1, ITGB3, MSN, SPN

1.00E+00	4.84	CANX, CYBB, PKM, RPS23
1.00E+00	6.88	CAMP, H2BC21, LTF
1.00E+00	6.88	CDC42, HNRNPU, RCC2
1.00E+00	6.88	MSN, NASP, THBS1
1.00E+00	2.75	ACTB, ARPC5, CALM3, CFL1, FLNA, FSCN1, HSP90AA1, HSPA8
1.00E+00	2.43	CALM3, FLNA, G6PD, ITGB1, ITGB3, SRI, STOM, UBQLN1, YWHAE, YWHAH
1.00E+00	3.34	ACTB, ADAM10, ITGB1, LRP1, SPTBN1, STOM
1.00E+00	3.34	B2M, BCAP31, CANX, HLA-A, HLA-B, RTN4
1.00E+00	3.88	HSPA1B, HSPA8, HSPA9, SPN, TFRC
1.00E+00	4.78	MYADM, PFN1, SPTBN1, VDAC2
1.00E+00	4.78	EIF5A, HINT1, RPL11, TMEM109
1.00E+00	4.78	ADH5, GSTP1, S100A8, S100A9
1.00E+00	3.32	EIF5A, HINT1, NPM1, RPL11, RPS15, TMEM109
1.00E+00	3.32	ARPC5, CALM3, CFL1, FLNA, FSCN1, HSP90AA1
1.00E+00	3.84	CSDE1, DHX9, HNRNPU, SYNCRIP, TNKS1BP1
1.00E+00	3.84	ACTB, ADAM10, ITGB1, LRP1, SPTBN1
1.00E+00	3.84	CYB5R3, CYBB, LDHA, LDHB, RAC2
1.00E+00	1.39	AACS, ACTB, ALDOA, ANXA2, ATIC, ATP5F1B, CCL5, CCT2, CCT4, CCT6A, CCT7, CCT8, CD3(
1.00E+00	2.54	CALM3, GNAQ, GSTP1, LRP1, MYADM, NPM1, PPIA, PTPRJ, YWHAG
1.00E+00	6.71	FLOT1, ITGB1, ITGB3
1.00E+00	6.71	CCL5, CD9, THBS1
1.00E+00	1.88	ADAM10, CD9, CDC42, ENO1, G6PD, HSP90AA1, HSPA1B, ITGB1, KRT17, NPM1, PTPRJ, R
1.00E+00	4.72	CD36, CD47, CYB5R3, HSP90AA1
1.00E+00	4.72	LRP1, S100A8, S100A9, VIM
1.00E+00	4.72	FSCN1, G3BP1, MSN, RHOA
1.00E+00	4.72	CAVIN2, CD36, FLOT1, SLC2A1
1.00E+00	4.72	FCN1, PSMA5, PSMA6, UBQLN1
1.00E+00	4.72	CD36, LGALS3BP, LRP1, TFRC
1.00E+00	2.95	ILK, LRP1, RHOA, S100A8, S100A9, SOD1, VIM
1.00E+00	2.00	ACTB, B2M, G6PD, HNRNPU, HSPA9, INPP5D, ITGB1, ITGB3, LTF, PRDX2, PTBP1, RCC2, RI
1.00E+00	3.28	CCL5, GNAI2, ITGB3, RHOA, THBS1, XRCC5
1.00E+00	1.70	CCL5, CD47, ENO1, G3BP1, GNAI2, GSTP1, HSPA1B, INPP5D, ITGA6, ITGB1, LRP1, LTF, MY
1.00E+00	3.78	ADAM10, CCL5, LBP, RAC2, THBS1
1.00E+00	3.78	CD36, CDC42, MYH9, RAC2, THBS1
1.00E+00	1.41	ACTB, ALDOA, ANXA2, ATIC, ATP5F1B, CCL5, CCT2, CCT4, CCT6A, CCT7, CCT8, CD36, CD4
1.00E+00	13.07	FSCN1, LRP1
1.00E+00	13.07	ADAM10, CCL5
1.00E+00	13.07	CFL1, MYH9
1.00E+00	13.07	CD53, FLOT1
1.00E+00	13.07	PLEK, SOD1
1.00E+00	13.07	DHX9, HSPD1
1.00E+00	13.07	YWHAG, YWHAH
1.00E+00	13.07	RAB10, RAB27B
1.00E+00	3.26	CD47, CDC42, HSPA1B, PFN1, PLEK, RHOA
1.00E+00	1.87	CDC42, CFL1, FLNA, GAPDH, HNRNPU, HSPA1B, HSPA8, ITGB1, MYH9, NPM1, PATJ, RAB2
1.00E+00	4.66	CALM3, HINT1, PLEK, PTBP1
1.00E+00	4.66	CD36, CD47, CYB5R3, HSP90AA1

1.00E+00	1.66	ACTB, ANXA2, ARL6IP5, ATP2A3, ATP5F1B, CALM3, CD36, CLIC1, CYBB, FLNA, G6PD, GP1
1.00E+00	6.55	DHX9, HNRNPU, PFN1
1.00E+00	6.55	CD53, MYH9, PTPRJ
1.00E+00	6.55	ATP5F1B, HSPA9, VDAC2
1.00E+00	6.55	ATP5F1B, HSPA9, VDAC2
1.00E+00	2.92	ALDOA, ENO1, G6PD, GALE, GAPDH, PGK1, PKM
1.00E+00	2.27	B2M, CD226, FCER1G, HLA-A, HLA-B, HSPD1, INPP5D, PRDX1, RAC2, TFRC, TUBB
1.00E+00	1.39	AACS, ACTB, ALDOA, ANXA2, ATIC, ATP5F1B, CCL5, CCT2, CCT4, CCT6A, CCT7, CCT8, CD3(
1.00E+00	1.58	ACTB, ANXA2, CCL5, CCT2, CCT4, CCT6A, CCT7, CCT8, CD36, CSDE1, DHX9, EEF2, EIF5A, E
1.00E+00	3.24	ATP5F1B, ITGB1, JUP, MYH9, RHOA, THBS1
1.00E+00	3.75	B2M, G6PD, HSPA1B, HSPA9, INPP5D
1.00E+00	3.75	CYBB, G6PD, RHOA, S100A8, SOD1
1.00E+00	1.64	ACTB, CALM3, CYB5R3, CYBB, DHX9, ENO1, FCN1, GNAI2, GNAQ, GNG5, HINT1, HNRNPA
1.00E+00	1.69	CCL5, CD47, ENO1, G3BP1, GNAI2, GSTP1, HSPA1B, INPP5D, ITGA6, ITGB1, LRP1, LTF, MY
1.00E+00	1.54	ANXA2, CALM3, CD36, CSDE1, DHX9, ENO1, FLNA, G6PD, GAPDH, GNAI2, GNAQ, GSTP1,
1.00E+00	4.60	HSPA1B, PPIA, RPL11, RPS15
1.00E+00	1.94	ARL6IP5, CCL5, CD36, CDC42, GNAI2, GSTP1, ITGB3, PPIA, PRDX1, PRDX2, PTPRJ, RAP1B,
1.00E+00	3.22	ADAM10, HNRNPU, ITGB3, NPM1, RCC2, RPS15
1.00E+00	3.72	CD53, CD9, FLOT1, ITGB1, MYH9
1.00E+00	2.09	ATP2A3, CALM3, DSP, ENO1, FLNA, G6PD, GNAI2, HSP90AA1, JUP, RHOA, SOD1, SRI, YWI
1.00E+00	3.20	B2M, CD226, HLA-A, HLA-B, HSPD1, TFRC
1.00E+00	3.20	CCL5, GNAI2, ITGB3, RHOA, THBS1, XRCC5
1.00E+00	4.54	ANXA2, GSDMA, OSBPL2, PFN1
1.00E+00	3.69	FLNA, JUP, MYL12B, SLC2A1, SRI
1.00E+00	1.74	ACTB, ANXA2, ATP2A3, ATP5F1B, B2M, CALM3, CCL5, FLNA, G6PD, GNAI2, GP1BA, GP1B
1.00E+00	2.08	ACTB, DHX9, ENO1, FLNA, HNRNPF, HNRNPU, JUP, NCL, NPM1, PSMA6, SRI, YWHAH, YW
1.00E+00	1.92	ANXA2, BCAP31, CSDE1, DHX9, EEF1A1, GAPDH, HNRNPC, HNRNPU, HSPA1B, NPM1, QS(
1.00E+00	2.34	ANPEP, CD36, CLTA, HLA-A, HLA-B, LBP, LRP1, PPIA, RPLP0, VDAC2
1.00E+00	12.19	LDHA, LDHB
1.00E+00	12.19	FCN1, LBP
1.00E+00	12.19	ADAM10, CCL5
1.00E+00	12.19	HLA-A, HLA-B
1.00E+00	12.19	PLEK, RAP1B
1.00E+00	12.19	RCC2, THBS1
1.00E+00	12.19	ARL6IP5, ITGB1
1.00E+00	12.19	RCC2, THBS1
1.00E+00	12.19	F11R, VCL
1.00E+00	12.19	F11R, VCL
1.00E+00	12.19	ANXA2, MYH9
1.00E+00	12.19	CYBB, RAC2
1.00E+00	12.19	CD36, SLC2A1
1.00E+00	12.19	CD36, THBS1
1.00E+00	12.19	ANXA2, ANXA5
1.00E+00	12.19	HNRNPA2B1, NPM1
1.00E+00	6.25	CAMP, CCL5, FCER1G
1.00E+00	6.25	CD226, HLA-A, HLA-B
1.00E+00	6.25	CD99, LBP, RAC2

1.00E+00	6.25	CD36, LGALS3BP, LRP1
1.00E+00	2.46	CAMP, CYBB, ITGB1, ITGB3, JUP, KRT1, PGK1, PKM, THBS1
1.00E+00	2.62	ACTB, CALM3, ITGB1, SRI, STOM, UBQLN1, YWHAE, YWHAH
1.00E+00	3.63	CD9, CLTA, HSPA8, M6PR, TFRC
1.00E+00	3.15	B2M, CD226, HLA-A, HLA-B, HSPD1, TFRC
1.00E+00	3.15	CALM3, FLNA, GP1BA, GP1BB, ITGB3, SRI
1.00E+00	2.01	ADAM10, B2M, BCAP31, CD59, CDC42, HLA-A, HLA-B, QSOX1, RAB10, RAB1B, RAB33B, R
1.00E+00	4.43	B2M, CD226, HLA-A, HLA-B
1.00E+00	4.43	CCL5, ITGB3, LRP1, RHOA
1.00E+00	3.13	HSP90AA1, MSN, NPM1, PLEK, RHOA, RTN4
1.00E+00	3.60	B2M, CD226, CD36, HLA-A, TFRC
1.00E+00	6.12	GNAI2, YWHAB, YWHAE
1.00E+00	6.12	INPP5D, ITGB3, TFRC
1.00E+00	2.31	ACTB, B2M, HLA-B, HSPA1B, HSPA9, INPP5D, LTF, PRDX2, RHOA, SOD1
1.00E+00	2.30	CCL5, CD47, DHX9, GSTP1, KRT1, LBP, PSMA6, S100A8, S100A9, SOD1
1.00E+00	2.30	ANXA2, B2M, FLOT1, HLA-A, HLA-B, HSPD1, LRP1, LTF, RCC2, TFRC
1.00E+00	1.83	ACTB, CALM3, CDC42, CFL1, FLNA, HNRNPU, HSPA1B, ITGB1, NASP, RCC2, RHOA, SPTBN1
1.00E+00	2.12	ANTXR2, ANXA2, B2M, CLTA, CTSD, HLA-A, HLA-B, ITGB1, RAB10, RAB27B, RCC2, TFRC
1.00E+00	11.43	CD47, CD99
1.00E+00	11.43	ARL6IP5, ITGB1
1.00E+00	11.43	FLOT1, UBQLN1
1.00E+00	11.43	ANXA2, LTF
1.00E+00	11.43	ITGB3, TFRC
1.00E+00	11.43	CYBB, SOD1
1.00E+00	11.43	LBP, RAC2
1.00E+00	11.43	DHX9, FLOT1
1.00E+00	11.43	CDC42, GNAI2
1.00E+00	11.43	ARL6IP5, ITGB1
1.00E+00	11.43	ITGB3, QSOX1
1.00E+00	11.43	ITGB3, QSOX1
1.00E+00	11.43	CD36, LRP1
1.00E+00	11.43	ANXA2, RAB10
1.00E+00	2.58	CYBB, ENO1, PGK1, RHOA, SLC2A1, TFRC, THBS1, UBQLN1
1.00E+00	2.58	B2M, G6PD, HSPA1B, HSPA9, INPP5D, PRDX1, PRDX2, SOD1
1.00E+00	5.98	CCL5, FERMT3, ITGB3
1.00E+00	5.98	CALM3, PPIA, PTBP1
1.00E+00	5.98	ENO1, HSP90AA1, RHOA
1.00E+00	4.33	B2M, HLA-A, HLA-B, HSPD1
1.00E+00	4.33	CALM3, SRI, UBQLN1, YWHAE
1.00E+00	4.33	CAMP, LBP, LTF, LYZ
1.00E+00	2.78	GNAQ, GSTP1, HNRNPU, NPM1, PPIA, PTPRJ, YWHAG
1.00E+00	3.08	HSP90AA1, HSPA1B, HSPD1, SOD1, THBS1, YWHAE
1.00E+00	2.10	B2M, CD36, FLNA, GP1BA, GP1BB, HSPD1, ITGA2B, ITGA6, ITGB1, ITGB3, LRP1, TFRC
1.00E+00	2.77	B2M, CD226, HLA-A, HLA-B, HSPD1, RAC2, TFRC
1.00E+00	1.73	ANXA2, ARL6IP5, ATP2A3, ATP5F1B, CALM3, CLIC1, CYBB, FLNA, G6PD, GP1BA, GP1BB, IT
1.00E+00	4.28	CCL5, GNAQ, LRP1, RHOA
1.00E+00	4.28	CD9, CLTA, M6PR, TFRC

1.00E+00	4.28	CANX, HNRNPU, RAB27B, SOD1
1.00E+00	2.27	ACTB, B2M, FCER1G, INPP5D, ITGB1, PRDX2, PTPRJ, RHOA, SOD1, SPN
1.00E+00	2.76	CALM3, GNAI2, MYH9, PLEK, RAB10, RAC2, RAP1B
1.00E+00	1.88	ACTB, ARPC5, ATIC, B2M, CALM3, CDC42, G6PD, ITGB1, RHOA, RTN3, RTN4, SLC2A1, TM:
1.00E+00	5.85	GSTP1, PNPLA8, PTGS1
1.00E+00	5.85	GSTP1, PNPLA8, PTGS1
1.00E+00	1.57	ACTB, ANXA2, CCL5, CCT2, CCT4, CCT6A, CCT7, CCT8, CSDE1, DHX9, EEF2, EIF5A, HNRNPU
1.00E+00	3.04	CALM3, G6PD, ITGB3, SRI, UBQLN1, YWHAE
1.00E+00	2.27	CALM3, CCL5, HSP90AA1, ITGB3, LTF, NPM1, PPIA, RHOA, THBS1, XRCC5
1.00E+00	2.17	ADAM10, CFL1, EIF5A, FLOT1, FSCN1, HSPA8, LRBA, LRP1, PLEK, SPTBN1, UBQLN1
1.00E+00	1.55	ACTB, ANXA2, CCL5, CCT2, CCT4, CCT6A, CCT7, CCT8, CD36, CSDE1, DHX9, EEF2, EIF5A, E
1.00E+00	1.36	AACS, ACTB, ALDOA, ANXA2, ATIC, ATP5F1B, CCL5, CCT2, CCT4, CCT6A, CCT7, CCT8, CD3(
1.00E+00	4.23	CCL5, FCN1, LTF, PPIA
1.00E+00	2.38	ANXA11, B2M, CALM3, CYBB, G6PD, S100A8, SOD1, TFRC, THBS1
1.00E+00	3.03	BCAP31, HSP90AA1, HSPD1, RAC2, UBE2L3, VDAC2
1.00E+00	3.03	CD36, FLNA, GAPDH, LTF, RHOA, TFRC
1.00E+00	10.76	G6PD, TKT
1.00E+00	10.76	GNAI2, GNAQ
1.00E+00	10.76	CCL5, THBS1
1.00E+00	10.76	RAC2, THBS1
1.00E+00	10.76	HLA-A, HLA-B
1.00E+00	10.76	ITGA6, ITGB3
1.00E+00	2.25	B2M, CALM3, CD59, CLTA, HLA-A, HLA-B, M6PR, RAB10, RAB1B, RAB27B
1.00E+00	5.73	B2M, HLA-A, HLA-B
1.00E+00	5.73	CD226, HLA-A, HLA-B
1.00E+00	5.73	DHX9, HNRNPA2B1, MATR3
1.00E+00	4.18	HSP90AA1, HSPA1B, PFN1, RHOA
1.00E+00	4.18	PFN1, YWHAB, YWHAE, YWHAZ
1.00E+00	3.44	CCL5, GNAI2, ITGB3, PLEK, YWHAB
1.00E+00	3.44	ANXA2, FLOT1, ITGB1, ITGB3, SRI
1.00E+00	2.51	CLTA, ENO1, FLNA, HSP90AA1, PFN1, RCC2, SOD1, SPTBN1
1.00E+00	3.41	ARPC5, MYADM, PFN1, RHOA, SPTBN1
1.00E+00	5.62	CD47, CDC42, RHOA
1.00E+00	5.62	HSPA8, M6PR, RTN4
1.00E+00	1.78	ACTB, B2M, CDC42, CFL1, FLNA, FLOT1, HSP90AA1, ITGA6, ITGB1, RAB10, RAC2, RHOA, R
1.00E+00	2.22	CSDE1, DHX9, HINT1, HNRNPC, HNRNPU, HSPA1B, NPM1, SYNCRIP, TNKS1BP1, VIM
1.00E+00	1.99	AACS, ANXA2, CALM3, CCL5, GNAI2, PPIA, RAB10, RAB27B, RAB33B, RAC2, RAP1B, S100/
1.00E+00	10.16	HLA-A, HLA-B
1.00E+00	10.16	CD226, HSPD1
1.00E+00	10.16	PRDX2, RAC2
1.00E+00	10.16	ANK1, ARPC5
1.00E+00	10.16	HNRNPC, HNRNPU
1.00E+00	10.16	CD36, LRP1
1.00E+00	10.16	KRT16, KRT2
1.00E+00	10.16	HLA-A, HLA-B
1.00E+00	10.16	BCAP31, UBQLN1
1.00E+00	10.16	PSMA5, PSMA6

1.00E+00	10.16	CD59, GP1BA
1.00E+00	2.48	CDC42, F11R, FLNA, ILK, ITGB1, ITGB3, MYADM, XRCC5
1.00E+00	3.36	B2M, FLNA, RHOA, RTN4, VIM
1.00E+00	3.36	CALM3, CPN1, HNRNPU, RHOA, UBE2L3
1.00E+00	2.95	B2M, FLNA, RHOA, RTN4, VIM, YWHAH
1.00E+00	1.49	ANXA2, CALM3, CD36, CSDE1, DHX9, ENO1, FLNA, G6PD, GAPDH, GNAI2, GNAQ, GSTP1,
1.00E+00	5.50	G6PD, GAPDH, GSR
1.00E+00	4.04	B2M, CD59, HLA-A, HLA-B
1.00E+00	2.93	CD47, CDC42, HSPA1B, PFN1, PLEK, RHOA
1.00E+00	1.84	CALM3, CCT4, CCT8, CDC42, DHX9, FLOT1, G6PD, GNAI2, HNRNPU, HSPA1B, MYH16, NPI
1.00E+00	2.46	CYBB, ENO1, PGK1, RHOA, SLC2A1, TFRC, THBS1, UBQLN1
1.00E+00	1.48	ACTB, ANXA2, ARPC5, ATIC, B2M, CALM3, CD9, CDC42, CFL1, FLNA, FLOT1, G6PD, GSTP1
1.00E+00	2.20	ACTB, ADAM10, CDC42, CFL1, FLNA, HSPA8, PFN1, RHOA, TUBB, YWHAZ
1.00E+00	2.11	CALM3, CCL5, CDC42, HSP90AA1, ITGB3, LTF, NPM1, PPIA, RHOA, THBS1, XRCC5
1.00E+00	3.31	ADAM10, CCL5, LBP, RAC2, THBS1
1.00E+00	4.00	HSPA1B, PPIA, RPL11, RPS15
1.00E+00	5.39	CFL1, ITGB1, MYH9
1.00E+00	5.39	DSP, FLNA, JUP
1.00E+00	2.03	ACTB, CDC42, CFL1, FLOT1, HSP90AA1, ITGB1, RAB10, RAC2, RTN4, SOD1, VCL, YWHAH
1.00E+00	2.19	CD36, CDC42, DSG1, HSP90AA1, ITGB3, MSN, MYL12B, PATJ, RAB27B, SLC2A1
1.00E+00	2.29	CD36, GNAI2, ITGB3, LRP1, NCL, PKM, RAB10, VIM, YWHAG
1.00E+00	91.12	CD59
1.00E+00	91.12	ARL6IP5
1.00E+00	91.12	B2M
1.00E+00	91.12	HLA-A
1.00E+00	91.12	YWHAE
1.00E+00	91.12	NPM1
1.00E+00	91.12	G6PD
1.00E+00	91.12	G6PD
1.00E+00	91.12	G6PD
1.00E+00	91.12	LBP
1.00E+00	91.12	G6PD
1.00E+00	91.12	PCYOX1
1.00E+00	91.12	PCYOX1
1.00E+00	91.12	MYH9
1.00E+00	91.12	ANXA2
1.00E+00	91.12	SRGN
1.00E+00	91.12	HSPD1
1.00E+00	91.12	F11R
1.00E+00	91.12	ADAM10
1.00E+00	91.12	LTF
1.00E+00	91.12	LTF
1.00E+00	91.12	LTF
1.00E+00	91.12	INPP5D
1.00E+00	91.12	TKT
1.00E+00	91.12	NANS
1.00E+00	91.12	SELPLG

1.00E+00	91.12	ACTB
1.00E+00	91.12	ITGB1
1.00E+00	91.12	ITGB1
1.00E+00	91.12	LBP
1.00E+00	91.12	MYH9
1.00E+00	91.12	PPIA
1.00E+00	91.12	G3BP1
1.00E+00	91.12	MSN
1.00E+00	91.12	PLEK
1.00E+00	91.12	CD36
1.00E+00	91.12	SOD1
1.00E+00	91.12	RHOA
1.00E+00	91.12	LTF
1.00E+00	91.12	LTF
1.00E+00	91.12	LTF
1.00E+00	91.12	LTF
1.00E+00	91.12	LTF
1.00E+00	91.12	LTF
1.00E+00	91.12	MYH9
1.00E+00	91.12	RHOA
1.00E+00	91.12	HNRNPU
1.00E+00	91.12	RAB33B
1.00E+00	91.12	ATP2A3
1.00E+00	91.12	MYH9
1.00E+00	91.12	MYH9
1.00E+00	91.12	LRP1
1.00E+00	91.12	LRP1
1.00E+00	91.12	RPLP0
1.00E+00	91.12	B2M
1.00E+00	91.12	B2M
1.00E+00	91.12	B2M
1.00E+00	91.12	B2M
1.00E+00	91.12	RHOA
1.00E+00	91.12	FLNA
1.00E+00	91.12	CD47
1.00E+00	91.12	RTN4
1.00E+00	91.12	ANXA2
1.00E+00	91.12	HNRNPA2B1
1.00E+00	91.12	DHX9
1.00E+00	91.12	RPS23
1.00E+00	91.12	RAB33B
1.00E+00	91.12	CD36
1.00E+00	91.12	CD36
1.00E+00	91.12	KRT6A
1.00E+00	91.12	HLA-A
1.00E+00	91.12	HLA-A
1.00E+00	91.12	CDC42

1.00E+00	91.12	ATP5F1B
1.00E+00	91.12	ITGB1
1.00E+00	91.12	ITGB1
1.00E+00	91.12	ITGB1
1.00E+00	91.12	ITGB1
1.00E+00	91.12	ITGB1
1.00E+00	91.12	ITGB1
1.00E+00	91.12	XRCC5
1.00E+00	91.12	ATP5F1B
1.00E+00	91.12	ITGB3
1.00E+00	91.12	GSTP1
1.00E+00	91.12	HSPA8
1.00E+00	91.12	CLTA
1.00E+00	91.12	ANXA2
1.00E+00	91.12	ANXA2
1.00E+00	91.12	DHX9
1.00E+00	91.12	HNRNPU
1.00E+00	91.12	PCYOX1
1.00E+00	91.12	HSP90AA1
1.00E+00	91.12	G6PD
1.00E+00	91.12	GAPDH
1.00E+00	91.12	PGK1
1.00E+00	91.12	PTGS1
1.00E+00	91.12	PKM
1.00E+00	91.12	TFRC
1.00E+00	91.12	RPSA
1.00E+00	91.12	NANS
1.00E+00	91.12	ATP2A3
1.00E+00	91.12	CCL5
1.00E+00	91.12	CDC42
1.00E+00	91.12	HSP90AA1
1.00E+00	91.12	GAPDH
1.00E+00	91.12	DHX9
1.00E+00	91.12	ADH5
1.00E+00	91.12	HLA-A
1.00E+00	91.12	THBS1
1.00E+00	91.12	TTLL12
1.00E+00	9.63	CD226, HSPD1
1.00E+00	9.63	GSTP1, PNPLA8
1.00E+00	9.63	CD36, LRP1
1.00E+00	9.63	ADAM10, CCL5
1.00E+00	9.63	CD53, FLOT1
1.00E+00	9.63	LGALS3BP, RAB27B
1.00E+00	2.88	B2M, CD226, HLA-A, HLA-B, HSPD1, TFRC
1.00E+00	2.02	AACS, ANXA2, CALM3, CCL5, GNAI2, PPIA, RAB10, RAB27B, RAB33B, RAC2, RAP1B, SRI
1.00E+00	3.27	CDC42, MYADM, PFN1, SPTBN1, VDAC2
1.00E+00	5.29	EEF1A1, EEF2, EIF5A

1.00E+00	5.29	CD47, FCER1G, PTPRJ
1.00E+00	5.29	GNAI2, GNG5, ITGB1
1.00E+00	2.61	CD36, FLNA, GAPDH, GSTP1, LTF, RHOA, TFRC
1.00E+00	2.87	CDC42, FSCN1, HSP90AA1, PFN1, RAC2, RCC2
1.00E+00	2.42	BCAP31, CALM3, CD36, HINT1, PLEK, PTBP1, PTPRJ, THBS1
1.00E+00	2.28	B2M, CD226, FCER1G, HLA-A, HLA-B, HSPD1, INPP5D, SPN, TFRC
1.00E+00	3.91	CCL5, GNAI2, ITGB3, THBS1
1.00E+00	1.58	ADAM10, B2M, BCAP31, CD36, CD59, CDC42, CLTA, CSDE1, CYBB, FLNA, GNAQ, HLA-A, H
1.00E+00	2.41	ANXA2, FCN1, GNAI2, GNAQ, GNG5, JUP, OSBPL2, RHOA
1.00E+00	2.84	CDC42, FSCN1, HSP90AA1, PFN1, RAC2, RCC2
1.00E+00	3.87	DHX9, G3BP1, HNRNPA2B1, XRCC5
1.00E+00	3.87	CD36, DHX9, HSPD1, LBP
1.00E+00	5.19	DHX9, FLOT1, NPM1
1.00E+00	5.19	RPL11, RPS15, UBE2L3
1.00E+00	2.58	CSDE1, DHX9, GAPDH, HNRNPU, NCL, SYNCRIP, TNKS1BP1
1.00E+00	2.83	ARL6IP5, CD36, ITGB1, SLC2A1, SLC2A3, THBS1
1.00E+00	9.14	G6PD, TKT
1.00E+00	9.14	ITGA6, KRT6B
1.00E+00	9.14	ARL6IP5, THBS1
1.00E+00	9.14	CCL5, ITGB3
1.00E+00	9.14	BCAP31, PTPRJ
1.00E+00	9.14	RPL11, RPS15
1.00E+00	9.14	HNRNPC, HNRNPU
1.00E+00	9.14	CFL1, ITGB3
1.00E+00	9.14	GSTP1, PRDX6
1.00E+00	9.14	GNAI2, GNAQ
1.00E+00	9.14	CANX, FLOT1
1.00E+00	9.14	HNRNPC, HNRNPU
1.00E+00	2.39	ADAM10, ANXA2, FLOT1, FSCN1, ITGB1, ITGB3, LRP1, QSOX1
1.00E+00	3.20	ANK1, HSP90AA1, JUP, SOD1, YWHAE
1.00E+00	3.83	ANXA2, INPP5D, LTF, TFRC
1.00E+00	3.83	HSPA1B, HSPA8, HSPA9, HSPD1
1.00E+00	3.83	ARL6IP5, PPIA, SOD1, UBQLN1
1.00E+00	3.83	HNRNPU, NCL, TFRC, XRCC5
1.00E+00	2.38	ADAM10, ANXA2, FLOT1, FSCN1, ITGB1, ITGB3, LRP1, QSOX1
1.00E+00	5.09	DHX9, FLOT1, HSP90AA1
1.00E+00	5.09	DHX9, FLOT1, HSP90AA1
1.00E+00	5.09	FSCN1, ITGB3, LRP1
1.00E+00	5.09	HNRNPA2B1, HNRNPU, PTBP1
1.00E+00	2.06	ARPC5, HSP90AA1, MSN, MYADM, NPM1, PFN1, PLEK, RHOA, RTN4, SOD1, SPTBN1
1.00E+00	2.06	CD36, CYBB, GNAI2, ITGB3, LRP1, NCL, PKM, RAB10, SLC2A1, VIM, YWHAG
1.00E+00	3.79	CD36, PNPLA8, SLC2A1, THBS1
1.00E+00	3.79	ITGB1, LRP1, SPTBN1, STOM
1.00E+00	3.79	HNRNPU, NCL, TFRC, XRCC5
1.00E+00	2.55	CALM3, GNAI2, PPIA, PTBP1, PTPRJ, YWHAB, YWHAE
1.00E+00	3.15	ACTB, F11R, JUP, PATJ, RHOA
1.00E+00	5.00	CYB5R3, G6PD, SOD1

1.00E+00	5.00	B2M, LTF, TFRC
1.00E+00	5.00	PFN1, PLEK, RCC2
1.00E+00	5.00	ITGB3, LRP1, PTPRJ
1.00E+00	5.00	CYB5R3, G6PD, SOD1
1.00E+00	3.75	CALM3, SRI, UBQLN1, YWHAE
1.00E+00	8.71	RHOA, SPN
1.00E+00	8.71	DHX9, HSPD1
1.00E+00	8.71	HNRNPU, HSPA1B
1.00E+00	8.71	FLNA, HSP90AA1
1.00E+00	8.71	HSP90AA1, LTF
1.00E+00	8.71	ANK1, JUP
1.00E+00	8.71	ITGB3, THBS1
1.00E+00	3.71	ACTB, ITGB1, LRP1, SPTBN1
1.00E+00	1.75	AACS, ADH5, ALDOA, ATIC, CD36, ENO1, GAPDH, GSTP1, LDHA, LDHB, OSBPL2, PGK1, PKI
1.00E+00	1.75	ANXA2, ATP2A3, ATP5F1B, CALM3, CLIC1, FLNA, G6PD, GP1BA, GP1BB, ITGB1, ITGB3, PC
1.00E+00	4.91	ANXA2, FLOT1, ITGB3
1.00E+00	4.91	CFL1, PLEK, SPTBN1
1.00E+00	4.91	FCN1, KRT6A, LTF
1.00E+00	4.91	ALDOA, B2M, OSBPL2
1.00E+00	4.91	CFL1, ITGB1, MYH9
1.00E+00	4.91	ANXA2, FLOT1, SPTBN1
1.00E+00	4.91	ITGB1, LRP1, SPTBN1
1.00E+00	4.91	PSMA5, PSMA6, UBQLN1
1.00E+00	4.91	RPL11, RPL8, RPLP0
1.00E+00	4.91	GSTP1, HMBS, NANS
1.00E+00	4.91	DHX9, HNRNPU, STOM
1.00E+00	2.74	BCAP31, CALM3, HINT1, PLEK, PTBP1, PTPRJ
1.00E+00	2.02	CSDE1, DHX9, HINT1, HNRNPC, HNRNPU, HSPA1B, NPM1, SYNCRIP, TNKS1BP1, VIM, YWI
1.00E+00	3.09	CD47, DHX9, LBP, S100A8, S100A9
1.00E+00	2.33	ACTB, CALM3, ITGB1, SRI, STOM, UBQLN1, YWHAE, YWHAH
1.00E+00	2.73	BCAP31, HSP90AA1, HSPA1B, LRP1, MSN, UBQLN1
1.00E+00	2.73	HNRNPU, HSPA1B, HSPA8, RHOA, UBE2L3, YWHAH
1.00E+00	2.01	ACTB, CALM3, CANX, CLTA, FLOT1, HSPA8, RAB10, RAB27B, RAB33B, SLC2A1, YWHAG
1.00E+00	3.07	ARL6IP5, CD36, ITGB1, SLC2A1, THBS1
1.00E+00	3.07	GSTP1, HSPA1B, ITGA6, PRDX2, THBS1
1.00E+00	4.83	CCL5, CD9, THBS1
1.00E+00	8.31	LRP1, PTPRJ
1.00E+00	8.31	CD53, FLOT1
1.00E+00	8.31	RPL11, RPS15
1.00E+00	8.31	CD59, GP1BA
1.00E+00	8.31	RAB10, RTN4
1.00E+00	8.31	SLC2A1, SLC2A3
1.00E+00	8.31	HNRNPU, SYNCRIP
1.00E+00	8.31	SLC2A1, SLC2A3
1.00E+00	3.64	EIF5A, NPM1, RPL11, RPS15
1.00E+00	3.05	ARL6IP5, CD36, ITGB1, SLC2A1, THBS1
1.00E+00	3.05	GALE, HSPD1, ITGB3, PPIA, QSOX1

1.00E+00	1.52	AACS, ADH5, ALDOA, ATIC, ATP5F1B, CD36, CYB5R3, ENO1, G6PD, GALE, GAPDH, GSTP1,
1.00E+00	1.84	ACTB, ARL6IP5, BCAP31, CD36, DHX9, ENO1, FLNA, GSTP1, NPM1, PNPLA8, PPIA, PRDX1,
1.00E+00	2.47	CCL5, GSTP1, HLA-A, INPP5D, RAC2, SPN, TFRC
1.00E+00	1.37	ACTB, ADAM10, ADH5, CALM3, CAMP, CCL5, CD36, CDC42, DSP, FLNA, FLOT1, G6PD, GA
1.00E+00	3.60	DHX9, G3BP1, HNRNPA2B1, XRCC5
1.00E+00	3.60	CALM3, EIF5A, HSPA1B, NPM1
1.00E+00	2.46	GSTP1, LRP1, LTF, PF4V1, PPIA, THBS1, TKT
1.00E+00	2.69	ACTB, CALM3, CANX, ITGB3, RAB27B, RAP1B
1.00E+00	1.69	ANXA2, BCAP31, CANX, CPN1, CTSD, EEF1A1, FLNA, HSP90AA1, HSPA1B, HSPA8, LRP1, M
1.00E+00	1.49	ANXA2, ANXA5, ATP2A3, CALM3, CCL5, CDC42, FLNA, GAPDH, GNAQ, GSTP1, HSP90AA1,
1.00E+00	2.67	ANXA2, CTSD, LRBA, RAB1B, RAB33B, UBQLN1
1.00E+00	2.16	ANXA2, ANXA5, GAPDH, LTF, NPM1, RPL11, RPS15, YWHAB, YWHAG
1.00E+00	1.92	ALDOA, ENO1, G6PD, GALE, GAPDH, LDHA, NANS, PGK1, PKM, PLEK, SLC2A1, SLC2A3
1.00E+00	7.95	HLA-A, HLA-B
1.00E+00	7.95	LBP, LYZ
1.00E+00	7.95	FLOT1, MYADM
1.00E+00	7.95	SOD1, SRI
1.00E+00	7.95	ALDOA, SOD1
1.00E+00	7.95	HNRNPU, RCC2
1.00E+00	7.95	CALM3, SRI
1.00E+00	7.95	LBP, RAC2
1.00E+00	7.95	GP1BA, RHOA
1.00E+00	7.95	CD36, THBS1
1.00E+00	4.66	HSPD1, LBP, THBS1
1.00E+00	4.66	CD36, CD47, HSP90AA1
1.00E+00	1.92	GNAI2, HNRNPU, HSPA1B, HSPA8, ITGB3, NCL, PKM, RAB10, RHOA, UBE2L3, YWHAG, YW
1.00E+00	1.98	BCAP31, CSDE1, DHX9, HNRNPU, HSP90AA1, HSPA1B, LRP1, MSN, SYNCRIP, TNKS1BP1, U
1.00E+00	2.65	B2M, CD226, FCER1G, HSPD1, INPP5D, TFRC
1.00E+00	2.64	GNAQ, GSTP1, NPM1, PPIA, PTPRJ, YWHAG
1.00E+00	1.55	ACTB, ARPC5, B2M, CDC42, CFL1, FLNA, FLOT1, FSCN1, HSP90AA1, ILK, ITGA6, ITGB1, MY
1.00E+00	4.58	FCN1, HSPA1B, LBP
1.00E+00	4.58	ANXA2, FLOT1, SPTBN1
1.00E+00	4.58	INPP5D, ITGB3, TFRC
1.00E+00	4.58	CALM3, SRI, UBQLN1
1.00E+00	3.50	EIF5A, GSTP1, HSPA1B, ILK
1.00E+00	3.50	CALM3, SRI, UBQLN1, YWHAE
1.00E+00	3.50	ANXA2, GSDMA, OSBPL2, PFN1
1.00E+00	2.13	ACTB, B2M, CCL5, CD47, HLA-A, HSPD1, INPP5D, RHOA, TFRC
1.00E+00	2.25	CYBB, ENO1, PGK1, RHOA, SLC2A1, TFRC, THBS1, UBQLN1
1.00E+00	45.56	CD59
1.00E+00	45.56	CD59
1.00E+00	45.56	THBS1
1.00E+00	45.56	THBS1
1.00E+00	45.56	CD36
1.00E+00	45.56	SPTBN1
1.00E+00	45.56	ARPC5
1.00E+00	45.56	ITGB1

1.00E+00	45.56	PCYOX1
1.00E+00	45.56	ITGB3
1.00E+00	45.56	FLOT1
1.00E+00	45.56	FLOT1
1.00E+00	45.56	GSTP1
1.00E+00	45.56	UBQLN1
1.00E+00	45.56	XRCC5
1.00E+00	45.56	HRNR
1.00E+00	45.56	HSP90AA1
1.00E+00	45.56	RHOA
1.00E+00	45.56	HSPD1
1.00E+00	45.56	KRT5
1.00E+00	45.56	RAB10
1.00E+00	45.56	RAB10
1.00E+00	45.56	INPP5D
1.00E+00	45.56	ADH5
1.00E+00	45.56	DHX9
1.00E+00	45.56	CD36
1.00E+00	45.56	CD36
1.00E+00	45.56	HSPA8
1.00E+00	45.56	HSPA8
1.00E+00	45.56	CD36
1.00E+00	45.56	CSDE1
1.00E+00	45.56	LTF
1.00E+00	45.56	CDC42
1.00E+00	45.56	RAB10
1.00E+00	45.56	YWHAE
1.00E+00	45.56	GNAI2
1.00E+00	45.56	RPLP0
1.00E+00	45.56	XRCC5
1.00E+00	45.56	RPLP0
1.00E+00	45.56	HSPA8
1.00E+00	45.56	CD47
1.00E+00	45.56	CYBB
1.00E+00	45.56	G6PD
1.00E+00	45.56	DHX9
1.00E+00	45.56	DHX9
1.00E+00	45.56	PTPRJ
1.00E+00	45.56	RTN4
1.00E+00	45.56	ANXA2
1.00E+00	45.56	HNRNPA2B1
1.00E+00	45.56	ANXA2
1.00E+00	45.56	HNRNPA2B1
1.00E+00	45.56	LTF
1.00E+00	45.56	ITGB3
1.00E+00	45.56	ANXA2
1.00E+00	45.56	B2M

1.00E+00	45.56	FLNA
1.00E+00	45.56	B2M
1.00E+00	45.56	FCER1G
1.00E+00	45.56	FCER1G
1.00E+00	45.56	ITGB1
1.00E+00	45.56	ITGB1
1.00E+00	45.56	ITGB3
1.00E+00	45.56	ITGB3
1.00E+00	45.56	ITGB3
1.00E+00	45.56	SRGN
1.00E+00	45.56	FSCN1
1.00E+00	45.56	XRCC5
1.00E+00	45.56	ITGB3
1.00E+00	45.56	ANXA5
1.00E+00	45.56	MYH9
1.00E+00	45.56	CYB5R3
1.00E+00	45.56	HSP90AA1
1.00E+00	45.56	ALDOA
1.00E+00	45.56	TKT
1.00E+00	45.56	YWHAG
1.00E+00	45.56	CD36
1.00E+00	45.56	PGK1
1.00E+00	45.56	QSOX1
1.00E+00	45.56	HSP90AA1
1.00E+00	45.56	LRP1
1.00E+00	45.56	CALM3
1.00E+00	45.56	CALM3
1.00E+00	45.56	HSP90AA1
1.00E+00	45.56	FLNA
1.00E+00	45.56	HLA-A
1.00E+00	45.56	XRCC5
1.00E+00	45.56	ADAM10
1.00E+00	45.56	DHX9
1.00E+00	7.62	RPL11, RPLP0
1.00E+00	7.62	CD47, PTPRJ
1.00E+00	7.62	B2M, HLA-A
1.00E+00	7.62	LTF, S100A9
1.00E+00	7.62	CD47, PTPRJ
1.00E+00	7.62	LTF, RHOA
1.00E+00	7.62	CALM3, SRI
1.00E+00	7.62	FLNA, YWHAE
1.00E+00	7.62	GNAI2, RAP1B
1.00E+00	7.62	FCN1, PPIA
1.00E+00	7.62	CCL5, THBS1
1.00E+00	7.62	ADAM10, VDAC2
1.00E+00	2.61	ANXA2, CDC42, INPP5D, LTF, MYH9, TFRC
1.00E+00	2.61	B2M, CD226, FCER1G, HSPD1, INPP5D, TFRC

1.00E+00	2.03	CSDE1, DHX9, HINT1, HNRNPC, HNRNPU, HSPA1B, NPM1, SYNCRIP, TNKS1BP1, VIM
1.00E+00	2.91	FLNA, HNRNPU, HSPA1B, ITGB1, RHOA
1.00E+00	1.69	AACS, ADH5, ALDOA, ATIC, CD36, ENO1, GAPDH, GSTP1, LDHA, LDHB, OSBPL2, PGK1, PKI
1.00E+00	4.44	CYB5R3, G6PD, SOD1
1.00E+00	4.44	CD36, CD47, HSP90AA1
1.00E+00	3.40	ANXA2, B2M, FLOT1, ITGB3
1.00E+00	7.31	CALM3, TNKS1BP1
1.00E+00	7.31	LBP, LTF
1.00E+00	7.31	KRT1, KRT10
1.00E+00	7.31	DHX9, GSDMA
1.00E+00	7.31	ADAM10, CCL5
1.00E+00	7.31	HNRNPC, HNRNPU
1.00E+00	7.31	HNRNPU, HSPA9
1.00E+00	7.31	SLC2A1, SLC2A3
1.00E+00	7.31	CD36, FCN1
1.00E+00	2.88	F11R, MYADM, NPM1, PFN1, SPTBN1
1.00E+00	2.36	CALM3, ITGB1, SRI, STOM, UBQLN1, YWHAE, YWHAH
1.00E+00	2.36	ATP5F1B, ITGB1, ITGB3, JUP, MYH9, RHOA, THBS1
1.00E+00	3.37	B2M, HLA-A, HLA-B, HSPD1
1.00E+00	1.68	AACS, ADH5, ALDOA, ATIC, CD36, ENO1, GAPDH, GSTP1, LDHA, LDHB, OSBPL2, PGK1, PKI
1.00E+00	4.36	SOD1, SRI, TFRC
1.00E+00	4.36	CCL5, GNAQ, LRP1
1.00E+00	4.36	RCC2, RHOA, THBS1
1.00E+00	4.36	GNAI2, MYH9, RAP1B
1.00E+00	4.36	FLNA, GP1BA, GP1BB
1.00E+00	4.36	MYADM, PFN1, SPTBN1
1.00E+00	4.36	ADAM10, ANPEP, CPN1
1.00E+00	1.63	ACTB, B2M, CDC42, CFL1, FLNA, FLOT1, HSP90AA1, ITGA6, ITGB1, RAB10, RAC2, RHOA, R
1.00E+00	2.86	ACTB, ADAM10, CDC42, CFL1, HSPA8
1.00E+00	2.00	CSDE1, DHX9, HINT1, HNRNPC, HNRNPU, HSPA1B, NPM1, SYNCRIP, TNKS1BP1, VIM
1.00E+00	2.08	CCL5, CFL1, DDX60L, DHX9, EIF5A, ENO1, G3BP1, HSP90AA1, RPS15A
1.00E+00	2.34	CSDE1, DHX9, GAPDH, HNRNPU, NCL, SYNCRIP, TNKS1BP1
1.00E+00	3.34	GSTP1, LBP, S100A8, TFRC
1.00E+00	3.34	HSPA9, INPP5D, LTF, PRDX2
1.00E+00	2.84	ARL6IP5, CALM3, SRI, UBQLN1, YWHAE
1.00E+00	1.99	ACTB, DHX9, FLNA, JUP, NCL, NPM1, PSMA6, SRI, YWHAH, YWHAZ
1.00E+00	4.30	ITGB1, LRP1, SPTBN1
1.00E+00	7.03	SLC2A1, XRCC5
1.00E+00	7.03	HNRNPU, KANSL3
1.00E+00	7.03	CCL5, THBS1
1.00E+00	7.03	ADAM10, CCL5
1.00E+00	7.03	HLA-A, HLA-B
1.00E+00	7.03	G6PD, TKT
1.00E+00	7.03	LBP, RAC2
1.00E+00	7.03	ARL6IP5, ITGB1
1.00E+00	7.03	ITGB3, MSN
1.00E+00	7.03	JUP, MYH9

1.00E+00	2.82	CDC42, HNRNPU, HSPA8, RAB27B, SOD1
1.00E+00	2.53	ACTB, H2AC20, H2BC21, JUP, NPM1, XRCC5
1.00E+00	3.31	CALM3, SRI, UBQLN1, YWHAE
1.00E+00	3.31	NPM1, RPL11, RPL8, RPLP0
1.00E+00	2.81	CANX, KRT14, KRT16, SOD1, TFRC
1.00E+00	2.81	F11R, HSPA8, MYADM, PFN1, SPTBN1
1.00E+00	4.23	ADAM10, RTN3, RTN4
1.00E+00	4.23	CCL5, GNAQ, RHOA
1.00E+00	2.79	ARPC5, MYADM, PFN1, RHOA, SPTBN1
1.00E+00	1.45	ACTB, ANXA2, CCT2, CCT4, CCT6A, CCT7, CCT8, CSDE1, DAZAP1, DHX9, EIF5A, ENO1, HNI
1.00E+00	2.30	CD36, FLNA, GAPDH, GSTP1, LTF, RHOA, TFRC
1.00E+00	1.79	ACTB, CD53, CD9, DSP, FLOT1, G6PD, HNRNPU, ITGB1, MYH9, MYL6, PTBP1, RHOA, SRI
1.00E+00	6.77	HSPA8, M6PR
1.00E+00	6.77	CD36, THBS1
1.00E+00	6.77	CALM3, TNKS1BP1
1.00E+00	6.77	ARL6IP5, ITGB1
1.00E+00	6.77	ILK, SOD1
1.00E+00	6.77	ILK, SOD1
1.00E+00	6.77	ANXA2, OSBPL2
1.00E+00	6.77	DHX9, HSPD1
1.00E+00	6.77	DHX9, HSPD1
1.00E+00	6.77	ARL6IP5, ITGB1
1.00E+00	6.77	PFN1, YWHAZ
1.00E+00	6.77	LTF, RHOA
1.00E+00	6.77	ITGB3, PECAM1
1.00E+00	6.77	GNAI2, GNAQ
1.00E+00	6.77	RTN3, RTN4
1.00E+00	6.77	SLC2A1, SLC2A3
1.00E+00	4.17	FCN1, FLNA, VCL
1.00E+00	2.29	ACTB, CALM3, CCL5, FLNA, G6PD, ITGB1, SRI
1.00E+00	2.77	ADH5, FCN1, GSTP1, S100A8, S100A9
1.00E+00	2.15	CDC42, FLNA, FSCN1, HSP90AA1, ITGA6, PFN1, RAC2, S100A9
1.00E+00	1.83	ADAM10, CANX, CDC42, CYBB, GNAI2, HNRNPU, HSP90AA1, HSPA8, ITGB1, RHOA, RPLP(
1.00E+00	1.52	ACTB, ARPC5, B2M, CDC42, CFL1, FLNA, FLOT1, FSCN1, HSP90AA1, ITGA6, ITGB1, MYH9,
1.00E+00	1.63	ACTB, CCT2, CCT4, CCT6A, CCT7, CCT8, DHX9, HNRNPA2B1, HNRNPC, HNRNPU, HSP90AA
1.00E+00	3.22	B2M, CD226, CD36, HLA-A
1.00E+00	3.22	CFL1, HSPA8, PLEK, SPTBN1
1.00E+00	3.22	FCN1, PSMA5, PSMA6, UBQLN1
1.00E+00	3.22	ACTB, DHX9, HNRNPC, HNRNPU
1.00E+00	4.10	ARL6IP5, CLIC1, SOD1
1.00E+00	4.10	NPM1, YWHAB, YWHAG
1.00E+00	1.83	ADAM10, CANX, CDC42, CYBB, GNAI2, HNRNPU, HSP90AA1, HSPA8, ITGB1, RHOA, RPLP(
1.00E+00	1.70	ANXA2, ATP2A3, ATP5F1B, CALM3, FLNA, G6PD, GP1BA, GP1BB, ITGB1, ITGB3, SRI, STON
1.00E+00	2.03	B2M, BCAP31, CANX, HLA-A, HLA-B, HSPA9, QSOX1, RAB27B, RTN4
1.00E+00	3.19	CD226, CD47, HLA-A, HSPD1
1.00E+00	3.19	CD226, CD47, HLA-A, HSPD1
1.00E+00	3.19	HSP90AA1, ITGB1, JUP, YWHAG

1.00E+00	2.27	ATP5F1B, ITGB3, JUP, PFN1, RHOA, RTN4, THBS1
1.00E+00	6.53	ANXA2, MYH9
1.00E+00	6.53	CALM3, SOD1
1.00E+00	6.53	RHOA, SPN
1.00E+00	6.53	HNRNPU, KANSL3
1.00E+00	6.53	HSP90AA1, XRCC5
1.00E+00	6.53	PRDX1, PRDX2
1.00E+00	6.53	CD53, FLOT1
1.00E+00	6.53	TMEM109, XRCC5
1.00E+00	6.53	ENO1, TMEM109
1.00E+00	2.26	CD53, CD9, FLOT1, G6PD, HNRNPU, ITGB1, MYH9
1.00E+00	2.72	ATIC, RHOA, RTN4, SLC2A1, YWHAE
1.00E+00	1.82	ACTB, CDC42, CFL1, FLOT1, HSP90AA1, ITGB1, RAB10, RAC2, RHOA, RTN4, VCL, YWHAH
1.00E+00	3.17	B2M, CD226, CD36, HLA-A
1.00E+00	3.17	CALM3, HSP90AA1, HSPA9, VDAC2
1.00E+00	3.17	HSPA1B, HSPA8, HSPA9, HSPD1
1.00E+00	1.56	ACTB, ADH5, CALM3, CCL5, CD36, GAPDH, HINT1, HMBS, HSP90AA1, ILK, ITGB3, KANSL3,
1.00E+00	1.94	CSDE1, DHX9, HINT1, HNRNPC, HNRNPU, HSPA1B, NPM1, SYNCRIP, TNKS1BP1, VIM
1.00E+00	1.94	DHX9, HSP90AA1, NPM1, RPL11, RPLP0, RPS15, RPSA, SF3A3, WBP11, XRCC5
1.00E+00	2.44	ANXA2, FLNA, GP1BA, GP1BB, LTF, RHOA
1.00E+00	30.37	ITGB1
1.00E+00	30.37	JUP
1.00E+00	30.37	LRP1
1.00E+00	30.37	SPN
1.00E+00	30.37	B2M
1.00E+00	30.37	THBS1
1.00E+00	30.37	ATP5F1B
1.00E+00	30.37	ITGB1
1.00E+00	30.37	G6PD
1.00E+00	30.37	ITGB1
1.00E+00	30.37	THBS1
1.00E+00	30.37	NPM1
1.00E+00	30.37	FSCN1
1.00E+00	30.37	CFL1
1.00E+00	30.37	ANXA2
1.00E+00	30.37	ANXA2
1.00E+00	30.37	SRGN
1.00E+00	30.37	GALE
1.00E+00	30.37	SOD1
1.00E+00	30.37	HLA-A
1.00E+00	30.37	FCER1G
1.00E+00	30.37	CDC42
1.00E+00	30.37	LBP
1.00E+00	30.37	EIF5A
1.00E+00	30.37	EIF5A
1.00E+00	30.37	ADH5
1.00E+00	30.37	PNPLA8

1.00E+00	30.37	ATIC
1.00E+00	30.37	ATP2A3
1.00E+00	30.37	ITGB3
1.00E+00	30.37	GSTP1
1.00E+00	30.37	ITGB1
1.00E+00	30.37	ITGB1
1.00E+00	30.37	NPM1
1.00E+00	30.37	ITGB1
1.00E+00	30.37	RAC2
1.00E+00	30.37	HSPA8
1.00E+00	30.37	JUP
1.00E+00	30.37	PECAM1
1.00E+00	30.37	GNG5
1.00E+00	30.37	MYADM
1.00E+00	30.37	YWHAZ
1.00E+00	30.37	DHX9
1.00E+00	30.37	GNAI2
1.00E+00	30.37	GNAI2
1.00E+00	30.37	THBS1
1.00E+00	30.37	ACTB
1.00E+00	30.37	GSTP1
1.00E+00	30.37	GSTP1
1.00E+00	30.37	YWHAE
1.00E+00	30.37	F11R
1.00E+00	30.37	BCAP31
1.00E+00	30.37	CYBB
1.00E+00	30.37	LRP1
1.00E+00	30.37	RHOA
1.00E+00	30.37	ANXA2
1.00E+00	30.37	RTN4
1.00E+00	30.37	RPL11
1.00E+00	30.37	CD47
1.00E+00	30.37	HLA-A
1.00E+00	30.37	LRP1
1.00E+00	30.37	ENO1
1.00E+00	30.37	DHX9
1.00E+00	30.37	MYH9
1.00E+00	30.37	EEF1A1
1.00E+00	30.37	TPM4
1.00E+00	30.37	ITGB3
1.00E+00	30.37	JUP
1.00E+00	30.37	ACTB
1.00E+00	30.37	ENO1
1.00E+00	30.37	SOD1
1.00E+00	30.37	SLC2A3
1.00E+00	30.37	CALM3
1.00E+00	30.37	SOD1

1.00E+00	30.37	HSP90AA1
1.00E+00	30.37	HSP90AA1
1.00E+00	30.37	CDC42
1.00E+00	30.37	DHX9
1.00E+00	30.37	HINT1
1.00E+00	30.37	ATP5F1B
1.00E+00	30.37	SACM1L
1.00E+00	30.37	NCL
1.00E+00	30.37	GSTP1
1.00E+00	30.37	JUP
1.00E+00	30.37	PPIA
1.00E+00	30.37	DHX9
1.00E+00	1.66	ACTB, CD36, CDC42, CLIC1, F11R, FLNA, HNRNPU, HSPA1B, ILK, INPP5D, ITGB1, ITGB3, LT
1.00E+00	3.14	ANXA2, CD36, LRP1, OSBPL2
1.00E+00	2.11	AACS, CANX, CCL5, M6PR, PLEK, PPIA, RAB10, SRI
1.00E+00	2.69	ATP5F1B, ITGB3, PFN1, RTN4, THBS1
1.00E+00	1.92	ADAM10, ANK1, FSCN1, HNRNPA2B1, HSPA8, ITGA2B, JUP, MYH9, NPM1, VIM
1.00E+00	3.98	CD9, M6PR, TFRC
1.00E+00	3.98	DHX9, G3BP1, XRCC5
1.00E+00	3.98	ALDOA, G6PD, SLC2A3
1.00E+00	2.24	ACTB, B2M, FCER1G, PRDX2, RHOA, SOD1, SPN
1.00E+00	2.10	AACS, CANX, CCL5, M6PR, PLEK, PPIA, RAB10, SRI
1.00E+00	2.41	CYB5R3, ENO1, HSPA9, RAC2, TMEM109, VDAC2
1.00E+00	3.11	CD47, CD59, HLA-A, HLA-B
1.00E+00	3.11	HSP90AA1, ITGB1, RTN4, VCL
1.00E+00	6.30	CALM3, SRI
1.00E+00	6.30	CDC42, FSCN1
1.00E+00	6.30	LRP1, SPTBN1
1.00E+00	6.30	ITGB3, PECAM1
1.00E+00	6.30	ITGB3, PECAM1
1.00E+00	6.30	FLNA, YWHAE
1.00E+00	6.30	PFN1, RCC2
1.00E+00	6.30	NPM1, RCC2
1.00E+00	6.30	GNAI2, GNAQ
1.00E+00	6.30	NPM1, PSMA6
1.00E+00	2.23	FCER1G, HSPD1, LBP, LRP1, RAC2, SPN, TFRC
1.00E+00	3.93	G6PD, LDHB, TKT
1.00E+00	3.93	B2M, ITGB1, RHOA
1.00E+00	3.93	G6PD, LDHB, TKT
1.00E+00	3.93	F11R, ITGB3, SLC2A1
1.00E+00	3.93	EEF2, EIF5A, RPSA
1.00E+00	1.68	ADAM10, CANX, CDC42, CYBB, FLNA, G3BP1, GNAI2, HNRNPU, HSP90AA1, HSPA8, ITGB1
1.00E+00	3.09	CCL5, CSDE1, NPM1, RPS3A
1.00E+00	2.39	CYB5R3, ENO1, HSPA9, RAC2, TMEM109, VDAC2
1.00E+00	1.51	ACTB, B2M, CDC42, CFL1, FLNA, FLOT1, HSP90AA1, ITGA6, ITGB1, PPIA, RAB10, RAC2, RH
1.00E+00	2.64	CCL5, DHX9, GSTP1, HSPA1B, TTLL12
1.00E+00	3.87	B2M, HSPD1, TFRC

1.00E+00	3.87	NPM1, YWHAB, YWHAG
1.00E+00	6.09	PNPLA8, PTGS1
1.00E+00	6.09	CALM3, SRI
1.00E+00	6.09	CD36, ITGB3
1.00E+00	6.09	ILK, SOD1
1.00E+00	6.09	ANXA2, OSBPL2
1.00E+00	6.09	CCL5, PPIA
1.00E+00	6.09	INPP5D, LTF
1.00E+00	6.09	PNPLA8, PTGS1
1.00E+00	6.09	INPP5D, SACM1L
1.00E+00	6.09	RCC2, THBS1
1.00E+00	6.09	RAC2, UBE2L3
1.00E+00	6.09	SLC2A1, SLC2A3
1.00E+00	2.37	CCL5, HLA-A, INPP5D, RAC2, SPN, TFRC
1.00E+00	1.52	ACTB, B2M, CDC42, CFL1, FLNA, FLOT1, HSP90AA1, ITGA6, ITGB1, PPIA, RAB10, RAC2, RH
1.00E+00	1.97	CYBB, GSTP1, HSP90AA1, ITGB3, RHOA, SOD1, TFRC, THBS1, XRCC5
1.00E+00	2.06	F11R, HNRNPC, HNRNPU, MYADM, NPM1, PFN1, SPTBN1, TFRC
1.00E+00	1.96	ANXA2, CD36, ITGB3, LBP, LRP1, OSBPL2, PNPLA8, SLC2A1, THBS1
1.00E+00	1.83	CDC42, CFL1, HNRNPU, ITGB1, MYH9, NPM1, PLEK, RAB10, RAC2, RPS15, YWHAZ
1.00E+00	3.82	ARL6IP5, ITGB1, THBS1
1.00E+00	3.82	CLIC1, ILK, LTF
1.00E+00	3.82	TMEM109, TNKS1BP1, XRCC5
1.00E+00	3.82	F11R, PATJ, PECAM1
1.00E+00	1.58	CCL5, CSDE1, DHX9, EEF2, EIF5A, GAPDH, HNRNPC, HNRNPU, KRT17, MATR3, NCL, NPM1
1.00E+00	2.35	ALDOA, ATIC, ATP5F1B, ENO1, G6PD, TKT
1.00E+00	2.05	AACS, CANX, CCL5, M6PR, PLEK, PPIA, RAB10, SRI
1.00E+00	2.05	CD36, DSG1, HSP90AA1, ITGB3, MSN, PATJ, RAB27B, SLC2A1
1.00E+00	3.01	ADH5, G6PD, LDHA, LDHB
1.00E+00	2.60	CCL5, HLA-A, RAC2, SPN, TFRC
1.00E+00	1.77	ACTB, CDC42, CFL1, FLOT1, HSP90AA1, ITGB1, RAB10, RAC2, RHOA, RTN4, VCL, YWHAH
1.00E+00	2.17	AACS, ANXA2, GNAI2, PPIA, RAB27B, S100A8, SRI
1.00E+00	2.34	CFL1, EIF5A, HSPA8, PLEK, SPTBN1, UBQLN1
1.00E+00	5.90	ANPEP, CPN1
1.00E+00	5.90	KRT10, KRT2
1.00E+00	5.90	LTF, RHOA
1.00E+00	5.90	F11R, PFN1
1.00E+00	5.90	HNRNPU, HSPA1B
1.00E+00	5.90	EIF5A, RPL11
1.00E+00	5.90	RTN3, RTN4
1.00E+00	5.90	ITGB1, ITGB3
1.00E+00	3.76	NPM1, RPL11, RPLP0
1.00E+00	3.76	EEF2, HSPA1B, UBQLN1
1.00E+00	2.17	ATP2A3, CLIC1, ENO1, GAPDH, GNAQ, MATR3, TMEM109
1.00E+00	2.58	CDC42, HNRNPU, RCC2, TUBB, TUBB1
1.00E+00	2.33	CCL5, HLA-A, INPP5D, RAC2, SPN, TFRC
1.00E+00	1.95	CDC42, CFL1, HNRNPU, ITGB1, MYH9, NPM1, RAC2, RPS15, YWHAZ
1.00E+00	1.87	CDC42, CFL1, F11R, FLOT1, G3BP1, ITGB1, RAB33B, RAC2, RAP1B, RHOA

1.00E+00	2.96	HSP90AA1, HSPA1B, YWHAB, YWHAE
1.00E+00	1.75	ACTB, CDC42, CFL1, FLOT1, HSP90AA1, ITGB1, RAB10, RAC2, RHOA, RTN4, VCL, YWHAH
1.00E+00	3.71	CD226, HLA-A, HSPD1
1.00E+00	3.71	HLA-A, HLA-B, TTLL12
1.00E+00	3.71	DDX60L, DHX9, G3BP1
1.00E+00	1.48	ADAM10, ANXA7, CD36, CSDE1, DHX9, GAPDH, GSTP1, HNRNPA2B1, HNRNPC, HNRNPU,
1.00E+00	2.55	ANXA2, CD36, ITGB3, LRP1, THBS1
1.00E+00	1.86	ARL6IP5, CD36, CD47, CLIC1, ITGB1, PCYOX1, PNPLA8, SLC2A1, SLC2A3, VDAC2
1.00E+00	2.02	ANXA2, F11R, GAPDH, GNAI2, LRP1, LTF, RCC2, THBS1
1.00E+00	5.71	ANXA2, CD36
1.00E+00	5.71	CCL5, CDC42
1.00E+00	5.71	HSPA1B, INPP5D
1.00E+00	5.71	PNPLA8, PRDX6
1.00E+00	5.71	ITGB1, ITGB3
1.00E+00	5.71	LBP, RAC2
1.00E+00	5.71	GNAI2, GNAQ
1.00E+00	5.71	MYH9, PGK1
1.00E+00	5.71	INPP5D, SACM1L
1.00E+00	2.94	CALM3, CCL5, G6PD, SRI
1.00E+00	22.78	RHOA
1.00E+00	22.78	HLA-A
1.00E+00	22.78	THBS1
1.00E+00	22.78	RHOA
1.00E+00	22.78	HSPA1B
1.00E+00	22.78	CD9
1.00E+00	22.78	SLC2A3
1.00E+00	22.78	GALE
1.00E+00	22.78	LTF
1.00E+00	22.78	MSN
1.00E+00	22.78	ANXA2
1.00E+00	22.78	RHOA
1.00E+00	22.78	RTN4
1.00E+00	22.78	MYADM
1.00E+00	22.78	S100A9
1.00E+00	22.78	DHX9
1.00E+00	22.78	ITGB3
1.00E+00	22.78	NPM1
1.00E+00	22.78	MYH9
1.00E+00	22.78	CALM3
1.00E+00	22.78	CDC42
1.00E+00	22.78	PPIA
1.00E+00	22.78	G3BP1
1.00E+00	22.78	HSPA1B
1.00E+00	22.78	HSPA1B
1.00E+00	22.78	JUP
1.00E+00	22.78	CD36
1.00E+00	22.78	CD36

1.00E+00	22.78	RTN4
1.00E+00	22.78	HNRNPU
1.00E+00	22.78	HNRNPU
1.00E+00	22.78	RHOA
1.00E+00	22.78	HSPA9
1.00E+00	22.78	SLC2A1
1.00E+00	22.78	ANXA2
1.00E+00	22.78	RTN4
1.00E+00	22.78	CD47
1.00E+00	22.78	CCL5
1.00E+00	22.78	B2M
1.00E+00	22.78	LTF
1.00E+00	22.78	EIF5A
1.00E+00	22.78	FCER1G
1.00E+00	22.78	HSPD1
1.00E+00	22.78	LTF
1.00E+00	22.78	HSP90AA1
1.00E+00	22.78	ACTB
1.00E+00	22.78	RTN4
1.00E+00	22.78	CLTA
1.00E+00	22.78	GP1BA
1.00E+00	22.78	GSR
1.00E+00	22.78	GP1BA
1.00E+00	22.78	VDAC2
1.00E+00	22.78	INPP5D
1.00E+00	22.78	CYB5R3
1.00E+00	22.78	TKT
1.00E+00	22.78	ATIC
1.00E+00	22.78	FCER1G
1.00E+00	22.78	CCL5
1.00E+00	22.78	CCL5
1.00E+00	22.78	HSP90AA1
1.00E+00	22.78	HSPD1
1.00E+00	22.78	CYB5R3
1.00E+00	22.78	XRCC5
1.00E+00	22.78	LBP
1.00E+00	22.78	CCT6A
1.00E+00	22.78	ITGB1
1.00E+00	22.78	ACTB
1.00E+00	22.78	CD36
1.00E+00	2.54	CALM3, FLNA, G6PD, ITGB1, SRI
1.00E+00	3.66	JUP, RHOA, THBS1
1.00E+00	3.66	BCAP31, RAB10, RTN4
1.00E+00	3.66	HSPA8, M6PR, RTN4
1.00E+00	3.66	RAC2, STOM, UBE2L3
1.00E+00	2.13	NPM1, RPL11, RPLP0, RPS15, RPSA, WBP11, XRCC5
1.00E+00	2.29	CCL5, GNAI2, ITGB3, RHOA, THBS1, XRCC5

1.00E+00	2.53	B2M, CD226, CD36, HLA-A, TFRC
1.00E+00	3.62	FLNA, GNAI2, ITGB3
1.00E+00	3.62	G6PD, LDHB, TKT
1.00E+00	3.62	GSTP1, LBP, LTF
1.00E+00	3.62	DDX60L, DHX9, G3BP1
1.00E+00	2.51	ENO1, G6PD, GAPDH, PGK1, PKM
1.00E+00	2.51	B2M, LTF, RCC2, RTN4, YWHAH
1.00E+00	2.51	ENO1, G6PD, HSPA1B, PTPRJ, RTN4
1.00E+00	2.12	CD226, CD47, FCER1G, FCN1, HLA-A, INPP5D, PTPRJ
1.00E+00	2.12	CD226, CD47, FCER1G, FCN1, HLA-A, INPP5D, PTPRJ
1.00E+00	5.54	CALM3, SRI
1.00E+00	5.54	HNRNPC, HNRNPU
1.00E+00	5.54	FLG2, KRT17
1.00E+00	5.54	HSPA9, XRCC5
1.00E+00	5.54	SOD1, SRI
1.00E+00	5.54	HSPA1B, PRDX2
1.00E+00	5.54	BCAP31, UBQLN1
1.00E+00	5.54	HSPA1B, PRDX2
1.00E+00	2.50	DAZAP1, DSG1, ITGB3, SLC2A1, SOD1
1.00E+00	2.50	HSP90AA1, ITGB1, PTPRJ, RTN4, THBS1
1.00E+00	1.90	B2M, CFL1, FLNA, ITGA6, RHOA, RTN4, S100A9, VIM, YWHAH
1.00E+00	2.87	CD36, ITGA6, ITGB3, THBS1
1.00E+00	3.57	PPIA, SOD1, UBQLN1
1.00E+00	3.57	BCAP31, CD59, RAB1B
1.00E+00	2.48	CD59, HLA-A, HLA-B, INPP5D, TTLL12
1.00E+00	2.10	CCL5, CD36, GNAI2, GSTP1, ITGB3, RAP1B, YWHAZ
1.00E+00	2.85	EEF2, HSPA9, PLEK, XRCC5
1.00E+00	2.85	FLNA, HNRNPU, HSPA1B, RHOA
1.00E+00	2.85	ANPEP, BCAP31, CD59, RAB1B
1.00E+00	1.57	BCAP31, CANX, CSDE1, DHX9, HNRNPC, HNRNPU, HSPA1B, NPM1, PCYOX1, PSMA5, PSM
1.00E+00	2.47	ADH5, ANPEP, PTGS1, RHOA, SOD1
1.00E+00	5.38	CALM3, PLEK
1.00E+00	5.38	CD36, THBS1
1.00E+00	5.38	INPP5D, MYH9
1.00E+00	5.38	F11R, PFN1
1.00E+00	5.38	F11R, RCC2
1.00E+00	5.38	EIF5A, PLEK
1.00E+00	5.38	ADH5, RHOA
1.00E+00	5.38	ANXA2, ITGB3
1.00E+00	5.38	DSP, JUP
1.00E+00	5.38	ITGB1, ITGB3
1.00E+00	5.38	CALM3, SRI
1.00E+00	5.38	RAC2, UBE2L3
1.00E+00	5.38	DHX9, HNRNPU
1.00E+00	5.38	PNPLA8, PRDX6
1.00E+00	5.38	LRP1, THBS1
1.00E+00	3.52	DHX9, HNRNPU, PTBP1

1.00E+00	3.52	F11R, PATJ, PECAM1
1.00E+00	2.08	CTSD, GAPDH, LRBA, QSOX1, RAB1B, RAB33B, UBQLN1
1.00E+00	2.08	ACTB, HLA-B, INPP5D, LTF, PRDX2, RHOA, SOD1
1.00E+00	2.46	ACTB, CALM3, CANX, RAB27B, RAP1B
1.00E+00	3.48	CCL5, CSDE1, NPM1
1.00E+00	3.48	CALM3, ITGB3, SRI
1.00E+00	3.48	LRP1, SPTBN1, VIM
1.00E+00	2.80	ANXA2, CD36, LRP1, OSBPL2
1.00E+00	2.44	CCL5, CD36, ITGB3, PECAM1, PTPRJ
1.00E+00	5.22	CALM3, CYBB
1.00E+00	5.22	CYBB, SOD1
1.00E+00	5.22	CALM3, HSP90AA1
1.00E+00	5.22	HNRNPC, HNRNPU
1.00E+00	5.22	CLTA, RAB27B
1.00E+00	5.22	MYH9, MYL6
1.00E+00	5.22	HNRNPA2B1, MATR3
1.00E+00	5.22	HLA-A, HLA-B
1.00E+00	1.70	ACTB, CDC42, CFL1, FLOT1, HSP90AA1, ITGB1, RAB10, RAC2, RHOA, RTN4, VCL, YWHAH
1.00E+00	2.42	ANK1, HSP90AA1, JUP, SOD1, YWHAE
1.00E+00	1.31	ADAM10, ANXA2, ANXA7, CALM3, CD36, CSDE1, DHX9, ENO1, FLNA, G6PD, GAPDH, GNA
1.00E+00	1.41	ACTB, ANXA11, CALM3, CDC42, CFL1, CLIC1, CLTA, FLNA, GNAI2, HNRNPU, HSPA1B, HSP
1.00E+00	18.22	SF3A3
1.00E+00	18.22	HSPD1
1.00E+00	18.22	CD226
1.00E+00	18.22	RHOA
1.00E+00	18.22	ATIC
1.00E+00	18.22	SRI
1.00E+00	18.22	FLNA
1.00E+00	18.22	TKT
1.00E+00	18.22	ADH5
1.00E+00	18.22	THBS1
1.00E+00	18.22	THBS1
1.00E+00	18.22	TKT
1.00E+00	18.22	CD9
1.00E+00	18.22	CCL5
1.00E+00	18.22	DHX9
1.00E+00	18.22	FSCN1
1.00E+00	18.22	ANXA2
1.00E+00	18.22	ANXA2
1.00E+00	18.22	ANXA2
1.00E+00	18.22	RTN4
1.00E+00	18.22	CCL5
1.00E+00	18.22	RTN4
1.00E+00	18.22	CD36
1.00E+00	18.22	S100A9
1.00E+00	18.22	AACS
1.00E+00	18.22	ITGB1

1.00E+00	18.22	ITGB1
1.00E+00	18.22	ITGB1
1.00E+00	18.22	RAB10
1.00E+00	18.22	SRI
1.00E+00	18.22	HSPA1B
1.00E+00	18.22	CAMP
1.00E+00	18.22	CD47
1.00E+00	18.22	GSTP1
1.00E+00	18.22	JUP
1.00E+00	18.22	GNAI2
1.00E+00	18.22	CYBB
1.00E+00	18.22	ITGB1
1.00E+00	18.22	F11R
1.00E+00	18.22	CALM3
1.00E+00	18.22	F11R
1.00E+00	18.22	ENO1
1.00E+00	18.22	ENO1
1.00E+00	18.22	GNAI2
1.00E+00	18.22	LRP1
1.00E+00	18.22	NPM1
1.00E+00	18.22	HSP90AA1
1.00E+00	18.22	ANXA2
1.00E+00	18.22	CD36
1.00E+00	18.22	RAP1B
1.00E+00	18.22	ATP5F1B
1.00E+00	18.22	RAB27B
1.00E+00	18.22	ITGB1
1.00E+00	18.22	ANXA2
1.00E+00	18.22	MYH9
1.00E+00	18.22	SYNCRIP
1.00E+00	18.22	CLTA
1.00E+00	18.22	DHX9
1.00E+00	18.22	CLTA
1.00E+00	18.22	QSOX1
1.00E+00	18.22	ANXA2
1.00E+00	18.22	HSP90AA1
1.00E+00	18.22	CDC42
1.00E+00	18.22	INPP5D
1.00E+00	18.22	ALDOA
1.00E+00	18.22	RPLP0
1.00E+00	18.22	TNKS1BP1
1.00E+00	18.22	CYB5R3
1.00E+00	18.22	DHX9
1.00E+00	2.41	B2M, ITGB1, RHOA, YWHAG, YWHAH
1.00E+00	2.19	CDC42, DSP, FLNA, GNG5, ILK, RTN4
1.00E+00	3.39	CALM3, GNAI2, PLEK
1.00E+00	3.39	EEF1A1, EEF2, EIF5A

1.00E+00	1.29	ACTB, ANXA2, CCL5, CCT2, CCT4, CCT6A, CCT7, CCT8, CD36, CD47, CSDE1, DHX9, EEF2, EI
1.00E+00	5.08	B2M, HLA-A
1.00E+00	5.08	B2M, HLA-A
1.00E+00	5.08	CD36, ITGB3
1.00E+00	5.08	ILK, SOD1
1.00E+00	5.08	FLNA, YWHAE
1.00E+00	5.08	GNAI2, GNAQ
1.00E+00	2.74	ADH5, G6PD, LDHA, LDHB
1.00E+00	1.93	B2M, BCAP31, CANX, HLA-A, HLA-B, HSPA9, QSOX1, RTN4
1.00E+00	2.39	CALM3, ITGB1, SRI, STOM, UBQLN1
1.00E+00	2.72	CD226, HLA-A, INPP5D, PTPRJ
1.00E+00	3.35	CFL1, RHOA, SPTBN1
1.00E+00	3.35	INPP5D, ITGB3, TFRC
1.00E+00	2.17	ATP2A3, BCAP31, CANX, FLOT1, THBS1, UBQLN1
1.00E+00	2.17	ADAM10, CDC42, KRT17, RHOA, S100A8, S100A9
1.00E+00	2.70	CDC42, CFL1, ITGB1, YWHAH
1.00E+00	2.70	FLNA, SRI, YWHAE, YWHAH
1.00E+00	4.94	ADH5, YWHAZ
1.00E+00	4.94	ANXA2, LRP1
1.00E+00	4.94	ANXA2, LRP1
1.00E+00	4.94	DSG1, THBS1
1.00E+00	4.94	CD36, ITGB3
1.00E+00	4.94	CD36, LRP1
1.00E+00	4.94	ADH5, GAPDH
1.00E+00	3.31	HSP90AA1, SLC2A1, XRCC5
1.00E+00	2.68	BCAP31, HSPA1B, RPL11, UBQLN1
1.00E+00	1.28	ACTB, ANXA2, CCL5, CCT2, CCT4, CCT6A, CCT7, CCT8, CD36, CSDE1, DHX9, EEF2, EIF5A, E
1.00E+00	1.71	CDC42, DSP, FLNA, G6PD, GNG5, HNRNPU, ILK, ITGB1, MATR3, RTN4, SRI
1.00E+00	3.27	BCAP31, CANX, UBQLN1
1.00E+00	3.27	CCL5, LTF, PPIA
1.00E+00	3.27	DHX9, GSTP1, ITGB3
1.00E+00	2.66	CCL5, FCN1, LTF, PPIA
1.00E+00	2.66	PNPLA8, SOD1, TKT, VIM
1.00E+00	2.66	PNPLA8, SOD1, TKT, VIM
1.00E+00	4.81	RCC2, THBS1
1.00E+00	4.81	S100A8, S100A9
1.00E+00	4.81	PLEK, TLN1
1.00E+00	4.81	RAB10, SPTBN1
1.00E+00	4.81	CCL5, THBS1
1.00E+00	4.81	FLNA, YWHAE
1.00E+00	4.81	GP1BA, RHOA
1.00E+00	4.81	B2M, CD36
1.00E+00	4.81	ACTB, HNRNPC
1.00E+00	2.64	CYB5R3, G6PD, PLEK, SOD1
1.00E+00	3.23	F11R, FLOT1, ITGB1
1.00E+00	2.12	CCL5, CD36, ITGB3, NCL, PECAM1, PTPRJ
1.00E+00	1.45	ANPEP, ANXA2, BCAP31, CANX, CPN1, CTSD, EEF1A1, FLNA, HINT1, HSP90AA1, HSPA1B, I

1.00E+00	2.62	CD36, ITGB1, ITGB3, THBS1
1.00E+00	2.31	CD36, GSTP1, PRDX1, PRDX2, SOD1
1.00E+00	3.19	CCL5, GSTP1, TTLL12
1.00E+00	3.19	INPP5D, ITGB3, TFRC
1.00E+00	3.19	ADAM10, CLTA, RAB27B
1.00E+00	3.19	QSOX1, TUBB, TUBB1
1.00E+00	4.68	LRP1, RHOA
1.00E+00	4.68	GNAI2, RAP1B
1.00E+00	4.68	SOD1, TFRC
1.00E+00	4.68	HINT1, PTBP1
1.00E+00	4.68	CALM3, SRI
1.00E+00	4.68	ACTB, KANSL3
1.00E+00	4.68	TKT, VDAC2
1.00E+00	1.46	ACTB, ANXA2, EIF5A, FLNA, HINT1, HNRNPA2B1, HNRNPA3, HNRNPC, HNRNPF, HNRNPH
1.00E+00	15.18	NPM1
1.00E+00	15.18	B2M
1.00E+00	15.18	FCN1
1.00E+00	15.18	CD226
1.00E+00	15.18	YWHAE
1.00E+00	15.18	SPTBN1
1.00E+00	15.18	CCL5
1.00E+00	15.18	ITGA6
1.00E+00	15.18	CPN1
1.00E+00	15.18	RHOA
1.00E+00	15.18	FSCN1
1.00E+00	15.18	NASP
1.00E+00	15.18	DHX9
1.00E+00	15.18	THBS1
1.00E+00	15.18	M6PR
1.00E+00	15.18	GNAI2
1.00E+00	15.18	NASP
1.00E+00	15.18	FLOT1
1.00E+00	15.18	RHOA
1.00E+00	15.18	FCN1
1.00E+00	15.18	NPM1
1.00E+00	15.18	FLOT1
1.00E+00	15.18	RAB33B
1.00E+00	15.18	KRT14
1.00E+00	15.18	CDC42
1.00E+00	15.18	PECAM1
1.00E+00	15.18	LBP
1.00E+00	15.18	MYH9
1.00E+00	15.18	MYH9
1.00E+00	15.18	CCL5
1.00E+00	15.18	CD47
1.00E+00	15.18	B2M
1.00E+00	15.18	XRCC5

1.00E+00	15.18	TUBB
1.00E+00	15.18	ITGB3
1.00E+00	15.18	ATIC
1.00E+00	15.18	ATIC
1.00E+00	15.18	ATIC
1.00E+00	15.18	SRI
1.00E+00	15.18	FLNA
1.00E+00	15.18	SRGN
1.00E+00	15.18	LRP1
1.00E+00	15.18	LTF
1.00E+00	15.18	ITGB1
1.00E+00	15.18	SOD1
1.00E+00	15.18	DHX9
1.00E+00	15.18	XRCC5
1.00E+00	15.18	FLNA
1.00E+00	15.18	ITGB3
1.00E+00	15.18	THBS1
1.00E+00	15.18	CD99
1.00E+00	15.18	KRT6A
1.00E+00	15.18	CDC42
1.00E+00	15.18	THBS1
1.00E+00	15.18	NPM1
1.00E+00	15.18	ATP5F1B
1.00E+00	15.18	HSPA8
1.00E+00	15.18	HSPA8
1.00E+00	15.18	CLTA
1.00E+00	15.18	ADH5
1.00E+00	15.18	CYB5R3
1.00E+00	15.18	INPP5D
1.00E+00	15.18	HINT1
1.00E+00	15.18	HSP90AA1
1.00E+00	15.18	FCER1G
1.00E+00	15.18	GAPDH
1.00E+00	15.18	TLN1
1.00E+00	15.18	SOD1
1.00E+00	15.18	CCL5
1.00E+00	15.18	INPP5D
1.00E+00	15.18	NCL
1.00E+00	15.18	INPP5D
1.00E+00	15.18	ITGB1
1.00E+00	1.74	BCAP31, CALM3, HSP90AA1, HSPA9, HSPD1, LRBA, RAC2, TFRC, UBE2L3, VDAC2
1.00E+00	2.60	CALM3, GNAI2, RAC2, RAP1B
1.00E+00	2.60	FLNA, SRI, YWHAE, YWHAH
1.00E+00	2.10	GNAI2, NCL, PKM, RAB10, SLC2A1, YWHAG
1.00E+00	1.97	F11R, ITGB3, NPM1, SLC2A1, TMEM109, TNKS1BP1, XRCC5
1.00E+00	1.97	F11R, ITGB3, NPM1, SLC2A1, TMEM109, TNKS1BP1, XRCC5
1.00E+00	3.16	DHX9, HNRNPA2B1, NPM1

1.00E+00	1.26	ACTB, ANXA2, CCL5, CCT2, CCT4, CCT6A, CCT7, CCT8, CD36, CSDE1, DHX9, EEF1A1, EEF2,
1.00E+00	4.57	CD226, TFRC
1.00E+00	4.57	CD226, TFRC
1.00E+00	4.57	HSPA8, M6PR
1.00E+00	4.57	CD36, HSPD1
1.00E+00	4.57	LBP, LTF
1.00E+00	4.57	FLNA, MSN
1.00E+00	4.57	CD36, HSP90AA1
1.00E+00	4.57	DHX9, HSP90AA1
1.00E+00	4.57	CD36, F11R
1.00E+00	4.57	S100A8, S100A9
1.00E+00	4.57	CALM3, SRI
1.00E+00	4.57	CAMP, SELPLG
1.00E+00	4.57	RHOA, THBS1
1.00E+00	4.57	CDC42, F11R
1.00E+00	4.57	MYL6, TPM4
1.00E+00	2.26	GNAI2, NCL, PKM, RAB10, YWHAG
1.00E+00	2.07	GSTP1, HLA-A, HLA-B, KRT1, SOD1, TTLL12
1.00E+00	1.60	ACTB, CD9, CDC42, CFL1, FLOT1, HSP90AA1, ITGB1, RAB10, RAC2, RHOA, RTN4, VCL, YWI
1.00E+00	2.25	DAZAP1, DSG1, ITGB3, SLC2A1, SOD1
1.00E+00	2.25	CFL1, HSPA8, RHOA, TUBB, YWHAZ
1.00E+00	2.25	CDC42, HNRNPU, HSPA8, RAB27B, SOD1
1.00E+00	1.94	CCL5, CD36, GNAI2, GSTP1, ITGB3, RAP1B, YWHAZ
1.00E+00	4.46	FLNA, NCL
1.00E+00	4.46	G6PD, TKT
1.00E+00	4.46	CALM3, TNKS1BP1
1.00E+00	4.46	HNRNPU, HSPA1B
1.00E+00	4.46	EEF1A1, NCL
1.00E+00	4.46	SLC2A1, SLC2A3
1.00E+00	4.46	DHX9, HNRNPU
1.00E+00	4.46	ACTB, HSP90AA1
1.00E+00	1.84	CD53, CD9, FLOT1, G6PD, HNRNPU, ITGB1, MYH9, PTBP1
1.00E+00	2.24	ACTB, INPP5D, PRDX2, RHOA, SOD1
1.00E+00	1.93	CD226, CD47, FCER1G, FCN1, HLA-A, INPP5D, PTPRJ
1.00E+00	2.05	ALDOA, CANX, FCN1, G6PD, KRT1, SLC2A3
1.00E+00	3.05	RAB10, RTN3, RTN4
1.00E+00	3.05	MSN, PLEK, RAB10
1.00E+00	3.05	DHX9, G3BP1, XRCC5
1.00E+00	3.05	CALM3, G6PD, SRI
1.00E+00	1.92	ADAM10, HSPA8, RPL8, RPLP0, RTN3, RTN4, SPTBN1
1.00E+00	2.51	CD36, PRDX1, PRDX2, SOD1
1.00E+00	2.51	ATP5F1B, JUP, RHOA, THBS1
1.00E+00	2.51	CALM3, HSP90AA1, HSPD1, VDAC2
1.00E+00	1.70	ENO1, GSTP1, ITGB1, MYADM, PLEK, PPIA, PTPRJ, RHOA, THBS1, VDAC2
1.00E+00	2.22	CALM3, FLOT1, ITGB1, ITGB3, RAP1B
1.00E+00	4.35	ADAM10, MYH9
1.00E+00	4.35	CALM3, SOD1

1.00E+00	4.35	CCL5, ITGB3
1.00E+00	4.35	CD226, PTPRJ
1.00E+00	4.35	RAC2, UBE2L3
1.00E+00	4.35	CD36, HSP90AA1
1.00E+00	1.75	ANXA2, CD36, ITGB3, LBP, LRP1, OSBPL2, PNPLA8, SLC2A1, THBS1
1.00E+00	3.02	ITGB1, RHOA, THBS1
1.00E+00	3.02	AACS, S100A8, SRI
1.00E+00	3.02	BCAP31, HSPA1B, UBQLN1
1.00E+00	3.02	CCL5, GSTP1, TTLL12
1.00E+00	2.49	CYBB, ENO1, PGK1, UBQLN1
1.00E+00	13.01	LBP
1.00E+00	13.01	HLA-B
1.00E+00	13.01	HLA-B
1.00E+00	13.01	CD226
1.00E+00	13.01	CD226
1.00E+00	13.01	HLA-B
1.00E+00	13.01	HLA-B
1.00E+00	13.01	DSP
1.00E+00	13.01	ATIC
1.00E+00	13.01	TCP1
1.00E+00	13.01	ITGB1
1.00E+00	13.01	TFRC
1.00E+00	13.01	THBS1
1.00E+00	13.01	CD36
1.00E+00	13.01	FLNA
1.00E+00	13.01	HSPA8
1.00E+00	13.01	ITGB1
1.00E+00	13.01	INPP5D
1.00E+00	13.01	NPM1
1.00E+00	13.01	FLOT1
1.00E+00	13.01	BCAP31
1.00E+00	13.01	CD36
1.00E+00	13.01	CD36
1.00E+00	13.01	B2M
1.00E+00	13.01	GSTP1
1.00E+00	13.01	HLA-A
1.00E+00	13.01	ATIC
1.00E+00	13.01	SOD1
1.00E+00	13.01	HSPA9
1.00E+00	13.01	INPP5D
1.00E+00	13.01	SRI
1.00E+00	13.01	ATIC
1.00E+00	13.01	ACTB
1.00E+00	13.01	KRT6A
1.00E+00	13.01	SOD1
1.00E+00	13.01	CALM3
1.00E+00	13.01	FLNA

1.00E+00	13.01	RAC2
1.00E+00	13.01	HSPA1B
1.00E+00	13.01	RAC2
1.00E+00	13.01	RAC2
1.00E+00	13.01	BCAP31
1.00E+00	13.01	CD36
1.00E+00	13.01	HSPA8
1.00E+00	13.01	HSPA1B
1.00E+00	13.01	FSCN1
1.00E+00	13.01	ANXA2
1.00E+00	13.01	SOD1
1.00E+00	13.01	RAB27B
1.00E+00	13.01	RAB27B
1.00E+00	13.01	SOD1
1.00E+00	13.01	THBS1
1.00E+00	13.01	LRP1
1.00E+00	13.01	UBQLN1
1.00E+00	13.01	HNRNPU
1.00E+00	13.01	HNRNPU
1.00E+00	13.01	LTF
1.00E+00	13.01	HSP90AA1
1.00E+00	13.01	MSN
1.00E+00	13.01	MSN
1.00E+00	13.01	RAB10
1.00E+00	13.01	CD59
1.00E+00	13.01	EEF1A1
1.00E+00	13.01	ITGB3
1.00E+00	13.01	CD99
1.00E+00	13.01	CCL5
1.00E+00	13.01	HLA-A
1.00E+00	13.01	LRP1
1.00E+00	13.01	MSN
1.00E+00	13.01	THBS1
1.00E+00	13.01	JUP
1.00E+00	13.01	MYL6
1.00E+00	13.01	FCN1
1.00E+00	13.01	HSPA8
1.00E+00	13.01	HSPA8
1.00E+00	13.01	DHX9
1.00E+00	13.01	HLA-A
1.00E+00	13.01	TKT
1.00E+00	13.01	PRDX6
1.00E+00	13.01	INPP5D
1.00E+00	13.01	DHX9
1.00E+00	13.01	ADAM10
1.00E+00	13.01	HNRNPU
1.00E+00	13.01	M6PR

1.00E+00	2.20	B2M, HSPA1B, HSPA9, INPP5D, LTF
1.00E+00	2.98	CALM3, CDC42, RHOA
1.00E+00	2.98	ADAM10, RTN3, RTN4
1.00E+00	2.98	HSPA9, INPP5D, LTF
1.00E+00	4.25	MATR3, RTN4
1.00E+00	4.25	PLEK, RAB10
1.00E+00	4.25	ITGB1, VIM
1.00E+00	4.25	CALM3, TNKS1BP1
1.00E+00	4.25	CD226, RAC2
1.00E+00	4.25	INPP5D, SACM1L
1.00E+00	4.25	ARL6IP5, ITGB1
1.00E+00	4.25	CAMP, SELPLG
1.00E+00	4.25	FLNA, MSN
1.00E+00	4.25	CD36, LRP1
1.00E+00	4.25	SRI, YWHAE
1.00E+00	1.90	ANK1, FSCN1, HNRNPA2B1, HSPA8, JUP, MYH9, NPM1
1.00E+00	2.19	CFL1, HSPA8, RHOA, TUBB, YWHAZ
1.00E+00	2.95	FCER1G, LBP, RAC2
1.00E+00	2.95	ACTB, ATP2A3, B2M
1.00E+00	2.95	GSTP1, INPP5D, SPN
1.00E+00	1.73	ACTB, FLNA, FLOT1, HSP90AA1, ITGB1, RAB10, RAC2, RTN4, VCL
1.00E+00	4.15	RHOA, RTN4
1.00E+00	4.15	CDC42, RHOA
1.00E+00	4.15	PFN1, RCC2
1.00E+00	4.15	HSPA1B, PRDX2
1.00E+00	4.15	SPN, TFRC
1.00E+00	2.92	JUP, RHOA, THBS1
1.00E+00	2.43	ANXA2, ITGB3, LRP1, THBS1
1.00E+00	2.43	HNRNPU, HSPA1B, PATJ, RHOA
1.00E+00	2.43	DDX60L, DHX9, G3BP1, XRCC5
1.00E+00	1.87	ADAM10, RPL8, RPLP0, RTN3, RTN4, SPTBN1, YWHAZ
1.00E+00	2.16	DAZAP1, DSG1, ITGB3, SLC2A1, SOD1
1.00E+00	1.79	CYBB, GNAI2, ITGB3, NCL, PKM, RAB10, SLC2A1, YWHAG
1.00E+00	1.43	ANXA2, ANXA5, ATP2A3, CALM3, CCL5, GAPDH, GNAQ, GSTP1, HSP90AA1, LTF, NPM1, R
1.00E+00	1.78	CDC42, DSP, FLOT1, G6PD, HNRNPU, ITGB1, MYL6, RHOA
1.00E+00	2.89	CCL5, PF4V1, RHOA
1.00E+00	2.89	CCL5, PF4V1, RHOA
1.00E+00	2.89	CTSD, HSP90AA1, HSPA8
1.00E+00	2.89	ADAM10, ANPEP, CPN1
1.00E+00	2.14	CALM3, FLOT1, ITGB1, ITGB3, RAP1B
1.00E+00	4.06	ANXA2, OSBPL2
1.00E+00	4.06	ITGB3, THBS1
1.00E+00	4.06	F11R, VCL
1.00E+00	4.06	HSP90AA1, SOD1
1.00E+00	4.06	ITGB3, RHOA
1.00E+00	4.06	SLC2A1, SLC2A3
1.00E+00	4.06	EEF1A1, NCL

1.00E+00	4.06	SLC2A1, XRCC5
1.00E+00	4.06	CANX, CLTA
1.00E+00	4.06	HNRNPU, HSPA1B
1.00E+00	4.06	ADH5, GAPDH
1.00E+00	1.25	ACTB, ANXA2, CCL5, CCT2, CCT4, CCT6A, CCT7, CCT8, CD36, CD47, CSDE1, DHX9, EEF2, EI
1.00E+00	11.39	CD226
1.00E+00	11.39	CD226
1.00E+00	11.39	PLEK
1.00E+00	11.39	THBS1
1.00E+00	11.39	CD226
1.00E+00	11.39	HSPD1
1.00E+00	11.39	CD226
1.00E+00	11.39	GALE
1.00E+00	11.39	ADH5
1.00E+00	11.39	HMBS
1.00E+00	11.39	DHX9
1.00E+00	11.39	CCL5
1.00E+00	11.39	CD9
1.00E+00	11.39	HINT1
1.00E+00	11.39	PTGS1
1.00E+00	11.39	LDHB
1.00E+00	11.39	ANXA2
1.00E+00	11.39	SOD1
1.00E+00	11.39	KRT6A
1.00E+00	11.39	UBQLN1
1.00E+00	11.39	TCP1
1.00E+00	11.39	RHOA
1.00E+00	11.39	AACS
1.00E+00	11.39	YWHAH
1.00E+00	11.39	GSTP1
1.00E+00	11.39	GSTP1
1.00E+00	11.39	RTN4
1.00E+00	11.39	SOD1
1.00E+00	11.39	PTPRJ
1.00E+00	11.39	LBP
1.00E+00	11.39	LRP1
1.00E+00	11.39	NPM1
1.00E+00	11.39	CASP14
1.00E+00	11.39	CD36
1.00E+00	11.39	CD36
1.00E+00	11.39	DHX9
1.00E+00	11.39	CD36
1.00E+00	11.39	CD36
1.00E+00	11.39	VIM
1.00E+00	11.39	B2M
1.00E+00	11.39	HSPA8
1.00E+00	11.39	GNAI2

1.00E+00	11.39	RHOA
1.00E+00	11.39	NPM1
1.00E+00	11.39	ADAM10
1.00E+00	11.39	ADH5
1.00E+00	11.39	ENO1
1.00E+00	11.39	ITGB3
1.00E+00	11.39	FCN1
1.00E+00	11.39	MYADM
1.00E+00	11.39	ARL6IP5
1.00E+00	11.39	RPLP0
1.00E+00	11.39	RPLP0
1.00E+00	11.39	RHOA
1.00E+00	11.39	ENO1
1.00E+00	11.39	HLA-A
1.00E+00	11.39	ARPC5
1.00E+00	11.39	SPTBN1
1.00E+00	11.39	FLOT1
1.00E+00	11.39	ATP2A3
1.00E+00	11.39	SPTBN1
1.00E+00	11.39	RAB10
1.00E+00	11.39	HSP90AA1
1.00E+00	11.39	XRCC5
1.00E+00	11.39	HSPA8
1.00E+00	11.39	ACTB
1.00E+00	11.39	XRCC5
1.00E+00	11.39	ADH5
1.00E+00	11.39	ATP2A3
1.00E+00	11.39	GSTP1
1.00E+00	11.39	ITGB3
1.00E+00	11.39	CASP14
1.00E+00	11.39	YWHAZ
1.00E+00	2.86	FLNA, ITGB1, YWHAE
1.00E+00	1.70	CCL5, EIF5A, FCER1G, GSTP1, HSPA1B, ILK, PF4V1, PTPRJ, TTLL12
1.00E+00	1.49	ANXA2, ATP2A3, ATP5F1B, CALM3, FLNA, G6PD, GP1BA, GP1BB, ITGB1, ITGB3, SRI, STOM
1.00E+00	2.83	CD36, LBP, PRDX2
1.00E+00	2.83	CYBB, JUP, RPLP0
1.00E+00	3.97	LBP, TFRC
1.00E+00	3.97	RTN4, YWHAZ
1.00E+00	3.97	PRDX2, SOD1
1.00E+00	3.97	RTN3, RTN4
1.00E+00	3.97	ITGB1, ITGB3
1.00E+00	2.37	CDC42, G6PD, HNRNPU, ITGB1
1.00E+00	2.37	CYBB, ENO1, PGK1, UBQLN1
1.00E+00	2.11	CD47, DHX9, FLOT1, HNRNPA2B1, NPM1
1.00E+00	2.11	CCL5, CD36, GNAI2, ITGB3, RAP1B
1.00E+00	2.80	ENO1, PPIA, VDAC2
1.00E+00	1.75	BCAP31, CSDE1, DHX9, HNRNPU, HSPA1B, SYNCRIP, TNKS1BP1, UBQLN1

1.00E+00	2.10	GSTP1, HSPA1B, ITGA6, PRDX2, THBS1
1.00E+00	1.26	ADAM10, ANXA2, ANXA7, CALM3, CD36, CSDE1, DHX9, ENO1, FLNA, G6PD, GAPDH, GNA
1.00E+00	3.89	RAB27B, SOD1
1.00E+00	3.89	DHX9, FLOT1
1.00E+00	3.89	CALM3, SRI
1.00E+00	3.89	CD36, RHOA
1.00E+00	3.89	G6PD, RHOA
1.00E+00	3.89	CCL5, PF4V1
1.00E+00	1.93	ACTB, ANXA2, CD36, ITGB3, LRP1, OSBPL2
1.00E+00	1.92	FCER1G, HSPD1, LBP, RAC2, SPN, TFRC
1.00E+00	1.30	ADH5, ALDOA, ANPEP, ARL6IP5, ATP2A3, B2M, CALM3, CD36, CDC42, CYB5R3, DSP, ENO
1.00E+00	2.74	CD36, FCN1, LBP
1.00E+00	2.74	FCN1, HSPA1B, LBP
1.00E+00	1.62	AACS, CYB5R3, G6PD, GSTP1, OSBPL2, PGK1, PLEK, PNPLA8, PTGS1, SOD1
1.00E+00	3.80	ARL6IP5, ITGB1
1.00E+00	3.80	RAC2, UBE2L3
1.00E+00	2.31	DHX9, FLOT1, HNRNPA2B1, NPM1
1.00E+00	2.31	DHX9, FLOT1, HNRNPA2B1, NPM1
1.00E+00	10.12	CD226
1.00E+00	10.12	CCL5
1.00E+00	10.12	RHOA
1.00E+00	10.12	CDC42
1.00E+00	10.12	GNG5
1.00E+00	10.12	MYH9
1.00E+00	10.12	ATIC
1.00E+00	10.12	NPM1
1.00E+00	10.12	INPP5D
1.00E+00	10.12	ALDOA
1.00E+00	10.12	NPM1
1.00E+00	10.12	ANXA2
1.00E+00	10.12	ANXA2
1.00E+00	10.12	GSTP1
1.00E+00	10.12	TFRC
1.00E+00	10.12	CCL5
1.00E+00	10.12	ITGA6
1.00E+00	10.12	CDC42
1.00E+00	10.12	CALM3
1.00E+00	10.12	ADH5
1.00E+00	10.12	ITGB1
1.00E+00	10.12	PTPRJ
1.00E+00	10.12	HSP90AA1
1.00E+00	10.12	CDC42
1.00E+00	10.12	CCL5
1.00E+00	10.12	F11R
1.00E+00	10.12	FLNA
1.00E+00	10.12	ACTB
1.00E+00	10.12	NCL

1.00E+00	10.12	AACS
1.00E+00	10.12	MSN
1.00E+00	10.12	RPLP0
1.00E+00	10.12	NPM1
1.00E+00	10.12	LRP1
1.00E+00	10.12	RAB27B
1.00E+00	10.12	CD36
1.00E+00	10.12	SLC2A1
1.00E+00	10.12	TUBB
1.00E+00	10.12	ANXA2
1.00E+00	10.12	MYH9
1.00E+00	10.12	ACTB
1.00E+00	10.12	SACM1L
1.00E+00	10.12	LYZ
1.00E+00	10.12	INPP5D
1.00E+00	10.12	RPL11
1.00E+00	10.12	ANXA11
1.00E+00	10.12	OSBPL2
1.00E+00	10.12	CD36
1.00E+00	10.12	DSG1
1.00E+00	10.12	CYBB
1.00E+00	10.12	CYB5R3
1.00E+00	10.12	ATIC
1.00E+00	10.12	ALDOA
1.00E+00	10.12	ADH5
1.00E+00	10.12	INPP5D
1.00E+00	10.12	SACM1L
1.00E+00	10.12	HLA-A
1.00E+00	10.12	PTPRJ
1.00E+00	10.12	NANS
1.00E+00	10.12	LBP
1.00E+00	10.12	UBE2L3
1.00E+00	10.12	HNRNPA2B1
1.00E+00	2.29	ACTB, ATP2A3, B2M, SRI
1.00E+00	2.72	B2M, LTF, TFRC
1.00E+00	2.72	FCN1, HSPA1B, LBP
1.00E+00	2.72	HSPD1, INPP5D, TFRC
1.00E+00	2.05	G6PD, HSP90AA1, ITGB1, RTN4, VCL
1.00E+00	3.73	CCL5, DHX9
1.00E+00	3.73	GSTP1, PPIA
1.00E+00	3.73	GSTP1, RHOA
1.00E+00	3.73	ANXA2, RAB1B
1.00E+00	3.73	RHOA, SPN
1.00E+00	3.73	CDC42, FSCN1
1.00E+00	3.73	GSTP1, PPIA
1.00E+00	3.73	OSBPL2, VDAC2
1.00E+00	3.73	ITGB3, QSOX1

1.00E+00	2.28	CD36, FLNA, HSP90AA1, JUP
1.00E+00	2.28	CDC42, RAB1B, RAB33B, YWHAZ
1.00E+00	2.28	CALM3, HSP90AA1, HSPD1, VDAC2
1.00E+00	1.71	ACTB, FLOT1, HSP90AA1, ITGB1, RAB10, RAC2, RTN4, VCL
1.00E+00	2.04	ATP5F1B, ITGB3, JUP, RHOA, THBS1
1.00E+00	2.04	ADAM10, CCL5, LBP, RAC2, THBS1
1.00E+00	1.89	ANK1, BCAP31, RAB10, RAB1B, RAB33B, SPTBN1
1.00E+00	2.26	DHX9, FLOT1, HNRNPA2B1, NPM1
1.00E+00	1.78	JUP, KRT16, LRP1, PTPRJ, RHOA, THBS1, VCL
1.00E+00	2.66	ALDOA, ATP5F1B, ENO1
1.00E+00	2.66	CCL5, HLA-A, TFRC
1.00E+00	3.65	INPP5D, LTF
1.00E+00	3.65	PNPLA8, PTGS1
1.00E+00	3.65	B2M, RHOA
1.00E+00	3.65	FLNA, YWHAE
1.00E+00	3.65	PLEK, SOD1
1.00E+00	2.25	CCL5, GSTP1, HSPA1B, TTLL12
1.00E+00	2.25	CCL5, HLA-A, RAC2, TFRC
1.00E+00	1.47	ALDOA, BCAP31, CCT2, CCT4, CCT7, CCT8, CD9, DAZAP1, ITGB1, KRT9, MYH9, SOD1, TCP:
1.00E+00	1.76	ARL6IP5, CD36, CD47, ITGB1, PNPLA8, SLC2A1, SLC2A3
1.00E+00	9.11	THBS1
1.00E+00	9.11	ATIC
1.00E+00	9.11	EIF5A
1.00E+00	9.11	ARL6IP5
1.00E+00	9.11	HSPA1B
1.00E+00	9.11	ANXA2
1.00E+00	9.11	ANXA2
1.00E+00	9.11	LRP1
1.00E+00	9.11	CD36
1.00E+00	9.11	GNAI2
1.00E+00	9.11	MYADM
1.00E+00	9.11	RTN4
1.00E+00	9.11	CANX
1.00E+00	9.11	ANK1
1.00E+00	9.11	ITGB3
1.00E+00	9.11	PPIA
1.00E+00	9.11	SOD1
1.00E+00	9.11	FLOT1
1.00E+00	9.11	ANK1
1.00E+00	9.11	HSP90AA1
1.00E+00	9.11	ARL6IP5
1.00E+00	9.11	HMBS
1.00E+00	9.11	RTN4
1.00E+00	9.11	BCAP31
1.00E+00	9.11	ITGB3
1.00E+00	9.11	VIM
1.00E+00	9.11	MYADM

1.00E+00	9.11	MSN
1.00E+00	9.11	ITGB3
1.00E+00	9.11	CDC42
1.00E+00	9.11	HSPA1B
1.00E+00	9.11	CD59
1.00E+00	9.11	FLNA
1.00E+00	9.11	KANSL3
1.00E+00	9.11	RHOA
1.00E+00	9.11	PPIA
1.00E+00	9.11	FCN1
1.00E+00	9.11	MSN
1.00E+00	9.11	FLOT1
1.00E+00	9.11	TCP1
1.00E+00	9.11	ANXA2
1.00E+00	9.11	RHOA
1.00E+00	9.11	LTF
1.00E+00	9.11	CDC42
1.00E+00	9.11	ATP2A3
1.00E+00	9.11	RAB27B
1.00E+00	9.11	KANSL3
1.00E+00	9.11	HSPA8
1.00E+00	9.11	FLOT1
1.00E+00	9.11	FCN1
1.00E+00	9.11	PFN1
1.00E+00	9.11	VCL
1.00E+00	9.11	CYB5R3
1.00E+00	9.11	GALE
1.00E+00	9.11	FCER1G
1.00E+00	9.11	CDC42
1.00E+00	9.11	XRCC5
1.00E+00	9.11	CALM3
1.00E+00	9.11	DHX9
1.00E+00	9.11	RPS15
1.00E+00	9.11	PNPLA8
1.00E+00	3.58	HSPD1, TFRC
1.00E+00	3.58	HSPD1, TFRC
1.00E+00	3.58	HSP90AA1, HSPD1
1.00E+00	3.58	PFN1, RHOA
1.00E+00	3.58	ANXA2, CD36
1.00E+00	3.58	HSPD1, TFRC
1.00E+00	3.58	DHX9, ITGB3
1.00E+00	3.58	ANXA2, B2M
1.00E+00	3.58	FCN1, KRT6A
1.00E+00	3.58	CDC42, RHOA
1.00E+00	3.58	EIF5A, HNRNPU
1.00E+00	3.58	ATP2A3, ATP5F1B
1.00E+00	3.58	GAPDH, LDHB

1.00E+00	3.58	HNRNPF, HNRNPU
1.00E+00	2.22	CD36, FLNA, HSP90AA1, JUP
1.00E+00	2.61	HSP90AA1, TFRC, TNKS1BP1
1.00E+00	2.61	INPP5D, LTF, PRDX2
1.00E+00	2.61	FLOT1, G6PD, PATJ
1.00E+00	2.61	CCL5, PNPLA8, PRDX6
1.00E+00	2.61	EEF1A1, EEF2, EIF5A
1.00E+00	2.21	DSC1, DSG1, ITGB1, PECAM1
1.00E+00	2.21	BCAP31, HSPA1B, RPL11, UBQLN1
1.00E+00	2.59	HSPD1, LBP, THBS1
1.00E+00	3.51	PNPLA8, PRDX6
1.00E+00	3.51	CD36, ITGB3
1.00E+00	3.51	PLEK, SPTBN1
1.00E+00	3.51	FLNA, YWHAE
1.00E+00	3.51	CALM3, LTF
1.00E+00	2.19	CALM3, FLNA, G6PD, SRI
1.00E+00	1.98	CDC42, CFL1, ITGB1, RHOA, YWHAH
1.00E+00	1.98	CALM3, FLNA, G6PD, ITGB1, SRI
1.00E+00	1.22	ACTB, ANXA2, CCT2, CCT4, CCT6A, CCT7, CCT8, CD36, CSDE1, DAZAP1, DHX9, EIF5A, ENC
1.00E+00	1.84	GNAI2, ITGB3, NCL, PKM, RAB10, YWHAG
1.00E+00	2.56	BCAP31, HSPA1B, UBQLN1
1.00E+00	2.18	HSP90AA1, ITGB1, RTN4, VCL
1.00E+00	1.97	DAZAP1, HNRNPA2B1, HNRNPA3, HNRNPC, HNRNPU
1.00E+00	3.44	CCL5, GNAQ
1.00E+00	3.44	RAP1B, RPLP0
1.00E+00	3.44	HNRNPU, UBE2L3
1.00E+00	3.44	RTN3, RTN4
1.00E+00	3.44	CD36, LRP1
1.00E+00	2.54	GSTP1, PNPLA8, PTGS1
1.00E+00	2.54	ANXA2, FLNA, RPL11
1.00E+00	2.54	CAMP, CCL5, CD47
1.00E+00	2.54	ILK, PRDX1, RHOA
1.00E+00	1.72	CCL5, CD36, ITGB3, NCL, PECAM1, PKM, PTPRJ
1.00E+00	8.28	ANXA2
1.00E+00	8.28	RAC2
1.00E+00	8.28	GSTP1
1.00E+00	8.28	GNG5
1.00E+00	8.28	GNG5
1.00E+00	8.28	SRI
1.00E+00	8.28	TFRC
1.00E+00	8.28	THBS1
1.00E+00	8.28	NPM1
1.00E+00	8.28	G6PD
1.00E+00	8.28	LBP
1.00E+00	8.28	THBS1
1.00E+00	8.28	GNAI2
1.00E+00	8.28	ITGA6

1.00E+00	8.28	NPM1
1.00E+00	8.28	RAP1B
1.00E+00	8.28	CDC42
1.00E+00	8.28	ARPC5
1.00E+00	8.28	LTF
1.00E+00	8.28	KRT17
1.00E+00	8.28	ITGB1
1.00E+00	8.28	LRP1
1.00E+00	8.28	CDC42
1.00E+00	8.28	HSPA8
1.00E+00	8.28	MYADM
1.00E+00	8.28	XRCC5
1.00E+00	8.28	ITGB3
1.00E+00	8.28	CD9
1.00E+00	8.28	ITGB3
1.00E+00	8.28	ACTB
1.00E+00	8.28	GNAI2
1.00E+00	8.28	CD36
1.00E+00	8.28	CD36
1.00E+00	8.28	GP1BA
1.00E+00	8.28	LRP1
1.00E+00	8.28	MSN
1.00E+00	8.28	XRCC5
1.00E+00	8.28	XRCC5
1.00E+00	8.28	HSPA8
1.00E+00	8.28	HLA-A
1.00E+00	8.28	CYB5R3
1.00E+00	8.28	HSPA8
1.00E+00	8.28	RAB27B
1.00E+00	8.28	CALM3
1.00E+00	8.28	TLN1
1.00E+00	8.28	PKM
1.00E+00	8.28	DHX9
1.00E+00	8.28	HNRNPA2B1
1.00E+00	8.28	INPP5D
1.00E+00	8.28	HNRNPU
1.00E+00	2.52	BCAP31, CANX, UBQLN1
1.00E+00	1.94	CDC42, DSP, G6PD, HNRNPU, ITGB1
1.00E+00	3.38	HLA-A, HLA-B
1.00E+00	3.38	RTN3, RTN4
1.00E+00	3.38	CALM3, GNAI2
1.00E+00	3.38	ANXA2, ITGB3
1.00E+00	2.14	CYBB, ENO1, PGK1, UBQLN1
1.00E+00	2.14	CANX, CDC42, ITGB1, RHOA
1.00E+00	1.72	CCL5, CD36, ITGB3, NCL, PECAM1, PKM, PTPRJ
1.00E+00	2.49	JUP, RHOA, THBS1
1.00E+00	2.49	CFL1, RHOA, SPTBN1

1.00E+00	2.49	ANXA2, CD36, LRP1
1.00E+00	1.71	ARPC5, ATIC, B2M, RHOA, RTN4, SLC2A1, YWHAE
1.00E+00	1.71	JUP, KRT16, LRP1, PTPRJ, RHOA, THBS1, VCL
1.00E+00	2.13	CANX, CDC42, ITGB1, RHOA
1.00E+00	1.80	ADAM10, B2M, CANX, QSOX1, THBS1, TSPAN14
1.00E+00	3.32	CD9, RTN4
1.00E+00	3.32	TMEM109, XRCC5
1.00E+00	3.32	CCL5, LTF
1.00E+00	3.32	CDC42, RHOA
1.00E+00	3.32	CD36, HSP90AA1
1.00E+00	2.47	FLNA, ITGB1, YWHAE
1.00E+00	2.47	ACTB, RAC2, TPM4
1.00E+00	2.47	CALM3, CLTA, RAB27B
1.00E+00	2.47	CALM3, CLTA, RAB27B
1.00E+00	2.11	CYB5R3, G6PD, OSBPL2, SOD1
1.00E+00	2.11	ACTB, H2AC20, H2BC21, XRCC5
1.00E+00	1.91	CYB5R3, G6PD, OSBPL2, PLEK, SOD1
1.00E+00	2.45	GSTP1, PNPLA8, PTGS1
1.00E+00	2.45	KRT14, KRT16, KRT17
1.00E+00	2.45	KRT14, KRT16, KRT17
1.00E+00	2.45	CALM3, CCL5, LTF
1.00E+00	1.69	BCAP31, CLTA, FLNA, HLA-A, M6PR, RAB10, RAB27B
1.00E+00	3.26	GSTP1, HSPA1B
1.00E+00	3.26	PNPLA8, PTGS1
1.00E+00	3.26	ADAM10, MYH9
1.00E+00	3.26	CDC42, CFL1
1.00E+00	3.26	RAP1B, STOM
1.00E+00	1.90	ARL6IP5, GSTP1, PPIA, PRDX1, RHOA
1.00E+00	2.09	ACTB, PRDX2, RHOA, SOD1
1.00E+00	2.09	FLNA, INPP5D, ITGB3, TFRC
1.00E+00	7.59	GNAI2
1.00E+00	7.59	PRDX2
1.00E+00	7.59	HLA-B
1.00E+00	7.59	RHOA
1.00E+00	7.59	KRT2
1.00E+00	7.59	CALM3
1.00E+00	7.59	HSP90AA1
1.00E+00	7.59	ITGB3
1.00E+00	7.59	RCC2
1.00E+00	7.59	PLEK
1.00E+00	7.59	CDC42
1.00E+00	7.59	CDC42
1.00E+00	7.59	CAMP
1.00E+00	7.59	FSCN1
1.00E+00	7.59	DHX9
1.00E+00	7.59	DHX9
1.00E+00	7.59	GNAI2

1.00E+00	7.59	GNAI2
1.00E+00	7.59	SOD1
1.00E+00	7.59	YWHAH
1.00E+00	7.59	HSP90AA1
1.00E+00	7.59	CD59
1.00E+00	7.59	DHX9
1.00E+00	7.59	FLNA
1.00E+00	7.59	ADH5
1.00E+00	7.59	THBS1
1.00E+00	7.59	CD36
1.00E+00	7.59	G6PD
1.00E+00	7.59	HSPA1B
1.00E+00	7.59	XRCC5
1.00E+00	7.59	HNRNPU
1.00E+00	7.59	RAB27B
1.00E+00	7.59	HNRNPU
1.00E+00	7.59	RAB27B
1.00E+00	7.59	RCC2
1.00E+00	7.59	CALM3
1.00E+00	7.59	HSPA1B
1.00E+00	7.59	MSN
1.00E+00	7.59	ITGB1
1.00E+00	7.59	CD36
1.00E+00	7.59	FLNA
1.00E+00	7.59	HLA-B
1.00E+00	7.59	HSPA9
1.00E+00	7.59	ANXA2
1.00E+00	7.59	SRGN
1.00E+00	7.59	HSP90AA1
1.00E+00	7.59	HSPA8
1.00E+00	7.59	HSPA9
1.00E+00	7.59	XRCC5
1.00E+00	7.59	FCN1
1.00E+00	7.59	CALM3
1.00E+00	7.59	INPP5D
1.00E+00	7.59	YWHAH
1.00E+00	7.59	NCL
1.00E+00	7.59	PFN1
1.00E+00	7.59	HNRNPA2B1
1.00E+00	7.59	ACTB
1.00E+00	2.43	MATR3, NASP, RTN4
1.00E+00	2.43	CCL5, EIF5A, HSP90AA1
1.00E+00	1.89	ALDOA, B2M, KRT1, KRT10, OSBPL2
1.00E+00	2.08	ANXA2, GSDMA, OSBPL2, PFN1
1.00E+00	1.89	CDC42, HNRNPU, HSPA9, ITGB1, XRCC5
1.00E+00	3.20	HSPA9, YWHAE
1.00E+00	3.20	ITGB3, SOD1

1.00E+00	3.20	CALM3, GNAI2
1.00E+00	3.20	HSPA8, M6PR
1.00E+00	2.41	ALDOA, ATP5F1B, ENO1
1.00E+00	1.88	ENO1, G6PD, HSPA1B, PTPRJ, RTN4
1.00E+00	2.06	AACS, CCL5, S100A8, SRI
1.00E+00	2.38	ALDOA, ATP5F1B, ENO1
1.00E+00	2.38	BCAP31, HSPA1B, UBQLN1
1.00E+00	3.15	RAB10, SPTBN1
1.00E+00	3.15	RHOA, RTN4
1.00E+00	3.15	GSTP1, PTPRJ
1.00E+00	3.15	CYBB, SOD1
1.00E+00	3.15	CALM3, GNAI2
1.00E+00	3.15	PKM, THBS1
1.00E+00	3.15	CALM3, RAP1B
1.00E+00	3.15	STOM, YWHAH
1.00E+00	1.75	ADAM10, RPL8, RPLP0, RTN3, RTN4, SPTBN1
1.00E+00	1.31	ADH5, ATP5F1B, BCAP31, CALM3, CASP14, CLIC1, CYB5R3, GSR, GSTP1, HSP90AA1, HSPA
1.00E+00	2.05	B2M, HLA-A, HLA-B, RCC2
1.00E+00	2.36	NPM1, UBE2L3, UBQLN1
1.00E+00	1.66	ACTB, CALM3, GSTP1, LTF, PTPRJ, RHOA, THBS1
1.00E+00	1.86	HNRNPU, HSPA1B, NPM1, PATJ, RHOA
1.00E+00	2.03	AACS, CCL5, S100A8, SRI
1.00E+00	7.00	B2M
1.00E+00	7.00	ALDOA
1.00E+00	7.00	ATIC
1.00E+00	7.00	ATIC
1.00E+00	7.00	RTN4
1.00E+00	7.00	RPLP0
1.00E+00	7.00	THBS1
1.00E+00	7.00	MYL6
1.00E+00	7.00	YWHAH
1.00E+00	7.00	RAP1B
1.00E+00	7.00	CD9
1.00E+00	7.00	LTF
1.00E+00	7.00	LRP1
1.00E+00	7.00	ADAM10
1.00E+00	7.00	XRCC5
1.00E+00	7.00	NPM1
1.00E+00	7.00	CCL5
1.00E+00	7.00	LTF
1.00E+00	7.00	ADH5
1.00E+00	7.00	FLNA
1.00E+00	7.00	RAB10
1.00E+00	7.00	RHOA
1.00E+00	7.00	VIM
1.00E+00	7.00	FLNA
1.00E+00	7.00	CYBB

1.00E+00	7.00	TCP1
1.00E+00	7.00	YWHAZ
1.00E+00	7.00	RTN4
1.00E+00	7.00	ITGB1
1.00E+00	7.00	SRI
1.00E+00	7.00	HSPA9
1.00E+00	7.00	THBS1
1.00E+00	7.00	HSP90AA1
1.00E+00	7.00	CYBB
1.00E+00	7.00	BCAP31
1.00E+00	7.00	LRP1
1.00E+00	7.00	ENO1
1.00E+00	7.00	ACTB
1.00E+00	7.00	TNKS1BP1
1.00E+00	7.00	CLTA
1.00E+00	7.00	SPTBN1
1.00E+00	7.00	LGALS3BP
1.00E+00	7.00	NPM1
1.00E+00	7.00	ACTB
1.00E+00	7.00	RAB27B
1.00E+00	7.00	SLC2A1
1.00E+00	7.00	ACTB
1.00E+00	7.00	HSP90AA1
1.00E+00	7.00	EEF1A1
1.00E+00	7.00	MMRN1
1.00E+00	7.00	CANX
1.00E+00	7.00	ACTB
1.00E+00	7.00	HNRNPU
1.00E+00	7.00	ITGB3
1.00E+00	7.00	CCL5
1.00E+00	7.00	MYL12B
1.00E+00	7.00	NPM1
1.00E+00	7.00	DHX9
1.00E+00	7.00	LYZ
1.00E+00	7.00	INPP5D
1.00E+00	7.00	YWHAZ
1.00E+00	7.00	DHX9
1.00E+00	3.09	HSPD1, TFRC
1.00E+00	3.09	PNPLA8, PTGS1
1.00E+00	3.09	DAZAP1, DSG1
1.00E+00	3.09	DHX9, HNRNPU
1.00E+00	3.09	DHX9, HNRNPU
1.00E+00	3.09	OSBPL2, VDAC2
1.00E+00	1.35	ACTB, ANXA11, CALM3, CDC42, CFL1, FLNA, HNRNPU, HSPA1B, ITGB1, MYH9, NASP, NPM
1.00E+00	1.73	ARL6IP5, CD36, ITGB1, PNPLA8, SLC2A1, THBS1
1.00E+00	2.02	ITGB1, PKM, RHOA, THBS1
1.00E+00	1.73	ACTB, CDC42, FLNA, HNRNPU, HSPA1B, RHOA

1.00E+00	2.32	DHX9, G3BP1, XRCC5
1.00E+00	2.32	CALM3, CCL5, LTF
1.00E+00	3.04	HNRNPU, PTBP1
1.00E+00	3.04	FLNA, NCL
1.00E+00	3.04	SLC2A1, SLC2A3
1.00E+00	3.04	RAB10, SPTBN1
1.00E+00	3.04	CD59, GP1BA
1.00E+00	3.04	RAB27B, SOD1
1.00E+00	1.83	ATIC, RHOA, RTN4, SLC2A1, YWHAE
1.00E+00	1.71	ADAM10, RPL8, RPLP0, RTN3, RTN4, SPTBN1
1.00E+00	2.30	ADH5, CYBB, GSR
1.00E+00	1.82	CDC42, DSP, G6PD, HNRNPU, ITGB1
1.00E+00	2.99	RHOA, RTN4
1.00E+00	2.99	CD36, XRCC5
1.00E+00	2.99	ADAM10, ITGB3
1.00E+00	2.99	DHX9, HNRNPU
1.00E+00	2.28	ALDOA, ATP5F1B, ENO1
1.00E+00	6.50	MYH9
1.00E+00	6.50	XRCC5
1.00E+00	6.50	MYH9
1.00E+00	6.50	MSN
1.00E+00	6.50	THBS1
1.00E+00	6.50	HLA-B
1.00E+00	6.50	CDC42
1.00E+00	6.50	THBS1
1.00E+00	6.50	RTN4
1.00E+00	6.50	FLOT1
1.00E+00	6.50	CD36
1.00E+00	6.50	ATIC
1.00E+00	6.50	CCL5
1.00E+00	6.50	SRI
1.00E+00	6.50	ACTB
1.00E+00	6.50	INPP5D
1.00E+00	6.50	HSP90AA1
1.00E+00	6.50	HSPD1
1.00E+00	6.50	YWHAZ
1.00E+00	6.50	YWHAZ
1.00E+00	6.50	RAP1B
1.00E+00	6.50	ARL6IP5
1.00E+00	6.50	CD36
1.00E+00	6.50	CDC42
1.00E+00	6.50	CDC42
1.00E+00	6.50	CCL5
1.00E+00	6.50	YWHAE
1.00E+00	6.50	BCAP31
1.00E+00	6.50	XRCC5
1.00E+00	6.50	XRCC5

1.00E+00	6.50	RAC2
1.00E+00	6.50	GNAI2
1.00E+00	6.50	PFN1
1.00E+00	6.50	ITGB3
1.00E+00	6.50	NCL
1.00E+00	6.50	CD36
1.00E+00	6.50	RHOA
1.00E+00	6.50	ADH5
1.00E+00	6.50	TUBB
1.00E+00	6.50	CASP14
1.00E+00	1.98	NPM1, TMEM109, TNKS1BP1, XRCC5
1.00E+00	2.27	G6PD, HNRNPU, ITGB1
1.00E+00	2.94	CD226, TFRC
1.00E+00	2.94	CD226, TFRC
1.00E+00	2.94	CCL5, PTPRJ
1.00E+00	2.94	HNRNPU, UBE2L3
1.00E+00	2.94	RAB27B, SOD1
1.00E+00	2.94	DSG1, JUP
1.00E+00	1.42	ACTB, CALM3, CDC42, CFL1, FLNA, HNRNPU, HSPA1B, ITGB1, NASP, RCC2, RHOA, SPTBN1
1.00E+00	1.80	CDC42, HNRNPU, NPM1, RCC2, RHOA
1.00E+00	1.35	ALDOA, ATIC, ATP5F1B, ENO1, G6PD, GAPDH, HINT1, HSPA1B, HSPA8, LDHA, NANS, PGK
1.00E+00	1.68	B2M, CD226, CD36, HLA-A, HSPD1, TFRC
1.00E+00	2.90	RHOA, SOD1
1.00E+00	2.90	ARL6IP5, ITGB1
1.00E+00	2.90	HNRNPU, SRGN
1.00E+00	2.90	CD47, HSPD1
1.00E+00	2.90	CD47, HSPD1
1.00E+00	2.90	ADAM10, CCL5
1.00E+00	2.90	RTN3, RTN4
1.00E+00	2.90	DHX9, HSPA1B
1.00E+00	2.90	ITGB3, THBS1
1.00E+00	2.23	GSTP1, PNPLA8, PTGS1
1.00E+00	2.23	EIF5A, PLEK, SPTBN1
1.00E+00	1.78	ANXA2, GAPDH, LRP1, LTF, THBS1
1.00E+00	6.07	MYH9
1.00E+00	6.07	B2M
1.00E+00	6.07	B2M
1.00E+00	6.07	PLEK
1.00E+00	6.07	ITGB1
1.00E+00	6.07	ITGB1
1.00E+00	6.07	FSCN1
1.00E+00	6.07	INPP5D
1.00E+00	6.07	HNRNPA2B1
1.00E+00	6.07	CDC42
1.00E+00	6.07	DHX9
1.00E+00	6.07	MYL6
1.00E+00	6.07	PNPLA8

1.00E+00	6.07	CTSD
1.00E+00	6.07	G6PD
1.00E+00	6.07	NASP
1.00E+00	6.07	GSTP1
1.00E+00	6.07	KANSL3
1.00E+00	6.07	KANSL3
1.00E+00	6.07	LTF
1.00E+00	6.07	INPP5D
1.00E+00	6.07	SOD1
1.00E+00	6.07	TKT
1.00E+00	6.07	THBS1
1.00E+00	6.07	CLTA
1.00E+00	6.07	CD226
1.00E+00	6.07	SOD1
1.00E+00	6.07	ITGB1
1.00E+00	6.07	HSPA1B
1.00E+00	6.07	HNRNPU
1.00E+00	6.07	ADAM10
1.00E+00	6.07	ADAM10
1.00E+00	6.07	SLC2A1
1.00E+00	6.07	HSPA9
1.00E+00	6.07	VCL
1.00E+00	6.07	ACTB
1.00E+00	6.07	HSPA8
1.00E+00	6.07	CALM3
1.00E+00	6.07	EIF5A
1.00E+00	6.07	CYBB
1.00E+00	6.07	CALM3
1.00E+00	2.85	KRT10, KRT2
1.00E+00	2.85	PLEK, RAB10
1.00E+00	2.85	CD226, PTPRJ
1.00E+00	2.85	DAZAP1, SLC2A1
1.00E+00	1.93	BCAP31, HSPA1B, RPL11, UBQLN1
1.00E+00	2.19	RAP1B, RPLP0, SOD1
1.00E+00	1.92	ARL6IP5, GSTP1, PPIA, PRDX1
1.00E+00	1.49	CDC42, FLNA, FSCN1, HSP90AA1, ITGB1, PFN1, RAC2, RCC2, VCL
1.00E+00	1.66	ARL6IP5, G6PD, GSR, GSTP1, HSPA9, SOD1
1.00E+00	2.81	ITGB1, RAB10
1.00E+00	2.81	INPP5D, TFRC
1.00E+00	2.81	CD226, RAC2
1.00E+00	2.81	ILK, RHOA
1.00E+00	1.75	ARL6IP5, CLIC1, ITGB1, PCYOX1, VDAC2
1.00E+00	2.18	HSPA8, LRP1, M6PR
1.00E+00	2.18	AACS, PPIA, SRI
1.00E+00	2.16	ACTB, INPP5D, RHOA
1.00E+00	2.16	CCL5, PNPLA8, PRDX6
1.00E+00	2.76	HLA-A, HLA-B

1.00E+00	2.76	AACS, SRI
1.00E+00	2.76	HSPA1B, PRDX2
1.00E+00	2.76	INPP5D, LTF
1.00E+00	2.76	PNPLA8, PRDX6
1.00E+00	2.76	HSPA1B, PRDX2
1.00E+00	2.76	ITGB1, MYH9
1.00E+00	5.69	ANXA2
1.00E+00	5.69	CD59
1.00E+00	5.69	CD47
1.00E+00	5.69	ITGB3
1.00E+00	5.69	CDC42
1.00E+00	5.69	PNPLA8
1.00E+00	5.69	CD36
1.00E+00	5.69	LRBA
1.00E+00	5.69	CALM3
1.00E+00	5.69	ITGB3
1.00E+00	5.69	MYH9
1.00E+00	5.69	HSPA1B
1.00E+00	5.69	ADH5
1.00E+00	5.69	ENO1
1.00E+00	5.69	ITGB3
1.00E+00	5.69	HSPA9
1.00E+00	5.69	G6PD
1.00E+00	5.69	GP1BA
1.00E+00	5.69	RAB33B
1.00E+00	5.69	B2M
1.00E+00	5.69	ALDOA
1.00E+00	5.69	CANX
1.00E+00	5.69	ADH5
1.00E+00	5.69	VDAC2
1.00E+00	5.69	ATP2A3
1.00E+00	5.69	PNPLA8
1.00E+00	5.69	ADAM10
1.00E+00	5.69	GALE
1.00E+00	5.69	PKM
1.00E+00	5.69	NPM1
1.00E+00	5.69	ATP5F1B
1.00E+00	5.69	CCL5
1.00E+00	5.69	PFN1
1.00E+00	1.89	ARL6IP5, GSTP1, PPIA, PRDX1
1.00E+00	2.14	ATP5F1B, ITGB3, THBS1
1.00E+00	1.73	CALM3, HSP90AA1, RAB10, SPTBN1, STOM
1.00E+00	2.72	ITGB3, PECAM1
1.00E+00	2.72	ADAM10, RAB27B
1.00E+00	2.72	CDC42, FSCN1
1.00E+00	2.72	G6PD, PTBP1
1.00E+00	2.72	F11R, PECAM1

1.00E+00	2.72	STOM, YWHAH
1.00E+00	2.72	ITGB1, THBS1
1.00E+00	2.72	CLTA, LRP1
1.00E+00	1.56	JUP, KRT16, LRP1, PTPRJ, RHOA, THBS1, VCL
1.00E+00	2.12	CD59, FCN1, KRT1
1.00E+00	2.12	ACTB, HNRNPU, RCC2
1.00E+00	2.12	ACTB, F11R, PATJ
1.00E+00	1.50	CANX, CDC42, CYBB, FLNA, G3BP1, HSP90AA1, SLC2A3, SOD1
1.00E+00	1.86	CLIC1, CYBB, TMEM109, VDAC2
1.00E+00	1.86	CLIC1, CYBB, TMEM109, VDAC2
1.00E+00	2.68	ADH5, TKT
1.00E+00	2.68	HSPD1, TFRC
1.00E+00	2.68	ITGB1, YWHAE
1.00E+00	2.68	ACTB, HNRNPC
1.00E+00	2.11	ACTB, H2AC20, H2BC21
1.00E+00	1.46	CDC42, FLNA, FSCN1, HSP90AA1, ITGB1, PFN1, RAC2, RCC2, VCL
1.00E+00	5.36	SOD1
1.00E+00	5.36	SPN
1.00E+00	5.36	RHOA
1.00E+00	5.36	EIF5A
1.00E+00	5.36	RAB10
1.00E+00	5.36	RCC2
1.00E+00	5.36	SOD1
1.00E+00	5.36	SOD1
1.00E+00	5.36	PTPRJ
1.00E+00	5.36	SOD1
1.00E+00	5.36	ATIC
1.00E+00	5.36	ATIC
1.00E+00	5.36	YWHAE
1.00E+00	5.36	INPP5D
1.00E+00	5.36	ITGB3
1.00E+00	5.36	THBS1
1.00E+00	5.36	RHOA
1.00E+00	5.36	ITGB3
1.00E+00	5.36	CCL5
1.00E+00	5.36	SOD1
1.00E+00	5.36	ACTB
1.00E+00	5.36	NPM1
1.00E+00	5.36	ADAM10
1.00E+00	5.36	RCC2
1.00E+00	5.36	LRP1
1.00E+00	5.36	MSN
1.00E+00	5.36	ITGB1
1.00E+00	5.36	CLTA
1.00E+00	5.36	MSN
1.00E+00	5.36	ITGB3
1.00E+00	5.36	ACTB

1.00E+00	5.36	SACM1L
1.00E+00	5.36	LRP1
1.00E+00	5.36	PF4V1
1.00E+00	5.36	INPP5D
1.00E+00	1.85	CD9, MYADM, PRDX2, SPN
1.00E+00	2.09	ANK1, BCAP31, RAB1B
1.00E+00	2.09	ALDOA, ATP5F1B, ENO1
1.00E+00	1.61	DSP, FLOT1, ITGB1, MYL6, RHOA, SRI
1.00E+00	2.64	HSPD1, TFRC
1.00E+00	2.64	HSPD1, TFRC
1.00E+00	2.64	CCL5, PF4V1
1.00E+00	2.64	CCL5, SLC2A1
1.00E+00	2.61	CDC42, RHOA
1.00E+00	2.61	ACTB, TFRC
1.00E+00	2.61	GNAI2, NCL
1.00E+00	2.61	GNAI2, NCL
1.00E+00	2.61	ACTB, ITGB1
1.00E+00	2.06	BCAP31, HSPA1B, UBQLN1
1.00E+00	1.68	ANXA2, GSDMA, OSBPL2, PFN1, TLN1
1.00E+00	1.59	ANXA2, GAPDH, LRP1, LTF, RPL11, THBS1
1.00E+00	1.68	AACS, ANXA2, PPIA, RAB27B, SRI
1.00E+00	5.06	LBP
1.00E+00	5.06	FCER1G
1.00E+00	5.06	ITGB3
1.00E+00	5.06	NANS
1.00E+00	5.06	CD9
1.00E+00	5.06	CD36
1.00E+00	5.06	CALM3
1.00E+00	5.06	CD36
1.00E+00	5.06	CD36
1.00E+00	5.06	INPP5D
1.00E+00	5.06	THBS1
1.00E+00	5.06	THBS1
1.00E+00	5.06	RTN4
1.00E+00	5.06	HNRNPU
1.00E+00	5.06	HNRNPU
1.00E+00	5.06	GNAI2
1.00E+00	5.06	DHX9
1.00E+00	5.06	CCL5
1.00E+00	5.06	KRT17
1.00E+00	5.06	G6PD
1.00E+00	5.06	FLNA
1.00E+00	5.06	FLNA
1.00E+00	5.06	FLNA
1.00E+00	5.06	MYADM
1.00E+00	5.06	ITGB3
1.00E+00	5.06	ITGB3

1.00E+00	5.06	PLEK
1.00E+00	5.06	PPIA
1.00E+00	5.06	GNG5
1.00E+00	5.06	CDC42
1.00E+00	5.06	RPL11
1.00E+00	5.06	B2M
1.00E+00	5.06	HLA-A
1.00E+00	5.06	ATP5F1B
1.00E+00	5.06	RAB1B
1.00E+00	5.06	RCC2
1.00E+00	5.06	SPTBN1
1.00E+00	5.06	SACM1L
1.00E+00	5.06	ACTB
1.00E+00	2.04	NPM1, UBE2L3, UBQLN1
1.00E+00	2.57	HLA-A, RHOA
1.00E+00	2.57	GNAI2, RAP1B
1.00E+00	2.57	ITGB1, LRP1
1.00E+00	1.80	FCER1G, HSPD1, SPN, TFRC
1.00E+00	1.80	CYB5R3, HSPA9, RAC2, VDAC2
1.00E+00	1.16	ACTB, ANXA11, CASP14, CAVIN2, CCT4, CCT8, DAZAP1, DHX9, GNAI2, H2BC21, HINT1, HN
1.00E+00	1.79	CALM3, CLTA, RAB10, RAB27B
1.00E+00	2.53	ITGB1, ITGB3
1.00E+00	2.53	CDC42, RHOA
1.00E+00	2.53	CDC42, RHOA
1.00E+00	2.53	ADH5, YWHAZ
1.00E+00	2.53	ACTB, KANSL3
1.00E+00	2.53	DHX9, HSP90AA1
1.00E+00	2.01	CYB5R3, G6PD, SOD1
1.00E+00	1.65	CDC42, ITGB1, RAB10, RHOA, SPN
1.00E+00	1.65	DSC1, DSG1, ITGB1, MYADM, PECAM1
1.00E+00	1.16	ACTB, ALDOA, ANXA2, ATIC, ATP5F1B, CCT2, CCT4, CCT6A, CCT7, CCT8, CD36, CYB5R3, D
1.00E+00	4.79	B2M
1.00E+00	4.79	B2M
1.00E+00	4.79	THBS1
1.00E+00	4.79	HLA-B
1.00E+00	4.79	EIF5A
1.00E+00	4.79	GNAQ
1.00E+00	4.79	INPP5D
1.00E+00	4.79	ATIC
1.00E+00	4.79	MYH9
1.00E+00	4.79	VIM
1.00E+00	4.79	INPP5D
1.00E+00	4.79	FLOT1
1.00E+00	4.79	TTLL12
1.00E+00	4.79	GNG5
1.00E+00	4.79	CAVIN2
1.00E+00	4.79	LRP1

1.00E+00	4.79	CD36
1.00E+00	4.79	ADAM10
1.00E+00	4.79	CALM3
1.00E+00	4.79	RHOA
1.00E+00	4.79	SRI
1.00E+00	4.79	CLTA
1.00E+00	4.79	PCYOX1
1.00E+00	4.79	PCYOX1
1.00E+00	4.79	CLTA
1.00E+00	4.79	ATP2A3
1.00E+00	4.79	PPIA
1.00E+00	4.79	ATP2A3
1.00E+00	4.79	VDAC2
1.00E+00	2.50	ATP5F1B, THBS1
1.00E+00	2.50	SLC2A1, SLC2A3
1.00E+00	2.50	MYH9, SOD1
1.00E+00	2.50	CANX, FLOT1
1.00E+00	1.65	CCL5, F11R, ITGA6, ITGB1, RCC2
1.00E+00	1.15	ACTB, ANXA11, ANXA2, CASP14, CAVIN2, CCT4, CCT8, CFL1, DAZAP1, DHX9, EEF1A1, FLN
1.00E+00	1.99	CLIC1, ILK, LTF
1.00E+00	1.99	CAMP, CCL5, CD47
1.00E+00	2.46	ARL6IP5, ITGB1
1.00E+00	2.46	ITGB1, ITGB3
1.00E+00	2.46	CALM3, GNAI2
1.00E+00	1.63	CANX, CLTA, HNRNPU, RAB27B, SOD1
1.00E+00	1.76	G6PD, NANS, PGK1, PLEK
1.00E+00	1.97	RAP1B, RPLP0, SOD1
1.00E+00	1.97	ILK, PRDX1, RHOA
1.00E+00	1.97	CD9, ILK, SOD1
1.00E+00	1.54	CDC42, HNRNPU, NPM1, RCC2, RHOA, RPS15A
1.00E+00	2.43	CALM3, GNAI2
1.00E+00	2.43	GSTP1, LBP
1.00E+00	2.43	F11R, VDAC2
1.00E+00	1.96	TMEM109, TNKS1BP1, XRCC5
1.00E+00	1.96	HSP90AA1, TFRC, TNKS1BP1
1.00E+00	4.55	ADH5
1.00E+00	4.55	GNAI2
1.00E+00	4.55	RTN4
1.00E+00	4.55	SOD1
1.00E+00	4.55	ITGB1
1.00E+00	4.55	CD36
1.00E+00	4.55	SRGN
1.00E+00	4.55	ANXA2
1.00E+00	4.55	RAB33B
1.00E+00	4.55	CD226
1.00E+00	4.55	GNAI2
1.00E+00	4.55	NCL

1.00E+00	4.55	SPN
1.00E+00	4.55	NPM1
1.00E+00	4.55	KANSL3
1.00E+00	4.55	ANXA2
1.00E+00	4.55	LRP1
1.00E+00	4.55	SPN
1.00E+00	4.55	PLEK
1.00E+00	4.55	HNRNPU
1.00E+00	4.55	CDC42
1.00E+00	4.55	G6PD
1.00E+00	4.55	GSTP1
1.00E+00	4.55	THBS1
1.00E+00	4.55	GSTP1
1.00E+00	4.55	CCL5
1.00E+00	4.55	RHOA
1.00E+00	4.55	RTN4
1.00E+00	4.55	FLOT1
1.00E+00	4.55	ATP5F1B
1.00E+00	4.55	TPM4
1.00E+00	4.55	JUP
1.00E+00	4.55	GNG5
1.00E+00	1.94	CD9, ILK, SOD1
1.00E+00	1.94	CD9, ILK, SOD1
1.00E+00	2.40	RHOA, THBS1
1.00E+00	2.40	CALM3, YWHAE
1.00E+00	2.40	CD47, LBP
1.00E+00	2.40	ARL6IP5, ITGB1
1.00E+00	2.40	TCP1, TNKS1BP1
1.00E+00	1.39	ADAM10, ANPEP, CASP14, CPN1, CTSD, DCD, HINT1, LTF, PSMA6
1.00E+00	2.37	FCER1G, SPN
1.00E+00	1.92	CCL5, HLA-A, TFRC
1.00E+00	1.92	B2M, RTN4, YWHAH
1.00E+00	4.33	ANXA2
1.00E+00	4.33	CDC42
1.00E+00	4.33	LRP1
1.00E+00	4.33	CD9
1.00E+00	4.33	ATIC
1.00E+00	4.33	TFRC
1.00E+00	4.33	CALM3
1.00E+00	4.33	ITGB3
1.00E+00	4.33	ANXA2
1.00E+00	4.33	ACTB
1.00E+00	4.33	CD59
1.00E+00	4.33	NPM1
1.00E+00	4.33	CD9
1.00E+00	4.33	CALM3
1.00E+00	4.33	LTF

1.00E+00	4.33	HNRNPU
1.00E+00	4.33	INPP5D
1.00E+00	4.33	ITGB1
1.00E+00	4.33	GNAI2
1.00E+00	4.33	MYH9
1.00E+00	4.33	GNAI2
1.00E+00	4.33	CD36
1.00E+00	4.33	FCN1
1.00E+00	4.33	GNAI2
1.00E+00	4.33	CD47
1.00E+00	4.33	CALM3
1.00E+00	4.33	GSTP1
1.00E+00	4.33	GSTP1
1.00E+00	4.33	SOD1
1.00E+00	4.33	PTBP1
1.00E+00	4.33	ITGB3
1.00E+00	4.33	FLNA
1.00E+00	4.33	LTF
1.00E+00	4.33	PTBP1
1.00E+00	4.33	SOD1
1.00E+00	4.33	CD36
1.00E+00	4.33	F11R
1.00E+00	4.33	FLNA
1.00E+00	4.33	TCP1
1.00E+00	4.33	HSPA9
1.00E+00	4.33	ATP5F1B
1.00E+00	4.33	XRCC5
1.00E+00	4.33	CDC42
1.00E+00	4.33	FCN1
1.00E+00	4.33	HSP90AA1
1.00E+00	4.33	OSBPL2
1.00E+00	1.52	ARL6IP5, CCL5, G6PD, GAPDH, RHOA, SOD1
1.00E+00	1.13	ACTB, ALDOA, ANXA2, ATIC, ATP5F1B, CCT2, CCT4, CCT6A, CCT7, CCT8, CD36, CSDE1, DA
1.00E+00	1.71	GSTP1, OSBPL2, PNPLA8, PTGS1
1.00E+00	1.90	ANXA11, CALM3, THBS1
1.00E+00	2.34	CDC42, ILK
1.00E+00	2.34	PTPRJ, RTN4
1.00E+00	2.34	ENO1, XRCC5
1.00E+00	1.45	CALM3, FLOT1, GNAI2, ITGB1, RAP1B, YWHAG, YWHAH
1.00E+00	1.89	CCL5, ITGB3, PPIA
1.00E+00	1.69	ALDOA, ATIC, ATP5F1B, ENO1
1.00E+00	1.50	GSTP1, INPP5D, LBP, LTF, SRGN, THBS1
1.00E+00	1.45	CALM3, FLOT1, GNAI2, ITGB1, RAP1B, YWHAG, YWHAH
1.00E+00	1.38	ALDOA, ATIC, ATP5F1B, ENO1, G6PD, INPP5D, PLEK, SACM1L, TKT
1.00E+00	2.31	HSPD1, TFRC
1.00E+00	2.31	ILK, SOD1
1.00E+00	2.31	ANXA2, LRP1

1.00E+00	2.31	ANXA2, LRP1
1.00E+00	2.31	CDC42, CFL1
1.00E+00	1.45	BCAP31, CANX, HSPA1B, PSMA5, PSMA6, RPL11, UBQLN1
1.00E+00	1.68	CD9, INPP5D, PRDX2, SPN
1.00E+00	1.88	CCL5, HLA-A, TFRC
1.00E+00	1.88	AACS, CCL5, SRI
1.00E+00	1.88	HNRNPC, HNRNPU, XRCC5
1.00E+00	1.88	CYB5R3, G6PD, SOD1
1.00E+00	4.14	RHOA
1.00E+00	4.14	DHX9
1.00E+00	4.14	ITGB1
1.00E+00	4.14	CFL1
1.00E+00	4.14	ACTB
1.00E+00	4.14	CD36
1.00E+00	4.14	SOD1
1.00E+00	4.14	NPM1
1.00E+00	4.14	CD36
1.00E+00	4.14	LRP1
1.00E+00	4.14	HNRNPU
1.00E+00	4.14	CCL5
1.00E+00	4.14	HNRNPU
1.00E+00	4.14	CD36
1.00E+00	4.14	SOD1
1.00E+00	4.14	NCL
1.00E+00	4.14	ILK
1.00E+00	4.14	ENO1
1.00E+00	4.14	HSPA9
1.00E+00	4.14	VIM
1.00E+00	4.14	DHX9
1.00E+00	4.14	CALM3
1.00E+00	4.14	OSBPL2
1.00E+00	1.44	GNAQ, ITGB1, ITGB3, NPM1, TMEM109, TNKS1BP1, XRCC5
1.00E+00	2.28	BCAP31, UBQLN1
1.00E+00	2.28	HNRNPU, HSPA9
1.00E+00	2.28	F11R, GNG5
1.00E+00	1.49	ALDOA, GAPDH, RCC2, S100A8, S100A9, TTLL12
1.00E+00	1.12	ACTB, ALDOA, ANXA2, ATIC, ATP5F1B, CCT2, CCT4, CCT6A, CCT7, CCT8, CD36, CSDE1, CYI
1.00E+00	1.85	B2M, RTN4, YWHAH
1.00E+00	1.85	GNAI2, RAB33B, RAP1B
1.00E+00	1.48	CCL5, F11R, ITGA6, ITGB1, RCC2, SOD1
1.00E+00	2.25	LTF, SRGN
1.00E+00	2.25	G6PD, HNRNPU
1.00E+00	2.25	LRBA, UBQLN1
1.00E+00	2.25	PLEK, RAB10
1.00E+00	1.48	ANXA2, B2M, ITGB1, RTN4, XRCC5, YWHAH
1.00E+00	1.84	INPP5D, ITGB1, PTPRJ
1.00E+00	1.84	ILK, ITGB3, PECAM1

1.00E+00	1.43	CDC42, G3BP1, GNAQ, ILK, JUP, LRP1, RHOA
1.00E+00	3.96	HSPD1
1.00E+00	3.96	TCP1
1.00E+00	3.96	CDC42
1.00E+00	3.96	SOD1
1.00E+00	3.96	GNAI2
1.00E+00	3.96	FSCN1
1.00E+00	3.96	LTF
1.00E+00	3.96	MSN
1.00E+00	3.96	ITGB3
1.00E+00	3.96	FCER1G
1.00E+00	3.96	THBS1
1.00E+00	3.96	SPN
1.00E+00	3.96	ITGB1
1.00E+00	3.96	PPIA
1.00E+00	3.96	CD36
1.00E+00	3.96	CCL5
1.00E+00	3.96	HSPA1B
1.00E+00	3.96	HNRNPC
1.00E+00	3.96	CD36
1.00E+00	3.96	CALM3
1.00E+00	3.96	FLOT1
1.00E+00	3.96	RHOA
1.00E+00	3.96	SF3A3
1.00E+00	3.96	ARL6IP5
1.00E+00	3.96	THBS1
1.00E+00	3.96	DHX9
1.00E+00	3.96	THBS1
1.00E+00	3.96	MYH9
1.00E+00	3.96	PNPLA8
1.00E+00	3.96	ATP5F1B
1.00E+00	3.96	PTGS1
1.00E+00	3.96	FCER1G
1.00E+00	3.96	ATP5F1B
1.00E+00	3.96	ATP5F1B
1.00E+00	3.96	ATP5F1B
1.00E+00	3.96	ATP2A3
1.00E+00	1.48	ADAM10, CANX, CLTA, HSPA8, ITGB1, ITGB3
1.00E+00	2.22	ACTB, ITGB3
1.00E+00	2.22	MYH9, RAC2
1.00E+00	2.22	ANXA2, CD36
1.00E+00	2.22	PLEK, RAB10
1.00E+00	2.22	MYH9, RAC2
1.00E+00	2.22	CSDE1, G3BP1
1.00E+00	1.65	CALM3, CLTA, RAB10, RAB27B
1.00E+00	1.83	BCAP31, HLA-A, RAB27B
1.00E+00	1.42	CDC42, G3BP1, GNAQ, ILK, JUP, LRP1, RHOA

1.00E+00	1.64	BCAP31, HSPA1B, RPL11, UBQLN1
1.00E+00	1.53	CD36, CLIC1, SLC2A1, SLC2A3, VDAC2
1.00E+00	1.81	CYBB, GNAI2, TFRC
1.00E+00	1.81	CYB5R3, G6PD, SOD1
1.00E+00	1.81	FLNA, G3BP1, SLC2A3
1.00E+00	2.20	CD36, LRP1
1.00E+00	1.53	G6PD, LTF, PFN1, RHOA, YWHAZ
1.00E+00	1.37	ANXA2, FLNA, GP1BA, GP1BB, LTF, PRDX1, RAB33B, RHOA
1.00E+00	1.80	CANX, ITGB1, ITGB3
1.00E+00	3.79	RHOA
1.00E+00	3.79	RHOA
1.00E+00	3.79	YWHAH
1.00E+00	3.79	RTN4
1.00E+00	3.79	RTN4
1.00E+00	3.79	BCAP31
1.00E+00	3.79	ATIC
1.00E+00	3.79	RHOA
1.00E+00	3.79	HLA-A
1.00E+00	3.79	PTPRJ
1.00E+00	3.79	BCAP31
1.00E+00	3.79	DHX9
1.00E+00	3.79	HSPA1B
1.00E+00	3.79	GNAI2
1.00E+00	3.79	DHX9
1.00E+00	3.79	RAP1B
1.00E+00	3.79	ITGB1
1.00E+00	3.79	TPM4
1.00E+00	3.79	CANX
1.00E+00	3.79	HNRNPU
1.00E+00	3.79	PTGS1
1.00E+00	3.79	GAPDH
1.00E+00	3.79	OSBPL2
1.00E+00	1.62	CALM3, LTF, RHOA, THBS1
1.00E+00	2.17	CD36, THBS1
1.00E+00	2.17	B2M, SOD1
1.00E+00	2.17	ANXA2, RAB27B
1.00E+00	1.79	H2BC21, NASP, NPM1
1.00E+00	1.62	AACS, CCL5, S100A8, SRI
1.00E+00	1.21	DAZAP1, DHX9, HNRNPA2B1, HNRNPA3, HNRNPC, HNRNPF, HNRNPH1, HNRNPH3, HNRN
1.00E+00	1.78	FLNA, ITGA6, S100A9
1.00E+00	1.78	CALM3, NPM1, RCC2
1.00E+00	2.14	ADH5, LRP1
1.00E+00	2.14	CD36, ITGB3
1.00E+00	2.14	INPP5D, SPN
1.00E+00	2.14	CFL1, HSPA8
1.00E+00	2.14	CALM3, HNRNPU
1.00E+00	2.14	PTPRJ, VCL

1.00E+00	2.14	CYB5R3, CYBB
1.00E+00	1.30	B2M, CD9, GSTP1, HSPA1B, ILK, INPP5D, NPM1, PTPRJ, RHOA, SPN, THBS1
1.00E+00	1.60	ANXA2, ATP2A3, GAPDH, LTF
1.00E+00	3.64	YWHAH
1.00E+00	3.64	ATP5F1B
1.00E+00	3.64	CALM3
1.00E+00	3.64	ENO1
1.00E+00	3.64	GNAI2
1.00E+00	3.64	RTN4
1.00E+00	3.64	HSPA1B
1.00E+00	3.64	ITGB3
1.00E+00	3.64	CD9
1.00E+00	3.64	SRI
1.00E+00	3.64	CD226
1.00E+00	3.64	PTPRJ
1.00E+00	3.64	ITGB1
1.00E+00	3.64	PECAM1
1.00E+00	3.64	ITGB3
1.00E+00	3.64	ENO1
1.00E+00	3.64	RAB1B
1.00E+00	3.64	THBS1
1.00E+00	3.64	RHOA
1.00E+00	3.64	LRP1
1.00E+00	3.64	SOD1
1.00E+00	3.64	CD59
1.00E+00	3.64	CTSD
1.00E+00	3.64	GSTP1
1.00E+00	3.64	CCL5
1.00E+00	3.64	HNRNPF
1.00E+00	3.64	ANK1
1.00E+00	3.64	INPP5D
1.00E+00	3.64	ANPEP
1.00E+00	3.64	HSPA8
1.00E+00	1.29	ATP5F1B, CALM3, CYB5R3, HSPA9, HSPD1, LDHB, PNPLA8, RAC2, SOD1, TMX2, VDAC2
1.00E+00	2.12	HSPD1, TFRC
1.00E+00	2.12	ANXA2, LRP1
1.00E+00	2.12	INPP5D, SPN
1.00E+00	2.12	PLEK, SPTBN1
1.00E+00	1.60	HSP90AA1, ITGB1, RTN4, VCL
1.00E+00	1.59	ALDOA, ATIC, ATP5F1B, ENO1
1.00E+00	1.49	INPP5D, PLEK, PNPLA8, PRDX6, SACM1L
1.00E+00	2.10	FLNA, SOD1
1.00E+00	1.74	GAPDH, QSOX1, UBQLN1
1.00E+00	1.74	GSTP1, PNPLA8, PTGS1
1.00E+00	3.50	FLOT1
1.00E+00	3.50	CCL5
1.00E+00	3.50	ATIC

1.00E+00	3.50	LRP1
1.00E+00	3.50	CD36
1.00E+00	3.50	RHOA
1.00E+00	3.50	GSTP1
1.00E+00	3.50	FCER1G
1.00E+00	3.50	VIM
1.00E+00	3.50	SOD1
1.00E+00	3.50	SRI
1.00E+00	3.50	HLA-A
1.00E+00	3.50	PTPRJ
1.00E+00	3.50	RPL11
1.00E+00	3.50	ITGB3
1.00E+00	3.50	CD226
1.00E+00	3.50	ATIC
1.00E+00	3.50	FLOT1
1.00E+00	3.50	GNAI2
1.00E+00	3.50	CD47
1.00E+00	3.50	KANSL3
1.00E+00	3.50	ARL6IP5
1.00E+00	3.50	BCAP31
1.00E+00	3.50	GNAI2
1.00E+00	3.50	FSCN1
1.00E+00	3.50	SRI
1.00E+00	3.50	ACTB
1.00E+00	3.50	ACTB
1.00E+00	3.50	ATP5F1B
1.00E+00	3.50	AACS
1.00E+00	3.50	CTSD
1.00E+00	1.73	HINT1, PNPLA8, PRDX6
1.00E+00	1.73	CD36, OSBPL2, SLC2A1
1.00E+00	2.07	ADH5, LRP1
1.00E+00	2.07	G6PD, PGK1
1.00E+00	2.07	RAP1B, RPLP0
1.00E+00	2.07	G6PD, HNRNPU
1.00E+00	2.07	CCL5, PF4V1
1.00E+00	2.07	OSBPL2, VDAC2
1.00E+00	1.42	ADAM10, ITGB1, MATR3, MYH9, NASP, RTN4
1.00E+00	1.57	LTF, PF4V1, PPIA, THBS1
1.00E+00	1.72	HSPA8, LRP1, M6PR
1.00E+00	1.72	INPP5D, PLEK, SACM1L
1.00E+00	1.34	ADH5, CYB5R3, G6PD, LDHA, LDHB, OSBPL2, PLEK, SOD1
1.00E+00	1.37	CYBB, G6PD, GNAI2, HSPA8, SLC2A1, SOD1, TFRC
1.00E+00	2.05	CALM3, RAP1B
1.00E+00	2.05	CDC42, CFL1
1.00E+00	2.05	LTF, THBS1
1.00E+00	2.05	ACTB, KANSL3
1.00E+00	2.05	CLIC1, VDAC2

1.00E+00	1.11	ACTB, ALDOA, ANXA2, ATIC, ATP5F1B, CCT2, CCT4, CCT6A, CCT7, CCT8, CD36, CSDE1, DA
1.00E+00	1.13	ACTB, ANXA2, CD36, CSDE1, DAZAP1, DHX9, EIF5A, ENO1, FLNA, FLOT1, HINT1, HNRNPA
1.00E+00	1.55	ARL6IP5, GSTP1, PPIA, PRDX1
1.00E+00	3.37	ITGB1
1.00E+00	3.37	CALM3
1.00E+00	3.37	GNAI2
1.00E+00	3.37	VIM
1.00E+00	3.37	SOD1
1.00E+00	3.37	RCC2
1.00E+00	3.37	CD47
1.00E+00	3.37	ANXA2
1.00E+00	3.37	RAP1B
1.00E+00	3.37	PLEK
1.00E+00	3.37	KRT17
1.00E+00	3.37	FLOT1
1.00E+00	3.37	TFRC
1.00E+00	3.37	PNPLA8
1.00E+00	3.37	CDC42
1.00E+00	3.37	STOM
1.00E+00	3.37	MSN
1.00E+00	3.37	THBS1
1.00E+00	3.37	PKM
1.00E+00	3.37	ATP2A3
1.00E+00	3.37	ACTB
1.00E+00	3.37	RAB27B
1.00E+00	3.37	PTPRJ
1.00E+00	3.37	GSDMA
1.00E+00	2.03	STOM, YWHAH
1.00E+00	2.03	CDC42, CFL1
1.00E+00	2.03	AACS, SRI
1.00E+00	1.70	GSTP1, PNPLA8, PTGS1
1.00E+00	1.70	FLOT1, MYL6, RHOA
1.00E+00	2.00	ARL6IP5, ITGB1
1.00E+00	2.00	ARL6IP5, ITGB1
1.00E+00	2.00	CDC42, RHOA
1.00E+00	2.00	GNAI2, RHOA
1.00E+00	2.00	CCL5, TCP1
1.00E+00	1.53	ALDOA, ATIC, ATP5F1B, ENO1
1.00E+00	1.68	INPP5D, PRDX2, SPN
1.00E+00	1.68	CANX, ITGB1, ITGB3
1.00E+00	1.39	AACS, ADH5, CD36, GSTP1, PNPLA8, PTGS1
1.00E+00	3.25	TFRC
1.00E+00	3.25	GSTP1
1.00E+00	3.25	THBS1
1.00E+00	3.25	CD47
1.00E+00	3.25	PPIA
1.00E+00	3.25	BCAP31

1.00E+00	3.25	PPIA
1.00E+00	3.25	GSTP1
1.00E+00	3.25	THBS1
1.00E+00	3.25	CCL5
1.00E+00	3.25	GNAI2
1.00E+00	3.25	FLNA
1.00E+00	3.25	THBS1
1.00E+00	3.25	BCAP31
1.00E+00	3.25	SF3A3
1.00E+00	3.25	CLTA
1.00E+00	3.25	CANX
1.00E+00	3.25	PFN1
1.00E+00	3.25	CPN1
1.00E+00	3.25	CCL5
1.00E+00	3.25	PLEK
1.00E+00	3.25	VDAC2
1.00E+00	3.25	LRP1
1.00E+00	1.19	ALDOA, BCAP31, CCT2, CCT4, CCT7, CCT8, CD9, CSDE1, DAZAP1, DSG1, ITGB1, ITGB3, KR
1.00E+00	1.98	SOD1, TUBB
1.00E+00	1.52	CALM3, HSP90AA1, HSPD1, VDAC2
1.00E+00	1.44	CYB5R3, G6PD, OSBPL2, SOD1, YWHAH
1.00E+00	1.96	GNAI2, RHOA
1.00E+00	1.65	CD59, GP1BA, RAB27B
1.00E+00	3.14	GNAQ
1.00E+00	3.14	RAC2
1.00E+00	3.14	HSPA9
1.00E+00	3.14	HSPA9
1.00E+00	3.14	RAB33B
1.00E+00	3.14	PCBP1
1.00E+00	3.14	SRGN
1.00E+00	3.14	UBE2L3
1.00E+00	3.14	PPIA
1.00E+00	3.14	CCL5
1.00E+00	3.14	CANX
1.00E+00	3.14	ACTB
1.00E+00	3.14	CLTA
1.00E+00	3.14	CCL5
1.00E+00	3.14	CALM3
1.00E+00	1.17	AACS, ADAM10, CALM3, CCL5, CDC42, FLNA, FLOT1, G3BP1, GNAI2, GNAQ, ILK, ITGB1, JU
1.00E+00	1.51	CANX, HNRNPU, RAB27B, SOD1
1.00E+00	1.18	ALDOA, BCAP31, CCT2, CCT4, CCT7, CCT8, CD9, CSDE1, DAZAP1, DSG1, ITGB1, ITGB3, KR
1.00E+00	1.94	CCL5, ITGB3
1.00E+00	1.26	AACS, CYB5R3, G6PD, GSTP1, INPP5D, OSBPL2, PNPLA8, PTGS1, SACM1L, SOD1
1.00E+00	1.18	AACS, ADH5, ATP5F1B, CD36, CDC42, CYB5R3, G6PD, GSTP1, INPP5D, LRP1, OSBPL2, PLE
1.00E+00	1.92	ADAM10, TSPAN14
1.00E+00	1.92	HNRNPC, HNRNPU
1.00E+00	1.92	CSDE1, PSMA6

1.00E+00	1.92	GNAQ, PTGS1
1.00E+00	1.50	FLNA, GNAI2, GNAQ, ITGB3
1.00E+00	1.63	FCN1, HLA-A, HLA-B
1.00E+00	1.63	LTF, PF4V1, THBS1
1.00E+00	3.03	PPIA
1.00E+00	3.03	FCN1
1.00E+00	3.03	HSP90AA1
1.00E+00	3.03	HLA-B
1.00E+00	3.03	G6PD
1.00E+00	3.03	G6PD
1.00E+00	3.03	HMBS
1.00E+00	3.03	KRT17
1.00E+00	3.03	PPIA
1.00E+00	3.03	THBS1
1.00E+00	3.03	SOD1
1.00E+00	3.03	CCL5
1.00E+00	3.03	RHOA
1.00E+00	3.03	FLNA
1.00E+00	3.03	G6PD
1.00E+00	3.03	ITGB3
1.00E+00	3.03	G6PD
1.00E+00	3.03	G6PD
1.00E+00	3.03	SRGN
1.00E+00	3.03	PTPRJ
1.00E+00	3.03	PTPRJ
1.00E+00	3.03	HNRNPU
1.00E+00	3.03	JUP
1.00E+00	3.03	AACS
1.00E+00	3.03	VDAC2
1.00E+00	3.03	VDAC2
1.00E+00	3.03	SLC2A1
1.00E+00	1.62	CALM3, HNRNPU, NPM1
1.00E+00	1.90	GSTP1, LBP
1.00E+00	1.90	ARL6IP5, ITGB1
1.00E+00	1.90	ATP2A3, ATP5F1B
1.00E+00	1.48	ALDOA, ATIC, ATP5F1B, ENO1
1.00E+00	1.88	GSTP1, LBP
1.00E+00	1.88	ANXA2, CD151
1.00E+00	1.88	H2AC20, H2BC21
1.00E+00	1.25	ATP5F1B, CALM3, CYB5R3, HSPA9, HSPD1, LDHB, PNPLA8, RAC2, TMX2, VDAC2
1.00E+00	1.60	HLA-A, RHOA, SPN
1.00E+00	1.47	ANXA2, GAPDH, LTF, THBS1
1.00E+00	1.47	CD226, HLA-A, INPP5D, PTPRJ
1.00E+00	2.93	CD226
1.00E+00	2.93	FCN1
1.00E+00	2.93	NASP
1.00E+00	2.93	NPM1

1.00E+00	2.93	XRCC5
1.00E+00	2.93	CANX
1.00E+00	2.93	HSPA1B
1.00E+00	2.93	CALM3
1.00E+00	2.93	SOD1
1.00E+00	2.93	PPIA
1.00E+00	2.93	NASP
1.00E+00	2.93	RAC2
1.00E+00	2.93	CDC42
1.00E+00	2.93	YWHAE
1.00E+00	2.93	PNPLA8
1.00E+00	2.93	UBE2L3
1.00E+00	2.93	RHOA
1.00E+00	2.93	PNPLA8
1.00E+00	2.93	CYBB
1.00E+00	2.93	DSC1
1.00E+00	2.93	XRCC5
1.00E+00	2.93	CCL5
1.00E+00	2.93	WBP11
1.00E+00	1.59	GNAI2, RAB33B, RAP1B
1.00E+00	1.46	AACS, CCL5, S100A8, SRI
1.00E+00	1.58	ADH5, CYBB, GSR
1.00E+00	1.58	FLOT1, MYL6, RHOA
1.00E+00	1.84	ADH5, LRP1
1.00E+00	1.84	CLIC1, PCYOX1
1.00E+00	1.84	ACTB, KANSL3
1.00E+00	1.84	CLTA, M6PR
1.00E+00	1.84	ACTB, KANSL3
1.00E+00	1.84	OSBPL2, VDAC2
1.00E+00	1.45	G6PD, GAPDH, RHOA, SOD1
1.00E+00	2.84	ITGB1
1.00E+00	2.84	ANXA2
1.00E+00	2.84	THBS1
1.00E+00	2.84	ITGB3
1.00E+00	2.84	TCP1
1.00E+00	2.84	ARL6IP5
1.00E+00	2.84	PCYOX1
1.00E+00	2.84	INPP5D
1.00E+00	2.84	RHOA
1.00E+00	2.84	NCL
1.00E+00	2.84	GSTP1
1.00E+00	2.84	PTPRJ
1.00E+00	2.84	HSP90AA1
1.00E+00	2.84	SOD1
1.00E+00	2.84	ARPC5
1.00E+00	2.84	ACTB
1.00E+00	2.84	RPLP0

1.00E+00	2.84	GNAI2
1.00E+00	2.84	GNAI2
1.00E+00	2.84	THBS1
1.00E+00	2.84	THBS1
1.00E+00	2.84	HLA-A
1.00E+00	2.84	YWHAG
1.00E+00	1.57	HSP90AA1, RHOA, YWHAE
1.00E+00	1.38	G3BP1, GNAQ, ILK, JUP, LRP1
1.00E+00	1.10	ACTB, ALDOA, ANXA2, ATIC, ATP5F1B, CCT2, CCT4, CCT6A, CCT7, CCT8, CD36, DHX9, EIF5
1.00E+00	1.44	AACS, CCL5, PPIA, SRI
1.00E+00	1.80	HSPA1B, INPP5D
1.00E+00	1.80	G6PD, SOD1
1.00E+00	1.80	RHOA, YWHAH
1.00E+00	1.80	LTF, SRGN
1.00E+00	1.55	AACS, CCL5, SRI
1.00E+00	2.76	TNKS1BP1
1.00E+00	2.76	RAB33B
1.00E+00	2.76	ATIC
1.00E+00	2.76	GNAQ
1.00E+00	2.76	ANXA2
1.00E+00	2.76	RTN4
1.00E+00	2.76	SPTBN1
1.00E+00	2.76	RAC2
1.00E+00	2.76	F11R
1.00E+00	2.76	PTPRJ
1.00E+00	2.76	GNAI2
1.00E+00	2.76	ATIC
1.00E+00	2.76	RTN4
1.00E+00	2.76	TUBB
1.00E+00	2.76	CD36
1.00E+00	2.76	FLNA
1.00E+00	2.76	STOM
1.00E+00	2.76	CD36
1.00E+00	2.76	YWHAZ
1.00E+00	2.76	DHX9
1.00E+00	1.29	CYBB, G6PD, GNAI2, HSPA8, SLC2A1, SOD1, TFRC
1.00E+00	1.54	ANXA2, GAPDH, LTF
1.00E+00	1.79	ARL6IP5, ITGB1
1.00E+00	1.79	RAB10, SPTBN1
1.00E+00	1.09	ACTB, ALDOA, ANXA2, ATIC, ATP5F1B, CCT2, CCT4, CCT6A, CCT7, CCT8, CD36, DHX9, EIF5
1.00E+00	1.42	G3BP1, GNAQ, ILK, JUP
1.00E+00	1.54	GSTP1, PPIA, PTPRJ
1.00E+00	1.09	ACTB, ALDOA, ANXA2, ATIC, ATP5F1B, CCT2, CCT4, CCT6A, CCT7, CCT8, CD36, DHX9, EIF5
1.00E+00	1.77	LTF, SRGN
1.00E+00	1.77	MSN, RPLP0
1.00E+00	2.68	DAZAP1
1.00E+00	2.68	HMBS

1.00E+00	2.68	QSOX1
1.00E+00	2.68	FCER1G
1.00E+00	2.68	FCER1G
1.00E+00	2.68	HMBS
1.00E+00	2.68	ITGB1
1.00E+00	2.68	CALM3
1.00E+00	2.68	GNG5
1.00E+00	2.68	XRCC5
1.00E+00	2.68	XRCC5
1.00E+00	2.68	ADAM10
1.00E+00	2.68	B2M
1.00E+00	1.41	CLTA, FLNA, M6PR, RAB10
1.00E+00	1.75	ARL6IP5, ITGB1
1.00E+00	1.75	CD36, F11R
1.00E+00	1.75	FLOT1, PTPRJ
1.00E+00	1.75	ARPC5, XRCC5
1.00E+00	1.52	AACS, CCL5, SRI
1.00E+00	1.52	GSTP1, ITGB3, TFRC
1.00E+00	1.20	DHX9, HNRNPA2B1, HNRNPU, HSPA1B, MYH9, NPM1, PCBP1, SF3A3, SH3BGRL3, SPN, TK
1.00E+00	1.51	GSTP1, KRT1, SOD1
1.00E+00	1.51	ACTB, INPP5D, RHOA
1.00E+00	1.51	ACTB, INPP5D, RHOA
1.00E+00	1.51	ADAM10, ANPEP, CPN1
1.00E+00	1.73	ITGB1, VIM
1.00E+00	1.73	ACTB, HNRNPU
1.00E+00	2.60	LRBA
1.00E+00	2.60	OSBPL2
1.00E+00	2.60	G6PD
1.00E+00	2.60	ADAM10
1.00E+00	2.60	RAB1B
1.00E+00	2.60	HSPA1B
1.00E+00	2.60	LRP1
1.00E+00	2.60	HSPD1
1.00E+00	2.60	PNPLA8
1.00E+00	2.60	INPP5D
1.00E+00	2.60	ADAM10
1.00E+00	2.60	VIM
1.00E+00	2.60	RPLP0
1.00E+00	2.60	ITGB1
1.00E+00	2.60	YWHAH
1.00E+00	2.60	CDC42
1.00E+00	2.60	VDAC2
1.00E+00	2.60	ENO1
1.00E+00	2.60	PCYOX1
1.00E+00	2.60	PCYOX1
1.00E+00	2.60	OSBPL2
1.00E+00	2.60	ATIC

1.00E+00	2.60	CCL5
1.00E+00	2.60	UBE2L3
1.00E+00	1.50	CASP14, CTSD, HINT1
1.00E+00	1.72	HSPD1, VDAC2
1.00E+00	1.72	CALM3, GNAI2
1.00E+00	1.72	PLEK, SOD1
1.00E+00	1.72	ILK, JUP
1.00E+00	1.72	B2M, NPM1
1.00E+00	1.49	CLIC1, PCYOX1, VDAC2
1.00E+00	1.49	GSTP1, PTPRJ, THBS1
1.00E+00	2.53	CDC42
1.00E+00	2.53	GNAI2
1.00E+00	2.53	NANS
1.00E+00	2.53	NCL
1.00E+00	2.53	GNAI2
1.00E+00	2.53	CD36
1.00E+00	2.53	CD36
1.00E+00	2.53	HSP90AA1
1.00E+00	2.53	RPL11
1.00E+00	2.53	RHOA
1.00E+00	2.53	CCL5
1.00E+00	2.53	FLNA
1.00E+00	2.53	RAB1B
1.00E+00	1.49	ANXA2, GAPDH, LTF
1.00E+00	1.32	HSPD1, INPP5D, ITGB1, PTPRJ, TFRC
1.00E+00	1.32	ACTB, DHX9, NPM1, PSMA6, YWHAH
1.00E+00	1.38	NCL, RPL11, RPS15, WBP11
1.00E+00	1.69	SLC2A1, SLC2A3
1.00E+00	1.69	NPM1, RCC2
1.00E+00	1.07	ACTB, ALDOA, ANXA2, ATIC, ATP5F1B, CCT2, CCT4, CCT6A, CCT7, CCT8, CD36, CSDE1, DA
1.00E+00	1.21	BCAP31, CANX, HSPA1B, PCYOX1, PSMA5, PSMA6, RPL11, UBE2L3, UBQLN1
1.00E+00	2.46	CD47
1.00E+00	2.46	HSPA1B
1.00E+00	2.46	G6PD
1.00E+00	2.46	INPP5D
1.00E+00	2.46	G6PD
1.00E+00	2.46	ITGB1
1.00E+00	2.46	THBS1
1.00E+00	2.46	SPN
1.00E+00	2.46	NPM1
1.00E+00	2.46	TFRC
1.00E+00	2.46	G6PD
1.00E+00	2.46	ADAM10
1.00E+00	2.46	ITGB1
1.00E+00	2.46	DHX9
1.00E+00	2.46	PPIA
1.00E+00	1.24	BCAP31, CANX, HSPA1B, PSMA5, PSMA6, RPL11, UBQLN1

1.00E+00	1.67	CALM3, RAP1B
1.00E+00	1.67	CALM3, TNKS1BP1
1.00E+00	1.67	ARPC5, YWHAE
1.00E+00	1.67	CD36, GSTP1
1.00E+00	1.67	CD36, GSTP1
1.00E+00	1.67	MYH9, MYL6
1.00E+00	1.37	GAPDH, RCC2, S100A8, S100A9
1.00E+00	1.46	FLNA, PRDX2, SRI
1.00E+00	1.46	ALDOA, B2M, OSBPL2
1.00E+00	1.46	CALM3, CDC42, RHOA
1.00E+00	1.46	CD36, SLC2A1, SLC2A3
1.00E+00	1.36	FLNA, PFN1, RCC2, SOD1
1.00E+00	1.66	ACTB, ITGB1
1.00E+00	1.45	G6PD, HNRNPU, ITGB1
1.00E+00	2.39	RHOA
1.00E+00	2.39	SRGN
1.00E+00	2.39	TCP1
1.00E+00	2.39	CD9
1.00E+00	2.39	GSTP1
1.00E+00	2.39	G6PD
1.00E+00	2.39	KANSL3
1.00E+00	2.39	ACTB
1.00E+00	2.39	THBS1
1.00E+00	2.39	SOD1
1.00E+00	2.39	ACTB
1.00E+00	2.39	XRCC5
1.00E+00	2.39	PCYOX1
1.00E+00	2.39	CLTA
1.00E+00	2.39	ANPEP
1.00E+00	1.45	ATIC, CD9, FLNA
1.00E+00	1.64	RHOA, SOD1
1.00E+00	1.64	SLC2A1, SLC2A3
1.00E+00	1.44	F11R, FLOT1, ITGB1
1.00E+00	1.63	CCL5, THBS1
1.00E+00	1.43	RHOA, SRI, THBS1
1.00E+00	1.43	ANXA2, GAPDH, LTF
1.00E+00	2.33	HSPA9
1.00E+00	2.33	FLNA
1.00E+00	2.33	GNAQ
1.00E+00	2.33	VIM
1.00E+00	2.33	DHX9
1.00E+00	2.33	HSP90AA1
1.00E+00	2.33	NCL
1.00E+00	2.33	G6PD
1.00E+00	2.33	ITGB1
1.00E+00	2.33	RTN4
1.00E+00	2.33	HSPA9

1.00E+00	2.33	ITGB1
1.00E+00	2.33	YWHAH
1.00E+00	2.33	ATP2A3
1.00E+00	2.33	UBE2L3
1.00E+00	1.61	HLA-A, RHOA
1.00E+00	1.61	G6PD, RTN4
1.00E+00	1.61	HSPA8, UBQLN1
1.00E+00	1.61	ACTB, HSPA8
1.00E+00	1.19	BCAP31, CANX, HSPA1B, PCYOX1, PSMA5, PSMA6, RPL11, UBE2L3, UBQLN1
1.00E+00	1.42	ITGB1, ITGB3, MYADM
1.00E+00	1.60	SLC2A1, SLC2A3
1.00E+00	1.60	RHOA, YWHAH
1.00E+00	1.60	ILK, PECAM1
1.00E+00	2.27	ILK
1.00E+00	2.27	HSPD1
1.00E+00	2.27	UBQLN1
1.00E+00	2.27	ACTB
1.00E+00	2.27	WBP11
1.00E+00	2.27	SPTBN1
1.00E+00	2.27	TTLL12
1.00E+00	2.27	CCL5
1.00E+00	2.27	PTBP1
1.00E+00	2.27	HNRNPU
1.00E+00	2.27	CD36
1.00E+00	2.27	HSP90AA1
1.00E+00	2.27	PPIA
1.00E+00	2.27	AACS
1.00E+00	1.58	ACTB, RHOA
1.00E+00	1.58	RHOA, SPN
1.00E+00	1.58	CCL5, TCP1
1.00E+00	1.40	INPP5D, PRDX2, SPN
1.00E+00	1.40	B2M, CYBB, SOD1
1.00E+00	1.32	DHX9, JUP, NPM1, UBE2L3
1.00E+00	1.13	ACTB, CALM3, CDC42, CLIC1, HNRNPU, HSPA1B, HSPA8, ITGB1, NPM1, RCC2, RHOA, RPS
1.00E+00	1.57	FCER1G, SPN
1.00E+00	1.57	CLIC1, PCYOX1
1.00E+00	1.57	CYBB, RHOA
1.00E+00	2.22	NPM1
1.00E+00	2.22	ATIC
1.00E+00	2.22	PRDX2
1.00E+00	2.22	CALM3
1.00E+00	2.22	PNPLA8
1.00E+00	2.22	PNPLA8
1.00E+00	2.22	THBS1
1.00E+00	2.22	PTBP1
1.00E+00	2.22	NCL
1.00E+00	2.22	GNAQ

1.00E+00	2.22	VDAC2
1.00E+00	2.22	RAB27B
1.00E+00	1.39	RHOA, SRI, THBS1
1.00E+00	1.39	ALDOA, ENO1, XRCC5
1.00E+00	1.26	ADH5, CYB5R3, G6PD, PLEK, SOD1
1.00E+00	1.39	EEF1A1, MYH9, SPTBN1
1.00E+00	1.56	CD9, ITGB1
1.00E+00	1.56	CD36, PNPLA8
1.00E+00	1.56	SLC2A1, SLC2A3
1.00E+00	2.17	RPLP0
1.00E+00	2.17	ATIC
1.00E+00	2.17	HINT1
1.00E+00	2.17	DSG1
1.00E+00	2.17	B2M
1.00E+00	2.17	GSTP1
1.00E+00	2.17	SOD1
1.00E+00	2.17	ANXA2
1.00E+00	2.17	SLC2A1
1.00E+00	2.17	ITGB1
1.00E+00	2.17	CDC42
1.00E+00	2.17	RCC2
1.00E+00	2.17	CD36
1.00E+00	2.17	ENO1
1.00E+00	2.17	HNRNPU
1.00E+00	2.17	THBS1
1.00E+00	2.17	CCL5
1.00E+00	1.54	CALM3, TNKS1BP1
1.00E+00	1.54	CCL5, THBS1
1.00E+00	1.54	BCAP31, HLA-A
1.00E+00	1.54	CASP14, CTSD
1.00E+00	1.22	ANXA2, B2M, ITGB1, RTN4, XRCC5, YWHAH
1.00E+00	1.53	H2BC21, NPM1
1.00E+00	1.53	ADH5, LRP1
1.00E+00	1.53	CALM3, SRI
1.00E+00	1.53	ILK, THBS1
1.00E+00	2.11	CDC42
1.00E+00	2.11	HSP90AA1
1.00E+00	2.11	ATIC
1.00E+00	2.11	RAC2
1.00E+00	2.11	ENO1
1.00E+00	2.11	TMEM109
1.00E+00	2.11	ITGB1
1.00E+00	2.11	ACTB
1.00E+00	2.11	HLA-A
1.00E+00	2.11	SOD1
1.00E+00	2.11	CFL1
1.00E+00	2.11	FLNA

1.00E+00	2.11	CD36
1.00E+00	2.11	VDAC2
1.00E+00	2.11	RHOA
1.00E+00	2.11	CLTA
1.00E+00	2.11	NPM1
1.00E+00	2.11	ENO1
1.00E+00	2.11	CPN1
1.00E+00	1.24	ACTB, H2BC21, HNRNPC, NASP, NPM1
1.00E+00	1.28	ANXA2, HSPA8, M6PR, RAB27B
1.00E+00	1.52	INPP5D, LTF
1.00E+00	1.52	GNAI2, NCL
1.00E+00	1.52	LTF, SRGN
1.00E+00	1.52	GSTP1, PTPRJ
1.00E+00	1.52	NPM1, RCC2
1.00E+00	1.52	GNAQ, PTGS1
1.00E+00	1.50	LTF, SRGN
1.00E+00	1.50	AACS, SRI
1.00E+00	1.27	ARL6IP5, CD36, ITGB1, PNPLA8
1.00E+00	2.07	LTF
1.00E+00	2.07	RTN4
1.00E+00	2.07	ITGB1
1.00E+00	2.07	SPTBN1
1.00E+00	2.07	TFRC
1.00E+00	2.07	CCL5
1.00E+00	2.07	DHX9
1.00E+00	2.07	CALM3
1.00E+00	2.07	FLNA
1.00E+00	2.07	RAB1B
1.00E+00	2.07	ATP2A3
1.00E+00	1.35	ITGB3, MYH9, SOD1
1.00E+00	1.49	HNRNPU, XRCC5
1.00E+00	1.34	RHOA, SRI, THBS1
1.00E+00	1.48	GNAI2, RHOA
1.00E+00	1.48	ENDOD1, G3BP1
1.00E+00	1.48	ADAM10, INPP5D
1.00E+00	2.02	CD59
1.00E+00	2.02	PLEK
1.00E+00	2.02	PTBP1
1.00E+00	2.02	SPN
1.00E+00	2.02	HMBS
1.00E+00	2.02	ATIC
1.00E+00	2.02	FCN1
1.00E+00	2.02	DSP
1.00E+00	2.02	QSOX1
1.00E+00	2.02	NPM1
1.00E+00	2.02	FLOT1
1.00E+00	2.02	RHOA

1.00E+00	2.02	ITGB1
1.00E+00	2.02	PFN1
1.00E+00	2.02	ACTB
1.00E+00	1.14	CALM3, CCT8, GNAQ, HSP90AA1, HSPD1, PKM, PTGS1, RAB10, VDAC2
1.00E+00	1.47	DSP, MATR3
1.00E+00	1.47	PTPRJ, RTN4
1.00E+00	1.47	CLIC1, PCYOX1
1.00E+00	1.98	ITGB1
1.00E+00	1.98	NPM1
1.00E+00	1.98	RHOA
1.00E+00	1.98	ARPC5
1.00E+00	1.98	VDAC2
1.00E+00	1.98	HLA-A
1.00E+00	1.98	RHOA
1.00E+00	1.98	HLA-B
1.00E+00	1.98	DHX9
1.00E+00	1.98	ACTB
1.00E+00	1.98	HSPA8
1.00E+00	1.98	CCL5
1.00E+00	1.46	ITGB1, ITGB3
1.00E+00	1.46	F11R, PATJ
1.00E+00	1.46	B2M, HLA-A
1.00E+00	1.25	AACS, CCL5, S100A8, SRI
1.00E+00	1.44	ACTB, DHX9
1.00E+00	1.44	PRDX2, SPN
1.00E+00	1.44	ATIC, HINT1
1.00E+00	1.93	FLNA
1.00E+00	1.93	GSTP1
1.00E+00	1.93	HINT1
1.00E+00	1.93	ANXA2
1.00E+00	1.93	PNPLA8
1.00E+00	1.93	BCAP31
1.00E+00	1.93	ITGB1
1.00E+00	1.93	HSP90AA1
1.00E+00	1.93	PRDX2
1.00E+00	1.93	TSPAN14
1.00E+00	1.93	ENO1
1.00E+00	1.93	DHX9
1.00E+00	1.93	RAB27B
1.00E+00	1.93	ENO1
1.00E+00	1.93	THBS1
1.00E+00	1.93	RHOA
1.00E+00	1.20	ADH5, ENO1, GALE, PGK1, PKM
1.00E+00	1.30	ADH5, INPP5D, SACM1L
1.00E+00	1.43	CDC42, RHOA
1.00E+00	1.42	CD36, GSTP1
1.00E+00	1.42	CD36, GSTP1

1.00E+00	1.42	DHX9, HNRNPA2B1
1.00E+00	1.89	RHOA
1.00E+00	1.89	GNAQ
1.00E+00	1.89	OSBPL2
1.00E+00	1.89	HINT1
1.00E+00	1.89	PPIA
1.00E+00	1.89	CALM3
1.00E+00	1.89	B2M
1.00E+00	1.89	SOD1
1.00E+00	1.89	PTPRJ
1.00E+00	1.89	RAC2
1.00E+00	1.89	RHOA
1.00E+00	1.89	SRI
1.00E+00	1.89	VDAC2
1.00E+00	1.89	CCL5
1.00E+00	1.19	ACTB, HNRNPA2B1, HNRNPU, RCC2, XRCC5
1.00E+00	1.19	INPP5D, PLEK, PNPLA8, PRDX6, SACM1L
1.00E+00	1.41	ACTB, HSPA8
1.00E+00	1.14	ATP2A3, CALM3, CCL5, GNAQ, LTF, UBE2L3, XRCC5
1.00E+00	1.18	ILK, LRP1, SPTBN1, THBS1, VIM
1.00E+00	1.86	SF3A3
1.00E+00	1.86	VIM
1.00E+00	1.86	CALM3
1.00E+00	1.86	DHX9
1.00E+00	1.86	CD36
1.00E+00	1.86	RAB10
1.00E+00	1.86	ADAM10
1.00E+00	1.86	OSBPL2
1.00E+00	1.86	AACS
1.00E+00	1.86	THBS1
1.00E+00	1.86	GNAI2
1.00E+00	1.86	RPL11
1.00E+00	1.86	THBS1
1.00E+00	1.86	ATP5F1B
1.00E+00	1.86	SF3A3
1.00E+00	1.86	OSBPL2
1.00E+00	1.40	SLC2A1, SLC2A3
1.00E+00	1.12	B2M, CD226, FCER1G, HLA-A, HLA-B, HSPD1, INPP5D, SPN, TFRC
1.00E+00	1.21	ALDOA, ATIC, ATP5F1B, ENO1
1.00E+00	1.21	DDX60L, DHX9, G3BP1, HSP90AA1
1.00E+00	1.27	G6PD, RHOA, SOD1
1.00E+00	1.21	DDX60L, DHX9, G3BP1, HSP90AA1
1.00E+00	1.39	ACTB, TFRC
1.00E+00	1.39	CYBB, RHOA
1.00E+00	1.39	TCP1, VDAC2
1.00E+00	1.15	ACTB, CDC42, FLNA, HNRNPU, HSPA1B, RHOA
1.00E+00	1.15	B2M, CDC42, ITGB3, KRT2, RTN4, THBS1

1.00E+00	1.26	B2M, HSPD1, TFRC
1.00E+00	1.21	ALDOA, ATIC, ATP5F1B, ENO1
1.00E+00	1.82	CDC42
1.00E+00	1.82	KANSL3
1.00E+00	1.82	GSTP1
1.00E+00	1.82	SOD1
1.00E+00	1.82	ITGB1
1.00E+00	1.82	HNRNPU
1.00E+00	1.82	JUP
1.00E+00	1.82	CDC42
1.00E+00	1.82	RTN4
1.00E+00	1.82	PTBP1
1.00E+00	1.82	RPL11
1.00E+00	1.82	ITGB1
1.00E+00	1.82	DAZAP1
1.00E+00	1.82	ALDOA
1.00E+00	1.38	INPP5D, SACM1L
1.00E+00	1.38	GNAQ, PTGS1
1.00E+00	1.14	ATP5F1B, GSR, HSPA9, HSPD1, SOD1, VDAC2
1.00E+00	1.37	ANXA2, ATP2A3
1.00E+00	1.78	GNAQ
1.00E+00	1.78	S100A8
1.00E+00	1.78	TFRC
1.00E+00	1.78	FLNA
1.00E+00	1.78	RAC2
1.00E+00	1.78	SRI
1.00E+00	1.78	PLEK
1.00E+00	1.78	YWHAE
1.00E+00	1.78	VDAC2
1.00E+00	1.78	SRI
1.00E+00	1.78	CLIC1
1.00E+00	1.19	ILK, ITGB3, PECAM1, RHOA
1.00E+00	1.36	ITGB1, ITGB3
1.00E+00	1.36	RHOA, YWHAH
1.00E+00	1.19	G3BP1, GNAQ, ILK, JUP
1.00E+00	1.12	ACTB, CALM3, ITGB1, NASP, NPM1, RCC2, UBE2L3
1.00E+00	1.24	FLNA, ITGB1, YWHAE
1.00E+00	1.15	INPP5D, PLEK, PNPLA8, PRDX6, SACM1L
1.00E+00	1.75	TFRC
1.00E+00	1.75	HMBS
1.00E+00	1.75	RAB33B
1.00E+00	1.75	ITGB1
1.00E+00	1.75	GNAQ
1.00E+00	1.75	LRP1
1.00E+00	1.75	RHOA
1.00E+00	1.75	HSPD1
1.00E+00	1.75	SRI

1.00E+00	1.75	SF3A3
1.00E+00	1.35	GNAQ, JUP
1.00E+00	1.35	S100A8, S100A9
1.00E+00	1.23	CDC42, ITGB3, RTN4
1.00E+00	1.34	RPLP0, RTN4
1.00E+00	1.23	AACS, CCL5, SRI
1.00E+00	1.23	ENO1, PKM, TKT
1.00E+00	1.18	HSP90AA1, ILK, TFRC, TNKS1BP1
1.00E+00	1.71	RAC2
1.00E+00	1.71	HSPA1B
1.00E+00	1.71	SLC2A1
1.00E+00	1.71	LTF
1.00E+00	1.71	NPM1
1.00E+00	1.71	LTF
1.00E+00	1.71	LRP1
1.00E+00	1.71	HINT1
1.00E+00	1.71	HNRNPU
1.00E+00	1.71	PRDX6
1.00E+00	1.71	UBQLN1
1.00E+00	1.33	CD36, F11R
1.00E+00	1.33	CYBB, PTGS1
1.00E+00	1.11	CDC42, G3BP1, GNAQ, ILK, JUP, LRP1, RHOA
1.00E+00	1.32	GNAQ, JUP
1.00E+00	1.32	CTSD, ITGB3
1.00E+00	1.32	ITGB3, PTPRJ
1.00E+00	1.12	ANXA2, ATP5F1B, CLIC1, CYBB, TMEM109, VDAC2
1.00E+00	1.68	THBS1
1.00E+00	1.68	RAC2
1.00E+00	1.68	DSP
1.00E+00	1.68	HSP90AA1
1.00E+00	1.68	FLNA
1.00E+00	1.68	RHOA
1.00E+00	1.68	KRT2
1.00E+00	1.68	PLEK
1.00E+00	1.68	GNAI2
1.00E+00	1.68	LTF
1.00E+00	1.68	PPIA
1.00E+00	1.68	CCL5
1.00E+00	1.68	HNRNPU
1.00E+00	1.68	B2M
1.00E+00	1.12	ANXA2, ATP5F1B, CLIC1, CYBB, TMEM109, VDAC2
1.00E+00	1.08	ANXA2, ATP2A3, ATP5F1B, CD36, CLIC1, CYBB, SLC2A1, SLC2A3, TMEM109, VDAC2
1.00E+00	1.21	RPL11, RPS15, WBP11
1.00E+00	1.21	B2M, CYBB, SOD1
1.00E+00	1.16	ILK, ITGB3, PECAM1, RHOA
1.00E+00	1.65	ATIC
1.00E+00	1.65	ACTB

1.00E+00	1.65	HNRNPU
1.00E+00	1.65	SLC2A1
1.00E+00	1.65	CDC42
1.00E+00	1.65	PNPLA8
1.00E+00	1.65	ADAM10
1.00E+00	1.65	LTF
1.00E+00	1.65	LTF
1.00E+00	1.30	CALM3, RCC2
1.00E+00	1.08	BCAP31, CANX, HSPA1B, PSMA5, PSMA6, RPL11, UBE2L3, UBQLN1
1.00E+00	1.15	ACTB, CDC42, HNRNPU, RCC2
1.00E+00	1.62	HNRNPU
1.00E+00	1.62	HSP90AA1
1.00E+00	1.62	CALM3
1.00E+00	1.62	HNRNPA2B1
1.00E+00	1.62	FLNA
1.00E+00	1.62	CALM3
1.00E+00	1.62	HMBS
1.00E+00	1.62	HSP90AA1
1.00E+00	1.62	HSP90AA1
1.00E+00	1.62	CCL5
1.00E+00	1.62	CLTA
1.00E+00	1.62	CLTA
1.00E+00	1.28	ILK, JUP
1.00E+00	1.19	CSDE1, NASP, SOD1
1.00E+00	1.18	RHOA, SRI, THBS1
1.00E+00	1.18	AACS, CCL5, SRI
1.00E+00	1.59	SOD1
1.00E+00	1.59	HSPA1B
1.00E+00	1.59	HNRNPU
1.00E+00	1.59	PRDX2
1.00E+00	1.59	ITGB3
1.00E+00	1.59	PTBP1
1.00E+00	1.59	ITGB1
1.00E+00	1.59	PTPRJ
1.00E+00	1.11	B2M, CDC42, ITGB3, RTN4, THBS1
1.00E+00	1.11	CDC42, HNRNPU, HSPA8, RAB27B, SOD1
1.00E+00	1.26	ILK, PECAM1
1.00E+00	1.13	CCL5, G6PD, RHOA, SOD1
1.00E+00	1.13	GSTP1, OSBPL2, PNPLA8, PTGS1
1.00E+00	1.57	CDC42
1.00E+00	1.57	ATIC
1.00E+00	1.57	SRGN
1.00E+00	1.57	SRI
1.00E+00	1.57	ARPC5
1.00E+00	1.57	CCL5
1.00E+00	1.57	UBQLN1
1.00E+00	1.25	ATP5F1B, RHOA

1.00E+00	1.25	ARL6IP5, ITGB1
1.00E+00	1.25	CSDE1, NASP
1.00E+00	1.25	RHOA, SOD1
1.00E+00	1.25	RHOA, SOD1
1.00E+00	1.25	PRDX2, SPN
1.00E+00	1.24	CD47, OSBPL2
1.00E+00	1.24	RHOA, SOD1
1.00E+00	1.24	CSDE1, NASP
1.00E+00	1.24	CANX, CLTA
1.00E+00	1.12	GSTP1, OSBPL2, PNPLA8, PTGS1
1.00E+00	1.12	F11R, HSPA8, ITGB3, SLC2A1
1.00E+00	1.16	CSDE1, NASP, SOD1
1.00E+00	1.54	ILK
1.00E+00	1.54	HSPA1B
1.00E+00	1.54	RAC2
1.00E+00	1.54	DSP
1.00E+00	1.54	THBS1
1.00E+00	1.54	HSPA9
1.00E+00	1.54	SPTBN1
1.00E+00	1.54	CD36
1.00E+00	1.10	HNRNPU, HSPA1B, NPM1, PCBP1, SF3A3
1.00E+00	1.08	DHX9, ENO1, HNRNPU, JUP, NPM1, UBE2L3
1.00E+00	1.05	CDC42, DSP, FLNA, GNG5, ILK, ITGA6, LTF, MSN, RAB33B, RHOA, RTN4, SOD1
1.00E+00	1.24	PGK1, THBS1
1.00E+00	1.24	CYBB, PTGS1
1.00E+00	1.51	ANXA2
1.00E+00	1.51	SOD1
1.00E+00	1.51	HNRNPA2B1
1.00E+00	1.51	CDC42
1.00E+00	1.51	HSPA9
1.00E+00	1.51	SOD1
1.00E+00	1.23	DAZAP1, SOD1
1.00E+00	1.23	NPM1, PATJ
1.00E+00	1.23	RAB10, SPTBN1
1.00E+00	1.15	DHX9, G3BP1, XRCC5
1.00E+00	1.11	ACTB, HSPD1, TFRC, XRCC5
1.00E+00	1.11	HSP90AA1, ILK, TFRC, TNKS1BP1
1.00E+00	1.05	BCAP31, CANX, HSPA1B, PCYOX1, PSMA5, PSMA6, RPL11, UBE2L3, UBQLN1
1.00E+00	1.22	CALM3, RAP1B
1.00E+00	1.22	CALM3, RAP1B
1.00E+00	1.22	PGK1, THBS1
1.00E+00	1.03	CD36, CSDE1, DHX9, ENO1, FLNA, GAPDH, GSTP1, HNRNPA2B1, HNRNPC, HNRNPU, HSP/
1.00E+00	1.14	H2BC21, NASP, NPM1
1.00E+00	1.49	FLNA
1.00E+00	1.49	RHOA
1.00E+00	1.49	CD36
1.00E+00	1.49	LRP1

1.00E+00	1.49	GSTP1
1.00E+00	1.49	VDAC2
1.00E+00	1.49	LRP1
1.00E+00	1.49	CCL5
1.00E+00	1.49	RAB1B
1.00E+00	1.49	CDC42
1.00E+00	1.21	PGK1, THBS1
1.00E+00	1.21	PNPLA8, PRDX6
1.00E+00	1.07	ACTB, CDC42, HNRNPU, RCC2, TTLL12, YWHAE
1.00E+00	1.08	ADAM10, CASP14, CTSD, LTF, PSMA6
1.00E+00	1.20	ATIC, ITGB1
1.00E+00	1.20	ANXA2, TFRC
1.00E+00	1.47	SPTBN1
1.00E+00	1.47	SPTBN1
1.00E+00	1.47	CD36
1.00E+00	1.47	PLEK
1.00E+00	1.47	F11R
1.00E+00	1.47	SOD1
1.00E+00	1.47	ATP2A3
1.00E+00	1.47	NCL
1.00E+00	1.47	MATR3
1.00E+00	1.47	RAB27B
1.00E+00	1.47	SPTBN1
1.00E+00	1.20	RHOA, SOD1
1.00E+00	1.44	RHOA
1.00E+00	1.44	ANXA2
1.00E+00	1.44	HSPD1
1.00E+00	1.44	PNPLA8
1.00E+00	1.44	QSOX1
1.00E+00	1.44	PNPLA8
1.00E+00	1.44	OSBPL2
1.00E+00	1.01	ADAM10, ANPEP, ANTXR2, ARL6IP5, ATP2A3, B2M, BCAP31, C16orf54, CALM3, CANX, CD
1.00E+00	1.19	PFN1, YWHAZ
1.00E+00	1.19	INPP5D, TFRC
1.00E+00	1.19	ANXA2, RAB27B
1.00E+00	1.12	FLNA, ITGB1, YWHAE
1.00E+00	1.18	SLC2A1, SLC2A3
1.00E+00	1.42	MATR3
1.00E+00	1.42	ATIC
1.00E+00	1.42	HMBS
1.00E+00	1.42	SOD1
1.00E+00	1.42	RTN4
1.00E+00	1.42	ENO1
1.00E+00	1.08	CLIC1, CYBB, TMEM109, VDAC2
1.00E+00	1.17	GNAI2, GNAQ
1.00E+00	1.17	HSPA1B, MYH16
1.00E+00	1.10	ACTB, ITGB1, NASP

1.00E+00	1.10	G6PD, LTF, RHOA
1.00E+00	1.10	ACTB, HNRNPU, RCC2
1.00E+00	1.40	SOD1
1.00E+00	1.40	YWHAH
1.00E+00	1.40	LRP1
1.00E+00	1.40	ILK
1.00E+00	1.40	THBS1
1.00E+00	1.40	RPL11
1.00E+00	1.16	ATP5F1B, HSPA9
1.00E+00	1.00	ADAM10, ANPEP, ANTXR2, ARL6IP5, ATP2A3, B2M, BCAP31, C16orf54, CALM3, CANX, CD
1.00E+00	1.04	ACTB, CDC42, FLNA, HNRNPU, HSPA1B, RHOA
1.00E+00	1.07	EEF1A1, GAPDH, QSOX1, UBQLN1
1.00E+00	1.16	CLIC1, VDAC2
1.00E+00	1.09	CDC42, FLNA, RTN4
1.00E+00	1.38	XRCC5
1.00E+00	1.38	HNRNPA2B1
1.00E+00	1.38	CCL5
1.00E+00	1.38	ATP5F1B
1.00E+00	1.38	HSP90AA1
1.00E+00	1.38	ILK
1.00E+00	1.38	HSPD1
1.00E+00	1.15	G6PD, PTBP1
1.00E+00	1.15	CD36, HNRNPU
1.00E+00	1.03	ACTB, H2BC21, HNRNPC, HNRNPU, KANSL3, NASP, NPM1
1.00E+00	1.36	GNAQ
1.00E+00	1.36	ITGB1
1.00E+00	1.36	SPN
1.00E+00	1.36	CDC42
1.00E+00	1.36	CDC42
1.00E+00	1.36	CD36
1.00E+00	1.36	PPIA
1.00E+00	1.04	MSN, PRDX2, RPLP0, RTN4, SOD1
1.00E+00	1.34	PRDX2
1.00E+00	1.34	G6PD
1.00E+00	1.34	GNG5
1.00E+00	1.34	THBS1
1.00E+00	1.34	ITGB1
1.00E+00	1.34	G6PD
1.00E+00	1.34	RTN4
1.00E+00	1.34	QSOX1
1.00E+00	1.34	CALM3
1.00E+00	1.13	HNRNPU, KANSL3
1.00E+00	1.00	ACTB, ANXA2, CCT2, CCT4, CCT6A, CCT7, CCT8, CD36, CSDE1, DAZAP1, DHX9, EIF5A, END
1.00E+00	1.12	F11R, VCL
1.00E+00	1.12	ATP5F1B, GNAI2
1.00E+00	1.12	ACTB, HNRNPU
1.00E+00	1.07	NASP, NPM1, TTLL12

1.00E+00	1.32	SPN
1.00E+00	1.32	ITGB3
1.00E+00	1.32	LBP
1.00E+00	1.32	SPN
1.00E+00	1.32	HSP90AA1
1.00E+00	1.32	HINT1
1.00E+00	1.11	AACS, ATP5F1B
1.00E+00	1.30	SPN
1.00E+00	1.30	SPN
1.00E+00	1.30	CCL5
1.00E+00	1.30	DSP
1.00E+00	1.30	INPP5D
1.00E+00	1.30	HNRNPU
1.00E+00	1.30	LRP1
1.00E+00	1.30	F11R
1.00E+00	1.30	HNRNPU
1.00E+00	1.30	ACTB
1.00E+00	1.30	SLC2A1
1.00E+00	1.30	XRCC5
1.00E+00	1.11	ACTB, DHX9
1.00E+00	0.99	ACTB, ANXA2, CSDE1, DAZAP1, DHX9, EIF5A, HNRNPU, HSPA8, ILK, ITGA6, JUP, KANSL3, I
1.00E+00	1.00	CDC42, HSP90AA1, HSPA1B, NPM1, PPIA, RPL11, RPS15, UBE2L3, UBQLN1
1.00E+00	1.10	DSP, MATR3
1.00E+00	1.28	ACTB
1.00E+00	1.28	G6PD
1.00E+00	1.28	RHOA
1.00E+00	1.28	HNRNPU
1.00E+00	1.28	SPTBN1
1.00E+00	1.28	FLOT1
1.00E+00	1.09	HNRNPA2B1, XRCC5
1.00E+00	1.09	GNAQ, PTGS1
1.00E+00	1.00	AACS, ADH5, CD36, GSTP1, INPP5D, LRP1, PLEK, PNPLA8, PRDX6, PTGS1, SACM1L
1.00E+00	1.26	M6PR
1.00E+00	1.26	CD9
1.00E+00	1.26	RHOA
1.00E+00	1.26	ACTB
1.00E+00	1.26	DSP
1.00E+00	1.26	ENO1
1.00E+00	1.26	CANX
1.00E+00	1.26	EEF1A1
1.00E+00	1.09	FLNA, RTN4
1.00E+00	1.09	ATP2A3, ATP5F1B
1.00E+00	1.00	ACTB, CALM3, CDC42, HNRNPU, HSPA1B, NPM1, RCC2, RHOA
1.00E+00	1.00	GNAI2, ITGB3, LRP1, NCL, PTPRJ, RTN4, THBS1
1.00E+00	1.02	ILK, ITGB3, PECAM1, RHOA
1.00E+00	1.24	MATR3
1.00E+00	1.24	NPM1

1.00E+00	1.24	CD36
1.00E+00	1.24	PTPRJ
1.00E+00	1.24	RHOA
1.00E+00	1.24	NPM1
1.00E+00	1.04	HSPA1B, RHOA, YWHAH
1.00E+00	1.07	FLNA, MSN
1.00E+00	1.07	CSDE1, NASP
1.00E+00	1.23	ACTB
1.00E+00	1.23	SOD1
1.00E+00	1.23	KANSL3
1.00E+00	1.23	TTLL12
1.00E+00	1.23	ILK
1.00E+00	1.07	TTLL12, UBQLN1
1.00E+00	1.02	CANX, HSPA8, ITGB3
1.00E+00	1.06	ACTB, XRCC5
1.00E+00	1.06	MMRN1, THBS1
1.00E+00	1.01	CCL5, INPP5D, PTPRJ, SACM1L
1.00E+00	1.21	THBS1
1.00E+00	1.21	TFRC
1.00E+00	1.21	HINT1
1.00E+00	1.21	ATP5F1B
1.00E+00	1.21	SOD1
1.00E+00	1.21	G6PD
1.00E+00	1.21	XRCC5
1.00E+00	1.06	B2M, HSP90AA1
1.00E+00	1.06	G3BP1, LRP1
1.00E+00	1.06	LTF, SRGN
1.00E+00	1.06	PNPLA8, RTN4
1.00E+00	1.06	ACTB, HNRNPU
1.00E+00	0.98	CD9, G6PD, HSP90AA1, ITGB1, RTN4, SOD1, VCL
1.00E+00	1.19	ATIC
1.00E+00	1.19	PNPLA8
1.00E+00	1.19	TTLL12
1.00E+00	1.19	ILK
1.00E+00	1.19	ARPC5
1.00E+00	1.19	YWHAG
1.00E+00	1.05	HSPA8, SLC2A1
1.00E+00	1.04	B2M, THBS1
1.00E+00	1.01	ARL6IP5, B2M, ITGB1
1.00E+00	1.01	CCL5, ITGB3, PF4V1
1.00E+00	0.98	ADAM10, ITGB1, MATR3, MYH9, NASP, PFN1, RTN4
1.00E+00	1.18	THBS1
1.00E+00	1.18	GSTP1
1.00E+00	1.18	AACS
1.00E+00	1.18	CLIC1
1.00E+00	1.04	ANXA2, CD9
1.00E+00	0.97	ADAM10, CDC42, CFL1, ITGB1, ITGB3, MATR3, MYADM, MYH9, NASP, PFN1, RTN4, SOD1

1.00E+00	1.16	RAC2
1.00E+00	1.16	LRP1
1.00E+00	1.16	ILK
1.00E+00	1.16	CALM3
1.00E+00	1.16	FLNA
1.00E+00	1.00	INPP5D, PTPRJ, SACM1L
1.00E+00	0.97	CCL5, ENDOD1, G3BP1, INPP5D, PNPLA8, PRDX6, PTPRJ, SACM1L
1.00E+00	0.99	H2BC21, NASP, NPM1
1.00E+00	0.97	AACS, CALM3, CCL5, RAP1B, SRI
1.00E+00	1.15	CCL5
1.00E+00	1.15	HMBS
1.00E+00	1.15	DSP
1.00E+00	1.15	CCL5
1.00E+00	1.15	CYBB
1.00E+00	1.15	ILK
1.00E+00	1.15	CALM3
1.00E+00	1.15	CANX
1.00E+00	1.15	TTLL12
1.00E+00	1.15	TTLL12
1.00E+00	0.97	CALM3, FLOT1, GNAI2, GNAQ, ITGB1, RAP1B, YWHAG, YWHAH
1.00E+00	1.02	ACTB, KANSL3
1.00E+00	1.02	G6PD, SOD1
1.00E+00	1.13	KANSL3
1.00E+00	1.13	THBS1
1.00E+00	1.13	ATIC
1.00E+00	1.13	HINT1
1.00E+00	1.13	HNRNPA2B1
1.00E+00	1.01	LTF, SRGN
1.00E+00	1.01	NCL, XRCC5
1.00E+00	0.97	ACTB, CALM3, ITGB1, NASP, RCC2
1.00E+00	0.98	ACTB, ITGB1, NASP
1.00E+00	1.12	YWHAE
1.00E+00	1.12	CCL5
1.00E+00	1.12	SPTBN1
1.00E+00	1.12	HNRNPU
1.00E+00	1.00	ITGB3, THBS1
1.00E+00	1.00	ADAM10, TSPAN14
1.00E+00	0.96	ALDOA, ATIC, ATP5F1B, ENO1, G6PD, NANS, TKT
1.00E+00	1.11	PGK1
1.00E+00	1.11	ANXA2
1.00E+00	1.11	CDC42
1.00E+00	1.11	TTLL12
1.00E+00	1.11	RHOA
1.00E+00	1.11	HSPA8
1.00E+00	1.00	STOM, YWHAH
1.00E+00	0.96	ACTB, CDC42, HNRNPU, RCC2
1.00E+00	1.09	ACTB

1.00E+00	1.09	ILK
1.00E+00	1.09	CDC42
1.00E+00	1.09	YWHAH
1.00E+00	1.09	CASP14
1.00E+00	1.09	SOD1
1.00E+00	0.95	CALM3, GNAQ, HLA-A, HLA-B, JUP, LBP, PTPRJ
1.00E+00	0.99	ACTB, KANSL3
1.00E+00	1.08	ITGB1
1.00E+00	1.08	THBS1
1.00E+00	1.08	HSP90AA1
1.00E+00	1.08	HSPA1B
1.00E+00	1.08	ATP5F1B
1.00E+00	1.08	HINT1
1.00E+00	1.08	ANK1
1.00E+00	0.98	CDC42, YWHAZ
1.00E+00	0.95	ADAM10, ITGB1, MATR3, MYH9, NASP, PFN1, RTN4
1.00E+00	0.98	ACTB, KANSL3
1.00E+00	0.95	CSDE1, NASP, SOD1
1.00E+00	1.07	VIM
1.00E+00	1.07	CCL5
1.00E+00	1.07	PGK1
1.00E+00	1.07	HNRNPU
1.00E+00	1.07	FCN1
1.00E+00	0.95	ILK, LRP1, THBS1
1.00E+00	1.06	THBS1
1.00E+00	1.06	QSOX1
1.00E+00	1.06	HSPA1B
1.00E+00	0.94	AACS, CCL5, SRI
1.00E+00	0.96	CDC42, YWHAZ
1.00E+00	0.96	ENO1, HNRNPU
1.00E+00	1.04	SPN
1.00E+00	1.04	CDC42
1.00E+00	1.04	ACTB
1.00E+00	1.04	CCL5
1.00E+00	0.95	RHOA, YWHAH
1.00E+00	1.03	RPLP0
1.00E+00	1.03	NPM1
1.00E+00	1.03	CDC42
1.00E+00	1.03	HNRNPU
1.00E+00	1.03	CYBB
1.00E+00	1.03	CLTA
1.00E+00	1.03	CALM3
1.00E+00	1.03	CLTA
1.00E+00	0.95	ADAM10, SOD1
1.00E+00	0.94	ATP5F1B, RHOA
1.00E+00	1.02	ATP5F1B
1.00E+00	1.02	CFL1

1.00E+00	1.02	ACTB
1.00E+00	1.01	KRT17
1.00E+00	1.01	RHOA
1.00E+00	1.01	FLNA
1.00E+00	1.01	HSPD1
1.00E+00	0.93	ACTB, KANSL3
1.00E+00	1.00	PFN1
1.00E+00	1.00	HSP90AA1
1.00E+00	1.00	ACTB
1.00E+00	1.00	HNRNPU
1.00E+00	1.00	FLNA
1.00E+00	1.00	SF3A3
1.00E+00	1.00	BCAP31
1.00E+00	1.00	HSPA9
1.00E+00	0.92	ACTB, TNKS1BP1, XRCC5
1.00E+00	0.92	AACS, CCL5, SRI
1.00E+00	0.99	LRBA
1.00E+00	0.99	RTN4
1.00E+00	0.99	RHOA
1.00E+00	0.99	RHOA
1.00E+00	0.99	G6PD
1.00E+00	0.99	PFN1
1.00E+00	0.99	LRBA
1.00E+00	0.99	PTGS1
1.00E+00	0.92	ITGB3, THBS1
1.00E+00	0.93	CALM3, CCL5, CDC42, FCN1, FLNA, GNAI2, GNAQ, GNG5, GP1BA, ITGB3, PLEK, RAC2, YW
1.00E+00	0.91	DHX9, GNAQ, HNRNPU
1.00E+00	0.98	KRT17
1.00E+00	0.98	KRT17
1.00E+00	0.98	ENO1
1.00E+00	0.98	HSPA9
1.00E+00	0.98	ACTB
1.00E+00	0.98	LYZ
1.00E+00	0.96	SF3A3
1.00E+00	0.96	ACTB
1.00E+00	0.96	PRDX2
1.00E+00	0.96	ATIC
1.00E+00	0.96	KRT17
1.00E+00	0.96	ACTB
1.00E+00	0.96	SF3A3
1.00E+00	0.92	CDC42, HSP90AA1, HSPA1B, NPM1, PPIA, RPL11, RPS15, UBE2L3, UBQLN1
1.00E+00	0.90	F11R, FLOT1, ITGB1
1.00E+00	0.90	CSDE1, NASP, SOD1
1.00E+00	0.90	ILK, RTN4
1.00E+00	0.95	RHOA
1.00E+00	0.95	CDC42
1.00E+00	0.95	G6PD

1.00E+00	0.95	ILK
1.00E+00	0.95	CALM3
1.00E+00	0.90	ACTB, HNRNPU
1.00E+00	0.90	INPP5D, TFRC
1.00E+00	0.91	ADH5, ANPEP, CPN1, FLG2, HRNR, LTF, MATR3, S100A8, S100A9, SF3A3, SOD1
1.00E+00	0.94	ILK
1.00E+00	0.94	G6PD
1.00E+00	0.94	ILK
1.00E+00	0.89	BCAP31, CD36
1.00E+00	0.93	ACTB
1.00E+00	0.93	PFN1
1.00E+00	0.93	ACTB
1.00E+00	0.93	LTF
1.00E+00	0.93	ILK
1.00E+00	0.93	ILK
1.00E+00	0.93	CD36
1.00E+00	0.93	PTPRJ
1.00E+00	0.93	ANXA2
1.00E+00	0.88	CSDE1, NASP, SOD1
1.00E+00	0.88	YWHAG, YWHAH
1.00E+00	0.93	G6PD
1.00E+00	0.93	ENO1
1.00E+00	0.93	ATP5F1B
1.00E+00	0.93	RCC2
1.00E+00	0.93	PRDX1
1.00E+00	0.93	HSP90AA1
1.00E+00	0.93	ILK
1.00E+00	0.88	ACTB, DHX9, NPM1
1.00E+00	0.88	ENDOD1, G3BP1
1.00E+00	0.88	CDC42, FLNA, RTN4
1.00E+00	0.92	PRDX2
1.00E+00	0.92	RCC2
1.00E+00	0.92	EIF5A
1.00E+00	0.90	GNAI2, ILK, ITGB3, LRP1, NCL, PTPRJ, RTN4, SPTBN1, THBS1, VIM
1.00E+00	0.87	ARL6IP5, B2M, ITGB1
1.00E+00	0.87	GNAQ, HNRNPU
1.00E+00	0.91	ATIC
1.00E+00	0.91	ACTB
1.00E+00	0.87	HSPA8, SLC2A1
1.00E+00	0.87	CDC42, YWHAZ
1.00E+00	0.90	ILK
1.00E+00	0.90	TMEM109
1.00E+00	0.90	G6PD
1.00E+00	0.90	SOD1
1.00E+00	0.90	KANSL3
1.00E+00	0.90	G6PD
1.00E+00	0.90	TUBA1C

1.00E+00	0.86	ACTB, DHX9
1.00E+00	0.89	SOD1
1.00E+00	0.89	PLEK
1.00E+00	0.89	ADH5, ANPEP, CPN1, MATR3, S100A8, S100A9, SF3A3, SOD1
1.00E+00	0.85	INPP5D, SACM1L
1.00E+00	0.88	G6PD
1.00E+00	0.88	OSBPL2
1.00E+00	0.88	HNRNPU
1.00E+00	0.88	CCL5
1.00E+00	0.91	CD36, CSDE1, DHX9, ENO1, FLNA, GAPDH, GSTP1, HNRNPA2B1, HNRNPC, HNRNPU, HSPA
1.00E+00	0.85	GNAQ, ITGB1, NPM1
1.00E+00	0.88	CALM3, FLOT1, GNAI2, ITGB1, RAP1B, YWHAG, YWHAH
1.00E+00	0.88	CALM3, FLOT1, GNAI2, ITGB1, RAP1B, YWHAG, YWHAH
1.00E+00	0.87	SOD1
1.00E+00	0.87	FLNA
1.00E+00	0.87	FLNA
1.00E+00	0.87	SPN
1.00E+00	0.87	SF3A3
1.00E+00	0.86	PFN1
1.00E+00	0.86	RAB33B
1.00E+00	0.90	CD36, CSDE1, DHX9, ENO1, FLNA, HNRNPA2B1, HNRNPC, HNRNPU, HSPA8, NPM1, PTBP
1.00E+00	0.84	RHOA, SOD1
1.00E+00	0.86	RAC2
1.00E+00	0.86	MATR3
1.00E+00	0.86	LRP1
1.00E+00	0.86	CLIC1
1.00E+00	0.87	CALM3, FLOT1, GNAI2, ITGB1, RAP1B, YWHAG, YWHAH
1.00E+00	0.85	TFRC
1.00E+00	0.85	ADH5
1.00E+00	0.85	RTN4
1.00E+00	0.85	FLNA
1.00E+00	0.85	FLNA
1.00E+00	0.83	ADAM10, SOD1
1.00E+00	0.84	ITGB1
1.00E+00	0.84	HNRNPU
1.00E+00	0.84	SOD1
1.00E+00	0.84	ENO1
1.00E+00	0.82	PNPLA8, PRDX6
1.00E+00	0.88	CDC42, HINT1, HSP90AA1, HSPA1B, NPM1, PPIA, RPL11, RPS15, UBE2L3, UBQLN1
1.00E+00	0.83	PTPRJ
1.00E+00	0.83	ADH5
1.00E+00	0.83	SOD1
1.00E+00	0.83	THBS1
1.00E+00	0.89	ANPEP, ANTXR2, CD36, CD47, FCER1G, FCN1, GP1BA, GP1BB, ITGB1, ITGB3, KRT1, LRP1,
1.00E+00	0.89	ANPEP, ANTXR2, CD36, CD47, FCER1G, FCN1, GP1BA, GP1BB, ITGB1, ITGB3, KRT1, LRP1,
1.00E+00	0.82	G6PD, RTN4, SOD1
1.00E+00	0.82	CCL5

1.00E+00	0.82	ATIC
1.00E+00	0.82	ILK
1.00E+00	0.82	ACTB
1.00E+00	0.82	ADAM10
1.00E+00	0.83	HNRNPA2B1, RPL11, RPS15, WBP11
1.00E+00	0.81	CALM3, TNKS1BP1
1.00E+00	0.82	ILK, PFN1, RHOA
1.00E+00	0.80	LTF, RAB33B
1.00E+00	0.81	PNPLA8, PRDX6, YWHAH
1.00E+00	0.80	ATP5F1B
1.00E+00	0.80	CALM3
1.00E+00	0.81	ILK, ITGB3, THBS1
1.00E+00	0.79	ITGB3
1.00E+00	0.79	CALM3
1.00E+00	0.79	SPN
1.00E+00	0.79	CCL5
1.00E+00	0.79	HSPA8
1.00E+00	0.79	ACTB, KANSL3
1.00E+00	0.81	ENO1, PLEK, SOD1
1.00E+00	0.82	ANXA2, ATP5F1B, CLIC1, VDAC2
1.00E+00	0.79	HSPA8, SLC2A1
1.00E+00	0.79	ITGB1, XRCC5
1.00E+00	0.79	TUBB
1.00E+00	0.79	ADH5
1.00E+00	0.79	SF3A3
1.00E+00	0.78	ACTB
1.00E+00	0.78	THBS1
1.00E+00	0.77	GSTP1
1.00E+00	0.77	CDC42
1.00E+00	0.77	PLEK
1.00E+00	0.77	CANX
1.00E+00	0.77	ANXA2
1.00E+00	0.77	ANXA2
1.00E+00	0.91	ADAM10, ADH5, ANPEP, ANTXR2, ANXA11, ANXA2, ANXA5, ANXA7, ATP2A3, CALM3, CA
1.00E+00	0.91	ADAM10, ADH5, ANPEP, ANTXR2, ANXA11, ANXA2, ANXA5, ANXA7, ATP2A3, CALM3, CA
1.00E+00	0.76	GNAQ
1.00E+00	0.76	THBS1
1.00E+00	0.76	ACTB
1.00E+00	0.77	CCL5, PF4V1
1.00E+00	0.81	ATP5F1B, HSPA9, HSPD1, LDHB, MATR3
1.00E+00	0.75	ANXA2
1.00E+00	0.76	ACTB, HNRNPU
1.00E+00	0.75	FLNA
1.00E+00	0.75	G6PD
1.00E+00	0.75	XRCC5
1.00E+00	0.76	ATP5F1B, RHOA
1.00E+00	0.74	MSN

1.00E+00	0.74	NPM1
1.00E+00	0.74	RCC2
1.00E+00	0.74	CANX
1.00E+00	0.76	DHX9, HNRNPU
1.00E+00	0.87	CD36, CSDE1, DHX9, ENO1, FLNA, GAPDH, HNRNPA2B1, HNRNPC, HNRNPU, HSPA8, NCL,
1.00E+00	0.74	HSPA1B
1.00E+00	0.74	SOD1
1.00E+00	0.74	SOD1
1.00E+00	0.72	DSP
1.00E+00	0.72	CALM3
1.00E+00	0.72	NANS
1.00E+00	0.74	PPIA, UBE2L3
1.00E+00	0.72	PFN1
1.00E+00	0.72	HINT1
1.00E+00	0.73	DHX9, HNRNPU
1.00E+00	0.71	RCC2
1.00E+00	0.71	CD36
1.00E+00	0.70	ARL6IP5
1.00E+00	0.70	KANSL3
1.00E+00	0.70	SOD1
1.00E+00	0.89	ACTB, ANXA2, CD36, DHX9, EIF5A, ENO1, FLNA, FLOT1, HINT1, HNRNPA2B1, HNRNPU, H!
1.00E+00	0.89	ACTB, ANXA2, CD36, DHX9, EIF5A, ENO1, FLNA, FLOT1, HINT1, HNRNPA2B1, HNRNPU, H!
1.00E+00	0.72	ENDOD1, G3BP1
1.00E+00	0.69	ARL6IP5
1.00E+00	0.69	LYZ
1.00E+00	0.72	INPP5D, SACM1L
1.00E+00	0.69	FLNA
1.00E+00	0.69	CD36
1.00E+00	0.71	STOM, YWHAH
1.00E+00	0.88	ACTB, ANXA2, CD36, DHX9, EIF5A, ENO1, FLNA, FLOT1, HINT1, HNRNPA2B1, HNRNPU, H!
1.00E+00	0.68	STOM
1.00E+00	0.68	RHOA
1.00E+00	0.68	NPM1
1.00E+00	0.68	RHOA
1.00E+00	0.68	ACTB
1.00E+00	0.68	ACTB
1.00E+00	0.77	ACTB, DHX9, NPM1, TNKS1BP1, XRCC5
1.00E+00	0.67	DHX9
1.00E+00	0.67	SRI
1.00E+00	0.67	XRCC5
1.00E+00	0.67	ATP5F1B
1.00E+00	0.70	INPP5D, SACM1L
1.00E+00	0.67	FLNA
1.00E+00	0.67	THBS1
1.00E+00	0.66	SRI
1.00E+00	0.66	PKM
1.00E+00	0.70	HSPA8, SLC2A1

1.00E+00	0.81	ANXA2, ATP2A3, ATP5F1B, CD36, CLIC1, CYBB, SLC2A1, SLC2A3, TMEM109, VDAC2
1.00E+00	0.76	ACTB, DHX9, HNRNPC, HNRNPU, NPM1
1.00E+00	0.66	RTN4
1.00E+00	0.66	PFN1
1.00E+00	0.66	LRBA
1.00E+00	0.66	YWHAH
1.00E+00	0.65	SRI
1.00E+00	0.81	ANXA2, ATP2A3, ATP5F1B, CD36, CLIC1, CYBB, OSBPL2, SLC2A1, SLC2A3, TMEM109, VDA
1.00E+00	0.65	GSTP1
1.00E+00	0.65	G3BP1
1.00E+00	0.71	DDX60L, DHX9, G3BP1
1.00E+00	0.76	CCL5, EEF1A1, ILK, ITGB3, NCL, THBS1
1.00E+00	0.63	KANSL3
1.00E+00	0.73	ATP5F1B, HSPA9, HSPD1, LDHB
1.00E+00	0.63	CCL5
1.00E+00	0.63	CDC42
1.00E+00	0.67	ACTB, KANSL3
1.00E+00	0.67	ACTB, HNRNPU
1.00E+00	0.62	THBS1
1.00E+00	0.62	SOD1
1.00E+00	0.62	HNRNPU
1.00E+00	0.62	CD36
1.00E+00	0.62	CD36
1.00E+00	0.62	GP1BA
1.00E+00	0.74	ITGB1, ITGB3, MYADM, PFN1, SOD1
1.00E+00	0.62	GSTP1
1.00E+00	0.62	FCER1G
1.00E+00	0.82	CD36, CSDE1, DHX9, ENO1, FLNA, HNRNPA2B1, HNRNPC, HNRNPU, HSPA8, NPM1, PTBP:
1.00E+00	0.61	NPM1
1.00E+00	0.61	TKT
1.00E+00	0.88	ACTB, ANXA2, CD36, CSDE1, DAZAP1, DHX9, EIF5A, ENO1, FLNA, FLOT1, HINT1, HNRNPA
1.00E+00	0.61	ITGB1
1.00E+00	0.61	TFRC
1.00E+00	0.61	THBS1
1.00E+00	0.65	ATP2A3, ATP5F1B
1.00E+00	0.84	ADAM10, ANPEP, CASP14, CCL5, CDC42, CPN1, CTSD, DCD, GAPDH, GSR, HINT1, ILK, ITGI
1.00E+00	0.65	ITGB1, XRCC5
1.00E+00	0.60	ENO1
1.00E+00	0.60	SRI
1.00E+00	0.60	PFN1
1.00E+00	0.60	GP1BA
1.00E+00	0.60	LTF
1.00E+00	0.80	DHX9, EEF1A1, FLNA, HSPA8, HSPA9, NCL, NPM1, PTBP1, RCC2, RPL11, RPS3A, SPTBN1, X
1.00E+00	0.59	CDC42
1.00E+00	0.59	HSP90AA1
1.00E+00	0.59	PLEK
1.00E+00	0.70	AACS, ADAM10, CCL5, SRI

1.00E+00	0.72	ARL6IP5, B2M, ITGB1, SOD1, THBS1
1.00E+00	0.58	RTN4
1.00E+00	0.64	DHX9, NASP
1.00E+00	0.58	ILK
1.00E+00	0.73	CCL5, EEF1A1, ILK, ITGB3, NCL, THBS1
1.00E+00	0.67	EEF1A1, EEF2, EIF5A
1.00E+00	0.58	SRI
1.00E+00	0.58	LRP1
1.00E+00	0.71	HNRNPA2B1, NCL, RPL11, RPS15, WBP11
1.00E+00	0.85	ACTB, ANXA2, CD36, DHX9, EIF5A, ENO1, FLNA, FLOT1, HINT1, HNRNPA2B1, HNRNPU, H
1.00E+00	0.85	ACTB, ANXA2, CD36, DHX9, EIF5A, ENO1, FLNA, FLOT1, HINT1, HNRNPA2B1, HNRNPU, H
1.00E+00	0.57	SOD1
1.00E+00	0.57	ATP5F1B
1.00E+00	0.66	ACTB, HINT1, KANSL3
1.00E+00	0.85	ACTB, ANXA2, CD36, DHX9, EIF5A, ENO1, FLNA, FLOT1, HINT1, HNRNPA2B1, HNRNPU, H
1.00E+00	0.62	ATP5F1B, HSPA9
1.00E+00	0.55	PFN1
1.00E+00	0.61	CALM3, CLIC1
1.00E+00	0.55	CD36
1.00E+00	0.55	CD36
1.00E+00	0.54	KANSL3
1.00E+00	0.54	ILK
1.00E+00	0.54	ACTB
1.00E+00	0.53	FLOT1
1.00E+00	0.53	GSTP1
1.00E+00	0.53	CCL5
1.00E+00	0.52	LTF
1.00E+00	0.52	ITGB1
1.00E+00	0.52	KANSL3
1.00E+00	0.52	HINT1
1.00E+00	0.52	HINT1
1.00E+00	0.51	ACTB
1.00E+00	0.51	PTPRJ
1.00E+00	0.51	CD36
1.00E+00	0.51	CCL5
1.00E+00	0.51	ITGA6
1.00E+00	0.51	ITGA6
1.00E+00	0.51	ILK
1.00E+00	0.51	ILK
1.00E+00	0.51	PTGS1
1.00E+00	0.50	SOD1
1.00E+00	0.50	NPM1
1.00E+00	0.50	ACTB
1.00E+00	0.50	SOD1
1.00E+00	0.50	CCL5
1.00E+00	0.50	CCL5
1.00E+00	0.49	ACTB

1.00E+00	0.49	PLEK
1.00E+00	0.49	G6PD
1.00E+00	0.73	ANXA2, DHX9, EIF5A, HNRNPU, ITGA6, JUP, KANSL3, NCL, NPM1, PCBP1
1.00E+00	0.48	ILK
1.00E+00	0.60	ACTB, NPM1, RCC2
1.00E+00	0.47	LTF
1.00E+00	0.68	BCAP31, CD9, DAZAP1, DSG1, ITGB1, KRT9, SOD1
1.00E+00	0.54	ATP5F1B, RHOA
1.00E+00	0.46	LTF
1.00E+00	0.46	SOD1
1.00E+00	0.61	DDX60L, DHX9, G3BP1, XRCC5
1.00E+00	0.74	ACTB, ANXA2, DHX9, EIF5A, HNRNPU, ILK, ITGA6, JUP, KANSL3, NCL, NPM1, PCBP1, UBE2
1.00E+00	0.74	ACTB, ANXA2, DHX9, EIF5A, HNRNPU, ILK, ITGA6, JUP, KANSL3, NCL, NPM1, PCBP1, UBE2
1.00E+00	0.61	BCAP31, DAZAP1, KRT9, SOD1
1.00E+00	0.45	CD36
1.00E+00	0.74	ACTB, ANXA2, DHX9, EIF5A, HNRNPU, ILK, ITGA6, JUP, KANSL3, NCL, NPM1, PCBP1, UBE2
1.00E+00	0.52	ANXA2, ATP5F1B
1.00E+00	0.52	CDC42, SOD1
1.00E+00	0.44	KANSL3
1.00E+00	0.44	KANSL3
1.00E+00	0.51	ANXA2, SRGN
1.00E+00	0.51	ACTB, RCC2
1.00E+00	0.43	MYH9
1.00E+00	0.43	ACTB
1.00E+00	0.59	BCAP31, DAZAP1, KRT9, SOD1
1.00E+00	0.42	THBS1
1.00E+00	0.61	ANXA2, ATP2A3, ATP5F1B, CLIC1, VDAC2
1.00E+00	0.41	PRDX6
1.00E+00	0.41	CALM3
1.00E+00	0.40	RCC2
1.00E+00	0.40	ATIC
1.00E+00	0.48	CALM3, CLIC1
1.00E+00	0.61	ACTB, DHX9, NPM1, TMEM109, TNKS1BP1, XRCC5
1.00E+00	0.61	BCAP31, DAZAP1, DSG1, ITGB1, KRT9, SOD1
1.00E+00	0.59	BCAP31, DAZAP1, ITGB1, KRT9, SOD1
1.00E+00	0.38	RAC2
1.00E+00	0.38	RAC2
1.00E+00	0.38	ADAM10
1.00E+00	0.37	HNRNPU
1.00E+00	0.37	NANS
1.00E+00	0.37	RAB10
1.00E+00	0.36	SRI
1.00E+00	0.36	PRDX6
1.00E+00	0.36	SRI
1.00E+00	0.36	ANXA2
1.00E+00	0.45	CALM3, CLIC1
1.00E+00	0.43	ATP2A3, ATP5F1B

1.00E+00	0.48	CCL5, ILK, PKM
1.00E+00	0.33	THBS1
1.00E+00	0.42	CD9, ITGB1
1.00E+00	0.33	SOD1
1.00E+00	0.47	ADAM10, SOD1, VIM
1.00E+00	0.33	ITGB3
1.00E+00	0.33	GNAQ
1.00E+00	0.41	ANXA2, ATP2A3
1.00E+00	0.56	BCAP31, CSDE1, DAZAP1, KRT9, NASP, SOD1
1.00E+00	0.33	THBS1
1.00E+00	0.66	ACTB, ARPC5, DHX9, H2AC20, H2BC21, HNRNPA2B1, HNRNPC, HNRNPU, NASP, NCL, RCC
1.00E+00	0.41	CDC42, UBE2L3
1.00E+00	0.32	MYH9
1.00E+00	0.40	CDC42, SOD1
1.00E+00	0.49	CCL5, ILK, PGK1, PKM
1.00E+00	0.31	SOD1
1.00E+00	0.31	XRCC5
1.00E+00	0.30	PLEK
1.00E+00	0.44	ANXA2, ATP2A3, ATP5F1B
1.00E+00	0.39	CDC42, UBE2L3
1.00E+00	0.51	CCL5, ILK, NANS, PGK1, PKM
1.00E+00	0.38	ACTB, KANSL3
1.00E+00	0.29	UBE2L3
1.00E+00	0.28	NPM1
1.00E+00	0.37	GNAQ, RCC2
1.00E+00	0.37	GNAQ, RCC2
1.00E+00	0.28	CDC42
1.00E+00	0.28	ENO1
1.00E+00	0.37	CCL5, PF4V1
1.00E+00	0.28	ENO1
1.00E+00	0.36	CCL5, PF4V1
1.00E+00	0.27	CDC42
1.00E+00	0.54	CD36, ENO1, FLNA, HNRNPA2B1, HNRNPU, HSPA8, XRCC5, YWHAZ
1.00E+00	0.41	ANXA2, ATP2A3, ATP5F1B
1.00E+00	0.54	CD36, ENO1, FLNA, HNRNPA2B1, HNRNPU, HSPA8, XRCC5, YWHAZ
1.00E+00	0.54	CD36, ENO1, FLNA, HNRNPA2B1, HNRNPU, HSPA8, XRCC5, YWHAZ
1.00E+00	0.54	ACTB, DHX9, H2AC20, H2BC21, HNRNPC, NASP, TCP1, TNKS1BP1
1.00E+00	0.26	VIM
1.00E+00	0.35	CALM3, LBP
1.00E+00	0.26	KANSL3
1.00E+00	0.40	CCL5, ILK, PKM
1.00E+00	0.47	CD36, ENO1, HNRNPA2B1, HNRNPU, YWHAZ
1.00E+00	0.25	FLNA
1.00E+00	0.33	B2M, CD36
1.00E+00	0.25	ILK
1.00E+00	0.42	ACTB, KANSL3, UBE2L3, XRCC5
1.00E+00	0.33	CCL5, PF4V1

1.00E+00	0.62	ACTB, DHX9, ENO1, G3BP1, H2AC20, H2BC21, HNRNPA2B1, HNRNPC, HNRNPU, HSPD1, I
1.00E+00	0.23	VIM
1.00E+00	0.23	KANSL3
1.00E+00	0.23	FLNA
1.00E+00	0.23	VIM
1.00E+00	0.23	RAB1B
1.00E+00	0.23	VIM
1.00E+00	0.22	NPM1
1.00E+00	0.22	DHX9
1.00E+00	0.22	DHX9
1.00E+00	0.47	ANTXR2, FCER1G, GP1BA, GP1BB, M6PR, PECAM1, SPN
1.00E+00	0.21	ILK
1.00E+00	0.20	B2M
1.00E+00	0.57	ACTB, ANXA2, CD36, DHX9, EIF5A, ENO1, HNRNPA2B1, HNRNPU, ITGA6, JUP, KANSL3, N(
1.00E+00	0.46	DHX9, ENO1, HNRNPA2B1, HNRNPU, HSPD1, NCL, NPM1, PCBP1, XRCC5
1.00E+00	0.26	DHX9, HNRNPA2B1
1.00E+00	0.17	ANXA2
1.00E+00	0.55	ACTB, ANXA2, CD36, DHX9, EIF5A, ENO1, HNRNPA2B1, HNRNPU, ITGA6, JUP, KANSL3, N(
1.00E+00	0.50	ATIC, CCL5, CDC42, GAPDH, GSTP1, HMBS, ILK, NANS, PGK1, PKM, PRDX6, TKT, UBE2L3
1.00E+00	0.26	B2M, CD36, SOD1
1.00E+00	0.14	DHX9
1.00E+00	0.14	DHX9
1.00E+00	0.14	DHX9
1.00E+00	0.34	ARL6IP5, B2M, CD36, HSP90AA1, ITGB1, SOD1
1.00E+00	0.33	DHX9, ENO1, HNRNPU, HSPD1, NPM1, XRCC5
1.00E+00	0.28	DHX9, ENO1, HNRNPU, NPM1, XRCC5
1.00E+00	0.28	DHX9, ENO1, HNRNPU, NPM1, XRCC5
1.00E+00	0.31	DHX9, ENO1, HNRNPU, HSPD1, NPM1, XRCC5
1.00E+00	0.10	GP1BA
1.00E+00	0.31	DHX9, ENO1, HNRNPU, JUP, NPM1, PCBP1, UBE2L3
1.00E+00	0.14	DHX9, HNRNPU
1.00E+00	0.18	DHX9, ENO1, HNRNPU
1.00E+00	0.14	DHX9, HNRNPU
1.00E+00	0.12	ENO1, PCBP1
1.00E+00	0.12	ENO1, PCBP1

TP5F1B, B2M, CAMP, CANX, CCT2, CCT4, CCT6A, CCT7, CCT8, CD47, CD53, CD59, CD9, CDC42, CFL1, CLI
TP5F1B, B2M, CAMP, CANX, CCT2, CCT4, CCT6A, CCT7, CCT8, CD47, CD53, CD59, CD9, CDC42, CFL1, CLI
TP5F1B, B2M, CAMP, CANX, CCT2, CCT4, CCT6A, CCT7, CCT8, CD47, CD53, CD59, CD9, CDC42, CFL1, CLI
TP5F1B, B2M, CAMP, CANX, CCT2, CCT4, CCT6A, CCT7, CCT8, CD47, CD53, CD59, CD9, CDC42, CFL1, CLI
5, ATIC, ATP5F1B, B2M, BCAP31, CALM3, CAMP, CANX, CCT2, CCT4, CCT6A, CCT7, CCT8, CD36, CD47, CD
TP5F1B, B2M, CAMP, CANX, CCL5, CCT2, CCT4, CCT6A, CCT7, CCT8, CD36, CD47, CD53, CD59, CD9, CDC
5, ATIC, ATP5F1B, B2M, CAMP, CANX, CCL5, CCT2, CCT4, CCT6A, CCT7, CCT8, CD36, CD47, CD53, CD59,
36, CD47, CD53, CD59, CD9, CTSD, CYB5R3, CYBB, DSC1, DSG1, DSP, EEF1A1, EEF2, FCER1G, FCN1, FER
CT8, CD36, CD47, CD53, CD59, CD9, CLTA, CTSD, CYB5R3, CYBB, DSC1, DSG1, DSP, EEF1A1, EEF2, FCER1
LNA, FLOT1, G3BP1, HSPA1B, HSPA8, HSPA9, ILK, ITGA2B, ITGA6, ITGB1, ITGB3, JUP, LRP1, MSN, MYH9,
T1, G3BP1, HSPA1B, HSPA8, HSPA9, ILK, ITGA2B, ITGA6, ITGB1, ITGB3, JUP, LRP1, MSN, MYH9, NPM1, P
M3, CAMP, CANX, CCT2, CCT4, CCT8, CD36, CD47, CD53, CD59, CD9, CDC42, CLTA, CTSD, CYB5R3, CYBB,
M3, CAMP, CANX, CCT2, CCT4, CCT8, CD36, CD47, CD53, CD59, CD9, CDC42, CLTA, CTSD, CYB5R3, CYBB,
F2, ENO1, F11R, FERMT3, FLNA, FSCN1, HSPA8, ICAM2, ITGA2B, ITGA6, ITGB1, ITGB3, JUP, LDHA, MSN,
ANXA7, ARL6IP5, ARPC5, ATIC, ATP2A3, ATP5F1B, B2M, BCAP31, CALM3, CAMP, CANX, CASP14, CAVIN2,
CASP14, CAVIN2, CCT2, CCT4, CCT6A, CCT7, CCT8, CD151, CDC42, CFL1, CLTA, CSDE1, CYB5R3, DAZAP1,
RMT3, GSTP1, HRNR, HSP90AA1, HSPA8, JUP, LGALS3BP, LTF, LYZ, MMRN1, PKM, PPIA, PRDX6, PSMA5,
RMT3, GSTP1, HRNR, HSP90AA1, HSPA8, JUP, LGALS3BP, LTF, LYZ, MMRN1, PKM, PPIA, PRDX6, PSMA5,
RMT3, GSTP1, HRNR, HSP90AA1, HSPA8, JUP, LGALS3BP, LTF, LYZ, MMRN1, PKM, PPIA, PRDX6, PSMA5,
FL1, DSC1, DSG1, DSP, F11R, FERMT3, FLNA, FLOT1, FSCN1, G3BP1, HSPA1B, HSPA8, HSPA9, ILK, ITGA2B
, JUP, LDHA, MYH9, PCBP1, PFN1, PKM, PRDX1, PRDX6, PTPRJ, RAB10, RTN4, SPTBN1, TAGLN2, TLN1, T
ARL6IP5, ARPC5, ATIC, ATP2A3, ATP5F1B, B2M, BCAP31, C16orf54, CALM3, CANX, CASP14, CAVIN2, CCL
ATP5F1B, B2M, BCAP31, CALM3, CCL5, CCT2, CCT4, CCT6A, CCT7, CCT8, CD36, CD59, CD9, CFL1, CLIC1,
D99, CDC42, CFL1, CLTA, DSC1, DSG1, DSP, EEF2, F11R, FERMT3, FLNA, FLOT1, FSCN1, G3BP1, GNAI2, G
P5, ATIC, ATP2A3, ATP5F1B, B2M, BCAP31, C16orf54, CALM3, CANX, CASP14, CAVIN2, CD151, CD226, C

1BA, GP1BB, ILK, ITGB3, KRT1, MMRN1, MYH9, PF4V1, PLEK, PPIA, PRDX2, THBS1, TLN1, VCL
1BA, GP1BB, ILK, ITGB3, KRT1, MMRN1, MYH9, PF4V1, PLEK, PPIA, PRDX2, THBS1, TLN1, VCL
1BA, GP1BB, ILK, ITGB3, KRT1, MMRN1, MYH9, PF4V1, PLEK, PPIA, PRDX2, THBS1, TLN1, VCL

T7, CCT8, CDC42, CFL1, CLTA, DHX9, DSP, EEF1A1, FERMT3, FLNA, FLOT1, FSCN1, G6PD, GAPDH, GNAI2,
9, CFL1, CLTA, DHX9, EEF2, EIF5A, F11R, FCER1G, FERMT3, FLNA, FSCN1, GNAI2, GNAQ, HLA-A, HNRNPC
IX9, DSP, EEF1A1, EEF2, EIF5A, ENO1, FLNA, FSCN1, G3BP1, HLA-A, HNRNPA2B1, HNRNPA3, HNRNPC, H
2A3, B2M, BCAP31, CALM3, CAMP, CANX, CCT2, CCT8, CD36, CD47, CD53, CD59, CD9, CDC42, CLIC1, C
AI2, GNAQ, GP1BA, GP1BB, HRNR, ILK, ITGB3, KRT1, KRT16, MMRN1, MYH9, PF4V1, PLEK, PPIA, PRDX2,
, KRT16, KRT17, KRT2, KRT5, KRT6A, KRT6B, KRT78, KRT9, MATR3, MMRN1, MSN, MYL6, RPL11, RPL8,

IA, GNAQ, GP1BA, GP1BB, ILK, ITGB1, ITGB3, KRT1, KRT6A, MMRN1, MYH9, PF4V1, PLEK, PPIA, PRDX2,

, DHX9, EEF1A1, EIF5A, ENDOD1, ENO1, F11R, FCER1G, FCN1, G3BP1, GAPDH, GSDMA, GSTP1, H2BC21,

:DC42, CLIC1, DSC1, DSG1, DSP, F11R, FERMT3, FLG2, FLNA, FLOT1, GP1BA, GP1BB, HLA-A, HSPD1, ICAM
, ARL6IP5, ARPC5, ATIC, ATP2A3, ATP5F1B, B2M, BCAP31, CALM3, CAMP, CANX, CASP14, CAVIN2, CCT2,
G, HLA-B, ITGA2B, ITGB3, PECAM1, PTPRJ, RAB10, RAB27B, RAP1B, RHOA, SLC2A3, SRI, STOM, TSPAN14

, ATP5F1B, B2M, BCAP31, CALM3, CANX, CASP14, CAVIN2, CD151, CD226, CD36, CD47, CD53, CD59, CD
IA, FLOT1, GP1BA, HLA-A, HSPD1, ICAM2, ILK, ITGA2B, ITGA6, ITGB1, ITGB3, JUP, MMRN1, MSN, MYADI

, HNRNPU, ILK, JUP, KRT1, KRT10, KRT14, KRT15, KRT16, KRT17, KRT2, KRT5, KRT6A, KRT6B, KRT78, KRT
, ARL6IP5, ARPC5, ATIC, ATP2A3, ATP5F1B, B2M, BCAP31, CALM3, CAMP, CANX, CASP14, CAVIN2, CCT2,
IA, GNAQ, GP1BA, GP1BB, ILK, ITGB1, ITGB3, KRT1, KRT6A, MMRN1, MYH9, PF4V1, PLEK, PPIA, PRDX2, F
, HNRNPU, ILK, JUP, KRT1, KRT10, KRT14, KRT15, KRT16, KRT17, KRT2, KRT5, KRT6A, KRT6B, KRT78, KRT
ANXA7, ARL6IP5, ARPC5, ATIC, ATP2A3, ATP5F1B, B2M, BCAP31, C16orf54, CALM3, CAMP, CANX, CASP1
HX9, DSP, ENO1, FLNA, G3BP1, HNRNPU, ILK, JUP, KRT1, KRT10, KRT14, KRT15, KRT16, KRT17, KRT2, KR

:D47, CDC42, CFL1, CSDE1, CTSD, DHX9, DSP, F11R, FLNA, FSCN1, G3BP1, GAPDH, HNRNPA2B1, HNRNPC
, CCT7, CCT8, CD151, CD36, CD47, CDC42, CLIC1, CTSD, CYBB, DHX9, FCER1G, FCN1, FLNA, FLOT1, G6PD
ER1G, FCN1, FLOT1, G6PD, GNAI2, GNAQ, GNG5, GP1BA, GSR, HLA-A, HLA-B, HSPA8, ITGA2B, ITGA6, ITG

EEF1A1, EEF2, ENO1, FLNA, FLOT1, GSTP1, HINT1, HNRNPU, HSP90AA1, HSPA1B, HSPA8, HSPA9, HSPD1
3CAP31, CALM3, CANX, CASP14, CAVIN2, CD151, CD226, CD36, CD47, CD53, CD59, CD9, CD99, CDC42, C
F1B, B2M, BCAP31, CALM3, CANX, CCL5, CCT2, CCT4, CCT6A, CCT7, CCT8, CD151, CD36, CD47, CD9, CDC
1, CD226, CD36, CD47, CD59, CD9, CDC42, CLIC1, CYBB, DCD, DDX60L, DHX9, DSP, ENDOD1, ENO1, F11
TA, CSDE1, CTSD, DHX9, DSG1, F11R, FERMT3, FLNA, FLOT1, FSCN1, G3BP1, H2BC21, HNRNPU, HSP90AA
:T2, CCT4, CCT6A, CCT7, CCT8, CD36, CDC42, CFL1, CLTA, CTSD, DHX9, DSP, F11R, FCN1, FLNA, FLOT1, G
RMT3, FLNA, GNAQ, GP1BA, GP1BB, HLA-A, HSPD1, ILK, INPP5D, ITGB1, ITGB3, KRT2, LBP, LRP1, MMRN1
B1, ITGB3, JUP, MSN, MYADM, MYH9, PATJ, PECAM1, PTPRJ, RAB10, RAP1B, RHOA, SLC2A1, TLN1, TNKS
9, CDC42, CTSD, CYBB, DHX9, EEF2, ENDOD1, F11R, FCER1G, FCN1, FLNA, FLOT1, G3BP1, G6PD, GAPDH,

, CCT7, CCT8, CD36, CDC42, CLTA, CTSD, DHX9, DSP, F11R, FCN1, FLNA, FLOT1, HNRNPU, HSP90AA1, HS
, CCT7, CCT8, CD36, CDC42, CLTA, CTSD, DHX9, DSP, F11R, FCN1, FLNA, FLOT1, HNRNPU, HSP90AA1, HS
ANXA7, ARL6IP5, ARPC5, ATIC, ATP2A3, ATP5F1B, B2M, BCAP31, CALM3, CAMP, CANX, CASP14, CAVIN2,

I, CCT6A, CCT7, CCT8, CD36, CDC42, CLTA, CTSD, DHX9, DSP, F11R, FCN1, FLNA, FLOT1, HNRNPA2B1, HN
CD53, CD59, CD9, ENO1, FCER1G, FCN1, FLOT1, GP1BA, GSR, HLA-A, HLA-B, HNRNPU, HSP90AA1, HSPD
, CCL5, CCT2, CCT4, CCT6A, CCT7, CCT8, CD151, CD226, CD36, CD47, CD53, CD59, CD99, CDC42, CFL1, C
I, BCAP31, CALM3, CANX, CCL5, CCT2, CCT4, CCT6A, CCT7, CCT8, CD151, CD36, CD47, CD9, CDC42, CFL

R1G, FCN1, FLOT1, G3BP1, GAPDH, HLA-A, HNRNPC, HNRNPU, HSP90AA1, HSPA1B, HSPA8, HSPD1, ITGB
T4, CCT6A, CCT7, CCT8, CD151, CD36, CD47, CD53, CD9, CDC42, CFL1, CLTA, CSDE1, CTSD, DHX9, DSG1,
CD53, CD59, CD9, CDC42, CLTA, CTSD, CYB5R3, CYBB, DSC1, DSG1, DSP, EEF1A1, ENO1, FCER1G, FLOT1,
:D36, CD47, CD53, CDC42, CFL1, EIF5A, ENO1, F11R, FLNA, FLOT1, FSCN1, G3BP1, G6PD, HNRNPA2B1, H
TA, CSDE1, CTSD, DHX9, DSG1, F11R, FERMT3, FLNA, FLOT1, FSCN1, G3BP1, H2BC21, HNRNPU, HSP90AA
:ALM3, CAMP, CANX, CASP14, CD151, CD36, CD53, CD9, CDC42, CFL1, CLIC1, CSDE1, CYBB, DAZAP1, DH
T4, CCT6A, CCT7, CCT8, CD151, CD36, CD47, CD53, CD9, CDC42, CFL1, CLTA, CSDE1, CTSD, DHX9, DSG1,

NPU, HSPA1B, ITGB1, ITGB3, KRT1, KRT14, KRT15, KRT16, KRT17, KRT2, KRT5, KRT6A, KRT6B, KRT78, KRT
M, BCAP31, CALM3, CANX, CCL5, CCT2, CCT4, CCT7, CCT8, CD151, CD36, CD47, CD9, CDC42, CLIC1, CLTA
CT2, CCT4, CCT6A, CCT7, CCT8, CD151, CD226, CD36, CD47, CD53, CD99, CDC42, CLIC1, CSDE1, CTSD, D

B, HSPA8, ITGB1, KRT1, KRT14, KRT15, KRT16, KRT17, KRT2, KRT5, KRT6A, KRT6B, KRT78, KRT9, MYADM
AMP, CASP14, CD151, CD36, CD53, CD9, CDC42, CFL1, CSDE1, CYBB, DAZAP1, DSP, EEF2, F11R, FCER1G,

A, HSP90AA1, HSPA8, ITGB1, ITGB3, JUP, KRT5, LRP1, MSN, NPM1, PECAM1, PPIA, RAC2, RTN4, SPTBN1,
6, KRT17, KRT2, KRT5, KRT6A, KRT6B, KRT78, KRT9, MSN, MYADM, PECAM1, PGK1, RAB10, RAP1B, RHO
5, ARPC5, ATP2A3, ATP5F1B, B2M, BCAP31, CALM3, CAMP, CANX, CASP14, CAVIN2, CCT2, CCT4, CCT6A,
GNAI2, GNAQ, GNG5, HSP90AA1, HSPA1B, HSPA8, HSPA9, HSPD1, RAB10, RAB1B, RAB27B, RAB33B, RAC
SD, DHX9, FLNA, HSP90AA1, HSPA8, ITGB1, ITGB3, JUP, KRT5, LRP1, MSN, NPM1, PECAM1, PPIA, RAC2, I

EIF5A, ENDOD1, ENO1, FCER1G, FCN1, G3BP1, GAPDH, GSDMA, GSTP1, H2BC21, HLA-A, HLA-B, HSP90A

1, HSP90AA1, ILK, ITGB1, ITGB3, JUP, LDHB, LRBA, MSN, NPM1, PLEK, PTPRJ, RAC2, RCC2, RHOA, SLC2A1
ANX, CCL5, CD36, CD47, CD9, CDC42, CPN1, CYBB, DHX9, DSG1, EEF1A1, EIF5A, FCER1G, FLNA, FLOT1, G
K, CASP14, CCT2, CCT4, CCT6A, CCT7, CCT8, CDC42, CFL1, CLTA, CSDE1, CYB5R3, DHX9, DSP, EEF1A1, EN
K, CASP14, CCT2, CCT4, CCT6A, CCT7, CCT8, CDC42, CFL1, CLTA, CSDE1, CYB5R3, DHX9, DSP, EEF1A1, EN
), KRT14, KRT15, KRT16, KRT17, KRT2, KRT5, KRT6A, KRT6B, KRT78, KRT9, RAC2, RCC2, TCP1, TPM4, TUB
, CLTA, CTSD, CYBB, DSC1, DSG1, DSP, FCER1G, HLA-A, HLA-B, HSPA8, ITGA2B, ITGB1, ITGB3, LRP1, M6PF

B, DCD, DDX60L, DHX9, EIF5A, ENDOD1, ENO1, F11R, FCER1G, FCN1, FLNA, G3BP1, G6PD, GAPDH, GNAI
, CLTA, CTSD, CYBB, DSC1, DSG1, DSP, FCER1G, HLA-A, HLA-B, HSPA8, ITGA2B, ITGB1, ITGB3, LRP1, M6PF

YB5R3, CYBB, DDX60L, DHX9, EEF1A1, EEF2, FCN1, G3BP1, GNAI2, GNAQ, GSDMA, GSR, GSTP1, HNRNP
CYBB, DHX9, EEF1A1, EIF5A, ENO1, FCER1G, FLNA, FLOT1, G6PD, GAPDH, GNAI2, GNAQ, GSR, GSTP1, H
EIF5A, ENDOD1, ENO1, FCER1G, FCN1, G3BP1, GAPDH, GSDMA, GSTP1, H2BC21, HLA-A, HLA-B, HSP90A
EIF5A, ENDOD1, ENO1, FCER1G, FCN1, G3BP1, GAPDH, GSDMA, GSTP1, H2BC21, HLA-A, HLA-B, HSP90A

H, GSR, GSTP1, HNRNPU, KRT17, NCL, NPM1, PKM, RHOA, RPL11, RPL8, RPLP0, RPS15, RPS15A, RPS23,

GNAI2, GNAQ, GNG5, HSP90AA1, HSPA1B, HSPA8, HSPA9, HSPD1, RAB10, RAB1B, RAB27B, RAB33B, RAC
GNAI2, GNAQ, GNG5, HSP90AA1, HSPA1B, HSPA8, HSPA9, HSPD1, RAB10, RAB1B, RAB27B, RAB33B, RAC
GNAI2, GNAQ, GNG5, HSP90AA1, HSPA1B, HSPA8, HSPA9, HSPD1, RAB10, RAB1B, RAB27B, RAB33B, RAC
2, EIF5A, ENO1, FLOT1, HNRNPU, HSP90AA1, HSPA1B, HSPD1, ILK, ITGB3, KANSL3, KRT17, LRP1, LTF, MS
LIC1, DAZAP1, DHX9, DSP, EEF2, F11R, FCER1G, FERMT3, FLNA, FLOT1, G6PD, GP1BA, GP1BB, HLA-B, HN
1, HSP90AA1, ILK, ITGB1, ITGB3, JUP, LRBA, MSN, NPM1, PLEK, PTPRJ, RAC2, RCC2, RHOA, TFRC, TUBA4A

B1, KRT1, KRT10, KRT14, KRT15, KRT16, KRT17, KRT2, KRT5, KRT6A, KRT6B, KRT78, KRT9, MSN, MYADM

CLIC1, DAZAP1, DHX9, DSP, EEF2, F11R, FCER1G, FERMT3, FLNA, FLOT1, G6PD, GP1BA, GP1BB, HLA-B, HN
MT3, FLNA, FSCN1, GNAI2, GP1BA, HSP90AA1, ILK, ITGA6, ITGB1, ITGB3, JUP, KRT16, KRT2, KRT5, LBP, LR
LOT1, GP1BA, HLA-A, HSPD1, ILK, ITGB3, JUP, MMRN1, MYADM, PRDX2, PTPRJ, RAC2, RCC2, RHOA, SPN,
DDX60L, DHX9, EEF1A1, EEF2, G3BP1, G6PD, GAPDH, GNAI2, GNAQ, GSR, HINT1, HNRNPU, HSP90AA1, H

ER1G, FCN1, FLNA, FLOT1, G6PD, GNAI2, HSP90AA1, ITGB1, JUP, LRP1, MSN, PPIA, PTPRJ, RAB27B, RAC2
X9, EEF1A1, EEF2, EIF5A, ENO1, FLNA, FLOT1, G6PD, GAPDH, GNAI2, GNAQ, GSTP1, HNRNPU, HSP90AA1,

ATP5F1B, B2M, BCAP31, CALM3, CAMP, CASP14, CD226, CD36, CD47, CD59, CD9, CDC42, CFL1, CLIC1, C

, CYBB, DDX60L, DHX9, EEF1A1, EEF2, G3BP1, G6PD, GAPDH, GNAI2, GNAQ, GSR, HINT1, HNRNPU, HSP9
DC42, F11R, FCN1, FERMT3, FLNA, FLOT1, GNAI2, GNAQ, HLA-A, HLA-B, HSP90AA1, HSPA1B, HSPA8, ICAM
ATP5F1B, B2M, BCAP31, CALM3, CAMP, CANX, CCL5, CD151, CD226, CD36, CD47, CD53, CD59, CD9, CDC

11R, FCER1G, FLNA, G6PD, GALE, GAPDH, GP1BB, HNRNPC, HNRNPH1, HNRNPU, HSP90AA1, ITGA2B, ITG
, DHX9, F11R, FLOT1, GNAI2, GNAQ, HNRNPA2B1, HNRNPU, HSP90AA1, HSPA1B, HSPD1, ITGA6, ITGB1, I

DDX60L, DHX9, EEF1A1, EEF2, G3BP1, G6PD, GAPDH, GNAI2, GNAQ, GSR, HINT1, HNRNPU, HSP90AA1, H

A, CCT7, CCT8, CD226, CD36, CSDE1, CTSD, CYBB, DAZAP1, DHX9, EEF2, EIF5A, ENO1, FCER1G, FCN1, FLO

A, CCT7, CCT8, CD226, CD36, CDC42, CSDE1, CTSD, CYBB, DAZAP1, DHX9, EEF2, EIF5A, ENO1, FCER1G, FC

FCN1, G3BP1, GAPDH, GSDMA, H2BC21, HLA-A, HLA-B, HSP90AA1, KRT1, KRT16, KRT6A, LBP, LTF, LYZ, M

ER1G, FCN1, G3BP1, GAPDH, GSDMA, GSTP1, H2BC21, HLA-A, HLA-B, HSP90AA1, ITGB1, KRT1, KRT16, K

T6A, CCT7, CCT8, CDC42, CSDE1, CYB5R3, CYBB, DAZAP1, DCD, DDX60L, DHX9, DSP, EEF1A1, EEF2, EIF5A
T6A, CCT7, CCT8, CDC42, CSDE1, CYB5R3, CYBB, DAZAP1, DCD, DDX60L, DHX9, DSP, EEF1A1, EEF2, EIF5A

P5D, M6PR, MYH9, NCL, RAB27B, RCC2, RHOA, TLN1, TNKS1BP1, TUBB, VIM, WBP11, YWHAB, YWHAE, Y
ANXA7, ARL6IP5, ARPC5, ATIC, ATP2A3, ATP5F1B, B2M, BCAP31, C16orf54, CALM3, CAMP, CANX, CASP1
, FERMT3, FLNA, FSCN1, GNAI2, GP1BA, HSP90AA1, ILK, ITGA6, ITGB1, ITGB3, JUP, KRT16, KRT2, KRT5, L
TA, CTSD, FLNA, HSP90AA1, HSPA8, HSPA9, HSPD1, JUP, LRP1, M6PR, MYH9, NASP, NPM1, PLEK, PPIA, R

CD9, CDC42, CLTA, FCER1G, FCN1, FLNA, FLOT1, GNAI2, HSPA1B, HSPA8, ITGB1, ITGB3, LBP, LGALS3BP, L
7, CCT8, CD36, CD9, CDC42, CLIC1, CLTA, CSDE1, CYB5R3, CYBB, DAZAP1, DHX9, EEF1A1, EEF2, EIF5A, EN
5, ITGB3, JUP, PATJ, PECAM1, PFN1, PTPRJ, RAP1B, RCC2, RHOA, THBS1, TLN1, TUBB, VCL, YWHAZ

J, HSP90AA1, HSPA8, HSPD1, JUP, M6PR, MSN, NPM1, RAB10, RAB33B, RAC2, RCC2, RPL11, RTN4, SRGN
, G3BP1, HNRNPA2B1, HNRNPC, HNRNPU, HSPA1B, ITGB3, LRP1, MSN, MYADM, NPM1, PATJ, PFN1, PLE

CLIC1, CYBB, DHX9, FCER1G, FCN1, FLNA, FLOT1, G6PD, GNAI2, HSP90AA1, HSPA8, ITGB1, ITGB3, JUP, LI

FLNA, FLOT1, FSCN1, G3BP1, HNRNPA2B1, HSP90AA1, HSPA1B, ITGA6, LRP1, MSN, PFN1, PLEK, PTPRJ, I

EIF5A, F11R, FCER1G, FCN1, FLNA, FLOT1, GAPDH, GNAI2, HINT1, HLA-A, HLA-B, HSP90AA1, HSPA1B, HS
CANX, CASP14, CCL5, CD36, CDC42, CPN1, CSDE1, CTSD, DCD, DHX9, DSP, EEF1A1, EEF2, EIF5A, ENO1, F
SPA1B, HSPA8, MSN, MYADM, NPM1, PFN1, PLEK, PTPRJ, RAB1B, RAC2, RAP1B, RCC2, RHOA, SPTBN1, TF

ALM3, CANX, CD36, CD47, CD53, CD59, CD9, CDC42, CLIC1, CLTA, CTSD, CYB5R3, CYBB, DSC1, DSG1, DSI

9, CD9, CDC42, DHX9, EIF5A, ENO1, F11R, FCER1G, FCN1, FLNA, FLOT1, G3BP1, G6PD, GAPDH, GNAI2, G
, ENO1, FLNA, G6PD, GAPDH, GNAI2, GSDMA, GSTP1, HINT1, HSP90AA1, HSPA1B, HSPA9, HSPD1, INPP5
CD47, CD9, CFL1, CYBB, DHX9, DSP, ENO1, F11R, FCER1G, FCN1, FLNA, FLOT1, G3BP1, G6PD, GAPDH, GN

5, ARPC5, ATP2A3, ATP5F1B, B2M, BCAP31, CALM3, CAMP, CANX, CASP14, CAVIN2, CCT2, CCT4, CCT8, C

Q, HRNR, HSP90AA1, HSPA8, LRBA, LRP1, LYZ, M6PR, PCYOX1, PRDX6, RAP1B, SOD1, SRGN, STOM, TUBE
, CAMP, CANX, CCL5, CD36, CD47, CD9, CDC42, CPN1, CYBB, DHX9, DSG1, EEF1A1, EIF5A, ENO1, FCER1G

CN1, FLOT1, G3BP1, GAPDH, H2BC21, HLA-A, HLA-B, HSP90AA1, HSPA1B, HSPD1, INPP5D, KRT1, KRT16,
G6PD, GNAI2, GP1BA, GP1BB, GSR, GSTP1, HRNR, HSPA1B, HSPA9, INPP5D, ITGB1, ITGB3, KRT1, KRT16,
L, FLOT1, GP1BA, HLA-A, HLA-B, HSP90AA1, HSPA1B, HSPA9, HSPD1, INPP5D, ITGB3, KRT1, LBP, LTF, MA
H2BC21, HSP90AA1, HSPA1B, HSPA8, HSPD1, KRT1, KRT10, KRT5, MSN, MYADM, NASP, NPM1, OSBPL2

AA1, HSPA1B, ILK, ITGB1, ITGB3, LTF, PPIA, PRDX2, PTBP1, PTPRJ, RAP1B, RHOA, RPL11, RPS15, RTN4, S1
MT3, FSCN1, H2BC21, HSP90AA1, HSPA1B, HSPA8, HSPD1, KRT1, KRT10, KRT5, MSN, MYADM, NASP, NF
H, GSR, GSTP1, HNRNPU, KRT17, NCL, NPM1, PKM, RHOA, RPL11, RPL8, RPLP0, RPS15, RPS15A, RPS23,

CAP31, CALM3, CAMP, CANX, CCL5, CD226, CD36, CD47, CD53, CD59, CD9, CDC42, CFL1, CLIC1, CYBB, DI

GNAQ, HNRNPA2B1, HNRNPU, HSP90AA1, HSPD1, ITGA6, ITGB1, ITGB3, LTF, NPM1, PLEK, PPIA, RCC2,

A6, ITGB1, ITGB3, JUP, KRT16, KRT5, LBP, LRP1, MSN, MYADM, PECAM1, PFN1, PTPRJ, RAC2, RCC2, RHOA

HNRNPU, HRNR, ILK, ITGA6, ITGB1, ITGB3, KRT1, KRT10, KRT14, KRT15, KRT16, KRT17, KRT2, KRT5, KRT6

DHX9, EEF1A1, EEF2, FCN1, G3BP1, GNAI2, GNAQ, HNRNPU, HSP90AA1, HSPA1B, HSPA8, HSPA9, HSPD
P, EEF2, FCER1G, FLG2, FLNA, FLOT1, G6PD, GNG5, GP1BA, GP1BB, HLA-B, HNRNPU, HRNR, HSPA1B, HS

, FLNA, G6PD, GAPDH, GNAI2, GSDMA, GSTP1, HINT1, HSP90AA1, HSPA1B, HSPA9, HSPD1, INPP5D, ITG
H, H2BC21, HLA-A, HLA-B, HSP90AA1, KRT1, KRT16, LBP, LTF, MATR3, PRDX1, S100A8, S100A9, SYNCRIP,

EEF1A1, EEF2, G3BP1, GNAI2, GNAQ, HNRNPU, HSP90AA1, HSPA1B, HSPA8, HSPA9, HSPD1, ILK, MYH9,

TP1, HLA-A, HLA-B, HSP90AA1, HSPA1B, HSPD1, INPP5D, LBP, LTF, PTPRJ, SOD1, SPN, SPTBN1, SRGN, TH

90AA1, ITGB1, KRT2, MSN, MYH9, MYL12B, PFN1, RAB10, RAB27B, RCC2, RHOA, S100A8, S100A9, SPTBN

TP1, HLA-A, HLA-B, HSP90AA1, HSPA1B, HSPD1, INPP5D, LBP, LTF, PTPRJ, SOD1, SPN, SPTBN1, SRGN, TH

RL6IP5, ARPC5, ATIC, ATP2A3, ATP5F1B, B2M, BCAP31, CALM3, CAMP, CANX, CASP14, CAVIN2, CCL5, CC

FLNA, HNRNPA2B1, HNRNPU, HSP90AA1, HSPA8, HSPA9, HSPD1, ITGB1, ITGB3, JUP, LRP1, M6PR, MSN, I

, HSP90AA1, HSPA8, LRBA, LRP1, LYZ, M6PR, PCYOX1, PRDX6, RAP1B, SOD1, SRGN, STOM, TUBB
, HSP90AA1, HSPA8, LRBA, LRP1, LYZ, M6PR, PCYOX1, PRDX6, RAP1B, SOD1, SRGN, STOM, TUBB

EIF5A, FCER1G, FLNA, FLOT1, GAPDH, GNAI2, GSTP1, HNRNPU, HSPA1B, HSPA8, HSPA9, HSPD1, ILK, ITGE
1, G3BP1, GAPDH, GNAI2, HLA-A, HSP90AA1, HSPA1B, HSPD1, INPP5D, ITGB1, ITGB3, JUP, KRT17, LBP, L

, G6PD, GAPDH, GNAI2, GSDMA, GSTP1, HINT1, HSP90AA1, HSPA1B, HSPA9, HSPD1, INPP5D, ITGA6, ITG
, LBP, LTF, MSN, NASP, PF4V1, PRDX2, RHOA, RPLP0, S100A8, S100A9, TFRC, THBS1, UBE2L3, VIM, XRCC

6, ITGB1, ITGB3, JUP, KRT16, KRT5, LBP, LRP1, MSN, MYADM, PECAM1, PFN1, PTPRJ, RAC2, RCC2, RHOA

0AA1, HSPA1B, HSPD1, INPP5D, KRT1, LBP, LTF, MATR3, PTPRJ, RAC2, RTN4, SPN, TFRC, TTLL12, UBQLN

HNRNPH1, HNRNPH3, HNRNPU, HSPA1B, HSPA8, NCL, NPM1, PCBP1, PSMA6, RPL11, RPL8, RPLP0, RPS
AI2, GSTP1, HSP90AA1, HSPA1B, HSPA9, HSPD1, INPP5D, ITGA6, ITGB1, ITGB3, LRP1, LTF, NPM1, PPIA, P

, GNAI2, GSTP1, HINT1, HSP90AA1, HSPA1B, ILK, ITGB1, ITGB3, LTF, MYADM, NPM1, PLEK, PPIA, PRDX1,
HOA, RPL11, RPL8, RPLP0, RPS15, RPS15A, RPS23, RPS3A, RPSA, SYNCRIP, THBS1, TNKS1BP1, VIM
EEF1A1, EEF2, G3BP1, GNAI2, GNAQ, HNRNPU, HSP90AA1, HSPA1B, HSPA8, HSPA9, HSPD1, ILK, MYH9, I

CYBB, DSP, F11R, FERMT3, FLG2, FLNA, FLOT1, GNG5, GP1BA, HSP90AA1, ILK, ITGA2B, ITGA6, ITGB1, ITG

PU, HSPA1B, ILK, NCL, PF4V1, PTPRJ, RHOA, RPLP0, SELPLG, SYNCRIP, TFRC, THBS1, TTLL12, VIM, XRCC5
BCAP31, CALM3, CAMP, CANX, CASP14, CCL5, CD36, CDC42, CPN1, CSDE1, CTSD, DCD, DHX9, DSP, EEF1
T1, HNRNPA2B1, HSP90AA1, HSPA8, HSPA9, HSPD1, ITGB1, ITGB3, JUP, LBP, LRP1, M6PR, MYH9, NASP,
HNRNPU, HSP90AA1, ILK, ITGA6, ITGB3, LRP1, LTF, MYADM, NCL, NPM1, PECAM1, PLEK, PPIA, PTBP1, P

HNRNPU, HSP90AA1, ILK, ITGA6, ITGB3, LRP1, LTF, MYADM, NCL, NPM1, PECAM1, PLEK, PPIA, PTBP1, P

A-A, HLA-B, HSP90AA1, KRT1, LBP, LTF, MATR3, MMRN1, PRDX2, PSMA6, RAC2, S100A8, S100A9, SOD1,
6, ITGB1, ITGB3, JUP, KRT16, KRT5, LBP, LRP1, MSN, MYADM, PECAM1, PFN1, PTPRJ, RAC2, RCC2, RHO

NA, GP1BA, GSTP1, HLA-A, HLA-B, HSP90AA1, ITGB1, KRT1, LBP, MATR3, MMRN1, NPM1, PNPLA8, PPIA,
SCN1, HSP90AA1, HSPA8, HSPD1, ICAM2, ITGB1, ITGB3, JUP, LRP1, MSN, MYH9, PATJ, PLEK, PTPRJ, RAB
O1, FLOT1, G6PD, GAPDH, GNAI2, GNAQ, GSTP1, HINT1, HNRNPU, HSP90AA1, HSPA1B, HSPA8, ILK, INP

HOA, RPL11, RPL8, RPLP0, RPS15, RPS15A, RPS23, RPS3A, RPSA, SYNCRIP, THBS1, TNKS1BP1, VIM
EEF1A1, EEF2, G3BP1, GNAI2, GNAQ, HNRNPU, HSP90AA1, HSPA1B, HSPA8, HSPA9, HSPD1, ILK, MYH9,
1, HSP90AA1, ITGB3, JUP, LBP, LRP1, LTF, MSN, NASP, NCL, PF4V1, PKM, PRDX1, PRDX2, RAB10, RAP1B,

IAI2, GNAQ, HSP90AA1, ITGB1, ITGB3, JUP, LRP1, NCL, NPM1, PKM, RAB10, RAP1B, RHOA, RPLP0, SLC2A
, CDC42, CTSD, DHX9, EEF1A1, F11R, FLNA, FLOT1, GAPDH, GNAI2, GNAQ, GSTP1, HNRNPA2B1, HNRNP
TP1, HSP90AA1, HSPA1B, HSPA9, HSPD1, INPP5D, ITGA6, ITGB1, LTF, NPM1, PPIA, PRDX2, RHOA, RPL11
-A, HLA-B, HSP90AA1, HSPD1, INPP5D, ITGB3, KRT1, LBP, MATR3, PTPRJ, RAC2, RHOA, SPN, TFRC, THBS
LBA, ITGA6, ITGB1, ITGB3, JUP, KRT16, KRT5, LBP, LRP1, MSN, MYADM, PECAM1, PF4V1, PFN1, PPIA, PT

NPA2B1, HSP90AA1, HSPA8, HSPA9, HSPD1, ITGB1, ITGB3, JUP, LRP1, M6PR, MYH9, NASP, NPM1, PLEK,

BB, EEF1A1, FERMT3, FLNA, FLOT1, FSCN1, GNAI2, GNAQ, HNRNPU, HSP90AA1, HSPA8, HSPD1, ICAM2,
EEF1A1, EEF2, G3BP1, GNAI2, GNAQ, HNRNPU, HSP90AA1, HSPA1B, HSPA8, HSPA9, HSPD1, ILK, MYH9,

J, HSP90AA1, HSPA8, HSPA9, HSPD1, JUP, LRP1, M6PR, MSN, NPM1, OSBPL2, RAB10, RAB1B, RAB27B, R

NPU, HSP90AA1, HSPA1B, HSPA8, JUP, MSN, NASP, NPM1, RAP1B, RHOA, RPLP0, SOD1, THBS1, UBE2L3,

O1, FLOT1, G6PD, GAPDH, GNAI2, GNAQ, GSTP1, HINT1, HNRNPU, HSP90AA1, HSPA1B, HSPA8, ILK, INPI

TP1, HNRNPU, HSPA1B, ILK, NCL, PF4V1, PTPRJ, RHOA, RPLP0, SELPLG, SYNCRIP, TFRC, THBS1, TTLL12, VI
2, CFL1, CSDE1, CYBB, DSP, EEF2, FCER1G, FLNA, FLOT1, G6PD, GNG5, GP1BA, GP1BB, GSTP1, HLA-B, HN

7, CCT8, CD36, CSDE1, CTSD, DAZAP1, DHX9, EEF2, EIF5A, ENO1, FLOT1, HNRNPA2B1, HNRNPU, HSP90A

9, CDC42, CFL1, CSDE1, DHX9, ENO1, F11R, FLNA, G3BP1, G6PD, GAPDH, GNAI2, GNAQ, GP1BA, GP1BB,

TP1, HSP90AA1, HSPA1B, HSPA9, HSPD1, INPP5D, ITGA6, ITGB1, LTF, NPM1, PPIA, PRDX2, RHOA, RPL11

CPN1, CTSD, DCD, DDX60L, DHX9, EEF1A1, EEF2, ENDOD1, G3BP1, GNAI2, GNAQ, GNG5, HINT1, HSP90A

3C21, HSP90AA1, ITGB1, JUP, KRT1, KRT10, MYH9, NPM1, PECAM1, PLEK, SRI, STOM, TFRC, TKT, TPM4,

D1, JUP, LRP1, M6PR, MYH9, NASP, NPM1, PLEK, PPIA, RAB10, RAB1B, RAB27B, RAB33B, RAC2, RPL11, S

PA1B, ITGB3, LRP1, LTF, MYADM, NCL, NPM1, PPIA, PTPRJ, RPL11, RPS15, RTN3, RTN4, SYNCRIP, THBS1,

HSP90AA1, ITGB3, LTF, NPM1, PPIA, PTPRJ, RHOA, RPL11, RPS15, SOD1, TCP1, THBS1, UBE2L3, XRCC5,

1B, ILK, ITGB3, KANSL3, LRP1, LTF, MYADM, NCL, NPM1, PECAM1, PPIA, PTBP1, PTPRJ, RHOA, RPL11, RI

HSPA9, INPP5D, ITGB1, LTF, MYH9, PLEK, PRDX2, PTPRJ, RHOA, SELPLG, SOD1, SPN, TFRC, XRCC5

CCT2, CCT4, CCT6A, CCT7, CCT8, CD226, CD36, CD47, CSDE1, CTSD, CYBB, DAZAP1, DHX9, EEF1A1, EEF2,

ANXA7, ARL6IP5, ARPC5, ATIC, ATP2A3, ATP5F1B, B2M, BCAP31, C16orf54, CALM3, CAMP, CANX, CASP1

3, B2M, BCAP31, CALM3, CAMP, CCL5, CCT2, CCT4, CCT6A, CCT7, CCT8, CD151, CD226, CD36, CD47, CD5

1R, FLNA, FLOT1, G3BP1, GAPDH, GNAI2, GNAQ, GSTP1, HINT1, HSP90AA1, HSPA1B, ILK, INPP5D, ITGA6,

2, CFL1, CSDE1, CYBB, DSP, EEF2, F11R, FCER1G, FLNA, FLOT1, G6PD, GNG5, GP1BA, GP1BB, GSTP1, HLA
, HINT1, HSP90AA1, HSPA1B, ILK, ITGB1, ITGB3, JUP, LBP, LTF, PPIA, PRDX2, PTBP1, PTPRJ, RAP1B, RHOA
CD47, CD59, CD9, CDC42, CFL1, CSDE1, DHX9, ENO1, F11R, FCN1, FLNA, G3BP1, G6PD, GAPDH, GNAI2, G

GAPDH, GNAI2, HINT1, HSP90AA1, HSPA1B, ILK, ITGB1, ITGB3, JUP, LBP, LTF, PPIA, PRDX2, PTBP1, PTPRJ

1R, FLNA, FLOT1, G6PD, HLA-B, HNRNPU, HSPA1B, HSPA9, ILK, INPP5D, ITGB1, ITGB3, JUP, KRT1, KRT10,

CAVIN2, CCT2, CCT4, CCT8, CFL1, CTSD, CYB5R3, DAZAP1, DHX9, EEF1A1, EEF2, FCN1, FERMT3, FLG2, FI
CAVIN2, CCT2, CCT4, CCT8, CFL1, CTSD, CYB5R3, DAZAP1, DHX9, EEF1A1, EEF2, FCN1, FERMT3, FLG2, FI
CAVIN2, CCT2, CCT4, CCT8, CFL1, CTSD, CYB5R3, DAZAP1, DHX9, EEF1A1, EEF2, FCN1, FERMT3, FLG2, FI

CDC42, EIF5A, ENO1, F11R, FLNA, FLOT1, G3BP1, GAPDH, GNAI2, GNAQ, GSTP1, HINT1, HSP90AA1, HSPA

HSPA9, INPP5D, ITGB1, LTF, MYH9, PLEK, PRDX2, PTPRJ, RHOA, SELPLG, SOD1, SPN, TFRC, XRCC5
CCT2, CCT4, CCT6A, CCT7, CCT8, CD226, CD36, CD47, CDC42, CSDE1, CTSD, CYBB, DAZAP1, DHX9, EEF1A

HSPA9, HSPD1, INPP5D, ITGB1, LTF, MYH9, PLEK, PRDX2, PTPRJ, RHOA, SELPLG, SOD1, SPN, TFRC, XRCC
FLNA, FLOT1, FSCN1, GNAI2, GNAQ, HNRNPU, HSP90AA1, HSPA8, HSPD1, ICAM2, ILK, ITGB1, ITGB3, MSN

NPU, ILK, ITGA2B, ITGB1, ITGB3, JUP, KRT1, LRP1, MATR3, MYH9, NCL, PECAM1, PGK1, PKM, RHOA, RTN

NG5, GSTP1, HLA-A, HNRNPU, HSPA1B, HSPD1, ILK, INPP5D, ITGB1, ITGB3, JUP, KRT2, KRT6A, LTF, MSN,
6, CDC42, CSDE1, DAZAP1, DHX9, EEF2, EIF5A, ENO1, FLOT1, GNAI2, GSTP1, HNRNPA2B1, HNRNPU, HSP

ATP2A3, ATP5F1B, B2M, BCAP31, CALM3, CAMP, CCL5, CCT2, CCT4, CCT6A, CCT7, CCT8, CD151, CD226,

P1, LTF, MSN, NCL, PF4V1, PKM, PRDX1, PRDX2, RAB10, RAP1B, RHOA, RPLP0, SOD1, SRI, VIM, XRCC5, Y

HNRNPH3, HNRNPU, HSPA1B, HSPA8, NCL, NPM1, PRDX6, PTBP1, SF3A3, SYNCRIP, TNKS1BP1, VIM, WBP

EEF1A1, ENO1, FLNA, GALE, GAPDH, HINT1, HNRNPC, HNRNPU, HSP90AA1, HSPA1B, HSPA8, ITGB1, LD

50L, DHX9, DSP, EEF1A1, EEF2, EIF5A, ENDOD1, ENO1, FLNA, FSCN1, G3BP1, H2AC20, H2BC21, HLA-A, H
HOA, RPL11, RPL8, RPLP0, RPS15, RPS15A, RPS23, RPS3A, RPSA, SYNCRIP, THBS1, TNKS1BP1, VIM
RPC5, ATP2A3, ATP5F1B, B2M, BCAP31, CALM3, CAMP, CCL5, CCT2, CCT4, CCT6A, CCT7, CCT8, CD151, C

GNAI2, HINT1, HSP90AA1, HSPA1B, ILK, ITGB1, ITGB3, JUP, LBP, LTF, PPIA, PRDX2, PTBP1, PTPRJ, RAP1B,
1, ITGB1, ITGB3, JUP, LRP1, NCL, PKM, RAB10, RAP1B, RHOA, RPLP0, SLC2A1, SOD1, UBQLN1, VIM, YWH

FLOT1, G3BP1, GAPDH, GNAI2, GSTP1, HINT1, HSP90AA1, HSPA1B, ILK, INPP5D, ITGB1, ITGB3, LTF, MYA
IF5A, ENO1, HLA-A, HLA-B, LRBA, PNPLA8, PTGS1, RAB10, RAB1B, RAC2, RHOA, RTN3, RTN4, SACM1L, SI

, HNRNPU, ITGB3, LRP1, LTF, NPM1, PPIA, PRDX2, PTPRJ, RCC2, RPL11, RPS15, SRI, THBS1, UBQLN1, YWI

ANXA7, ARL6IP5, ARPC5, ATIC, ATP2A3, ATP5F1B, B2M, BCAP31, CALM3, CAMP, CANX, CASP14, CAVIN2,

D, ITGB1, LBP, MSN, MYH9, PRDX1, PRDX2, PTPRJ, RAC2, RHOA, SELPLG, SOD1, SPN, TFRC, THBS1

DH, GNAQ, GSTP1, HNRNPU, HSP90AA1, ILK, ITGA6, ITGB3, LDHA, LRP1, LTF, MYADM, NCL, NPM1, PECA
90AA1, ILK, ITGA6, ITGB3, LRP1, LTF, MYADM, NCL, NPM1, PECAM1, PPIA, PTPRJ, RHOA, SOD1, TFRC, TH

D1, F11R, FLNA, FLOT1, G3BP1, GAPDH, GNAI2, GNAQ, GSTP1, HINT1, HSP90AA1, HSPA1B, ILK, INPP5D,

CYB5R3, CYBB, EIF5A, HLA-A, HLA-B, HSPA1B, LRBA, PKM, PNPLA8, PTGS1, QSOX1, RAB10, RAB1B, RAC2

IF5A, HLA-A, HLA-B, LRBA, PNPLA8, PTGS1, RAB10, RAB1B, RAC2, RHOA, RTN3, RTN4, SACM1L, SRI, TKT,

IF5A, HLA-A, HLA-B, LRBA, PNPLA8, PTGS1, RAB10, RAB1B, RAC2, RHOA, RTN3, RTN4, SACM1L, SRI, TKT,

5D, DHX9, EEF1A1, F11R, GAPDH, GNAI2, GNAQ, GSTP1, HNRNPA2B1, HNRNPU, HSP90AA1, HSPD1, ITGA

I2, HNRNPU, HSP90AA1, HSPA1B, HSPA8, ILK, ITGB1, ITGB3, JUP, LRP1, NCL, PKM, RAB10, RAP1B, RHOA,

3, ATP5F1B, CALM3, CANX, CCT2, CCT4, CCT6A, CCT7, CCT8, CDC42, CPN1, CYB5R3, CYBB, DCD, DDX60L,

ITGB3, MSN, MYADM, MYH9, MYL12B, RAB10, RAC2, RCC2, RHOA, RTN4, SOD1, VCL, YWHAH

YBB, EIF5A, FLNA, HLA-A, HLA-B, LRBA, M6PR, PNPLA8, PTGS1, RAB10, RAB1B, RAB27B, RAC2, RHOA, RT

TGB3, LTF, NPM1, PPIA, PRDX2, RHOA, RPS3A, SOD1, TFRC, THBS1, TMEM109, VDAC2, YWHAZ

, CD47, CD53, CD59, CDC42, CFL1, CLIC1, CYBB, EIF5A, ENO1, F11R, FCER1G, FCN1, FERMT3, FLNA, FLOT

7, CCT8, CD36, CD47, CSDE1, CTSD, DAZAP1, DHX9, EEF1A1, EEF2, EIF5A, ENO1, FLNA, FLOT1, G6PD, GA

F, MYADM, NCL, NPM1, PECAM1, PPIA, PTPRJ, RHOA, SOD1, TFRC, THBS1, TNKS1BP1, XRCC5, YWHAG

, FLNA, G6PD, GNAI2, GP1BA, GP1BB, ITGB1, ITGB3, LTF, PCYOX1, PNPLA8, RHOA, SLC2A1, SLC2A3, SRI,

P1, HRNR, ITGB3, KRT1, KRT16, LTF, PNPLA8, RTN4, S100A8, S100A9, SOD1, SRI, TFRC, YWHAE

TP5F1B, B2M, BCAP31, CALM3, CAMP, CANX, CASP14, CCL5, CCT2, CCT4, CCT6A, CCT7, CCT8, CD226, CD

AI2, GNAQ, GNG5, GP1BA, GP1BB, HLA-A, HLA-B, HSPA9, HSPD1, ITGA2B, ITGA6, ITGB1, ITGB3, JUP, LRP1

, ITGB3, MYH9, PFN1, RAB10, RAB27B, RAB33B, RHOA, RPL8, RPLP0, RPS23, RPS3A, RTN3, RTN4, SLC2A1

7, CCT8, CD36, CDC42, CSDE1, CTSD, DAZAP1, DHX9, EEF1A1, EEF2, EIF5A, ENO1, FLNA, FLOT1, G6PD, GA

6, CD47, CDC42, CSDE1, DAZAP1, DHX9, EEF1A1, EEF2, EIF5A, ENO1, FLNA, FLOT1, G6PD, GAPDH, GNAI2

, GAPDH, HINT1, HNRNPC, HNRNPU, HSP90AA1, HSPA1B, HSPA8, LRBA, LRP1, NPM1, PCYOX1, PNPLA8,
.NA, GP1BA, GP1BB, HLA-A, HLA-B, ICAM2, ITGA2B, ITGA6, ITGB1, ITGB3, LRP1, M6PR, PECAM1, PTPRJ, I

TGB1, ITGB3, JUP, KRT1, MATR3, MYH9, NCL, PFN1, PGK1, PKM, RHOA, RTN4, SPTBN1, THBS1, YWHAZ

.SP14, CAVIN2, CCT4, CCT8, CFL1, CLIC1, CYBB, DAZAP1, DHX9, DSP, EEF1A1, EEF2, EIF5A, ENO1, FLG2, FI

R1G, FLNA, GP1BA, GP1BB, HLA-A, HLA-B, ICAM2, ITGA2B, ITGA6, ITGB1, ITGB3, LRP1, M6PR, PECAM1, P

A1B, ILK, INPP5D, ITGB1, ITGB3, JUP, KRT10, KRT17, KRT2, LTF, MYADM, PKM, RHOA, RTN4, TFRC, THBS1

NPP5D, ITGB1, ITGB3, JUP, KRT1, KRT17, LTF, PGK1, PKM, PRDX2, RHOA, RTN4, SOD1, SRGN, THBS1, VCL

ANXA7, ARL6IP5, ARPC5, ATIC, ATP2A3, ATP5F1B, B2M, BCAP31, C16orf54, CALM3, CAMP, CANX, CASP1

, CD36, CD47, CD53, CD59, CDC42, CFL1, CLIC1, CYBB, DSP, EIF5A, ENO1, F11R, FCER1G, FCN1, FERMT3,

., FLNA, GALE, GAPDH, HINT1, HNRNPC, HNRNPU, HSP90AA1, HSPA1B, HSPA8, LDHA, LRP1, MSN, NPM1

, CD226, CD36, CD47, CD53, CD59, CDC42, CFL1, CLIC1, CYBB, DSP, EIF5A, ENO1, F11R, FCER1G, FCN1, F

PA1B, INPP5D, ITGA6, ITGB1, KRT1, LRP1, LTF, MMRN1, MYADM, NCL, PLEK, PPIA, PRDX2, PTPRJ, RHOA,

.PDH, GNAI2, HNRNPU, HSPA1B, MYH16, MYH9, NPM1, PATJ, RCC2, TCP1, TTLL12, TUBA1C, TUBA4A, TU

.K, ITGB3, LRP1, LTF, MYADM, NCL, NPM1, PECAM1, PKM, PPIA, PTPRJ, RHOA, SOD1, SPTBN1, TFRC, THE

NRNPU, HSP90AA1, ILK, ITGA6, ITGB1, ITGB3, KRT2, LRP1, LTF, MSN, MYADM, MYH9, PECAM1, RAB10, F

DHX9, EEF2, EIF5A, ENO1, F11R, FCER1G, FCN1, FLNA, FLOT1, G3BP1, GAPDH, GSTP1, HINT1, HLA-A, HL

B2M, BCAP31, CALM3, CAMP, CANX, CASP14, CCL5, CCT2, CCT4, CCT6A, CCT7, CCT8, CD36, CDC42, CPN

32M, BCAP31, CALM3, CAMP, CANX, CCL5, CCT2, CCT4, CCT6A, CCT7, CCT8, CD36, CD47, CDC42, CPN1, (

TP5F1B, B2M, BCAP31, CALM3, CAMP, CANX, CASP14, CCL5, CCT2, CCT4, CCT6A, CCT7, CCT8, CD226, CD

-A, HNRNPU, HSPA1B, ILK, INPP5D, ITGB1, ITGB3, JUP, KRT6A, LTF, NPM1, PTGS1, PTPRJ, RAC2, RHOA, R

HOA, RPL11, RPL8, RPLP0, RPS15, RPS15A, RPS23, RPS3A, RPSA, SYNCRIP, THBS1, TNKS1BP1, VIM

, CAMP, CANX, CASP14, CCL5, CCT2, CCT4, CCT6A, CCT7, CCT8, CD226, CD36, CD47, CDC42, CPN1, CSDE

BCAP31, CALM3, CAMP, CANX, CASP14, CCL5, CCT2, CCT4, CCT6A, CCT7, CCT8, CD36, CD47, CDC42, CPN

GB3, JUP, LRP1, NCL, PKM, RAB10, RAP1B, RHOA, RPLP0, SOD1, THBS1, UBE2L3, VIM, YWHAG, YWHAH

T4, CCT6A, CCT7, CCT8, CDC42, CPN1, CTSD, CYB5R3, CYBB, DCD, DDX60L, DHX9, EEF1A1, EEF2, ENDOD

6PD, GSR, GSTP1, HSP90AA1, HSPA1B, HSPA8, HSPA9, HSPD1, NPM1, PGK1, PNPLA8, PPIA, PRDX1, PRD

, G3BP1, GNAI2, GNAQ, GP1BA, GP1BB, GSTP1, HLA-A, HSPA1B, ILK, INPP5D, ITGA2B, ITGA6, ITGB1, ITGI

ENO1, FCN1, GAPDH, HINT1, HSPA1B, HSPD1, LRP1, LTF, MYH9, PCYOX1, PGK1, PSMA5, PSMA6, RHOA, I

GNAQ, HNRNPU, HSP90AA1, HSPA8, ITGB1, PTGS1, RAB27B, RHOA, RPLP0, RTN3, RTN4, SOD1, SPTBN1,

SP90AA1, HSPA1B, HSPA8, LRP1, MSN, NPM1, PCYOX1, PSMA5, PSMA6, RPL11, SYNCRIP, TNKS1BP1, UBI

HNRNPU, HSP90AA1, HSPA1B, HSPA8, HSPA9, HSPD1, ILK, MYH9, PGK1, PKM, PNPLA8, TCP1, TTLL12, U

HNRNPU, HSP90AA1, HSPA1B, HSPA8, HSPA9, HSPD1, ILK, MYH9, PFN1, PGK1, PKM, PNPLA8, TCP1, TTL

AZAP1, DHX9, EEF1A1, EEF2, EIF5A, ENO1, F11R, FCER1G, FCN1, FLNA, FLOT1, G3BP1, GAPDH, GP1BA, G

ISPA9, ILK, INPP5D, ITGB1, ITGB3, LTF, MYADM, PRDX2, PTBP1, RCC2, RHOA, RTN4, SOD1, VCL, XRCC5, Y

A1, EEF2, EIF5A, GAPDH, HNRNPA2B1, HNRNPC, HNRNPU, HSP90AA1, HSPA1B, HSPD1, KANSL3, KRT17,
6A, CCT7, CCT8, CD36, CD47, CPN1, CSDE1, CYB5R3, DAZAP1, DHX9, EEF1A1, EEF2, EIF5A, ENDOD1, EN

HMBS, HNRNPU, KRT17, NCL, NPM1, PKM, RHOA, RPL11, RPL8, RPLP0, RPS15, RPS15A, RPS23, RPS3A, F

, PPIA, RAB10, RAC2, RHOA, RTN3, RTN4, S100A8, S100A9, SOD1, VCL, VIM, XRCC5, YWHAE, YWHAG, YV

HNRNPU, HSP90AA1, HSPA1B, HSPA8, HSPA9, HSPD1, ILK, MYH9, PGK1, PKM, PNPLA8, TCP1, TTLL12, U

PDH, GNAI2, GNAQ, GSTP1, HINT1, HMBS, HSP90AA1, HSPA1B, ILK, ITGB3, KANSL3, KRT1, KRT10, KRT2,

6, CD47, CSDE1, CYB5R3, CYBB, DHX9, EEF1A1, EEF2, EIF5A, ENO1, FLNA, FLOT1, G6PD, GAPDH, GSTP1, I

'ADM, NCL, PLEK, PPIA, PRDX2, PTPRJ, RAP1B, RHOA, THBS1, TTLL12, UBQLN1, VDAC2, YWHAB

7, CSDE1, CYB5R3, DHX9, EEF1A1, EEF2, EIF5A, ENO1, FLNA, FLOT1, GAPDH, HINT1, HMBS, HNRNPA2B1,

BA, GP1BB, HSPD1, ITGB1, ITGB3, PCYOX1, SLC2A1, SLC2A3, SRI, STOM, THBS1, TMEM109, UBQLN1, VD

6, CD47, CSDE1, CYB5R3, CYBB, DHX9, EEF1A1, EEF2, EIF5A, ENO1, FLNA, FLOT1, GAPDH, GSTP1, HINT1,
NO1, HNRNPU, HSP90AA1, ILK, ITGA6, JUP, KANSL3, KRT17, NCL, NPM1, PCBP1, PKM, RHOA, SYNCRIP, T

2B1, HNRNPA3, HNRNPC, HNRNPF, HNRNPH1, HNRNPU, KANSL3, LDHA, LDHB, PSMA5, PSMA6, RAC2, S
'ADM, NCL, PLEK, PPIA, PRDX2, PTPRJ, RAP1B, RHOA, THBS1, TTLL12, UBQLN1, VDAC2, YWHAB
HNRNPA2B1, HNRNPC, HNRNPU, HSPA8, ITGB3, LRP1, MYADM, NCL, NPM1, PLEK, PPIA, PTBP1, PTPRJ, (

J, HSP90AA1, ILK, ITGA6, JUP, KANSL3, KRT17, NCL, NPM1, PCBP1, PKM, RHOA, SYNCRIP, TCP1, THBS1, U

NO1, HNRNPU, HSP90AA1, ILK, ITGA6, JUP, KANSL3, KRT17, NCL, NPM1, PCBP1, PKM, RHOA, SYNCRIP, T
6, CSDE1, CYB5R3, DHX9, EEF1A1, EEF2, EIF5A, ENO1, FLNA, FLOT1, G6PD, GAPDH, GSTP1, HINT1, HMBS

HNRNPA2B1, HNRNPC, HNRNPU, HSPA1B, HSPA8, ITGB3, LRP1, LTF, MYADM, NCL, NPM1, PPIA, PTBP1,

, HSP90AA1, ILK, ITGA6, ITGB1, LRP1, PFN1, PPIA, RAB10, RAC2, RHOA, RTN3, RTN4, S100A8, S100A9, SL

ILA-B, LRBA, LRP1, M6PR, PTGS1, QSOX1, RAB10, RAB1B, RAB27B, RAB33B, RTN3, SACM1L, SLC2A1, SRG

HINT1, HSPA1B, HSPA8, LDHA, LDHB, NANS, OSBPL2, PGK1, PKM, PLEK, PNPLA8, PTGS1, RHOA, SLC2A1,

PDH, GNAI2, GNAQ, GSTP1, HINT1, HMBS, HSP90AA1, HSPA1B, ILK, ITGB3, KANSL3, KRT1, KRT10, KRT2,

, LTF, NPM1, PF4V1, PFN1, RAB10, RAB1B, RAB27B, RAC2, RAP1B, RCC2, RHOA, RPL11, RPS15, SRI, UBE2

RNPA2B1, HNRNPU, HSP90AA1, HSPA8, ILK, ITGA6, JUP, KANSL3, NCL, NPM1, PCBP1, PRDX6, SYNCRIP, T

HSPA1B, INPP5D, LBP, LRP1, LTF, MYADM, NCL, NPM1, PTBP1, SRGN, SYNCRIP, THBS1, TNKS1BP1, VIM

AI2, GNAQ, GSTP1, HNRNPA2B1, HNRNPC, HNRNPU, HSPA1B, HSPA8, INPP5D, ITGB3, LBP, LRP1, LTF, MY
A8, ITGB1, MYH9, NASP, NPM1, RCC2, RHOA, RPS15A, SPTBN1, TTLL12, TUBA1C, TUBA4A, TUBB, TUBB1,

IF5A, ENO1, FLNA, FLOT1, GAPDH, GSTP1, HINT1, HNRNPA2B1, HNRNPC, HNRNPU, HSP90AA1, HSPA1B,

NO1, FLNA, FLOT1, GAPDH, GSTP1, HINT1, HNRNPA2B1, HNRNPC, HNRNPU, HSP90AA1, HSPA1B, HSPA8

EIF5A, ENO1, FLNA, FLOT1, GAPDH, GSTP1, HINT1, HNRNPA2B1, HNRNPC, HNRNPU, HSP90AA1, HSPA1

IF5A, ENO1, FLNA, FLOT1, GAPDH, HINT1, HNRNPA2B1, HNRNPC, HNRNPU, HSP90AA1, HSPA1B, HSPA8,

I2, GNAQ, GSTP1, HNRNPA2B1, HNRNPC, HNRNPU, HSPA1B, HSPA8, INPP5D, ITGB3, LBP, LRP1, LTF, MY

1, F11R, FLNA, G6PD, GNAI2, HSP90AA1, ITGB1, JUP, LRP1, MYL6, PTGS1, PTPRJ, RHOA, SLC2A1, SLC2A3

)1, FLNA, FLOT1, HINT1, HNRNPA2B1, HNRNPC, HNRNPF, HNRNPH1, HNRNPH3, HNRNPU, HSP90AA1, HS

NRNPA2B1, HNRNPA3, HNRNPC, HNRNPF, HNRNPH1, HNRNPH3, HNRNPU, HSP90AA1, HSPA1B, HSPA8, I

HX9, EIF5A, ENO1, FLNA, FLOT1, G6PD, HINT1, HMBS, HNRNPA2B1, HNRNPC, HNRNPU, HSP90AA1, HSP.

A, GNAI2, H2BC21, HINT1, HNRNPA2B1, HNRNPA3, HNRNPC, HNRNPF, HNRNPH1, HNRNPH3, HNRNPU,

ZAP1, DHX9, EIF5A, ENDOD1, ENO1, FLNA, FLOT1, G3BP1, G6PD, GAPDH, HINT1, HMBS, HNRNPA2B1, H

B5R3, DAZAP1, DHX9, EIF5A, ENDOD1, ENO1, FLNA, FLOT1, G3BP1, G6PD, GAPDH, HINT1, HMBS, HNRNI

ZAP1, DHX9, EIF5A, ENDOD1, ENO1, FLNA, FLOT1, G3BP1, G6PD, GAPDH, HINT1, HNRNPA2B1, HNRNPA2B1, HNRNPC, HNRNPF, HNRNPH1, HNRNPH3, HNRNPU, HSPA1B, HSPA8, ILK, ITGA6, JUP, KANSL3, LTF,

5A, ENO1, FLNA, FLOT1, HINT1, HMBS, HNRNPA2B1, HNRNPC, HNRNPU, HSP90AA1, HSPA1B, HSPA8, ILK

5A, ENO1, FLNA, FLOT1, HINT1, HMBS, HNRNPA2B1, HNRNPC, HNRNPU, HSP90AA1, HSPA1B, HSPA8, ILK

5A, ENO1, FLNA, FLOT1, HINT1, HNRNPA2B1, HNRNPC, HNRNPU, HSP90AA1, HSPA1B, HSPA8, ILK, ITGA6

ZAP1, DHX9, EIF5A, ENDOD1, ENO1, FLNA, FLOT1, G3BP1, G6PD, GAPDH, HINT1, HMBS, HNRNPA2B1, H

)151, CD226, CD36, CD47, CD53, CD59, CD9, CD99, CLIC1, CYBB, DCD, DSC1, DSG1, F11R, FCER1G, FLNA,

)151, CD226, CD36, CD47, CD53, CD9, CD99, CLIC1, CYBB, DCD, DSC1, DSG1, F11R, FCER1G, FLNA, GP1B

OD1, ENO1, FLNA, FLOT1, G3BP1, HINT1, HNRNPA2B1, HNRNPA3, HNRNPC, HNRNPF, HNRNPH1, HNRN

NX, CPN1, CYBB, DCD, DHX9, DSC1, DSG1, ENDOD1, ENO1, FCN1, FLG2, GNAI2, GNAQ, HRNR, ITGA2B, IT
NX, CPN1, CYBB, DCD, DHX9, DSC1, DSG1, ENDOD1, ENO1, FCN1, FLG2, GNAI2, GNAQ, HRNR, ITGA2B, IT

SPA1B, HSPA8, ILK, ITGA6, JUP, KANSL3, LTF, NCL, NPM1, PCBP1, PFN1, PPIA, PRDX2, PSMA6, RHOA, S10
SPA1B, HSPA8, ILK, ITGA6, JUP, KANSL3, LTF, NCL, NPM1, PCBP1, PFN1, PPIA, PRDX2, PSMA6, RHOA, S10

SPA1B, HSPA8, ILK, ITGA6, JUP, KANSL3, LTF, NCL, NPM1, PCBP1, PFN1, PPIA, PRDX2, PSMA6, RHOA, S10

2B1, HNRNPA3, HNRNPC, HNRNPF, HNRNPH1, HNRNPH3, HNRNPU, HSPA1B, HSPA8, ILK, ITGA6, JUP, KA

SPA1B, HSPA8, ILK, ITGA6, JUP, KANSL3, LTF, NCL, NPM1, PCBP1, PFN1, PPIA, PRDX2, PSMA6, RHOA, S10
SPA1B, HSPA8, ILK, ITGA6, JUP, KANSL3, LTF, NCL, NPM1, PCBP1, PFN1, PPIA, PRDX2, PSMA6, RHOA, S10

SPA1B, HSPA8, ILK, ITGA6, JUP, KANSL3, LTF, NCL, NPM1, PCBP1, PFN1, PPIA, PRDX2, PSMA6, RHOA, S10

C1, CTSD, DCD, DSC1, DSP, EEF1A1, EEF2, ENDOD1, ENO1, F11R, FERMT3, FLNA, FLOT1, FSCN1, G6PD, G
C1, CTSD, DCD, DSC1, DSP, EEF1A1, EEF2, ENDOD1, ENO1, F11R, FERMT3, FLNA, FLOT1, FSCN1, G6PD, G
C1, CTSD, DCD, DSC1, DSP, EEF1A1, EEF2, ENDOD1, ENO1, F11R, FERMT3, FLNA, FLOT1, FSCN1, G6PD, G
C1, CTSD, DCD, DSC1, DSP, EEF1A1, EEF2, ENDOD1, ENO1, F11R, FERMT3, FLNA, FLOT1, FSCN1, G6PD, G
D53, CD59, CD9, CDC42, CFL1, CLIC1, CLTA, CTSD, CYB5R3, CYBB, DCD, DSC1, DSG1, DSP, EEF1A1, EEF2, E
42, CFL1, CLIC1, CPN1, CTSD, DCD, DSC1, DSP, EEF1A1, EEF2, ENDOD1, ENO1, F11R, FCN1, FERMT3, FLN
CD9, CDC42, CFL1, CLIC1, CPN1, CTSD, CYB5R3, DCD, DSC1, DSP, EEF1A1, EEF2, ENDOD1, ENO1, F11R, FC
MT3, FLG2, GSTP1, HLA-B, HRNR, HSP90AA1, HSPA1B, HSPA8, HSPD1, ITGA2B, ITGB3, JUP, KRT1, LGALS3E
G, FCN1, FERMT3, FLG2, GSTP1, HLA-B, HRNR, HSP90AA1, HSPA1B, HSPA8, HSPD1, ITGA2B, ITGB3, JUP,
NPM1, PFN1, PPIA, RAB10, RAC2, RHOA, RPL8, RPLP0, RPS15, RPS3A, TLN1, TPM4, VCL, VIM, YWHAB, Y
FN1, PPIA, RAB10, RAC2, RHOA, RPL8, RPLP0, RPS15, RPS3A, TLN1, TPM4, VCL, VIM, YWHAB, YWHAE, Y
DSC1, DSG1, DSP, EEF1A1, EEF2, FCER1G, FCN1, FERMT3, FLG2, FLOT1, GSTP1, HLA-A, HLA-B, HRNR, HS
DSC1, DSG1, DSP, EEF1A1, EEF2, FCER1G, FCN1, FERMT3, FLG2, FLOT1, GSTP1, HLA-A, HLA-B, HRNR, HS
MYH9, PCBP1, PFN1, PKM, PPIA, PRDX1, PRDX6, PTPRJ, RAB10, RPSA, RTN4, SPTBN1, TAGLN2, THBS1, T
, CCT2, CCT4, CCT6A, CCT7, CCT8, CD151, CD36, CD47, CD53, CD59, CD9, CD99, CDC42, CFL1, CLIC1, CLT
DHX9, DSG1, EEF1A1, EEF2, EIF5A, ENDOD1, ENO1, FLNA, FSCN1, G3BP1, G6PD, GALE, GAPDH, GNAI2,

, ITGA6, ITGB1, ITGB3, JUP, LRP1, MSN, MYADM, MYH9, NPM1, PATJ, PECAM1, PFN1, PPIA, PTPRJ, RAB

5, CCT2, CCT4, CCT6A, CCT7, CCT8, CD151, CD226, CD36, CD47, CD53, CD59, CD9, CD99, CDC42, CFL1, C
CSDE1, DHX9, DSG1, DSP, F11R, FCER1G, FERMT3, FLG2, FLNA, FLOT1, G6PD, GAPDH, GNAI2, GNAQ, GP
NAQ, HSPA1B, HSPA8, HSPA9, ILK, ITGA2B, ITGA6, ITGB1, ITGB3, JUP, LRP1, MSN, MYADM, MYH9, NPM
D36, CD47, CD53, CD59, CD9, CD99, CDC42, CFL1, CLIC1, CLTA, CSDE1, CTSD, CYB5R3, CYBB, DCD, DHX9,

HINT1, HNRNPC, HNRNPU, HSPA1B, ILK, INPP5D, JUP, KRT1, KRT10, KRT14, KRT15, KRT16, KRT17, KRT2,
, HNRNPU, HSP90AA1, HSPA8, HSPD1, ICAM2, ITGA2B, ITGA6, ITGB1, ITGB3, JUP, KRT14, LRP1, M6PR, M
NRNPF, HNRNPH1, HNRNPH3, HNRNPU, HSP90AA1, HSPA1B, HSPA8, HSPA9, HSPD1, LRP1, MATR3, MSN
TA, CSDE1, CTSD, CYB5R3, CYBB, DSC1, DSG1, DSP, EEF1A1, EEF2, EIF5A, ENO1, FCER1G, FCN1, FERMT3

RPLP0, RPS15, RPS15A, RPS23, RPS3A, RPSA, SPTBN1, THBS1, TLN1, TPM4, TUBA1C, TUBA4A, TUBB, TUB

HLA-A, HLA-B, HSP90AA1, HSPA1B, HSPA8, HSPD1, ITGB1, ITGB3, KRT1, KRT16, KRT6A, LBP, LTF, LYZ, M

I2, ILK, ITGA2B, ITGA6, ITGB1, ITGB3, JUP, LGALS3BP, MMRN1, MSN, MYADM, MYH9, PECAM1, PLEK, PP
, CCT4, CCT6A, CCT7, CCT8, CD36, CD47, CD53, CD59, CD9, CDC42, CFL1, CLIC1, CLTA, CSDE1, CTSD, CYB5

9, CD99, CDC42, CFL1, CLIC1, CLTA, CSDE1, CTSD, CYBB, DSC1, DSG1, DSP, EEF1A1, EEF2, ENO1, F11R, FC
M, MYH9, PECAM1, PLEK, PPIA, PRDX2, RAB10, RAC2, RHOA, S100A8, S100A9, SELPLG, SPN, TFRC, TLN1,

9, MYH9, MYL12B, PSMA6, RAC2, RCC2, SLC2A1, SPTBN1, SRI, TCP1, TPM4, TUBA1C, TUBA4A, TUBB, TUI
, CCT4, CCT6A, CCT7, CCT8, CD36, CD47, CD53, CD59, CD9, CDC42, CFL1, CLIC1, CLTA, CSDE1, CTSD, CYB5

9, MYH9, MYL12B, PSMA6, RAC2, RCC2, SLC2A1, SPTBN1, SRI, TCP1, TPM4, TUBA1C, TUBA4A, TUBB, TUI
.4, CAVIN2, CCL5, CCT2, CCT4, CCT6A, CCT7, CCT8, CD151, CD226, CD36, CD47, CD53, CD59, CD9, CD99,
T5, KRT6A, KRT6B, KRT78, KRT9, MYH9, MYL12B, NCL, PCBP1, PSMA6, RAC2, RCC2, RPLP0, SLC2A1, SPTB

C, HNRNPU, HSP90AA1, HSPA1B, HSPA9, HSPD1, ITGB1, ITGB3, KRT1, KRT14, KRT15, KRT16, KRT17, KRT2
), GNAI2, GP1BA, GP1BB, HNRNPU, HSP90AA1, HSPA8, ITGB1, ITGB3, JUP, KRT5, LRP1, MSN, NPM1, PEC

, ILK, ITGB1, ITGB3, JUP, LDHB, LRBA, MSN, NCL, NPM1, PFN1, PLEK, PRDX6, PTPRJ, RAC2, RCC2, RHOA, I
CFL1, CLIC1, CLTA, CSDE1, CYBB, DSC1, DSG1, DSP, EEF1A1, EEF2, ENO1, F11R, FCER1G, FCN1, FLG2, FLNA
42, CFL1, CLIC1, CLTA, CTSD, CYBB, DHX9, DSP, F11R, FCER1G, FCN1, FLNA, FLOT1, G6PD, GNAI2, GP1BA
R, FCER1G, FCN1, FERMT3, FLNA, FLOT1, G3BP1, G6PD, GAPDH, GNAQ, GP1BA, GP1BB, GSDMA, GSR, GS
.1, HSPA1B, HSPA8, HSPA9, HSPD1, ITGA6, ITGB1, ITGB3, JUP, KRT1, KRT10, KRT14, KRT5, LRBA, MSN, M
P1BA, GP1BB, HNRNPA2B1, HNRNPU, HSP90AA1, HSPA8, HSPA9, HSPD1, ITGB1, ITGB3, JUP, KRT5, LRBA
., MSN, MYH9, PF4V1, PLEK, PPIA, PRDX1, PRDX2, PTPRJ, RAC2, RHOA, SELPLG, SOD1, SPN, TFRC, THBS1,

, GP1BA, GP1BB, H2BC21, HLA-A, HLA-B, HSP90AA1, HSPA1B, HSPA9, HSPD1, INPP5D, ITGA6, ITGB1, ITG

PA8, HSPA9, HSPD1, ITGB1, ITGB3, JUP, KRT5, LRBA, LRP1, M6PR, MSN, MYADM, MYH9, NASP, NPM1, P
PA8, HSPA9, HSPD1, ITGB1, ITGB3, JUP, KRT5, LRBA, LRP1, M6PR, MSN, MYADM, MYH9, NASP, NPM1, P
, CCT2, CCT4, CCT6A, CCT7, CCT8, CD151, CD36, CD47, CD53, CD59, CD9, CD99, CDC42, CFL1, CLIC1, CLT.

IRNPU, HSP90AA1, HSPA8, HSPA9, HSPD1, ITGB1, ITGB3, JUP, KRT5, LBP, LRBA, LRP1, M6PR, MSN, MYAI
1, ITGA2B, ITGA6, ITGB1, ITGB3, KRT10, LBP, LTF, MSN, PTPRJ, SPN, TFRC, THBS1, TLN1, TSPAN14
CLIC1, CSDE1, CTSD, CYBB, DAZAP1, DHX9, EEF2, EIF5A, ENO1, F11R, FCER1G, FCN1, FERMT3, FLNA, FLOT
1, CLIC1, CLTA, CTSD, CYBB, DHX9, F11R, FCER1G, FCN1, FLNA, FLOT1, G6PD, GNAI2, GP1BA, GP1BB, HNI

3, KRT17, LBP, MSN, MYH9, NCL, NPM1, PKM, PRDX6, PTPRJ, RHOA, RPL11, SOD1, SPN, SPTBN1, SYNCRI
DSP, EIF5A, ENO1, F11R, FERMT3, FLNA, FLOT1, FSCN1, G3BP1, G6PD, GAPDH, H2BC21, HNRNPA2B1, H
, GNAQ, HLA-A, HLA-B, HSPA8, HSPA9, ITGA2B, ITGB1, ITGB3, LRBA, LRP1, M6PR, PECAM1, PNPLA8, PTP
NRNPC, HNRNPU, HSP90AA1, HSPA1B, HSPA8, ITGA6, ITGB3, KRT17, LRP1, MSN, MYADM, MYH9, NPM1
.1, HSPA1B, HSPA8, HSPA9, HSPD1, ITGA6, ITGB1, ITGB3, JUP, KRT1, KRT10, KRT14, KRT5, LRBA, MSN, M
X9, DSP, EEF2, F11R, FCER1G, FERMT3, FLG2, FLNA, FLOT1, G6PD, GNG5, GP1BA, GP1BB, GSTP1, HLA-B,
DSP, EIF5A, ENO1, F11R, FERMT3, FLNA, FLOT1, FSCN1, G3BP1, G6PD, GAPDH, H2BC21, HNRNPA2B1, H

'9, LRP1, MSN, MYADM, MYH9, NPM1, PATJ, PFN1, PLEK, RAC2, RHOA, S100A8, S100A9, SOD1, SPTBN1,
, CYBB, DHX9, F11R, FCER1G, FCN1, FLNA, FLOT1, G6PD, GNAI2, GP1BA, GP1BB, HNRNPA2B1, HNRNPU,
AZAP1, DHX9, EEF2, EIF5A, ENO1, F11R, FCN1, FERMT3, FLNA, FLOT1, FSCN1, G3BP1, G6PD, GAPDH, GN

FERMT3, FLG2, FLNA, FLOT1, G6PD, GNG5, GP1BA, GP1BB, GSTP1, HLA-B, HNRNPH3, HNRNPU, HRNR, H

, CCT7, CCT8, CD36, CD47, CD53, CD59, CD9, CDC42, CFL1, CLIC1, CLTA, CSDE1, CTSD, CYB5R3, CYBB, DA

A1, HSPD1, KRT1, KRT16, KRT6A, LBP, LTF, LYZ, MATR3, PF4V1, PRDX1, PRDX2, PTPRJ, RHOA, RPS15A, S1

G6PD, GAPDH, GNAI2, GNAQ, GSTP1, HNRNPU, HSP90AA1, HSPA1B, HSPA8, HSPA9, HSPD1, ILK, ITGB1, I
O1, FERMT3, FLNA, FLOT1, FSCN1, G3BP1, G6PD, GAPDH, GNAI2, H2AC20, H2BC21, HINT1, HNRNPA2B1
O1, FERMT3, FLNA, FLOT1, FSCN1, G3BP1, G6PD, GAPDH, GNAI2, H2AC20, H2BC21, HINT1, HNRNPA2B1

R, PECAM1, PTPRJ, RAB10, RAB27B, RAC2, RAP1B, RCC2, RHOA, SLC2A3, SRI, STOM, TFRC, TSPAN14

2, GNAQ, GP1BA, GSDMA, GSTP1, H2BC21, HLA-A, HLA-B, HSP90AA1, HSPA8, ITGB3, JUP, KRT1, KRT16, I
R, PECAM1, PTPRJ, RAB10, RAB27B, RAC2, RAP1B, RCC2, RHOA, SLC2A3, SRI, STOM, TFRC, TSPAN14

U, HSP90AA1, HSPA1B, HSPA8, HSPA9, HSPD1, ILK, MYH9, OSBPL2, PCYOX1, PFN1, PGK1, PKM, PLEK, PN
NRNPU, HSPA1B, HSPA8, HSPA9, HSPD1, ILK, ITGB3, JUP, LBP, LRP1, LTF, MSN, NCL, NPM1, PF4V1, PGK1
A1, KRT1, KRT16, KRT6A, LBP, LTF, LYZ, MATR3, PF4V1, PRDX1, PRDX2, RHOA, RPS15A, S100A8, S100A9,
A1, KRT1, KRT16, KRT6A, LBP, LTF, LYZ, MATR3, PF4V1, PRDX1, PRDX2, RHOA, RPS15A, S100A8, S100A9,

N, MYH9, NPM1, PECAM1, PKM, PPIA, PTBP1, PTPRJ, RAB1B, RHOA, S100A8, S100A9, SYNCRIP, TFRC, TH
NRNPC, HNRNPH3, HNRNPU, HRNR, HSP90AA1, HSPA1B, HSPA9, ILK, INPP5D, ITGA6, ITGB1, ITGB3, KRT1

NRNPC, HNRNPH3, HNRNPU, HRNR, HSP90AA1, HSPA1B, HSPA9, ILK, INPP5D, ITGA6, ITGB1, ITGB3, KRT1
P1, MSN, MYADM, MYH9, PECAM1, PF4V1, PFN1, PPIA, PTPRJ, RAC2, RCC2, RHOA, RTN4, S100A8, S100A

ISPA1B, HSPA8, HSPA9, HSPD1, ILK, LDHB, MYH9, PCYOX1, PFN1, PGK1, PKM, PNPLA8, PSMA6, QSOX1, F

, HSPA1B, HSPD1, ILK, ITGB3, KANSL3, KRT17, LRP1, LTF, MSN, MYADM, MYH9, NCL, NPM1, PECAM1, PK

SDE1, CYB5R3, CYBB, DAZAP1, DHX9, DSG1, DSP, EEF2, ENO1, F11R, FCER1G, FCN1, FERMT3, FLG2, FLNA

90AA1, HSPA1B, HSPA8, HSPA9, HSPD1, ILK, LDHB, MYH9, OSBPL2, PCYOX1, PFN1, PGK1, PKM, PNPLA8,
M2, ITGA2B, ITGA6, ITGB1, ITGB3, LBP, LRP1, MSN, MYH9, PF4V1, PPIA, PTPRJ, S100A8, S100A9, SELPLG,
C42, CFL1, CLIC1, CPN1, CYBB, DCD, DDX60L, DHX9, DSG1, DSP, EEF1A1, EIF5A, ENDOD1, ENO1, F11R, FC

GB3, JUP, LDHA, LDHB, LYZ, MATR3, MYH9, NCL, NPM1, PECAM1, PLEK, PRDX1, PRDX6, SLC2A1, SOD1, S
TGB3, JUP, LRP1, LTF, NPM1, PFN1, PLEK, PPIA, PSMA6, RCC2, RHOA, RPL11, S100A8, S100A9, SOD1, TC

ISPA1B, HSPA8, HSPA9, HSPD1, ILK, LDHB, MYH9, PCYOX1, PFN1, PGK1, PKM, PNPLA8, QSOX1, RAB10, R

T1, G3BP1, GAPDH, HLA-A, HNRNPA2B1, HNRNPC, HNRNPU, HSP90AA1, HSPA1B, HSPA8, HSPD1, ILK, IT

N1, FLOT1, G3BP1, GAPDH, GNAI2, GSTP1, HLA-A, HNRNPA2B1, HNRNPC, HNRNPU, HSP90AA1, HSPA1B

RT6A, LBP, LGALS3BP, LRP1, LTF, LYZ, MATR3, PF4V1, PRDX1, PRDX2, PSMA6, PTGS1, S100A8, S100A9, S

, ENDOD1, ENO1, FLNA, FSCN1, G3BP1, G6PD, GAPDH, GNAI2, GNAQ, GSR, H2AC20, H2BC21, HINT1, HL
, ENDOD1, ENO1, FLNA, FSCN1, G3BP1, G6PD, GAPDH, GNAI2, GNAQ, GSR, H2AC20, H2BC21, HINT1, HL

4, CAVIN2, CCL5, CCT2, CCT4, CCT6A, CCT7, CCT8, CD151, CD226, CD36, CD47, CD53, CD59, CD9, CD99,
BP, LRP1, MSN, MYADM, MYH9, PECAM1, PF4V1, PFN1, PPIA, PTPRJ, RAC2, RCC2, RHOA, RTN4, S100A8,
AB10, RAB1B, RAB27B, RAB33B, RAC2, RCC2, RPL11, SPTBN1, SRI, STOM, TCP1, TFRC, UBE2L3, YWHAB,

.RP1, M6PR, MSN, MYH9, PECAM1, PLEK, PTPRJ, RAB10, RAB1B, RAB27B, RAB33B, RAC2, RAP1B, RTN3, S
O1, F11R, FCER1G, FCN1, FLNA, FLOT1, G3BP1, GAPDH, GNAI2, GNAQ, GNG5, GP1BA, GP1BB, GSTP1, H2

RP1, MSN, PPIA, PTPRJ, RAB10, RAB27B, RAB33B, RAC2, RAP1B, RHOA, S100A8, SOD1, SRI, STOM, THBS1

SPD1, ILK, INPP5D, ITGB1, ITGB3, JUP, KRT1, LBP, LTF, MATR3, PPIA, PRDX2, PTBP1, PTPRJ, RAC2, RAP1B
CN1, FLNA, FLOT1, G6PD, GAPDH, GNAI2, GNAQ, GP1BA, GP1BB, GSTP1, HINT1, HMBS, HNRNPU, HSP90

P, EEF1A1, EIF5A, ENO1, FCER1G, FLOT1, GAPDH, GNAQ, HLA-A, HLA-B, HSPA8, HSPA9, HSPD1, ITGA2B, I

NAQ, GP1BA, GSTP1, HINT1, HLA-A, HLA-B, HSP90AA1, HSPA1B, HSPD1, ILK, INPP5D, ITGA6, ITGB1, ITGB
D, ITGA6, ITGB1, ITGB3, LRP1, LTF, NPM1, PKM, PNPLA8, PPIA, PRDX2, RHOA, RPL11, RPS3A, RTN3, RTN
IAI2, GP1BA, GSTP1, HLA-A, HLA-B, HSP90AA1, HSPA1B, HSPA9, HSPD1, INPP5D, ITGB1, ITGB3, JUP, KRT1

CD36, CD47, CD53, CD59, CD9, CDC42, CFL1, CLIC1, CLTA, CSDE1, CTSD, CYB5R3, CYBB, DAZAP1, DHX9, D

;, FLNA, FLOT1, G6PD, GAPDH, GNAI2, GNAQ, GSR, GSTP1, HNRNPU, HSP90AA1, HSPA1B, HSPA8, HSPA9

KRT6A, LBP, LRP1, LTF, LYZ, MATR3, PF4V1, PRDX1, PTPRJ, RAC2, RTN4, S100A8, S100A9, SPN, SYNCRIP,
LTF, LYZ, NPM1, PECAM1, PNPLA8, PRDX1, PRDX2, PRDX6, RTN4, S100A8, S100A9, SLC2A1, SOD1, SRI, T
TR3, MSN, PRDX2, PTPRJ, RAC2, RHOA, RTN4, SOD1, SPN, TFRC, THBS1, TTLL12, UBQLN1, XRCC5
, PFN1, PLEK, RAP1B, RHOA, RPL11, RPLP0, RPS15, RPSA, RTN4, SF3A3, SLC2A1, SPTBN1, TCP1, TFRC, TLI

'M1, OSBPL2, PFN1, PLEK, RAP1B, RHOA, RPL11, RPLP0, RPS15, RPSA, RTN4, SF3A3, SLC2A1, SPTBN1, TC

HX9, EEF1A1, EIF5A, ENO1, F11R, FCER1G, FCN1, FERMT3, FLNA, FLOT1, G3BP1, G6PD, GAPDH, GNAI2, G

5A, KRT6B, KRT78, KRT9, LTF, MSN, MYADM, MYL6, PECAM1, PFN1, PGK1, RAB10, RAP1B, RHOA, RTN4,

1, ILK, LBP, LRP1, LTF, MYH9, PF4V1, PGK1, PKM, PNPLA8, PPIA, RAB10, RAB1B, RAB27B, RAB33B, RAC2,
PA9, ILK, INPP5D, ITGA6, ITGB1, ITGB3, JUP, KRT1, KRT10, KRT16, KRT17, KRT2, KRT5, KRT6A, KRT6B, KR

A6, ITGB1, LTF, NPM1, PKM, PPIA, PRDX2, RHOA, RPL11, RPS3A, RTN3, RTN4, S100A8, S100A9, SOD1, SR

PGK1, PKM, PNPLA8, PSMA6, RAB10, RAB1B, RAB27B, RAB33B, RAC2, RAP1B, RHOA, TCP1, TTLL12, TUB

:T2, CCT4, CCT6A, CCT7, CCT8, CD151, CD226, CD36, CD47, CD53, CD59, CD9, CD99, CDC42, CFL1, CLIC1,

MYH9, NPM1, OSBPL2, PLEK, RAB10, RAB1B, RAB27B, RAC2, RAP1B, RPL11, RPS15, SOD1, SRI, STOM, TU

33, JUP, LBP, LRP1, LTF, MSN, NCL, NPM1, PF4V1, PKM, PRDX2, PTPRJ, RAB10, RAP1B, RHOA, RPLP0, SEL

B1, LTF, NPM1, PPIA, PRDX2, RHOA, RPL11, RPS3A, RTN3, RTN4, S100A8, S100A9, SOD1, SRGN, TCP1, TF

RDX2, RHOA, RPL11, RPS3A, RTN4, S100A8, S100A9, SOD1, TCP1, TFRC, THBS1, TMEM109, UBQLN1, VD

PRDX2, PTBP1, PTPRJ, RAP1B, RHOA, RPL11, RPS15, RTN4, S100A8, S100A9, SOD1, TFRC, THBS1, UBQLN

PFN1, PGK1, PKM, PNPLA8, RAB10, RAB1B, RAB27B, RAB33B, RAC2, RAP1B, RHOA, TCP1, TTLL12, TUBA1

iB3, JUP, KRT1, KRT10, KRT16, KRT17, KRT6A, LRP1, LTF, MATR3, MSN, MYADM, MYH9, MYL12B, NCL, PI

A1, EEF2, EIF5A, ENO1, FCN1, FLNA, FLOT1, G6PD, GAPDH, GNAI2, GNAQ, GP1BA, GP1BB, GSR, GSTP1, H
NPM1, OSBPL2, PLEK, PNPLA8, PPIA, RAB10, RAB1B, RAB27B, RAB33B, RAC2, RPL11, S100A8, SLC2A1, S

P5D, ITGA6, ITGB3, LDHA, LDHB, LRP1, LTF, MYADM, NANS, NCL, NPM1, PECAM1, PGK1, PKM, PLEK, PNP

PGK1, PKM, PNPLA8, RAB10, RAB1B, RAB27B, RAB33B, RAC2, RAP1B, RHOA, TCP1, TTLL12, TUBA1C, TUE

J, HSP90AA1, HSPA1B, HSPD1, ITGA6, ITGB1, ITGB3, JUP, LRP1, LTF, NPM1, PFN1, PLEK, PPIA, PRDX2, PS

PPIA, RAB10, RAB1B, RAB27B, RAB33B, RAC2, RPL11, S100A8, SPTBN1, SRI, STOM, TCP1, TFRC, UBE2L3

ILK, ITGB1, ITGB3, MSN, MYADM, MYH9, PKM, PLEK, PTGS1, PTPRJ, RAB10, RAB27B, RAC2, RHOA, RPLP
PGK1, PKM, PNPLA8, RAB10, RAB1B, RAB27B, RAB33B, RAC2, RAP1B, RHOA, TCP1, TTLL12, TUBA1C, TUE

AC2, RPL11, RPS15, SOD1, STOM, TUBA1C, TUBB, UBE2L3, VDAC2, YWHAB, YWHAE, YWHAG, YWHAH, Y

P5D, ITGA6, ITGB3, LDHA, LDHB, LRP1, LTF, MYADM, NCL, NPM1, PECAM1, PGK1, PKM, PLEK, PNPLA8, P

NRNPU, HSP90AA1, HSPA1B, HSPA9, HSPD1, ILK, INPP5D, ITGA2B, ITGA6, ITGB1, ITGB3, JUP, KRT1, LRP1,

A1, HSPA1B, HSPA8, HSPD1, ILK, ITGA6, ITGB3, JUP, KANSL3, KRT17, LRP1, LTF, MSN, MYH9, NCL, NPM1,

GSTP1, HLA-A, HLA-B, HNRNPA2B1, HNRNPC, HNRNPU, HSPA1B, HSPA8, HSPA9, HSPD1, ILK, INPP5D, IT

AA1, HSPA1B, HSPA8, HSPA9, HSPD1, INPP5D, LTF, LYZ, PNPLA8, PRDX6, PSMA6, PTPRJ, RAB10, RAB1B, F

EIF5A, ENO1, F11R, FCER1G, FCN1, FLNA, FLOT1, G3BP1, G6PD, GAPDH, GNAI2, GNAQ, GSTP1, HINT1, H

.4, CAVIN2, CCL5, CCT2, CCT4, CCT6A, CCT7, CCT8, CD151, CD226, CD36, CD47, CD53, CD59, CD9, CD99,

53, CD59, CD9, CD99, CDC42, CFL1, CLIC1, CSDE1, CTSD, CYBB, DAZAP1, DHX9, DSP, EEF1A1, EEF2, EIF5A,

ITGB1, ITGB3, JUP, LBP, LRP1, LTF, MYADM, NCL, NPM1, PLEK, PPIA, PRDX1, PRDX2, PTBP1, PTPRJ, RAP

-B, HNRNPU, HSP90AA1, HSPA1B, HSPA9, HSPD1, ILK, INPP5D, ITGA2B, ITGA6, ITGB1, ITGB3, JUP, KRT1,

NAQ, GP1BA, GP1BB, GSTP1, HLA-A, HLA-B, HNRNPA2B1, HNRNPC, HNRNPU, HSPA1B, HSPA8, HSPA9, H

, KRT17, KRT2, LTF, MSN, MYADM, MYH9, MYL12B, PGK1, PKM, PRDX2, PTBP1, RAC2, RCC2, RHOA, RTN

LNA, GNAI2, GSR, GSTP1, H2BC21, HINT1, HNRNPA2B1, HNRNPA3, HNRNPC, HNRNPF, HNRNPH1, HNRNI
LNA, GNAI2, GSR, GSTP1, H2BC21, HINT1, HNRNPA2B1, HNRNPA3, HNRNPC, HNRNPF, HNRNPH1, HNRNI
LNA, GNAI2, GSR, GSTP1, H2BC21, HINT1, HNRNPA2B1, HNRNPA3, HNRNPC, HNRNPF, HNRNPH1, HNRNI

1B, ILK, INPP5D, ITGA6, ITGB1, ITGB3, JUP, LBP, LRP1, LTF, MYADM, NCL, NPM1, PLEK, PPIA, PRDX1, PRI

1, EEF2, EIF5A, ENO1, F11R, FCER1G, FCN1, FLNA, FLOT1, G3BP1, G6PD, GAPDH, GNAI2, GNAQ, GSTP1, I

N, MYADM, MYH9, PKM, PLEK, PTGS1, PTPRJ, RAB10, RAB27B, RAC2, RHOA, RPLP0, RTN3, RTN4, SELPLG

NPM1, PRDX1, PRDX2, PTGS1, PTPRJ, RAB10, RAC2, RAP1B, RHOA, RPS15A, RTN4, SPN, TFRC, THBS1, U
90AA1, HSPA1B, HSPA8, ILK, ITGA6, ITGB3, JUP, KANSL3, KRT17, LTF, NCL, NPM1, PCBP1, PECAM1, PKM

CD36, CD47, CD53, CD59, CD9, CD99, CDC42, CFL1, CLIC1, CSDE1, CTSD, CYBB, DAZAP1, DHX9, DSP, EEF1

HA, LRBA, LRP1, MSN, NPM1, PCYOX1, PGK1, PKM, PNPLA8, PRDX1, PRDX2, PRDX6, PSMA5, PSMA6, QS(

NRNPA2B1, HNRNPA3, HNRNPC, HNRNPF, HNRNPH1, HNRNPH3, HNRNPU, HSP90AA1, HSPA1B, HSPA8,

D226, CD36, CD47, CD53, CD59, CD9, CD99, CDC42, CFL1, CLIC1, CSDE1, CTSD, CYBB, DAZAP1, DHX9, DS

DM, NPM1, PATJ, PLEK, PPIA, PRDX1, PRDX2, PTBP1, PTPRJ, RAB33B, RAC2, RAP1B, RHOA, RPL11, RPS15

, CCL5, CCT2, CCT4, CCT6A, CCT7, CCT8, CD151, CD226, CD36, CD47, CD53, CD59, CD9, CD99, CDC42, CF

AM1, PGK1, PKM, PPIA, PTPRJ, RHOA, SOD1, SPTBN1, TFRC, THBS1, TNKS1BP1, XRCC5, YWHAG, YWHAZ

ITGA6, ITGB1, ITGB3, JUP, LBP, LRP1, LTF, MYADM, NCL, NPM1, PLEK, PPIA, PRDX1, PRDX2, PTBP1, PTPR

2, RHOA, RPLP0, RPS23, RPS3A, RTN3, RTN4, SACM1L, SRI, STOM, SYNCRIP, THBS1, TKT, TMEM109, TMX

A6, ITGB1, ITGB3, LRP1, LTF, NPM1, PLEK, PPIA, PTPRJ, RAC2, RCC2, RHOA, RPL11, RPS15, S100A8, S100A

DHX9, DSC1, DSG1, EEF1A1, EEF2, ENDOD1, ENO1, FCN1, FLG2, G3BP1, GNAI2, GNAQ, GSDMA, GSR, GS

1, G3BP1, GAPDH, GNAI2, GNAQ, GNG5, GP1BA, GP1BB, GSTP1, HINT1, HLA-A, HSP90AA1, HSPA1B, HSP

PDH, GNAI2, GNAQ, GSTP1, HINT1, HNRNPA2B1, HNRNPC, HNRNPF, HNRNPH1, HNRNPH3, HNRNPU, HSI

)36, CD47, CDC42, CPN1, CSDE1, CTSD, CYB5R3, CYBB, DAZAP1, DCD, DHX9, DSP, EEF1A1, EEF2, EIF5A, E

APDH, GNAI2, GNAQ, GSTP1, HINT1, HNRNPA2B1, HNRNPC, HNRNPF, HNRNPH1, HNRNPH3, HNRNPU, H

!, GNAQ, GSTP1, HINT1, HNRNPA2B1, HNRNPC, HNRNPF, HNRNPH1, HNRNPH3, HNRNPU, HSP90AA1, HS

PRDX1, PRDX2, PRDX6, PSMA5, PSMA6, QSOX1, RAB1B, RAB33B, RPL11, S100A8, S100A9, SYNCRIP, TNK

LNA, G3BP1, G6PD, GAPDH, GNAI2, GNAQ, GSTP1, H2AC20, H2BC21, HINT1, HNRNPA2B1, HNRNPA3, HN

4, CAVIN2, CCL5, CCT2, CCT4, CCT6A, CCT7, CCT8, CD151, CD226, CD36, CD47, CD53, CD59, CD9, CD99,

FLNA, FLOT1, G3BP1, GAPDH, GNAI2, GNAQ, GNG5, GP1BA, GP1BB, GSTP1, HINT1, HLA-A, HSP90AA1, H

, PCYOX1, PGK1, PKM, PNPLA8, PRDX6, PSMA5, PSMA6, RPL11, SYNCRIP, TNKS1BP1, UBE2L3, UBQLN1,

ERMT3, FLNA, FLOT1, G3BP1, GAPDH, GNAI2, GNAQ, GNG5, GP1BA, GP1BB, GSTP1, HINT1, HLA-A, HSP9

RAC2, RAP1B, RCC2, RHOA, RTN4, S100A8, S100A9, SOD1, VCL, VIM, XRCC5, YWHAH, YWHAZ

A-B, HNRNPA2B1, HNRNPC, HNRNPF, HNRNPH1, HNRNPH3, HNRNPU, HSP90AA1, HSPA1B, HSPA8, HSPI

1, CSDE1, CTSD, CYB5R3, DAZAP1, DCD, DHX9, DSP, EEF1A1, EEF2, EIF5A, ENDOD1, ENO1, FCN1, FLNA, I

CSDE1, CTSD, CYB5R3, CYBB, DAZAP1, DHX9, EEF1A1, EEF2, EIF5A, ENDOD1, ENO1, FLNA, FLOT1, G3BP1,

)36, CD47, CDC42, CPN1, CSDE1, CTSD, CYB5R3, CYBB, DAZAP1, DCD, DHX9, DSP, EEF1A1, EEF2, EIF5A, E

1, CTSD, CYBB, DAZAP1, DCD, DHX9, DSP, EEF1A1, EEF2, EIF5A, ENDOD1, ENO1, F11R, FCER1G, FCN1, FL

1, CSDE1, CTSD, CYB5R3, DAZAP1, DCD, DHX9, DSP, EEF1A1, EEF2, EIF5A, ENDOD1, ENO1, FCN1, FLNA, F

1, ENO1, G3BP1, G6PD, GALE, GAPDH, GNAI2, GNAQ, GNG5, GSR, GSTP1, HINT1, HMBS, HSP90AA1, HSP

B3, JUP, LBP, LRP1, LTF, MYH9, NCL, PECAM1, PF4V1, PLEK, PRDX2, PTPRJ, RCC2, RHOA, RTN4, SPN, SPTI

P1BB, GSTP1, HINT1, HLA-A, HLA-B, HNRNPA2B1, HNRNPA3, HNRNPC, HNRNPF, HNRNPH1, HNRNPH3, H

NASP, NCL, NPM1, PCYOX1, PKM, PSMA5, PSMA6, RAB1B, RHOA, RPL11, RPL8, RPLP0, RPS15, RPS15A,
D1, FLNA, FLOT1, G3BP1, G6PD, GAPDH, GSR, GSTP1, HINT1, HMBS, HNRNPA2B1, HNRNPA3, HNRNPC, H

LRP1, LTF, MYADM, NCL, NPM1, PECAM1, PKM, PPIA, PTBP1, PTPRJ, RHOA, RPL11, RPS15, S100A8, S10(

HINT1, HMBS, HNRNPA2B1, HNRNPC, HNRNPU, HSP90AA1, HSPA1B, HSPA8, ILK, INPP5D, ITGA6, JUP, KA

, HNRNPC, HNRNPU, HSP90AA1, HSPA1B, HSPA8, ILK, ITGA6, JUP, KANSL3, KRT17, LTF, NANS, NCL, NPM

HMBS, HNRNPA2B1, HNRNPC, HNRNPU, HSP90AA1, HSPA1B, HSPA8, ILK, INPP5D, ITGA6, JUP, KANSL3,

QSOX1, RHOA, RPL11, RTN3, RTN4, SYNCRIP, TNKS1BP1, XRCC5, YWHAB, YWHAE, YWHAG, YWHAZ

, HNRNPA2B1, HNRNPC, HNRNPU, HSP90AA1, HSPA1B, HSPA8, ILK, INPP5D, ITGA6, JUP, KANSL3, KRT17

PTPRJ, RPL11, RPS15, RTN3, RTN4, SYNCRIP, THBS1, TNKS1BP1, XRCC5, YWHAB, YWHAE, YWHAG, YWH

LRP1, LTF, MYADM, NCL, NPM1, PECAM1, PKM, PPIA, PTBP1, PTPRJ, RHOA, RPL11, RPS15, S100A8, S10(

ADM, NCL, NPM1, PLEK, PPIA, PTBP1, PTPRJ, QSOX1, RHOA, RPL11, RPS15, RTN3, RTN4, SOD1, SRGN, SY

HSPA8, ILK, ITGA6, JUP, KANSL3, KRT17, LTF, NCL, NPM1, PCBP1, PFN1, PKM, PLEK, PPIA, PRDX2, PSMA(

}, ILK, ITGA6, JUP, KANSL3, KRT17, LTF, NCL, NPM1, PCBP1, PFN1, PKM, PPIA, PRDX2, PSMA6, RHOA, S10

B, HSPA8, ILK, ITGA6, JUP, KANSL3, KRT17, LTF, NCL, NPM1, PCBP1, PFN1, PKM, PPIA, PRDX2, PSMA6, RI

ILK, ITGA6, JUP, KANSL3, KRT17, LTF, NCL, NPM1, PCBP1, PFN1, PKM, PLEK, PPIA, PRDX2, PSMA6, RHOA

ADM, NCL, NPM1, PPIA, PTBP1, PTPRJ, RPL11, RPS15, RTN3, RTN4, SRGN, SYNCRIP, THBS1, TNKS1BP1, V

;PA1B, HSPA8, ILK, ITGA6, JUP, KANSL3, LTF, NCL, NPM1, PCBP1, PFN1, PPIA, PRDX2, PRDX6, PSMA6, PTI

ILK, ITGB3, KANSL3, MYH9, NASP, NCL, NPM1, PCBP1, PSMA5, PSMA6, PTBP1, RPL11, RPS15, RPS15A, RI

A1B, HSPA8, ILK, ITGA6, JUP, KANSL3, LTF, NANS, NCL, NPM1, OSBPL2, PCBP1, PFN1, PPIA, PRDX2, PSMA

HSP90AA1, HSPA1B, HSPA8, HSPA9, ILK, ITGB3, KANSL3, MATR3, MYH9, NASP, NCL, NPM1, PCBP1, PSM

NRNPA3, HNRNPC, HNRNPF, HNRNPH1, HNRNPH3, HNRNPU, HSP90AA1, HSPA1B, HSPA8, HSPD1, ILK, IT

PA2B1, HNRNPA3, HNRNPC, HNRNPF, HNRNPH1, HNRNPH3, HNRNPU, HSP90AA1, HSPA1B, HSPA8, HSPI

3, HNRNPC, HNRNPF, HNRNPH1, HNRNPH3, HNRNPU, HSP90AA1, HSPA1B, HSPA8, HSPD1, ILK, ITGA6, JU
NCL, NPM1, PCBP1, PFN1, PPIA, PRDX2, PRDX6, PSMA6, PTBP1, RHOA, S100A8, S100A9, SRI, SYNCRIP, T

, ITGA6, JUP, KANSL3, LTF, NANS, NCL, NPM1, PCBP1, PFN1, PPIA, PRDX2, PSMA6, RHOA, S100A8, S100A

, ITGA6, JUP, KANSL3, LTF, NANS, NCL, NPM1, PCBP1, PFN1, PPIA, PRDX2, PSMA6, RHOA, S100A8, S100A

, JUP, KANSL3, LTF, NANS, NCL, NPM1, PCBP1, PFN1, PPIA, PRDX2, PSMA6, RHOA, S100A8, S100A9, SRI,

NRNPA3, HNRNPC, HNRNPF, HNRNPH1, HNRNPH3, HNRNPU, HSP90AA1, HSPA1B, HSPA8, HSPD1, ILK, IT

, GP1BA, GP1BB, GSDMA, HLA-A, HLA-B, HSPA9, HSPD1, ICAM2, ITGA2B, ITGA6, ITGB1, ITGB3, LRBA, LRF

A, GP1BB, GSDMA, HLA-A, HLA-B, HSPA9, HSPD1, ICAM2, ITGA2B, ITGA6, ITGB1, ITGB3, LRBA, LRP1, M6

PH3, HNRNPU, HSP90AA1, HSPA1B, HSPA8, HSPD1, ILK, ITGA6, JUP, KANSL3, LTF, NASP, NCL, NPM1, PCE

'GA6, ITGB1, LRP1, LTF, MATR3, MMRN1, MYL12B, MYL6, PKM, PTGS1, S100A8, S100A9, SF3A3, SOD1, S
'GA6, ITGB1, LRP1, LTF, MATR3, MMRN1, MYL12B, MYL6, PKM, PTGS1, S100A8, S100A9, SF3A3, SOD1, S

ANSL3, LTF, NCL, NPM1, PCBP1, PFN1, PPIA, PRDX2, PRDX6, PSMA6, PTBP1, RHOA, RPL11, RPS15, S100A8

APDH, GNAI2, GNAQ, GNG5, GP1BA, GSR, GSTP1, H2AC20, H2BC21, HINT1, HLA-A, HLA-B, HNRNPA2B1,
APDH, GNAI2, GNAQ, GNG5, GP1BA, GSR, GSTP1, H2AC20, H2BC21, HINT1, HLA-A, HLA-B, HNRNPA2B1,
APDH, GNAI2, GNAQ, GNG5, GP1BA, GSR, GSTP1, H2AC20, H2BC21, HINT1, HLA-A, HLA-B, HNRNPA2B1,
APDH, GNAI2, GNAQ, GNG5, GP1BA, GSR, GSTP1, H2AC20, H2BC21, HINT1, HLA-A, HLA-B, HNRNPA2B1,
NDOD1, ENO1, F11R, FCER1G, FCN1, FERMT3, FLG2, FLNA, FLOT1, FSCN1, G6PD, GAPDH, GNAI2, GNAQ,
A, FLOT1, FSCN1, G6PD, GAPDH, GNAI2, GNAQ, GNG5, GP1BA, GSR, GSTP1, H2AC20, H2BC21, HINT1, HL
CN1, FERMT3, FLG2, FLNA, FLOT1, FSCN1, G6PD, GAPDH, GNAI2, GNAQ, GNG5, GP1BA, GSR, GSTP1, H2A
3P, LTF, LYZ, MMRN1, MYH9, PECAM1, PKM, PPIA, PRDX6, PSMA5, PTPRJ, QSOX1, RAB10, RAB27B, RAP1
KRT1, LGALS3BP, LTF, LYZ, MMRN1, MYH9, PECAM1, PKM, PPIA, PRDX6, PSMA5, PTPRJ, QSOX1, RAB10,

P90AA1, HSPA1B, HSPA8, HSPD1, ITGA2B, ITGB1, ITGB3, JUP, KRT1, LGALS3BP, LRP1, LTF, LYZ, M6PR, MI
P90AA1, HSPA1B, HSPA8, HSPD1, ITGA2B, ITGB1, ITGB3, JUP, KRT1, LGALS3BP, LRP1, LTF, LYZ, M6PR, MI

A, CSDE1, CTSD, CYB5R3, CYBB, DAZAP1, DDX60L, DHX9, DSC1, DSG1, DSP, EEF1A1, EEF2, EIF5A, ENDOD
GSDMA, GSR, GSTP1, H2BC21, HINT1, HMBS, HNRNPC, HNRNPF, HNRNPH1, HNRNPU, HSP90AA1, HSPA1

10, RAC2, RAP1B, RHOA, RPL8, RPLP0, RPS15, RPS3A, RTN4, SLC2A1, TLN1, TNKS1BP1, TPM4, VCL, VIM,

LIC1, CLTA, CPN1, CSDE1, CTSD, CYB5R3, CYBB, DAZAP1, DCD, DHX9, DSC1, DSG1, DSP, EEF1A1, EEF2, EI
1BA, GP1BB, GSR, GSTP1, HNRNPC, HNRNPU, HRNR, HSP90AA1, HSPA1B, HSPA8, HSPA9, HSPD1, ILK, IN
I1, PATJ, PECAM1, PFN1, PPIA, PTPRJ, RAB10, RAB27B, RAB33B, RAC2, RAP1B, RHOA, RPL8, RPLP0, RPS1
, DSC1, DSG1, DSP, EEF1A1, EEF2, EIF5A, ENDOD1, ENO1, F11R, FCER1G, FCN1, FERMT3, FLG2, FLNA, FLO

KRT5, KRT6A, KRT6B, KRT78, KRT9, LRP1, MSN, MYADM, MYH16, MYH9, MYL12B, MYL6, NPM1, PATJ, P
MYH9, NPM1, PKM, PPIA, RAP1B, RPSA, SPTBN1, TFRC, THBS1, TLN1, TNKS1BP1, TPM4, TUBB, VIM, XRCC
I, MYH9, NCL, NPM1, PCBP1, PFN1, PKM, PPIA, PRDX1, PSMA6, PTBP1, RCC2, RPL11, RPL8, RPLP0, RPS1
, FLG2, FLNA, FLOT1, GAPDH, GNAQ, GSTP1, HLA-A, HLA-B, HRNR, HSP90AA1, HSPA1B, HSPA8, HSPD1, I

ATR3, PF4V1, PPIA, PRDX1, PRDX2, RHOA, RPS15A, RPSA, S100A8, S100A9, SELPLG, SPN, STOM, SYNCRI

IA, PRDX2, PTPRJ, RAB10, RAC2, RCC2, RHOA, RPSA, S100A8, S100A9, SELPLG, SPN, TFRC, THBS1, TLN1,
5R3, CYBB, DAZAP1, DCD, DHX9, DSC1, DSG1, DSP, EEF1A1, EEF2, EIF5A, ENDOD1, ENO1, F11R, FCER1G,

CER1G, FCN1, FLG2, FLNA, FLOT1, FSCN1, G6PD, GAPDH, GNAI2, GNAQ, GNG5, GP1BA, GP1BB, GSDMA,

5R3, CYBB, DAZAP1, DCD, DHX9, DSC1, DSG1, DSP, EEF1A1, EEF2, EIF5A, ENDOD1, ENO1, F11R, FCER1G,

CDC42, CFL1, CLIC1, CLTA, CPN1, CSDE1, CTSD, CYB5R3, CYBB, DAZAP1, DCD, DDX60L, DHX9, DSC1, DSG

, KRT5, KRT6A, KRT6B, KRT78, KRT9, LRBA, LRP1, MSN, MYADM, MYH9, NASP, NPM1, PATJ, PFN1, PLEK,
AM1, PPIA, PTPRJ, RAB10, RAB27B, RAB33B, RAC2, RAP1B, RHOA, RTN4, S100A8, SOD1, SPTBN1, SRI, ST

RPL11, RTN4, SLC2A1, SOD1, SPTBN1, SRI, STOM, TCP1, TFRC, TNKS1BP1, TSPAN14, TUBA4A, TUBB, UBE
A, FLOT1, FSCN1, G6PD, GAPDH, GNAI2, GNAQ, GNG5, GP1BA, GP1BB, GSDMA, GSR, HINT1, HLA-A, HLA-
, GP1BB, GSTP1, HNRNPA2B1, HNRNPU, HSP90AA1, HSPA1B, HSPA8, HSPA9, HSPD1, ITGB1, ITGB3, JUP,
STP1, H2BC21, HLA-A, HLA-B, HSP90AA1, HSPA1B, HSPA8, HSPA9, HSPD1, ILK, ITGB1, ITGB3, KRT1, KRT16
YADM, NASP, NPM1, OSBPL2, PATJ, PECAM1, PFN1, PLEK, PTPRJ, QSOX1, RAB1B, RAB33B, RAC2, RAP1B
, LRP1, M6PR, MSN, MYADM, MYH9, NASP, NPM1, OSBPL2, PECAM1, PLEK, PPIA, RAB10, RAB1B, RAB27

B3, KRT1, KRT16, KRT6A, LBP, LRP1, LTF, LYZ, MATR3, MSN, MYH9, PECAM1, PF4V1, PLEK, PPIA, PRDX1,

ECAM1, PLEK, PPIA, RAB10, RAB1B, RAB27B, RAB33B, RAC2, RCC2, RPL11, RTN4, SPTBN1, SRGN, SRI, ST
ECAM1, PLEK, PPIA, RAB10, RAB1B, RAB27B, RAB33B, RAC2, RCC2, RPL11, RTN4, SPTBN1, SRGN, SRI, ST
A, CSDE1, CTSD, CYB5R3, CYBB, DAZAP1, DDX60L, DHX9, DSC1, DSG1, DSP, EEF1A1, EEF2, EIF5A, ENDOD

DM, MYH9, NASP, NPM1, OSBPL2, PECAM1, PLEK, PNPLA8, PPIA, RAB10, RAB1B, RAB27B, RAB33B, RAC

T1, FSCN1, G3BP1, G6PD, GAPDH, GNAI2, GNG5, GP1BA, GP1BB, GSTP1, HINT1, HLA-A, HLA-B, HNRNPA2
RNPA2B1, HNRNPU, HSP90AA1, HSPA1B, HSPA8, HSPA9, HSPD1, ITGB1, ITGB3, JUP, LBP, LGALS3BP, LRP

NRNPC, HNRNPU, HRNR, HSP90AA1, HSPA1B, HSPA8, HSPA9, HSPD1, ILK, ITGA6, ITGB1, ITGB3, JUP, KAN
RJ, QSOX1, RAB10, RAB1B, RAB27B, RAB33B, RAC2, RAP1B, RCC2, RHOA, RTN3, SACM1L, SLC2A1, SLC2A
, PATJ, PFN1, PLEK, PTPRJ, RAB1B, RAB33B, RAC2, RAP1B, RCC2, RHOA, RTN4, S100A8, S100A9, SPTBN1,
YADM, NASP, NPM1, OSBPL2, PATJ, PECAM1, PFN1, PLEK, PTPRJ, QSOX1, RAB1B, RAB33B, RAC2, RAP1B
HNRNPC, HNRNPH3, HNRNPU, HRNR, HSP90AA1, HSPA1B, HSPA9, HSPD1, ILK, INPP5D, ITGA2B, ITGA6,
NRNPC, HNRNPU, HRNR, HSP90AA1, HSPA1B, HSPA8, HSPA9, HSPD1, ILK, ITGA6, ITGB1, ITGB3, JUP, KAN

HSP90AA1, HSPA1B, HSPA8, HSPA9, HSPD1, ITGB1, ITGB3, JUP, LBP, LGALS3BP, LRP1, LTF, M6PR, MSN,
AI2, GNG5, GP1BA, GP1BB, GSTP1, HINT1, HLA-A, HLA-B, HNRNPA2B1, HNRNPU, HSP90AA1, HSPA1B, H

HSP90AA1, HSPA1B, HSPA9, HSPD1, ILK, INPP5D, ITGA2B, ITGA6, ITGB1, ITGB3, JUP, KRT1, KRT10, KRT14

ZAP1, DHX9, DSC1, DSG1, DSP, EEF1A1, EEF2, EIF5A, ENO1, FCER1G, FCN1, FERMT3, FLG2, FLNA, FLOT1,

TGB3, JUP, LBP, LRP1, LTF, MSN, NASP, NCL, NPM1, PF4V1, PKM, PRDX2, PTPRJ, RAB10, RAP1B, RHOA, R
, HNRNPC, HNRNPU, HSPA1B, HSPA8, HSPA9, ILK, INPP5D, JUP, KRT1, KRT10, KRT14, KRT15, KRT16, KRT
, HNRNPC, HNRNPU, HSPA1B, HSPA8, HSPA9, ILK, INPP5D, JUP, KRT1, KRT10, KRT14, KRT15, KRT16, KRT

KRT5, KRT6A, LBP, LTF, LYZ, MATR3, MMRN1, PF4V1, PPIA, PRDX1, PRDX2, PSMA6, PTPRJ, RAC2, RHOA,

IPLA8, PSMA6, QSOX1, RAB10, RAB1B, RAB27B, RAB33B, RAC2, RAP1B, RHOA, S100A8, S100A9, TCP1, TI
., PKM, PNPLA8, PPIA, PRDX1, PRDX2, PRDX6, PTGS1, PTPRJ, RAB10, RAC2, RAP1B, RHOA, RPLP0, S100A

., KRT10, KRT14, KRT15, KRT16, KRT17, KRT2, KRT5, KRT6A, KRT6B, KRT78, KRT9, LRP1, LTF, MSN, MYAD

., KRT10, KRT14, KRT15, KRT16, KRT17, KRT2, KRT5, KRT6A, KRT6B, KRT78, KRT9, LRP1, LTF, MSN, MYAD

RAB10, RAB1B, RAB27B, RAB33B, RAC2, RAP1B, RHOA, TCP1, TTLL12, TUBA1C, TUBA4A, TUBB, TUBB1, U

KM, PPIA, PTBP1, PTPRJ, RAB1B, RHOA, RPL11, RPS15, RTN3, RTN4, S100A8, S100A9, SOD1, SYNCRIP, TFI

A, FLOT1, G3BP1, G6PD, GAPDH, GNAI2, GNAQ, GNG5, GP1BA, GP1BB, GSTP1, HLA-A, HLA-B, HNRNPC, H

QSOX1, RAB10, RAB1B, RAB27B, RAB33B, RAC2, RAP1B, RHOA, S100A8, S100A9, SLC2A3, TCP1, TKT, TTL

ER1G, FCN1, FERMT3, FLNA, FLOT1, G3BP1, G6PD, GAPDH, GNAI2, GNAQ, GNG5, GP1BA, GP1BB, GSDM

TOM, TFRC, THBS1, TKT, TMX2, TPM4, UBQLN1, VIM, YWHAB, YWHAE, YWHAG, YWHAH, YWHAZ

AB1B, RAB27B, RAB33B, RAC2, RAP1B, RHOA, TCP1, TTLL12, TUBA1C, TUBA4A, TUBB, TUBB1, UBE2L3, V

GA6, ITGB3, JUP, KANSL3, KRT17, LBP, LRP1, LTF, MSN, MYH9, NCL, NPM1, PCBP1, PECAM1, PKM, PPIA,

, HSPA8, HSPD1, ILK, ITGA6, ITGB3, JUP, KANSL3, KRT17, LBP, LRP1, LTF, MSN, MYH9, NCL, NPM1, PCBP1

A-A, HNRNPA2B1, HNRNPA3, HNRNPC, HNRNPF, HNRNPH1, HNRNPH3, HNRNPU, HSP90AA1, HSPA1B, H
A-A, HNRNPA2B1, HNRNPA3, HNRNPC, HNRNPF, HNRNPH1, HNRNPH3, HNRNPU, HSP90AA1, HSPA1B, H

CDC42, CFL1, CLIC1, CLTA, CPN1, CSDE1, CTSD, CYB5R3, CYBB, DAZAP1, DCD, DDX60L, DHX9, DSC1, DSG

2AC20, H2BC21, HINT1, HLA-A, HLA-B, HNRNPA2B1, HNRNPA3, HNRNPC, HNRNPF, HNRNPH1, HNRNPH3

, RHOA, RPL11, RPS15, RTN4, S100A8, S100A9, SOD1, TFRC, THBS1, TSPAN14, UBQLN1, XRCC5
0AA1, HSPA1B, HSPA8, HSPD1, ILK, ITGB3, KANSL3, KRT1, KRT10, KRT17, KRT2, LRP1, LTF, MSN, MYADM

ITGB1, ITGB3, LDHB, LRBA, LRP1, M6PR, MATR3, PECAM1, PNPLA8, PTGS1, PTPRJ, QSOX1, RAB10, RAB1

33, JUP, KRT1, LBP, LRP1, LTF, MATR3, MMRN1, MYADM, NCL, NPM1, PLEK, PNPLA8, PPIA, PRDX1, PRDX

1, KRT17, LBP, LRP1, LTF, PFN1, PGK1, PKM, PRDX2, PTPRJ, RHOA, RTN4, SOD1, SPN, SPTBN1, SRGN, SRI,

DSC1, DSG1, DSP, EEF1A1, EEF2, EIF5A, ENO1, FCER1G, FCN1, FERMT3, FLG2, FLNA, FLOT1, G3BP1, G6PD

, HSPD1, ILK, ITGB1, ITGB3, JUP, LBP, LRP1, LTF, MSN, NASP, NCL, NPM1, PF4V1, PGK1, PKM, PNPLA8, P

GNAQ, GNG5, GP1BA, GP1BB, GSR, GSTP1, HINT1, HLA-A, HNRNPU, HSP90AA1, HSPA1B, HSPA8, HSPA9,

T78, KRT9, LTF, MATR3, MSN, MYH9, MYL6, NASP, PECAM1, PLEK, PRDX2, PTPRJ, RAB33B, RHOA, RPLP

, CLTA, CPN1, CSDE1, CTSD, CYB5R3, CYBB, DAZAP1, DCD, DHX9, DSC1, DSG1, DSP, EEF1A1, EEF2, EIF5A,

FN1, PGK1, PKM, RAB10, RAB33B, RAC2, RCC2, RHOA, RTN4, SOD1, SPTBN1, TFRC, THBS1, VCL, YWHAH,

INT1, HMBS, HNRNPU, HSP90AA1, HSPA1B, HSPA8, HSPD1, ILK, ITGB3, KANSL3, KRT1, KRT10, KRT17, KF
LC2A3, SPTBN1, SRI, STOM, TCP1, TFRC, THBS1, UBE2L3, YWHAB, YWHAE, YWHAG, YWHAH, YWHAZ

LA8, PPIA, PRDX6, PTBP1, PTPRJ, RHOA, SACM1L, SOD1, SPTBN1, TFRC, THBS1, TKT, TNKS1BP1, XRCC5,

MA6, PTPRJ, RAC2, RCC2, RHOA, RPL11, RPS15, S100A8, S100A9, SOD1, SRI, STOM, TCP1, TFRC, THBS1,

PIA, PRDX6, PTBP1, PTPRJ, RHOA, SACM1L, SOD1, SPTBN1, TFRC, THBS1, TKT, TNKS1BP1, XRCC5, YWHAI

LTF, MATR3, MYH9, NASP, NCL, PECAM1, PFN1, PGK1, PKM, PLEK, PPIA, PRDX1, PRDX2, PTPRJ, RAB10,

, PCBP1, PECAM1, PKM, PPIA, PRDX6, PTBP1, PTPRJ, RAB1B, RHOA, S100A8, S100A9, SYNCRIP, TCP1, TFI

GA6, ITGB1, ITGB3, JUP, KRT16, LRP1, LTF, MYADM, MYH9, NCL, NPM1, PFN1, PLEK, PPIA, PRDX2, PTBP

HLA-A, HLA-B, HNRNPA2B1, HNRNPC, HNRNPF, HNRNPH1, HNRNPH3, HNRNPU, HSP90AA1, HSPA1B, HSP

CDC42, CFL1, CLIC1, CLTA, CPN1, CSDE1, CTSD, CYB5R3, CYBB, DAZAP1, DCD, DDX60L, DHX9, DSC1, DSG

, ENO1, F11R, FCER1G, FCN1, FERMT3, FLNA, FLOT1, FSCN1, G3BP1, G6PD, GAPDH, GNAI2, GNAQ, GNG5

1B, RHOA, RPL11, RPS15, RTN4, S100A8, S100A9, SOD1, SPTBN1, TFRC, THBS1, TSPAN14, TTLL12, UBQLN

KRT17, LRP1, LTF, MATR3, MYADM, MYH9, NASP, NCL, PECAM1, PFN1, PGK1, PKM, PLEK, PPIA, PRDX1,

ISPD1, ILK, INPP5D, ITGA6, ITGB1, ITGB3, JUP, KRT1, KRT16, KRT6A, LBP, LRP1, LTF, MMRN1, MYADM, M

PH3, HNRNPU, HRNR, HSP90AA1, HSPA1B, HSPA8, HSPA9, HSPD1, ILK, ITGB3, JUP, KANSL3, KRT1, LGALS
PH3, HNRNPU, HRNR, HSP90AA1, HSPA1B, HSPA8, HSPA9, HSPD1, ILK, ITGB3, JUP, KANSL3, KRT1, LGALS
PH3, HNRNPU, HRNR, HSP90AA1, HSPA1B, HSPA8, HSPA9, HSPD1, ILK, ITGB3, JUP, KANSL3, KRT1, LGALS

DX2, PTBP1, PTPRJ, RAP1B, RHOA, RPL11, RPS15, RTN4, S100A8, S100A9, SOD1, SPTBN1, SRI, TFRC, THBS

HINT1, HLA-A, HLA-B, HNRNPA2B1, HNRNPC, HNRNPF, HNRNPH1, HNRNPH3, HNRNPU, HSP90AA1, HSP/

, PLEK, PPIA, PRDX6, PTBP1, PTPRJ, RAB1B, RHOA, SOD1, SYNCRIP, TCP1, TFRC, THBS1, TNKS1BP1, UBE2

1A1, EEF2, EIF5A, ENO1, F11R, FCER1G, FCN1, FERMT3, FLNA, FLOT1, FSCN1, G3BP1, G6PD, GAPDH, GN/

OX1, RAB1B, RAB33B, RPL11, S100A8, S100A9, SYNCRIP, TNKS1BP1, UBE2L3, UBQLN1, VIM, YWHAH

HSPA9, HSPD1, LRP1, LTF, MATR3, MSN, MYH9, NCL, NPM1, PCBP1, PFN1, PKM, PPIA, PRDX1, PSMA6, F

G1, DSP, EEF1A1, EEF2, EIF5A, ENO1, F11R, FCER1G, FCN1, FERMT3, FLG2, FLNA, FLOT1, FSCN1, G3BP1,

5, RTN4, S100A8, S100A9, SOD1, TFRC, THBS1, TMEM109, UBQLN1, VDAC2, YWHAE, YWHAZ

L1, CLIC1, CLTA, CPN1, CSDE1, CTSD, CYB5R3, CYBB, DAZAP1, DCD, DDX60L, DHX9, DSC1, DSG1, DSP, EE

RJ, RAP1B, RHOA, RPL11, RPS15, RTN4, S100A8, S100A9, SOD1, SPTBN1, SRI, TFRC, THBS1, TSPAN14, TTL

TP1, HNRNPU, HRNR, HSP90AA1, HSPA1B, HSPA8, HSPA9, HSPD1, ILK, ITGA2B, ITGA6, ITGB1, LRP1, LTF,

D1, ILK, INPP5D, ITGA2B, ITGA6, ITGB1, ITGB3, JUP, LBP, LGALS3BP, LRP1, LTF, MYADM, MYH9, NCL, NPI

P90AA1, HSPA1B, HSPA8, HSPD1, ILK, ITGA6, ITGB3, JUP, KANSL3, KRT17, LRP1, LTF, MSN, MYADM, MYH

NDOD1, ENO1, F11R, FCER1G, FCN1, FLNA, FLOT1, G3BP1, G6PD, GALE, GAPDH, GNAI2, GNAQ, GP1BA,

SP90AA1, HSPA1B, HSPA8, HSPD1, ILK, ITGA6, ITGB3, JUP, KANSL3, KRT17, LRP1, LTF, MSN, MYADM, MY

;PA1B, HSPA8, ILK, ITGA6, ITGB3, JUP, KANSL3, KRT17, LRP1, LTF, MYADM, NCL, NPM1, PCBP1, PECAM1,

NRNPC, HNRNPF, HNRNPH1, HNRNPH3, HNRNPU, HRNR, HSP90AA1, HSPA1B, HSPA8, HSPA9, ILK, ITGB3,

CDC42, CFL1, CLIC1, CLTA, CPN1, CSDE1, CTSD, CYB5R3, CYBB, DAZAP1, DCD, DDX60L, DHX9, DSC1, DSG

SPA1B, HSPA8, HSPD1, ILK, INPP5D, ITGA2B, ITGA6, ITGB1, ITGB3, JUP, LBP, LGALS3BP, LRP1, LTF, MYAD

0AA1, HSPA1B, HSPD1, ILK, INPP5D, ITGA2B, ITGA6, ITGB1, ITGB3, JUP, LBP, LGALS3BP, LRP1, LTF, MYAD

)1, ILK, INPP5D, ITGA6, ITGB3, JUP, KANSL3, KRT17, LBP, LRP1, LTF, MATR3, MSN, MYADM, MYH9, NCL,

FLOT1, G3BP1, G6PD, GALE, GAPDH, GNAI2, GNAQ, GP1BA, GP1BB, GSTP1, HINT1, HMBS, HNRNPA2B1,

, G6PD, GAPDH, GNAI2, GNAQ, GSR, GSTP1, HINT1, HMBS, HNRNPA2B1, HNRNPA3, HNRNPC, HNRNPF, H

NDOD1, ENO1, F11R, FCER1G, FCN1, FLNA, FLOT1, G3BP1, G6PD, GALE, GAPDH, GNAI2, GNAQ, GP1BA,

NA, FLOT1, G3BP1, G6PD, GAPDH, GNAI2, GNAQ, GP1BA, GP1BB, GSTP1, HINT1, HLA-A, HLA-B, HMBS, H

FLOT1, G3BP1, G6PD, GAPDH, GNAI2, GNAQ, GP1BA, GP1BB, GSR, GSTP1, HINT1, HMBS, HNRNPA2B1, H

'A1B, HSPA8, HSPA9, HSPD1, ILK, INPP5D, ITGB3, LDHA, LDHB, LTF, LYZ, NANS, PCYOX1, PGK1, PKM, PNF

HNRNPU, HSP90AA1, HSPA1B, HSPA8, HSPD1, ILK, INPP5D, ITGA6, ITGB3, JUP, KANSL3, KRT17, LBP, LRP1

RPS23, RPS3A, RPSA, SYNCRIP, TCP1, TFRC, THBS1, TNKS1BP1, UBE2L3, UBQLN1, VIM, XRCC5
INRNPF, HNRNPH1, HNRNPH3, HNRNPU, HSP90AA1, HSPA1B, HSPA8, HSPD1, ILK, ITGA6, JUP, KANSL3, K

0A9, SOD1, SPTBN1, TFRC, THBS1, TNKS1BP1, TTLL12, UBE2L3, UBQLN1, XRCC5, YWHAB, YWHAE, YWHA

ANSL3, KRT17, LTF, NANS, NCL, NPM1, OSBPL2, PCBP1, PFN1, PGK1, PKM, PLEK, PNPLA8, PPIA, PRDX2, P

1, PCBP1, PFN1, PKM, PPIA, PRDX2, PSMA6, RHOA, RPL11, RPL8, RPLP0, RPS15, RPS15A, RPS23, RPS3A,

KRT17, LTF, NANS, NCL, NPM1, OSBPL2, PCBP1, PFN1, PKM, PLEK, PNPLA8, PPIA, PRDX2, PSMA6, PTGS1

, LTF, NANS, NCL, NPM1, OSBPL2, PCBP1, PFN1, PGK1, PKM, PLEK, PNPLA8, PPIA, PRDX2, PSMA6, PTGS1

0A9, SOD1, SPTBN1, TFRC, THBS1, TNKS1BP1, TTLL12, UBE2L3, UBQLN1, XRCC5, YWHAB, YWHAE, YWHA

5, RHOA, S100A8, S100A9, SOD1, SRI, SYNCRIP, TCP1, TFRC, THBS1, TNKS1BP1, UBE2L3, VIM, XRCC5, YW

)0A8, S100A9, SRI, SYNCRIP, TCP1, TFRC, THBS1, TNKS1BP1, UBE2L3, VIM, XRCC5, YWHAH, YWHAZ

HOA, RPL11, RPL8, RPLP0, RPS15, RPS15A, RPS23, RPS3A, RPSA, S100A8, S100A9, SRI, SYNCRIP, TCP1, TF

, S100A8, S100A9, SRI, SYNCRIP, TCP1, TFRC, THBS1, TNKS1BP1, UBE2L3, VIM, XRCC5, YWHAH, YWHAZ

BP1, RHOA, S100A8, S100A9, SRI, SYNCRIP, TCP1, TFRC, TNKS1BP1, UBE2L3, VIM, XRCC5, YWHAH, YWHA

PS23, RPS3A, RPSA, S100A9, SF3A3, SH3BGRL3, SOD1, SPN, SRI, SYNCRIP, TKT, UBE2L3, UBQLN1, WBP11

A6, RHOA, S100A8, S100A9, SOD1, SRI, TCP1, TFRC, TNKS1BP1, UBE2L3, XRCC5, YWHAH, YWHAZ

A5, PSMA6, PTBP1, RCC2, RPL11, RPS15, RPS15A, RPS23, RPS3A, RPSA, S100A9, SF3A3, SH3BGRL3, SOD

TGA6, JUP, KANSL3, LDHA, LDHB, LTF, NANS, NASP, NCL, NPM1, PCBP1, PFN1, PGK1, PKM, PPIA, PRDX2,

ɔ1, ILK, ITGA6, JUP, KANSL3, LDHA, LDHB, LTF, NANS, NASP, NCL, NPM1, OSBPL2, PCBP1, PFN1, PGK1, PI

JP, KANSL3, LDHA, LDHB, LTF, NANS, NASP, NCL, NPM1, PCBP1, PFN1, PGK1, PKM, PPIA, PRDX2, PRDX6,

TGA6, JUP, KANSL3, LDHA, LDHB, LTF, NANS, NASP, NCL, NPM1, PCBP1, PFN1, PGK1, PKM, PPIA, PRDX2,

ᵓ1, M6PR, MYADM, PECAM1, PNPLA8, PTPRJ, QSOX1, RAB27B, RAC2, RTN3, RTN4, SACM1L, SELPLG, SLC

PR, MYADM, PECAM1, PNPLA8, PTPRJ, QSOX1, RAC2, RTN3, RTN4, SACM1L, SELPLG, SLC2A1, SLC2A3, SI

3P1, PFN1, PPIA, PRDX2, PRDX6, PSMA6, PTBP1, RHOA, RPL11, RPS15, S100A8, S100A9, SF3A3, SRI, SYN(

8, S100A9, SF3A3, SRI, SYNCRIP, TFRC, TNKS1BP1, UBE2L3, VIM, WBP11, XRCC5, YWHAH, YWHAZ

HNRNPC, HRNR, HSP90AA1, HSPA1B, HSPA8, HSPA9, HSPD1, ITGA2B, ITGB1, ITGB3, JUP, KPRP, KRT1, KR
HNRNPC, HRNR, HSP90AA1, HSPA1B, HSPA8, HSPA9, HSPD1, ITGA2B, ITGB1, ITGB3, JUP, KPRP, KRT1, KR
HNRNPC, HRNR, HSP90AA1, HSPA1B, HSPA8, HSPA9, HSPD1, ITGA2B, ITGB1, ITGB3, JUP, KPRP, KRT1, KR
HNRNPC, HRNR, HSP90AA1, HSPA1B, HSPA8, HSPA9, HSPD1, ITGA2B, ITGB1, ITGB3, JUP, KPRP, KRT1, KR
, GNG5, GP1BA, GSR, GSTP1, H2AC20, H2BC21, HINT1, HLA-A, HLA-B, HNRNPA2B1, HNRNPC, HRNR, HSP
A-A, HLA-B, HNRNPA2B1, HNRNPC, HRNR, HSP90AA1, HSPA1B, HSPA8, HSPA9, HSPD1, ITGA2B, ITGB1, IT
C20, H2BC21, HINT1, HLA-A, HLA-B, HNRNPA2B1, HNRNPC, HRNR, HSP90AA1, HSPA1B, HSPA8, HSPA9, I
1B, RHOA, S100A8, S100A9, SLC2A3, SOD1, SRGN, SRI, STOM, TCP1, THBS1, TSPAN14, TUBB, VCL, VDAC2
RAB27B, RAP1B, RHOA, S100A8, S100A9, SLC2A3, SOD1, SRGN, SRI, STOM, TCP1, THBS1, TSPAN14, TUB

MRN1, MYH9, PECAM1, PKM, PPIA, PRDX1, PRDX6, PSMA5, PTPRJ, QSOX1, RAB10, RAB1B, RAB27B, RAB
MRN1, MYH9, PECAM1, PKM, PPIA, PRDX1, PRDX6, PSMA5, PTPRJ, QSOX1, RAB10, RAB1B, RAB27B, RAB

1, ENO1, FCER1G, FCN1, FERMT3, FLG2, FLNA, FLOT1, FSCN1, G3BP1, G6PD, GALE, GAPDH, GNAI2, GNA
1B, HSPA8, HSPD1, ILK, INPP5D, JUP, KRT1, KRT10, KRT14, KRT15, KRT16, KRT17, KRT2, KRT5, KRT6A, KR

F5A, ENDOD1, ENO1, F11R, FCER1G, FCN1, FERMT3, FLNA, FLOT1, FSCN1, G3BP1, G6PD, GALE, GAPDH,
PP5D, ITGB1, ITGB3, JUP, KRT1, KRT16, LTF, LYZ, MMRN1, MSN, MYADM, MYH9, NPM1, PECAM1, PF4V1
5, RPS23, RPS3A, RTN3, RTN4, S100A9, SLC2A1, SPTBN1, TLN1, TNKS1BP1, TPM4, VCL, VIM, YWHAB, YW
OT1, FSCN1, G6PD, GAPDH, GNAI2, GNAQ, GNG5, GP1BA, GP1BB, GSDMA, GSR, HINT1, HLA-A, HLA-B, HI

PFN1, RAB10, RAC2, RCC2, RHOA, S100A8, S100A9, SLC2A1, SPTBN1, STOM, TAGLN2, TCP1, TLN1, TNKS1

5, RPS15A, RPS23, RPS3A, RPSA, RTN4, SF3A3, SPTBN1, SYNCRIP, TCP1, TFRC, UBE2L3, VIM, WBP11, XRC
TGA2B, ITGB1, ITGB3, JUP, KRT1, LGALS3BP, LRBA, LRP1, LTF, LYZ, M6PR, MATR3, MMRN1, MYH9, PECA

FCN1, FERMT3, FLG2, FLNA, FLOT1, FSCN1, G3BP1, G6PD, GAPDH, GNAI2, GNAQ, GNG5, GP1BA, GSR, G

GSR, HINT1, HLA-A, HLA-B, HNRNPF, HRNR, HSP90AA1, HSPA1B, HSPA8, HSPD1, ICAM2, ILK, INPP5D, ITG

FCN1, FERMT3, FLG2, FLNA, FLOT1, FSCN1, G3BP1, G6PD, GAPDH, GNAI2, GNAQ, GNG5, GP1BA, GSR, G

1, DSP, EEF1A1, EEF2, EIF5A, ENDOD1, ENO1, F11R, FCER1G, FCN1, FERMT3, FLG2, FLNA, FLOT1, FSCN1,

PPIA, RAB10, RAB1B, RAB33B, RAC2, RHOA, RPL11, RPLP0, RPS15, RPS23, RPSA, RTN3, RTN4, S100A8, S

B, HNRNPF, HRNR, HSP90AA1, HSPA1B, HSPA8, HSPD1, ICAM2, ILK, INPP5D, ITGA2B, ITGA6, ITGB1, ITGB
KRT5, LBP, LGALS3BP, LRBA, LRP1, LTF, M6PR, MSN, MYADM, MYH9, NASP, NPM1, OSBPL2, PCYOX1, PI
5, KRT6A, LBP, LGALS3BP, LRP1, LTF, LYZ, MATR3, MMRN1, MYH9, NPM1, PF4V1, PGK1, PLEK, PNPLA8, P
, RCC2, RHOA, RPL11, RPLP0, RPS15, RPS23, RPSA, RTN3, RTN4, SF3A3, SLC2A1, SPTBN1, TCP1, TFRC, TH
7B, RAB33B, RAC2, RAP1B, RCC2, RPL11, RPS15, RTN4, S100A8, S100A9, SOD1, SPTBN1, SRGN, SRI, STOM

PRDX2, PTPRJ, RAB10, RAC2, RHOA, RTN4, S100A8, S100A9, SELPLG, SOD1, SPN, SYNCRIP, TFRC, THBS1,

OM, TCP1, TFRC, TSPAN14, UBE2L3, VCL, XRCC5, YWHAB, YWHAE, YWHAG, YWHAH, YWHAZ
OM, TCP1, TFRC, TSPAN14, UBE2L3, VCL, XRCC5, YWHAB, YWHAE, YWHAG, YWHAH, YWHAZ
1, ENO1, FCER1G, FCN1, FERMT3, FLG2, FLNA, FLOT1, FSCN1, G3BP1, G6PD, GALE, GAPDH, GNAI2, GNA

2, RCC2, RPL11, RTN4, SLC2A1, SPTBN1, SRGN, SRI, STOM, TCP1, TFRC, THBS1, TSPAN14, UBE2L3, VCL, X

2B1, HNRNPC, HNRNPU, HSP90AA1, HSPA1B, HSPA8, HSPD1, ILK, INPP5D, ITGA6, ITGB1, ITGB3, JUP, KAN
1, LTF, M6PR, MSN, MYH9, NASP, NPM1, OSBPL2, PCYOX1, PECAM1, PLEK, PNPLA8, PPIA, PTPRJ, RAB10

NSL3, KRT1, KRT10, KRT14, KRT15, KRT16, KRT17, KRT2, KRT5, KRT6A, KRT6B, KRT78, KRT9, LRBA, LRP1, I

, RCC2, RHOA, RPL11, RPLP0, RPS15, RPS23, RPSA, RTN3, RTN4, SF3A3, SLC2A1, SPTBN1, TCP1, TFRC, TH
ITGB1, ITGB3, JUP, KRT1, KRT10, KRT14, KRT15, KRT16, KRT17, KRT2, KRT5, KRT6A, KRT6B, KRT78, KRT9,
NSL3, KRT1, KRT10, KRT14, KRT15, KRT16, KRT17, KRT2, KRT5, KRT6A, KRT6B, KRT78, KRT9, LRBA, LRP1, I

MYH9, NASP, NPM1, OSBPL2, PCYOX1, PECAM1, PLEK, PNPLA8, PPIA, PTPRJ, RAB10, RAB1B, RAB27B, R
SPA8, HSPD1, ILK, INPP5D, ITGA6, ITGB1, ITGB3, JUP, KANSL3, KRT17, KRT6A, LBP, LRP1, LTF, MMRN1, M

, KRT15, KRT16, KRT17, KRT2, KRT5, KRT6A, KRT6B, KRT78, KRT9, LRP1, LTF, MATR3, MSN, MYADM, MY

FSCN1, G3BP1, G6PD, GAPDH, GNAI2, GNAQ, GSR, GSTP1, H2AC20, H2BC21, HINT1, HLA-A, HLA-B, HNR

RPLP0, S100A8, S100A9, SELPLG, SLC2A1, SOD1, SRI, SYNCRIP, TFRC, THBS1, TTLL12, UBE2L3, UBQLN1, V
17, KRT2, KRT5, KRT6A, KRT6B, KRT78, KRT9, LRP1, MSN, MYADM, MYH16, MYH9, MYL12B, MYL6, NAS
17, KRT2, KRT5, KRT6A, KRT6B, KRT78, KRT9, LRP1, MSN, MYADM, MYH16, MYH9, MYL12B, MYL6, NAS

RPS15A, S100A8, S100A9, SLC2A1, SOD1, SPN, SYNCRIP, TFRC, THBS1, TTLL12, TUBB, VIM, XRCC5

8, S100A9, SELPLG, SLC2A1, SOD1, SRI, SYNCRIP, TFRC, THBS1, TTLL12, UBE2L3, UBQLN1, VIM, XRCC5, Y

M, MYH9, PECAM1, PGK1, PLEK, PPIA, PRDX2, PTBP1, PTPRJ, RAB10, RAC2, RAP1B, RCC2, RHOA, RPS15,

M, MYH9, PECAM1, PGK1, PLEK, PPIA, PRDX2, PTBP1, PTPRJ, RAB10, RAC2, RAP1B, RCC2, RHOA, RPS15,

HNRNPU, HRNR, HSP90AA1, HSPA1B, HSPA9, HSPD1, ILK, INPP5D, ITGA2B, ITGA6, ITGB1, ITGB3, JUP, KRT

1A, GSR, GSTP1, H2BC21, HINT1, HLA-A, HLA-B, HNRNPU, HSP90AA1, HSPA1B, HSPA8, HSPA9, HSPD1, ILK

PRDX6, PTBP1, PTPRJ, RAB1B, RHOA, RPL11, S100A8, S100A9, SOD1, SPN, SPTBN1, SYNCRIP, TCP1, TFRC

1, PECAM1, PKM, PLEK, PPIA, PRDX6, PTBP1, PTPRJ, RAB1B, RHOA, RPL11, S100A8, S100A9, SOD1, SPN,

HSPA8, HSPA9, HSPD1, ILK, LDHB, LRP1, LTF, MATR3, MSN, MYH9, NCL, NPM1, OSBPL2, PCBP1, PCYOX1,
HSPA8, HSPA9, HSPD1, ILK, LDHB, LRP1, LTF, MATR3, MSN, MYH9, NCL, NPM1, PCBP1, PCYOX1, PFN1, PG

1, DSP, EEF1A1, EEF2, EIF5A, ENDOD1, ENO1, F11R, FCER1G, FCN1, FERMT3, FLG2, FLNA, FLOT1, FSCN1,

, HNRNPU, HSP90AA1, HSPA1B, HSPA8, HSPA9, HSPD1, ITGA2B, ITGA6, ITGB1, ITGB3, JUP, KANSL3, LDH

, MYH9, NCL, NPM1, PCYOX1, PECAM1, PGK1, PKM, PPIA, PSMA5, PSMA6, PTBP1, PTPRJ, RAB1B, RHOA,

B, RAB27B, RAB33B, RAC2, RAP1B, RCC2, RHOA, RTN3, RTN4, SACM1L, SLC2A1, SLC2A3, SRI, STOM, TFR

2, PSMA6, PTBP1, PTPRJ, RAC2, RAP1B, RHOA, RPL11, RPS15, RTN4, S100A8, S100A9, SOD1, SPN, SPTB

, GAPDH, GNAI2, GNAQ, GSR, GSTP1, H2AC20, H2BC21, HINT1, HLA-A, HLA-B, HNRNPA2B1, HNRNPA3,

PIA, PRDX1, PRDX2, PRDX6, PTGS1, PTPRJ, RAB10, RAC2, RAP1B, RHOA, RPLP0, S100A8, S100A9, SELPLG

HSPD1, ILK, INPP5D, ITGA2B, ITGA6, ITGB1, ITGB3, JUP, LBP, LGALS3BP, LRP1, LTF, MSN, MYADM, MYH9

), RTN3, RTN4, SELPLG, SLC2A1, SOD1, SPN, SRGN, SRI, TFRC, TMX2, VIM, XRCC5, YWHAE, YWHAH, YWH

ENDOD1, ENO1, F11R, FCER1G, FCN1, FERMT3, FLG2, FLNA, FLOT1, FSCN1, G3BP1, G6PD, GAPDH, GNA

RT2, LDHA, LDHB, LRP1, LTF, MSN, MYADM, MYH9, NCL, NPM1, PCYOX1, PECAM1, PGK1, PKM, PNPLA8,

RAB33B, RAC2, RHOA, RTN3, RTN4, S100A8, S100A9, SELPLG, SLC2A1, SOD1, SPN, SPTBN1, SRI, TFRC, TH

1, PTPRJ, QSOX1, RAB33B, RAP1B, RCC2, RHOA, RPL11, RPS3A, RTN3, RTN4, SOD1, SPN, SPTBN1, SRI, SY

PA8, HSPD1, ILK, INPP5D, ITGA6, ITGB3, JUP, KANSL3, KRT17, LBP, LRP1, LTF, MATR3, MSN, MYADM, MY

i1, DSP, EEF1A1, EEF2, EIF5A, ENDOD1, ENO1, F11R, FCER1G, FCN1, FERMT3, FLG2, FLNA, FLOT1, FSCN1,

5, GP1BA, GP1BB, GSR, GSTP1, HINT1, HLA-A, HLA-B, HNRNPA2B1, HNRNPC, HNRNPF, HNRNPH1, HNRNI

PRDX2, PTPRJ, RAB10, RAB33B, RAC2, RHOA, RTN3, RTN4, S100A8, S100A9, SELPLG, SLC2A1, SOD1, SPI

IYH9, NCL, NPM1, PFN1, PGK1, PLEK, PPIA, PRDX2, PTBP1, PTPRJ, QSOX1, RAB33B, RAP1B, RCC2, RHOA,

3BP, LTF, LYZ, MATR3, MMRN1, MYH9, NASP, NCL, NPM1, PCBP1, PKM, PPIA, PRDX6, PSMA5, PSMA6, F
3BP, LTF, LYZ, MATR3, MMRN1, MYH9, NASP, NCL, NPM1, PCBP1, PKM, PPIA, PRDX6, PSMA5, PSMA6, F
3BP, LTF, LYZ, MATR3, MMRN1, MYH9, NASP, NCL, NPM1, PCBP1, PKM, PPIA, PRDX6, PSMA5, PSMA6, F

A1B, HSPA8, HSPD1, ILK, INPP5D, ITGA6, ITGB1, ITGB3, JUP, KANSL3, KRT17, LBP, LRP1, LTF, MATR3, MSI

AI2, GNAQ, GNG5, GP1BA, GP1BB, GSR, GSTP1, HINT1, HLA-A, HLA-B, HNRNPA2B1, HNRNPC, HNRNPF, H

PTBP1, RCC2, RPL11, RPL8, RPLP0, RPS15, RPS15A, RPS23, RPS3A, RPSA, RTN4, SF3A3, SPTBN1, SYNCRIP,

G6PD, GAPDH, GNAI2, GNAQ, GNG5, GP1BA, GP1BB, GSR, GSTP1, HINT1, HLA-A, HLA-B, HNRNPA2B1, H

F1A1, EEF2, EIF5A, ENDOD1, ENO1, F11R, FCER1G, FCN1, FERMT3, FLG2, FLNA, FLOT1, FSCN1, G3BP1, G

MATR3, MMRN1, MYH9, MYL12B, MYL6, OSBPL2, PCYOX1, PFN1, PGK1, PKM, PLEK, PNPLA8, PSMA6, P

M1, PATJ, PECAM1, PF4V1, PLEK, PPIA, PRDX1, PRDX2, PTBP1, PTPRJ, RAB33B, RAC2, RAP1B, RCC2, RHO

H9, NCL, NPM1, PCBP1, PECAM1, PFN1, PKM, PPIA, PRDX2, PRDX6, PSMA6, PTBP1, PTPRJ, RAB1B, RHOA

GP1BB, GSR, GSTP1, HINT1, HLA-A, HLA-B, HMBS, HNRNPA2B1, HNRNPA3, HNRNPC, HNRNPF, HNRNPH1

YH9, NCL, NPM1, PCBP1, PECAM1, PFN1, PKM, PLEK, PPIA, PRDX2, PRDX6, PSMA6, PTBP1, PTPRJ, RAB1B

, PFN1, PKM, PLEK, PPIA, PRDX2, PRDX6, PSMA6, PTBP1, PTPRJ, QSOX1, RAB1B, RAC2, RHOA, RPL11, RT

JUP, KANSL3, KRT1, KRT10, KRT14, KRT15, KRT16, KRT2, KRT5, KRT6A, KRT9, LDHA, LRP1, LTF, MATR3, M

;1, DSP, EEF1A1, EEF2, EIF5A, ENDOD1, ENO1, F11R, FCER1G, FCN1, FERMT3, FLG2, FLNA, FLOT1, FSCN1,

OM, MYH9, NCL, NPM1, PATJ, PECAM1, PF4V1, PLEK, PPIA, PRDX1, PRDX2, PTBP1, PTPRJ, RAB33B, RAC2,

OM, MYH9, NCL, NPM1, PATJ, PECAM1, PF4V1, PLEK, PPIA, PRDX1, PRDX2, PTBP1, PTPRJ, RAB33B, RAC2

NPM1, PCBP1, PFN1, PKM, PPIA, PRDX2, PRDX6, PSMA6, PTBP1, PTPRJ, RHOA, RPL11, S100A8, S100A9,

HNRNPA3, HNRNPC, HNRNPF, HNRNPH1, HNRNPH3, HNRNPU, HSP90AA1, HSPA1B, HSPA8, HSPD1, ILK,

HNRNPH1, HNRNPH3, HNRNPU, HSP90AA1, HSPA1B, HSPA8, HSPA9, HSPD1, ILK, INPP5D, ITGA6, ITGB3,

GP1BB, GSR, GSTP1, HINT1, HLA-A, HLA-B, HMBS, HNRNPA2B1, HNRNPA3, HNRNPC, HNRNPF, HNRNPH1

HNRNPA2B1, HNRNPA3, HNRNPC, HNRNPF, HNRNPH1, HNRNPH3, HNRNPU, HSP90AA1, HSPA1B, HSPA8,

HNRNPA3, HNRNPC, HNRNPF, HNRNPH1, HNRNPH3, HNRNPU, HSP90AA1, HSPA1B, HSPA8, HSPD1, ILK, I

PLA8, PPIA, PRDX1, PRDX2, PRDX6, PSMA6, PTGS1, PTPRJ, QSOX1, RAB10, RAB1B, RAB27B, RAB33B, RAC

., LTF, MATR3, MSN, MYADM, MYH9, NCL, NPM1, PCBP1, PFN1, PGK1, PKM, PPIA, PRDX2, PRDX6, PSMA

KRT17, LDHA, LDHB, LTF, NANS, NASP, NCL, NPM1, PCBP1, PFN1, PGK1, PKM, PPIA, PRDX2, PRDX6, PSMA

SMA6, PTGS1, RHOA, RPL11, RPL8, RPLP0, RPS15, RPS15A, RPS23, RPS3A, RPSA, S100A8, S100A9, SACM

RPSA, S100A8, S100A9, SRI, SYNCRIP, TCP1, TFRC, THBS1, TNKS1BP1, UBE2L3, VIM, XRCC5, YWHAH, YW

, RHOA, RPL11, RPL8, RPLP0, RPS15, RPS15A, RPS23, RPS3A, RPSA, S100A8, S100A9, SACM1L, SOD1, SRI

L, RHOA, RPL11, RPL8, RPLP0, RPS15, RPS15A, RPS23, RPS3A, RPSA, S100A8, S100A9, SACM1L, SOD1, SR

PRDX6, PSMA6, PTBP1, RHOA, RPL11, RPS15, S100A8, S100A9, SF3A3, SLC2A1, SLC2A3, SRI, SYNCRIP, TC

‹M, PPIA, PRDX2, PRDX6, PSMA6, PTBP1, RHOA, RPL11, RPS15, S100A8, S100A9, SF3A3, SLC2A1, SLC2A3

PSMA6, PTBP1, RHOA, RPL11, RPS15, S100A8, S100A9, SF3A3, SRI, SYNCRIP, TCP1, TFRC, TKT, TNKS1BP

PRDX6, PSMA6, PTBP1, RHOA, RPL11, RPS15, S100A8, S100A9, SF3A3, SRI, SYNCRIP, TCP1, TFRC, TKT, TI

RT10, KRT14, KRT15, KRT16, KRT2, KRT5, KRT6A, KRT6B, KRT78, KRT9, LBP, LDHA, LDHB, LGALS3BP, LTF,
RT10, KRT14, KRT15, KRT16, KRT2, KRT5, KRT6A, KRT6B, KRT78, KRT9, LBP, LDHA, LDHB, LGALS3BP, LTF,
RT10, KRT14, KRT15, KRT16, KRT2, KRT5, KRT6A, KRT6B, KRT78, KRT9, LBP, LDHA, LDHB, LGALS3BP, LTF,
RT10, KRT14, KRT15, KRT16, KRT2, KRT5, KRT6A, KRT6B, KRT78, KRT9, LBP, LDHA, LDHB, LGALS3BP, LTF,
90AA1, HSPA1B, HSPA8, HSPA9, HSPD1, ITGA2B, ITGB1, ITGB3, JUP, KPRP, KRT1, KRT10, KRT14, KRT15, I
TGB3, JUP, KPRP, KRT1, KRT10, KRT14, KRT15, KRT16, KRT2, KRT5, KRT6A, KRT6B, KRT78, KRT9, LBP, LDH
HSPD1, ITGA2B, ITGB1, ITGB3, JUP, KPRP, KRT1, KRT10, KRT14, KRT15, KRT16, KRT2, KRT5, KRT6A, KRT6I

33B, RAC2, RAP1B, RCC2, RHOA, S100A8, S100A9, SLC2A1, SLC2A3, SOD1, SRGN, SRI, STOM, TCP1, TFR(
33B, RAC2, RAP1B, RCC2, RHOA, S100A8, S100A9, SLC2A1, SLC2A3, SOD1, SRGN, SRI, STOM, TCP1, TFR(

Q, GSDMA, GSR, GSTP1, H2BC21, HINT1, HLA-A, HLA-B, HMBS, HNRNPA2B1, HNRNPC, HNRNPF, HNRNPI
T6B, KRT78, KRT9, LDHA, LDHB, LRBA, MSN, MYH9, MYL12B, MYL6, NANS, NPM1, OSBPL2, PATJ, PCBP1,

GNAI2, GNAQ, GNG5, GP1BA, GP1BB, GSDMA, GSTP1, H2AC20, H2BC21, HINT1, HLA-A, HLA-B, HMBS, H
L, PFN1, PLEK, PNPLA8, PPIA, PRDX1, PRDX2, PRDX6, PTGS1, PTPRJ, RAP1B, RHOA, RPL11, RPLP0, RTN4,

NRNPA2B1, HNRNPC, HNRNPF, HNRNPH1, HNRNPU, HRNR, HSP90AA1, HSPA1B, HSPA8, HSPA9, HSPD1,

M1, PKM, PNPLA8, PPIA, PRDX6, PSMA5, PTGS1, PTPRJ, QSOX1, RAB10, RAB1B, RAB27B, RAB33B, RAC2

STP1, H2AC20, H2BC21, HINT1, HLA-A, HLA-B, HNRNPA2B1, HNRNPA3, HNRNPC, HNRNPF, HNRNPH1, H

iA2B, ITGA6, ITGB1, ITGB3, JUP, KRT1, KRT10, KRT16, KRT17, KRT2, LGALS3BP, LRBA, LRP1, LTF, M6PR, N

STP1, H2AC20, H2BC21, HINT1, HLA-A, HLA-B, HNRNPA2B1, HNRNPA3, HNRNPC, HNRNPF, HNRNPH1, H

, G3BP1, G6PD, GALE, GAPDH, GNAI2, GNAQ, GNG5, GP1BA, GP1BB, GSDMA, GSR, GSTP1, H2AC20, H2B

;100A9, SOD1, SPTBN1, SRGN, TCP1, TFRC, TLN1, TNKS1BP1, TPM4, TUBA1C, TUBA4A, TUBB, TUBB1, UB

33, JUP, KRT1, KRT10, KRT16, KRT17, KRT2, LRBA, LRP1, LTF, M6PR, MSN, MYADM, MYH9, NCL, OSBPL2,
ECAM1, PLEK, PNPLA8, PPIA, PTPRJ, RAB10, RAB1B, RAB27B, RAB33B, RAC2, RAP1B, RCC2, RHOA, RPL11
PIA, PRDX1, PRDX2, PRDX6, PSMA6, PTGS1, RHOA, S100A8, S100A9, SLC2A1, SOD1, SPN, SYNCRIP, TFR(

И, TCP1, TFRC, TSPAN14, TUBA1C, TUBB, UBE2L3, VCL, VDAC2, XRCC5, YWHAB, YWHAE, YWHAG, YWHA

Q, GSDMA, GSR, GSTP1, H2AC20, H2BC21, HINT1, HLA-A, HLA-B, HMBS, HNRNPA2B1, HNRNPA3, HNRNI

ISL3, KRT1, KRT10, KRT17, KRT2, KRT6A, LBP, LRP1, LTF, MATR3, MMRN1, MSN, MYADM, MYH9, NCL, N
, RAB1B, RAB27B, RAB33B, RAC2, RAP1B, RCC2, RHOA, RPL11, RPLP0, RPS15, RTN3, S100A8, SLC2A1, SL(

MSN, MYADM, MYH9, NASP, NPM1, OSBPL2, PATJ, PECAM1, PFN1, PLEK, PPIA, PTPRJ, QSOX1, RAB10, R.

, LRP1, LTF, MATR3, MSN, MYADM, MYH9, MYL12B, MYL6, NASP, NCL, PECAM1, PFN1, PGK1, PKM, PLEI
MSN, MYADM, MYH9, NASP, NPM1, OSBPL2, PATJ, PECAM1, PFN1, PLEK, PPIA, PTPRJ, QSOX1, RAB10, R.

AB33B, RAC2, RAP1B, RHOA, RPL11, RPLP0, RPS15, RTN3, S100A8, SLC2A1, SLC2A3, SOD1, SPTBN1, SRI,
MSN, MYADM, NCL, NPM1, PCBP1, PECAM1, PFN1, PKM, PLEK, PPIA, PRDX2, PRDX6, PTBP1, PTPRJ, RAB1

H9, MYL12B, MYL6, NASP, NCL, PECAM1, PFN1, PGK1, PKM, PLEK, PPIA, PRDX1, PRDX2, PTBP1, PTPRJ, R

NPA2B1, HNRNPA3, HNRNPC, HNRNPF, HNRNPH1, HNRNPH3, HNRNPU, HRNR, HSP90AA1, HSPA1B, HSI

, NCL, NPM1, OSBPL2, PATJ, PCBP1, PFN1, PSMA6, PTBP1, RAB10, RAC2, RAP1B, RCC2, RHOA, RPL11, R
, NCL, NPM1, OSBPL2, PATJ, PCBP1, PFN1, PSMA6, PTBP1, RAB10, RAC2, RAP1B, RCC2, RHOA, RPL11, R

RPS3A, RTN3, RTN4, S100A8, S100A9, SOD1, SPN, SYNCRIP, TAGLN2, TFRC, TPM4, TUBB, VCL, VIM, XRC

, RPS3A, RTN3, RTN4, S100A8, S100A9, SOD1, SPN, SYNCRIP, TAGLN2, TFRC, TPM4, TUBB, VCL, VIM, XRC

T1, KRT14, KRT16, KRT17, KRT2, KRT5, KRT6A, KRT6B, KRT78, KRT9, LBP, LRP1, LTF, LYZ, MATR3, MMRN1

K, INPP5D, ITGA2B, ITGA6, ITGB1, ITGB3, JUP, KRT1, KRT16, KRT5, KRT6A, LBP, LGALS3BP, LRP1, LTF, LYZ,

PFN1, PGK1, PKM, PNPLA8, PPIA, PRDX1, PSMA6, PTBP1, PTGS1, QSOX1, RAB10, RAB1B, RAB27B, RAB3

GK1, PKM, PNPLA8, PPIA, PRDX1, PSMA6, PTBP1, PTGS1, QSOX1, RAB10, RAB1B, RAB27B, RAB33B, RAC2

, G3BP1, G6PD, GALE, GAPDH, GNAI2, GNAQ, GNG5, GP1BA, GP1BB, GSDMA, GSR, GSTP1, H2AC20, H2B

A, LDHB, LRP1, LTF, MMRN1, MYH16, MYH9, MYL12B, MYL6, NASP, NCL, NPM1, PCBP1, PCYOX1, PECAI

, RPL11, RPL8, RPLP0, RPS15, RPS15A, RPS23, RPS3A, RPSA, RTN3, RTN4, S100A8, S100A9, SOD1, SPTBN:

HNRNPC, HNRNPF, HNRNPH1, HNRNPH3, HNRNPU, HRNR, HSP90AA1, HSPA1B, HSPA8, HSPA9, HSPD1, II

;, SLC2A1, SOD1, SPN, SRI, SYNCRIP, TFRC, THBS1, TTLL12, UBE2L3, UBQLN1, VIM, XRCC5, YWHAG, YWH/

), NCL, NPM1, PATJ, PECAM1, PF4V1, PGK1, PKM, PLEK, PNPLA8, PPIA, PRDX1, PRDX2, PRDX6, PTBP1, PT

I2, GNAQ, GNG5, GP1BA, GP1BB, GSDMA, GSR, GSTP1, H2BC21, HINT1, HLA-A, HLA-B, HMBS, HNRNPA2I

PPIA, PSMA5, PSMA6, PTBP1, PTPRJ, RAB1B, RHOA, RPL11, RPL8, RPLP0, RPS15, RPS15A, RPS23, RPS3A,

NCRIP, TFRC, THBS1, TMEM109, TNKS1BP1, TTLL12, UBQLN1, VCL, VDAC2, VIM, XRCC5, YWHAB, YWHAI

H9, NCL, NPM1, PCBP1, PECAM1, PFN1, PKM, PPIA, PRDX2, PRDX6, PSMA6, PTBP1, PTPRJ, RAB1B, RHO

G3BP1, G6PD, GALE, GAPDH, GNAI2, GNAQ, GNG5, GP1BA, GP1BB, GSDMA, GSR, GSTP1, H2AC20, H2B

H3, HNRNPU, HSP90AA1, HSPA1B, HSPA8, HSPA9, HSPD1, ILK, INPP5D, ITGA2B, ITGA6, ITGB1, ITGB3, JU

, SPTBN1, SRGN, SRI, TFRC, THBS1, TMX2, VCL, VIM, XRCC5, YWHAE, YWHAG, YWHAH, YWHAZ

RPL11, RPS15, RPS3A, RTN3, RTN4, SOD1, SPN, SPTBN1, SRGN, SRI, SYNCRIP, TFRC, THBS1, TMEM109, T

TBP1, QSOX1, RAB33B, RCC2, RPL11, RPS15, RPS15A, RPS23, RPS3A, RPSA, S100A8, S100A9, SF3A3, SH
TBP1, QSOX1, RAB33B, RCC2, RPL11, RPS15, RPS15A, RPS23, RPS3A, RPSA, S100A8, S100A9, SF3A3, SH
TBP1, QSOX1, RAB33B, RCC2, RPL11, RPS15, RPS15A, RPS23, RPS3A, RPSA, S100A8, S100A9, SF3A3, SH

ı, MYADM, MYH9, NCL, NPM1, PCBP1, PECAM1, PFN1, PKM, PLEK, PPIA, PRDX2, PRDX6, PSMA6, PTBP1

NRNPH1, HNRNPH3, HNRNPU, HSP90AA1, HSPA1B, HSPA8, HSPA9, HSPD1, ILK, INPP5D, ITGA2B, ITGA6,

INRNPC, HNRNPF, HNRNPH1, HNRNPH3, HNRNPU, HRNR, HSP90AA1, HSPA1B, HSPA8, HSPA9, HSPD1, IL

6PD, GALE, GAPDH, GNAI2, GNAQ, GNG5, GP1BA, GP1BB, GSDMA, GSR, GSTP1, H2AC20, H2BC21, HINT1

TGS1, QSOX1, RAB10, RAB1B, RAB27B, RAB33B, RAC2, RAP1B, RHOA, S100A8, S100A9, SF3A3, SOD1, SR

)A, RPL11, RPS15, RTN4, S100A8, S100A9, SOD1, SPN, SPTBN1, SRGN, SRI, TFRC, THBS1, TLN1, TMEM10

, RPL11, RPS15, RTN3, RTN4, S100A8, S100A9, SOD1, SRI, SYNCRIP, TCP1, TFRC, THBS1, TNKS1BP1, UBE

l, HNRNPH3, HNRNPU, HSP90AA1, HSPA1B, HSPA8, HSPA9, HSPD1, ILK, INPP5D, ITGA6, ITGB1, ITGB3, JU

3, RHOA, RPL11, RPS15, RTN3, RTN4, S100A8, S100A9, SOD1, SRI, SYNCRIP, TCP1, TFRC, THBS1, TNKS1BP

N3, RTN4, S100A8, S100A9, SOD1, SRI, SYNCRIP, TCP1, TFRC, THBS1, TNKS1BP1, UBE2L3, UBQLN1, VIM,

MSN, MYH9, NASP, NCL, NPM1, PCBP1, PFN1, PKM, PPIA, PRDX1, PRDX6, PSMA5, PSMA6, PTBP1, RAC2,

, G3BP1, G6PD, GALE, GAPDH, GNAI2, GNAQ, GNG5, GP1BA, GP1BB, GSDMA, GSR, GSTP1, H2AC20, H2B

, RAP1B, RCC2, RHOA, RPL11, RPS15, RTN4, S100A8, S100A9, SLC2A1, SOD1, SPN, SPTBN1, SRGN, SRI, TF

, RAP1B, RCC2, RHOA, RPL11, RPS15, RTN4, S100A8, S100A9, SOD1, SPN, SPTBN1, SRGN, SRI, TFRC, THB

SOD1, SPN, SPTBN1, SRGN, SRI, SYNCRIP, TFRC, THBS1, TNKS1BP1, UBE2L3, VIM, XRCC5, YWHAH, YWHA

INPP5D, ITGA6, ITGB3, JUP, KANSL3, KRT1, KRT10, KRT17, KRT2, LDHA, LDHB, LRP1, LTF, MSN, MYADM,

JUP, KANSL3, KRT17, LDHA, LDHB, LRBA, LRP1, LTF, MYADM, NANS, NASP, NCL, NPM1, OSBPL2, PCBP1,

L, HNRNPH3, HNRNPU, HSP90AA1, HSPA1B, HSPA8, HSPD1, ILK, INPP5D, ITGA6, ITGB3, JUP, KANSL3, KR

, HSPD1, ILK, INPP5D, ITGA6, ITGB3, JUP, KANSL3, KRT1, KRT10, KRT17, KRT2, LBP, LRP1, LTF, MATR3, MS

TGA6, ITGB3, JUP, KANSL3, KRT1, KRT10, KRT17, KRT2, LDHA, LDHB, LRP1, LTF, MSN, MYADM, MYH9, NA

\6, PTBP1, PTPRJ, RHOA, RPL11, RPL8, RPLP0, RPS15, RPS15A, RPS23, RPS3A, RPSA, S100A8, S100A9, SF3

A6, PTBP1, RHOA, RPL11, RPL8, RPLP0, RPS15, RPS15A, RPS23, RPS3A, RPSA, RTN3, RTN4, S100A8, S100/

1L, SOD1, SRI, SYNCRIP, TCP1, TFRC, THBS1, TKT, TNKS1BP1, UBE2L3, VIM, XRCC5, YWHAH, YWHAZ

3, SOD1, SRI, SYNCRIP, TCP1, TFRC, TKT, TNKS1BP1, UBE2L3, VIM, WBP11, XRCC5, YWHAH, YWHAZ

LYZ, MSN, MYH9, MYL12B, MYL6, NANS, NCL, PATJ, PCBP1, PCYOX1, PECAM1, PFN1, PGK1, PKM, PPIA, F
LYZ, MSN, MYH9, MYL12B, MYL6, NANS, NCL, PATJ, PCBP1, PCYOX1, PECAM1, PFN1, PGK1, PKM, PPIA, F
LYZ, MSN, MYH9, MYL12B, MYL6, NANS, NCL, PATJ, PCBP1, PCYOX1, PECAM1, PFN1, PGK1, PKM, PPIA, F
LYZ, MSN, MYH9, MYL12B, MYL6, NANS, NCL, PATJ, PCBP1, PCYOX1, PECAM1, PFN1, PGK1, PKM, PPIA, F
KRT16, KRT2, KRT5, KRT6A, KRT6B, KRT78, KRT9, LBP, LDHA, LDHB, LGALS3BP, LRP1, LTF, LYZ, M6PR, MN
HA, LDHB, LGALS3BP, LTF, LYZ, MSN, MYH9, MYL12B, MYL6, NANS, NCL, PATJ, PCBP1, PCYOX1, PECAM1,
B, KRT78, KRT9, LBP, LDHA, LDHB, LGALS3BP, LTF, LYZ, MMRN1, MSN, MYH9, MYL12B, MYL6, NANS, NCL

H1, HNRNPU, HRNR, HSP90AA1, HSPA1B, HSPA8, HSPA9, HSPD1, ILK, INPP5D, ITGA2B, ITGB1, ITGB3, JUI
PFN1, PGK1, PKM, PLEK, PPIA, PRDX1, PRDX2, PRDX6, PSMA5, PSMA6, RAB10, RAB1B, RAC2, RAP1B, R

NRNPA2B1, HNRNPA3, HNRNPC, HNRNPF, HNRNPH1, HNRNPH3, HNRNPU, HSP90AA1, HSPA1B, HSPA8,
S100A8, S100A9, SLC2A1, SOD1, SPTBN1, SRI, SYNCRIP, TCP1, TFRC, THBS1, TLN1, TNKS1BP1, TUBB, VCL

ICAM2, ILK, INPP5D, ITGA2B, ITGA6, ITGB1, ITGB3, JUP, KRT1, KRT10, KRT16, KRT17, KRT2, KRT5, KRT6A

, RAP1B, RCC2, RHOA, RPLP0, RPS23, RPS3A, RTN3, RTN4, S100A8, S100A9, SACM1L, SLC2A1, SLC2A3, S

NRNPH3, HNRNPU, HRNR, HSP90AA1, HSPA1B, HSPA8, HSPA9, HSPD1, ILK, ITGA2B, ITGB1, ITGB3, JUP, K

MRN1, MSN, MYADM, MYH9, MYL12B, NCL, OSBPL2, PATJ, PCYOX1, PECAM1, PFN1, PKM, PLEK, PTPRJ,

NRNPH3, HNRNPU, HRNR, HSP90AA1, HSPA1B, HSPA8, HSPA9, HSPD1, ILK, INPP5D, ITGA2B, ITGB1, ITGB

C21, HINT1, HLA-A, HLA-B, HMBS, HNRNPA2B1, HNRNPA3, HNRNPC, HNRNPF, HNRNPH1, HNRNPH3, HN

PATJ, PCYOX1, PECAM1, PLEK, PTPRJ, RAB10, RAB27B, RAC2, RAP1B, RCC2, RHOA, RPSA, RTN3, RTN4, S
., RPLP0, RPS15, RTN3, RTN4, S100A8, S100A9, SLC2A1, SLC2A3, SOD1, SPTBN1, SRGN, SRI, STOM, TCP1,

PC, HNRNPF, HNRNPH1, HNRNPH3, HNRNPU, HRNR, HSP90AA1, HSPA1B, HSPA8, HSPA9, HSPD1, ILK, INF

PM1, PCBP1, PECAM1, PFN1, PKM, PLEK, PPIA, PRDX2, PRDX6, PTBP1, PTPRJ, RAB1B, RAB27B, RAC2, RA
C2A3, SOD1, SPTBN1, SRI, STOM, TCP1, TFRC, THBS1, TMEM109, TUBA1C, TUBB, UBE2L3, UBQLN1, VDA

AB1B, RAB33B, RAC2, RAP1B, RCC2, RHOA, RPL11, RPLP0, RPS15, RPS23, RPSA, RTN3, RTN4, S100A8, S1

K, PPIA, PRDX1, PRDX2, PTBP1, PTPRJ, RAB10, RAB33B, RAC2, RAP1B, RCC2, RHOA, RPLP0, RPS15, RPS3A
AB1B, RAB33B, RAC2, RAP1B, RCC2, RHOA, RPL11, RPLP0, RPS15, RPS23, RPSA, RTN3, RTN4, S100A8, S1

STOM, TCP1, TFRC, THBS1, TMEM109, TUBA1C, TUBB, UBE2L3, UBQLN1, VDAC2, YWHAB, YWHAE, YWH
B, RAB27B, RAC2, RAP1B, RCC2, RHOA, RPL11, RPS15, RPS15A, RTN4, S100A8, S100A9, SOD1, SPN, SPTB

AB10, RAB33B, RAC2, RAP1B, RCC2, RHOA, RPLP0, RTN3, RTN4, S100A8, S100A9, SELPLG, SLC2A1, SOD1

PA8, HSPA9, HSPD1, ILK, INPP5D, ITGA2B, ITGB1, ITGB3, JUP, KANSL3, KRT1, KRT10, KRT14, KRT15, KRT1

PL8, RPLP0, RPS15, RPS15A, RPS23, RPS3A, RPSA, S100A8, S100A9, SLC2A1, SPTBN1, SRI, STOM, TAGLN2
PL8, RPLP0, RPS15, RPS15A, RPS23, RPS3A, RPSA, S100A8, S100A9, SLC2A1, SPTBN1, SRI, STOM, TAGLN2

l, MYADM, MYH9, MYL6, NASP, NCL, PECAM1, PF4V1, PFN1, PGK1, PKM, PLEK, PPIA, PRDX1, PRDX2, PT(

, MATR3, MMRN1, MSN, MYADM, MYH9, NASP, NCL, NPM1, PATJ, PECAM1, PF4V1, PGK1, PKM, PLEK, P

3B, RAC2, RAP1B, RCC2, RHOA, RPL11, RPL8, RPLP0, RPS15, RPS15A, RPS23, RPS3A, RPSA, RTN4, SF3A3,
:, RAP1B, RCC2, RHOA, RPL11, RPL8, RPLP0, RPS15, RPS15A, RPS23, RPS3A, RPSA, RTN4, SF3A3, SPTBN1,

C21, HINT1, HLA-A, HLA-B, HMBS, HNRNPA2B1, HNRNPA3, HNRNPC, HNRNPF, HNRNPH1, HNRNPH3, HN

/1, PPIA, PSMA5, PSMA6, RAC2, RPL11, RPL8, RPLP0, RPS15, RPS15A, RPS23, RPS3A, RPSA, SF3A3, SOD1

1, SRGN, SYNCRIP, TFRC, THBS1, TNKS1BP1, TSPAN14, TTLL12, UBE2L3, UBQLN1, VIM, XRCC5, YWHAB, Y

-K, ITGA2B, ITGB1, ITGB3, JUP, KANSL3, KRT1, KRT10, KRT14, KRT15, KRT16, KRT2, KRT5, KRT6A, KRT9, L

ΓGS1, PTPRJ, RAB10, RAB33B, RAC2, RAP1B, RCC2, RHOA, RPL11, RPLP0, RPS15, RTN4, S100A8, S100A9,

B1, HNRNPA3, HNRNPC, HNRNPF, HNRNPH1, HNRNPH3, HNRNPU, HRNR, HSP90AA1, HSPA1B, HSPA8, H

, RPSA, RTN3, RTN4, S100A8, S100A9, SOD1, SPTBN1, SRGN, SYNCRIP, TFRC, THBS1, TKT, TNKS1BP1, TSP

A, RPL11, RPS15, RTN3, RTN4, S100A8, S100A9, SOD1, SPN, SPTBN1, SRGN, SRI, SYNCRIP, TCP1, TFRC, TH

C21, HINT1, HLA-A, HLA-B, HMBS, HNRNPA2B1, HNRNPA3, HNRNPC, HNRNPF, HNRNPH1, HNRNPH3, HN

JP, KANSL3, KRT16, KRT17, KRT5, KRT6A, LBP, LGALS3BP, LRP1, LTF, MMRN1, MSN, MYADM, MYH9, NC

NKS1BP1, TTLL12, UBQLN1, VCL, VDAC2, VIM, XRCC5, YWHAB, YWHAE, YWHAG, YWHAH, YWHAZ

BGRL3, SOD1, SPN, SPTBN1, SRGN, SRI, SYNCRIP, THBS1, TKT, TSPAN14, TUBB, UBE2L3, UBQLN1, VCL, V
BGRL3, SOD1, SPN, SPTBN1, SRGN, SRI, SYNCRIP, THBS1, TKT, TSPAN14, TUBB, UBE2L3, UBQLN1, VCL, V
BGRL3, SOD1, SPN, SPTBN1, SRGN, SRI, SYNCRIP, THBS1, TKT, TSPAN14, TUBB, UBE2L3, UBQLN1, VCL, V

, PTPRJ, QSOX1, RAB1B, RAC2, RHOA, RPL11, RPS15, RTN3, RTN4, S100A8, S100A9, SOD1, SPN, SPTBN1,

ITGB1, ITGB3, JUP, KANSL3, KRT1, KRT10, KRT16, KRT17, KRT2, KRT5, KRT6A, LBP, LGALS3BP, LRP1, LTF,

.K, INPP5D, ITGA2B, ITGA6, ITGB1, ITGB3, JUP, KANSL3, KRT1, KRT10, KRT16, KRT17, KRT2, KRT5, KRT6A,

1, HLA-A, HLA-B, HMBS, HNRNPA2B1, HNRNPA3, HNRNPC, HNRNPF, HNRNPH1, HNRNPH3, HNRNPU, HR

RI, TCP1, THBS1, TKT, TPM4, TTLL12, TUBA1C, TUBA4A, TUBB, TUBB1, UBE2L3, VDAC2, XRCC5

JP, KANSL3, KRT1, KRT10, KRT17, KRT2, LBP, LDHA, LDHB, LRBA, LRP1, LTF, LYZ, MATR3, MSN, MYADM, I

RCC2, RHOA, RPL11, RPLP0, RPS15, RPS15A, RPS23, RPS3A, RPSA, RTN4, S100A8, S100A9, SF3A3, SH3B(

C21, HINT1, HLA-A, HLA-B, HMBS, HNRNPA2B1, HNRNPA3, HNRNPC, HNRNPF, HNRNPH1, HNRNPH3, HN

FRC, THBS1, TLN1, TMEM109, TSPAN14, TTLL12, UBQLN1, VDAC2, VIM, YWHAB, YWHAE, YWHAG, YWHA

S1, TLN1, TMEM109, TSPAN14, TTLL12, UBQLN1, VDAC2, VIM, YWHAB, YWHAE, YWHAG, YWHAH, YWH

MYH9, NANS, NASP, NCL, NPM1, OSBPL2, PCBP1, PCYOX1, PECAM1, PFN1, PGK1, PKM, PLEK, PNPLA8, P

PCYOX1, PECAM1, PFN1, PGK1, PKM, PLEK, PNPLA8, PPIA, PRDX1, PRDX2, PRDX6, PSMA5, PSMA6, PTBP

T1, KRT10, KRT17, KRT2, LBP, LDHA, LDHB, LRP1, LTF, MATR3, MSN, MYADM, MYH9, NANS, NASP, NCL, I

SN, MYADM, MYH9, NASP, NCL, NPM1, PCBP1, PCYOX1, PECAM1, PFN1, PGK1, PKM, PPIA, PRDX2, PRD

ANS, NASP, NCL, NPM1, PCBP1, PCYOX1, PECAM1, PFN1, PGK1, PKM, PNPLA8, PPIA, PRDX2, PRDX6, PS

3A3, SOD1, SPN, SPTBN1, SRGN, SRI, SYNCRIP, TFRC, THBS1, TNKS1BP1, TSPAN14, UBE2L3, VIM, WBP11,

A9, SF3A3, SOD1, SRI, SYNCRIP, TCP1, TFRC, THBS1, TKT, TNKS1BP1, UBE2L3, VIM, WBP11, XRCC5, YWHA

PRDX1, PRDX2, PRDX6, PSMA5, PSMA6, PTBP1, PTGS1, PTPRJ, QSOX1, RAB10, RAB1B, RAB27B, RAC2, RA
PRDX1, PRDX2, PRDX6, PSMA5, PSMA6, PTBP1, PTGS1, PTPRJ, QSOX1, RAB10, RAB1B, RAB27B, RAC2, RA
PRDX1, PRDX2, PRDX6, PSMA5, PSMA6, PTBP1, PTGS1, PTPRJ, QSOX1, RAB10, RAB1B, RAB27B, RAC2, RA
PRDX1, PRDX2, PRDX6, PSMA5, PSMA6, PTBP1, PTGS1, PTPRJ, QSOX1, RAB10, RAB1B, RAB27B, RAC2, RA
MRN1, MSN, MYH9, MYL12B, MYL6, NANS, NCL, PATJ, PCBP1, PCYOX1, PECAM1, PFN1, PGK1, PKM, PPIA
PF4V1, PFN1, PGK1, PKM, PPIA, PRDX1, PRDX2, PRDX6, PSMA5, PSMA6, PTBP1, PTGS1, PTPRJ, QSOX1,
L, PATJ, PCBP1, PCYOX1, PECAM1, PF4V1, PFN1, PGK1, PKM, PLEK, PPIA, PRDX1, PRDX2, PRDX6, PSMA5,

P, KPRP, KRT1, KRT10, KRT14, KRT15, KRT16, KRT17, KRT2, KRT5, KRT6A, KRT6B, KRT78, KRT9, LDHA, LDH
CC2, RHOA, RPL11, RPL8, RPLP0, RPS15, RPS15A, RPS23, RPS3A, RPSA, S100A8, S100A9, SLC2A1, SOD1, S

HSPA9, HSPD1, ICAM2, ILK, INPP5D, ITGA2B, ITGA6, ITGB1, ITGB3, JUP, KPRP, KRT1, KRT10, KRT14, KRT1

A, KRT9, LBP, LDHA, LDHB, LGALS3BP, LRBA, LRP1, LTF, M6PR, MATR3, MSN, MYADM, MYH9, MYL6, NCL,

OD1, SRGN, SRI, STOM, SYNCRIP, TCP1, TFRC, THBS1, TKT, TMEM109, TMX2, TSPAN14, TUBB, UBQLN1, V

ANSL3, KPRP, KRT1, KRT10, KRT14, KRT15, KRT16, KRT2, KRT5, KRT6A, KRT6B, KRT78, KRT9, LBP, LDHA,

, RAB10, RAB27B, RAC2, RAP1B, RCC2, RHOA, RPSA, RTN3, RTN4, S100A8, S100A9, SELPLG, SLC2A1, SLC

33, JUP, KANSL3, KPRP, KRT1, KRT10, KRT14, KRT15, KRT16, KRT17, KRT2, KRT5, KRT6A, KRT6B, KRT78, K

RNPU, HRNR, HSP90AA1, HSPA1B, HSPA8, HSPA9, HSPD1, ICAM2, ILK, INPP5D, ITGA2B, ITGA6, ITGB1, I

100A8, S100A9, SELPLG, SLC2A1, SLC2A3, SOD1, SPN, SPTBN1, SRI, STOM, TFRC, THBS1, TLN1, TNKS1BP

TFRC, THBS1, TMEM109, TSPAN14, TUBA1C, TUBB, UBE2L3, UBQLN1, VCL, VDAC2, XRCC5, YWHAB, YW

P5D, ITGA2B, ITGB1, ITGB3, JUP, KANSL3, KPRP, KRT1, KRT10, KRT14, KRT15, KRT16, KRT17, KRT2, KRT

P1B, RCC2, RHOA, RPL11, RPS15, RPS15A, RTN4, S100A8, S100A9, SOD1, SPN, SPTBN1, SRI, STOM, SYN

00A9, SF3A3, SLC2A1, SOD1, SPTBN1, SRGN, TCP1, TFRC, THBS1, TLN1, TNKS1BP1, TPM4, TUBA1C, TUB

, RTN3, RTN4, S100A8, S100A9, SELPLG, SLC2A1, SOD1, SPN, SPTBN1, SRGN, SRI, SYNCRIP, TAGLN2, TFR

00A9, SF3A3, SLC2A1, SOD1, SPTBN1, SRGN, TCP1, TFRC, THBS1, TLN1, TNKS1BP1, TPM4, TUBA1C, TUB

3N1, SRI, STOM, SYNCRIP, TCP1, TFRC, THBS1, TNKS1BP1, TSPAN14, UBE2L3, UBQLN1, VIM, XRCC5, YWH

l, SPN, SPTBN1, SRGN, SRI, TAGLN2, TFRC, THBS1, TMX2, TUBB, VCL, VIM, XRCC5, YWHAE, YWHAG, YWH

.6, KRT17, KRT2, KRT5, KRT6A, KRT6B, KRT78, KRT9, LDHA, LDHB, LGALS3BP, LRBA, LRP1, LTF, LYZ, M6PR

2, TCP1, TLN1, TNKS1BP1, TPM4, TTLL12, TUBA1C, TUBA4A, TUBB, TUBB1, VCL, VDAC2, VIM, XRCC5
2, TCP1, TLN1, TNKS1BP1, TPM4, TTLL12, TUBA1C, TUBA4A, TUBB, TUBB1, VCL, VDAC2, VIM, XRCC5

GS1, PTPRJ, RAB10, RAB33B, RAC2, RHOA, RPS15, RTN3, RTN4, S100A8, S100A9, SELPLG, SLC2A1, SLC2A

PNPLA8, PPIA, PRDX1, PRDX2, PRDX6, PSMA6, PTBP1, PTGS1, PTPRJ, RAB10, RAB33B, RAC2, RAP1B, RCC

SPTBN1, SYNCRIP, TCP1, TFRC, TKT, TTLL12, TUBA1C, TUBA4A, TUBB, TUBB1, UBE2L3, VDAC2, VIM, WB
SYNCRIP, TCP1, TFRC, TKT, TTLL12, TUBA1C, TUBA4A, TUBB, TUBB1, UBE2L3, VDAC2, VIM, WBP11, XRC

NRNPU, HRNR, HSP90AA1, HSPA1B, HSPA8, HSPA9, HSPD1, ICAM2, ILK, INPP5D, ITGA2B, ITGA6, ITGB1, IT

l, SPTBN1, SYNCRIP, TCP1, TFRC, THBS1, TNKS1BP1, TPM4, TUBB, UBE2L3, UBQLN1, VCL, VDAC2, VIM, W

DHA, LDHB, LGALS3BP, LRBA, LRP1, LTF, LYZ, M6PR, MATR3, MMRN1, MSN, MYH9, NASP, NCL, NPM1, C

SELPLG, SLC2A1, SOD1, SPN, SPTBN1, SRGN, SRI, SYNCRIP, TFRC, THBS1, TLN1, TMEM109, TNKS1BP1, TS

ISPA9, HSPD1, ICAM2, ILK, INPP5D, ITGA2B, ITGA6, ITGB1, ITGB3, JUP, KANSL3, KRT1, KRT10, KRT14, KRT

ıRNPU, HRNR, HSP90AA1, HSPA1B, HSPA8, HSPA9, HSPD1, ICAM2, ILK, INPP5D, ITGA2B, ITGA6, ITGB1, IT

L, NPM1, PATJ, PCBP1, PECAM1, PF4V1, PFN1, PKM, PLEK, PNPLA8, PPIA, PRDX1, PRDX2, PRDX6, PSMA6

SRGN, SRI, SYNCRIP, TCP1, TFRC, THBS1, TNKS1BP1, UBE2L3, UBQLN1, VIM, XRCC5, YWHAB, YWHAE, YV

MATR3, MMRN1, MSN, MYADM, MYH9, MYL12B, NCL, NPM1, PATJ, PCBP1, PECAM1, PF4V1, PFN1, PG

LBP, LGALS3BP, LRP1, LTF, LYZ, MATR3, MMRN1, MSN, MYADM, MYH9, MYL12B, NCL, NPM1, PATJ, PCI

NR, HSP90AA1, HSPA1B, HSPA8, HSPA9, HSPD1, ICAM2, ILK, INPP5D, ITGA2B, ITGA6, ITGB1, ITGB3, JUP,

MYH9, NANS, NASP, NCL, NPM1, OSBPL2, PCBP1, PCYOX1, PECAM1, PFN1, PGK1, PKM, PLEK, PNPLA8, P

GRL3, SLC2A1, SOD1, SPN, SPTBN1, SRI, SYNCRIP, TFRC, TKT, TMEM109, TNKS1BP1, TTLL12, TUBA1C, TU

IRNPU, HRNR, HSP90AA1, HSPA1B, HSPA8, HSPA9, HSPD1, ICAM2, ILK, INPP5D, ITGA2B, ITGA6, ITGB1, IT

ᐳPIA, PRDX2, PRDX6, PSMA5, PSMA6, PTBP1, PTGS1, PTPRJ, RAB1B, RHOA, RPL11, RPL8, RPLP0, RPS15, I

ᐳ1, PTGS1, PTPRJ, QSOX1, RAB1B, RAB33B, RAC2, RHOA, RPL11, RPL8, RPLP0, RPS15, RPS15A, RPS23, RP

NPM1, OSBPL2, PCBP1, PCYOX1, PECAM1, PFN1, PGK1, PKM, PLEK, PNPLA8, PPIA, PRDX2, PRDX6, PSMA

‹6, PSMA5, PSMA6, PTBP1, PTPRJ, RAB1B, RHOA, RPL11, RPL8, RPLP0, RPS15, RPS15A, RPS23, RPS3A, RF

ɪA5, PSMA6, PTBP1, PTPRJ, RAB1B, RHOA, RPL11, RPL8, RPLP0, RPS15, RPS15A, RPS23, RPS3A, RPSA, RT

P1B, RHOA, RPL11, RPLP0, RPS15A, RPS3A, RPSA, S100A8, S100A9, SBSN, SH3BGRL3, SLC2A1, SLC2A3,
P1B, RHOA, RPL11, RPLP0, RPS15A, RPS3A, RPSA, S100A8, S100A9, SBSN, SH3BGRL3, SLC2A1, SLC2A3,
P1B, RHOA, RPL11, RPLP0, RPS15A, RPS3A, RPSA, S100A8, S100A9, SBSN, SH3BGRL3, SLC2A1, SLC2A3,
P1B, RHOA, RPL11, RPLP0, RPS15A, RPS3A, RPSA, S100A8, S100A9, SBSN, SH3BGRL3, SLC2A1, SLC2A3,
, PRDX1, PRDX2, PRDX6, PSMA5, PSMA6, PTBP1, PTGS1, PTPRJ, QSOX1, RAB10, RAB1B, RAB27B, RAB33
RAB10, RAB1B, RAB27B, RAC2, RAP1B, RHOA, RPL11, RPLP0, RPS15A, RPS3A, RPSA, RTN3, S100A8, S100
PSMA6, PTBP1, PTGS1, PTPRJ, QSOX1, RAB10, RAB1B, RAB27B, RAC2, RAP1B, RHOA, RPL11, RPLP0, RP

HB, LGALS3BP, LRBA, LRP1, LTF, LYZ, M6PR, MMRN1, MSN, MYADM, MYH9, MYL12B, MYL6, NANS, NAS
PTBN1, SRI, SYNCRIP, TAGLN2, TCP1, TKT, TLN1, TNKS1BP1, TPM4, TUBA4A, TUBB, UBE2L3, UBQLN1, V

15, KRT16, KRT17, KRT2, KRT5, KRT6A, KRT6B, KRT78, LBP, LDHA, LDHB, LGALS3BP, LRBA, LRP1, LTF, LYZ

NPM1, OSBPL2, PATJ, PCBP1, PCYOX1, PECAM1, PFN1, PGK1, PLEK, PNPLA8, PPIA, PRDX6, PTBP1, PTG

LDHB, LGALS3BP, LRBA, LRP1, LTF, LYZ, M6PR, MATR3, MMRN1, MSN, MYH9, MYL12B, MYL6, NANS, NA

2A3, SOD1, SPN, SPTBN1, SRI, STOM, TFRC, THBS1, TLN1, TNKS1BP1, TPM4, TSPAN14, UBQLN1, VCL, VIN

RT9, LBP, LDHA, LDHB, LGALS3BP, LRBA, LRP1, LTF, LYZ, M6PR, MATR3, MMRN1, MSN, MYADM, MYH1

TGB3, JUP, KPRP, KRT1, KRT10, KRT14, KRT15, KRT16, KRT17, KRT2, KRT5, KRT6A, KRT6B, KRT78, LBP, LD

, KRT6A, KRT6B, KRT78, KRT9, LDHA, LDHB, LGALS3BP, LRBA, LRP1, LTF, LYZ, M6PR, MATR3, MMRN1, M

RIP, TCP1, TFRC, THBS1, TNKS1BP1, TSPAN14, UBE2L3, UBQLN1, VIM, XRCC5, YWHAE, YWHAH

, MATR3, MMRN1, MSN, MYADM, MYH16, MYH9, MYL12B, MYL6, NASP, NCL, NPM1, OSBPL2, PATJ, PC

3, SOD1, SPN, SPTBN1, SRGN, SRI, SYNCRIP, TFRC, THBS1, TLN1, TMX2, TPM4, VCL, VIM, XRCC5, YWHAE

2, RHOA, RPL11, RPLP0, RPS15, RPS15A, RTN4, S100A8, S100A9, SELPLG, SLC2A1, SOD1, SPN, SPTBN1, S

TGB3, JUP, KPRP, KRT1, KRT10, KRT14, KRT15, KRT16, KRT17, KRT2, KRT5, KRT6A, KRT6B, KRT78, KRT9, L

)SBPL2, PCBP1, PCYOX1, PECAM1, PFN1, PKM, PNPLA8, PPIA, PRDX1, PRDX6, PSMA5, PSMA6, PTBP1, PT

SPAN14, TTLL12, UBE2L3, UBQLN1, VDAC2, VIM, XRCC5, YWHAB, YWHAE, YWHAG, YWHAH, YWHAZ

T15, KRT16, KRT17, KRT2, KRT5, KRT6A, KRT6B, KRT78, KRT9, LBP, LDHA, LDHB, LGALS3BP, LRBA, LRP1, L

TGB3, JUP, KANSL3, KPRP, KRT1, KRT10, KRT14, KRT15, KRT16, KRT17, KRT2, KRT5, KRT6A, KRT6B, KRT78

, PTBP1, PTGS1, PTPRJ, QSOX1, RAB10, RAB1B, RAB27B, RAB33B, RAC2, RAP1B, RCC2, RHOA, RPL11, RP

K1, PKM, PLEK, PNPLA8, PPIA, PRDX1, PRDX2, PRDX6, PSMA6, PTBP1, PTGS1, PTPRJ, QSOX1, RAB10, RA

BP1, PECAM1, PF4V1, PFN1, PGK1, PKM, PLEK, PNPLA8, PPIA, PRDX1, PRDX2, PRDX6, PSMA6, PTBP1, PT

KANSL3, KRT1, KRT10, KRT14, KRT15, KRT16, KRT17, KRT2, KRT5, KRT6A, KRT6B, KRT78, KRT9, LBP, LDH

PIA, PRDX1, PRDX2, PRDX6, PSMA5, PSMA6, PTBP1, PTGS1, PTPRJ, QSOX1, RAB1B, RAB33B, RAC2, RHO/

TGB3, JUP, KANSL3, KPRP, KRT1, KRT10, KRT14, KRT15, KRT16, KRT17, KRT2, KRT5, KRT6A, KRT6B, KRT78

ᴚPS15A, RPS23, RPS3A, RPSA, RTN3, RTN4, S100A8, S100A9, SACM1L, SF3A3, SLC2A1, SLC2A3, SOD1, SP

S3A, RPSA, RTN3, RTN4, S100A8, S100A9, SACM1L, SF3A3, SLC2A1, SLC2A3, SOD1, SPTBN1, SRI, SYNCRIF

5, PSMA6, PTBP1, PTGS1, PTPRJ, RAB1B, RHOA, RPL11, RPL8, RPLP0, RPS15, RPS15A, RPS23, RPS3A, RPS

ᴾSA, RTN3, RTN4, S100A8, S100A9, SF3A3, SOD1, SPN, SPTBN1, SRGN, SRI, SYNCRIP, TCP1, TFRC, THBS1,

N3, RTN4, S100A8, S100A9, SF3A3, SOD1, SPTBN1, SRGN, SRI, SYNCRIP, TCP1, TFRC, THBS1, TKT, TNKS1I

SOD1, SPN, SPTBN1, SRI, STOM, TAGLN2, TCP1, TFRC, THBS1, TKT, TLN1, TMEM109, TPM4, TUBA4A, TUE
SOD1, SPN, SPTBN1, SRI, STOM, TAGLN2, TCP1, TFRC, THBS1, TKT, TLN1, TMEM109, TPM4, TUBA4A, TUE
SOD1, SPN, SPTBN1, SRI, STOM, TAGLN2, TCP1, TFRC, THBS1, TKT, TLN1, TMEM109, TPM4, TUBA4A, TUE
SOD1, SPN, SPTBN1, SRI, STOM, TAGLN2, TCP1, TFRC, THBS1, TKT, TLN1, TMEM109, TPM4, TUBA4A, TUE
B, RAC2, RAP1B, RCC2, RHOA, RPL11, RPLP0, RPS15A, RPS3A, RPSA, S100A8, S100A9, SBSN, SH3BGRL3, S
A9, SBSN, SH3BGRL3, SLC2A1, SLC2A3, SOD1, SPN, SPTBN1, SRGN, SRI, STOM, TAGLN2, TCP1, TFRC, THB
S15A, RPS3A, RPSA, RTN3, S100A8, S100A9, SBSN, SH3BGRL3, SLC2A1, SLC2A3, SOD1, SPN, SPTBN1, SRG

P, NCL, NPM1, OSBPL2, PATJ, PCBP1, PCYOX1, PECAM1, PFN1, PGK1, PKM, PLEK, PNPLA8, PPIA, PRDX1, I

, M6PR, MATR3, MMRN1, MSN, MYADM, MYH9, MYL12B, MYL6, NASP, NCL, NPM1, OSBPL2, PATJ, PCBI

S1, PTPRJ, QSOX1, RAB10, RAB1B, RAB27B, RAB33B, RAC2, RAP1B, RCC2, RHOA, RPL11, RPL8, RPLP0, RPS

ASP, NCL, NPM1, OSBPL2, PATJ, PCBP1, PCYOX1, PECAM1, PFN1, PGK1, PKM, PNPLA8, PPIA, PRDX1, PRD

5, MYH9, MYL12B, MYL6, NANS, NASP, NCL, NPM1, OSBPL2, PATJ, PCBP1, PCYOX1, PECAM1, PFN1, PGK1

DHA, LDHB, LGALS3BP, LRBA, LRP1, LTF, LYZ, M6PR, MATR3, MMRN1, MSN, MYADM, MYH9, MYL12B, MY

MSN, MYADM, MYH16, MYH9, MYL12B, MYL6, NANS, NASP, NCL, NPM1, OSBPL2, PATJ, PCBP1, PCYOX1,

BP1, PCYOX1, PECAM1, PFN1, PKM, PNPLA8, PPIA, PRDX1, PRDX6, PSMA5, PSMA6, PTBP1, PTGS1, PTPR

RGN, SRI, SYNCRIP, TFRC, THBS1, TLN1, TMEM109, TNKS1BP1, TSPAN14, TTLL12, TUBB, UBE2L3, UBQLN

.BP, LDHA, LDHB, LGALS3BP, LRBA, LRP1, LTF, LYZ, M6PR, MATR3, MMRN1, MSN, MYADM, MYH9, MYL1

TGS1, PTPRJ, QSOX1, RAB10, RAB1B, RAB27B, RAB33B, RAC2, RAP1B, RCC2, RHOA, RPL11, RPLP0, RPS15

.TF, LYZ, M6PR, MMRN1, MSN, MYADM, MYH9, MYL6, NANS, NASP, NCL, NPM1, OSBPL2, PATJ, PCBP1, F

3, KRT9, LBP, LDHA, LDHB, LGALS3BP, LRBA, LRP1, LTF, LYZ, M6PR, MATR3, MMRN1, MSN, MYADM, MYH

S15, RPS15A, RPS3A, RTN3, RTN4, S100A8, S100A9, SOD1, SPN, SPTBN1, SRGN, SRI, STOM, SYNCRIP, TCI

B1B, RAB27B, RAB33B, RAC2, RAP1B, RCC2, RHOA, RPL11, RPS15, RPS15A, RPS3A, RTN3, RTN4, S100A8,

'GS1, PTPRJ, QSOX1, RAB10, RAB1B, RAB27B, RAB33B, RAC2, RAP1B, RCC2, RHOA, RPL11, RPLP0, RPS15,

A, LDHB, LGALS3BP, LRBA, LRP1, LTF, LYZ, M6PR, MATR3, MMRN1, MSN, MYADM, MYH9, MYL12B, MYL

A, RPL11, RPL8, RPLP0, RPS15, RPS15A, RPS23, RPS3A, RPSA, RTN3, RTN4, S100A8, S100A9, SACM1L, SF3

3, KRT9, LBP, LDHA, LDHB, LGALS3BP, LRBA, LRP1, LTF, LYZ, M6PR, MATR3, MMRN1, MSN, MYADM, MYH

TBN1, SRGN, SRI, SYNCRIP, TCP1, TFRC, THBS1, TKT, TNKS1BP1, TSPAN14, TTLL12, UBE2L3, UBQLN1, VIM

P, TCP1, TFRC, THBS1, TKT, TNKS1BP1, UBE2L3, UBQLN1, VIM, WBP11, XRCC5, YWHAB, YWHAE, YWHAG

SA, RTN3, RTN4, S100A8, S100A9, SACM1L, SF3A3, SLC2A1, SLC2A3, SOD1, SPN, SPTBN1, SRGN, SRI, SYN

TNKS1BP1, TSPAN14, TTLL12, UBE2L3, UBQLN1, VIM, WBP11, XRCC5, YWHAB, YWHAE, YWHAG, YWHAI

3P1, TSPAN14, TTLL12, UBE2L3, UBQLN1, VIM, WBP11, XRCC5, YWHAB, YWHAE, YWHAG, YWHAH, YWH

SLC2A1, SLC2A3, SOD1, SPN, SPTBN1, SRGN, SRI, STOM, TAGLN2, TCP1, TFRC, THBS1, TKT, TLN1, TMEM1
S1, TKT, TLN1, TMEM109, TPM4, TUBA4A, TUBB, TUBB1, VCL, VIM, YWHAB, YWHAE, YWHAG, YWHAH,
N, SRI, STOM, TAGLN2, TCP1, TFRC, THBS1, TKT, TLN1, TMEM109, TPM4, TUBA4A, TUBB, TUBB1, VCL, V

PRDX2, PRDX6, PSMA5, PSMA6, PTGS1, PTPRJ, QSOX1, RAB10, RAB1B, RAB27B, RAB33B, RAC2, RAP1B,

P1, PCYOX1, PECAM1, PF4V1, PFN1, PGK1, PKM, PLEK, PPIA, PRDX1, PRDX2, PRDX6, PSMA5, PSMA6, PT

S15, RPS15A, RPS23, RPSA, RTN3, RTN4, S100A8, S100A9, SACM1L, SELPLG, SLC2A1, SLC2A3, SOD1, SPN,

)X2, PRDX6, PSMA5, PSMA6, PTBP1, PTGS1, PTPRJ, QSOX1, RAB10, RAB1B, RAB27B, RAB33B, RAC2, RAP

1, PKM, PNPLA8, PPIA, PRDX1, PRDX2, PRDX6, PSMA5, PSMA6, PTBP1, PTGS1, PTPRJ, QSOX1, RAB10, RA

YL6, NASP, NCL, NPM1, OSBPL2, PATJ, PCBP1, PCYOX1, PECAM1, PF4V1, PFN1, PGK1, PKM, PLEK, PNPLA

PECAM1, PFN1, PGK1, PKM, PLEK, PNPLA8, PPIA, PRDX1, PRDX2, PRDX6, PSMA5, PSMA6, PTBP1, PTGS1

J, QSOX1, RAB10, RAB1B, RAB27B, RAB33B, RAC2, RAP1B, RCC2, RHOA, RPL11, RPL8, RPLP0, RPS15, RPS

.2B, MYL6, NANS, NASP, NCL, NPM1, OSBPL2, PATJ, PCBP1, PCYOX1, PECAM1, PF4V1, PFN1, PGK1, PKM,

, RPS15A, RPS23, RPS3A, RPSA, RTN3, RTN4, S100A8, S100A9, SACM1L, SF3A3, SH3BGRL3, SLC2A1, SLC2

PCYOX1, PECAM1, PF4V1, PFN1, PGK1, PKM, PLEK, PNPLA8, PPIA, PRDX1, PRDX2, PRDX6, PSMA5, PSMA

H16, MYH9, MYL12B, MYL6, NANS, NASP, NCL, NPM1, OSBPL2, PATJ, PCBP1, PCYOX1, PECAM1, PF4V1, P

P1, TFRC, THBS1, TLN1, TMEM109, TNKS1BP1, TSPAN14, TTLL12, TUBB, UBE2L3, UBQLN1, VCL, VDAC2, V

S100A9, SOD1, SPN, SPTBN1, SRGN, SRI, STOM, SYNCRIP, TCP1, TFRC, THBS1, TKT, TLN1, TMEM109, TN

, RPS15A, RPS3A, RTN3, RTN4, S100A8, S100A9, SLC2A1, SOD1, SPN, SPTBN1, SRGN, SRI, STOM, SYNCRIP

.6, NANS, NASP, NCL, NPM1, OSBPL2, PATJ, PCBP1, PCYOX1, PECAM1, PF4V1, PFN1, PGK1, PKM, PLEK, P

3A3, SLC2A1, SLC2A3, SOD1, SPN, SPTBN1, SRGN, SRI, SYNCRIP, TCP1, TFRC, THBS1, TKT, TNKS1BP1, TSP/

H16, MYH9, MYL12B, MYL6, NANS, NASP, NCL, NPM1, OSBPL2, PATJ, PCBP1, PCYOX1, PECAM1, PF4V1, P

CRIP, TCP1, TFRC, THBS1, TKT, TNKS1BP1, TSPAN14, TTLL12, UBE2L3, UBQLN1, VIM, WBP11, XRCC5, YW

RCC2, RHOA, R

BP1, PTGS1, PT

1B, RCC2, RHOA

\B1B, RAB27B, RA

\8, PPIA, PRDX1

., PTPRJ, QSOX1, R

;15A, RPS23, RP

, PLEK, PNPLA8,

, PLEK, PNPLA8,

.A3, SOD1, SPN

6, PTBP1, PTGS

PFN1, PGK1, PKM

VIM, XRCC5

'NPLA8, PPIA, P

ꟼFN1, PGK1, PKM

/HAB, YWHAE,

Table 4. REACTOME pathways associated with T-cell-originated proteins identified in CD3

REACTOME ID	REACTOME pathway
R-HSA-6798695	Neutrophil degranulation
R-HSA-76002	Platelet activation, signaling and aggregation
R-HSA-109582	Hemostasis
R-HSA-114608	Platelet degranulation
R-HSA-76005	Response to elevated platelet cytosolic Ca2+
R-HSA-168249	Innate Immune System
R-HSA-168256	Immune System
R-HSA-8950505	Gene and protein expression by JAK-STAT signaling after Interleukin-12 stimu
R-HSA-447115	Interleukin-12 family signaling
R-HSA-389957	Prefoldin mediated transfer of substrate to CCT/TriC
R-HSA-9020591	Interleukin-12 signaling
R-HSA-194315	Signaling by Rho GTPases
R-HSA-390450	Folding of actin by CCT/TriC
R-HSA-9716542	Signaling by Rho GTPases, Miro GTPases and RHOBTB3
R-HSA-195258	RHO GTPase Effectors
R-HSA-389958	Cooperation of Prefoldin and TriC/CCT in actin and tubulin folding
R-HSA-389960	Formation of tubulin folding intermediates by CCT/TriC
R-HSA-6809371	Formation of the cornified envelope
R-HSA-422475	Axon guidance
R-HSA-1445148	Translocation of SLC2A4 (GLUT4) to the plasma membrane
R-HSA-9675108	Nervous system development
R-HSA-1266738	Developmental Biology
R-HSA-9692914	SARS-CoV-1-host interactions
R-HSA-390466	Chaperonin-mediated protein folding
R-HSA-6802946	Signaling by moderate kinase activity BRAF mutants
R-HSA-6802949	Signaling by RAS mutants
R-HSA-6802955	Paradoxical activation of RAF signaling by kinase inactive BRAF
R-HSA-9649948	Signaling downstream of RAS mutants
R-HSA-391251	Protein folding
R-HSA-1643685	Disease
R-HSA-6814122	Cooperation of PDCL (PhLP1) and TRiC/CCT in G-protein beta folding
R-HSA-9678108	SARS-CoV-1 Infection
R-HSA-6805567	Keratinization
R-HSA-5663205	Infectious disease
R-HSA-9656223	Signaling by RAF1 mutants
R-HSA-9755779	SARS-CoV-2 targets host intracellular signalling and regulatory pathways
R-HSA-5625740	RHO GTPases activate PKNs
R-HSA-1280215	Cytokine Signaling in Immune system
R-HSA-75035	Chk1/Chk2(Cds1) mediated inactivation of Cyclin B:Cdk1 complex
R-HSA-202733	Cell surface interactions at the vascular wall
R-HSA-373760	L1CAM interactions
R-HSA-6802948	Signaling by high-kinase activity BRAF mutants
R-HSA-5627123	RHO GTPases activate PAKs
R-HSA-3299685	Detoxification of Reactive Oxygen Species
R-HSA-111447	Activation of BAD and translocation to mitochondria
R-HSA-9735871	SARS-CoV-1 targets host intracellular signalling and regulatory pathways
R-HSA-449147	Signaling by Interleukins

R-HSA-156902	Peptide chain elongation
R-HSA-5674135	MAP2K and MAPK activation
R-HSA-9705683	SARS-CoV-2-host interactions
R-HSA-2682334	EPH-Ephrin signaling
R-HSA-156842	Eukaryotic Translation Elongation
R-HSA-445355	Smooth Muscle Contraction
R-HSA-5626467	RHO GTPases activate IQGAPs
R-HSA-5627117	RHO GTPases Activate ROCKs
R-HSA-373755	Semaphorin interactions
R-HSA-6802957	Oncogenic MAPK signaling
R-HSA-446107	Type I hemidesmosome assembly
R-HSA-216083	Integrin cell surface interactions
R-HSA-6802952	Signaling by BRAF and RAF1 fusions
R-HSA-1500931	Cell-Cell communication
R-HSA-5628897	TP53 Regulates Metabolic Genes
R-HSA-9706574	RHOBTB GTPase Cycle
R-HSA-430116	GP1b-IX-V activation signalling
R-HSA-9013418	RHOBTB2 GTPase cycle
R-HSA-9679506	SARS-CoV Infections
R-HSA-390471	Association of TriC/CCT with target proteins during biosynthesis
R-HSA-9735869	SARS-CoV-1 modulates host translation machinery
R-HSA-446728	Cell junction organization
R-HSA-76009	Platelet Aggregation (Plug Formation)
R-HSA-5357801	Programmed Cell Death
R-HSA-9824446	Viral Infection Pathways
R-HSA-168255	Influenza Infection
R-HSA-5336415	Uptake and function of diphtheria toxin
R-HSA-6799990	Metal sequestration by antimicrobial proteins
R-HSA-372708	p130Cas linkage to MAPK signaling for integrins
R-HSA-354194	GRB2:SOS provides linkage to MAPK signaling for Integrins
R-HSA-75892	Platelet Adhesion to exposed collagen
R-HSA-983170	Antigen Presentation: Folding, assembly and peptide loading of class I MHC
R-HSA-9012999	RHO GTPase cycle
R-HSA-1236975	Antigen processing-Cross presentation
R-HSA-114452	Activation of BH3-only proteins
R-HSA-2262752	Cellular responses to stress
R-HSA-437239	Recycling pathway of L1
R-HSA-9694516	SARS-CoV-2 Infection
R-HSA-446353	Cell-extracellular matrix interactions
R-HSA-196836	Vitamin C (ascorbate) metabolism
R-HSA-5653656	Vesicle-mediated transport
R-HSA-8953897	Cellular responses to stimuli
R-HSA-376176	Signaling by ROBO receptors
R-HSA-1236974	ER-Phagosome pathway
R-HSA-8876725	Protein methylation
R-HSA-2408557	Selenocysteine synthesis
R-HSA-72764	Eukaryotic Translation Termination
R-HSA-5625900	RHO GTPases activate CIT
R-HSA-447041	CHL1 interactions
R-HSA-2408522	Selenoamino acid metabolism

R-HSA-975956	Nonsense Mediated Decay (NMD) independent of the Exon Junction Comple›
R-HSA-1280218	Adaptive Immune System
R-HSA-5686938	Regulation of TLR by endogenous ligand
R-HSA-416572	Sema4D induced cell migration and growth-cone collapse
R-HSA-3371497	HSP90 chaperone cycle for steroid hormone receptors (SHR) in the presence
R-HSA-4420097	VEGFA-VEGFR2 Pathway
R-HSA-9613829	Chaperone Mediated Autophagy
R-HSA-5663202	Diseases of signal transduction by growth factor receptors and second messe
R-HSA-5339562	Uptake and actions of bacterial toxins
R-HSA-72163	mRNA Splicing - Major Pathway
R-HSA-192823	Viral mRNA Translation
R-HSA-72689	Formation of a pool of free 40S subunits
R-HSA-9633012	Response of EIF2AK4 (GCN2) to amino acid deficiency
R-HSA-9833482	PKR-mediated signaling
R-HSA-9013422	RHOBTB1 GTPase cycle
R-HSA-5620922	BBSome-mediated cargo-targeting to cilium
R-HSA-109581	Apoptosis
R-HSA-9614399	Regulation of localization of FOXO transcription factors
R-HSA-1236977	Endosomal/Vacuolar pathway
R-HSA-5668599	RHO GTPases Activate NADPH Oxidases
R-HSA-72172	mRNA Splicing
R-HSA-2029482	Regulation of actin dynamics for phagocytic cup formation
R-HSA-8953854	Metabolism of RNA
R-HSA-400685	Sema4D in semaphorin signaling
R-HSA-210991	Basigin interactions
R-HSA-3928662	EPHB-mediated forward signaling
R-HSA-194138	Signaling by VEGF
R-HSA-3371511	HSF1 activation
R-HSA-210990	PECAM1 interactions
R-HSA-9646399	Aggrephagy
R-HSA-156827	L13a-mediated translational silencing of Ceruloplasmin expression
R-HSA-72706	GTP hydrolysis and joining of the 60S ribosomal subunit
R-HSA-1799339	SRP-dependent cotranslational protein targeting to membrane
R-HSA-354192	Integrin signaling
R-HSA-3000170	Syndecan interactions
R-HSA-199991	Membrane Trafficking
R-HSA-5663220	RHO GTPases Activate Formins
R-HSA-927802	Nonsense-Mediated Decay (NMD)
R-HSA-975957	Nonsense Mediated Decay (NMD) enhanced by the Exon Junction Complex (E
R-HSA-9636667	Manipulation of host energy metabolism
R-HSA-446343	Localization of the PINCH-ILK-PARVIN complex to focal adhesions
R-HSA-5617833	Cilium Assembly
R-HSA-190828	Gap junction trafficking
R-HSA-9010553	Regulation of expression of SLITs and ROBOs
R-HSA-3371568	Attenuation phase
R-HSA-418359	Reduction of cytosolic Ca++ levels
R-HSA-1852241	Organelle biogenesis and maintenance
R-HSA-9619483	Activation of AMPK downstream of NMDARs
R-HSA-380320	Recruitment of NuMA to mitotic centrosomes
R-HSA-168273	Influenza Viral RNA Transcription and Replication

R-HSA-72613	Eukaryotic Translation Initiation
R-HSA-72737	Cap-dependent Translation Initiation
R-HSA-3928663	EPHA-mediated growth cone collapse
R-HSA-157858	Gap junction trafficking and regulation
R-HSA-9754678	SARS-CoV-2 modulates host translation machinery
R-HSA-392517	Rap1 signalling
R-HSA-72695	Formation of the ternary complex, and subsequently, the 43S complex
R-HSA-8869496	TFAP2A acts as a transcriptional repressor during retinoic acid induced cell di
R-HSA-9711123	Cellular response to chemical stress
R-HSA-6807878	COPI-mediated anterograde transport
R-HSA-8852276	The role of GTSE1 in G2/M progression after G2 checkpoint
R-HSA-109606	Intrinsic Pathway for Apoptosis
R-HSA-70263	Gluconeogenesis
R-HSA-114604	GPVI-mediated activation cascade
R-HSA-5602498	MyD88 deficiency (TLR2/4)
R-HSA-190840	Microtubule-dependent trafficking of connexons from Golgi to the plasma me
R-HSA-9663891	Selective autophagy
R-HSA-9673221	Defective F9 activation
R-HSA-392499	Metabolism of proteins
R-HSA-6785807	Interleukin-4 and Interleukin-13 signaling
R-HSA-5603041	IRAK4 deficiency (TLR2/4)
R-HSA-190872	Transport of connexons to the plasma membrane
R-HSA-9824439	Bacterial Infection Pathways
R-HSA-72649	Translation initiation complex formation
R-HSA-72702	Ribosomal scanning and start codon recognition
R-HSA-977225	Amyloid fiber formation
R-HSA-111465	Apoptotic cleavage of cellular proteins
R-HSA-72662	Activation of the mRNA upon binding of the cap-binding complex and eIFs, ar
R-HSA-2029480	Fcgamma receptor (FCGR) dependent phagocytosis
R-HSA-9845620	Enhanced binding of GP1BA variant to VWF multimer:collagen
R-HSA-9846298	Defective binding of VWF variant to GPIb:IX:V
R-HSA-445144	Signal transduction by L1
R-HSA-397014	Muscle contraction
R-HSA-450531	Regulation of mRNA stability by proteins that bind AU-rich elements
R-HSA-3371556	Cellular response to heat stress
R-HSA-3858494	Beta-catenin independent WNT signaling
R-HSA-8862803	Deregulated CDK5 triggers multiple neurodegenerative pathways in Alzheime
R-HSA-8863678	Neurodegenerative Diseases
R-HSA-389977	Post-chaperonin tubulin folding pathway
R-HSA-8955332	Carboxyterminal post-translational modifications of tubulin
R-HSA-9700206	Signaling by ALK in cancer
R-HSA-9725370	Signaling by ALK fusions and activated point mutants
R-HSA-4086400	PCP/CE pathway
R-HSA-9006934	Signaling by Receptor Tyrosine Kinases
R-HSA-9823587	Defects of platelet adhesion to exposed collagen
R-HSA-9818028	NFE2L2 regulates pentose phosphate pathway genes
R-HSA-1236973	Cross-presentation of particulate exogenous antigens (phagosomes)
R-HSA-5673001	RAF/MAP kinase cascade
R-HSA-69473	G2/M DNA damage checkpoint
R-HSA-3371571	HSF1-dependent transactivation

R-HSA-72203	Processing of Capped Intron-Containing Pre-mRNA
R-HSA-1169410	Antiviral mechanism by IFN-stimulated genes
R-HSA-5684996	MAPK1/MAPK3 signaling
R-HSA-6791226	Major pathway of rRNA processing in the nucleolus and cytosol
R-HSA-380259	Loss of Nlp from mitotic centrosomes
R-HSA-380284	Loss of proteins required for interphase microtubule organization from the ce
R-HSA-9759194	Nuclear events mediated by NFE2L2
R-HSA-9668250	Defective factor IX causes hemophilia B
R-HSA-193634	Axonal growth inhibition (RHOA activation)
R-HSA-9734009	Defective Intrinsic Pathway for Apoptosis
R-HSA-9711097	Cellular response to starvation
R-HSA-5683057	MAPK family signaling cascades
R-HSA-6803157	Antimicrobial peptides
R-HSA-6803529	FGFR2 alternative splicing
R-HSA-70171	Glycolysis
R-HSA-8854518	AURKA Activation by TPX2
R-HSA-9013106	RHOC GTPase cycle
R-HSA-193697	p75NTR regulates axonogenesis
R-HSA-162582	Signal Transduction
R-HSA-1474244	Extracellular matrix organization
R-HSA-8868773	rRNA processing in the nucleus and cytosol
R-HSA-69275	G2/M Transition
R-HSA-198933	Immunoregulatory interactions between a Lymphoid and a non-Lymphoid ce
R-HSA-453274	Mitotic G2-G2/M phases
R-HSA-5620920	Cargo trafficking to the periciliary membrane
R-HSA-69481	G2/M Checkpoints
R-HSA-351906	Apoptotic cleavage of cell adhesion proteins
R-HSA-164940	Nef mediated downregulation of MHC class I complex cell surface expression
R-HSA-9013700	NOTCH4 Activation and Transmission of Signal to the Nucleus
R-HSA-196025	Formation of annular gap junctions
R-HSA-5625970	RHO GTPases activate KTN1
R-HSA-400451	Free fatty acids regulate insulin secretion
R-HSA-2025928	Calcineurin activates NFAT
R-HSA-5218920	VEGFR2 mediated vascular permeability
R-HSA-422356	Regulation of insulin secretion
R-HSA-75153	Apoptotic execution phase
R-HSA-5609977	Defective GALE causes EDG
R-HSA-140180	COX reactions
R-HSA-5619043	Defective SLC2A1 causes GLUT1 deficiency syndrome 1 (GLUT1DS1)
R-HSA-163685	Integration of energy metabolism
R-HSA-418360	Platelet calcium homeostasis
R-HSA-913531	Interferon Signaling
R-HSA-9662361	Sensory processing of sound by outer hair cells of the cochlea
R-HSA-203615	eNOS activation
R-HSA-190873	Gap junction degradation
R-HSA-1474151	Tetrahydrobiopterin (BH4) synthesis, recycling, salvage and regulation
R-HSA-9668328	Sealing of the nuclear envelope (NE) by ESCRT-III
R-HSA-445095	Interaction between L1 and Ankyrins
R-HSA-72312	rRNA processing
R-HSA-9013409	RHOJ GTPase cycle

R-HSA-380270	Recruitment of mitotic centrosome proteins and complexes
R-HSA-380287	Centrosome maturation
R-HSA-450408	AUF1 (hnRNP D0) binds and destabilizes mRNA
R-HSA-397795	G-protein beta:gamma signalling
R-HSA-5675482	Regulation of necroptotic cell death
R-HSA-425561	Sodium/Calcium exchangers
R-HSA-8856828	Clathrin-mediated endocytosis
R-HSA-5260271	Diseases of Immune System
R-HSA-5602358	Diseases associated with the TLR signaling cascade
R-HSA-392518	Signal amplification
R-HSA-5213460	RIPK1-mediated regulated necrosis
R-HSA-9013406	RHOQ GTPase cycle
R-HSA-3000171	Non-integrin membrane-ECM interactions
R-HSA-162592	Integration of provirus
R-HSA-9013404	RAC2 GTPase cycle
R-HSA-2565942	Regulation of PLK1 Activity at G2/M Transition
R-HSA-418346	Platelet homeostasis
R-HSA-9612973	Autophagy
R-HSA-432720	Lysosome Vesicle Biogenesis
R-HSA-163841	Gamma carboxylation, hypusinylation, hydroxylation, and arylsulfatase activa
R-HSA-5663213	RHO GTPases Activate WASPs and WAVEs
R-HSA-190861	Gap junction assembly
R-HSA-5673000	RAF activation
R-HSA-199977	ER to Golgi Anterograde Transport
R-HSA-9664407	Parasite infection
R-HSA-9664417	Leishmania phagocytosis
R-HSA-9664422	FCGR3A-mediated phagocytosis
R-HSA-71336	Pentose phosphate pathway
R-HSA-2132295	MHC class II antigen presentation
R-HSA-70326	Glucose metabolism
R-HSA-9828642	Respiratory syncytial virus genome transcription
R-HSA-166020	Transfer of LPS from LBP carrier to CD14
R-HSA-163754	Insulin effects increased synthesis of Xylulose-5-Phosphate
R-HSA-5210891	Uptake and function of anthrax toxins
R-HSA-399954	Sema3A PAK dependent Axon repulsion
R-HSA-202131	Metabolism of nitric oxide: NOS3 activation and regulation
R-HSA-9651496	Defects of contact activation system (CAS) and kallikrein/kinin system (KKS)
R-HSA-2173791	TGF-beta receptor signaling in EMT (epithelial to mesenchymal transition)
R-HSA-9013407	RHOH GTPase cycle
R-HSA-9013424	RHOV GTPase cycle
R-HSA-8936459	RUNX1 regulates genes involved in megakaryocyte differentiation and platel
R-HSA-9755511	KEAP1-NFE2L2 pathway
R-HSA-8873719	RAB geranylgeranylation
R-HSA-140877	Formation of Fibrin Clot (Clotting Cascade)
R-HSA-5620912	Anchoring of the basal body to the plasma membrane
R-HSA-9609736	Assembly and cell surface presentation of NMDA receptors
R-HSA-3371453	Regulation of HSF1-mediated heat shock response
R-HSA-9662360	Sensory processing of sound by inner hair cells of the cochlea
R-HSA-2022377	Metabolism of Angiotensinogen to Angiotensins
R-HSA-9658195	Leishmania infection

R-HSA-9824443	Parasitic Infection Pathways
R-HSA-2173782	Binding and Uptake of Ligands by Scavenger Receptors
R-HSA-9013026	RHOB GTPase cycle
R-HSA-68877	Mitotic Prometaphase
R-HSA-9671793	Diseases of hemostasis
R-HSA-9818027	NFE2L2 regulating anti-oxidant/detoxification enzymes
R-HSA-9820965	Respiratory syncytial virus (RSV) genome replication, transcription and transla
R-HSA-1632852	Macroautophagy
R-HSA-199992	trans-Golgi Network Vesicle Budding
R-HSA-162909	Host Interactions of HIV factors
R-HSA-434313	Intracellular metabolism of fatty acids regulates insulin secretion
R-HSA-5653890	Lactose synthesis
R-HSA-9820962	Assembly and release of respiratory syncytial virus (RSV) virions
R-HSA-9629569	Protein hydroxylation
R-HSA-2028269	Signaling by Hippo
R-HSA-8964616	G beta:gamma signalling through CDC42
R-HSA-6804115	TP53 regulates transcription of additional cell cycle genes whose exact role ir
R-HSA-9634597	GPER1 signaling
R-HSA-8849932	Synaptic adhesion-like molecules
R-HSA-8978934	Metabolism of cofactors
R-HSA-162594	Early Phase of HIV Life Cycle
R-HSA-189200	Cellular hexose transport
R-HSA-9645723	Diseases of programmed cell death
R-HSA-9659379	Sensory processing of sound
R-HSA-9009391	Extra-nuclear estrogen signaling
R-HSA-72766	Translation
R-HSA-195721	Signaling by WNT
R-HSA-164938	Nef-mediates down modulation of cell surface receptors by recruiting them t
R-HSA-392170	ADP signalling through P2Y purinoceptor 12
R-HSA-68886	M Phase
R-HSA-8980692	RHOA GTPase cycle
R-HSA-5610787	Hedgehog 'off' state
R-HSA-438064	Post NMDA receptor activation events
R-HSA-8949215	Mitochondrial calcium ion transport
R-HSA-140837	Intrinsic Pathway of Fibrin Clot Formation
R-HSA-71387	Metabolism of carbohydrates
R-HSA-9652282	Drug-mediated inhibition of ERBB2 signaling
R-HSA-9665230	Drug resistance in ERBB2 KD mutants
R-HSA-9665233	Resistance of ERBB2 KD mutants to trastuzumab
R-HSA-9665244	Resistance of ERBB2 KD mutants to sapitinib
R-HSA-9665245	Resistance of ERBB2 KD mutants to tesevatinib
R-HSA-9665246	Resistance of ERBB2 KD mutants to neratinib
R-HSA-9665247	Resistance of ERBB2 KD mutants to osimertinib
R-HSA-9665249	Resistance of ERBB2 KD mutants to afatinib
R-HSA-9665250	Resistance of ERBB2 KD mutants to AEE788
R-HSA-9665251	Resistance of ERBB2 KD mutants to lapatinib
R-HSA-9665737	Drug resistance in ERBB2 TMD/JMD mutants
R-HSA-204626	Hypusine synthesis from eIF5A-lysine
R-HSA-70370	Galactose catabolism
R-HSA-2408550	Metabolism of ingested H2SeO4 and H2SeO3 into H2Se

R-HSA-9834752	Respiratory syncytial virus genome replication
R-HSA-209563	Axonal growth stimulation
R-HSA-8985586	SLIT2:ROBO1 increases RHOA activity
R-HSA-948021	Transport to the Golgi and subsequent modification
R-HSA-983169	Class I MHC mediated antigen processing & presentation
R-HSA-9013694	Signaling by NOTCH4
R-HSA-9662851	Anti-inflammatory response favouring Leishmania parasite infection
R-HSA-9664433	Leishmania parasite growth and survival
R-HSA-6811436	COPI-independent Golgi-to-ER retrograde traffic
R-HSA-3928665	EPH-ephrin mediated repulsion of cells
R-HSA-428930	Thromboxane signalling through TP receptor
R-HSA-202040	G-protein activation
R-HSA-1168372	Downstream signaling events of B Cell Receptor (BCR)
R-HSA-392451	G beta:gamma signalling through PI3Kgamma
R-HSA-418592	ADP signalling through P2Y purinoceptor 1
R-HSA-9604323	Negative regulation of NOTCH4 signaling
R-HSA-5620924	Intraflagellar transport
R-HSA-2855086	Ficolins bind to repetitive carbohydrate structures on the target cell surface
R-HSA-1300645	Acrosome Reaction and Sperm:Oocyte Membrane Binding
R-HSA-1483248	Synthesis of PIPs at the ER membrane
R-HSA-9683686	Maturation of spike protein
R-HSA-8875513	MET interacts with TNS proteins
R-HSA-432722	Golgi Associated Vesicle Biogenesis
R-HSA-5578775	Ion homeostasis
R-HSA-112040	G-protein mediated events
R-HSA-9007101	Rab regulation of trafficking
R-HSA-936837	Ion transport by P-type ATPases
R-HSA-8876198	RAB GEFs exchange GTP for GDP on RABs
R-HSA-400042	Adrenaline,noradrenaline inhibits insulin secretion
R-HSA-1855204	Synthesis of IP3 and IP4 in the cytosol
R-HSA-166658	Complement cascade
R-HSA-3700989	Transcriptional Regulation by TP53
R-HSA-9705671	SARS-CoV-2 activates/modulates innate and adaptive immune responses
R-HSA-111885	Opioid Signalling
R-HSA-164952	The role of Nef in HIV-1 replication and disease pathogenesis
R-HSA-983189	Kinesins
R-HSA-3000484	Scavenging by Class F Receptors
R-HSA-1566977	Fibronectin matrix formation
R-HSA-162589	Reverse Transcription of HIV RNA
R-HSA-164516	Minus-strand DNA synthesis
R-HSA-164525	Plus-strand DNA synthesis
R-HSA-205025	NADE modulates death signalling
R-HSA-3000471	Scavenging by Class B Receptors
R-HSA-192905	vRNP Assembly
R-HSA-9022535	Loss of phosphorylation of MECP2 at T308
R-HSA-111957	Cam-PDE 1 activation
R-HSA-442755	Activation of NMDA receptors and postsynaptic events
R-HSA-9013423	RAC3 GTPase cycle
R-HSA-170834	Signaling by TGF-beta Receptor Complex
R-HSA-3000157	Laminin interactions

R-HSA-5218859	Regulated Necrosis
R-HSA-8949613	Cristae formation
R-HSA-70268	Pyruvate metabolism
R-HSA-69620	Cell Cycle Checkpoints
R-HSA-71291	Metabolism of amino acids and derivatives
R-HSA-9833576	CDH11 homotypic and heterotypic interactions
R-HSA-2660825	Signaling by NOTCH1 t(7;9)(NOTCH1:M1580_K2555) Translocation Mutant
R-HSA-2660826	Constitutive Signaling by NOTCH1 t(7;9)(NOTCH1:M1580_K2555) Translocati
R-HSA-447043	Neurofascin interactions
R-HSA-170984	ARMS-mediated activation
R-HSA-9764302	Regulation of CDH19 Expression and Function
R-HSA-162585	Uncoating of the HIV Virion
R-HSA-3134963	DEx/H-box helicases activate type I IFN and inflammatory cytokines productio
R-HSA-447038	NrCAM interactions
R-HSA-168316	Assembly of Viral Components at the Budding Site
R-HSA-456926	Thrombin signalling through proteinase activated receptors (PARs)
R-HSA-451326	Activation of kainate receptors upon glutamate binding
R-HSA-6811434	COPI-dependent Golgi-to-ER retrograde traffic
R-HSA-9614085	FOXO-mediated transcription
R-HSA-1268020	Mitochondrial protein import
R-HSA-2871809	FCERI mediated Ca+2 mobilization
R-HSA-5689603	UCH proteinases
R-HSA-416550	Sema4D mediated inhibition of cell attachment and migration
R-HSA-166662	Lectin pathway of complement activation
R-HSA-5358493	Synthesis of diphthamide-EEF2
R-HSA-9660537	Signaling by MRAS-complex mutants
R-HSA-9726840	SHOC2 M1731 mutant abolishes MRAS complex function
R-HSA-9726842	Gain-of-function MRAS complexes activate RAF signaling
R-HSA-434316	Fatty Acids bound to GPR40 (FFAR1) regulate insulin secretion
R-HSA-428543	Inactivation of CDC42 and RAC1
R-HSA-77111	Synthesis of Ketone Bodies
R-HSA-168330	Viral RNP Complexes in the Host Cell Nucleus
R-HSA-9615710	Late endosomal microautophagy
R-HSA-446203	Asparagine N-linked glycosylation
R-HSA-8939211	ESR-mediated signaling
R-HSA-1222556	ROS and RNS production in phagocytes
R-HSA-390522	Striated Muscle Contraction
R-HSA-5621481	C-type lectin receptors (CLRs)
R-HSA-8856825	Cargo recognition for clathrin-mediated endocytosis
R-HSA-9613354	Lipophagy
R-HSA-9619229	Activation of RAC1 downstream of NMDARs
R-HSA-9662834	CD163 mediating an anti-inflammatory response
R-HSA-5666185	RHO GTPases Activate Rhotekin and Rhophilins
R-HSA-198203	PI3K/AKT activation
R-HSA-2129379	Molecules associated with elastic fibres
R-HSA-112315	Transmission across Chemical Synapses
R-HSA-8864260	Transcriptional regulation by the AP-2 (TFAP2) family of transcription factors
R-HSA-5358351	Signaling by Hedgehog
R-HSA-606279	Deposition of new CENPA-containing nucleosomes at the centromere
R-HSA-774815	Nucleosome assembly

R-HSA-5654738	Signaling by FGFR2
R-HSA-2467813	Separation of Sister Chromatids
R-HSA-9635486	Infection with Mycobacterium tuberculosis
R-HSA-442729	CREB1 phosphorylation through the activation of CaMKII/CaMKK/CaMKIV cas
R-HSA-180689	APOBEC3G mediated resistance to HIV-1 infection
R-HSA-399997	Acetylcholine regulates insulin secretion
R-HSA-8983711	OAS antiviral response
R-HSA-74182	Ketone body metabolism
R-HSA-1222449	Mtb iron assimilation by chelation
R-HSA-9013408	RHOG GTPase cycle
R-HSA-983705	Signaling by the B Cell Receptor (BCR)
R-HSA-2995410	Nuclear Envelope (NE) Reassembly
R-HSA-9013420	RHOU GTPase cycle
R-HSA-157118	Signaling by NOTCH
R-HSA-196854	Metabolism of vitamins and cofactors
R-HSA-597592	Post-translational protein modification
R-HSA-8878171	Transcriptional regulation by RUNX1
R-HSA-3000178	ECM proteoglycans
R-HSA-8875878	MET promotes cell motility
R-HSA-9762292	Regulation of CDH11 function
R-HSA-9020956	Interleukin-27 signaling
R-HSA-73817	Purine ribonucleoside monophosphate biosynthesis
R-HSA-9634285	Constitutive Signaling by Overexpressed ERBB2
R-HSA-168275	Entry of Influenza Virion into Host Cell via Endocytosis
R-HSA-173107	Binding and entry of HIV virion
R-HSA-8875555	MET activates RAP1 and RAC1
R-HSA-168268	Virus Assembly and Release
R-HSA-162906	HIV Infection
R-HSA-9696264	RND3 GTPase cycle
R-HSA-9696273	RND1 GTPase cycle
R-HSA-9648025	EML4 and NUDC in mitotic spindle formation
R-HSA-6806834	Signaling by MET
R-HSA-9660821	ADORA2B mediated anti-inflammatory cytokines production
R-HSA-977444	GABA B receptor activation
R-HSA-991365	Activation of GABAB receptors
R-HSA-9696270	RND2 GTPase cycle
R-HSA-8984722	Interleukin-35 Signalling
R-HSA-111932	CaMK IV-mediated phosphorylation of CREB
R-HSA-170968	Frs2-mediated activation
R-HSA-164843	2-LTR circle formation
R-HSA-264870	Caspase-mediated cleavage of cytoskeletal proteins
R-HSA-9700645	ALK mutants bind TKIs
R-HSA-71384	Ethanol oxidation
R-HSA-451306	Ionotropic activity of kainate receptors
R-HSA-451308	Activation of Ca-permeable Kainate Receptor
R-HSA-389359	CD28 dependent Vav1 pathway
R-HSA-6811442	Intra-Golgi and retrograde Golgi-to-ER traffic
R-HSA-202403	TCR signaling
R-HSA-1640170	Cell Cycle
R-HSA-1566948	Elastic fibre formation

R-HSA-2871837	FCERI mediated NF-kB activation
R-HSA-2172127	DAP12 interactions
R-HSA-112314	Neurotransmitter receptors and postsynaptic signal transmission
R-HSA-5607763	CLEC7A (Dectin-1) induces NFAT activation
R-HSA-3270619	IRF3-mediated induction of type I IFN
R-HSA-5140745	WNT5A-dependent internalization of FZD2, FZD5 and ROR2
R-HSA-2168880	Scavenging of heme from plasma
R-HSA-75205	Dissolution of Fibrin Clot
R-HSA-9820952	Respiratory Syncytial Virus Infection Pathway
R-HSA-9637690	Response of Mtb to phagocytosis
R-HSA-9006936	Signaling by TGFB family members
R-HSA-977606	Regulation of Complement cascade
R-HSA-2173789	TGF-beta receptor signaling activates SMADs
R-HSA-2500257	Resolution of Sister Chromatid Cohesion
R-HSA-983231	Factors involved in megakaryocyte development and platelet production
R-HSA-177504	Retrograde neurotrophin signalling
R-HSA-399955	SEMA3A-Plexin repulsion signaling by inhibiting Integrin adhesion
R-HSA-166786	Creation of C4 and C2 activators
R-HSA-8956320	Nucleotide biosynthesis
R-HSA-170670	Adenylate cyclase inhibitory pathway
R-HSA-442720	CREB1 phosphorylation through the activation of Adenylate Cyclase
R-HSA-169893	Prolonged ERK activation events
R-HSA-189451	Heme biosynthesis
R-HSA-8849471	PTK6 Regulates RHO GTPases, RAS GTPase and MAP kinases
R-HSA-418885	DCC mediated attractive signaling
R-HSA-9005891	Loss of function of MECP2 in Rett syndrome
R-HSA-9005895	Pervasive developmental disorders
R-HSA-9675151	Disorders of Developmental Biology
R-HSA-9697154	Disorders of Nervous System Development
R-HSA-162658	Golgi Cisternae Pericentriolar Stack Reorganization
R-HSA-9637628	Modulation by Mtb of host immune system
R-HSA-6791312	TP53 Regulates Transcription of Cell Cycle Genes
R-HSA-418597	G alpha (z) signalling events
R-HSA-512988	Interleukin-3, Interleukin-5 and GM-CSF signaling
R-HSA-1236978	Cross-presentation of soluble exogenous antigens (endosomes)
R-HSA-421270	Cell-cell junction organization
R-HSA-8939236	RUNX1 regulates transcription of genes involved in differentiation of HSCs
R-HSA-9706369	Negative regulation of FLT3
R-HSA-5609975	Diseases associated with glycosylation precursor biosynthesis
R-HSA-2691230	Signaling by NOTCH1 HD Domain Mutants in Cancer
R-HSA-2691232	Constitutive Signaling by NOTCH1 HD Domain Mutants
R-HSA-6785631	ERBB2 Regulates Cell Motility
R-HSA-2162123	Synthesis of Prostaglandins (PG) and Thromboxanes (TX)
R-HSA-5637810	Constitutive Signaling by EGFRvIII
R-HSA-5637812	Signaling by EGFRvIII in Cancer
R-HSA-5099900	WNT5A-dependent internalization of FZD4
R-HSA-5687128	MAPK6/MAPK4 signaling
R-HSA-190236	Signaling by FGFR
R-HSA-1227986	Signaling by ERBB2
R-HSA-211733	Regulation of activated PAK-2p34 by proteasome mediated degradation

R-HSA-1483249	Inositol phosphate metabolism
R-HSA-5250924	B-WICH complex positively regulates rRNA expression
R-HSA-350562	Regulation of ornithine decarboxylase (ODC)
R-HSA-171306	Packaging Of Telomere Ends
R-HSA-112310	Neurotransmitter release cycle
R-HSA-112043	PLC beta mediated events
R-HSA-8856688	Golgi-to-ER retrograde transport
R-HSA-8866427	VLDLR internalisation and degradation
R-HSA-391160	Signal regulatory protein family interactions
R-HSA-8949664	Processing of SMDT1
R-HSA-9665348	Signaling by ERBB2 ECD mutants
R-HSA-1834941	STING mediated induction of host immune responses
R-HSA-70221	Glycogen breakdown (glycogenolysis)
R-HSA-2730905	Role of LAT2/NTAL/LAB on calcium mobilization
R-HSA-75815	Ubiquitin-dependent degradation of Cyclin D
R-HSA-349425	Autodegradation of the E3 ubiquitin ligase COP1
R-HSA-69601	Ubiquitin Mediated Degradation of Phosphorylated Cdc25A
R-HSA-69610	p53-Independent DNA Damage Response
R-HSA-69613	p53-Independent G1/S DNA damage checkpoint
R-HSA-9762114	GSK3B and BTRC:CUL1-mediated-degradation of NFE2L2
R-HSA-2454202	Fc epsilon receptor (FCERI) signaling
R-HSA-169911	Regulation of Apoptosis
R-HSA-180534	Vpu mediated degradation of CD4
R-HSA-177929	Signaling by EGFR
R-HSA-1810476	RIP-mediated NFkB activation via ZBP1
R-HSA-450385	Butyrate Response Factor 1 (BRF1) binds and destabilizes mRNA
R-HSA-450513	Tristetraprolin (TTP, ZFP36) binds and destabilizes mRNA
R-HSA-450604	KSRP (KHSRP) binds and destabilizes mRNA
R-HSA-9022699	MECP2 regulates neuronal receptors and channels
R-HSA-432142	Platelet sensitization by LDL
R-HSA-1592230	Mitochondrial biogenesis
R-HSA-877300	Interferon gamma signaling
R-HSA-174113	SCF-beta-TrCP mediated degradation of Emi1
R-HSA-180585	Vif-mediated degradation of APOBEC3G
R-HSA-4641257	Degradation of AXIN
R-HSA-8854050	FBXL7 down-regulates AURKA during mitotic entry and in early mitosis
R-HSA-8941858	Regulation of RUNX3 expression and activity
R-HSA-9824272	Somitogenesis
R-HSA-110330	Recognition and association of DNA glycosylase with site containing an affect
R-HSA-110331	Cleavage of the damaged purine
R-HSA-73927	Depurination
R-HSA-71406	Pyruvate metabolism and Citric Acid (TCA) cycle
R-HSA-112316	Neuronal System
R-HSA-1912420	Pre-NOTCH Processing in Golgi
R-HSA-163210	Formation of ATP by chemiosmotic coupling
R-HSA-8964315	G beta:gamma signalling through BTK
R-HSA-1660514	Synthesis of PIPs at the Golgi membrane
R-HSA-193704	p75 NTR receptor-mediated signalling
R-HSA-5362768	Hh mutants are degraded by ERAD
R-HSA-2151201	Transcriptional activation of mitochondrial biogenesis

R-HSA-68867	Assembly of the pre-replicative complex
R-HSA-3214847	HATs acetylate histones
R-HSA-202424	Downstream TCR signaling
R-HSA-9837999	Mitochondrial protein degradation
R-HSA-418990	Adherens junctions interactions
R-HSA-4641258	Degradation of DVL
R-HSA-69541	Stabilization of p53
R-HSA-9679191	Potential therapeutics for SARS
R-HSA-416700	Other semaphorin interactions
R-HSA-9703465	Signaling by FLT3 fusion proteins
R-HSA-8964038	LDL clearance
R-HSA-392851	Prostacyclin signalling through prostacyclin receptor
R-HSA-888590	GABA synthesis, release, reuptake and degradation
R-HSA-1236382	Constitutive Signaling by Ligand-Responsive EGFR Cancer Variants
R-HSA-5637815	Signaling by Ligand-Responsive EGFR Variants in Cancer
R-HSA-5607764	CLEC7A (Dectin-1) signaling
R-HSA-69278	Cell Cycle, Mitotic
R-HSA-68882	Mitotic Anaphase
R-HSA-9609690	HCMV Early Events
R-HSA-351202	Metabolism of polyamines
R-HSA-5387390	Hh mutants abrogate ligand secretion
R-HSA-5676590	NIK-->noncanonical NF-kB signaling
R-HSA-8866654	E3 ubiquitin ligases ubiquitinate target proteins
R-HSA-418217	G beta:gamma signalling through PLC beta
R-HSA-163615	PKA activation
R-HSA-2555396	Mitotic Metaphase and Anaphase
R-HSA-187577	SCF(Skp2)-mediated degradation of p27/p21
R-HSA-5607761	Dectin-1 mediated noncanonical NF-kB signaling
R-HSA-5610780	Degradation of GLI1 by the proteasome
R-HSA-5610783	Degradation of GLI2 by the proteasome
R-HSA-5610785	GLI3 is processed to GLI3R by the proteasome
R-HSA-110328	Recognition and association of DNA glycosylase with site containing an affect
R-HSA-110329	Cleavage of the damaged pyrimidine
R-HSA-73928	Depyrimidination
R-HSA-977443	GABA receptor activation
R-HSA-166016	Toll Like Receptor 4 (TLR4) Cascade
R-HSA-5678895	Defective CFTR causes cystic fibrosis
R-HSA-2022090	Assembly of collagen fibrils and other multimeric structures
R-HSA-6807004	Negative regulation of MET activity
R-HSA-500657	Presynaptic function of Kainate receptors
R-HSA-9830674	Formation of the ureteric bud
R-HSA-5218921	VEGFR2 mediated cell proliferation
R-HSA-1606322	ZBP1(DAI) mediated induction of type I IFNs
R-HSA-5621575	CD209 (DC-SIGN) signaling
R-HSA-442982	Ras activation upon Ca2+ influx through NMDA receptor
R-HSA-9018519	Estrogen-dependent gene expression
R-HSA-73728	RNA Polymerase I Promoter Opening
R-HSA-73929	Base-Excision Repair, AP Site Formation
R-HSA-4086398	Ca2+ pathway
R-HSA-8986944	Transcriptional Regulation by MECP2

R-HSA-211945	Phase I - Functionalization of compounds
R-HSA-5250913	Positive epigenetic regulation of rRNA expression
R-HSA-2979096	NOTCH2 Activation and Transmission of Signal to the Nucleus
R-HSA-140875	Common Pathway of Fibrin Clot Formation
R-HSA-111931	PKA-mediated phosphorylation of CREB
R-HSA-9665686	Signaling by ERBB2 TMD/JMD mutants
R-HSA-202427	Phosphorylation of CD3 and TCR zeta chains
R-HSA-438066	Unblocking of NMDA receptors, glutamate binding and activation
R-HSA-9617324	Negative regulation of NMDA receptor-mediated neuronal transmission
R-HSA-174084	Autodegradation of Cdh1 by Cdh1:APC/C
R-HSA-1912422	Pre-NOTCH Expression and Processing
R-HSA-4608870	Asymmetric localization of PCP proteins
R-HSA-1442490	Collagen degradation
R-HSA-5334118	DNA methylation
R-HSA-1834949	Cytosolic sensors of pathogen-associated DNA
R-HSA-166663	Initial triggering of complement
R-HSA-175474	Assembly Of The HIV Virion
R-HSA-373753	Nephrin family interactions
R-HSA-2672351	Stimuli-sensing channels
R-HSA-5358346	Hedgehog ligand biogenesis
R-HSA-9013148	CDC42 GTPase cycle
R-HSA-73887	Death Receptor Signaling
R-HSA-69563	p53-Dependent G1 DNA Damage Response
R-HSA-69580	p53-Dependent G1/S DNA damage checkpoint
R-HSA-1234176	Oxygen-dependent proline hydroxylation of Hypoxia-inducible Factor Alpha
R-HSA-5625886	Activated PKN1 stimulates transcription of AR (androgen receptor) regulated
R-HSA-5688426	Deubiquitination
R-HSA-9820960	Respiratory syncytial virus (RSV) attachment and entry
R-HSA-166208	mTORC1-mediated signalling
R-HSA-210500	Glutamate Neurotransmitter Release Cycle
R-HSA-429947	Deadenylation of mRNA
R-HSA-9820841	M-decay: degradation of maternal mRNAs by maternally stored factors
R-HSA-9620244	Long-term potentiation
R-HSA-9006931	Signaling by Nuclear Receptors
R-HSA-69002	DNA Replication Pre-Initiation
R-HSA-1169091	Activation of NF-kappaB in B cells
R-HSA-174154	APC/C:Cdc20 mediated degradation of Securin
R-HSA-427359	SIRT1 negatively regulates rRNA expression
R-HSA-9670095	Inhibition of DNA recombination at telomere
R-HSA-5693571	Nonhomologous End-Joining (NHEJ)
R-HSA-204005	COPII-mediated vesicle transport
R-HSA-5658442	Regulation of RAS by GAPs
R-HSA-69615	G1/S DNA Damage Checkpoints
R-HSA-68616	Assembly of the ORC complex at the origin of replication
R-HSA-9013507	NOTCH3 Activation and Transmission of Signal to the Nucleus
R-HSA-9664565	Signaling by ERBB2 KD Mutants
R-HSA-1187000	Fertilization
R-HSA-1643713	Signaling by EGFR in Cancer
R-HSA-166058	MyD88:MAL(TIRAP) cascade initiated on plasma membrane
R-HSA-168188	Toll Like Receptor TLR6:TLR2 Cascade

R-HSA-8948751	Regulation of PTEN stability and activity
R-HSA-9793380	Formation of paraxial mesoderm
R-HSA-187037	Signaling by NTRK1 (TRKA)
R-HSA-180024	DARPP-32 events
R-HSA-901042	Calnexin/calreticulin cycle
R-HSA-1227990	Signaling by ERBB2 in Cancer
R-HSA-9821002	Chromatin modifications during the maternal to zygotic transition (MZT)
R-HSA-9816359	Maternal to zygotic transition (MZT)
R-HSA-168179	Toll Like Receptor TLR1:TLR2 Cascade
R-HSA-181438	Toll Like Receptor 2 (TLR2) Cascade
R-HSA-68949	Orc1 removal from chromatin
R-HSA-9648002	RAS processing
R-HSA-189445	Metabolism of porphyrins
R-HSA-912526	Interleukin receptor SHC signaling
R-HSA-8982491	Glycogen metabolism
R-HSA-69017	CDK-mediated phosphorylation and removal of Cdc6
R-HSA-174184	Cdc20:Phospho-APC/C mediated degradation of Cyclin A
R-HSA-212300	PRC2 methylates histones and DNA
R-HSA-9710421	Defective pyroptosis
R-HSA-2299718	Condensation of Prophase Chromosomes
R-HSA-174178	APC/C:Cdh1 mediated degradation of Cdc20 and other APC/C:Cdh1 targeted
R-HSA-179419	APC:Cdc20 mediated degradation of cell cycle proteins prior to satisfation of
R-HSA-8939902	Regulation of RUNX2 expression and activity
R-HSA-1482788	Acyl chain remodelling of PC
R-HSA-9759475	Regulation of CDH11 Expression and Function
R-HSA-9682385	FLT3 signaling in disease
R-HSA-168898	Toll-like Receptor Cascades
R-HSA-9753281	Paracetamol ADME
R-HSA-8863795	Downregulation of ERBB2 signaling
R-HSA-5621480	Dectin-2 family
R-HSA-1296041	Activation of G protein gated Potassium channels
R-HSA-1296059	G protein gated Potassium channels
R-HSA-997272	Inhibition of voltage gated Ca2+ channels via Gbeta/gamma subunits
R-HSA-420029	Tight junction interactions
R-HSA-5601884	PIWI-interacting RNA (piRNA) biogenesis
R-HSA-442742	CREB1 phosphorylation through NMDA receptor-mediated activation of RAS
R-HSA-1234174	Cellular response to hypoxia
R-HSA-176409	APC/C:Cdc20 mediated degradation of mitotic proteins
R-HSA-5689880	Ub-specific processing proteases
R-HSA-176814	Activation of APC/C and APC/C:Cdc20 mediated degradation of mitotic protei
R-HSA-427389	ERCC6 (CSB) and EHMT2 (G9a) positively regulate rRNA expression
R-HSA-525793	Myogenesis
R-HSA-1482839	Acyl chain remodelling of PE
R-HSA-2424491	DAP12 signaling
R-HSA-499943	Interconversion of nucleotide di- and triphosphates
R-HSA-77387	Insulin receptor recycling
R-HSA-8874081	MET activates PTK2 signaling
R-HSA-8866652	Synthesis of active ubiquitin: roles of E1 and E2 enzymes
R-HSA-9683701	Translation of Structural Proteins
R-HSA-909733	Interferon alpha/beta signaling

R-HSA-5619084	ABC transporter disorders
R-HSA-1221632	Meiotic synapsis
R-HSA-9759476	Regulation of Homotypic Cell-Cell Adhesion
R-HSA-9764260	Regulation of Expression and Function of Type II Classical Cadherins
R-HSA-2122948	Activated NOTCH1 Transmits Signal to the Nucleus
R-HSA-182971	EGFR downregulation
R-HSA-917977	Transferrin endocytosis and recycling
R-HSA-9680350	Signaling by CSF1 (M-CSF) in myeloid cells
R-HSA-196849	Metabolism of water-soluble vitamins and cofactors
R-HSA-2559586	DNA Damage/Telomere Stress Induced Senescence
R-HSA-8852135	Protein ubiquitination
R-HSA-2871796	FCERI mediated MAPK activation
R-HSA-983695	Antigen activates B Cell Receptor (BCR) leading to generation of second mess
R-HSA-416482	G alpha (12/13) signalling events
R-HSA-176408	Regulation of APC/C activators between G1/S and early anaphase
R-HSA-389356	CD28 co-stimulation
R-HSA-4085001	Sialic acid metabolism
R-HSA-1980145	Signaling by NOTCH2
R-HSA-5694530	Cargo concentration in the ER
R-HSA-9725371	Nuclear events stimulated by ALK signaling in cancer
R-HSA-420092	Glucagon-type ligand receptors
R-HSA-163359	Glucagon signaling in metabolic regulation
R-HSA-9022692	Regulation of MECP2 expression and activity
R-HSA-1430728	Metabolism
R-HSA-5576891	Cardiac conduction
R-HSA-201681	TCF dependent signaling in response to WNT
R-HSA-69202	Cyclin E associated events during G1/S transition
R-HSA-195253	Degradation of beta-catenin by the destruction complex
R-HSA-192105	Synthesis of bile acids and bile salts
R-HSA-180746	Nuclear import of Rev protein
R-HSA-187687	Signalling to ERKs
R-HSA-5576892	Phase 0 - rapid depolarisation
R-HSA-1483255	PI Metabolism
R-HSA-166520	Signaling by NTRKs
R-HSA-1296065	Inwardly rectifying K+ channels
R-HSA-532668	N-glycan trimming in the ER and Calnexin/Calreticulin cycle
R-HSA-9013149	RAC1 GTPase cycle
R-HSA-69656	Cyclin A:Cdk2-associated events at S phase entry
R-HSA-912446	Meiotic recombination
R-HSA-5632684	Hedgehog 'on' state
R-HSA-983712	Ion channel transport
R-HSA-69306	DNA Replication
R-HSA-6794362	Protein-protein interactions at synapses
R-HSA-162588	Budding and maturation of HIV virion
R-HSA-2514859	Inactivation, recovery and regulation of the phototransduction cascade
R-HSA-156590	Glutathione conjugation
R-HSA-211000	Gene Silencing by RNA
R-HSA-174143	APC/C-mediated degradation of cell cycle proteins
R-HSA-453276	Regulation of mitotic cell cycle
R-HSA-9609646	HCMV Infection

R-HSA-212165	Epigenetic regulation of gene expression
R-HSA-2514856	The phototransduction cascade
R-HSA-8964043	Plasma lipoprotein clearance
R-HSA-5689901	Metalloprotease DUBs
R-HSA-5083635	Defective B3GALTL causes PpS
R-HSA-9694548	Maturation of spike protein
R-HSA-111933	Calmodulin induced events
R-HSA-111997	CaM pathway
R-HSA-177243	Interactions of Rev with host cellular proteins
R-HSA-73886	Chromosome Maintenance
R-HSA-2980736	Peptide hormone metabolism
R-HSA-9616222	Transcriptional regulation of granulopoiesis
R-HSA-9607240	FLT3 Signaling
R-HSA-5696394	DNA Damage Recognition in GG-NER
R-HSA-5173214	O-glycosylation of TSR domain-containing proteins
R-HSA-68875	Mitotic Prophase
R-HSA-73772	RNA Polymerase I Promoter Escape
R-HSA-201722	Formation of the beta-catenin:TCF transactivating complex
R-HSA-1474290	Collagen formation
R-HSA-5689896	Ovarian tumor domain proteases
R-HSA-111996	Ca-dependent events
R-HSA-168274	Export of Viral Ribonucleoproteins from Nucleus
R-HSA-9840310	Glycosphingolipid catabolism
R-HSA-73884	Base Excision Repair
R-HSA-69052	Switching of origins to a post-replicative state
R-HSA-1912408	Pre-NOTCH Transcription and Translation
R-HSA-3214815	HDACs deacetylate histones
R-HSA-165159	MTOR signalling
R-HSA-9772755	Formation of WDR5-containing histone-modifying complexes
R-HSA-9035034	RHOF GTPase cycle
R-HSA-9821993	Replacement of protamines by nucleosomes in the male pronucleus
R-HSA-381676	Glucagon-like Peptide-1 (GLP1) regulates insulin secretion
R-HSA-9692916	SARS-CoV-1 activates/modulates innate immune responses
R-HSA-3769402	Deactivation of the beta-catenin transactivating complex
R-HSA-8878159	Transcriptional regulation by RUNX3
R-HSA-1489509	DAG and IP3 signaling
R-HSA-432040	Vasopressin regulates renal water homeostasis via Aquaporins
R-HSA-9664323	FCGR3A-mediated IL10 synthesis
R-HSA-5675221	Negative regulation of MAPK pathway
R-HSA-975634	Retinoid metabolism and transport
R-HSA-6811438	Intra-Golgi traffic
R-HSA-451927	Interleukin-2 family signaling
R-HSA-15869	Metabolism of nucleotides
R-HSA-194068	Bile acid and bile salt metabolism
R-HSA-3899300	SUMOylation of transcription cofactors
R-HSA-1474165	Reproduction
R-HSA-8854214	TBC/RABGAPs
R-HSA-9830369	Kidney development
R-HSA-5668541	TNFR2 non-canonical NF-kB pathway
R-HSA-382556	ABC-family proteins mediated transport

R-HSA-4570464	SUMOylation of RNA binding proteins
R-HSA-6783783	Interleukin-10 signaling
R-HSA-418555	G alpha (s) signalling events
R-HSA-2187338	Visual phototransduction
R-HSA-6806667	Metabolism of fat-soluble vitamins
R-HSA-5578749	Transcriptional regulation by small RNAs
R-HSA-9012852	Signaling by NOTCH3
R-HSA-6811440	Retrograde transport at the Trans-Golgi-Network
R-HSA-381038	XBP1(S) activates chaperone genes
R-HSA-5357905	Regulation of TNFR1 signaling
R-HSA-373752	Netrin-1 signaling
R-HSA-8957275	Post-translational protein phosphorylation
R-HSA-427413	NoRC negatively regulates rRNA expression
R-HSA-425393	Transport of inorganic cations/anions and amino acids/oligopeptides
R-HSA-9609507	Protein localization
R-HSA-2559582	Senescence-Associated Secretory Phenotype (SASP)
R-HSA-73854	RNA Polymerase I Promoter Clearance
R-HSA-381070	IRE1alpha activates chaperones
R-HSA-445717	Aquaporin-mediated transport
R-HSA-211859	Biological oxidations
R-HSA-5250941	Negative epigenetic regulation of rRNA expression
R-HSA-1660499	Synthesis of PIPs at the plasma membrane
R-HSA-157579	Telomere Maintenance
R-HSA-73864	RNA Polymerase I Transcription
R-HSA-8848021	Signaling by PTK6
R-HSA-9006927	Signaling by Non-Receptor Tyrosine Kinases
R-HSA-429914	Deadenylation-dependent mRNA decay
R-HSA-1222499	Latent infection - Other responses of Mtb to phagocytosis
R-HSA-9020702	Interleukin-1 signaling
R-HSA-1500620	Meiosis
R-HSA-5619115	Disorders of transmembrane transporters
R-HSA-2644602	Signaling by NOTCH1 PEST Domain Mutants in Cancer
R-HSA-2644603	Signaling by NOTCH1 in Cancer
R-HSA-2644606	Constitutive Signaling by NOTCH1 PEST Domain Mutants
R-HSA-2894858	Signaling by NOTCH1 HD+PEST Domain Mutants in Cancer
R-HSA-2894862	Constitutive Signaling by NOTCH1 HD+PEST Domain Mutants
R-HSA-917937	Iron uptake and transport
R-HSA-9694635	Translation of Structural Proteins
R-HSA-1660662	Glycosphingolipid metabolism
R-HSA-186797	Signaling by PDGF
R-HSA-1428517	The citric acid (TCA) cycle and respiratory electron transport
R-HSA-69239	Synthesis of DNA
R-HSA-75893	TNF signaling
R-HSA-380108	Chemokine receptors bind chemokines
R-HSA-2142753	Arachidonic acid metabolism
R-HSA-8878166	Transcriptional regulation by RUNX2
R-HSA-5617472	Activation of anterior HOX genes in hindbrain development during early emb
R-HSA-5619507	Activation of HOX genes during differentiation
R-HSA-382551	Transport of small molecules
R-HSA-381426	Regulation of Insulin-like Growth Factor (IGF) transport and uptake by Insulin

R-HSA-446219	Synthesis of substrates in N-glycan biosythesis
R-HSA-375165	NCAM signaling for neurite out-growth
R-HSA-9758941	Gastrulation
R-HSA-2559580	Oxidative Stress Induced Senescence
R-HSA-425407	SLC-mediated transmembrane transport
R-HSA-69206	G1/S Transition
R-HSA-388841	Costimulation by the CD28 family
R-HSA-3906995	Diseases associated with O-glycosylation of proteins
R-HSA-9772573	Late SARS-CoV-2 Infection Events
R-HSA-418594	G alpha (i) signalling events
R-HSA-6807070	PTEN Regulation
R-HSA-1474228	Degradation of the extracellular matrix
R-HSA-1980143	Signaling by NOTCH1
R-HSA-174824	Plasma lipoprotein assembly, remodeling, and clearance
R-HSA-204998	Cell death signalling via NRAGE, NRIF and NADE
R-HSA-3781865	Diseases of glycosylation
R-HSA-5693565	Recruitment and ATM-mediated phosphorylation of repair and signaling prot
R-HSA-3247509	Chromatin modifying enzymes
R-HSA-4839726	Chromatin organization
R-HSA-446193	Biosynthesis of the N-glycan precursor (dolichol lipid-linked oligosaccharide, l
R-HSA-5693606	DNA Double Strand Break Response
R-HSA-3214858	RMTs methylate histone arginines
R-HSA-2219530	Constitutive Signaling by Aberrant PI3K in Cancer
R-HSA-1483257	Phospholipid metabolism
R-HSA-453279	Mitotic G1 phase and G1/S transition
R-HSA-168928	DDX58/IFIH1-mediated induction of interferon-alpha/beta
R-HSA-74752	Signaling by Insulin receptor
R-HSA-446652	Interleukin-1 family signaling
R-HSA-381340	Transcriptional regulation of white adipocyte differentiation
R-HSA-5696399	Global Genome Nucleotide Excision Repair (GG-NER)
R-HSA-162587	HIV Life Cycle
R-HSA-212436	Generic Transcription Pathway
R-HSA-69242	S Phase
R-HSA-5693532	DNA Double-Strand Break Repair
R-HSA-381119	Unfolded Protein Response (UPR)
R-HSA-373080	Class B/2 (Secretin family receptors)
R-HSA-141424	Amplification of signal from the kinetochores
R-HSA-141444	Amplification of signal from unattached kinetochores via a MAD2 inhibitory
R-HSA-5693607	Processing of DNA double-strand break ends
R-HSA-5619102	SLC transporter disorders
R-HSA-9833110	RSV-host interactions
R-HSA-9006925	Intracellular signaling by second messengers
R-HSA-1296071	Potassium Channels
R-HSA-2219528	PI3K/AKT Signaling in Cancer
R-HSA-428157	Sphingolipid metabolism
R-HSA-6811558	PI5P, PP2A and IER3 Regulate PI3K/AKT Signaling
R-HSA-3108232	SUMO E3 ligases SUMOylate target proteins
R-HSA-156580	Phase II - Conjugation of compounds
R-HSA-9748784	Drug ADME
R-HSA-5696398	Nucleotide Excision Repair

R-HSA-2990846	SUMOylation
R-HSA-69618	Mitotic Spindle Checkpoint
R-HSA-5173105	O-linked glycosylation
R-HSA-199418	Negative regulation of the PI3K/AKT network
R-HSA-73894	DNA Repair
R-HSA-1989781	PPARA activates gene expression
R-HSA-1257604	PIP3 activates AKT signaling
R-HSA-2559583	Cellular Senescence
R-HSA-400206	Regulation of lipid metabolism by PPARalpha
R-HSA-9610379	HCMV Late Events
R-HSA-163200	Respiratory electron transport, ATP synthesis by chemiosmotic coupling, and
R-HSA-1483206	Glycerophospholipid biosynthesis
R-HSA-73857	RNA Polymerase II Transcription
R-HSA-74160	Gene expression (Transcription)
R-HSA-5693567	HDR through Homologous Recombination (HRR) or Single Strand Annealing (S
R-HSA-416476	G alpha (q) signalling events
R-HSA-5693538	Homology Directed Repair
R-HSA-388396	GPCR downstream signalling
R-HSA-162599	Late Phase of HIV Life Cycle
R-HSA-983168	Antigen processing: Ubiquitination & Proteasome degradation
R-HSA-8957322	Metabolism of steroids
R-HSA-8951664	Neddylation
R-HSA-5668914	Diseases of metabolism
R-HSA-8978868	Fatty acid metabolism
R-HSA-372790	Signaling by GPCR
R-HSA-375276	Peptide ligand-binding receptors
R-HSA-9709957	Sensory Perception
R-HSA-556833	Metabolism of lipids
R-HSA-373076	Class A/1 (Rhodopsin-like receptors)
R-HSA-500792	GPCR ligand binding

3(+)sEV4

arbitrary category	# all protei	# detected	p.val	FDR	OR
immune-related	480	52	1.94E-24	1.89E-21	7.45
cancer-related	263	37	2.52E-21	2.46E-18	9.41
cancer-related	623	52	3.03E-19	2.96E-16	5.51
cancer-related	129	26	3.13E-19	3.05E-16	13.87
cancer-related	134	26	8.61E-19	8.38E-16	13.22
immune-related	1076	64	3.54E-16	3.44E-13	3.93
immune-related	2068	90	1.69E-14	1.64E-11	3.05
immune-related	38	12	5.15E-12	4.99E-09	23.86
immune-related	57	13	6.41E-11	6.21E-08	15.32
cancer-related	28	10	7.92E-11	7.67E-08	28.48
immune-related	47	12	8.48E-11	8.20E-08	17.71
cancer-related	706	42	1.23E-10	1.19E-07	3.58
cancer-related	10	7	1.32E-10	1.27E-07	118.14
cancer-related	722	42	2.47E-10	2.38E-07	3.49
cancer-related	326	27	3.26E-10	3.14E-07	4.90
cancer-related	32	10	3.63E-10	3.49E-07	23.29
cancer-related	25	9	6.67E-10	6.41E-07	28.71
cancer-related	129	17	6.85E-10	6.57E-07	7.97
other	553	33	1.46E-08	1.40E-05	3.48
cancer-related	72	12	1.61E-08	1.54E-05	10.31
other	578	33	4.25E-08	4.07E-05	3.31
cancer-related	1271	54	4.36E-08	4.17E-05	2.55
other	98	13	6.83E-08	6.52E-05	7.90
other	90	12	2.09E-07	1.99E-04	7.92
cancer-related	47	9	3.03E-07	2.89E-04	12.06
cancer-related	47	9	3.03E-07	2.89E-04	12.06
cancer-related	47	9	3.03E-07	2.89E-04	12.06
cancer-related	47	9	3.03E-07	2.89E-04	12.06
other	96	12	4.28E-07	4.06E-04	7.35
other	2074	72	5.36E-07	5.08E-04	2.12
cancer-related	38	8	6.52E-07	6.18E-04	13.53
other	144	14	1.06E-06	1.01E-03	5.57
cancer-related	215	17	1.39E-06	1.32E-03	4.47
other	1273	50	1.71E-06	1.62E-03	2.29
cancer-related	43	8	1.77E-06	1.67E-03	11.59
other	12	5	2.15E-06	2.02E-03	35.82
cancer-related	94	11	2.54E-06	2.39E-03	6.79
immune-related	792	36	2.61E-06	2.45E-03	2.59
cancer-related	13	5	3.43E-06	3.22E-03	31.34
immune-related	138	13	3.71E-06	3.48E-03	5.35
cancer-related	119	12	4.34E-06	4.07E-03	5.76
cancer-related	36	7	5.88E-06	5.51E-03	12.19
cancer-related	24	6	5.91E-06	5.53E-03	16.78
cancer-related	37	7	7.13E-06	6.66E-03	11.78
cancer-related	15	5	7.75E-06	7.23E-03	25.07
other	15	5	7.75E-06	7.23E-03	25.07
immune-related	473	25	8.39E-06	7.81E-03	2.95

other	90	10	1.17E-05	1.09E-02	6.37
cancer-related	40	7	1.23E-05	1.14E-02	10.71
other	204	15	1.42E-05	1.32E-02	4.10
cancer-related	92	10	1.43E-05	1.32E-02	6.22
other	94	10	1.73E-05	1.60E-02	6.07
other	45	7	2.75E-05	2.54E-02	9.30
cancer-related	32	6	3.48E-05	3.22E-02	11.61
cancer-related	20	5	3.69E-05	3.40E-02	16.71
other	65	8	4.23E-05	3.90E-02	7.10
cancer-related	84	9	4.32E-05	3.98E-02	6.09
cancer-related	11	4	4.58E-05	4.21E-02	28.53
cancer-related	85	9	4.75E-05	4.37E-02	6.01
cancer-related	67	8	5.29E-05	4.85E-02	6.86
immune-related	153	12	5.56E-05	5.10E-02	4.35
cancer-related	87	9	5.72E-05	5.24E-02	5.86
cancer-related	35	6	5.93E-05	5.43E-02	10.40
cancer-related	12	4	6.76E-05	6.18E-02	24.96
cancer-related	23	5	7.62E-05	6.96E-02	13.92
other	417	21	9.19E-05	8.38E-02	2.77
cancer-related	38	6	9.60E-05	8.74E-02	9.43
other	38	6	9.60E-05	8.74E-02	9.43
other	115	10	9.88E-05	8.98E-02	4.84
cancer-related	39	6	1.12E-04	1.01E-01	9.14
cancer-related	217	14	1.15E-04	1.04E-01	3.54
other	985	37	1.26E-04	1.14E-01	2.09
other	168	12	1.37E-04	1.24E-01	3.93
other	6	3	1.50E-04	1.36E-01	49.72
other	6	3	1.50E-04	1.36E-01	49.72
cancer-related	15	4	1.78E-04	1.60E-01	18.15
cancer-related	15	4	1.78E-04	1.60E-01	18.15
cancer-related	16	4	2.33E-04	2.10E-01	16.64
immune-related	29	5	2.44E-04	2.19E-01	10.43
cancer-related	449	21	2.56E-04	2.30E-01	2.55
immune-related	106	9	2.64E-04	2.37E-01	4.70
cancer-related	30	5	2.88E-04	2.58E-01	10.01
other	807	31	3.21E-04	2.87E-01	2.11
other	47	6	3.21E-04	2.87E-01	7.35
other	300	16	3.41E-04	3.04E-01	2.90
cancer-related	18	4	3.80E-04	3.39E-01	14.26
other	8	3	4.09E-04	3.64E-01	29.83
other	673	27	4.11E-04	3.66E-01	2.19
other	821	31	4.32E-04	3.84E-01	2.07
cancer-related	219	13	4.51E-04	4.00E-01	3.22
immune-related	91	8	4.57E-04	4.06E-01	4.87
other	19	4	4.74E-04	4.20E-01	13.30
other	94	8	5.69E-04	5.04E-01	4.70
other	94	8	5.69E-04	5.04E-01	4.70
cancer-related	20	4	5.83E-04	5.15E-01	12.47
other	9	3	6.04E-04	5.33E-01	24.85
other	119	9	6.21E-04	5.47E-01	4.14

other	96	8	6.55E-04	5.77E-01	4.59
immune-related	770	29	6.98E-04	6.13E-01	2.06
immune-related	21	4	7.10E-04	6.23E-01	11.74
cancer-related	21	4	7.10E-04	6.23E-01	11.74
other	55	6	7.60E-04	6.66E-01	6.15
cancer-related	99	8	8.04E-04	7.03E-01	4.44
other	22	4	8.54E-04	7.46E-01	11.08
cancer-related	458	20	8.54E-04	7.46E-01	2.36
other	38	5	8.91E-04	7.77E-01	7.58
other	207	12	9.16E-04	7.98E-01	3.13
other	101	8	9.17E-04	7.98E-01	4.34
other	102	8	9.78E-04	8.50E-01	4.29
other	102	8	9.78E-04	8.50E-01	4.29
cancer-related	79	7	9.88E-04	8.57E-01	4.89
cancer-related	23	4	1.02E-03	8.81E-01	10.50
other	23	4	1.02E-03	8.81E-01	10.50
cancer-related	182	11	1.06E-03	9.17E-01	3.27
cancer-related	11	3	1.15E-03	9.94E-01	18.64
other	11	3	1.15E-03	9.94E-01	18.64
cancer-related	24	4	1.20E-03	1.00E+00	9.97
other	215	12	1.27E-03	1.00E+00	3.01
cancer-related	61	6	1.32E-03	1.00E+00	5.47
other	729	27	1.38E-03	1.00E+00	2.01
cancer-related	25	4	1.41E-03	1.00E+00	9.50
cancer-related	25	4	1.41E-03	1.00E+00	9.50
immune-related	42	5	1.42E-03	1.00E+00	6.76
cancer-related	108	8	1.42E-03	1.00E+00	4.03
cancer-related	12	3	1.51E-03	1.00E+00	16.57
immune-related	12	3	1.51E-03	1.00E+00	16.57
other	43	5	1.58E-03	1.00E+00	6.58
immune-related	112	8	1.79E-03	1.00E+00	3.88
other	113	8	1.89E-03	1.00E+00	3.84
other	113	8	1.89E-03	1.00E+00	3.84
cancer-related	27	4	1.89E-03	1.00E+00	8.67
cancer-related	27	4	1.89E-03	1.00E+00	8.67
other	634	24	1.92E-03	1.00E+00	2.04
cancer-related	140	9	1.96E-03	1.00E+00	3.47
other	116	8	2.23E-03	1.00E+00	3.73
other	116	8	2.23E-03	1.00E+00	3.73
other	4	2	2.32E-03	1.00E+00	49.50
cancer-related	4	2	2.32E-03	1.00E+00	49.50
other	201	11	2.34E-03	1.00E+00	2.94
other	47	5	2.35E-03	1.00E+00	5.95
other	172	10	2.37E-03	1.00E+00	3.12
cancer-related	14	3	2.43E-03	1.00E+00	13.55
other	14	3	2.43E-03	1.00E+00	13.55
cancer-related	296	14	2.48E-03	1.00E+00	2.53
other	29	4	2.48E-03	1.00E+00	7.98
other	94	7	2.71E-03	1.00E+00	4.04
other	147	9	2.74E-03	1.00E+00	3.29

other	120	8	2.76E-03	1.00E+00	3.60
other	120	8	2.76E-03	1.00E+00	3.60
cancer-related	30	4	2.82E-03	1.00E+00	7.67
other	49	5	2.83E-03	1.00E+00	5.68
other	51	5	3.38E-03	1.00E+00	5.43
immune-related	16	3	3.63E-03	1.00E+00	11.46
other	52	5	3.68E-03	1.00E+00	5.32
other	5	2	3.82E-03	1.00E+00	33.00
cancer-related	215	11	3.93E-03	1.00E+00	2.73
other	101	7	4.05E-03	1.00E+00	3.74
cancer-related	77	6	4.32E-03	1.00E+00	4.23
cancer-related	55	5	4.70E-03	1.00E+00	5.00
other	35	4	4.99E-03	1.00E+00	6.43
cancer-related	35	4	4.99E-03	1.00E+00	6.43
immune-related	18	3	5.14E-03	1.00E+00	9.93
other	18	3	5.14E-03	1.00E+00	9.93
other	81	6	5.54E-03	1.00E+00	4.01
other	6	2	5.65E-03	1.00E+00	24.75
cancer-related	2077	57	5.85E-03	1.00E+00	1.51
immune-related	108	7	5.85E-03	1.00E+00	3.48
immune-related	19	3	6.01E-03	1.00E+00	9.31
other	19	3	6.01E-03	1.00E+00	9.31
other	166	9	6.08E-03	1.00E+00	2.89
other	59	5	6.34E-03	1.00E+00	4.62
other	59	5	6.34E-03	1.00E+00	4.62
other	110	7	6.46E-03	1.00E+00	3.41
cancer-related	38	4	6.72E-03	1.00E+00	5.86
other	60	5	6.80E-03	1.00E+00	4.54
cancer-related	86	6	7.40E-03	1.00E+00	3.75
other	7	2	7.80E-03	1.00E+00	19.80
other	7	2	7.80E-03	1.00E+00	19.80
cancer-related	21	3	8.01E-03	1.00E+00	8.28
other	205	10	8.11E-03	1.00E+00	2.59
other	88	6	8.25E-03	1.00E+00	3.66
cancer-related	89	6	8.71E-03	1.00E+00	3.62
cancer-related	146	8	8.93E-03	1.00E+00	2.91
other	22	3	9.14E-03	1.00E+00	7.84
other	22	3	9.14E-03	1.00E+00	7.84
other	22	3	9.14E-03	1.00E+00	7.84
cancer-related	42	4	9.57E-03	1.00E+00	5.24
cancer-related	91	6	9.67E-03	1.00E+00	3.53
cancer-related	91	6	9.67E-03	1.00E+00	3.53
cancer-related	92	6	1.02E-02	1.00E+00	3.49
cancer-related	532	19	1.02E-02	1.00E+00	1.89
other	8	2	1.03E-02	1.00E+00	16.50
other	8	2	1.03E-02	1.00E+00	16.50
immune-related	8	2	1.03E-02	1.00E+00	16.50
cancer-related	280	12	1.05E-02	1.00E+00	2.26
cancer-related	94	6	1.12E-02	1.00E+00	3.41
cancer-related	24	3	1.17E-02	1.00E+00	7.09

other	285	12	1.20E-02	1.00E+00	2.22
immune-related	154	8	1.21E-02	1.00E+00	2.75
cancer-related	286	12	1.23E-02	1.00E+00	2.21
other	187	9	1.28E-02	1.00E+00	2.54
other	70	5	1.29E-02	1.00E+00	3.84
other	70	5	1.29E-02	1.00E+00	3.84
cancer-related	97	6	1.30E-02	1.00E+00	3.30
other	9	2	1.30E-02	1.00E+00	14.14
other	9	2	1.30E-02	1.00E+00	14.14
cancer-related	25	3	1.31E-02	1.00E+00	6.77
other	157	8	1.35E-02	1.00E+00	2.69
cancer-related	325	13	1.36E-02	1.00E+00	2.11
other	98	6	1.36E-02	1.00E+00	3.26
cancer-related	26	3	1.46E-02	1.00E+00	6.47
other	73	5	1.52E-02	1.00E+00	3.67
other	73	5	1.52E-02	1.00E+00	3.67
cancer-related	74	5	1.61E-02	1.00E+00	3.61
other	10	2	1.61E-02	1.00E+00	12.37
cancer-related	2599	66	1.67E-02	1.00E+00	1.39
cancer-related	300	12	1.74E-02	1.00E+00	2.10
other	197	9	1.74E-02	1.00E+00	2.41
other	197	9	1.74E-02	1.00E+00	2.41
immune-related	134	7	1.79E-02	1.00E+00	2.76
other	199	9	1.85E-02	1.00E+00	2.38
other	51	4	1.86E-02	1.00E+00	4.23
other	167	8	1.89E-02	1.00E+00	2.52
cancer-related	11	2	1.94E-02	1.00E+00	10.99
other	11	2	1.94E-02	1.00E+00	10.99
cancer-related	11	2	1.94E-02	1.00E+00	10.99
other	11	2	1.94E-02	1.00E+00	10.99
cancer-related	11	2	1.94E-02	1.00E+00	10.99
cancer-related	11	2	1.94E-02	1.00E+00	10.99
immune-related	11	2	1.94E-02	1.00E+00	10.99
cancer-related	29	3	1.96E-02	1.00E+00	5.73
other	78	5	1.97E-02	1.00E+00	3.41
cancer-related	52	4	1.98E-02	1.00E+00	4.15
other	1	1	2.00E-02	1.00E+00	Inf
other	1	1	2.00E-02	1.00E+00	Inf
other	1	1	2.00E-02	1.00E+00	Inf
other	108	6	2.10E-02	1.00E+00	2.94
other	30	3	2.15E-02	1.00E+00	5.51
immune-related	274	11	2.17E-02	1.00E+00	2.11
other	54	4	2.25E-02	1.00E+00	3.98
cancer-related	12	2	2.30E-02	1.00E+00	9.89
other	12	2	2.30E-02	1.00E+00	9.89
other	12	2	2.30E-02	1.00E+00	9.89
other	31	3	2.34E-02	1.00E+00	5.32
cancer-related	31	3	2.34E-02	1.00E+00	5.32
other	208	9	2.38E-02	1.00E+00	2.27
cancer-related	55	4	2.39E-02	1.00E+00	3.90

other	82	5	2.39E-02	1.00E+00	3.24
other	82	5	2.39E-02	1.00E+00	3.24
cancer-related	56	4	2.53E-02	1.00E+00	3.83
cancer-related	32	3	2.55E-02	1.00E+00	5.13
cancer-related	32	3	2.55E-02	1.00E+00	5.13
other	13	2	2.68E-02	1.00E+00	8.99
cancer-related	146	7	2.71E-02	1.00E+00	2.52
immune-related	33	3	2.76E-02	1.00E+00	4.96
immune-related	33	3	2.76E-02	1.00E+00	4.96
other	33	3	2.76E-02	1.00E+00	4.96
cancer-related	34	3	2.98E-02	1.00E+00	4.80
cancer-related	59	4	3.00E-02	1.00E+00	3.62
cancer-related	59	4	3.00E-02	1.00E+00	3.62
other	14	2	3.09E-02	1.00E+00	8.24
cancer-related	88	5	3.13E-02	1.00E+00	3.00
other	88	5	3.13E-02	1.00E+00	3.00
cancer-related	88	5	3.13E-02	1.00E+00	3.00
other	151	7	3.18E-02	1.00E+00	2.43
other	35	3	3.22E-02	1.00E+00	4.65
other	61	4	3.33E-02	1.00E+00	3.49
cancer-related	36	3	3.46E-02	1.00E+00	4.51
other	36	3	3.46E-02	1.00E+00	4.51
cancer-related	36	3	3.46E-02	1.00E+00	4.51
other	154	7	3.48E-02	1.00E+00	2.38
other	62	4	3.51E-02	1.00E+00	3.43
other	62	4	3.51E-02	1.00E+00	3.43
other	62	4	3.51E-02	1.00E+00	3.43
cancer-related	15	2	3.51E-02	1.00E+00	7.61
immune-related	123	6	3.67E-02	1.00E+00	2.56
other	93	5	3.83E-02	1.00E+00	2.83
other	2	1	3.95E-02	1.00E+00	49.29
immune-related	2	1	3.95E-02	1.00E+00	49.29
other	2	1	3.95E-02	1.00E+00	49.29
other	16	2	3.96E-02	1.00E+00	7.06
other	16	2	3.96E-02	1.00E+00	7.06
other	16	2	3.96E-02	1.00E+00	7.06
other	16	2	3.96E-02	1.00E+00	7.06
cancer-related	16	2	3.96E-02	1.00E+00	7.06
cancer-related	38	3	3.97E-02	1.00E+00	4.25
cancer-related	38	3	3.97E-02	1.00E+00	4.25
cancer-related	94	5	3.99E-02	1.00E+00	2.80
cancer-related	126	6	4.05E-02	1.00E+00	2.49
cancer-related	65	4	4.07E-02	1.00E+00	3.26
other	39	3	4.24E-02	1.00E+00	4.13
other	98	5	4.63E-02	1.00E+00	2.68
other	41	3	4.81E-02	1.00E+00	3.91
cancer-related	69	4	4.89E-02	1.00E+00	3.06
other	69	4	4.89E-02	1.00E+00	3.06
other	18	2	4.92E-02	1.00E+00	6.18
other	167	7	5.02E-02	1.00E+00	2.18

other	167	7	5.02E-02	1.00E+00	2.18
cancer-related	42	3	5.10E-02	1.00E+00	3.81
cancer-related	70	4	5.11E-02	1.00E+00	3.01
other	204	8	5.22E-02	1.00E+00	2.04
other	19	2	5.43E-02	1.00E+00	5.82
other	19	2	5.43E-02	1.00E+00	5.82
other	19	2	5.43E-02	1.00E+00	5.82
other	136	6	5.50E-02	1.00E+00	2.30
other	72	4	5.56E-02	1.00E+00	2.92
other	137	6	5.66E-02	1.00E+00	2.28
other	3	1	5.87E-02	1.00E+00	24.64
other	3	1	5.87E-02	1.00E+00	24.64
other	3	1	5.87E-02	1.00E+00	24.64
other	20	2	5.96E-02	1.00E+00	5.49
cancer-related	20	2	5.96E-02	1.00E+00	5.49
cancer-related	20	2	5.96E-02	1.00E+00	5.49
cancer-related	20	2	5.96E-02	1.00E+00	5.49
cancer-related	45	3	6.04E-02	1.00E+00	3.54
other	21	2	6.50E-02	1.00E+00	5.20
other	21	2	6.50E-02	1.00E+00	5.20
other	21	2	6.50E-02	1.00E+00	5.20
other	21	2	6.50E-02	1.00E+00	5.20
cancer-related	108	5	6.50E-02	1.00E+00	2.41
other	76	4	6.53E-02	1.00E+00	2.76
other	77	4	6.79E-02	1.00E+00	2.72
other	292	10	6.82E-02	1.00E+00	1.77
cancer-related	331	11	6.82E-02	1.00E+00	1.72
other	22	2	7.06E-02	1.00E+00	4.94
other	22	2	7.06E-02	1.00E+00	4.94
other	416	13	7.39E-02	1.00E+00	1.62
cancer-related	147	6	7.43E-02	1.00E+00	2.12
cancer-related	113	5	7.58E-02	1.00E+00	2.30
other	80	4	7.58E-02	1.00E+00	2.61
other	23	2	7.64E-02	1.00E+00	4.71
other	23	2	7.64E-02	1.00E+00	4.71
other	299	10	7.71E-02	1.00E+00	1.73
cancer-related	4	1	7.75E-02	1.00E+00	16.43
cancer-related	4	1	7.75E-02	1.00E+00	16.43
cancer-related	4	1	7.75E-02	1.00E+00	16.43
cancer-related	4	1	7.75E-02	1.00E+00	16.43
cancer-related	4	1	7.75E-02	1.00E+00	16.43
cancer-related	4	1	7.75E-02	1.00E+00	16.43
cancer-related	4	1	7.75E-02	1.00E+00	16.43
cancer-related	4	1	7.75E-02	1.00E+00	16.43
cancer-related	4	1	7.75E-02	1.00E+00	16.43
cancer-related	4	1	7.75E-02	1.00E+00	16.43
cancer-related	4	1	7.75E-02	1.00E+00	16.43
other	4	1	7.75E-02	1.00E+00	16.43
other	4	1	7.75E-02	1.00E+00	16.43
other	4	1	7.75E-02	1.00E+00	16.43

other	4	1	7.75E-02	1.00E+00	16.43
other	4	1	7.75E-02	1.00E+00	16.43
other	4	1	7.75E-02	1.00E+00	16.43
other	185	7	7.76E-02	1.00E+00	1.96
immune-related	382	12	8.13E-02	1.00E+00	1.62
cancer-related	82	4	8.14E-02	1.00E+00	2.54
other	82	4	8.14E-02	1.00E+00	2.54
other	82	4	8.14E-02	1.00E+00	2.54
other	51	3	8.14E-02	1.00E+00	3.10
cancer-related	51	3	8.14E-02	1.00E+00	3.10
cancer-related	24	2	8.22E-02	1.00E+00	4.49
cancer-related	24	2	8.22E-02	1.00E+00	4.49
immune-related	83	4	8.42E-02	1.00E+00	2.51
cancer-related	25	2	8.82E-02	1.00E+00	4.30
cancer-related	25	2	8.82E-02	1.00E+00	4.30
cancer-related	54	3	9.30E-02	1.00E+00	2.91
other	54	3	9.30E-02	1.00E+00	2.91
cancer-related	5	1	9.60E-02	1.00E+00	12.32
other	5	1	9.60E-02	1.00E+00	12.32
other	5	1	9.60E-02	1.00E+00	12.32
other	5	1	9.60E-02	1.00E+00	12.32
cancer-related	5	1	9.60E-02	1.00E+00	12.32
other	56	3	1.01E-01	1.00E+00	2.80
other	56	3	1.01E-01	1.00E+00	2.80
cancer-related	56	3	1.01E-01	1.00E+00	2.80
cancer-related	124	5	1.03E-01	1.00E+00	2.09
other	57	3	1.05E-01	1.00E+00	2.75
cancer-related	90	4	1.06E-01	1.00E+00	2.31
other	28	2	1.07E-01	1.00E+00	3.80
other	28	2	1.07E-01	1.00E+00	3.80
immune-related	58	3	1.09E-01	1.00E+00	2.70
cancer-related	362	11	1.10E-01	1.00E+00	1.57
other	127	5	1.11E-01	1.00E+00	2.03
other	92	4	1.12E-01	1.00E+00	2.25
other	29	2	1.13E-01	1.00E+00	3.66
other	59	3	1.14E-01	1.00E+00	2.65
immune-related	6	1	1.14E-01	1.00E+00	9.85
cancer-related	6	1	1.14E-01	1.00E+00	9.85
other	6	1	1.14E-01	1.00E+00	9.85
other	6	1	1.14E-01	1.00E+00	9.85
other	6	1	1.14E-01	1.00E+00	9.85
cancer-related	6	1	1.14E-01	1.00E+00	9.85
immune-related	6	1	1.14E-01	1.00E+00	9.85
other	6	1	1.14E-01	1.00E+00	9.85
other	6	1	1.14E-01	1.00E+00	9.85
other	6	1	1.14E-01	1.00E+00	9.85
other	93	4	1.15E-01	1.00E+00	2.23
cancer-related	94	4	1.19E-01	1.00E+00	2.20
cancer-related	94	4	1.19E-01	1.00E+00	2.20
cancer-related	30	2	1.20E-01	1.00E+00	3.53

cancer-related	62	3	1.27E-01	1.00E+00	2.52
other	31	2	1.27E-01	1.00E+00	3.41
other	31	2	1.27E-01	1.00E+00	3.41
cancer-related	292	9	1.31E-01	1.00E+00	1.58
cancer-related	375	11	1.31E-01	1.00E+00	1.51
other	7	1	1.32E-01	1.00E+00	8.21
cancer-related	7	1	1.32E-01	1.00E+00	8.21
cancer-related	7	1	1.32E-01	1.00E+00	8.21
other	7	1	1.32E-01	1.00E+00	8.21
cancer-related	7	1	1.32E-01	1.00E+00	8.21
other	7	1	1.32E-01	1.00E+00	8.21
other	7	1	1.32E-01	1.00E+00	8.21
other	7	1	1.32E-01	1.00E+00	8.21
other	7	1	1.32E-01	1.00E+00	8.21
other	7	1	1.32E-01	1.00E+00	8.21
cancer-related	32	2	1.33E-01	1.00E+00	3.29
other	32	2	1.33E-01	1.00E+00	3.29
other	99	4	1.36E-01	1.00E+00	2.09
cancer-related	65	3	1.40E-01	1.00E+00	2.39
other	65	3	1.40E-01	1.00E+00	2.39
immune-related	33	2	1.40E-01	1.00E+00	3.19
cancer-related	102	4	1.47E-01	1.00E+00	2.02
other	8	1	1.49E-01	1.00E+00	7.04
immune-related	8	1	1.49E-01	1.00E+00	7.04
other	8	1	1.49E-01	1.00E+00	7.04
cancer-related	8	1	1.49E-01	1.00E+00	7.04
cancer-related	8	1	1.49E-01	1.00E+00	7.04
cancer-related	8	1	1.49E-01	1.00E+00	7.04
other	8	1	1.49E-01	1.00E+00	7.04
immune-related	8	1	1.49E-01	1.00E+00	7.04
other	8	1	1.49E-01	1.00E+00	7.04
other	8	1	1.49E-01	1.00E+00	7.04
other	35	2	1.54E-01	1.00E+00	2.99
cancer-related	304	9	1.55E-01	1.00E+00	1.52
cancer-related	222	7	1.57E-01	1.00E+00	1.62
cancer-related	36	2	1.61E-01	1.00E+00	2.90
other	36	2	1.61E-01	1.00E+00	2.90
immune-related	144	5	1.61E-01	1.00E+00	1.78
cancer-related	106	4	1.62E-01	1.00E+00	1.94
other	9	1	1.66E-01	1.00E+00	6.16
other	9	1	1.66E-01	1.00E+00	6.16
immune-related	9	1	1.66E-01	1.00E+00	6.16
other	9	1	1.66E-01	1.00E+00	6.16
other	9	1	1.66E-01	1.00E+00	6.16
cancer-related	37	2	1.68E-01	1.00E+00	2.82
other	270	8	1.73E-01	1.00E+00	1.52
other	38	2	1.75E-01	1.00E+00	2.74
cancer-related	149	5	1.78E-01	1.00E+00	1.72
other	73	3	1.79E-01	1.00E+00	2.12
other	73	3	1.79E-01	1.00E+00	2.12

cancer-related	73	3	1.79E-01	1.00E+00	2.12
other	190	6	1.80E-01	1.00E+00	1.62
other	111	4	1.82E-01	1.00E+00	1.85
other	10	1	1.83E-01	1.00E+00	5.47
other	10	1	1.83E-01	1.00E+00	5.47
other	10	1	1.83E-01	1.00E+00	5.47
immune-related	10	1	1.83E-01	1.00E+00	5.47
other	10	1	1.83E-01	1.00E+00	5.47
cancer-related	10	1	1.83E-01	1.00E+00	5.47
cancer-related	74	3	1.84E-01	1.00E+00	2.09
immune-related	112	4	1.86E-01	1.00E+00	1.83
other	75	3	1.89E-01	1.00E+00	2.06
cancer-related	40	2	1.90E-01	1.00E+00	2.60
cancer-related	235	7	1.91E-01	1.00E+00	1.52
other	194	6	1.92E-01	1.00E+00	1.58
cancer-related	1462	34	1.93E-01	1.00E+00	1.20
cancer-related	236	7	1.93E-01	1.00E+00	1.52
cancer-related	76	3	1.94E-01	1.00E+00	2.03
cancer-related	41	2	1.97E-01	1.00E+00	2.53
other	11	1	1.99E-01	1.00E+00	4.92
immune-related	11	1	1.99E-01	1.00E+00	4.92
other	11	1	1.99E-01	1.00E+00	4.92
cancer-related	11	1	1.99E-01	1.00E+00	4.92
other	11	1	1.99E-01	1.00E+00	4.92
other	11	1	1.99E-01	1.00E+00	4.92
cancer-related	11	1	1.99E-01	1.00E+00	4.92
other	11	1	1.99E-01	1.00E+00	4.92
other	239	7	2.02E-01	1.00E+00	1.50
cancer-related	42	2	2.04E-01	1.00E+00	2.47
cancer-related	42	2	2.04E-01	1.00E+00	2.47
other	117	4	2.06E-01	1.00E+00	1.75
cancer-related	79	3	2.09E-01	1.00E+00	1.95
other	43	2	2.12E-01	1.00E+00	2.41
other	43	2	2.12E-01	1.00E+00	2.41
other	43	2	2.12E-01	1.00E+00	2.41
cancer-related	43	2	2.12E-01	1.00E+00	2.41
immune-related	12	1	2.15E-01	1.00E+00	4.48
other	12	1	2.15E-01	1.00E+00	4.48
cancer-related	12	1	2.15E-01	1.00E+00	4.48
other	12	1	2.15E-01	1.00E+00	4.48
cancer-related	12	1	2.15E-01	1.00E+00	4.48
other	12	1	2.15E-01	1.00E+00	4.48
other	12	1	2.15E-01	1.00E+00	4.48
other	12	1	2.15E-01	1.00E+00	4.48
other	12	1	2.15E-01	1.00E+00	4.48
immune-related	12	1	2.15E-01	1.00E+00	4.48
other	202	6	2.17E-01	1.00E+00	1.52
immune-related	120	4	2.19E-01	1.00E+00	1.70
cancer-related	691	17	2.19E-01	1.00E+00	1.26
cancer-related	44	2	2.19E-01	1.00E+00	2.35

immune-related	82	3	2.25E-01	1.00E+00	1.88
immune-related	45	2	2.27E-01	1.00E+00	2.29
other	205	6	2.27E-01	1.00E+00	1.49
other	13	1	2.31E-01	1.00E+00	4.10
other	13	1	2.31E-01	1.00E+00	4.10
other	13	1	2.31E-01	1.00E+00	4.10
other	13	1	2.31E-01	1.00E+00	4.10
other	13	1	2.31E-01	1.00E+00	4.10
other	123	4	2.31E-01	1.00E+00	1.66
cancer-related	46	2	2.34E-01	1.00E+00	2.24
cancer-related	124	4	2.36E-01	1.00E+00	1.65
immune-related	47	2	2.41E-01	1.00E+00	2.19
immune-related	47	2	2.41E-01	1.00E+00	2.19
other	126	4	2.44E-01	1.00E+00	1.62
cancer-related	168	5	2.45E-01	1.00E+00	1.52
other	14	1	2.46E-01	1.00E+00	3.79
cancer-related	14	1	2.46E-01	1.00E+00	3.79
immune-related	14	1	2.46E-01	1.00E+00	3.79
other	14	1	2.46E-01	1.00E+00	3.79
cancer-related	14	1	2.46E-01	1.00E+00	3.79
other	14	1	2.46E-01	1.00E+00	3.79
cancer-related	14	1	2.46E-01	1.00E+00	3.79
other	14	1	2.46E-01	1.00E+00	3.79
other	14	1	2.46E-01	1.00E+00	3.79
immune-related	14	1	2.46E-01	1.00E+00	3.79
other	14	1	2.46E-01	1.00E+00	3.79
other	14	1	2.46E-01	1.00E+00	3.79
other	14	1	2.46E-01	1.00E+00	3.79
other	14	1	2.46E-01	1.00E+00	3.79
other	14	1	2.46E-01	1.00E+00	3.79
immune-related	14	1	2.46E-01	1.00E+00	3.79
cancer-related	48	2	2.49E-01	1.00E+00	2.14
other	48	2	2.49E-01	1.00E+00	2.14
immune-related	48	2	2.49E-01	1.00E+00	2.14
other	49	2	2.56E-01	1.00E+00	2.10
cancer-related	88	3	2.57E-01	1.00E+00	1.74
cancer-related	129	4	2.57E-01	1.00E+00	1.58
cancer-related	15	1	2.61E-01	1.00E+00	3.52
other	15	1	2.61E-01	1.00E+00	3.52
cancer-related	15	1	2.61E-01	1.00E+00	3.52
cancer-related	15	1	2.61E-01	1.00E+00	3.52
other	15	1	2.61E-01	1.00E+00	3.52
other	15	1	2.61E-01	1.00E+00	3.52
cancer-related	15	1	2.61E-01	1.00E+00	3.52
cancer-related	15	1	2.61E-01	1.00E+00	3.52
other	15	1	2.61E-01	1.00E+00	3.52
cancer-related	89	3	2.62E-01	1.00E+00	1.72
cancer-related	89	3	2.62E-01	1.00E+00	1.72
cancer-related	50	2	2.64E-01	1.00E+00	2.05
other	50	2	2.64E-01	1.00E+00	2.05

other	50	2	2.64E-01	1.00E+00	2.05
other	90	3	2.68E-01	1.00E+00	1.70
other	51	2	2.71E-01	1.00E+00	2.01
other	51	2	2.71E-01	1.00E+00	2.01
other	51	2	2.71E-01	1.00E+00	2.01
cancer-related	51	2	2.71E-01	1.00E+00	2.01
other	133	4	2.75E-01	1.00E+00	1.53
other	16	1	2.76E-01	1.00E+00	3.28
cancer-related	16	1	2.76E-01	1.00E+00	3.28
other	16	1	2.76E-01	1.00E+00	3.28
cancer-related	16	1	2.76E-01	1.00E+00	3.28
other	16	1	2.76E-01	1.00E+00	3.28
other	16	1	2.76E-01	1.00E+00	3.28
immune-related	16	1	2.76E-01	1.00E+00	3.28
other	52	2	2.79E-01	1.00E+00	1.97
other	52	2	2.79E-01	1.00E+00	1.97
other	52	2	2.79E-01	1.00E+00	1.97
other	52	2	2.79E-01	1.00E+00	1.97
other	52	2	2.79E-01	1.00E+00	1.97
other	52	2	2.79E-01	1.00E+00	1.97
other	134	4	2.79E-01	1.00E+00	1.52
other	53	2	2.86E-01	1.00E+00	1.93
other	53	2	2.86E-01	1.00E+00	1.93
cancer-related	53	2	2.86E-01	1.00E+00	1.93
other	17	1	2.90E-01	1.00E+00	3.08
cancer-related	17	1	2.90E-01	1.00E+00	3.08
cancer-related	17	1	2.90E-01	1.00E+00	3.08
cancer-related	17	1	2.90E-01	1.00E+00	3.08
other	17	1	2.90E-01	1.00E+00	3.08
other	17	1	2.90E-01	1.00E+00	3.08
other	95	3	2.95E-01	1.00E+00	1.61
immune-related	96	3	3.01E-01	1.00E+00	1.59
other	55	2	3.01E-01	1.00E+00	1.86
other	55	2	3.01E-01	1.00E+00	1.86
other	55	2	3.01E-01	1.00E+00	1.86
other	55	2	3.01E-01	1.00E+00	1.86
cancer-related	55	2	3.01E-01	1.00E+00	1.86
other	55	2	3.01E-01	1.00E+00	1.86
other	55	2	3.01E-01	1.00E+00	1.86
other	55	2	3.01E-01	1.00E+00	1.86
other	55	2	3.01E-01	1.00E+00	1.86
other	55	2	3.01E-01	1.00E+00	1.86
other	410	10	3.04E-01	1.00E+00	1.24
cancer-related	18	1	3.05E-01	1.00E+00	2.90
other	18	1	3.05E-01	1.00E+00	2.90
other	18	1	3.05E-01	1.00E+00	2.90
other	18	1	3.05E-01	1.00E+00	2.90
other	97	3	3.06E-01	1.00E+00	1.57
other	56	2	3.08E-01	1.00E+00	1.82
other	56	2	3.08E-01	1.00E+00	1.82

other	141	4	3.11E-01	1.00E+00	1.44
cancer-related	141	4	3.11E-01	1.00E+00	1.44
immune-related	98	3	3.11E-01	1.00E+00	1.56
other	98	3	3.11E-01	1.00E+00	1.56
cancer-related	57	2	3.16E-01	1.00E+00	1.79
other	57	2	3.16E-01	1.00E+00	1.79
other	57	2	3.16E-01	1.00E+00	1.79
other	99	3	3.17E-01	1.00E+00	1.54
cancer-related	19	1	3.19E-01	1.00E+00	2.73
other	19	1	3.19E-01	1.00E+00	2.73
other	19	1	3.19E-01	1.00E+00	2.73
other	19	1	3.19E-01	1.00E+00	2.73
other	19	1	3.19E-01	1.00E+00	2.73
cancer-related	19	1	3.19E-01	1.00E+00	2.73
cancer-related	19	1	3.19E-01	1.00E+00	2.73
other	101	3	3.28E-01	1.00E+00	1.51
other	560	13	3.28E-01	1.00E+00	1.18
other	235	6	3.29E-01	1.00E+00	1.29
other	235	6	3.29E-01	1.00E+00	1.29
other	59	2	3.30E-01	1.00E+00	1.73
other	59	2	3.30E-01	1.00E+00	1.73
other	59	2	3.30E-01	1.00E+00	1.73
other	59	2	3.30E-01	1.00E+00	1.73
other	20	1	3.32E-01	1.00E+00	2.59
other	20	1	3.32E-01	1.00E+00	2.59
other	236	6	3.33E-01	1.00E+00	1.29
other	60	2	3.38E-01	1.00E+00	1.70
other	60	2	3.38E-01	1.00E+00	1.70
other	60	2	3.38E-01	1.00E+00	1.70
other	60	2	3.38E-01	1.00E+00	1.70
other	60	2	3.38E-01	1.00E+00	1.70
other	60	2	3.38E-01	1.00E+00	1.70
other	60	2	3.38E-01	1.00E+00	1.70
other	60	2	3.38E-01	1.00E+00	1.70
other	60	2	3.38E-01	1.00E+00	1.70
immune-related	147	4	3.38E-01	1.00E+00	1.38
other	61	2	3.45E-01	1.00E+00	1.67
cancer-related	61	2	3.45E-01	1.00E+00	1.67
cancer-related	21	1	3.46E-01	1.00E+00	2.46
other	21	1	3.46E-01	1.00E+00	2.46
other	21	1	3.46E-01	1.00E+00	2.46
other	21	1	3.46E-01	1.00E+00	2.46
other	21	1	3.46E-01	1.00E+00	2.46
immune-related	21	1	3.46E-01	1.00E+00	2.46
other	21	1	3.46E-01	1.00E+00	2.46
other	149	4	3.47E-01	1.00E+00	1.36
other	62	2	3.52E-01	1.00E+00	1.64
other	62	2	3.52E-01	1.00E+00	1.64
cancer-related	62	2	3.52E-01	1.00E+00	1.64
other	62	2	3.52E-01	1.00E+00	1.64

other	106	3	3.55E-01	1.00E+00	1.44
other	106	3	3.55E-01	1.00E+00	1.44
cancer-related	22	1	3.59E-01	1.00E+00	2.34
cancer-related	22	1	3.59E-01	1.00E+00	2.34
other	22	1	3.59E-01	1.00E+00	2.34
cancer-related	22	1	3.59E-01	1.00E+00	2.34
immune-related	22	1	3.59E-01	1.00E+00	2.34
other	22	1	3.59E-01	1.00E+00	2.34
other	22	1	3.59E-01	1.00E+00	2.34
other	63	2	3.59E-01	1.00E+00	1.61
cancer-related	108	3	3.66E-01	1.00E+00	1.41
other	64	2	3.67E-01	1.00E+00	1.59
cancer-related	64	2	3.67E-01	1.00E+00	1.59
other	64	2	3.67E-01	1.00E+00	1.59
other	64	2	3.67E-01	1.00E+00	1.59
immune-related	23	1	3.71E-01	1.00E+00	2.24
other	23	1	3.71E-01	1.00E+00	2.24
other	23	1	3.71E-01	1.00E+00	2.24
other	109	3	3.72E-01	1.00E+00	1.39
other	65	2	3.74E-01	1.00E+00	1.56
cancer-related	155	4	3.74E-01	1.00E+00	1.31
cancer-related	156	4	3.79E-01	1.00E+00	1.30
other	66	2	3.81E-01	1.00E+00	1.54
other	66	2	3.81E-01	1.00E+00	1.54
other	66	2	3.81E-01	1.00E+00	1.54
cancer-related	66	2	3.81E-01	1.00E+00	1.54
other	297	7	3.82E-01	1.00E+00	1.19
other	24	1	3.84E-01	1.00E+00	2.14
cancer-related	24	1	3.84E-01	1.00E+00	2.14
other	24	1	3.84E-01	1.00E+00	2.14
other	24	1	3.84E-01	1.00E+00	2.14
other	24	1	3.84E-01	1.00E+00	2.14
other	24	1	3.84E-01	1.00E+00	2.14
other	298	7	3.86E-01	1.00E+00	1.19
other	158	4	3.88E-01	1.00E+00	1.28
other	67	2	3.88E-01	1.00E+00	1.51
other	67	2	3.88E-01	1.00E+00	1.51
other	67	2	3.88E-01	1.00E+00	1.51
other	67	2	3.88E-01	1.00E+00	1.51
cancer-related	68	2	3.95E-01	1.00E+00	1.49
other	68	2	3.95E-01	1.00E+00	1.49
other	68	2	3.95E-01	1.00E+00	1.49
other	68	2	3.95E-01	1.00E+00	1.49
cancer-related	68	2	3.95E-01	1.00E+00	1.49
cancer-related	25	1	3.96E-01	1.00E+00	2.05
cancer-related	25	1	3.96E-01	1.00E+00	2.05
other	25	1	3.96E-01	1.00E+00	2.05
cancer-related	25	1	3.96E-01	1.00E+00	2.05
immune-related	114	3	3.99E-01	1.00E+00	1.33
immune-related	114	3	3.99E-01	1.00E+00	1.33

other	69	2	4.02E-01	1.00E+00	1.47
other	69	2	4.02E-01	1.00E+00	1.47
cancer-related	115	3	4.04E-01	1.00E+00	1.32
other	26	1	4.08E-01	1.00E+00	1.97
cancer-related	26	1	4.08E-01	1.00E+00	1.97
cancer-related	26	1	4.08E-01	1.00E+00	1.97
other	70	2	4.09E-01	1.00E+00	1.45
other	117	3	4.15E-01	1.00E+00	1.30
immune-related	117	3	4.15E-01	1.00E+00	1.30
immune-related	117	3	4.15E-01	1.00E+00	1.30
other	71	2	4.16E-01	1.00E+00	1.43
other	27	1	4.20E-01	1.00E+00	1.89
other	27	1	4.20E-01	1.00E+00	1.89
immune-related	27	1	4.20E-01	1.00E+00	1.89
other	27	1	4.20E-01	1.00E+00	1.89
other	72	2	4.23E-01	1.00E+00	1.41
other	72	2	4.23E-01	1.00E+00	1.41
other	72	2	4.23E-01	1.00E+00	1.41
other	72	2	4.23E-01	1.00E+00	1.41
other	73	2	4.30E-01	1.00E+00	1.39
other	73	2	4.30E-01	1.00E+00	1.39
other	73	2	4.30E-01	1.00E+00	1.39
other	73	2	4.30E-01	1.00E+00	1.39
other	28	1	4.32E-01	1.00E+00	1.82
other	28	1	4.32E-01	1.00E+00	1.82
other	28	1	4.32E-01	1.00E+00	1.82
immune-related	170	4	4.42E-01	1.00E+00	1.19
other	29	1	4.43E-01	1.00E+00	1.76
cancer-related	29	1	4.43E-01	1.00E+00	1.76
other	29	1	4.43E-01	1.00E+00	1.76
other	29	1	4.43E-01	1.00E+00	1.76
other	29	1	4.43E-01	1.00E+00	1.76
other	29	1	4.43E-01	1.00E+00	1.76
other	29	1	4.43E-01	1.00E+00	1.76
other	29	1	4.43E-01	1.00E+00	1.76
other	29	1	4.43E-01	1.00E+00	1.76
other	75	2	4.44E-01	1.00E+00	1.35
other	75	2	4.44E-01	1.00E+00	1.35
cancer-related	219	5	4.45E-01	1.00E+00	1.15
other	76	2	4.50E-01	1.00E+00	1.33
other	76	2	4.50E-01	1.00E+00	1.33
other	30	1	4.54E-01	1.00E+00	1.70
other	30	1	4.54E-01	1.00E+00	1.70
immune-related	30	1	4.54E-01	1.00E+00	1.70
other	30	1	4.54E-01	1.00E+00	1.70
other	30	1	4.54E-01	1.00E+00	1.70
cancer-related	30	1	4.54E-01	1.00E+00	1.70
other	30	1	4.54E-01	1.00E+00	1.70
immune-related	30	1	4.54E-01	1.00E+00	1.70
immune-related	77	2	4.57E-01	1.00E+00	1.31

other	78	2	4.64E-01	1.00E+00	1.29
other	78	2	4.64E-01	1.00E+00	1.29
other	31	1	4.65E-01	1.00E+00	1.64
other	31	1	4.65E-01	1.00E+00	1.64
cancer-related	31	1	4.65E-01	1.00E+00	1.64
immune-related	31	1	4.65E-01	1.00E+00	1.64
other	31	1	4.65E-01	1.00E+00	1.64
other	31	1	4.65E-01	1.00E+00	1.64
other	127	3	4.68E-01	1.00E+00	1.19
other	79	2	4.70E-01	1.00E+00	1.28
other	79	2	4.70E-01	1.00E+00	1.28
cancer-related	32	1	4.76E-01	1.00E+00	1.59
other	32	1	4.76E-01	1.00E+00	1.59
cancer-related	80	2	4.77E-01	1.00E+00	1.26
other	80	2	4.77E-01	1.00E+00	1.26
immune-related	33	1	4.87E-01	1.00E+00	1.54
other	33	1	4.87E-01	1.00E+00	1.54
cancer-related	33	1	4.87E-01	1.00E+00	1.54
other	33	1	4.87E-01	1.00E+00	1.54
other	33	1	4.87E-01	1.00E+00	1.54
other	33	1	4.87E-01	1.00E+00	1.54
other	33	1	4.87E-01	1.00E+00	1.54
other	33	1	4.87E-01	1.00E+00	1.54
other	2127	43	4.91E-01	1.00E+00	1.02
other	132	3	4.93E-01	1.00E+00	1.14
cancer-related	232	5	4.95E-01	1.00E+00	1.08
other	83	2	4.96E-01	1.00E+00	1.21
other	83	2	4.96E-01	1.00E+00	1.21
other	34	1	4.97E-01	1.00E+00	1.49
other	34	1	4.97E-01	1.00E+00	1.49
cancer-related	34	1	4.97E-01	1.00E+00	1.49
other	34	1	4.97E-01	1.00E+00	1.49
other	84	2	5.03E-01	1.00E+00	1.20
cancer-related	134	3	5.03E-01	1.00E+00	1.13
other	35	1	5.07E-01	1.00E+00	1.45
cancer-related	35	1	5.07E-01	1.00E+00	1.45
cancer-related	185	4	5.07E-01	1.00E+00	1.09
other	85	2	5.09E-01	1.00E+00	1.18
other	85	2	5.09E-01	1.00E+00	1.18
other	85	2	5.09E-01	1.00E+00	1.18
other	186	4	5.12E-01	1.00E+00	1.08
other	186	4	5.12E-01	1.00E+00	1.08
other	86	2	5.15E-01	1.00E+00	1.17
other	36	1	5.17E-01	1.00E+00	1.40
other	36	1	5.17E-01	1.00E+00	1.40
other	36	1	5.17E-01	1.00E+00	1.40
other	137	3	5.18E-01	1.00E+00	1.10
other	87	2	5.21E-01	1.00E+00	1.16
other	87	2	5.21E-01	1.00E+00	1.16
other	290	6	5.23E-01	1.00E+00	1.04

other	189	4	5.24E-01	1.00E+00	1.06
other	37	1	5.26E-01	1.00E+00	1.36
other	37	1	5.26E-01	1.00E+00	1.36
cancer-related	37	1	5.26E-01	1.00E+00	1.36
other	37	1	5.26E-01	1.00E+00	1.36
other	37	1	5.26E-01	1.00E+00	1.36
other	37	1	5.26E-01	1.00E+00	1.36
other	37	1	5.26E-01	1.00E+00	1.36
other	37	1	5.26E-01	1.00E+00	1.36
other	140	3	5.33E-01	1.00E+00	1.08
other	89	2	5.34E-01	1.00E+00	1.13
immune-related	89	2	5.34E-01	1.00E+00	1.13
cancer-related	38	1	5.36E-01	1.00E+00	1.33
cancer-related	38	1	5.36E-01	1.00E+00	1.33
other	38	1	5.36E-01	1.00E+00	1.33
other	141	3	5.37E-01	1.00E+00	1.07
other	90	2	5.40E-01	1.00E+00	1.12
other	90	2	5.40E-01	1.00E+00	1.12
cancer-related	90	2	5.40E-01	1.00E+00	1.12
other	39	1	5.45E-01	1.00E+00	1.29
other	39	1	5.45E-01	1.00E+00	1.29
other	39	1	5.45E-01	1.00E+00	1.29
other	39	1	5.45E-01	1.00E+00	1.29
other	91	2	5.46E-01	1.00E+00	1.10
other	91	2	5.46E-01	1.00E+00	1.10
cancer-related	92	2	5.52E-01	1.00E+00	1.09
other	94	2	5.63E-01	1.00E+00	1.07
cancer-related	41	1	5.63E-01	1.00E+00	1.23
other	41	1	5.63E-01	1.00E+00	1.23
cancer-related	42	1	5.72E-01	1.00E+00	1.20
other	42	1	5.72E-01	1.00E+00	1.20
other	42	1	5.72E-01	1.00E+00	1.20
other	42	1	5.72E-01	1.00E+00	1.20
cancer-related	42	1	5.72E-01	1.00E+00	1.20
cancer-related	97	2	5.80E-01	1.00E+00	1.03
other	43	1	5.81E-01	1.00E+00	1.17
other	43	1	5.81E-01	1.00E+00	1.17
other	43	1	5.81E-01	1.00E+00	1.17
cancer-related	43	1	5.81E-01	1.00E+00	1.17
other	44	1	5.89E-01	1.00E+00	1.14
other	44	1	5.89E-01	1.00E+00	1.14
immune-related	44	1	5.89E-01	1.00E+00	1.14
other	99	2	5.92E-01	1.00E+00	1.01
other	45	1	5.97E-01	1.00E+00	1.12
other	45	1	5.97E-01	1.00E+00	1.12
other	154	3	5.97E-01	1.00E+00	0.97
cancer-related	46	1	6.05E-01	1.00E+00	1.09
other	46	1	6.05E-01	1.00E+00	1.09
cancer-related	102	2	6.08E-01	1.00E+00	0.98
other	103	2	6.13E-01	1.00E+00	0.97

other	47	1	6.13E-01	1.00E+00	1.07
other	47	1	6.13E-01	1.00E+00	1.07
cancer-related	158	3	6.15E-01	1.00E+00	0.95
other	104	2	6.19E-01	1.00E+00	0.96
other	48	1	6.21E-01	1.00E+00	1.04
other	105	2	6.24E-01	1.00E+00	0.95
cancer-related	49	1	6.29E-01	1.00E+00	1.02
other	49	1	6.29E-01	1.00E+00	1.02
immune-related	50	1	6.36E-01	1.00E+00	1.00
other	50	1	6.36E-01	1.00E+00	1.00
immune-related	50	1	6.36E-01	1.00E+00	1.00
other	108	2	6.39E-01	1.00E+00	0.93
other	108	2	6.39E-01	1.00E+00	0.93
other	108	2	6.39E-01	1.00E+00	0.93
other	164	3	6.40E-01	1.00E+00	0.91
other	110	2	6.49E-01	1.00E+00	0.91
other	110	2	6.49E-01	1.00E+00	0.91
immune-related	52	1	6.51E-01	1.00E+00	0.96
other	52	1	6.51E-01	1.00E+00	0.96
other	222	4	6.51E-01	1.00E+00	0.90
other	111	2	6.54E-01	1.00E+00	0.90
cancer-related	53	1	6.58E-01	1.00E+00	0.94
other	112	2	6.59E-01	1.00E+00	0.89
other	113	2	6.63E-01	1.00E+00	0.88
other	54	1	6.64E-01	1.00E+00	0.93
other	54	1	6.64E-01	1.00E+00	0.93
other	55	1	6.71E-01	1.00E+00	0.91
other	55	1	6.71E-01	1.00E+00	0.91
other	115	2	6.73E-01	1.00E+00	0.87
other	117	2	6.82E-01	1.00E+00	0.85
other	176	3	6.87E-01	1.00E+00	0.85
cancer-related	58	1	6.91E-01	1.00E+00	0.86
cancer-related	58	1	6.91E-01	1.00E+00	0.86
cancer-related	58	1	6.91E-01	1.00E+00	0.86
cancer-related	58	1	6.91E-01	1.00E+00	0.86
cancer-related	58	1	6.91E-01	1.00E+00	0.86
other	58	1	6.91E-01	1.00E+00	0.86
immune-related	58	1	6.91E-01	1.00E+00	0.86
other	58	1	6.91E-01	1.00E+00	0.86
cancer-related	58	1	6.91E-01	1.00E+00	0.86
other	178	3	6.94E-01	1.00E+00	0.84
other	120	2	6.95E-01	1.00E+00	0.83
other	59	1	6.97E-01	1.00E+00	0.85
other	59	1	6.97E-01	1.00E+00	0.85
other	59	1	6.97E-01	1.00E+00	0.85
other	121	2	7.00E-01	1.00E+00	0.82
cancer-related	121	2	7.00E-01	1.00E+00	0.82
cancer-related	121	2	7.00E-01	1.00E+00	0.82
other	735	13	7.15E-01	1.00E+00	0.88
other	125	2	7.17E-01	1.00E+00	0.80

other	63	1	7.20E-01	1.00E+00	0.79
other	63	1	7.20E-01	1.00E+00	0.79
other	126	2	7.21E-01	1.00E+00	0.79
other	126	2	7.21E-01	1.00E+00	0.79
other	247	4	7.31E-01	1.00E+00	0.80
other	131	2	7.41E-01	1.00E+00	0.76
immune-related	69	1	7.52E-01	1.00E+00	0.72
other	69	1	7.52E-01	1.00E+00	0.72
other	71	1	7.62E-01	1.00E+00	0.70
cancer-related	318	5	7.66E-01	1.00E+00	0.78
cancer-related	140	2	7.73E-01	1.00E+00	0.71
cancer-related	140	2	7.73E-01	1.00E+00	0.71
cancer-related	74	1	7.76E-01	1.00E+00	0.67
other	75	1	7.81E-01	1.00E+00	0.66
cancer-related	76	1	7.85E-01	1.00E+00	0.65
other	144	2	7.86E-01	1.00E+00	0.69
cancer-related	77	1	7.90E-01	1.00E+00	0.64
other	270	4	7.92E-01	1.00E+00	0.73
other	270	4	7.92E-01	1.00E+00	0.73
other	78	1	7.94E-01	1.00E+00	0.64
cancer-related	78	1	7.94E-01	1.00E+00	0.64
cancer-related	78	1	7.94E-01	1.00E+00	0.64
cancer-related	78	1	7.94E-01	1.00E+00	0.64
other	212	3	8.00E-01	1.00E+00	0.70
other	149	2	8.02E-01	1.00E+00	0.66
immune-related	82	1	8.10E-01	1.00E+00	0.60
other	82	1	8.10E-01	1.00E+00	0.60
other	153	2	8.14E-01	1.00E+00	0.65
other	84	1	8.17E-01	1.00E+00	0.59
cancer-related	84	1	8.17E-01	1.00E+00	0.59
other	157	2	8.25E-01	1.00E+00	0.63
other	1253	21	8.33E-01	1.00E+00	0.82
other	162	2	8.38E-01	1.00E+00	0.61
cancer-related	168	2	8.53E-01	1.00E+00	0.59
immune-related	95	1	8.54E-01	1.00E+00	0.52
other	95	1	8.54E-01	1.00E+00	0.52
other	96	1	8.57E-01	1.00E+00	0.51
other	96	1	8.57E-01	1.00E+00	0.51
cancer-related	97	1	8.60E-01	1.00E+00	0.51
other	98	1	8.63E-01	1.00E+00	0.50
other	100	1	8.68E-01	1.00E+00	0.49
other	310	4	8.71E-01	1.00E+00	0.64
other	103	1	8.76E-01	1.00E+00	0.48
cancer-related	104	1	8.78E-01	1.00E+00	0.47
other	106	1	8.83E-01	1.00E+00	0.47
cancer-related	106	1	8.83E-01	1.00E+00	0.47
other	183	2	8.84E-01	1.00E+00	0.54
other	109	1	8.90E-01	1.00E+00	0.45
other	110	1	8.92E-01	1.00E+00	0.45
cancer-related	111	1	8.95E-01	1.00E+00	0.44

other	189	2	8.95E-01	1.00E+00	0.52
other	112	1	8.97E-01	1.00E+00	0.44
other	112	1	8.97E-01	1.00E+00	0.44
cancer-related	113	1	8.99E-01	1.00E+00	0.44
cancer-related	335	4	9.06E-01	1.00E+00	0.59
other	117	1	9.07E-01	1.00E+00	0.42
cancer-related	268	3	9.07E-01	1.00E+00	0.55
other	197	2	9.08E-01	1.00E+00	0.50
other	119	1	9.10E-01	1.00E+00	0.41
other	204	2	9.18E-01	1.00E+00	0.48
other	127	1	9.24E-01	1.00E+00	0.39
other	129	1	9.27E-01	1.00E+00	0.38
other	1377	21	9.29E-01	1.00E+00	0.74
cancer-related	1547	24	9.30E-01	1.00E+00	0.75
cancer-related	132	1	9.31E-01	1.00E+00	0.37
cancer-related	216	2	9.33E-01	1.00E+00	0.45
cancer-related	138	1	9.39E-01	1.00E+00	0.36
cancer-related	634	8	9.43E-01	1.00E+00	0.61
other	144	1	9.46E-01	1.00E+00	0.34
other	309	3	9.49E-01	1.00E+00	0.47
other	155	1	9.57E-01	1.00E+00	0.32
other	246	2	9.59E-01	1.00E+00	0.40
other	250	2	9.62E-01	1.00E+00	0.39
other	177	1	9.73E-01	1.00E+00	0.28
cancer-related	709	8	9.76E-01	1.00E+00	0.54
other	201	1	9.83E-01	1.00E+00	0.24
other	609	6	9.85E-01	1.00E+00	0.47
other	758	8	9.87E-01	1.00E+00	0.51
other	334	1	9.99E-01	1.00E+00	0.14
cancer-related	468	2	9.99E-01	1.00E+00	0.20

ProteinName

ADAM10, ALDOA, ANPEP, ANXA2, ARPC5, B2M, CAMP, CCT2, CCT8, CD36, CD47, CD53, CD59, CTSD,
ALDOA, ANXA5, CALM3, CD36, CD9, CDC42, CFL1, ENDOD1, FCER1G, FERMT3, FLNA, GNAI2, GNAQ,
ACTB, ALDOA, ANXA2, ANXA5, ATP2A3, CALM3, CD36, CD47, CD9, CD99, CDC42, CFL1, ENDOD1, F1
ALDOA, ANXA5, CALM3, CD36, CD9, CFL1, ENDOD1, FERMT3, FLNA, ITGA2B, ITGB3, LGALS3BP, MMI
ALDOA, ANXA5, CALM3, CD36, CD9, CFL1, ENDOD1, FERMT3, FLNA, ITGA2B, ITGB3, LGALS3BP, MMI
ACTB, ADAM10, ALDOA, ANPEP, ANXA2, ARPC5, B2M, CALM3, CAMP, CCT2, CCT8, CD36, CD47, CD5
ACTB, ADAM10, ALDOA, ANPEP, ANXA2, ARPC5, B2M, BCAP31, CALM3, CAMP, CANX, CCL5, CCT2, C
ANXA2, CDC42, CFL1, HNRNPA2B1, HNRNPF, HSPA9, MSN, PPIA, RAP1B, RPLP0, SOD1, TCP1
ANXA2, CANX, CDC42, CFL1, HNRNPA2B1, HNRNPF, HSPA9, MSN, PPIA, RAP1B, RPLP0, SOD1, TCP1
ACTB, CCT2, CCT4, CCT6A, CCT7, CCT8, TCP1, TUBA1C, TUBA4A, TUBB1
ANXA2, CDC42, CFL1, HNRNPA2B1, HNRNPF, HSPA9, MSN, PPIA, RAP1B, RPLP0, SOD1, TCP1
ACTB, ARPC5, BCAP31, CALM3, CCT2, CCT6A, CCT7, CDC42, CFL1, CYBB, DSG1, DSP, FLNA, FLOT1, H2
ACTB, CCT2, CCT4, CCT6A, CCT7, CCT8, TCP1
ACTB, ARPC5, BCAP31, CALM3, CCT2, CCT6A, CCT7, CDC42, CFL1, CYBB, DSG1, DSP, FLNA, FLOT1, H2
ACTB, ARPC5, CALM3, CDC42, CFL1, CYBB, FLNA, H2AC20, H2BC21, ITGB1, MYH9, MYL12B, MYL6, PI
ACTB, CCT2, CCT4, CCT6A, CCT7, CCT8, TCP1, TUBA1C, TUBA4A, TUBB1
CCT2, CCT4, CCT6A, CCT7, CCT8, TCP1, TUBA1C, TUBA4A, TUBB1
CASP14, DSC1, DSG1, DSP, JUP, KRT1, KRT10, KRT14, KRT15, KRT16, KRT17, KRT2, KRT5, KRT6A, KRT
ACTB, ADAM10, ANK1, ARPC5, CDC42, CFL1, CLTA, HSP90AA1, HSPA8, ITGA2B, ITGB1, ITGB3, MSN,
ACTB, CALM3, MYH9, RAB10, TUBA1C, TUBA4A, TUBB1, YWHAB, YWHAE, YWHAG, YWHAH, YWHAZ
ACTB, ADAM10, ANK1, ARPC5, CDC42, CFL1, CLTA, HSP90AA1, HSPA8, ITGA2B, ITGB1, ITGB3, MSN,
ACTB, ADAM10, ANK1, ARPC5, CASP14, CD36, CDC42, CFL1, CLTA, DSC1, DSG1, DSP, H2AC20, H2BC
EEF1A1, NPM1, PPIA, RPS15, RPS15A, RPS23, RPS3A, RPSA, YWHAB, YWHAE, YWHAG, YWHAH, YWH
ACTB, CCT2, CCT4, CCT6A, CCT7, CCT8, GNAQ, GNG5, TCP1, TUBA1C, TUBA4A, TUBB1
ACTB, CALM3, ITGA2B, ITGB3, PHB, RAP1B, TLN1, VCL, YWHAB
ACTB, CALM3, ITGA2B, ITGB3, PHB, RAP1B, TLN1, VCL, YWHAB
ACTB, CALM3, ITGA2B, ITGB3, PHB, RAP1B, TLN1, VCL, YWHAB
ACTB, CALM3, ITGA2B, ITGB3, PHB, RAP1B, TLN1, VCL, YWHAB
ACTB, CCT2, CCT4, CCT6A, CCT7, CCT8, GNAQ, GNG5, TCP1, TUBA1C, TUBA4A, TUBB1
ACTB, ADAM10, ANTXR2, ARPC5, ATIC, B2M, BCAP31, CALM3, CANX, CD36, CD9, CDC42, CLTA, EEF1
CCT2, CCT4, CCT6A, CCT7, CCT8, GNAQ, GNG5, TCP1
CANX, EEF1A1, NPM1, PPIA, RPS15, RPS15A, RPS23, RPS3A, RPSA, YWHAB, YWHAE, YWHAG, YWHAI
CASP14, DSC1, DSG1, DSP, JUP, KRT1, KRT10, KRT14, KRT15, KRT16, KRT17, KRT2, KRT5, KRT6A, KRT
ACTB, ANTXR2, ARPC5, B2M, BCAP31, CALM3, CANX, CD9, CDC42, CLTA, EEF1A1, EEF2, ENO1, G3BP
ACTB, CALM3, ITGA2B, ITGB3, RAP1B, TLN1, VCL, YWHAB
YWHAB, YWHAE, YWHAG, YWHAH, YWHAZ
H2AC20, H2BC21, MYH9, MYL12B, MYL6, RHOA, YWHAB, YWHAE, YWHAG, YWHAH, YWHAZ
ANXA2, B2M, CANX, CCL5, CD36, CDC42, CFL1, DHX9, FLNA, FSCN1, HLA-A, HLA-B, HNRNPA2B1, HN
YWHAB, YWHAE, YWHAG, YWHAH, YWHAZ
CD47, CD99, F11R, FCER1G, INPP5D, ITGA6, ITGB1, ITGB3, PECAM1, PF4V1, PPIA, SELPLG, SPN
ACTB, ANK1, CLTA, HSPA8, ITGA2B, ITGB1, ITGB3, MSN, SPTBN1, TUBA1C, TUBA4A, TUBB1
ACTB, ITGA2B, ITGB3, RAP1B, TLN1, VCL, YWHAB
CALM3, CDC42, FLNA, MYH9, MYL12B, MYL6
CYBB, GSR, GSTP1, PRDX1, PRDX2, PRDX6, SOD1
YWHAB, YWHAE, YWHAG, YWHAH, YWHAZ
YWHAB, YWHAE, YWHAG, YWHAH, YWHAZ
ANXA2, CANX, CCL5, CD36, CDC42, CFL1, FSCN1, HNRNPA2B1, HNRNPF, HSP90AA1, HSPA8, HSPA9,

EEF1A1, EEF2, RPL11, RPL8, RPLP0, RPS15, RPS15A, RPS23, RPS3A, RPSA
ACTB, ITGA2B, ITGB3, RAP1B, TLN1, VCL, YWHAB
B2M, G3BP1, HLA-A, HLA-B, HSP90AA1, RPS15, RPS15A, RPS23, RPS3A, RPSA, YWHAB, YWHAE, YWH
ACTB, ADAM10, ARPC5, CDC42, CFL1, CLTA, MYH9, MYL12B, MYL6, RHOA
EEF1A1, EEF2, RPL11, RPL8, RPLP0, RPS15, RPS15A, RPS23, RPS3A, RPSA
ANXA2, CALM3, MYL12B, MYL6, TLN1, TPM4, VCL
ACTB, CALM3, CDC42, TUBA1C, TUBA4A, TUBB1
CFL1, MYH9, MYL12B, MYL6, RHOA
CFL1, HSP90AA1, ITGB1, MYH9, MYL12B, MYL6, RHOA, TLN1
ACTB, CALM3, ITGA2B, ITGB3, PHB, RAP1B, TLN1, VCL, YWHAB
CD151, ITGA6, KRT14, KRT5
CD47, F11R, ICAM2, ITGA2B, ITGA6, ITGB1, ITGB3, PECAM1, THBS1
ACTB, CALM3, ITGA2B, ITGB3, RAP1B, TLN1, VCL, YWHAB
ACTB, CD151, CD47, F11R, FLNA, ILK, ITGA6, ITGB1, JUP, KRT14, KRT5, SPTBN1
G6PD, GSR, PRDX1, PRDX2, YWHAB, YWHAE, YWHAG, YWHAH, YWHAZ
CCT2, CCT6A, CCT7, HNRNPC, HSP90AA1, VIM
FLNA, GP1BA, GP1BB, YWHAZ
CCT2, CCT6A, CCT7, HNRNPC, HSP90AA1
B2M, CANX, EEF1A1, G3BP1, HLA-A, HLA-B, HSP90AA1, ITGB1, NPM1, PPIA, RPS15, RPS15A, RPS23,
CCT2, CCT4, CCT6A, CCT7, CCT8, TCP1
EEF1A1, RPS15, RPS15A, RPS23, RPS3A, RPSA
ACTB, CD151, F11R, FLNA, ILK, ITGA6, ITGB1, JUP, KRT14, KRT5
GP1BA, GP1BB, ITGA2B, ITGB3, RAP1B, TLN1
BCAP31, DSG1, DSP, FLOT1, HSP90AA1, PSMA5, PSMA6, UBE2L3, VIM, YWHAB, YWHAE, YWHAG, YV
B2M, BCAP31, CANX, CLTA, EEF1A1, G3BP1, H2AC20, H2BC21, HLA-A, HLA-B, HSP90AA1, HSPA1B, H
CANX, CLTA, HSP90AA1, HSPA1B, RPL11, RPL8, RPLP0, RPS15, RPS15A, RPS23, RPS3A, RPSA
CD9, EEF2, HSP90AA1
LTF, S100A8, S100A9
ITGA2B, ITGB3, RAP1B, TLN1
ITGA2B, ITGB3, RAP1B, TLN1
FCER1G, GP1BA, GP1BB, ITGB1
B2M, BCAP31, CANX, HLA-A, HLA-B
ACTB, BCAP31, CCT2, CCT6A, CCT7, CDC42, CYBB, DSG1, DSP, FLOT1, HNRNPC, HSP90AA1, ITGB1, JU
B2M, CD36, CYBB, HLA-A, HLA-B, PSMA5, PSMA6, S100A8, S100A9
YWHAB, YWHAE, YWHAG, YWHAH, YWHAZ
CYBB, EEF1A1, G6PD, GSR, GSTP1, H2AC20, H2BC21, HSP90AA1, HSPA1B, HSPA8, HSPA9, PRDX1, PR
ACTB, CLTA, MSN, TUBA1C, TUBA4A, TUBB1
B2M, CANX, G3BP1, HLA-A, HLA-B, HSP90AA1, RPS15, RPS15A, RPS23, RPS3A, RPSA, YWHAB, YWHA
ACTB, FLNA, ILK, ITGB1
CYB5R3, SLC2A1, SLC2A3
ACTB, ANK1, ARPC5, CALM3, CD36, CD59, CLTA, HSP90AA1, HSPA8, LRP1, M6PR, MYH9, RAB10, RAI
CYBB, EEF1A1, G6PD, GSR, GSTP1, H2AC20, H2BC21, HSP90AA1, HSPA1B, HSPA8, HSPA9, PRDX1, PR
CDC42, PFN1, PSMA5, PSMA6, RHOA, RPL11, RPL8, RPLP0, RPS15, RPS15A, RPS23, RPS3A, RPSA
B2M, CD36, HLA-A, HLA-B, PSMA5, PSMA6, S100A8, S100A9
CALM3, EEF1A1, EEF2, HSPA8
RPL11, RPL8, RPLP0, RPS15, RPS15A, RPS23, RPS3A, RPSA
RPL11, RPL8, RPLP0, RPS15, RPS15A, RPS23, RPS3A, RPSA
MYH9, MYL12B, MYL6, RHOA
ANK1, HSPA8, ITGB1
GSR, RPL11, RPL8, RPLP0, RPS15, RPS15A, RPS23, RPS3A, RPSA

RPL11, RPL8, RPLP0, RPS15, RPS15A, RPS23, RPS3A, RPSA
B2M, BCAP31, CALM3, CANX, CD226, CD36, CD99, CDC42, CLTA, CTSD, CYBB, HLA-A, HLA-B, ICAM2,
CD36, LBP, S100A8, S100A9
MYH9, MYL12B, MYL6, RHOA
HSP90AA1, HSPA1B, HSPA8, TUBA1C, TUBA4A, TUBB1
ACTB, CALM3, CDC42, CYBB, HSP90AA1, ITGB3, JUP, RHOA
EEF1A1, HSP90AA1, HSPA8, VIM
ACTB, ADAM10, ATIC, CALM3, HSP90AA1, ITGA2B, ITGB3, MSN, MYH9, NPM1, PHB, PSMA5, PSMA6
ANTXR2, CALM3, CD9, EEF2, HSP90AA1
DHX9, HNRNPA2B1, HNRNPA3, HNRNPC, HNRNPF, HNRNPH1, HNRNPU, HSPA8, PCBP1, PTBP1, SF3A
RPL11, RPL8, RPLP0, RPS15, RPS15A, RPS23, RPS3A, RPSA
RPL11, RPL8, RPLP0, RPS15, RPS15A, RPS23, RPS3A, RPSA
RPL11, RPL8, RPLP0, RPS15, RPS15A, RPS23, RPS3A, RPSA
DHX9, HSPA1B, HSPA8, NPM1, TUBA1C, TUBA4A, TUBB1
CCT2, CCT7, HNRNPC, VIM
CCT2, CCT4, CCT8, TCP1
BCAP31, DSG1, DSP, PSMA5, PSMA6, VIM, YWHAB, YWHAE, YWHAG, YWHAH, YWHAZ
YWHAB, YWHAG, YWHAZ
B2M, HLA-A, HLA-B
CYBB, RAC2, S100A8, S100A9
DHX9, HNRNPA2B1, HNRNPA3, HNRNPC, HNRNPF, HNRNPH1, HNRNPU, HSPA8, PCBP1, PTBP1, SF3A
ACTB, ARPC5, CDC42, CFL1, HSP90AA1, MYH9
DHX9, HNRNPA2B1, HNRNPA3, HNRNPC, HNRNPF, HNRNPH1, HNRNPU, HSPA1B, HSPA8, NCL, PCBP
MYH9, MYL12B, MYL6, RHOA
ITGA6, ITGB1, PPIA, SPN
ACTB, ARPC5, CDC42, CFL1, RHOA
ACTB, CALM3, CDC42, CYBB, HSP90AA1, ITGB3, JUP, RHOA
EEF1A1, HSP90AA1, YWHAE
INPP5D, ITGB3, PECAM1
HSP90AA1, TUBA1C, TUBA4A, TUBB1, VIM
RPL11, RPL8, RPLP0, RPS15, RPS15A, RPS23, RPS3A, RPSA
RPL11, RPL8, RPLP0, RPS15, RPS15A, RPS23, RPS3A, RPSA
RPL11, RPL8, RPLP0, RPS15, RPS15A, RPS23, RPS3A, RPSA
ITGA2B, ITGB3, RAP1B, TLN1
ITGA6, ITGB1, ITGB3, THBS1
ACTB, ANK1, ARPC5, CALM3, CD59, CLTA, HSPA8, M6PR, MYH9, RAB10, RAB1B, RAB27B, RAB33B, S
ACTB, CDC42, ITGB1, PFN1, RCC2, RHOA, TUBA1C, TUBA4A, TUBB1
RPL11, RPL8, RPLP0, RPS15, RPS15A, RPS23, RPS3A, RPSA
RPL11, RPL8, RPLP0, RPS15, RPS15A, RPS23, RPS3A, RPSA
ENO1, PGK1
ILK, ITGB1
CCT2, CCT4, CCT8, HSP90AA1, TCP1, TUBA1C, TUBA4A, TUBB, TUBB1, YWHAE, YWHAG
ACTB, CLTA, TUBA1C, TUBA4A, TUBB1
PSMA5, PSMA6, RPL11, RPL8, RPLP0, RPS15, RPS15A, RPS23, RPS3A, RPSA
HSP90AA1, HSPA1B, HSPA8
ATP2A3, CALM3, SRI
ATP5F1B, CALM3, CCT2, CCT4, CCT8, HSP90AA1, HSPA9, TCP1, TUBA1C, TUBA4A, TUBB, TUBB1, YW
CALM3, TUBA1C, TUBA4A, TUBB1
HSP90AA1, TUBA1C, TUBA4A, TUBB, TUBB1, YWHAE, YWHAG
HSP90AA1, RPL11, RPL8, RPLP0, RPS15, RPS15A, RPS23, RPS3A, RPSA

RPL11, RPL8, RPLP0, RPS15, RPS15A, RPS23, RPS3A, RPSA
RPL11, RPL8, RPLP0, RPS15, RPS15A, RPS23, RPS3A, RPSA
MYH9, MYL12B, MYL6, RHOA
ACTB, CLTA, TUBA1C, TUBA4A, TUBB1
RPS15, RPS15A, RPS23, RPS3A, RPSA
RAP1B, YWHAB, YWHAZ
RPS15, RPS15A, RPS23, RPS3A, RPSA
HSPD1, NPM1
CYBB, G6PD, GSR, GSTP1, PRDX1, PRDX2, PRDX6, PSMA5, PSMA6, SOD1, TKT
ANK1, CD59, RAB1B, SPTBN1, TUBA1C, TUBA4A, TUBB1
HSP90AA1, PSMA5, PSMA6, TUBA1C, TUBA4A, TUBB1
YWHAB, YWHAE, YWHAG, YWHAH, YWHAZ
ALDOA, ENO1, GAPDH, PGK1
CDC42, FCER1G, RAC2, RHOA
CD36, S100A8, S100A9
TUBA1C, TUBA4A, TUBB1
HSP90AA1, HSPA8, TUBA1C, TUBA4A, TUBB1, VIM
GP1BA, GP1BB
ACTB, ADAM10, ANK1, ANPEP, ATP5F1B, B2M, CALM3, CANX, CCT2, CCT4, CCT6A, CCT7, CCT8, CD5
CD36, FSCN1, HSP90AA1, HSPA8, ITGB1, LBP, VIM
CD36, S100A8, S100A9
TUBA1C, TUBA4A, TUBB1
ANTXR2, B2M, CALM3, CD9, EEF2, ENO1, HSP90AA1, LTF, PGK1
RPS15, RPS15A, RPS23, RPS3A, RPSA
RPS15, RPS15A, RPS23, RPS3A, RPSA
ADAM10, B2M, H2AC20, H2BC21, LTF, LYZ, TSPAN14
BCAP31, DSG1, DSP, VIM
RPS15, RPS15A, RPS23, RPS3A, RPSA
ACTB, ARPC5, CDC42, CFL1, HSP90AA1, MYH9
GP1BA, GP1BB
GP1BA, GP1BB
ITGA2B, ITGB1, ITGB3
ANXA2, ATP2A3, CALM3, MYL12B, MYL6, SRI, TLN1, TPM4, VCL, VIM
HSPA1B, HSPA8, PSMA5, PSMA6, YWHAB, YWHAZ
EEF1A1, HSP90AA1, HSPA1B, HSPA8, HSPA9, YWHAE
CALM3, CLTA, GNG5, PFN1, PSMA5, PSMA6, RAC2, RHOA
PRDX1, PRDX2, YWHAE
PRDX1, PRDX2, YWHAE
TUBA1C, TUBA4A, TUBB1
TTLL12, TUBA1C, TUBA4A, TUBB1
ATIC, MSN, MYH9, NPM1, TPM4, VCL
ATIC, MSN, MYH9, NPM1, TPM4, VCL
CLTA, PFN1, PSMA5, PSMA6, RAC2, RHOA
ACTB, ADAM10, CALM3, CDC42, CLTA, CTSD, CYBB, HNRNPF, HNRNPH1, HSP90AA1, ITGB1, ITGB3, J
GP1BA, GP1BB
G6PD, TKT
CD36, CYBB
ACTB, CALM3, ITGA2B, ITGB3, PHB, PSMA5, PSMA6, RAP1B, SPTBN1, TLN1, VCL, YWHAB
H2BC21, YWHAB, YWHAE, YWHAG, YWHAH, YWHAZ
HSP90AA1, HSPA1B, HSPA8

DHX9, HNRNPA2B1, HNRNPA3, HNRNPC, HNRNPF, HNRNPH1, HNRNPU, HSPA8, PCBP1, PTBP1, SF3A
DHX9, FLNA, HSPA1B, HSPA8, NPM1, TUBA1C, TUBA4A, TUBB1
ACTB, CALM3, ITGA2B, ITGB3, PHB, PSMA5, PSMA6, RAP1B, SPTBN1, TLN1, VCL, YWHAB
NCL, RPL11, RPL8, RPLP0, RPS15, RPS15A, RPS23, RPS3A, RPSA
HSP90AA1, TUBA4A, TUBB, YWHAE, YWHAG
HSP90AA1, TUBA4A, TUBB, YWHAE, YWHAG
G6PD, GSR, PRDX1, PSMA5, PSMA6, TKT
GP1BA, GP1BB
RHOA, RTN4
PRDX1, PRDX2, YWHAE
RPL11, RPL8, RPLP0, RPS15, RPS15A, RPS23, RPS3A, RPSA
ACTB, CALM3, CDC42, ITGA2B, ITGB3, PHB, PSMA5, PSMA6, RAP1B, SPTBN1, TLN1, VCL, YWHAB
CAMP, DCD, LTF, LYZ, S100A8, S100A9
HNRNPF, HNRNPH1, PTBP1
ALDOA, ENO1, GAPDH, PGK1, PKM
HSP90AA1, TUBA4A, TUBB, YWHAE, YWHAG
FLOT1, JUP, RHOA, STOM, TFRC
RHOA, RTN4
ACTB, ADAM10, ARPC5, ATP2A3, BCAP31, CALM3, CCL5, CCT2, CCT6A, CCT7, CDC42, CFL1, CLTA, CT
ADAM10, CD151, CD47, CTSD, F11R, ICAM2, ITGA2B, ITGA6, ITGB1, ITGB3, PECAM1, THBS1
NCL, RPL11, RPL8, RPLP0, RPS15, RPS15A, RPS23, RPS3A, RPSA
HSP90AA1, PSMA5, PSMA6, TUBA1C, TUBA4A, TUBB, TUBB1, YWHAE, YWHAG
B2M, CD226, CD99, HLA-A, HLA-B, ICAM2, ITGB1
HSP90AA1, PSMA5, PSMA6, TUBA1C, TUBA4A, TUBB, TUBB1, YWHAE, YWHAG
CCT2, CCT4, CCT8, TCP1
H2BC21, PSMA5, PSMA6, YWHAB, YWHAE, YWHAG, YWHAH, YWHAZ
DSG1, DSP
B2M, HLA-A
ADAM10, YWHAZ
ACTB, CLTA
CDC42, RHOA
CD36, GNAQ
CALM3, PPIA
CALM3, HSP90AA1, JUP
CD36, GNAI2, GNAQ, GNG5, SLC2A1
BCAP31, DSG1, DSP, VIM
GALE
PTGS1
SLC2A1
CD36, GNAI2, GNAQ, GNG5, SLC2A1, TKT
ATP2A3, CALM3, SRI
B2M, DHX9, FLNA, HLA-A, HLA-B, HSPA1B, HSPA8, NPM1, TUBA1C, TUBA4A, TUBB1
ACTB, MSN, MYH9, SPTBN1
CALM3, HSP90AA1
ACTB, CLTA
CALM3, HSP90AA1
TUBA1C, TUBA4A, TUBB1
ACTB, ANK1, SPTBN1
NCL, RPL11, RPL8, RPLP0, RPS15, RPS15A, RPS23, RPS3A, RPSA
CDC42, JUP, STOM, TFRC

HSP90AA1, TUBA4A, TUBB, YWHAE, YWHAG
HSP90AA1, TUBA4A, TUBB, YWHAE, YWHAG
HSPA1B, HSPA8, PSMA5, PSMA6
CDC42, GNG5, RHOA
FLOT1, HSP90AA1, UBE2L3
CALM3, SRI
ACTB, ARPC5, CLTA, HSPA8, M6PR, TFRC, UBQLN1
CD36, S100A8, S100A9
CD36, S100A8, S100A9
GNAI2, GNAQ, GNG5
FLOT1, HSP90AA1, UBE2L3
CDC42, JUP, STOM, TFRC
ITGA6, ITGB1, ITGB3, THBS1
PPIA, XRCC5
CDC42, CYBB, ITGB1, RAC2, TFRC
HSP90AA1, TUBA4A, TUBB, YWHAE, YWHAG
ATP2A3, CALM3, GNG5, PECAM1, SRI
EEF1A1, HSP90AA1, HSPA8, TUBA1C, TUBA4A, TUBB1, VIM
CLTA, HSPA8, M6PR
EEF2, EIF5A, RPL8, RPS23
ACTB, ARPC5, CDC42
TUBA1C, TUBA4A, TUBB1
CALM3, PHB, YWHAB
ANK1, CD59, RAB1B, SPTBN1, TUBA1C, TUBA4A, TUBB1
ACTB, ARPC5, CDC42, MYH9
ACTB, ARPC5, CDC42, MYH9
ACTB, ARPC5, CDC42, MYH9
G6PD, TKT
CANX, CLTA, CTSD, TUBA1C, TUBA4A, TUBB1
ALDOA, ENO1, GAPDH, PGK1, PKM
HSPA8
LBP
TKT
ANTXR2, CALM3
CFL1, HSP90AA1
CALM3, HSP90AA1
GP1BA, GP1BB
F11R, RHOA
JUP, STOM, TFRC
CDC42, SPTBN1, TPM4
GP1BA, H2AC20, H2BC21, ITGA2B, THBS1
G6PD, GSR, PRDX1, PSMA5, PSMA6, TKT
RAB10, RAB1B, RAB27B, RAB33B
GP1BA, GP1BB, PF4V1
HSP90AA1, TUBA4A, TUBB, YWHAE, YWHAG
TUBA1C, TUBA4A, TUBB1
HSPA1B, HSPA8, HSPA9, YWHAE
ACTB, MSN, MYH9, SPTBN1
ANPEP, CTSD
ACTB, ARPC5, CALM3, CDC42, GNAI2, GNG5, MYH9

ACTB, ARPC5, CALM3, CDC42, GNAI2, GNG5, MYH9
CD36, HSP90AA1, LRP1
FLOT1, JUP, STOM, TFRC
HSP90AA1, RCC2, TUBA1C, TUBA4A, TUBB, TUBB1, YWHAE, YWHAG
GP1BA, GP1BB
GSR, PRDX1
HSP90AA1, HSPA8
HSP90AA1, HSPA8, TUBA1C, TUBA4A, TUBB1, VIM
CLTA, HSPA8, M6PR, TFRC
B2M, HLA-A, NPM1, PPIA, PSMA5, PSMA6
CD36
SLC2A1
HSP90AA1
RPL8, RPS23
YWHAB, YWHAE
CDC42, GNG5
NPM1, TNKS1BP1
GNAI2, GNG5, ITGB1
FLOT1, RTN3
CALM3, HSP90AA1
PPIA, XRCC5
SLC2A1, SLC2A3
H2AC20, H2BC21, PRDX1, PRDX2, YWHAE
ACTB, MSN, MYH9, SPTBN1
CALM3, GNAI2, GNG5, HSP90AA1
EEF1A1, EEF2, RPL11, RPL8, RPLP0, RPS15, RPS15A, RPS23, RPS3A, RPSA
CALM3, CLTA, GNG5, H2AC20, H2BC21, PFN1, PSMA5, PSMA6, RAC2, RHOA, YWHAZ
B2M, HLA-A
GNAI2, GNG5
H2AC20, H2BC21, HSP90AA1, PSMA5, PSMA6, RAB1B, RCC2, TUBA1C, TUBA4A, TUBB, TUBB1, YWH
BCAP31, FLOT1, JUP, RHOA, STOM, TFRC
PSMA5, PSMA6, TUBA1C, TUBA4A, TUBB1
CALM3, TUBA1C, TUBA4A, TUBB1
PHB, VDAC2
GP1BA, GP1BB
ALDOA, CALM3, ENO1, G6PD, GALE, GAPDH, PGK1, PKM, SLC2A1, TKT
HSP90AA1
HSP90AA1
HSP90AA1
HSP90AA1
HSP90AA1
HSP90AA1
HSP90AA1
HSP90AA1
HSP90AA1
HSP90AA1
HSP90AA1
EIF5A
GALE
GSR

HSP90AA1
RHOA
RHOA
ANK1, CD59, RAB1B, SPTBN1, TUBA1C, TUBA4A, TUBB1
B2M, BCAP31, CANX, CD36, CYBB, HLA-A, HLA-B, PSMA5, PSMA6, S100A8, S100A9, UBE2L3
ADAM10, PSMA5, PSMA6, YWHAZ
CALM3, GNAI2, GNG5, MYH9
CALM3, GNAI2, GNG5, MYH9
TUBA1C, TUBA4A, TUBB1
ACTB, ADAM10, CLTA
GNAQ, GNG5
GNAQ, GNG5
CALM3, PPIA, PSMA5, PSMA6
GNG5, RHOA
GNAQ, GNG5
PSMA5, PSMA6, YWHAZ
TUBA1C, TUBA4A, TUBB1
FCN1
CD9
SACM1L
CANX
ITGB1
CLTA, HSPA8, TFRC
ATP2A3, CALM3, SRI
CALM3, GNAI2, GNAQ
RAB10, RAB1B, RAB27B, RAB33B, YWHAE
ATP2A3, CALM3, SRI
RAB10, RAB1B, RAB27B, YWHAE
GNAI2, GNG5
CALM3, INPP5D
CD59, CPN1, FCN1
G6PD, GSR, NPM1, PRDX1, PRDX2, TNKS1BP1, YWHAB, YWHAE, YWHAG, YWHAH, YWHAZ
B2M, G3BP1, HLA-A, HLA-B, HSP90AA1
CALM3, GNAI2, GNAQ, GNG5
B2M, HLA-A
TUBA1C, TUBA4A, TUBB1
HSP90AA1
ITGB1
PPIA
PPIA
PPIA
YWHAE
CD36
HSP90AA1
CALM3
CALM3
CALM3, TUBA1C, TUBA4A, TUBB1
CDC42, CYBB, ITGB1, TFRC
F11R, ITGB1, ITGB3, RHOA
ITGA6, ITGB1

FLOT1, HSP90AA1, UBE2L3
ATP5F1B, HSPA9
LDHA, LDHB
H2BC21, PSMA5, PSMA6, RCC2, YWHAB, YWHAE, YWHAG, YWHAH, YWHAZ
GSR, PSMA5, PSMA6, RPL11, RPL8, RPLP0, RPS15, RPS15A, RPS23, RPS3A, RPSA
JUP
ADAM10
ADAM10
ANK1
YWHAB
JUP
PPIA
DHX9
ANK1
CANX
GNAQ, GNG5
CALM3, GNG5
RAB1B, TUBA1C, TUBA4A, TUBB1
YWHAB, YWHAG, YWHAZ
ATP5F1B, HSPA9, HSPD1
CALM3, FCER1G
ACTB, H2AC20, PSMA5, PSMA6
RHOA
FCN1
EEF2
YWHAB
YWHAB
YWHAB
GNAQ
CDC42
AACS
HSPA1B
HSPA8, VIM
ANK1, CANX, CD59, NANS, RAB1B, SPTBN1, TUBA1C, TUBA4A, TUBB1
CALM3, CTSD, GNAI2, GNG5, H2AC20, H2BC21, HSP90AA1
CYBB, RAC2
TPM4, VIM
CALM3, FCER1G, ICAM2, PSMA5, PSMA6
CLTA, M6PR, TFRC, UBQLN1
HSPA8
CALM3
MYH9
RHOA
RHOA
ITGB1, ITGB3
ARL6IP5, CALM3, GNAI2, GNG5, HSPA8, TUBA1C, TUBA4A, TUBB1
HSPD1, NPM1
PSMA5, PSMA6, TUBA1C, TUBA4A, TUBB1
H2AC20, H2BC21, NPM1
H2AC20, H2BC21, NPM1

HNRNPF, HNRNPH1, PTBP1
PSMA5, PSMA6, RCC2, TUBA1C, TUBA4A, TUBB1
B2M, ENO1, LTF, PGK1
CALM3
PPIA
GNAQ
FLNA
AACS
LTF
CDC42, ITGB1, TFRC
CALM3, PPIA, PSMA5, PSMA6
TUBA1C, TUBA4A, TUBB1
CDC42, SPTBN1
ADAM10, ATP2A3, H2AC20, H2BC21, PSMA5, PSMA6, YWHAZ
CALM3, CYB5R3, HSP90AA1, LRP1, SLC2A1, SLC2A3
ACTB, ADAM10, ANK1, CALM3, CANX, CD59, EEF1A1, EEF2, EIF5A, H2AC20, H2BC21, HLA-A, HNRNP
GP1BA, H2AC20, H2BC21, ITGA2B, PSMA5, PSMA6, THBS1
ITGA2B, ITGB1, ITGB3
ITGB1, RAP1B
JUP
CANX
ATIC
HSP90AA1
CLTA
PPIA
RAP1B
CANX
B2M, HLA-A, NPM1, PPIA, PSMA5, PSMA6, XRCC5
DSG1, DSP
DSP, TFRC
RCC2, TUBA1C, TUBA4A, TUBB1
ITGB1, PTPRJ, RAP1B
GNAI2, GNG5
GNAI2, GNG5
GNAI2, GNG5
DSG1, TFRC
CANX
CALM3
YWHAB
XRCC5
VIM
NPM1
ADH5
CALM3
CALM3
CDC42
M6PR, RAB1B, RAB33B, TUBA1C, TUBA4A, TUBB1
INPP5D, PSMA5, PSMA6, PTPRJ
H2AC20, H2BC21, HSP90AA1, NPM1, PSMA5, PSMA6, RAB1B, RCC2, TUBA1C, TUBA4A, TUBB, TUBB
ITGB1, ITGB3

FCER1G, PSMA5, PSMA6
B2M, HLA-B
CALM3, GNAI2, GNG5, TUBA1C, TUBA4A, TUBB1
CALM3
XRCC5
CLTA
LRP1
ANXA2
BCAP31, HSP90AA1, HSPA8, NCL
ENO1, PGK1
F11R, ITGB1, ITGB3, RHOA
CD59, CPN1
ITGB1, ITGB3
RCC2, TUBA1C, TUBA4A, TUBB1
ACTB, CDC42, TUBA1C, TUBA4A, TUBB1
CLTA
TLN1
FCN1
ATIC
GNAI2
CALM3
YWHAB
HMBS
RHOA
CDC42
CALM3
CALM3
CALM3
CALM3
RAB1B
B2M
NPM1, TNKS1BP1
GNAI2, GNG5
INPP5D, YWHAZ
PSMA5, PSMA6
ACTB, F11R, JUP
H2AC20, H2BC21, PSMA5, PSMA6
PTPRJ
GALE
ADAM10
ADAM10
RHOA
PTGS1
HSP90AA1
HSP90AA1
CLTA
CDC42, PSMA5, PSMA6
HNRNPF, HNRNPH1, PTBP1
HSP90AA1, RHOA
PSMA5, PSMA6

CALM3, INPP5D
ACTB, H2AC20, H2BC21
PSMA5, PSMA6
H2AC20, H2BC21
ARL6IP5, HSPA8
CALM3, GNAQ
RAB1B, TUBA1C, TUBA4A, TUBB1
CLTA
CD47
PHB
HSP90AA1
XRCC5
CALM3
FCER1G
PSMA5, PSMA6
PSMA5, PSMA6
PSMA5, PSMA6
PSMA5, PSMA6
PSMA5, PSMA6
PSMA5, PSMA6
CALM3, FCER1G, PSMA5, PSMA6
PSMA5, PSMA6
PSMA5, PSMA6
ADAM10, CDC42
DHX9
YWHAB
YWHAB
YWHAZ
SLC2A3
PECAM1
ATP5F1B, CALM3, HSPA9
B2M, HLA-A, HLA-B
PSMA5, PSMA6
PSMA5, PSMA6
PSMA5, PSMA6
PSMA5, PSMA6
PSMA5, PSMA6
PSMA5, PSMA6
H2AC20, H2BC21
H2AC20, H2BC21
H2AC20, H2BC21
LDHA, LDHB
ARL6IP5, CALM3, FLOT1, GNAI2, GNG5, HSPA8, RTN3, TUBA1C, TUBA4A, TUBB1
ATP2A3
ATP5F1B
GNG5
SACM1L
RHOA, RTN4, YWHAE
PSMA5, PSMA6
ATP5F1B, CALM3

H2AC20, H2BC21, PSMA5, PSMA6
ACTB, H2AC20, H2BC21, KANSL3
INPP5D, PSMA5, PSMA6
ATP5F1B, HSPA9, HSPD1
ACTB, JUP
PSMA5, PSMA6
PSMA5, PSMA6
HSP90AA1, ITGB1, TUBB
ITGB1
SPTBN1
CLTA
GNG5
HSPA8
HSP90AA1
HSP90AA1
CALM3, PSMA5, PSMA6
H2AC20, H2BC21, HSP90AA1, PSMA5, PSMA6, RAB1B, RCC2, TUBA1C, TUBA4A, TUBB, TUBB1, YWH/
PSMA5, PSMA6, RCC2, TUBA1C, TUBA4A, TUBB1
H2AC20, H2BC21, ITGB1, TUBA1C, TUBA4A, TUBB1
PSMA5, PSMA6
PSMA5, PSMA6
PSMA5, PSMA6
HLA-A, UBE2L3
GNG5
CALM3
PSMA5, PSMA6, RCC2, TUBA1C, TUBA4A, TUBB1
PSMA5, PSMA6
PSMA5, PSMA6
PSMA5, PSMA6
PSMA5, PSMA6
PSMA5, PSMA6
H2AC20, H2BC21
H2AC20, H2BC21
H2AC20, H2BC21
GNAI2, GNG5
CD36, LBP, S100A8, S100A9
PSMA5, PSMA6
CD151, ITGA6
PTPRJ
GNG5
ITGB1
CALM3
DHX9
ICAM2
CALM3
CTSD, H2AC20, H2BC21, HSP90AA1
H2AC20, H2BC21
H2AC20, H2BC21
CALM3, GNG5
CALM3, SLC2A3

ADH5, CYB5R3, PTGS1
ACTB, H2AC20, H2BC21
ADAM10
PF4V1
CALM3
HSP90AA1
PTPRJ
CALM3
CALM3
PSMA5, PSMA6
ATP2A3, H2AC20, H2BC21
PSMA5, PSMA6
ADAM10, CTSD
H2AC20, H2BC21
DHX9, XRCC5
FCN1
PPIA
SPTBN1
CALM3, SRI, STOM
PSMA5, PSMA6
CDC42, JUP, STOM, TFRC
RHOA, RTN4, UBE2L3, YWHAE
PSMA5, PSMA6
PSMA5, PSMA6
PSMA5, PSMA6
H2AC20, H2BC21
ACTB, H2AC20, H2BC21, PSMA5, PSMA6, RHOA, VDAC2
NCL
YWHAB
ARL6IP5
TNKS1BP1
TNKS1BP1
CALM3
CALM3, CTSD, GNAI2, GNG5, H2AC20, H2BC21, HSP90AA1
H2AC20, H2BC21, PSMA5, PSMA6
PSMA5, PSMA6
PSMA5, PSMA6
H2AC20, H2BC21
H2AC20, H2BC21
H2BC21, XRCC5
CD59, RAB1B
PSMA5, PSMA6
PSMA5, PSMA6
H2AC20, H2BC21
ADAM10
HSP90AA1
CD9
HSP90AA1
CD36, S100A8, S100A9
CD36, S100A8, S100A9

PSMA5, PSMA6
PSMA5, PSMA6
CLTA, RHOA, YWHAB
CALM3
CANX
HSP90AA1
H2AC20, H2BC21
H2AC20, H2BC21, TNKS1BP1
CD36, S100A8, S100A9
CD36, S100A8, S100A9
PSMA5, PSMA6
CALM3
HMBS
INPP5D
CALM3
PSMA5, PSMA6
PSMA5, PSMA6
H2AC20, H2BC21
H2AC20, H2BC21
H2AC20, H2BC21
PSMA5, PSMA6
PSMA5, PSMA6
PSMA5, PSMA6
PNPLA8
JUP
SPTBN1
CD36, LBP, S100A8, S100A9
GSTP1
HSP90AA1
FCER1G
GNG5
GNG5
GNG5
F11R
HSP90AA1
CALM3
PSMA5, PSMA6
PSMA5, PSMA6
H2AC20, H2BC21, PSMA5, PSMA6, VDAC2
PSMA5, PSMA6
H2AC20, H2BC21
CDC42
PNPLA8
B2M
GSR
CTSD
ITGB1
UBE2L3
CANX
HLA-A, HLA-B

PSMA5, PSMA6
H2AC20, H2BC21
JUP
JUP
ADAM10
CDC42
TFRC
INPP5D
CYB5R3, SLC2A1, SLC2A3
H2AC20, H2BC21
HLA-A, UBE2L3
FCER1G
CALM3
GNG5, RHOA
PSMA5, PSMA6
CDC42
NANS
ADAM10
CD59
NPM1
GNG5
GNG5
CALM3
AACS, ADH5, ALDOA, ATIC, ATP5F1B, CALM3, CD36, CYB5R3, ENO1, G6PD, GALE, GAPDH, GNAI2, GN
ATP2A3, CALM3, SRI
H2AC20, H2BC21, PSMA5, PSMA6, YWHAZ
PSMA5, PSMA6
PSMA5, PSMA6
OSBPL2
NPM1
YWHAB
CALM3
INPP5D, SACM1L
CLTA, RHOA, YWHAB
GNG5
CANX
CDC42, CYBB, ITGB1, TFRC
PSMA5, PSMA6
H2AC20, H2BC21
PSMA5, PSMA6
ATP2A3, CALM3, SRI, STOM
H2AC20, H2BC21, PSMA5, PSMA6
FLOT1, RTN3
PPIA
CALM3
GSTP1
H2AC20, H2BC21, HSP90AA1
PSMA5, PSMA6
PSMA5, PSMA6
H2AC20, H2BC21, ITGB1, TUBA1C, TUBA4A, TUBB1

ACTB, H2AC20, H2BC21, KANSL3
CALM3
CLTA
H2AC20
THBS1
CANX
CALM3
CALM3
NPM1
H2AC20, H2BC21, NPM1
ANPEP, CTSD
H2AC20, H2BC21
PTPRJ
ACTB
THBS1
H2AC20, H2BC21, RAB1B
H2AC20, H2BC21
H2AC20, H2BC21
CD151, ITGA6
RHOA
CALM3
HSPA1B
M6PR
H2AC20, H2BC21
PSMA5, PSMA6
H2AC20, H2BC21
H2AC20, H2BC21
YWHAB
KANSL3
ACTB
H2BC21
GNG5
PPIA
YWHAZ
PSMA5, PSMA6
CALM3
GNG5
CALM3
YWHAB
LRP1
RAB33B
INPP5D
ATIC, GSR
OSBPL2
NPM1
CD9, H2AC20, H2BC21
RAB33B
ITGB1
PSMA5, PSMA6
PSMA5, PSMA6

HNRNPC
CCL5
GNAI2, GNG5, ITGB1
CALM3, LRP1
LRP1
H2AC20, H2BC21
ADAM10
M6PR
TLN1
UBE2L3
CDC42
ADAM10, QSOX1
H2AC20, H2BC21
CALM3, SRI
ATP5F1B, HSPA9, HSPD1
H2AC20, H2BC21
H2AC20, H2BC21
TLN1
GNG5
ADH5, CYB5R3, GSTP1, PTGS1
H2AC20, H2BC21
INPP5D
H2AC20, H2BC21
H2AC20, H2BC21
RHOA
RHOA
TNKS1BP1
LTF
PSMA5, PSMA6
H2AC20, H2BC21
PSMA5, PSMA6, SLC2A1
ADAM10
ADAM10
ADAM10
ADAM10
ADAM10
TFRC
CANX
M6PR
THBS1
ATP5F1B, LDHA, LDHB
PSMA5, PSMA6
UBE2L3
CCL5
PTGS1
PSMA5, PSMA6
H2AC20, H2BC21
H2AC20, H2BC21
ATP2A3, CALM3, CLTA, GNG5, PHB, PSMA5, PSMA6, SLC2A1, SLC2A3, SRI, STOM, TFRC, VDAC2
ADAM10, QSOX1

NANS
SPTBN1
PSMA5, PSMA6
H2AC20, H2BC21
CALM3, SLC2A1, SLC2A3, SRI
PSMA5, PSMA6
CDC42
THBS1
CANX
CALM3, CCL5, GNAI2, GNAQ, GNG5
PSMA5, PSMA6
ADAM10, CTSD
ADAM10
CLTA
YWHAE
GALE, THBS1
H2BC21
ACTB, H2AC20, H2BC21, KANSL3
ACTB, H2AC20, H2BC21, KANSL3
NANS
H2BC21
H2AC20
RAC2
INPP5D, PNPLA8, SACM1L
PSMA5, PSMA6
HSP90AA1
CTSD
PSMA5, PSMA6
CD36
ACTB
PPIA, XRCC5
CALM3, G6PD, GP1BA, GSR, H2AC20, H2BC21, HSPD1, ITGA2B, NPM1, PRDX1, PRDX2, PSMA5, PSM
PSMA5, PSMA6
H2BC21, XRCC5
TLN1
GNG5
RCC2
RCC2
H2BC21
SLC2A1
BCAP31
CALM3, PSMA5, PSMA6, RAC2
GNG5
RAC2
M6PR
RAC2
HNRNPC, NPM1
GSTP1
GSTP1
ACTB

HNRNPC, NPM1
RCC2
THBS1
RAC2
ACTB, H2AC20, H2BC21, XRCC5
CD36
PSMA5, PSMA6, RAC2
H2AC20, H2BC21
CD36
H2AC20, H2BC21
ATP5F1B
PNPLA8
CALM3, G6PD, GP1BA, GSR, H2AC20, H2BC21, HSPD1, ITGA2B, NPM1, PRDX1, PRDX2, PSMA5, PSM
ACTB, CALM3, G6PD, GP1BA, GSR, H2AC20, H2BC21, HSP90AA1, HSPD1, ITGA2B, KANSL3, NPM1, P
H2BC21
GNAQ, GNG5
H2BC21
CALM3, CCL5, CDC42, GNAI2, GNAQ, GNG5, ITGB1, RHOA
PPIA
PSMA5, PSMA6, UBE2L3
OSBPL2
PSMA5, PSMA6
GALE, THBS1
PTGS1
CALM3, CCL5, CDC42, GNAI2, GNAQ, GNG5, ITGB1, RHOA
CCL5
ACTB, CALM3, LRP1, MSN, MYH9, SPTBN1
AACS, CD36, INPP5D, M6PR, OSBPL2, PNPLA8, PTGS1, SACM1L
CCL5
CCL5, GNG5

, CYB5R3, CYBB, DSC1, DSG1, DSP, EEF1A1, EEF2, FCER1G, FCN1, FLG2, GSTP1, HLA-B, HRNR, HSP90AA1, I
GNG5, GP1BA, GP1BB, ITGA2B, ITGB3, LGALS3BP, MMRN1, PECAM1, PFN1, PLEK, PPIA, QSOX1, RAB27B,
1R, FCER1G, FERMT3, FLNA, GNAI2, GNAQ, GNG5, GP1BA, GP1BB, INPP5D, ITGA2B, ITGA6, ITGB1, ITGB3,
RN1, PECAM1, PFN1, PLEK, PPIA, QSOX1, RAB27B, SOD1, SRGN, TAGLN2, THBS1, TLN1, TUBA4A, VCL
RN1, PECAM1, PFN1, PLEK, PPIA, QSOX1, RAB27B, SOD1, SRGN, TAGLN2, THBS1, TLN1, TUBA4A, VCL
3, CD59, CDC42, CFL1, CPN1, CTSD, CYB5R3, CYBB, DCD, DHX9, DSC1, DSG1, DSP, EEF1A1, EEF2, FCER1G,
CT8, CD226, CD36, CD47, CD53, CD59, CD99, CDC42, CFL1, CLTA, CPN1, CTSD, CYB5R3, CYBB, DCD, DHX9

2AC20, H2BC21, HNRNPC, HSP90AA1, ITGB1, JUP, MYH9, MYL12B, MYL6, PFN1, RAC2, RCC2, RHOA, S100

2AC20, H2BC21, HNRNPC, HSP90AA1, ITGB1, JUP, MYH9, MYL12B, MYL6, PFN1, RAC2, RCC2, RHOA, S100
FN1, RAC2, RCC2, RHOA, S100A8, S100A9, TUBA1C, TUBA4A, TUBB1, YWHAB, YWHAE, YWHAG, YWHAH,

MYH9, MYL12B, MYL6, PFN1, PSMA5, PSMA6, RHOA, RPL11, RPL8, RPLP0, RPS15, RPS15A, RPS23, RPS3A

MYH9, MYL12B, MYL6, PFN1, PSMA5, PSMA6, RHOA, RPL11, RPL8, RPLP0, RPS15, RPS15A, RPS23, RPS3A
21, HSP90AA1, HSPA8, ITGA2B, ITGB1, ITGB3, JUP, KRT1, KRT10, KRT14, KRT15, KRT16, KRT17, KRT2, KRT

A1, EEF2, ENO1, G3BP1, GALE, GNAI2, GNG5, GP1BA, GP1BB, H2AC20, H2BC21, HLA-A, HLA-B, HSP90AA

1, GNAI2, GNG5, H2AC20, H2BC21, HLA-A, HLA-B, HSP90AA1, HSPA1B, HSPA8, ITGB1, LTF, MYH9, NCL, N

RNPF, HSP90AA1, HSPA1B, HSPA8, HSPA9, INPP5D, ITGB1, LBP, MSN, NPM1, PPIA, PSMA5, PSMA6, PTPR

SPA8, ITGB1, NCL, NPM1, PPIA, PSMA5, PSMA6, RPL11, RPL8, RPLP0, RPS15, RPS15A, RPS23, RPS3A, RPS

DX2, PRDX6, PSMA5, PSMA6, RPL11, RPL8, RPLP0, RPS15, RPS15A, RPS23, RPS3A, RPSA, SOD1, TKT, TLN

31B, RAB27B, RAB33B, SPTBN1, TFRC, TUBA1C, TUBA4A, TUBB1, UBQLN1, YWHAB, YWHAE, YWHAG, YW
DX2, PRDX6, PSMA5, PSMA6, RPL11, RPL8, RPLP0, RPS15, RPS15A, RPS23, RPS3A, RPSA, SOD1, TKT, TLN

INPP5D, ITGB1, PPIA, PSMA5, PSMA6, PTPRJ, RAP1B, S100A8, S100A9, TUBA1C, TUBA4A, TUBB1, UBE2L

1, PSMA5, PSMA6, PTBP1, RPL11, RPL8, RPLP0, RPS15, RPS15A, RPS23, RPS3A, RPSA, SF3A3, TNKS1BP1, \

PTBN1, TFRC, TUBA1C, TUBA4A, TUBB1, UBQLN1, YWHAB, YWHAE, YWHAG, YWHAH, YWHAZ

9, CTSD, EEF1A1, EEF2, EIF5A, GNAQ, GNG5, H2AC20, H2BC21, HLA-A, HNRNPC, HSPA8, HSPA9, HSPD1, L

SD, CYBB, DSG1, DSP, F11R, FLNA, FLOT1, GNAI2, GNAQ, GNG5, H2AC20, H2BC21, HNRNPC, HNRNPF, HN

C, HSPA8, NANS, NPM1, PSMA5, PSMA6, QSOX1, RAB10, RAB1B, RAB27B, RAB33B, RHOA, RPL8, RPS23, S

NAQ, GNG5, GSR, GSTP1, HMBS, HSP90AA1, INPP5D, LDHA, LDHB, LRP1, M6PR, OSBPL2, PGK1, PKM, PNP

RDX1, PRDX2, PSMA5, PSMA6, SLC2A3, THBS1, TNKS1BP1, YWHAB, YWHAE, YWHAG, YWHAH, YWHAZ

HSPA1B, HSPA8, JUP, KRT1, LTF, LYZ, PECAM1, PKM, PPIA, PRDX6, PSMA5, PTPRJ, QSOX1, RAB10, RAP1B,

LGALS3BP, MMRN1, PECAM1, PF4V1, PFN1, PLEK, PPIA, QSOX1, RAB27B, RAC2, RAP1B, RHOA, SELPLG,

, FCN1, FLG2, GSTP1, HLA-B, HRNR, HSP90AA1, HSPA1B, HSPA8, ICAM2, JUP, KRT1, LBP, LTF, LYZ, MYH9,

, DSC1, DSG1, DSP, EEF1A1, EEF2, FCER1G, FCN1, FLG2, FLNA, FSCN1, GSTP1, HLA-A, HLA-B, HNRNPA2B1

A8, S100A9, SPTBN1, STOM, TFRC, TPM4, TUBA1C, TUBA4A, TUBB1, VIM, YWHAB, YWHAE, YWHAG, YW

A8, S100A9, SPTBN1, STOM, TFRC, TPM4, TUBA1C, TUBA4A, TUBB1, VIM, YWHAB, YWHAE, YWHAG, YW

5, KRT6A, KRT6B, KRT78, KRT9, MSN, MYH9, MYL12B, MYL6, PFN1, PSMA5, PSMA6, RHOA, RPL11, RPL8,

1, HSPA1B, HSPA8, ITGA2B, ITGB1, ITGB3, LTF, MSN, MYH9, NCL, NPM1, PGK1, PHB, PPIA, PRDX1, PRDX2

PM1, PGK1, PPIA, PSMA5, PSMA6, RPL11, RPL8, RPLP0, RPS15, RPS15A, RPS23, RPS3A, RPSA, TUBA1C,

TF, LYZ, NANS, NPM1, PSMA5, PSMA6, QSOX1, RAB10, RAB1B, RAB27B, RAB33B, RHOA, RPL11, RPL8, RP

ıRNPH1, HSP90AA1, ITGA2B, ITGB1, ITGB3, JUP, MYH9, MYL12B, MYL6, PFN1, PHB, PSMA5, PSMA6, PTBI

LA8, PSMA5, PSMA6, PTGS1, RPL11, RPL8, RPLP0, RPS15, RPS15A, RPS23, RPS3A, RPSA, SACM1L, SLC2A1

PECAM1, PKM, PPIA, PRDX6, PSMA5, PSMA6, PTPRJ, QSOX1, RAB10, RAC2, RAP1B, RHOA, S100A8, S100/
., HNRNPF, HRNR, HSP90AA1, HSPA1B, HSPA8, HSPA9, ICAM2, INPP5D, ITGB1, JUP, KRT1, LBP, LTF, LYZ, N

RPLP0, RPS15, RPS15A, RPS23, RPS3A, RPSA, SPTBN1, TLN1, TNKS1BP1, TUBA1C, TUBA4A, TUBB1

:, PSMA5, PSMA6, RAC2, RAP1B, RPL11, RPL8, RPLP0, RPS15, RPS15A, RPS23, RPS3A, RPSA, S100A8, S100

LP0, RPS15, RPS15A, RPS23, RPS3A, RPSA, SPTBN1, TCP1, THBS1, TSPAN14, TTLL12, TUBA1C, TUBA4A, TU

P1, PTPRJ, RAC2, RAP1B, RCC2, RHOA, RTN4, S100A8, S100A9, SPTBN1, STOM, TFRC, THBS1, TLN1, TPM4,

MSN, MYH9, NPM1, PECAM1, PKM, PPIA, PRDX6, PSMA5, PSMA6, PTPRJ, QSOX1, RAB10, RAC2, RAP1B, R

A9, SLC2A1, SPTBN1, THBS1, TLN1, TPM4, TUBA1C, TUBA4A, TUBB, TUBB1, VCL, XRCC5, YWHAB, YWHAE

HOA, RPLP0, S100A8, S100A9, SLC2A3, SOD1, STOM, TCP1, TSPAN14, TUBA1C, TUBA4A, TUBB, TUBB1, U

BE2L3, VCL, VIM, XRCC5, YWHAB, YWHAZ

Table 5. Gene Ontology terms associated with non-T-cell proteins identified in CD3(+)sEV

GO ID	Gene Ontology term	# all protei	# detected	p.val
GO:0005198	structural molecule activity	819	27	1.24E-20
GO:0005201	extracellular matrix structural constituent	173	15	1.08E-17
GO:0062023	collagen-containing extracellular matrix	433	18	1.97E-15
GO:0031012	extracellular matrix	573	19	1.74E-14
GO:0030312	external encapsulating structure	574	19	1.80E-14
GO:0005581	collagen trimer	86	10	2.11E-13
GO:0005576	extracellular region	4648	41	1.92E-11
GO:0005615	extracellular space	3652	36	4.09E-11
GO:0071944	cell periphery	6372	45	1.82E-09
GO:0030020	extracellular matrix structural constituent conferring te	41	6	4.09E-09
GO:0007155	cell adhesion	1539	21	1.06E-08
GO:0048407	platelet-derived growth factor binding	11	4	3.44E-08
GO:0001867	complement activation, lectin pathway	12	4	5.14E-08
GO:0043589	skin morphogenesis	12	4	5.14E-08
GO:0009888	tissue development	1990	22	1.87E-07
GO:0005509	calcium ion binding	710	13	4.42E-07
GO:0098644	complex of collagen trimers	22	4	7.41E-07
GO:0005788	endoplasmic reticulum lumen	312	9	7.99E-07
GO:0005604	basement membrane	99	6	8.78E-07
GO:0009653	anatomical structure morphogenesis	2790	25	1.13E-06
GO:0048856	anatomical structure development	5933	38	2.25E-06
GO:0032502	developmental process	6527	40	2.94E-06
GO:0005583	fibrillar collagen trimer	12	3	7.27E-06
GO:0098643	banded collagen fibril	12	3	7.27E-06
GO:0032501	multicellular organismal process	7674	43	1.01E-05
GO:0005584	collagen type I trimer	2	2	1.06E-05
GO:0005589	collagen type VI trimer	2	2	1.06E-05
GO:0098647	collagen beaded filament	2	2	1.06E-05
GO:0048513	animal organ development	3591	27	1.07E-05
GO:0099512	supramolecular fiber	1029	13	2.54E-05
GO:0099081	supramolecular polymer	1038	13	2.78E-05
GO:0099080	supramolecular complex	1390	15	3.50E-05
GO:0007369	gastrulation	196	6	4.45E-05
GO:0043588	skin development	302	7	5.89E-05
GO:0070062	extracellular exosome	2210	19	6.11E-05
GO:0019838	growth factor binding	132	5	7.34E-05
GO:0001503	ossification	429	8	7.87E-05
GO:0045229	external encapsulating structure organization	321	7	8.64E-05
GO:0030154	cell differentiation	4302	28	1.03E-04
GO:1903561	extracellular vesicle	2307	19	1.09E-04
GO:0043230	extracellular organelle	2308	19	1.10E-04
GO:0065010	extracellular membrane-bounded organelle	2308	19	1.10E-04
GO:0048869	cellular developmental process	4329	28	1.15E-04

GO:0060429	epithelium development	1204	13	1.27E-04
GO:0031982	vesicle	4146	27	1.47E-04
GO:0001649	osteoblast differentiation	246	6	1.56E-04
GO:0110112	regulation of lipid transporter activity	6	2	1.58E-04
GO:0031424	keratinization	84	4	1.68E-04
GO:0008544	epidermis development	362	7	1.82E-04
GO:0002752	cell surface pattern recognition receptor signaling pathv	7	2	2.20E-04
GO:0043394	proteoglycan binding	36	3	2.23E-04
GO:0009898	cytoplasmic side of plasma membrane	173	5	2.61E-04
GO:0030155	regulation of cell adhesion	803	10	2.74E-04
GO:0030246	carbohydrate binding	275	6	2.84E-04
GO:0097435	supramolecular fiber organization	815	10	3.08E-04
GO:0050764	regulation of phagocytosis	103	4	3.67E-04
GO:1903028	positive regulation of opsonization	9	2	3.76E-04
GO:0008091	spectrin	9	2	3.76E-04
GO:0014731	spectrin-associated cytoskeleton	9	2	3.76E-04
GO:0003810	protein-glutamine gamma-glutamyltransferase activity	9	2	3.76E-04
GO:0001501	skeletal system development	541	8	3.82E-04
GO:0008236	serine-type peptidase activity	192	5	4.21E-04
GO:0030863	cortical cytoskeleton	108	4	4.39E-04
GO:0007275	multicellular organism development	4922	29	4.41E-04
GO:0017171	serine hydrolase activity	196	5	4.62E-04
GO:0031235	intrinsic component of the cytoplasmic side of the plasr	10	2	4.69E-04
GO:0030855	epithelial cell differentiation	715	9	5.25E-04
GO:0098562	cytoplasmic side of membrane	204	5	5.54E-04
GO:0035987	endodermal cell differentiation	49	3	5.58E-04
GO:0110011	regulation of basement membrane organization	11	2	5.72E-04
GO:1903027	regulation of opsonization	11	2	5.72E-04
GO:1905370	serine-type endopeptidase complex	11	2	5.72E-04
GO:0009611	response to wounding	577	8	5.85E-04
GO:0042060	wound healing	442	7	6.04E-04
GO:0030198	extracellular matrix organization	318	6	6.12E-04
GO:0043062	extracellular structure organization	319	6	6.23E-04
GO:0048646	anatomical structure formation involved in morphogen	1242	12	6.52E-04
GO:0098636	protein complex involved in cell adhesion	52	3	6.65E-04
GO:0010745	negative regulation of macrophage derived foam cell di	13	2	8.08E-04
GO:0097475	motor neuron migration	13	2	8.08E-04
GO:1905286	serine-type peptidase complex	13	2	8.08E-04
GO:0001704	formation of primary germ layer	127	4	8.08E-04
GO:0032991	protein-containing complex	6153	33	8.11E-04
GO:0050840	extracellular matrix binding	56	3	8.27E-04
GO:0048598	embryonic morphogenesis	614	8	8.76E-04
GO:0001706	endoderm formation	58	3	9.16E-04
GO:0006956	complement activation	132	4	9.34E-04
GO:0098637	protein complex involved in cell-matrix adhesion	14	2	9.41E-04
GO:0001101	response to acid chemical	133	4	9.60E-04
GO:0009790	embryo development	1130	11	1.06E-03

GO:0030199	collagen fibril organization	64	3	1.22E-03
GO:0008228	opsonization	17	2	1.40E-03
GO:0038065	collagen-activated signaling pathway	17	2	1.40E-03
GO:0016755	aminoacyltransferase activity	17	2	1.40E-03
GO:0045111	intermediate filament cytoskeleton	253	5	1.45E-03
GO:0045109	intermediate filament organization	69	3	1.52E-03
GO:0005518	collagen binding	69	3	1.52E-03
GO:0007584	response to nutrient	154	4	1.65E-03
GO:0032101	regulation of response to external stimulus	1014	10	1.65E-03
GO:0031347	regulation of defense response	680	8	1.68E-03
GO:0030506	ankyrin binding	19	2	1.75E-03
GO:0098552	side of membrane	685	8	1.76E-03
GO:0030334	regulation of cell migration	1025	10	1.79E-03
GO:0009605	response to external stimulus	2886	19	1.85E-03
GO:0050766	positive regulation of phagocytosis	74	3	1.85E-03
GO:0005886	plasma membrane	5891	31	1.89E-03
GO:0005783	endoplasmic reticulum	2020	15	1.97E-03
GO:0061448	connective tissue development	274	5	2.06E-03
GO:0008104	protein localization	2469	17	2.12E-03
GO:0071559	response to transforming growth factor beta	277	5	2.16E-03
GO:0030099	myeloid cell differentiation	407	6	2.17E-03
GO:0070727	cellular macromolecule localization	2479	17	2.22E-03
GO:0009887	animal organ morphogenesis	1056	10	2.23E-03
GO:0071230	cellular response to amino acid stimulus	80	3	2.32E-03
GO:0005537	mannose binding	22	2	2.35E-03
GO:0030021	extracellular matrix structural constituent conferring co	22	2	2.35E-03
GO:0033691	sialic acid binding	22	2	2.35E-03
GO:0030216	keratinocyte differentiation	170	4	2.36E-03
GO:0048306	calcium-dependent protein binding	81	3	2.40E-03
GO:0005765	lysosomal membrane	418	6	2.48E-03
GO:0098852	lytic vacuole membrane	418	6	2.48E-03
GO:0007492	endoderm development	83	3	2.57E-03
GO:0061515	myeloid cell development	83	3	2.57E-03
GO:2000145	regulation of cell motility	1086	10	2.73E-03
GO:0005540	hyaluronic acid binding	24	2	2.79E-03
GO:0048731	system development	4454	25	2.82E-03
GO:0000323	lytic vacuole	746	8	2.98E-03
GO:0005764	lysosome	746	8	2.98E-03
GO:0045104	intermediate filament cytoskeleton organization	89	3	3.14E-03
GO:0071229	cellular response to acid chemical	89	3	3.14E-03
GO:0045103	intermediate filament-based process	90	3	3.24E-03
GO:0033343	positive regulation of collagen binding	1	1	3.28E-03
GO:0045852	pH elevation	1	1	3.28E-03
GO:0060739	mesenchymal-epithelial cell signaling involved in prosta	1	1	3.28E-03
GO:0070208	protein heterotrimerization	1	1	3.28E-03
GO:0071306	cellular response to vitamin E	1	1	3.28E-03
GO:0072217	negative regulation of metanephros development	1	1	3.28E-03

GO:1902076	regulation of lateral motor column neuron migration	1	1	3.28E-03
GO:1902078	positive regulation of lateral motor column neuron migr	1	1	3.28E-03
GO:1904538	regulation of glycolytic process through fructose-6-phos	1	1	3.28E-03
GO:1904539	negative regulation of glycolytic process through fructos	1	1	3.28E-03
GO:1904597	negative regulation of connective tissue replacement in	1	1	3.28E-03
GO:1905204	negative regulation of connective tissue replacement	1	1	3.28E-03
GO:1905483	regulation of motor neuron migration	1	1	3.28E-03
GO:1905485	positive regulation of motor neuron migration	1	1	3.28E-03
GO:1905715	regulation of cornification	1	1	3.28E-03
GO:1905716	negative regulation of cornification	1	1	3.28E-03
GO:2000477	regulation of metanephric podocyte development	1	1	3.28E-03
GO:2000478	positive regulation of metanephric podocyte developme	1	1	3.28E-03
GO:2000534	positive regulation of renal albumin absorption	1	1	3.28E-03
GO:2000590	negative regulation of metanephric mesenchymal cell m	1	1	3.28E-03
GO:0005585	collagen type II trimer	1	1	3.28E-03
GO:0005590	collagen type VII trimer	1	1	3.28E-03
GO:0098648	collagen anchoring fibril	1	1	3.28E-03
GO:0098652	collagen type VII anchoring fibril	1	1	3.28E-03
GO:0110157	reelin complex	1	1	3.28E-03
GO:0001792	polymeric immunoglobulin receptor activity	1	1	3.28E-03
GO:0032448	DNA hairpin binding	1	1	3.28E-03
GO:0120153	calcium-dependent carbohydrate binding	1	1	3.28E-03
GO:0031349	positive regulation of defense response	307	5	3.36E-03
GO:0007162	negative regulation of cell adhesion	308	5	3.40E-03
GO:0010875	positive regulation of cholesterol efflux	27	2	3.53E-03
GO:0005938	cell cortex	312	5	3.60E-03
GO:0040012	regulation of locomotion	1130	10	3.63E-03
GO:0006909	phagocytosis	314	5	3.70E-03
GO:0002682	regulation of immune system process	1525	12	3.72E-03
GO:0043236	laminin binding	28	2	3.79E-03
GO:0051393	alpha-actinin binding	28	2	3.79E-03
GO:0005774	vacuolar membrane	461	6	4.01E-03
GO:0001958	endochondral ossification	29	2	4.07E-03
GO:0036075	replacement ossification	29	2	4.07E-03
GO:0048265	response to pain	29	2	4.07E-03
GO:0032103	positive regulation of response to external stimulus	464	6	4.13E-03
GO:0051216	cartilage development	201	4	4.31E-03
GO:0002220	innate immune response activating cell surface recepto	31	2	4.64E-03
GO:0042802	identical protein binding	1997	14	4.80E-03
GO:0007166	cell surface receptor signaling pathway	2907	18	4.91E-03
GO:0002758	innate immune response-activating signal transduction	32	2	4.94E-03
GO:0010743	regulation of macrophage derived foam cell differentiat	32	2	4.94E-03
GO:0006508	proteolysis	1791	13	4.99E-03
GO:0060351	cartilage development involved in endochondral bone r	33	2	5.24E-03
GO:0032963	collagen metabolic process	107	3	5.25E-03
GO:0005200	structural constituent of cytoskeleton	107	3	5.25E-03
GO:0014812	muscle cell migration	108	3	5.39E-03

GO:0005882	intermediate filament	215	4	5.46E-03
GO:0033036	macromolecule localization	2946	18	5.66E-03
GO:0006897	endocytosis	657	7	5.66E-03
GO:0007601	visual perception	220	4	5.92E-03
GO:0005773	vacuole	838	8	5.99E-03
GO:0048584	positive regulation of response to stimulus	2272	15	6.05E-03
GO:0018149	peptide cross-linking	36	2	6.22E-03
GO:0071711	basement membrane organization	36	2	6.22E-03
GO:0042805	actinin binding	36	2	6.22E-03
GO:0002062	chondrocyte differentiation	114	3	6.26E-03
GO:0050953	sensory perception of light stimulus	224	4	6.30E-03
GO:0007596	blood coagulation	225	4	6.40E-03
GO:0001581	detection of chemical stimulus involved in sensory perc	2	1	6.55E-03
GO:0002415	immunoglobulin transcytosis in epithelial cells mediated	2	1	6.55E-03
GO:0003017	lymph circulation	2	1	6.55E-03
GO:0003336	corneocyte desquamation	2	1	6.55E-03
GO:0033341	regulation of collagen binding	2	1	6.55E-03
GO:0044269	glycerol ether catabolic process	2	1	6.55E-03
GO:0060694	regulation of cholesterol transporter activity	2	1	6.55E-03
GO:0062234	platelet activating factor catabolic process	2	1	6.55E-03
GO:0070994	detection of oxidative stress	2	1	6.55E-03
GO:0110113	positive regulation of lipid transporter activity	2	1	6.55E-03
GO:1901502	ether catabolic process	2	1	6.55E-03
GO:1902618	cellular response to fluoride	2	1	6.55E-03
GO:1904596	regulation of connective tissue replacement involved in	2	1	6.55E-03
GO:2000532	regulation of renal albumin absorption	2	1	6.55E-03
GO:2000584	negative regulation of platelet-derived growth factor re	2	1	6.55E-03
GO:0001855	complement component C4b binding	2	1	6.55E-03
GO:0033040	sour taste receptor activity	2	1	6.55E-03
GO:0046326	positive regulation of glucose import	37	2	6.56E-03
GO:0030280	structural constituent of skin epidermis	37	2	6.56E-03
GO:0043200	response to amino acid	118	3	6.89E-03
GO:0050817	coagulation	230	4	6.91E-03
GO:0010742	macrophage derived foam cell differentiation	38	2	6.91E-03
GO:0048821	erythrocyte development	38	2	6.91E-03
GO:0007599	hemostasis	231	4	7.01E-03
GO:0010639	negative regulation of organelle organization	366	5	7.01E-03
GO:0040011	locomotion	1445	11	7.05E-03
GO:0032373	positive regulation of sterol transport	39	2	7.27E-03
GO:0032376	positive regulation of cholesterol transport	39	2	7.27E-03
GO:0090077	foam cell differentiation	39	2	7.27E-03
GO:0009913	epidermal cell differentiation	235	4	7.44E-03
GO:0045923	positive regulation of fatty acid metabolic process	40	2	7.63E-03
GO:0051693	actin filament capping	41	2	8.01E-03
GO:0050912	detection of chemical stimulus involved in sensory perc	43	2	8.78E-03
GO:0050878	regulation of body fluid levels	390	5	9.09E-03
GO:0010828	positive regulation of glucose transmembrane transport	44	2	9.18E-03

GO:1904706	negative regulation of vascular associated smooth musc	44	2	9.18E-03
GO:0030835	negative regulation of actin filament depolymerization	45	2	9.59E-03
GO:0002414	immunoglobulin transcytosis in epithelial cells	3	1	9.81E-03
GO:0003335	corneocyte development	3	1	9.81E-03
GO:0033326	cerebrospinal fluid secretion	3	1	9.81E-03
GO:0034441	plasma lipoprotein particle oxidation	3	1	9.81E-03
GO:0043163	cell envelope organization	3	1	9.81E-03
GO:0060025	regulation of synaptic activity	3	1	9.81E-03
GO:0070165	positive regulation of adiponectin secretion	3	1	9.81E-03
GO:0070543	response to linoleic acid	3	1	9.81E-03
GO:0072244	metanephric glomerular epithelium development	3	1	9.81E-03
GO:0072248	metanephric podocyte differentiation	3	1	9.81E-03
GO:0072249	metanephric podocyte development	3	1	9.81E-03
GO:0072312	metanephric glomerular epithelial cell differentiation	3	1	9.81E-03
GO:0072313	metanephric glomerular epithelial cell development	3	1	9.81E-03
GO:0097477	lateral motor column neuron migration	3	1	9.81E-03
GO:0030934	anchoring collagen complex	3	1	9.81E-03
GO:0034666	integrin alpha2-beta1 complex	3	1	9.81E-03
GO:0090733	tenascin complex	3	1	9.81E-03
GO:0000293	ferric-chelate reductase activity	3	1	9.81E-03
GO:0005006	epidermal growth factor receptor activity	3	1	9.81E-03
GO:0008823	cupric reductase activity	3	1	9.81E-03
GO:0050692	DNA binding domain binding	3	1	9.81E-03
GO:0052851	ferric-chelate reductase (NADPH) activity	3	1	9.81E-03
GO:0010677	negative regulation of cellular carbohydrate metabolic p	46	2	1.00E-02
GO:0085029	extracellular matrix assembly	46	2	1.00E-02
GO:0042383	sarcolemma	136	3	1.01E-02
GO:0005044	scavenger receptor activity	47	2	1.04E-02
GO:0002376	immune system process	2892	17	1.07E-02
GO:0010171	body morphogenesis	48	2	1.09E-02
GO:0050727	regulation of inflammatory response	414	5	1.16E-02
GO:0032964	collagen biosynthetic process	50	2	1.17E-02
GO:0050921	positive regulation of chemotaxis	144	3	1.18E-02
GO:0071560	cellular response to transforming growth factor beta sti	271	4	1.21E-02
GO:0050982	detection of mechanical stimulus	51	2	1.22E-02
GO:0005102	signaling receptor binding	1565	11	1.24E-02
GO:0045912	negative regulation of carbohydrate metabolic process	52	2	1.27E-02
GO:0002687	positive regulation of leukocyte migration	148	3	1.27E-02
GO:0005737	cytoplasm	12069	49	1.28E-02
GO:0035583	sequestering of TGFbeta in extracellular matrix	4	1	1.31E-02
GO:0035789	metanephric mesenchymal cell migration	4	1	1.31E-02
GO:0048014	Tie signaling pathway	4	1	1.31E-02
GO:0071603	endothelial cell-cell adhesion	4	1	1.31E-02
GO:0090258	negative regulation of mitochondrial fission	4	1	1.31E-02
GO:0097018	renal albumin absorption	4	1	1.31E-02
GO:0097114	NMDA glutamate receptor clustering	4	1	1.31E-02
GO:0097476	spinal cord motor neuron migration	4	1	1.31E-02

GO:1902340	negative regulation of chromosome condensation	4	1	1.31E-02
GO:1903690	negative regulation of wound healing, spreading of epid	4	1	1.31E-02
GO:2000589	regulation of metanephric mesenchymal cell migration	4	1	1.31E-02
GO:0050436	microfibril binding	4	1	1.31E-02
GO:0070326	very-low-density lipoprotein particle receptor binding	4	1	1.31E-02
GO:0031226	intrinsic component of plasma membrane	1794	12	1.31E-02
GO:0010874	regulation of cholesterol efflux	53	2	1.31E-02
GO:0030834	regulation of actin filament depolymerization	54	2	1.36E-02
GO:0002684	positive regulation of immune system process	971	8	1.39E-02
GO:0016477	cell migration	1595	11	1.42E-02
GO:0006952	defense response	1820	12	1.45E-02
GO:0005178	integrin binding	156	3	1.47E-02
GO:0043113	receptor clustering	57	2	1.51E-02
GO:0043388	positive regulation of DNA binding	57	2	1.51E-02
GO:1903053	regulation of extracellular matrix organization	57	2	1.51E-02
GO:0060350	endochondral bone morphogenesis	58	2	1.56E-02
GO:0035577	azurophil granule membrane	58	2	1.56E-02
GO:0016020	membrane	9649	41	1.56E-02
GO:0006935	chemotaxis	621	6	1.61E-02
GO:0030042	actin filament depolymerization	59	2	1.61E-02
GO:0046324	regulation of glucose import	59	2	1.61E-02
GO:0021999	neural plate anterior/posterior regionalization	5	1	1.63E-02
GO:0031508	pericentric heterochromatin formation	5	1	1.63E-02
GO:0032224	positive regulation of synaptic transmission, cholinergic	5	1	1.63E-02
GO:0035788	cell migration involved in metanephros development	5	1	1.63E-02
GO:0035811	negative regulation of urine volume	5	1	1.63E-02
GO:0042494	detection of bacterial lipoprotein	5	1	1.63E-02
GO:0090131	mesenchyme migration	5	1	1.63E-02
GO:0090185	negative regulation of kidney development	5	1	1.63E-02
GO:1902617	response to fluoride	5	1	1.63E-02
GO:1903845	negative regulation of cellular response to transforming	5	1	1.63E-02
GO:1904259	regulation of basement membrane assembly involved ir	5	1	1.63E-02
GO:1904261	positive regulation of basement membrane assembly in	5	1	1.63E-02
GO:1905599	positive regulation of low-density lipoprotein receptor a	5	1	1.63E-02
GO:2000467	positive regulation of glycogen (starch) synthase activity	5	1	1.63E-02
GO:2000583	regulation of platelet-derived growth factor receptor-al	5	1	1.63E-02
GO:2001197	basement membrane assembly involved in embryonic b	5	1	1.63E-02
GO:0005592	collagen type XI trimer	5	1	1.63E-02
GO:0045180	basal cortex	5	1	1.63E-02
GO:0071746	IgA immunoglobulin complex, circulating	5	1	1.63E-02
GO:0071749	polymeric IgA immunoglobulin complex	5	1	1.63E-02
GO:0071751	secretory IgA immunoglobulin complex	5	1	1.63E-02
GO:0098966	perisynaptic extracellular matrix	5	1	1.63E-02
GO:0099535	synapse-associated extracellular matrix	5	1	1.63E-02
GO:1990779	glycoprotein Ib-IX-V complex	5	1	1.63E-02
GO:0004758	serine C-palmitoyltransferase activity	5	1	1.63E-02
GO:0016454	C-palmitoyltransferase activity	5	1	1.63E-02

GO:0016723	oxidoreductase activity, acting on metal ions, NAD or N/	5	1	1.63E-02
GO:0042806	fucose binding	5	1	1.63E-02
GO:0045545	syndecan binding	5	1	1.63E-02
GO:0098639	collagen binding involved in cell-matrix adhesion	5	1	1.63E-02
GO:0042330	taxis	623	6	1.63E-02
GO:0043407	negative regulation of MAP kinase activity	60	2	1.66E-02
GO:0051494	negative regulation of cytoskeleton organization	165	3	1.70E-02
GO:0007167	enzyme-linked receptor protein signaling pathway	1010	8	1.72E-02
GO:0048729	tissue morphogenesis	633	6	1.75E-02
GO:0021700	developmental maturation	304	4	1.77E-02
GO:1902904	negative regulation of supramolecular fiber organizatio	169	3	1.81E-02
GO:0031406	carboxylic acid binding	171	3	1.87E-02
GO:0002218	activation of innate immune response	64	2	1.88E-02
GO:0035306	positive regulation of dephosphorylation	64	2	1.88E-02
GO:0010694	positive regulation of alkaline phosphatase activity	6	1	1.95E-02
GO:0010891	negative regulation of sequestering of triglyceride	6	1	1.95E-02
GO:0015677	copper ion import	6	1	1.95E-02
GO:0021800	cerebral cortex tangential migration	6	1	1.95E-02
GO:0033591	response to L-ascorbic acid	6	1	1.95E-02
GO:0035787	cell migration involved in kidney development	6	1	1.95E-02
GO:0060897	neural plate regionalization	6	1	1.95E-02
GO:0072215	regulation of metanephros development	6	1	1.95E-02
GO:0090230	regulation of centromere complex assembly	6	1	1.95E-02
GO:2000481	positive regulation of cAMP-dependent protein kinase a	6	1	1.95E-02
GO:0000939	inner kinetochore	6	1	1.95E-02
GO:0001740	Barr body	6	1	1.95E-02
GO:0033165	interphotoreceptor matrix	6	1	1.95E-02
GO:0071439	clathrin complex	6	1	1.95E-02
GO:0097443	sorting endosome	6	1	1.95E-02
GO:0038064	collagen receptor activity	6	1	1.95E-02
GO:0042289	MHC class II protein binding	6	1	1.95E-02
GO:0050543	icosatetraenoic acid binding	6	1	1.95E-02
GO:0050544	arachidonic acid binding	6	1	1.95E-02
GO:0004252	serine-type endopeptidase activity	174	3	1.96E-02
GO:0048660	regulation of smooth muscle cell proliferation	175	3	1.99E-02
GO:0010812	negative regulation of cell-substrate adhesion	66	2	1.99E-02
GO:0030219	megakaryocyte differentiation	66	2	1.99E-02
GO:0030837	negative regulation of actin filament polymerization	66	2	1.99E-02
GO:0051099	positive regulation of binding	177	3	2.05E-02
GO:0051641	cellular localization	3351	18	2.05E-02
GO:0005856	cytoskeleton	2374	14	2.05E-02
GO:0006959	humoral immune response	320	4	2.10E-02
GO:0050909	sensory perception of taste	68	2	2.10E-02
GO:0048659	smooth muscle cell proliferation	179	3	2.11E-02
GO:0048583	regulation of response to stimulus	4131	21	2.12E-02
GO:0050954	sensory perception of mechanical stimulus	180	3	2.14E-02
GO:0014823	response to activity	70	2	2.22E-02

GO:0001957	intramembranous ossification	7	1	2.27E-02
GO:0002248	connective tissue replacement involved in inflammatory	7	1	2.27E-02
GO:0009744	response to sucrose	7	1	2.27E-02
GO:0032222	regulation of synaptic transmission, cholinergic	7	1	2.27E-02
GO:0034285	response to disaccharide	7	1	2.27E-02
GO:0035790	platelet-derived growth factor receptor-alpha signaling	7	1	2.27E-02
GO:0036072	direct ossification	7	1	2.27E-02
GO:0044691	tooth eruption	7	1	2.27E-02
GO:0048050	post-embryonic eye morphogenesis	7	1	2.27E-02
GO:0051971	positive regulation of transmission of nerve impulse	7	1	2.27E-02
GO:0060232	delamination	7	1	2.27E-02
GO:0060331	negative regulation of response to interferon-gamma	7	1	2.27E-02
GO:0060336	negative regulation of interferon-gamma-mediated sign	7	1	2.27E-02
GO:0060638	mesenchymal-epithelial cell signaling	7	1	2.27E-02
GO:0070162	adiponectin secretion	7	1	2.27E-02
GO:0070163	regulation of adiponectin secretion	7	1	2.27E-02
GO:0097017	renal protein absorption	7	1	2.27E-02
GO:0097065	anterior head development	7	1	2.27E-02
GO:1905598	negative regulation of low-density lipoprotein receptor	7	1	2.27E-02
GO:0019763	immunoglobulin receptor activity	7	1	2.27E-02
GO:0032051	clathrin light chain binding	7	1	2.27E-02
GO:0050542	icosanoid binding	7	1	2.27E-02
GO:0050693	LBD domain binding	7	1	2.27E-02
GO:0098634	cell-matrix adhesion mediator activity	7	1	2.27E-02
GO:0033344	cholesterol efflux	71	2	2.28E-02
GO:1901880	negative regulation of protein depolymerization	72	2	2.34E-02
GO:0048029	monosaccharide binding	72	2	2.34E-02
GO:0048469	cell maturation	187	3	2.36E-02
GO:0051668	localization within membrane	679	6	2.38E-02
GO:0002833	positive regulation of response to biotic stimulus	188	3	2.39E-02
GO:0071260	cellular response to mechanical stimulus	73	2	2.40E-02
GO:0044419	biological process involved in interspecies interaction b	1726	11	2.41E-02
GO:0048662	negative regulation of smooth muscle cell proliferation	74	2	2.46E-02
GO:0070482	response to oxygen levels	337	4	2.48E-02
GO:0008217	regulation of blood pressure	191	3	2.49E-02
GO:0046323	glucose import	75	2	2.52E-02
GO:0050731	positive regulation of peptidyl-tyrosine phosphorylation	193	3	2.56E-02
GO:0030853	negative regulation of granulocyte differentiation	8	1	2.59E-02
GO:0031509	subtelomeric heterochromatin formation	8	1	2.59E-02
GO:0038026	reelin-mediated signaling pathway	8	1	2.59E-02
GO:0045650	negative regulation of macrophage differentiation	8	1	2.59E-02
GO:0046469	platelet activating factor metabolic process	8	1	2.59E-02
GO:0060896	neural plate pattern specification	8	1	2.59E-02
GO:0071694	maintenance of protein location in extracellular region	8	1	2.59E-02
GO:0097119	postsynaptic density protein 95 clustering	8	1	2.59E-02
GO:1901388	regulation of transforming growth factor beta activation	8	1	2.59E-02
GO:1904179	positive regulation of adipose tissue development	8	1	2.59E-02

GO:2000465	regulation of glycogen (starch) synthase activity	8	1	2.59E-02
GO:2000969	positive regulation of AMPA receptor activity	8	1	2.59E-02
GO:0017059	serine C-palmitoyltransferase complex	8	1	2.59E-02
GO:0031211	endoplasmic reticulum palmitoyltransferase complex	8	1	2.59E-02
GO:0003847	1-alkyl-2-acetylglycerophosphocholine esterase activity	8	1	2.59E-02
GO:0016043	cellular component organization	6395	29	2.62E-02
GO:0010827	regulation of glucose transmembrane transport	77	2	2.65E-02
GO:0001618	virus receptor activity	77	2	2.65E-02
GO:0046332	SMAD binding	78	2	2.71E-02
GO:0140272	exogenous protein binding	78	2	2.71E-02
GO:0019199	transmembrane receptor protein kinase activity	79	2	2.78E-02
GO:0046872	metal ion binding	4246	21	2.81E-02
GO:0051050	positive regulation of transport	904	7	2.83E-02
GO:0030864	cortical actin cytoskeleton	80	2	2.84E-02
GO:0032272	negative regulation of protein polymerization	81	2	2.91E-02
GO:0032371	regulation of sterol transport	81	2	2.91E-02
GO:0032374	regulation of cholesterol transport	81	2	2.91E-02
GO:0033555	multicellular organismal response to stress	81	2	2.91E-02
GO:0002246	wound healing involved in inflammatory response	9	1	2.91E-02
GO:0007597	blood coagulation, intrinsic pathway	9	1	2.91E-02
GO:0010616	negative regulation of cardiac muscle adaptation	9	1	2.91E-02
GO:0010692	regulation of alkaline phosphatase activity	9	1	2.91E-02
GO:0033197	response to vitamin E	9	1	2.91E-02
GO:0050915	sensory perception of sour taste	9	1	2.91E-02
GO:0060392	negative regulation of SMAD protein signal transductior	9	1	2.91E-02
GO:0070268	cornification	9	1	2.91E-02
GO:0090129	positive regulation of synapse maturation	9	1	2.91E-02
GO:1903243	negative regulation of cardiac muscle hypertrophy in re	9	1	2.91E-02
GO:1903689	regulation of wound healing, spreading of epidermal ce	9	1	2.91E-02
GO:0032437	cuticular plate	9	1	2.91E-02
GO:0030492	hemoglobin binding	9	1	2.91E-02
GO:0047499	calcium-independent phospholipase A2 activity	9	1	2.91E-02
GO:0043242	negative regulation of protein-containing complex disas	82	2	2.98E-02
GO:1905369	endopeptidase complex	82	2	2.98E-02
GO:0038024	cargo receptor activity	82	2	2.98E-02
GO:0010562	positive regulation of phosphorus metabolic process	914	7	2.98E-02
GO:0045937	positive regulation of phosphate metabolic process	914	7	2.98E-02
GO:0051129	negative regulation of cellular component organization	717	6	3.00E-02
GO:0048870	cell motility	1791	11	3.06E-02
GO:0030097	hemopoiesis	920	7	3.08E-02
GO:0002831	regulation of response to biotic stimulus	361	4	3.08E-02
GO:0097006	regulation of plasma lipoprotein particle levels	84	2	3.11E-02
GO:0051179	localization	5370	25	3.13E-02
GO:0034103	regulation of tissue remodeling	85	2	3.18E-02
GO:0048041	focal adhesion assembly	85	2	3.18E-02
GO:0001540	amyloid-beta binding	85	2	3.18E-02
GO:0045087	innate immune response	927	7	3.19E-02

GO:0009612	response to mechanical stimulus	211	3	3.21E-02
GO:0010572	positive regulation of platelet activation	10	1	3.23E-02
GO:0014012	peripheral nervous system axon regeneration	10	1	3.23E-02
GO:0033034	positive regulation of myeloid cell apoptotic process	10	1	3.23E-02
GO:0033572	transferrin transport	10	1	3.23E-02
GO:0060346	bone trabecula formation	10	1	3.23E-02
GO:1901203	positive regulation of extracellular matrix assembly	10	1	3.23E-02
GO:1902412	regulation of mitotic cytokinesis	10	1	3.23E-02
GO:1905461	positive regulation of vascular associated smooth muscl	10	1	3.23E-02
GO:0000805	X chromosome	10	1	3.23E-02
GO:0071745	IgA immunoglobulin complex	10	1	3.23E-02
GO:0004955	prostaglandin receptor activity	10	1	3.23E-02
GO:0033273	response to vitamin	86	2	3.25E-02
GO:0006950	response to stress	4044	20	3.25E-02
GO:0043235	receptor complex	544	5	3.30E-02
GO:0014910	regulation of smooth muscle cell migration	87	2	3.32E-02
GO:0051098	regulation of binding	372	4	3.39E-02
GO:0032370	positive regulation of lipid transport	88	2	3.39E-02
GO:0055072	iron ion homeostasis	88	2	3.39E-02
GO:1901879	regulation of protein depolymerization	88	2	3.39E-02
GO:0007179	transforming growth factor beta receptor signaling path	216	3	3.41E-02
GO:0001960	negative regulation of cytokine-mediated signaling path	89	2	3.46E-02
GO:0070848	response to growth factor	742	6	3.46E-02
GO:0043169	cation binding	4337	21	3.47E-02
GO:0045995	regulation of embryonic development	90	2	3.53E-02
GO:0051591	response to cAMP	90	2	3.53E-02
GO:0010172	embryonic body morphogenesis	11	1	3.55E-02
GO:0010739	positive regulation of protein kinase A signaling	11	1	3.55E-02
GO:0010887	negative regulation of cholesterol storage	11	1	3.55E-02
GO:0032493	response to bacterial lipoprotein	11	1	3.55E-02
GO:0034115	negative regulation of heterotypic cell-cell adhesion	11	1	3.55E-02
GO:0035581	sequestering of extracellular ligand from receptor	11	1	3.55E-02
GO:0036363	transforming growth factor beta activation	11	1	3.55E-02
GO:0048563	post-embryonic animal organ morphogenesis	11	1	3.55E-02
GO:0060174	limb bud formation	11	1	3.55E-02
GO:0060272	embryonic skeletal joint morphogenesis	11	1	3.55E-02
GO:0060623	regulation of chromosome condensation	11	1	3.55E-02
GO:0070587	regulation of cell-cell adhesion involved in gastrulation	11	1	3.55E-02
GO:0071872	cellular response to epinephrine stimulus	11	1	3.55E-02
GO:0002178	palmitoyltransferase complex	11	1	3.55E-02
GO:0005614	interstitial matrix	11	1	3.55E-02
GO:0004954	prostanoid receptor activity	11	1	3.55E-02
GO:0018108	peptidyl-tyrosine phosphorylation	380	4	3.62E-02
GO:0018212	peptidyl-tyrosine modification	382	4	3.68E-02
GO:0098609	cell-cell adhesion	957	7	3.69E-02
GO:1904705	regulation of vascular associated smooth muscle cell pr	93	2	3.75E-02
GO:0048534	hematopoietic or lymphoid organ development	962	7	3.78E-02

GO:0030335	positive regulation of cell migration	566	5	3.81E-02
GO:0060761	negative regulation of response to cytokine stimulus	94	2	3.82E-02
GO:0043167	ion binding	6021	27	3.82E-02
GO:0003008	system process	2333	13	3.83E-02
GO:0006971	hypotonic response	12	1	3.87E-02
GO:0014745	negative regulation of muscle adaptation	12	1	3.87E-02
GO:0031953	negative regulation of protein autophosphorylation	12	1	3.87E-02
GO:0032490	detection of molecule of bacterial origin	12	1	3.87E-02
GO:0033327	Leydig cell differentiation	12	1	3.87E-02
GO:0038063	collagen-activated tyrosine kinase receptor signaling pa	12	1	3.87E-02
GO:0060394	negative regulation of pathway-restricted SMAD protei	12	1	3.87E-02
GO:0070586	cell-cell adhesion involved in gastrulation	12	1	3.87E-02
GO:0071107	response to parathyroid hormone	12	1	3.87E-02
GO:0072015	podocyte development	12	1	3.87E-02
GO:0140719	constitutive heterochromatin formation	12	1	3.87E-02
GO:1904177	regulation of adipose tissue development	12	1	3.87E-02
GO:0034362	low-density lipoprotein particle	12	1	3.87E-02
GO:0015269	calcium-activated potassium channel activity	12	1	3.87E-02
GO:0097677	STAT family protein binding	12	1	3.87E-02
GO:0007044	cell-substrate junction assembly	95	2	3.89E-02
GO:0014909	smooth muscle cell migration	95	2	3.89E-02
GO:0045638	negative regulation of myeloid cell differentiation	95	2	3.89E-02
GO:1990874	vascular associated smooth muscle cell proliferation	95	2	3.89E-02
GO:0098542	defense response to other organism	1183	8	3.92E-02
GO:0071840	cellular component organization or biogenesis	6601	29	3.94E-02
GO:0019217	regulation of fatty acid metabolic process	96	2	3.97E-02
GO:0008037	cell recognition	230	3	3.99E-02
GO:0050920	regulation of chemotaxis	230	3	3.99E-02
GO:0009719	response to endogenous stimulus	1637	10	4.01E-02
GO:0002685	regulation of leukocyte migration	231	3	4.03E-02
GO:0002690	positive regulation of leukocyte chemotaxis	97	2	4.04E-02
GO:0060349	bone morphogenesis	98	2	4.12E-02
GO:0043202	lysosomal lumen	98	2	4.12E-02
GO:0004867	serine-type endopeptidase inhibitor activity	98	2	4.12E-02
GO:0060348	bone development	233	3	4.12E-02
GO:0002253	activation of immune response	397	4	4.14E-02
GO:0003334	keratinocyte development	13	1	4.18E-02
GO:0009415	response to water	13	1	4.18E-02
GO:0010642	negative regulation of platelet-derived growth factor re	13	1	4.18E-02
GO:0051451	myoblast migration	13	1	4.18E-02
GO:0060100	positive regulation of phagocytosis, engulfment	13	1	4.18E-02
GO:0061430	bone trabecula morphogenesis	13	1	4.18E-02
GO:0072310	glomerular epithelial cell development	13	1	4.18E-02
GO:1900115	extracellular regulation of signal transduction	13	1	4.18E-02
GO:1900116	extracellular negative regulation of signal transduction	13	1	4.18E-02
GO:1900121	negative regulation of receptor binding	13	1	4.18E-02
GO:1905155	positive regulation of membrane invagination	13	1	4.18E-02

GO:2001046	positive regulation of integrin-mediated signaling pathw	13	1	4.18E-02
GO:0001527	microfibril	13	1	4.18E-02
GO:0005024	transforming growth factor beta receptor activity	13	1	4.18E-02
GO:0031386	protein tag	13	1	4.18E-02
GO:0030512	negative regulation of transforming growth factor beta	99	2	4.20E-02
GO:0031234	extrinsic component of cytoplasmic side of plasma mem	99	2	4.20E-02
GO:0030527	structural constituent of chromatin	99	2	4.20E-02
GO:0022603	regulation of anatomical structure morphogenesis	987	7	4.25E-02
GO:0034764	positive regulation of transmembrane transport	237	3	4.30E-02
GO:0005539	glycosaminoglycan binding	237	3	4.30E-02
GO:0072657	protein localization to membrane	586	5	4.32E-02
GO:0080134	regulation of response to stress	1431	9	4.35E-02
GO:0150115	cell-substrate junction organization	101	2	4.35E-02
GO:0045095	keratin filament	101	2	4.35E-02
GO:0012505	endomembrane system	4715	22	4.42E-02
GO:0045088	regulation of innate immune response	240	3	4.43E-02
GO:2000147	positive regulation of cell motility	592	5	4.48E-02
GO:0021819	layer formation in cerebral cortex	14	1	4.50E-02
GO:0030238	male sex determination	14	1	4.50E-02
GO:0045820	negative regulation of glycolytic process	14	1	4.50E-02
GO:0050966	detection of mechanical stimulus involved in sensory pe	14	1	4.50E-02
GO:0071871	response to epinephrine	14	1	4.50E-02
GO:0090128	regulation of synapse maturation	14	1	4.50E-02
GO:1905203	regulation of connective tissue replacement	14	1	4.50E-02
GO:0006910	phagocytosis, recognition	103	2	4.51E-02
GO:0032677	regulation of interleukin-8 production	103	2	4.51E-02
GO:0032984	protein-containing complex disassembly	242	3	4.53E-02
GO:0032637	interleukin-8 production	104	2	4.58E-02
GO:0007600	sensory perception	1006	7	4.63E-02
GO:0008233	peptidase activity	601	5	4.72E-02
GO:0006958	complement activation, classical pathway	106	2	4.74E-02
GO:0005796	Golgi lumen	106	2	4.74E-02
GO:0033002	muscle cell proliferation	247	3	4.76E-02
GO:0016746	acyltransferase activity	248	3	4.81E-02
GO:0003674	molecular_function	18432	65	4.81E-02
GO:0009886	post-embryonic animal morphogenesis	15	1	4.81E-02
GO:0010889	regulation of sequestering of triglyceride	15	1	4.81E-02
GO:0046512	sphingosine biosynthetic process	15	1	4.81E-02
GO:0046520	sphingoid biosynthetic process	15	1	4.81E-02
GO:0060099	regulation of phagocytosis, engulfment	15	1	4.81E-02
GO:0060330	regulation of response to interferon-gamma	15	1	4.81E-02
GO:0060334	regulation of interferon-gamma-mediated signaling pat	15	1	4.81E-02
GO:0070831	basement membrane assembly	15	1	4.81E-02
GO:0071599	otic vesicle development	15	1	4.81E-02
GO:0071639	positive regulation of monocyte chemotactic protein-1	15	1	4.81E-02
GO:0072498	embryonic skeletal joint development	15	1	4.81E-02
GO:1900426	positive regulation of defense response to bacterium	15	1	4.81E-02

GO:1904936	interneuron migration	15	1	4.81E-02
GO:1905153	regulation of membrane invagination	15	1	4.81E-02
GO:0004953	icosanoid receptor activity	15	1	4.81E-02
GO:0036041	long-chain fatty acid binding	15	1	4.81E-02
GO:0046965	nuclear retinoid X receptor binding	15	1	4.81E-02
GO:0051371	muscle alpha-actinin binding	15	1	4.81E-02
GO:0070492	oligosaccharide binding	15	1	4.81E-02
GO:0010717	regulation of epithelial to mesenchymal transition	107	2	4.82E-02
GO:0040017	positive regulation of locomotion	607	5	4.89E-02
GO:0071695	anatomical structure maturation	250	3	4.90E-02
GO:0062014	negative regulation of small molecule metabolic proces	108	2	4.91E-02
GO:0090263	positive regulation of canonical Wnt signaling pathway	108	2	4.91E-02
GO:0002520	immune system development	1024	7	5.01E-02
GO:0042592	homeostatic process	1705	10	5.05E-02
GO:0007173	epidermal growth factor receptor signaling pathway	110	2	5.07E-02
GO:0060322	head development	815	6	5.07E-02
GO:0006825	copper ion transport	16	1	5.12E-02
GO:0030852	regulation of granulocyte differentiation	16	1	5.12E-02
GO:0034374	low-density lipoprotein particle remodeling	16	1	5.12E-02
GO:0034393	positive regulation of smooth muscle cell apoptotic pro	16	1	5.12E-02
GO:0034638	phosphatidylcholine catabolic process	16	1	5.12E-02
GO:0043249	erythrocyte maturation	16	1	5.12E-02
GO:0048266	behavioral response to pain	16	1	5.12E-02
GO:0050872	white fat cell differentiation	16	1	5.12E-02
GO:0070365	hepatocyte differentiation	16	1	5.12E-02
GO:0072578	neurotransmitter-gated ion channel clustering	16	1	5.12E-02
GO:1901201	regulation of extracellular matrix assembly	16	1	5.12E-02
GO:0016722	oxidoreductase activity, acting on metal ions	16	1	5.12E-02
GO:0001944	vasculature development	818	6	5.14E-02
GO:0021761	limbic system development	111	2	5.15E-02
GO:0005887	integral component of plasma membrane	1713	10	5.19E-02
GO:0006898	receptor-mediated endocytosis	257	3	5.24E-02
GO:0007229	integrin-mediated signaling pathway	113	2	5.32E-02
GO:1904659	glucose transmembrane transport	113	2	5.32E-02
GO:1905954	positive regulation of lipid localization	113	2	5.32E-02
GO:0010470	regulation of gastrulation	17	1	5.44E-02
GO:0016045	detection of bacterium	17	1	5.44E-02
GO:0020027	hemoglobin metabolic process	17	1	5.44E-02
GO:0051895	negative regulation of focal adhesion assembly	17	1	5.44E-02
GO:0051969	regulation of transmission of nerve impulse	17	1	5.44E-02
GO:0070206	protein trimerization	17	1	5.44E-02
GO:0071468	cellular response to acidic pH	17	1	5.44E-02
GO:0072224	metanephric glomerulus development	17	1	5.44E-02
GO:0072378	blood coagulation, fibrin clot formation	17	1	5.44E-02
GO:0090136	epithelial cell-cell adhesion	17	1	5.44E-02
GO:0090594	inflammatory response to wounding	17	1	5.44E-02
GO:0150118	negative regulation of cell-substrate junction organizati	17	1	5.44E-02

GO:1905288	vascular associated smooth muscle cell apoptotic proce	17	1	5.44E-02
GO:1905459	regulation of vascular associated smooth muscle cell ap	17	1	5.44E-02
GO:0005869	dynactin complex	17	1	5.44E-02
GO:0008150	biological_process	18903	66	5.44E-02
GO:0008645	hexose transmembrane transport	116	2	5.57E-02
GO:0034440	lipid oxidation	116	2	5.57E-02
GO:0050730	regulation of peptidyl-tyrosine phosphorylation	266	3	5.70E-02
GO:0050830	defense response to Gram-positive bacterium	118	2	5.74E-02
GO:0051261	protein depolymerization	118	2	5.74E-02
GO:1905368	peptidase complex	118	2	5.74E-02
GO:0010612	regulation of cardiac muscle adaptation	18	1	5.75E-02
GO:0030730	sequestering of triglyceride	18	1	5.75E-02
GO:0030903	notochord development	18	1	5.75E-02
GO:0045721	negative regulation of gluconeogenesis	18	1	5.75E-02
GO:0048569	post-embryonic animal organ development	18	1	5.75E-02
GO:0060391	positive regulation of SMAD protein signal transduction	18	1	5.75E-02
GO:0061644	protein localization to CENP-A containing chromatin	18	1	5.75E-02
GO:1903242	regulation of cardiac muscle hypertrophy in response to	18	1	5.75E-02
GO:2001224	positive regulation of neuron migration	18	1	5.75E-02
GO:0030130	clathrin coat of trans-Golgi network vesicle	18	1	5.75E-02
GO:0043083	synaptic cleft	18	1	5.75E-02
GO:0043505	CENP-A containing nucleosome	18	1	5.75E-02
GO:0061638	CENP-A containing chromatin	18	1	5.75E-02
GO:0043395	heparan sulfate proteoglycan binding	18	1	5.75E-02
GO:0065008	regulation of biological quality	3773	18	5.82E-02
GO:0007417	central nervous system development	1060	7	5.82E-02
GO:0002455	humoral immune response mediated by circulating imm	119	2	5.83E-02
GO:0015749	monosaccharide transmembrane transport	119	2	5.83E-02
GO:0032868	response to insulin	269	3	5.85E-02
GO:0006954	inflammatory response	848	6	5.93E-02
GO:0007169	transmembrane receptor protein tyrosine kinase signali	642	5	5.95E-02
GO:0006334	nucleosome assembly	121	2	6.00E-02
GO:0030301	cholesterol transport	121	2	6.00E-02
GO:0032355	response to estradiol	121	2	6.00E-02
GO:0051101	regulation of DNA binding	121	2	6.00E-02
GO:0045178	basal part of cell	272	3	6.01E-02
GO:0001502	cartilage condensation	19	1	6.06E-02
GO:0010738	regulation of protein kinase A signaling	19	1	6.06E-02
GO:0010885	regulation of cholesterol storage	19	1	6.06E-02
GO:0021511	spinal cord patterning	19	1	6.06E-02
GO:0061003	positive regulation of dendritic spine morphogenesis	19	1	6.06E-02
GO:0061615	glycolytic process through fructose-6-phosphate	19	1	6.06E-02
GO:1902894	negative regulation of miRNA transcription	19	1	6.06E-02
GO:0034506	chromosome, centromeric core domain	19	1	6.06E-02
GO:0004675	transmembrane receptor protein serine/threonine kina	19	1	6.06E-02
GO:0005452	solute:inorganic anion antiporter activity	19	1	6.06E-02
GO:0046983	protein dimerization activity	1070	7	6.07E-02

GO:0071901	negative regulation of protein serine/threonine kinase a	122	2	6.09E-02
GO:0044877	protein-containing complex binding	1299	8	6.16E-02
GO:0001894	tissue homeostasis	275	3	6.17E-02
GO:0110053	regulation of actin filament organization	278	3	6.33E-02
GO:0003014	renal system process	125	2	6.35E-02
GO:0006662	glycerol ether metabolic process	20	1	6.36E-02
GO:0032793	positive regulation of CREB transcription factor activity	20	1	6.36E-02
GO:0042953	lipoprotein transport	20	1	6.36E-02
GO:0045653	negative regulation of megakaryocyte differentiation	20	1	6.36E-02
GO:0046485	ether lipid metabolic process	20	1	6.36E-02
GO:0055093	response to hyperoxia	20	1	6.36E-02
GO:0072376	protein activation cascade	20	1	6.36E-02
GO:0097709	connective tissue replacement	20	1	6.36E-02
GO:0098543	detection of other organism	20	1	6.36E-02
GO:1903579	negative regulation of ATP metabolic process	20	1	6.36E-02
GO:2000479	regulation of cAMP-dependent protein kinase activity	20	1	6.36E-02
GO:2000629	negative regulation of miRNA metabolic process	20	1	6.36E-02
GO:0012510	trans-Golgi network transport vesicle membrane	20	1	6.36E-02
GO:0000979	RNA polymerase II core promoter sequence-specific DN	20	1	6.36E-02
GO:0016408	C-acyltransferase activity	20	1	6.36E-02
GO:0004888	transmembrane signaling receptor activity	1309	8	6.39E-02
GO:0071417	cellular response to organonitrogen compound	656	5	6.40E-02
GO:0032411	positive regulation of transporter activity	126	2	6.44E-02
GO:0038127	ERBB signaling pathway	126	2	6.44E-02
GO:0043244	regulation of protein-containing complex disassembly	126	2	6.44E-02
GO:0051707	response to other organism	1548	9	6.53E-02
GO:0002688	regulation of leukocyte chemotaxis	127	2	6.53E-02
GO:0006911	phagocytosis, engulfment	127	2	6.53E-02
GO:0098742	cell-cell adhesion via plasma-membrane adhesion mole	282	3	6.55E-02
GO:0043207	response to external biotic stimulus	1551	9	6.59E-02
GO:0030218	erythrocyte differentiation	128	2	6.62E-02
GO:0046683	response to organophosphorus	128	2	6.62E-02
GO:0048706	embryonic skeletal system development	128	2	6.62E-02
GO:0006670	sphingosine metabolic process	21	1	6.67E-02
GO:0010878	cholesterol storage	21	1	6.67E-02
GO:0035902	response to immobilization stress	21	1	6.67E-02
GO:0044872	lipoprotein localization	21	1	6.67E-02
GO:0045056	transcytosis	21	1	6.67E-02
GO:0060008	Sertoli cell differentiation	21	1	6.67E-02
GO:0071605	monocyte chemotactic protein-1 production	21	1	6.67E-02
GO:0071637	regulation of monocyte chemotactic protein-1 producti	21	1	6.67E-02
GO:0090026	positive regulation of monocyte chemotaxis	21	1	6.67E-02
GO:0001846	opsonin binding	21	1	6.67E-02
GO:0072659	protein localization to plasma membrane	285	3	6.72E-02
GO:0019538	protein metabolic process	5494	24	6.88E-02
GO:0005770	late endosome	288	3	6.88E-02
GO:0007530	sex determination	22	1	6.98E-02

GO:0010888	negative regulation of lipid storage	22	1	6.98E-02
GO:0034104	negative regulation of tissue remodeling	22	1	6.98E-02
GO:0035313	wound healing, spreading of epidermal cells	22	1	6.98E-02
GO:0035809	regulation of urine volume	22	1	6.98E-02
GO:0035855	megakaryocyte development	22	1	6.98E-02
GO:0050765	negative regulation of phagocytosis	22	1	6.98E-02
GO:0061318	renal filtration cell differentiation	22	1	6.98E-02
GO:0071404	cellular response to low-density lipoprotein particle stin	22	1	6.98E-02
GO:0072112	podocyte differentiation	22	1	6.98E-02
GO:0098743	cell aggregation	22	1	6.98E-02
GO:0140374	antiviral innate immune response	22	1	6.98E-02
GO:1900424	regulation of defense response to bacterium	22	1	6.98E-02
GO:2000310	regulation of NMDA receptor activity	22	1	6.98E-02
GO:2001044	regulation of integrin-mediated signaling pathway	22	1	6.98E-02
GO:0090665	glycoprotein complex	22	1	6.98E-02
GO:0015106	bicarbonate transmembrane transporter activity	22	1	6.98E-02
GO:0034219	carbohydrate transmembrane transport	132	2	6.98E-02
GO:0031667	response to nutrient levels	474	4	7.02E-02
GO:0035303	regulation of dephosphorylation	133	2	7.08E-02
GO:0000786	nucleosome	133	2	7.08E-02
GO:0015918	sterol transport	135	2	7.26E-02
GO:0042476	odontogenesis	135	2	7.26E-02
GO:0034312	diol biosynthetic process	23	1	7.28E-02
GO:0046519	sphingoid metabolic process	23	1	7.28E-02
GO:0060965	negative regulation of miRNA-mediated gene silencing	23	1	7.28E-02
GO:0072243	metanephric nephron epithelium development	23	1	7.28E-02
GO:0072311	glomerular epithelial cell differentiation	23	1	7.28E-02
GO:1900120	regulation of receptor binding	23	1	7.28E-02
GO:1900273	positive regulation of long-term synaptic potentiation	23	1	7.28E-02
GO:1900543	negative regulation of purine nucleotide metabolic proc	23	1	7.28E-02
GO:0001666	response to hypoxia	296	3	7.34E-02
GO:0048872	homeostasis of number of cells	296	3	7.34E-02
GO:0010565	regulation of cellular ketone metabolic process	136	2	7.36E-02
GO:0099024	plasma membrane invagination	136	2	7.36E-02
GO:0009581	detection of external stimulus	137	2	7.45E-02
GO:0034101	erythrocyte homeostasis	137	2	7.45E-02
GO:0050793	regulation of developmental process	2584	13	7.52E-02
GO:0009607	response to biotic stimulus	1593	9	7.53E-02
GO:0010640	regulation of platelet-derived growth factor receptor sig	24	1	7.59E-02
GO:0014850	response to muscle activity	24	1	7.59E-02
GO:0035357	peroxisome proliferator activated receptor signaling pat	24	1	7.59E-02
GO:0036303	lymph vessel morphogenesis	24	1	7.59E-02
GO:0060149	negative regulation of post-transcriptional gene silencin	24	1	7.59E-02
GO:0060967	negative regulation of gene silencing by RNA	24	1	7.59E-02
GO:0072202	cell differentiation involved in metanephros developme	24	1	7.59E-02
GO:1900369	negative regulation of post-transcriptional gene silencin	24	1	7.59E-02
GO:0032982	myosin filament	24	1	7.59E-02

GO:0042974	nuclear retinoic acid receptor binding	24	1	7.59E-02
GO:0070412	R-SMAD binding	24	1	7.59E-02
GO:0065007	biological regulation	13067	49	7.62E-02
GO:0042803	protein homodimerization activity	691	5	7.62E-02
GO:0002020	protease binding	139	2	7.64E-02
GO:0016922	nuclear receptor binding	139	2	7.64E-02
GO:0022407	regulation of cell-cell adhesion	490	4	7.72E-02
GO:0009582	detection of abiotic stimulus	140	2	7.73E-02
GO:0030833	regulation of actin filament polymerization	140	2	7.73E-02
GO:0048813	dendrite morphogenesis	140	2	7.73E-02
GO:0048018	receptor ligand activity	491	4	7.77E-02
GO:0051234	establishment of localization	4734	21	7.85E-02
GO:0017121	plasma membrane phospholipid scrambling	25	1	7.89E-02
GO:0018904	ether metabolic process	25	1	7.89E-02
GO:0021801	cerebral cortex radial glia-guided migration	25	1	7.89E-02
GO:0021854	hypothalamus development	25	1	7.89E-02
GO:0022030	telencephalon glial cell migration	25	1	7.89E-02
GO:0032331	negative regulation of chondrocyte differentiation	25	1	7.89E-02
GO:0034114	regulation of heterotypic cell-cell adhesion	25	1	7.89E-02
GO:0045649	regulation of macrophage differentiation	25	1	7.89E-02
GO:0045662	negative regulation of myoblast differentiation	25	1	7.89E-02
GO:0045980	negative regulation of nucleotide metabolic process	25	1	7.89E-02
GO:0050927	positive regulation of positive chemotaxis	25	1	7.89E-02
GO:0062149	detection of stimulus involved in sensory perception of	25	1	7.89E-02
GO:0090023	positive regulation of neutrophil chemotaxis	25	1	7.89E-02
GO:0098581	detection of external biotic stimulus	25	1	7.89E-02
GO:1905563	negative regulation of vascular endothelial cell prolifera	25	1	7.89E-02
GO:2000311	regulation of AMPA receptor activity	25	1	7.89E-02
GO:0030057	desmosome	25	1	7.89E-02
GO:0005227	calcium activated cation channel activity	25	1	7.89E-02
GO:0050431	transforming growth factor beta binding	25	1	7.89E-02
GO:0014074	response to purine-containing compound	142	2	7.92E-02
GO:0048608	reproductive structure development	306	3	7.93E-02
GO:0044403	biological process involved in symbiotic interaction	307	3	7.99E-02
GO:0010324	membrane invagination	143	2	8.02E-02
GO:0055076	transition metal ion homeostasis	143	2	8.02E-02
GO:0030546	signaling receptor activator activity	498	4	8.08E-02
GO:0036293	response to decreased oxygen levels	309	3	8.11E-02
GO:0030177	positive regulation of Wnt signaling pathway	144	2	8.12E-02
GO:0034330	cell junction organization	705	5	8.15E-02
GO:0050776	regulation of immune response	922	6	8.15E-02
GO:0061458	reproductive system development	310	3	8.18E-02
GO:0006929	substrate-dependent cell migration	26	1	8.19E-02
GO:0050926	regulation of positive chemotaxis	26	1	8.19E-02
GO:0060333	interferon-gamma-mediated signaling pathway	26	1	8.19E-02
GO:0060343	trabecula formation	26	1	8.19E-02
GO:0060740	prostate gland epithelium morphogenesis	26	1	8.19E-02

GO:0072010	glomerular epithelium development	26	1	8.19E-02
GO:1904753	negative regulation of vascular associated smooth musc	26	1	8.19E-02
GO:0001848	complement binding	26	1	8.19E-02
GO:0008527	taste receptor activity	26	1	8.19E-02
GO:0016192	vesicle-mediated transport	1621	9	8.20E-02
GO:0031644	regulation of nervous system process	145	2	8.21E-02
GO:0045089	positive regulation of innate immune response	145	2	8.21E-02
GO:0072562	blood microparticle	145	2	8.21E-02
GO:0015629	actin cytoskeleton	502	4	8.27E-02
GO:0022604	regulation of cell morphogenesis	312	3	8.30E-02
GO:0031333	negative regulation of protein-containing complex asser	146	2	8.31E-02
GO:0071495	cellular response to endogenous stimulus	1387	8	8.32E-02
GO:0060485	mesenchyme development	313	3	8.36E-02
GO:0009991	response to extracellular stimulus	504	4	8.36E-02
GO:1901699	cellular response to nitrogen compound	711	5	8.38E-02
GO:0098588	bounding membrane of organelle	2122	11	8.40E-02
GO:0008584	male gonad development	147	2	8.41E-02
GO:0017015	regulation of transforming growth factor beta receptor	147	2	8.41E-02
GO:0045598	regulation of fat cell differentiation	147	2	8.41E-02
GO:0071363	cellular response to growth factor stimulus	712	5	8.41E-02
GO:0030325	adrenal gland development	27	1	8.50E-02
GO:0032967	positive regulation of collagen biosynthetic process	27	1	8.50E-02
GO:0036296	response to increased oxygen levels	27	1	8.50E-02
GO:0051291	protein heterooligomerization	27	1	8.50E-02
GO:0071467	cellular response to pH	27	1	8.50E-02
GO:0072207	metanephric epithelium development	27	1	8.50E-02
GO:1903055	positive regulation of extracellular matrix organization	27	1	8.50E-02
GO:0034364	high-density lipoprotein particle	27	1	8.50E-02
GO:0038187	pattern recognition receptor activity	27	1	8.50E-02
GO:0080025	phosphatidylinositol-3,5-bisphosphate binding	27	1	8.50E-02
GO:0046546	development of primary male sexual characteristics	148	2	8.50E-02
GO:0016525	negative regulation of angiogenesis	149	2	8.60E-02
GO:0045595	regulation of cell differentiation	1639	9	8.65E-02
GO:0045596	negative regulation of cell differentiation	718	5	8.65E-02
GO:1903844	regulation of cellular response to transforming growth f	150	2	8.70E-02
GO:0010714	positive regulation of collagen metabolic process	28	1	8.80E-02
GO:0010894	negative regulation of steroid biosynthetic process	28	1	8.80E-02
GO:0060512	prostate gland morphogenesis	28	1	8.80E-02
GO:0071624	positive regulation of granulocyte chemotaxis	28	1	8.80E-02
GO:0043495	protein-membrane adaptor activity	28	1	8.80E-02
GO:0046718	viral entry into host cell	151	2	8.80E-02
GO:2000181	negative regulation of blood vessel morphogenesis	151	2	8.80E-02
GO:0062013	positive regulation of small molecule metabolic process	152	2	8.90E-02
GO:1901343	negative regulation of vasculature development	152	2	8.90E-02
GO:0010804	negative regulation of tumor necrosis factor-mediated s	29	1	9.10E-02
GO:0015701	bicarbonate transport	29	1	9.10E-02
GO:0051873	killing by host of symbiont cells	29	1	9.10E-02

GO:0060074	synapse maturation	29	1	9.10E-02
GO:0090025	regulation of monocyte chemotaxis	29	1	9.10E-02
GO:0070325	lipoprotein particle receptor binding	29	1	9.10E-02
GO:0008360	regulation of cell shape	154	2	9.10E-02
GO:0045834	positive regulation of lipid metabolic process	154	2	9.10E-02
GO:0050729	positive regulation of inflammatory response	154	2	9.10E-02
GO:0048468	cell development	2155	11	9.14E-02
GO:0032409	regulation of transporter activity	326	3	9.18E-02
GO:0002009	morphogenesis of an epithelium	521	4	9.18E-02
GO:0010675	regulation of cellular carbohydrate metabolic process	155	2	9.20E-02
GO:0032368	regulation of lipid transport	155	2	9.20E-02
GO:0005766	primary lysosome	155	2	9.20E-02
GO:0042582	azurophil granule	155	2	9.20E-02
GO:0008643	carbohydrate transport	156	2	9.30E-02
GO:0034728	nucleosome organization	156	2	9.30E-02
GO:0019220	regulation of phosphate metabolic process	1423	8	9.32E-02
GO:0051174	regulation of phosphorus metabolic process	1424	8	9.35E-02
GO:0001556	oocyte maturation	30	1	9.39E-02
GO:0007271	synaptic transmission, cholinergic	30	1	9.39E-02
GO:0010447	response to acidic pH	30	1	9.39E-02
GO:0034368	protein-lipid complex remodeling	30	1	9.39E-02
GO:0034369	plasma lipoprotein particle remodeling	30	1	9.39E-02
GO:0060325	face morphogenesis	30	1	9.39E-02
GO:0070293	renal absorption	30	1	9.39E-02
GO:0090140	regulation of mitochondrial fission	30	1	9.39E-02
GO:2000463	positive regulation of excitatory postsynaptic potential	30	1	9.39E-02
GO:0030125	clathrin vesicle coat	30	1	9.39E-02
GO:0042277	peptide binding	330	3	9.43E-02
GO:0030659	cytoplasmic vesicle membrane	1190	7	9.44E-02
GO:1901564	organonitrogen compound metabolic process	6535	27	9.45E-02
GO:0045184	establishment of protein localization	1671	9	9.48E-02
GO:0044409	entry into host	158	2	9.50E-02
GO:0090101	negative regulation of transmembrane receptor protein	158	2	9.50E-02
GO:0007267	cell-cell signaling	1672	9	9.51E-02
GO:0007605	sensory perception of sound	159	2	9.60E-02
GO:0008064	regulation of actin polymerization or depolymerization	159	2	9.60E-02
GO:0060249	anatomical structure homeostasis	333	3	9.63E-02
GO:0001945	lymph vessel development	31	1	9.69E-02
GO:0003299	muscle hypertrophy in response to stress	31	1	9.69E-02
GO:0014887	cardiac muscle adaptation	31	1	9.69E-02
GO:0014898	cardiac muscle hypertrophy in response to stress	31	1	9.69E-02
GO:0021591	ventricular system development	31	1	9.69E-02
GO:0031069	hair follicle morphogenesis	31	1	9.69E-02
GO:0034505	tooth mineralization	31	1	9.69E-02
GO:0045939	negative regulation of steroid metabolic process	31	1	9.69E-02
GO:0045987	positive regulation of smooth muscle contraction	31	1	9.69E-02
GO:0048384	retinoic acid receptor signaling pathway	31	1	9.69E-02

GO:0060390	regulation of SMAD protein signal transduction	31	1	9.69E-02
GO:0071295	cellular response to vitamin	31	1	9.69E-02
GO:0097106	postsynaptic density organization	31	1	9.69E-02
GO:0000803	sex chromosome	31	1	9.69E-02
GO:0008305	integrin complex	31	1	9.69E-02
GO:0042788	polysomal ribosome	31	1	9.69E-02
GO:0001968	fibronectin binding	31	1	9.69E-02
GO:0140323	solute:anion antiporter activity	31	1	9.69E-02
GO:0006810	transport	4578	20	9.74E-02
GO:0071214	cellular response to abiotic stimulus	335	3	9.76E-02
GO:0104004	cellular response to environmental stimulus	335	3	9.76E-02
GO:0051604	protein maturation	336	3	9.82E-02
GO:1990778	protein localization to cell periphery	337	3	9.89E-02
GO:0030832	regulation of actin filament length	162	2	9.91E-02
GO:0008083	growth factor activity	162	2	9.91E-02
GO:0048871	multicellular organismal homeostasis	536	4	9.92E-02
GO:0050896	response to stimulus	9216	36	9.93E-02
GO:0006335	DNA replication-dependent chromatin assembly	32	1	9.99E-02
GO:0034311	diol metabolic process	32	1	9.99E-02
GO:0034367	protein-containing complex remodeling	32	1	9.99E-02
GO:0034390	smooth muscle cell apoptotic process	32	1	9.99E-02
GO:0034391	regulation of smooth muscle cell apoptotic process	32	1	9.99E-02
GO:0034508	centromere complex assembly	32	1	9.99E-02
GO:0061037	negative regulation of cartilage development	32	1	9.99E-02
GO:1901889	negative regulation of cell junction assembly	32	1	9.99E-02
GO:0001934	positive regulation of protein phosphorylation	751	5	1.00E-01
GO:0048545	response to steroid hormone	339	3	1.00E-01
GO:0062012	regulation of small molecule metabolic process	339	3	1.00E-01
GO:0072359	circulatory system development	1209	7	1.01E-01
GO:0012506	vesicle membrane	1209	7	1.01E-01
GO:0002768	immune response-regulating cell surface receptor signa	341	3	1.02E-01
GO:0010008	endosome membrane	541	4	1.02E-01
GO:0031960	response to corticosteroid	165	2	1.02E-01
GO:0051235	maintenance of location	342	3	1.02E-01
GO:0033032	regulation of myeloid cell apoptotic process	33	1	1.03E-01
GO:0050974	detection of mechanical stimulus involved in sensory pe	33	1	1.03E-01
GO:0051497	negative regulation of stress fiber assembly	33	1	1.03E-01
GO:0051968	positive regulation of synaptic transmission, glutamater	33	1	1.03E-01
GO:0090183	regulation of kidney development	33	1	1.03E-01
GO:0098801	regulation of renal system process	33	1	1.03E-01
GO:1904893	negative regulation of receptor signaling pathway via ST	33	1	1.03E-01
GO:0005154	epidermal growth factor receptor binding	33	1	1.03E-01
GO:0030139	endocytic vesicle	343	3	1.03E-01
GO:0007009	plasma membrane organization	166	2	1.03E-01
GO:0060627	regulation of vesicle-mediated transport	544	4	1.03E-01
GO:0050789	regulation of biological process	12322	46	1.04E-01
GO:0001959	regulation of cytokine-mediated signaling pathway	167	2	1.04E-01

GO:0030545	signaling receptor regulator activity	546	4	1.04E-01
GO:0000781	chromosome, telomeric region	168	2	1.05E-01
GO:0097730	non-motile cilium	168	2	1.05E-01
GO:0019216	regulation of lipid metabolic process	347	3	1.06E-01
GO:0030674	protein-macromolecule adaptor activity	347	3	1.06E-01
GO:0021799	cerebral cortex radially oriented cell migration	34	1	1.06E-01
GO:0034383	low-density lipoprotein particle clearance	34	1	1.06E-01
GO:0046475	glycerophospholipid catabolic process	34	1	1.06E-01
GO:0048333	mesodermal cell differentiation	34	1	1.06E-01
GO:0055094	response to lipoprotein particle	34	1	1.06E-01
GO:0090022	regulation of neutrophil chemotaxis	34	1	1.06E-01
GO:0099084	postsynaptic specialization organization	34	1	1.06E-01
GO:1902624	positive regulation of neutrophil migration	34	1	1.06E-01
GO:0005834	heterotrimeric G-protein complex	34	1	1.06E-01
GO:0090543	Flemming body	34	1	1.06E-01
GO:0001837	epithelial to mesenchymal transition	169	2	1.06E-01
GO:0002262	myeloid cell homeostasis	169	2	1.06E-01
GO:2000026	regulation of multicellular organismal development	1467	8	1.06E-01
GO:0008092	cytoskeletal protein binding	993	6	1.07E-01
GO:0007156	homophilic cell adhesion via plasma membrane adhesio	170	2	1.07E-01
GO:0030041	actin filament polymerization	170	2	1.07E-01
GO:0050806	positive regulation of synaptic transmission	170	2	1.07E-01
GO:0046661	male sex differentiation	171	2	1.08E-01
GO:0006779	porphyrin-containing compound biosynthetic process	35	1	1.09E-01
GO:0010661	positive regulation of muscle cell apoptotic process	35	1	1.09E-01
GO:0010737	protein kinase A signaling	35	1	1.09E-01
GO:0033014	tetrapyrrole biosynthetic process	35	1	1.09E-01
GO:0035633	maintenance of blood-brain barrier	35	1	1.09E-01
GO:0048048	embryonic eye morphogenesis	35	1	1.09E-01
GO:0048730	epidermis morphogenesis	35	1	1.09E-01
GO:0051354	negative regulation of oxidoreductase activity	35	1	1.09E-01
GO:1901099	negative regulation of signal transduction in absence of	35	1	1.09E-01
GO:2001240	negative regulation of extrinsic apoptotic signaling path	35	1	1.09E-01
GO:0005488	binding	16619	59	1.09E-01
GO:0019897	extrinsic component of plasma membrane	172	2	1.09E-01
GO:0051093	negative regulation of developmental process	1002	6	1.10E-01
GO:0050839	cell adhesion molecule binding	558	4	1.11E-01
GO:0031214	biomineral tissue development	174	2	1.12E-01
GO:0007616	long-term memory	36	1	1.12E-01
GO:0010614	negative regulation of cardiac muscle hypertrophy	36	1	1.12E-01
GO:0010922	positive regulation of phosphatase activity	36	1	1.12E-01
GO:0016486	peptide hormone processing	36	1	1.12E-01
GO:0030224	monocyte differentiation	36	1	1.12E-01
GO:0031646	positive regulation of nervous system process	36	1	1.12E-01
GO:0032232	negative regulation of actin filament bundle assembly	36	1	1.12E-01
GO:0048261	negative regulation of receptor-mediated endocytosis	36	1	1.12E-01
GO:0048536	spleen development	36	1	1.12E-01

GO:0050775	positive regulation of dendrite morphogenesis	36	1	1.12E-01
GO:0060323	head morphogenesis	36	1	1.12E-01
GO:0034358	plasma lipoprotein particle	36	1	1.12E-01
GO:1990777	lipoprotein particle	36	1	1.12E-01
GO:0000217	DNA secondary structure binding	36	1	1.12E-01
GO:0004623	phospholipase A2 activity	36	1	1.12E-01
GO:0010469	regulation of signaling receptor activity	175	2	1.13E-01
GO:0005775	vacuolar lumen	176	2	1.14E-01
GO:0001568	blood vessel development	784	5	1.14E-01
GO:0001580	detection of chemical stimulus involved in sensory perc	37	1	1.15E-01
GO:0001941	postsynaptic membrane organization	37	1	1.15E-01
GO:0033144	negative regulation of intracellular steroid hormone rec	37	1	1.15E-01
GO:0044319	wound healing, spreading of cells	37	1	1.15E-01
GO:0060999	positive regulation of dendritic spine development	37	1	1.15E-01
GO:0071402	cellular response to lipoprotein particle stimulus	37	1	1.15E-01
GO:0090505	epiboly involved in wound healing	37	1	1.15E-01
GO:2000279	negative regulation of DNA biosynthetic process	37	1	1.15E-01
GO:0030140	trans-Golgi network transport vesicle	37	1	1.15E-01
GO:0016409	palmitoyltransferase activity	37	1	1.15E-01
GO:0001764	neuron migration	177	2	1.15E-01
GO:0071772	response to BMP	178	2	1.16E-01
GO:0071773	cellular response to BMP stimulus	178	2	1.16E-01
GO:0044815	DNA packaging complex	178	2	1.16E-01
GO:0099513	polymeric cytoskeletal fiber	789	5	1.17E-01
GO:0031401	positive regulation of protein modification process	1019	6	1.17E-01
GO:0048771	tissue remodeling	179	2	1.17E-01
GO:0060759	regulation of response to cytokine stimulus	179	2	1.17E-01
GO:0014741	negative regulation of muscle hypertrophy	38	1	1.17E-01
GO:0030851	granulocyte differentiation	38	1	1.17E-01
GO:0032717	negative regulation of interleukin-8 production	38	1	1.17E-01
GO:0045652	regulation of megakaryocyte differentiation	38	1	1.17E-01
GO:0090504	epiboly	38	1	1.17E-01
GO:0140448	signaling receptor ligand precursor processing	38	1	1.17E-01
GO:0004866	endopeptidase inhibitor activity	180	2	1.18E-01
GO:0032956	regulation of actin cytoskeleton organization	365	3	1.18E-01
GO:0043409	negative regulation of MAPK cascade	181	2	1.19E-01
GO:0110148	biomineralization	181	2	1.19E-01
GO:0043086	negative regulation of catalytic activity	796	5	1.20E-01
GO:0030336	negative regulation of cell migration	368	3	1.20E-01
GO:0071692	protein localization to extracellular region	368	3	1.20E-01
GO:0002385	mucosal immune response	39	1	1.20E-01
GO:0009595	detection of biotic stimulus	39	1	1.20E-01
GO:0033028	myeloid cell apoptotic process	39	1	1.20E-01
GO:0071634	regulation of transforming growth factor beta productio	39	1	1.20E-01
GO:0097242	amyloid-beta clearance	39	1	1.20E-01
GO:0032994	protein-lipid complex	39	1	1.20E-01
GO:0099738	cell cortex region	39	1	1.20E-01

GO:0005515	protein binding	14027	51	1.21E-01
GO:0031589	cell-substrate adhesion	369	3	1.21E-01
GO:0001936	regulation of endothelial cell proliferation	183	2	1.21E-01
GO:0044000	movement in host	183	2	1.21E-01
GO:0050728	negative regulation of inflammatory response	184	2	1.22E-01
GO:0000266	mitochondrial fission	40	1	1.23E-01
GO:0001953	negative regulation of cell-matrix adhesion	40	1	1.23E-01
GO:0032941	secretion by tissue	40	1	1.23E-01
GO:0032965	regulation of collagen biosynthetic process	40	1	1.23E-01
GO:0071392	cellular response to estradiol stimulus	40	1	1.23E-01
GO:0098751	bone cell development	40	1	1.23E-01
GO:1990000	amyloid fibril formation	40	1	1.23E-01
GO:1905360	GTPase complex	40	1	1.23E-01
GO:0031492	nucleosomal DNA binding	40	1	1.23E-01
GO:0071407	cellular response to organic cyclic compound	581	4	1.23E-01
GO:0032200	telomere organization	185	2	1.23E-01
GO:1905952	regulation of lipid localization	185	2	1.23E-01
GO:1905114	cell surface receptor signaling pathway involved in cell-c	582	4	1.24E-01
GO:0006109	regulation of carbohydrate metabolic process	186	2	1.24E-01
GO:0043405	regulation of MAP kinase activity	186	2	1.24E-01
GO:0090130	tissue migration	374	3	1.25E-01
GO:0002221	pattern recognition receptor signaling pathway	187	2	1.26E-01
GO:0030414	peptidase inhibitor activity	187	2	1.26E-01
GO:0014912	negative regulation of smooth muscle cell migration	41	1	1.26E-01
GO:0072210	metanephric nephron development	41	1	1.26E-01
GO:0038023	signaling receptor activity	1531	8	1.27E-01
GO:0060089	molecular transducer activity	1531	8	1.27E-01
GO:0007565	female pregnancy	189	2	1.28E-01
GO:0051302	regulation of cell division	190	2	1.29E-01
GO:0021983	pituitary gland development	42	1	1.29E-01
GO:0031670	cellular response to nutrient	42	1	1.29E-01
GO:0050913	sensory perception of bitter taste	42	1	1.29E-01
GO:0071604	transforming growth factor beta production	42	1	1.29E-01
GO:0042287	MHC protein binding	42	1	1.29E-01
GO:0042326	negative regulation of phosphorylation	381	3	1.30E-01
GO:0042742	defense response to bacterium	382	3	1.30E-01
GO:0044057	regulation of system process	594	4	1.31E-01
GO:0050877	nervous system process	1541	8	1.31E-01
GO:0009755	hormone-mediated signaling pathway	192	2	1.31E-01
GO:2000146	negative regulation of cell motility	383	3	1.31E-01
GO:0010243	response to organonitrogen compound	1054	6	1.31E-01
GO:1902903	regulation of supramolecular fiber organization	384	3	1.32E-01
GO:0002251	organ or tissue specific immune response	43	1	1.32E-01
GO:0031952	regulation of protein autophosphorylation	43	1	1.32E-01
GO:0042789	mRNA transcription by RNA polymerase II	43	1	1.32E-01
GO:0071364	cellular response to epidermal growth factor stimulus	43	1	1.32E-01
GO:0071459	protein localization to chromosome, centromeric regior	43	1	1.32E-01

GO:0030666	endocytic vesicle membrane	194	2	1.33E-01
GO:0061135	endopeptidase regulator activity	194	2	1.33E-01
GO:0048878	chemical homeostasis	1060	6	1.34E-01
GO:0014075	response to amine	44	1	1.35E-01
GO:0014911	positive regulation of smooth muscle cell migration	44	1	1.35E-01
GO:0021762	substantia nigra development	44	1	1.35E-01
GO:0061001	regulation of dendritic spine morphogenesis	44	1	1.35E-01
GO:0090317	negative regulation of intracellular protein transport	44	1	1.35E-01
GO:0098815	modulation of excitatory postsynaptic potential	44	1	1.35E-01
GO:0030118	clathrin coat	44	1	1.35E-01
GO:0001046	core promoter sequence-specific DNA binding	44	1	1.35E-01
GO:0022839	ion gated channel activity	44	1	1.35E-01
GO:0055080	cation homeostasis	601	4	1.35E-01
GO:0042327	positive regulation of phosphorylation	828	5	1.35E-01
GO:0007178	transmembrane receptor protein serine/threonine kina	390	3	1.36E-01
GO:0001825	blastocyst formation	45	1	1.38E-01
GO:0009268	response to pH	45	1	1.38E-01
GO:0010712	regulation of collagen metabolic process	45	1	1.38E-01
GO:0060964	regulation of miRNA-mediated gene silencing	45	1	1.38E-01
GO:0071827	plasma lipoprotein particle organization	45	1	1.38E-01
GO:0022627	cytosolic small ribosomal subunit	45	1	1.38E-01
GO:0050778	positive regulation of immune response	607	4	1.38E-01
GO:0001935	endothelial cell proliferation	199	2	1.39E-01
GO:0003013	circulatory system process	609	4	1.39E-01
GO:0008154	actin polymerization or depolymerization	200	2	1.40E-01
GO:0043393	regulation of protein binding	200	2	1.40E-01
GO:0021517	ventral spinal cord development	46	1	1.40E-01
GO:0021795	cerebral cortex cell migration	46	1	1.40E-01
GO:1900271	regulation of long-term synaptic potentiation	46	1	1.40E-01
GO:1904752	regulation of vascular associated smooth muscle cell mi	46	1	1.40E-01
GO:2000273	positive regulation of signaling receptor activity	46	1	1.40E-01
GO:2001239	regulation of extrinsic apoptotic signaling pathway in ab	46	1	1.40E-01
GO:0005272	sodium channel activity	46	1	1.40E-01
GO:0032271	regulation of protein polymerization	201	2	1.41E-01
GO:0022408	negative regulation of cell-cell adhesion	202	2	1.42E-01
GO:0050900	leukocyte migration	398	3	1.42E-01
GO:0006110	regulation of glycolytic process	47	1	1.43E-01
GO:0061383	trabecula morphogenesis	47	1	1.43E-01
GO:0070849	response to epidermal growth factor	47	1	1.43E-01
GO:0120163	negative regulation of cold-induced thermogenesis	47	1	1.43E-01
GO:1900368	regulation of post-transcriptional gene silencing by RNA	47	1	1.43E-01
GO:1902622	regulation of neutrophil migration	47	1	1.43E-01
GO:1902895	positive regulation of miRNA transcription	47	1	1.43E-01
GO:2001222	regulation of neuron migration	47	1	1.43E-01
GO:0051701	biological process involved in interaction with host	203	2	1.43E-01
GO:0098771	inorganic ion homeostasis	616	4	1.44E-01
GO:0034762	regulation of transmembrane transport	618	4	1.45E-01

GO:0006953	acute-phase response	48	1	1.46E-01
GO:0007528	neuromuscular junction development	48	1	1.46E-01
GO:0009299	mRNA transcription	48	1	1.46E-01
GO:0032008	positive regulation of TOR signaling	48	1	1.46E-01
GO:0035850	epithelial cell differentiation involved in kidney develop	48	1	1.46E-01
GO:0045776	negative regulation of blood pressure	48	1	1.46E-01
GO:0060147	regulation of post-transcriptional gene silencing	48	1	1.46E-01
GO:0071825	protein-lipid complex subunit organization	48	1	1.46E-01
GO:1904738	vascular associated smooth muscle cell migration	48	1	1.46E-01
GO:0034284	response to monosaccharide	207	2	1.48E-01
GO:0032970	regulation of actin filament-based process	406	3	1.48E-01
GO:0051128	regulation of cellular component organization	2369	11	1.49E-01
GO:0010862	positive regulation of pathway-restricted SMAD protein	49	1	1.49E-01
GO:0030261	chromosome condensation	49	1	1.49E-01
GO:0035307	positive regulation of protein dephosphorylation	49	1	1.49E-01
GO:0045933	positive regulation of muscle contraction	49	1	1.49E-01
GO:0005504	fatty acid binding	49	1	1.49E-01
GO:0070888	E-box binding	49	1	1.49E-01
GO:0032869	cellular response to insulin stimulus	208	2	1.49E-01
GO:0050801	ion homeostasis	626	4	1.50E-01
GO:0051239	regulation of multicellular organismal process	2907	13	1.50E-01
GO:0033218	amide binding	408	3	1.50E-01
GO:0030100	regulation of endocytosis	209	2	1.50E-01
GO:0044703	multi-organism reproductive process	209	2	1.50E-01
GO:0007623	circadian rhythm	210	2	1.51E-01
GO:0043254	regulation of protein-containing complex assembly	410	3	1.52E-01
GO:0060966	regulation of gene silencing by RNA	50	1	1.52E-01
GO:0009725	response to hormone	861	5	1.52E-01
GO:0030225	macrophage differentiation	51	1	1.54E-01
GO:0030850	prostate gland development	51	1	1.54E-01
GO:0043124	negative regulation of I-kappaB kinase/NF-kappaB signa	51	1	1.54E-01
GO:0043277	apoptotic cell clearance	51	1	1.54E-01
GO:0050873	brown fat cell differentiation	51	1	1.54E-01
GO:0060324	face development	51	1	1.54E-01
GO:0101023	vascular endothelial cell proliferation	51	1	1.54E-01
GO:1905562	regulation of vascular endothelial cell proliferation	51	1	1.54E-01
GO:0002064	epithelial cell development	213	2	1.55E-01
GO:0030258	lipid modification	213	2	1.55E-01
GO:0045637	regulation of myeloid cell differentiation	214	2	1.56E-01
GO:0032879	regulation of localization	2129	10	1.56E-01
GO:0016064	immunoglobulin mediated immune response	215	2	1.57E-01
GO:0002762	negative regulation of myeloid leukocyte differentiation	52	1	1.57E-01
GO:0010543	regulation of platelet activation	52	1	1.57E-01
GO:0010823	negative regulation of mitochondrion organization	52	1	1.57E-01
GO:0060612	adipose tissue development	52	1	1.57E-01
GO:0004879	nuclear receptor activity	52	1	1.57E-01
GO:0030159	signaling receptor complex adaptor activity	52	1	1.57E-01

GO:0098531	ligand-activated transcription factor activity	52	1	1.57E-01
GO:0006469	negative regulation of protein kinase activity	216	2	1.58E-01
GO:0040013	negative regulation of locomotion	419	3	1.59E-01
GO:0006111	regulation of gluconeogenesis	53	1	1.60E-01
GO:0006778	porphyrin-containing compound metabolic process	53	1	1.60E-01
GO:0034381	plasma lipoprotein particle clearance	53	1	1.60E-01
GO:0045332	phospholipid translocation	53	1	1.60E-01
GO:0060071	Wnt signaling pathway, planar cell polarity pathway	53	1	1.60E-01
GO:0071622	regulation of granulocyte chemotaxis	53	1	1.60E-01
GO:0016032	viral process	421	3	1.60E-01
GO:0043434	response to peptide hormone	421	3	1.60E-01
GO:0002573	myeloid leukocyte differentiation	218	2	1.60E-01
GO:0019724	B cell mediated immunity	218	2	1.60E-01
GO:0042180	cellular ketone metabolic process	218	2	1.60E-01
GO:0044706	multi-multicellular organism process	218	2	1.60E-01
GO:0005925	focal adhesion	422	3	1.61E-01
GO:0051015	actin filament binding	219	2	1.62E-01
GO:0061024	membrane organization	881	5	1.62E-01
GO:0009395	phospholipid catabolic process	54	1	1.63E-01
GO:0010883	regulation of lipid storage	54	1	1.63E-01
GO:0032330	regulation of chondrocyte differentiation	54	1	1.63E-01
GO:0071168	protein localization to chromatin	54	1	1.63E-01
GO:0015459	potassium channel regulator activity	54	1	1.63E-01
GO:0004175	endopeptidase activity	426	3	1.64E-01
GO:0010810	regulation of cell-substrate adhesion	222	2	1.65E-01
GO:0044242	cellular lipid catabolic process	222	2	1.65E-01
GO:0034703	cation channel complex	222	2	1.65E-01
GO:0014888	striated muscle adaptation	55	1	1.66E-01
GO:0031103	axon regeneration	55	1	1.66E-01
GO:0038093	Fc receptor signaling pathway	55	1	1.66E-01
GO:0045668	negative regulation of osteoblast differentiation	55	1	1.66E-01
GO:0071320	cellular response to cAMP	55	1	1.66E-01
GO:0072132	mesenchyme morphogenesis	55	1	1.66E-01
GO:0030170	pyridoxal phosphate binding	55	1	1.66E-01
GO:0052548	regulation of endopeptidase activity	428	3	1.66E-01
GO:0071805	potassium ion transmembrane transport	223	2	1.66E-01
GO:0010718	positive regulation of epithelial to mesenchymal transit	56	1	1.68E-01
GO:0043470	regulation of carbohydrate catabolic process	56	1	1.68E-01
GO:0045806	negative regulation of endocytosis	56	1	1.68E-01
GO:0060986	endocrine hormone secretion	56	1	1.68E-01
GO:0060998	regulation of dendritic spine development	56	1	1.68E-01
GO:0097120	receptor localization to synapse	56	1	1.68E-01
GO:2000630	positive regulation of miRNA metabolic process	56	1	1.68E-01
GO:0070279	vitamin B6 binding	56	1	1.68E-01
GO:0032993	protein-DNA complex	225	2	1.68E-01
GO:0009410	response to xenobiotic stimulus	432	3	1.69E-01
GO:0030055	cell-substrate junction	432	3	1.69E-01

GO:0006869	lipid transport	433	3	1.70E-01
GO:0006979	response to oxidative stress	434	3	1.71E-01
GO:0048705	skeletal system morphogenesis	227	2	1.71E-01
GO:0008347	glial cell migration	57	1	1.71E-01
GO:0090175	regulation of establishment of planar polarity	57	1	1.71E-01
GO:0030120	vesicle coat	57	1	1.71E-01
GO:0030660	Golgi-associated vesicle membrane	57	1	1.71E-01
GO:1901698	response to nitrogen compound	1142	6	1.71E-01
GO:0032102	negative regulation of response to external stimulus	436	3	1.72E-01
GO:0006826	iron ion transport	58	1	1.74E-01
GO:0010803	regulation of tumor necrosis factor-mediated signaling	58	1	1.74E-01
GO:0034204	lipid translocation	58	1	1.74E-01
GO:0048008	platelet-derived growth factor receptor signaling pathw	58	1	1.74E-01
GO:0048599	oocyte development	58	1	1.74E-01
GO:0060997	dendritic spine morphogenesis	58	1	1.74E-01
GO:0061005	cell differentiation involved in kidney development	58	1	1.74E-01
GO:0045936	negative regulation of phosphate metabolic process	440	3	1.75E-01
GO:0010563	negative regulation of phosphorus metabolic process	441	3	1.76E-01
GO:0048732	gland development	441	3	1.76E-01
GO:0007566	embryo implantation	59	1	1.76E-01
GO:0051289	protein homotetramerization	59	1	1.76E-01
GO:0061098	positive regulation of protein tyrosine kinase activity	59	1	1.76E-01
GO:0097352	autophagosome maturation	59	1	1.76E-01
GO:0001533	cornified envelope	59	1	1.76E-01
GO:0022625	cytosolic large ribosomal subunit	59	1	1.76E-01
GO:0005840	ribosome	232	2	1.77E-01
GO:0061134	peptidase regulator activity	232	2	1.77E-01
GO:0008406	gonad development	233	2	1.78E-01
GO:0009743	response to carbohydrate	234	2	1.79E-01
GO:0046883	regulation of hormone secretion	234	2	1.79E-01
GO:0071356	cellular response to tumor necrosis factor	234	2	1.79E-01
GO:0002011	morphogenesis of an epithelial sheet	60	1	1.79E-01
GO:0022029	telencephalon cell migration	60	1	1.79E-01
GO:0045599	negative regulation of fat cell differentiation	60	1	1.79E-01
GO:0004714	transmembrane receptor protein tyrosine kinase activit	60	1	1.79E-01
GO:0051606	detection of stimulus	673	4	1.79E-01
GO:0045597	positive regulation of cell differentiation	915	5	1.81E-01
GO:0007411	axon guidance	236	2	1.81E-01
GO:0034113	heterotypic cell-cell adhesion	61	1	1.82E-01
GO:1902893	regulation of miRNA transcription	61	1	1.82E-01
GO:0050804	modulation of chemical synaptic transmission	448	3	1.82E-01
GO:0016358	dendrite development	237	2	1.83E-01
GO:0097485	neuron projection guidance	237	2	1.83E-01
GO:0005125	cytokine activity	237	2	1.83E-01
GO:0033993	response to lipid	918	5	1.83E-01
GO:0099177	regulation of trans-synaptic signaling	449	3	1.83E-01
GO:0007160	cell-matrix adhesion	238	2	1.84E-01

GO:0045137	development of primary sexual characteristics	238	2	1.84E-01
GO:0031410	cytoplasmic vesicle	2479	11	1.85E-01
GO:0009994	oocyte differentiation	62	1	1.85E-01
GO:0031102	neuron projection regeneration	62	1	1.85E-01
GO:0032387	negative regulation of intracellular transport	62	1	1.85E-01
GO:0046888	negative regulation of hormone secretion	62	1	1.85E-01
GO:0060393	regulation of pathway-restricted SMAD protein phospho	62	1	1.85E-01
GO:0061614	miRNA transcription	62	1	1.85E-01
GO:0099601	regulation of neurotransmitter receptor activity	62	1	1.85E-01
GO:0097708	intracellular vesicle	2482	11	1.86E-01
GO:0030595	leukocyte chemotaxis	240	2	1.86E-01
GO:0033673	negative regulation of kinase activity	240	2	1.86E-01
GO:0007015	actin filament organization	454	3	1.87E-01
GO:0051090	regulation of DNA-binding transcription factor activity	454	3	1.87E-01
GO:0021885	forebrain cell migration	63	1	1.87E-01
GO:0030514	negative regulation of BMP signaling pathway	63	1	1.87E-01
GO:0032757	positive regulation of interleukin-8 production	63	1	1.87E-01
GO:0046173	polyol biosynthetic process	63	1	1.87E-01
GO:0051055	negative regulation of lipid biosynthetic process	63	1	1.87E-01
GO:0070542	response to fatty acid	63	1	1.87E-01
GO:0097035	regulation of membrane lipid distribution	63	1	1.87E-01
GO:0005771	multivesicular body	63	1	1.87E-01
GO:0010628	positive regulation of gene expression	1177	6	1.88E-01
GO:0065004	protein-DNA complex assembly	242	2	1.88E-01
GO:0097529	myeloid leukocyte migration	242	2	1.88E-01
GO:0016055	Wnt signaling pathway	456	3	1.88E-01
GO:0061061	muscle structure development	688	4	1.89E-01
GO:0050918	positive chemotaxis	64	1	1.90E-01
GO:0051893	regulation of focal adhesion assembly	64	1	1.90E-01
GO:0090109	regulation of cell-substrate junction assembly	64	1	1.90E-01
GO:0098631	cell adhesion mediator activity	64	1	1.90E-01
GO:0198738	cell-cell signaling by wnt	458	3	1.90E-01
GO:0045444	fat cell differentiation	244	2	1.91E-01
GO:0052547	regulation of peptidase activity	459	3	1.91E-01
GO:0006940	regulation of smooth muscle contraction	65	1	1.93E-01
GO:0033013	tetrapyrrole metabolic process	65	1	1.93E-01
GO:0048857	neural nucleus development	65	1	1.93E-01
GO:1905953	negative regulation of lipid localization	65	1	1.93E-01
GO:2000272	negative regulation of signaling receptor activity	65	1	1.93E-01
GO:0033043	regulation of organelle organization	1186	6	1.93E-01
GO:0044092	negative regulation of molecular function	1187	6	1.93E-01
GO:0009897	external side of plasma membrane	462	3	1.93E-01
GO:0006813	potassium ion transport	247	2	1.94E-01
GO:0016197	endosomal transport	247	2	1.94E-01
GO:0051259	protein complex oligomerization	247	2	1.94E-01
GO:0048814	regulation of dendrite morphogenesis	66	1	1.95E-01
GO:0060389	pathway-restricted SMAD protein phosphorylation	66	1	1.95E-01

GO:0071300	cellular response to retinoic acid	66	1	1.95E-01
GO:0005844	polysome	66	1	1.95E-01
GO:0042593	glucose homeostasis	248	2	1.96E-01
GO:0033500	carbohydrate homeostasis	249	2	1.97E-01
GO:0000228	nuclear chromosome	249	2	1.97E-01
GO:0002252	immune effector process	700	4	1.97E-01
GO:0015909	long-chain fatty acid transport	67	1	1.98E-01
GO:0031638	zymogen activation	67	1	1.98E-01
GO:0016485	protein processing	250	2	1.98E-01
GO:0000775	chromosome, centromeric region	250	2	1.98E-01
GO:0010951	negative regulation of endopeptidase activity	252	2	2.00E-01
GO:0048762	mesenchymal cell differentiation	252	2	2.00E-01
GO:0038034	signal transduction in absence of ligand	68	1	2.01E-01
GO:0046503	glycerolipid catabolic process	68	1	2.01E-01
GO:0046513	ceramide biosynthetic process	68	1	2.01E-01
GO:0097192	extrinsic apoptotic signaling pathway in absence of ligar	68	1	2.01E-01
GO:0031594	neuromuscular junction	68	1	2.01E-01
GO:0097367	carbohydrate derivative binding	2255	10	2.01E-01
GO:0034612	response to tumor necrosis factor	254	2	2.03E-01
GO:0002260	lymphocyte homeostasis	69	1	2.03E-01
GO:0010611	regulation of cardiac muscle hypertrophy	69	1	2.03E-01
GO:0032835	glomerulus development	69	1	2.03E-01
GO:0035418	protein localization to synapse	69	1	2.03E-01
GO:0150116	regulation of cell-substrate junction organization	69	1	2.03E-01
GO:0034704	calcium channel complex	69	1	2.03E-01
GO:0030276	clathrin binding	69	1	2.03E-01
GO:0090257	regulation of muscle system process	256	2	2.05E-01
GO:0042221	response to chemical	4494	18	2.05E-01
GO:0032722	positive regulation of chemokine production	70	1	2.06E-01
GO:0031491	nucleosome binding	70	1	2.06E-01
GO:0098797	plasma membrane protein complex	713	4	2.06E-01
GO:0009986	cell surface	962	5	2.08E-01
GO:0002548	monocyte chemotaxis	71	1	2.08E-01
GO:0051057	positive regulation of small GTPase mediated signal trar	71	1	2.08E-01
GO:0005905	clathrin-coated pit	71	1	2.08E-01
GO:0043621	protein self-association	71	1	2.08E-01
GO:0060828	regulation of canonical Wnt signaling pathway	260	2	2.10E-01
GO:1901701	cellular response to oxygen-containing compound	1220	6	2.10E-01
GO:0014743	regulation of muscle hypertrophy	72	1	2.11E-01
GO:0033143	regulation of intracellular steroid hormone receptor sig	72	1	2.11E-01
GO:0035567	non-canonical Wnt signaling pathway	72	1	2.11E-01
GO:0045600	positive regulation of fat cell differentiation	72	1	2.11E-01
GO:1900076	regulation of cellular response to insulin stimulus	72	1	2.11E-01
GO:0002699	positive regulation of immune effector process	261	2	2.11E-01
GO:0010876	lipid localization	483	3	2.11E-01
GO:0022411	cellular component disassembly	483	3	2.11E-01
GO:0045785	positive regulation of cell adhesion	484	3	2.12E-01

GO:0005802	trans-Golgi network	262	2	2.12E-01
GO:0010466	negative regulation of peptidase activity	263	2	2.14E-01
GO:0050795	regulation of behavior	73	1	2.14E-01
GO:0050805	negative regulation of synaptic transmission	73	1	2.14E-01
GO:0061035	regulation of cartilage development	73	1	2.14E-01
GO:0071677	positive regulation of mononuclear cell migration	73	1	2.14E-01
GO:1903578	regulation of ATP metabolic process	73	1	2.14E-01
GO:0016529	sarcoplasmic reticulum	73	1	2.14E-01
GO:0010647	positive regulation of cell communication	1753	8	2.15E-01
GO:0001707	mesoderm formation	74	1	2.16E-01
GO:0001736	establishment of planar polarity	74	1	2.16E-01
GO:0007164	establishment of tissue polarity	74	1	2.16E-01
GO:2000628	regulation of miRNA metabolic process	74	1	2.16E-01
GO:0023056	positive regulation of signaling	1757	8	2.16E-01
GO:0030522	intracellular receptor signaling pathway	266	2	2.17E-01
GO:0051091	positive regulation of DNA-binding transcription factor a	266	2	2.17E-01
GO:0061564	axon development	490	3	2.17E-01
GO:0045661	regulation of myoblast differentiation	75	1	2.19E-01
GO:0050810	regulation of steroid biosynthetic process	75	1	2.19E-01
GO:0060090	molecular adaptor activity	492	3	2.19E-01
GO:0007010	cytoskeleton organization	1500	7	2.20E-01
GO:0005575	cellular_component	19869	67	2.21E-01
GO:0001937	negative regulation of endothelial cell proliferation	76	1	2.21E-01
GO:0006635	fatty acid beta-oxidation	76	1	2.21E-01
GO:0019226	transmission of nerve impulse	76	1	2.21E-01
GO:0021536	diencephalon development	76	1	2.21E-01
GO:0048332	mesoderm morphogenesis	76	1	2.21E-01
GO:0042571	immunoglobulin complex, circulating	76	1	2.21E-01
GO:0060170	ciliary membrane	76	1	2.21E-01
GO:1901681	sulfur compound binding	270	2	2.22E-01
GO:0006919	activation of cysteine-type endopeptidase activity invol	77	1	2.24E-01
GO:0032720	negative regulation of tumor necrosis factor production	77	1	2.24E-01
GO:0045824	negative regulation of innate immune response	77	1	2.24E-01
GO:0046470	phosphatidylcholine metabolic process	77	1	2.24E-01
GO:0015935	small ribosomal subunit	77	1	2.24E-01
GO:0031348	negative regulation of defense response	272	2	2.24E-01
GO:0090596	sensory organ morphogenesis	272	2	2.24E-01
GO:0043537	negative regulation of blood vessel endothelial cell migr	78	1	2.26E-01
GO:0051966	regulation of synaptic transmission, glutamatergic	78	1	2.26E-01
GO:0061045	negative regulation of wound healing	78	1	2.26E-01
GO:0070373	negative regulation of ERK1 and ERK2 cascade	78	1	2.26E-01
GO:0051247	positive regulation of protein metabolic process	1516	7	2.28E-01
GO:0002764	immune response-regulating signaling pathway	503	3	2.28E-01
GO:0007193	adenylate cyclase-inhibiting G protein-coupled receptor	79	1	2.29E-01
GO:1903556	negative regulation of tumor necrosis factor superfamily	79	1	2.29E-01
GO:0008076	voltage-gated potassium channel complex	79	1	2.29E-01
GO:0051348	negative regulation of transferase activity	276	2	2.29E-01

GO:0071824	protein-DNA complex subunit organization	277	2	2.30E-01
GO:0001895	retina homeostasis	80	1	2.32E-01
GO:0014068	positive regulation of phosphatidylinositol 3-kinase sign	80	1	2.32E-01
GO:2001259	positive regulation of cation channel activity	80	1	2.32E-01
GO:0005791	rough endoplasmic reticulum	80	1	2.32E-01
GO:0033116	endoplasmic reticulum-Golgi intermediate compartmen	80	1	2.32E-01
GO:0051049	regulation of transport	1791	8	2.32E-01
GO:0008283	cell population proliferation	2062	9	2.32E-01
GO:0042325	regulation of phosphorylation	1264	6	2.33E-01
GO:1901652	response to peptide	509	3	2.34E-01
GO:0097061	dendritic spine organization	81	1	2.34E-01
GO:0120193	tight junction organization	81	1	2.34E-01
GO:0033293	monocarboxylic acid binding	81	1	2.34E-01
GO:0051258	protein polymerization	281	2	2.35E-01
GO:0009593	detection of chemical stimulus	512	3	2.36E-01
GO:0010770	positive regulation of cell morphogenesis involved in di	82	1	2.37E-01
GO:0021766	hippocampus development	82	1	2.37E-01
GO:0051149	positive regulation of muscle cell differentiation	82	1	2.37E-01
GO:0060395	SMAD protein signal transduction	82	1	2.37E-01
GO:0006094	gluconeogenesis	83	1	2.39E-01
GO:0006096	glycolytic process	83	1	2.39E-01
GO:0031507	heterochromatin formation	83	1	2.39E-01
GO:0010817	regulation of hormone levels	517	3	2.41E-01
GO:0000902	cell morphogenesis	1018	5	2.41E-01
GO:0006757	ATP generation from ADP	84	1	2.42E-01
GO:0010921	regulation of phosphatase activity	84	1	2.42E-01
GO:0097194	execution phase of apoptosis	84	1	2.42E-01
GO:0055065	metal ion homeostasis	519	3	2.42E-01
GO:0007548	sex differentiation	288	2	2.44E-01
GO:0000281	mitotic cytokinesis	85	1	2.44E-01
GO:0008306	associative learning	85	1	2.44E-01
GO:0051453	regulation of intracellular pH	85	1	2.44E-01
GO:0007420	brain development	768	4	2.44E-01
GO:0032989	cellular component morphogenesis	768	4	2.44E-01
GO:0090092	regulation of transmembrane receptor protein serine/t	290	2	2.46E-01
GO:0006970	response to osmotic stress	86	1	2.47E-01
GO:0019319	hexose biosynthetic process	86	1	2.47E-01
GO:0032092	positive regulation of protein binding	86	1	2.47E-01
GO:0032456	endocytic recycling	86	1	2.47E-01
GO:0035591	signaling adaptor activity	86	1	2.47E-01
GO:0008015	blood circulation	524	3	2.47E-01
GO:0046879	hormone secretion	291	2	2.47E-01
GO:0010632	regulation of epithelial cell migration	292	2	2.49E-01
GO:0015850	organic hydroxy compound transport	292	2	2.49E-01
GO:0010660	regulation of muscle cell apoptotic process	87	1	2.49E-01
GO:0019915	lipid storage	87	1	2.49E-01
GO:0099175	regulation of postsynapse organization	87	1	2.49E-01

GO:0009967	positive regulation of signal transduction	1559	7	2.49E-01
GO:0005768	endosome	1031	5	2.49E-01
GO:0048562	embryonic organ morphogenesis	294	2	2.51E-01
GO:0033627	cell adhesion mediated by integrin	88	1	2.52E-01
GO:0015031	protein transport	1564	7	2.52E-01
GO:0044262	cellular carbohydrate metabolic process	296	2	2.53E-01
GO:0034702	ion channel complex	296	2	2.53E-01
GO:0007589	body fluid secretion	89	1	2.54E-01
GO:0048678	response to axon injury	89	1	2.54E-01
GO:0050829	defense response to Gram-negative bacterium	89	1	2.54E-01
GO:0005798	Golgi-associated vesicle	89	1	2.54E-01
GO:0016528	sarcoplasm	89	1	2.54E-01
GO:0030117	membrane coat	89	1	2.54E-01
GO:0034705	potassium channel complex	89	1	2.54E-01
GO:0048475	coated membrane	89	1	2.54E-01
GO:0007165	signal transduction	6120	23	2.56E-01
GO:0006029	proteoglycan metabolic process	90	1	2.56E-01
GO:0010586	miRNA metabolic process	90	1	2.56E-01
GO:0030641	regulation of cellular pH	90	1	2.56E-01
GO:0046364	monosaccharide biosynthetic process	90	1	2.56E-01
GO:0051262	protein tetramerization	90	1	2.56E-01
GO:0045334	clathrin-coated endocytic vesicle	90	1	2.56E-01
GO:0071013	catalytic step 2 spliceosome	90	1	2.56E-01
GO:0043933	protein-containing complex organization	1845	8	2.57E-01
GO:0055085	transmembrane transport	1575	7	2.57E-01
GO:0009617	response to bacterium	786	4	2.57E-01
GO:0009914	hormone transport	300	2	2.58E-01
GO:0001942	hair follicle development	91	1	2.59E-01
GO:0030901	midbrain development	91	1	2.59E-01
GO:0048525	negative regulation of viral process	91	1	2.59E-01
GO:0060021	roof of mouth development	91	1	2.59E-01
GO:0070828	heterochromatin organization	91	1	2.59E-01
GO:0106027	neuron projection organization	91	1	2.59E-01
GO:0035579	specific granule membrane	91	1	2.59E-01
GO:0051493	regulation of cytoskeleton organization	539	3	2.60E-01
GO:0048511	rhythmic process	302	2	2.61E-01
GO:0001656	metanephros development	92	1	2.61E-01
GO:0009791	post-embryonic development	92	1	2.61E-01
GO:0010657	muscle cell apoptotic process	92	1	2.61E-01
GO:0046031	ADP metabolic process	92	1	2.61E-01
GO:0060996	dendritic spine development	92	1	2.61E-01
GO:0050906	detection of stimulus involved in sensory perception	542	3	2.63E-01
GO:0042147	retrograde transport, endosome to Golgi	93	1	2.64E-01
GO:0045185	maintenance of protein location	93	1	2.64E-01
GO:0045814	negative regulation of gene expression, epigenetic	93	1	2.64E-01
GO:0050886	endocrine process	93	1	2.64E-01
GO:0051781	positive regulation of cell division	93	1	2.64E-01

GO:0015297	antiporter activity	93	1	2.64E-01
GO:0071375	cellular response to peptide hormone stimulus	306	2	2.66E-01
GO:0022404	molting cycle process	94	1	2.66E-01
GO:0022405	hair cycle process	94	1	2.66E-01
GO:0035304	regulation of protein dephosphorylation	94	1	2.66E-01
GO:0048704	embryonic skeletal system morphogenesis	94	1	2.66E-01
GO:0051492	regulation of stress fiber assembly	94	1	2.66E-01
GO:1900542	regulation of purine nucleotide metabolic process	94	1	2.66E-01
GO:1903035	negative regulation of response to wounding	94	1	2.66E-01
GO:0010720	positive regulation of cell development	307	2	2.67E-01
GO:0032465	regulation of cytokinesis	95	1	2.69E-01
GO:0098773	skin epidermis development	95	1	2.69E-01
GO:0030134	COPII-coated ER to Golgi transport vesicle	95	1	2.69E-01
GO:0098796	membrane protein complex	1328	6	2.69E-01
GO:0060070	canonical Wnt signaling pathway	310	2	2.71E-01
GO:0030135	coated vesicle	310	2	2.71E-01
GO:0001738	morphogenesis of a polarized epithelium	96	1	2.71E-01
GO:0032091	negative regulation of protein binding	96	1	2.71E-01
GO:0045807	positive regulation of endocytosis	96	1	2.71E-01
GO:0061097	regulation of protein tyrosine kinase activity	96	1	2.71E-01
GO:0002429	immune response-activating cell surface receptor signal	312	2	2.73E-01
GO:0002757	immune response-activating signal transduction	312	2	2.73E-01
GO:0010596	negative regulation of endothelial cell migration	97	1	2.73E-01
GO:0043502	regulation of muscle adaptation	97	1	2.73E-01
GO:2001251	negative regulation of chromosome organization	97	1	2.73E-01
GO:0001750	photoreceptor outer segment	97	1	2.73E-01
GO:0055038	recycling endosome membrane	97	1	2.73E-01
GO:0030667	secretory granule membrane	313	2	2.74E-01
GO:0021510	spinal cord development	98	1	2.76E-01
GO:0032642	regulation of chemokine production	98	1	2.76E-01
GO:0048661	positive regulation of smooth muscle cell proliferation	98	1	2.76E-01
GO:0120162	positive regulation of cold-induced thermogenesis	98	1	2.76E-01
GO:1902414	protein localization to cell junction	98	1	2.76E-01
GO:0070372	regulation of ERK1 and ERK2 cascade	315	2	2.77E-01
GO:0019898	extrinsic component of membrane	316	2	2.78E-01
GO:0003300	cardiac muscle hypertrophy	99	1	2.78E-01
GO:0006140	regulation of nucleotide metabolic process	99	1	2.78E-01
GO:0006885	regulation of pH	99	1	2.78E-01
GO:0032602	chemokine production	99	1	2.78E-01
GO:0032755	positive regulation of interleukin-6 production	99	1	2.78E-01
GO:1901379	regulation of potassium ion transmembrane transport	99	1	2.78E-01
GO:2001237	negative regulation of extrinsic apoptotic signaling path	99	1	2.78E-01
GO:0048519	negative regulation of biological process	5903	22	2.79E-01
GO:0001776	leukocyte homeostasis	100	1	2.81E-01
GO:0006165	nucleoside diphosphate phosphorylation	100	1	2.81E-01
GO:0010769	regulation of cell morphogenesis involved in differentia	100	1	2.81E-01
GO:0019058	viral life cycle	319	2	2.81E-01

GO:0060326	cell chemotaxis	319	2	2.81E-01
GO:0098802	plasma membrane signaling receptor complex	319	2	2.81E-01
GO:0048667	cell morphogenesis involved in neuron differentiation	564	3	2.82E-01
GO:0009062	fatty acid catabolic process	101	1	2.83E-01
GO:0015908	fatty acid transport	101	1	2.83E-01
GO:1902476	chloride transmembrane transport	101	1	2.83E-01
GO:0014897	striated muscle hypertrophy	102	1	2.85E-01
GO:0019218	regulation of steroid metabolic process	102	1	2.85E-01
GO:0035249	synaptic transmission, glutamatergic	102	1	2.85E-01
GO:0046939	nucleotide phosphorylation	102	1	2.85E-01
GO:0001817	regulation of cytokine production	826	4	2.87E-01
GO:0019233	sensory perception of pain	103	1	2.88E-01
GO:0042472	inner ear morphogenesis	103	1	2.88E-01
GO:0043255	regulation of carbohydrate biosynthetic process	103	1	2.88E-01
GO:0048477	oogenesis	103	1	2.88E-01
GO:0050773	regulation of dendrite development	103	1	2.88E-01
GO:0000041	transition metal ion transport	104	1	2.90E-01
GO:0009135	purine nucleoside diphosphate metabolic process	104	1	2.90E-01
GO:0009179	purine ribonucleoside diphosphate metabolic process	104	1	2.90E-01
GO:0014896	muscle hypertrophy	104	1	2.90E-01
GO:0015914	phospholipid transport	104	1	2.90E-01
GO:0060291	long-term synaptic potentiation	104	1	2.90E-01
GO:0110020	regulation of actomyosin structure organization	104	1	2.90E-01
GO:0071868	cellular response to monoamine stimulus	105	1	2.92E-01
GO:0071870	cellular response to catecholamine stimulus	105	1	2.92E-01
GO:0022626	cytosolic ribosome	105	1	2.92E-01
GO:0071496	cellular response to external stimulus	328	2	2.92E-01
GO:0030593	neutrophil chemotaxis	106	1	2.95E-01
GO:0042102	positive regulation of T cell proliferation	106	1	2.95E-01
GO:0001816	cytokine production	839	4	2.96E-01
GO:0006672	ceramide metabolic process	107	1	2.97E-01
GO:0010906	regulation of glucose metabolic process	107	1	2.97E-01
GO:0015108	chloride transmembrane transporter activity	107	1	2.97E-01
GO:0007399	nervous system development	2495	10	2.99E-01
GO:0006090	pyruvate metabolic process	108	1	2.99E-01
GO:0032006	regulation of TOR signaling	108	1	2.99E-01
GO:0032231	regulation of actin filament bundle assembly	108	1	2.99E-01
GO:0032526	response to retinoic acid	108	1	2.99E-01
GO:0051341	regulation of oxidoreductase activity	108	1	2.99E-01
GO:0071867	response to monoamine	108	1	2.99E-01
GO:0071869	response to catecholamine	108	1	2.99E-01
GO:1902106	negative regulation of leukocyte differentiation	108	1	2.99E-01
GO:0030175	filopodium	108	1	2.99E-01
GO:0034451	centriolar satellite	108	1	2.99E-01
GO:0004620	phospholipase activity	108	1	2.99E-01
GO:0030038	contractile actin filament bundle assembly	109	1	3.02E-01
GO:0033209	tumor necrosis factor-mediated signaling pathway	109	1	3.02E-01

GO:0043149	stress fiber assembly	109	1	3.02E-01
GO:0001221	transcription coregulator binding	109	1	3.02E-01
GO:1902936	phosphatidylinositol bisphosphate binding	109	1	3.02E-01
GO:0016042	lipid catabolic process	336	2	3.02E-01
GO:0030111	regulation of Wnt signaling pathway	336	2	3.02E-01
GO:0051094	positive regulation of developmental process	1387	6	3.03E-01
GO:0019395	fatty acid oxidation	110	1	3.04E-01
GO:0045833	negative regulation of lipid metabolic process	110	1	3.04E-01
GO:0001933	negative regulation of protein phosphorylation	338	2	3.05E-01
GO:0090287	regulation of cellular response to growth factor stimulu	338	2	3.05E-01
GO:0014066	regulation of phosphatidylinositol 3-kinase signaling	111	1	3.06E-01
GO:0030148	sphingolipid biosynthetic process	111	1	3.06E-01
GO:0030510	regulation of BMP signaling pathway	111	1	3.06E-01
GO:0001932	regulation of protein phosphorylation	1121	5	3.07E-01
GO:0070371	ERK1 and ERK2 cascade	340	2	3.07E-01
GO:0046982	protein heterodimerization activity	341	2	3.08E-01
GO:0007283	spermatogenesis	593	3	3.08E-01
GO:0045445	myoblast differentiation	112	1	3.09E-01
GO:0048259	regulation of receptor-mediated endocytosis	112	1	3.09E-01
GO:0006955	immune response	1955	8	3.10E-01
GO:0032870	cellular response to hormone stimulus	595	3	3.10E-01
GO:0006939	smooth muscle contraction	113	1	3.11E-01
GO:0010633	negative regulation of epithelial cell migration	113	1	3.11E-01
GO:0061640	cytoskeleton-dependent cytokinesis	113	1	3.11E-01
GO:0003682	chromatin binding	597	3	3.12E-01
GO:0070588	calcium ion transmembrane transport	345	2	3.13E-01
GO:0002526	acute inflammatory response	114	1	3.13E-01
GO:0009185	ribonucleoside diphosphate metabolic process	114	1	3.13E-01
GO:0030004	cellular monovalent inorganic cation homeostasis	114	1	3.13E-01
GO:0043266	regulation of potassium ion transport	114	1	3.13E-01
GO:0044344	cellular response to fibroblast growth factor stimulus	114	1	3.13E-01
GO:1903707	negative regulation of hemopoiesis	114	1	3.13E-01
GO:0001938	positive regulation of endothelial cell proliferation	115	1	3.15E-01
GO:0032414	positive regulation of ion transmembrane transporter a	115	1	3.15E-01
GO:0034502	protein localization to chromosome	115	1	3.15E-01
GO:0042303	molting cycle	115	1	3.15E-01
GO:0042633	hair cycle	115	1	3.15E-01
GO:1904892	regulation of receptor signaling pathway via STAT	115	1	3.15E-01
GO:0015934	large ribosomal subunit	115	1	3.15E-01
GO:0043679	axon terminus	115	1	3.15E-01
GO:0007517	muscle organ development	348	2	3.17E-01
GO:0023052	signaling	6622	24	3.18E-01
GO:0001824	blastocyst development	116	1	3.18E-01
GO:0002832	negative regulation of response to biotic stimulus	116	1	3.18E-01
GO:0030518	intracellular steroid hormone receptor signaling pathwa	116	1	3.18E-01
GO:0051702	biological process involved in interaction with symbiont	116	1	3.18E-01
GO:0072009	nephron epithelium development	116	1	3.18E-01

GO:0010033	response to organic substance	3109	12	3.18E-01
GO:0045765	regulation of angiogenesis	349	2	3.18E-01
GO:1901700	response to oxygen-containing compound	1691	7	3.18E-01
GO:0061629	RNA polymerase II-specific DNA-binding transcription fa	350	2	3.19E-01
GO:0140096	catalytic activity, acting on a protein	2257	9	3.20E-01
GO:0021987	cerebral cortex development	117	1	3.20E-01
GO:0001525	angiogenesis	607	3	3.21E-01
GO:0031346	positive regulation of cell projection organization	352	2	3.22E-01
GO:0045861	negative regulation of proteolysis	352	2	3.22E-01
GO:0071396	cellular response to lipid	608	3	3.22E-01
GO:0006821	chloride transport	118	1	3.22E-01
GO:0007088	regulation of mitotic nuclear division	118	1	3.22E-01
GO:0019751	polyol metabolic process	118	1	3.22E-01
GO:0071346	cellular response to interferon-gamma	118	1	3.22E-01
GO:0031490	chromatin DNA binding	118	1	3.22E-01
GO:0009628	response to abiotic stimulus	1148	5	3.24E-01
GO:0010927	cellular component assembly involved in morphogenesi	119	1	3.24E-01
GO:0005262	calcium channel activity	119	1	3.24E-01
GO:1901342	regulation of vasculature development	355	2	3.25E-01
GO:0048232	male gamete generation	613	3	3.26E-01
GO:0007613	memory	120	1	3.27E-01
GO:0042752	regulation of circadian rhythm	120	1	3.27E-01
GO:0071774	response to fibroblast growth factor	120	1	3.27E-01
GO:0090288	negative regulation of cellular response to growth facto	120	1	3.27E-01
GO:0016311	dephosphorylation	357	2	3.28E-01
GO:0090100	positive regulation of transmembrane receptor protein	121	1	3.29E-01
GO:2000278	regulation of DNA biosynthetic process	121	1	3.29E-01
GO:0032787	monocarboxylic acid metabolic process	616	3	3.29E-01
GO:0009306	protein secretion	359	2	3.30E-01
GO:0002761	regulation of myeloid leukocyte differentiation	122	1	3.31E-01
GO:0043500	muscle adaptation	122	1	3.31E-01
GO:0097733	photoreceptor cell cilium	122	1	3.31E-01
GO:0005179	hormone activity	122	1	3.31E-01
GO:0005267	potassium channel activity	122	1	3.31E-01
GO:0001655	urogenital system development	360	2	3.31E-01
GO:0003002	regionalization	360	2	3.31E-01
GO:0035592	establishment of protein localization to extracellular reg	360	2	3.31E-01
GO:0022612	gland morphogenesis	123	1	3.33E-01
GO:0030282	bone mineralization	123	1	3.33E-01
GO:0046887	positive regulation of hormone secretion	123	1	3.33E-01
GO:0071675	regulation of mononuclear cell migration	123	1	3.33E-01
GO:0042471	ear morphogenesis	124	1	3.35E-01
GO:0051209	release of sequestered calcium ion into cytosol	124	1	3.35E-01
GO:0060079	excitatory postsynaptic potential	124	1	3.35E-01
GO:0007154	cell communication	6682	24	3.36E-01
GO:0034220	ion transmembrane transport	1167	5	3.37E-01
GO:0001952	regulation of cell-matrix adhesion	125	1	3.38E-01

GO:0019730	antimicrobial humoral response	125	1	3.38E-01
GO:0043280	positive regulation of cysteine-type endopeptidase activ	125	1	3.38E-01
GO:0051283	negative regulation of sequestering of calcium ion	125	1	3.38E-01
GO:0051241	negative regulation of multicellular organismal process	1169	5	3.38E-01
GO:0048812	neuron projection morphogenesis	626	3	3.38E-01
GO:0010631	epithelial cell migration	366	2	3.38E-01
GO:0002449	lymphocyte mediated immunity	367	2	3.40E-01
GO:0032535	regulation of cellular component size	367	2	3.40E-01
GO:0072329	monocarboxylic acid catabolic process	126	1	3.40E-01
GO:0098661	inorganic anion transmembrane transport	126	1	3.40E-01
GO:0090132	epithelium migration	369	2	3.42E-01
GO:0051282	regulation of sequestering of calcium ion	127	1	3.42E-01
GO:0140534	endoplasmic reticulum protein-containing complex	127	1	3.42E-01
GO:0045471	response to ethanol	128	1	3.44E-01
GO:0001818	negative regulation of cytokine production	371	2	3.44E-01
GO:0071705	nitrogen compound transport	2025	8	3.45E-01
GO:0050714	positive regulation of protein secretion	129	1	3.46E-01
GO:0051224	negative regulation of protein transport	129	1	3.46E-01
GO:2000027	regulation of animal organ morphogenesis	129	1	3.46E-01
GO:0006338	chromatin remodeling	373	2	3.47E-01
GO:0051346	negative regulation of hydrolase activity	373	2	3.47E-01
GO:1901653	cellular response to peptide	373	2	3.47E-01
GO:0048518	positive regulation of biological process	6421	23	3.48E-01
GO:0031929	TOR signaling	130	1	3.49E-01
GO:0042542	response to hydrogen peroxide	130	1	3.49E-01
GO:0051028	mRNA transport	130	1	3.49E-01
GO:0071621	granulocyte chemotaxis	130	1	3.49E-01
GO:0099565	chemical synaptic transmission, postsynaptic	130	1	3.49E-01
GO:1990266	neutrophil migration	130	1	3.49E-01
GO:0030018	Z disc	130	1	3.49E-01
GO:0016298	lipase activity	130	1	3.49E-01
GO:0030001	metal ion transport	909	4	3.49E-01
GO:0051301	cell division	638	3	3.49E-01
GO:0002460	adaptive immune response based on somatic recombin	375	2	3.49E-01
GO:0051208	sequestering of calcium ion	131	1	3.51E-01
GO:0044306	neuron projection terminus	131	1	3.51E-01
GO:0120039	plasma membrane bounded cell projection morphogen	642	3	3.52E-01
GO:0009132	nucleoside diphosphate metabolic process	132	1	3.53E-01
GO:0051983	regulation of chromosome segregation	132	1	3.53E-01
GO:0030665	clathrin-coated vesicle membrane	132	1	3.53E-01
GO:0002697	regulation of immune effector process	379	2	3.54E-01
GO:1902495	transmembrane transporter complex	379	2	3.54E-01
GO:0030168	platelet activation	133	1	3.55E-01
GO:1904950	negative regulation of establishment of protein localizat	133	1	3.55E-01
GO:0005793	endoplasmic reticulum-Golgi intermediate compartmen	133	1	3.55E-01
GO:0048858	cell projection morphogenesis	647	3	3.57E-01
GO:0043467	regulation of generation of precursor metabolites and e	134	1	3.57E-01

GO:0097731	9+0 non-motile cilium	134	1	3.57E-01
GO:0030900	forebrain development	383	2	3.59E-01
GO:0035270	endocrine system development	135	1	3.59E-01
GO:0015085	calcium ion transmembrane transporter activity	135	1	3.59E-01
GO:0031224	intrinsic component of membrane	5564	20	3.59E-01
GO:0042127	regulation of cell population proliferation	1769	7	3.61E-01
GO:0007498	mesoderm development	136	1	3.61E-01
GO:0043401	steroid hormone mediated signaling pathway	136	1	3.61E-01
GO:0098791	Golgi apparatus subcompartment	387	2	3.63E-01
GO:0061041	regulation of wound healing	137	1	3.63E-01
GO:0043177	organic acid binding	137	1	3.63E-01
GO:0043009	chordate embryonic development	655	3	3.64E-01
GO:0098660	inorganic ion transmembrane transport	930	4	3.64E-01
GO:0098687	chromosomal region	388	2	3.65E-01
GO:0014070	response to organic cyclic compound	931	4	3.65E-01
GO:0030879	mammary gland development	138	1	3.66E-01
GO:0004713	protein tyrosine kinase activity	138	1	3.66E-01
GO:0006997	nucleus organization	139	1	3.68E-01
GO:0007266	Rho protein signal transduction	140	1	3.70E-01
GO:0034341	response to interferon-gamma	140	1	3.70E-01
GO:0070851	growth factor receptor binding	140	1	3.70E-01
GO:0099106	ion channel regulator activity	140	1	3.70E-01
GO:0006812	cation transport	1219	5	3.71E-01
GO:1904062	regulation of cation transmembrane transport	394	2	3.72E-01
GO:0001889	liver development	141	1	3.72E-01
GO:0006986	response to unfolded protein	141	1	3.72E-01
GO:0045667	regulation of osteoblast differentiation	141	1	3.72E-01
GO:0071900	regulation of protein serine/threonine kinase activity	395	2	3.73E-01
GO:0004857	enzyme inhibitor activity	395	2	3.73E-01
GO:0035107	appendage morphogenesis	142	1	3.74E-01
GO:0035108	limb morphogenesis	142	1	3.74E-01
GO:0051384	response to glucocorticoid	142	1	3.74E-01
GO:0032990	cell part morphogenesis	666	3	3.74E-01
GO:0045727	positive regulation of translation	143	1	3.76E-01
GO:0046165	alcohol biosynthetic process	143	1	3.76E-01
GO:2001056	positive regulation of cysteine-type endopeptidase activ	143	1	3.76E-01
GO:0031674	I band	143	1	3.76E-01
GO:0098655	cation transmembrane transport	948	4	3.78E-01
GO:0007224	smoothened signaling pathway	144	1	3.78E-01
GO:0061008	hepaticobiliary system development	144	1	3.78E-01
GO:0019955	cytokine binding	144	1	3.78E-01
GO:0006631	fatty acid metabolic process	400	2	3.79E-01
GO:0045859	regulation of protein kinase activity	672	3	3.79E-01
GO:0014065	phosphatidylinositol 3-kinase signaling	145	1	3.80E-01
GO:0051783	regulation of nuclear division	145	1	3.80E-01
GO:0060078	regulation of postsynaptic membrane potential	145	1	3.80E-01
GO:0016247	channel regulator activity	145	1	3.80E-01

GO:0046903	secretion	951	4	3.80E-01
GO:0006996	organelle organization	3556	13	3.82E-01
GO:0050671	positive regulation of lymphocyte proliferation	146	1	3.82E-01
GO:0072073	kidney epithelium development	146	1	3.82E-01
GO:0043226	organelle	14728	50	3.82E-01
GO:0009792	embryo development ending in birth or egg hatching	677	3	3.84E-01
GO:0035264	multicellular organism growth	147	1	3.84E-01
GO:0106106	cold-induced thermogenesis	147	1	3.84E-01
GO:0120161	regulation of cold-induced thermogenesis	147	1	3.84E-01
GO:1990351	transporter complex	405	2	3.85E-01
GO:0015748	organophosphate ester transport	148	1	3.86E-01
GO:0140375	immune receptor activity	148	1	3.86E-01
GO:0003006	developmental process involved in reproduction	959	4	3.86E-01
GO:0044087	regulation of cellular component biogenesis	959	4	3.86E-01
GO:0032946	positive regulation of mononuclear cell proliferation	149	1	3.88E-01
GO:0046467	membrane lipid biosynthetic process	149	1	3.88E-01
GO:0051053	negative regulation of DNA metabolic process	149	1	3.88E-01
GO:0019842	vitamin binding	149	1	3.88E-01
GO:0042692	muscle cell differentiation	409	2	3.89E-01
GO:0001890	placenta development	150	1	3.90E-01
GO:0007612	learning	150	1	3.90E-01
GO:0010821	regulation of mitochondrion organization	150	1	3.90E-01
GO:0043535	regulation of blood vessel endothelial cell migration	150	1	3.90E-01
GO:0098876	vesicle-mediated transport to the plasma membrane	150	1	3.90E-01
GO:0071456	cellular response to hypoxia	151	1	3.92E-01
GO:1903531	negative regulation of secretion by cell	151	1	3.92E-01
GO:0031175	neuron projection development	967	4	3.92E-01
GO:1903706	regulation of hemopoiesis	413	2	3.94E-01
GO:0072006	nephron development	152	1	3.94E-01
GO:0052689	carboxylic ester hydrolase activity	152	1	3.94E-01
GO:0050678	regulation of epithelial cell proliferation	414	2	3.95E-01
GO:0019904	protein domain specific binding	693	3	3.98E-01
GO:0043408	regulation of MAPK cascade	694	3	3.99E-01
GO:0016052	carbohydrate catabolic process	155	1	4.00E-01
GO:0070507	regulation of microtubule cytoskeleton organization	155	1	4.00E-01
GO:2001236	regulation of extrinsic apoptotic signaling pathway	155	1	4.00E-01
GO:0031902	late endosome membrane	155	1	4.00E-01
GO:0051240	positive regulation of multicellular organismal process	1553	6	4.01E-01
GO:0110165	cellular anatomical entity	19469	65	4.01E-01
GO:0045860	positive regulation of protein kinase activity	420	2	4.02E-01
GO:0015081	sodium ion transmembrane transporter activity	156	1	4.02E-01
GO:0031252	cell leading edge	421	2	4.03E-01
GO:0010976	positive regulation of neuron projection development	157	1	4.04E-01
GO:0001558	regulation of cell growth	422	2	4.04E-01
GO:0048514	blood vessel morphogenesis	701	3	4.05E-01
GO:0097530	granulocyte migration	158	1	4.06E-01
GO:0015079	potassium ion transmembrane transporter activity	158	1	4.06E-01

GO:0008284	positive regulation of cell population proliferation	986	4	4.07E-01
GO:0016310	phosphorylation	1853	7	4.07E-01
GO:0007292	female gamete generation	159	1	4.08E-01
GO:0036294	cellular response to decreased oxygen levels	159	1	4.08E-01
GO:0048592	eye morphogenesis	159	1	4.08E-01
GO:0015103	inorganic anion transmembrane transporter activity	159	1	4.08E-01
GO:0005819	spindle	426	2	4.09E-01
GO:0007338	single fertilization	160	1	4.10E-01
GO:0051017	actin filament bundle assembly	160	1	4.10E-01
GO:0051147	regulation of muscle cell differentiation	160	1	4.10E-01
GO:1990845	adaptive thermogenesis	160	1	4.10E-01
GO:0042581	specific granule	160	1	4.10E-01
GO:0002181	cytoplasmic translation	161	1	4.12E-01
GO:0046434	organophosphate catabolic process	161	1	4.12E-01
GO:0034329	cell junction assembly	430	2	4.14E-01
GO:0050792	regulation of viral process	162	1	4.14E-01
GO:0050808	synapse organization	432	2	4.16E-01
GO:0006665	sphingolipid metabolic process	163	1	4.16E-01
GO:0035966	response to topologically incorrect protein	163	1	4.16E-01
GO:0040029	epigenetic regulation of gene expression	163	1	4.16E-01
GO:0050657	nucleic acid transport	163	1	4.16E-01
GO:0050658	RNA transport	163	1	4.16E-01
GO:0006468	protein phosphorylation	1578	6	4.16E-01
GO:0031399	regulation of protein modification process	1579	6	4.17E-01
GO:0051100	negative regulation of binding	164	1	4.18E-01
GO:0055067	monovalent inorganic cation homeostasis	164	1	4.18E-01
GO:0061572	actin filament bundle organization	164	1	4.18E-01
GO:0000776	kinetochore	164	1	4.18E-01
GO:0006811	ion transport	1581	6	4.18E-01
GO:0071310	cellular response to organic substance	2464	9	4.20E-01
GO:0071702	organic substance transport	2466	9	4.21E-01
GO:0051236	establishment of RNA localization	166	1	4.22E-01
GO:0099173	postsynapse organization	166	1	4.22E-01
GO:0005875	microtubule associated complex	166	1	4.22E-01
GO:0019814	immunoglobulin complex	166	1	4.22E-01
GO:0007409	axonogenesis	438	2	4.23E-01
GO:0030307	positive regulation of cell growth	167	1	4.24E-01
GO:0030509	BMP signaling pathway	167	1	4.24E-01
GO:0070665	positive regulation of leukocyte proliferation	167	1	4.24E-01
GO:0003779	actin binding	439	2	4.24E-01
GO:0006816	calcium ion transport	440	2	4.25E-01
GO:0000904	cell morphogenesis involved in differentiation	724	3	4.26E-01
GO:0044255	cellular lipid metabolic process	1013	4	4.27E-01
GO:0007268	chemical synaptic transmission	727	3	4.28E-01
GO:0098916	anterograde trans-synaptic signaling	727	3	4.28E-01
GO:0034250	positive regulation of cellular amide metabolic process	170	1	4.29E-01
GO:0035637	multicellular organismal signaling	170	1	4.29E-01

GO:0016482	cytosolic transport	171	1	4.31E-01
GO:1904064	positive regulation of cation transmembrane transport	171	1	4.31E-01
GO:0008201	heparin binding	171	1	4.31E-01
GO:0030036	actin cytoskeleton organization	731	3	4.32E-01
GO:1903034	regulation of response to wounding	172	1	4.33E-01
GO:0099537	trans-synaptic signaling	734	3	4.34E-01
GO:0002683	negative regulation of immune system process	449	2	4.35E-01
GO:0048568	embryonic organ development	449	2	4.35E-01
GO:0051336	regulation of hydrolase activity	1025	4	4.36E-01
GO:0006937	regulation of muscle contraction	174	1	4.37E-01
GO:0010950	positive regulation of endopeptidase activity	174	1	4.37E-01
GO:0021543	pallium development	174	1	4.37E-01
GO:0055088	lipid homeostasis	174	1	4.37E-01
GO:0000779	condensed chromosome, centromeric region	174	1	4.37E-01
GO:0009968	negative regulation of signal transduction	1319	5	4.37E-01
GO:0051048	negative regulation of secretion	175	1	4.39E-01
GO:0071453	cellular response to oxygen levels	175	1	4.39E-01
GO:0010634	positive regulation of epithelial cell migration	176	1	4.41E-01
GO:0032635	interleukin-6 production	176	1	4.41E-01
GO:0032675	regulation of interleukin-6 production	176	1	4.41E-01
GO:0050680	negative regulation of epithelial cell proliferation	176	1	4.41E-01
GO:0003735	structural constituent of ribosome	176	1	4.41E-01
GO:0006796	phosphate-containing compound metabolic process	2806	10	4.41E-01
GO:0003012	muscle system process	455	2	4.42E-01
GO:0043534	blood vessel endothelial cell migration	177	1	4.42E-01
GO:0001659	temperature homeostasis	178	1	4.44E-01
GO:0006694	steroid biosynthetic process	178	1	4.44E-01
GO:0031503	protein-containing complex localization	178	1	4.44E-01
GO:0048015	phosphatidylinositol-mediated signaling	178	1	4.44E-01
GO:0048736	appendage development	178	1	4.44E-01
GO:0060173	limb development	178	1	4.44E-01
GO:0070887	cellular response to chemical stimulus	3116	11	4.47E-01
GO:1901981	phosphatidylinositol phosphate binding	180	1	4.48E-01
GO:0030162	regulation of proteolysis	750	3	4.48E-01
GO:0008022	protein C-terminus binding	181	1	4.50E-01
GO:0002443	leukocyte mediated immunity	463	2	4.51E-01
GO:0042129	regulation of T cell proliferation	182	1	4.52E-01
GO:0046890	regulation of lipid biosynthetic process	182	1	4.52E-01
GO:0048017	inositol lipid-mediated signaling	182	1	4.52E-01
GO:0097553	calcium ion transmembrane import into cytosol	182	1	4.52E-01
GO:0071466	cellular response to xenobiotic stimulus	183	1	4.53E-01
GO:0097696	receptor signaling pathway via STAT	183	1	4.53E-01
GO:0031901	early endosome membrane	183	1	4.53E-01
GO:0072686	mitotic spindle	183	1	4.53E-01
GO:0006793	phosphorus metabolic process	2832	10	4.54E-01
GO:0099536	synaptic signaling	757	3	4.54E-01
GO:0048523	negative regulation of cellular process	4934	17	4.55E-01

GO:0050867	positive regulation of cell activation	467	2	4.55E-01
GO:0032640	tumor necrosis factor production	184	1	4.55E-01
GO:0032680	regulation of tumor necrosis factor production	184	1	4.55E-01
GO:0035725	sodium ion transmembrane transport	184	1	4.55E-01
GO:0051716	cellular response to stimulus	7653	26	4.56E-01
GO:0023061	signal release	470	2	4.58E-01
GO:0015698	inorganic anion transport	186	1	4.59E-01
GO:0034767	positive regulation of ion transmembrane transport	186	1	4.59E-01
GO:0065003	protein-containing complex assembly	1650	6	4.59E-01
GO:0000910	cytokinesis	187	1	4.61E-01
GO:0007389	pattern specification process	472	2	4.61E-01
GO:0007276	gamete generation	765	3	4.61E-01
GO:0043170	macromolecule metabolic process	10395	35	4.62E-01
GO:0006006	glucose metabolic process	188	1	4.62E-01
GO:0044391	ribosomal subunit	188	1	4.62E-01
GO:0005543	phospholipid binding	474	2	4.63E-01
GO:0007286	spermatid development	189	1	4.64E-01
GO:0043123	positive regulation of I-kappaB kinase/NF-kappaB signal	189	1	4.64E-01
GO:0071706	tumor necrosis factor superfamily cytokine production	189	1	4.64E-01
GO:1903555	regulation of tumor necrosis factor superfamily cytokine	189	1	4.64E-01
GO:0140297	DNA-binding transcription factor binding	476	2	4.65E-01
GO:0043433	negative regulation of DNA-binding transcription factor	190	1	4.66E-01
GO:0050777	negative regulation of immune response	190	1	4.66E-01
GO:0051260	protein homooligomerization	190	1	4.66E-01
GO:0008514	organic anion transmembrane transporter activity	190	1	4.66E-01
GO:0050907	detection of chemical stimulus involved in sensory perc	477	2	4.66E-01
GO:0003714	transcription corepressor activity	191	1	4.68E-01
GO:0031099	regeneration	192	1	4.69E-01
GO:0050673	epithelial cell proliferation	481	2	4.70E-01
GO:0050794	regulation of cellular process	11335	38	4.71E-01
GO:0048839	inner ear development	193	1	4.71E-01
GO:0043229	intracellular organelle	13770	46	4.72E-01
GO:0009749	response to glucose	194	1	4.73E-01
GO:0010952	positive regulation of peptidase activity	194	1	4.73E-01
GO:0003341	cilium movement	195	1	4.75E-01
GO:0005681	spliceosomal complex	195	1	4.75E-01
GO:0043005	neuron projection	1378	5	4.75E-01
GO:0001819	positive regulation of cytokine production	486	2	4.76E-01
GO:0045664	regulation of neuron differentiation	196	1	4.76E-01
GO:0048515	spermatid differentiation	196	1	4.76E-01
GO:0007626	locomotory behavior	197	1	4.78E-01
GO:2001257	regulation of cation channel activity	197	1	4.78E-01
GO:0043549	regulation of kinase activity	786	3	4.79E-01
GO:0071897	DNA biosynthetic process	198	1	4.80E-01
GO:0030662	coated vesicle membrane	198	1	4.80E-01
GO:0055037	recycling endosome	198	1	4.80E-01
GO:0009746	response to hexose	199	1	4.82E-01

GO:0001667	ameboidal-type cell migration	492	2	4.82E-01
GO:0016049	cell growth	493	2	4.83E-01
GO:0000165	MAPK cascade	792	3	4.84E-01
GO:0009566	fertilization	201	1	4.85E-01
GO:0019221	cytokine-mediated signaling pathway	496	2	4.87E-01
GO:0033674	positive regulation of kinase activity	496	2	4.87E-01
GO:0006403	RNA localization	202	1	4.87E-01
GO:1903828	negative regulation of protein localization	203	1	4.88E-01
GO:0030027	lamellipodium	203	1	4.88E-01
GO:0030496	midbody	203	1	4.88E-01
GO:0098772	molecular function regulator activity	2003	7	4.89E-01
GO:0030003	cellular cation homeostasis	499	2	4.90E-01
GO:0000302	response to reactive oxygen species	204	1	4.90E-01
GO:0031032	actomyosin structure organization	204	1	4.90E-01
GO:1901888	regulation of cell junction assembly	204	1	4.90E-01
GO:0043281	regulation of cysteine-type endopeptidase activity invol	205	1	4.92E-01
GO:0051223	regulation of protein transport	501	2	4.92E-01
GO:0048585	negative regulation of response to stimulus	1706	6	4.92E-01
GO:0071674	mononuclear cell migration	206	1	4.94E-01
GO:0048167	regulation of synaptic plasticity	207	1	4.95E-01
GO:0005741	mitochondrial outer membrane	207	1	4.95E-01
GO:0006091	generation of precursor metabolites and energy	505	2	4.96E-01
GO:0045216	cell-cell junction organization	208	1	4.97E-01
GO:0007264	small GTPase mediated signal transduction	506	2	4.97E-01
GO:0006643	membrane lipid metabolic process	209	1	4.99E-01
GO:0050807	regulation of synapse organization	209	1	4.99E-01
GO:0071383	cellular response to steroid hormone stimulus	209	1	4.99E-01
GO:0048666	neuron development	1112	4	4.99E-01
GO:0006629	lipid metabolic process	1415	5	4.99E-01
GO:0090066	regulation of anatomical structure size	509	2	5.00E-01
GO:0030136	clathrin-coated vesicle	211	1	5.02E-01
GO:0016051	carbohydrate biosynthetic process	212	1	5.04E-01
GO:0042098	T cell proliferation	212	1	5.04E-01
GO:0010648	negative regulation of cell communication	1424	5	5.05E-01
GO:0032940	secretion by cell	817	3	5.05E-01
GO:0030017	sarcomere	214	1	5.07E-01
GO:0006873	cellular ion homeostasis	516	2	5.08E-01
GO:0060284	regulation of cell development	516	2	5.08E-01
GO:0050803	regulation of synapse structure or activity	215	1	5.08E-01
GO:0030029	actin filament-based process	821	3	5.08E-01
GO:0023057	negative regulation of signaling	1430	5	5.09E-01
GO:0031400	negative regulation of protein modification process	519	2	5.11E-01
GO:0046034	ATP metabolic process	217	1	5.12E-01
GO:0034765	regulation of ion transmembrane transport	520	2	5.12E-01
GO:0009952	anterior/posterior pattern specification	218	1	5.13E-01
GO:0043232	intracellular non-membrane-bounded organelle	5700	19	5.13E-01
GO:0016021	integral component of membrane	5397	18	5.14E-01

GO:0043228	non-membrane-bounded organelle	5701	19	5.14E-01
GO:0008289	lipid binding	831	3	5.17E-01
GO:0070201	regulation of establishment of protein localization	526	2	5.18E-01
GO:0043583	ear development	221	1	5.18E-01
GO:0016747	acyltransferase activity, transferring groups other than a	221	1	5.18E-01
GO:0051246	regulation of protein metabolic process	2668	9	5.19E-01
GO:0051248	negative regulation of protein metabolic process	1141	4	5.19E-01
GO:0030658	transport vesicle membrane	222	1	5.20E-01
GO:0048285	organelle fission	528	2	5.20E-01
GO:0042995	cell projection	2368	8	5.21E-01
GO:0015931	nucleobase-containing compound transport	223	1	5.21E-01
GO:0070374	positive regulation of ERK1 and ERK2 cascade	223	1	5.21E-01
GO:0098858	actin-based cell projection	223	1	5.21E-01
GO:0005829	cytosol	5418	18	5.21E-01
GO:0098662	inorganic cation transmembrane transport	837	3	5.22E-01
GO:0071345	cellular response to cytokine stimulus	838	3	5.22E-01
GO:0050679	positive regulation of epithelial cell proliferation	224	1	5.23E-01
GO:0097191	extrinsic apoptotic signaling pathway	224	1	5.23E-01
GO:0043065	positive regulation of apoptotic process	532	2	5.24E-01
GO:0033157	regulation of intracellular protein transport	225	1	5.24E-01
GO:0032880	regulation of protein localization	842	3	5.26E-01
GO:0046777	protein autophosphorylation	226	1	5.26E-01
GO:0051651	maintenance of location in cell	226	1	5.26E-01
GO:0010001	glial cell differentiation	227	1	5.28E-01
GO:0035295	tube development	1154	4	5.28E-01
GO:0007606	sensory perception of chemical stimulus	537	2	5.29E-01
GO:0019318	hexose metabolic process	229	1	5.31E-01
GO:0071902	positive regulation of protein serine/threonine kinase a	229	1	5.31E-01
GO:0016323	basolateral plasma membrane	229	1	5.31E-01
GO:0048522	positive regulation of cellular process	5749	19	5.31E-01
GO:0010594	regulation of endothelial cell migration	230	1	5.32E-01
GO:0009987	cellular process	17266	57	5.33E-01
GO:0031669	cellular response to nutrient levels	231	1	5.34E-01
GO:0050769	positive regulation of neurogenesis	231	1	5.34E-01
GO:0005622	intracellular anatomical structure	15464	51	5.35E-01
GO:2001234	negative regulation of apoptotic signaling pathway	233	1	5.37E-01
GO:0030016	myofibril	233	1	5.37E-01
GO:0043068	positive regulation of programmed cell death	547	2	5.39E-01
GO:2000116	regulation of cysteine-type endopeptidase activity	235	1	5.40E-01
GO:0031968	organelle outer membrane	235	1	5.40E-01
GO:0019867	outer membrane	237	1	5.43E-01
GO:0003730	mRNA 3'-UTR binding	238	1	5.45E-01
GO:1902532	negative regulation of intracellular signal transduction	554	2	5.46E-01
GO:0050670	regulation of lymphocyte proliferation	239	1	5.46E-01
GO:0022414	reproductive process	1491	5	5.46E-01
GO:0030054	cell junction	2112	7	5.47E-01
GO:0042445	hormone metabolic process	241	1	5.49E-01

GO:0030141	secretory granule	872	3	5.50E-01
GO:0009205	purine ribonucleoside triphosphate metabolic process	242	1	5.51E-01
GO:0043292	contractile fiber	242	1	5.51E-01
GO:0000003	reproduction	1500	5	5.52E-01
GO:0032944	regulation of mononuclear cell proliferation	243	1	5.52E-01
GO:0046395	carboxylic acid catabolic process	243	1	5.52E-01
GO:0010035	response to inorganic substance	561	2	5.53E-01
GO:0031090	organelle membrane	3667	12	5.54E-01
GO:0043085	positive regulation of catalytic activity	1191	4	5.54E-01
GO:1901617	organic hydroxy compound biosynthetic process	245	1	5.55E-01
GO:0015291	secondary active transmembrane transporter activity	246	1	5.57E-01
GO:0044297	cell body	565	2	5.57E-01
GO:0004672	protein kinase activity	565	2	5.57E-01
GO:0048609	multicellular organismal reproductive process	882	3	5.57E-01
GO:1903530	regulation of secretion by cell	566	2	5.58E-01
GO:0009144	purine nucleoside triphosphate metabolic process	247	1	5.58E-01
GO:0016054	organic acid catabolic process	247	1	5.58E-01
GO:0005996	monosaccharide metabolic process	248	1	5.59E-01
GO:0007188	adenylate cyclase-modulating G protein-coupled recept	248	1	5.59E-01
GO:0009199	ribonucleoside triphosphate metabolic process	249	1	5.61E-01
GO:0090575	RNA polymerase II transcription regulator complex	249	1	5.61E-01
GO:0140352	export from cell	890	3	5.64E-01
GO:0050870	positive regulation of T cell activation	251	1	5.64E-01
GO:0009966	regulation of signal transduction	3075	10	5.64E-01
GO:0007423	sensory organ development	573	2	5.64E-01
GO:0032886	regulation of microtubule-based process	252	1	5.65E-01
GO:0033044	regulation of chromosome organization	252	1	5.65E-01
GO:0097305	response to alcohol	253	1	5.67E-01
GO:0019953	sexual reproduction	896	3	5.68E-01
GO:0009925	basal plasma membrane	254	1	5.68E-01
GO:0006814	sodium ion transport	255	1	5.70E-01
GO:0021537	telencephalon development	255	1	5.70E-01
GO:0043122	regulation of I-kappaB kinase/NF-kappaB signaling	255	1	5.70E-01
GO:0044283	small molecule biosynthetic process	579	2	5.70E-01
GO:0050708	regulation of protein secretion	257	1	5.72E-01
GO:0003007	heart morphogenesis	258	1	5.74E-01
GO:0006470	protein dephosphorylation	261	1	5.78E-01
GO:0034976	response to endoplasmic reticulum stress	261	1	5.78E-01
GO:0045927	positive regulation of growth	261	1	5.78E-01
GO:0051347	positive regulation of transferase activity	588	2	5.79E-01
GO:0051345	positive regulation of hydrolase activity	589	2	5.80E-01
GO:0031668	cellular response to extracellular stimulus	262	1	5.80E-01
GO:0051338	regulation of transferase activity	911	3	5.80E-01
GO:0030030	cell projection organization	1548	5	5.81E-01
GO:0005975	carbohydrate metabolic process	593	2	5.83E-01
GO:0010256	endomembrane system organization	594	2	5.84E-01
GO:0098656	anion transmembrane transport	266	1	5.85E-01

GO:0002521	leukocyte differentiation	596	2	5.86E-01
GO:0009141	nucleoside triphosphate metabolic process	267	1	5.86E-01
GO:0008134	transcription factor binding	597	2	5.87E-01
GO:0043603	cellular amide metabolic process	1242	4	5.88E-01
GO:0032504	multicellular organism reproduction	922	3	5.88E-01
GO:0070663	regulation of leukocyte proliferation	269	1	5.89E-01
GO:0008017	microtubule binding	271	1	5.92E-01
GO:0019752	carboxylic acid metabolic process	930	3	5.94E-01
GO:0007611	learning or memory	273	1	5.95E-01
GO:0000793	condensed chromosome	273	1	5.95E-01
GO:0043604	amide biosynthetic process	932	3	5.95E-01
GO:0034097	response to cytokine	933	3	5.96E-01
GO:1903039	positive regulation of leukocyte cell-cell adhesion	275	1	5.97E-01
GO:0010646	regulation of cell communication	3464	11	5.98E-01
GO:0051276	chromosome organization	609	2	5.98E-01
GO:0035091	phosphatidylinositol binding	277	1	6.00E-01
GO:1903532	positive regulation of secretion by cell	278	1	6.01E-01
GO:0150034	distal axon	278	1	6.01E-01
GO:0022853	active ion transmembrane transporter activity	278	1	6.01E-01
GO:0010942	positive regulation of cell death	614	2	6.02E-01
GO:0051962	positive regulation of nervous system development	279	1	6.03E-01
GO:0023051	regulation of signaling	3477	11	6.03E-01
GO:0044093	positive regulation of molecular function	1587	5	6.03E-01
GO:0035239	tube morphogenesis	943	3	6.03E-01
GO:0032412	regulation of ion transmembrane transporter activity	281	1	6.05E-01
GO:0043542	endothelial cell migration	281	1	6.05E-01
GO:0000278	mitotic cell cycle	946	3	6.06E-01
GO:0045165	cell fate commitment	282	1	6.07E-01
GO:0030425	dendrite	621	2	6.09E-01
GO:0006874	cellular calcium ion homeostasis	284	1	6.09E-01
GO:0051046	regulation of secretion	623	2	6.10E-01
GO:0097447	dendritic tree	623	2	6.10E-01
GO:0099568	cytoplasmic region	285	1	6.11E-01
GO:0007186	G protein-coupled receptor signaling pathway	1278	4	6.11E-01
GO:0006325	chromatin organization	624	2	6.11E-01
GO:0040008	regulation of growth	624	2	6.11E-01
GO:0060341	regulation of cellular localization	955	3	6.12E-01
GO:0043436	oxoacid metabolic process	957	3	6.13E-01
GO:0007249	I-kappaB kinase/NF-kappaB signaling	288	1	6.14E-01
GO:0007610	behavior	629	2	6.16E-01
GO:0043270	positive regulation of ion transport	289	1	6.16E-01
GO:0001664	G protein-coupled receptor binding	289	1	6.16E-01
GO:0018193	peptidyl-amino acid modification	1287	4	6.16E-01
GO:0022898	regulation of transmembrane transporter activity	291	1	6.18E-01
GO:0006082	organic acid metabolic process	964	3	6.18E-01
GO:0006518	peptide metabolic process	966	3	6.20E-01
GO:0120025	plasma membrane bounded cell projection	2258	7	6.21E-01

GO:0051222	positive regulation of protein transport	294	1	6.22E-01
GO:0030424	axon	642	2	6.27E-01
GO:0008152	metabolic process	12433	40	6.28E-01
GO:0000785	chromatin	1306	4	6.28E-01
GO:0031625	ubiquitin protein ligase binding	299	1	6.28E-01
GO:0008509	anion transmembrane transporter activity	303	1	6.33E-01
GO:0051047	positive regulation of secretion	304	1	6.34E-01
GO:0051607	defense response to virus	304	1	6.34E-01
GO:0046651	lymphocyte proliferation	305	1	6.36E-01
GO:0051056	regulation of small GTPase mediated signal transductior	305	1	6.36E-01
GO:0140546	defense response to symbiont	305	1	6.36E-01
GO:0034504	protein localization to nucleus	306	1	6.37E-01
GO:0005794	Golgi apparatus	1652	5	6.40E-01
GO:1902494	catalytic complex	1653	5	6.40E-01
GO:0010604	positive regulation of macromolecule metabolic process	3570	11	6.40E-01
GO:0001822	kidney development	309	1	6.40E-01
GO:0055074	calcium ion homeostasis	309	1	6.40E-01
GO:0031344	regulation of cell projection organization	659	2	6.41E-01
GO:0042063	gliogenesis	310	1	6.42E-01
GO:1904951	positive regulation of establishment of protein localizat	310	1	6.42E-01
GO:0070161	anchoring junction	1330	4	6.42E-01
GO:0006650	glycerophospholipid metabolic process	311	1	6.43E-01
GO:0032943	mononuclear cell proliferation	312	1	6.44E-01
GO:0050890	cognition	314	1	6.46E-01
GO:0007281	germ cell development	316	1	6.49E-01
GO:0016236	macroautophagy	317	1	6.50E-01
GO:1902105	regulation of leukocyte differentiation	317	1	6.50E-01
GO:0072001	renal system development	318	1	6.51E-01
GO:0044389	ubiquitin-like protein ligase binding	318	1	6.51E-01
GO:0012501	programmed cell death	2000	6	6.52E-01
GO:0016773	phosphotransferase activity, alcohol group as acceptor	672	2	6.52E-01
GO:1902533	positive regulation of intracellular signal transduction	1013	3	6.52E-01
GO:0055082	cellular chemical homeostasis	675	2	6.54E-01
GO:0022409	positive regulation of cell-cell adhesion	322	1	6.56E-01
GO:0008202	steroid metabolic process	323	1	6.57E-01
GO:0072503	cellular divalent inorganic cation homeostasis	323	1	6.57E-01
GO:0001227	DNA-binding transcription repressor activity, RNA polym	323	1	6.57E-01
GO:0015849	organic acid transport	324	1	6.58E-01
GO:0031647	regulation of protein stability	325	1	6.59E-01
GO:0140014	mitotic nuclear division	325	1	6.59E-01
GO:0015630	microtubule cytoskeleton	1362	4	6.61E-01
GO:0001217	DNA-binding transcription repressor activity	327	1	6.61E-01
GO:0000377	RNA splicing, via transesterification reactions with bulge	329	1	6.64E-01
GO:0000398	mRNA splicing, via spliceosome	329	1	6.64E-01
GO:0009895	negative regulation of catabolic process	332	1	6.67E-01
GO:0000375	RNA splicing, via transesterification reactions	333	1	6.68E-01
GO:0045296	cadherin binding	333	1	6.68E-01

GO:0099503	secretory vesicle	1041	3	6.71E-01
GO:0022607	cellular component assembly	3011	9	6.71E-01
GO:0071704	organic substance metabolic process	11974	38	6.71E-01
GO:0032386	regulation of intracellular transport	336	1	6.71E-01
GO:0030182	neuron differentiation	1381	4	6.72E-01
GO:0043010	camera-type eye development	340	1	6.76E-01
GO:0051130	positive regulation of cellular component organization	1050	3	6.76E-01
GO:0022836	gated channel activity	341	1	6.77E-01
GO:0050790	regulation of catalytic activity	2380	7	6.77E-01
GO:0031253	cell projection membrane	344	1	6.80E-01
GO:0043168	anion binding	2388	7	6.81E-01
GO:0005261	cation channel activity	346	1	6.82E-01
GO:0070661	leukocyte proliferation	348	1	6.84E-01
GO:0006936	muscle contraction	351	1	6.88E-01
GO:0043269	regulation of ion transport	718	2	6.88E-01
GO:0007265	Ras protein signal transduction	353	1	6.90E-01
GO:0072507	divalent inorganic cation homeostasis	356	1	6.93E-01
GO:0050865	regulation of cell activation	727	2	6.94E-01
GO:0005929	cilium	728	2	6.95E-01
GO:0016301	kinase activity	728	2	6.95E-01
GO:0002237	response to molecule of bacterial origin	360	1	6.97E-01
GO:0060271	cilium assembly	362	1	6.99E-01
GO:0008610	lipid biosynthetic process	735	2	7.00E-01
GO:0010564	regulation of cell cycle process	735	2	7.00E-01
GO:0006066	alcohol metabolic process	367	1	7.04E-01
GO:0015318	inorganic molecular entity transmembrane transporter	743	2	7.06E-01
GO:0002250	adaptive immune response	744	2	7.06E-01
GO:0015711	organic anion transport	372	1	7.09E-01
GO:0045862	positive regulation of proteolysis	372	1	7.09E-01
GO:2001233	regulation of apoptotic signaling pathway	374	1	7.11E-01
GO:0048699	generation of neurons	1453	4	7.11E-01
GO:0016071	mRNA metabolic process	751	2	7.11E-01
GO:0015631	tubulin binding	375	1	7.12E-01
GO:0050863	regulation of T cell activation	376	1	7.13E-01
GO:0050767	regulation of neurogenesis	377	1	7.14E-01
GO:1903037	regulation of leukocyte cell-cell adhesion	377	1	7.14E-01
GO:0065009	regulation of molecular function	3131	9	7.17E-01
GO:0007059	chromosome segregation	382	1	7.18E-01
GO:0044282	small molecule catabolic process	385	1	7.21E-01
GO:0001654	eye development	388	1	7.24E-01
GO:0006412	translation	770	2	7.24E-01
GO:0006644	phospholipid metabolic process	389	1	7.25E-01
GO:0019725	cellular homeostasis	773	2	7.26E-01
GO:0044782	cilium organization	391	1	7.27E-01
GO:0008219	cell death	2166	6	7.27E-01
GO:0150063	visual system development	392	1	7.28E-01
GO:0031984	organelle subcompartment	1486	4	7.28E-01

GO:0001701	in utero embryonic development	393	1	7.28E-01
GO:0009100	glycoprotein metabolic process	395	1	7.30E-01
GO:0048880	sensory system development	398	1	7.33E-01
GO:0044281	small molecule metabolic process	1841	5	7.33E-01
GO:0046486	glycerolipid metabolic process	399	1	7.34E-01
GO:0051251	positive regulation of lymphocyte activation	400	1	7.35E-01
GO:1990904	ribonucleoprotein complex	1152	3	7.36E-01
GO:1903047	mitotic cell cycle process	789	2	7.37E-01
GO:0008285	negative regulation of cell population proliferation	790	2	7.37E-01
GO:0120036	plasma membrane bounded cell projection organizatior	1509	4	7.39E-01
GO:0031974	membrane-enclosed lumen	6090	18	7.42E-01
GO:0043233	organelle lumen	6090	18	7.42E-01
GO:0070013	intracellular organelle lumen	6090	18	7.42E-01
GO:0009615	response to virus	409	1	7.43E-01
GO:0043043	peptide biosynthetic process	801	2	7.44E-01
GO:0007159	leukocyte cell-cell adhesion	414	1	7.47E-01
GO:0005769	early endosome	414	1	7.47E-01
GO:0007018	microtubule-based movement	415	1	7.48E-01
GO:0022804	active transmembrane transporter activity	417	1	7.49E-01
GO:0005694	chromosome	1880	5	7.50E-01
GO:0016607	nuclear speck	419	1	7.51E-01
GO:0006875	cellular metal ion homeostasis	420	1	7.52E-01
GO:0060255	regulation of macromolecule metabolic process	6762	20	7.54E-01
GO:0043067	regulation of programmed cell death	1541	4	7.54E-01
GO:0010959	regulation of metal ion transport	423	1	7.54E-01
GO:0030133	transport vesicle	423	1	7.54E-01
GO:0009893	positive regulation of metabolic process	3897	11	7.56E-01
GO:0045934	negative regulation of nucleobase-containing compoun	1550	4	7.58E-01
GO:0010629	negative regulation of gene expression	1551	4	7.59E-01
GO:0010467	gene expression	7096	21	7.59E-01
GO:0022412	cellular process involved in reproduction in multicellula	430	1	7.60E-01
GO:0004674	protein serine/threonine kinase activity	430	1	7.60E-01
GO:0042391	regulation of membrane potential	431	1	7.61E-01
GO:0046873	metal ion transmembrane transporter activity	436	1	7.65E-01
GO:0044085	cellular component biogenesis	3273	9	7.67E-01
GO:0010975	regulation of neuron projection development	446	1	7.73E-01
GO:0005216	ion channel activity	446	1	7.73E-01
GO:0036477	somatodendritic compartment	849	2	7.73E-01
GO:0009150	purine ribonucleotide metabolic process	447	1	7.73E-01
GO:0006915	apoptotic process	1941	5	7.75E-01
GO:0002696	positive regulation of leukocyte activation	450	1	7.76E-01
GO:1903829	positive regulation of protein localization	451	1	7.76E-01
GO:0015075	ion transmembrane transporter activity	855	2	7.77E-01
GO:0036211	protein modification process	3645	10	7.80E-01
GO:0051960	regulation of nervous system development	456	1	7.80E-01
GO:0006886	intracellular protein transport	868	2	7.84E-01
GO:0004930	G protein-coupled receptor activity	868	2	7.84E-01

GO:0043227	membrane-bounded organelle	13883	43	7.86E-01
GO:0098590	plasma membrane region	1250	3	7.86E-01
GO:0009259	ribonucleotide metabolic process	466	1	7.87E-01
GO:0005874	microtubule	466	1	7.87E-01
GO:0016772	transferase activity, transferring phosphorus-containing	876	2	7.88E-01
GO:0008380	RNA splicing	469	1	7.90E-01
GO:0006807	nitrogen compound metabolic process	10310	31	7.90E-01
GO:1903131	mononuclear cell differentiation	473	1	7.92E-01
GO:0019693	ribose phosphate metabolic process	475	1	7.94E-01
GO:0006163	purine nucleotide metabolic process	476	1	7.95E-01
GO:0016787	hydrolase activity	2344	6	7.96E-01
GO:0051051	negative regulation of transport	479	1	7.97E-01
GO:0000280	nuclear division	481	1	7.98E-01
GO:0006417	regulation of translation	486	1	8.01E-01
GO:0015267	channel activity	494	1	8.07E-01
GO:0022803	passive transmembrane transporter activity	495	1	8.07E-01
GO:0022008	neurogenesis	1669	4	8.08E-01
GO:0043410	positive regulation of MAPK cascade	496	1	8.08E-01
GO:0019222	regulation of metabolic process	7297	21	8.08E-01
GO:0045892	negative regulation of DNA-templated transcription	1302	3	8.09E-01
GO:0044089	positive regulation of cellular component biogenesis	498	1	8.09E-01
GO:0033365	protein localization to organelle	917	2	8.09E-01
GO:1903507	negative regulation of nucleic acid-templated transcript	1304	3	8.10E-01
GO:1902679	negative regulation of RNA biosynthetic process	1306	3	8.10E-01
GO:0040007	growth	921	2	8.11E-01
GO:0010468	regulation of gene expression	5401	15	8.12E-01
GO:0007017	microtubule-based process	925	2	8.13E-01
GO:0005911	cell-cell junction	505	1	8.14E-01
GO:0006397	mRNA processing	507	1	8.15E-01
GO:0072521	purine-containing compound metabolic process	507	1	8.15E-01
GO:0006820	anion transport	508	1	8.15E-01
GO:0005667	transcription regulator complex	509	1	8.16E-01
GO:0003712	transcription coregulator activity	510	1	8.17E-01
GO:0010941	regulation of cell death	1698	4	8.19E-01
GO:0043069	negative regulation of programmed cell death	938	2	8.19E-01
GO:0007346	regulation of mitotic cell cycle	517	1	8.21E-01
GO:0098793	presynapse	519	1	8.22E-01
GO:0031325	positive regulation of cellular metabolic process	3133	8	8.26E-01
GO:0000122	negative regulation of transcription by RNA polymerase	956	2	8.28E-01
GO:0051052	regulation of DNA metabolic process	540	1	8.34E-01
GO:0007005	mitochondrion organization	542	1	8.35E-01
GO:0051173	positive regulation of nitrogen compound metabolic pro	3175	8	8.37E-01
GO:0042110	T cell activation	548	1	8.39E-01
GO:0036094	small molecule binding	2482	6	8.39E-01
GO:0034248	regulation of cellular amide metabolic process	551	1	8.40E-01
GO:0043412	macromolecule modification	3869	10	8.40E-01
GO:0051172	negative regulation of nitrogen compound metabolic pr	2490	6	8.41E-01

GO:1902531	regulation of intracellular signal transduction	1766	4	8.42E-01
GO:0006914	autophagy	554	1	8.42E-01
GO:0061919	process utilizing autophagic mechanism	554	1	8.42E-01
GO:1901615	organic hydroxy compound metabolic process	555	1	8.43E-01
GO:0120031	plasma membrane bounded cell projection assembly	564	1	8.47E-01
GO:0051249	regulation of lymphocyte activation	573	1	8.52E-01
GO:2000112	regulation of cellular macromolecule biosynthetic proce	574	1	8.52E-01
GO:0051253	negative regulation of RNA metabolic process	1418	3	8.53E-01
GO:0030031	cell projection assembly	575	1	8.53E-01
GO:1901566	organonitrogen compound biosynthetic process	1817	4	8.58E-01
GO:0019637	organophosphate metabolic process	1034	2	8.60E-01
GO:0007049	cell cycle	1823	4	8.60E-01
GO:0003729	mRNA binding	596	1	8.63E-01
GO:0060548	negative regulation of cell death	1043	2	8.63E-01
GO:0009117	nucleotide metabolic process	597	1	8.63E-01
GO:0097190	apoptotic signaling pathway	600	1	8.65E-01
GO:0006753	nucleoside phosphate metabolic process	605	1	8.67E-01
GO:0007507	heart development	607	1	8.68E-01
GO:0022890	inorganic cation transmembrane transporter activity	607	1	8.68E-01
GO:0016740	transferase activity	2242	5	8.72E-01
GO:0005813	centrosome	623	1	8.75E-01
GO:0010608	post-transcriptional regulation of gene expression	1080	2	8.76E-01
GO:0035195	miRNA-mediated gene silencing	626	1	8.76E-01
GO:0034660	ncRNA metabolic process	634	1	8.79E-01
GO:0035194	post-transcriptional gene silencing by RNA	634	1	8.79E-01
GO:0042981	regulation of apoptotic process	1506	3	8.80E-01
GO:0016441	post-transcriptional gene silencing	639	1	8.81E-01
GO:0000226	microtubule cytoskeleton organization	642	1	8.83E-01
GO:0120035	regulation of plasma membrane bounded cell projectio	642	1	8.83E-01
GO:1901135	carbohydrate derivative metabolic process	1111	2	8.86E-01
GO:0048589	developmental growth	652	1	8.87E-01
GO:0035556	intracellular signal transduction	2673	6	8.87E-01
GO:0022857	transmembrane transporter activity	1118	2	8.88E-01
GO:0008324	cation transmembrane transporter activity	656	1	8.88E-01
GO:0002694	regulation of leukocyte activation	670	1	8.93E-01
GO:0055086	nucleobase-containing small molecule metabolic proces	673	1	8.94E-01
GO:0051726	regulation of cell cycle	1145	2	8.96E-01
GO:0001775	cell activation	1153	2	8.98E-01
GO:0019941	modification-dependent protein catabolic process	690	1	9.00E-01
GO:0031047	gene silencing by RNA	690	1	9.00E-01
GO:0005789	endoplasmic reticulum membrane	1163	2	9.01E-01
GO:0046907	intracellular transport	1587	3	9.01E-01
GO:0098827	endoplasmic reticulum subcompartment	1166	2	9.02E-01
GO:0043632	modification-dependent macromolecule catabolic proce	702	1	9.04E-01
GO:0010558	negative regulation of macromolecule biosynthetic proc	1604	3	9.05E-01
GO:0042175	nuclear outer membrane-endoplasmic reticulum memb	1186	2	9.07E-01
GO:0048471	perinuclear region of cytoplasm	729	1	9.13E-01

GO:0000978	RNA polymerase II cis-regulatory region sequence-speci	1213	2	9.14E-01
GO:0016491	oxidoreductase activity	735	1	9.14E-01
GO:0009892	negative regulation of metabolic process	3554	8	9.16E-01
GO:0005215	transporter activity	1225	2	9.17E-01
GO:0031966	mitochondrial membrane	744	1	9.17E-01
GO:0034645	cellular macromolecule biosynthetic process	1231	2	9.18E-01
GO:0000987	cis-regulatory region sequence-specific DNA binding	1234	2	9.19E-01
GO:0031327	negative regulation of cellular biosynthetic process	1673	3	9.20E-01
GO:0016788	hydrolase activity, acting on ester bonds	755	1	9.20E-01
GO:0019899	enzyme binding	2086	4	9.21E-01
GO:0030234	enzyme regulator activity	1253	2	9.23E-01
GO:0022402	cell cycle process	1256	2	9.24E-01
GO:0009890	negative regulation of biosynthetic process	1702	3	9.26E-01
GO:1901575	organic substance catabolic process	2120	4	9.27E-01
GO:0044238	primary metabolic process	10825	30	9.28E-01
GO:0003677	DNA binding	2522	5	9.28E-01
GO:0051603	proteolysis involved in protein catabolic process	788	1	9.29E-01
GO:0044248	cellular catabolic process	2134	4	9.29E-01
GO:0005740	mitochondrial envelope	793	1	9.30E-01
GO:0140513	nuclear protein-containing complex	1295	2	9.32E-01
GO:0051171	regulation of nitrogen compound metabolic process	5792	14	9.36E-01
GO:0010605	negative regulation of macromolecule metabolic proces	3336	7	9.36E-01
GO:0008270	zinc ion binding	823	1	9.37E-01
GO:0009056	catabolic process	2579	5	9.37E-01
GO:0016567	protein ubiquitination	824	1	9.37E-01
GO:0005815	microtubule organizing center	832	1	9.39E-01
GO:0045202	synapse	1341	2	9.40E-01
GO:1990234	transferase complex	839	1	9.40E-01
GO:0046649	lymphocyte activation	846	1	9.41E-01
GO:0016604	nuclear body	849	1	9.42E-01
GO:1901565	organonitrogen compound catabolic process	1371	2	9.45E-01
GO:0044271	cellular nitrogen compound biosynthetic process	4865	11	9.46E-01
GO:0006355	regulation of DNA-templated transcription	3443	7	9.49E-01
GO:1903506	regulation of nucleic acid-templated transcription	3445	7	9.49E-01
GO:2001141	regulation of RNA biosynthetic process	3454	7	9.50E-01
GO:0032446	protein modification by small protein conjugation	898	1	9.51E-01
GO:0080090	regulation of primary metabolic process	5954	14	9.51E-01
GO:0000977	RNA polymerase II transcription regulatory region seque	1431	2	9.54E-01
GO:0043066	negative regulation of apoptotic process	914	1	9.54E-01
GO:0070925	organelle assembly	935	1	9.57E-01
GO:0031324	negative regulation of cellular metabolic process	2345	4	9.57E-01
GO:0010557	positive regulation of macromolecule biosynthetic proc	1918	3	9.57E-01
GO:0005524	ATP binding	1472	2	9.59E-01
GO:0043231	intracellular membrane-bounded organelle	12663	35	9.59E-01
GO:0006351	DNA-templated transcription	3557	7	9.60E-01
GO:0097659	nucleic acid-templated transcription	3559	7	9.60E-01
GO:0032774	RNA biosynthetic process	3576	7	9.61E-01

GO:0003723	RNA binding	1971	3	9.63E-01
GO:0033554	cellular response to stress	1979	3	9.64E-01
GO:0009894	regulation of catabolic process	997	1	9.65E-01
GO:0000976	transcription cis-regulatory region binding	1529	2	9.65E-01
GO:0010556	regulation of macromolecule biosynthetic process	4002	8	9.65E-01
GO:0001067	transcription regulatory region nucleic acid binding	1531	2	9.65E-01
GO:0032559	adenyl ribonucleotide binding	1536	2	9.66E-01
GO:0045321	leukocyte activation	1009	1	9.67E-01
GO:0030554	adenyl nucleotide binding	1548	2	9.67E-01
GO:0030163	protein catabolic process	1014	1	9.67E-01
GO:0003824	catalytic activity	5488	12	9.68E-01
GO:0031328	positive regulation of cellular biosynthetic process	2032	3	9.69E-01
GO:0044265	cellular macromolecule catabolic process	1029	1	9.69E-01
GO:0070647	protein modification by small protein conjugation or rer	1039	1	9.70E-01
GO:0019219	regulation of nucleobase-containing compound metabo	4076	8	9.70E-01
GO:1990837	sequence-specific double-stranded DNA binding	1587	2	9.71E-01
GO:0018130	heterocycle biosynthetic process	4086	8	9.71E-01
GO:0006259	DNA metabolic process	1054	1	9.71E-01
GO:0019438	aromatic compound biosynthetic process	4100	8	9.72E-01
GO:0009891	positive regulation of biosynthetic process	2074	3	9.72E-01
GO:0051252	regulation of RNA metabolic process	3754	7	9.74E-01
GO:0051649	establishment of localization in cell	2108	3	9.74E-01
GO:0005654	nucleoplasm	4155	8	9.75E-01
GO:0046914	transition metal ion binding	1097	1	9.75E-01
GO:0031326	regulation of cellular biosynthetic process	4175	8	9.76E-01
GO:0003690	double-stranded DNA binding	1686	2	9.78E-01
GO:0043565	sequence-specific DNA binding	1689	2	9.78E-01
GO:0045893	positive regulation of DNA-templated transcription	1692	2	9.79E-01
GO:1903508	positive regulation of nucleic acid-templated transcripti	1692	2	9.79E-01
GO:1902680	positive regulation of RNA biosynthetic process	1698	2	9.79E-01
GO:0009889	regulation of biosynthetic process	4239	8	9.79E-01
GO:1901362	organic cyclic compound biosynthetic process	4253	8	9.80E-01
GO:0031323	regulation of cellular metabolic process	5737	12	9.81E-01
GO:0045944	positive regulation of transcription by RNA polymerase	1237	1	9.85E-01
GO:0035639	purine ribonucleoside triphosphate binding	1813	2	9.85E-01
GO:0034654	nucleobase-containing compound biosynthetic process	4013	7	9.86E-01
GO:0031967	organelle envelope	1255	1	9.86E-01
GO:0031975	envelope	1255	1	9.86E-01
GO:0009059	macromolecule biosynthetic process	4806	9	9.86E-01
GO:0051254	positive regulation of RNA metabolic process	1843	2	9.87E-01
GO:0044260	cellular macromolecule metabolic process	3266	5	9.88E-01
GO:0032555	purine ribonucleotide binding	1883	2	9.88E-01
GO:0017076	purine nucleotide binding	1897	2	9.89E-01
GO:0032553	ribonucleotide binding	1900	2	9.89E-01
GO:0140110	transcription regulator activity	1939	2	9.90E-01
GO:0000981	DNA-binding transcription factor activity, RNA polymera	1382	1	9.91E-01
GO:0009057	macromolecule catabolic process	1385	1	9.91E-01

GO:0003700	DNA-binding transcription factor activity	1430	1	9.92E-01
GO:0044249	cellular biosynthetic process	5807	11	9.92E-01
GO:0045935	positive regulation of nucleobase-containing compound	2052	2	9.93E-01
GO:0006396	RNA processing	1460	1	9.93E-01
GO:1901576	organic substance biosynthetic process	5877	11	9.94E-01
GO:0000166	nucleotide binding	2140	2	9.95E-01
GO:1901265	nucleoside phosphate binding	2141	2	9.95E-01
GO:0009058	biosynthetic process	5962	11	9.95E-01
GO:0034641	cellular nitrogen compound metabolic process	7053	14	9.95E-01
GO:0031981	nuclear lumen	4964	8	9.96E-01
GO:0005739	mitochondrion	1660	1	9.97E-01
GO:0016070	RNA metabolic process	5053	8	9.97E-01
GO:0003676	nucleic acid binding	4309	6	9.98E-01
GO:0006357	regulation of transcription by RNA polymerase II	2581	2	9.99E-01
GO:0006366	transcription by RNA polymerase II	2677	2	9.99E-01
GO:0046483	heterocycle metabolic process	6336	10	9.99E-01
GO:0006725	cellular aromatic compound metabolic process	6392	10	9.99E-01
GO:0090304	nucleic acid metabolic process	5606	8	9.99E-01
GO:0006139	nucleobase-containing compound metabolic process	6165	9	1.00E+00
GO:1901360	organic cyclic compound metabolic process	6632	10	1.00E+00
GO:1901363	heterocyclic compound binding	6259	9	1.00E+00
GO:0097159	organic cyclic compound binding	6330	9	1.00E+00
GO:0044237	cellular metabolic process	10271	20	1.00E+00
GO:0005634	nucleus	8188	11	1.00E+00

FDR	OR	ProteinName
3.18E-17	16.52	ACAN, ADIPOQ, CLTCL1, COL1A1, COL1A2, COL2A1, COL6A1, COL6A3, COL7A1, EFEMP
2.77E-14	36.72	ACAN, ADIPOQ, COL1A1, COL1A2, COL2A1, COL6A1, COL6A3, COL7A1, EFEMP1, LAMA
5.05E-12	17.56	ACAN, ADIPOQ, COL1A1, COL1A2, COL2A1, COL6A1, COL6A3, COL7A1, EFEMP1, LAMA
4.45E-11	14.07	ACAN, ADIPOQ, COL1A1, COL1A2, COL2A1, COL6A1, COL6A3, COL7A1, EFEMP1, LAMA
4.59E-11	14.05	ACAN, ADIPOQ, COL1A1, COL1A2, COL2A1, COL6A1, COL6A3, COL7A1, EFEMP1, LAMA
5.38E-10	46.70	ADIPOQ, COL1A1, COL1A2, COL2A1, COL6A1, COL6A3, COL7A1, COLEC11, MARCO, ME
4.90E-08	5.29	ACAN, ACTG2, ADIPOQ, C1orf56, CDSN, CLTCL1, CMTM5, COL1A1, COL1A2, COL2A1, C
1.04E-07	5.30	ACTG2, ADIPOQ, CLTCL1, CMTM5, COL1A1, COL1A2, COL2A1, COL6A1, COL6A3, COL7
4.65E-06	4.43	ACAN, ACTG2, ADIPOQ, CDSN, CELSR2, CLTCL1, CMTM6, COL1A1, COL1A2, COL2A1, CC
1.04E-05	57.03	COL1A1, COL1A2, COL2A1, COL6A1, COL6A3, COL7A1
2.71E-05	5.63	ACAN, ADIPOQ, CDSN, CELSR2, COL1A1, COL6A1, COL6A3, COL7A1, GP9, ITGA2, LAMA
8.75E-05	184.40	COL1A1, COL1A2, COL2A1, COL6A1
1.31E-04	161.34	COLEC11, MASP1, MASP2, MBL2
1.31E-04	161.34	CDSN, COL1A1, COL1A2, ITGA2
4.75E-04	4.54	ACTG2, ADIPOQ, CDSN, CELSR2, COL1A1, COL1A2, COL2A1, COL6A1, COL7A1, EFEMP1
1.13E-03	6.77	CELSR2, COLEC11, EFEMP1, LTBP1, MASP1, MASP2, PCDHGB7, PKD2L1, SPTA1, SVEP1,
1.88E-03	71.67	COL1A1, COL1A2, COL2A1, COL7A1
2.03E-03	10.25	COL1A1, COL1A2, COL2A1, COL6A1, COL6A3, COL7A1, LTBP1, TNC, VCAN
2.23E-03	21.40	COL2A1, COL7A1, LAMA2, TGFBI, THBS4, TNC
2.87E-03	3.76	ACTG2, ADIPOQ, CDSN, CELSR2, CLTCL1, COL1A1, COL1A2, COL2A1, COL6A1, COL7A1,
5.71E-03	3.17	ACAN, ACTG2, ADIPOQ, CDSN, CELSR2, CLTCL1, CMTM5, COL1A1, COL1A2, COL2A1, CC
7.46E-03	3.12	ACAN, ACTG2, ADIPOQ, CDSN, CELSR2, CLTCL1, CMTM5, COL1A1, COL1A2, COL2A1, CC
1.84E-02	105.90	COL1A1, COL1A2, COL2A1
1.84E-02	105.90	COL1A1, COL1A2, COL2A1
2.55E-02	2.94	ACAN, ACTG2, ADIPOQ, CDSN, CELSR2, COL1A1, COL1A2, COL2A1, COL6A1, COL7A1, E
2.69E-02	Inf	COL1A1, COL1A2
2.69E-02	Inf	COL6A1, COL6A3
2.69E-02	Inf	COL6A1, COL6A3
2.69E-02	3.16	ACTG2, ADIPOQ, CDSN, CELSR2, COL1A1, COL1A2, COL2A1, COL6A3, EFEMP1, EPB42, (
6.42E-02	4.57	ACTG2, COL1A1, COL1A2, COL2A1, COL6A1, COL6A3, DCDC1, KRT24, KRT32, KRT85, KF
7.03E-02	4.53	ACTG2, COL1A1, COL1A2, COL2A1, COL6A1, COL6A3, DCDC1, KRT24, KRT32, KRT85, KF
8.85E-02	3.97	ACTG2, COL1A1, COL1A2, COL2A1, COL6A1, COL6A3, COL7A1, DCDC1, H3-3A, KRT24, K
1.12E-01	10.43	ADIPOQ, COL6A1, COL7A1, ITGA2, NR0B1, PHLDB2
1.49E-01	7.92	CDSN, COL1A1, COL1A2, ITGA2, KRT85, KRT86, TGM3
1.54E-01	3.27	ACTG2, CLTCL1, COL1A2, COL6A1, COL6A3, EFEMP1, GNB4, H3-3A, H4C1, KRT24, KRT3
1.85E-01	12.83	COL1A1, COL1A2, COL2A1, COL6A1, LTBP1
1.98E-01	6.41	COL1A1, COL1A2, COL2A1, COL6A1, H3-3A, PPARG, TNC, VCAN
2.18E-01	7.44	COL1A1, COL1A2, COL2A1, LAMA2, PHLDB2, TGFBI, TGM3
2.59E-01	2.68	ADIPOQ, CDSN, CELSR2, CMTM5, COL1A1, COL2A1, COL6A1, COL7A1, EFEMP1, EPB42,
2.75E-01	3.11	ACTG2, CLTCL1, COL1A2, COL6A1, COL6A3, EFEMP1, GNB4, H3-3A, H4C1, KRT24, KRT3
2.77E-01	3.11	ACTG2, CLTCL1, COL1A2, COL6A1, COL6A3, EFEMP1, GNB4, H3-3A, H4C1, KRT24, KRT3
2.77E-01	3.11	ACTG2, CLTCL1, COL1A2, COL6A1, COL6A3, EFEMP1, GNB4, H3-3A, H4C1, KRT24, KRT3
2.90E-01	2.66	ADIPOQ, CDSN, CELSR2, CMTM5, COL1A1, COL2A1, COL6A1, COL7A1, EFEMP1, EPB42,

3.18E-01	3.86	ADIPOQ, CDSN, CELSR2, COL2A1, ITGA2, KRT32, KRT85, KRT86, NR0B1, PHLDB2, PPAR(
3.70E-01	2.64	ACTG2, CLTCL1, CMTM6, COL1A1, COL1A2, COL6A1, COL6A3, COL7A1, EFEMP1, GNB4,
3.91E-01	8.23	COL1A1, COL6A1, H3-3A, PPARG, TNC, VCAN
3.96E-01	156.48	ADIPOQ, PPARG
4.21E-01	16.08	CDSN, KRT85, KRT86, TGM3
4.56E-01	6.56	CDSN, COL7A1, KRT32, KRT85, KRT86, SVEP1, TGM3
5.53E-01	125.18	COLEC11, MBL2
5.60E-01	28.85	COL2A1, ITGA2, TNC
6.53E-01	9.68	GNB4, SLC4A1, SPTA1, SPTB, TGM3
6.85E-01	4.32	ADIPOQ, CDSN, CELSR2, COL1A1, ITGA2, LAMA2, PHLDB2, SPTA1, TGFBI, TNC
7.11E-01	7.34	ACAN, COLEC11, DCDC1, MBL2, SIGLEC16, VCAN
7.71E-01	4.25	CDSN, COL1A1, COL1A2, COL2A1, KRT32, KRT85, KRT86, PHLDB2, SPTA1, SPTB
9.18E-01	12.98	ADIPOQ, COLEC11, ITGA2, MBL2
9.41E-01	89.41	COLEC11, MBL2
9.41E-01	89.41	SPTA1, SPTB
9.41E-01	89.41	SPTA1, SPTB
9.41E-01	89.41	EPB42, TGM3
9.54E-01	5.03	ACAN, COL1A1, COL1A2, COL2A1, EFEMP1, GP9, TGFBI, VCAN
1.00E+00	8.69	DPP6, HGFAC, MASP1, MASP2, RELN
1.00E+00	12.35	EPB42, SLC4A1, SPTA1, SPTB
1.00E+00	2.40	ACAN, ADIPOQ, CELSR2, COL1A1, COL1A2, COL2A1, COL6A1, COL7A1, EFEMP1, EPB42,
1.00E+00	8.51	DPP6, HGFAC, MASP1, MASP2, RELN
1.00E+00	78.23	SPTA1, SPTB
1.00E+00	4.31	ADIPOQ, CDSN, ITGA2, KRT32, KRT85, KRT86, NR0B1, PPARG, TGM3
1.00E+00	8.16	GNB4, SLC4A1, SPTA1, SPTB, TGM3
1.00E+00	20.68	COL6A1, COL7A1, NR0B1
1.00E+00	69.53	LAMA2, PHLDB2
1.00E+00	69.53	COLEC11, MBL2
1.00E+00	69.53	COLEC11, MBL2
1.00E+00	4.71	COL1A1, GP9, HGFAC, ITGA2, PHLDB2, PPARG, SLC4A1, TNC
1.00E+00	5.34	COL1A1, GP9, HGFAC, ITGA2, PHLDB2, PPARG, SLC4A1
1.00E+00	6.31	COL1A1, COL1A2, COL2A1, LAMA2, PHLDB2, TGFBI
1.00E+00	6.29	COL1A1, COL1A2, COL2A1, LAMA2, PHLDB2, TGFBI
1.00E+00	3.39	COL1A1, COL2A1, COL6A1, COL7A1, ITGA2, NR0B1, PHLDB2, PPARG, RELN, TGFBI, THB
1.00E+00	19.41	ITGA2, LAMA2, TNC
1.00E+00	56.88	ADIPOQ, PPARG
1.00E+00	56.88	CELSR2, RELN
1.00E+00	56.88	COLEC11, MBL2
1.00E+00	10.44	COL6A1, COL7A1, ITGA2, NR0B1
1.00E+00	2.24	ACTG2, ADIPOQ, CLTCL1, COL1A1, COL1A2, COL2A1, COL6A1, COL6A3, COL7A1, COLEC
1.00E+00	17.95	ITGA2, SSC5D, TGFBI
1.00E+00	4.41	ADIPOQ, COL2A1, COL6A1, COL7A1, EFEMP1, ITGA2, NR0B1, PHLDB2
1.00E+00	17.29	COL6A1, COL7A1, NR0B1
1.00E+00	10.03	COLEC11, MASP1, MASP2, MBL2
1.00E+00	52.14	LAMA2, TNC
1.00E+00	9.95	COL1A1, COL1A2, COL6A1, PKD2L1
1.00E+00	3.37	ADIPOQ, COL1A1, COL2A1, COL6A1, COL7A1, EFEMP1, ITGA2, LAMA2, NR0B1, PHLDB2

1.00E+00	15.59	COL1A1, COL1A2, COL2A1
1.00E+00	41.71	COLEC11, MBL2
1.00E+00	41.71	COL1A1, ITGA2
1.00E+00	41.71	EPB42, TGM3
1.00E+00	6.53	KRT24, KRT32, KRT85, KRT86, PHLDB2
1.00E+00	14.40	KRT32, KRT85, KRT86
1.00E+00	14.40	COL6A1, ITGA2, TGFBI
1.00E+00	8.55	ADIPOQ, COL1A1, ITGA2, PPARG
1.00E+00	3.38	ADIPOQ, COLEC11, ITGA2, MBL2, PHLDB2, PLA2G7, PPARG, SIGLEC16, THBS4, TNC
1.00E+00	3.97	ADIPOQ, COLEC11, ITGA2, MBL2, PLA2G7, PPARG, SIGLEC16, TNC
1.00E+00	36.80	SLC4A1, SPTB
1.00E+00	3.94	COLEC11, GNB4, ITGA2, MBL2, SLC4A1, SPTA1, SPTB, TGM3
1.00E+00	3.34	ADIPOQ, COL1A1, ITGA2, LAMA2, PHLDB2, PLA2G7, PPARG, RELN, THBS4, TNC
1.00E+00	2.41	ADIPOQ, CMTM5, COL1A1, COLEC11, ITGA2, LAMA2, MARCO, MASP1, MASP2, MBL2,
1.00E+00	13.38	COLEC11, ITGA2, MBL2
1.00E+00	2.12	CDSN, CELSR2, CLTCL1, CMTM6, COL6A1, COL6A3, COLEC11, DPP6, EPB42, GNB4, GP9
1.00E+00	2.63	ADIPOQ, COL1A1, COL1A2, COL2A1, COL6A1, COL6A3, COL7A1, HGFAC, LTBP1, PKD2L1
1.00E+00	6.02	COL1A1, COL2A1, EFEMP1, PPARG, TGFBI
1.00E+00	2.48	ADIPOQ, CELSR2, CLTCL1, CMTM6, COL1A1, DPP6, H4C1, ITGA2, LTBP1, NR0B1, PHLDE
1.00E+00	5.95	COL1A1, COL1A2, LTBP1, MXRA5, PPARG
1.00E+00	4.89	ADIPOQ, EPB42, GP9, H4C1, PPARG, SLC4A1
1.00E+00	2.46	ADIPOQ, CELSR2, CLTCL1, CMTM6, COL1A1, DPP6, H4C1, ITGA2, LTBP1, NR0B1, PHLDE
1.00E+00	3.23	ACTG2, CDSN, CELSR2, COL1A1, COL1A2, COL2A1, EFEMP1, ITGA2, LAMA2, TNC
1.00E+00	12.34	COL1A1, COL1A2, COL6A1
1.00E+00	31.27	COLEC11, MBL2
1.00E+00	31.27	ACAN, VCAN
1.00E+00	31.27	ADIPOQ, SIGLEC16
1.00E+00	7.72	CDSN, KRT85, KRT86, TGM3
1.00E+00	12.18	MASP1, MASP2, MBL2
1.00E+00	4.76	CMTM6, COL6A1, GNB4, PIGR, SNX14, UBA52
1.00E+00	4.76	CMTM6, COL6A1, GNB4, PIGR, SNX14, UBA52
1.00E+00	11.87	COL6A1, COL7A1, NR0B1
1.00E+00	11.87	EPB42, GP9, SLC4A1
1.00E+00	3.14	ADIPOQ, COL1A1, ITGA2, LAMA2, PHLDB2, PLA2G7, PPARG, RELN, THBS4, TNC
1.00E+00	28.43	ACAN, VCAN
1.00E+00	2.13	ACAN, ADIPOQ, CELSR2, COL1A1, COL1A2, COL2A1, EFEMP1, EPB42, GNB4, GP9, H3-3A
1.00E+00	3.60	ACAN, CMTM6, COL6A1, GNB4, PIGR, SNX14, UBA52, VCAN
1.00E+00	3.60	ACAN, CMTM6, COL6A1, GNB4, PIGR, SNX14, UBA52, VCAN
1.00E+00	11.04	KRT32, KRT85, KRT86
1.00E+00	11.04	COL1A1, COL1A2, COL6A1
1.00E+00	10.91	KRT32, KRT85, KRT86
1.00E+00	Inf	ITGA2
1.00E+00	Inf	SLC4A1
1.00E+00	Inf	TNC
1.00E+00	Inf	COL1A2
1.00E+00	Inf	COL1A1
1.00E+00	Inf	ADIPOQ

1.00E+00	Inf	RELN
1.00E+00	Inf	RELN
1.00E+00	Inf	SLC4A1
1.00E+00	Inf	SLC4A1
1.00E+00	Inf	PPARG
1.00E+00	Inf	PPARG
1.00E+00	Inf	RELN
1.00E+00	Inf	RELN
1.00E+00	Inf	CDSN
1.00E+00	Inf	CDSN
1.00E+00	Inf	ADIPOQ
1.00E+00	Inf	ADIPOQ
1.00E+00	Inf	ADIPOQ
1.00E+00	Inf	ADIPOQ
1.00E+00	Inf	COL2A1
1.00E+00	Inf	COL7A1
1.00E+00	Inf	COL7A1
1.00E+00	Inf	COL7A1
1.00E+00	Inf	RELN
1.00E+00	Inf	PIGR
1.00E+00	Inf	NR0B1
1.00E+00	Inf	COLEC11
1.00E+00	5.35	COLEC11, ITGA2, MBL2, PLA2G7, SIGLEC16
1.00E+00	5.33	ADIPOQ, CDSN, COL1A1, PHLDB2, TGFBI
1.00E+00	25.01	ADIPOQ, PPARG
1.00E+00	5.26	EPB42, PHLDB2, SLC4A1, SPTA1, SPTB
1.00E+00	3.01	ADIPOQ, COL1A1, ITGA2, LAMA2, PHLDB2, PLA2G7, PPARG, RELN, THBS4, TNC
1.00E+00	5.23	ADIPOQ, COLEC11, ITGA2, MARCO, MBL2
1.00E+00	2.71	ADIPOQ, COLEC11, H4C1, ITGA2, MASP1, MASP2, MBL2, PIGR, PLA2G7, PPARG, SPTA1
1.00E+00	24.05	ITGA2, SSC5D
1.00E+00	24.05	PKD2L1, PPARG
1.00E+00	4.30	CMTM6, COL6A1, GNB4, PIGR, SNX14, UBA52
1.00E+00	23.16	COL1A1, COL2A1
1.00E+00	23.16	COL1A1, COL2A1
1.00E+00	23.16	RELN, THBS4
1.00E+00	4.27	COLEC11, ITGA2, MBL2, PLA2G7, SIGLEC16, THBS4
1.00E+00	6.49	COL1A1, COL2A1, EFEMP1, TGFBI
1.00E+00	21.56	COLEC11, MBL2
1.00E+00	2.44	ADIPOQ, CDSN, COL1A1, COL1A2, COL2A1, COLEC11, GYPA, MASP1, MBL2, NR0B1, PK
1.00E+00	2.21	ADIPOQ, CELSR2, COL1A1, COL1A2, COL2A1, COLEC11, EFEMP1, ITGA2, LAMA2, LTBP1
1.00E+00	20.84	COLEC11, MBL2
1.00E+00	20.84	ADIPOQ, PPARG
1.00E+00	2.51	ACAN, COL6A3, COL7A1, COLEC11, DPP6, HGFAC, MASP1, MASP2, MBL2, PLA2G7, PPA
1.00E+00	20.17	COL1A1, COL2A1
1.00E+00	9.12	COL1A1, COL1A2, ITGA2
1.00E+00	9.12	EPB42, SPTA1, SPTB
1.00E+00	9.04	ADIPOQ, ITGA2, THBS4

1.00E+00	6.06	KRT24, KRT32, KRT85, KRT86
1.00E+00	2.18	ADIPOQ, CELSR2, CLTCL1, CMTM6, COL1A1, DPP6, H4C1, HNRNPA1L2, ITGA2, LTBP1, I
1.00E+00	3.53	ADIPOQ, CLTCL1, COLEC11, ITGA2, MARCO, MBL2, SSC5D
1.00E+00	5.92	COL1A1, COL2A1, EFEMP1, TGFBI
1.00E+00	3.19	ACAN, CMTM6, COL6A1, GNB4, PIGR, SNX14, UBA52, VCAN
1.00E+00	2.31	ADIPOQ, COL1A1, COLEC11, ITGA2, LAMA2, MARCO, MASP1, MASP2, MBL2, PLA2G7, I
1.00E+00	18.38	EPB42, TGM3
1.00E+00	18.38	LAMA2, PHLDB2
1.00E+00	18.38	PKD2L1, PPARG
1.00E+00	8.54	COL2A1, EFEMP1, TGFBI
1.00E+00	5.81	COL1A1, COL2A1, EFEMP1, TGFBI
1.00E+00	5.78	GP9, HGFAC, ITGA2, SLC4A1
1.00E+00	308.34	PKD2L1
1.00E+00	308.34	PIGR
1.00E+00	308.34	SVEP1
1.00E+00	308.34	CDSN
1.00E+00	308.34	ITGA2
1.00E+00	308.34	PLA2G7
1.00E+00	308.34	PPARG
1.00E+00	308.34	PLA2G7
1.00E+00	308.34	ADIPOQ
1.00E+00	308.34	ADIPOQ
1.00E+00	308.34	PLA2G7
1.00E+00	308.34	COL1A1
1.00E+00	308.34	PPARG
1.00E+00	308.34	ADIPOQ
1.00E+00	308.34	ADIPOQ
1.00E+00	308.34	MASP2
1.00E+00	308.34	PKD2L1
1.00E+00	17.86	ADIPOQ, CLTCL1
1.00E+00	17.86	KRT85, KRT86
1.00E+00	8.25	COL1A1, COL1A2, COL6A1
1.00E+00	5.65	GP9, HGFAC, ITGA2, SLC4A1
1.00E+00	17.36	ADIPOQ, PPARG
1.00E+00	17.36	EPB42, SLC4A1
1.00E+00	5.63	GP9, HGFAC, ITGA2, SLC4A1
1.00E+00	4.46	H3-3A, PHLDB2, PPARG, SPTA1, SPTB
1.00E+00	2.59	ADIPOQ, CMTM5, COL1A1, ITGA2, LAMA2, PHLDB2, PLA2G7, PPARG, RELN, THBS4, TN
1.00E+00	16.89	ADIPOQ, PPARG
1.00E+00	16.89	ADIPOQ, PPARG
1.00E+00	16.89	ADIPOQ, PPARG
1.00E+00	5.53	CDSN, KRT85, KRT86, TGM3
1.00E+00	16.44	ADIPOQ, PPARG
1.00E+00	16.02	SPTA1, SPTB
1.00E+00	15.24	PIGR, PKD2L1
1.00E+00	4.18	CELSR2, GP9, HGFAC, ITGA2, SLC4A1
1.00E+00	14.88	ADIPOQ, CLTCL1

1.00E+00	14.88	ADIPOQ, PPARG
1.00E+00	14.53	SPTA1, SPTB
1.00E+00	154.16	PIGR
1.00E+00	154.16	CDSN
1.00E+00	154.16	CELSR2
1.00E+00	154.16	PLA2G7
1.00E+00	154.16	TGM3
1.00E+00	154.16	RELN
1.00E+00	154.16	PPARG
1.00E+00	154.16	ADIPOQ
1.00E+00	154.16	ADIPOQ
1.00E+00	154.16	ADIPOQ
1.00E+00	154.16	ADIPOQ
1.00E+00	154.16	ADIPOQ
1.00E+00	154.16	ADIPOQ
1.00E+00	154.16	RELN
1.00E+00	154.16	COL7A1
1.00E+00	154.16	ITGA2
1.00E+00	154.16	TNC
1.00E+00	154.16	STEAP3
1.00E+00	154.16	EFEMP1
1.00E+00	154.16	STEAP3
1.00E+00	154.16	PPARG
1.00E+00	154.16	STEAP3
1.00E+00	14.20	ADIPOQ, SLC4A1
1.00E+00	14.20	COL1A2, PHLDB2
1.00E+00	7.12	COL6A1, COL6A3, LAMA2
1.00E+00	13.88	MARCO, SSC5D
1.00E+00	2.06	ADIPOQ, COLEC11, EPB42, GP9, H4C1, ITGA2, MARCO, MASP1, MASP2, MBL2, PIGR, P
1.00E+00	13.58	COL1A1, PHLDB2
1.00E+00	3.93	ADIPOQ, ITGA2, PLA2G7, PPARG, TNC
1.00E+00	13.01	COL1A1, ITGA2
1.00E+00	6.72	ITGA2, PLA2G7, THBS4
1.00E+00	4.77	COL1A1, COL1A2, LTBP1, PPARG
1.00E+00	12.75	ITGA2, PKD2L1
1.00E+00	2.37	ADIPOQ, CMTM5, COL2A1, EFEMP1, ITGA2, LAMA2, MARCO, MBL2, RELN, TGFBI, THB
1.00E+00	12.49	ADIPOQ, SLC4A1
1.00E+00	6.53	ITGA2, PLA2G7, THBS4
1.00E+00	1.85	ACAN, ACTG2, ADIPOQ, CDCA2, CELSR2, CLTCL1, CMTM6, COL1A1, COL1A2, COL2A1, C
1.00E+00	102.77	LTBP1
1.00E+00	102.77	ADIPOQ
1.00E+00	102.77	SVEP1
1.00E+00	102.77	THBS4
1.00E+00	102.77	PPARG
1.00E+00	102.77	ADIPOQ
1.00E+00	102.77	RELN
1.00E+00	102.77	RELN

1.00E+00	102.77	H3-3A
1.00E+00	102.77	PHLDB2
1.00E+00	102.77	ADIPOQ
1.00E+00	102.77	LTBP1
1.00E+00	102.77	RELN
1.00E+00	2.27	DPP6, GP9, GYPA, ITGA2, MARCO, PCDHGB7, PIGR, PKD2L1, SLC4A1, SPTA1, SPTB, TM
1.00E+00	12.25	ADIPOQ, PPARG
1.00E+00	12.01	SPTA1, SPTB
1.00E+00	2.73	COLEC11, ITGA2, MASP1, MASP2, MBL2, PLA2G7, SPTA1, THBS4
1.00E+00	2.32	ADIPOQ, CELSR2, COL1A1, ITGA2, LAMA2, PHLDB2, PLA2G7, PPARG, RELN, THBS4, TN
1.00E+00	2.23	ADIPOQ, COLEC11, ITGA2, MARCO, MASP1, MASP2, MBL2, PLA2G7, PPARG, SIGLEC16,
1.00E+00	6.19	ITGA2, TGFBI, THBS4
1.00E+00	11.35	PIGR, RELN
1.00E+00	11.35	ITGA2, PPARG
1.00E+00	11.35	LAMA2, PHLDB2
1.00E+00	11.15	COL1A1, COL2A1
1.00E+00	11.15	CMTM6, PIGR
1.00E+00	1.75	CDSN, CELSR2, CLTCL1, CMTM5, CMTM6, COL6A1, COL6A3, COL7A1, COLEC11, DPP6,
1.00E+00	3.15	CMTM5, ITGA2, LAMA2, PLA2G7, RELN, THBS4
1.00E+00	10.95	SPTA1, SPTB
1.00E+00	10.95	ADIPOQ, CLTCL1
1.00E+00	77.07	CELSR2
1.00E+00	77.07	H3-3A
1.00E+00	77.07	LAMA2
1.00E+00	77.07	ADIPOQ
1.00E+00	77.07	SLC4A1
1.00E+00	77.07	SSC5D
1.00E+00	77.07	ACTG2
1.00E+00	77.07	ADIPOQ
1.00E+00	77.07	COL1A1
1.00E+00	77.07	PPARG
1.00E+00	77.07	PHLDB2
1.00E+00	77.07	PHLDB2
1.00E+00	77.07	PPARG
1.00E+00	77.07	ADIPOQ
1.00E+00	77.07	ADIPOQ
1.00E+00	77.07	PHLDB2
1.00E+00	77.07	COL2A1
1.00E+00	77.07	PHLDB2
1.00E+00	77.07	PIGR
1.00E+00	77.07	PIGR
1.00E+00	77.07	PIGR
1.00E+00	77.07	TNC
1.00E+00	77.07	TNC
1.00E+00	77.07	GP9
1.00E+00	77.07	SPTLC3
1.00E+00	77.07	SPTLC3

1.00E+00	77.07	STEAP3
1.00E+00	77.07	COLEC11
1.00E+00	77.07	TNC
1.00E+00	77.07	ITGA2
1.00E+00	3.14	CMTM5, ITGA2, LAMA2, PLA2G7, RELN, THBS4
1.00E+00	10.76	ADIPOQ, PPARG
1.00E+00	5.84	PHLDB2, SPTA1, SPTB
1.00E+00	2.62	ADIPOQ, COL1A1, COL1A2, EFEMP1, LTBP1, PIGR, PPARG, SVEP1
1.00E+00	3.09	ACTG2, CELSR2, ITGA2, PHLDB2, TGM3, TNC
1.00E+00	4.24	EPB42, H3-3A, PPARG, RELN
1.00E+00	5.70	PHLDB2, SPTA1, SPTB
1.00E+00	5.63	ADIPOQ, PPARG, SIGLEC16
1.00E+00	10.07	COLEC11, MBL2
1.00E+00	10.07	CDCA2, ITGA2
1.00E+00	61.66	ITGA2
1.00E+00	61.66	PPARG
1.00E+00	61.66	STEAP3
1.00E+00	61.66	RELN
1.00E+00	61.66	ITGA2
1.00E+00	61.66	ADIPOQ
1.00E+00	61.66	CELSR2
1.00E+00	61.66	ADIPOQ
1.00E+00	61.66	H3-3A
1.00E+00	61.66	ADIPOQ
1.00E+00	61.66	H3-3A
1.00E+00	61.66	H3-3A
1.00E+00	61.66	VCAN
1.00E+00	61.66	CLTCL1
1.00E+00	61.66	CLTCL1
1.00E+00	61.66	ITGA2
1.00E+00	61.66	COL2A1
1.00E+00	61.66	PPARG
1.00E+00	61.66	PPARG
1.00E+00	5.53	HGFAC, MASP1, MASP2
1.00E+00	5.50	ADIPOQ, ITGA2, PPARG
1.00E+00	9.75	COL1A1, PHLDB2
1.00E+00	9.75	GP9, H4C1
1.00E+00	9.75	SPTA1, SPTB
1.00E+00	5.43	ITGA2, PPARG, SPTA1
1.00E+00	1.87	ADIPOQ, CELSR2, CLTCL1, CMTM6, COL1A1, DPP6, GP9, H4C1, ITGA2, LTBP1, NR0B1, P
1.00E+00	2.01	ACTG2, CLTCL1, DCDC1, EPB42, KRT24, KRT32, KRT85, KRT86, NR0B1, PHLDB2, PKD2L1
1.00E+00	4.02	COLEC11, MASP1, MASP2, MBL2
1.00E+00	9.46	PIGR, PKD2L1
1.00E+00	5.37	ADIPOQ, ITGA2, PPARG
1.00E+00	1.80	ADIPOQ, COL1A1, COL2A1, COLEC11, ITGA2, LAMA2, LTBP1, MARCO, MASP1, MASP2,
1.00E+00	5.34	COL1A1, COL2A1, ITGA2
1.00E+00	9.18	ADIPOQ, ITGA2

1.00E+00	51.38	COL1A1
1.00E+00	51.38	PPARG
1.00E+00	51.38	ADIPOQ
1.00E+00	51.38	LAMA2
1.00E+00	51.38	ADIPOQ
1.00E+00	51.38	ADIPOQ
1.00E+00	51.38	COL1A1
1.00E+00	51.38	COL1A1
1.00E+00	51.38	EFEMP1
1.00E+00	51.38	ITGA2
1.00E+00	51.38	CDSN
1.00E+00	51.38	PPARG
1.00E+00	51.38	PPARG
1.00E+00	51.38	TNC
1.00E+00	51.38	PPARG
1.00E+00	51.38	PPARG
1.00E+00	51.38	ADIPOQ
1.00E+00	51.38	COL2A1
1.00E+00	51.38	ADIPOQ
1.00E+00	51.38	PIGR
1.00E+00	51.38	CLTCL1
1.00E+00	51.38	PPARG
1.00E+00	51.38	PPARG
1.00E+00	51.38	ITGA2
1.00E+00	9.04	ADIPOQ, PPARG
1.00E+00	8.91	SPTA1, SPTB
1.00E+00	8.91	COLEC11, MBL2
1.00E+00	5.14	EPB42, H3-3A, PPARG
1.00E+00	2.87	ADIPOQ, CMTM6, DPP6, PIGR, RELN, SLC4A1
1.00E+00	5.11	COLEC11, MBL2, SIGLEC16
1.00E+00	8.79	COL1A1, ITGA2
1.00E+00	2.13	ADIPOQ, COLEC11, GYPA, ITGA2, MARCO, MASP1, MASP2, MBL2, PPARG, SIGLEC16, S
1.00E+00	8.66	ADIPOQ, PPARG
1.00E+00	3.82	ADIPOQ, COL1A1, ITGA2, PPARG
1.00E+00	5.03	ADIPOQ, COL1A2, PPARG
1.00E+00	8.55	ADIPOQ, CLTCL1
1.00E+00	4.97	ADIPOQ, RELN, THBS4
1.00E+00	44.04	ADIPOQ
1.00E+00	44.04	H3-3A
1.00E+00	44.04	RELN
1.00E+00	44.04	ADIPOQ
1.00E+00	44.04	PLA2G7
1.00E+00	44.04	CELSR2
1.00E+00	44.04	LTBP1
1.00E+00	44.04	RELN
1.00E+00	44.04	LTBP1
1.00E+00	44.04	PPARG

1.00E+00	44.04	ADIPOQ
1.00E+00	44.04	RELN
1.00E+00	44.04	SPTLC3
1.00E+00	44.04	SPTLC3
1.00E+00	44.04	PLA2G7
1.00E+00	1.67	ADIPOQ, CDCA2, CDSN, CELSR2, COL1A1, COL1A2, COL2A1, EPB42, H3-3A, H4C1, ITGA
1.00E+00	8.32	ADIPOQ, CLTCL1
1.00E+00	8.32	GYPA, ITGA2
1.00E+00	8.21	COL1A2, PPARG
1.00E+00	8.21	GYPA, ITGA2
1.00E+00	8.10	EFEMP1, LTBP1
1.00E+00	1.74	ACAN, CELSR2, COL1A1, COL1A2, COL2A1, COLEC11, EFEMP1, ITGA2, LTBP1, MASP1, N
1.00E+00	2.53	ADIPOQ, CLTCL1, COLEC11, ITGA2, MBL2, PPARG, RELN
1.00E+00	8.00	SPTA1, SPTB
1.00E+00	7.89	SPTA1, SPTB
1.00E+00	7.89	ADIPOQ, PPARG
1.00E+00	7.89	ADIPOQ, PPARG
1.00E+00	7.89	RELN, THBS4
1.00E+00	38.53	PPARG
1.00E+00	38.53	GP9
1.00E+00	38.53	PPARG
1.00E+00	38.53	ITGA2
1.00E+00	38.53	COL1A1
1.00E+00	38.53	PKD2L1
1.00E+00	38.53	PPARG
1.00E+00	38.53	CDSN
1.00E+00	38.53	RELN
1.00E+00	38.53	PPARG
1.00E+00	38.53	PHLDB2
1.00E+00	38.53	SPTA1
1.00E+00	38.53	SLC4A1
1.00E+00	38.53	PLA2G7
1.00E+00	7.80	SPTA1, SPTB
1.00E+00	7.80	COLEC11, MBL2
1.00E+00	7.80	MARCO, SSC5D
1.00E+00	2.50	ADIPOQ, CDCA2, ITGA2, MARCO, PPARG, RELN, THBS4
1.00E+00	2.50	ADIPOQ, CDCA2, ITGA2, MARCO, PPARG, RELN, THBS4
1.00E+00	2.72	ADIPOQ, H3-3A, PHLDB2, PPARG, SPTA1, SPTB
1.00E+00	2.05	ADIPOQ, CELSR2, COL1A1, ITGA2, LAMA2, PHLDB2, PLA2G7, PPARG, RELN, THBS4, TN(
1.00E+00	2.48	ADIPOQ, EPB42, GP9, H4C1, PPARG, SLC4A1, SPTA1
1.00E+00	3.55	COLEC11, MBL2, PPARG, SIGLEC16
1.00E+00	7.60	ADIPOQ, PLA2G7
1.00E+00	1.67	ADIPOQ, CELSR2, CLTCL1, CMTM6, COL1A1, COLEC11, DPP6, GP9, H4C1, HNRNPA1L2,
1.00E+00	7.51	PPARG, THBS4
1.00E+00	7.51	ITGA2, PHLDB2
1.00E+00	7.51	ITGA2, MARCO
1.00E+00	2.46	COLEC11, MARCO, MASP1, MASP2, MBL2, PPARG, SSC5D

1.00E+00	4.54	COL1A1, ITGA2, PKD2L1
1.00E+00	34.25	GP9
1.00E+00	34.25	TNC
1.00E+00	34.25	ADIPOQ
1.00E+00	34.25	STEAP3
1.00E+00	34.25	COL1A1
1.00E+00	34.25	PHLDB2
1.00E+00	34.25	DCDC1
1.00E+00	34.25	PPARG
1.00E+00	34.25	H3-3A
1.00E+00	34.25	PIGR
1.00E+00	34.25	PPARG
1.00E+00	7.42	COL1A1, ITGA2
1.00E+00	1.72	ADIPOQ, COL1A1, COLEC11, GP9, HGFAC, ITGA2, MARCO, MASP1, MASP2, MBL2, NR0
1.00E+00	2.96	GP9, ITGA2, PIGR, PKD2L1, PPARG
1.00E+00	7.34	ADIPOQ, ITGA2
1.00E+00	3.45	ADIPOQ, ITGA2, PPARG, SPTA1
1.00E+00	7.25	ADIPOQ, PPARG
1.00E+00	7.25	EPB42, STEAP3
1.00E+00	7.25	SPTA1, SPTB
1.00E+00	4.43	COL1A2, LTBP1, PPARG
1.00E+00	7.17	ADIPOQ, PPARG
1.00E+00	2.62	COL1A1, COL1A2, COL2A1, LTBP1, MXRA5, PPARG
1.00E+00	1.69	ACAN, CELSR2, COL1A1, COL1A2, COL2A1, COLEC11, EFEMP1, ITGA2, LTBP1, MASP1, M
1.00E+00	7.08	LAMA2, PHLDB2
1.00E+00	7.08	ADIPOQ, COL1A1
1.00E+00	30.82	PHLDB2
1.00E+00	30.82	ADIPOQ
1.00E+00	30.82	PPARG
1.00E+00	30.82	SSC5D
1.00E+00	30.82	ADIPOQ
1.00E+00	30.82	LTBP1
1.00E+00	30.82	LTBP1
1.00E+00	30.82	EFEMP1
1.00E+00	30.82	COL2A1
1.00E+00	30.82	COL2A1
1.00E+00	30.82	H3-3A
1.00E+00	30.82	ADIPOQ
1.00E+00	30.82	ADIPOQ
1.00E+00	30.82	SPTLC3
1.00E+00	30.82	TNC
1.00E+00	30.82	PPARG
1.00E+00	3.37	ADIPOQ, EFEMP1, RELN, THBS4
1.00E+00	3.35	ADIPOQ, EFEMP1, RELN, THBS4
1.00E+00	2.38	ADIPOQ, CDSN, CELSR2, ITGA2, PCDHGB7, SPTA1, THBS4
1.00E+00	6.85	ADIPOQ, PPARG
1.00E+00	2.37	ADIPOQ, EPB42, GP9, H4C1, PPARG, SLC4A1, SPTA1

1.00E+00	2.84	COL1A1, ITGA2, PLA2G7, RELN, THBS4
1.00E+00	6.77	ADIPOQ, PPARG
1.00E+00	1.61	ACAN, ACTG2, ADIPOQ, CELSR2, COL1A1, COL1A2, COL2A1, COLEC11, EFEMP1, EPB42,
1.00E+00	1.87	ADIPOQ, COL1A1, COL1A2, COL2A1, EFEMP1, ITGA2, PIGR, PKD2L1, PPARG, RELN, SLC
1.00E+00	28.02	ITGA2
1.00E+00	28.02	PPARG
1.00E+00	28.02	ADIPOQ
1.00E+00	28.02	SSC5D
1.00E+00	28.02	NR0B1
1.00E+00	28.02	COL1A1
1.00E+00	28.02	PPARG
1.00E+00	28.02	ADIPOQ
1.00E+00	28.02	ITGA2
1.00E+00	28.02	ADIPOQ
1.00E+00	28.02	H3-3A
1.00E+00	28.02	PPARG
1.00E+00	28.02	PLA2G7
1.00E+00	28.02	PKD2L1
1.00E+00	28.02	PPARG
1.00E+00	6.70	ITGA2, PHLDB2
1.00E+00	6.70	ADIPOQ, ITGA2
1.00E+00	6.70	ADIPOQ, H4C1
1.00E+00	6.70	ADIPOQ, PPARG
1.00E+00	2.21	COLEC11, MARCO, MASP1, MASP2, MBL2, PPARG, SIGLEC16, SSC5D
1.00E+00	1.59	ADIPOQ, CDCA2, CDSN, CELSR2, COL1A1, COL1A2, COL2A1, EPB42, H3-3A, H4C1, ITGA
1.00E+00	6.63	ADIPOQ, PPARG
1.00E+00	4.15	COLEC11, MBL2, VCAN
1.00E+00	4.15	ITGA2, PLA2G7, THBS4
1.00E+00	2.02	ADIPOQ, COL1A1, COL1A2, COL2A1, COL6A1, ITGA2, LTBP1, MXRA5, NR0B1, PPARG
1.00E+00	4.14	ITGA2, PLA2G7, THBS4
1.00E+00	6.56	PLA2G7, THBS4
1.00E+00	6.49	COL1A1, COL2A1
1.00E+00	6.49	ACAN, VCAN
1.00E+00	6.49	COL6A3, COL7A1
1.00E+00	4.10	COL1A1, COL2A1, GP9
1.00E+00	3.22	COLEC11, MASP1, MASP2, MBL2
1.00E+00	25.68	CDSN
1.00E+00	25.68	PKD2L1
1.00E+00	25.68	ADIPOQ
1.00E+00	25.68	THBS4
1.00E+00	25.68	ITGA2
1.00E+00	25.68	COL1A1
1.00E+00	25.68	ADIPOQ
1.00E+00	25.68	LTBP1
1.00E+00	25.68	LTBP1
1.00E+00	25.68	ADIPOQ
1.00E+00	25.68	ITGA2

1.00E+00	25.68	LAMA2
1.00E+00	25.68	LTBP1
1.00E+00	25.68	LTBP1
1.00E+00	25.68	UBA52
1.00E+00	6.42	LTBP1, PPARG
1.00E+00	6.42	GNB4, TGM3
1.00E+00	6.42	H3-3A, H4C1
1.00E+00	2.30	CELSR2, EPB42, PHLDB2, PPARG, RELN, SPTA1, THBS4
1.00E+00	4.03	ADIPOQ, CLTCL1, RELN
1.00E+00	4.03	ACAN, THBS4, VCAN
1.00E+00	2.74	ADIPOQ, DPP6, PIGR, RELN, SLC4A1
1.00E+00	2.06	ADIPOQ, COLEC11, ITGA2, MBL2, PHLDB2, PLA2G7, PPARG, SIGLEC16, TNC
1.00E+00	6.29	ITGA2, PHLDB2
1.00E+00	6.29	KRT85, KRT86
1.00E+00	1.63	ACAN, ADIPOQ, CLTCL1, CMTM6, COL1A1, COL1A2, COL2A1, COL6A1, COL6A3, COL7A
1.00E+00	3.98	COLEC11, MBL2, PPARG
1.00E+00	2.71	COL1A1, ITGA2, PLA2G7, RELN, THBS4
1.00E+00	23.70	RELN
1.00E+00	23.70	NR0B1
1.00E+00	23.70	SLC4A1
1.00E+00	23.70	ITGA2
1.00E+00	23.70	ADIPOQ
1.00E+00	23.70	RELN
1.00E+00	23.70	PPARG
1.00E+00	6.17	COLEC11, MBL2
1.00E+00	6.17	ADIPOQ, SSC5D
1.00E+00	3.94	SNX14, SPTA1, SPTB
1.00E+00	6.11	ADIPOQ, SSC5D
1.00E+00	2.26	COL1A1, COL2A1, EFEMP1, ITGA2, PIGR, PKD2L1, TGFBI
1.00E+00	2.67	DPP6, HGFAC, MASP1, MASP2, RELN
1.00E+00	5.99	MASP2, MBL2
1.00E+00	5.99	ACAN, VCAN
1.00E+00	3.86	ADIPOQ, ITGA2, PPARG
1.00E+00	3.85	EPB42, SPTLC3, TGM3
1.00E+00	2.70	ACAN, ACTG2, ADIPOQ, C1orf56, CDSN, CELSR2, CLTCL1, CMTM5, CMTM6, COL1A1, C
1.00E+00	22.01	EFEMP1
1.00E+00	22.01	PPARG
1.00E+00	22.01	SPTLC3
1.00E+00	22.01	SPTLC3
1.00E+00	22.01	ITGA2
1.00E+00	22.01	PPARG
1.00E+00	22.01	PPARG
1.00E+00	22.01	PHLDB2
1.00E+00	22.01	COL2A1
1.00E+00	22.01	ADIPOQ
1.00E+00	22.01	COL2A1
1.00E+00	22.01	SIGLEC16

1.00E+00	22.01	RELN
1.00E+00	22.01	ITGA2
1.00E+00	22.01	PPARG
1.00E+00	22.01	PPARG
1.00E+00	22.01	PPARG
1.00E+00	22.01	PKD2L1
1.00E+00	22.01	COLEC11
1.00E+00	5.93	COL1A1, PHLDB2
1.00E+00	2.64	COL1A1, ITGA2, PLA2G7, RELN, THBS4
1.00E+00	3.81	EPB42, H3-3A, PPARG
1.00E+00	5.88	ADIPOQ, SLC4A1
1.00E+00	5.88	COL1A1, TMEM198
1.00E+00	2.22	ADIPOQ, EPB42, GP9, H4C1, PPARG, SLC4A1, SPTA1
1.00E+00	1.93	ADIPOQ, COL2A1, EPB42, GP9, LAMA2, PIGR, PPARG, SLC4A1, SPTA1, STEAP3
1.00E+00	5.77	EFEMP1, PIGR
1.00E+00	2.37	CELSR2, COL1A1, COL2A1, GNB4, NR0B1, RELN
1.00E+00	20.54	STEAP3
1.00E+00	20.54	ADIPOQ
1.00E+00	20.54	PLA2G7
1.00E+00	20.54	PPARG
1.00E+00	20.54	PLA2G7
1.00E+00	20.54	EPB42
1.00E+00	20.54	THBS4
1.00E+00	20.54	PPARG
1.00E+00	20.54	ITGA2
1.00E+00	20.54	RELN
1.00E+00	20.54	PHLDB2
1.00E+00	20.54	STEAP3
1.00E+00	2.37	COL1A1, COL1A2, PPARG, SVEP1, TGFBI, THBS4
1.00E+00	5.71	NR0B1, RELN
1.00E+00	1.92	DPP6, GP9, GYPA, ITGA2, MARCO, PCDHGB7, PIGR, PKD2L1, SLC4A1, TM4SF1
1.00E+00	3.71	ADIPOQ, CLTCL1, MARCO
1.00E+00	5.61	ITGA2, LAMA2
1.00E+00	5.61	ADIPOQ, CLTCL1
1.00E+00	5.61	ADIPOQ, PPARG
1.00E+00	19.26	PHLDB2
1.00E+00	19.26	SSC5D
1.00E+00	19.26	EPB42
1.00E+00	19.26	PHLDB2
1.00E+00	19.26	ITGA2
1.00E+00	19.26	COL1A2
1.00E+00	19.26	PKD2L1
1.00E+00	19.26	ADIPOQ
1.00E+00	19.26	GP9
1.00E+00	19.26	THBS4
1.00E+00	19.26	PPARG
1.00E+00	19.26	PHLDB2

1.00E+00	19.26	PPARG
1.00E+00	19.26	PPARG
1.00E+00	19.26	ACTG2
1.00E+00	3.19	ACAN, ACTG2, ADIPOQ, C1orf56, CDCA2, CDSN, CELSR2, CLTCL1, CMTM5, CMTM6, CO
1.00E+00	5.46	ADIPOQ, CLTCL1
1.00E+00	5.46	ADIPOQ, PLA2G7
1.00E+00	3.58	ADIPOQ, RELN, THBS4
1.00E+00	5.37	MBL2, SSC5D
1.00E+00	5.37	SPTA1, SPTB
1.00E+00	5.37	COLEC11, MBL2
1.00E+00	18.12	PPARG
1.00E+00	18.12	PPARG
1.00E+00	18.12	COL2A1
1.00E+00	18.12	ADIPOQ
1.00E+00	18.12	EFEMP1
1.00E+00	18.12	PPARG
1.00E+00	18.12	H4C1
1.00E+00	18.12	PPARG
1.00E+00	18.12	RELN
1.00E+00	18.12	CLTCL1
1.00E+00	18.12	LAMA2
1.00E+00	18.12	H4C1
1.00E+00	18.12	H4C1
1.00E+00	18.12	ITGA2
1.00E+00	1.62	ADIPOQ, CELSR2, CMTM6, COL1A2, COL2A1, EPB42, GP9, HGFAC, ITGA2, LAMA2, PIGR
1.00E+00	2.14	ACAN, CELSR2, COL2A1, GNB4, NR0B1, RELN, VCAN
1.00E+00	5.32	MASP2, MBL2
1.00E+00	5.32	ADIPOQ, CLTCL1
1.00E+00	3.54	ADIPOQ, COL1A1, PPARG
1.00E+00	2.28	ADIPOQ, ITGA2, MBL2, PLA2G7, PPARG, TNC
1.00E+00	2.49	ADIPOQ, COL1A1, EFEMP1, PIGR, SVEP1
1.00E+00	5.23	H3-3A, H4C1
1.00E+00	5.23	ADIPOQ, PPARG
1.00E+00	5.23	COL1A1, ITGA2
1.00E+00	5.23	ITGA2, PPARG
1.00E+00	3.50	ITGA2, PHLDB2, SLC4A1
1.00E+00	17.12	COL2A1
1.00E+00	17.12	ADIPOQ
1.00E+00	17.12	PPARG
1.00E+00	17.12	RELN
1.00E+00	17.12	RELN
1.00E+00	17.12	SLC4A1
1.00E+00	17.12	PPARG
1.00E+00	17.12	H4C1
1.00E+00	17.12	LTBP1
1.00E+00	17.12	SLC4A1
1.00E+00	2.12	ADIPOQ, CDSN, H3-3A, H4C1, MASP1, NR0B1, SLC4A1

1.00E+00	5.19	ADIPOQ, PPARG
1.00E+00	2.00	COL6A1, GNB4, H3-3A, ITGA2, SPTA1, SPTB, TGFBI, THBS4
1.00E+00	3.46	COL2A1, LAMA2, PIGR
1.00E+00	3.42	PHLDB2, SPTA1, SPTB
1.00E+00	5.06	ADIPOQ, SLC4A1
1.00E+00	16.21	PLA2G7
1.00E+00	16.21	RELN
1.00E+00	16.21	PPARG
1.00E+00	16.21	H4C1
1.00E+00	16.21	PLA2G7
1.00E+00	16.21	COL1A1
1.00E+00	16.21	GP9
1.00E+00	16.21	PPARG
1.00E+00	16.21	SSC5D
1.00E+00	16.21	SLC4A1
1.00E+00	16.21	ADIPOQ
1.00E+00	16.21	PPARG
1.00E+00	16.21	CLTCL1
1.00E+00	16.21	H3-3A
1.00E+00	16.21	SPTLC3
1.00E+00	1.98	CELSR2, EFEMP1, ITGA2, LTBP1, MARCO, PIGR, PKD2L1, PPARG
1.00E+00	2.44	ADIPOQ, COL1A1, COL1A2, COL6A1, PPARG
1.00E+00	5.02	ADIPOQ, RELN
1.00E+00	5.02	EFEMP1, PIGR
1.00E+00	5.02	SPTA1, SPTB
1.00E+00	1.90	ADIPOQ, COLEC11, MARCO, MASP1, MASP2, MBL2, PPARG, SIGLEC16, SSC5D
1.00E+00	4.98	PLA2G7, THBS4
1.00E+00	4.98	ITGA2, MARCO
1.00E+00	3.37	ADIPOQ, CELSR2, PCDHGB7
1.00E+00	1.89	ADIPOQ, COLEC11, MARCO, MASP1, MASP2, MBL2, PPARG, SIGLEC16, SSC5D
1.00E+00	4.94	EPB42, SLC4A1
1.00E+00	4.94	ADIPOQ, COL1A1
1.00E+00	4.94	COL1A1, COL2A1
1.00E+00	15.40	SPTLC3
1.00E+00	15.40	PPARG
1.00E+00	15.40	NR0B1
1.00E+00	15.40	PPARG
1.00E+00	15.40	PIGR
1.00E+00	15.40	NR0B1
1.00E+00	15.40	ADIPOQ
1.00E+00	15.40	ADIPOQ
1.00E+00	15.40	PLA2G7
1.00E+00	15.40	MASP2
1.00E+00	3.34	ADIPOQ, DPP6, SLC4A1
1.00E+00	1.51	ACAN, ADIPOQ, CDCA2, CDSN, COL2A1, COL6A3, COL7A1, COLEC11, DPP6, EFEMP1, EI
1.00E+00	3.30	CLTCL1, SNX14, STEAP3
1.00E+00	14.67	NR0B1

1.00E+00	14.67	PPARG
1.00E+00	14.67	PPARG
1.00E+00	14.67	PHLDB2
1.00E+00	14.67	SLC4A1
1.00E+00	14.67	GP9
1.00E+00	14.67	ADIPOQ
1.00E+00	14.67	ADIPOQ
1.00E+00	14.67	PPARG
1.00E+00	14.67	ADIPOQ
1.00E+00	14.67	COL2A1
1.00E+00	14.67	MBL2
1.00E+00	14.67	SIGLEC16
1.00E+00	14.67	RELN
1.00E+00	14.67	LAMA2
1.00E+00	14.67	GP9
1.00E+00	14.67	SLC4A1
1.00E+00	4.79	ADIPOQ, CLTCL1
1.00E+00	2.68	ADIPOQ, COL1A1, ITGA2, PPARG
1.00E+00	4.75	CDCA2, ITGA2
1.00E+00	4.75	H3-3A, H4C1
1.00E+00	4.68	ADIPOQ, PPARG
1.00E+00	4.68	COL1A1, COL1A2
1.00E+00	14.00	SPTLC3
1.00E+00	14.00	SPTLC3
1.00E+00	14.00	PPARG
1.00E+00	14.00	ADIPOQ
1.00E+00	14.00	ADIPOQ
1.00E+00	14.00	ADIPOQ
1.00E+00	14.00	RELN
1.00E+00	14.00	SLC4A1
1.00E+00	3.21	ADIPOQ, ITGA2, PPARG
1.00E+00	3.21	EPB42, SLC4A1, SPTA1
1.00E+00	4.64	ADIPOQ, PPARG
1.00E+00	4.64	ITGA2, MARCO
1.00E+00	4.61	ITGA2, PKD2L1
1.00E+00	4.61	EPB42, SLC4A1
1.00E+00	1.66	ADIPOQ, CELSR2, CMTM5, COL1A1, EFEMP1, EPB42, H4C1, LAMA2, PHLDB2, PPARG, R
1.00E+00	1.84	ADIPOQ, COLEC11, MARCO, MASP1, MASP2, MBL2, PPARG, SIGLEC16, SSC5D
1.00E+00	13.39	ADIPOQ
1.00E+00	13.39	ITGA2
1.00E+00	13.39	PPARG
1.00E+00	13.39	SVEP1
1.00E+00	13.39	PPARG
1.00E+00	13.39	PPARG
1.00E+00	13.39	ADIPOQ
1.00E+00	13.39	PPARG
1.00E+00	13.39	ACTG2

1.00E+00	13.39	PPARG
1.00E+00	13.39	PPARG
1.00E+00	1.51	ACTG2, ADIPOQ, C1orf56, CDCA2, CDSN, CELSR2, CLTCL1, CMTM5, CMTM6, COL1A1, C
1.00E+00	2.31	ADIPOQ, CDSN, MASP1, NR0B1, SLC4A1
1.00E+00	4.54	COL1A1, COL1A2
1.00E+00	4.54	NR0B1, PPARG
1.00E+00	2.59	ADIPOQ, CDSN, CELSR2, SPTA1
1.00E+00	4.51	ITGA2, PKD2L1
1.00E+00	4.51	SPTA1, SPTB
1.00E+00	4.51	CELSR2, RELN
1.00E+00	2.59	ADIPOQ, CMTM5, EFEMP1, THBS4
1.00E+00	1.51	ADIPOQ, CELSR2, CLTCL1, CMTM6, COL1A1, COLEC11, DPP6, GP9, HNRNPA1L2, ITGA2,
1.00E+00	12.83	SLC4A1
1.00E+00	12.83	PLA2G7
1.00E+00	12.83	RELN
1.00E+00	12.83	NR0B1
1.00E+00	12.83	RELN
1.00E+00	12.83	EFEMP1
1.00E+00	12.83	ADIPOQ
1.00E+00	12.83	ADIPOQ
1.00E+00	12.83	CMTM5
1.00E+00	12.83	SLC4A1
1.00E+00	12.83	ITGA2
1.00E+00	12.83	ITGA2
1.00E+00	12.83	THBS4
1.00E+00	12.83	SSC5D
1.00E+00	12.83	PPARG
1.00E+00	12.83	RELN
1.00E+00	12.83	CDSN
1.00E+00	12.83	PKD2L1
1.00E+00	12.83	LTBP1
1.00E+00	4.44	ADIPOQ, COL1A1
1.00E+00	3.10	H3-3A, NR0B1, TNC
1.00E+00	3.09	GYPA, ITGA2, MBL2
1.00E+00	4.41	ITGA2, MARCO
1.00E+00	4.41	EPB42, STEAP3
1.00E+00	2.55	ADIPOQ, CMTM5, EFEMP1, THBS4
1.00E+00	3.07	ADIPOQ, ITGA2, PPARG
1.00E+00	4.38	COL1A1, TMEM198
1.00E+00	2.26	ITGA2, PHLDB2, RELN, SVEP1, TNC
1.00E+00	2.09	COLEC11, MASP1, MASP2, MBL2, PIGR, PPARG
1.00E+00	3.06	H3-3A, NR0B1, TNC
1.00E+00	12.32	ITGA2
1.00E+00	12.32	ITGA2
1.00E+00	12.32	PPARG
1.00E+00	12.32	COL1A1
1.00E+00	12.32	TNC

1.00E+00	12.32	ADIPOQ
1.00E+00	12.32	ADIPOQ
1.00E+00	12.32	MASP2
1.00E+00	12.32	PKD2L1
1.00E+00	1.80	ADIPOQ, CLTCL1, CMTM6, COLEC11, ITGA2, MARCO, MBL2, PIGR, SSC5D
1.00E+00	4.35	ITGA2, RELN
1.00E+00	4.35	COLEC11, MBL2
1.00E+00	4.35	ACTG2, SLC4A1
1.00E+00	2.53	ACTG2, PKD2L1, SPTA1, SPTB
1.00E+00	3.04	EPB42, RELN, SPTA1
1.00E+00	4.32	SPTA1, SPTB
1.00E+00	1.86	ADIPOQ, COL1A1, COL1A2, COL2A1, COL6A1, LTBP1, NR0B1, PPARG
1.00E+00	3.03	ACTG2, COL1A1, PHLDB2
1.00E+00	2.52	ADIPOQ, COL1A1, ITGA2, PPARG
1.00E+00	2.24	ADIPOQ, COL1A1, COL1A2, COL6A1, PPARG
1.00E+00	1.70	CLTCL1, CMTM6, COL6A1, COL7A1, GNB4, MARCO, PIGR, PKD2L1, SNX14, STEAP3, UB/
1.00E+00	4.29	H3-3A, NR0B1
1.00E+00	4.29	LTBP1, PPARG
1.00E+00	4.29	ADIPOQ, PPARG
1.00E+00	2.24	COL1A1, COL1A2, COL2A1, LTBP1, PPARG
1.00E+00	11.85	NR0B1
1.00E+00	11.85	ITGA2
1.00E+00	11.85	COL1A1
1.00E+00	11.85	COL1A2
1.00E+00	11.85	PKD2L1
1.00E+00	11.85	ADIPOQ
1.00E+00	11.85	PHLDB2
1.00E+00	11.85	PLA2G7
1.00E+00	11.85	MARCO
1.00E+00	11.85	SNX14
1.00E+00	4.26	H3-3A, NR0B1
1.00E+00	4.23	PPARG, THBS4
1.00E+00	1.78	ADIPOQ, CMTM5, COL1A1, EFEMP1, H4C1, LAMA2, PHLDB2, PPARG, RELN
1.00E+00	2.22	ADIPOQ, CMTM5, EFEMP1, H4C1, PPARG
1.00E+00	4.20	LTBP1, PPARG
1.00E+00	11.41	ITGA2
1.00E+00	11.41	NR0B1
1.00E+00	11.41	TNC
1.00E+00	11.41	THBS4
1.00E+00	11.41	SLC4A1
1.00E+00	4.17	GYPA, ITGA2
1.00E+00	4.17	PPARG, THBS4
1.00E+00	4.14	ADIPOQ, PPARG
1.00E+00	4.14	PPARG, THBS4
1.00E+00	11.00	ADIPOQ
1.00E+00	11.00	SLC4A1
1.00E+00	11.00	MBL2

1.00E+00	11.00	RELN
1.00E+00	11.00	PLA2G7
1.00E+00	11.00	RELN
1.00E+00	4.09	EPB42, SPTA1
1.00E+00	4.09	ADIPOQ, PPARG
1.00E+00	4.09	ITGA2, PLA2G7
1.00E+00	1.67	ADIPOQ, CDSN, CELSR2, EPB42, GP9, H3-3A, LAMA2, PPARG, RELN, SLC4A1, TNC
1.00E+00	2.91	ADIPOQ, PPARG, RELN
1.00E+00	2.44	CELSR2, PHLDB2, TGM3, TNC
1.00E+00	4.06	ADIPOQ, SLC4A1
1.00E+00	4.06	ADIPOQ, PPARG
1.00E+00	4.06	CMTM6, PIGR
1.00E+00	4.06	CMTM6, PIGR
1.00E+00	4.04	ADIPOQ, CLTCL1
1.00E+00	4.04	H3-3A, H4C1
1.00E+00	1.81	ADIPOQ, CDCA2, ITGA2, MARCO, PPARG, RELN, SLC4A1, THBS4
1.00E+00	1.81	ADIPOQ, CDCA2, ITGA2, MARCO, PPARG, RELN, SLC4A1, THBS4
1.00E+00	10.62	H3-3A
1.00E+00	10.62	LAMA2
1.00E+00	10.62	PKD2L1
1.00E+00	10.62	PLA2G7
1.00E+00	10.62	PLA2G7
1.00E+00	10.62	COL1A1
1.00E+00	10.62	ADIPOQ
1.00E+00	10.62	PPARG
1.00E+00	10.62	RELN
1.00E+00	10.62	CLTCL1
1.00E+00	2.87	ITGA2, MARCO, PPARG
1.00E+00	1.89	CLTCL1, CMTM6, MARCO, PIGR, SNX14, STEAP3, UBA52
1.00E+00	1.43	ACAN, ADIPOQ, CDCA2, CDSN, COL2A1, COL6A3, COL7A1, COLEC11, DPP6, EFEMP1, EI
1.00E+00	1.74	ADIPOQ, CLTCL1, CMTM6, COL1A1, ITGA2, PHLDB2, PPARG, SNX14, STEAP3
1.00E+00	3.98	GYPA, ITGA2
1.00E+00	3.98	LTBP1, PPARG
1.00E+00	1.74	ADIPOQ, CELSR2, COL1A1, LAMA2, LTBP1, PPARG, RELN, TMEM198, TNC
1.00E+00	3.96	COL1A1, COL2A1
1.00E+00	3.96	SPTA1, SPTB
1.00E+00	2.84	COL2A1, LAMA2, PIGR
1.00E+00	10.26	SVEP1
1.00E+00	10.26	PPARG
1.00E+00	10.26	PPARG
1.00E+00	10.26	PPARG
1.00E+00	10.26	CELSR2
1.00E+00	10.26	TGM3
1.00E+00	10.26	COL1A1
1.00E+00	10.26	NR0B1
1.00E+00	10.26	ITGA2
1.00E+00	10.26	PPARG

1.00E+00	10.26	PPARG
1.00E+00	10.26	COL1A1
1.00E+00	10.26	RELN
1.00E+00	10.26	H3-3A
1.00E+00	10.26	ITGA2
1.00E+00	10.26	NR0B1
1.00E+00	10.26	SSC5D
1.00E+00	10.26	SLC4A1
1.00E+00	1.47	ADIPOQ, CELSR2, CLTCL1, CMTM6, COL1A1, COLEC11, DPP6, GP9, HNRNPA1L2, ITGA2,
1.00E+00	2.83	COL1A1, ITGA2, PKD2L1
1.00E+00	2.83	COL1A1, ITGA2, PKD2L1
1.00E+00	2.82	GP9, HGFAC, PLA2G7
1.00E+00	2.81	ADIPOQ, DPP6, SLC4A1
1.00E+00	3.88	SPTA1, SPTB
1.00E+00	3.88	EFEMP1, THBS4
1.00E+00	2.36	ADIPOQ, COL2A1, LAMA2, PIGR
1.00E+00	1.41	ADIPOQ, CELSR2, CMTM5, COL1A1, COL1A2, COL2A1, COL6A1, COLEC11, DCDC1, EFEN
1.00E+00	9.93	H4C1
1.00E+00	9.93	SPTLC3
1.00E+00	9.93	PLA2G7
1.00E+00	9.93	PPARG
1.00E+00	9.93	PPARG
1.00E+00	9.93	H3-3A
1.00E+00	9.93	EFEMP1
1.00E+00	9.93	PHLDB2
1.00E+00	2.12	ADIPOQ, MARCO, PPARG, RELN, THBS4
1.00E+00	2.79	ADIPOQ, COL1A1, NR0B1
1.00E+00	2.79	ADIPOQ, PPARG, SLC4A1
1.00E+00	1.86	COL1A1, COL1A2, COL2A1, PPARG, SVEP1, TGFBI, THBS4
1.00E+00	1.86	CLTCL1, CMTM6, MARCO, PIGR, SNX14, STEAP3, UBA52
1.00E+00	2.77	COLEC11, MBL2, PIGR
1.00E+00	2.34	CMTM6, SNX14, STEAP3, UBA52
1.00E+00	3.81	ADIPOQ, COL1A1
1.00E+00	2.77	GP9, LTBP1, PPARG
1.00E+00	9.62	ADIPOQ
1.00E+00	9.62	ITGA2
1.00E+00	9.62	PHLDB2
1.00E+00	9.62	RELN
1.00E+00	9.62	ADIPOQ
1.00E+00	9.62	ADIPOQ
1.00E+00	9.62	PPARG
1.00E+00	9.62	EFEMP1
1.00E+00	2.76	CLTCL1, MARCO, UBA52
1.00E+00	3.79	SLC4A1, SPTA1
1.00E+00	2.33	ADIPOQ, COLEC11, ITGA2, MBL2
1.00E+00	1.43	ACTG2, ADIPOQ, C1orf56, CDCA2, CDSN, CELSR2, CLTCL1, CMTM5, COL1A1, COL1A2, C
1.00E+00	3.76	ADIPOQ, PPARG

1.00E+00	2.32	ADIPOQ, CMTM5, EFEMP1, THBS4
1.00E+00	3.74	H3-3A, H4C1
1.00E+00	3.74	PKD2L1, VCAN
1.00E+00	2.73	ADIPOQ, NR0B1, PPARG
1.00E+00	2.73	COL1A2, GNB4, SLC4A1
1.00E+00	9.33	RELN
1.00E+00	9.33	ADIPOQ
1.00E+00	9.33	PLA2G7
1.00E+00	9.33	ITGA2
1.00E+00	9.33	PPARG
1.00E+00	9.33	THBS4
1.00E+00	9.33	RELN
1.00E+00	9.33	THBS4
1.00E+00	9.33	GNB4
1.00E+00	9.33	DCDC1
1.00E+00	3.72	COL1A1, PHLDB2
1.00E+00	3.72	EPB42, SLC4A1
1.00E+00	1.75	ADIPOQ, EFEMP1, H4C1, LAMA2, PHLDB2, PPARG, RELN, THBS4
1.00E+00	1.93	DCDC1, PKD2L1, PPARG, SLC4A1, SPTA1, SPTB
1.00E+00	3.70	CELSR2, PCDHGB7
1.00E+00	3.70	SPTA1, SPTB
1.00E+00	3.70	LAMA2, RELN
1.00E+00	3.67	H3-3A, NR0B1
1.00E+00	9.05	SPTA1
1.00E+00	9.05	PPARG
1.00E+00	9.05	ADIPOQ
1.00E+00	9.05	SPTA1
1.00E+00	9.05	LAMA2
1.00E+00	9.05	EFEMP1
1.00E+00	9.05	TGM3
1.00E+00	9.05	SLC4A1
1.00E+00	9.05	COL2A1
1.00E+00	9.05	COL2A1
1.00E+00	1.62	ACAN, ACTG2, ADIPOQ, C1orf56, CDSN, CELSR2, CLTCL1, CMTM5, CMTM6, COL1A1, C(
1.00E+00	3.65	GNB4, TGM3
1.00E+00	1.91	ADIPOQ, CMTM5, EFEMP1, H4C1, PPARG, THBS4
1.00E+00	2.27	ITGA2, PHLDB2, TGFBI, THBS4
1.00E+00	3.61	COL1A1, COL1A2
1.00E+00	8.80	RELN
1.00E+00	8.80	PPARG
1.00E+00	8.80	ITGA2
1.00E+00	8.80	PLA2G7
1.00E+00	8.80	PPARG
1.00E+00	8.80	ITGA2
1.00E+00	8.80	PHLDB2
1.00E+00	8.80	ADIPOQ
1.00E+00	8.80	EPB42

1.00E+00	8.80	RELN
1.00E+00	8.80	COL1A1
1.00E+00	8.80	PLA2G7
1.00E+00	8.80	PLA2G7
1.00E+00	8.80	NR0B1
1.00E+00	8.80	PLA2G7
1.00E+00	3.59	PPARG, RELN
1.00E+00	3.57	ACAN, VCAN
1.00E+00	2.03	COL1A1, COL1A2, PPARG, TGFBI, THBS4
1.00E+00	8.55	PIGR
1.00E+00	8.55	RELN
1.00E+00	8.55	NR0B1
1.00E+00	8.55	PHLDB2
1.00E+00	8.55	RELN
1.00E+00	8.55	PPARG
1.00E+00	8.55	PHLDB2
1.00E+00	8.55	ADIPOQ
1.00E+00	8.55	CLTCL1
1.00E+00	8.55	SPTLC3
1.00E+00	3.55	CELSR2, RELN
1.00E+00	3.53	COL2A1, PPARG
1.00E+00	3.53	COL2A1, PPARG
1.00E+00	3.53	H3-3A, H4C1
1.00E+00	2.01	DCDC1, KRT24, KRT32, KRT85, KRT86
1.00E+00	1.88	ADIPOQ, CDCA2, MARCO, PPARG, RELN, THBS4
1.00E+00	3.51	PPARG, THBS4
1.00E+00	3.51	ADIPOQ, PPARG
1.00E+00	8.32	PPARG
1.00E+00	8.32	ADIPOQ
1.00E+00	8.32	SSC5D
1.00E+00	8.32	H4C1
1.00E+00	8.32	PHLDB2
1.00E+00	8.32	PLA2G7
1.00E+00	3.49	COL6A3, COL7A1
1.00E+00	2.59	PHLDB2, SPTA1, SPTB
1.00E+00	3.47	ADIPOQ, PPARG
1.00E+00	3.47	COL1A1, COL1A2
1.00E+00	1.99	ADIPOQ, COL6A3, COL7A1, PPARG, SLC4A1
1.00E+00	2.57	ADIPOQ, PHLDB2, PPARG
1.00E+00	2.57	LTBP1, PPARG, STEAP3
1.00E+00	8.10	PIGR
1.00E+00	8.10	SSC5D
1.00E+00	8.10	ADIPOQ
1.00E+00	8.10	LTBP1
1.00E+00	8.10	MARCO
1.00E+00	8.10	PLA2G7
1.00E+00	8.10	PHLDB2

1.00E+00	1.43	ACAN, ADIPOQ, C1orf56, CDSN, CLTCL1, CMTM5, CMTM6, COL1A1, COL1A2, COL2A1,
1.00E+00	2.56	COL1A1, ITGA2, PHLDB2
1.00E+00	3.43	PPARG, THBS4
1.00E+00	3.43	GYPA, ITGA2
1.00E+00	3.41	ADIPOQ, PPARG
1.00E+00	7.89	PPARG
1.00E+00	7.89	PHLDB2
1.00E+00	7.89	CELSR2
1.00E+00	7.89	ITGA2
1.00E+00	7.89	ITGA2
1.00E+00	7.89	GP9
1.00E+00	7.89	CDSN
1.00E+00	7.89	GNB4
1.00E+00	7.89	H3-3A
1.00E+00	2.18	ADIPOQ, COL1A1, ITGA2, NR0B1
1.00E+00	3.39	H3-3A, H4C1
1.00E+00	3.39	ADIPOQ, PPARG
1.00E+00	2.17	CELSR2, COL1A1, RELN, TMEM198
1.00E+00	3.37	ADIPOQ, SLC4A1
1.00E+00	3.37	ADIPOQ, PPARG
1.00E+00	2.52	ACTG2, ITGA2, PPARG
1.00E+00	3.35	COLEC11, MBL2
1.00E+00	3.35	COL6A3, COL7A1
1.00E+00	7.69	ADIPOQ
1.00E+00	7.69	ADIPOQ
1.00E+00	1.68	CELSR2, EFEMP1, ITGA2, LTBP1, MARCO, PIGR, PKD2L1, PPARG
1.00E+00	1.68	CELSR2, EFEMP1, ITGA2, LTBP1, MARCO, PIGR, PKD2L1, PPARG
1.00E+00	3.32	H3-3A, ITGA2
1.00E+00	3.30	DCDC1, THBS4
1.00E+00	7.51	NR0B1
1.00E+00	7.51	COL1A1
1.00E+00	7.51	PIGR
1.00E+00	7.51	LTBP1
1.00E+00	7.51	COL2A1
1.00E+00	2.48	ADIPOQ, PPARG, SLC4A1
1.00E+00	2.47	MBL2, SIGLEC16, SSC5D
1.00E+00	2.13	ADIPOQ, ITGA2, PPARG, RELN
1.00E+00	1.66	COL1A1, COL2A1, EFEMP1, ITGA2, PIGR, PKD2L1, RELN, TGFBI
1.00E+00	3.26	NR0B1, PPARG
1.00E+00	2.46	ADIPOQ, PHLDB2, PPARG
1.00E+00	1.81	ADIPOQ, COL1A1, COL1A2, COL6A1, ITGA2, PPARG
1.00E+00	2.46	PHLDB2, SPTA1, SPTB
1.00E+00	7.33	PIGR
1.00E+00	7.33	ADIPOQ
1.00E+00	7.33	PPARG
1.00E+00	7.33	COL1A1
1.00E+00	7.33	H4C1

1.00E+00	3.23	MARCO, UBA52
1.00E+00	3.23	COL6A3, COL7A1
1.00E+00	1.80	ADIPOQ, EPB42, GP9, PPARG, SLC4A1, STEAP3
1.00E+00	7.16	ITGA2
1.00E+00	7.16	ITGA2
1.00E+00	7.16	GNB4
1.00E+00	7.16	RELN
1.00E+00	7.16	ADIPOQ
1.00E+00	7.16	RELN
1.00E+00	7.16	CLTCL1
1.00E+00	7.16	H3-3A
1.00E+00	7.16	PKD2L1
1.00E+00	2.10	EPB42, GP9, SLC4A1, STEAP3
1.00E+00	1.91	ADIPOQ, MARCO, PPARG, RELN, THBS4
1.00E+00	2.42	COL1A2, LTBP1, PPARG
1.00E+00	6.99	TM4SF1
1.00E+00	6.99	PKD2L1
1.00E+00	6.99	ITGA2
1.00E+00	6.99	PPARG
1.00E+00	6.99	PLA2G7
1.00E+00	6.99	UBA52
1.00E+00	2.08	COLEC11, MASP1, MASP2, MBL2
1.00E+00	3.15	PPARG, THBS4
1.00E+00	2.07	ADIPOQ, COL1A2, PPARG, SVEP1
1.00E+00	3.13	SPTA1, SPTB
1.00E+00	3.13	ADIPOQ, SPTA1
1.00E+00	6.84	RELN
1.00E+00	6.84	RELN
1.00E+00	6.84	RELN
1.00E+00	6.84	ADIPOQ
1.00E+00	6.84	RELN
1.00E+00	6.84	COL2A1
1.00E+00	6.84	PKD2L1
1.00E+00	3.12	SPTA1, SPTB
1.00E+00	3.10	ADIPOQ, CDSN
1.00E+00	2.37	ITGA2, PLA2G7, THBS4
1.00E+00	6.69	SLC4A1
1.00E+00	6.69	COL1A1
1.00E+00	6.69	COL1A1
1.00E+00	6.69	ADIPOQ
1.00E+00	6.69	PPARG
1.00E+00	6.69	THBS4
1.00E+00	6.69	PPARG
1.00E+00	6.69	RELN
1.00E+00	3.08	GYPA, ITGA2
1.00E+00	2.05	EPB42, GP9, SLC4A1, STEAP3
1.00E+00	2.04	ADIPOQ, CLTCL1, DPP6, RELN

1.00E+00	6.55	MBL2
1.00E+00	6.55	TNC
1.00E+00	6.55	PPARG
1.00E+00	6.55	RELN
1.00E+00	6.55	ADIPOQ
1.00E+00	6.55	ADIPOQ
1.00E+00	6.55	PPARG
1.00E+00	6.55	PLA2G7
1.00E+00	6.55	ADIPOQ
1.00E+00	3.02	ADIPOQ, ITGA2
1.00E+00	2.32	PHLDB2, SPTA1, SPTB
1.00E+00	1.50	ADIPOQ, CDCA2, H3-3A, ITGA2, LAMA2, PHLDB2, PPARG, RELN, SPTA1, SPTB, TNC
1.00E+00	6.41	PPARG
1.00E+00	6.41	H3-3A
1.00E+00	6.41	CDCA2
1.00E+00	6.41	ITGA2
1.00E+00	6.41	PPARG
1.00E+00	6.41	PPARG
1.00E+00	3.01	ADIPOQ, PPARG
1.00E+00	2.01	EPB42, GP9, SLC4A1, STEAP3
1.00E+00	1.45	ADIPOQ, COL1A1, EFEMP1, H4C1, ITGA2, LAMA2, LTBP1, PHLDB2, PPARG, RELN, SIGLE
1.00E+00	2.31	ITGA2, MARCO, PPARG
1.00E+00	2.99	ADIPOQ, ITGA2
1.00E+00	2.99	H3-3A, ITGA2
1.00E+00	2.98	ADIPOQ, PPARG
1.00E+00	2.30	H3-3A, SPTA1, SPTB
1.00E+00	6.28	PPARG
1.00E+00	1.84	ADIPOQ, COL1A1, ITGA2, NR0B1, PPARG
1.00E+00	6.15	ADIPOQ
1.00E+00	6.15	TNC
1.00E+00	6.15	ADIPOQ
1.00E+00	6.15	MARCO
1.00E+00	6.15	ADIPOQ
1.00E+00	6.15	COL1A1
1.00E+00	6.15	PPARG
1.00E+00	6.15	PPARG
1.00E+00	2.94	ADIPOQ, CDSN
1.00E+00	2.94	ADIPOQ, PLA2G7
1.00E+00	2.92	ADIPOQ, H4C1
1.00E+00	1.51	ADIPOQ, CELSR2, CLTCL1, COLEC11, DPP6, GP9, ITGA2, MBL2, PPARG, RELN
1.00E+00	2.91	MASP2, MBL2
1.00E+00	6.03	ADIPOQ
1.00E+00	6.03	GP9
1.00E+00	6.03	PPARG
1.00E+00	6.03	PPARG
1.00E+00	6.03	PPARG
1.00E+00	6.03	GNB4

1.00E+00	6.03	PPARG
1.00E+00	2.90	ADIPOQ, PPARG
1.00E+00	2.25	ADIPOQ, PHLDB2, PPARG
1.00E+00	5.92	ADIPOQ
1.00E+00	5.92	SPTA1
1.00E+00	5.92	ADIPOQ
1.00E+00	5.92	SLC4A1
1.00E+00	5.92	CELSR2
1.00E+00	5.92	THBS4
1.00E+00	2.24	GYPA, ITGA2, MBL2
1.00E+00	2.24	ADIPOQ, COL1A1, PPARG
1.00E+00	2.87	ADIPOQ, PPARG
1.00E+00	2.87	MASP2, MBL2
1.00E+00	2.87	ADIPOQ, PPARG
1.00E+00	2.87	H3-3A, ITGA2
1.00E+00	2.23	ITGA2, PHLDB2, TNC
1.00E+00	2.85	SPTA1, SPTB
1.00E+00	1.79	ITGA2, MARCO, RELN, SLC4A1, SPTA1
1.00E+00	5.80	PLA2G7
1.00E+00	5.80	PPARG
1.00E+00	5.80	EFEMP1
1.00E+00	5.80	H4C1
1.00E+00	5.80	DPP6
1.00E+00	2.21	HGFAC, MASP1, MASP2
1.00E+00	2.82	COL1A1, PHLDB2
1.00E+00	2.82	ADIPOQ, PLA2G7
1.00E+00	2.82	DPP6, PKD2L1
1.00E+00	5.70	PPARG
1.00E+00	5.70	TNC
1.00E+00	5.70	PIGR
1.00E+00	5.70	PPARG
1.00E+00	5.70	ADIPOQ
1.00E+00	5.70	ACTG2
1.00E+00	5.70	SPTLC3
1.00E+00	2.20	COL6A3, COL7A1, PPARG
1.00E+00	2.80	DPP6, PKD2L1
1.00E+00	5.59	COL1A1
1.00E+00	5.59	SLC4A1
1.00E+00	5.59	ADIPOQ
1.00E+00	5.59	PPARG
1.00E+00	5.59	RELN
1.00E+00	5.59	RELN
1.00E+00	5.59	PPARG
1.00E+00	5.59	SPTLC3
1.00E+00	2.78	H3-3A, H4C1
1.00E+00	2.18	ADIPOQ, COL1A1, ITGA2
1.00E+00	2.18	ITGA2, PHLDB2, TNC

1.00E+00	2.17	ADIPOQ, PPARG, SLC4A1
1.00E+00	2.17	ADIPOQ, COL1A1, MBL2
1.00E+00	2.75	COL1A1, COL2A1
1.00E+00	5.49	RELN
1.00E+00	5.49	CELSR2
1.00E+00	5.49	CLTCL1
1.00E+00	5.49	CLTCL1
1.00E+00	1.66	ADIPOQ, COL1A1, COL1A2, COL6A1, ITGA2, PPARG
1.00E+00	2.16	ADIPOQ, PHLDB2, PPARG
1.00E+00	5.39	STEAP3
1.00E+00	5.39	ADIPOQ
1.00E+00	5.39	SLC4A1
1.00E+00	5.39	ADIPOQ
1.00E+00	5.39	H3-3A
1.00E+00	5.39	RELN
1.00E+00	5.39	ADIPOQ
1.00E+00	2.14	ADIPOQ, PPARG, SLC4A1
1.00E+00	2.13	ADIPOQ, PPARG, SLC4A1
1.00E+00	2.13	ITGA2, NR0B1, TNC
1.00E+00	5.30	H3-3A
1.00E+00	5.30	PKD2L1
1.00E+00	5.30	RELN
1.00E+00	5.30	SNX14
1.00E+00	5.30	CDSN
1.00E+00	5.30	UBA52
1.00E+00	2.69	NR0B1, UBA52
1.00E+00	2.69	COL6A3, COL7A1
1.00E+00	2.68	H3-3A, NR0B1
1.00E+00	2.67	ADIPOQ, ITGA2
1.00E+00	2.67	ADIPOQ, PPARG
1.00E+00	2.67	ADIPOQ, COL1A1
1.00E+00	5.21	PHLDB2
1.00E+00	5.21	RELN
1.00E+00	5.21	ADIPOQ
1.00E+00	5.21	EFEMP1
1.00E+00	1.87	ITGA2, PIGR, PKD2L1, SSC5D
1.00E+00	1.72	ADIPOQ, COL1A1, LAMA2, PPARG, RELN
1.00E+00	2.65	LAMA2, RELN
1.00E+00	5.12	ADIPOQ
1.00E+00	5.12	PPARG
1.00E+00	2.10	ADIPOQ, LAMA2, RELN
1.00E+00	2.63	CELSR2, RELN
1.00E+00	2.63	LAMA2, RELN
1.00E+00	2.63	ADIPOQ, CMTM5
1.00E+00	1.72	ADIPOQ, COL1A1, ITGA2, NR0B1, PPARG
1.00E+00	2.09	ADIPOQ, LAMA2, RELN
1.00E+00	2.62	ITGA2, PHLDB2

1.00E+00	2.62	H3-3A, NR0B1
1.00E+00	1.42	CLTCL1, CMTM6, COL1A1, COL7A1, MARCO, PIGR, PKD2L1, SNX14, STEAP3, TMEM198
1.00E+00	5.04	H3-3A
1.00E+00	5.04	TNC
1.00E+00	5.04	ADIPOQ
1.00E+00	5.04	ADIPOQ
1.00E+00	5.04	PPARG
1.00E+00	5.04	PPARG
1.00E+00	5.04	RELN
1.00E+00	1.42	CLTCL1, CMTM6, COL1A1, COL7A1, MARCO, PIGR, PKD2L1, SNX14, STEAP3, TMEM198
1.00E+00	2.60	PLA2G7, THBS4
1.00E+00	2.60	ADIPOQ, PPARG
1.00E+00	2.07	PHLDB2, SPTA1, SPTB
1.00E+00	2.07	NR0B1, PPARG, RELN
1.00E+00	4.96	RELN
1.00E+00	4.96	PPARG
1.00E+00	4.96	ADIPOQ
1.00E+00	4.96	SPTLC3
1.00E+00	4.96	NR0B1
1.00E+00	4.96	ADIPOQ
1.00E+00	4.96	SLC4A1
1.00E+00	4.96	STEAP3
1.00E+00	1.61	ACTG2, ADIPOQ, ITGA2, PPARG, SIGLEC16, TNC
1.00E+00	2.58	H3-3A, H4C1
1.00E+00	2.58	PLA2G7, THBS4
1.00E+00	2.06	CELSR2, COL1A1, TMEM198
1.00E+00	1.83	CMTM5, COL6A3, H3-3A, LAMA2
1.00E+00	4.88	ITGA2
1.00E+00	4.88	PHLDB2
1.00E+00	4.88	PHLDB2
1.00E+00	4.88	ITGA2
1.00E+00	2.05	CELSR2, COL1A1, TMEM198
1.00E+00	2.56	ADIPOQ, PPARG
1.00E+00	2.04	COL6A3, COL7A1, PPARG
1.00E+00	4.80	ITGA2
1.00E+00	4.80	SPTA1
1.00E+00	4.80	GNB4
1.00E+00	4.80	PPARG
1.00E+00	4.80	PPARG
1.00E+00	1.60	CDCA2, H3-3A, PHLDB2, PPARG, SPTA1, SPTB
1.00E+00	1.60	ADIPOQ, COL6A3, COL7A1, NR0B1, PPARG, SLC4A1
1.00E+00	2.03	COLEC11, ITGA2, MBL2
1.00E+00	2.53	DPP6, PKD2L1
1.00E+00	2.53	CLTCL1, CMTM6
1.00E+00	2.53	COL1A2, PKD2L1
1.00E+00	4.73	RELN
1.00E+00	4.73	PPARG

1.00E+00	4.73	COL1A1
1.00E+00	4.73	NR0B1
1.00E+00	2.51	ADIPOQ, PPARG
1.00E+00	2.50	ADIPOQ, PPARG
1.00E+00	2.50	H3-3A, H4C1
1.00E+00	1.79	COLEC11, MASP1, MASP2, MBL2
1.00E+00	4.66	PPARG
1.00E+00	4.66	HGFAC
1.00E+00	2.49	HGFAC, PLA2G7
1.00E+00	2.49	H3-3A, H4C1
1.00E+00	2.47	COL6A3, COL7A1
1.00E+00	2.47	COL1A1, PHLDB2
1.00E+00	4.59	COL2A1
1.00E+00	4.59	PLA2G7
1.00E+00	4.59	SPTLC3
1.00E+00	4.59	COL2A1
1.00E+00	4.59	LAMA2
1.00E+00	1.41	ACAN, ACTG2, ADIPOQ, COL2A1, EPB42, ITGA2, SIGLEC16, THBS4, TNC, VCAN
1.00E+00	2.45	ADIPOQ, COL1A1
1.00E+00	4.52	SPTA1
1.00E+00	4.52	PPARG
1.00E+00	4.52	ADIPOQ
1.00E+00	4.52	RELN
1.00E+00	4.52	PHLDB2
1.00E+00	4.52	PKD2L1
1.00E+00	4.52	CLTCL1
1.00E+00	2.43	ITGA2, PPARG
1.00E+00	1.30	ADIPOQ, CMTM5, COL1A1, COL1A2, COL2A1, COL6A1, ITGA2, LAMA2, LTBP1, MXRA5,
1.00E+00	4.45	ADIPOQ
1.00E+00	4.45	H3-3A
1.00E+00	1.76	DPP6, GNB4, GP9, ITGA2
1.00E+00	1.63	ADIPOQ, COLEC11, ITGA2, MBL2, PKD2L1
1.00E+00	4.39	PLA2G7
1.00E+00	4.39	RELN
1.00E+00	4.39	CLTCL1
1.00E+00	4.39	PPARG
1.00E+00	2.40	COL1A1, TMEM198
1.00E+00	1.55	ADIPOQ, COL1A1, COL1A2, COL6A1, ITGA2, PPARG
1.00E+00	4.33	PPARG
1.00E+00	4.33	NR0B1
1.00E+00	4.33	CELSR2
1.00E+00	4.33	PPARG
1.00E+00	4.33	PPARG
1.00E+00	2.39	COLEC11, MBL2
1.00E+00	1.94	ADIPOQ, PPARG, SLC4A1
1.00E+00	1.94	SNX14, SPTA1, SPTB
1.00E+00	1.94	ITGA2, LAMA2, SPTA1

1.00E+00	2.38	CLTCL1, TGFBI
1.00E+00	2.37	COL6A3, COL7A1
1.00E+00	4.27	RELN
1.00E+00	4.27	ADIPOQ
1.00E+00	4.27	EFEMP1
1.00E+00	4.27	PLA2G7
1.00E+00	4.27	SLC4A1
1.00E+00	4.27	THBS4
1.00E+00	1.45	ADIPOQ, COL1A1, ITGA2, LAMA2, MARCO, PPARG, RELN, TMEM198
1.00E+00	4.21	ITGA2
1.00E+00	4.21	CELSR2
1.00E+00	4.21	CELSR2
1.00E+00	4.21	PPARG
1.00E+00	1.44	ADIPOQ, COL1A1, ITGA2, LAMA2, MARCO, PPARG, RELN, TMEM198
1.00E+00	2.34	NR0B1, PPARG
1.00E+00	2.34	PPARG, RELN
1.00E+00	1.91	LAMA2, RELN, TNC
1.00E+00	4.15	CMTM5
1.00E+00	4.15	NR0B1
1.00E+00	1.90	COL1A2, GNB4, SLC4A1
1.00E+00	1.47	EPB42, KRT32, KRT85, KRT86, PHLDB2, SPTA1, SPTB
1.00E+00	2.90	ACAN, ACTG2, ADIPOQ, C1orf56, CDCA2, CDSN, CELSR2, CLTCL1, CMTM5, CMTM6, CO
1.00E+00	4.10	PPARG
1.00E+00	4.10	ADIPOQ
1.00E+00	4.10	ITGA2
1.00E+00	4.10	NR0B1
1.00E+00	4.10	ITGA2
1.00E+00	4.10	PIGR
1.00E+00	4.10	PKD2L1
1.00E+00	2.31	ITGA2, THBS4
1.00E+00	4.04	PPARG
1.00E+00	4.04	ADIPOQ
1.00E+00	4.04	PPARG
1.00E+00	4.04	PLA2G7
1.00E+00	4.04	UBA52
1.00E+00	2.29	ADIPOQ, PPARG
1.00E+00	2.29	COL2A1, EFEMP1
1.00E+00	3.99	PPARG
1.00E+00	3.99	RELN
1.00E+00	3.99	PHLDB2
1.00E+00	3.99	ADIPOQ
1.00E+00	1.46	ADIPOQ, CDCA2, ITGA2, MARCO, PPARG, RELN, THBS4
1.00E+00	1.86	COLEC11, MBL2, PIGR
1.00E+00	3.94	MARCO
1.00E+00	3.94	ADIPOQ
1.00E+00	3.94	DPP6
1.00E+00	2.25	ADIPOQ, PPARG

1.00E+00	2.25	H3-3A, H4C1
1.00E+00	3.89	PIGR
1.00E+00	3.89	RELN
1.00E+00	3.89	RELN
1.00E+00	3.89	HGFAC
1.00E+00	3.89	COL7A1
1.00E+00	1.41	ADIPOQ, CLTCL1, COLEC11, DPP6, ITGA2, MBL2, PPARG, RELN
1.00E+00	1.38	ADIPOQ, C1orf56, H3-3A, ITGA2, PPARG, SPTA1, TGFBI, THBS4, TNC
1.00E+00	1.49	ADIPOQ, MARCO, PPARG, RELN, SLC4A1, THBS4
1.00E+00	1.84	ADIPOQ, COL1A1, PPARG
1.00E+00	3.84	RELN
1.00E+00	3.84	SVEP1
1.00E+00	3.84	PPARG
1.00E+00	2.21	SPTA1, SPTB
1.00E+00	1.83	PIGR, PKD2L1, SSC5D
1.00E+00	3.79	RELN
1.00E+00	3.79	RELN
1.00E+00	3.79	LAMA2
1.00E+00	3.79	PPARG
1.00E+00	3.75	ADIPOQ
1.00E+00	3.75	SLC4A1
1.00E+00	3.75	H3-3A
1.00E+00	1.81	ADIPOQ, PLA2G7, PPARG
1.00E+00	1.54	CELSR2, EPB42, LAMA2, RELN, SPTA1
1.00E+00	3.70	SLC4A1
1.00E+00	3.70	ITGA2
1.00E+00	3.70	COLEC11
1.00E+00	1.80	EPB42, GP9, STEAP3
1.00E+00	2.16	H3-3A, NR0B1
1.00E+00	3.66	DCDC1
1.00E+00	3.66	RELN
1.00E+00	3.66	SLC4A1
1.00E+00	1.63	CELSR2, GNB4, NR0B1, RELN
1.00E+00	1.63	CELSR2, LAMA2, PHLDB2, RELN
1.00E+00	2.14	LTBP1, PPARG
1.00E+00	3.61	ITGA2
1.00E+00	3.61	ADIPOQ
1.00E+00	3.61	SPTA1
1.00E+00	3.61	CMTM6
1.00E+00	3.61	GNB4
1.00E+00	1.78	ADIPOQ, COL1A2, PPARG
1.00E+00	2.14	ADIPOQ, PPARG
1.00E+00	2.13	ITGA2, PPARG
1.00E+00	2.13	ADIPOQ, PPARG
1.00E+00	3.57	PPARG
1.00E+00	3.57	PPARG
1.00E+00	3.57	RELN

1.00E+00	1.41	ADIPOQ, COL1A1, LAMA2, MARCO, PPARG, RELN, TMEM198
1.00E+00	1.52	CLTCL1, CMTM6, SNX14, STEAP3, UBA52
1.00E+00	2.11	COL2A1, EFEMP1
1.00E+00	3.53	ITGA2
1.00E+00	1.41	ADIPOQ, CLTCL1, CMTM6, COL1A1, PPARG, SNX14, STEAP3
1.00E+00	2.10	ADIPOQ, SLC4A1
1.00E+00	2.10	DPP6, PKD2L1
1.00E+00	3.49	CELSR2
1.00E+00	3.49	TNC
1.00E+00	3.49	SSC5D
1.00E+00	3.49	CLTCL1
1.00E+00	3.49	THBS4
1.00E+00	3.49	CLTCL1
1.00E+00	3.49	DPP6
1.00E+00	3.49	CLTCL1
1.00E+00	1.22	ADIPOQ, CELSR2, CMTM5, COL1A1, COL1A2, COL2A1, COLEC11, DCDC1, EFEMP1, GNB
1.00E+00	3.45	COL2A1
1.00E+00	3.45	PPARG
1.00E+00	3.45	SLC4A1
1.00E+00	3.45	ADIPOQ
1.00E+00	3.45	PKD2L1
1.00E+00	3.45	CLTCL1
1.00E+00	3.45	HNRNPA1L2
1.00E+00	1.37	COL1A2, H3-3A, H4C1, PKD2L1, PLA2G7, SNX14, SPTA1, SPTB
1.00E+00	1.40	ADIPOQ, CLTCL1, DPP6, GP9, PKD2L1, RELN, SLC4A1
1.00E+00	1.59	ADIPOQ, MBL2, SIGLEC16, SSC5D
1.00E+00	2.07	ADIPOQ, PPARG
1.00E+00	3.41	TGM3
1.00E+00	3.41	GNB4
1.00E+00	3.41	MBL2
1.00E+00	3.41	COL2A1
1.00E+00	3.41	H3-3A
1.00E+00	3.41	RELN
1.00E+00	3.41	CMTM6
1.00E+00	1.73	PHLDB2, SPTA1, SPTB
1.00E+00	2.06	ADIPOQ, PPARG
1.00E+00	3.37	ADIPOQ
1.00E+00	3.37	EFEMP1
1.00E+00	3.37	PPARG
1.00E+00	3.37	SLC4A1
1.00E+00	3.37	RELN
1.00E+00	1.72	ITGA2, PIGR, PKD2L1
1.00E+00	3.34	CLTCL1
1.00E+00	3.34	LTBP1
1.00E+00	3.34	H3-3A
1.00E+00	3.34	PPARG
1.00E+00	3.34	THBS4

1.00E+00	3.34	SLC4A1
1.00E+00	2.03	ADIPOQ, PPARG
1.00E+00	3.30	TGM3
1.00E+00	3.30	TGM3
1.00E+00	3.30	CDCA2
1.00E+00	3.30	COL2A1
1.00E+00	3.30	PHLDB2
1.00E+00	3.30	SLC4A1
1.00E+00	3.30	PHLDB2
1.00E+00	2.02	ADIPOQ, RELN
1.00E+00	3.27	DCDC1
1.00E+00	3.27	TGM3
1.00E+00	3.27	COL7A1
1.00E+00	1.42	CLTCL1, DPP6, GNB4, GP9, ITGA2, PKD2L1
1.00E+00	2.00	COL1A1, TMEM198
1.00E+00	2.00	CLTCL1, COL7A1
1.00E+00	3.23	CELSR2
1.00E+00	3.23	ADIPOQ
1.00E+00	3.23	ITGA2
1.00E+00	3.23	RELN
1.00E+00	1.99	COLEC11, MBL2
1.00E+00	1.99	COLEC11, MBL2
1.00E+00	3.20	PPARG
1.00E+00	3.20	PPARG
1.00E+00	3.20	H3-3A
1.00E+00	3.20	VCAN
1.00E+00	3.20	CMTM6
1.00E+00	1.98	CMTM6, PIGR
1.00E+00	3.16	RELN
1.00E+00	3.16	ADIPOQ
1.00E+00	3.16	ITGA2
1.00E+00	3.16	ADIPOQ
1.00E+00	3.16	RELN
1.00E+00	1.97	ADIPOQ, MARCO
1.00E+00	1.96	GNB4, TGM3
1.00E+00	3.13	PPARG
1.00E+00	3.13	SLC4A1
1.00E+00	3.13	SLC4A1
1.00E+00	3.13	ADIPOQ
1.00E+00	3.13	SIGLEC16
1.00E+00	3.13	DPP6
1.00E+00	3.13	COL2A1
1.00E+00	1.20	ADIPOQ, CDSN, CMTM5, COL1A1, COL2A1, COL6A3, COL7A1, EFEMP1, GP9, H3-3A, H4
1.00E+00	3.10	SPTA1
1.00E+00	3.10	SLC4A1
1.00E+00	3.10	RELN
1.00E+00	1.94	GYPA, ITGA2

1.00E+00	1.94	PLA2G7, THBS4
1.00E+00	1.94	GP9, ITGA2
1.00E+00	1.65	CELSR2, LAMA2, RELN
1.00E+00	3.07	ADIPOQ
1.00E+00	3.07	PPARG
1.00E+00	3.07	SLC4A1
1.00E+00	3.04	PPARG
1.00E+00	3.04	NR0B1
1.00E+00	3.04	RELN
1.00E+00	3.04	SLC4A1
1.00E+00	1.51	ADIPOQ, LTBP1, SIGLEC16, SSC5D
1.00E+00	3.01	ITGA2
1.00E+00	3.01	COL2A1
1.00E+00	3.01	ADIPOQ
1.00E+00	3.01	H3-3A
1.00E+00	3.01	RELN
1.00E+00	2.98	STEAP3
1.00E+00	2.98	SLC4A1
1.00E+00	2.98	SLC4A1
1.00E+00	2.98	PPARG
1.00E+00	2.98	SLC4A1
1.00E+00	2.98	RELN
1.00E+00	2.98	PHLDB2
1.00E+00	2.95	ADIPOQ
1.00E+00	2.95	ADIPOQ
1.00E+00	2.95	UBA52
1.00E+00	1.89	COL1A1, ITGA2
1.00E+00	2.92	THBS4
1.00E+00	2.92	SPTA1
1.00E+00	1.48	ADIPOQ, LTBP1, SIGLEC16, SSC5D
1.00E+00	2.89	SPTLC3
1.00E+00	2.89	ADIPOQ
1.00E+00	2.89	SLC4A1
1.00E+00	1.26	ACAN, CELSR2, COL2A1, GNB4, LAMA2, NR0B1, PCDHGB7, RELN, TNC, VCAN
1.00E+00	2.87	SLC4A1
1.00E+00	2.87	RELN
1.00E+00	2.87	PHLDB2
1.00E+00	2.87	COL1A1
1.00E+00	2.87	SLC4A1
1.00E+00	2.87	ADIPOQ
1.00E+00	2.87	ADIPOQ
1.00E+00	2.87	ADIPOQ
1.00E+00	2.87	ACTG2
1.00E+00	2.87	NR0B1
1.00E+00	2.87	PLA2G7
1.00E+00	2.84	PHLDB2
1.00E+00	2.84	ADIPOQ

1.00E+00	2.84	PHLDB2
1.00E+00	2.84	PPARG
1.00E+00	2.84	SNX14
1.00E+00	1.84	ADIPOQ, PLA2G7
1.00E+00	1.84	COL1A1, TMEM198
1.00E+00	1.35	ADIPOQ, COL1A1, LAMA2, PHLDB2, PPARG, RELN
1.00E+00	2.81	ADIPOQ
1.00E+00	2.81	NR0B1
1.00E+00	1.83	ADIPOQ, PPARG
1.00E+00	1.83	LTBP1, PPARG
1.00E+00	2.79	RELN
1.00E+00	2.79	SPTLC3
1.00E+00	2.79	PPARG
1.00E+00	1.39	ADIPOQ, MARCO, PPARG, RELN, THBS4
1.00E+00	1.82	ADIPOQ, MARCO
1.00E+00	1.82	H3-3A, H4C1
1.00E+00	1.57	H3-3A, NR0B1, SPATA48
1.00E+00	2.76	CMTM5
1.00E+00	2.76	ADIPOQ
1.00E+00	1.28	COLEC11, MARCO, MASP1, MASP2, MBL2, PIGR, PPARG, SSC5D
1.00E+00	1.56	ADIPOQ, NR0B1, PPARG
1.00E+00	2.74	ITGA2
1.00E+00	2.74	PPARG
1.00E+00	2.74	DCDC1
1.00E+00	1.56	H3-3A, PPARG, SVEP1
1.00E+00	1.79	GP9, PKD2L1
1.00E+00	2.71	MBL2
1.00E+00	2.71	SLC4A1
1.00E+00	2.71	SLC4A1
1.00E+00	2.71	DPP6
1.00E+00	2.71	COL1A1
1.00E+00	2.71	ADIPOQ
1.00E+00	2.69	THBS4
1.00E+00	2.69	RELN
1.00E+00	2.69	H4C1
1.00E+00	2.69	TGM3
1.00E+00	2.69	TGM3
1.00E+00	2.69	PPARG
1.00E+00	2.69	UBA52
1.00E+00	2.69	ITGA2
1.00E+00	1.78	COL6A3, LAMA2
1.00E+00	1.16	ADIPOQ, CELSR2, CMTM5, COL1A1, COL1A2, COL2A1, COLEC11, DCDC1, EFEMP1, GNB
1.00E+00	2.67	TM4SF1
1.00E+00	2.67	PPARG
1.00E+00	2.67	NR0B1
1.00E+00	2.67	MBL2
1.00E+00	2.67	ADIPOQ

1.00E+00	1.22	ADIPOQ, COL1A1, COL1A2, COL2A1, COL6A1, ITGA2, LTBP1, MXRA5, NR0B1, PPARG, S
1.00E+00	1.77	PPARG, THBS4
1.00E+00	1.29	ADIPOQ, COL1A1, COL1A2, COL6A1, ITGA2, PKD2L1, PPARG
1.00E+00	1.77	NR0B1, PPARG
1.00E+00	1.25	DPP6, EFEMP1, EPB42, HGFAC, LTBP1, MASP1, MASP2, RELN, TGM3
1.00E+00	2.64	RELN
1.00E+00	1.53	PPARG, TGFBI, THBS4
1.00E+00	1.76	ITGA2, RELN
1.00E+00	1.76	COL6A3, COL7A1
1.00E+00	1.53	COL1A1, ITGA2, NR0B1
1.00E+00	2.62	SLC4A1
1.00E+00	2.62	CDCA2
1.00E+00	2.62	SPTLC3
1.00E+00	2.62	PPARG
1.00E+00	2.62	H3-3A
1.00E+00	1.36	ADIPOQ, COL1A1, ITGA2, PKD2L1, PPARG
1.00E+00	2.60	PHLDB2
1.00E+00	2.60	PKD2L1
1.00E+00	1.74	PPARG, THBS4
1.00E+00	1.52	H3-3A, NR0B1, SPATA48
1.00E+00	2.58	RELN
1.00E+00	2.58	PPARG
1.00E+00	2.58	COL1A1
1.00E+00	2.58	PPARG
1.00E+00	1.73	CDCA2, ITGA2
1.00E+00	2.55	PPARG
1.00E+00	2.55	ADIPOQ
1.00E+00	1.51	ADIPOQ, PPARG, SLC4A1
1.00E+00	1.72	PPARG, STEAP3
1.00E+00	2.53	ADIPOQ
1.00E+00	2.53	PPARG
1.00E+00	2.53	VCAN
1.00E+00	2.53	ADIPOQ
1.00E+00	2.53	PKD2L1
1.00E+00	1.72	ADIPOQ, TNC
1.00E+00	1.72	CELSR2, RELN
1.00E+00	1.72	PPARG, STEAP3
1.00E+00	2.51	TNC
1.00E+00	2.51	COL1A2
1.00E+00	2.51	PPARG
1.00E+00	2.51	PLA2G7
1.00E+00	2.49	COL2A1
1.00E+00	2.49	GP9
1.00E+00	2.49	RELN
1.00E+00	1.15	ADIPOQ, CELSR2, CMTM5, COL1A1, COL1A2, COL2A1, COLEC11, DCDC1, EFEMP1, GNB
1.00E+00	1.33	DPP6, GP9, PKD2L1, RELN, SLC4A1
1.00E+00	2.47	PHLDB2

1.00E+00	2.47	COLEC11
1.00E+00	2.47	PPARG
1.00E+00	2.47	GP9
1.00E+00	1.33	ADIPOQ, EFEMP1, PPARG, SSC5D, THBS4
1.00E+00	1.48	CELSR2, LAMA2, RELN
1.00E+00	1.69	ITGA2, PPARG
1.00E+00	1.68	MASP2, MBL2
1.00E+00	1.68	SPTA1, SPTB
1.00E+00	2.45	ADIPOQ
1.00E+00	2.45	SLC4A1
1.00E+00	1.68	ITGA2, PPARG
1.00E+00	2.43	GP9
1.00E+00	2.43	SPTLC3
1.00E+00	2.41	ADIPOQ
1.00E+00	1.67	ADIPOQ, SSC5D
1.00E+00	1.23	ADIPOQ, CLTCL1, CMTM6, COL1A1, HNRNPA1L2, PPARG, SNX14, STEAP3
1.00E+00	2.39	PPARG
1.00E+00	2.39	ADIPOQ
1.00E+00	2.39	CELSR2
1.00E+00	1.66	H3-3A, H4C1
1.00E+00	1.66	COL6A3, COL7A1
1.00E+00	1.66	ADIPOQ, PPARG
1.00E+00	1.14	ACTG2, ADIPOQ, CDCA2, CLTCL1, COL1A1, COLEC11, GP9, H3-3A, ITGA2, LAMA2, MAR
1.00E+00	2.38	RELN
1.00E+00	2.38	COL1A1
1.00E+00	2.38	HNRNPA1L2
1.00E+00	2.38	THBS4
1.00E+00	2.38	RELN
1.00E+00	2.38	THBS4
1.00E+00	2.38	SLC4A1
1.00E+00	2.38	PLA2G7
1.00E+00	1.36	DPP6, GP9, PKD2L1, STEAP3
1.00E+00	1.46	CDCA2, DCDC1, THBS4
1.00E+00	1.65	MASP2, MBL2
1.00E+00	2.36	GP9
1.00E+00	2.36	ITGA2
1.00E+00	1.45	CELSR2, LAMA2, RELN
1.00E+00	2.34	SLC4A1
1.00E+00	2.34	CDCA2
1.00E+00	2.34	CLTCL1
1.00E+00	1.63	COLEC11, MBL2
1.00E+00	1.63	DPP6, PKD2L1
1.00E+00	2.32	GP9
1.00E+00	2.32	ADIPOQ
1.00E+00	2.32	COL7A1
1.00E+00	1.43	CELSR2, LAMA2, RELN
1.00E+00	2.30	SLC4A1

1.00E+00	2.30	VCAN
1.00E+00	1.61	NR0B1, RELN
1.00E+00	2.29	NR0B1
1.00E+00	2.29	PKD2L1
1.00E+00	1.14	CELSR2, CMTM5, CMTM6, DPP6, GP9, GYPA, ITGA2, MARCO, PCDHGB7, PIGR, PKD2L1
1.00E+00	1.23	ADIPOQ, C1orf56, ITGA2, PPARG, SPTA1, THBS4, TNC
1.00E+00	2.27	ITGA2
1.00E+00	2.27	NR0B1
1.00E+00	1.60	CLTCL1, TGFBI
1.00E+00	2.25	PHLDB2
1.00E+00	2.25	PPARG
1.00E+00	1.42	COL1A1, COL2A1, TM4SF1
1.00E+00	1.33	DPP6, GP9, PKD2L1, SLC4A1
1.00E+00	1.59	H3-3A, H4C1
1.00E+00	1.33	ADIPOQ, COL1A1, ITGA2, NR0B1
1.00E+00	2.24	ITGA2
1.00E+00	2.24	EFEMP1
1.00E+00	2.22	H3-3A
1.00E+00	2.20	COL1A2
1.00E+00	2.20	PPARG
1.00E+00	2.20	EFEMP1
1.00E+00	2.20	DPP6
1.00E+00	1.27	DPP6, GP9, PKD2L1, RELN, STEAP3
1.00E+00	1.57	DPP6, RELN
1.00E+00	2.19	ITGA2
1.00E+00	2.19	THBS4
1.00E+00	2.19	PPARG
1.00E+00	1.56	ADIPOQ, PPARG
1.00E+00	1.56	COL6A3, COL7A1
1.00E+00	2.17	COL2A1
1.00E+00	2.17	COL2A1
1.00E+00	2.17	ADIPOQ
1.00E+00	1.39	CELSR2, LAMA2, RELN
1.00E+00	2.16	ITGA2
1.00E+00	2.16	SPTLC3
1.00E+00	2.16	PPARG
1.00E+00	2.16	SLC4A1
1.00E+00	1.31	DPP6, GP9, PKD2L1, RELN
1.00E+00	2.14	PKD2L1
1.00E+00	2.14	ITGA2
1.00E+00	2.14	LTBP1
1.00E+00	1.54	ADIPOQ, PPARG
1.00E+00	1.38	ADIPOQ, PPARG, RELN
1.00E+00	2.13	RELN
1.00E+00	2.13	CDCA2
1.00E+00	2.13	RELN
1.00E+00	2.13	DPP6

1.00E+00	1.30	ADIPOQ, CELSR2, PPARG, STEAP3
1.00E+00	1.14	CDCA2, CELSR2, EPB42, H3-3A, H4C1, KRT32, KRT85, KRT86, PHLDB2, PPARG, RELN, SP
1.00E+00	2.11	SPTA1
1.00E+00	2.11	ADIPOQ
1.00E+00	1.13	ACAN, ACTG2, ADIPOQ, CDCA2, CLTCL1, CMTM6, COL1A1, COL1A2, COL2A1, COL6A1,
1.00E+00	1.37	COL1A1, COL2A1, TM4SF1
1.00E+00	2.10	H3-3A
1.00E+00	2.10	ADIPOQ
1.00E+00	2.10	ADIPOQ
1.00E+00	1.52	DPP6, PKD2L1
1.00E+00	2.08	SLC4A1
1.00E+00	2.08	PIGR
1.00E+00	1.29	H3-3A, NR0B1, SPATA48, TNC
1.00E+00	1.29	H3-3A, PHLDB2, SPTA1, SPTB
1.00E+00	2.07	SPTA1
1.00E+00	2.07	SPTLC3
1.00E+00	2.07	ADIPOQ
1.00E+00	2.07	SPTLC3
1.00E+00	1.51	H3-3A, LAMA2
1.00E+00	2.05	PPARG
1.00E+00	2.05	RELN
1.00E+00	2.05	PPARG
1.00E+00	2.05	PPARG
1.00E+00	2.05	CMTM6
1.00E+00	2.04	PPARG
1.00E+00	2.04	ADIPOQ
1.00E+00	1.28	CELSR2, LAMA2, RELN, TNC
1.00E+00	1.49	ADIPOQ, H4C1
1.00E+00	2.03	ADIPOQ
1.00E+00	2.03	PLA2G7
1.00E+00	1.49	PPARG, THBS4
1.00E+00	1.34	H4C1, NR0B1, PPARG
1.00E+00	1.33	ADIPOQ, MARCO, PPARG
1.00E+00	1.99	SLC4A1
1.00E+00	1.99	PHLDB2
1.00E+00	1.99	COL2A1
1.00E+00	1.99	SNX14
1.00E+00	1.20	ADIPOQ, COL1A1, ITGA2, PPARG, RELN, SIGLEC16
1.00E+00	1.40	ACAN, ACTG2, ADIPOQ, C1orf56, CDCA2, CDSN, CELSR2, CLTCL1, CMTM5, CMTM6, CO
1.00E+00	1.47	ADIPOQ, RELN
1.00E+00	1.97	PKD2L1
1.00E+00	1.46	ACTG2, PHLDB2
1.00E+00	1.96	RELN
1.00E+00	1.46	H3-3A, TNC
1.00E+00	1.32	PPARG, TGFBI, THBS4
1.00E+00	1.95	THBS4
1.00E+00	1.95	PKD2L1

1.00E+00	1.25	ITGA2, SPTA1, THBS4, TNC
1.00E+00	1.17	ADIPOQ, EFEMP1, MARCO, PPARG, RELN, SLC4A1, THBS4
1.00E+00	1.94	H3-3A
1.00E+00	1.94	PPARG
1.00E+00	1.94	EFEMP1
1.00E+00	1.94	SLC4A1
1.00E+00	1.45	CLTCL1, DCDC1
1.00E+00	1.92	H3-3A
1.00E+00	1.92	PHLDB2
1.00E+00	1.92	LAMA2
1.00E+00	1.92	ADIPOQ
1.00E+00	1.92	CMTM6
1.00E+00	1.91	UBA52
1.00E+00	1.91	PLA2G7
1.00E+00	1.43	ITGA2, PHLDB2
1.00E+00	1.90	MBL2
1.00E+00	1.43	RELN, TNC
1.00E+00	1.89	SPTLC3
1.00E+00	1.89	THBS4
1.00E+00	1.89	H3-3A
1.00E+00	1.89	HNRNPA1L2
1.00E+00	1.89	HNRNPA1L2
1.00E+00	1.18	ADIPOQ, EFEMP1, MARCO, PPARG, RELN, THBS4
1.00E+00	1.17	ADIPOQ, CDCA2, MARCO, PPARG, RELN, THBS4
1.00E+00	1.88	ADIPOQ
1.00E+00	1.88	SLC4A1
1.00E+00	1.88	PHLDB2
1.00E+00	1.88	H3-3A
1.00E+00	1.17	DPP6, GP9, PKD2L1, RELN, SLC4A1, STEAP3
1.00E+00	1.13	ADIPOQ, COL1A1, COL1A2, COL2A1, COL6A1, ITGA2, LTBP1, NR0B1, PPARG
1.00E+00	1.13	ADIPOQ, CLTCL1, CMTM6, COL1A1, HNRNPA1L2, PPARG, SLC4A1, SNX14, STEAP3
1.00E+00	1.85	HNRNPA1L2
1.00E+00	1.85	RELN
1.00E+00	1.85	ACTG2
1.00E+00	1.85	PIGR
1.00E+00	1.41	LAMA2, RELN
1.00E+00	1.84	H3-3A
1.00E+00	1.84	PPARG
1.00E+00	1.84	SPTA1
1.00E+00	1.40	SPTA1, SPTB
1.00E+00	1.40	GP9, PKD2L1
1.00E+00	1.28	CELSR2, LAMA2, RELN
1.00E+00	1.22	ADIPOQ, PLA2G7, PPARG, SPTLC3
1.00E+00	1.27	ADIPOQ, LAMA2, RELN
1.00E+00	1.27	ADIPOQ, LAMA2, RELN
1.00E+00	1.81	ITGA2
1.00E+00	1.81	ITGA2

1.00E+00	1.80	CLTCL1
1.00E+00	1.80	RELN
1.00E+00	1.80	THBS4
1.00E+00	1.26	PHLDB2, SPTA1, SPTB
1.00E+00	1.79	PHLDB2
1.00E+00	1.26	ADIPOQ, LAMA2, RELN
1.00E+00	1.37	ADIPOQ, PPARG
1.00E+00	1.37	COL2A1, EFEMP1
1.00E+00	1.20	COL6A3, COL7A1, ITGA2, PPARG
1.00E+00	1.77	ITGA2
1.00E+00	1.77	PPARG
1.00E+00	1.77	RELN
1.00E+00	1.77	PPARG
1.00E+00	1.77	H3-3A
1.00E+00	1.17	ADIPOQ, COL2A1, LTBP1, NR0B1, PPARG
1.00E+00	1.76	ADIPOQ
1.00E+00	1.76	PPARG
1.00E+00	1.75	ITGA2
1.00E+00	1.75	SIGLEC16
1.00E+00	1.75	SIGLEC16
1.00E+00	1.75	PPARG
1.00E+00	1.75	UBA52
1.00E+00	1.10	ADIPOQ, CDCA2, EFEMP1, ITGA2, MARCO, PLA2G7, PPARG, RELN, SLC4A1, THBS4
1.00E+00	1.35	ITGA2, PPARG
1.00E+00	1.74	PPARG
1.00E+00	1.73	ADIPOQ
1.00E+00	1.73	NR0B1
1.00E+00	1.73	RELN
1.00E+00	1.73	RELN
1.00E+00	1.73	COL2A1
1.00E+00	1.73	COL2A1
1.00E+00	1.09	ADIPOQ, COL1A1, COL1A2, COL2A1, COL6A1, ITGA2, LTBP1, NR0B1, PLA2G7, PPARG, T
1.00E+00	1.71	SNX14
1.00E+00	1.23	COL6A3, COL7A1, PPARG
1.00E+00	1.70	PPARG
1.00E+00	1.33	MASP2, MBL2
1.00E+00	1.69	SPTA1
1.00E+00	1.69	NR0B1
1.00E+00	1.69	RELN
1.00E+00	1.69	GP9
1.00E+00	1.68	ADIPOQ
1.00E+00	1.68	PPARG
1.00E+00	1.68	CMTM6
1.00E+00	1.68	DCDC1
1.00E+00	1.09	ADIPOQ, CDCA2, EFEMP1, ITGA2, MARCO, PLA2G7, PPARG, RELN, SLC4A1, THBS4
1.00E+00	1.22	ADIPOQ, LAMA2, RELN
1.00E+00	1.07	ADIPOQ, CDSN, CMTM5, COL1A1, COL2A1, EFEMP1, GP9, H3-3A, H4C1, LTBP1, NR0B1,

1.00E+00	1.32	GP9, SPTA1
1.00E+00	1.67	ADIPOQ
1.00E+00	1.67	ADIPOQ
1.00E+00	1.67	PKD2L1
1.00E+00	1.06	ADIPOQ, CELSR2, CMTM5, COL1A1, COL1A2, COL2A1, COL6A1, COLEC11, DCDC1, EFEN
1.00E+00	1.31	ADIPOQ, PPARG
1.00E+00	1.65	SLC4A1
1.00E+00	1.65	RELN
1.00E+00	1.12	COL1A2, H3-3A, H4C1, PKD2L1, SPTA1, SPTB
1.00E+00	1.64	DCDC1
1.00E+00	1.30	CELSR2, RELN
1.00E+00	1.21	H3-3A, NR0B1, SPATA48
1.00E+00	1.05	ACAN, ACTG2, ADIPOQ, CDCA2, CDSN, CELSR2, COL1A1, COL2A1, COL6A3, COL7A1, CC
1.00E+00	1.63	ADIPOQ
1.00E+00	1.63	UBA52
1.00E+00	1.30	PLA2G7, SNX14
1.00E+00	1.63	H3-3A
1.00E+00	1.63	ADIPOQ
1.00E+00	1.63	ADIPOQ
1.00E+00	1.63	ADIPOQ
1.00E+00	1.29	NR0B1, PPARG
1.00E+00	1.62	NR0B1
1.00E+00	1.62	PPARG
1.00E+00	1.62	PKD2L1
1.00E+00	1.62	SLC4A1
1.00E+00	1.29	PIGR, PKD2L1
1.00E+00	1.61	NR0B1
1.00E+00	1.60	TNC
1.00E+00	1.28	PPARG, THBS4
1.00E+00	1.05	ADIPOQ, C1orf56, CDCA2, CDSN, CELSR2, CLTCL1, CMTM5, COL1A1, COL1A2, COL2A1,
1.00E+00	1.59	COL2A1
1.00E+00	1.06	ACAN, ACTG2, ADIPOQ, CDCA2, CLTCL1, CMTM6, COL1A1, COL1A2, COL2A1, COL6A1,
1.00E+00	1.58	ADIPOQ
1.00E+00	1.58	PPARG
1.00E+00	1.57	CELSR2
1.00E+00	1.57	HNRNPA1L2
1.00E+00	1.11	ITGA2, RELN, SNX14, SPTA1, VCAN
1.00E+00	1.26	ADIPOQ, SIGLEC16
1.00E+00	1.57	RELN
1.00E+00	1.57	H3-3A
1.00E+00	1.56	RELN
1.00E+00	1.56	RELN
1.00E+00	1.17	ADIPOQ, PPARG, RELN
1.00E+00	1.55	ADIPOQ
1.00E+00	1.55	CLTCL1
1.00E+00	1.55	CMTM6
1.00E+00	1.54	ADIPOQ

1.00E+00	1.25	ITGA2, PPARG
1.00E+00	1.24	H3-3A, TNC
1.00E+00	1.16	ADIPOQ, MARCO, PPARG
1.00E+00	1.53	H3-3A
1.00E+00	1.24	ADIPOQ, PPARG
1.00E+00	1.24	ADIPOQ, RELN
1.00E+00	1.52	HNRNPA1L2
1.00E+00	1.51	ADIPOQ
1.00E+00	1.51	ACTG2
1.00E+00	1.51	DCDC1
1.00E+00	1.07	ADIPOQ, CMTM5, COL6A3, COL7A1, DPP6, EFEMP1, THBS4
1.00E+00	1.23	GP9, SLC4A1
1.00E+00	1.50	COL1A1
1.00E+00	1.50	PHLDB2
1.00E+00	1.50	PHLDB2
1.00E+00	1.50	PPARG
1.00E+00	1.22	ADIPOQ, PPARG
1.00E+00	1.08	ADIPOQ, COL2A1, LTBP1, NR0B1, PHLDB2, PPARG
1.00E+00	1.49	PLA2G7
1.00E+00	1.48	RELN
1.00E+00	1.48	UBA52
1.00E+00	1.21	ADIPOQ, SLC4A1
1.00E+00	1.47	SVEP1
1.00E+00	1.21	COL1A2, RELN
1.00E+00	1.47	SPTLC3
1.00E+00	1.47	RELN
1.00E+00	1.47	NR0B1
1.00E+00	1.10	CELSR2, LAMA2, RELN, TNC
1.00E+00	1.08	ADIPOQ, NR0B1, PLA2G7, PPARG, SPTLC3
1.00E+00	1.20	SPTA1, SPTB
1.00E+00	1.45	CLTCL1
1.00E+00	1.45	ADIPOQ
1.00E+00	1.45	SPTA1
1.00E+00	1.08	ADIPOQ, COL2A1, LTBP1, NR0B1, PPARG
1.00E+00	1.13	ADIPOQ, PPARG, STEAP3
1.00E+00	1.43	SLC4A1
1.00E+00	1.19	GP9, SLC4A1
1.00E+00	1.19	ADIPOQ, RELN
1.00E+00	1.43	RELN
1.00E+00	1.12	PHLDB2, SPTA1, SPTB
1.00E+00	1.07	ADIPOQ, COL2A1, LTBP1, NR0B1, PPARG
1.00E+00	1.18	ADIPOQ, PPARG
1.00E+00	1.41	SLC4A1
1.00E+00	1.18	DPP6, RELN
1.00E+00	1.41	CELSR2
1.00E+00	1.02	ACTG2, CDCA2, CLTCL1, DCDC1, EPB42, H3-3A, H4C1, KRT24, KRT32, KRT85, KRT86, NF
1.00E+00	1.02	CELSR2, CMTM5, CMTM6, DPP6, GP9, GYPA, ITGA2, MARCO, PCDHGB7, PIGR, PKD2L1

1.00E+00	1.02	ACTG2, CDCA2, CLTCL1, DCDC1, EPB42, H3-3A, H4C1, KRT24, KRT32, KRT85, KRT86, NF
1.00E+00	1.11	PLA2G7, PPARG, SNX14
1.00E+00	1.16	ADIPOQ, PPARG
1.00E+00	1.39	COL2A1
1.00E+00	1.39	SPTLC3
1.00E+00	1.03	ADIPOQ, CDCA2, COL6A3, COL7A1, ITGA2, MARCO, PPARG, RELN, THBS4
1.00E+00	1.07	ADIPOQ, COL6A3, COL7A1, PPARG
1.00E+00	1.38	CLTCL1
1.00E+00	1.16	CDCA2, PPARG
1.00E+00	1.03	ACTG2, ITGA2, PKD2L1, RELN, SNX14, SPTA1, SPTB, VCAN
1.00E+00	1.37	HNRNPA1L2
1.00E+00	1.37	MARCO
1.00E+00	1.37	ACTG2
1.00E+00	1.02	ACTG2, CDCA2, CLTCL1, GNB4, GYPA, HGFAC, KRT24, KRT32, KRT85, KRT86, MASP1, PI
1.00E+00	1.10	DPP6, GP9, PKD2L1
1.00E+00	1.10	ADIPOQ, COL1A1, PPARG
1.00E+00	1.37	THBS4
1.00E+00	1.37	COL2A1
1.00E+00	1.15	ADIPOQ, PPARG
1.00E+00	1.36	ADIPOQ
1.00E+00	1.09	ADIPOQ, CELSR2, PPARG
1.00E+00	1.36	ADIPOQ
1.00E+00	1.36	GP9
1.00E+00	1.35	RELN
1.00E+00	1.06	COL2A1, PPARG, TGFBI, THBS4
1.00E+00	1.14	PIGR, PKD2L1
1.00E+00	1.34	ADIPOQ
1.00E+00	1.34	ADIPOQ
1.00E+00	1.34	SLC4A1
1.00E+00	1.01	ADIPOQ, CDCA2, CLTCL1, COL1A1, COLEC11, GP9, H3-3A, ITGA2, LAMA2, MARCO, MBI
1.00E+00	1.33	PPARG
1.00E+00	1.04	ACAN, ADIPOQ, C1orf56, CDCA2, CDSN, CELSR2, CLTCL1, CMTM5, CMTM6, COL1A1, C(
1.00E+00	1.33	COL1A1
1.00E+00	1.33	RELN
1.00E+00	1.02	ACAN, ACTG2, ADIPOQ, CDCA2, CELSR2, CLTCL1, CMTM6, COL1A1, COL1A2, COL2A1, (
1.00E+00	1.31	COL2A1
1.00E+00	1.31	SLC4A1
1.00E+00	1.12	ADIPOQ, PPARG
1.00E+00	1.30	PPARG
1.00E+00	1.30	UBA52
1.00E+00	1.29	UBA52
1.00E+00	1.29	HNRNPA1L2
1.00E+00	1.10	ADIPOQ, PPARG
1.00E+00	1.28	SPTA1
1.00E+00	1.02	H3-3A, ITGA2, NR0B1, SPATA48, TNC
1.00E+00	1.01	CDSN, ITGA2, LAMA2, PHLDB2, SPTA1, SPTB, TNC
1.00E+00	1.27	PLA2G7

1.00E+00	1.05	CMTM6, COL1A1, PIGR
1.00E+00	1.26	SLC4A1
1.00E+00	1.26	SLC4A1
1.00E+00	1.02	H3-3A, ITGA2, NR0B1, SPATA48, TNC
1.00E+00	1.26	SPTA1
1.00E+00	1.26	ADIPOQ
1.00E+00	1.09	COL1A1, PKD2L1
1.00E+00	1.00	CLTCL1, CMTM6, COL6A1, COL7A1, GNB4, MARCO, PIGR, PKD2L1, SNX14, SPTLC3, STE
1.00E+00	1.03	ADIPOQ, ITGA2, PPARG, RELN
1.00E+00	1.25	SPTLC3
1.00E+00	1.24	SLC4A1
1.00E+00	1.08	ACTG2, GNB4
1.00E+00	1.08	EFEMP1, LTBP1
1.00E+00	1.04	H3-3A, NR0B1, SPATA48
1.00E+00	1.08	ADIPOQ, PPARG
1.00E+00	1.24	SLC4A1
1.00E+00	1.24	ADIPOQ
1.00E+00	1.23	ADIPOQ
1.00E+00	1.23	MARCO
1.00E+00	1.23	SLC4A1
1.00E+00	1.23	PPARG
1.00E+00	1.03	ADIPOQ, PPARG, STEAP3
1.00E+00	1.22	SPTA1
1.00E+00	0.99	ADIPOQ, COL1A1, COL2A1, LAMA2, LTBP1, MARCO, NR0B1, PPARG, RELN, TMEM198
1.00E+00	1.07	COL2A1, EFEMP1
1.00E+00	1.21	PHLDB2
1.00E+00	1.21	H3-3A
1.00E+00	1.21	ADIPOQ
1.00E+00	1.02	H3-3A, NR0B1, SPATA48
1.00E+00	1.20	SLC4A1
1.00E+00	1.20	PKD2L1
1.00E+00	1.20	RELN
1.00E+00	1.20	ADIPOQ
1.00E+00	1.05	ADIPOQ, SPTLC3
1.00E+00	1.19	PPARG
1.00E+00	1.18	COL2A1
1.00E+00	1.17	CDCA2
1.00E+00	1.17	THBS4
1.00E+00	1.17	H3-3A
1.00E+00	1.04	ADIPOQ, RELN
1.00E+00	1.04	ITGA2, PPARG
1.00E+00	1.17	COL1A1
1.00E+00	1.00	ADIPOQ, PPARG, RELN
1.00E+00	0.98	CELSR2, ITGA2, LAMA2, RELN, TNC
1.00E+00	1.03	ADIPOQ, SLC4A1
1.00E+00	1.03	SLC4A1, SPTA1
1.00E+00	1.15	SLC4A1

1.00E+00	1.02	ADIPOQ, PPARG
1.00E+00	1.14	SLC4A1
1.00E+00	1.02	NR0B1, PPARG
1.00E+00	0.98	ITGA2, PLA2G7, SPTLC3, UBA52
1.00E+00	0.99	H3-3A, NR0B1, SPATA48
1.00E+00	1.14	SPTA1
1.00E+00	1.13	DCDC1
1.00E+00	0.98	ADIPOQ, PPARG, SLC4A1
1.00E+00	1.12	RELN
1.00E+00	1.12	H3-3A
1.00E+00	0.98	ITGA2, SPTLC3, UBA52
1.00E+00	0.98	ADIPOQ, COL1A1, PPARG
1.00E+00	1.11	SPTA1
1.00E+00	0.96	ADIPOQ, COL1A1, COL2A1, ITGA2, LAMA2, LTBP1, MARCO, NR0B1, PPARG, RELN, TME
1.00E+00	1.00	H3-3A, H4C1
1.00E+00	1.10	SNX14
1.00E+00	1.10	PPARG
1.00E+00	1.10	ITGA2
1.00E+00	1.10	SLC4A1
1.00E+00	0.99	ADIPOQ, PPARG
1.00E+00	1.09	RELN
1.00E+00	0.96	ADIPOQ, COL1A1, COL2A1, ITGA2, LAMA2, LTBP1, MARCO, NR0B1, PPARG, RELN, TME
1.00E+00	0.96	ADIPOQ, ITGA2, PPARG, RELN, SPTA1
1.00E+00	0.97	PPARG, TGFBI, THBS4
1.00E+00	1.09	RELN
1.00E+00	1.09	PPARG
1.00E+00	0.97	CDCA2, CLTCL1, DCDC1
1.00E+00	1.08	PPARG
1.00E+00	0.98	RELN, SNX14
1.00E+00	1.07	GP9
1.00E+00	0.98	ADIPOQ, PPARG
1.00E+00	0.98	RELN, SNX14
1.00E+00	1.07	PHLDB2
1.00E+00	0.95	CELSR2, GNB4, MARCO, PPARG
1.00E+00	0.98	H3-3A, H4C1
1.00E+00	0.98	H3-3A, TNC
1.00E+00	0.96	ADIPOQ, CELSR2, PPARG
1.00E+00	0.95	ADIPOQ, PPARG, SLC4A1
1.00E+00	1.06	ADIPOQ
1.00E+00	0.97	RELN, THBS4
1.00E+00	1.06	RELN
1.00E+00	1.06	MARCO
1.00E+00	0.94	ADIPOQ, EFEMP1, RELN, THBS4
1.00E+00	1.05	RELN
1.00E+00	0.95	ADIPOQ, PPARG, SLC4A1
1.00E+00	0.94	ITGA2, PLA2G7, UBA52
1.00E+00	0.94	ACTG2, ITGA2, PKD2L1, RELN, SNX14, SPTA1, VCAN

1.00E+00	1.04	PPARG
1.00E+00	0.95	ITGA2, SPTA1
1.00E+00	0.95	ACAN, ACTG2, ADIPOQ, CDCA2, CDSN, CELSR2, COL1A1, COL1A2, COL2A1, COL6A3, CC
1.00E+00	0.93	H3-3A, H4C1, NR0B1, PPARG
1.00E+00	1.02	UBA52
1.00E+00	1.01	SLC4A1
1.00E+00	1.00	PPARG
1.00E+00	1.00	MBL2
1.00E+00	1.00	SPTA1
1.00E+00	1.00	RELN
1.00E+00	1.00	MBL2
1.00E+00	1.00	COL1A1
1.00E+00	0.92	ACAN, CLTCL1, COL1A1, TGFBI, VCAN
1.00E+00	0.92	COLEC11, GNB4, HNRNPA1L2, MBL2, SPTLC3
1.00E+00	0.93	ACTG2, ADIPOQ, CDCA2, COL1A1, ITGA2, MARCO, PPARG, RELN, SIGLEC16, THBS4, TN
1.00E+00	0.99	ADIPOQ
1.00E+00	0.99	GP9
1.00E+00	0.92	ITGA2, RELN
1.00E+00	0.98	RELN
1.00E+00	0.98	PPARG
1.00E+00	0.91	CDSN, ITGA2, PHLDB2, TNC
1.00E+00	0.98	PLA2G7
1.00E+00	0.98	SPTA1
1.00E+00	0.97	RELN
1.00E+00	0.96	H3-3A
1.00E+00	0.96	SNX14
1.00E+00	0.96	ADIPOQ
1.00E+00	0.96	ADIPOQ
1.00E+00	0.96	UBA52
1.00E+00	0.91	ADIPOQ, CDSN, COL2A1, COLEC11, PPARG, STEAP3
1.00E+00	0.90	EFEMP1, LTBP1
1.00E+00	0.90	ADIPOQ, MARCO, RELN
1.00E+00	0.90	GP9, SLC4A1
1.00E+00	0.95	SPTA1
1.00E+00	0.94	NR0B1
1.00E+00	0.94	GP9
1.00E+00	0.94	PPARG
1.00E+00	0.94	PPARG
1.00E+00	0.94	CMTM6
1.00E+00	0.94	CDCA2
1.00E+00	0.89	ACTG2, CLTCL1, DCDC1, NR0B1
1.00E+00	0.93	PPARG
1.00E+00	0.93	HNRNPA1L2
1.00E+00	0.93	HNRNPA1L2
1.00E+00	0.92	SLC4A1
1.00E+00	0.91	HNRNPA1L2
1.00E+00	0.91	PHLDB2

1.00E+00	0.87	CMTM6, COL1A1, PIGR
1.00E+00	0.90	CELSR2, COL1A2, H3-3A, H4C1, ITGA2, PHLDB2, PKD2L1, SPTA1, SPTB
1.00E+00	0.93	ACAN, ACTG2, ADIPOQ, CDCA2, CDSN, CELSR2, COL1A1, COL2A1, COL6A3, COL7A1, CC
1.00E+00	0.91	ADIPOQ
1.00E+00	0.88	CELSR2, LAMA2, RELN, TNC
1.00E+00	0.89	EFEMP1
1.00E+00	0.86	ITGA2, PHLDB2, RELN
1.00E+00	0.89	PKD2L1
1.00E+00	0.88	ADIPOQ, COL6A3, COL7A1, ITGA2, PPARG, RELN, SLC4A1
1.00E+00	0.88	PKD2L1
1.00E+00	0.88	ACTG2, ADIPOQ, EPB42, PPARG, SIGLEC16, SNX14, SPTLC3
1.00E+00	0.88	PKD2L1
1.00E+00	0.87	SPTA1
1.00E+00	0.87	ITGA2
1.00E+00	0.84	DPP6, RELN
1.00E+00	0.86	COL1A2
1.00E+00	0.85	GP9
1.00E+00	0.83	GP9, SPTA1
1.00E+00	0.83	PKD2L1, VCAN
1.00E+00	0.83	EFEMP1, LTBP1
1.00E+00	0.84	SSC5D
1.00E+00	0.84	CELSR2
1.00E+00	0.82	NR0B1, SPTLC3
1.00E+00	0.82	CDCA2, DCDC1
1.00E+00	0.83	SPTLC3
1.00E+00	0.81	PKD2L1, SLC4A1
1.00E+00	0.81	MASP2, MBL2
1.00E+00	0.82	SLC4A1
1.00E+00	0.82	PPARG
1.00E+00	0.81	COL2A1
1.00E+00	0.83	CELSR2, LAMA2, RELN, TNC
1.00E+00	0.81	HNRNPA1L2, PPARG
1.00E+00	0.81	DCDC1
1.00E+00	0.81	SPTA1
1.00E+00	0.81	RELN
1.00E+00	0.81	SPTA1
1.00E+00	0.86	ADIPOQ, COL6A3, COL7A1, ITGA2, NR0B1, PPARG, RELN, SLC4A1, SPTA1
1.00E+00	0.79	CDCA2
1.00E+00	0.79	ADIPOQ
1.00E+00	0.78	EFEMP1
1.00E+00	0.78	ITGA2, UBA52
1.00E+00	0.78	PLA2G7
1.00E+00	0.78	GP9, SLC4A1
1.00E+00	0.78	CELSR2
1.00E+00	0.83	ADIPOQ, CDSN, COL2A1, COLEC11, PPARG, STEAP3
1.00E+00	0.77	EFEMP1
1.00E+00	0.81	CLTCL1, SPTLC3, TGFBI, UBA52

1.00E+00	0.77	TM4SF1
1.00E+00	0.77	COL2A1
1.00E+00	0.76	EFEMP1
1.00E+00	0.81	ADIPOQ, PLA2G7, PPARG, SLC4A1, SPTLC3
1.00E+00	0.76	PLA2G7
1.00E+00	0.76	SPTA1
1.00E+00	0.78	HNRNPA1L2, NR0B1, UBA52
1.00E+00	0.77	CDCA2, DCDC1
1.00E+00	0.76	ADIPOQ, PPARG
1.00E+00	0.80	CELSR2, LAMA2, RELN, TNC
1.00E+00	0.86	ACAN, CDCA2, COL1A1, COL1A2, COL2A1, COL6A1, COL6A3, COL7A1, GYPA, H3-3A, H4
1.00E+00	0.86	ACAN, CDCA2, COL1A1, COL1A2, COL2A1, COL6A1, COL6A3, COL7A1, GYPA, H3-3A, H4
1.00E+00	0.86	ACAN, CDCA2, COL1A1, COL1A2, COL2A1, COL6A1, COL6A3, COL7A1, GYPA, H3-3A, H4
1.00E+00	0.74	MBL2
1.00E+00	0.75	ITGA2, UBA52
1.00E+00	0.73	SPTA1
1.00E+00	0.73	CMTM6
1.00E+00	0.73	CELSR2
1.00E+00	0.73	SLC4A1
1.00E+00	0.80	CDCA2, H3-3A, H4C1, NR0B1, PPARG
1.00E+00	0.72	NR0B1
1.00E+00	0.72	GP9
1.00E+00	0.86	ACTG2, ADIPOQ, CDCA2, CELSR2, COL1A1, COL2A1, COL6A3, COL7A1, EFEMP1, H3-3A,
1.00E+00	0.78	ADIPOQ, CDSN, COL2A1, PPARG
1.00E+00	0.72	DPP6
1.00E+00	0.72	CLTCL1
1.00E+00	0.83	ACTG2, ADIPOQ, CDCA2, COL1A1, ITGA2, MARCO, PPARG, RELN, SIGLEC16, THBS4, TN
1.00E+00	0.77	ADIPOQ, NR0B1, PPARG, SLC4A1
1.00E+00	0.77	ADIPOQ, H3-3A, PPARG, SSC5D
1.00E+00	0.86	ACTG2, ADIPOQ, CELSR2, COL1A1, COL2A1, EFEMP1, GP9, H3-3A, HGFAC, HNRNPA1L2
1.00E+00	0.70	H3-3A
1.00E+00	0.70	LTBP1
1.00E+00	0.70	RELN
1.00E+00	0.69	PKD2L1
1.00E+00	0.81	CELSR2, COL1A2, H3-3A, H4C1, ITGA2, PHLDB2, PKD2L1, SPTA1, SPTB
1.00E+00	0.68	RELN
1.00E+00	0.68	PKD2L1
1.00E+00	0.71	RELN, SNX14
1.00E+00	0.68	SLC4A1
1.00E+00	0.77	ADIPOQ, COL2A1, COLEC11, PPARG, STEAP3
1.00E+00	0.67	SPTA1
1.00E+00	0.67	PPARG
1.00E+00	0.70	PKD2L1, SLC4A1
1.00E+00	0.81	ADIPOQ, CDCA2, EFEMP1, EPB42, MARCO, PPARG, RELN, TGM3, THBS4, UBA52
1.00E+00	0.66	RELN
1.00E+00	0.69	ADIPOQ, CLTCL1
1.00E+00	0.69	CELSR2, PPARG

1.00E+00	0.85	ACAN, ACTG2, ADIPOQ, CDCA2, CLTCL1, CMTM6, COL1A1, COL1A2, COL2A1, COL6A1,
1.00E+00	0.72	CLTCL1, PKD2L1, SLC4A1
1.00E+00	0.65	SLC4A1
1.00E+00	0.65	DCDC1
1.00E+00	0.69	EFEMP1, LTBP1
1.00E+00	0.64	HNRNPA1L2
1.00E+00	0.85	ACAN, ADIPOQ, CDCA2, CDSN, CELSR2, COL1A1, COL2A1, COL6A3, COL7A1, COLEC11,
1.00E+00	0.64	PPARG
1.00E+00	0.64	SLC4A1
1.00E+00	0.63	SLC4A1
1.00E+00	0.76	DPP6, HGFAC, MASP1, MASP2, PLA2G7, RELN
1.00E+00	0.63	ADIPOQ
1.00E+00	0.63	CDCA2
1.00E+00	0.62	ITGA2
1.00E+00	0.61	PKD2L1
1.00E+00	0.61	PKD2L1
1.00E+00	0.71	CELSR2, LAMA2, RELN, TNC
1.00E+00	0.61	MARCO
1.00E+00	0.82	ACTG2, ADIPOQ, CDCA2, CELSR2, COL1A1, COL2A1, COL6A3, COL7A1, EFEMP1, H3-3A,
1.00E+00	0.69	ADIPOQ, NR0B1, PPARG
1.00E+00	0.61	PHLDB2
1.00E+00	0.65	COL1A1, H4C1
1.00E+00	0.69	ADIPOQ, NR0B1, PPARG
1.00E+00	0.69	ADIPOQ, NR0B1, PPARG
1.00E+00	0.65	H3-3A, TNC
1.00E+00	0.80	ACTG2, ADIPOQ, CELSR2, COL1A1, COL2A1, EFEMP1, H3-3A, ITGA2, LTBP1, NR0B1, PP/
1.00E+00	0.65	CELSR2, PHLDB2
1.00E+00	0.60	CDSN
1.00E+00	0.59	HNRNPA1L2
1.00E+00	0.59	SLC4A1
1.00E+00	0.59	SLC4A1
1.00E+00	0.59	PPARG
1.00E+00	0.59	NR0B1
1.00E+00	0.70	ADIPOQ, CDSN, COL2A1, PPARG
1.00E+00	0.64	CDSN, COL2A1
1.00E+00	0.58	CDCA2
1.00E+00	0.58	ITGA2
1.00E+00	0.75	ADIPOQ, CDCA2, COL1A1, ITGA2, MARCO, PPARG, RELN, THBS4
1.00E+00	0.63	NR0B1, PPARG
1.00E+00	0.56	ADIPOQ
1.00E+00	0.56	PPARG
1.00E+00	0.74	ADIPOQ, CDCA2, COL1A1, ITGA2, MARCO, PPARG, RELN, THBS4
1.00E+00	0.55	SPTA1
1.00E+00	0.71	ACTG2, COLEC11, EPB42, MBL2, PPARG, SPTLC3
1.00E+00	0.55	ITGA2
1.00E+00	0.75	ADIPOQ, CDCA2, EFEMP1, EPB42, MARCO, PPARG, RELN, TGM3, THBS4, UBA52
1.00E+00	0.71	ADIPOQ, COL6A3, COL7A1, NR0B1, PPARG, SLC4A1

1.00E+00	0.67	ADIPOQ, MARCO, PPARG, RELN
1.00E+00	0.54	SNX14
1.00E+00	0.54	SNX14
1.00E+00	0.54	SPTLC3
1.00E+00	0.53	CELSR2
1.00E+00	0.52	SPTA1
1.00E+00	0.52	ITGA2
1.00E+00	0.63	ADIPOQ, NR0B1, PPARG
1.00E+00	0.52	CELSR2
1.00E+00	0.65	ITGA2, SPTA1, SPTLC3, UBA52
1.00E+00	0.58	PLA2G7, SLC4A1
1.00E+00	0.65	CDCA2, CLTCL1, DCDC1, STEAP3
1.00E+00	0.50	HNRNPA1L2
1.00E+00	0.57	CDSN, COL2A1
1.00E+00	0.50	SLC4A1
1.00E+00	0.50	COL2A1
1.00E+00	0.50	SLC4A1
1.00E+00	0.49	COL2A1
1.00E+00	0.49	PKD2L1
1.00E+00	0.65	EFEMP1, EPB42, LTBP1, SPTLC3, TGM3
1.00E+00	0.48	NR0B1
1.00E+00	0.55	ITGA2, PPARG
1.00E+00	0.48	PPARG
1.00E+00	0.47	PPARG
1.00E+00	0.47	PPARG
1.00E+00	0.59	ADIPOQ, COL2A1, PPARG
1.00E+00	0.47	PPARG
1.00E+00	0.47	PHLDB2
1.00E+00	0.47	RELN
1.00E+00	0.53	COL2A1, SLC4A1
1.00E+00	0.46	H3-3A
1.00E+00	0.65	ADIPOQ, COL1A2, DCDC1, MARCO, PPARG, RELN
1.00E+00	0.53	PKD2L1, SLC4A1
1.00E+00	0.46	PKD2L1
1.00E+00	0.45	SPTA1
1.00E+00	0.44	SLC4A1
1.00E+00	0.52	CDCA2, DCDC1
1.00E+00	0.51	GP9, SPTA1
1.00E+00	0.43	UBA52
1.00E+00	0.43	PPARG
1.00E+00	0.51	SPTLC3, UBA52
1.00E+00	0.56	ADIPOQ, CLTCL1, CMTM6
1.00E+00	0.51	SPTLC3, UBA52
1.00E+00	0.42	UBA52
1.00E+00	0.55	ADIPOQ, NR0B1, PPARG
1.00E+00	0.50	SPTLC3, UBA52
1.00E+00	0.41	ITGA2

1.00E+00	0.49	H3-3A, PPARG
1.00E+00	0.41	STEAP3
1.00E+00	0.64	ADIPOQ, COL6A3, COL7A1, H3-3A, NR0B1, PPARG, SLC4A1, SSC5D
1.00E+00	0.48	PKD2L1, SLC4A1
1.00E+00	0.40	UBA52
1.00E+00	0.48	ITGA2, UBA52
1.00E+00	0.48	H3-3A, PPARG
1.00E+00	0.52	ADIPOQ, NR0B1, PPARG
1.00E+00	0.39	PLA2G7
1.00E+00	0.56	COL1A1, COL1A2, PPARG, UBA52
1.00E+00	0.47	COL6A3, COL7A1
1.00E+00	0.47	CDCA2, DCDC1
1.00E+00	0.52	ADIPOQ, NR0B1, PPARG
1.00E+00	0.55	ADIPOQ, PLA2G7, SLC4A1, UBA52
1.00E+00	0.72	ACAN, ADIPOQ, CDCA2, CDSN, CELSR2, COL1A1, COL2A1, COL6A3, COL7A1, COLEC11,
1.00E+00	0.57	COLEC11, H3-3A, H4C1, NR0B1, PPARG
1.00E+00	0.38	UBA52
1.00E+00	0.54	ADIPOQ, PLA2G7, SNX14, UBA52
1.00E+00	0.37	UBA52
1.00E+00	0.45	HNRNPA1L2, PPARG
1.00E+00	0.67	ADIPOQ, CDCA2, CELSR2, COL1A1, COL6A3, COL7A1, EFEMP1, ITGA2, MARCO, NR0B1,
1.00E+00	0.60	ADIPOQ, COL6A3, COL7A1, H3-3A, NR0B1, PPARG, SSC5D
1.00E+00	0.36	PPARG
1.00E+00	0.56	ADIPOQ, PLA2G7, SLC4A1, SNX14, UBA52
1.00E+00	0.36	UBA52
1.00E+00	0.36	NR0B1
1.00E+00	0.44	ITGA2, LAMA2
1.00E+00	0.35	SPTLC3
1.00E+00	0.35	SPTA1
1.00E+00	0.35	NR0B1
1.00E+00	0.43	PLA2G7, UBA52
1.00E+00	0.63	ADIPOQ, CELSR2, COL1A1, EFEMP1, ITGA2, NR0B1, PPARG, RELN, SPTA1, SPTLC3, UBA
1.00E+00	0.58	ADIPOQ, CELSR2, COL1A1, EFEMP1, NR0B1, PPARG, RELN
1.00E+00	0.57	ADIPOQ, CELSR2, COL1A1, EFEMP1, NR0B1, PPARG, RELN
1.00E+00	0.57	ADIPOQ, CELSR2, COL1A1, EFEMP1, NR0B1, PPARG, RELN
1.00E+00	0.33	UBA52
1.00E+00	0.64	ADIPOQ, CDCA2, CELSR2, COL1A1, COL6A3, COL7A1, EFEMP1, ITGA2, MARCO, NR0B1,
1.00E+00	0.41	H3-3A, PPARG
1.00E+00	0.32	COL2A1
1.00E+00	0.32	CELSR2
1.00E+00	0.49	ADIPOQ, NR0B1, PPARG, SLC4A1
1.00E+00	0.45	COL1A1, ITGA2, PPARG
1.00E+00	0.40	ACTG2, EPB42
1.00E+00	0.67	ACAN, ADIPOQ, CDCA2, CLTCL1, CMTM6, COL1A1, COL1A2, COL2A1, COL6A1, COL6A3
1.00E+00	0.55	ADIPOQ, CELSR2, COL1A1, EFEMP1, NR0B1, PPARG, RELN
1.00E+00	0.55	ADIPOQ, CELSR2, COL1A1, EFEMP1, NR0B1, PPARG, RELN
1.00E+00	0.55	ADIPOQ, CELSR2, COL1A1, EFEMP1, NR0B1, PPARG, RELN

1.00E+00	0.44	H4C1, HNRNPA1L2, NR0B1
1.00E+00	0.44	PPARG, THBS4, TNC
1.00E+00	0.29	SLC4A1
1.00E+00	0.38	H3-3A, PPARG
1.00E+00	0.56	ADIPOQ, CELSR2, COL1A1, EFEMP1, ITGA2, NR0B1, PPARG, RELN
1.00E+00	0.38	H3-3A, PPARG
1.00E+00	0.38	ACTG2, EPB42
1.00E+00	0.29	SPTA1
1.00E+00	0.37	ACTG2, EPB42
1.00E+00	0.29	UBA52
1.00E+00	0.59	DPP6, EFEMP1, EPB42, HGFAC, LTBP1, MASP1, MASP2, PLA2G7, RELN, SPTLC3, STEAP3
1.00E+00	0.42	COL1A1, ITGA2, PPARG
1.00E+00	0.29	UBA52
1.00E+00	0.28	UBA52
1.00E+00	0.54	ADIPOQ, CELSR2, COL1A1, EFEMP1, NR0B1, PPARG, RELN, SLC4A1
1.00E+00	0.36	H3-3A, PPARG
1.00E+00	0.54	ADIPOQ, CELSR2, COL1A1, EFEMP1, NR0B1, PPARG, RELN, SPTA1
1.00E+00	0.28	ADIPOQ
1.00E+00	0.54	ADIPOQ, CELSR2, COL1A1, EFEMP1, NR0B1, PPARG, RELN, SPTA1
1.00E+00	0.41	COL1A1, ITGA2, PPARG
1.00E+00	0.52	ADIPOQ, CELSR2, COL1A1, EFEMP1, NR0B1, PPARG, RELN
1.00E+00	0.41	ADIPOQ, CLTCL1, CMTM6
1.00E+00	0.53	CDCA2, GYPA, H3-3A, H4C1, MASP1, NR0B1, PPARG, UBA52
1.00E+00	0.27	PPARG
1.00E+00	0.53	ADIPOQ, CELSR2, COL1A1, EFEMP1, ITGA2, NR0B1, PPARG, RELN
1.00E+00	0.34	H3-3A, PPARG
1.00E+00	0.34	H3-3A, PPARG
1.00E+00	0.34	COL1A1, PPARG
1.00E+00	0.34	COL1A1, PPARG
1.00E+00	0.34	COL1A1, PPARG
1.00E+00	0.52	ADIPOQ, CELSR2, COL1A1, EFEMP1, ITGA2, NR0B1, PPARG, RELN
1.00E+00	0.52	ADIPOQ, CELSR2, COL1A1, EFEMP1, NR0B1, PPARG, RELN, SPTA1
1.00E+00	0.56	ADIPOQ, CDCA2, CELSR2, COL1A1, EFEMP1, ITGA2, MARCO, NR0B1, PPARG, RELN, SLC
1.00E+00	0.23	PPARG
1.00E+00	0.32	ACTG2, EPB42
1.00E+00	0.48	ADIPOQ, CELSR2, COL1A1, EFEMP1, NR0B1, PPARG, RELN
1.00E+00	0.23	UBA52
1.00E+00	0.23	UBA52
1.00E+00	0.50	ADIPOQ, CELSR2, COL1A1, EFEMP1, ITGA2, NR0B1, PPARG, RELN, UBA52
1.00E+00	0.31	COL1A1, PPARG
1.00E+00	0.42	ADIPOQ, COL2A1, EPB42, ITGA2, UBA52
1.00E+00	0.30	ACTG2, EPB42
1.00E+00	0.30	ACTG2, EPB42
1.00E+00	0.30	ACTG2, EPB42
1.00E+00	0.29	NR0B1, PPARG
1.00E+00	0.21	PPARG
1.00E+00	0.21	UBA52

1.00E+00	0.20	PPARG
1.00E+00	0.49	ADIPOQ, CELSR2, COL1A1, EFEMP1, ITGA2, NR0B1, PPARG, RELN, SPTA1, SPTLC3, UBA
1.00E+00	0.28	COL1A1, PPARG
1.00E+00	0.20	HNRNPA1L2
1.00E+00	0.49	ADIPOQ, CELSR2, COL1A1, EFEMP1, ITGA2, NR0B1, PPARG, RELN, SPTA1, SPTLC3, UBA
1.00E+00	0.26	ACTG2, EPB42
1.00E+00	0.26	ACTG2, EPB42
1.00E+00	0.48	ADIPOQ, CELSR2, COL1A1, EFEMP1, ITGA2, NR0B1, PPARG, RELN, SPTA1, SPTLC3, UBA
1.00E+00	0.50	ADIPOQ, CELSR2, COL1A1, EFEMP1, HNRNPA1L2, ITGA2, NR0B1, PLA2G7, PPARG, RELN
1.00E+00	0.42	CDCA2, GYPA, H3-3A, H4C1, MASP1, NR0B1, PPARG, UBA52
1.00E+00	0.17	UBA52
1.00E+00	0.41	ADIPOQ, CELSR2, COL1A1, EFEMP1, HNRNPA1L2, NR0B1, PPARG, RELN
1.00E+00	0.37	COLEC11, H3-3A, H4C1, HNRNPA1L2, NR0B1, PPARG
1.00E+00	0.21	NR0B1, PPARG
1.00E+00	0.20	NR0B1, PPARG
1.00E+00	0.39	ADIPOQ, CELSR2, COL1A1, EFEMP1, HNRNPA1L2, NR0B1, PPARG, RELN, SLC4A1, SPTA1
1.00E+00	0.39	ADIPOQ, CELSR2, COL1A1, EFEMP1, HNRNPA1L2, NR0B1, PPARG, RELN, SLC4A1, SPTA1
1.00E+00	0.36	ADIPOQ, CELSR2, COL1A1, EFEMP1, HNRNPA1L2, NR0B1, PPARG, RELN
1.00E+00	0.36	ADIPOQ, CELSR2, COL1A1, EFEMP1, HNRNPA1L2, NR0B1, PPARG, RELN, SLC4A1
1.00E+00	0.37	ADIPOQ, CELSR2, COL1A1, EFEMP1, HNRNPA1L2, NR0B1, PPARG, RELN, SLC4A1, SPTA1
1.00E+00	0.35	ACTG2, COLEC11, EPB42, H3-3A, H4C1, HNRNPA1L2, NR0B1, PPARG, SPTLC3
1.00E+00	0.35	ACTG2, COLEC11, EPB42, H3-3A, H4C1, HNRNPA1L2, NR0B1, PPARG, SPTLC3
1.00E+00	0.42	ADIPOQ, CDCA2, CELSR2, COL1A1, COL2A1, EFEMP1, EPB42, HNRNPA1L2, ITGA2, MAR
1.00E+00	0.29	CDCA2, GYPA, H3-3A, H4C1, HNRNPA1L2, ITGA2, MASP1, NR0B1, PPARG, SVEP1, UBA5

1, EPB42, H3-3A, H4C1, KRT24, KRT32, KRT85, KRT86, LAMA2, LTBP1, MXRA5, SPTA1, SPTB, TGFBI, TGM

OL6A1, COL6A3, COL7A1, COLEC11, EFEMP1, GNB4, H3-3A, H4C1, HGFAC, KRT24, KRT32, KRT85, KRT86,
A1, COLEC11, EFEMP1, GNB4, H3-3A, H4C1, HGFAC, KRT24, KRT32, KRT85, KRT86, MASP1, MASP2, MBL2
OL6A1, COL6A3, COL7A1, COLEC11, DPP6, EFEMP1, EPB42, GNB4, GP9, GYPA, ITGA2, LAMA2, LTBP1, MA

EFEMP1, EPB42, ITGA2, LAMA2, NR0B1, PHLDB2, PPARG, RELN, SPTA1, SVEP1, TGFBI, TGM3, THBS4, TM
OL6A1, COL6A3, COL7A1, EFEMP1, EPB42, GNB4, GP9, H3-3A, H4C1, ITGA2, KRT32, KRT85, KRT86, LAMA
OL6A1, COL6A3, COL7A1, COLEC11, EFEMP1, EPB42, GNB4, GP9, H3-3A, H4C1, ITGA2, KRT32, KRT85, KRT

FEMP1, EPB42, GNB4, GP9, H3-3A, H4C1, HGFAC, ITGA2, KRT85, KRT86, LAMA2, LTBP1, MARCO, NR0B1

GNB4, GP9, H3-3A, H4C1, ITGA2, KRT85, KRT86, LAMA2, NR0B1, PHLDB2, PPARG, RELN, SLC4A1, SPTA1,

, GP9, H3-3A, H4C1, ITGA2, KRT32, KRT85, KRT86, LAMA2, NR0B1, PHLDB2, PPARG, RELN, SLC4A1, SPTA

, GP9, H3-3A, H4C1, ITGA2, KRT32, KRT85, KRT86, LAMA2, NR0B1, PHLDB2, PPARG, RELN, SLC4A1, SPTA

, H3-3A, H4C1, KRT24, KRT32, MARCO, MASP2, MXRA5, PIGR, PKD2L1, SLC4A1, SNX14, STEAP3, TGFBI, T

, GNB4, GP9, H3-3A, H4C1, ITGA2, LAMA2, NR0B1, PCDHGB7, PHLDB2, PPARG, RELN, SLC4A1, SPTA1, SV

C11, DPP6, GNB4, GP9, H3-3A, H4C1, HNRNPA1L2, ITGA2, LAMA2, LTBP1, MARCO, MBL2, NR0B1, PIGR, I

, GYPA, ITGA2, LAMA2, MARCO, MBL2, PCDHGB7, PHLDB2, PIGR, PKD2L1, RELN, SIGLEC16, SLC4A1, SPT.

A, H4C1, ITGA2, LAMA2, NR0B1, PCDHGB7, PPARG, RELN, SLC4A1, SPTA1, SVEP1, TGFBI, THBS4, TNC, VC

:OL6A1, COL6A3, COL7A1, DCDC1, EPB42, GNB4, GYPA, HGFAC, HNRNPA1L2, ITGA2, KRT24, KRT32, KRT

EPB42, GNB4, GP9, GYPA, H4C1, ITGA2, LAMA2, MARCO, MBL2, NR0B1, PCDHGB7, PHLDB2, PIGR, PKD2

2, KRT32, KRT85, KRT86, LAMA2, MARCO, PHLDB2, PKD2L1, PLA2G7, PPARG, RELN, SLC4A1, SNX14, SPT

ITGA2, LTBP1, MARCO, MBL2, NR0B1, PHLDB2, PIGR, PKD2L1, PPARG, RELN, SLC4A1, SNX14, SSC5D, STI

, ITGA2, LTBP1, MASP1, MASP2, PCDHGB7, PKD2L1, PPARG, RELN, SIGLEC16, SNX14, SPTA1, SPTLC3, STE

2, KRT32, KRT85, KRT86, LAMA2, MARCO, PHLDB2, PKD2L1, PLA2G7, PPARG, RELN, SLC4A1, SNX14, SPT

ƆL1A2, COL2A1, COL6A1, COL6A3, COL7A1, COLEC11, DCDC1, DPP6, EFEMP1, EPB42, GNB4, GP9, GYPA,

L1A1, COL1A2, COL2A1, COL6A1, COL6A3, COL7A1, COLEC11, DCDC1, DPP6, EFEMP1, EPB42, GNB4, GP

ᴾB42, GP9, HGFAC, ITGA2, MARCO, MASP1, MASP2, MBL2, PLA2G7, PPARG, RELN, TGM3, THBS4, UBA52

COL1A2, COL2A1, COL6A3, COL7A1, COLEC11, DCDC1, DPP6, EFEMP1, EPB42, GNB4, GP9, H3-3A, H4C1,

PB42, GP9, HGFAC, ITGA2, MARCO, MASP1, MASP2, MBL2, PLA2G7, PPARG, RELN, SLC4A1, SPTA1, SPTL(

MP1, GNB4, GP9, HGFAC, ITGA2, LAMA2, LTBP1, MARCO, MASP1, MASP2, MBL2, MXRA5, NR0B1, PHLDE

COL2A1, COL6A3, COL7A1, COLEC11, DCDC1, DPP6, EFEMP1, EPB42, GNB4, GP9, H3-3A, H4C1, ITGA2, LA

ͻL1A2, COL2A1, COL6A1, COL7A1, COLEC11, DCDC1, EFEMP1, EPB42, GNB4, GP9, GYPA, H3-3A, H4C1, H

COL6A1, COL7A1, COLEC11, DCDC1, EFEMP1, EPB42, GNB4, GP9, GYPA, H3-3A, H4C1, HGFAC, ITGA2, KF

L1A1, COL1A2, COL2A1, COL6A1, COL6A3, COL7A1, COLEC11, DCDC1, DPP6, EFEMP1, EPB42, GNB4, GP

;4, ITGA2, LAMA2, LTBP1, MARCO, MBL2, NR0B1, PIGR, PKD2L1, PPARG, RELN, SVEP1, THBS4, TMEM198

;4, ITGA2, LAMA2, LTBP1, MARCO, MBL2, NR0B1, PIGR, PKD2L1, PPARG, RELN, SVEP1, THBS4, TMEM198

4, ITGA2, LAMA2, LTBP1, MARCO, MBL2, NR0B1, PIGR, PKD2L1, PPARG, RELN, SVEP1, THBS4, TMEM19

CO, MASP1, MASP2, MBL2, PHLDB2, PLA2G7, PPARG, RELN, SIGLEC16, SPTA1, THBS4, TMEM198, TNC

COL6A3, COL7A1, DCDC1, EFEMP1, EPB42, GNB4, GYPA, H3-3A, H4C1, HGFAC, HNRNPA1L2, ITGA2, KRT2

L1A1, COL1A2, COL2A1, COL6A1, COL6A3, COL7A1, COLEC11, DCDC1, DPP6, EFEMP1, EPB42, GNB4, GP9

MP1, GNB4, ITGA2, LAMA2, LTBP1, MARCO, MBL2, NR0B1, PIGR, PKD2L1, PLA2G7, PPARG, RELN, SVEP1,

OLEC11, DPP6, EFEMP1, EPB42, GP9, H3-3A, HGFAC, HNRNPA1L2, ITGA2, LTBP1, MARCO, MASP1, MASP2

COLEC11, DCDC1, DPP6, EFEMP1, GNB4, GP9, H3-3A, H4C1, ITGA2, LAMA2, LTBP1, MARCO, MBL2, NR0

COL6A3, COL7A1, DCDC1, EPB42, GNB4, GYPA, H3-3A, H4C1, HGFAC, HNRNPA1L2, ITGA2, KRT24, KRT32

OL1A2, COL2A1, COL6A1, COL6A3, COL7A1, COLEC11, DCDC1, DPP6, EFEMP1, EPB42, GNB4, GP9, H3-3A

COL6A1, COL6A3, COL7A1, DCDC1, EPB42, GNB4, GYPA, H3-3A, H4C1, HGFAC, HNRNPA1L2, ITGA2, KRT2

)L7A1, COLEC11, DPP6, EFEMP1, EPB42, GP9, H3-3A, HGFAC, HNRNPA1L2, ITGA2, LTBP1, MARCO, MASF

)LEC11, DPP6, EFEMP1, EPB42, GP9, H3-3A, HGFAC, HNRNPA1L2, ITGA2, LTBP1, MARCO, MASP1, MASP:

COL6A3, COL7A1, EFEMP1, GNB4, GYPA, H3-3A, H4C1, HGFAC, HNRNPA1L2, ITGA2, KRT24, KRT32, LTBP

DPP6, EFEMP1, EPB42, GP9, HGFAC, HNRNPA1L2, ITGA2, MARCO, MASP1, MASP2, MBL2, NR0B1, PLA2(

DPP6, EFEMP1, EPB42, GP9, HGFAC, HNRNPA1L2, ITGA2, MARCO, MASP1, MASP2, MBL2, NR0B1, PLA2(

, COL7A1, GNB4, GYPA, H3-3A, H4C1, HGFAC, HNRNPA1L2, ITGA2, LTBP1, MARCO, MASP1, NR0B1, PIGR

, LAMA2, LTBP1, MASP1, MASP2, MBL2, MXRA5, PIGR, PLA2G7, RELN, SLC4A1, SSC5D, SVEP1, TGFBI, TG
2, MXRA5, PIGR, PLA2G7, RELN, SLC4A1, SSC5D, SVEP1, TGFBI, TGM3, THBS4, TNC, UBA52, VCAN
ARCO, MBL2, MXRA5, PCDHGB7, PHLDB2, PIGR, PKD2L1, RELN, SIGLEC16, SLC4A1, SPTA1, SPTB, SSC5D, S

A2, NR0B1, PCDHGB7, PHLDB2, PPARG, RELN, SLC4A1, SPTA1, SVEP1, TGFBI, TGM3, THBS4, TM4SF1, TNC
T86, LAMA2, NR0B1, PCDHGB7, PHLDB2, PPARG, RELN, SLC4A1, SPATA48, SPTA1, SVEP1, TGFBI, TGM3,

, PCDHGB7, PHLDB2, PIGR, PKD2L1, PLA2G7, PPARG, RELN, SIGLEC16, SLC4A1, SPATA48, SPTA1, SSC5D,

85, KRT86, LTBP1, MARCO, MASP1, NR0B1, PHLDB2, PIGR, PKD2L1, PLA2G7, PPARG, RELN, SLC4A1, SNX

L1, RELN, SIGLEC16, SLC4A1, SNX14, SPTA1, SPTB, SPTLC3, SSC5D, STEAP3, SVEP1, TGFBI, TGM3, TM4SF

H3-3A, H4C1, HGFAC, HNRNPA1L2, ITGA2, KRT24, KRT32, KRT85, KRT86, LAMA2, LTBP1, MARCO, MASF

9, GYPA, H3-3A, H4C1, HGFAC, HNRNPA1L2, ITGA2, KRT24, KRT32, KRT85, KRT86, LAMA2, LTBP1, MARC

HGFAC, ITGA2, LAMA2, LTBP1, MARCO, MASP1, MASP2, MBL2, NR0B1, PHLDB2, PIGR, PKD2L1, PLA2G7,

32, PIGR, PKD2L1, PLA2G7, PPARG, RELN, SIGLEC16, SLC4A1, SSC5D, SVEP1, TGFBI, THBS4, TMEM198, TN

MA2, LTBP1, MARCO, MASP1, MASP2, MBL2, NR0B1, PHLDB2, PIGR, PKD2L1, PLA2G7, PPARG, RELN, SI

IGFAC, HNRNPA1L2, ITGA2, KRT24, KRT32, KRT85, KRT86, LAMA2, LTBP1, MARCO, MASP1, MASP2, MBL

RT24, KRT32, KRT85, KRT86, LAMA2, LTBP1, MARCO, MASP1, MASP2, MBL2, NR0B1, PHLDB2, PKD2L1, P

), GYPA, H3-3A, H4C1, HGFAC, HNRNPA1L2, ITGA2, KRT24, KRT32, KRT85, KRT86, LAMA2, LTBP1, MARC

24, KRT32, KRT85, KRT86, LTBP1, MARCO, MASP1, MASP2, MXRA5, NR0B1, PHLDB2, PIGR, PKD2L1, PPA

9, GYPA, H3-3A, H4C1, HGFAC, HNRNPA1L2, ITGA2, KRT24, KRT32, KRT85, KRT86, LAMA2, LTBP1, MARC

2, MBL2, NR0B1, PLA2G7, PPARG, RELN, SIGLEC16, SSC5D, SVEP1, TGM3, THBS4, TNC, UBA52

B1, PHLDB2, PIGR, PKD2L1, PLA2G7, PPARG, RELN, SLC4A1, SPTA1, SPTB, SVEP1, TGFBI, THBS4, TMEM1

, KRT85, KRT86, LTBP1, MARCO, MASP1, NR0B1, PHLDB2, PIGR, PKD2L1, PPARG, SLC4A1, SNX14, SPTA1

, H4C1, HNRNPA1L2, ITGA2, KRT32, KRT85, KRT86, LAMA2, LTBP1, MARCO, MBL2, NR0B1, PCDHGB7, PI

4, KRT32, KRT85, KRT86, LTBP1, MARCO, MASP1, NR0B1, PHLDB2, PIGR, PKD2L1, PLA2G7, PPARG, RELN

?1, MASP2, MBL2, NR0B1, PLA2G7, PPARG, RELN, SIGLEC16, SLC4A1, SNX14, SPTA1, SPTLC3, SSC5D, SVE

2, MBL2, NR0B1, PLA2G7, PPARG, RELN, SIGLEC16, SLC4A1, SPTA1, SPTLC3, SSC5D, SVEP1, TGM3, THBS4

1, MARCO, MASP1, MASP2, MXRA5, NR0B1, PIGR, PKD2L1, PPARG, SLC4A1, SNX14, SPTLC3, STEAP3, SV

:, PKD2L1, PPARG, SNX14, SPTLC3, STEAP3, SVEP1, TGFBI, THBS4, TMEM198, TNC, UBA52, VCAN

14, SPTA1, SPTB, SPTLC3, SSC5D, STEAP3, SVEP1, TGFBI, TGM3, THBS4, TMEM198, TNC, UBA52, VCAN

P1, MASP2, MBL2, MXRA5, NR0B1, PCDHGB7, PHLDB2, PIGR, PKD2L1, PLA2G7, PPARG, RELN, SIGLEC16,

O, MASP1, MASP2, MBL2, MXRA5, NR0B1, PCDHGB7, PHLDB2, PIGR, PKD2L1, PLA2G7, PPARG, RELN, SI(

, PPARG, RELN, SIGLEC16, SLC4A1, SPTA1, SPTB, SSC5D, STEAP3, SVEP1, TGFBI, THBS4, TMEM198, TNC

.2, NR0B1, PCDHGB7, PHLDB2, PKD2L1, PLA2G7, PPARG, RELN, SIGLEC16, SLC4A1, SNX14, SPTA1, SPTB, S

PARG, RELN, SIGLEC16, SLC4A1, SPTA1, SPTB, SPTLC3, SSC5D, STEAP3, TGFBI, THBS4, TM4SF1, TNC, UBA

O, MASP1, MASP2, MBL2, MXRA5, NR0B1, PCDHGB7, PHLDB2, PIGR, PKD2L1, PLA2G7, PPARG, RELN, SI(

RG, SLC4A1, SNX14, SPTA1, SPTB, SPTLC3, STEAP3, SVEP1, TGFBI, TGM3, THBS4, TMEM198, TNC, UBA52

O, MASP1, MASP2, MBL2, MXRA5, NR0B1, PCDHGB7, PHLDB2, PIGR, PKD2L1, PLA2G7, PPARG, RELN, SI(

HLDB2, PIGR, PKD2L1, PLA2G7, PPARG, RELN, SIGLEC16, SLC4A1, SNX14, SPATA48, SPTA1, SPTB, SPTLC3,

, SLC4A1, SNX14, SPTA1, SPTB, SPTLC3, SSC5D, STEAP3, SVEP1, TGFBI, TGM3, THBS4, TMEM198, TNC, U

SLC4A1, SNX14, SPATA3, SPATA48, SPTA1, SPTB, SPTLC3, SSC5D, STEAP3, SVEP1, TGFBI, TGM3, THBS4, T

;LEC16, SLC4A1, SNX14, SPATA48, SPTA1, SPTB, SPTLC3, SSC5D, STEAP3, SVEP1, TGFBI, TGM3, THBS4, TI

GLEC16, SLC4A1, SNX14, SPATA3, SPATA48, SPTA1, SPTB, SPTLC3, SSC5D, STEAP3, SVEP1, TGFBI, TGM3,

GLEC16, SLC4A1, SNX14, SPTA1, SPTB, SPTLC3, SSC5D, STEAP3, SVEP1, TGFBI, TGM3, THBS4, TM4SF1, TM

THBS4, TM4SF1, TMEM198, TNC, UBA52, VCAN

Table 6. REACTOME pathways associated with non-T-cell proteins identified in CD3(+)sEV

REACTOME ID	REACTOME pathway	arbitrary category	# all protei
R-HSA-3000178	ECM proteoglycans	cancer-related	76
R-HSA-1474244	Extracellular matrix organization	cancer-related	300
R-HSA-216083	Integrin cell surface interactions	cancer-related	85
R-HSA-3000480	Scavenging by Class A Receptors	other	19
R-HSA-166662	Lectin pathway of complement activation	immune-related	8
R-HSA-2173782	Binding and Uptake of Ligands by Scavenger Receptor	cancer-related	42
R-HSA-8948216	Collagen chain trimerization	other	44
R-HSA-6806834	Signaling by MET	cancer-related	79
R-HSA-8874081	MET activates PTK2 signaling	cancer-related	30
R-HSA-166786	Creation of C4 and C2 activators	immune-related	14
R-HSA-3000171	Non-integrin membrane-ECM interactions	cancer-related	59
R-HSA-2022090	Assembly of collagen fibrils and other multimeric stru	cancer-related	61
R-HSA-75892	Platelet Adhesion to exposed collagen	cancer-related	16
R-HSA-1442490	Collagen degradation	cancer-related	64
R-HSA-1650814	Collagen biosynthesis and modifying enzymes	other	67
R-HSA-8875878	MET promotes cell motility	cancer-related	41
R-HSA-9845620	Enhanced binding of GP1BA variant to VWF multimer:	other	7
R-HSA-9846298	Defective binding of VWF variant to GPIb:IX:V	other	7
R-HSA-166663	Initial triggering of complement	immune-related	23
R-HSA-9823587	Defects of platelet adhesion to exposed collagen	other	8
R-HSA-1474290	Collagen formation	cancer-related	90
R-HSA-3000170	Syndecan interactions	cancer-related	27
R-HSA-1474228	Degradation of the extracellular matrix	cancer-related	140
R-HSA-375165	NCAM signaling for neurite out-growth	other	63
R-HSA-430116	GP1b-IX-V activation signalling	cancer-related	12
R-HSA-2214320	Anchoring fibril formation	other	15
R-HSA-9845622	Defective VWF binding to collagen type I	other	3
R-HSA-1266738	Developmental Biology	cancer-related	1271
R-HSA-9671793	Diseases of hemostasis	other	19
R-HSA-9845619	Enhanced cleavage of VWF variant by ADAMTS13	other	4
R-HSA-9845621	Defective VWF cleavage by ADAMTS13 variant	other	4
R-HSA-2855086	Ficolins bind to repetitive carbohydrate structures on	cancer-related	5
R-HSA-166658	Complement cascade	immune-related	58
R-HSA-186797	Signaling by PDGF	cancer-related	58
R-HSA-3000157	Laminin interactions	cancer-related	30
R-HSA-6809371	Formation of the cornified envelope	cancer-related	129
R-HSA-9006934	Signaling by Receptor Tyrosine Kinases	cancer-related	532
R-HSA-76009	Platelet Aggregation (Plug Formation)	cancer-related	39
R-HSA-419037	NCAM1 interactions	other	42
R-HSA-5653656	Vesicle-mediated transport	other	673
R-HSA-9675108	Nervous system development	other	578
R-HSA-977225	Amyloid fiber formation	other	110
R-HSA-2243919	Crosslinking of collagen fibrils	other	18

R-HSA-422475	Axon guidance	other	553
R-HSA-5619050	Defective SLC4A1 causes hereditary spherocytosis typ	other	1
R-HSA-9705671	SARS-CoV-2 activates/modulates innate and adaptive	other	127
R-HSA-6805567	Keratinization	cancer-related	215
R-HSA-445095	Interaction between L1 and Ankyrins	cancer-related	31
R-HSA-114604	GPVI-mediated activation cascade	cancer-related	35
R-HSA-170834	Signaling by TGF-beta Receptor Complex	cancer-related	94
R-HSA-2173796	SMAD2/SMAD3:SMAD4 heterotrimer regulates transc	other	36
R-HSA-2129379	Molecules associated with elastic fibres	cancer-related	37
R-HSA-9683683	Maturation of protein E	other	4
R-HSA-9694493	Maturation of protein E	other	4
R-HSA-3560782	Diseases associated with glycosaminoglycan metaboli	other	41
R-HSA-9821993	Replacement of protamines by nucleosomes in the ma	other	42
R-HSA-8957275	Post-translational protein phosphorylation	other	108
R-HSA-2559582	Senescence-Associated Secretory Phenotype (SASP)	other	110
R-HSA-1566948	Elastic fibre formation	cancer-related	44
R-HSA-9022707	MECP2 regulates transcription factors	other	5
R-HSA-8866376	Reelin signalling pathway	other	5
R-HSA-2173789	TGF-beta receptor signaling activates SMADs	immune-related	47
R-HSA-9705683	SARS-CoV-2-host interactions	other	204
R-HSA-373760	L1CAM interactions	cancer-related	119
R-HSA-6806942	MET Receptor Activation	other	6
R-HSA-9673221	Defective F9 activation	other	6
R-HSA-3000471	Scavenging by Class B Receptors	immune-related	6
R-HSA-2173793	Transcriptional activity of SMAD2/SMAD3:SMAD4 het	other	51
R-HSA-9006936	Signaling by TGFB family members	cancer-related	124
R-HSA-383280	Nuclear Receptor transcription pathway	other	52
R-HSA-381426	Regulation of Insulin-like Growth Factor (IGF) transpo	other	125
R-HSA-2559580	Oxidative Stress Induced Senescence	other	126
R-HSA-9706377	FLT3 signaling by CBL mutants	other	7
R-HSA-8939236	RUNX1 regulates transcription of genes involved in dif	cancer-related	129
R-HSA-198933	Immunoregulatory interactions between a Lymphoid a	immune-related	134
R-HSA-3595172	Defective CHST3 causes SEDCJD	other	8
R-HSA-3595174	Defective CHST14 causes EDS, musculocontractural ty	other	8
R-HSA-3595177	Defective CHSY1 causes TPBS	other	8
R-HSA-3656225	Defective CHST6 causes MCDC1	other	8
R-HSA-3656243	Defective ST3GAL3 causes MCT12 and EIEE15	other	8
R-HSA-3656244	Defective B4GALT1 causes B4GALT1-CDG (CDG-2d)	other	8
R-HSA-163680	AMPK inhibits chREBP transcriptional activation activi	other	8
R-HSA-917937	Iron uptake and transport	other	58
R-HSA-202733	Cell surface interactions at the vascular wall	immune-related	138
R-HSA-68867	Assembly of the pre-replicative complex	other	141
R-HSA-447041	CHL1 interactions	other	9
R-HSA-1253288	Downregulation of ERBB4 signaling	cancer-related	9
R-HSA-8849469	PTK6 Regulates RTKs and Their Effectors AKT1 and DO	other	9
R-HSA-9668250	Defective factor IX causes hemophilia B	other	9
R-HSA-3785653	Myoclonic epilepsy of Lafora	other	9

R-HSA-1247673	Erythrocytes take up oxygen and release carbon dioxi	other	9
R-HSA-8948747	Regulation of PTEN localization	other	9
R-HSA-73728	RNA Polymerase I Promoter Opening	other	62
R-HSA-5334118	DNA methylation	other	64
R-HSA-937042	IRAK2 mediated activation of TAK1 complex	other	10
R-HSA-8876493	InlA-mediated entry of Listeria monocytogenes into h	other	10
R-HSA-425381	Bicarbonate transporters	other	10
R-HSA-5625886	Activated PKN1 stimulates transcription of AR (androg	cancer-related	66
R-HSA-427359	SIRT1 negatively regulates rRNA expression	other	67
R-HSA-9670095	Inhibition of DNA recombination at telomere	other	67
R-HSA-68616	Assembly of the ORC complex at the origin of replicati	cancer-related	68
R-HSA-2022923	Dermatan sulfate biosynthesis	other	11
R-HSA-9014325	TICAM1,TRAF6-dependent induction of TAK1 complex	other	11
R-HSA-9645460	Alpha-protein kinase 1 signaling pathway	other	11
R-HSA-196025	Formation of annular gap junctions	other	11
R-HSA-9664873	Pexophagy	other	11
R-HSA-9708530	Regulation of BACH1 activity	cancer-related	11
R-HSA-199977	ER to Golgi Anterograde Transport	other	154
R-HSA-9821002	Chromatin modifications during the maternal to zygot	other	70
R-HSA-69002	DNA Replication Pre-Initiation	other	158
R-HSA-212300	PRC2 methylates histones and DNA	other	72
R-HSA-9710421	Defective pyroptosis	other	72
R-HSA-9013973	TICAM1-dependent activation of IRF3/IRF7	other	12
R-HSA-190873	Gap junction degradation	other	12
R-HSA-5689877	Josephin domain DUBs	other	12
R-HSA-2299718	Condensation of Prophase Chromosomes	other	73
R-HSA-76002	Platelet activation, signaling and aggregation	cancer-related	263
R-HSA-1358803	Downregulation of ERBB2:ERBB3 signaling	cancer-related	13
R-HSA-1237044	Erythrocytes take up carbon dioxide and release oxyg	other	13
R-HSA-1480926	O2/CO2 exchange in erythrocytes	other	13
R-HSA-209543	p75NTR recruits signalling complexes	other	13
R-HSA-209560	NF-kB is activated and signals survival	cancer-related	13
R-HSA-427389	ERCC6 (CSB) and EHMT2 (G9a) positively regulate rRN	other	76
R-HSA-5693565	Recruitment and ATM-mediated phosphorylation of r	cancer-related	77
R-HSA-109582	Hemostasis	cancer-related	623
R-HSA-5693606	DNA Double Strand Break Response	cancer-related	78
R-HSA-2024101	CS/DS degradation	other	14
R-HSA-3322077	Glycogen synthesis	other	14
R-HSA-975110	TRAF6 mediated IRF7 activation in TLR7/8 or 9 signali	other	14
R-HSA-9637628	Modulation by Mtb of host immune system	immune-related	14
R-HSA-9706369	Negative regulation of FLT3	cancer-related	15
R-HSA-2691230	Signaling by NOTCH1 HD Domain Mutants in Cancer	cancer-related	15
R-HSA-2691232	Constitutive Signaling by NOTCH1 HD Domain Mutant	cancer-related	15
R-HSA-2022857	Keratan sulfate degradation	other	15
R-HSA-3134975	Regulation of innate immune responses to cytosolic D	immune-related	15
R-HSA-937039	IRAK1 recruits IKK complex	immune-related	15
R-HSA-975144	IRAK1 recruits IKK complex upon TLR7/8 or 9 stimulat	immune-related	15

R-HSA-174490	Membrane binding and targetting of GAG proteins	other	15
R-HSA-174495	Synthesis And Processing Of GAG, GAGPOL Polyprotei	other	15
R-HSA-381340	Transcriptional regulation of white adipocyte differen	other	84
R-HSA-975163	IRAK2 mediated activation of TAK1 complex upon TLR	immune-related	16
R-HSA-8866427	VLDLR internalisation and degradation	other	16
R-HSA-1295596	Spry regulation of FGF signaling	other	16
R-HSA-8875360	InlB-mediated entry of Listeria monocytogenes into h	other	16
R-HSA-110312	Translesion synthesis by REV1	other	16
R-HSA-5684264	MAP3K8 (TPL2)-dependent MAPK1/3 activation	cancer-related	16
R-HSA-9651496	Defects of contact activation system (CAS) and kallikre	other	16
R-HSA-2173791	TGF-beta receptor signaling in EMT (epithelial to mese	cancer-related	16
R-HSA-205043	NRIF signals cell death from the nucleus	other	16
R-HSA-3229121	Glycogen storage diseases	other	16
R-HSA-193639	p75NTR signals via NF-kB	other	16
R-HSA-912446	Meiotic recombination	other	85
R-HSA-937072	TRAF6-mediated induction of TAK1 complex within TL	other	17
R-HSA-5655862	Translesion synthesis by POLK	other	17
R-HSA-5656121	Translesion synthesis by POLI	other	17
R-HSA-948021	Transport to the Golgi and subsequent modification	other	185
R-HSA-9616222	Transcriptional regulation of granulopoiesis	immune-related	89
R-HSA-69306	DNA Replication	other	186
R-HSA-73772	RNA Polymerase I Promoter Escape	other	90
R-HSA-201722	Formation of the beta-catenin:TCF transactivating cor	other	90
R-HSA-5250924	B-WICH complex positively regulates rRNA expression	other	90
R-HSA-936964	Activation of IRF3, IRF7 mediated by TBK1, IKBKE	other	18
R-HSA-8964315	G beta:gamma signalling through BTK	other	18
R-HSA-9758274	Regulation of NF-kappa B signaling	other	18
R-HSA-1912408	Pre-NOTCH Transcription and Translation	cancer-related	92
R-HSA-9694516	SARS-CoV-2 Infection	other	300
R-HSA-6804760	Regulation of TP53 Activity through Methylation	other	19
R-HSA-392851	Prostacyclin signalling through prostacyclin receptor	other	19
R-HSA-1236382	Constitutive Signaling by Ligand-Responsive EGFR Can	cancer-related	19
R-HSA-5637815	Signaling by Ligand-Responsive EGFR Variants in Cance	cancer-related	19
R-HSA-422085	Synthesis, secretion, and deacylation of Ghrelin	other	19
R-HSA-110320	Translesion Synthesis by POLH	other	19
R-HSA-5625740	RHO GTPases activate PKNs	cancer-related	94
R-HSA-8936459	RUNX1 regulates genes involved in megakaryocyte dif	cancer-related	94
R-HSA-446203	Asparagine N-linked glycosylation	cancer-related	304
R-HSA-3560783	Defective B4GALT7 causes EDS, progeroid type	other	20
R-HSA-3560801	Defective B3GAT3 causes JDSSDHD	other	20
R-HSA-4420332	Defective B3GALT6 causes EDSP2 and SEMDJL1	other	20
R-HSA-6803204	TP53 Regulates Transcription of Genes Involved in Cyt	other	20
R-HSA-2022870	Chondroitin sulfate biosynthesis	other	20
R-HSA-418217	G beta:gamma signalling through PLC beta	other	20
R-HSA-8964616	G beta:gamma signalling through CDC42	cancer-related	20
R-HSA-168927	TICAM1, RIP1-mediated IKK complex recruitment	other	20
R-HSA-2559583	Cellular Senescence	other	197

R-HSA-5693607	Processing of DNA double-strand break ends	cancer-related	97
R-HSA-901032	ER Quality Control Compartment (ERQC)	other	21
R-HSA-6807004	Negative regulation of MET activity	cancer-related	21
R-HSA-500657	Presynaptic function of Kainate receptors	other	21
R-HSA-4641263	Regulation of FZD by ubiquitination	other	21
R-HSA-6807878	COPI-mediated anterograde transport	other	101
R-HSA-9613829	Chaperone Mediated Autophagy	other	22
R-HSA-5205685	PINK1-PRKN Mediated Mitophagy	other	22
R-HSA-2979096	NOTCH2 Activation and Transmission of Signal to the I	cancer-related	22
R-HSA-8876384	Listeria monocytogenes entry into host cells	other	22
R-HSA-392170	ADP signalling through P2Y purinoceptor 12	other	22
R-HSA-912631	Regulation of signaling by CBL	other	22
R-HSA-450321	JNK (c-Jun kinases) phosphorylation and activation m	other	23
R-HSA-174048	APC/C:Cdc20 mediated degradation of Cyclin B	other	23
R-HSA-175474	Assembly Of The HIV Virion	other	23
R-HSA-140837	Intrinsic Pathway of Fibrin Clot Formation	other	23
R-HSA-5578749	Transcriptional regulation by small RNAs	other	105
R-HSA-8856825	Cargo recognition for clathrin-mediated endocytosis	cancer-related	106
R-HSA-5250913	Positive epigenetic regulation of rRNA expression	other	106
R-HSA-8940973	RUNX2 regulates osteoblast differentiation	other	24
R-HSA-450302	activated TAK1 mediates p38 MAPK activation	cancer-related	24
R-HSA-428930	Thromboxane signalling through TP receptor	cancer-related	24
R-HSA-202040	G-protein activation	cancer-related	24
R-HSA-9636383	Prevention of phagosomal-lysosomal fusion	other	24
R-HSA-9645723	Diseases of programmed cell death	cancer-related	108
R-HSA-1912422	Pre-NOTCH Expression and Processing	cancer-related	108
R-HSA-427413	NoRC negatively regulates rRNA expression	other	108
R-HSA-163685	Integration of energy metabolism	other	108
R-HSA-195721	Signaling by WNT	cancer-related	331
R-HSA-392451	G beta:gamma signalling through PI3Kgamma	cancer-related	25
R-HSA-937041	IKK complex recruitment mediated by RIP1	other	25
R-HSA-9013507	NOTCH3 Activation and Transmission of Signal to the I	cancer-related	25
R-HSA-179409	APC-Cdc20 mediated degradation of Nek2A	other	25
R-HSA-418592	ADP signalling through P2Y purinoceptor 1	cancer-related	25
R-HSA-5696397	Gap-filling DNA repair synthesis and ligation in GG-NEI	other	25
R-HSA-1643713	Signaling by EGFR in Cancer	cancer-related	25
R-HSA-9705462	Inactivation of CSF3 (G-CSF) signaling	other	25
R-HSA-73854	RNA Polymerase I Promoter Clearance	other	110
R-HSA-5250941	Negative epigenetic regulation of rRNA expression	other	111
R-HSA-1971475	A tetrasaccharide linker sequence is required for GAG	other	26
R-HSA-901042	Calnexin/calreticulin cycle	cancer-related	26
R-HSA-2173788	Downregulation of TGF-beta receptor signaling	other	26
R-HSA-157579	Telomere Maintenance	other	112
R-HSA-73864	RNA Polymerase I Transcription	other	113
R-HSA-8939211	ESR-mediated signaling	cancer-related	222
R-HSA-9648002	RAS processing	other	27
R-HSA-8982491	Glycogen metabolism	other	27

R-HSA-9682385	FLT3 signaling in disease	other	28
R-HSA-2022854	Keratan sulfate biosynthesis	other	28
R-HSA-9619665	EGR2 and SOX10-mediated initiation of Schwann cell r	other	28
R-HSA-400042	Adrenaline,noradrenaline inhibits insulin secretion	other	28
R-HSA-9816359	Maternal to zygotic transition (MZT)	other	117
R-HSA-1500620	Meiosis	other	117
R-HSA-8863795	Downregulation of ERBB2 signaling	cancer-related	29
R-HSA-5205647	Mitophagy	other	29
R-HSA-5654732	Negative regulation of FGFR3 signaling	other	29
R-HSA-1296041	Activation of G protein gated Potassium channels	other	29
R-HSA-1296059	G protein gated Potassium channels	other	29
R-HSA-997272	Inhibition of voltage gated Ca2+ channels via Gbeta/g	other	29
R-HSA-9637687	Suppression of phagosomal maturation	other	29
R-HSA-201681	TCF dependent signaling in response to WNT	cancer-related	232
R-HSA-8878166	Transcriptional regulation by RUNX2	other	121
R-HSA-5617472	Activation of anterior HOX genes in hindbrain develop	other	121
R-HSA-5619507	Activation of HOX genes during differentiation	cancer-related	121
R-HSA-4090294	SUMOylation of intracellular receptors	other	30
R-HSA-8866652	Synthesis of active ubiquitin: roles of E1 and E2 enzym	other	30
R-HSA-9683701	Translation of Structural Proteins	immune-related	30
R-HSA-110314	Recognition of DNA damage by PCNA-containing repli	other	30
R-HSA-2173795	Downregulation of SMAD2/3:SMAD4 transcriptional a	other	30
R-HSA-9674555	Signaling by CSF3 (G-CSF)	other	30
R-HSA-157118	Signaling by NOTCH	cancer-related	235
R-HSA-8878171	Transcriptional regulation by RUNX1	cancer-related	236
R-HSA-2122948	Activated NOTCH1 Transmits Signal to the Nucleus	cancer-related	31
R-HSA-182971	EGFR downregulation	immune-related	31
R-HSA-5654733	Negative regulation of FGFR4 signaling	other	31
R-HSA-917977	Transferrin endocytosis and recycling	other	31
R-HSA-9680350	Signaling by CSF1 (M-CSF) in myeloid cells	other	31
R-HSA-1630316	Glycosaminoglycan metabolism	other	126
R-HSA-397795	G-protein beta:gamma signalling	cancer-related	32
R-HSA-8941326	RUNX2 regulates bone development	other	32
R-HSA-5675482	Regulation of necroptotic cell death	cancer-related	32
R-HSA-917729	Endosomal Sorting Complex Required For Transport (E	other	32
R-HSA-456926	Thrombin signalling through proteinase activated rece	cancer-related	32
R-HSA-451326	Activation of kainate receptors upon glutamate bindir	other	32
R-HSA-5656169	Termination of translesion DNA synthesis	other	32
R-HSA-1980145	Signaling by NOTCH2	cancer-related	33
R-HSA-5694530	Cargo concentration in the ER	other	33
R-HSA-5357956	TNFR1-induced NF-kappa-B signaling pathway	other	33
R-HSA-5654726	Negative regulation of FGFR1 signaling	other	33
R-HSA-392518	Signal amplification	other	33
R-HSA-420092	Glucagon-type ligand receptors	other	33
R-HSA-163359	Glucagon signaling in metabolic regulation	other	33
R-HSA-5213460	RIPK1-mediated regulated necrosis	cancer-related	34
R-HSA-5654727	Negative regulation of FGFR2 signaling	other	34

R-HSA-5663084	Diseases of carbohydrate metabolism	other	34
R-HSA-5693567	HDR through Homologous Recombination (HRR) or Sir	cancer-related	132
R-HSA-5668914	Diseases of metabolism	other	250
R-HSA-9615710	Late endosomal microautophagy	other	35
R-HSA-1296065	Inwardly rectifying K+ channels	other	35
R-HSA-2559585	Oncogene Induced Senescence	other	35
R-HSA-532668	N-glycan trimming in the ER and Calnexin/Calreticulin	cancer-related	35
R-HSA-9833109	Evasion by RSV of host interferon responses	other	35
R-HSA-936440	Negative regulators of DDX58/IFIH1 signaling	other	35
R-HSA-6804757	Regulation of TP53 Degradation	other	36
R-HSA-162588	Budding and maturation of HIV virion	other	36
R-HSA-1638074	Keratan sulfate/keratin metabolism	other	36
R-HSA-211000	Gene Silencing by RNA	other	137
R-HSA-6806003	Regulation of TP53 Expression and Degradation	other	37
R-HSA-8964043	Plasma lipoprotein clearance	other	37
R-HSA-1660661	Sphingolipid de novo biosynthesis	other	37
R-HSA-5689901	Metalloprotease DUBs	cancer-related	37
R-HSA-168638	NOD1/2 Signaling Pathway	other	37
R-HSA-5693538	Homology Directed Repair	cancer-related	138
R-HSA-9607240	FLT3 Signaling	cancer-related	38
R-HSA-5696394	DNA Damage Recognition in GG-NER	cancer-related	38
R-HSA-6814122	Cooperation of PDCL (PhLP1) and TRiC/CCT in G-prote	cancer-related	38
R-HSA-6807070	PTEN Regulation	other	140
R-HSA-73886	Chromosome Maintenance	other	140
R-HSA-68875	Mitotic Prophase	other	141
R-HSA-5689896	Ovarian tumor domain proteases	other	39
R-HSA-110313	Translesion synthesis by Y family DNA polymerases by	other	39
R-HSA-140877	Formation of Fibrin Clot (Clotting Cascade)	other	39
R-HSA-3781865	Diseases of glycosylation	other	144
R-HSA-6783310	Fanconi Anemia Pathway	other	40
R-HSA-5654741	Signaling by FGFR3	other	40
R-HSA-8856828	Clathrin-mediated endocytosis	cancer-related	146
R-HSA-3858494	Beta-catenin independent WNT signaling	cancer-related	146
R-HSA-5696400	Dual Incision in GG-NER	other	41
R-HSA-5654743	Signaling by FGFR4	other	41
R-HSA-9035034	RHOF GTPase cycle	cancer-related	42
R-HSA-381676	Glucagon-like Peptide-1 (GLP1) regulates insulin secre	other	42
R-HSA-9692916	SARS-CoV-1 activates/modulates innate immune resp	other	42
R-HSA-3769402	Deactivation of the beta-catenin transactivating comp	cancer-related	42
R-HSA-9018519	Estrogen-dependent gene expression	other	149
R-HSA-9646399	Aggrephagy	other	43
R-HSA-5696395	Formation of Incision Complex in GG-NER	other	43
R-HSA-432040	Vasopressin regulates renal water homeostasis via Aq	other	43
R-HSA-9660821	ADORA2B mediated anti-inflammatory cytokines proc	other	43
R-HSA-977444	GABA B receptor activation	other	43
R-HSA-991365	Activation of GABAB receptors	other	43
R-HSA-5675221	Negative regulation of MAPK pathway	cancer-related	43

R-HSA-5633008	TP53 Regulates Transcription of Cell Death Genes	other	44
R-HSA-1474165	Reproduction	other	154
R-HSA-5673001	RAF/MAP kinase cascade	cancer-related	280
R-HSA-445355	Smooth Muscle Contraction	other	45
R-HSA-445989	TAK1-dependent IKK and NF-kappa-B activation	other	45
R-HSA-9634597	GPER1 signaling	cancer-related	45
R-HSA-9679506	SARS-CoV Infections	other	417
R-HSA-9637690	Response of Mtb to phagocytosis	cancer-related	46
R-HSA-5684996	MAPK1/MAPK3 signaling	cancer-related	286
R-HSA-190828	Gap junction trafficking	other	47
R-HSA-69231	Cyclin D associated events in G1	other	47
R-HSA-69236	G1 Phase	other	47
R-HSA-2122947	NOTCH1 Intracellular Domain Regulates Transcription	cancer-related	48
R-HSA-418597	G alpha (z) signalling events	other	48
R-HSA-73893	DNA Damage Bypass	other	48
R-HSA-512988	Interleukin-3, Interleukin-5 and GM-CSF signaling	immune-related	48
R-HSA-9012852	Signaling by NOTCH3	cancer-related	49
R-HSA-157858	Gap junction trafficking and regulation	other	49
R-HSA-1227986	Signaling by ERBB2	cancer-related	50
R-HSA-1793185	Chondroitin sulfate/dermatan sulfate metabolism	other	50
R-HSA-5357905	Regulation of TNFR1 signaling	other	50
R-HSA-211733	Regulation of activated PAK-2p34 by proteasome mec	other	50
R-HSA-3214842	HDMs demethylate histones	other	50
R-HSA-3928665	EPH-ephrin mediated repulsion of cells	cancer-related	51
R-HSA-171306	Packaging Of Telomere Ends	other	51
R-HSA-69481	G2/M Checkpoints	other	167
R-HSA-9006931	Signaling by Nuclear Receptors	other	298
R-HSA-71387	Metabolism of carbohydrates	other	299
R-HSA-5693532	DNA Double-Strand Break Repair	cancer-related	168
R-HSA-75815	Ubiquitin-dependent degradation of Cyclin D	other	52
R-HSA-349425	Autodegradation of the E3 ubiquitin ligase COP1	other	52
R-HSA-69601	Ubiquitin Mediated Degradation of Phosphorylated C	other	52
R-HSA-69610	p53-Independent DNA Damage Response	cancer-related	52
R-HSA-69613	p53-Independent G1/S DNA damage checkpoint	cancer-related	52
R-HSA-9762114	GSK3B and BTRC:CUL1-mediated-degradation of NFE2	other	52
R-HSA-5654736	Signaling by FGFR1	other	52
R-HSA-445717	Aquaporin-mediated transport	other	52
R-HSA-169911	Regulation of Apoptosis	other	53
R-HSA-180534	Vpu mediated degradation of CD4	other	53
R-HSA-177929	Signaling by EGFR	cancer-related	53
R-HSA-6781823	Formation of TC-NER Pre-Incision Complex	other	54
R-HSA-9013405	RHOD GTPase cycle	cancer-related	54
R-HSA-8848021	Signaling by PTK6	other	54
R-HSA-9006927	Signaling by Non-Receptor Tyrosine Kinases	other	54
R-HSA-9604323	Negative regulation of NOTCH4 signaling	cancer-related	54
R-HSA-174113	SCF-beta-TrCP mediated degradation of Emi1	other	55
R-HSA-180585	Vif-mediated degradation of APOBEC3G	other	55

R-HSA-4641257	Degradation of AXIN	other	55
R-HSA-8854050	FBXL7 down-regulates AURKA during mitotic entry an	other	55
R-HSA-8941858	Regulation of RUNX3 expression and activity	other	55
R-HSA-110330	Recognition and association of DNA glycosylase with s	other	55
R-HSA-110331	Cleavage of the damaged purine	other	55
R-HSA-73927	Depurination	other	55
R-HSA-9013409	RHOJ GTPase cycle	cancer-related	55
R-HSA-5619115	Disorders of transmembrane transporters	other	176
R-HSA-162582	Signal Transduction	cancer-related	2599
R-HSA-450408	AUF1 (hnRNP D0) binds and destabilizes mRNA	cancer-related	56
R-HSA-5362768	Hh mutants are degraded by ERAD	other	56
R-HSA-1638091	Heparan sulfate/heparin (HS-GAG) metabolism	other	57
R-HSA-4641258	Degradation of DVL	other	57
R-HSA-69541	Stabilization of p53	cancer-related	57
R-HSA-2644602	Signaling by NOTCH1 PEST Domain Mutants in Cancer	cancer-related	58
R-HSA-2644603	Signaling by NOTCH1 in Cancer	cancer-related	58
R-HSA-2644606	Constitutive Signaling by NOTCH1 PEST Domain Mutai	cancer-related	58
R-HSA-2894858	Signaling by NOTCH1 HD+PEST Domain Mutants in Ca	cancer-related	58
R-HSA-2894862	Constitutive Signaling by NOTCH1 HD+PEST Domain M	cancer-related	58
R-HSA-1236394	Signaling by ERBB4	cancer-related	58
R-HSA-9694635	Translation of Structural Proteins	immune-related	58
R-HSA-75893	TNF signaling	other	59
R-HSA-5387390	Hh mutants abrogate ligand secretion	other	59
R-HSA-5676590	NIK-->noncanonical NF-kB signaling	other	59
R-HSA-9013406	RHOQ GTPase cycle	cancer-related	59
R-HSA-8866654	E3 ubiquitin ligases ubiquitinate target proteins	other	59
R-HSA-3108232	SUMO E3 ligases SUMOylate target proteins	other	183
R-HSA-187577	SCF(Skp2)-mediated degradation of p27/p21	other	60
R-HSA-5607761	Dectin-1 mediated noncanonical NF-kB signaling	other	60
R-HSA-5610780	Degradation of GLI1 by the proteasome	other	60
R-HSA-5610783	Degradation of GLI2 by the proteasome	other	60
R-HSA-5610785	GLI3 is processed to GLI3R by the proteasome	other	60
R-HSA-110328	Recognition and association of DNA glycosylase with s	other	60
R-HSA-110329	Cleavage of the damaged pyrimidine	other	60
R-HSA-73928	Depyrimidination	other	60
R-HSA-168643	Nucleotide-binding domain, leucine rich repeat contai	other	60
R-HSA-977443	GABA receptor activation	other	60
R-HSA-8943724	Regulation of PTEN gene transcription	other	61
R-HSA-5678895	Defective CFTR causes cystic fibrosis	other	61
R-HSA-5683057	MAPK family signaling cascades	cancer-related	325
R-HSA-5218859	Regulated Necrosis	cancer-related	62
R-HSA-73929	Base-Excision Repair, AP Site Formation	other	62
R-HSA-4086398	Ca2+ pathway	cancer-related	62
R-HSA-8986944	Transcriptional Regulation by MECP2	other	62
R-HSA-2990846	SUMOylation	other	189
R-HSA-212165	Epigenetic regulation of gene expression	other	189
R-HSA-9033241	Peroxisomal protein import	other	63

R-HSA-174084	Autodegradation of Cdh1 by Cdh1:APC/C	other	63
R-HSA-4608870	Asymmetric localization of PCP proteins	other	64
R-HSA-450294	MAP kinase activation	other	64
R-HSA-1834949	Cytosolic sensors of pathogen-associated DNA	other	64
R-HSA-6782210	Gap-filling DNA repair synthesis and ligation in TC-NEF	other	65
R-HSA-5358346	Hedgehog ligand biogenesis	other	65
R-HSA-6782135	Dual incision in TC-NER	other	66
R-HSA-69563	p53-Dependent G1 DNA Damage Response	cancer-related	66
R-HSA-69580	p53-Dependent G1/S DNA damage checkpoint	cancer-related	66
R-HSA-1234176	Oxygen-dependent proline hydroxylation of Hypoxia-i	other	66
R-HSA-199991	Membrane Trafficking	other	634
R-HSA-1169091	Activation of NF-kappaB in B cells	immune-related	67
R-HSA-174154	APC/C:Cdc20 mediated degradation of Securin	other	67
R-HSA-5685942	HDR through Homologous Recombination (HRR)	other	68
R-HSA-5693571	Nonhomologous End-Joining (NHEJ)	cancer-related	68
R-HSA-204005	COPII-mediated vesicle transport	other	68
R-HSA-5658442	Regulation of RAS by GAPs	cancer-related	68
R-HSA-69615	G1/S DNA Damage Checkpoints	cancer-related	68
R-HSA-8948751	Regulation of PTEN stability and activity	cancer-related	69
R-HSA-3214841	PKMTs methylate histone lysines	other	69
R-HSA-400253	Circadian Clock	other	70
R-HSA-4551638	SUMOylation of chromatin organization proteins	other	71
R-HSA-68949	Orc1 removal from chromatin	other	71
R-HSA-9772573	Late SARS-CoV-2 Infection Events	other	71
R-HSA-69017	CDK-mediated phosphorylation and removal of Cdc6	other	72
R-HSA-174184	Cdc20:Phospho-APC/C mediated degradation of Cyclir	other	72
R-HSA-448424	Interleukin-17 signaling	immune-related	72
R-HSA-606279	Deposition of new CENPA-containing nucleosomes at	other	73
R-HSA-774815	Nucleosome assembly	other	73
R-HSA-174178	APC/C:Cdh1 mediated degradation of Cdc20 and othe	other	73
R-HSA-179419	APC:Cdc20 mediated degradation of cell cycle protein	other	73
R-HSA-8939902	Regulation of RUNX2 expression and activity	other	73
R-HSA-5654738	Signaling by FGFR2	cancer-related	73
R-HSA-1980143	Signaling by NOTCH1	cancer-related	74
R-HSA-1169408	ISG15 antiviral mechanism	other	74
R-HSA-174824	Plasma lipoprotein assembly, remodeling, and clearan	other	75
R-HSA-1234174	Cellular response to hypoxia	other	75
R-HSA-176409	APC/C:Cdc20 mediated degradation of mitotic proteir	other	75
R-HSA-204998	Cell death signalling via NRAGE, NRIF and NADE	cancer-related	76
R-HSA-176814	Activation of APC/C and APC/C:Cdc20 mediated degra	other	76
R-HSA-9009391	Extra-nuclear estrogen signaling	other	77
R-HSA-8852276	The role of GTSE1 in G2/M progression after G2 check	cancer-related	77
R-HSA-909733	Interferon alpha/beta signaling	immune-related	77
R-HSA-5619084	ABC transporter disorders	other	78
R-HSA-3214858	RMTs methylate histone arginines	cancer-related	78
R-HSA-1221632	Meiotic synapsis	other	78
R-HSA-422356	Regulation of insulin secretion	other	78

R-HSA-6781827	Transcription-Coupled Nucleotide Excision Repair (TC-	other	79
R-HSA-2559586	DNA Damage/Telomere Stress Induced Senescence	other	79
R-HSA-8852135	Protein ubiquitination	other	79
R-HSA-416482	G alpha (12/13) signalling events	cancer-related	80
R-HSA-176408	Regulation of APC/C activators between G1/S and ear	other	80
R-HSA-9663891	Selective autophagy	other	81
R-HSA-168928	DDX58/IFIH1-mediated induction of interferon-alpha/	immune-related	82
R-HSA-9013694	Signaling by NOTCH4	cancer-related	82
R-HSA-2871837	FCERI mediated NF-kB activation	immune-related	82
R-HSA-9662851	Anti-inflammatory response favouring Leishmania par	other	82
R-HSA-9664433	Leishmania parasite growth and survival	other	82
R-HSA-69202	Cyclin E associated events during G1/S transition	other	83
R-HSA-195253	Degradation of beta-catenin by the destruction compl	other	83
R-HSA-1168372	Downstream signaling events of B Cell Receptor (BCR)	immune-related	83
R-HSA-5696399	Global Genome Nucleotide Excision Repair (GG-NER)	cancer-related	84
R-HSA-69656	Cyclin A:Cdk2-associated events at S phase entry	other	85
R-HSA-5632684	Hedgehog 'on' state	other	85
R-HSA-174143	APC/C-mediated degradation of cell cycle proteins	other	87
R-HSA-453276	Regulation of mitotic cell cycle	other	87
R-HSA-2565942	Regulation of PLK1 Activity at G2/M Transition	other	88
R-HSA-450531	Regulation of mRNA stability by proteins that bind AU	other	88
R-HSA-418346	Platelet homeostasis	cancer-related	88
R-HSA-2980736	Peptide hormone metabolism	other	89
R-HSA-5687128	MAPK6/MAPK4 signaling	cancer-related	89
R-HSA-190236	Signaling by FGFR	cancer-related	89
R-HSA-390466	Chaperonin-mediated protein folding	other	90
R-HSA-156902	Peptide chain elongation	other	90
R-HSA-73884	Base Excision Repair	other	91
R-HSA-69052	Switching of origins to a post-replicative state	other	91
R-HSA-1236974	ER-Phagosome pathway	immune-related	91
R-HSA-9700206	Signaling by ALK in cancer	cancer-related	91
R-HSA-9725370	Signaling by ALK fusions and activated point mutants	cancer-related	91
R-HSA-2682334	EPH-Ephrin signaling	cancer-related	92
R-HSA-6804756	Regulation of TP53 Activity through Phosphorylation	cancer-related	92
R-HSA-4086400	PCP/CE pathway	cancer-related	92
R-HSA-111885	Opioid Signalling	other	92
R-HSA-69473	G2/M DNA damage checkpoint	cancer-related	94
R-HSA-3214815	HDACs deacetylate histones	other	94
R-HSA-156842	Eukaryotic Translation Elongation	other	94
R-HSA-2408557	Selenocysteine synthesis	other	94
R-HSA-72764	Eukaryotic Translation Termination	other	94
R-HSA-373080	Class B/2 (Secretin family receptors)	other	95
R-HSA-391251	Protein folding	other	96
R-HSA-975956	Nonsense Mediated Decay (NMD) independent of the	other	96
R-HSA-193704	p75 NTR receptor-mediated signalling	other	97
R-HSA-8878159	Transcriptional regulation by RUNX3	other	97
R-HSA-9759194	Nuclear events mediated by NFE2L2	cancer-related	97

R-HSA-202424	Downstream TCR signaling	immune-related	98
R-HSA-168142	Toll Like Receptor 10 (TLR10) Cascade	immune-related	98
R-HSA-168176	Toll Like Receptor 5 (TLR5) Cascade	immune-related	98
R-HSA-975871	MyD88 cascade initiated on plasma membrane	immune-related	98
R-HSA-9692914	SARS-CoV-1-host interactions	other	98
R-HSA-5619102	SLC transporter disorders	other	98
R-HSA-9833110	RSV-host interactions	other	100
R-HSA-5607764	CLEC7A (Dectin-1) signaling	other	101
R-HSA-192823	Viral mRNA Translation	other	101
R-HSA-68886	M Phase	other	416
R-HSA-5668541	TNFR2 non-canonical NF-kB pathway	other	102
R-HSA-5689603	UCH proteinases	cancer-related	102
R-HSA-72689	Formation of a pool of free 40S subunits	other	102
R-HSA-9633012	Response of EIF2AK4 (GCN2) to amino acid deficiency	other	102
R-HSA-1296071	Potassium Channels	other	103
R-HSA-975138	TRAF6 mediated induction of NFkB and MAP kinases u	other	103
R-HSA-382556	ABC-family proteins mediated transport	other	103
R-HSA-975155	MyD88 dependent cascade initiated on endosome	immune-related	104
R-HSA-168164	Toll Like Receptor 3 (TLR3) Cascade	immune-related	105
R-HSA-168181	Toll Like Receptor 7/8 (TLR7/8) Cascade	immune-related	105
R-HSA-168249	Innate Immune System	immune-related	1076
R-HSA-428157	Sphingolipid metabolism	other	106
R-HSA-1236975	Antigen processing-Cross presentation	immune-related	106
R-HSA-168138	Toll Like Receptor 9 (TLR9) Cascade	immune-related	108
R-HSA-6785807	Interleukin-4 and Interleukin-13 signaling	immune-related	108
R-HSA-425393	Transport of inorganic cations/anions and amino acids	other	108
R-HSA-1257604	PIP3 activates AKT signaling	cancer-related	268
R-HSA-1643685	Disease	other	2074
R-HSA-2672351	Stimuli-sensing channels	other	109
R-HSA-212436	Generic Transcription Pathway	other	1253
R-HSA-392499	Metabolism of proteins	cancer-related	2077
R-HSA-166166	MyD88-independent TLR4 cascade	immune-related	110
R-HSA-937061	TRIF(TICAM1)-mediated TLR4 signaling	immune-related	110
R-HSA-5696398	Nucleotide Excision Repair	cancer-related	111
R-HSA-9635486	Infection with Mycobacterium tuberculosis	other	111
R-HSA-983705	Signaling by the B Cell Receptor (BCR)	immune-related	112
R-HSA-156827	L13a-mediated translational silencing of Ceruloplasmi	immune-related	112
R-HSA-5610787	Hedgehog 'off' state	cancer-related	113
R-HSA-72706	GTP hydrolysis and joining of the 60S ribosomal subur	other	113
R-HSA-1799339	SRP-dependent cotranslational protein targeting to m	other	113
R-HSA-166058	MyD88:MAL(TIRAP) cascade initiated on plasma mem	immune-related	114
R-HSA-168188	Toll Like Receptor TLR6:TLR2 Cascade	immune-related	114
R-HSA-9020702	Interleukin-1 signaling	immune-related	115
R-HSA-927802	Nonsense-Mediated Decay (NMD)	other	116
R-HSA-975957	Nonsense Mediated Decay (NMD) enhanced by the Ex	other	116
R-HSA-1989781	PPARA activates gene expression	other	117
R-HSA-168179	Toll Like Receptor TLR1:TLR2 Cascade	immune-related	117

R-HSA-181438	Toll Like Receptor 2 (TLR2) Cascade	immune-related	117
R-HSA-400206	Regulation of lipid metabolism by PPARalpha	other	119
R-HSA-2408522	Selenoamino acid metabolism	other	119
R-HSA-69239	Synthesis of DNA	other	120
R-HSA-202403	TCR signaling	immune-related	120
R-HSA-72613	Eukaryotic Translation Initiation	other	120
R-HSA-72737	Cap-dependent Translation Initiation	other	120
R-HSA-597592	Post-translational protein modification	cancer-related	1462
R-HSA-9820952	Respiratory Syncytial Virus Infection Pathway	other	123
R-HSA-69620	Cell Cycle Checkpoints	cancer-related	292
R-HSA-9755511	KEAP1-NFE2L2 pathway	cancer-related	126
R-HSA-69206	G1/S Transition	other	131
R-HSA-2454202	Fc epsilon receptor (FCERI) signaling	other	134
R-HSA-9006925	Intracellular signaling by second messengers	other	310
R-HSA-1632852	Macroautophagy	other	136
R-HSA-162909	Host Interactions of HIV factors	other	137
R-HSA-3214847	HATs acetylate histones	cancer-related	141
R-HSA-162599	Late Phase of HIV Life Cycle	other	144
R-HSA-9678108	SARS-CoV-1 Infection	other	144
R-HSA-5621481	C-type lectin receptors (CLRs)	immune-related	144
R-HSA-195258	RHO GTPase Effectors	cancer-related	326
R-HSA-166016	Toll Like Receptor 4 (TLR4) Cascade	immune-related	147
R-HSA-168273	Influenza Viral RNA Transcription and Replication	other	147
R-HSA-453279	Mitotic G1 phase and G1/S transition	other	149
R-HSA-5358351	Signaling by Hedgehog	cancer-related	149
R-HSA-73857	RNA Polymerase II Transcription	other	1377
R-HSA-9612973	Autophagy	other	151
R-HSA-73894	DNA Repair	cancer-related	335
R-HSA-446652	Interleukin-1 family signaling	other	153
R-HSA-1169410	Antiviral mechanism by IFN-stimulated genes	immune-related	154
R-HSA-9013148	CDC42 GTPase cycle	cancer-related	155
R-HSA-73887	Death Receptor Signaling	cancer-related	156
R-HSA-162587	HIV Life Cycle	other	157
R-HSA-9711097	Cellular response to starvation	other	157
R-HSA-418555	G alpha (s) signalling events	cancer-related	158
R-HSA-5633007	Regulation of TP53 Activity	other	160
R-HSA-69242	S Phase	other	162
R-HSA-9609507	Protein localization	other	164
R-HSA-9824439	Bacterial Infection Pathways	other	166
R-HSA-9658195	Leishmania infection	other	167
R-HSA-9824443	Parasitic Infection Pathways	other	167
R-HSA-983231	Factors involved in megakaryocyte development and	cancer-related	168
R-HSA-168255	Influenza Infection	other	168
R-HSA-168898	Toll-like Receptor Cascades	immune-related	170
R-HSA-3700989	Transcriptional Regulation by TP53	cancer-related	362
R-HSA-9010553	Regulation of expression of SLITs and ROBOs	other	172
R-HSA-382551	Transport of small molecules	other	735

R-HSA-69278	Cell Cycle, Mitotic	other	560
R-HSA-109581	Apoptosis	cancer-related	182
R-HSA-983712	Ion channel transport	other	186
R-HSA-6791226	Major pathway of rRNA processing in the nucleolus ar	other	187
R-HSA-2467813	Separation of Sister Chromatids	other	190
R-HSA-1280218	Adaptive Immune System	immune-related	770
R-HSA-8868773	rRNA processing in the nucleus and cytosol	other	197
R-HSA-69275	G2/M Transition	other	197
R-HSA-453274	Mitotic G2-G2/M phases	other	199
R-HSA-9610379	HCMV Late Events	other	204
R-HSA-397014	Muscle contraction	other	205
R-HSA-112314	Neurotransmitter receptors and postsynaptic signal tr	other	205
R-HSA-9824446	Viral Infection Pathways	other	985
R-HSA-72312	rRNA processing	other	208
R-HSA-74160	Gene expression (Transcription)	cancer-related	1547
R-HSA-9711123	Cellular response to chemical stress	cancer-related	215
R-HSA-416476	G alpha (q) signalling events	cancer-related	216
R-HSA-5357801	Programmed Cell Death	cancer-related	217
R-HSA-5689880	Ub-specific processing proteases	cancer-related	219
R-HSA-376176	Signaling by ROBO receptors	cancer-related	219
R-HSA-68882	Mitotic Anaphase	other	235
R-HSA-9609690	HCMV Early Events	other	235
R-HSA-2555396	Mitotic Metaphase and Anaphase	other	236
R-HSA-162906	HIV Infection	other	239
R-HSA-8951664	Neddylation	other	246
R-HSA-425407	SLC-mediated transmembrane transport	other	247
R-HSA-449147	Signaling by Interleukins	immune-related	473
R-HSA-6798695	Neutrophil degranulation	immune-related	480
R-HSA-5663205	Infectious disease	other	1273
R-HSA-1640170	Cell Cycle	cancer-related	691
R-HSA-194315	Signaling by Rho GTPases	cancer-related	706
R-HSA-3247509	Chromatin modifying enzymes	other	270
R-HSA-4839726	Chromatin organization	other	270
R-HSA-112315	Transmission across Chemical Synapses	other	270
R-HSA-913531	Interferon Signaling	immune-related	274
R-HSA-9716542	Signaling by Rho GTPases, Miro GTPases and RHOBTB:	cancer-related	722
R-HSA-168256	Immune System	immune-related	2068
R-HSA-9609646	HCMV Infection	other	290
R-HSA-72766	Translation	other	292
R-HSA-5688426	Deubiquitination	other	297
R-HSA-983168	Antigen processing: Ubiquitination & Proteasome deg	immune-related	309
R-HSA-418594	G alpha (i) signalling events	cancer-related	318
R-HSA-2262752	Cellular responses to stress	other	807
R-HSA-8953897	Cellular responses to stimuli	other	821
R-HSA-71291	Metabolism of amino acids and derivatives	cancer-related	375
R-HSA-983169	Class I MHC mediated antigen processing & presentat	immune-related	382
R-HSA-112316	Neuronal System	other	410

R-HSA-9012999	RHO GTPase cycle	cancer-related	449
R-HSA-5663202	Diseases of signal transduction by growth factor recep	cancer-related	458
R-HSA-500792	GPCR ligand binding	cancer-related	468
R-HSA-556833	Metabolism of lipids	other	758
R-HSA-1280215	Cytokine Signaling in Immune system	immune-related	792
R-HSA-388396	GPCR downstream signalling	cancer-related	634
R-HSA-1430728	Metabolism	other	2127
R-HSA-372790	Signaling by GPCR	cancer-related	709
R-HSA-8953854	Metabolism of RNA	other	729

# detected	p.val	FDR	OR	ProteinName
10	2.15E-11	1.43E-08	29.21	ACAN, COL1A1, COL1A2, COL2A1, COL6A1, COL6A3, ITGA2, LAM
13	2.09E-08	1.38E-05	9.03	ACAN, COL1A1, COL1A2, COL2A1, COL6A1, COL6A3, COL7A1, EFI
8	3.71E-08	2.45E-05	19.35	COL1A1, COL1A2, COL2A1, COL6A1, COL6A3, COL7A1, ITGA2, TN
5	7.35E-08	4.85E-05	63.69	COL1A1, COL1A2, COLEC11, MARCO, MASP1
4	8.20E-08	5.40E-05	175.70	COLEC11, MASP1, MASP2, MBL2
6	1.66E-07	1.09E-04	30.14	COL1A1, COL1A2, COLEC11, MARCO, MASP1, SSC5D
6	2.22E-07	1.46E-04	28.55	COL1A1, COL1A2, COL2A1, COL6A1, COL6A3, COL7A1
7	4.25E-07	2.79E-04	17.81	COL1A1, COL1A2, COL2A1, HGFAC, ITGA2, LAMA2, UBA52
5	8.56E-07	5.60E-04	35.63	COL1A1, COL1A2, COL2A1, ITGA2, LAMA2
4	1.14E-06	7.46E-04	70.24	COLEC11, MASP1, MASP2, MBL2
6	1.32E-06	8.61E-04	20.44	COL1A1, COL1A2, COL2A1, ITGA2, LAMA2, TNC
6	1.61E-06	1.05E-03	19.70	COL1A1, COL1A2, COL2A1, COL6A1, COL6A3, COL7A1
4	2.05E-06	1.34E-03	58.53	COL1A1, COL1A2, GP9, ITGA2
6	2.14E-06	1.39E-03	18.67	COL1A1, COL1A2, COL2A1, COL6A1, COL6A3, COL7A1
6	2.81E-06	1.83E-03	17.75	COL1A1, COL1A2, COL2A1, COL6A1, COL6A3, COL7A1
5	4.28E-06	2.77E-03	24.72	COL1A1, COL1A2, COL2A1, ITGA2, LAMA2
3	7.14E-06	4.62E-03	129.75	COL1A1, COL1A2, GP9
3	7.14E-06	4.62E-03	129.75	COL1A1, COL1A2, GP9
4	9.69E-06	6.25E-03	36.94	COLEC11, MASP1, MASP2, MBL2
3	1.14E-05	7.32E-03	103.79	COL1A1, COL1A2, GP9
6	1.58E-05	1.01E-02	12.86	COL1A1, COL1A2, COL2A1, COL6A1, COL6A3, COL7A1
4	1.89E-05	1.21E-02	30.51	COL1A1, COL1A2, ITGA2, TNC
7	1.97E-05	1.26E-02	9.59	ACAN, COL1A1, COL1A2, COL2A1, COL6A1, COL6A3, COL7A1
5	3.62E-05	2.32E-02	15.31	COL2A1, COL6A1, COL6A3, SPTA1, SPTB
3	4.39E-05	2.81E-02	57.64	COL1A1, COL1A2, GP9
3	8.97E-05	5.72E-02	43.22	COL1A1, COL1A2, COL7A1
2	1.06E-04	6.77E-02	340.85	COL1A1, COL1A2
19	1.20E-04	7.64E-02	3.10	ADIPOQ, CDSN, CLTCL1, COL2A1, COL6A1, COL6A3, H3-3A, H4C1
3	1.88E-04	1.19E-01	32.40	COL1A1, COL1A2, GP9
2	2.12E-04	1.34E-01	170.41	COL1A1, COL1A2
2	2.12E-04	1.34E-01	170.41	COL1A1, COL1A2
2	3.52E-04	2.22E-01	113.60	MASP1, MASP2
4	3.96E-04	2.50E-01	12.96	COLEC11, MASP1, MASP2, MBL2
4	3.96E-04	2.50E-01	12.96	COL2A1, COL6A1, COL6A3, THBS4
3	7.50E-04	4.72E-01	19.18	COL7A1, ITGA2, LAMA2
5	1.05E-03	6.56E-01	7.12	CDSN, KRT24, KRT32, KRT85, KRT86
10	1.20E-03	7.55E-01	3.54	COL1A1, COL1A2, COL2A1, COL6A1, COL6A3, HGFAC, ITGA2, LAM
3	1.62E-03	1.00E+00	14.38	COL1A1, COL1A2, GP9
3	2.01E-03	1.00E+00	13.27	COL2A1, COL6A1, COL6A3
11	2.08E-03	1.00E+00	3.09	CLTCL1, COL1A1, COL1A2, COL7A1, COLEC11, MARCO, MASP1, S
10	2.23E-03	1.00E+00	3.24	CLTCL1, COL2A1, COL6A1, COL6A3, ITGA2, LAMA2, RELN, SPTA1,
4	4.27E-03	1.00E+00	6.57	H3-3A, H4C1, TGFBI, UBA52
2	5.12E-03	1.00E+00	21.27	COL1A1, COL1A2

9	5.59E-03	1.00E+00	3.00	CLTCL1, COL2A1, COL6A1, COL6A3, ITGA2, RELN, SPTA1, SPTB, U
1	6.01E-03	1.00E+00	Inf	SLC4A1
4	7.08E-03	1.00E+00	5.65	MASP1, MASP2, MBL2, UBA52
5	9.34E-03	1.00E+00	4.17	CDSN, KRT24, KRT32, KRT85, KRT86
2	1.48E-02	1.00E+00	11.72	SPTA1, SPTB
2	1.86E-02	1.00E+00	10.30	COL1A1, COL1A2
3	1.89E-02	1.00E+00	5.66	COL1A2, LTBP1, UBA52
2	1.97E-02	1.00E+00	10.00	COL1A2, UBA52
2	2.07E-02	1.00E+00	9.71	EFEMP1, LTBP1
1	2.38E-02	1.00E+00	55.95	UBA52
1	2.38E-02	1.00E+00	55.95	UBA52
2	2.51E-02	1.00E+00	8.71	ACAN, VCAN
2	2.62E-02	1.00E+00	8.49	H3-3A, H4C1
3	2.71E-02	1.00E+00	4.90	LTBP1, TNC, VCAN
3	2.84E-02	1.00E+00	4.81	H3-3A, H4C1, UBA52
2	2.86E-02	1.00E+00	8.09	EFEMP1, LTBP1
1	2.97E-02	1.00E+00	41.96	PPARG
1	2.97E-02	1.00E+00	41.96	RELN
2	3.23E-02	1.00E+00	7.54	LTBP1, UBA52
4	3.41E-02	1.00E+00	3.45	MASP1, MASP2, MBL2, UBA52
3	3.47E-02	1.00E+00	4.43	ITGA2, SPTA1, SPTB
1	3.55E-02	1.00E+00	33.56	HGFAC
1	3.55E-02	1.00E+00	33.56	GP9
1	3.55E-02	1.00E+00	33.56	SSC5D
2	3.76E-02	1.00E+00	6.93	COL1A2, UBA52
3	3.84E-02	1.00E+00	4.24	COL1A2, LTBP1, UBA52
2	3.89E-02	1.00E+00	6.79	NR0B1, PPARG
3	3.92E-02	1.00E+00	4.21	LTBP1, TNC, VCAN
3	4.00E-02	1.00E+00	4.17	H3-3A, H4C1, UBA52
1	4.13E-02	1.00E+00	27.97	UBA52
3	4.24E-02	1.00E+00	4.07	H3-3A, H4C1, UBA52
3	4.66E-02	1.00E+00	3.92	COL1A1, COL1A2, COL2A1
1	4.71E-02	1.00E+00	23.97	VCAN
1	4.71E-02	1.00E+00	23.97	VCAN
1	4.71E-02	1.00E+00	23.97	VCAN
1	4.71E-02	1.00E+00	23.97	ACAN
1	4.71E-02	1.00E+00	23.97	ACAN
1	4.71E-02	1.00E+00	23.97	ACAN
1	4.71E-02	1.00E+00	23.97	ADIPOQ
2	4.74E-02	1.00E+00	6.06	STEAP3, UBA52
3	5.01E-02	1.00E+00	3.80	COL1A1, COL1A2, GYPA
3	5.28E-02	1.00E+00	3.72	H3-3A, H4C1, UBA52
1	5.28E-02	1.00E+00	20.97	ITGA2
1	5.28E-02	1.00E+00	20.97	UBA52
1	5.28E-02	1.00E+00	20.97	UBA52
1	5.28E-02	1.00E+00	20.97	GP9
1	5.28E-02	1.00E+00	20.97	UBA52

1	5.28E-02	1.00E+00	20.97	SLC4A1
1	5.28E-02	1.00E+00	20.97	UBA52
2	5.34E-02	1.00E+00	5.65	H3-3A, H4C1
2	5.65E-02	1.00E+00	5.47	H3-3A, H4C1
1	5.85E-02	1.00E+00	18.64	UBA52
1	5.85E-02	1.00E+00	18.64	UBA52
1	5.85E-02	1.00E+00	18.64	SLC4A1
2	5.97E-02	1.00E+00	5.30	H3-3A, H4C1
2	6.13E-02	1.00E+00	5.21	H3-3A, H4C1
2	6.13E-02	1.00E+00	5.21	H3-3A, H4C1
2	6.29E-02	1.00E+00	5.13	H3-3A, H4C1
1	6.42E-02	1.00E+00	16.77	VCAN
1	6.42E-02	1.00E+00	16.77	UBA52
1	6.42E-02	1.00E+00	16.77	UBA52
1	6.42E-02	1.00E+00	16.77	CLTCL1
1	6.42E-02	1.00E+00	16.77	UBA52
1	6.42E-02	1.00E+00	16.77	UBA52
3	6.53E-02	1.00E+00	3.39	COL7A1, SPTA1, SPTB
2	6.62E-02	1.00E+00	4.98	H3-3A, H4C1
3	6.94E-02	1.00E+00	3.30	H3-3A, H4C1, UBA52
2	6.95E-02	1.00E+00	4.84	H3-3A, H4C1
2	6.95E-02	1.00E+00	4.84	H3-3A, H4C1
1	6.98E-02	1.00E+00	15.25	UBA52
1	6.98E-02	1.00E+00	15.25	CLTCL1
1	6.98E-02	1.00E+00	15.25	UBA52
2	7.12E-02	1.00E+00	4.77	H3-3A, H4C1
4	7.33E-02	1.00E+00	2.65	COL1A1, COL1A2, GNB4, GP9
1	7.54E-02	1.00E+00	13.98	UBA52
1	7.54E-02	1.00E+00	13.98	SLC4A1
1	7.54E-02	1.00E+00	13.98	SLC4A1
1	7.54E-02	1.00E+00	13.98	UBA52
1	7.54E-02	1.00E+00	13.98	UBA52
2	7.63E-02	1.00E+00	4.58	H3-3A, H4C1
2	7.81E-02	1.00E+00	4.51	H4C1, UBA52
7	7.93E-02	1.00E+00	1.98	COL1A1, COL1A2, GNB4, GP9, GYPA, H3-3A, ITGA2
2	7.98E-02	1.00E+00	4.45	H4C1, UBA52
1	8.10E-02	1.00E+00	12.90	VCAN
1	8.10E-02	1.00E+00	12.90	UBA52
1	8.10E-02	1.00E+00	12.90	UBA52
1	8.10E-02	1.00E+00	12.90	UBA52
1	8.65E-02	1.00E+00	11.98	UBA52
1	8.65E-02	1.00E+00	11.98	UBA52
1	8.65E-02	1.00E+00	11.98	UBA52
1	8.65E-02	1.00E+00	11.98	ACAN
1	8.65E-02	1.00E+00	11.98	UBA52
1	8.65E-02	1.00E+00	11.98	UBA52
1	8.65E-02	1.00E+00	11.98	UBA52

1	8.65E-02	1.00E+00	11.98	UBA52
1	8.65E-02	1.00E+00	11.98	UBA52
2	9.06E-02	1.00E+00	4.13	ADIPOQ, PPARG
1	9.20E-02	1.00E+00	11.18	UBA52
1	9.20E-02	1.00E+00	11.18	UBA52
1	9.20E-02	1.00E+00	11.18	UBA52
1	9.20E-02	1.00E+00	11.18	UBA52
1	9.20E-02	1.00E+00	11.18	UBA52
1	9.20E-02	1.00E+00	11.18	UBA52
1	9.20E-02	1.00E+00	11.18	GP9
1	9.20E-02	1.00E+00	11.18	UBA52
1	9.20E-02	1.00E+00	11.18	UBA52
1	9.20E-02	1.00E+00	11.18	UBA52
1	9.20E-02	1.00E+00	11.18	UBA52
2	9.24E-02	1.00E+00	4.08	H3-3A, H4C1
1	9.74E-02	1.00E+00	10.48	UBA52
1	9.74E-02	1.00E+00	10.48	UBA52
1	9.74E-02	1.00E+00	10.48	UBA52
3	9.99E-02	1.00E+00	2.81	COL7A1, SPTA1, SPTB
2	9.99E-02	1.00E+00	3.89	H3-3A, H4C1
3	1.01E-01	1.00E+00	2.79	H3-3A, H4C1, UBA52
2	1.02E-01	1.00E+00	3.84	H3-3A, H4C1
2	1.02E-01	1.00E+00	3.84	H3-3A, H4C1
2	1.02E-01	1.00E+00	3.84	H3-3A, H4C1
1	1.03E-01	1.00E+00	9.86	UBA52
1	1.03E-01	1.00E+00	9.86	GNB4
1	1.03E-01	1.00E+00	9.86	UBA52
2	1.06E-01	1.00E+00	3.76	H3-3A, H4C1
4	1.06E-01	1.00E+00	2.31	MASP1, MASP2, MBL2, UBA52
1	1.08E-01	1.00E+00	9.31	UBA52
1	1.08E-01	1.00E+00	9.31	GNB4
1	1.08E-01	1.00E+00	9.31	UBA52
1	1.08E-01	1.00E+00	9.31	UBA52
1	1.08E-01	1.00E+00	9.31	PLA2G7
1	1.08E-01	1.00E+00	9.31	UBA52
2	1.09E-01	1.00E+00	3.67	H3-3A, H4C1
2	1.09E-01	1.00E+00	3.67	H3-3A, H4C1
4	1.10E-01	1.00E+00	2.28	COL7A1, SPTA1, SPTB, UBA52
1	1.14E-01	1.00E+00	8.82	VCAN
1	1.14E-01	1.00E+00	8.82	VCAN
1	1.14E-01	1.00E+00	8.82	VCAN
1	1.14E-01	1.00E+00	8.82	STEAP3
1	1.14E-01	1.00E+00	8.82	VCAN
1	1.14E-01	1.00E+00	8.82	GNB4
1	1.14E-01	1.00E+00	8.82	GNB4
1	1.14E-01	1.00E+00	8.82	UBA52
3	1.15E-01	1.00E+00	2.63	H3-3A, H4C1, UBA52

2	1.15E-01	1.00E+00	3.56	H4C1, UBA52
1	1.19E-01	1.00E+00	8.38	UBA52
1	1.19E-01	1.00E+00	8.38	UBA52
1	1.19E-01	1.00E+00	8.38	GNB4
1	1.19E-01	1.00E+00	8.38	UBA52
2	1.23E-01	1.00E+00	3.41	SPTA1, SPTB
1	1.24E-01	1.00E+00	7.98	UBA52
1	1.24E-01	1.00E+00	7.98	UBA52
1	1.24E-01	1.00E+00	7.98	UBA52
1	1.24E-01	1.00E+00	7.98	UBA52
1	1.24E-01	1.00E+00	7.98	GNB4
1	1.24E-01	1.00E+00	7.98	UBA52
1	1.30E-01	1.00E+00	7.62	UBA52
1	1.30E-01	1.00E+00	7.62	UBA52
1	1.30E-01	1.00E+00	7.62	UBA52
1	1.30E-01	1.00E+00	7.62	GP9
2	1.31E-01	1.00E+00	3.28	H3-3A, H4C1
2	1.33E-01	1.00E+00	3.25	CLTCL1, UBA52
2	1.33E-01	1.00E+00	3.25	H3-3A, H4C1
1	1.35E-01	1.00E+00	7.28	COL1A1
1	1.35E-01	1.00E+00	7.28	UBA52
1	1.35E-01	1.00E+00	7.28	GNB4
1	1.35E-01	1.00E+00	7.28	GNB4
1	1.35E-01	1.00E+00	7.28	UBA52
2	1.37E-01	1.00E+00	3.19	H3-3A, H4C1
2	1.37E-01	1.00E+00	3.19	H3-3A, H4C1
2	1.37E-01	1.00E+00	3.19	H3-3A, H4C1
2	1.37E-01	1.00E+00	3.19	ADIPOQ, GNB4
4	1.38E-01	1.00E+00	2.09	GNB4, H3-3A, H4C1, UBA52
1	1.40E-01	1.00E+00	6.98	GNB4
1	1.40E-01	1.00E+00	6.98	UBA52
1	1.40E-01	1.00E+00	6.98	UBA52
1	1.40E-01	1.00E+00	6.98	UBA52
1	1.40E-01	1.00E+00	6.98	GNB4
1	1.40E-01	1.00E+00	6.98	UBA52
1	1.40E-01	1.00E+00	6.98	UBA52
1	1.40E-01	1.00E+00	6.98	UBA52
2	1.41E-01	1.00E+00	3.13	H3-3A, H4C1
2	1.43E-01	1.00E+00	3.10	H3-3A, H4C1
1	1.45E-01	1.00E+00	6.70	VCAN
1	1.45E-01	1.00E+00	6.70	UBA52
1	1.45E-01	1.00E+00	6.70	UBA52
2	1.46E-01	1.00E+00	3.07	H3-3A, H4C1
2	1.48E-01	1.00E+00	3.04	H3-3A, H4C1
3	1.49E-01	1.00E+00	2.32	GNB4, H3-3A, H4C1
1	1.50E-01	1.00E+00	6.44	UBA52
1	1.50E-01	1.00E+00	6.44	UBA52

1	1.55E-01	1.00E+00	6.20	UBA52
1	1.55E-01	1.00E+00	6.20	ACAN
1	1.55E-01	1.00E+00	6.20	LAMA2
1	1.55E-01	1.00E+00	6.20	GNB4
2	1.56E-01	1.00E+00	2.93	H3-3A, H4C1
2	1.56E-01	1.00E+00	2.93	H3-3A, H4C1
1	1.61E-01	1.00E+00	5.98	UBA52
1	1.61E-01	1.00E+00	5.98	UBA52
1	1.61E-01	1.00E+00	5.98	UBA52
1	1.61E-01	1.00E+00	5.98	GNB4
1	1.61E-01	1.00E+00	5.98	GNB4
1	1.61E-01	1.00E+00	5.98	GNB4
1	1.61E-01	1.00E+00	5.98	UBA52
3	1.63E-01	1.00E+00	2.22	H3-3A, H4C1, UBA52
2	1.64E-01	1.00E+00	2.83	COL1A1, UBA52
2	1.64E-01	1.00E+00	2.83	H3-3A, H4C1
2	1.64E-01	1.00E+00	2.83	H3-3A, H4C1
1	1.66E-01	1.00E+00	5.77	PPARG
1	1.66E-01	1.00E+00	5.77	UBA52
1	1.66E-01	1.00E+00	5.77	UBA52
1	1.66E-01	1.00E+00	5.77	UBA52
1	1.66E-01	1.00E+00	5.77	UBA52
1	1.66E-01	1.00E+00	5.77	UBA52
3	1.67E-01	1.00E+00	2.19	H3-3A, H4C1, UBA52
3	1.69E-01	1.00E+00	2.18	H3-3A, H4C1, UBA52
1	1.71E-01	1.00E+00	5.58	UBA52
1	1.71E-01	1.00E+00	5.58	UBA52
1	1.71E-01	1.00E+00	5.58	UBA52
1	1.71E-01	1.00E+00	5.58	STEAP3
1	1.71E-01	1.00E+00	5.58	UBA52
2	1.75E-01	1.00E+00	2.72	ACAN, VCAN
1	1.76E-01	1.00E+00	5.40	GNB4
1	1.76E-01	1.00E+00	5.40	COL1A1
1	1.76E-01	1.00E+00	5.40	UBA52
1	1.76E-01	1.00E+00	5.40	UBA52
1	1.76E-01	1.00E+00	5.40	GNB4
1	1.76E-01	1.00E+00	5.40	GNB4
1	1.76E-01	1.00E+00	5.40	UBA52
1	1.81E-01	1.00E+00	5.23	UBA52
1	1.81E-01	1.00E+00	5.23	COL7A1
1	1.81E-01	1.00E+00	5.23	UBA52
1	1.81E-01	1.00E+00	5.23	UBA52
1	1.81E-01	1.00E+00	5.23	GNB4
1	1.81E-01	1.00E+00	5.23	GNB4
1	1.81E-01	1.00E+00	5.23	GNB4
1	1.86E-01	1.00E+00	5.07	UBA52
1	1.86E-01	1.00E+00	5.07	UBA52

1	1.86E-01	1.00E+00	5.07	UBA52
2	1.88E-01	1.00E+00	2.59	H4C1, UBA52
3	1.90E-01	1.00E+00	2.06	ACAN, UBA52, VCAN
1	1.90E-01	1.00E+00	4.92	UBA52
1	1.90E-01	1.00E+00	4.92	GNB4
1	1.90E-01	1.00E+00	4.92	UBA52
1	1.90E-01	1.00E+00	4.92	UBA52
1	1.90E-01	1.00E+00	4.92	UBA52
1	1.90E-01	1.00E+00	4.92	UBA52
1	1.95E-01	1.00E+00	4.78	UBA52
1	1.95E-01	1.00E+00	4.78	UBA52
1	1.95E-01	1.00E+00	4.78	ACAN
2	1.99E-01	1.00E+00	2.49	H3-3A, H4C1
1	2.00E-01	1.00E+00	4.65	UBA52
1	2.00E-01	1.00E+00	4.65	UBA52
1	2.00E-01	1.00E+00	4.65	SPTLC3
1	2.00E-01	1.00E+00	4.65	UBA52
1	2.00E-01	1.00E+00	4.65	UBA52
2	2.01E-01	1.00E+00	2.48	H4C1, UBA52
1	2.05E-01	1.00E+00	4.52	UBA52
1	2.05E-01	1.00E+00	4.52	UBA52
1	2.05E-01	1.00E+00	4.52	GNB4
2	2.06E-01	1.00E+00	2.44	PPARG, UBA52
2	2.06E-01	1.00E+00	2.44	H3-3A, H4C1
2	2.08E-01	1.00E+00	2.42	H3-3A, H4C1
1	2.10E-01	1.00E+00	4.40	UBA52
1	2.10E-01	1.00E+00	4.40	UBA52
1	2.10E-01	1.00E+00	4.40	GP9
2	2.14E-01	1.00E+00	2.37	ACAN, VCAN
1	2.15E-01	1.00E+00	4.29	UBA52
1	2.15E-01	1.00E+00	4.29	UBA52
2	2.19E-01	1.00E+00	2.34	CLTCL1, UBA52
2	2.19E-01	1.00E+00	2.34	GNB4, UBA52
1	2.19E-01	1.00E+00	4.18	UBA52
1	2.19E-01	1.00E+00	4.18	UBA52
1	2.24E-01	1.00E+00	4.08	STEAP3
1	2.24E-01	1.00E+00	4.08	GNB4
1	2.24E-01	1.00E+00	4.08	UBA52
1	2.24E-01	1.00E+00	4.08	UBA52
2	2.25E-01	1.00E+00	2.29	H3-3A, H4C1
1	2.29E-01	1.00E+00	3.98	UBA52
1	2.29E-01	1.00E+00	3.98	UBA52
1	2.29E-01	1.00E+00	3.98	GNB4
1	2.29E-01	1.00E+00	3.98	GNB4
1	2.29E-01	1.00E+00	3.98	GNB4
1	2.29E-01	1.00E+00	3.98	GNB4
1	2.29E-01	1.00E+00	3.98	UBA52

1	2.33E-01	1.00E+00	3.89	STEAP3
2	2.37E-01	1.00E+00	2.21	H3-3A, H4C1
3	2.37E-01	1.00E+00	1.83	SPTA1, SPTB, UBA52
1	2.38E-01	1.00E+00	3.80	ACTG2
1	2.38E-01	1.00E+00	3.80	UBA52
1	2.38E-01	1.00E+00	3.80	GNB4
4	2.41E-01	1.00E+00	1.64	MASP1, MASP2, MBL2, UBA52
1	2.43E-01	1.00E+00	3.72	UBA52
3	2.46E-01	1.00E+00	1.79	SPTA1, SPTB, UBA52
1	2.47E-01	1.00E+00	3.63	CLTCL1
1	2.47E-01	1.00E+00	3.63	UBA52
1	2.47E-01	1.00E+00	3.63	UBA52
1	2.52E-01	1.00E+00	3.56	UBA52
1	2.52E-01	1.00E+00	3.56	GNB4
1	2.52E-01	1.00E+00	3.56	UBA52
1	2.52E-01	1.00E+00	3.56	UBA52
1	2.56E-01	1.00E+00	3.48	UBA52
1	2.56E-01	1.00E+00	3.48	CLTCL1
1	2.61E-01	1.00E+00	3.41	UBA52
1	2.61E-01	1.00E+00	3.41	VCAN
1	2.61E-01	1.00E+00	3.41	UBA52
1	2.61E-01	1.00E+00	3.41	UBA52
1	2.61E-01	1.00E+00	3.41	H4C1
1	2.65E-01	1.00E+00	3.34	CLTCL1
1	2.65E-01	1.00E+00	3.34	H4C1
2	2.65E-01	1.00E+00	2.04	H4C1, UBA52
3	2.66E-01	1.00E+00	1.71	GNB4, H3-3A, H4C1
3	2.68E-01	1.00E+00	1.71	ACAN, UBA52, VCAN
2	2.68E-01	1.00E+00	2.02	H4C1, UBA52
1	2.70E-01	1.00E+00	3.28	UBA52
1	2.70E-01	1.00E+00	3.28	UBA52
1	2.70E-01	1.00E+00	3.28	UBA52
1	2.70E-01	1.00E+00	3.28	UBA52
1	2.70E-01	1.00E+00	3.28	UBA52
1	2.70E-01	1.00E+00	3.28	UBA52
1	2.70E-01	1.00E+00	3.28	UBA52
1	2.70E-01	1.00E+00	3.28	GNB4
1	2.74E-01	1.00E+00	3.21	UBA52
1	2.74E-01	1.00E+00	3.21	UBA52
1	2.74E-01	1.00E+00	3.21	UBA52
1	2.78E-01	1.00E+00	3.15	UBA52
1	2.78E-01	1.00E+00	3.15	STEAP3
1	2.78E-01	1.00E+00	3.15	UBA52
1	2.78E-01	1.00E+00	3.15	UBA52
1	2.78E-01	1.00E+00	3.15	UBA52
1	2.83E-01	1.00E+00	3.09	UBA52
1	2.83E-01	1.00E+00	3.09	UBA52

1	2.83E-01	1.00E+00	3.09	UBA52
1	2.83E-01	1.00E+00	3.09	UBA52
1	2.83E-01	1.00E+00	3.09	UBA52
1	2.83E-01	1.00E+00	3.09	H4C1
1	2.83E-01	1.00E+00	3.09	H4C1
1	2.83E-01	1.00E+00	3.09	H4C1
1	2.83E-01	1.00E+00	3.09	STEAP3
2	2.86E-01	1.00E+00	1.93	SLC4A1, UBA52
18	2.87E-01	1.00E+00	1.21	COL1A1, COL1A2, COL2A1, COL6A1, COL6A3, GNB4, H3-3A, H4C
1	2.87E-01	1.00E+00	3.04	UBA52
1	2.87E-01	1.00E+00	3.04	UBA52
1	2.91E-01	1.00E+00	2.98	VCAN
1	2.91E-01	1.00E+00	2.98	UBA52
1	2.91E-01	1.00E+00	2.98	UBA52
1	2.96E-01	1.00E+00	2.93	UBA52
1	2.96E-01	1.00E+00	2.93	UBA52
1	2.96E-01	1.00E+00	2.93	UBA52
1	2.96E-01	1.00E+00	2.93	UBA52
1	2.96E-01	1.00E+00	2.93	UBA52
1	2.96E-01	1.00E+00	2.93	UBA52
1	2.96E-01	1.00E+00	2.93	UBA52
1	3.00E-01	1.00E+00	2.88	UBA52
1	3.00E-01	1.00E+00	2.88	UBA52
1	3.00E-01	1.00E+00	2.88	UBA52
1	3.00E-01	1.00E+00	2.88	STEAP3
1	3.00E-01	1.00E+00	2.88	UBA52
2	3.01E-01	1.00E+00	1.85	H4C1, PPARG
1	3.04E-01	1.00E+00	2.83	UBA52
1	3.04E-01	1.00E+00	2.83	UBA52
1	3.04E-01	1.00E+00	2.83	UBA52
1	3.04E-01	1.00E+00	2.83	UBA52
1	3.04E-01	1.00E+00	2.83	UBA52
1	3.04E-01	1.00E+00	2.83	H4C1
1	3.04E-01	1.00E+00	2.83	H4C1
1	3.04E-01	1.00E+00	2.83	H4C1
1	3.04E-01	1.00E+00	2.83	UBA52
1	3.04E-01	1.00E+00	2.83	GNB4
1	3.08E-01	1.00E+00	2.78	PPARG
1	3.08E-01	1.00E+00	2.78	UBA52
3	3.10E-01	1.00E+00	1.57	SPTA1, SPTB, UBA52
1	3.12E-01	1.00E+00	2.74	UBA52
1	3.12E-01	1.00E+00	2.74	H4C1
1	3.12E-01	1.00E+00	2.74	GNB4
1	3.12E-01	1.00E+00	2.74	PPARG
2	3.15E-01	1.00E+00	1.79	H4C1, PPARG
2	3.15E-01	1.00E+00	1.79	H3-3A, H4C1
1	3.17E-01	1.00E+00	2.69	UBA52

1	3.17E-01	1.00E+00	2.69	UBA52
1	3.21E-01	1.00E+00	2.65	UBA52
1	3.21E-01	1.00E+00	2.65	UBA52
1	3.21E-01	1.00E+00	2.65	UBA52
1	3.25E-01	1.00E+00	2.61	UBA52
1	3.25E-01	1.00E+00	2.61	UBA52
1	3.29E-01	1.00E+00	2.57	UBA52
1	3.29E-01	1.00E+00	2.57	UBA52
1	3.29E-01	1.00E+00	2.57	UBA52
1	3.29E-01	1.00E+00	2.57	UBA52
5	3.33E-01	1.00E+00	1.34	CLTCL1, COL7A1, SPTA1, SPTB, UBA52
1	3.33E-01	1.00E+00	2.53	UBA52
1	3.33E-01	1.00E+00	2.53	UBA52
1	3.37E-01	1.00E+00	2.49	UBA52
1	3.37E-01	1.00E+00	2.49	H4C1
1	3.37E-01	1.00E+00	2.49	COL7A1
1	3.37E-01	1.00E+00	2.49	UBA52
1	3.37E-01	1.00E+00	2.49	UBA52
1	3.41E-01	1.00E+00	2.45	UBA52
1	3.41E-01	1.00E+00	2.45	H4C1
1	3.45E-01	1.00E+00	2.42	UBA52
1	3.49E-01	1.00E+00	2.38	H4C1
1	3.49E-01	1.00E+00	2.38	UBA52
1	3.49E-01	1.00E+00	2.38	UBA52
1	3.53E-01	1.00E+00	2.35	UBA52
1	3.53E-01	1.00E+00	2.35	UBA52
1	3.53E-01	1.00E+00	2.35	UBA52
1	3.57E-01	1.00E+00	2.32	H4C1
1	3.57E-01	1.00E+00	2.32	H4C1
1	3.57E-01	1.00E+00	2.32	UBA52
1	3.57E-01	1.00E+00	2.32	UBA52
1	3.57E-01	1.00E+00	2.32	UBA52
1	3.57E-01	1.00E+00	2.32	UBA52
1	3.61E-01	1.00E+00	2.29	UBA52
1	3.61E-01	1.00E+00	2.29	UBA52
1	3.65E-01	1.00E+00	2.25	UBA52
1	3.65E-01	1.00E+00	2.25	UBA52
1	3.65E-01	1.00E+00	2.25	UBA52
1	3.68E-01	1.00E+00	2.22	UBA52
1	3.68E-01	1.00E+00	2.22	UBA52
1	3.72E-01	1.00E+00	2.19	GNB4
1	3.72E-01	1.00E+00	2.19	UBA52
1	3.72E-01	1.00E+00	2.19	UBA52
1	3.76E-01	1.00E+00	2.17	UBA52
1	3.76E-01	1.00E+00	2.17	H4C1
1	3.76E-01	1.00E+00	2.17	H4C1
1	3.76E-01	1.00E+00	2.17	GNB4

1	3.80E-01	1.00E+00	2.14	UBA52
1	3.80E-01	1.00E+00	2.14	H4C1
1	3.80E-01	1.00E+00	2.14	UBA52
1	3.84E-01	1.00E+00	2.11	GNB4
1	3.84E-01	1.00E+00	2.11	UBA52
1	3.87E-01	1.00E+00	2.08	UBA52
1	3.91E-01	1.00E+00	2.06	UBA52
1	3.91E-01	1.00E+00	2.06	UBA52
1	3.91E-01	1.00E+00	2.06	UBA52
1	3.91E-01	1.00E+00	2.06	GNB4
1	3.91E-01	1.00E+00	2.06	GNB4
1	3.95E-01	1.00E+00	2.03	UBA52
1	3.95E-01	1.00E+00	2.03	UBA52
1	3.95E-01	1.00E+00	2.03	UBA52
1	3.98E-01	1.00E+00	2.01	UBA52
1	4.02E-01	1.00E+00	1.98	UBA52
1	4.02E-01	1.00E+00	1.98	UBA52
1	4.09E-01	1.00E+00	1.94	UBA52
1	4.09E-01	1.00E+00	1.94	UBA52
1	4.13E-01	1.00E+00	1.91	UBA52
1	4.13E-01	1.00E+00	1.91	UBA52
1	4.13E-01	1.00E+00	1.91	GNB4
1	4.16E-01	1.00E+00	1.89	PLA2G7
1	4.16E-01	1.00E+00	1.89	UBA52
1	4.16E-01	1.00E+00	1.89	UBA52
1	4.20E-01	1.00E+00	1.87	GNB4
1	4.20E-01	1.00E+00	1.87	UBA52
1	4.23E-01	1.00E+00	1.85	H4C1
1	4.23E-01	1.00E+00	1.85	UBA52
1	4.23E-01	1.00E+00	1.85	UBA52
1	4.23E-01	1.00E+00	1.85	UBA52
1	4.23E-01	1.00E+00	1.85	UBA52
1	4.27E-01	1.00E+00	1.83	CLTCL1
1	4.27E-01	1.00E+00	1.83	UBA52
1	4.27E-01	1.00E+00	1.83	UBA52
1	4.27E-01	1.00E+00	1.83	GNB4
1	4.34E-01	1.00E+00	1.79	H4C1
1	4.34E-01	1.00E+00	1.79	H4C1
1	4.34E-01	1.00E+00	1.79	UBA52
1	4.34E-01	1.00E+00	1.79	UBA52
1	4.34E-01	1.00E+00	1.79	UBA52
1	4.37E-01	1.00E+00	1.77	GNB4
1	4.41E-01	1.00E+00	1.75	GNB4
1	4.41E-01	1.00E+00	1.75	UBA52
1	4.44E-01	1.00E+00	1.73	UBA52
1	4.44E-01	1.00E+00	1.73	UBA52
1	4.44E-01	1.00E+00	1.73	UBA52

1	4.47E-01	1.00E+00	1.72	UBA52
1	4.47E-01	1.00E+00	1.72	UBA52
1	4.47E-01	1.00E+00	1.72	UBA52
1	4.47E-01	1.00E+00	1.72	UBA52
1	4.47E-01	1.00E+00	1.72	UBA52
1	4.47E-01	1.00E+00	1.72	SLC4A1
1	4.54E-01	1.00E+00	1.68	UBA52
1	4.57E-01	1.00E+00	1.66	UBA52
1	4.57E-01	1.00E+00	1.66	UBA52
3	4.59E-01	1.00E+00	1.21	H3-3A, H4C1, UBA52
1	4.61E-01	1.00E+00	1.65	UBA52
1	4.61E-01	1.00E+00	1.65	UBA52
1	4.61E-01	1.00E+00	1.65	UBA52
1	4.61E-01	1.00E+00	1.65	UBA52
1	4.64E-01	1.00E+00	1.63	GNB4
1	4.64E-01	1.00E+00	1.63	UBA52
1	4.64E-01	1.00E+00	1.63	UBA52
1	4.67E-01	1.00E+00	1.62	UBA52
1	4.70E-01	1.00E+00	1.60	UBA52
1	4.70E-01	1.00E+00	1.60	UBA52
7	4.72E-01	1.00E+00	1.09	CMTM6, COLEC11, MASP1, MASP2, MBL2, PIGR, UBA52
1	4.74E-01	1.00E+00	1.58	SPTLC3
1	4.74E-01	1.00E+00	1.58	UBA52
1	4.80E-01	1.00E+00	1.55	UBA52
1	4.80E-01	1.00E+00	1.55	COL1A2
1	4.80E-01	1.00E+00	1.55	SLC4A1
2	4.81E-01	1.00E+00	1.25	PPARG, UBA52
13	4.82E-01	1.00E+00	1.05	ACAN, COL1A1, COL1A2, GNB4, GP9, H3-3A, H4C1, MASP1, MAS
1	4.83E-01	1.00E+00	1.54	UBA52
8	4.84E-01	1.00E+00	1.07	COL1A1, COL1A2, H3-3A, H4C1, NR0B1, PPARG, STEAP3, UBA52
13	4.84E-01	1.00E+00	1.05	COL7A1, GNB4, H3-3A, H4C1, LTBP1, PLA2G7, PPARG, SPTA1, SP
1	4.86E-01	1.00E+00	1.53	UBA52
1	4.86E-01	1.00E+00	1.53	UBA52
1	4.89E-01	1.00E+00	1.51	UBA52
1	4.89E-01	1.00E+00	1.51	UBA52
1	4.93E-01	1.00E+00	1.50	UBA52
1	4.93E-01	1.00E+00	1.50	UBA52
1	4.96E-01	1.00E+00	1.48	UBA52
1	4.96E-01	1.00E+00	1.48	UBA52
1	4.96E-01	1.00E+00	1.48	UBA52
1	4.99E-01	1.00E+00	1.47	UBA52
1	4.99E-01	1.00E+00	1.47	UBA52
1	5.02E-01	1.00E+00	1.46	UBA52
1	5.05E-01	1.00E+00	1.45	UBA52
1	5.05E-01	1.00E+00	1.45	UBA52
1	5.08E-01	1.00E+00	1.43	PPARG
1	5.08E-01	1.00E+00	1.43	UBA52

1	5.08E-01	1.00E+00	1.43	UBA52
1	5.14E-01	1.00E+00	1.41	PPARG
1	5.14E-01	1.00E+00	1.41	UBA52
1	5.17E-01	1.00E+00	1.40	UBA52
1	5.17E-01	1.00E+00	1.40	UBA52
1	5.17E-01	1.00E+00	1.40	UBA52
1	5.17E-01	1.00E+00	1.40	UBA52
9	5.23E-01	1.00E+00	1.03	COL7A1, H4C1, LTBP1, PPARG, SPTA1, SPTB, TNC, UBA52, VCAN
1	5.25E-01	1.00E+00	1.36	UBA52
2	5.27E-01	1.00E+00	1.15	H4C1, UBA52
1	5.34E-01	1.00E+00	1.33	UBA52
1	5.48E-01	1.00E+00	1.28	UBA52
1	5.56E-01	1.00E+00	1.25	UBA52
2	5.60E-01	1.00E+00	1.08	PPARG, UBA52
1	5.62E-01	1.00E+00	1.23	UBA52
1	5.64E-01	1.00E+00	1.22	UBA52
1	5.75E-01	1.00E+00	1.18	H4C1
1	5.82E-01	1.00E+00	1.16	UBA52
1	5.82E-01	1.00E+00	1.16	UBA52
1	5.82E-01	1.00E+00	1.16	UBA52
2	5.87E-01	1.00E+00	1.02	H3-3A, H4C1
1	5.90E-01	1.00E+00	1.14	UBA52
1	5.90E-01	1.00E+00	1.14	UBA52
1	5.95E-01	1.00E+00	1.12	UBA52
1	5.95E-01	1.00E+00	1.12	UBA52
8	5.96E-01	1.00E+00	0.96	COL1A1, COL1A2, H3-3A, H4C1, NR0B1, PPARG, STEAP3, UBA52
1	6.00E-01	1.00E+00	1.10	UBA52
2	6.02E-01	1.00E+00	0.99	H4C1, UBA52
1	6.05E-01	1.00E+00	1.09	UBA52
1	6.07E-01	1.00E+00	1.08	UBA52
1	6.10E-01	1.00E+00	1.08	STEAP3
1	6.12E-01	1.00E+00	1.07	UBA52
1	6.14E-01	1.00E+00	1.06	UBA52
1	6.14E-01	1.00E+00	1.06	UBA52
1	6.17E-01	1.00E+00	1.05	GNB4
1	6.21E-01	1.00E+00	1.04	UBA52
1	6.26E-01	1.00E+00	1.03	UBA52
1	6.30E-01	1.00E+00	1.02	UBA52
1	6.35E-01	1.00E+00	1.00	UBA52
1	6.37E-01	1.00E+00	1.00	GNB4
1	6.37E-01	1.00E+00	1.00	GNB4
1	6.39E-01	1.00E+00	0.99	H3-3A
1	6.39E-01	1.00E+00	0.99	UBA52
1	6.44E-01	1.00E+00	0.98	UBA52
2	6.45E-01	1.00E+00	0.92	STEAP3, UBA52
1	6.48E-01	1.00E+00	0.97	UBA52
4	6.52E-01	1.00E+00	0.90	GNB4, SLC4A1, STEAP3, UBA52

3	6.61E-01	1.00E+00	0.89	H3-3A, H4C1, UBA52
1	6.69E-01	1.00E+00	0.91	UBA52
1	6.77E-01	1.00E+00	0.89	UBA52
1	6.79E-01	1.00E+00	0.89	UBA52
1	6.85E-01	1.00E+00	0.87	UBA52
4	6.89E-01	1.00E+00	0.86	COL1A1, COL1A2, COL2A1, UBA52
1	6.98E-01	1.00E+00	0.84	UBA52
1	6.98E-01	1.00E+00	0.84	UBA52
1	7.02E-01	1.00E+00	0.83	UBA52
1	7.11E-01	1.00E+00	0.81	H4C1
1	7.13E-01	1.00E+00	0.81	ACTG2
1	7.13E-01	1.00E+00	0.81	GNB4
5	7.17E-01	1.00E+00	0.83	H4C1, MASP1, MASP2, MBL2, UBA52
1	7.18E-01	1.00E+00	0.80	UBA52
8	7.29E-01	1.00E+00	0.84	COL1A1, COL1A2, H3-3A, H4C1, NR0B1, PPARG, STEAP3, UBA52
1	7.30E-01	1.00E+00	0.77	UBA52
1	7.31E-01	1.00E+00	0.77	GNB4
1	7.33E-01	1.00E+00	0.76	UBA52
1	7.36E-01	1.00E+00	0.76	UBA52
1	7.36E-01	1.00E+00	0.76	UBA52
1	7.61E-01	1.00E+00	0.70	UBA52
1	7.61E-01	1.00E+00	0.70	H4C1
1	7.62E-01	1.00E+00	0.70	UBA52
1	7.67E-01	1.00E+00	0.69	UBA52
1	7.77E-01	1.00E+00	0.67	UBA52
1	7.78E-01	1.00E+00	0.67	SLC4A1
2	7.83E-01	1.00E+00	0.69	COL1A2, UBA52
2	7.90E-01	1.00E+00	0.68	CMTM6, PIGR
6	7.92E-01	1.00E+00	0.76	GNB4, H4C1, MASP1, MASP2, MBL2, UBA52
3	7.93E-01	1.00E+00	0.71	H3-3A, H4C1, UBA52
3	8.05E-01	1.00E+00	0.69	H3-3A, H4C1, STEAP3
1	8.07E-01	1.00E+00	0.61	H4C1
1	8.07E-01	1.00E+00	0.61	H4C1
1	8.07E-01	1.00E+00	0.61	GNB4
1	8.12E-01	1.00E+00	0.60	UBA52
3	8.18E-01	1.00E+00	0.68	H3-3A, H4C1, STEAP3
10	8.20E-01	1.00E+00	0.77	CMTM6, COL1A1, COL1A2, COL2A1, COLEC11, MASP1, MASP2, M
1	8.30E-01	1.00E+00	0.57	H4C1
1	8.32E-01	1.00E+00	0.56	UBA52
1	8.37E-01	1.00E+00	0.55	UBA52
1	8.49E-01	1.00E+00	0.53	UBA52
1	8.57E-01	1.00E+00	0.51	GNB4
3	8.72E-01	1.00E+00	0.60	H3-3A, H4C1, UBA52
3	8.80E-01	1.00E+00	0.59	H3-3A, H4C1, UBA52
1	9.00E-01	1.00E+00	0.43	UBA52
1	9.04E-01	1.00E+00	0.43	UBA52
1	9.19E-01	1.00E+00	0.40	GNB4

1	9.37E-01	1.00E+00	0.36	STEAP3
1	9.40E-01	1.00E+00	0.35	UBA52
1	9.44E-01	1.00E+00	0.34	GNB4
2	9.48E-01	1.00E+00	0.42	PPARG, SPTLC3
2	9.56E-01	1.00E+00	0.40	COL1A2, UBA52
1	9.80E-01	1.00E+00	0.25	GNB4
7	9.81E-01	1.00E+00	0.49	ACAN, ADIPOQ, GNB4, PPARG, SPTLC3, UBA52, VCAN
1	9.88E-01	1.00E+00	0.22	GNB4
1	9.89E-01	1.00E+00	0.22	UBA52

., ITGA2, KRT24, KRT32, KRT85, KRT86, LAMA2, PPARG, RELN, SPTA1, SPTB, UBA52

Table 7. Gene Ontology terms associated with Differentially Expressed Proteins

GO ID	Gene Ontology term	# all proteins	# DEPs	p.val
GO:0045216	cell-cell junction organization	208	15	0.0003
GO:0007043	cell-cell junction assembly	152	11	0.0003
GO:0034329	cell junction assembly	430	19	0.0014
GO:0005102	signaling receptor binding	1565	45	0.0015
GO:0005903	brush border	102	7	0.0028
GO:0098862	cluster of actin-based cell projections	159	7	0.0028
GO:0005178	integrin binding	156	16	0.0043
GO:0030168	platelet activation	133	19	0.0061
GO:0006606	protein import into nucleus	165	4	0.0068
GO:0042306	regulation of protein import into nucleus	59	4	0.0068
GO:0051170	import into nucleus	169	4	0.0068
GO:1990782	protein tyrosine kinase binding	119	4	0.0068
GO:0034330	cell junction organization	705	27	0.0072
GO:0010647	positive regulation of cell communication	1753	38	0.0074
GO:0030010	establishment of cell polarity	150	10	0.0077
GO:0140297	DNA-binding transcription factor binding	476	10	0.0077
GO:0030182	neuron differentiation	1381	22	0.0079
GO:0031346	positive regulation of cell projection organiza	352	8	0.0086
GO:0030048	actin filament-based movement	134	6	0.0088
GO:0032432	actin filament bundle	79	6	0.0088
GO:0070527	platelet aggregation	71	15	0.0101
GO:0023056	positive regulation of signaling	1757	39	0.0105
GO:0048699	generation of neurons	1453	23	0.0126
GO:0008134	transcription factor binding	597	13	0.0129
GO:0007596	blood coagulation	225	26	0.0145
GO:0007599	hemostasis	231	26	0.0145
GO:0050817	coagulation	230	26	0.0145
GO:0051222	positive regulation of protein transport	294	11	0.0162
GO:0022008	neurogenesis	1669	29	0.0162
GO:0005911	cell-cell junction	505	29	0.0162
GO:0007267	cell-cell signaling	1672	21	0.0166
GO:0140096	catalytic activity, acting on a protein	2257	21	0.0166
GO:0042060	wound healing	442	32	0.0175
GO:0007163	establishment or maintenance of cell polarity	234	16	0.0177
GO:0019730	antimicrobial humoral response	125	9	0.0199
GO:0098802	plasma membrane signaling receptor comple	319	9	0.0199
GO:0042277	peptide binding	330	9	0.0199
GO:0048870	cell motility	1791	47	0.0204
GO:0034109	homotypic cell-cell adhesion	95	19	0.0219
GO:0048666	neuron development	1112	19	0.0219
GO:0042581	specific granule	160	19	0.0219
GO:0007229	integrin-mediated signaling pathway	113	14	0.0230
GO:0051223	regulation of protein transport	501	14	0.0230

GO:0072359	circulatory system development	1209	30	0.0231
GO:0050830	defense response to Gram-positive bacterium	118	7	0.0237
GO:0002532	production of molecular mediator involved ir	100	3	0.0241
GO:0007597	blood coagulation, intrinsic pathway	9	3	0.0241
GO:0010976	positive regulation of neuron projection deve	157	3	0.0241
GO:0030219	megakaryocyte differentiation	66	3	0.0241
GO:0035855	megakaryocyte development	22	3	0.0241
GO:0042307	positive regulation of protein import into nuc	40	3	0.0241
GO:0043433	negative regulation of DNA-binding transcript	190	3	0.0241
GO:0048762	mesenchymal cell differentiation	252	3	0.0241
GO:0050714	positive regulation of protein secretion	129	3	0.0241
GO:0051220	cytoplasmic sequestering of protein	22	3	0.0241
GO:0060485	mesenchyme development	313	3	0.0241
GO:0072376	protein activation cascade	20	3	0.0241
GO:0072378	blood coagulation, fibrin clot formation	17	3	0.0241
GO:0120192	tight junction assembly	75	3	0.0241
GO:0120193	tight junction organization	81	3	0.0241
GO:0016460	myosin II complex	26	3	0.0241
GO:0070160	tight junction	132	3	0.0241
GO:0090665	glycoprotein complex	22	3	0.0241
GO:1990779	glycoprotein Ib-IX-V complex	5	3	0.0241
GO:0009611	response to wounding	577	33	0.0245
GO:0006886	intracellular protein transport	868	22	0.0254
GO:0035295	tube development	1154	22	0.0254
GO:0009967	positive regulation of signal transduction	1559	36	0.0255
GO:0048468	cell development	2155	39	0.0262
GO:0035633	maintenance of blood-brain barrier	35	5	0.0264
GO:0070252	actin-mediated cell contraction	104	5	0.0264
GO:1903036	positive regulation of response to wounding	77	5	0.0264
GO:0001725	stress fiber	70	5	0.0264
GO:0030018	Z disc	130	5	0.0264
GO:0031092	platelet alpha granule membrane	17	5	0.0264
GO:0042641	actomyosin	78	5	0.0264
GO:0043296	apical junction complex	151	5	0.0264
GO:0097517	contractile actin filament bundle	70	5	0.0264
GO:0071705	nitrogen compound transport	2025	45	0.0268
GO:0000902	cell morphogenesis	1018	25	0.0282
GO:0022603	regulation of anatomical structure morphoge	987	25	0.0282
GO:0030030	cell projection organization	1548	25	0.0282
GO:0050900	leukocyte migration	398	25	0.0282
GO:0051235	maintenance of location	342	12	0.0297
GO:0043235	receptor complex	544	12	0.0297
GO:0010646	regulation of cell communication	3464	61	0.0319
GO:0070161	anchoring junction	1330	61	0.0319
GO:0005615	extracellular space	3652	153	0.0327
GO:0001568	blood vessel development	784	20	0.0334
GO:0006351	DNA-templated transcription	3557	34	0.0334

GO:0006355	regulation of DNA-templated transcription	3443	34	0.0334
GO:0032774	RNA biosynthetic process	3576	34	0.0334
GO:0097659	nucleic acid-templated transcription	3559	34	0.0334
GO:1903506	regulation of nucleic acid-templated transcrip	3445	34	0.0334
GO:2001141	regulation of RNA biosynthetic process	3454	34	0.0334
GO:0005887	integral component of plasma membrane	1713	34	0.0334
GO:0071702	organic substance transport	2466	52	0.0342
GO:0015031	protein transport	1564	37	0.0342
GO:0016477	cell migration	1595	46	0.0349
GO:0098609	cell-cell adhesion	957	43	0.0349
GO:0031344	regulation of cell projection organization	659	15	0.0376
GO:0051090	regulation of DNA-binding transcription facto	454	15	0.0376
GO:0120035	regulation of plasma membrane bounded cel	642	15	0.0376
GO:1903034	regulation of response to wounding	172	10	0.0382
GO:0045177	apical part of cell	435	10	0.0382
GO:0033218	amide binding	408	10	0.0382
GO:0030667	secretory granule membrane	313	26	0.0399
GO:0007155	cell adhesion	1539	56	0.0423
GO:0045296	cadherin binding	333	32	0.0435
GO:0001525	angiogenesis	607	18	0.0438
GO:0031175	neuron projection development	967	18	0.0438
GO:0031589	cell-substrate adhesion	369	18	0.0438
GO:0042470	melanosome	110	18	0.0438
GO:0048770	pigment granule	110	18	0.0438
GO:0023052	signaling	6622	95	0.0444
GO:0009719	response to endogenous stimulus	1637	35	0.0445
GO:0031226	intrinsic component of plasma membrane	1794	35	0.0445
GO:0007166	cell surface receptor signaling pathway	2907	47	0.0447
GO:0050839	cell adhesion molecule binding	558	47	0.0447
GO:0001775	cell activation	1153	44	0.0451
GO:0007165	signal transduction	6120	92	0.0472
GO:0001944	vasculature development	818	21	0.0486
GO:0018193	peptidyl-amino acid modification	1287	21	0.0486
GO:0035239	tube morphogenesis	943	21	0.0486
GO:0010720	positive regulation of cell development	307	8	0.0488
GO:0010811	positive regulation of cell-substrate adhesion	125	8	0.0488
GO:0022898	regulation of transmembrane transporter act	291	8	0.0488
GO:0032409	regulation of transporter activity	326	8	0.0488
GO:0035580	specific granule lumen	62	8	0.0488
GO:0043025	neuronal cell body	497	8	0.0488
GO:0006914	autophagy	554	13	0.0493
GO:0032989	cellular component morphogenesis	768	13	0.0493
GO:0061919	process utilizing autophagic mechanism	554	13	0.0493
GO:0023051	regulation of signaling	3477	63	0.0494
GO:0005576	extracellular region	4648	159	0.0504
GO:0120036	plasma membrane bounded cell projection o	1509	24	0.0521
GO:0009653	anatomical structure morphogenesis	2790	57	0.0527

GO:0007154	cell communication	6682	96	0.0533
GO:0030054	cell junction	2112	73	0.0536
GO:0071944	cell periphery	6372	127	0.0546
GO:0010243	response to organonitrogen compound	1054	27	0.0547
GO:0048646	anatomical structure formation involved in m	1242	27	0.0547
GO:1901701	cellular response to oxygen-containing comp	1220	30	0.0564
GO:0051649	establishment of localization in cell	2108	48	0.0564
GO:0031410	cytoplasmic vesicle	2479	93	0.0566
GO:0097708	intracellular vesicle	2482	93	0.0566
GO:0003007	heart morphogenesis	258	6	0.0618
GO:0030032	lamellipodium assembly	73	6	0.0618
GO:0031532	actin cytoskeleton reorganization	111	6	0.0618
GO:0046822	regulation of nucleocytoplasmic transport	109	6	0.0618
GO:0051208	sequestering of calcium ion	131	6	0.0618
GO:0051209	release of sequestered calcium ion into cytos	124	6	0.0618
GO:0051282	regulation of sequestering of calcium ion	127	6	0.0618
GO:0051283	negative regulation of sequestering of calciur	125	6	0.0618
GO:0060048	cardiac muscle contraction	140	6	0.0618
GO:0097553	calcium ion transmembrane import into cyto	182	6	0.0618
GO:0031674	I band	143	6	0.0618
GO:0048514	blood vessel morphogenesis	701	19	0.0636
GO:0007507	heart development	607	11	0.0647
GO:0044877	protein-containing complex binding	1299	55	0.0668
GO:0043230	extracellular organelle	2308	146	0.0672
GO:0065010	extracellular membrane-bounded organelle	2308	146	0.0672
GO:0070062	extracellular exosome	2210	146	0.0672
GO:1903561	extracellular vesicle	2307	146	0.0672
GO:0030029	actin filament-based process	821	25	0.0709
GO:1901700	response to oxygen-containing compound	1691	40	0.0738
GO:0012506	vesicle membrane	1209	40	0.0738
GO:0030659	cytoplasmic vesicle membrane	1190	40	0.0738
GO:0050878	regulation of body fluid levels	390	31	0.0740
GO:1902533	positive regulation of intracellular signal tran	1013	31	0.0740
GO:0007160	cell-matrix adhesion	238	14	0.0756
GO:0042742	defense response to bacterium	382	14	0.0756
GO:0000303	response to superoxide	28	4	0.0760
GO:0000305	response to oxygen radical	29	4	0.0760
GO:0002011	morphogenesis of an epithelial sheet	60	4	0.0760
GO:0006809	nitric oxide biosynthetic process	76	4	0.0760
GO:0007030	Golgi organization	165	4	0.0760
GO:0007156	homophilic cell adhesion via plasma membra	170	4	0.0760
GO:0010572	positive regulation of platelet activation	10	4	0.0760
GO:0010591	regulation of lamellipodium assembly	42	4	0.0760
GO:0010592	positive regulation of lamellipodium assembl	29	4	0.0760
GO:0019430	removal of superoxide radicals	23	4	0.0760
GO:0034614	cellular response to reactive oxygen species	150	4	0.0760
GO:0044319	wound healing, spreading of cells	37	4	0.0760

GO:0046209	nitric oxide metabolic process	82	4	0.0760
GO:0048675	axon extension	122	4	0.0760
GO:0048813	dendrite morphogenesis	140	4	0.0760
GO:0050708	regulation of protein secretion	257	4	0.0760
GO:0050829	defense response to Gram-negative bacteriu	89	4	0.0760
GO:0055094	response to lipoprotein particle	34	4	0.0760
GO:0060560	developmental growth involved in morphoge	233	4	0.0760
GO:0071402	cellular response to lipoprotein particle stimu	37	4	0.0760
GO:0071404	cellular response to low-density lipoprotein p	22	4	0.0760
GO:0071450	cellular response to oxygen radical	25	4	0.0760
GO:0071451	cellular response to superoxide	25	4	0.0760
GO:0086003	cardiac muscle cell contraction	76	4	0.0760
GO:0090303	positive regulation of wound healing	62	4	0.0760
GO:0090504	epiboly	38	4	0.0760
GO:0090505	epiboly involved in wound healing	37	4	0.0760
GO:1902743	regulation of lamellipodium organization	54	4	0.0760
GO:1902745	positive regulation of lamellipodium organiza	37	4	0.0760
GO:1990138	neuron projection extension	172	4	0.0760
GO:2001057	reactive nitrogen species metabolic process	83	4	0.0760
GO:0008305	integrin complex	31	4	0.0760
GO:0016459	myosin complex	56	4	0.0760
GO:0043197	dendritic spine	175	4	0.0760
GO:0044309	neuron spine	176	4	0.0760
GO:0016247	channel regulator activity	145	4	0.0760
GO:0099106	ion channel regulator activity	140	4	0.0760
GO:0010256	endomembrane system organization	594	17	0.0832
GO:0051668	localization within membrane	679	17	0.0832
GO:0071417	cellular response to organonitrogen compou	656	17	0.0832
GO:0072657	protein localization to membrane	586	17	0.0832
GO:0030312	external encapsulating structure	574	17	0.0832
GO:0031012	extracellular matrix	573	17	0.0832
GO:0051641	cellular localization	3351	82	0.0836
GO:0001763	morphogenesis of a branching structure	203	2	0.0844
GO:0001837	epithelial to mesenchymal transition	169	2	0.0844
GO:0002028	regulation of sodium ion transport	92	2	0.0844
GO:0002536	respiratory burst involved in inflammatory re	8	2	0.0844
GO:0002679	respiratory burst involved in defense respons	15	2	0.0844
GO:0003151	outflow tract morphogenesis	80	2	0.0844
GO:0006473	protein acetylation	230	2	0.0844
GO:0006475	internal protein amino acid acetylation	188	2	0.0844
GO:0006814	sodium ion transport	255	2	0.0844
GO:0007586	digestion	139	2	0.0844
GO:0008038	neuron recognition	48	2	0.0844
GO:0008347	glial cell migration	57	2	0.0844
GO:0010927	cellular component assembly involved in mor	119	2	0.0844
GO:0016570	histone modification	474	2	0.0844
GO:0016573	histone acetylation	180	2	0.0844

GO:0018393	internal peptidyl-lysine acetylation	186	2	0.0844
GO:0018394	peptidyl-lysine acetylation	197	2	0.0844
GO:0019835	cytolysis	25	2	0.0844
GO:0022412	cellular process involved in reproduction in m	430	2	0.0844
GO:0022600	digestive system process	106	2	0.0844
GO:0030177	positive regulation of Wnt signaling pathway	144	2	0.0844
GO:0030323	respiratory tube development	191	2	0.0844
GO:0030324	lung development	187	2	0.0844
GO:0032024	positive regulation of insulin secretion	68	2	0.0844
GO:0035606	peptidyl-cysteine S-trans-nitrosylation	2	2	0.0844
GO:0035725	sodium ion transmembrane transport	184	2	0.0844
GO:0042744	hydrogen peroxide catabolic process	30	2	0.0844
GO:0043171	peptide catabolic process	33	2	0.0844
GO:0043543	protein acylation	272	2	0.0844
GO:0043967	histone H4 acetylation	74	2	0.0844
GO:0045429	positive regulation of nitric oxide biosyntheti	42	2	0.0844
GO:0045445	myoblast differentiation	112	2	0.0844
GO:0046847	filopodium assembly	69	2	0.0844
GO:0046887	positive regulation of hormone secretion	123	2	0.0844
GO:0048167	regulation of synaptic plasticity	207	2	0.0844
GO:0050892	intestinal absorption	42	2	0.0844
GO:0051489	regulation of filopodium assembly	51	2	0.0844
GO:0051491	positive regulation of filopodium assembly	31	2	0.0844
GO:0051645	Golgi localization	14	2	0.0844
GO:0051683	establishment of Golgi localization	8	2	0.0844
GO:0060541	respiratory system development	211	2	0.0844
GO:0070391	response to lipoteichoic acid	10	2	0.0844
GO:0070830	bicellular tight junction assembly	69	2	0.0844
GO:0071223	cellular response to lipoteichoic acid	10	2	0.0844
GO:0072009	nephron epithelium development	116	2	0.0844
GO:0072073	kidney epithelium development	146	2	0.0844
GO:0072583	clathrin-dependent endocytosis	47	2	0.0844
GO:0090263	positive regulation of canonical Wnt signaling	108	2	0.0844
GO:0090277	positive regulation of peptide hormone secre	92	2	0.0844
GO:1902305	regulation of sodium ion transmembrane tra	69	2	0.0844
GO:1904407	positive regulation of nitric oxide metabolic p	44	2	0.0844
GO:2000249	regulation of actin cytoskeleton reorganizatio	42	2	0.0844
GO:2000649	regulation of sodium ion transmembrane tra	60	2	0.0844
GO:0000123	histone acetyltransferase complex	91	2	0.0844
GO:0005923	bicellular tight junction	127	2	0.0844
GO:0016328	lateral plasma membrane	64	2	0.0844
GO:0031248	protein acetyltransferase complex	101	2	0.0844
GO:0031526	brush border membrane	57	2	0.0844
GO:0031594	neuromuscular junction	68	2	0.0844
GO:0035577	azurophil granule membrane	58	2	0.0844
GO:0042583	chromaffin granule	13	2	0.0844
GO:0042584	chromaffin granule membrane	9	2	0.0844

GO:0042734	presynaptic membrane	148	2	0.0844
GO:0044295	axonal growth cone	23	2	0.0844
GO:0098685	Schaffer collateral - CA1 synapse	72	2	0.0844
GO:1902493	acetyltransferase complex	101	2	0.0844
GO:1902562	H4 histone acetyltransferase complex	41	2	0.0844
GO:0000146	microfilament motor activity	37	2	0.0844
GO:0003774	cytoskeletal motor activity	111	2	0.0844
GO:0004197	cysteine-type endopeptidase activity	120	2	0.0844
GO:0005159	insulin-like growth factor receptor binding	16	2	0.0844
GO:0043495	protein-membrane adaptor activity	28	2	0.0844
GO:0048156	tau protein binding	43	2	0.0844
GO:0001508	action potential	146	9	0.0850
GO:0010543	regulation of platelet activation	52	9	0.0850
GO:0010975	regulation of neuron projection development	446	9	0.0850
GO:0030031	cell projection assembly	575	9	0.0850
GO:0033157	regulation of intracellular protein transport	225	9	0.0850
GO:0061041	regulation of wound healing	137	9	0.0850
GO:0061564	axon development	490	9	0.0850
GO:0120031	plasma membrane bounded cell projection a	564	9	0.0850
GO:0008233	peptidase activity	601	9	0.0850
GO:0044325	transmembrane transporter binding	128	9	0.0850
GO:0000904	cell morphogenesis involved in differentiatio	724	20	0.0883
GO:0045785	positive regulation of cell adhesion	484	20	0.0883
GO:0003008	system process	2333	32	0.0948
GO:0006959	humoral immune response	320	12	0.0996
GO:0032990	cell part morphogenesis	666	12	0.0996
GO:0048667	cell morphogenesis involved in neuron differ	564	12	0.0996
GO:0048812	neuron projection morphogenesis	626	12	0.0996
GO:0048858	cell projection morphogenesis	647	12	0.0996
GO:0072659	protein localization to plasma membrane	285	12	0.0996
GO:0120039	plasma membrane bounded cell projection m	642	12	0.0996
GO:0008104	protein localization	2469	67	0.1046
GO:0070727	cellular macromolecule localization	2479	67	0.1046
GO:0043408	regulation of MAPK cascade	694	15	0.1089
GO:0060284	regulation of cell development	516	15	0.1089
GO:0098655	cation transmembrane transport	948	15	0.1089
GO:0098662	inorganic cation transmembrane transport	837	15	0.1089
GO:0045184	establishment of protein localization	1671	45	0.1105
GO:0006941	striated muscle contraction	181	7	0.1125
GO:0007268	chemical synaptic transmission	727	7	0.1125
GO:0016236	macroautophagy	317	7	0.1125
GO:0030193	regulation of blood coagulation	70	7	0.1125
GO:0031032	actomyosin structure organization	204	7	0.1125
GO:0032412	regulation of ion transmembrane transporter	281	7	0.1125
GO:0035088	establishment or maintenance of apical/basa	55	7	0.1125
GO:0043270	positive regulation of ion transport	289	7	0.1125
GO:0043491	protein kinase B signaling	218	7	0.1125

GO:0045766	positive regulation of angiogenesis	187	7	0.1125
GO:0048589	developmental growth	652	7	0.1125
GO:0050804	modulation of chemical synaptic transmissio	448	7	0.1125
GO:0061245	establishment or maintenance of bipolar cell	55	7	0.1125
GO:0061844	antimicrobial humoral immune response me	81	7	0.1125
GO:0097581	lamellipodium organization	92	7	0.1125
GO:0098916	anterograde trans-synaptic signaling	727	7	0.1125
GO:0099177	regulation of trans-synaptic signaling	449	7	0.1125
GO:0099537	trans-synaptic signaling	734	7	0.1125
GO:1900046	regulation of hemostasis	72	7	0.1125
GO:1904018	positive regulation of vasculature developme	187	7	0.1125
GO:0030674	protein-macromolecule adaptor activity	347	7	0.1125
GO:0002376	immune system process	2892	74	0.1132
GO:0033036	macromolecule localization	2946	74	0.1132
GO:0009966	regulation of signal transduction	3075	58	0.1172
GO:0080134	regulation of response to stress	1431	36	0.1174
GO:0070201	regulation of establishment of protein localiz	526	21	0.1181
GO:0099503	secretory vesicle	1041	71	0.1182
GO:0006897	endocytosis	657	24	0.1199
GO:0031982	vesicle	4146	168	0.1239
GO:0006913	nucleocytoplasmic transport	329	10	0.1319
GO:0051169	nuclear transport	329	10	0.1319
GO:0051651	maintenance of location in cell	226	10	0.1319
GO:0060047	heart contraction	250	10	0.1319
GO:0071216	cellular response to biotic stimulus	261	10	0.1319
GO:0071219	cellular response to molecule of bacterial ori	234	10	0.1319
GO:0071222	cellular response to lipopolysaccharide	222	10	0.1319
GO:1904062	regulation of cation transmembrane transpo	394	10	0.1319
GO:1990266	neutrophil migration	130	10	0.1319
GO:0060090	molecular adaptor activity	492	10	0.1319
GO:0016021	integral component of membrane	5397	59	0.1384
GO:0032496	response to lipopolysaccharide	339	13	0.1427
GO:0051604	protein maturation	336	13	0.1427
GO:0061061	muscle structure development	688	13	0.1427
GO:0097529	myeloid leukocyte migration	242	13	0.1427
GO:0030017	sarcomere	214	13	0.1427
GO:0030141	secretory granule	872	69	0.1439
GO:0032880	regulation of protein localization	842	34	0.1467
GO:0000165	MAPK cascade	792	16	0.1488
GO:0006909	phagocytosis	314	16	0.1488
GO:0008015	blood circulation	524	16	0.1488
GO:0045597	positive regulation of cell differentiation	915	16	0.1488
GO:0062023	collagen-containing extracellular matrix	433	16	0.1488
GO:0030155	regulation of cell adhesion	803	31	0.1492
GO:1901698	response to nitrogen compound	1142	31	0.1492
GO:0000122	negative regulation of transcription by RNA p	956	5	0.1503
GO:0000302	response to reactive oxygen species	204	5	0.1503

GO:0000768	syncytium formation by plasma membrane fu	64	5	0.1503
GO:0006911	phagocytosis, engulfment	127	5	0.1503
GO:0006942	regulation of striated muscle contraction	97	5	0.1503
GO:0006949	syncytium formation	68	5	0.1503
GO:0007520	myoblast fusion	50	5	0.1503
GO:0010770	positive regulation of cell morphogenesis inv	82	5	0.1503
GO:0014902	myotube differentiation	129	5	0.1503
GO:0016358	dendrite development	237	5	0.1503
GO:0023061	signal release	470	5	0.1503
GO:0031663	lipopolysaccharide-mediated signaling pathw	63	5	0.1503
GO:0034764	positive regulation of transmembrane transp	237	5	0.1503
GO:0034767	positive regulation of ion transmembrane tra	186	5	0.1503
GO:0035089	establishment of apical/basal cell polarity	22	5	0.1503
GO:0043547	positive regulation of GTPase activity	283	5	0.1503
GO:0045185	maintenance of protein location	93	5	0.1503
GO:0045664	regulation of neuron differentiation	196	5	0.1503
GO:0046824	positive regulation of nucleocytoplasmic tran	62	5	0.1503
GO:0048588	developmental cell growth	229	5	0.1503
GO:0051896	regulation of protein kinase B signaling	189	5	0.1503
GO:0051897	positive regulation of protein kinase B signali	120	5	0.1503
GO:0055117	regulation of cardiac muscle contraction	78	5	0.1503
GO:0061162	establishment of monopolar cell polarity	24	5	0.1503
GO:0061339	establishment or maintenance of monopolar	25	5	0.1503
GO:0086065	cell communication involved in cardiac condu	61	5	0.1503
GO:0098742	cell-cell adhesion via plasma-membrane adhe	282	5	0.1503
GO:0098751	bone cell development	40	5	0.1503
GO:0098900	regulation of action potential	61	5	0.1503
GO:0099504	synaptic vesicle cycle	192	5	0.1503
GO:0120034	positive regulation of plasma membrane bou	111	5	0.1503
GO:0140253	cell-cell fusion	64	5	0.1503
GO:0150105	protein localization to cell-cell junction	21	5	0.1503
GO:1900026	positive regulation of substrate adhesion-dep	44	5	0.1503
GO:1903532	positive regulation of secretion by cell	278	5	0.1503
GO:2001257	regulation of cation channel activity	197	5	0.1503
GO:0030175	filopodium	108	5	0.1503
GO:0098636	protein complex involved in cell adhesion	52	5	0.1503
GO:0004175	endopeptidase activity	426	5	0.1503
GO:0061629	RNA polymerase II-specific DNA-binding trans	350	5	0.1503
GO:0005773	vacuole	838	28	0.1512
GO:1903829	positive regulation of protein localization	451	25	0.1525
GO:0003013	circulatory system process	609	22	0.1529
GO:0006954	inflammatory response	848	22	0.1529
GO:0009056	catabolic process	2579	50	0.1536
GO:0098588	bounding membrane of organelle	2122	60	0.1618
GO:0005925	focal adhesion	422	44	0.1635
GO:0007399	nervous system development	2495	38	0.1730
GO:0008150	biological_process	18903	221	0.1746

GO:0001501	skeletal system development	541	8	0.1761
GO:0007409	axonogenesis	438	8	0.1761
GO:0008016	regulation of heart contraction	214	8	0.1761
GO:0009306	protein secretion	359	8	0.1761
GO:0030593	neutrophil chemotaxis	106	8	0.1761
GO:0035592	establishment of protein localization to extra	360	8	0.1761
GO:0045123	cellular extravasation	74	8	0.1761
GO:0045892	negative regulation of DNA-templated transc	1302	8	0.1761
GO:0050818	regulation of coagulation	75	8	0.1761
GO:0051017	actin filament bundle assembly	160	8	0.1761
GO:0061572	actin filament bundle organization	164	8	0.1761
GO:0071692	protein localization to extracellular region	368	8	0.1761
GO:0090316	positive regulation of intracellular protein tra	157	8	0.1761
GO:0099536	synaptic signaling	757	8	0.1761
GO:1902679	negative regulation of RNA biosynthetic proc	1306	8	0.1761
GO:1903507	negative regulation of nucleic acid-templated	1304	8	0.1761
GO:0016324	apical plasma membrane	368	8	0.1761
GO:0031301	integral component of organelle membrane	390	8	0.1761
GO:0150034	distal axon	278	8	0.1761
GO:1904724	tertiary granule lumen	55	8	0.1761
GO:0051015	actin filament binding	219	8	0.1761
GO:0045595	regulation of cell differentiation	1639	29	0.1859
GO:0051094	positive regulation of developmental process	1387	29	0.1859
GO:0046983	protein dimerization activity	1070	29	0.1859
GO:0005886	plasma membrane	5891	118	0.1867
GO:0065009	regulation of molecular function	3131	61	0.1874
GO:0031224	intrinsic component of membrane	5564	61	0.1874
GO:0003015	heart process	261	11	0.1879
GO:0097530	granulocyte migration	158	11	0.1879
GO:0005766	primary lysosome	155	11	0.1879
GO:0005819	spindle	426	11	0.1879
GO:0031091	platelet alpha granule	91	11	0.1879
GO:0042582	azurophil granule	155	11	0.1879
GO:0009617	response to bacterium	786	23	0.1922
GO:0098797	plasma membrane protein complex	713	23	0.1922
GO:0008289	lipid binding	831	23	0.1922
GO:0051252	regulation of RNA metabolic process	3754	45	0.1927
GO:0030055	cell-substrate junction	432	45	0.1927
GO:0000226	microtubule cytoskeleton organization	642	14	0.1931
GO:0001667	ameboidal-type cell migration	492	14	0.1931
GO:0002237	response to molecule of bacterial origin	360	14	0.1931
GO:0010810	regulation of cell-substrate adhesion	222	14	0.1931
GO:0022604	regulation of cell morphogenesis	312	14	0.1931
GO:0045893	positive regulation of DNA-templated transcr	1692	14	0.1931
GO:1902680	positive regulation of RNA biosynthetic proce	1698	14	0.1931
GO:1903508	positive regulation of nucleic acid-templated	1692	14	0.1931
GO:1990778	protein localization to cell periphery	337	14	0.1931

GO:0005774	vacuolar membrane	461	14	0.1931
GO:0030016	myofibril	233	14	0.1931
GO:0043292	contractile fiber	242	14	0.1931
GO:0098794	postsynapse	619	14	0.1931
GO:0007159	leukocyte cell-cell adhesion	414	20	0.1942
GO:0042327	positive regulation of phosphorylation	828	20	0.1942
GO:0005509	calcium ion binding	710	20	0.1942
GO:0006357	regulation of transcription by RNA polymeras	2581	17	0.1947
GO:0006366	transcription by RNA polymerase II	2677	17	0.1947
GO:0098660	inorganic ion transmembrane transport	930	17	0.1947
GO:1904951	positive regulation of establishment of prote	310	17	0.1947
GO:0030139	endocytic vesicle	343	17	0.1947
GO:1902531	regulation of intracellular signal transduction	1766	42	0.1990
GO:0048731	system development	4454	78	0.2007
GO:0000082	G1/S transition of mitotic cell cycle	250	3	0.2049
GO:0002523	leukocyte migration involved in inflammatory	19	3	0.2049
GO:0002793	positive regulation of peptide secretion	94	3	0.2049
GO:0006813	potassium ion transport	247	3	0.2049
GO:0006910	phagocytosis, recognition	103	3	0.2049
GO:0007272	ensheathment of neurons	144	3	0.2049
GO:0008366	axon ensheathment	144	3	0.2049
GO:0009914	hormone transport	300	3	0.2049
GO:0016241	regulation of macroautophagy	160	3	0.2049
GO:0017156	calcium-ion regulated exocytosis	66	3	0.2049
GO:0018205	peptidyl-lysine modification	414	3	0.2049
GO:0030072	peptide hormone secretion	225	3	0.2049
GO:0030073	insulin secretion	183	3	0.2049
GO:0030194	positive regulation of blood coagulation	28	3	0.2049
GO:0031099	regeneration	192	3	0.2049
GO:0031664	regulation of lipopolysaccharide-mediated sig	28	3	0.2049
GO:0032506	cytokinetic process	40	3	0.2049
GO:0035023	regulation of Rho protein signal transduction	88	3	0.2049
GO:0038061	NIK/NF-kappaB signaling	142	3	0.2049
GO:0042552	myelination	142	3	0.2049
GO:0043266	regulation of potassium ion transport	114	3	0.2049
GO:0044843	cell cycle G1/S phase transition	280	3	0.2049
GO:0045428	regulation of nitric oxide biosynthetic proces	62	3	0.2049
GO:0046578	regulation of Ras protein signal transduction	193	3	0.2049
GO:0046879	hormone secretion	291	3	0.2049
GO:0046883	regulation of hormone secretion	234	3	0.2049
GO:0050679	positive regulation of epithelial cell proliferat	224	3	0.2049
GO:0050768	negative regulation of neurogenesis	146	3	0.2049
GO:0050796	regulation of insulin secretion	149	3	0.2049
GO:0050820	positive regulation of coagulation	30	3	0.2049
GO:0051056	regulation of small GTPase mediated signal tr	305	3	0.2049
GO:0051238	sequestering of metal ion	26	3	0.2049
GO:0051293	establishment of spindle localization	54	3	0.2049

GO:0051653	spindle localization	59	3	0.2049
GO:0051961	negative regulation of nervous system develo	151	3	0.2049
GO:0071805	potassium ion transmembrane transport	223	3	0.2049
GO:0072006	nephron development	152	3	0.2049
GO:0080164	regulation of nitric oxide metabolic process	65	3	0.2049
GO:0086002	cardiac muscle cell action potential involved	54	3	0.2049
GO:0086004	regulation of cardiac muscle cell contraction	34	3	0.2049
GO:0090276	regulation of peptide hormone secretion	179	3	0.2049
GO:0090330	regulation of platelet aggregation	30	3	0.2049
GO:0098581	detection of external biotic stimulus	25	3	0.2049
GO:1900048	positive regulation of hemostasis	28	3	0.2049
GO:1901222	regulation of NIK/NF-kappaB signaling	111	3	0.2049
GO:1901379	regulation of potassium ion transmembrane	99	3	0.2049
GO:1903115	regulation of actin filament-based movement	40	3	0.2049
GO:0000786	nucleosome	133	3	0.2049
GO:0043202	lysosomal lumen	98	3	0.2049
GO:0043204	perikaryon	154	3	0.2049
GO:0045171	intercellular bridge	89	3	0.2049
GO:0099240	intrinsic component of synaptic membrane	166	3	0.2049
GO:0099699	integral component of synaptic membrane	155	3	0.2049
GO:0004672	protein kinase activity	565	3	0.2049
GO:0004860	protein kinase inhibitor activity	70	3	0.2049
GO:0008234	cysteine-type peptidase activity	185	3	0.2049
GO:0008235	metalloexopeptidase activity	66	3	0.2049
GO:0008237	metallopeptidase activity	184	3	0.2049
GO:0008238	exopeptidase activity	98	3	0.2049
GO:0008379	thioredoxin peroxidase activity	7	3	0.2049
GO:0008514	organic anion transmembrane transporter ac	190	3	0.2049
GO:0016773	phosphotransferase activity, alcohol group as	672	3	0.2049
GO:0019210	kinase inhibitor activity	74	3	0.2049
GO:0035325	Toll-like receptor binding	12	3	0.2049
GO:0050815	phosphoserine residue binding	10	3	0.2049
GO:0051920	peroxiredoxin activity	8	3	0.2049
GO:0071949	FAD binding	39	3	0.2049
GO:0097718	disordered domain specific binding	35	3	0.2049
GO:0140824	thioredoxin-dependent peroxiredoxin activity	7	3	0.2049
GO:0060429	epithelium development	1204	39	0.2053
GO:0048856	anatomical structure development	5933	112	0.2070
GO:0042802	identical protein binding	1997	52	0.2093
GO:0040011	locomotion	1445	36	0.2116
GO:0060341	regulation of cellular localization	955	36	0.2116
GO:0009987	cellular process	17266	213	0.2125
GO:0051050	positive regulation of transport	904	33	0.2178
GO:0098590	plasma membrane region	1250	33	0.2178
GO:0007275	multicellular organism development	4922	82	0.2180
GO:0008092	cytoskeletal protein binding	993	30	0.2239
GO:0048583	regulation of response to stimulus	4131	79	0.2273

GO:0055085	transmembrane transport	1575	27	0.2299
GO:0071495	cellular response to endogenous stimulus	1387	27	0.2299
GO:0019904	protein domain specific binding	693	27	0.2299
GO:0000280	nuclear division	481	6	0.2389
GO:0001701	in utero embryonic development	393	6	0.2389
GO:0001885	endothelial cell development	65	6	0.2389
GO:0007266	Rho protein signal transduction	140	6	0.2389
GO:0007517	muscle organ development	348	6	0.2389
GO:0010324	membrane invagination	143	6	0.2389
GO:0010769	regulation of cell morphogenesis involved in	100	6	0.2389
GO:0031345	negative regulation of cell projection organiz	194	6	0.2389
GO:0034121	regulation of toll-like receptor signaling path	77	6	0.2389
GO:0043087	regulation of GTPase activity	376	6	0.2389
GO:0043123	positive regulation of I-kappaB kinase/NF-ka	189	6	0.2389
GO:0043534	blood vessel endothelial cell migration	177	6	0.2389
GO:0045197	establishment or maintenance of epithelial c	51	6	0.2389
GO:0045446	endothelial cell differentiation	121	6	0.2389
GO:0048285	organelle fission	528	6	0.2389
GO:0050767	regulation of neurogenesis	377	6	0.2389
GO:0050877	nervous system process	1541	6	0.2389
GO:0051960	regulation of nervous system development	456	6	0.2389
GO:0060348	bone development	233	6	0.2389
GO:0060491	regulation of cell projection assembly	201	6	0.2389
GO:0061028	establishment of endothelial barrier	48	6	0.2389
GO:0061515	myeloid cell development	83	6	0.2389
GO:0098739	import across plasma membrane	202	6	0.2389
GO:0099003	vesicle-mediated transport in synapse	212	6	0.2389
GO:0099024	plasma membrane invagination	136	6	0.2389
GO:0120032	regulation of plasma membrane bounded cel	199	6	0.2389
GO:0140014	mitotic nuclear division	325	6	0.2389
GO:1900024	regulation of substrate adhesion-dependent	61	6	0.2389
GO:1905475	regulation of protein localization to membra	172	6	0.2389
GO:0005788	endoplasmic reticulum lumen	312	6	0.2389
GO:0005811	lipid droplet	102	6	0.2389
GO:0014704	intercalated disc	50	6	0.2389
GO:0030176	integral component of endoplasmic reticulun	164	6	0.2389
GO:0030426	growth cone	167	6	0.2389
GO:0030427	site of polarized growth	173	6	0.2389
GO:0031227	intrinsic component of endoplasmic reticulur	172	6	0.2389
GO:0032993	protein-DNA complex	225	6	0.2389
GO:0097060	synaptic membrane	378	6	0.2389
GO:0001786	phosphatidylserine binding	61	6	0.2389
GO:0003925	G protein activity	41	6	0.2389
GO:0023023	MHC protein complex binding	36	6	0.2389
GO:0023026	MHC class II protein complex binding	27	6	0.2389
GO:0048306	calcium-dependent protein binding	81	6	0.2389
GO:0072341	modified amino acid binding	94	6	0.2389

GO:0044248	cellular catabolic process	2134	40	0.2402
GO:0010556	regulation of macromolecule biosynthetic pr	4002	53	0.2406
GO:0048584	positive regulation of response to stimulus	2272	53	0.2406
GO:0030036	actin cytoskeleton organization	731	21	0.2410
GO:0061024	membrane organization	881	21	0.2410
GO:1901699	cellular response to nitrogen compound	711	21	0.2410
GO:0048471	perinuclear region of cytoplasm	729	21	0.2410
GO:0070820	tertiary granule	164	21	0.2410
GO:0050794	regulation of cellular process	11335	155	0.2421
GO:0007017	microtubule-based process	925	18	0.2456
GO:0022402	cell cycle process	1256	18	0.2456
GO:0032970	regulation of actin filament-based process	406	18	0.2456
GO:0031090	organelle membrane	3667	73	0.2463
GO:0007009	plasma membrane organization	166	9	0.2490
GO:0042063	gliogenesis	310	9	0.2490
GO:0045765	regulation of angiogenesis	349	9	0.2490
GO:0051656	establishment of organelle localization	437	9	0.2490
GO:0071621	granulocyte chemotaxis	130	9	0.2490
GO:0098657	import into cell	252	9	0.2490
GO:0098869	cellular oxidant detoxification	100	9	0.2490
GO:1903522	regulation of blood circulation	263	9	0.2490
GO:0031300	intrinsic component of organelle membrane	420	9	0.2490
GO:0001894	tissue homeostasis	275	15	0.2492
GO:0036477	somatodendritic compartment	849	15	0.2492
GO:0003779	actin binding	439	15	0.2492
GO:0038023	signaling receptor activity	1531	15	0.2492
GO:0060089	molecular transducer activity	1531	15	0.2492
GO:0006936	muscle contraction	351	12	0.2509
GO:0009887	animal organ morphogenesis	1056	12	0.2509
GO:0032386	regulation of intracellular transport	336	12	0.2509
GO:0042391	regulation of membrane potential	431	12	0.2509
GO:0043410	positive regulation of MAPK cascade	496	12	0.2509
GO:0051091	positive regulation of DNA-binding transcript	266	12	0.2509
GO:0072503	cellular divalent inorganic cation homeostasis	323	12	0.2509
GO:0072507	divalent inorganic cation homeostasis	356	12	0.2509
GO:0050896	response to stimulus	9216	145	0.2567
GO:0009888	tissue development	1990	47	0.2586
GO:0034654	nucleobase-containing compound biosynthet	4013	47	0.2586
GO:0051716	cellular response to stimulus	7653	121	0.2632
GO:0006468	protein phosphorylation	1578	31	0.2650
GO:0098772	molecular function regulator activity	2003	31	0.2650
GO:0016192	vesicle-mediated transport	1621	44	0.2679
GO:0031326	regulation of cellular biosynthetic process	4175	54	0.2739
GO:0050865	regulation of cell activation	727	25	0.2824
GO:0000323	lytic vacuole	746	25	0.2824
GO:0005764	lysosome	746	25	0.2824
GO:0050793	regulation of developmental process	2584	51	0.2841

GO:0046907	intracellular transport	1587	38	0.2875
GO:0006812	cation transport	1219	22	0.2913
GO:0019725	cellular homeostasis	773	22	0.2913
GO:0000079	regulation of cyclin-dependent protein serine	117	1	0.2920
GO:0000132	establishment of mitotic spindle orientation	33	1	0.2920
GO:0000212	meiotic spindle organization	15	1	0.2920
GO:0000413	protein peptidyl-prolyl isomerization	31	1	0.2920
GO:0000724	double-strand break repair via homologous re	169	1	0.2920
GO:0001657	ureteric bud development	97	1	0.2920
GO:0001658	branching involved in ureteric bud morphoge	60	1	0.2920
GO:0001659	temperature homeostasis	178	1	0.2920
GO:0001678	cellular glucose homeostasis	158	1	0.2920
GO:0001708	cell fate specification	109	1	0.2920
GO:0001709	cell fate determination	44	1	0.2920
GO:0001755	neural crest cell migration	59	1	0.2920
GO:0001767	establishment of lymphocyte polarity	16	1	0.2920
GO:0001768	establishment of T cell polarity	15	1	0.2920
GO:0001776	leukocyte homeostasis	100	1	0.2920
GO:0001823	mesonephros development	102	1	0.2920
GO:0001974	blood vessel remodeling	48	1	0.2920
GO:0002038	positive regulation of L-glutamate import acr	4	1	0.2920
GO:0002052	positive regulation of neuroblast proliferation	28	1	0.2920
GO:0002159	desmosome assembly	4	1	0.2920
GO:0002230	positive regulation of defense response to vi	31	1	0.2920
GO:0002232	leukocyte chemotaxis involved in inflammato	8	1	0.2920
GO:0002260	lymphocyte homeostasis	69	1	0.2920
GO:0002281	macrophage activation involved in immune re	19	1	0.2920
GO:0002357	defense response to tumor cell	13	1	0.2920
GO:0002419	T cell mediated cytotoxicity directed against t	5	1	0.2920
GO:0002485	antigen processing and presentation of endog	2	1	0.2920
GO:0003009	skeletal muscle contraction	44	1	0.2920
GO:0003161	cardiac conduction system development	37	1	0.2920
GO:0003253	cardiac neural crest cell migration involved in	10	1	0.2920
GO:0006289	nucleotide-excision repair	84	1	0.2920
GO:0006406	mRNA export from nucleus	67	1	0.2920
GO:0006476	protein deacetylation	122	1	0.2920
GO:0006479	protein methylation	208	1	0.2920
GO:0006612	protein targeting to membrane	134	1	0.2920
GO:0006706	steroid catabolic process	26	1	0.2920
GO:0006713	glucocorticoid catabolic process	1	1	0.2920
GO:0006880	intracellular sequestering of iron ion	6	1	0.2920
GO:0006907	pinocytosis	22	1	0.2920
GO:0006997	nucleus organization	139	1	0.2920
GO:0006999	nuclear pore organization	14	1	0.2920
GO:0007008	outer mitochondrial membrane organization	13	1	0.2920
GO:0007088	regulation of mitotic nuclear division	118	1	0.2920
GO:0007091	metaphase/anaphase transition of mitotic ce	95	1	0.2920

GO:0007097	nuclear migration	24	1	0.2920
GO:0007161	calcium-independent cell-matrix adhesion	4	1	0.2920
GO:0007195	adenylate cyclase-inhibiting dopamine recept	6	1	0.2920
GO:0007212	dopamine receptor signaling pathway	40	1	0.2920
GO:0007342	fusion of sperm to egg plasma membrane inv	22	1	0.2920
GO:0007343	egg activation	10	1	0.2920
GO:0007413	axonal fasciculation	21	1	0.2920
GO:0007612	learning	150	1	0.2920
GO:0007632	visual behavior	53	1	0.2920
GO:0008039	synaptic target recognition	1	1	0.2920
GO:0008211	glucocorticoid metabolic process	25	1	0.2920
GO:0008213	protein alkylation	208	1	0.2920
GO:0008306	associative learning	85	1	0.2920
GO:0008354	germ cell migration	8	1	0.2920
GO:0008542	visual learning	47	1	0.2920
GO:0009100	glycoprotein metabolic process	395	1	0.2920
GO:0010002	cardioblast differentiation	21	1	0.2920
GO:0010459	negative regulation of heart rate	12	1	0.2920
GO:0010469	regulation of signaling receptor activity	175	1	0.2920
GO:0010507	negative regulation of autophagy	87	1	0.2920
GO:0010544	negative regulation of platelet activation	19	1	0.2920
GO:0010569	regulation of double-strand break repair via h	72	1	0.2920
GO:0010657	muscle cell apoptotic process	92	1	0.2920
GO:0010658	striated muscle cell apoptotic process	57	1	0.2920
GO:0010659	cardiac muscle cell apoptotic process	55	1	0.2920
GO:0010668	ectodermal cell differentiation	7	1	0.2920
GO:0010710	regulation of collagen catabolic process	4	1	0.2920
GO:0010744	positive regulation of macrophage derived fo	19	1	0.2920
GO:0010815	bradykinin catabolic process	7	1	0.2920
GO:0010878	cholesterol storage	21	1	0.2920
GO:0010884	positive regulation of lipid storage	27	1	0.2920
GO:0010885	regulation of cholesterol storage	19	1	0.2920
GO:0010886	positive regulation of cholesterol storage	8	1	0.2920
GO:0010934	macrophage cytokine production	37	1	0.2920
GO:0010935	regulation of macrophage cytokine productio	37	1	0.2920
GO:0010965	regulation of mitotic sister chromatid separat	98	1	0.2920
GO:0014032	neural crest cell development	83	1	0.2920
GO:0014033	neural crest cell differentiation	96	1	0.2920
GO:0014905	myoblast fusion involved in skeletal muscle r	5	1	0.2920
GO:0014908	myotube differentiation involved in skeletal r	9	1	0.2920
GO:0015695	organic cation transport	56	1	0.2920
GO:0015757	galactose transmembrane transport	5	1	0.2920
GO:0015874	norepinephrine transport	22	1	0.2920
GO:0015912	short-chain fatty acid transport	3	1	0.2920
GO:0015920	lipopolysaccharide transport	2	1	0.2920
GO:0016242	negative regulation of macroautophagy	35	1	0.2920
GO:0016339	calcium-dependent cell-cell adhesion via plas	43	1	0.2920

GO:0016479	negative regulation of transcription by RNA p	8	1	0.2920
GO:0016571	histone methylation	167	1	0.2920
GO:0018022	peptidyl-lysine methylation	144	1	0.2920
GO:0018208	peptidyl-proline modification	49	1	0.2920
GO:0019068	virion assembly	36	1	0.2920
GO:0019076	viral release from host cell	29	1	0.2920
GO:0019082	viral protein processing	32	1	0.2920
GO:0019827	stem cell population maintenance	177	1	0.2920
GO:0021535	cell migration in hindbrain	16	1	0.2920
GO:0021675	nerve development	84	1	0.2920
GO:0021799	cerebral cortex radially oriented cell migratio	34	1	0.2920
GO:0021801	cerebral cortex radial glia-guided migration	25	1	0.2920
GO:0021932	hindbrain radial glia guided cell migration	8	1	0.2920
GO:0021943	formation of radial glial scaffolds	3	1	0.2920
GO:0021954	central nervous system neuron development	85	1	0.2920
GO:0021955	central nervous system neuron axonogenesis	37	1	0.2920
GO:0022030	telencephalon glial cell migration	25	1	0.2920
GO:0023035	CD40 signaling pathway	16	1	0.2920
GO:0030033	microvillus assembly	16	1	0.2920
GO:0030035	microspike assembly	4	1	0.2920
GO:0030046	parallel actin filament bundle assembly	7	1	0.2920
GO:0030049	muscle filament sliding	14	1	0.2920
GO:0030071	regulation of mitotic metaphase/anaphase tr	92	1	0.2920
GO:0030101	natural killer cell activation	99	1	0.2920
GO:0030220	platelet formation	20	1	0.2920
GO:0030225	macrophage differentiation	51	1	0.2920
GO:0030239	myofibril assembly	68	1	0.2920
GO:0030299	intestinal cholesterol absorption	19	1	0.2920
GO:0030509	BMP signaling pathway	167	1	0.2920
GO:0030510	regulation of BMP signaling pathway	111	1	0.2920
GO:0030513	positive regulation of BMP signaling pathway	41	1	0.2920
GO:0030516	regulation of axon extension	93	1	0.2920
GO:0030517	negative regulation of axon extension	45	1	0.2920
GO:0030574	collagen catabolic process	45	1	0.2920
GO:0030913	paranodal junction assembly	6	1	0.2920
GO:0031053	primary miRNA processing	16	1	0.2920
GO:0031056	regulation of histone modification	175	1	0.2920
GO:0031058	positive regulation of histone modification	102	1	0.2920
GO:0031060	regulation of histone methylation	81	1	0.2920
GO:0031062	positive regulation of histone methylation	51	1	0.2920
GO:0031102	neuron projection regeneration	62	1	0.2920
GO:0031103	axon regeneration	55	1	0.2920
GO:0031268	pseudopodium organization	17	1	0.2920
GO:0031269	pseudopodium assembly	16	1	0.2920
GO:0031272	regulation of pseudopodium assembly	13	1	0.2920
GO:0031274	positive regulation of pseudopodium assemb	13	1	0.2920
GO:0031958	corticosteroid receptor signaling pathway	14	1	0.2920

GO:0032088	negative regulation of NF-kappaB transcriptio	95	1	0.2920
GO:0032148	activation of protein kinase B activity	31	1	0.2920
GO:0032259	methylation	390	1	0.2920
GO:0032288	myelin assembly	22	1	0.2920
GO:0032387	negative regulation of intracellular transport	62	1	0.2920
GO:0032486	Rap protein signal transduction	14	1	0.2920
GO:0032488	Cdc42 protein signal transduction	15	1	0.2920
GO:0032490	detection of molecule of bacterial origin	12	1	0.2920
GO:0032493	response to bacterial lipoprotein	11	1	0.2920
GO:0032494	response to peptidoglycan	13	1	0.2920
GO:0032528	microvillus organization	24	1	0.2920
GO:0032530	regulation of microvillus organization	13	1	0.2920
GO:0032534	regulation of microvillus assembly	6	1	0.2920
GO:0032722	positive regulation of chemokine production	70	1	0.2920
GO:0032731	positive regulation of interleukin-1 beta prod	63	1	0.2920
GO:0032732	positive regulation of interleukin-1 productio	74	1	0.2920
GO:0032796	uropod organization	2	1	0.2920
GO:0032878	regulation of establishment or maintenance c	28	1	0.2920
GO:0032892	positive regulation of organic acid transport	48	1	0.2920
GO:0033194	response to hydroperoxide	17	1	0.2920
GO:0033275	actin-myosin filament sliding	16	1	0.2920
GO:0033500	carbohydrate homeostasis	249	1	0.2920
GO:0033598	mammary gland epithelial cell proliferation	26	1	0.2920
GO:0033599	regulation of mammary gland epithelial cell p	15	1	0.2920
GO:0033601	positive regulation of mammary gland epithe	6	1	0.2920
GO:0034111	negative regulation of homotypic cell-cell adh	14	1	0.2920
GO:0034162	toll-like receptor 9 signaling pathway	19	1	0.2920
GO:0034163	regulation of toll-like receptor 9 signaling pat	11	1	0.2920
GO:0034165	positive regulation of toll-like receptor 9 sign	6	1	0.2920
GO:0034196	acylglycerol transport	8	1	0.2920
GO:0034197	triglyceride transport	8	1	0.2920
GO:0034389	lipid droplet organization	32	1	0.2920
GO:0034968	histone lysine methylation	130	1	0.2920
GO:0034975	protein folding in endoplasmic reticulum	11	1	0.2920
GO:0035024	negative regulation of Rho protein signal trar	23	1	0.2920
GO:0035025	positive regulation of Rho protein signal trans	34	1	0.2920
GO:0035050	embryonic heart tube development	84	1	0.2920
GO:0035196	miRNA processing	57	1	0.2920
GO:0035313	wound healing, spreading of epidermal cells	22	1	0.2920
GO:0035601	protein deacylation	133	1	0.2920
GO:0035634	response to stilbenoid	6	1	0.2920
GO:0035683	memory T cell extravasation	2	1	0.2920
GO:0035709	memory T cell activation	4	1	0.2920
GO:0035740	CD8-positive, alpha-beta T cell proliferation	8	1	0.2920
GO:0035773	insulin secretion involved in cellular response	59	1	0.2920
GO:0035774	positive regulation of insulin secretion involv	27	1	0.2920
GO:0035878	nail development	10	1	0.2920

GO:0035891	exit from host cell	29	1	0.2920
GO:0036037	CD8-positive, alpha-beta T cell activation	27	1	0.2920
GO:0036344	platelet morphogenesis	22	1	0.2920
GO:0038129	ERBB3 signaling pathway	5	1	0.2920
GO:0038189	neuropilin signaling pathway	4	1	0.2920
GO:0040001	establishment of mitotic spindle localization	38	1	0.2920
GO:0042118	endothelial cell activation	11	1	0.2920
GO:0042159	lipoprotein catabolic process	16	1	0.2920
GO:0042220	response to cocaine	48	1	0.2920
GO:0042246	tissue regeneration	73	1	0.2920
GO:0042268	regulation of cytolysis	4	1	0.2920
GO:0042308	negative regulation of protein import into nu	15	1	0.2920
GO:0042593	glucose homeostasis	248	1	0.2920
GO:0042921	glucocorticoid receptor signaling pathway	13	1	0.2920
GO:0042994	cytoplasmic sequestering of transcription fac	15	1	0.2920
GO:0043029	T cell homeostasis	42	1	0.2920
GO:0043113	receptor clustering	57	1	0.2920
GO:0043268	positive regulation of potassium ion transpor	52	1	0.2920
GO:0043276	anoikis	35	1	0.2920
GO:0043279	response to alkaloid	99	1	0.2920
GO:0043335	protein unfolding	3	1	0.2920
GO:0043392	negative regulation of DNA binding	52	1	0.2920
GO:0043403	skeletal muscle tissue regeneration	39	1	0.2920
GO:0043414	macromolecule methylation	348	1	0.2920
GO:0043550	regulation of lipid kinase activity	68	1	0.2920
GO:0043551	regulation of phosphatidylinositol 3-kinase ac	56	1	0.2920
GO:0043552	positive regulation of phosphatidylinositol 3-	32	1	0.2920
GO:0043589	skin morphogenesis	12	1	0.2920
GO:0043627	response to estrogen	70	1	0.2920
GO:0043968	histone H2A acetylation	23	1	0.2920
GO:0043981	histone H4-K5 acetylation	16	1	0.2920
GO:0043982	histone H4-K8 acetylation	16	1	0.2920
GO:0043984	histone H4-K16 acetylation	21	1	0.2920
GO:0044241	lipid digestion	23	1	0.2920
GO:0044546	NLRP3 inflammasome complex assembly	22	1	0.2920
GO:0044782	cilium organization	391	1	0.2920
GO:0044784	metaphase/anaphase transition of cell cycle	98	1	0.2920
GO:0045023	G0 to G1 transition	41	1	0.2920
GO:0045026	plasma membrane fusion	26	1	0.2920
GO:0045040	protein insertion into mitochondrial outer me	13	1	0.2920
GO:0045112	integrin biosynthetic process	6	1	0.2920
GO:0045113	regulation of integrin biosynthetic process	5	1	0.2920
GO:0045176	apical protein localization	15	1	0.2920
GO:0045214	sarcomere organization	44	1	0.2920
GO:0045581	negative regulation of T cell differentiation	48	1	0.2920
GO:0045620	negative regulation of lymphocyte differentia	58	1	0.2920
GO:0045661	regulation of myoblast differentiation	75	1	0.2920

GO:0045663	positive regulation of myoblast differentiatio	44	1	0.2920
GO:0045747	positive regulation of Notch signaling pathwa	48	1	0.2920
GO:0045793	positive regulation of cell size	11	1	0.2920
GO:0045822	negative regulation of heart contraction	26	1	0.2920
GO:0045823	positive regulation of heart contraction	40	1	0.2920
GO:0045834	positive regulation of lipid metabolic process	154	1	0.2920
GO:0045919	positive regulation of cytolysis	1	1	0.2920
GO:0045988	negative regulation of striated muscle contra	8	1	0.2920
GO:0045989	positive regulation of striated muscle contrac	15	1	0.2920
GO:0046579	positive regulation of Ras protein signal trans	63	1	0.2920
GO:0046580	negative regulation of Ras protein signal tran	51	1	0.2920
GO:0046633	alpha-beta T cell proliferation	47	1	0.2920
GO:0046640	regulation of alpha-beta T cell proliferation	44	1	0.2920
GO:0046641	positive regulation of alpha-beta T cell prolife	25	1	0.2920
GO:0046823	negative regulation of nucleocytoplasmic tra	23	1	0.2920
GO:0046929	negative regulation of neurotransmitter secre	12	1	0.2920
GO:0046950	cellular ketone body metabolic process	9	1	0.2920
GO:0046951	ketone body biosynthetic process	6	1	0.2920
GO:0048268	clathrin coat assembly	16	1	0.2920
GO:0048308	organelle inheritance	15	1	0.2920
GO:0048313	Golgi inheritance	15	1	0.2920
GO:0048548	regulation of pinocytosis	12	1	0.2920
GO:0048549	positive regulation of pinocytosis	7	1	0.2920
GO:0048625	myoblast fate commitment	6	1	0.2920
GO:0048626	myoblast fate specification	1	1	0.2920
GO:0048663	neuron fate commitment	68	1	0.2920
GO:0048664	neuron fate determination	10	1	0.2920
GO:0048679	regulation of axon regeneration	31	1	0.2920
GO:0048680	positive regulation of axon regeneration	13	1	0.2920
GO:0048736	appendage development	178	1	0.2920
GO:0048754	branching morphogenesis of an epithelial tub	157	1	0.2920
GO:0048755	branching morphogenesis of a nerve	11	1	0.2920
GO:0048814	regulation of dendrite morphogenesis	66	1	0.2920
GO:0048864	stem cell development	89	1	0.2920
GO:0050770	regulation of axonogenesis	156	1	0.2920
GO:0050771	negative regulation of axonogenesis	65	1	0.2920
GO:0050774	negative regulation of dendrite morphogenes	9	1	0.2920
GO:0050879	multicellular organismal movement	57	1	0.2920
GO:0050881	musculoskeletal movement	57	1	0.2920
GO:0050904	diapedesis	7	1	0.2920
GO:0050905	neuromuscular process	154	1	0.2920
GO:0050909	sensory perception of taste	68	1	0.2920
GO:0050982	detection of mechanical stimulus	51	1	0.2920
GO:0051057	positive regulation of small GTPase mediated	71	1	0.2920
GO:0051058	negative regulation of small GTPase mediate	58	1	0.2920
GO:0051204	protein insertion into mitochondrial membra	33	1	0.2920
GO:0051205	protein insertion into membrane	67	1	0.2920

GO:0051224	negative regulation of protein transport	129	1	0.2920
GO:0051292	nuclear pore complex assembly	9	1	0.2920
GO:0051294	establishment of spindle orientation	40	1	0.2920
GO:0051295	establishment of meiotic spindle localization	5	1	0.2920
GO:0051304	chromosome separation	135	1	0.2920
GO:0051306	mitotic sister chromatid separation	101	1	0.2920
GO:0051321	meiotic cell cycle	285	1	0.2920
GO:0051568	histone H3-K4 methylation	75	1	0.2920
GO:0051569	regulation of histone H3-K4 methylation	39	1	0.2920
GO:0051571	positive regulation of histone H3-K4 methyla	27	1	0.2920
GO:0051582	positive regulation of neurotransmitter uptak	6	1	0.2920
GO:0051589	negative regulation of neurotransmitter trans	15	1	0.2920
GO:0051590	positive regulation of neurotransmitter trans	24	1	0.2920
GO:0051620	norepinephrine uptake	8	1	0.2920
GO:0051621	regulation of norepinephrine uptake	2	1	0.2920
GO:0051623	positive regulation of norepinephrine uptake	1	1	0.2920
GO:0051647	nucleus localization	28	1	0.2920
GO:0051764	actin crosslink formation	14	1	0.2920
GO:0051783	regulation of nuclear division	145	1	0.2920
GO:0051899	membrane depolarization	84	1	0.2920
GO:0051933	amino acid neurotransmitter reuptake	10	1	0.2920
GO:0051935	glutamate reuptake	6	1	0.2920
GO:0051937	catecholamine transport	73	1	0.2920
GO:0051941	regulation of amino acid uptake involved in s	4	1	0.2920
GO:0051943	positive regulation of amino acid uptake invo	2	1	0.2920
GO:0051946	regulation of glutamate uptake involved in tr	4	1	0.2920
GO:0051951	positive regulation of glutamate uptake invol	2	1	0.2920
GO:0051954	positive regulation of amine transport	43	1	0.2920
GO:0051957	positive regulation of amino acid transport	26	1	0.2920
GO:0055002	striated muscle cell development	69	1	0.2920
GO:0055095	lipoprotein particle mediated signaling	3	1	0.2920
GO:0055096	low-density lipoprotein particle mediated sig	3	1	0.2920
GO:0055118	negative regulation of cardiac muscle contrac	6	1	0.2920
GO:0060099	regulation of phagocytosis, engulfment	15	1	0.2920
GO:0060100	positive regulation of phagocytosis, engulfme	13	1	0.2920
GO:0060173	limb development	178	1	0.2920
GO:0060264	regulation of respiratory burst involved in inf	7	1	0.2920
GO:0060265	positive regulation of respiratory burst involv	2	1	0.2920
GO:0060267	positive regulation of respiratory burst	9	1	0.2920
GO:0060271	cilium assembly	362	1	0.2920
GO:0060317	cardiac epithelial to mesenchymal transition	34	1	0.2920
GO:0060352	cell adhesion molecule production	24	1	0.2920
GO:0060372	regulation of atrial cardiac muscle cell memb	8	1	0.2920
GO:0060379	cardiac muscle cell myoblast differentiation	12	1	0.2920
GO:0060425	lung morphogenesis	51	1	0.2920
GO:0060428	lung epithelium development	43	1	0.2920
GO:0060452	positive regulation of cardiac muscle contrac	10	1	0.2920

GO:0060467	negative regulation of fertilization	7	1	0.2920
GO:0060468	prevention of polyspermy	7	1	0.2920
GO:0060471	cortical granule exocytosis	2	1	0.2920
GO:0060501	positive regulation of epithelial cell proliferat	5	1	0.2920
GO:0060502	epithelial cell proliferation involved in lung m	10	1	0.2920
GO:0060675	ureteric bud morphogenesis	66	1	0.2920
GO:0060688	regulation of morphogenesis of a branching s	51	1	0.2920
GO:0060907	positive regulation of macrophage cytokine p	24	1	0.2920
GO:0060911	cardiac cell fate commitment	16	1	0.2920
GO:0060912	cardiac cell fate specification	4	1	0.2920
GO:0060973	cell migration involved in heart development	21	1	0.2920
GO:0060993	kidney morphogenesis	97	1	0.2920
GO:0061081	positive regulation of myeloid leukocyte cyto	34	1	0.2920
GO:0061082	myeloid leukocyte cytokine production	50	1	0.2920
GO:0061138	morphogenesis of a branching epithelium	188	1	0.2920
GO:0061178	regulation of insulin secretion involved in cell	49	1	0.2920
GO:0061180	mammary gland epithelium development	69	1	0.2920
GO:0061307	cardiac neural crest cell differentiation involv	15	1	0.2920
GO:0061308	cardiac neural crest cell development involve	15	1	0.2920
GO:0061309	cardiac neural crest cell development involve	12	1	0.2920
GO:0061326	renal tubule development	99	1	0.2920
GO:0061333	renal tubule morphogenesis	79	1	0.2920
GO:0061387	regulation of extent of cell growth	108	1	0.2920
GO:0061944	negative regulation of protein K48-linked ubi	2	1	0.2920
GO:0061945	regulation of protein K48-linked ubiquitinatio	5	1	0.2920
GO:0070268	cornification	9	1	0.2920
GO:0070316	regulation of G0 to G1 transition	39	1	0.2920
GO:0070339	response to bacterial lipopeptide	9	1	0.2920
GO:0070508	cholesterol import	9	1	0.2920
GO:0070543	response to linoleic acid	3	1	0.2920
GO:0070570	regulation of neuron projection regeneration	34	1	0.2920
GO:0070572	positive regulation of neuron projection rege	14	1	0.2920
GO:0070633	transepithelial transport	33	1	0.2920
GO:0070918	small regulatory ncRNA processing	61	1	0.2920
GO:0070936	protein K48-linked ubiquitination	68	1	0.2920
GO:0071220	cellular response to bacterial lipoprotein	9	1	0.2920
GO:0071221	cellular response to bacterial lipopeptide	9	1	0.2920
GO:0071224	cellular response to peptidoglycan	6	1	0.2920
GO:0071322	cellular response to carbohydrate stimulus	152	1	0.2920
GO:0071326	cellular response to monosaccharide stimulus	140	1	0.2920
GO:0071331	cellular response to hexose stimulus	138	1	0.2920
GO:0071333	cellular response to glucose stimulus	136	1	0.2920
GO:0071447	cellular response to hydroperoxide	8	1	0.2920
GO:0071526	semaphorin-plexin signaling pathway	44	1	0.2920
GO:0071603	endothelial cell-cell adhesion	4	1	0.2920
GO:0071680	response to indole-3-methanol	6	1	0.2920
GO:0071681	cellular response to indole-3-methanol	5	1	0.2920

GO:0071711	basement membrane organization	36	1	0.2920
GO:0071724	response to diacyl bacterial lipopeptide	5	1	0.2920
GO:0071726	cellular response to diacyl bacterial lipopepti	5	1	0.2920
GO:0071772	response to BMP	178	1	0.2920
GO:0071773	cellular response to BMP stimulus	178	1	0.2920
GO:0071867	response to monoamine	108	1	0.2920
GO:0071868	cellular response to monoamine stimulus	105	1	0.2920
GO:0071869	response to catecholamine	108	1	0.2920
GO:0071870	cellular response to catecholamine stimulus	105	1	0.2920
GO:0072010	glomerular epithelium development	26	1	0.2920
GO:0072011	glomerular endothelium development	4	1	0.2920
GO:0072028	nephron morphogenesis	80	1	0.2920
GO:0072078	nephron tubule morphogenesis	75	1	0.2920
GO:0072080	nephron tubule development	96	1	0.2920
GO:0072088	nephron epithelium morphogenesis	77	1	0.2920
GO:0072163	mesonephric epithelium development	98	1	0.2920
GO:0072164	mesonephric tubule development	98	1	0.2920
GO:0072171	mesonephric tubule morphogenesis	67	1	0.2920
GO:0072384	organelle transport along microtubule	88	1	0.2920
GO:0072564	blood microparticle formation	2	1	0.2920
GO:0072749	cellular response to cytochalasin B	1	1	0.2920
GO:0080154	regulation of fertilization	17	1	0.2920
GO:0085029	extracellular matrix assembly	46	1	0.2920
GO:0086010	membrane depolarization during action pote	36	1	0.2920
GO:0086014	atrial cardiac muscle cell action potential	19	1	0.2920
GO:0086019	cell-cell signaling involved in cardiac conducti	37	1	0.2920
GO:0086026	atrial cardiac muscle cell to AV node cell sign	19	1	0.2920
GO:0086066	atrial cardiac muscle cell to AV node cell com	19	1	0.2920
GO:0086100	endothelin receptor signaling pathway	11	1	0.2920
GO:0086101	endothelin receptor signaling pathway involv	3	1	0.2920
GO:0090042	tubulin deacetylation	22	1	0.2920
GO:0090091	positive regulation of extracellular matrix dis	8	1	0.2920
GO:0090128	regulation of synapse maturation	14	1	0.2920
GO:0090156	cellular sphingolipid homeostasis	5	1	0.2920
GO:0090158	endoplasmic reticulum membrane organizati	14	1	0.2920
GO:0090168	Golgi reassembly	4	1	0.2920
GO:0090218	positive regulation of lipid kinase activity	36	1	0.2920
GO:0090313	regulation of protein targeting to membrane	34	1	0.2920
GO:0090314	positive regulation of protein targeting to me	28	1	0.2920
GO:0090317	negative regulation of intracellular protein tr	44	1	0.2920
GO:0090331	negative regulation of platelet aggregation	12	1	0.2920
GO:0090493	catecholamine uptake	18	1	0.2920
GO:0090497	mesenchymal cell migration	61	1	0.2920
GO:0090557	establishment of endothelial intestinal barrie	10	1	0.2920
GO:0097009	energy homeostasis	43	1	0.2920
GO:0097194	execution phase of apoptosis	84	1	0.2920
GO:0097320	plasma membrane tubulation	20	1	0.2920

GO:0097577	sequestering of iron ion	7	1	0.2920
GO:0098727	maintenance of cell number	181	1	0.2920
GO:0098732	macromolecule deacylation	137	1	0.2920
GO:0098814	spontaneous synaptic transmission	14	1	0.2920
GO:0098856	intestinal lipid absorption	23	1	0.2920
GO:0098903	regulation of membrane repolarization durin	10	1	0.2920
GO:0098914	membrane repolarization during atrial cardia	7	1	0.2920
GO:0098974	postsynaptic actin cytoskeleton organization	10	1	0.2920
GO:0099188	postsynaptic cytoskeleton organization	12	1	0.2920
GO:0099623	regulation of cardiac muscle cell membrane r	29	1	0.2920
GO:0099624	atrial cardiac muscle cell membrane repolariz	11	1	0.2920
GO:0106030	neuron projection fasciculation	21	1	0.2920
GO:0106106	cold-induced thermogenesis	147	1	0.2920
GO:0120161	regulation of cold-induced thermogenesis	147	1	0.2920
GO:0120162	positive regulation of cold-induced thermoge	98	1	0.2920
GO:0140052	cellular response to oxidised low-density lipo	12	1	0.2920
GO:0140632	inflammasome complex assembly	24	1	0.2920
GO:0150003	regulation of spontaneous synaptic transmiss	6	1	0.2920
GO:0150024	oxidised low-density lipoprotein particle clea	1	1	0.2920
GO:0150103	reactive gliosis	1	1	0.2920
GO:0150107	positive regulation of protein localization to c	1	1	0.2920
GO:0150111	regulation of transepithelial transport	4	1	0.2920
GO:1900095	regulation of dosage compensation by inactiv	11	1	0.2920
GO:1900181	negative regulation of protein localization to	34	1	0.2920
GO:1900225	regulation of NLRP3 inflammasome complex	20	1	0.2920
GO:1900227	positive regulation of NLRP3 inflammasome c	12	1	0.2920
GO:1900756	protein processing in phagocytic vesicle	2	1	0.2920
GO:1901031	regulation of response to reactive oxygen spe	41	1	0.2920
GO:1901077	regulation of relaxation of muscle	14	1	0.2920
GO:1901186	positive regulation of ERBB signaling pathway	40	1	0.2920
GO:1901328	response to cytochalasin B	1	1	0.2920
GO:1901381	positive regulation of potassium ion transme	45	1	0.2920
GO:1901552	positive regulation of endothelial cell develop	6	1	0.2920
GO:1901568	fatty acid derivative metabolic process	78	1	0.2920
GO:1901570	fatty acid derivative biosynthetic process	50	1	0.2920
GO:1901585	regulation of acid-sensing ion channel activity	1	1	0.2920
GO:1901979	regulation of inward rectifier potassium chan	7	1	0.2920
GO:1902099	regulation of metaphase/anaphase transition	95	1	0.2920
GO:1902176	negative regulation of oxidative stress-induce	19	1	0.2920
GO:1902224	ketone body metabolic process	10	1	0.2920
GO:1902396	protein localization to bicellular tight junction	4	1	0.2920
GO:1902459	positive regulation of stem cell population m	47	1	0.2920
GO:1902692	regulation of neuroblast proliferation	40	1	0.2920
GO:1902806	regulation of cell cycle G1/S phase transition	213	1	0.2920
GO:1902914	regulation of protein polyubiquitination	30	1	0.2920
GO:1902915	negative regulation of protein polyubiquitina	11	1	0.2920
GO:1902947	regulation of tau-protein kinase activity	14	1	0.2920

GO:1902949	positive regulation of tau-protein kinase activ	8	1	0.2920
GO:1903018	regulation of glycoprotein metabolic process	62	1	0.2920
GO:1903020	positive regulation of glycoprotein metabolic	26	1	0.2920
GO:1903046	meiotic cell cycle process	212	1	0.2920
GO:1903055	positive regulation of extracellular matrix org	27	1	0.2920
GO:1903142	positive regulation of establishment of endot	6	1	0.2920
GO:1903202	negative regulation of oxidative stress-induce	55	1	0.2920
GO:1903236	regulation of leukocyte tethering or rolling	17	1	0.2920
GO:1903238	positive regulation of leukocyte tethering or	12	1	0.2920
GO:1903350	response to dopamine	92	1	0.2920
GO:1903351	cellular response to dopamine	91	1	0.2920
GO:1903428	positive regulation of reactive oxygen species	22	1	0.2920
GO:1903523	negative regulation of blood circulation	27	1	0.2920
GO:1903524	positive regulation of blood circulation	41	1	0.2920
GO:1903672	positive regulation of sprouting angiogenesis	28	1	0.2920
GO:1903793	positive regulation of anion transport	51	1	0.2920
GO:1903828	negative regulation of protein localization	203	1	0.2920
GO:1903918	regulation of actin filament severing	1	1	0.2920
GO:1903919	negative regulation of actin filament severing	1	1	0.2920
GO:1903921	regulation of protein processing in phagocyti	2	1	0.2920
GO:1903923	positive regulation of protein processing in p	2	1	0.2920
GO:1903961	positive regulation of anion transmembrane	13	1	0.2920
GO:1904029	regulation of cyclin-dependent protein kinase	120	1	0.2920
GO:1904950	negative regulation of establishment of prote	133	1	0.2920
GO:1905000	regulation of membrane repolarization durin	2	1	0.2920
GO:1905031	regulation of membrane repolarization durin	7	1	0.2920
GO:1905153	regulation of membrane invagination	15	1	0.2920
GO:1905155	positive regulation of membrane invaginatior	13	1	0.2920
GO:1905168	positive regulation of double-strand break re	39	1	0.2920
GO:1905323	telomerase holoenzyme complex assembly	6	1	0.2920
GO:1905550	regulation of protein localization to endoplas	3	1	0.2920
GO:1905552	positive regulation of protein localization to e	2	1	0.2920
GO:1905578	regulation of ERBB3 signaling pathway	1	1	0.2920
GO:1905580	positive regulation of ERBB3 signaling pathwa	1	1	0.2920
GO:1905661	regulation of telomerase RNA reverse transcr	3	1	0.2920
GO:1905663	positive regulation of telomerase RNA revers	2	1	0.2920
GO:1905818	regulation of chromosome separation	111	1	0.2920
GO:1990379	lipid transport across blood-brain barrier	6	1	0.2920
GO:1990428	miRNA transport	3	1	0.2920
GO:1990809	endoplasmic reticulum tubular network mem	4	1	0.2920
GO:2000036	regulation of stem cell population maintenan	71	1	0.2920
GO:2000045	regulation of G1/S transition of mitotic cell cy	185	1	0.2920
GO:2000114	regulation of establishment of cell polarity	25	1	0.2920
GO:2000121	regulation of removal of superoxide radicals	10	1	0.2920
GO:2000172	regulation of branching morphogenesis of a r	5	1	0.2920
GO:2000178	negative regulation of neural precursor cell p	23	1	0.2920
GO:2000209	regulation of anoikis	25	1	0.2920

GO:2000251	positive regulation of actin cytoskeleton reor	19	1	0.2920
GO:2000273	positive regulation of signaling receptor activ	46	1	0.2920
GO:2000301	negative regulation of synaptic vesicle exocyt	6	1	0.2920
GO:2000332	regulation of blood microparticle formation	2	1	0.2920
GO:2000334	positive regulation of blood microparticle for	2	1	0.2920
GO:2000564	regulation of CD8-positive, alpha-beta T cell p	8	1	0.2920
GO:2000566	positive regulation of CD8-positive, alpha-bet	4	1	0.2920
GO:2000567	regulation of memory T cell activation	2	1	0.2920
GO:2000568	positive regulation of memory T cell activatio	2	1	0.2920
GO:2000677	regulation of transcription regulatory region	53	1	0.2920
GO:2000678	negative regulation of transcription regulator	20	1	0.2920
GO:2000779	regulation of double-strand break repair	135	1	0.2920
GO:2000781	positive regulation of double-strand break re	88	1	0.2920
GO:2000785	regulation of autophagosome assembly	45	1	0.2920
GO:2000794	regulation of epithelial cell proliferation invol	7	1	0.2920
GO:2000810	regulation of bicellular tight junction assembl	22	1	0.2920
GO:2000811	negative regulation of anoikis	18	1	0.2920
GO:2000819	regulation of nucleotide-excision repair	28	1	0.2920
GO:2001044	regulation of integrin-mediated signaling pat	22	1	0.2920
GO:2001046	positive regulation of integrin-mediated signa	13	1	0.2920
GO:2001185	regulation of CD8-positive, alpha-beta T cell a	19	1	0.2920
GO:2001187	positive regulation of CD8-positive, alpha-bet	9	1	0.2920
GO:0000322	storage vacuole	2	1	0.2920
GO:0000407	phagophore assembly site	37	1	0.2920
GO:0005790	smooth endoplasmic reticulum	30	1	0.2920
GO:0005797	Golgi medial cisterna	12	1	0.2920
GO:0005826	actomyosin contractile ring	4	1	0.2920
GO:0005833	hemoglobin complex	12	1	0.2920
GO:0005915	zonula adherens	8	1	0.2920
GO:0012510	trans-Golgi network transport vesicle membr	20	1	0.2920
GO:0015030	Cajal body	81	1	0.2920
GO:0016327	apicolateral plasma membrane	21	1	0.2920
GO:0016342	catenin complex	31	1	0.2920
GO:0016461	unconventional myosin complex	8	1	0.2920
GO:0016514	SWI/SNF complex	30	1	0.2920
GO:0016586	RSC-type complex	14	1	0.2920
GO:0017119	Golgi transport complex	11	1	0.2920
GO:0030117	membrane coat	89	1	0.2920
GO:0030118	clathrin coat	44	1	0.2920
GO:0030120	vesicle coat	57	1	0.2920
GO:0030125	clathrin vesicle coat	30	1	0.2920
GO:0030130	clathrin coat of trans-Golgi network vesicle	18	1	0.2920
GO:0030132	clathrin coat of coated pit	14	1	0.2920
GO:0030173	integral component of Golgi membrane	64	1	0.2920
GO:0030315	T-tubule	52	1	0.2920
GO:0030660	Golgi-associated vesicle membrane	57	1	0.2920
GO:0031228	intrinsic component of Golgi membrane	69	1	0.2920

GO:0031523	Myb complex	3	1	0.2920
GO:0032982	myosin filament	24	1	0.2920
GO:0034045	phagophore assembly site membrane	19	1	0.2920
GO:0034665	integrin alpha1-beta1 complex	2	1	0.2920
GO:0034666	integrin alpha2-beta1 complex	3	1	0.2920
GO:0034667	integrin alpha3-beta1 complex	2	1	0.2920
GO:0034668	integrin alpha4-beta1 complex	3	1	0.2920
GO:0034674	integrin alpha5-beta1 complex	2	1	0.2920
GO:0034677	integrin alpha7-beta1 complex	1	1	0.2920
GO:0034678	integrin alpha8-beta1 complex	2	1	0.2920
GO:0034680	integrin alpha10-beta1 complex	2	1	0.2920
GO:0034681	integrin alpha11-beta1 complex	2	1	0.2920
GO:0035060	brahma complex	14	1	0.2920
GO:0035267	NuA4 histone acetyltransferase complex	27	1	0.2920
GO:0035748	myelin sheath abaxonal region	6	1	0.2920
GO:0036019	endolysosome	29	1	0.2920
GO:0036020	endolysosome membrane	20	1	0.2920
GO:0043189	H4/H2A histone acetyltransferase complex	27	1	0.2920
GO:0044292	dendrite terminus	13	1	0.2920
GO:0044294	dendritic growth cone	9	1	0.2920
GO:0044305	calyx of Held	18	1	0.2920
GO:0044322	endoplasmic reticulum quality control compa	25	1	0.2920
GO:0044393	microspike	3	1	0.2920
GO:0044545	NSL complex	11	1	0.2920
GO:0048475	coated membrane	89	1	0.2920
GO:0048787	presynaptic active zone membrane	29	1	0.2920
GO:0060473	cortical granule	6	1	0.2920
GO:0070603	SWI/SNF superfamily-type complex	90	1	0.2920
GO:0070938	contractile ring	10	1	0.2920
GO:0070971	endoplasmic reticulum exit site	33	1	0.2920
GO:0071439	clathrin complex	6	1	0.2920
GO:0071564	npBAF complex	14	1	0.2920
GO:0071565	nBAF complex	16	1	0.2920
GO:0071664	catenin-TCF7L2 complex	4	1	0.2920
GO:0071665	gamma-catenin-TCF7L2 complex	1	1	0.2920
GO:0097226	sperm mitochondrial sheath	5	1	0.2920
GO:0097386	glial cell projection	38	1	0.2920
GO:0097433	dense body	5	1	0.2920
GO:0097513	myosin II filament	3	1	0.2920
GO:0097524	sperm plasma membrane	14	1	0.2920
GO:0098686	hippocampal mossy fiber to CA3 synapse	34	1	0.2920
GO:0098826	endoplasmic reticulum tubular network mem	5	1	0.2920
GO:0098833	presynaptic endocytic zone	7	1	0.2920
GO:0098835	presynaptic endocytic zone membrane	6	1	0.2920
GO:0098843	postsynaptic endocytic zone	5	1	0.2920
GO:0098871	postsynaptic actin cytoskeleton	4	1	0.2920
GO:0098889	intrinsic component of presynaptic membran	80	1	0.2920

GO:0098936	intrinsic component of postsynaptic membra	123	1	0.2920
GO:0098945	intrinsic component of presynaptic active zor	17	1	0.2920
GO:0099023	vesicle tethering complex	62	1	0.2920
GO:0099055	integral component of postsynaptic membra	118	1	0.2920
GO:0099056	integral component of presynaptic membran	73	1	0.2920
GO:0099059	integral component of presynaptic active zor	14	1	0.2920
GO:0099571	postsynaptic cytoskeleton	9	1	0.2920
GO:0099631	postsynaptic endocytic zone cytoplasmic com	2	1	0.2920
GO:0099738	cell cortex region	39	1	0.2920
GO:0140092	bBAF complex	10	1	0.2920
GO:0140288	GBAF complex	14	1	0.2920
GO:1903958	nitric-oxide synthase complex	3	1	0.2920
GO:1904949	ATPase complex	94	1	0.2920
GO:0001648	proteinase-activated receptor activity	5	1	0.2920
GO:0001653	peptide receptor activity	153	1	0.2920
GO:0002134	UTP binding	3	1	0.2920
GO:0002135	CTP binding	2	1	0.2920
GO:0003755	peptidyl-prolyl cis-trans isomerase activity	38	1	0.2920
GO:0003796	lysozyme activity	10	1	0.2920
GO:0004128	cytochrome-b5 reductase activity, acting on I	7	1	0.2920
GO:0004177	aminopeptidase activity	39	1	0.2920
GO:0004180	carboxypeptidase activity	44	1	0.2920
GO:0004181	metallocarboxypeptidase activity	29	1	0.2920
GO:0004190	aspartic-type endopeptidase activity	26	1	0.2920
GO:0004365	glyceraldehyde-3-phosphate dehydrogenase	2	1	0.2920
GO:0004553	hydrolase activity, hydrolyzing O-glycosyl cor	94	1	0.2920
GO:0004674	protein serine/threonine kinase activity	430	1	0.2920
GO:0004713	protein tyrosine kinase activity	138	1	0.2920
GO:0004743	pyruvate kinase activity	2	1	0.2920
GO:0004930	G protein-coupled receptor activity	868	1	0.2920
GO:0005342	organic acid transmembrane transporter acti	166	1	0.2920
GO:0005354	galactose transmembrane transporter activit	4	1	0.2920
GO:0008028	monocarboxylic acid transmembrane transpo	60	1	0.2920
GO:0008426	protein kinase C inhibitor activity	3	1	0.2920
GO:0008429	phosphatidylethanolamine binding	10	1	0.2920
GO:0008528	G protein-coupled peptide receptor activity	147	1	0.2920
GO:0015057	thrombin-activated receptor activity	5	1	0.2920
GO:0015245	fatty acid transmembrane transporter activit	10	1	0.2920
GO:0015636	short-chain fatty acid transmembrane transp	3	1	0.2920
GO:0015643	toxic substance binding	10	1	0.2920
GO:0016018	cyclosporin A binding	20	1	0.2920
GO:0016405	CoA-ligase activity	27	1	0.2920
GO:0016653	oxidoreductase activity, acting on NAD(P)H, h	10	1	0.2920
GO:0016661	oxidoreductase activity, acting on other nitro	11	1	0.2920
GO:0016662	oxidoreductase activity, acting on other nitro	5	1	0.2920
GO:0016769	transferase activity, transferring nitrogenous	25	1	0.2920
GO:0016798	hydrolase activity, acting on glycosyl bonds	132	1	0.2920

GO:0016859	cis-trans isomerase activity	41	1	0.2920
GO:0016877	ligase activity, forming carbon-sulfur bonds	41	1	0.2920
GO:0016878	acid-thiol ligase activity	31	1	0.2920
GO:0016922	nuclear receptor binding	139	1	0.2920
GO:0016971	flavin-linked sulfhydryl oxidase activity	3	1	0.2920
GO:0016972	thiol oxidase activity	6	1	0.2920
GO:0017025	TBP-class protein binding	26	1	0.2920
GO:0017080	sodium channel regulator activity	40	1	0.2920
GO:0017098	sulfonylurea receptor binding	3	1	0.2920
GO:0017166	vinculin binding	12	1	0.2920
GO:0019103	pyrimidine nucleotide binding	7	1	0.2920
GO:0019828	aspartic-type endopeptidase inhibitor activity	7	1	0.2920
GO:0019894	kinesin binding	46	1	0.2920
GO:0030274	LIM domain binding	7	1	0.2920
GO:0030291	protein serine/threonine kinase inhibitor acti	33	1	0.2920
GO:0030729	acetoacetate-CoA ligase activity	1	1	0.2920
GO:0030742	GTP-dependent protein binding	22	1	0.2920
GO:0030881	beta-2-microglobulin binding	8	1	0.2920
GO:0030911	TPR domain binding	6	1	0.2920
GO:0030955	potassium ion binding	12	1	0.2920
GO:0030971	receptor tyrosine kinase binding	77	1	0.2920
GO:0031420	alkali metal ion binding	17	1	0.2920
GO:0031996	thioesterase binding	11	1	0.2920
GO:0032036	myosin heavy chain binding	14	1	0.2920
GO:0032427	GBD domain binding	2	1	0.2920
GO:0032552	deoxyribonucleotide binding	5	1	0.2920
GO:0032554	purine deoxyribonucleotide binding	4	1	0.2920
GO:0032557	pyrimidine ribonucleotide binding	4	1	0.2920
GO:0032558	adenyl deoxyribonucleotide binding	3	1	0.2920
GO:0032564	dATP binding	2	1	0.2920
GO:0034190	apolipoprotein receptor binding	6	1	0.2920
GO:0034191	apolipoprotein A-I receptor binding	4	1	0.2920
GO:0034987	immunoglobulin receptor binding	79	1	0.2920
GO:0034988	Fc-gamma receptor I complex binding	3	1	0.2920
GO:0035259	nuclear glucocorticoid receptor binding	13	1	0.2920
GO:0035605	peptidyl-cysteine S-nitrosylase activity	1	1	0.2920
GO:0042608	T cell receptor binding	10	1	0.2920
GO:0042610	CD8 receptor binding	3	1	0.2920
GO:0043047	single-stranded telomeric DNA binding	12	1	0.2920
GO:0043891	glyceraldehyde-3-phosphate dehydrogenase	2	1	0.2920
GO:0045236	CXCR chemokine receptor binding	18	1	0.2920
GO:0046943	carboxylic acid transmembrane transporter a	165	1	0.2920
GO:0050421	nitrite reductase (NO-forming) activity	5	1	0.2920
GO:0061630	ubiquitin protein ligase activity	322	1	0.2920
GO:0061659	ubiquitin-like protein ligase activity	334	1	0.2920
GO:0061783	peptidoglycan muralytic activity	14	1	0.2920
GO:0062061	TAP complex binding	2	1	0.2920

GO:0070001	aspartic-type peptidase activity	27	1	0.2920
GO:0070006	metalloaminopeptidase activity	26	1	0.2920
GO:0070182	DNA polymerase binding	22	1	0.2920
GO:0070273	phosphatidylinositol-4-phosphate binding	28	1	0.2920
GO:0070891	lipoteichoic acid binding	5	1	0.2920
GO:0070892	lipoteichoic acid immune receptor activity	1	1	0.2920
GO:0071723	lipopeptide binding	10	1	0.2920
GO:0097153	cysteine-type endopeptidase activity involve	15	1	0.2920
GO:0097157	pre-mRNA intronic binding	13	1	0.2920
GO:0097200	cysteine-type endopeptidase activity involve	9	1	0.2920
GO:0098505	G-rich strand telomeric DNA binding	10	1	0.2920
GO:0098634	cell-matrix adhesion mediator activity	7	1	0.2920
GO:0098639	collagen binding involved in cell-matrix adhes	5	1	0.2920
GO:0098809	nitrite reductase activity	6	1	0.2920
GO:0098918	structural constituent of synapse	19	1	0.2920
GO:0098973	structural constituent of postsynaptic actin c	5	1	0.2920
GO:0099186	structural constituent of postsynapse	13	1	0.2920
GO:0106006	cytoskeletal protein-membrane anchor activi	4	1	0.2920
GO:0106310	protein serine kinase activity	363	1	0.2920
GO:0140311	protein sequestering activity	9	1	0.2920
GO:0140313	molecular sequestering activity	14	1	0.2920
GO:0150025	oxidised low-density lipoprotein particle rece	1	1	0.2920
GO:1901480	oleate transmembrane transporter activity	5	1	0.2920
GO:1904399	heparan sulfate binding	4	1	0.2920
GO:0006952	defense response	1820	48	0.2946
GO:0018130	heterocycle biosynthetic process	4086	48	0.2946
GO:0019438	aromatic compound biosynthetic process	4100	48	0.2946
GO:0098552	side of membrane	685	35	0.2978
GO:0048871	multicellular organismal homeostasis	536	19	0.3004
GO:0009897	external side of plasma membrane	462	19	0.3004
GO:0031252	cell leading edge	421	19	0.3004
GO:0065007	biological regulation	13067	171	0.3004
GO:0006810	transport	4578	95	0.3006
GO:0050789	regulation of biological process	12322	164	0.3020
GO:0002009	morphogenesis of an epithelium	521	16	0.3095
GO:0008037	cell recognition	230	16	0.3095
GO:0034762	regulation of transmembrane transport	618	16	0.3095
GO:0050867	positive regulation of cell activation	467	16	0.3095
GO:0055082	cellular chemical homeostasis	675	16	0.3095
GO:0060249	anatomical structure homeostasis	333	16	0.3095
GO:0003012	muscle system process	455	13	0.3185
GO:0006605	protein targeting	325	13	0.3185
GO:0006898	receptor-mediated endocytosis	257	13	0.3185
GO:0030099	myeloid cell differentiation	407	13	0.3185
GO:0005765	lysosomal membrane	418	13	0.3185
GO:0031253	cell projection membrane	344	13	0.3185
GO:0098852	lytic vacuole membrane	418	13	0.3185

GO:0005543	phospholipid binding	474	13	0.3185
GO:0051234	establishment of localization	4734	99	0.3191
GO:0010033	response to organic substance	3109	72	0.3197
GO:0032101	regulation of response to external stimulus	1014	29	0.3198
GO:0019901	protein kinase binding	697	29	0.3198
GO:0019219	regulation of nucleobase-containing compou	4076	52	0.3204
GO:1901362	organic cyclic compound biosynthetic proces	4253	52	0.3204
GO:0080090	regulation of primary metabolic process	5954	89	0.3245
GO:0006874	cellular calcium ion homeostasis	284	10	0.3270
GO:0007264	small GTPase mediated signal transduction	506	10	0.3270
GO:0007265	Ras protein signal transduction	353	10	0.3270
GO:0030595	leukocyte chemotaxis	240	10	0.3270
GO:0031334	positive regulation of protein-containing com	192	10	0.3270
GO:0050808	synapse organization	432	10	0.3270
GO:0055074	calcium ion homeostasis	309	10	0.3270
GO:0060326	cell chemotaxis	319	10	0.3270
GO:0097237	cellular response to toxic substance	123	10	0.3270
GO:0098754	detoxification	154	10	0.3270
GO:1901342	regulation of vasculature development	355	10	0.3270
GO:1990748	cellular detoxification	115	10	0.3270
GO:0030027	lamellipodium	203	10	0.3270
GO:0035579	specific granule membrane	91	10	0.3270
GO:0046982	protein heterodimerization activity	341	10	0.3270
GO:0016310	phosphorylation	1853	39	0.3286
GO:0009986	cell surface	962	39	0.3286
GO:0045321	leukocyte activation	1009	26	0.3316
GO:0000910	cytokinesis	187	7	0.3337
GO:0002224	toll-like receptor signaling pathway	123	7	0.3337
GO:0003158	endothelium development	139	7	0.3337
GO:0006801	superoxide metabolic process	74	7	0.3337
GO:0007051	spindle organization	195	7	0.3337
GO:0007249	I-kappaB kinase/NF-kappaB signaling	288	7	0.3337
GO:0008360	regulation of cell shape	154	7	0.3337
GO:0009792	embryo development ending in birth or egg h	677	7	0.3337
GO:0015711	organic anion transport	372	7	0.3337
GO:0016055	Wnt signaling pathway	456	7	0.3337
GO:0018108	peptidyl-tyrosine phosphorylation	380	7	0.3337
GO:0018212	peptidyl-tyrosine modification	382	7	0.3337
GO:0021762	substantia nigra development	44	7	0.3337
GO:0030901	midbrain development	91	7	0.3337
GO:0032231	regulation of actin filament bundle assembly	108	7	0.3337
GO:0032504	multicellular organism reproduction	922	7	0.3337
GO:0033627	cell adhesion mediated by integrin	88	7	0.3337
GO:0043009	chordate embryonic development	655	7	0.3337
GO:0043122	regulation of I-kappaB kinase/NF-kappaB sigr	255	7	0.3337
GO:0043542	endothelial cell migration	281	7	0.3337
GO:0045807	positive regulation of endocytosis	96	7	0.3337

GO:0048857	neural nucleus development	65	7	0.3337
GO:0051047	positive regulation of secretion	304	7	0.3337
GO:0051146	striated muscle cell differentiation	293	7	0.3337
GO:0051606	detection of stimulus	673	7	0.3337
GO:0061337	cardiac conduction	101	7	0.3337
GO:0062207	regulation of pattern recognition receptor sig	107	7	0.3337
GO:0070371	ERK1 and ERK2 cascade	340	7	0.3337
GO:0070372	regulation of ERK1 and ERK2 cascade	315	7	0.3337
GO:0198738	cell-cell signaling by wnt	458	7	0.3337
GO:1902414	protein localization to cell junction	98	7	0.3337
GO:1905114	cell surface receptor signaling pathway involv	582	7	0.3337
GO:0044291	cell-cell contact zone	72	7	0.3337
GO:0098791	Golgi apparatus subcompartment	387	7	0.3337
GO:0098858	actin-based cell projection	223	7	0.3337
GO:0004888	transmembrane signaling receptor activity	1309	7	0.3337
GO:0005080	protein kinase C binding	55	7	0.3337
GO:0001655	urogenital system development	360	4	0.3338
GO:0001822	kidney development	309	4	0.3338
GO:0001954	positive regulation of cell-matrix adhesion	56	4	0.3338
GO:0002040	sprouting angiogenesis	185	4	0.3338
GO:0002347	response to tumor cell	43	4	0.3338
GO:0002367	cytokine production involved in immune resp	120	4	0.3338
GO:0002478	antigen processing and presentation of exoge	40	4	0.3338
GO:0002718	regulation of cytokine production involved in	118	4	0.3338
GO:0002720	positive regulation of cytokine production inv	79	4	0.3338
GO:0002790	peptide secretion	230	4	0.3338
GO:0002791	regulation of peptide secretion	182	4	0.3338
GO:0006968	cellular defense response	52	4	0.3338
GO:0007059	chromosome segregation	382	4	0.3338
GO:0009595	detection of biotic stimulus	39	4	0.3338
GO:0010506	regulation of autophagy	344	4	0.3338
GO:0010817	regulation of hormone levels	517	4	0.3338
GO:0015833	peptide transport	254	4	0.3338
GO:0017014	protein nitrosylation	17	4	0.3338
GO:0018105	peptidyl-serine phosphorylation	313	4	0.3338
GO:0018119	peptidyl-cysteine S-nitrosylation	17	4	0.3338
GO:0018209	peptidyl-serine modification	332	4	0.3338
GO:0019731	antibacterial humoral response	63	4	0.3338
GO:0019884	antigen processing and presentation of exoge	49	4	0.3338
GO:0022408	negative regulation of cell-cell adhesion	202	4	0.3338
GO:0031640	killing of cells of another organism	46	4	0.3338
GO:0032755	positive regulation of interleukin-6 productio	99	4	0.3338
GO:0032872	regulation of stress-activated MAPK cascade	195	4	0.3338
GO:0033622	integrin activation	26	4	0.3338
GO:0034110	regulation of homotypic cell-cell adhesion	37	4	0.3338
GO:0034123	positive regulation of toll-like receptor signal	29	4	0.3338
GO:0035051	cardiocyte differentiation	159	4	0.3338

GO:0036465	synaptic vesicle recycling	79	4	0.3338
GO:0042886	amide transport	296	4	0.3338
GO:0043297	apical junction assembly	77	4	0.3338
GO:0045454	cell redox homeostasis	41	4	0.3338
GO:0045580	regulation of T cell differentiation	179	4	0.3338
GO:0045730	respiratory burst	39	4	0.3338
GO:0046942	carboxylic acid transport	290	4	0.3338
GO:0048488	synaptic vesicle endocytosis	66	4	0.3338
GO:0048524	positive regulation of viral process	64	4	0.3338
GO:0050866	negative regulation of cell activation	221	4	0.3338
GO:0050901	leukocyte tethering or rolling	33	4	0.3338
GO:0051100	negative regulation of binding	164	4	0.3338
GO:0051403	stress-activated MAPK cascade	239	4	0.3338
GO:0051873	killing by host of symbiont cells	29	4	0.3338
GO:0060070	canonical Wnt signaling pathway	310	4	0.3338
GO:0060828	regulation of canonical Wnt signaling pathwa	260	4	0.3338
GO:0070302	regulation of stress-activated protein kinase s	198	4	0.3338
GO:0072001	renal system development	318	4	0.3338
GO:0090087	regulation of peptide transport	184	4	0.3338
GO:0098813	nuclear chromosome segregation	321	4	0.3338
GO:0140238	presynaptic endocytosis	66	4	0.3338
GO:1903076	regulation of protein localization to plasma m	103	4	0.3338
GO:1904064	positive regulation of cation transmembrane	171	4	0.3338
GO:1905477	positive regulation of protein localization to r	101	4	0.3338
GO:0005791	rough endoplasmic reticulum	80	4	0.3338
GO:0005793	endoplasmic reticulum-Golgi intermediate co	133	4	0.3338
GO:0005802	trans-Golgi network	262	4	0.3338
GO:0005901	caveola	82	4	0.3338
GO:0030057	desmosome	25	4	0.3338
GO:0032153	cell division site	72	4	0.3338
GO:0032154	cleavage furrow	54	4	0.3338
GO:0043209	myelin sheath	47	4	0.3338
GO:0044815	DNA packaging complex	178	4	0.3338
GO:0045334	clathrin-coated endocytic vesicle	90	4	0.3338
GO:0071556	integral component of lumenal side of endop	29	4	0.3338
GO:0098553	lumenal side of endoplasmic reticulum meml	29	4	0.3338
GO:1990234	transferase complex	839	4	0.3338
GO:0001530	lipopolysaccharide binding	37	4	0.3338
GO:0005539	glycosaminoglycan binding	237	4	0.3338
GO:0005544	calcium-dependent phospholipid binding	54	4	0.3338
GO:0008017	microtubule binding	271	4	0.3338
GO:0016301	kinase activity	728	4	0.3338
GO:0019838	growth factor binding	132	4	0.3338
GO:0019955	cytokine binding	144	4	0.3338
GO:0042826	histone deacetylase binding	128	4	0.3338
GO:0045309	protein phosphorylated amino acid binding	58	4	0.3338
GO:0051219	phosphoprotein binding	92	4	0.3338

GO:0031323	regulation of cellular metabolic process	5737	86	0.3368
GO:0019222	regulation of metabolic process	7297	110	0.3435
GO:0048522	positive regulation of cellular process	5749	110	0.3435
GO:0010562	positive regulation of phosphorus metabolic	914	23	0.3441
GO:0044403	biological process involved in symbiotic inter	307	23	0.3441
GO:0045937	positive regulation of phosphate metabolic p	914	23	0.3441
GO:0051241	negative regulation of multicellular organism	1169	23	0.3441
GO:0015629	actin cytoskeleton	502	23	0.3441
GO:0009889	regulation of biosynthetic process	4239	56	0.3453
GO:0042221	response to chemical	4494	83	0.3493
GO:0042592	homeostatic process	1705	43	0.3576
GO:0046872	metal ion binding	4246	43	0.3576
GO:0030001	metal ion transport	909	20	0.3576
GO:0046649	lymphocyte activation	846	20	0.3576
GO:0042803	protein homodimerization activity	691	20	0.3576
GO:0032502	developmental process	6527	121	0.3670
GO:0042325	regulation of phosphorylation	1264	30	0.3677
GO:0030154	cell differentiation	4302	87	0.3697
GO:0048869	cellular developmental process	4329	87	0.3697
GO:0016070	RNA metabolic process	5053	50	0.3712
GO:0032501	multicellular organismal process	7674	128	0.3715
GO:0019058	viral life cycle	319	17	0.3721
GO:0022407	regulation of cell-cell adhesion	490	17	0.3721
GO:0032940	secretion by cell	817	17	0.3721
GO:0005938	cell cortex	312	17	0.3721
GO:0045121	membrane raft	326	17	0.3721
GO:0098857	membrane microdomain	327	17	0.3721
GO:0051179	localization	5370	111	0.3755
GO:0014070	response to organic cyclic compound	931	27	0.3825
GO:0048523	negative regulation of cellular process	4934	84	0.3828
GO:0012501	programmed cell death	2000	47	0.3850
GO:0098542	defense response to other organism	1183	37	0.3854
GO:0006875	cellular metal ion homeostasis	420	14	0.3882
GO:0010959	regulation of metal ion transport	423	14	0.3882
GO:0034765	regulation of ion transmembrane transport	520	14	0.3882
GO:0080135	regulation of cellular response to stress	726	14	0.3882
GO:0043169	cation binding	4337	44	0.3994
GO:0051171	regulation of nitrogen compound metabolic p	5792	88	0.4034
GO:0001906	cell killing	189	11	0.4065
GO:0009636	response to toxic substance	250	11	0.4065
GO:0010631	epithelial cell migration	366	11	0.4065
GO:0032388	positive regulation of intracellular transport	199	11	0.4065
GO:0034599	cellular response to oxidative stress	288	11	0.4065
GO:0044000	movement in host	183	11	0.4065
GO:0051092	positive regulation of NF-kappaB transcriptio	154	11	0.4065
GO:0051640	organelle localization	570	11	0.4065
GO:0070588	calcium ion transmembrane transport	345	11	0.4065

GO:0090130	tissue migration	374	11	0.4065
GO:0090132	epithelium migration	369	11	0.4065
GO:1903037	regulation of leukocyte cell-cell adhesion	377	11	0.4065
GO:0001726	ruffle	178	11	0.4065
GO:0005775	vacuolar lumen	176	11	0.4065
GO:0005912	adherens junction	179	11	0.4065
GO:0045335	phagocytic vesicle	139	11	0.4065
GO:0098793	presynapse	519	11	0.4065
GO:0098978	glutamatergic synapse	324	11	0.4065
GO:0008219	cell death	2166	51	0.4111
GO:0035556	intracellular signal transduction	2673	51	0.4111
GO:0034220	ion transmembrane transport	1167	21	0.4159
GO:1901565	organonitrogen compound catabolic process	1371	21	0.4159
GO:0030334	regulation of cell migration	1025	31	0.4166
GO:0040012	regulation of locomotion	1130	31	0.4166
GO:2000145	regulation of cell motility	1086	31	0.4166
GO:0001895	retina homeostasis	80	8	0.4282
GO:0002064	epithelial cell development	213	8	0.4282
GO:0006937	regulation of muscle contraction	174	8	0.4282
GO:0030198	extracellular matrix organization	318	8	0.4282
GO:0034446	substrate adhesion-dependent cell spreading	111	8	0.4282
GO:0035637	multicellular organismal signaling	170	8	0.4282
GO:0042098	T cell proliferation	212	8	0.4282
GO:0042692	muscle cell differentiation	409	8	0.4282
GO:0043062	extracellular structure organization	319	8	0.4282
GO:0060537	muscle tissue development	422	8	0.4282
GO:0016363	nuclear matrix	126	8	0.4282
GO:0034399	nuclear periphery	145	8	0.4282
GO:0001618	virus receptor activity	77	8	0.4282
GO:0008270	zinc ion binding	823	8	0.4282
GO:0016209	antioxidant activity	85	8	0.4282
GO:0051020	GTPase binding	303	8	0.4282
GO:0140272	exogenous protein binding	78	8	0.4282
GO:0033993	response to lipid	918	28	0.4340
GO:0005488	binding	16619	220	0.4353
GO:0001934	positive regulation of protein phosphorylatio	751	18	0.4354
GO:0030163	protein catabolic process	1014	18	0.4354
GO:0040007	growth	921	18	0.4354
GO:0048729	tissue morphogenesis	633	18	0.4354
GO:0071396	cellular response to lipid	608	18	0.4354
GO:0140352	export from cell	890	18	0.4354
GO:0006915	apoptotic process	1941	45	0.4418
GO:0000045	autophagosome assembly	110	5	0.4561
GO:0001505	regulation of neurotransmitter levels	219	5	0.4561
GO:0001738	morphogenesis of a polarized epithelium	96	5	0.4561
GO:0002027	regulation of heart rate	107	5	0.4561
GO:0002228	natural killer cell mediated immunity	77	5	0.4561

GO:0002700	regulation of production of molecular mediat	187	5	0.4561
GO:0002702	positive regulation of production of molecula	133	5	0.4561
GO:0002832	negative regulation of response to biotic stim	116	5	0.4561
GO:0006006	glucose metabolic process	188	5	0.4561
GO:0006836	neurotransmitter transport	212	5	0.4561
GO:0008202	steroid metabolic process	323	5	0.4561
GO:0009620	response to fungus	67	5	0.4561
GO:0010721	negative regulation of cell development	188	5	0.4561
GO:0010761	fibroblast migration	55	5	0.4561
GO:0010977	negative regulation of neuron projection dev	142	5	0.4561
GO:0014706	striated muscle tissue development	257	5	0.4561
GO:0018198	peptidyl-cysteine modification	50	5	0.4561
GO:0030038	contractile actin filament bundle assembly	109	5	0.4561
GO:0030111	regulation of Wnt signaling pathway	336	5	0.4561
GO:0030195	negative regulation of blood coagulation	50	5	0.4561
GO:0031098	stress-activated protein kinase signaling casc	246	5	0.4561
GO:0032869	cellular response to insulin stimulus	208	5	0.4561
GO:0034332	adherens junction organization	51	5	0.4561
GO:0042267	natural killer cell mediated cytotoxicity	74	5	0.4561
GO:0042743	hydrogen peroxide metabolic process	55	5	0.4561
GO:0043149	stress fiber assembly	109	5	0.4561
GO:0044772	mitotic cell cycle phase transition	473	5	0.4561
GO:0045165	cell fate commitment	282	5	0.4561
GO:0045619	regulation of lymphocyte differentiation	210	5	0.4561
GO:0046916	cellular transition metal ion homeostasis	116	5	0.4561
GO:0048002	antigen processing and presentation of pepti	64	5	0.4561
GO:0048732	gland development	441	5	0.4561
GO:0048738	cardiac muscle tissue development	241	5	0.4561
GO:0048863	stem cell differentiation	248	5	0.4561
GO:0050678	regulation of epithelial cell proliferation	414	5	0.4561
GO:0050729	positive regulation of inflammatory response	154	5	0.4561
GO:0050731	positive regulation of peptidyl-tyrosine phosp	193	5	0.4561
GO:0050832	defense response to fungus	56	5	0.4561
GO:0051492	regulation of stress fiber assembly	94	5	0.4561
GO:0051588	regulation of neurotransmitter transport	105	5	0.4561
GO:0061756	leukocyte adhesion to vascular endothelial ce	57	5	0.4561
GO:0062208	positive regulation of pattern recognition rec	45	5	0.4561
GO:0070374	positive regulation of ERK1 and ERK2 cascade	223	5	0.4561
GO:0086001	cardiac muscle cell action potential	79	5	0.4561
GO:0090150	establishment of protein localization to mem	270	5	0.4561
GO:0097191	extrinsic apoptotic signaling pathway	224	5	0.4561
GO:0099173	postsynapse organization	166	5	0.4561
GO:0110020	regulation of actomyosin structure organizati	104	5	0.4561
GO:1900047	negative regulation of hemostasis	51	5	0.4561
GO:1900407	regulation of cellular response to oxidative st	95	5	0.4561
GO:1902850	microtubule cytoskeleton organization involv	163	5	0.4561
GO:1902882	regulation of response to oxidative stress	104	5	0.4561

GO:1903825	organic acid transmembrane transport	156	5	0.4561
GO:1904375	regulation of protein localization to cell perip	125	5	0.4561
GO:1905037	autophagosome organization	117	5	0.4561
GO:1905039	carboxylic acid transmembrane transport	155	5	0.4561
GO:2000177	regulation of neural precursor cell proliferati	90	5	0.4561
GO:2001236	regulation of extrinsic apoptotic signaling pat	155	5	0.4561
GO:2001237	negative regulation of extrinsic apoptotic sigr	99	5	0.4561
GO:0030665	clathrin-coated vesicle membrane	132	5	0.4561
GO:0044853	plasma membrane raft	113	5	0.4561
GO:0072686	mitotic spindle	183	5	0.4561
GO:0098576	lumenal side of membrane	35	5	0.4561
GO:0098687	chromosomal region	388	5	0.4561
GO:0099568	cytoplasmic region	285	5	0.4561
GO:0002020	protease binding	139	5	0.4561
GO:0004601	peroxidase activity	55	5	0.4561
GO:0008509	anion transmembrane transporter activity	303	5	0.4561
GO:0016684	oxidoreductase activity, acting on peroxide a	57	5	0.4561
GO:0016772	transferase activity, transferring phosphorus-	876	5	0.4561
GO:0016853	isomerase activity	156	5	0.4561
GO:0019902	phosphatase binding	195	5	0.4561
GO:0019903	protein phosphatase binding	149	5	0.4561
GO:0035091	phosphatidylinositol binding	277	5	0.4561
GO:0050660	flavin adenine dinucleotide binding	87	5	0.4561
GO:0050840	extracellular matrix binding	56	5	0.4561
GO:0002831	regulation of response to biotic stimulus	361	15	0.4577
GO:0006873	cellular ion homeostasis	516	15	0.4577
GO:0016049	cell growth	493	15	0.4577
GO:0030003	cellular cation homeostasis	499	15	0.4577
GO:0045596	negative regulation of cell differentiation	718	15	0.4577
GO:0030424	axon	642	15	0.4577
GO:0044297	cell body	565	15	0.4577
GO:0031984	organelle subcompartment	1486	32	0.4658
GO:0019900	kinase binding	781	32	0.4658
GO:0008284	positive regulation of cell population prolifer	986	22	0.4740
GO:0016032	viral process	421	22	0.4740
GO:0031401	positive regulation of protein modification pr	1019	22	0.4740
GO:0051240	positive regulation of multicellular organisma	1553	39	0.4759
GO:0010468	regulation of gene expression	5401	80	0.4808
GO:0009790	embryo development	1130	12	0.4843
GO:0022409	positive regulation of cell-cell adhesion	322	12	0.4843
GO:0051702	biological process involved in interaction witl	116	12	0.4843
GO:1900180	regulation of protein localization to nucleus	135	12	0.4843
GO:1903047	mitotic cell cycle process	789	12	0.4843
GO:1903131	mononuclear cell differentiation	473	12	0.4843
GO:1903530	regulation of secretion by cell	566	12	0.4843
GO:0010008	endosome membrane	541	12	0.4843
GO:0030425	dendrite	621	12	0.4843

GO:0097447	dendritic tree	623	12	0.4843
GO:0051049	regulation of transport	1791	46	0.4844
GO:0044092	negative regulation of molecular function	1187	29	0.4854
GO:0045202	synapse	1341	29	0.4854
GO:0098796	membrane protein complex	1328	29	0.4854
GO:0012505	endomembrane system	4715	111	0.4900
GO:0071310	cellular response to organic substance	2464	53	0.4918
GO:0048519	negative regulation of biological process	5903	94	0.4928
GO:0019220	regulation of phosphate metabolic process	1423	36	0.4945
GO:0051174	regulation of phosphorus metabolic process	1424	36	0.4945
GO:0022607	cellular component assembly	3011	77	0.4956
GO:0009725	response to hormone	861	19	0.4977
GO:0043269	regulation of ion transport	718	19	0.4977
GO:0044419	biological process involved in interspecies int	1726	60	0.4986
GO:0000018	regulation of DNA recombination	133	2	0.4997
GO:0000070	mitotic sister chromatid segregation	204	2	0.4997
GO:0000209	protein polyubiquitination	246	2	0.4997
GO:0000725	recombinational repair	173	2	0.4997
GO:0000819	sister chromatid segregation	239	2	0.4997
GO:0001704	formation of primary germ layer	127	2	0.4997
GO:0001707	mesoderm formation	74	2	0.4997
GO:0001736	establishment of planar polarity	74	2	0.4997
GO:0001778	plasma membrane repair	30	2	0.4997
GO:0001825	blastocyst formation	45	2	0.4997
GO:0001911	negative regulation of leukocyte mediated cy	25	2	0.4997
GO:0002036	regulation of L-glutamate import across plasr	8	2	0.4997
GO:0002369	T cell cytokine production	38	2	0.4997
GO:0002424	T cell mediated immune response to tumor c	12	2	0.4997
GO:0002484	antigen processing and presentation of endo	9	2	0.4997
GO:0002486	antigen processing and presentation of endo	8	2	0.4997
GO:0002704	negative regulation of leukocyte mediated im	68	2	0.4997
GO:0002707	negative regulation of lymphocyte mediated	56	2	0.4997
GO:0002716	negative regulation of natural killer cell medi	20	2	0.4997
GO:0002724	regulation of T cell cytokine production	38	2	0.4997
GO:0002726	positive regulation of T cell cytokine producti	26	2	0.4997
GO:0002934	desmosome organization	10	2	0.4997
GO:0003016	respiratory system process	39	2	0.4997
GO:0003333	amino acid transmembrane transport	104	2	0.4997
GO:0006282	regulation of DNA repair	212	2	0.4997
GO:0006356	regulation of transcription by RNA polymeras	43	2	0.4997
GO:0006360	transcription by RNA polymerase I	62	2	0.4997
GO:0006509	membrane protein ectodomain proteolysis	44	2	0.4997
GO:0006766	vitamin metabolic process	110	2	0.4997
GO:0006767	water-soluble vitamin metabolic process	62	2	0.4997
GO:0006835	dicarboxylic acid transport	93	2	0.4997
GO:0006865	amino acid transport	147	2	0.4997
GO:0006882	cellular zinc ion homeostasis	40	2	0.4997

GO:0006953	acute-phase response	48	2	0.4997
GO:0007164	establishment of tissue polarity	74	2	0.4997
GO:0007204	positive regulation of cytosolic calcium ion co	205	2	0.4997
GO:0007219	Notch signaling pathway	183	2	0.4997
GO:0007263	nitric oxide mediated signal transduction	29	2	0.4997
GO:0007269	neurotransmitter secretion	151	2	0.4997
GO:0007398	ectoderm development	22	2	0.4997
GO:0007405	neuroblast proliferation	67	2	0.4997
GO:0007422	peripheral nervous system development	81	2	0.4997
GO:0007498	mesoderm development	136	2	0.4997
GO:0007549	dosage compensation	30	2	0.4997
GO:0007585	respiratory gaseous exchange by respiratory	74	2	0.4997
GO:0007606	sensory perception of chemical stimulus	537	2	0.4997
GO:0008228	opsonization	17	2	0.4997
GO:0008593	regulation of Notch signaling pathway	97	2	0.4997
GO:0008643	carbohydrate transport	156	2	0.4997
GO:0008645	hexose transmembrane transport	116	2	0.4997
GO:0009048	dosage compensation by inactivation of X chr	28	2	0.4997
GO:0009409	response to cold	53	2	0.4997
GO:0009581	detection of external stimulus	137	2	0.4997
GO:0009582	detection of abiotic stimulus	140	2	0.4997
GO:0009593	detection of chemical stimulus	512	2	0.4997
GO:0009651	response to salt stress	30	2	0.4997
GO:0009755	hormone-mediated signaling pathway	192	2	0.4997
GO:0010310	regulation of hydrogen peroxide metabolic p	20	2	0.4997
GO:0010644	cell communication by electrical coupling	32	2	0.4997
GO:0010649	regulation of cell communication by electrica	15	2	0.4997
GO:0010712	regulation of collagen metabolic process	45	2	0.4997
GO:0010715	regulation of extracellular matrix disassembly	16	2	0.4997
GO:0010742	macrophage derived foam cell differentiation	38	2	0.4997
GO:0010743	regulation of macrophage derived foam cell d	32	2	0.4997
GO:0010880	regulation of release of sequestered calcium	31	2	0.4997
GO:0010883	regulation of lipid storage	54	2	0.4997
GO:0010958	regulation of amino acid import across plasm	18	2	0.4997
GO:0014037	Schwann cell differentiation	38	2	0.4997
GO:0014044	Schwann cell development	32	2	0.4997
GO:0014808	release of sequestered calcium ion into cytos	35	2	0.4997
GO:0015718	monocarboxylic acid transport	119	2	0.4997
GO:0015749	monosaccharide transmembrane transport	119	2	0.4997
GO:0015800	acidic amino acid transport	65	2	0.4997
GO:0015807	L-amino acid transport	76	2	0.4997
GO:0015813	L-glutamate transmembrane transport	29	2	0.4997
GO:0015837	amine transport	106	2	0.4997
GO:0015844	monoamine transport	84	2	0.4997
GO:0016045	detection of bacterium	17	2	0.4997
GO:0016079	synaptic vesicle exocytosis	111	2	0.4997
GO:0016482	cytosolic transport	171	2	0.4997

GO:0017158	regulation of calcium ion-dependent exocyto	41	2	0.4997
GO:0019216	regulation of lipid metabolic process	347	2	0.4997
GO:0019852	L-ascorbic acid metabolic process	9	2	0.4997
GO:0019915	lipid storage	87	2	0.4997
GO:0019934	cGMP-mediated signaling	36	2	0.4997
GO:0019935	cyclic-nucleotide-mediated signaling	86	2	0.4997
GO:0021795	cerebral cortex cell migration	46	2	0.4997
GO:0021885	forebrain cell migration	63	2	0.4997
GO:0021953	central nervous system neuron differentiatio	174	2	0.4997
GO:0022011	myelination in peripheral nervous system	29	2	0.4997
GO:0022029	telencephalon cell migration	60	2	0.4997
GO:0030224	monocyte differentiation	36	2	0.4997
GO:0030518	intracellular steroid hormone receptor signal	116	2	0.4997
GO:0030856	regulation of epithelial cell differentiation	164	2	0.4997
GO:0030866	cortical actin cytoskeleton organization	40	2	0.4997
GO:0030879	mammary gland development	138	2	0.4997
GO:0030902	hindbrain development	153	2	0.4997
GO:0031023	microtubule organizing center organization	150	2	0.4997
GO:0031047	gene silencing by RNA	690	2	0.4997
GO:0031342	negative regulation of cell killing	28	2	0.4997
GO:0031665	negative regulation of lipopolysaccharide-me	14	2	0.4997
GO:0032119	sequestering of zinc ion	8	2	0.4997
GO:0032232	negative regulation of actin filament bundle a	36	2	0.4997
GO:0032292	peripheral nervous system axon ensheathme	29	2	0.4997
GO:0032418	lysosome localization	84	2	0.4997
GO:0032467	positive regulation of cytokinesis	46	2	0.4997
GO:0032570	response to progesterone	39	2	0.4997
GO:0032602	chemokine production	99	2	0.4997
GO:0032611	interleukin-1 beta production	111	2	0.4997
GO:0032612	interleukin-1 production	130	2	0.4997
GO:0032642	regulation of chemokine production	98	2	0.4997
GO:0032651	regulation of interleukin-1 beta production	111	2	0.4997
GO:0032652	regulation of interleukin-1 production	130	2	0.4997
GO:0032720	negative regulation of tumor necrosis factor	77	2	0.4997
GO:0032735	positive regulation of interleukin-12 producti	42	2	0.4997
GO:0032835	glomerulus development	69	2	0.4997
GO:0032873	negative regulation of stress-activated MAPK	51	2	0.4997
GO:0032963	collagen metabolic process	107	2	0.4997
GO:0033045	regulation of sister chromatid segregation	107	2	0.4997
GO:0033619	membrane protein proteolysis	58	2	0.4997
GO:0033623	regulation of integrin activation	17	2	0.4997
GO:0033625	positive regulation of integrin activation	11	2	0.4997
GO:0034142	toll-like receptor 4 signaling pathway	42	2	0.4997
GO:0034143	regulation of toll-like receptor 4 signaling pat	27	2	0.4997
GO:0034145	positive regulation of toll-like receptor 4 sign	12	2	0.4997
GO:0034205	amyloid-beta formation	56	2	0.4997
GO:0034219	carbohydrate transmembrane transport	132	2	0.4997

GO:0034260	negative regulation of GTPase activity	36	2	0.4997
GO:0034333	adherens junction assembly	13	2	0.4997
GO:0034381	plasma lipoprotein particle clearance	53	2	0.4997
GO:0034383	low-density lipoprotein particle clearance	34	2	0.4997
GO:0035148	tube formation	155	2	0.4997
GO:0035425	autocrine signaling	7	2	0.4997
GO:0035567	non-canonical Wnt signaling pathway	72	2	0.4997
GO:0035696	monocyte extravasation	8	2	0.4997
GO:0035821	modulation of process of another organism	26	2	0.4997
GO:0036336	dendritic cell migration	34	2	0.4997
GO:0038034	signal transduction in absence of ligand	68	2	0.4997
GO:0038127	ERBB signaling pathway	126	2	0.4997
GO:0040029	epigenetic regulation of gene expression	163	2	0.4997
GO:0042157	lipoprotein metabolic process	140	2	0.4997
GO:0042270	protection from natural killer cell mediated c	6	2	0.4997
GO:0042590	antigen processing and presentation of exoge	9	2	0.4997
GO:0042953	lipoprotein transport	20	2	0.4997
GO:0043401	steroid hormone mediated signaling pathway	136	2	0.4997
GO:0044070	regulation of anion transport	93	2	0.4997
GO:0044088	regulation of vacuole organization	51	2	0.4997
GO:0044806	G-quadruplex DNA unwinding	7	2	0.4997
GO:0044872	lipoprotein localization	21	2	0.4997
GO:0045022	early endosome to late endosome transport	42	2	0.4997
GO:0045070	positive regulation of viral genome replicatio	32	2	0.4997
GO:0045582	positive regulation of T cell differentiation	117	2	0.4997
GO:0045601	regulation of endothelial cell differentiation	47	2	0.4997
GO:0045739	positive regulation of DNA repair	128	2	0.4997
GO:0045911	positive regulation of DNA recombination	72	2	0.4997
GO:0045932	negative regulation of muscle contraction	25	2	0.4997
GO:0045953	negative regulation of natural killer cell medi	19	2	0.4997
GO:0045955	negative regulation of calcium ion-dependen	8	2	0.4997
GO:0046323	glucose import	75	2	0.4997
GO:0046634	regulation of alpha-beta T cell activation	115	2	0.4997
GO:0046635	positive regulation of alpha-beta T cell activa	73	2	0.4997
GO:0046677	response to antibiotic	47	2	0.4997
GO:0046928	regulation of neurotransmitter secretion	91	2	0.4997
GO:0048332	mesoderm morphogenesis	76	2	0.4997
GO:0048333	mesodermal cell differentiation	34	2	0.4997
GO:0048538	thymus development	48	2	0.4997
GO:0048568	embryonic organ development	449	2	0.4997
GO:0048640	negative regulation of developmental growth	115	2	0.4997
GO:0048678	response to axon injury	89	2	0.4997
GO:0050435	amyloid-beta metabolic process	65	2	0.4997
GO:0050688	regulation of defense response to virus	74	2	0.4997
GO:0050691	regulation of defense response to virus by ho	42	2	0.4997
GO:0050769	positive regulation of neurogenesis	231	2	0.4997
GO:0050773	regulation of dendrite development	103	2	0.4997

GO:0050805	negative regulation of synaptic transmission	73	2	0.4997
GO:0050868	negative regulation of T cell activation	128	2	0.4997
GO:0051014	actin filament severing	16	2	0.4997
GO:0051028	mRNA transport	130	2	0.4997
GO:0051101	regulation of DNA binding	121	2	0.4997
GO:0051180	vitamin transport	47	2	0.4997
GO:0051280	negative regulation of release of sequestered	13	2	0.4997
GO:0051281	positive regulation of release of sequestered	41	2	0.4997
GO:0051284	positive regulation of sequestering of calcium	15	2	0.4997
GO:0051497	negative regulation of stress fiber assembly	33	2	0.4997
GO:0051591	response to cAMP	90	2	0.4997
GO:0051781	positive regulation of cell division	93	2	0.4997
GO:0051938	L-glutamate import	29	2	0.4997
GO:0051952	regulation of amine transport	98	2	0.4997
GO:0051955	regulation of amino acid transport	45	2	0.4997
GO:0051962	positive regulation of nervous system develo	279	2	0.4997
GO:0055069	zinc ion homeostasis	42	2	0.4997
GO:0055088	lipid homeostasis	174	2	0.4997
GO:0060071	Wnt signaling pathway, planar cell polarity pa	53	2	0.4997
GO:0060074	synapse maturation	29	2	0.4997
GO:0060135	maternal process involved in female pregnan	61	2	0.4997
GO:0060263	regulation of respiratory burst	18	2	0.4997
GO:0060306	regulation of membrane repolarization	38	2	0.4997
GO:0060314	regulation of ryanodine-sensitive calcium-rel	26	2	0.4997
GO:0060315	negative regulation of ryanodine-sensitive ca	11	2	0.4997
GO:0060996	dendritic spine development	92	2	0.4997
GO:0060997	dendritic spine morphogenesis	58	2	0.4997
GO:0061025	membrane fusion	177	2	0.4997
GO:0061437	renal system vasculature development	31	2	0.4997
GO:0061440	kidney vasculature development	31	2	0.4997
GO:0070098	chemokine-mediated signaling pathway	90	2	0.4997
GO:0070269	pyroptosis	27	2	0.4997
GO:0070296	sarcoplasmic reticulum calcium ion transport	42	2	0.4997
GO:0070303	negative regulation of stress-activated protei	51	2	0.4997
GO:0070488	neutrophil aggregation	2	2	0.4997
GO:0070493	thrombin-activated receptor signaling pathw	12	2	0.4997
GO:0070542	response to fatty acid	63	2	0.4997
GO:0070741	response to interleukin-6	45	2	0.4997
GO:0070837	dehydroascorbic acid transport	8	2	0.4997
GO:0071320	cellular response to cAMP	55	2	0.4997
GO:0071354	cellular response to interleukin-6	42	2	0.4997
GO:0071624	positive regulation of granulocyte chemotaxi	28	2	0.4997
GO:0071787	endoplasmic reticulum tubular network form	4	2	0.4997
GO:0071896	protein localization to adherens junction	6	2	0.4997
GO:0072012	glomerulus vasculature development	29	2	0.4997
GO:0072672	neutrophil extravasation	14	2	0.4997
GO:0072683	T cell extravasation	13	2	0.4997

GO:0086005	ventricular cardiac muscle cell action potenti	36	2	0.4997
GO:0086009	membrane repolarization	52	2	0.4997
GO:0086011	membrane repolarization during action poter	31	2	0.4997
GO:0086013	membrane repolarization during cardiac mus	26	2	0.4997
GO:0086042	cardiac muscle cell-cardiac muscle cell adhes	7	2	0.4997
GO:0086064	cell communication by electrical coupling inv	25	2	0.4997
GO:0086069	bundle of His cell to Purkinje myocyte comm	15	2	0.4997
GO:0086073	bundle of His cell-Purkinje myocyte adhesion	6	2	0.4997
GO:0086103	G protein-coupled receptor signaling pathwa	18	2	0.4997
GO:0089718	amino acid import across plasma membrane	50	2	0.4997
GO:0090022	regulation of neutrophil chemotaxis	34	2	0.4997
GO:0090023	positive regulation of neutrophil chemotaxis	25	2	0.4997
GO:0090075	relaxation of muscle	35	2	0.4997
GO:0090077	foam cell differentiation	39	2	0.4997
GO:0090100	positive regulation of transmembrane recept	121	2	0.4997
GO:0090151	establishment of protein localization to mito	37	2	0.4997
GO:0090175	regulation of establishment of planar polarity	57	2	0.4997
GO:0090559	regulation of membrane permeability	77	2	0.4997
GO:0097006	regulation of plasma lipoprotein particle leve	84	2	0.4997
GO:0097061	dendritic spine organization	81	2	0.4997
GO:0097192	extrinsic apoptotic signaling pathway in abse	68	2	0.4997
GO:0097242	amyloid-beta clearance	39	2	0.4997
GO:0098543	detection of other organism	20	2	0.4997
GO:0098704	carbohydrate import across plasma membra	6	2	0.4997
GO:0098708	glucose import across plasma membrane	5	2	0.4997
GO:0098712	L-glutamate import across plasma membrane	17	2	0.4997
GO:0098810	neurotransmitter reuptake	36	2	0.4997
GO:0098911	regulation of ventricular cardiac muscle cell a	12	2	0.4997
GO:0098927	vesicle-mediated transport between endoso	45	2	0.4997
GO:0099622	cardiac muscle cell membrane repolarization	40	2	0.4997
GO:0099643	signal release from synapse	151	2	0.4997
GO:0106027	neuron projection organization	91	2	0.4997
GO:0140271	hexose import across plasma membrane	6	2	0.4997
GO:0150076	neuroinflammatory response	73	2	0.4997
GO:0150094	amyloid-beta clearance by cellular catabolic p	8	2	0.4997
GO:0150106	regulation of protein localization to cell-cell j	3	2	0.4997
GO:1901016	regulation of potassium ion transmembrane	70	2	0.4997
GO:1901099	negative regulation of signal transduction in a	35	2	0.4997
GO:1901184	regulation of ERBB signaling pathway	80	2	0.4997
GO:1901224	positive regulation of NIK/NF-kappaB signalin	67	2	0.4997
GO:1901264	carbohydrate derivative transport	78	2	0.4997
GO:1901334	lactone metabolic process	9	2	0.4997
GO:1901385	regulation of voltage-gated calcium channel a	49	2	0.4997
GO:1901550	regulation of endothelial cell development	17	2	0.4997
GO:1901731	positive regulation of platelet aggregation	11	2	0.4997
GO:1901841	regulation of high voltage-gated calcium char	14	2	0.4997
GO:1901844	regulation of cell communication by electrica	11	2	0.4997

GO:1901990	regulation of mitotic cell cycle phase transitio	354	2	0.4997
GO:1902003	regulation of amyloid-beta formation	48	2	0.4997
GO:1902430	negative regulation of amyloid-beta formatio	29	2	0.4997
GO:1902475	L-alpha-amino acid transmembrane transpor	78	2	0.4997
GO:1902991	regulation of amyloid precursor protein catab	55	2	0.4997
GO:1902992	negative regulation of amyloid precursor pro	33	2	0.4997
GO:1903038	negative regulation of leukocyte cell-cell adh	147	2	0.4997
GO:1903140	regulation of establishment of endothelial ba	17	2	0.4997
GO:1903306	negative regulation of regulated secretory pa	26	2	0.4997
GO:1903426	regulation of reactive oxygen species biosynt	49	2	0.4997
GO:1903514	release of sequestered calcium ion into cytos	36	2	0.4997
GO:1903670	regulation of sprouting angiogenesis	60	2	0.4997
GO:1903729	regulation of plasma membrane organization	17	2	0.4997
GO:1903789	regulation of amino acid transmembrane tra	18	2	0.4997
GO:1903901	negative regulation of viral life cycle	26	2	0.4997
GO:1903959	regulation of anion transmembrane transpor	28	2	0.4997
GO:1904645	response to amyloid-beta	55	2	0.4997
GO:1904646	cellular response to amyloid-beta	45	2	0.4997
GO:1904659	glucose transmembrane transport	113	2	0.4997
GO:1904994	regulation of leukocyte adhesion to vascular	40	2	0.4997
GO:1904996	positive regulation of leukocyte adhesion to v	25	2	0.4997
GO:1905684	regulation of plasma membrane repair	6	2	0.4997
GO:1990000	amyloid fibril formation	40	2	0.4997
GO:1990845	adaptive thermogenesis	160	2	0.4997
GO:1990849	vacuolar localization	84	2	0.4997
GO:2000027	regulation of animal organ morphogenesis	129	2	0.4997
GO:2000242	negative regulation of reproductive process	75	2	0.4997
GO:2000300	regulation of synaptic vesicle exocytosis	60	2	0.4997
GO:2001022	positive regulation of response to DNA dama	166	2	0.4997
GO:2001239	regulation of extrinsic apoptotic signaling pat	46	2	0.4997
GO:2001240	negative regulation of extrinsic apoptotic sign	35	2	0.4997
GO:0000776	kinetochore	164	2	0.4997
GO:0000779	condensed chromosome, centromeric region	174	2	0.4997
GO:0000781	chromosome, telomeric region	168	2	0.4997
GO:0000793	condensed chromosome	273	2	0.4997
GO:0000932	P-body	97	2	0.4997
GO:0005604	basement membrane	99	2	0.4997
GO:0005839	proteasome core complex	20	2	0.4997
GO:0019773	proteasome core complex, alpha-subunit com	8	2	0.4997
GO:0030140	trans-Golgi network transport vesicle	37	2	0.4997
GO:0031233	intrinsic component of external side of plasm	24	2	0.4997
GO:0031362	anchored component of external side of plas	20	2	0.4997
GO:0031985	Golgi cisterna	120	2	0.4997
GO:0032588	trans-Golgi network membrane	101	2	0.4997
GO:0042824	MHC class I peptide loading complex	8	2	0.4997
GO:0043679	axon terminus	115	2	0.4997
GO:0044306	neuron projection terminus	131	2	0.4997

GO:0046658	anchored component of plasma membrane	62	2	0.4997
GO:0048786	presynaptic active zone	75	2	0.4997
GO:0071682	endocytic vesicle lumen	23	2	0.4997
GO:0071782	endoplasmic reticulum tubular network	24	2	0.4997
GO:0097197	tetraspanin-enriched microdomain	10	2	0.4997
GO:0097452	GAIT complex	4	2	0.4997
GO:0140534	endoplasmic reticulum protein-containing co	127	2	0.4997
GO:0001540	amyloid-beta binding	85	2	0.4997
GO:0003756	protein disulfide isomerase activity	18	2	0.4997
GO:0004842	ubiquitin-protein transferase activity	441	2	0.4997
GO:0005041	low-density lipoprotein particle receptor acti	15	2	0.4997
GO:0005125	cytokine activity	237	2	0.4997
GO:0005246	calcium channel regulator activity	45	2	0.4997
GO:0005324	long-chain fatty acid transporter activity	17	2	0.4997
GO:0005355	glucose transmembrane transporter activity	24	2	0.4997
GO:0005518	collagen binding	69	2	0.4997
GO:0005520	insulin-like growth factor binding	19	2	0.4997
GO:0005536	glucose binding	11	2	0.4997
GO:0008009	chemokine activity	49	2	0.4997
GO:0008035	high-density lipoprotein particle binding	12	2	0.4997
GO:0008093	cytoskeletal anchor activity	23	2	0.4997
GO:0008307	structural constituent of muscle	42	2	0.4997
GO:0015026	coreceptor activity	48	2	0.4997
GO:0015144	carbohydrate transmembrane transporter ac	43	2	0.4997
GO:0015145	monosaccharide transmembrane transporter	27	2	0.4997
GO:0015149	hexose transmembrane transporter activity	24	2	0.4997
GO:0015459	potassium channel regulator activity	54	2	0.4997
GO:0016620	oxidoreductase activity, acting on the aldehy	39	2	0.4997
GO:0016651	oxidoreductase activity, acting on NAD(P)H	87	2	0.4997
GO:0016670	oxidoreductase activity, acting on a sulfur gro	11	2	0.4997
GO:0016860	intramolecular oxidoreductase activity	51	2	0.4997
GO:0016864	intramolecular oxidoreductase activity, trans	18	2	0.4997
GO:0016874	ligase activity	165	2	0.4997
GO:0016903	oxidoreductase activity, acting on the aldehy	47	2	0.4997
GO:0019787	ubiquitin-like protein transferase activity	465	2	0.4997
GO:0019956	chemokine binding	33	2	0.4997
GO:0019960	C-X3-C chemokine binding	5	2	0.4997
GO:0030165	PDZ domain binding	82	2	0.4997
GO:0030169	low-density lipoprotein particle binding	17	2	0.4997
GO:0030228	lipoprotein particle receptor activity	18	2	0.4997
GO:0030235	nitric-oxide synthase regulator activity	7	2	0.4997
GO:0030276	clathrin binding	69	2	0.4997
GO:0030527	structural constituent of chromatin	99	2	0.4997
GO:0030545	signaling receptor regulator activity	546	2	0.4997
GO:0030546	signaling receptor activator activity	498	2	0.4997
GO:0030957	Tat protein binding	10	2	0.4997
GO:0031491	nucleosome binding	70	2	0.4997

GO:0031492	nucleosomal DNA binding	40	2	0.4997
GO:0031994	insulin-like growth factor I binding	13	2	0.4997
GO:0032050	clathrin heavy chain binding	10	2	0.4997
GO:0033300	dehydroascorbic acid transmembrane transp	8	2	0.4997
GO:0035198	miRNA binding	37	2	0.4997
GO:0035255	ionotropic glutamate receptor binding	22	2	0.4997
GO:0035662	Toll-like receptor 4 binding	4	2	0.4997
GO:0036041	long-chain fatty acid binding	15	2	0.4997
GO:0038132	neuregulin binding	5	2	0.4997
GO:0038187	pattern recognition receptor activity	27	2	0.4997
GO:0042379	chemokine receptor binding	71	2	0.4997
GO:0042605	peptide antigen binding	37	2	0.4997
GO:0043177	organic acid binding	137	2	0.4997
GO:0043531	ADP binding	34	2	0.4997
GO:0044548	S100 protein binding	14	2	0.4997
GO:0045294	alpha-catenin binding	10	2	0.4997
GO:0045295	gamma-catenin binding	13	2	0.4997
GO:0046790	virion binding	12	2	0.4997
GO:0046977	TAP binding	8	2	0.4997
GO:0048018	receptor ligand activity	491	2	0.4997
GO:0050431	transforming growth factor beta binding	25	2	0.4997
GO:0050542	icosanoid binding	7	2	0.4997
GO:0050543	icosatetraenoic acid binding	6	2	0.4997
GO:0050544	arachidonic acid binding	6	2	0.4997
GO:0050786	RAGE receptor binding	10	2	0.4997
GO:0050998	nitric-oxide synthase binding	13	2	0.4997
GO:0051021	GDP-dissociation inhibitor binding	5	2	0.4997
GO:0051022	Rho GDP-dissociation inhibitor binding	4	2	0.4997
GO:0051059	NF-kappaB binding	31	2	0.4997
GO:0051119	sugar transmembrane transporter activity	29	2	0.4997
GO:0051287	NAD binding	53	2	0.4997
GO:0055056	D-glucose transmembrane transporter activit	12	2	0.4997
GO:0070053	thrombospondin receptor activity	4	2	0.4997
GO:0086083	cell adhesive protein binding involved in bun	5	2	0.4997
GO:0090482	vitamin transmembrane transporter activity	32	2	0.4997
GO:0098847	sequence-specific single stranded DNA bindir	14	2	0.4997
GO:0140296	general transcription initiation factor binding	53	2	0.4997
GO:0140693	molecular condensate scaffold activity	17	2	0.4997
GO:1990247	N6-methyladenosine-containing RNA binding	10	2	0.4997
GO:0008283	cell population proliferation	2062	43	0.5021
GO:0001932	regulation of protein phosphorylation	1121	26	0.5070
GO:0030097	hemopoiesis	920	26	0.5070
GO:0048534	hematopoietic or lymphoid organ developme	962	26	0.5070
GO:0043005	neuron projection	1378	26	0.5070
GO:0042995	cell projection	2368	50	0.5088
GO:0006811	ion transport	1581	33	0.5144
GO:0051130	positive regulation of cellular component org	1050	33	0.5144

GO:0001952	regulation of cell-matrix adhesion	125	9	0.5178
GO:0002221	pattern recognition receptor signaling pathw	187	9	0.5178
GO:0005996	monosaccharide metabolic process	248	9	0.5178
GO:0016567	protein ubiquitination	824	9	0.5178
GO:0030100	regulation of endocytosis	209	9	0.5178
GO:0031623	receptor internalization	121	9	0.5178
GO:0032446	protein modification by small protein conjuga	898	9	0.5178
GO:0045229	external encapsulating structure organization	321	9	0.5178
GO:0046718	viral entry into host cell	151	9	0.5178
GO:0090257	regulation of muscle system process	256	9	0.5178
GO:1901653	cellular response to peptide	373	9	0.5178
GO:1901888	regulation of cell junction assembly	204	9	0.5178
GO:1903039	positive regulation of leukocyte cell-cell adhe	275	9	0.5178
GO:2001234	negative regulation of apoptotic signaling pat	233	9	0.5178
GO:0030662	coated vesicle membrane	198	9	0.5178
GO:0070821	tertiary granule membrane	73	9	0.5178
GO:0004857	enzyme inhibitor activity	395	9	0.5178
GO:0060255	regulation of macromolecule metabolic proc	6762	105	0.5186
GO:0032879	regulation of localization	2129	64	0.5207
GO:0051128	regulation of cellular component organizatio	2369	64	0.5207
GO:0002521	leukocyte differentiation	596	16	0.5251
GO:0007420	brain development	768	16	0.5251
GO:0010558	negative regulation of macromolecule biosyn	1604	16	0.5251
GO:0031327	negative regulation of cellular biosynthetic pr	1673	16	0.5251
GO:0042110	T cell activation	548	16	0.5251
GO:0043254	regulation of protein-containing complex ass	410	16	0.5251
GO:0044089	positive regulation of cellular component bio	498	16	0.5251
GO:0051345	positive regulation of hydrolase activity	589	16	0.5251
GO:0060322	head development	815	16	0.5251
GO:0007417	central nervous system development	1060	23	0.5308
GO:0101002	ficolin-1-rich granule	185	23	0.5308
GO:0044085	cellular component biogenesis	3273	78	0.5315
GO:0036211	protein modification process	3645	54	0.5319
GO:0043412	macromolecule modification	3869	54	0.5319
GO:0065008	regulation of biological quality	3773	102	0.5329
GO:2000026	regulation of multicellular organismal develo	1467	30	0.5359
GO:0042981	regulation of apoptotic process	1506	37	0.5406
GO:0043067	regulation of programmed cell death	1541	37	0.5406
GO:0009605	response to external stimulus	2886	68	0.5420
GO:0043167	ion binding	6021	92	0.5420
GO:0030335	positive regulation of cell migration	566	20	0.5578
GO:0040017	positive regulation of locomotion	607	20	0.5578
GO:0046903	secretion	951	20	0.5578
GO:0051254	positive regulation of RNA metabolic process	1843	20	0.5578
GO:0051493	regulation of cytoskeleton organization	539	20	0.5578
GO:2000147	positive regulation of cell motility	592	20	0.5578
GO:0140513	nuclear protein-containing complex	1295	20	0.5578

GO:0007186	G protein-coupled receptor signaling pathwa	1278	13	0.5580
GO:0032102	negative regulation of response to external s	436	13	0.5580
GO:0044057	regulation of system process	594	13	0.5580
GO:0051046	regulation of secretion	623	13	0.5580
GO:0051701	biological process involved in interaction with	203	13	0.5580
GO:0062197	cellular response to chemical stress	345	13	0.5580
GO:0016740	transferase activity	2242	13	0.5580
GO:0002520	immune system development	1024	27	0.5594
GO:0007049	cell cycle	1823	27	0.5594
GO:0010467	gene expression	7096	96	0.5617
GO:0043085	positive regulation of catalytic activity	1191	34	0.5618
GO:0031983	vesicle lumen	327	34	0.5618
GO:0034774	secretory granule lumen	322	34	0.5618
GO:0060205	cytoplasmic vesicle lumen	325	34	0.5618
GO:0010941	regulation of cell death	1698	41	0.5647
GO:0005783	endoplasmic reticulum	2020	41	0.5647
GO:0001909	leukocyte mediated cytotoxicity	134	6	0.5647
GO:0002440	production of molecular mediator of immune	333	6	0.5647
GO:0002573	myeloid leukocyte differentiation	218	6	0.5647
GO:0006096	glycolytic process	83	6	0.5647
GO:0006165	nucleoside diphosphate phosphorylation	100	6	0.5647
GO:0006469	negative regulation of protein kinase activity	216	6	0.5647
GO:0006631	fatty acid metabolic process	400	6	0.5647
GO:0006757	ATP generation from ADP	84	6	0.5647
GO:0007033	vacuole organization	213	6	0.5647
GO:0007346	regulation of mitotic cell cycle	517	6	0.5647
GO:0008361	regulation of cell size	183	6	0.5647
GO:0009132	nucleoside diphosphate metabolic process	132	6	0.5647
GO:0009135	purine nucleoside diphosphate metabolic pro	104	6	0.5647
GO:0009179	purine ribonucleoside diphosphate metabolic	104	6	0.5647
GO:0009185	ribonucleoside diphosphate metabolic proces	114	6	0.5647
GO:0010232	vascular transport	87	6	0.5647
GO:0015849	organic acid transport	324	6	0.5647
GO:0015850	organic hydroxy compound transport	292	6	0.5647
GO:0021782	glial cell development	117	6	0.5647
GO:0030307	positive regulation of cell growth	167	6	0.5647
GO:0032479	regulation of type I interferon production	105	6	0.5647
GO:0032481	positive regulation of type I interferon produ	63	6	0.5647
GO:0032606	type I interferon production	105	6	0.5647
GO:0032868	response to insulin	269	6	0.5647
GO:0034341	response to interferon-gamma	140	6	0.5647
GO:0035304	regulation of protein dephosphorylation	94	6	0.5647
GO:0044788	modulation by host of viral process	46	6	0.5647
GO:0045927	positive regulation of growth	261	6	0.5647
GO:0046031	ADP metabolic process	92	6	0.5647
GO:0046939	nucleotide phosphorylation	102	6	0.5647
GO:0048609	multicellular organismal reproductive proces	882	6	0.5647

GO:0050673	epithelial cell proliferation	481	6	0.5647
GO:0050730	regulation of peptidyl-tyrosine phosphorylati	266	6	0.5647
GO:0050792	regulation of viral process	162	6	0.5647
GO:0050819	negative regulation of coagulation	54	6	0.5647
GO:0051225	spindle assembly	128	6	0.5647
GO:0051972	regulation of telomerase activity	47	6	0.5647
GO:0061045	negative regulation of wound healing	78	6	0.5647
GO:0061351	neural precursor cell proliferation	149	6	0.5647
GO:0071346	cellular response to interferon-gamma	118	6	0.5647
GO:0071375	cellular response to peptide hormone stimulu	306	6	0.5647
GO:0150104	transport across blood-brain barrier	87	6	0.5647
GO:1903035	negative regulation of response to wounding	94	6	0.5647
GO:0042383	sarcolemma	136	6	0.5647
GO:0101003	ficolin-1-rich granule membrane	61	6	0.5647
GO:0015631	tubulin binding	375	6	0.5647
GO:0030246	carbohydrate binding	275	6	0.5647
GO:0097110	scaffold protein binding	66	6	0.5647
GO:0009059	macromolecule biosynthetic process	4806	62	0.5748
GO:0051239	regulation of multicellular organismal proces	2907	62	0.5748
GO:0006950	response to stress	4044	93	0.5762
GO:0044087	regulation of cellular component biogenesis	959	31	0.5847
GO:0044093	positive regulation of molecular function	1587	45	0.5871
GO:0006082	organic acid metabolic process	964	17	0.5888
GO:0010942	positive regulation of cell death	614	17	0.5888
GO:0019752	carboxylic acid metabolic process	930	17	0.5888
GO:0043436	oxoacid metabolic process	957	17	0.5888
GO:0055065	metal ion homeostasis	519	17	0.5888
GO:1904813	ficolin-1-rich granule lumen	124	17	0.5888
GO:0005525	GTP binding	379	17	0.5888
GO:0019001	guanyl nucleotide binding	401	17	0.5888
GO:0032561	guanyl ribonucleotide binding	401	17	0.5888
GO:0005829	cytosol	5418	131	0.5899
GO:0019538	protein metabolic process	5494	97	0.5955
GO:0048518	positive regulation of biological process	6421	121	0.5974
GO:0006820	anion transport	508	10	0.5996
GO:0030098	lymphocyte differentiation	419	10	0.5996
GO:0032943	mononuclear cell proliferation	312	10	0.5996
GO:0044409	entry into host	158	10	0.5996
GO:0045944	positive regulation of transcription by RNA po	1237	10	0.5996
GO:0046651	lymphocyte proliferation	305	10	0.5996
GO:0050727	regulation of inflammatory response	414	10	0.5996
GO:0070647	protein modification by small protein conjuga	1039	10	0.5996
GO:0110053	regulation of actin filament organization	278	10	0.5996
GO:0009898	cytoplasmic side of plasma membrane	173	10	0.5996
GO:0030133	transport vesicle	423	10	0.5996
GO:0031256	leading edge membrane	177	10	0.5996
GO:0016020	membrane	9649	172	0.6033

GO:0005789	endoplasmic reticulum membrane	1163	28	0.6095
GO:0098827	endoplasmic reticulum subcompartment	1166	28	0.6095
GO:0043170	macromolecule metabolic process	10395	135	0.6100
GO:0051246	regulation of protein metabolic process	2668	63	0.6116
GO:0006816	calcium ion transport	440	14	0.6259
GO:0006935	chemotaxis	621	14	0.6259
GO:0010035	response to inorganic substance	561	14	0.6259
GO:0032787	monocarboxylic acid metabolic process	616	14	0.6259
GO:0032956	regulation of actin cytoskeleton organization	365	14	0.6259
GO:0042330	taxis	623	14	0.6259
GO:0045934	negative regulation of nucleobase-containing	1550	14	0.6259
GO:0051253	negative regulation of RNA metabolic proces	1418	14	0.6259
GO:0051301	cell division	638	14	0.6259
GO:0000139	Golgi membrane	667	14	0.6259
GO:0072562	blood microparticle	145	14	0.6259
GO:0043207	response to external biotic stimulus	1551	46	0.6275
GO:0050790	regulation of catalytic activity	2380	46	0.6275
GO:0051707	response to other organism	1548	46	0.6275
GO:0120025	plasma membrane bounded cell projection	2258	46	0.6275
GO:0006508	proteolysis	1791	32	0.6312
GO:0001504	neurotransmitter uptake	44	3	0.6471
GO:0001764	neuron migration	177	3	0.6471
GO:0001824	blastocyst development	116	3	0.6471
GO:0001913	T cell mediated cytotoxicity	51	3	0.6471
GO:0001914	regulation of T cell mediated cytotoxicity	41	3	0.6471
GO:0001916	positive regulation of T cell mediated cytotox	31	3	0.6471
GO:0002042	cell migration involved in sprouting angiogen	94	3	0.6471
GO:0002227	innate immune response in mucosa	28	3	0.6471
GO:0002251	organ or tissue specific immune response	43	3	0.6471
GO:0002275	myeloid cell activation involved in immune re	96	3	0.6471
GO:0002385	mucosal immune response	39	3	0.6471
GO:0002418	immune response to tumor cell	27	3	0.6471
GO:0002483	antigen processing and presentation of endo	19	3	0.6471
GO:0002495	antigen processing and presentation of pepti	34	3	0.6471
GO:0002691	regulation of cellular extravasation	41	3	0.6471
GO:0002695	negative regulation of leukocyte activation	198	3	0.6471
GO:0002715	regulation of natural killer cell mediated imm	51	3	0.6471
GO:0006007	glucose catabolic process	25	3	0.6471
GO:0006302	double-strand break repair	300	3	0.6471
GO:0006405	RNA export from nucleus	90	3	0.6471
GO:0006695	cholesterol biosynthetic process	58	3	0.6471
GO:0006734	NADH metabolic process	37	3	0.6471
GO:0006735	NADH regeneration	16	3	0.6471
GO:0006879	cellular iron ion homeostasis	66	3	0.6471
GO:0006888	endoplasmic reticulum to Golgi vesicle-media	134	3	0.6471
GO:0006970	response to osmotic stress	86	3	0.6471
GO:0007029	endoplasmic reticulum organization	93	3	0.6471

GO:0007193	adenylate cyclase-inhibiting G protein-couple	79	3	0.6471
GO:0007369	gastrulation	196	3	0.6471
GO:0007519	skeletal muscle tissue development	164	3	0.6471
GO:0007600	sensory perception	1006	3	0.6471
GO:0007611	learning or memory	273	3	0.6471
GO:0008203	cholesterol metabolic process	139	3	0.6471
GO:0008608	attachment of spindle microtubules to kinetc	43	3	0.6471
GO:0009416	response to light stimulus	321	3	0.6471
GO:0009743	response to carbohydrate	234	3	0.6471
GO:0009746	response to hexose	199	3	0.6471
GO:0009749	response to glucose	194	3	0.6471
GO:0010596	negative regulation of endothelial cell migrat	97	3	0.6471
GO:0010633	negative regulation of epithelial cell migratio	113	3	0.6471
GO:0010763	positive regulation of fibroblast migration	17	3	0.6471
GO:0010954	positive regulation of protein processing	26	3	0.6471
GO:0014074	response to purine-containing compound	142	3	0.6471
GO:0015911	long-chain fatty acid import across plasma m	10	3	0.6471
GO:0016042	lipid catabolic process	336	3	0.6471
GO:0016125	sterol metabolic process	154	3	0.6471
GO:0016126	sterol biosynthetic process	65	3	0.6471
GO:0019883	antigen processing and presentation of endo	26	3	0.6471
GO:0019885	antigen processing and presentation of endo	17	3	0.6471
GO:0019886	antigen processing and presentation of exoge	30	3	0.6471
GO:0030183	B cell differentiation	152	3	0.6471
GO:0030433	ubiquitin-dependent ERAD pathway	87	3	0.6471
GO:0030522	intracellular receptor signaling pathway	266	3	0.6471
GO:0032091	negative regulation of protein binding	96	3	0.6471
GO:0032147	activation of protein kinase activity	148	3	0.6471
GO:0032465	regulation of cytokinesis	95	3	0.6471
GO:0032608	interferon-beta production	57	3	0.6471
GO:0032637	interleukin-8 production	104	3	0.6471
GO:0032648	regulation of interferon-beta production	57	3	0.6471
GO:0032677	regulation of interleukin-8 production	103	3	0.6471
GO:0032728	positive regulation of interferon-beta produc	39	3	0.6471
GO:0032729	positive regulation of interferon-gamma proc	77	3	0.6471
GO:0032757	positive regulation of interleukin-8 productio	63	3	0.6471
GO:0032890	regulation of organic acid transport	75	3	0.6471
GO:0032946	positive regulation of mononuclear cell prolif	149	3	0.6471
GO:0033119	negative regulation of RNA splicing	28	3	0.6471
GO:0033135	regulation of peptidyl-serine phosphorylation	143	3	0.6471
GO:0033138	positive regulation of peptidyl-serine phosph	108	3	0.6471
GO:0033631	cell-cell adhesion mediated by integrin	17	3	0.6471
GO:0034112	positive regulation of homotypic cell-cell adh	16	3	0.6471
GO:0034284	response to monosaccharide	207	3	0.6471
GO:0034394	protein localization to cell surface	69	3	0.6471
GO:0035305	negative regulation of dephosphorylation	48	3	0.6471
GO:0035307	positive regulation of protein dephosphoryla	49	3	0.6471

GO:0035308	negative regulation of protein dephosphoryla	34	3	0.6471
GO:0036230	granulocyte activation	47	3	0.6471
GO:0036503	ERAD pathway	112	3	0.6471
GO:0042102	positive regulation of T cell proliferation	106	3	0.6471
GO:0042116	macrophage activation	109	3	0.6471
GO:0042119	neutrophil activation	41	3	0.6471
GO:0042177	negative regulation of protein catabolic proc	111	3	0.6471
GO:0042269	regulation of natural killer cell mediated cyto	47	3	0.6471
GO:0042982	amyloid precursor protein metabolic process	95	3	0.6471
GO:0042987	amyloid precursor protein catabolic process	68	3	0.6471
GO:0043030	regulation of macrophage activation	62	3	0.6471
GO:0043032	positive regulation of macrophage activation	30	3	0.6471
GO:0043409	negative regulation of MAPK cascade	181	3	0.6471
GO:0043537	negative regulation of blood vessel endotheli	78	3	0.6471
GO:0044539	long-chain fatty acid import into cell	17	3	0.6471
GO:0044827	modulation by host of viral genome replicatio	23	3	0.6471
GO:0044829	positive regulation by host of viral genome re	9	3	0.6471
GO:0045069	regulation of viral genome replication	87	3	0.6471
GO:0045198	establishment of epithelial cell apical/basal p	17	3	0.6471
GO:0045621	positive regulation of lymphocyte differentiat	130	3	0.6471
GO:0045665	negative regulation of neuron differentiation	73	3	0.6471
GO:0045667	regulation of osteoblast differentiation	141	3	0.6471
GO:0045669	positive regulation of osteoblast differentiati	75	3	0.6471
GO:0045824	negative regulation of innate immune respon	77	3	0.6471
GO:0045920	negative regulation of exocytosis	38	3	0.6471
GO:0045933	positive regulation of muscle contraction	49	3	0.6471
GO:0046631	alpha-beta T cell activation	174	3	0.6471
GO:0046683	response to organophosphorus	128	3	0.6471
GO:0046931	pore complex assembly	20	3	0.6471
GO:0048025	negative regulation of mRNA splicing, via spli	23	3	0.6471
GO:0048638	regulation of developmental growth	332	3	0.6471
GO:0050671	positive regulation of lymphocyte proliferatio	146	3	0.6471
GO:0050686	negative regulation of mRNA processing	25	3	0.6471
GO:0050890	cognition	314	3	0.6471
GO:0051048	negative regulation of secretion	175	3	0.6471
GO:0051131	chaperone-mediated protein complex assem	23	3	0.6471
GO:0051250	negative regulation of lymphocyte activation	166	3	0.6471
GO:0051279	regulation of release of sequestered calcium	82	3	0.6471
GO:0051302	regulation of cell division	190	3	0.6471
GO:0051496	positive regulation of stress fiber assembly	52	3	0.6471
GO:0051580	regulation of neurotransmitter uptake	18	3	0.6471
GO:0051592	response to calcium ion	147	3	0.6471
GO:0051983	regulation of chromosome segregation	132	3	0.6471
GO:0051988	regulation of attachment of spindle microtub	18	3	0.6471
GO:0055001	muscle cell development	191	3	0.6471
GO:0055007	cardiac muscle cell differentiation	124	3	0.6471
GO:0060538	skeletal muscle organ development	176	3	0.6471

GO:0060562	epithelial tube morphogenesis	335	3	0.6471
GO:0061462	protein localization to lysosome	52	3	0.6471
GO:0061615	glycolytic process through fructose-6-phosph	19	3	0.6471
GO:0061620	glycolytic process through glucose-6-phospha	18	3	0.6471
GO:0061621	canonical glycolysis	16	3	0.6471
GO:0061684	chaperone-mediated autophagy	16	3	0.6471
GO:0061718	glucose catabolic process to pyruvate	16	3	0.6471
GO:0070555	response to interleukin-1	141	3	0.6471
GO:0070972	protein localization to endoplasmic reticulum	78	3	0.6471
GO:0071260	cellular response to mechanical stimulus	73	3	0.6471
GO:0071347	cellular response to interleukin-1	111	3	0.6471
GO:0071786	endoplasmic reticulum tubular network orga	18	3	0.6471
GO:0071800	podosome assembly	19	3	0.6471
GO:0071801	regulation of podosome assembly	14	3	0.6471
GO:0071803	positive regulation of podosome assembly	11	3	0.6471
GO:0072665	protein localization to vacuole	78	3	0.6471
GO:0086091	regulation of heart rate by cardiac conductio	42	3	0.6471
GO:0090092	regulation of transmembrane receptor prote	290	3	0.6471
GO:0090136	epithelial cell-cell adhesion	17	3	0.6471
GO:0090162	establishment of epithelial cell polarity	34	3	0.6471
GO:0090287	regulation of cellular response to growth fact	338	3	0.6471
GO:0097306	cellular response to alcohol	100	3	0.6471
GO:0098586	cellular response to virus	116	3	0.6471
GO:0098901	regulation of cardiac muscle cell action poter	31	3	0.6471
GO:0099563	modification of synaptic structure	19	3	0.6471
GO:0140354	lipid import into cell	19	3	0.6471
GO:1901987	regulation of cell cycle phase transition	453	3	0.6471
GO:1902001	fatty acid transmembrane transport	18	3	0.6471
GO:1902106	negative regulation of leukocyte differentiati	108	3	0.6471
GO:1902107	positive regulation of leukocyte differentiatio	184	3	0.6471
GO:1902175	regulation of oxidative stress-induced intrinsi	29	3	0.6471
GO:1902622	regulation of neutrophil migration	47	3	0.6471
GO:1902624	positive regulation of neutrophil migration	34	3	0.6471
GO:1902652	secondary alcohol metabolic process	149	3	0.6471
GO:1902653	secondary alcohol biosynthetic process	58	3	0.6471
GO:1903053	regulation of extracellular matrix organizatio	57	3	0.6471
GO:1903078	positive regulation of protein localization to p	59	3	0.6471
GO:1903201	regulation of oxidative stress-induced cell de	80	3	0.6471
GO:1903319	positive regulation of protein maturation	28	3	0.6471
GO:1903531	negative regulation of secretion by cell	151	3	0.6471
GO:1903533	regulation of protein targeting	78	3	0.6471
GO:1903556	negative regulation of tumor necrosis factor s	79	3	0.6471
GO:1903708	positive regulation of hemopoiesis	184	3	0.6471
GO:1904377	positive regulation of protein localization to c	67	3	0.6471
GO:1904427	positive regulation of calcium ion transmemb	93	3	0.6471
GO:1905517	macrophage migration	60	3	0.6471
GO:1905521	regulation of macrophage migration	44	3	0.6471

GO:1905954	positive regulation of lipid localization	113	3	0.6471
GO:1990868	response to chemokine	98	3	0.6471
GO:1990869	cellular response to chemokine	98	3	0.6471
GO:2000179	positive regulation of neural precursor cell pr	55	3	0.6471
GO:2000241	regulation of reproductive process	206	3	0.6471
GO:2001020	regulation of response to DNA damage stimu	312	3	0.6471
GO:2001243	negative regulation of intrinsic apoptotic sigr	101	3	0.6471
GO:2001258	negative regulation of cation channel activity	63	3	0.6471
GO:0000502	proteasome complex	59	3	0.6471
GO:0000775	chromosome, centromeric region	250	3	0.6471
GO:0001772	immunological synapse	45	3	0.6471
GO:0005795	Golgi stack	153	3	0.6471
GO:0005798	Golgi-associated vesicle	89	3	0.6471
GO:0005884	actin filament	114	3	0.6471
GO:0005916	fascia adherens	9	3	0.6471
GO:0030669	clathrin-coated endocytic vesicle membrane	72	3	0.6471
GO:0030672	synaptic vesicle membrane	114	3	0.6471
GO:0031225	anchored component of membrane	169	3	0.6471
GO:0032839	dendrite cytoplasm	31	3	0.6471
GO:0033017	sarcoplasmic reticulum membrane	41	3	0.6471
GO:0033116	endoplasmic reticulum-Golgi intermediate co	80	3	0.6471
GO:0034451	centriolar satellite	108	3	0.6471
GO:0042611	MHC protein complex	25	3	0.6471
GO:0042612	MHC class I protein complex	9	3	0.6471
GO:0044233	mitochondria-associated endoplasmic reticul	21	3	0.6471
GO:0045211	postsynaptic membrane	269	3	0.6471
GO:0051233	spindle midzone	36	3	0.6471
GO:0099501	exocytic vesicle membrane	114	3	0.6471
GO:0000287	magnesium ion binding	225	3	0.6471
GO:0001968	fibronectin binding	31	3	0.6471
GO:0003823	antigen binding	171	3	0.6471
GO:0004866	endopeptidase inhibitor activity	180	3	0.6471
GO:0005044	scavenger receptor activity	47	3	0.6471
GO:0005126	cytokine receptor binding	273	3	0.6471
GO:0005319	lipid transporter activity	161	3	0.6471
GO:0005516	calmodulin binding	200	3	0.6471
GO:0008201	heparin binding	171	3	0.6471
GO:0015035	protein-disulfide reductase activity	39	3	0.6471
GO:0030414	peptidase inhibitor activity	187	3	0.6471
GO:0034185	apolipoprotein binding	18	3	0.6471
GO:0035254	glutamate receptor binding	42	3	0.6471
GO:0036002	pre-mRNA binding	57	3	0.6471
GO:0042162	telomeric DNA binding	37	3	0.6471
GO:0043236	laminin binding	28	3	0.6471
GO:0048029	monosaccharide binding	72	3	0.6471
GO:0050661	NADP binding	53	3	0.6471
GO:0061135	endopeptidase regulator activity	194	3	0.6471

GO:0061980	regulatory RNA binding	51	3	0.6471
GO:0070063	RNA polymerase binding	59	3	0.6471
GO:0071813	lipoprotein particle binding	30	3	0.6471
GO:0071814	protein-lipid complex binding	30	3	0.6471
GO:0086080	protein binding involved in heterotypic cell-c	12	3	0.6471
GO:0000278	mitotic cell cycle	946	18	0.6479
GO:0006629	lipid metabolic process	1415	18	0.6479
GO:0031347	regulation of defense response	680	18	0.6479
GO:0050801	ion homeostasis	626	18	0.6479
GO:0050821	protein stabilization	214	18	0.6479
GO:0055080	cation homeostasis	601	18	0.6479
GO:0071407	cellular response to organic cyclic compound	581	18	0.6479
GO:0098771	inorganic ion homeostasis	616	18	0.6479
GO:0016491	oxidoreductase activity	735	18	0.6479
GO:0043227	membrane-bounded organelle	13883	203	0.6559
GO:0045087	innate immune response	927	29	0.6568
GO:0042175	nuclear outer membrane-endoplasmic reticu	1186	29	0.6568
GO:0006090	pyruvate metabolic process	108	7	0.6572
GO:0006325	chromatin organization	624	7	0.6572
GO:0006470	protein dephosphorylation	261	7	0.6572
GO:0009612	response to mechanical stimulus	211	7	0.6572
GO:0010001	glial cell differentiation	227	7	0.6572
GO:0010498	proteasomal protein catabolic process	521	7	0.6572
GO:0010632	regulation of epithelial cell migration	292	7	0.6572
GO:0016052	carbohydrate catabolic process	155	7	0.6572
GO:0019079	viral genome replication	130	7	0.6572
GO:0019318	hexose metabolic process	229	7	0.6572
GO:0019882	antigen processing and presentation	108	7	0.6572
GO:0030217	T cell differentiation	296	7	0.6572
GO:0032635	interleukin-6 production	176	7	0.6572
GO:0032675	regulation of interleukin-6 production	176	7	0.6572
GO:0032760	positive regulation of tumor necrosis factor p	105	7	0.6572
GO:0033673	negative regulation of kinase activity	240	7	0.6572
GO:0034113	heterotypic cell-cell adhesion	61	7	0.6572
GO:0035303	regulation of dephosphorylation	133	7	0.6572
GO:0043161	proteasome-mediated ubiquitin-dependent p	450	7	0.6572
GO:0044770	cell cycle phase transition	576	7	0.6572
GO:0045055	regulated exocytosis	242	7	0.6572
GO:0051851	modulation by host of symbiont process	84	7	0.6572
GO:0055076	transition metal ion homeostasis	143	7	0.6572
GO:1902105	regulation of leukocyte differentiation	317	7	0.6572
GO:1903557	positive regulation of tumor necrosis factor s	109	7	0.6572
GO:0030658	transport vesicle membrane	222	7	0.6572
GO:0055037	recycling endosome	198	7	0.6572
GO:0098984	neuron to neuron synapse	351	7	0.6572
GO:0019887	protein kinase regulator activity	218	7	0.6572
GO:0098631	cell adhesion mediator activity	64	7	0.6572

GO:1901681	sulfur compound binding	270	7	0.6572
GO:0051173	positive regulation of nitrogen compound me	3175	61	0.6642
GO:0090304	nucleic acid metabolic process	5606	61	0.6642
GO:0044271	cellular nitrogen compound biosynthetic pro	4865	68	0.6648
GO:0002699	positive regulation of immune effector proce	261	11	0.6720
GO:0031349	positive regulation of defense response	307	11	0.6720
GO:0033674	positive regulation of kinase activity	496	11	0.6720
GO:0045088	regulation of innate immune response	240	11	0.6720
GO:0048545	response to steroid hormone	339	11	0.6720
GO:0051098	regulation of binding	372	11	0.6720
GO:0070661	leukocyte proliferation	348	11	0.6720
GO:0072593	reactive oxygen species metabolic process	235	11	0.6720
GO:1900182	positive regulation of protein localization to r	87	11	0.6720
GO:1901652	response to peptide	509	11	0.6720
GO:0030666	endocytic vesicle membrane	194	11	0.6720
GO:0035770	ribonucleoprotein granule	265	11	0.6720
GO:0036464	cytoplasmic ribonucleoprotein granule	248	11	0.6720
GO:0098562	cytoplasmic side of membrane	204	11	0.6720
GO:0046914	transition metal ion binding	1097	11	0.6720
GO:0016043	cellular component organization	6395	120	0.6749
GO:0009891	positive regulation of biosynthetic process	2074	33	0.6751
GO:0031328	positive regulation of cellular biosynthetic pr	2032	33	0.6751
GO:0042127	regulation of cell population proliferation	1769	33	0.6751
GO:0005654	nucleoplasm	4155	51	0.6833
GO:0097367	carbohydrate derivative binding	2255	51	0.6833
GO:0002764	immune response-regulating signaling pathw	503	15	0.6870
GO:0006457	protein folding	223	15	0.6870
GO:0006979	response to oxidative stress	434	15	0.6870
GO:0007015	actin filament organization	454	15	0.6870
GO:0032103	positive regulation of response to external st	464	15	0.6870
GO:0034504	protein localization to nucleus	306	15	0.6870
GO:0043065	positive regulation of apoptotic process	532	15	0.6870
GO:0043068	positive regulation of programmed cell death	547	15	0.6870
GO:0003924	GTPase activity	331	15	0.6870
GO:0008152	metabolic process	12433	161	0.6906
GO:0031324	negative regulation of cellular metabolic proc	2345	37	0.6919
GO:0031399	regulation of protein modification process	1579	37	0.6919
GO:0009890	negative regulation of biosynthetic process	1702	19	0.7015
GO:0031647	regulation of protein stability	325	19	0.7015
GO:0043086	negative regulation of catalytic activity	796	19	0.7015
GO:0043549	regulation of kinase activity	786	19	0.7015
GO:0007010	cytoskeleton organization	1500	48	0.7026
GO:0009607	response to biotic stimulus	1593	48	0.7026
GO:0071840	cellular component organization or biogenes	6601	121	0.7052
GO:0005575	cellular_component	19869	225	0.7080
GO:0110165	cellular anatomical entity	19469	225	0.7080
GO:0043066	negative regulation of apoptotic process	914	23	0.7153

GO:0043069	negative regulation of programmed cell deat	938	23	0.7153
GO:0070925	organelle assembly	935	23	0.7153
GO:0051247	positive regulation of protein metabolic proc	1516	45	0.7226
GO:0005794	Golgi apparatus	1652	27	0.7285
GO:0019899	enzyme binding	2086	63	0.7299
GO:0006511	ubiquitin-dependent protein catabolic proces	680	8	0.7335
GO:0007044	cell-substrate junction assembly	95	8	0.7335
GO:0010564	regulation of cell cycle process	735	8	0.7335
GO:0010950	positive regulation of endopeptidase activity	174	8	0.7335
GO:0010952	positive regulation of peptidase activity	194	8	0.7335
GO:0016485	protein processing	250	8	0.7335
GO:0031396	regulation of protein ubiquitination	210	8	0.7335
GO:0043280	positive regulation of cysteine-type endopep	125	8	0.7335
GO:0043393	regulation of protein binding	200	8	0.7335
GO:0043434	response to peptide hormone	421	8	0.7335
GO:0045089	positive regulation of innate immune respons	145	8	0.7335
GO:0048872	homeostasis of number of cells	296	8	0.7335
GO:0050870	positive regulation of T cell activation	251	8	0.7335
GO:0051346	negative regulation of hydrolase activity	373	8	0.7335
GO:0071356	cellular response to tumor necrosis factor	234	8	0.7335
GO:0150115	cell-substrate junction organization	101	8	0.7335
GO:1902115	regulation of organelle assembly	207	8	0.7335
GO:1903320	regulation of protein modification by small pi	252	8	0.7335
GO:2001056	positive regulation of cysteine-type endopep	143	8	0.7335
GO:0000785	chromatin	1306	8	0.7335
GO:0030136	clathrin-coated vesicle	211	8	0.7335
GO:0030863	cortical cytoskeleton	108	8	0.7335
GO:0032587	ruffle membrane	97	8	0.7335
GO:0035578	azurophil granule lumen	91	8	0.7335
GO:0071013	catalytic step 2 spliceosome	90	8	0.7335
GO:0019207	kinase regulator activity	246	8	0.7335
GO:0001558	regulation of cell growth	422	12	0.7346
GO:0002696	positive regulation of leukocyte activation	450	12	0.7346
GO:0005975	carbohydrate metabolic process	593	12	0.7346
GO:0032870	cellular response to hormone stimulus	595	12	0.7346
GO:0050863	regulation of T cell activation	376	12	0.7346
GO:0051054	positive regulation of DNA metabolic process	301	12	0.7346
GO:0052548	regulation of endopeptidase activity	428	12	0.7346
GO:0071674	mononuclear cell migration	206	12	0.7346
GO:1902903	regulation of supramolecular fiber organizati	384	12	0.7346
GO:0002694	regulation of leukocyte activation	670	16	0.7410
GO:0045859	regulation of protein kinase activity	672	16	0.7410
GO:0051347	positive regulation of transferase activity	588	16	0.7410
GO:2001233	regulation of apoptotic signaling pathway	374	16	0.7410
GO:0010557	positive regulation of macromolecule biosynt	1918	31	0.7412
GO:0030855	epithelial cell differentiation	715	31	0.7412
GO:0045935	positive regulation of nucleobase-containing	2052	31	0.7412

GO:0001819	positive regulation of cytokine production	486	20	0.7494
GO:0051093	negative regulation of developmental proces	1002	20	0.7494
GO:0005515	protein binding	14027	211	0.7508
GO:0001910	regulation of leukocyte mediated cytotoxicity	87	4	0.7516
GO:0001912	positive regulation of leukocyte mediated cyt	59	4	0.7516
GO:0002218	activation of innate immune response	64	4	0.7516
GO:0002456	T cell mediated immunity	113	4	0.7516
GO:0002474	antigen processing and presentation of pepti	30	4	0.7516
GO:0002504	antigen processing and presentation of pepti	36	4	0.7516
GO:0002526	acute inflammatory response	114	4	0.7516
GO:0002544	chronic inflammatory response	19	4	0.7516
GO:0002698	negative regulation of immune effector proce	122	4	0.7516
GO:0002709	regulation of T cell mediated immunity	89	4	0.7516
GO:0002711	positive regulation of T cell mediated immun	60	4	0.7516
GO:0006310	DNA recombination	332	4	0.7516
GO:0006694	steroid biosynthetic process	178	4	0.7516
GO:0006919	activation of cysteine-type endopeptidase ac	77	4	0.7516
GO:0007006	mitochondrial membrane organization	120	4	0.7516
GO:0007052	mitotic spindle organization	133	4	0.7516
GO:0007188	adenylate cyclase-modulating G protein-coup	248	4	0.7516
GO:0008631	intrinsic apoptotic signaling pathway in respo	45	4	0.7516
GO:0010762	regulation of fibroblast migration	40	4	0.7516
GO:0010951	negative regulation of endopeptidase activity	252	4	0.7516
GO:0014002	astrocyte development	42	4	0.7516
GO:0015908	fatty acid transport	101	4	0.7516
GO:0015909	long-chain fatty acid transport	67	4	0.7516
GO:0015918	sterol transport	135	4	0.7516
GO:0019320	hexose catabolic process	42	4	0.7516
GO:0022617	extracellular matrix disassembly	62	4	0.7516
GO:0030301	cholesterol transport	121	4	0.7516
GO:0030859	polarized epithelial cell differentiation	24	4	0.7516
GO:0030865	cortical cytoskeleton organization	61	4	0.7516
GO:0031343	positive regulation of cell killing	66	4	0.7516
GO:0031397	negative regulation of protein ubiquitination	84	4	0.7516
GO:0032273	positive regulation of protein polymerization	92	4	0.7516
GO:0032392	DNA geometric change	99	4	0.7516
GO:0032410	negative regulation of transporter activity	115	4	0.7516
GO:0032413	negative regulation of ion transmembrane tr	89	4	0.7516
GO:0032609	interferon-gamma production	119	4	0.7516
GO:0032649	regulation of interferon-gamma production	119	4	0.7516
GO:0033209	tumor necrosis factor-mediated signaling pat	109	4	0.7516
GO:0034470	ncRNA processing	439	4	0.7516
GO:0034605	cellular response to heat	66	4	0.7516
GO:0035306	positive regulation of dephosphorylation	64	4	0.7516
GO:0036473	cell death in response to oxidative stress	100	4	0.7516
GO:0042730	fibrinolysis	27	4	0.7516
GO:0043535	regulation of blood vessel endothelial cell mi	150	4	0.7516

GO:0044794	positive regulation by host of viral process	20	4	0.7516
GO:0045744	negative regulation of G protein-coupled rece	54	4	0.7516
GO:0046165	alcohol biosynthetic process	143	4	0.7516
GO:0046365	monosaccharide catabolic process	46	4	0.7516
GO:0048525	negative regulation of viral process	91	4	0.7516
GO:0048708	astrocyte differentiation	82	4	0.7516
GO:0048771	tissue remodeling	179	4	0.7516
GO:0050657	nucleic acid transport	163	4	0.7516
GO:0050658	RNA transport	163	4	0.7516
GO:0050851	antigen receptor-mediated signaling pathway	252	4	0.7516
GO:0050852	T cell receptor signaling pathway	139	4	0.7516
GO:0051236	establishment of RNA localization	166	4	0.7516
GO:0051402	neuron apoptotic process	255	4	0.7516
GO:0051607	defense response to virus	304	4	0.7516
GO:0051926	negative regulation of calcium ion transport	74	4	0.7516
GO:0051928	positive regulation of calcium ion transport	129	4	0.7516
GO:0070486	leukocyte aggregation	13	4	0.7516
GO:0070507	regulation of microtubule cytoskeleton orgar	155	4	0.7516
GO:0070613	regulation of protein processing	67	4	0.7516
GO:0070665	positive regulation of leukocyte proliferation	167	4	0.7516
GO:0071103	DNA conformation change	106	4	0.7516
GO:0071496	cellular response to external stimulus	328	4	0.7516
GO:0071622	regulation of granulocyte chemotaxis	53	4	0.7516
GO:0090307	mitotic spindle assembly	74	4	0.7516
GO:0090322	regulation of superoxide metabolic process	34	4	0.7516
GO:0140546	defense response to symbiont	305	4	0.7516
GO:1901019	regulation of calcium ion transmembrane tra	102	4	0.7516
GO:1901020	negative regulation of calcium ion transmem	44	4	0.7516
GO:1902117	positive regulation of organelle assembly	82	4	0.7516
GO:1903170	negative regulation of calcium ion transmem	56	4	0.7516
GO:1903305	regulation of regulated secretory pathway	145	4	0.7516
GO:1903317	regulation of protein maturation	72	4	0.7516
GO:1903321	negative regulation of protein modification b	96	4	0.7516
GO:1903409	reactive oxygen species biosynthetic process	62	4	0.7516
GO:1903707	negative regulation of hemopoiesis	114	4	0.7516
GO:1903900	regulation of viral life cycle	142	4	0.7516
GO:1904063	negative regulation of cation transmembrane	109	4	0.7516
GO:0002102	podosome	30	4	0.7516
GO:0005741	mitochondrial outer membrane	207	4	0.7516
GO:0005905	clathrin-coated pit	71	4	0.7516
GO:0008021	synaptic vesicle	208	4	0.7516
GO:0012507	ER to Golgi transport vesicle membrane	63	4	0.7516
GO:0030134	COPII-coated ER to Golgi transport vesicle	95	4	0.7516
GO:0031514	motile cilium	244	4	0.7516
GO:0031901	early endosome membrane	183	4	0.7516
GO:0032838	plasma membrane bounded cell projection c	246	4	0.7516
GO:0036126	sperm flagellum	150	4	0.7516

GO:0044232	organelle membrane contact site	48	4	0.7516
GO:0070382	exocytic vesicle	226	4	0.7516
GO:0097225	sperm midpiece	50	4	0.7516
GO:0097729	9+2 motile cilium	165	4	0.7516
GO:0120111	neuron projection cytoplasm	90	4	0.7516
GO:1905368	peptidase complex	118	4	0.7516
GO:1905369	endopeptidase complex	82	4	0.7516
GO:0003713	transcription coactivator activity	281	4	0.7516
GO:0005504	fatty acid binding	49	4	0.7516
GO:0005546	phosphatidylinositol-4,5-bisphosphate bindin	85	4	0.7516
GO:0015036	disulfide oxidoreductase activity	44	4	0.7516
GO:0017022	myosin binding	74	4	0.7516
GO:0019003	GDP binding	73	4	0.7516
GO:0031267	small GTPase binding	272	4	0.7516
GO:0031490	chromatin DNA binding	118	4	0.7516
GO:0033293	monocarboxylic acid binding	81	4	0.7516
GO:0038024	cargo receptor activity	82	4	0.7516
GO:0061134	peptidase regulator activity	232	4	0.7516
GO:1901981	phosphatidylinositol phosphate binding	180	4	0.7516
GO:1902936	phosphatidylinositol bisphosphate binding	109	4	0.7516
GO:1901360	organic cyclic compound metabolic process	6632	78	0.7572
GO:0070887	cellular response to chemical stimulus	3116	71	0.7579
GO:0051336	regulation of hydrolase activity	1025	24	0.7586
GO:1901575	organic substance catabolic process	2120	39	0.7650
GO:0015630	microtubule cytoskeleton	1362	28	0.7682
GO:0010604	positive regulation of macromolecule metabo	3570	75	0.7712
GO:0046483	heterocycle metabolic process	6336	75	0.7712
GO:0009893	positive regulation of metabolic process	3897	79	0.7841
GO:0006139	nucleobase-containing compound metabolic	6165	72	0.7852
GO:0006955	immune response	1955	47	0.7871
GO:0033043	regulation of organelle organization	1186	36	0.7874
GO:0002685	regulation of leukocyte migration	231	13	0.7875
GO:0002697	regulation of immune effector process	379	13	0.7875
GO:0045862	positive regulation of proteolysis	372	13	0.7875
GO:0051051	negative regulation of transport	479	13	0.7875
GO:0051249	regulation of lymphocyte activation	573	13	0.7875
GO:0052547	regulation of peptidase activity	459	13	0.7875
GO:0005635	nuclear envelope	488	13	0.7875
GO:0030135	coated vesicle	310	13	0.7875
GO:0002252	immune effector process	700	21	0.7916
GO:0009894	regulation of catabolic process	997	21	0.7916
GO:0031325	positive regulation of cellular metabolic proc	3133	58	0.7917
GO:0001933	negative regulation of protein phosphorylatio	338	9	0.7951
GO:0002274	myeloid leukocyte activation	237	9	0.7951
GO:0002833	positive regulation of response to biotic stim	188	9	0.7951
GO:0003018	vascular process in circulatory system	270	9	0.7951
GO:0006869	lipid transport	433	9	0.7951

GO:0007162	negative regulation of cell adhesion	308	9	0.7951
GO:0007338	single fertilization	160	9	0.7951
GO:0009566	fertilization	201	9	0.7951
GO:0009895	negative regulation of catabolic process	332	9	0.7951
GO:0010876	lipid localization	483	9	0.7951
GO:0019221	cytokine-mediated signaling pathway	496	9	0.7951
GO:0019941	modification-dependent protein catabolic pro	690	9	0.7951
GO:0034249	negative regulation of cellular amide metabo	285	9	0.7951
GO:0034612	response to tumor necrosis factor	254	9	0.7951
GO:0043281	regulation of cysteine-type endopeptidase ac	205	9	0.7951
GO:0043632	modification-dependent macromolecule cata	702	9	0.7951
GO:0046034	ATP metabolic process	217	9	0.7951
GO:0051251	positive regulation of lymphocyte activation	400	9	0.7951
GO:0051348	negative regulation of transferase activity	276	9	0.7951
GO:0051603	proteolysis involved in protein catabolic proc	788	9	0.7951
GO:2000377	regulation of reactive oxygen species metabo	149	9	0.7951
GO:0005929	cilium	728	9	0.7951
GO:0030496	midbody	203	9	0.7951
GO:0001664	G protein-coupled receptor binding	289	9	0.7951
GO:0003674	molecular_function	18432	223	0.7951
GO:0010648	negative regulation of cell communication	1424	25	0.7971
GO:0023057	negative regulation of signaling	1430	25	0.7971
GO:0048878	chemical homeostasis	1060	25	0.7971
GO:0060548	negative regulation of cell death	1043	25	0.7971
GO:0005856	cytoskeleton	2374	76	0.7973
GO:0010605	negative regulation of macromolecule metab	3336	44	0.8061
GO:0099081	supramolecular polymer	1038	44	0.8061
GO:0099512	supramolecular fiber	1029	44	0.8061
GO:0035639	purine ribonucleoside triphosphate binding	1813	44	0.8061
GO:1901265	nucleoside phosphate binding	2141	55	0.8079
GO:0006725	cellular aromatic compound metabolic proce	6392	73	0.8105
GO:0003676	nucleic acid binding	4309	73	0.8105
GO:0048585	negative regulation of response to stimulus	1706	33	0.8107
GO:0009892	negative regulation of metabolic process	3554	48	0.8153
GO:1901564	organonitrogen compound metabolic process	6535	105	0.8232
GO:0048513	animal organ development	3591	70	0.8237
GO:0002688	regulation of leukocyte chemotaxis	127	5	0.8254
GO:0002690	positive regulation of leukocyte chemotaxis	97	5	0.8254
GO:0006066	alcohol metabolic process	367	5	0.8254
GO:0006281	DNA repair	591	5	0.8254
GO:0006338	chromatin remodeling	373	5	0.8254
GO:0006626	protein targeting to mitochondrion	104	5	0.8254
GO:0007018	microtubule-based movement	415	5	0.8254
GO:0007178	transmembrane receptor protein serine/thre	390	5	0.8254
GO:0007276	gamete generation	765	5	0.8254
GO:0007565	female pregnancy	189	5	0.8254
GO:0007568	aging	169	5	0.8254

GO:0007610	behavior	629	5	0.8254
GO:0008217	regulation of blood pressure	191	5	0.8254
GO:0008277	regulation of G protein-coupled receptor sign	139	5	0.8254
GO:0009408	response to heat	114	5	0.8254
GO:0010466	negative regulation of peptidase activity	263	5	0.8254
GO:0010594	regulation of endothelial cell migration	230	5	0.8254
GO:0010634	positive regulation of epithelial cell migration	176	5	0.8254
GO:0010970	transport along microtubule	168	5	0.8254
GO:0015931	nucleobase-containing compound transport	223	5	0.8254
GO:0019080	viral gene expression	100	5	0.8254
GO:0021537	telencephalon development	255	5	0.8254
GO:0021543	pallium development	174	5	0.8254
GO:0021700	developmental maturation	304	5	0.8254
GO:0021987	cerebral cortex development	117	5	0.8254
GO:0030308	negative regulation of cell growth	188	5	0.8254
GO:0031333	negative regulation of protein-containing cor	146	5	0.8254
GO:0031341	regulation of cell killing	102	5	0.8254
GO:0032233	positive regulation of actin filament bundle a	62	5	0.8254
GO:0032615	interleukin-12 production	63	5	0.8254
GO:0032655	regulation of interleukin-12 production	63	5	0.8254
GO:0032886	regulation of microtubule-based process	252	5	0.8254
GO:0034660	ncRNA metabolic process	634	5	0.8254
GO:0034766	negative regulation of ion transmembrane tra	115	5	0.8254
GO:0042113	B cell activation	350	5	0.8254
GO:0042129	regulation of T cell proliferation	182	5	0.8254
GO:0043271	negative regulation of ion transport	167	5	0.8254
GO:0044282	small molecule catabolic process	385	5	0.8254
GO:0044703	multi-organism reproductive process	209	5	0.8254
GO:0044706	multi-multicellular organism process	218	5	0.8254
GO:0045926	negative regulation of growth	249	5	0.8254
GO:0048144	fibroblast proliferation	103	5	0.8254
GO:0048598	embryonic morphogenesis	614	5	0.8254
GO:0050777	negative regulation of immune response	190	5	0.8254
GO:0050803	regulation of synapse structure or activity	215	5	0.8254
GO:0050807	regulation of synapse organization	209	5	0.8254
GO:0050920	regulation of chemotaxis	230	5	0.8254
GO:0050921	positive regulation of chemotaxis	144	5	0.8254
GO:0051384	response to glucocorticoid	142	5	0.8254
GO:0051494	negative regulation of cytoskeleton organizat	165	5	0.8254
GO:0051973	positive regulation of telomerase activity	34	5	0.8254
GO:0055072	iron ion homeostasis	88	5	0.8254
GO:0090068	positive regulation of cell cycle process	260	5	0.8254
GO:0098656	anion transmembrane transport	266	5	0.8254
GO:0099111	microtubule-based transport	209	5	0.8254
GO:1901617	organic hydroxy compound biosynthetic proc	245	5	0.8254
GO:1902904	negative regulation of supramolecular fiber o	169	5	0.8254
GO:1905952	regulation of lipid localization	185	5	0.8254

GO:2000379	positive regulation of reactive oxygen species	71	5	0.8254
GO:2000765	regulation of cytoplasmic translation	29	5	0.8254
GO:2000767	positive regulation of cytoplasmic translation	15	5	0.8254
GO:0005902	microvillus	93	5	0.8254
GO:0016528	sarcoplasm	89	5	0.8254
GO:0016529	sarcoplasmic reticulum	73	5	0.8254
GO:0055038	recycling endosome membrane	97	5	0.8254
GO:1990204	oxidoreductase complex	125	5	0.8254
GO:0003682	chromatin binding	597	5	0.8254
GO:0003730	mRNA 3'-UTR binding	238	5	0.8254
GO:0016667	oxidoreductase activity, acting on a sulfur gro	59	5	0.8254
GO:0031406	carboxylic acid binding	171	5	0.8254
GO:0098632	cell-cell adhesion mediator activity	54	5	0.8254
GO:0030162	regulation of proteolysis	750	18	0.8278
GO:0072594	establishment of protein localization to orgar	449	18	0.8278
GO:0140694	non-membrane-bounded organelle assembly	386	18	0.8278
GO:0009057	macromolecule catabolic process	1385	26	0.8308
GO:0051338	regulation of transferase activity	911	26	0.8308
GO:0006091	generation of precursor metabolites and ene	505	14	0.8316
GO:0010563	negative regulation of phosphorus metabolic	441	14	0.8316
GO:0019953	sexual reproduction	896	14	0.8316
GO:0040008	regulation of growth	624	14	0.8316
GO:0045936	negative regulation of phosphate metabolic p	440	14	0.8316
GO:0051726	regulation of cell cycle	1145	14	0.8316
GO:0001533	cornified envelope	59	14	0.8316
GO:0005694	chromosome	1880	14	0.8316
GO:0003729	mRNA binding	596	14	0.8316
GO:0051082	unfolded protein binding	124	14	0.8316
GO:0006796	phosphate-containing compound metabolic p	2806	56	0.8330
GO:0071704	organic substance metabolic process	11974	153	0.8402
GO:0031981	nuclear lumen	4964	60	0.8416
GO:0036094	small molecule binding	2482	60	0.8416
GO:0043168	anion binding	2388	60	0.8416
GO:0002449	lymphocyte mediated immunity	367	10	0.8440
GO:0007167	enzyme-linked receptor protein signaling pat	1010	10	0.8440
GO:0008610	lipid biosynthetic process	735	10	0.8440
GO:0009141	nucleoside triphosphate metabolic process	267	10	0.8440
GO:0009144	purine nucleoside triphosphate metabolic pro	247	10	0.8440
GO:0009199	ribonucleoside triphosphate metabolic proce	249	10	0.8440
GO:0009205	purine ribonucleoside triphosphate metaboli	242	10	0.8440
GO:0016311	dephosphorylation	357	10	0.8440
GO:0032535	regulation of cellular component size	367	10	0.8440
GO:0032640	tumor necrosis factor production	184	10	0.8440
GO:0032680	regulation of tumor necrosis factor productio	184	10	0.8440
GO:0042176	regulation of protein catabolic process	361	10	0.8440
GO:0042326	negative regulation of phosphorylation	381	10	0.8440
GO:0044283	small molecule biosynthetic process	579	10	0.8440

GO:0045860	positive regulation of protein kinase activity	420	10	0.8440
GO:0071706	tumor necrosis factor superfamily cytokine p	189	10	0.8440
GO:1902532	negative regulation of intracellular signal trar	554	10	0.8440
GO:1903555	regulation of tumor necrosis factor superfam	189	10	0.8440
GO:1903706	regulation of hemopoiesis	413	10	0.8440
GO:2000116	regulation of cysteine-type endopeptidase ac	235	10	0.8440
GO:2001242	regulation of intrinsic apoptotic signaling pat	172	10	0.8440
GO:0015075	ion transmembrane transporter activity	855	10	0.8440
GO:0022857	transmembrane transporter activity	1118	10	0.8440
GO:0051172	negative regulation of nitrogen compound m	2490	38	0.8454
GO:0005737	cytoplasm	12069	201	0.8475
GO:0031974	membrane-enclosed lumen	6090	96	0.8524
GO:0043233	organelle lumen	6090	96	0.8524
GO:0070013	intracellular organelle lumen	6090	96	0.8524
GO:0006793	phosphorus metabolic process	2832	57	0.8558
GO:0003723	RNA binding	1971	68	0.8583
GO:0051129	negative regulation of cellular component or	717	23	0.8595
GO:0005739	mitochondrion	1660	23	0.8595
GO:0001816	cytokine production	839	27	0.8601
GO:0001817	regulation of cytokine production	826	27	0.8601
GO:0005524	ATP binding	1472	27	0.8601
GO:0032559	adenyl ribonucleotide binding	1536	27	0.8601
GO:0006807	nitrogen compound metabolic process	10310	134	0.8602
GO:0000003	reproduction	1500	19	0.8615
GO:0022414	reproductive process	1491	19	0.8615
GO:0030234	enzyme regulator activity	1253	19	0.8615
GO:0044249	cellular biosynthetic process	5807	79	0.8637
GO:1901576	organic substance biosynthetic process	5877	79	0.8637
GO:0003824	catalytic activity	5488	79	0.8637
GO:0032991	protein-containing complex	6153	107	0.8640
GO:0033554	cellular response to stress	1979	35	0.8655
GO:0044238	primary metabolic process	10825	141	0.8664
GO:0051052	regulation of DNA metabolic process	540	15	0.8678
GO:0000166	nucleotide binding	2140	54	0.8698
GO:0032553	ribonucleotide binding	1900	43	0.8740
GO:0032555	purine ribonucleotide binding	1883	43	0.8740
GO:0001818	negative regulation of cytokine production	371	6	0.8776
GO:0002366	leukocyte activation involved in immune resp	293	6	0.8776
GO:0002705	positive regulation of leukocyte mediated im	140	6	0.8776
GO:0002706	regulation of lymphocyte mediated immunity	179	6	0.8776
GO:0002708	positive regulation of lymphocyte mediated i	119	6	0.8776
GO:0002819	regulation of adaptive immune response	198	6	0.8776
GO:0002821	positive regulation of adaptive immune respo	120	6	0.8776
GO:0002822	regulation of adaptive immune response bas	182	6	0.8776
GO:0002824	positive regulation of adaptive immune respo	114	6	0.8776
GO:0006839	mitochondrial transport	189	6	0.8776
GO:0006974	cellular response to DNA damage stimulus	879	6	0.8776

GO:0006986	response to unfolded protein	141	6	0.8776
GO:0009266	response to temperature stimulus	186	6	0.8776
GO:0031348	negative regulation of defense response	272	6	0.8776
GO:0031960	response to corticosteroid	165	6	0.8776
GO:0032944	regulation of mononuclear cell proliferation	243	6	0.8776
GO:0034101	erythrocyte homeostasis	137	6	0.8776
GO:0034763	negative regulation of transmembrane transp	154	6	0.8776
GO:0034976	response to endoplasmic reticulum stress	261	6	0.8776
GO:0035966	response to topologically incorrect protein	163	6	0.8776
GO:0042026	protein refolding	27	6	0.8776
GO:0043277	apoptotic cell clearance	51	6	0.8776
GO:0045732	positive regulation of protein catabolic proce	209	6	0.8776
GO:0045787	positive regulation of cell cycle	362	6	0.8776
GO:0045861	negative regulation of proteolysis	352	6	0.8776
GO:0048041	focal adhesion assembly	85	6	0.8776
GO:0048193	Golgi vesicle transport	298	6	0.8776
GO:0050670	regulation of lymphocyte proliferation	239	6	0.8776
GO:0050764	regulation of phagocytosis	103	6	0.8776
GO:0050766	positive regulation of phagocytosis	74	6	0.8776
GO:0051168	nuclear export	165	6	0.8776
GO:0051495	positive regulation of cytoskeleton organizati	195	6	0.8776
GO:0051893	regulation of focal adhesion assembly	64	6	0.8776
GO:0070585	protein localization to mitochondrion	128	6	0.8776
GO:0070848	response to growth factor	742	6	0.8776
GO:0070997	neuron death	368	6	0.8776
GO:0071363	cellular response to growth factor stimulus	712	6	0.8776
GO:0071383	cellular response to steroid hormone stimulu	209	6	0.8776
GO:0072655	establishment of protein localization to mito	123	6	0.8776
GO:0090109	regulation of cell-substrate junction assembly	64	6	0.8776
GO:0150116	regulation of cell-substrate junction organiza	69	6	0.8776
GO:1902905	positive regulation of supramolecular fiber o	176	6	0.8776
GO:1903169	regulation of calcium ion transmembrane tra	188	6	0.8776
GO:2001244	positive regulation of intrinsic apoptotic sign	63	6	0.8776
GO:0001931	uropod	13	6	0.8776
GO:0014069	postsynaptic density	321	6	0.8776
GO:0019867	outer membrane	237	6	0.8776
GO:0030670	phagocytic vesicle membrane	77	6	0.8776
GO:0030864	cortical actin cytoskeleton	80	6	0.8776
GO:0031093	platelet alpha granule lumen	67	6	0.8776
GO:0031254	cell trailing edge	13	6	0.8776
GO:0031968	organelle outer membrane	235	6	0.8776
GO:0032279	asymmetric synapse	327	6	0.8776
GO:0003697	single-stranded DNA binding	124	6	0.8776
GO:0003712	transcription coregulator activity	510	6	0.8776
GO:0044237	cellular metabolic process	10271	135	0.8789
GO:0002443	leukocyte mediated immunity	463	11	0.8822
GO:0008285	negative regulation of cell population prolife	790	11	0.8822

GO:0033044	regulation of chromosome organization	252	11	0.8822
GO:0043484	regulation of RNA splicing	180	11	0.8822
GO:0044255	cellular lipid metabolic process	1013	11	0.8822
GO:0090066	regulation of anatomical structure size	509	11	0.8822
GO:2000278	regulation of DNA biosynthetic process	121	11	0.8822
GO:0016604	nuclear body	849	11	0.8822
GO:0005215	transporter activity	1225	11	0.8822
GO:0099080	supramolecular complex	1390	51	0.8838
GO:1902494	catalytic complex	1653	28	0.8851
GO:0030554	adenyl nucleotide binding	1548	28	0.8851
GO:0043226	organelle	14728	208	0.8853
GO:0009968	negative regulation of signal transduction	1319	24	0.8860
GO:0010629	negative regulation of gene expression	1551	24	0.8860
GO:0097190	apoptotic signaling pathway	600	20	0.8895
GO:0005200	structural constituent of cytoskeleton	107	20	0.8895
GO:0002682	regulation of immune system process	1525	40	0.8906
GO:0017076	purine nucleotide binding	1897	44	0.8939
GO:0031400	negative regulation of protein modification p	519	16	0.8970
GO:0043588	skin development	302	16	0.8970
GO:0051276	chromosome organization	609	16	0.8970
GO:1901135	carbohydrate derivative metabolic process	1111	16	0.8970
GO:0005882	intermediate filament	215	16	0.8970
GO:0031625	ubiquitin protein ligase binding	299	16	0.8970
GO:0044389	ubiquitin-like protein ligase binding	318	16	0.8970
GO:1901363	heterocyclic compound binding	6259	109	0.8978
GO:0009058	biosynthetic process	5962	81	0.8983
GO:0044281	small molecule metabolic process	1841	29	0.9064
GO:0000723	telomere maintenance	154	12	0.9117
GO:0002253	activation of immune response	397	12	0.9117
GO:0002683	negative regulation of immune system proce	449	12	0.9117
GO:0006163	purine nucleotide metabolic process	476	12	0.9117
GO:0006887	exocytosis	369	12	0.9117
GO:0009150	purine ribonucleotide metabolic process	447	12	0.9117
GO:0009259	ribonucleotide metabolic process	466	12	0.9117
GO:0010833	telomere maintenance via telomere lengther	81	12	0.9117
GO:0032200	telomere organization	185	12	0.9117
GO:0071897	DNA biosynthetic process	198	12	0.9117
GO:0072521	purine-containing compound metabolic proc	507	12	0.9117
GO:0005681	spliceosomal complex	195	12	0.9117
GO:0097159	organic cyclic compound binding	6330	110	0.9122
GO:0060627	regulation of vesicle-mediated transport	544	21	0.9125
GO:0005768	endosome	1031	21	0.9125
GO:0016787	hydrolase activity	2344	49	0.9141
GO:0002262	myeloid cell homeostasis	169	7	0.9143
GO:0002263	cell activation involved in immune response	297	7	0.9143
GO:0002429	immune response-activating cell surface rece	312	7	0.9143
GO:0002703	regulation of leukocyte mediated immunity	241	7	0.9143

GO:0002757	immune response-activating signal transduct	312	7	0.9143
GO:0002768	immune response-regulating cell surface rece	341	7	0.9143
GO:0007169	transmembrane receptor protein tyrosine kir	642	7	0.9143
GO:0009314	response to radiation	452	7	0.9143
GO:0017148	negative regulation of translation	258	7	0.9143
GO:0030336	negative regulation of cell migration	368	7	0.9143
GO:0030705	cytoskeleton-dependent intracellular transpo	208	7	0.9143
GO:0030900	forebrain development	383	7	0.9143
GO:0040013	negative regulation of locomotion	419	7	0.9143
GO:0070663	regulation of leukocyte proliferation	269	7	0.9143
GO:0071214	cellular response to abiotic stimulus	335	7	0.9143
GO:0071900	regulation of protein serine/threonine kinase	395	7	0.9143
GO:0104004	cellular response to environmental stimulus	335	7	0.9143
GO:1901654	response to ketone	205	7	0.9143
GO:2000113	negative regulation of cellular macromolecul	283	7	0.9143
GO:2000146	negative regulation of cell motility	383	7	0.9143
GO:2001235	positive regulation of apoptotic signaling patl	135	7	0.9143
GO:0005844	polysome	66	7	0.9143
GO:0031965	nuclear membrane	305	7	0.9143
GO:0099572	postsynaptic specialization	342	7	0.9143
GO:0140110	transcription regulator activity	1939	7	0.9143
GO:0043228	non-membrane-bounded organelle	5701	107	0.9182
GO:0043232	intracellular non-membrane-bounded organe	5700	107	0.9182
GO:0097435	supramolecular fiber organization	815	34	0.9234
GO:0051248	negative regulation of protein metabolic proc	1141	30	0.9243
GO:0071345	cellular response to cytokine stimulus	838	26	0.9267
GO:0006259	DNA metabolic process	1054	18	0.9390
GO:0010638	positive regulation of organelle organization	508	18	0.9390
GO:0034248	regulation of cellular amide metabolic proces	551	18	0.9390
GO:0045111	intermediate filament cytoskeleton	253	18	0.9390
GO:0003677	DNA binding	2522	18	0.9390
GO:0010639	negative regulation of organelle organization	366	8	0.9401
GO:0017157	regulation of exocytosis	213	8	0.9401
GO:0019932	second-messenger-mediated signaling	314	8	0.9401
GO:0022618	ribonucleoprotein complex assembly	234	8	0.9401
GO:0031330	negative regulation of cellular catabolic proce	247	8	0.9401
GO:0032206	positive regulation of telomere maintenance	67	8	0.9401
GO:0032271	regulation of protein polymerization	201	8	0.9401
GO:0035036	sperm-egg recognition	50	8	0.9401
GO:0048024	regulation of mRNA splicing, via spliceosome	109	8	0.9401
GO:0071826	ribonucleoprotein complex subunit organizat	242	8	0.9401
GO:0072676	lymphocyte migration	124	8	0.9401
GO:0072678	T cell migration	73	8	0.9401
GO:0097305	response to alcohol	253	8	0.9401
GO:1901615	organic hydroxy compound metabolic proces	555	8	0.9401
GO:1903312	negative regulation of mRNA metabolic proce	94	8	0.9401
GO:1904358	positive regulation of telomere maintenance	37	8	0.9401

GO:2000573	positive regulation of DNA biosynthetic proce	74	8	0.9401
GO:2001252	positive regulation of chromosome organizat	106	8	0.9401
GO:0019897	extrinsic component of plasma membrane	172	8	0.9401
GO:0019898	extrinsic component of membrane	316	8	0.9401
GO:0034641	cellular nitrogen compound metabolic proces	7053	95	0.9407
GO:0034097	response to cytokine	933	27	0.9418
GO:0005622	intracellular anatomical structure	15464	210	0.9425
GO:0043229	intracellular organelle	13770	193	0.9425
GO:0009628	response to abiotic stimulus	1148	23	0.9464
GO:0031967	organelle envelope	1255	23	0.9464
GO:0031975	envelope	1255	23	0.9464
GO:0019693	ribose phosphate metabolic process	475	14	0.9514
GO:0097193	intrinsic apoptotic signaling pathway	298	14	0.9514
GO:0005813	centrosome	623	14	0.9514
GO:0050778	positive regulation of immune response	607	19	0.9535
GO:0140535	intracellular protein-containing complex	892	19	0.9535
GO:0140657	ATP-dependent activity	552	19	0.9535
GO:0002684	positive regulation of immune system proces	971	28	0.9541
GO:0050776	regulation of immune response	922	28	0.9541
GO:0002250	adaptive immune response	744	9	0.9582
GO:0002460	adaptive immune response based on somatic	375	9	0.9582
GO:0009410	response to xenobiotic stimulus	432	9	0.9582
GO:0009615	response to virus	409	9	0.9582
GO:0009988	cell-cell recognition	71	9	0.9582
GO:0010038	response to metal ion	360	9	0.9582
GO:0050684	regulation of mRNA processing	139	9	0.9582
GO:0051258	protein polymerization	281	9	0.9582
GO:0051924	regulation of calcium ion transport	268	9	0.9582
GO:0009925	basal plasma membrane	254	9	0.9582
GO:0016323	basolateral plasma membrane	229	9	0.9582
GO:0045178	basal part of cell	272	9	0.9582
GO:0003727	single-stranded RNA binding	89	9	0.9582
GO:0043565	sequence-specific DNA binding	1689	9	0.9582
GO:0006996	organelle organization	3556	87	0.9633
GO:0006753	nucleoside phosphate metabolic process	605	15	0.9642
GO:0009117	nucleotide metabolic process	597	15	0.9642
GO:0065003	protein-containing complex assembly	1650	42	0.9672
GO:0001649	osteoblast differentiation	246	10	0.9709
GO:0002687	positive regulation of leukocyte migration	148	10	0.9709
GO:0007005	mitochondrion organization	542	10	0.9709
GO:0022613	ribonucleoprotein complex biogenesis	489	10	0.9709
GO:0031424	keratinization	84	10	0.9709
GO:0032204	regulation of telomere maintenance	101	10	0.9709
GO:1904356	regulation of telomere maintenance via telor	61	10	0.9709
GO:0005769	early endosome	414	10	0.9709
GO:0031966	mitochondrial membrane	744	10	0.9709
GO:0016462	pyrophosphatase activity	737	30	0.9721

GO:0016817	hydrolase activity, acting on acid anhydrides	738	30	0.9721
GO:0016818	hydrolase activity, acting on acid anhydrides,	738	30	0.9721
GO:0017111	ribonucleoside triphosphate phosphatase act	677	30	0.9721
GO:0043231	intracellular membrane-bounded organelle	12663	175	0.9734
GO:0005198	structural molecule activity	819	43	0.9738
GO:0006417	regulation of translation	486	16	0.9738
GO:0031329	regulation of cellular catabolic process	798	16	0.9738
GO:0055086	nucleobase-containing small molecule metab	673	16	0.9738
GO:2000112	regulation of cellular macromolecule biosynt	574	16	0.9738
GO:0005815	microtubule organizing center	832	16	0.9738
GO:0033365	protein localization to organelle	917	31	0.9784
GO:0006278	RNA-templated DNA biosynthetic process	75	11	0.9798
GO:0006403	RNA localization	202	11	0.9798
GO:0007004	telomere maintenance via telomerase	69	11	0.9798
GO:0009896	positive regulation of catabolic process	528	11	0.9798
GO:0022411	cellular component disassembly	483	11	0.9798
GO:1901361	organic cyclic compound catabolic process	517	11	0.9798
GO:0005740	mitochondrial envelope	793	11	0.9798
GO:0005840	ribosome	232	11	0.9798
GO:0045095	keratin filament	101	11	0.9798
GO:0044183	protein folding chaperone	68	11	0.9798
GO:0140662	ATP-dependent protein folding chaperone	41	11	0.9798
GO:0000375	RNA splicing, via transesterification reactions	333	17	0.9810
GO:0000377	RNA splicing, via transesterification reactions	329	17	0.9810
GO:0000398	mRNA splicing, via spliceosome	329	17	0.9810
GO:0006397	mRNA processing	507	17	0.9810
GO:0008380	RNA splicing	469	17	0.9810
GO:0008544	epidermis development	362	17	0.9810
GO:0044265	cellular macromolecule catabolic process	1029	17	0.9810
GO:0099513	polymeric cytoskeletal fiber	789	32	0.9834
GO:1990904	ribonucleoprotein complex	1152	32	0.9834
GO:0043933	protein-containing complex organization	1845	45	0.9837
GO:0001503	ossification	429	12	0.9860
GO:0030216	keratinocyte differentiation	170	12	0.9860
GO:0010608	post-transcriptional regulation of gene expre	1080	18	0.9862
GO:0044260	cellular macromolecule metabolic process	3266	50	0.9867
GO:0006396	RNA processing	1460	19	0.9901
GO:0002181	cytoplasmic translation	161	13	0.9903
GO:0009913	epidermal cell differentiation	235	13	0.9903
GO:0034250	positive regulation of cellular amide metabol	170	13	0.9903
GO:0045109	intermediate filament organization	69	13	0.9903
GO:0045727	positive regulation of translation	143	13	0.9903
GO:0005730	nucleolus	1463	13	0.9903
GO:0005874	microtubule	466	13	0.9903
GO:0010628	positive regulation of gene expression	1177	43	0.9907
GO:0006518	peptide metabolic process	966	35	0.9927
GO:0043603	cellular amide metabolic process	1242	35	0.9927

GO:0019637	organophosphate metabolic process	1034	20	0.9929
GO:0045103	intermediate filament-based process	90	14	0.9933
GO:0045104	intermediate filament cytoskeleton organizat	89	14	0.9933
GO:1903311	regulation of mRNA metabolic process	309	14	0.9933
GO:0016887	ATP hydrolysis activity	347	15	0.9954
GO:0016071	mRNA metabolic process	751	22	0.9964
GO:0005634	nucleus	8188	114	0.9980
GO:0006412	translation	770	25	0.9987
GO:0034645	cellular macromolecule biosynthetic process	1231	25	0.9987
GO:0043043	peptide biosynthetic process	801	25	0.9987
GO:0043604	amide biosynthetic process	932	25	0.9987
GO:1901566	organonitrogen compound biosynthetic proc	1817	31	0.9999

FDR	OR	ProteinID
1.0000	7.80	ACTB, ADAM10, CD9, CDC42, DSG1, DSP, F11R, FLNA, FSCN1, JUP, PATJ, PECAM1, RHOA,
1.0000	12.47	ACTB, CD9, DSG1, F11R, FSCN1, JUP, PATJ, PECAM1, RHOA, TLN1, VCL
1.0000	4.86	ACTB, CD9, CDC42, DSG1, F11R, FLOT1, FSCN1, ITGA6, ITGB3, JUP, PATJ, PECAM1, PTPRJ,
1.0000	2.98	ADAM10, ANXA7, ATP5F1B, BCAP31, CALM3, CANX, CCL5, CD151, CD226, CD36, CD9, CD
1.0000	15.90	CD36, CLIC1, FLNA, HSP90AA1, MYH9, MYL12B, MYL6
1.0000	15.90	CD36, CLIC1, FLNA, HSP90AA1, MYH9, MYL12B, MYL6
1.0000	4.58	ADAM10, ANXA7, CD151, CD226, CD9, F11R, FERMT3, ICAM2, ITGA2B, ITGA6, ITGB1, ITG
1.0000	3.80	ACTB, CD9, CLIC1, F11R, FCER1G, FERMT3, FLNA, GNAQ, GP1BA, GP1BB, ILK, ITGB3, MM
1.0000	Inf	CD36, FLNA, HSP90AA1, JUP
1.0000	Inf	CD36, FLNA, HSP90AA1, JUP
1.0000	Inf	CD36, FLNA, HSP90AA1, JUP
1.0000	Inf	HSP90AA1, ITGB1, JUP, YWHAG
1.0000	3.04	ACTB, ADAM10, CD9, CDC42, CFL1, DSG1, DSP, F11R, FLNA, FLOT1, FSCN1, HSPA8, ITGA6
1.0000	2.63	AACS, ARL6IP5, BCAP31, CCL5, CD226, CD36, CDC42, EIF5A, F11R, FLNA, FLOT1, GAPDH,
1.0000	6.21	CDC42, FSCN1, HSP90AA1, ITGB1, MSN, MYH9, PATJ, RAB10, RAP1B, RHOA
1.0000	6.21	ACTB, DHX9, FLNA, JUP, NCL, NPM1, PSMA6, SRI, YWHAH, YWHAZ
1.0000	3.33	ACTB, B2M, CDC42, CFL1, FLNA, FLOT1, HSP90AA1, ITGA6, ITGB1, PPIA, RAB10, RAC2, RH
1.0000	7.90	CDC42, FLNA, FSCN1, HSP90AA1, ITGA6, PFN1, RAC2, S100A9
1.0000	13.03	DSP, FLNA, JUP, MYH9, MYL6, SRI
1.0000	13.03	FLNA, FSCN1, ILK, MYH9, MYL12B, TPM4
1.0000	4.05	ACTB, CD9, CLIC1, F11R, FERMT3, FLNA, GP1BA, ILK, ITGB3, MMRN1, MYH9, PLEK, PPIA,
1.0000	2.48	AACS, ANXA2, ARL6IP5, BCAP31, CCL5, CD226, CD36, CDC42, EIF5A, F11R, FLNA, FLOT1,
1.0000	3.01	ACTB, B2M, CDC42, CFL1, FLNA, FLOT1, HSP90AA1, ITGA6, ITGB1, PPIA, RAB10, RAC2, RH
1.0000	4.28	ACTB, DHX9, ENO1, FLNA, HNRNPF, HNRNPU, JUP, NCL, NPM1, PSMA6, SRI, YWHAH, YW
1.0000	2.77	ACTB, ANXA2, ANXA5, CD36, CD59, CD9, CLIC1, F11R, FCER1G, FERMT3, FLNA, GNAQ, GP
1.0000	2.77	ACTB, ANXA2, ANXA5, CD36, CD59, CD9, CLIC1, F11R, FCER1G, FERMT3, FLNA, GNAQ, GP
1.0000	2.77	ACTB, ANXA2, ANXA5, CD36, CD59, CD9, CLIC1, F11R, FCER1G, FERMT3, FLNA, GNAQ, GP
1.0000	4.63	AACS, BCAP31, FLNA, HSP90AA1, JUP, PPIA, RAC2, SRI, STOM, UBE2L3, YWHAE
1.0000	2.60	ACTB, ANXA2, B2M, CD9, CDC42, CFL1, FLNA, FLOT1, HSP90AA1, ILK, ITGA6, ITGB1, LRP1
1.0000	2.60	ACTB, ADAM10, ANXA2, CD53, CDC42, DSC1, DSG1, DSP, F11R, FLNA, FLOT1, FSCN1, ITG
1.0000	3.00	AACS, ADAM10, CALM3, CCL5, CDC42, FLNA, FLOT1, G3BP1, GNAI2, GNAQ, ILK, ITGB1, JU
1.0000	3.00	ADAM10, ANPEP, CASP14, CCL5, CDC42, CPN1, CTSD, DCD, GAPDH, GSR, HINT1, ILK, ITG
1.0000	2.47	ACTB, ANXA2, ANXA5, CD151, CD36, CD59, CD9, CLIC1, DSP, F11R, FCER1G, FERMT3, FLN
1.0000	3.45	ACTB, ANK1, ARPC5, CDC42, FSCN1, HSP90AA1, ILK, ITGB1, MSN, MYH9, PATJ, RAB10, RA
1.0000	5.23	CAMP, GAPDH, H2BC21, HLA-A, KRT6A, LTF, LYZ, PF4V1, S100A9
1.0000	5.23	B2M, FLNA, GP1BA, GP1BB, ITGA2B, ITGA6, ITGB1, ITGB3, TFRC
1.0000	5.23	ANPEP, CD36, CLTA, HLA-A, HLA-B, LBP, LRP1, PPIA, RPLP0
1.0000	2.14	ACTB, ADAM10, ARPC5, ATP5F1B, CCL5, CD151, CD47, CD9, CD99, CDC42, F11R, FCER1G
1.0000	3.00	ACTB, CCL5, CD9, CD99, CLIC1, DSP, F11R, FERMT3, FLNA, GP1BA, ILK, ITGB3, JUP, MMR
1.0000	3.00	ACTB, B2M, CDC42, CFL1, FLNA, FLOT1, HSP90AA1, ITGA6, ITGB1, RAB10, RAC2, RHOA, R
1.0000	3.00	ADAM10, ANXA11, B2M, CAMP, CD36, CD47, CD53, CD59, CTSD, CYBB, JUP, LTF, LYZ, PT
1.0000	3.54	ADAM10, CD47, CDC42, FERMT3, FLNA, ILK, ITGA2B, ITGA6, ITGB1, ITGB3, MYH9, PLEK,
1.0000	3.54	AACS, BCAP31, CCL5, CD36, FLNA, HSP90AA1, HSPA8, JUP, PPIA, RAC2, SRI, STOM, UBE2

1.0000	2.42	ADAM10, ANPEP, ANXA2, ATP5F1B, CAMP, CDC42, CYBB, DSP, FLNA, G6PD, GNG5, HNRI
1.0000	6.48	CAMP, CD36, H2BC21, HLA-A, KRT6A, LBP, LYZ
1.0000	Inf	CD36, LBP, PRDX2
1.0000	Inf	FLNA, GP1BA, GP1BB
1.0000	Inf	FLNA, ITGA6, S100A9
1.0000	Inf	FLNA, GP1BA, GP1BB
1.0000	Inf	FLNA, GP1BA, GP1BB
1.0000	Inf	FLNA, HSP90AA1, JUP
1.0000	Inf	FLNA, PRDX2, SRI
1.0000	Inf	CDC42, FLNA, RTN4
1.0000	Inf	AACS, PPIA, SRI
1.0000	Inf	FLNA, SRI, YWHAB
1.0000	Inf	CDC42, FLNA, RTN4
1.0000	Inf	FLNA, GP1BA, GP1BB
1.0000	Inf	FLNA, GP1BA, GP1BB
1.0000	Inf	F11R, PATJ, PECAM1
1.0000	Inf	F11R, PATJ, PECAM1
1.0000	Inf	MYH9, MYL12B, MYL6
1.0000	Inf	ACTB, F11R, PATJ
1.0000	Inf	FLNA, GP1BA, GP1BB
1.0000	Inf	FLNA, GP1BA, GP1BB
1.0000	2.32	ACTB, ANXA2, ANXA5, CD151, CD36, CD59, CD9, CLIC1, DSP, F11R, FCER1G, FERMT3, FLN
1.0000	2.71	BCAP31, CD36, CLTA, FLNA, HSP90AA1, HSPA8, HSPA9, HSPD1, JUP, M6PR, NPM1, RAB1I
1.0000	2.71	ANPEP, ANXA2, ATP5F1B, CAMP, CDC42, CYBB, FLNA, ILK, ITGA2B, ITGB1, ITGB3, JUP, KR
1.0000	2.24	ARL6IP5, BCAP31, CCL5, CD226, CD36, CDC42, EIF5A, F11R, FLNA, FLOT1, GAPDH, GNAI2
1.0000	2.18	ACTB, ANXA2, B2M, CDC42, CFL1, F11R, FERMT3, FLNA, FLOT1, G6PD, GP1BA, GP1BB, HI
1.0000	10.26	ACTB, F11R, ITGB1, PECAM1, VCL
1.0000	10.26	DSP, FLNA, JUP, MYL6, SRI
1.0000	10.26	CD36, FLNA, ITGB1, PRDX2, THBS1
1.0000	10.26	FSCN1, ILK, MYH9, MYL12B, TPM4
1.0000	10.26	FLNA, JUP, MYL12B, SLC2A1, SRI
1.0000	10.26	CD36, CD9, ITGA2B, ITGB3, PECAM1
1.0000	10.26	FSCN1, ILK, MYH9, MYL12B, TPM4
1.0000	10.26	ACTB, F11R, JUP, PATJ, RHOA
1.0000	10.26	FSCN1, ILK, MYH9, MYL12B, TPM4
1.0000	2.08	AACS, ACTB, ARL6IP5, BCAP31, CANX, CCL5, CD36, CD47, CLTA, DHX9, FLNA, FLOT1, HNR
1.0000	2.51	ACTB, ALDOA, CDC42, CFL1, F11R, FERMT3, FLNA, FLOT1, GP1BA, HSP90AA1, ILK, ITGB1,
1.0000	2.51	ALDOA, CAMP, CDC42, CFL1, CYBB, F11R, FLNA, ILK, ITGB1, ITGB3, JUP, KRT1, MSN, MYA
1.0000	2.51	ACTB, ARPC5, B2M, CDC42, CFL1, FLNA, FLOT1, FSCN1, HSP90AA1, ILK, ITGA6, ITGB1, MY
1.0000	2.51	ADAM10, CCL5, CD47, CD9, CD99, CDC42, F11R, FCER1G, GP1BA, ITGA6, ITGB1, ITGB3, L
1.0000	3.68	CALM3, CD36, FLNA, GP1BA, GP1BB, HNRNPU, ITGB3, S100A8, S100A9, SRGN, SRI, YWH/
1.0000	3.68	B2M, CD36, FLNA, GP1BA, GP1BB, HSPD1, ITGA2B, ITGA6, ITGB1, ITGB3, LRP1, TFRC
1.0000	1.90	AACS, ADAM10, ARL6IP5, BCAP31, CALM3, CCL5, CD226, CD36, CD47, CDC42, EIF5A, EN(
1.0000	1.90	ACTB, ADAM10, ANXA2, ANXA5, ARPC5, B2M, CD151, CD53, CD59, CD9, CD99, CDC42, C
1.0000	1.93	ACTB, ADAM10, ADH5, ALDOA, ANPEP, ANXA11, ANXA2, ANXA5, ANXA7, ARPC5, ATIC, A
1.0000	2.68	ANPEP, ANXA2, ATP5F1B, CAMP, CYBB, FLNA, ITGA2B, ITGB1, ITGB3, JUP, KRT1, LRP1, M
1.0000	2.18	ACTB, ANXA2, CD36, DHX9, EIF5A, ENO1, FLNA, FLOT1, HINT1, HNRNPA2B1, HNRNPU, H!

1.0000	2.18	ACTB, ANXA2, CD36, DHX9, EIF5A, ENO1, FLNA, FLOT1, HINT1, HNRNPA2B1, HNRNPU, H
1.0000	2.18	ACTB, ANXA2, CD36, DHX9, EIF5A, ENO1, FLNA, FLOT1, HINT1, HNRNPA2B1, HNRNPU, H
1.0000	2.18	ACTB, ANXA2, CD36, DHX9, EIF5A, ENO1, FLNA, FLOT1, HINT1, HNRNPA2B1, HNRNPU, H
1.0000	2.18	ACTB, ANXA2, CD36, DHX9, EIF5A, ENO1, FLNA, FLOT1, HINT1, HNRNPA2B1, HNRNPU, H
1.0000	2.18	ACTB, ANXA2, CD36, DHX9, EIF5A, ENO1, FLNA, FLOT1, HINT1, HNRNPA2B1, HNRNPU, H
1.0000	2.18	BCAP31, CALM3, CANX, CD151, CD226, CD36, CD47, CD53, CD9, CD99, CYBB, FCER1G, FL
1.0000	1.94	AACS, ACTB, ANXA2, ARL6IP5, BCAP31, CANX, CCL5, CD36, CD47, CLTA, DHX9, FLNA, FLO
1.0000	2.12	AACS, ARL6IP5, BCAP31, CANX, CCL5, CD36, CLTA, FLNA, HSP90AA1, HSPA8, HSPA9, HSP
1.0000	1.99	ADAM10, ARPC5, ATP5F1B, CCL5, CD151, CD47, CD9, CD99, CDC42, F11R, FCER1G, FERM
1.0000	2.03	ACTB, ANXA2, B2M, CCL5, CD47, CD9, CD99, CLIC1, DSC1, DSG1, DSP, F11R, FERMT3, FLN
1.0000	3.01	B2M, CDC42, CFL1, FLNA, FSCN1, HSP90AA1, ITGA6, PFN1, RAC2, RCC2, RHOA, RTN4, S10
1.0000	3.01	CD36, DHX9, FLNA, FLOT1, HSPA1B, JUP, LTF, NPM1, PPIA, PRDX2, PSMA6, S100A8, S100
1.0000	3.01	B2M, CDC42, CFL1, FLNA, FSCN1, HSP90AA1, ITGA6, PFN1, RAC2, RCC2, RHOA, RTN4, S10
1.0000	3.90	ANXA2, CD36, CD9, FLNA, GP1BA, ITGB1, KRT1, MMRN1, PRDX2, THBS1
1.0000	3.90	CD36, CDC42, DSG1, HSP90AA1, ITGB3, MSN, MYL12B, PATJ, RAB27B, SLC2A1
1.0000	3.90	ANPEP, CD36, CLTA, HLA-A, HLA-B, LBP, LRP1, PPIA, RPLP0, VDAC2
1.0000	2.32	ADAM10, ANPEP, ANXA7, CD36, CD47, CD53, CD59, CD9, CYBB, DSC1, DSG1, DSP, FCER1
1.0000	1.85	ACTB, ADAM10, ANXA2, ATP5F1B, B2M, CCL5, CD151, CD226, CD36, CD47, CD9, CD99, C
1.0000	2.12	ALDOA, ANXA2, ATIC, CCT8, CLIC1, EEF2, ENO1, F11R, FLNA, FSCN1, HSPA8, ITGA6, ITGB1
1.0000	2.65	ANPEP, ANXA2, ATP5F1B, CAMP, CYBB, FLNA, ITGA2B, ITGB1, ITGB3, JUP, KRT1, MYH9, N
1.0000	2.65	ACTB, B2M, CDC42, CFL1, FLNA, FLOT1, HSP90AA1, ITGA6, ITGB1, RAB10, RAC2, RHOA, R
1.0000	2.65	CD36, CDC42, FERMT3, FLNA, ILK, ITGA2B, ITGA6, ITGB1, ITGB3, JUP, MYADM, PTPRJ, RA
1.0000	2.65	ANXA11, ANXA2, CANX, CCT4, CTSD, FLOT1, HSP90AA1, HSPA8, ITGB1, ITGB3, PRDX1, RA
1.0000	2.65	ANXA11, ANXA2, CANX, CCT4, CTSD, FLOT1, HSP90AA1, HSPA8, ITGB1, ITGB3, PRDX1, RA
1.0000	1.72	AACS, ADAM10, ANK1, ANPEP, ANXA2, ANXA5, ARL6IP5, ATP2A3, BCAP31, CALM3, CCL5
1.0000	2.06	ACTB, CALM3, CAMP, CCL5, CD36, CD9, CPN1, CYBB, DSG1, EEF1A1, FCER1G, FLNA, GNA
1.0000	2.06	BCAP31, CALM3, CANX, CD151, CD226, CD36, CD47, CD53, CD59, CD9, CD99, CYBB, FCEF
1.0000	1.91	ADAM10, CCL5, CD226, CD36, CD47, CD59, CDC42, EIF5A, FCER1G, FCN1, FERMT3, FLNA
1.0000	1.91	ADAM10, ALDOA, ANXA2, ANXA7, ATIC, CCT8, CD151, CD226, CD47, CD9, CLIC1, DSP, EE
1.0000	1.93	ACTB, ADAM10, B2M, CAMP, CCL5, CD151, CD226, CD47, CD9, CLIC1, F11R, FCER1G, FEF
1.0000	1.71	ADAM10, ANK1, ANPEP, ANXA5, ARL6IP5, ATP2A3, BCAP31, CALM3, CCL5, CD226, CD36,
1.0000	2.42	ADAM10, ANPEP, ANXA2, ATP5F1B, CAMP, CYBB, FLNA, ITGA2B, ITGB1, ITGB3, JUP, KRT1
1.0000	2.42	ACTB, ADH5, CALM3, CCL5, CD36, GAPDH, HINT1, HMBS, HSP90AA1, ILK, ITGB3, KANSL3,
1.0000	2.42	ANPEP, ANXA2, ATP5F1B, CAMP, CYBB, FLNA, ILK, ITGA2B, ITGB1, ITGB3, JUP, KRT1, LRP1
1.0000	4.29	CDC42, F11R, FLNA, ILK, ITGB1, ITGB3, MYADM, XRCC5
1.0000	4.29	CD36, CDC42, FLNA, ILK, ITGB3, JUP, MYADM, PTPRJ
1.0000	4.29	ACTB, CALM3, ITGB1, SRI, STOM, UBQLN1, YWHAE, YWHAH
1.0000	4.29	ACTB, CALM3, ITGB1, SRI, STOM, UBQLN1, YWHAE, YWHAH
1.0000	4.29	B2M, CAMP, CTSD, JUP, LTF, LYZ, QSOX1, VCL
1.0000	4.29	CANX, CDC42, CYBB, FLNA, G3BP1, HSP90AA1, SLC2A3, SOD1
1.0000	3.05	ANXA7, CTSD, EEF1A1, GAPDH, HSP90AA1, HSPA8, LRBA, QSOX1, RAB1B, RAB33B, S100A
1.0000	3.05	ACTB, CD9, CDC42, CFL1, FLOT1, HSP90AA1, ITGB1, RAB10, RAC2, RHOA, RTN4, VCL, YW
1.0000	3.05	ANXA7, CTSD, EEF1A1, GAPDH, HSP90AA1, HSPA8, LRBA, QSOX1, RAB1B, RAB33B, S100A
1.0000	1.77	AACS, ADAM10, ANXA2, ARL6IP5, ATP2A3, BCAP31, CALM3, CCL5, CD226, CD36, CD47, C
1.0000	1.84	ACTB, ADAM10, ADH5, ALDOA, ANPEP, ANTXR2, ANXA11, ANXA2, ANXA5, ANXA7, ARPC
1.0000	2.26	ACTB, ARPC5, B2M, CDC42, CFL1, FLNA, FLOT1, FSCN1, HSP90AA1, ITGA6, ITGB1, MYH9,
1.0000	1.79	ACTB, ALDOA, ANPEP, ANXA2, ATP5F1B, CAMP, CD151, CD36, CD53, CD9, CDC42, CFL1,

1.0000	1.68	AACS, ADAM10, ANK1, ANPEP, ANXA5, ARL6IP5, ATP2A3, BCAP31, CALM3, CCL5, CD226,
1.0000	1.72	ACTB, ADAM10, ANXA2, ANXA5, ARPC5, B2M, CALM3, CANX, CD151, CD53, CD59, CD9, (
1.0000	1.69	ACTB, ADAM10, ANK1, ANPEP, ANTXR2, ANXA11, ANXA2, ANXA5, ANXA7, ARL6IP5, ATIC
1.0000	2.15	ACTB, BCAP31, CALM3, CAMP, CANX, CD36, CD9, CYBB, FCER1G, FLNA, GNAI2, HSP90AA
1.0000	2.15	ANPEP, ANXA2, ATP5F1B, CAMP, CD36, CD53, CD9, CYBB, FLNA, FLOT1, GNG5, ITGA2B, I
1.0000	2.06	ACTB, ADH5, CAMP, CCL5, CD36, CYBB, FLNA, GNAI2, GNAQ, GSTP1, ITGB3, JUP, LBP, LRI
1.0000	1.83	ACTB, ANK1, ANXA2, ATP2A3, BCAP31, CALM3, CANX, CD36, CDC42, CFL1, CLTA, DHX9, I
1.0000	1.67	ADAM10, ALDOA, ANPEP, ANTXR2, ANXA11, ANXA2, ANXA7, ARPC5, B2M, BCAP31, CALI
1.0000	1.67	ADAM10, ALDOA, ANPEP, ANTXR2, ANXA11, ANXA2, ANXA7, ARPC5, B2M, BCAP31, CALI
1.0000	5.10	CDC42, DSP, FLNA, GNG5, ILK, RTN4
1.0000	5.10	CDC42, FSCN1, HSP90AA1, ITGB1, RAC2, VCL
1.0000	5.10	CDC42, F11R, FLNA, MYH9, PLEK, RHOA
1.0000	5.10	CD36, DHX9, FLNA, HSP90AA1, JUP, YWHAE
1.0000	5.10	CALM3, FLNA, GP1BA, GP1BB, ITGB3, SRI
1.0000	5.10	CALM3, FLNA, GP1BA, GP1BB, ITGB3, SRI
1.0000	5.10	CALM3, FLNA, GP1BA, GP1BB, ITGB3, SRI
1.0000	5.10	CALM3, FLNA, GP1BA, GP1BB, ITGB3, SRI
1.0000	5.10	CALM3, DSP, FLNA, HSP90AA1, JUP, SRI
1.0000	5.10	CALM3, FLNA, GP1BA, GP1BB, ITGB3, SRI
1.0000	5.10	ALDOA, FLNA, JUP, MYL12B, SLC2A1, SRI
1.0000	2.37	ANPEP, ANXA2, ATP5F1B, CAMP, CYBB, FLNA, ITGA2B, ITGB1, ITGB3, JUP, KRT1, LRP1, M
1.0000	3.10	CDC42, DSP, FLNA, G6PD, GNG5, HNRNPU, ILK, ITGB1, MATR3, RTN4, SRI
1.0000	1.73	ACTB, ADAM10, ANK1, ANXA11, ANXA7, ARPC5, B2M, BCAP31, CD151, CD226, CD36, CD
1.0000	1.69	ACTB, ADAM10, ADH5, ALDOA, ANPEP, ANXA11, ANXA2, ANXA5, ANXA7, ARPC5, ATIC, A
1.0000	1.69	ACTB, ADAM10, ADH5, ALDOA, ANPEP, ANXA11, ANXA2, ANXA5, ANXA7, ARPC5, ATIC, A
1.0000	1.69	ACTB, ADAM10, ADH5, ALDOA, ANPEP, ANXA11, ANXA2, ANXA5, ANXA7, ARPC5, ATIC, A
1.0000	1.69	ACTB, ADAM10, ADH5, ALDOA, ANPEP, ANXA11, ANXA2, ANXA5, ANXA7, ARPC5, ATIC, A
1.0000	2.09	ACTB, ALDOA, ARPC5, CD47, CDC42, CFL1, DSP, F11R, FLNA, FSCN1, ITGB1, ITGB3, JUP, LI
1.0000	1.81	ACTB, ADH5, CALM3, CAMP, CCL5, CD36, CYBB, DSG1, FLNA, G6PD, GNAI2, GNAQ, GSTP:
1.0000	1.81	ADAM10, ANPEP, ANTXR2, ANXA2, ANXA7, B2M, CALM3, CD36, CD47, CD53, CD59, CD9,
1.0000	1.81	ADAM10, ANPEP, ANTXR2, ANXA2, ANXA7, B2M, CALM3, CD36, CD47, CD53, CD59, CD9,
1.0000	1.94	ACTB, ANXA2, ANXA5, CD36, CD59, CD9, CLIC1, F11R, FCER1G, FERMT3, FLG2, FLNA, GN/
1.0000	1.94	ARL6IP5, BCAP31, CCL5, CD36, CDC42, EIF5A, F11R, FLNA, GAPDH, GNAI2, HINT1, HSP90/
1.0000	2.59	CD36, FERMT3, ILK, ITGA2B, ITGA6, ITGB1, ITGB3, JUP, PTPRJ, RCC2, RHOA, THBS1, TLN1
1.0000	2.59	CAMP, CD36, DCD, FCER1G, GSDMA, H2BC21, HLA-A, KRT6A, LBP, LTF, LYZ, S100A8, S10(
1.0000	7.57	CD36, PRDX1, PRDX2, SOD1
1.0000	7.57	CD36, PRDX1, PRDX2, SOD1
1.0000	7.57	CD151, FLNA, ITGB1, RHOA
1.0000	7.57	CD36, CD47, CYB5R3, HSP90AA1
1.0000	7.57	CDC42, RAB1B, RAB33B, YWHAZ
1.0000	7.57	DSC1, DSG1, ITGB1, PECAM1
1.0000	7.57	FLNA, GP1BA, GP1BB, PLEK
1.0000	7.57	CDC42, FSCN1, HSP90AA1, RAC2
1.0000	7.57	CDC42, FSCN1, HSP90AA1, RAC2
1.0000	7.57	CD36, PRDX1, PRDX2, SOD1
1.0000	7.57	CD36, PRDX1, PRDX2, SOD1
1.0000	7.57	CD151, FLNA, ITGB1, RHOA

1.0000	7.57	CD36, CD47, CYB5R3, HSP90AA1
1.0000	7.57	HSP90AA1, ITGB1, RTN4, VCL
1.0000	7.57	CDC42, CFL1, ITGB1, YWHAH
1.0000	7.57	AACS, CCL5, PPIA, SRI
1.0000	7.57	CAMP, LBP, LTF, LYZ
1.0000	7.57	CD36, CD9, FCER1G, ITGB1
1.0000	7.57	HSP90AA1, ITGB1, RTN4, VCL
1.0000	7.57	CD36, CD9, FCER1G, ITGB1
1.0000	7.57	CD36, CD9, FCER1G, ITGB1
1.0000	7.57	CD36, PRDX1, PRDX2, SOD1
1.0000	7.57	CD36, PRDX1, PRDX2, SOD1
1.0000	7.57	DSP, FLNA, JUP, SRI
1.0000	7.57	CD36, ITGB1, PRDX2, THBS1
1.0000	7.57	CD151, FLNA, ITGB1, RHOA
1.0000	7.57	CD151, FLNA, ITGB1, RHOA
1.0000	7.57	CDC42, FSCN1, HSP90AA1, RAC2
1.0000	7.57	CDC42, FSCN1, HSP90AA1, RAC2
1.0000	7.57	HSP90AA1, ITGB1, RTN4, VCL
1.0000	7.57	CD36, CD47, CYB5R3, HSP90AA1
1.0000	7.57	ITGA2B, ITGA6, ITGB1, ITGB3
1.0000	7.57	MYH16, MYH9, MYL12B, MYL6
1.0000	7.57	CANX, CDC42, ITGB1, RHOA
1.0000	7.57	CANX, CDC42, ITGB1, RHOA
1.0000	7.57	FLNA, SRI, YWHAE, YWHAH
1.0000	7.57	FLNA, SRI, YWHAE, YWHAH
1.0000	2.31	ANXA2, CAVIN2, CD9, CDC42, FLOT1, MYH9, OSBPL2, RAB10, RAB1B, RAB33B, RTN3, RTN
1.0000	2.31	ACTB, ADAM10, ANK1, ARL6IP5, CALM3, F11R, FLNA, FLOT1, HSP90AA1, ITGB1, JUP, LRP
1.0000	2.31	ACTB, CAMP, CD36, CYBB, FLNA, GNAI2, ITGB3, JUP, LRP1, NCL, PKM, RAB10, RAP1B, RPL
1.0000	2.31	ACTB, ADAM10, ANK1, ARL6IP5, CALM3, F11R, FLNA, FLOT1, HSP90AA1, ITGB1, JUP, LRP
1.0000	2.31	ANXA11, ANXA2, ANXA5, ANXA7, CD151, CTSD, FCN1, GP1BA, HRNR, HSP90AA1, KRT1, L
1.0000	2.31	ANXA11, ANXA2, ANXA5, ANXA7, CD151, CTSD, FCN1, GP1BA, HRNR, HSP90AA1, KRT1, L
1.0000	1.58	AACS, ACTB, ADAM10, ANK1, ANXA2, ARL6IP5, ATP2A3, BCAP31, CALM3, CANX, CCL5, C
1.0000	Inf	ILK, RTN4
1.0000	Inf	FLNA, RTN4
1.0000	Inf	STOM, YWHAH
1.0000	Inf	LBP, PRDX2
1.0000	Inf	LBP, PRDX2
1.0000	Inf	CDC42, ILK
1.0000	Inf	ACTB, KANSL3
1.0000	Inf	ACTB, KANSL3
1.0000	Inf	STOM, YWHAH
1.0000	Inf	CD36, F11R
1.0000	Inf	RTN4, YWHAZ
1.0000	Inf	CD9, RTN4
1.0000	Inf	CD9, ITGB1
1.0000	Inf	ACTB, KANSL3
1.0000	Inf	ACTB, KANSL3

1.0000	Inf	ACTB, KANSL3
1.0000	Inf	ACTB, KANSL3
1.0000	Inf	LBP, LYZ
1.0000	Inf	CD9, ITGB1
1.0000	Inf	CD36, F11R
1.0000	Inf	ILK, JUP
1.0000	Inf	CDC42, YWHAZ
1.0000	Inf	CDC42, YWHAZ
1.0000	Inf	AACS, SRI
1.0000	Inf	GAPDH, S100A9
1.0000	Inf	STOM, YWHAH
1.0000	Inf	PRDX1, PRDX2
1.0000	Inf	ANPEP, CPN1
1.0000	Inf	ACTB, KANSL3
1.0000	Inf	ACTB, KANSL3
1.0000	Inf	CD36, HSP90AA1
1.0000	Inf	ACTB, ITGB1
1.0000	Inf	CDC42, FSCN1
1.0000	Inf	AACS, SRI
1.0000	Inf	YWHAG, YWHAH
1.0000	Inf	CD36, F11R
1.0000	Inf	CDC42, FSCN1
1.0000	Inf	CDC42, FSCN1
1.0000	Inf	CDC42, YWHAZ
1.0000	Inf	CDC42, YWHAZ
1.0000	Inf	CDC42, YWHAZ
1.0000	Inf	CD36, LBP
1.0000	Inf	F11R, PECAM1
1.0000	Inf	CD36, LBP
1.0000	Inf	ILK, PECAM1
1.0000	Inf	ILK, PECAM1
1.0000	Inf	CANX, CLTA
1.0000	Inf	ILK, JUP
1.0000	Inf	AACS, SRI
1.0000	Inf	STOM, YWHAH
1.0000	Inf	CD36, HSP90AA1
1.0000	Inf	CDC42, F11R
1.0000	Inf	STOM, YWHAH
1.0000	Inf	ACTB, KANSL3
1.0000	Inf	F11R, PATJ
1.0000	Inf	DSG1, JUP
1.0000	Inf	ACTB, KANSL3
1.0000	Inf	CD36, HSP90AA1
1.0000	Inf	ITGB1, MYH9
1.0000	Inf	RAP1B, STOM
1.0000	Inf	ANXA7, SRI
1.0000	Inf	ANXA7, SRI

1.0000	Inf	CANX, CLTA
1.0000	Inf	FLNA, HSP90AA1
1.0000	Inf	ACTB, ITGB1
1.0000	Inf	ACTB, KANSL3
1.0000	Inf	ACTB, KANSL3
1.0000	Inf	MYH9, MYL6
1.0000	Inf	MYH9, MYL6
1.0000	Inf	CASP14, CTSD
1.0000	Inf	YWHAG, YWHAH
1.0000	Inf	JUP, MYH9
1.0000	Inf	ACTB, HSP90AA1
1.0000	3.20	CALM3, CD36, DSP, FLNA, GNAQ, JUP, SRI, YWHAE, YWHAH
1.0000	3.20	CD9, F11R, FCER1G, FLNA, GNAQ, GP1BA, GP1BB, MMRN1, PLEK
1.0000	3.20	B2M, CFL1, FLNA, ITGA6, RHOA, RTN4, S100A9, VIM, YWHAH
1.0000	3.20	CDC42, FLNA, FSCN1, HSP90AA1, ITGB1, PFN1, RAC2, RCC2, VCL
1.0000	3.20	BCAP31, CD36, FLNA, HSP90AA1, JUP, RAC2, STOM, UBE2L3, YWHAE
1.0000	3.20	ANXA2, CD36, CD9, GP1BA, ITGB1, KRT1, MMRN1, PRDX2, THBS1
1.0000	3.20	ACTB, FLNA, FLOT1, HSP90AA1, ITGB1, RAB10, RAC2, RTN4, VCL
1.0000	3.20	CDC42, FLNA, FSCN1, HSP90AA1, ITGB1, PFN1, RAC2, RCC2, VCL
1.0000	3.20	ADAM10, ANPEP, CASP14, CPN1, CTSD, DCD, HINT1, LTF, PSMA6
1.0000	3.20	ANK1, ATP2A3, CALM3, FLNA, HSP90AA1, SRI, YWHAE, YWHAH, YWHAZ
1.0000	2.14	ACTB, CDC42, CFL1, FERMT3, FLNA, FLOT1, HSP90AA1, ILK, ITGB1, ITGB3, MYADM, MYH9
1.0000	2.14	ACTB, B2M, CCL5, CD36, CD47, CDC42, F11R, FLNA, FLOT1, GP1BA, HLA-A, HSPD1, ILK, IT
1.0000	1.82	ADH5, ALDOA, ANPEP, ARL6IP5, ATP2A3, B2M, CALM3, CD36, CDC42, CYB5R3, DSP, ENO
1.0000	2.57	CAMP, CD59, FCN1, GAPDH, H2BC21, HLA-A, KRT1, KRT6A, LTF, LYZ, PF4V1, S100A9
1.0000	2.57	ACTB, CDC42, CFL1, FLOT1, HSP90AA1, ITGB1, RAB10, RAC2, RHOA, RTN4, VCL, YWHAH
1.0000	2.57	ACTB, CDC42, CFL1, FLOT1, HSP90AA1, ITGB1, RAB10, RAC2, RTN4, SOD1, VCL, YWHAH
1.0000	2.57	ACTB, CDC42, CFL1, FLOT1, HSP90AA1, ITGB1, RAB10, RAC2, RHOA, RTN4, VCL, YWHAH
1.0000	2.57	ACTB, CDC42, CFL1, FLOT1, HSP90AA1, ITGB1, RAB10, RAC2, RHOA, RTN4, VCL, YWHAH
1.0000	2.57	ACTB, ANK1, ARL6IP5, F11R, FLNA, FLOT1, ITGB1, JUP, LRP1, RAB10, SPTBN1, TSPAN14
1.0000	2.57	ACTB, CDC42, CFL1, FLOT1, HSP90AA1, ITGB1, RAB10, RAC2, RHOA, RTN4, VCL, YWHAH
1.0000	1.55	AACS, ACTB, ADAM10, ANK1, ARL6IP5, BCAP31, CALM3, CANX, CCL5, CCT2, CCT4, CCT6A
1.0000	1.55	AACS, ACTB, ADAM10, ANK1, ARL6IP5, BCAP31, CALM3, CANX, CCL5, CCT2, CCT4, CCT6A
1.0000	2.25	ARL6IP5, CCL5, CD36, CDC42, GNAI2, GSTP1, ITGB3, PPIA, PRDX1, PRDX2, PTPRJ, RAP1B,
1.0000	2.25	ANXA2, B2M, CDC42, F11R, FLNA, ILK, ITGB1, ITGB3, LTF, MYADM, RCC2, RTN4, VCL, XRC
1.0000	2.25	ANXA2, ATP2A3, ATP5F1B, CALM3, FLNA, G6PD, GP1BA, GP1BB, ITGB1, ITGB3, SRI, STOM
1.0000	2.25	ANXA2, ATP2A3, ATP5F1B, CALM3, FLNA, G6PD, GP1BA, GP1BB, ITGB1, ITGB3, SRI, STOM
1.0000	1.64	AACS, ARL6IP5, BCAP31, CALM3, CANX, CCL5, CCT2, CCT4, CCT6A, CCT7, CCT8, CD36, CLT
1.0000	3.38	ALDOA, CALM3, DSP, FLNA, HSP90AA1, JUP, SRI
1.0000	3.38	CALM3, FLOT1, GNAI2, ITGB1, RAP1B, YWHAG, YWHAH
1.0000	3.38	CTSD, GAPDH, LRBA, QSOX1, RAB1B, RAB33B, UBQLN1
1.0000	3.38	ANXA2, CD36, CD9, GP1BA, KRT1, PRDX2, THBS1
1.0000	3.38	CD47, CDC42, F11R, ITGB1, MYH9, PFN1, RHOA
1.0000	3.38	CALM3, ITGB1, SRI, STOM, UBQLN1, YWHAE, YWHAH
1.0000	3.38	ANK1, CDC42, FSCN1, ILK, MSN, PATJ, RHOA
1.0000	3.38	ACTB, CALM3, CCL5, FLNA, G6PD, ITGB1, SRI
1.0000	3.38	CCL5, HSP90AA1, ILK, ITGB1, PTPRJ, RTN4, THBS1

1.0000	3.38	CAMP, CYBB, ITGB1, ITGB3, JUP, PKM, THBS1
1.0000	3.38	CD9, G6PD, HSP90AA1, ITGB1, RTN4, SOD1, VCL
1.0000	3.38	CALM3, FLOT1, GNAI2, ITGB1, RAP1B, YWHAG, YWHAH
1.0000	3.38	ANK1, CDC42, FSCN1, ILK, MSN, PATJ, RHOA
1.0000	3.38	CAMP, GAPDH, H2BC21, KRT6A, LTF, PF4V1, S100A9
1.0000	3.38	ARPC5, CDC42, FSCN1, HSP90AA1, ITGB1, RAC2, VCL
1.0000	3.38	CALM3, FLOT1, GNAI2, ITGB1, RAP1B, YWHAG, YWHAH
1.0000	3.38	CALM3, FLOT1, GNAI2, ITGB1, RAP1B, YWHAG, YWHAH
1.0000	3.38	CALM3, FLOT1, GNAI2, ITGB1, RAP1B, YWHAG, YWHAH
1.0000	3.38	ANXA2, CD36, CD9, GP1BA, KRT1, PRDX2, THBS1
1.0000	3.38	CAMP, CYBB, ITGB1, ITGB3, JUP, PKM, THBS1
1.0000	3.38	ANK1, FSCN1, HNRNPA2B1, HSPA8, JUP, MYH9, NPM1
1.0000	1.52	ACTB, ADAM10, ANXA2, B2M, CAMP, CCL5, CD151, CD226, CD36, CD47, CD59, CD9, CD9
1.0000	1.52	AACS, ACTB, ADAM10, ANK1, ANXA2, ARL6IP5, BCAP31, CALM3, CANX, CCL5, CCT2, CCT
1.0000	1.55	ADAM10, ARL6IP5, BCAP31, CALM3, CCL5, CD226, CD36, CD47, CDC42, EIF5A, ENO1, F1
1.0000	1.69	ACTB, ANXA2, ARL6IP5, BCAP31, CCL5, CD226, CD36, CD47, CD9, DHX9, ENO1, FCN1, FL
1.0000	1.95	AACS, BCAP31, CCL5, CCT2, CCT4, CCT6A, CCT7, CCT8, CD36, CTSD, FLNA, HSP90AA1, HS
1.0000	1.51	ADAM10, ALDOA, ANPEP, ANXA11, ANXA2, ANXA7, ARPC5, B2M, CALM3, CAMP, CCT2, C
1.0000	1.86	ACTB, ANXA2, B2M, CALM3, CANX, CD151, CD36, CD9, CDC42, CLTA, FCER1G, FCN1, FLO
1.0000	1.60	ACTB, ADAM10, ADH5, ALDOA, ANPEP, ANTXR2, ANXA11, ANXA2, ANXA5, ANXA7, ARPC
1.0000	2.54	CD36, DHX9, FLNA, HNRNPA2B1, HSP90AA1, HSPA9, JUP, NPM1, RPS15, YWHAE
1.0000	2.54	CD36, DHX9, FLNA, HNRNPA2B1, HSP90AA1, HSPA9, JUP, NPM1, RPS15, YWHAE
1.0000	2.54	CALM3, FLNA, GP1BA, GP1BB, HNRNPU, ITGB3, S100A8, S100A9, SRGN, SRI
1.0000	2.54	ATP2A3, CALM3, CDC42, DSP, FLNA, HSP90AA1, JUP, SOD1, SRI, YWHAE
1.0000	2.54	CAMP, CCL5, CD36, GSTP1, LBP, LTF, PF4V1, PRDX2, RHOA, VIM
1.0000	2.54	CAMP, CCL5, CD36, GSTP1, LBP, LTF, PF4V1, PRDX2, RHOA, VIM
1.0000	2.54	CAMP, CCL5, CD36, GSTP1, LBP, LTF, PF4V1, PRDX2, RHOA, VIM
1.0000	2.54	CALM3, FLNA, G6PD, ITGB1, ITGB3, SRI, STOM, UBQLN1, YWHAE, YWHAH
1.0000	2.54	CCL5, CD99, FCER1G, LBP, PECAM1, PF4V1, PPIA, RAC2, S100A8, S100A9
1.0000	2.54	ADAM10, ANK1, FSCN1, HNRNPA2B1, HSPA8, ITGA2B, JUP, MYH9, NPM1, VIM
1.0000	1.50	ADAM10, ANPEP, ANTXR2, ARL6IP5, ATP2A3, B2M, BCAP31, C16orf54, CALM3, CANX, CD
1.0000	2.19	ADH5, CAMP, CCL5, CD36, GSTP1, LBP, LTF, PF4V1, PRDX2, RHOA, S100A8, S100A9, VIM
1.0000	2.19	ADAM10, ANXA2, CPN1, ENO1, FLNA, GP1BA, GP1BB, HSPD1, MYH9, PGK1, SRGN, THBS
1.0000	2.19	ACTB, CD53, CD9, DSP, FLOT1, G6PD, HNRNPU, ITGB1, MYH9, MYL6, PTBP1, RHOA, SRI
1.0000	2.19	CCL5, CD47, CD9, CD99, FCER1G, LBP, PECAM1, PF4V1, PPIA, RAC2, S100A8, S100A9, TH
1.0000	2.19	ALDOA, ANK1, CALM3, ENO1, FLNA, ILK, JUP, MYL12B, PSMA6, SLC2A1, SPTBN1, SRI, TP
1.0000	1.46	ADAM10, ALDOA, ANPEP, ANXA11, ANXA2, ANXA7, ARPC5, B2M, CAMP, CCT2, CCT8, CD
1.0000	1.62	AACS, ACTB, ADAM10, BCAP31, CCL5, CCT2, CCT4, CCT6A, CCT7, CCT8, CD36, CTSD, FLN
1.0000	1.99	ARL6IP5, CCL5, CD36, CDC42, GNAI2, GSTP1, ITGB3, PPIA, PRDX1, PRDX2, PTPRJ, RAP1B,
1.0000	1.99	ANXA11, CD36, CD47, CDC42, FCER1G, FCN1, ITGB1, ITGB3, LBP, LRP1, MYH9, PECAM1,
1.0000	1.99	ADH5, ANPEP, ATP2A3, CALM3, CDC42, CYB5R3, DSP, FLNA, HSP90AA1, JUP, PTGS1, PTP
1.0000	1.99	ACTB, CD36, CDC42, CLIC1, F11R, FLNA, HNRNPU, HSPA1B, ILK, INPP5D, ITGB1, ITGB3, LT
1.0000	1.99	ANXA11, ANXA2, ANXA5, ANXA7, CD151, CTSD, FCN1, HRNR, HSP90AA1, KRT1, LGALS3B
1.0000	1.65	ACTB, ADAM10, ATP5F1B, B2M, CCL5, CD36, CD47, CD9, CDC42, F11R, FERMT3, FLNA, F
1.0000	1.65	ACTB, BCAP31, CALM3, CAMP, CANX, CD36, CD9, CYBB, DHX9, FCER1G, FLNA, FLOT1, G
1.0000	3.76	CD36, ENO1, HNRNPA2B1, HNRNPU, YWHAZ
1.0000	3.76	CD36, GSTP1, PRDX1, PRDX2, SOD1

1.0000	3.76	CD53, CD9, FLOT1, ITGB1, MYH9
1.0000	3.76	CD36, CDC42, MYH9, RAC2, THBS1
1.0000	3.76	CALM3, DSP, HSP90AA1, JUP, SRI
1.0000	3.76	CD53, CD9, FLOT1, ITGB1, MYH9
1.0000	3.76	CD53, CD9, FLOT1, ITGB1, MYH9
1.0000	3.76	CDC42, FLNA, ILK, ITGB3, MYADM
1.0000	3.76	CD53, CD9, FLOT1, ITGB1, MYH9
1.0000	3.76	CDC42, CFL1, ITGB1, RHOA, YWHAH
1.0000	3.76	AACS, CALM3, CCL5, RAP1B, SRI
1.0000	3.76	CCL5, CD36, LBP, LTF, PRDX2
1.0000	3.76	CALM3, FLNA, G6PD, ITGB1, SRI
1.0000	3.76	CALM3, FLNA, G6PD, ITGB1, SRI
1.0000	3.76	CDC42, FSCN1, MSN, PATJ, RHOA
1.0000	3.76	CCL5, F11R, ITGA6, ITGB1, RCC2
1.0000	3.76	FLNA, HNRNPU, SRGN, SRI, YWHAB
1.0000	3.76	B2M, ITGB1, RHOA, YWHAG, YWHAH
1.0000	3.76	DHX9, FLNA, HSP90AA1, JUP, YWHAE
1.0000	3.76	G6PD, HSP90AA1, ITGB1, RTN4, VCL
1.0000	3.76	HSP90AA1, ITGB1, PTPRJ, RTN4, THBS1
1.0000	3.76	HSP90AA1, ITGB1, PTPRJ, RTN4, THBS1
1.0000	3.76	CALM3, DSP, HSP90AA1, JUP, SRI
1.0000	3.76	CDC42, FSCN1, MSN, PATJ, RHOA
1.0000	3.76	CDC42, FSCN1, MSN, PATJ, RHOA
1.0000	3.76	CALM3, DSP, FLNA, JUP, SRI
1.0000	3.76	DSC1, DSG1, ITGB1, MYADM, PECAM1
1.0000	3.76	ANXA2, FLNA, GP1BA, GP1BB, LTF
1.0000	3.76	CALM3, CD36, DSP, FLNA, JUP
1.0000	3.76	ACTB, CALM3, CANX, RAB27B, RAP1B
1.0000	3.76	CDC42, FSCN1, HSP90AA1, PFN1, RAC2
1.0000	3.76	CD53, CD9, FLOT1, ITGB1, MYH9
1.0000	3.76	ACTB, DSP, F11R, PECAM1, VCL
1.0000	3.76	CDC42, FLNA, ILK, ITGB3, MYADM
1.0000	3.76	AACS, ANXA2, PPIA, RAB27B, SRI
1.0000	3.76	CALM3, ITGB1, SRI, STOM, UBQLN1
1.0000	3.76	CDC42, FSCN1, ITGB1, ITGB3, MSN
1.0000	3.76	ITGA2B, ITGA6, ITGB1, ITGB3, MMRN1
1.0000	3.76	ADAM10, CASP14, CTSD, LTF, PSMA6
1.0000	3.76	ACTB, DHX9, NPM1, PSMA6, YWHAH
1.0000	1.68	ANPEP, ANXA11, ANXA2, CCT2, CCT8, CDC42, CLTA, CTSD, CYB5R3, EEF1A1, FLOT1, GNA(
1.0000	1.73	AACS, BCAP31, CCT2, CCT4, CCT6A, CCT7, CCT8, FLNA, HSP90AA1, ITGB1, JUP, LRP1, MSN
1.0000	1.79	ADH5, ANPEP, ATP2A3, CALM3, CD36, CDC42, CYB5R3, DSP, FLNA, GNAI2, HSP90AA1, JU
1.0000	1.79	CCL5, CD36, CD47, CYBB, DHX9, F11R, GSTP1, ITGB1, KRT1, KRT16, LBP, LRP1, LYZ, PF4V1
1.0000	1.50	ADH5, ALDOA, ANPEP, ANXA2, ANXA7, BCAP31, CANX, CD36, CPN1, CSDE1, CTSD, DHX9,
1.0000	1.45	ADAM10, ANPEP, ANTXR2, ANXA2, ANXA7, ATP2A3, B2M, BCAP31, CALM3, CD36, CD47,
1.0000	1.51	ACTB, ADAM10, ANXA5, ARPC5, B2M, CD151, CD59, CD9, CD99, CDC42, CFL1, FLNA, FLO
1.0000	1.53	ACTB, ANXA2, ARPC5, ATIC, B2M, CALM3, CD9, CDC42, CFL1, FLNA, FLOT1, G6PD, GSTP1
1.0000	Inf	AACS, ACTB, ADAM10, ADH5, ALDOA, ANK1, ANPEP, ANTXR2, ANXA11, ANXA2, ANXA5, /

1.0000	2.52	ANXA2, FLNA, GP1BA, GP1BB, LTF, PRDX1, RAB33B, RHOA
1.0000	2.52	ACTB, FLOT1, HSP90AA1, ITGB1, RAB10, RAC2, RTN4, VCL
1.0000	2.52	ATP2A3, CALM3, DSP, FLNA, HSP90AA1, JUP, SRI, YWHAE
1.0000	2.52	AACS, CANX, CCL5, M6PR, PLEK, PPIA, RAB10, SRI
1.0000	2.52	CCL5, FCER1G, LBP, PF4V1, PPIA, RAC2, S100A8, S100A9
1.0000	2.52	AACS, CANX, CCL5, M6PR, PLEK, PPIA, RAB10, SRI
1.0000	2.52	CD47, CD99, F11R, GP1BA, ITGB1, PECAM1, SELPLG, SPN
1.0000	2.52	CD36, ENO1, FLNA, HNRNPA2B1, HNRNPU, HSPA8, XRCC5, YWHAZ
1.0000	2.52	ANXA2, ANXA5, CD36, CD9, GP1BA, KRT1, PRDX2, THBS1
1.0000	2.52	CD47, CDC42, F11R, FLNA, FSCN1, PFN1, PLEK, RHOA
1.0000	2.52	CD47, CDC42, F11R, FLNA, FSCN1, PFN1, PLEK, RHOA
1.0000	2.52	AACS, CANX, CCL5, M6PR, PLEK, PPIA, RAB10, SRI
1.0000	2.52	BCAP31, FLNA, HSP90AA1, JUP, RAC2, STOM, UBE2L3, YWHAE
1.0000	2.52	CALM3, FLOT1, GNAI2, GNAQ, ITGB1, RAP1B, YWHAG, YWHAH
1.0000	2.52	CD36, ENO1, FLNA, HNRNPA2B1, HNRNPU, HSPA8, XRCC5, YWHAZ
1.0000	2.52	CD36, ENO1, FLNA, HNRNPA2B1, HNRNPU, HSPA8, XRCC5, YWHAZ
1.0000	2.52	CD36, DSG1, HSP90AA1, ITGB3, MSN, PATJ, RAB27B, SLC2A1
1.0000	2.52	B2M, BCAP31, CANX, HLA-A, HLA-B, HSPA9, QSOX1, RTN4
1.0000	2.52	ACTB, ARPC5, CALM3, CFL1, FLNA, FSCN1, HSP90AA1, HSPA8
1.0000	2.52	ALDOA, B2M, CAMP, CTSD, FLG2, LTF, LYZ, QSOX1
1.0000	2.52	ARPC5, CFL1, FLNA, FSCN1, MYH9, SPTBN1, TLN1, TPM4
1.0000	1.58	ACTB, ANXA2, B2M, CD36, CDC42, CLIC1, F11R, FLNA, G6PD, HLA-B, HNRNPU, HSPA1B, H
1.0000	1.58	ACTB, CAMP, CD36, CD53, CDC42, CFL1, CLIC1, CYBB, F11R, FLNA, FLOT1, HNRNPU, HSPA
1.0000	1.58	ADAM10, ATIC, B2M, CCL5, CYBB, ENO1, F11R, FCER1G, FLNA, G6PD, GALE, H2AC20, H2B
1.0000	1.36	ACTB, ADAM10, ANK1, ANPEP, ANTXR2, ANXA2, ANXA5, ARL6IP5, ATIC, ATP5F1B, B2M,
1.0000	1.40	ACTB, ANXA2, ANXA5, ARL6IP5, ATP2A3, B2M, BCAP31, CALM3, CCL5, CCT2, CCT4, CD36
1.0000	1.40	ADAM10, ANPEP, ANTXR2, ARL6IP5, ATP2A3, B2M, BCAP31, C16orf54, CALM3, CANX, CD
1.0000	2.10	ATP2A3, CALM3, CDC42, DSP, FLNA, GNAI2, HSP90AA1, JUP, SOD1, SRI, YWHAE
1.0000	2.10	CCL5, CD99, FCER1G, LBP, PECAM1, PF4V1, PPIA, RAC2, S100A8, S100A9, THBS1
1.0000	2.10	ANXA11, ANXA2, CCT2, CCT8, CYB5R3, HRNR, LYZ, PRDX6, RAP1B, STOM, TUBB
1.0000	2.10	ANXA11, CALM3, CDC42, CLTA, HNRNPU, MYH9, NPM1, RCC2, TTLL12, TUBB, TUBB1
1.0000	2.10	ALDOA, CD36, CD9, FERMT3, ITGA2B, ITGB3, MMRN1, PECAM1, QSOX1, SRGN, THBS1
1.0000	2.10	ANXA11, ANXA2, CCT2, CCT8, CYB5R3, HRNR, LYZ, PRDX6, RAP1B, STOM, TUBB
1.0000	1.65	ADH5, B2M, CAMP, CCL5, CD36, DCD, FCER1G, GSDMA, GSTP1, H2BC21, HLA-A, HLA-B, K
1.0000	1.65	ANXA2, B2M, CALM3, CLTA, CYBB, FCER1G, FLNA, FLOT1, GNAI2, GNAQ, GNG5, GP1BA, G
1.0000	1.65	ADH5, ANXA11, ANXA2, ANXA5, ANXA7, CAMP, CAVIN2, CD36, FERMT3, GSDMA, GSTP1,
1.0000	1.45	ACTB, ANXA2, CD36, CSDE1, DAZAP1, DHX9, EIF5A, ENO1, FLNA, FLOT1, HINT1, HNRNPA
1.0000	1.45	ACTB, ADAM10, ANXA5, ARPC5, B2M, CD151, CD59, CD9, CD99, CDC42, CFL1, FERMT3, F
1.0000	1.90	CFL1, FLNA, GAPDH, HNRNPU, HSPA1B, ITGB1, MYH9, NPM1, PATJ, RHOA, TUBA1C, TUBA
1.0000	1.90	ATP5F1B, CDC42, ILK, ITGB1, ITGB3, JUP, KRT16, KRT2, MYH9, PFN1, RCC2, RHOA, RTN4,
1.0000	1.90	ADH5, B2M, CAMP, CCL5, CD36, GSTP1, LBP, LTF, PF4V1, PRDX2, RHOA, S100A8, S100A9
1.0000	1.90	CD36, CDC42, FLNA, ILK, ITGB3, JUP, MYADM, PTPRJ, RAC2, RCC2, RHOA, THBS1, TLN1, V
1.0000	1.90	ALDOA, CDC42, CFL1, F11R, FLNA, ILK, ITGB3, MSN, MYADM, MYH9, MYL12B, RAC2, RCC
1.0000	1.90	ACTB, ANXA2, DHX9, EIF5A, HNRNPU, ILK, ITGA6, JUP, KANSL3, NCL, NPM1, PCBP1, UBE2
1.0000	1.90	ACTB, ANXA2, DHX9, EIF5A, HNRNPU, ILK, ITGA6, JUP, KANSL3, NCL, NPM1, PCBP1, UBE2
1.0000	1.90	ACTB, ANXA2, DHX9, EIF5A, HNRNPU, ILK, ITGA6, JUP, KANSL3, NCL, NPM1, PCBP1, UBE2
1.0000	1.90	ACTB, ADAM10, ANK1, ARL6IP5, F11R, FLNA, FLOT1, ITGB1, JUP, LRP1, MYADM, RAB10, S

1.0000	1.90	ANPEP, ANXA2, CLTA, CTSD, EEF1A1, FLOT1, GNAQ, HSPA8, LRBA, LRP1, M6PR, RAP1B, S
1.0000	1.90	ALDOA, ANK1, CALM3, ENO1, FLNA, ILK, JUP, MYL12B, PSMA6, SLC2A1, SPTBN1, SRI, TPN
1.0000	1.90	ALDOA, ANK1, CALM3, ENO1, FLNA, ILK, JUP, MYL12B, PSMA6, SLC2A1, SPTBN1, SRI, TPN
1.0000	1.90	ACTB, ADAM10, CANX, CDC42, CLTA, HSPA8, ITGB1, ITGB3, RHOA, RPL8, RPLP0, RTN3, R
1.0000	1.70	ACTB, B2M, CCL5, CD47, F11R, FERMT3, GP1BA, HLA-A, HSPD1, ITGB1, MSN, PECAM1, P
1.0000	1.70	CALM3, CAMP, CCL5, CD36, CDC42, FLOT1, HSP90AA1, ILK, ITGA6, ITGB3, LTF, NPM1, PE
1.0000	1.70	ANXA11, ANXA2, ANXA5, ANXA7, CALM3, CANX, DSC1, DSG1, FLG2, HRNR, LRP1, MMRN
1.0000	1.78	ACTB, ANXA2, CD36, DHX9, EIF5A, ENO1, HNRNPA2B1, HNRNPU, ITGA6, JUP, KANSL3, N
1.0000	1.78	ACTB, ANXA2, CD36, DHX9, EIF5A, ENO1, HNRNPA2B1, HNRNPU, ITGA6, JUP, KANSL3, N
1.0000	1.78	ANXA2, ATP2A3, ATP5F1B, CALM3, CLIC1, FLNA, G6PD, GP1BA, GP1BB, ITGB1, ITGB3, PC
1.0000	1.78	AACS, BCAP31, CCT2, CCT4, CCT6A, CCT7, CCT8, FLNA, HSP90AA1, JUP, PPIA, RAC2, SRI, S
1.0000	1.78	ANXA11, B2M, CD36, CD9, CDC42, CLTA, CYBB, HLA-A, HLA-B, HSP90AA1, LRP1, LTF, M6I
1.0000	1.45	ARL6IP5, BCAP31, CALM3, CCL5, CD36, CDC42, EIF5A, ENO1, F11R, FLNA, FLOT1, GAPDH,
1.0000	1.35	ACTB, ADAM10, ANPEP, ANXA2, ARPC5, ATIC, ATP5F1B, B2M, CALM3, CAMP, CD9, CDC4
1.0000	4.97	ACTB, ITGB1, NASP
1.0000	4.97	LBP, S100A8, S100A9
1.0000	4.97	AACS, S100A8, SRI
1.0000	4.97	FLNA, ITGB1, YWHAE
1.0000	4.97	CD36, FCN1, LBP
1.0000	4.97	CD9, ILK, SOD1
1.0000	4.97	CD9, ILK, SOD1
1.0000	4.97	AACS, CCL5, SRI
1.0000	4.97	GAPDH, QSOX1, UBQLN1
1.0000	4.97	GNAI2, MYH9, RAP1B
1.0000	4.97	ACTB, HINT1, KANSL3
1.0000	4.97	AACS, CCL5, SRI
1.0000	4.97	AACS, CCL5, SRI
1.0000	4.97	CD36, PRDX2, THBS1
1.0000	4.97	ATIC, CD9, FLNA
1.0000	4.97	CD36, LTF, PRDX2
1.0000	4.97	ANXA11, MYH9, RHOA
1.0000	4.97	F11R, FLOT1, ITGB1
1.0000	4.97	ILK, PRDX1, RHOA
1.0000	4.97	CD9, ILK, SOD1
1.0000	4.97	FLNA, ITGB1, YWHAE
1.0000	4.97	ACTB, ITGB1, NASP
1.0000	4.97	CD36, CD47, HSP90AA1
1.0000	4.97	F11R, FLOT1, ITGB1
1.0000	4.97	AACS, CCL5, SRI
1.0000	4.97	AACS, CCL5, SRI
1.0000	4.97	CDC42, ITGB3, RTN4
1.0000	4.97	B2M, RTN4, YWHAH
1.0000	4.97	AACS, CCL5, SRI
1.0000	4.97	CD36, PRDX2, THBS1
1.0000	4.97	F11R, FLOT1, ITGB1
1.0000	4.97	S100A8, S100A9, SRI
1.0000	4.97	CFL1, ITGB1, MYH9

1.0000	4.97	CFL1, ITGB1, MYH9
1.0000	4.97	B2M, RTN4, YWHAH
1.0000	4.97	FLNA, ITGB1, YWHAE
1.0000	4.97	ILK, ITGB3, PECAM1
1.0000	4.97	CD36, CD47, HSP90AA1
1.0000	4.97	DSP, FLNA, JUP
1.0000	4.97	DSP, JUP, SRI
1.0000	4.97	AACS, CCL5, SRI
1.0000	4.97	CD9, F11R, MMRN1
1.0000	4.97	HLA-A, HLA-B, LBP
1.0000	4.97	CD36, PRDX2, THBS1
1.0000	4.97	ILK, PRDX1, RHOA
1.0000	4.97	FLNA, ITGB1, YWHAE
1.0000	4.97	DSP, JUP, SRI
1.0000	4.97	ACTB, H2AC20, H2BC21
1.0000	4.97	CTSD, HSP90AA1, HSPA8
1.0000	4.97	FLNA, G3BP1, SLC2A3
1.0000	4.97	QSOX1, TUBB, TUBB1
1.0000	4.97	CANX, ITGB1, ITGB3
1.0000	4.97	CANX, ITGB1, ITGB3
1.0000	4.97	CCL5, ILK, PKM
1.0000	4.97	NPM1, YWHAB, YWHAG
1.0000	4.97	CASP14, CTSD, HINT1
1.0000	4.97	ADAM10, ANPEP, CPN1
1.0000	4.97	ADAM10, ANPEP, CPN1
1.0000	4.97	ADAM10, ANPEP, CPN1
1.0000	4.97	PRDX1, PRDX2, PRDX6
1.0000	4.97	CD36, SLC2A1, SLC2A3
1.0000	4.97	CCL5, ILK, PKM
1.0000	4.97	NPM1, YWHAB, YWHAG
1.0000	4.97	CD36, S100A8, S100A9
1.0000	4.97	YWHAB, YWHAE, YWHAZ
1.0000	4.97	PRDX1, PRDX2, PRDX6
1.0000	4.97	CYB5R3, PCYOX1, QSOX1
1.0000	4.97	CALM3, GAPDH, HSP90AA1
1.0000	4.97	PRDX1, PRDX2, PRDX6
1.0000	1.45	ACTB, ANXA7, CASP14, CD151, CDC42, DSP, F11R, FLG2, FLNA, HNRNPH3, HRNR, ILK, ITG
1.0000	1.33	ACTB, ADAM10, ALDOA, ANPEP, ANXA2, ANXA7, ARPC5, ATIC, ATP5F1B, B2M, CALM3, C
1.0000	1.39	ACTB, ADAM10, ADH5, ALDOA, ANXA2, ATIC, B2M, CCL5, CD226, CD53, CDC42, ENO1, F
1.0000	1.46	ADAM10, ATP5F1B, CCL5, CD151, CD47, CD9, CD99, FCER1G, FERMT3, FLNA, GNAI2, GP1
1.0000	1.46	AACS, ACTB, ADAM10, ANXA2, BCAP31, CCL5, CCT2, CCT4, CCT6A, CCT7, CCT8, CD36, CT
1.0000	2.36	AACS, ACTB, ADAM10, ADH5, ALDOA, ANK1, ANPEP, ANXA11, ANXA2, ANXA5, ANXA7, A
1.0000	1.47	AACS, ACTB, ANXA2, B2M, BCAP31, CALM3, CCL5, CD151, CD36, CD47, CDC42, DHX9, FC
1.0000	1.47	ADAM10, ANK1, ANXA2, CANX, CAVIN2, CD36, CFL1, CLTA, DSG1, DSP, EEF1A1, FLOT1, F
1.0000	1.32	ACTB, ADAM10, ANPEP, ANXA2, ARPC5, ATIC, ATP5F1B, B2M, CALM3, CAMP, CD9, CDC4
1.0000	1.48	ACTB, ALDOA, ANK1, ANXA2, ARPC5, CALM3, CFL1, FLNA, FSCN1, GAPDH, HNRNPU, HSP
1.0000	1.31	ACTB, ADAM10, ANXA2, ARL6IP5, B2M, BCAP31, CALM3, CCL5, CD226, CD36, CD47, CD5

1.0000	1.50	ACTB, ANXA2, ARL6IP5, ATP2A3, ATP5F1B, CALM3, CD36, CLIC1, CYBB, FLNA, G6PD, GP1
1.0000	1.50	ACTB, CAMP, CCL5, CD36, CYBB, EEF1A1, FLNA, GNAI2, HNRNPU, HSPA1B, HSPA8, ILK, IT
1.0000	1.50	ADAM10, CALM3, CCT6A, CDC42, F11R, GAPDH, GNG5, HSP90AA1, HSPA1B, HSPA8, INPI
1.0000	2.49	ACTB, CDC42, FLNA, HNRNPU, HSPA1B, RHOA
1.0000	2.49	ADAM10, ITGB1, MATR3, MYH9, NASP, RTN4
1.0000	2.49	F11R, MSN, MYADM, PECAM1, RAP1B, VCL
1.0000	2.49	CDC42, CFL1, F11R, FLOT1, ITGB1, RHOA
1.0000	2.49	DSP, FLOT1, ITGB1, MYL6, RHOA, SRI
1.0000	2.49	CD36, CDC42, MYH9, RAC2, RHOA, THBS1
1.0000	2.49	CDC42, FLNA, ILK, ITGB3, MYADM, RCC2
1.0000	2.49	B2M, FLNA, RHOA, RTN4, VIM, YWHAH
1.0000	2.49	CD36, FLOT1, LBP, LTF, RTN4, UBQLN1
1.0000	2.49	CCL5, F11R, ITGA6, ITGB1, RCC2, SOD1
1.0000	2.49	CD36, FLNA, GAPDH, LTF, RHOA, TFRC
1.0000	2.49	ATP5F1B, ITGB1, JUP, MYH9, RHOA, THBS1
1.0000	2.49	ANK1, CDC42, ILK, MSN, PATJ, RHOA
1.0000	2.49	F11R, MSN, MYADM, PECAM1, RAP1B, VCL
1.0000	2.49	ACTB, CDC42, FLNA, HNRNPU, HSPA1B, RHOA
1.0000	2.49	ANXA2, B2M, ITGB1, RTN4, XRCC5, YWHAH
1.0000	2.49	ARL6IP5, B2M, CD36, HSP90AA1, ITGB1, SOD1
1.0000	2.49	ANXA2, B2M, ITGB1, RTN4, XRCC5, YWHAH
1.0000	2.49	ANXA2, FLNA, GP1BA, GP1BB, LTF, RHOA
1.0000	2.49	CDC42, FSCN1, HSP90AA1, PFN1, RAC2, RCC2
1.0000	2.49	F11R, MSN, MYADM, PECAM1, RAP1B, VCL
1.0000	2.49	ANXA2, FLNA, G6PD, GP1BA, GP1BB, LTF
1.0000	2.49	ARL6IP5, CD36, ITGB1, SLC2A1, SLC2A3, THBS1
1.0000	2.49	ACTB, CALM3, CANX, ITGB3, RAB27B, RAP1B
1.0000	2.49	CD36, CDC42, MYH9, RAC2, RHOA, THBS1
1.0000	2.49	CDC42, FSCN1, HSP90AA1, PFN1, RAC2, RCC2
1.0000	2.49	ACTB, CDC42, FLNA, HNRNPU, HSPA1B, RHOA
1.0000	2.49	CDC42, FLNA, ILK, ITGB3, MYADM, RCC2
1.0000	2.49	ACTB, ADAM10, ITGB1, LRP1, SPTBN1, STOM
1.0000	2.49	ADAM10, B2M, CANX, QSOX1, THBS1, TSPAN14
1.0000	2.49	ANXA2, BCAP31, CYB5R3, GAPDH, OSBPL2, RAP1B
1.0000	2.49	DSP, ITGB1, JUP, SLC2A1, VCL, YWHAH
1.0000	2.49	B2M, BCAP31, CANX, HLA-A, HLA-B, RTN4
1.0000	2.49	ARPC5, CALM3, CFL1, FLNA, FSCN1, HSP90AA1
1.0000	2.49	ARPC5, CALM3, CFL1, FLNA, FSCN1, HSP90AA1
1.0000	2.49	B2M, BCAP31, CANX, HLA-A, HLA-B, RTN4
1.0000	2.49	ACTB, H2AC20, H2BC21, JUP, NPM1, XRCC5
1.0000	2.49	ADAM10, CANX, CLTA, HSPA8, ITGB1, ITGB3
1.0000	2.49	ANXA2, CAVIN2, GSDMA, HSPA8, THBS1, TLN1
1.0000	2.49	CDC42, RAB10, RAB1B, RAB27B, RAP1B, RHOA
1.0000	2.49	ANXA11, B2M, HSP90AA1, HSPA8, PKM, YWHAE
1.0000	2.49	ANXA11, B2M, HSP90AA1, HSPA8, PKM, YWHAE
1.0000	2.49	ANXA11, ANXA2, ANXA7, CALM3, S100A8, S100A9
1.0000	2.49	ANXA2, CAVIN2, GSDMA, HSPA8, THBS1, TLN1

1.0000	1.39	ADH5, ANPEP, ANXA2, ANXA7, BCAP31, CANX, CD36, CPN1, CSDE1, CTSD, DHX9, EEF1A1
1.0000	1.34	ACTB, ANXA2, CCL5, CCT2, CCT4, CCT6A, CCT7, CCT8, CD36, CSDE1, DHX9, EEF2, EIF5A, E
1.0000	1.34	ACTB, ADAM10, ARL6IP5, B2M, BCAP31, CCL5, CD226, CD36, CD47, CD59, CDC42, DHX9,
1.0000	1.56	ACTB, ALDOA, ARPC5, CD47, CDC42, CFL1, F11R, FLNA, FSCN1, ITGB1, ITGB3, LRP1, MYA
1.0000	1.56	ANXA2, CALM3, CAVIN2, CD36, CD9, CDC42, FLOT1, HSP90AA1, HSPA8, HSPA9, MYADM,
1.0000	1.56	ACTB, CAMP, CD36, CYBB, DHX9, FLNA, FLOT1, GNAI2, GNAQ, ITGB3, JUP, LRP1, NCL, NP
1.0000	1.56	ADAM10, BCAP31, CLIC1, CYBB, GAPDH, GSDMA, HRNR, HSP90AA1, HSPA1B, HSPA8, ITG
1.0000	1.56	ADAM10, ALDOA, B2M, CAMP, CD47, CD53, CD59, CTSD, CYBB, DSC1, DSG1, DSP, FCER1
1.0000	1.32	AACS, ACTB, ADAM10, ANK1, ANPEP, ANXA2, ANXA5, ARL6IP5, ARPC5, ATP2A3, ATP5F1
1.0000	1.61	CDC42, CFL1, FLNA, GAPDH, HNRNPU, HSPA1B, HSPA8, ITGB1, MYH9, NPM1, PATJ, RAB2
1.0000	1.61	ACTB, ANXA11, CALM3, CDC42, CFL1, FLNA, HNRNPU, HSPA1B, ITGB1, MYH9, NASP, NP
1.0000	1.61	ARPC5, CD47, CDC42, DSP, F11R, FLNA, FSCN1, ITGB3, JUP, LRP1, MYADM, MYH9, PFN1,
1.0000	1.29	ADAM10, ANPEP, ANTXR2, ANXA2, ANXA7, ARL6IP5, ATP2A3, ATP5F1B, B2M, BCAP31, C
1.0000	2.00	ANXA2, CAVIN2, CD9, FLOT1, MYH9, OSBPL2, S100A9, SOD1, SPTBN1
1.0000	2.00	CD9, ILK, LRP1, RHOA, RTN4, S100A8, S100A9, SOD1, VIM
1.0000	2.00	CAMP, CYBB, ITGB1, ITGB3, JUP, KRT1, PGK1, PKM, THBS1
1.0000	2.00	CDC42, CFL1, HNRNPU, ITGB1, MYH9, NPM1, RAC2, RPS15, YWHAZ
1.0000	2.00	CCL5, FCER1G, LBP, PF4V1, PPIA, RAC2, S100A8, S100A9, THBS1
1.0000	2.00	ACTB, ARL6IP5, CD36, FLOT1, ITGB1, ITGB3, SLC2A1, SLC2A3, THBS1
1.0000	2.00	CD36, GSR, GSTP1, PRDX1, PRDX2, PRDX6, PTGS1, S100A9, SOD1
1.0000	2.00	ATP2A3, CALM3, DSP, FLNA, HSP90AA1, JUP, RHOA, SRI, YWHAE
1.0000	2.00	B2M, BCAP31, CANX, HLA-A, HLA-B, HSPA9, QSOX1, RAB27B, RTN4
1.0000	1.68	ACTB, B2M, F11R, INPP5D, ITGB1, ITGB3, KRT1, LTF, LYZ, PECAM1, PRDX1, SLC2A1, SOD1
1.0000	1.68	ADAM10, CANX, CDC42, CYBB, FLNA, G3BP1, GNAI2, HNRNPU, HSP90AA1, HSPA8, ITGB1
1.0000	1.68	ALDOA, ARPC5, CFL1, FLNA, FSCN1, HNRNPU, ITGB1, MSN, MYH9, PFN1, SPTBN1, TLN1,
1.0000	1.68	ANPEP, ANTXR2, CD36, CD47, FCER1G, FCN1, GP1BA, GP1BB, ITGB1, ITGB3, KRT1, LRP1,
1.0000	1.68	ANPEP, ANTXR2, CD36, CD47, FCER1G, FCN1, GP1BA, GP1BB, ITGB1, ITGB3, KRT1, LRP1,
1.0000	1.79	ALDOA, CALM3, DSP, ENO1, FLNA, HSP90AA1, JUP, MYL6, RHOA, SOD1, SRI, TPM4
1.0000	1.79	CDC42, DSP, FLNA, GNG5, ILK, ITGA6, LTF, MSN, RAB33B, RHOA, RTN4, SOD1
1.0000	1.79	ANXA2, BCAP31, CD36, DHX9, FLNA, HSP90AA1, JUP, MSN, RAC2, STOM, UBE2L3, YWHA
1.0000	1.79	ARL6IP5, CALM3, CD36, CLIC1, DSP, FLNA, GNAQ, JUP, SOD1, SRI, YWHAE, YWHAH
1.0000	1.79	ARL6IP5, CCL5, CD36, CDC42, GNAI2, ITGB3, PPIA, PRDX2, PTPRJ, RAP1B, SOD1, THBS1
1.0000	1.79	CD36, DHX9, FLOT1, HSPA1B, JUP, LTF, NPM1, PPIA, PSMA6, S100A8, S100A9, TFRC
1.0000	1.79	ATP2A3, BCAP31, CALM3, CCL5, FLNA, GP1BA, GP1BB, ITGB3, S100A8, S100A9, SRI, YWH
1.0000	1.79	ATP2A3, BCAP31, CALM3, CCL5, FLNA, GP1BA, GP1BB, ITGB3, S100A8, S100A9, SRI, YWH
1.0000	1.29	ACTB, ADAM10, ADH5, ANK1, ANPEP, ANXA11, ANXA2, ANXA5, ARL6IP5, ATIC, ATP2A3,
1.0000	1.33	ACTB, ANXA7, CASP14, CD151, CDC42, DSP, F11R, FLG2, FLNA, FLOT1, G6PD, HNRNPH3,
1.0000	1.33	ACTB, ALDOA, ANXA2, ATIC, ATP5F1B, CCT2, CCT4, CCT6A, CCT7, CCT8, CD36, DHX9, EIF
1.0000	1.26	ACTB, ADAM10, ADH5, ANK1, ANPEP, ANXA5, ARL6IP5, ATIC, ATP2A3, ATP5F1B, B2M, B
1.0000	1.40	ACTB, ADAM10, CALM3, CAMP, CCL5, CD36, CDC42, FLOT1, GNAQ, GSTP1, HSP90AA1, IL
1.0000	1.40	ANXA2, ANXA5, ATP2A3, CALM3, CCL5, CDC42, FLNA, GAPDH, GNAQ, GSTP1, HSP90AA1,
1.0000	1.33	ACTB, ANK1, ANXA11, ANXA2, B2M, BCAP31, CALM3, CANX, CCL5, CD151, CD36, CD47,
1.0000	1.29	ACTB, ANXA2, CCL5, CCT2, CCT4, CCT6A, CCT7, CCT8, CD36, CD47, CSDE1, DHX9, EEF2, E
1.0000	1.42	ACTB, B2M, CCL5, CD226, CD47, CD9, F11R, FCER1G, FLNA, GNAQ, GP1BA, GP1BB, HLA-
1.0000	1.42	ANPEP, ANXA11, ANXA2, CCT2, CCT8, CLTA, CTSD, CYB5R3, EEF1A1, FLOT1, GNAQ, HRNR
1.0000	1.42	ANPEP, ANXA11, ANXA2, CCT2, CCT8, CLTA, CTSD, CYB5R3, EEF1A1, FLOT1, GNAQ, HRNR
1.0000	1.29	ACTB, ADAM10, ALDOA, ANXA2, B2M, CAMP, CD36, CD53, CDC42, CFL1, CLIC1, CYBB, F1

1.0000	1.32	ANK1, ANXA2, ATP2A3, BCAP31, CD36, CDC42, CLTA, DHX9, FLNA, HNRNPA2B1, HNRNPU
1.0000	1.44	ACTB, ANXA2, ATP2A3, ATP5F1B, B2M, CALM3, CCL5, FLNA, G6PD, GNAI2, GP1BA, GP1B
1.0000	1.44	ALDOA, ATP2A3, ATP5F1B, BCAP31, CALM3, CCL5, FLNA, GP1BA, GP1BB, GSR, ITGB3, NP
1.0000	Inf	ACTB
1.0000	Inf	ITGB1
1.0000	Inf	MYH9
1.0000	Inf	PPIA
1.0000	Inf	ACTB
1.0000	Inf	ILK
1.0000	Inf	ILK
1.0000	Inf	CD36
1.0000	Inf	SRI
1.0000	Inf	ITGB1
1.0000	Inf	CDC42
1.0000	Inf	CDC42
1.0000	Inf	MYH9
1.0000	Inf	MYH9
1.0000	Inf	PRDX2
1.0000	Inf	ILK
1.0000	Inf	FLNA
1.0000	Inf	ITGB1
1.0000	Inf	ITGB1
1.0000	Inf	JUP
1.0000	Inf	HSP90AA1
1.0000	Inf	LBP
1.0000	Inf	PRDX2
1.0000	Inf	LBP
1.0000	Inf	PRDX2
1.0000	Inf	HLA-A
1.0000	Inf	HLA-A
1.0000	Inf	HSP90AA1
1.0000	Inf	CDC42
1.0000	Inf	CDC42
1.0000	Inf	ACTB
1.0000	Inf	HNRNPA2B1
1.0000	Inf	FLNA
1.0000	Inf	KANSL3
1.0000	Inf	STOM
1.0000	Inf	YWHAH
1.0000	Inf	YWHAH
1.0000	Inf	SRI
1.0000	Inf	CDC42
1.0000	Inf	RTN4
1.0000	Inf	RTN4
1.0000	Inf	HSP90AA1
1.0000	Inf	CDC42
1.0000	Inf	ACTB

1.0000	Inf	CDC42
1.0000	Inf	ITGB1
1.0000	Inf	FLNA
1.0000	Inf	FLNA
1.0000	Inf	CD9
1.0000	Inf	MYH9
1.0000	Inf	RTN4
1.0000	Inf	ITGB1
1.0000	Inf	ITGB1
1.0000	Inf	YWHAZ
1.0000	Inf	YWHAH
1.0000	Inf	KANSL3
1.0000	Inf	ITGB1
1.0000	Inf	ITGB1
1.0000	Inf	ITGB1
1.0000	Inf	RAB1B
1.0000	Inf	ITGB1
1.0000	Inf	SRI
1.0000	Inf	ITGB1
1.0000	Inf	QSOX1
1.0000	Inf	CD9
1.0000	Inf	ACTB
1.0000	Inf	HSP90AA1
1.0000	Inf	HSP90AA1
1.0000	Inf	HSP90AA1
1.0000	Inf	ITGA6
1.0000	Inf	ITGB1
1.0000	Inf	CD36
1.0000	Inf	CPN1
1.0000	Inf	CD36
1.0000	Inf	CD36
1.0000	Inf	CD36
1.0000	Inf	CD36
1.0000	Inf	CD36
1.0000	Inf	CD36
1.0000	Inf	ACTB
1.0000	Inf	CDC42
1.0000	Inf	CDC42
1.0000	Inf	CD9
1.0000	Inf	CD9
1.0000	Inf	ACTB
1.0000	Inf	SLC2A3
1.0000	Inf	ACTB
1.0000	Inf	CD36
1.0000	Inf	LBP
1.0000	Inf	QSOX1
1.0000	Inf	DSG1

1.0000	Inf	FLNA
1.0000	Inf	KANSL3
1.0000	Inf	KANSL3
1.0000	Inf	PPIA
1.0000	Inf	RAB1B
1.0000	Inf	PPIA
1.0000	Inf	CANX
1.0000	Inf	ACTB
1.0000	Inf	ITGB1
1.0000	Inf	ILK
1.0000	Inf	RTN4
1.0000	Inf	RTN4
1.0000	Inf	ITGB1
1.0000	Inf	ITGB1
1.0000	Inf	HSP90AA1
1.0000	Inf	HSP90AA1
1.0000	Inf	RTN4
1.0000	Inf	ITGB1
1.0000	Inf	FSCN1
1.0000	Inf	FSCN1
1.0000	Inf	FSCN1
1.0000	Inf	MYL6
1.0000	Inf	ACTB
1.0000	Inf	PRDX1
1.0000	Inf	MYH9
1.0000	Inf	CDC42
1.0000	Inf	ITGB1
1.0000	Inf	CD36
1.0000	Inf	ILK
1.0000	Inf	ILK
1.0000	Inf	ILK
1.0000	Inf	RTN4
1.0000	Inf	RTN4
1.0000	Inf	ITGB1
1.0000	Inf	CD9
1.0000	Inf	HNRNPA2B1
1.0000	Inf	KANSL3
1.0000	Inf	KANSL3
1.0000	Inf	KANSL3
1.0000	Inf	KANSL3
1.0000	Inf	FLNA
1.0000	Inf	FLNA
1.0000	Inf	CDC42
1.0000	Inf	CDC42
1.0000	Inf	CDC42
1.0000	Inf	CDC42
1.0000	Inf	YWHAH

1.0000	Inf	PRDX2
1.0000	Inf	PPIA
1.0000	Inf	KANSL3
1.0000	Inf	CD9
1.0000	Inf	CD36
1.0000	Inf	RAP1B
1.0000	Inf	CDC42
1.0000	Inf	LBP
1.0000	Inf	CD36
1.0000	Inf	CAMP
1.0000	Inf	FSCN1
1.0000	Inf	FSCN1
1.0000	Inf	FSCN1
1.0000	Inf	LBP
1.0000	Inf	CD36
1.0000	Inf	CD36
1.0000	Inf	MYH9
1.0000	Inf	RAP1B
1.0000	Inf	ITGB1
1.0000	Inf	CD36
1.0000	Inf	MYL6
1.0000	Inf	SRI
1.0000	Inf	RTN4
1.0000	Inf	RTN4
1.0000	Inf	RTN4
1.0000	Inf	CD9
1.0000	Inf	RTN4
1.0000	Inf	RTN4
1.0000	Inf	RTN4
1.0000	Inf	CD36
1.0000	Inf	CD36
1.0000	Inf	PPIA
1.0000	Inf	KANSL3
1.0000	Inf	CANX
1.0000	Inf	ITGB1
1.0000	Inf	F11R
1.0000	Inf	CDC42
1.0000	Inf	HNRNPA2B1
1.0000	Inf	ITGB1
1.0000	Inf	FLNA
1.0000	Inf	CD36
1.0000	Inf	F11R
1.0000	Inf	HLA-A
1.0000	Inf	HLA-A
1.0000	Inf	SRI
1.0000	Inf	SRI
1.0000	Inf	ITGA6

1.0000	Inf	PPIA
1.0000	Inf	HLA-A
1.0000	Inf	MYH9
1.0000	Inf	RTN4
1.0000	Inf	CDC42
1.0000	Inf	ITGB1
1.0000	Inf	PPIA
1.0000	Inf	CTSD
1.0000	Inf	HSP90AA1
1.0000	Inf	CD9
1.0000	Inf	LBP
1.0000	Inf	CD36
1.0000	Inf	SRI
1.0000	Inf	YWHAH
1.0000	Inf	SRI
1.0000	Inf	PRDX2
1.0000	Inf	FLNA
1.0000	Inf	FLNA
1.0000	Inf	ITGB1
1.0000	Inf	HSP90AA1
1.0000	Inf	HSP90AA1
1.0000	Inf	SRI
1.0000	Inf	CD9
1.0000	Inf	KANSL3
1.0000	Inf	CDC42
1.0000	Inf	CDC42
1.0000	Inf	CDC42
1.0000	Inf	ITGA6
1.0000	Inf	HSP90AA1
1.0000	Inf	ACTB
1.0000	Inf	KANSL3
1.0000	Inf	KANSL3
1.0000	Inf	KANSL3
1.0000	Inf	CD36
1.0000	Inf	CD36
1.0000	Inf	FLNA
1.0000	Inf	ACTB
1.0000	Inf	ACTB
1.0000	Inf	CD9
1.0000	Inf	HSP90AA1
1.0000	Inf	S100A9
1.0000	Inf	S100A9
1.0000	Inf	ACTB
1.0000	Inf	ITGB1
1.0000	Inf	PRDX2
1.0000	Inf	PRDX2
1.0000	Inf	ACTB

1.0000	Inf	ACTB
1.0000	Inf	TSPAN14
1.0000	Inf	HSP90AA1
1.0000	Inf	SRI
1.0000	Inf	HSP90AA1
1.0000	Inf	CDC42
1.0000	Inf	LBP
1.0000	Inf	SRI
1.0000	Inf	HSP90AA1
1.0000	Inf	F11R
1.0000	Inf	ITGB1
1.0000	Inf	HLA-A
1.0000	Inf	HLA-A
1.0000	Inf	HLA-A
1.0000	Inf	CD36
1.0000	Inf	RAP1B
1.0000	Inf	AACS
1.0000	Inf	AACS
1.0000	Inf	CLTA
1.0000	Inf	YWHAZ
1.0000	Inf	YWHAZ
1.0000	Inf	CDC42
1.0000	Inf	CDC42
1.0000	Inf	ITGB1
1.0000	Inf	ITGB1
1.0000	Inf	CDC42
1.0000	Inf	CDC42
1.0000	Inf	FLNA
1.0000	Inf	FLNA
1.0000	Inf	ITGA6
1.0000	Inf	ILK
1.0000	Inf	RTN4
1.0000	Inf	YWHAH
1.0000	Inf	CDC42
1.0000	Inf	RTN4
1.0000	Inf	RTN4
1.0000	Inf	YWHAH
1.0000	Inf	HSP90AA1
1.0000	Inf	HSP90AA1
1.0000	Inf	PECAM1
1.0000	Inf	HSP90AA1
1.0000	Inf	CD36
1.0000	Inf	JUP
1.0000	Inf	F11R
1.0000	Inf	ITGB1
1.0000	Inf	HSP90AA1
1.0000	Inf	HSP90AA1

1.0000	Inf	CD36
1.0000	Inf	RTN4
1.0000	Inf	ITGB1
1.0000	Inf	MYH9
1.0000	Inf	ACTB
1.0000	Inf	ACTB
1.0000	Inf	MYH9
1.0000	Inf	KANSL3
1.0000	Inf	KANSL3
1.0000	Inf	KANSL3
1.0000	Inf	ITGB1
1.0000	Inf	RAP1B
1.0000	Inf	ITGB1
1.0000	Inf	ACTB
1.0000	Inf	ACTB
1.0000	Inf	ACTB
1.0000	Inf	CDC42
1.0000	Inf	FLNA
1.0000	Inf	CDC42
1.0000	Inf	YWHAH
1.0000	Inf	ITGB1
1.0000	Inf	ITGB1
1.0000	Inf	ACTB
1.0000	Inf	ITGB1
1.0000	Inf	ITGB1
1.0000	Inf	ITGB1
1.0000	Inf	ITGB1
1.0000	Inf	ITGB1
1.0000	Inf	ITGB1
1.0000	Inf	ITGB1
1.0000	Inf	CD36
1.0000	Inf	CD36
1.0000	Inf	SRI
1.0000	Inf	CD36
1.0000	Inf	CD36
1.0000	Inf	ITGA6
1.0000	Inf	LBP
1.0000	Inf	LBP
1.0000	Inf	LBP
1.0000	Inf	FLNA
1.0000	Inf	RTN4
1.0000	Inf	PPIA
1.0000	Inf	FLNA
1.0000	Inf	ITGB1
1.0000	Inf	CDC42
1.0000	Inf	CDC42
1.0000	Inf	HSP90AA1

1.0000	Inf	MYH9
1.0000	Inf	MYH9
1.0000	Inf	MYH9
1.0000	Inf	CDC42
1.0000	Inf	CDC42
1.0000	Inf	ILK
1.0000	Inf	RTN4
1.0000	Inf	CD36
1.0000	Inf	ITGB1
1.0000	Inf	ITGB1
1.0000	Inf	CDC42
1.0000	Inf	ILK
1.0000	Inf	CD36
1.0000	Inf	CD36
1.0000	Inf	ILK
1.0000	Inf	SRI
1.0000	Inf	RTN4
1.0000	Inf	CDC42
1.0000	Inf	CDC42
1.0000	Inf	CDC42
1.0000	Inf	ILK
1.0000	Inf	ILK
1.0000	Inf	RTN4
1.0000	Inf	PPIA
1.0000	Inf	PPIA
1.0000	Inf	CASP14
1.0000	Inf	ACTB
1.0000	Inf	CD36
1.0000	Inf	CD36
1.0000	Inf	CD36
1.0000	Inf	FLNA
1.0000	Inf	FLNA
1.0000	Inf	ACTB
1.0000	Inf	HNRNPA2B1
1.0000	Inf	PPIA
1.0000	Inf	CD36
1.0000	Inf	CD36
1.0000	Inf	CAMP
1.0000	Inf	SRI
1.0000	Inf	SRI
1.0000	Inf	SRI
1.0000	Inf	SRI
1.0000	Inf	CD36
1.0000	Inf	FLNA
1.0000	Inf	JUP
1.0000	Inf	JUP
1.0000	Inf	JUP

1.0000	Inf	ITGB1
1.0000	Inf	CD36
1.0000	Inf	CD36
1.0000	Inf	ILK
1.0000	Inf	ILK
1.0000	Inf	FLNA
1.0000	Inf	FLNA
1.0000	Inf	FLNA
1.0000	Inf	FLNA
1.0000	Inf	PECAM1
1.0000	Inf	PECAM1
1.0000	Inf	ILK
1.0000	Inf	ILK
1.0000	Inf	ILK
1.0000	Inf	ILK
1.0000	Inf	ILK
1.0000	Inf	ILK
1.0000	Inf	ILK
1.0000	Inf	CDC42
1.0000	Inf	CD36
1.0000	Inf	ACTB
1.0000	Inf	MYH9
1.0000	Inf	QSOX1
1.0000	Inf	YWHAH
1.0000	Inf	FLNA
1.0000	Inf	FLNA
1.0000	Inf	FLNA
1.0000	Inf	FLNA
1.0000	Inf	CDC42
1.0000	Inf	CDC42
1.0000	Inf	FLNA
1.0000	Inf	FSCN1
1.0000	Inf	YWHAZ
1.0000	Inf	RTN4
1.0000	Inf	RTN4
1.0000	Inf	YWHAZ
1.0000	Inf	CDC42
1.0000	Inf	STOM
1.0000	Inf	STOM
1.0000	Inf	CD36
1.0000	Inf	CD9
1.0000	Inf	ACTB
1.0000	Inf	CDC42
1.0000	Inf	F11R
1.0000	Inf	CD36
1.0000	Inf	CASP14
1.0000	Inf	CAVIN2

1.0000	Inf	SRI
1.0000	Inf	ACTB
1.0000	Inf	FLNA
1.0000	Inf	ITGB1
1.0000	Inf	CD36
1.0000	Inf	FLNA
1.0000	Inf	FLNA
1.0000	Inf	ACTB
1.0000	Inf	ACTB
1.0000	Inf	FLNA
1.0000	Inf	FLNA
1.0000	Inf	RTN4
1.0000	Inf	CD36
1.0000	Inf	CD36
1.0000	Inf	CD36
1.0000	Inf	CD36
1.0000	Inf	CD36
1.0000	Inf	ITGB1
1.0000	Inf	CD36
1.0000	Inf	ITGB1
1.0000	Inf	PECAM1
1.0000	Inf	ACTB
1.0000	Inf	KANSL3
1.0000	Inf	CD36
1.0000	Inf	CD36
1.0000	Inf	CD36
1.0000	Inf	MYH9
1.0000	Inf	CD36
1.0000	Inf	SRI
1.0000	Inf	RTN4
1.0000	Inf	ACTB
1.0000	Inf	FLNA
1.0000	Inf	F11R
1.0000	Inf	AACS
1.0000	Inf	AACS
1.0000	Inf	STOM
1.0000	Inf	ITGB1
1.0000	Inf	ACTB
1.0000	Inf	PPIA
1.0000	Inf	AACS
1.0000	Inf	F11R
1.0000	Inf	ACTB
1.0000	Inf	ITGB1
1.0000	Inf	ACTB
1.0000	Inf	PPIA
1.0000	Inf	PPIA
1.0000	Inf	HSP90AA1

1.0000	Inf	HSP90AA1
1.0000	Inf	RAB1B
1.0000	Inf	RAB1B
1.0000	Inf	MYH9
1.0000	Inf	FSCN1
1.0000	Inf	F11R
1.0000	Inf	PPIA
1.0000	Inf	GP1BA
1.0000	Inf	GP1BA
1.0000	Inf	FLNA
1.0000	Inf	FLNA
1.0000	Inf	CD36
1.0000	Inf	SRI
1.0000	Inf	HSP90AA1
1.0000	Inf	PKM
1.0000	Inf	ITGB1
1.0000	Inf	CD36
1.0000	Inf	MYH9
1.0000	Inf	MYH9
1.0000	Inf	MYH9
1.0000	Inf	MYH9
1.0000	Inf	ITGB1
1.0000	Inf	ACTB
1.0000	Inf	CD36
1.0000	Inf	FLNA
1.0000	Inf	FLNA
1.0000	Inf	CD36
1.0000	Inf	CD36
1.0000	Inf	ACTB
1.0000	Inf	HSP90AA1
1.0000	Inf	RTN4
1.0000	Inf	RTN4
1.0000	Inf	RTN4
1.0000	Inf	RTN4
1.0000	Inf	HNRNPA2B1
1.0000	Inf	HNRNPA2B1
1.0000	Inf	ACTB
1.0000	Inf	CD36
1.0000	Inf	HNRNPA2B1
1.0000	Inf	RTN4
1.0000	Inf	ACTB
1.0000	Inf	ACTB
1.0000	Inf	RAP1B
1.0000	Inf	CD36
1.0000	Inf	RTN4
1.0000	Inf	ILK
1.0000	Inf	ITGB1

1.0000	Inf	CDC42
1.0000	Inf	ITGB1
1.0000	Inf	RAP1B
1.0000	Inf	CD36
1.0000	Inf	CD36
1.0000	Inf	HLA-A
1.0000	Inf	HLA-A
1.0000	Inf	HLA-A
1.0000	Inf	HLA-A
1.0000	Inf	SRI
1.0000	Inf	SRI
1.0000	Inf	ACTB
1.0000	Inf	ACTB
1.0000	Inf	RAB1B
1.0000	Inf	CDC42
1.0000	Inf	F11R
1.0000	Inf	ITGB1
1.0000	Inf	ACTB
1.0000	Inf	FLNA
1.0000	Inf	FLNA
1.0000	Inf	HLA-A
1.0000	Inf	HLA-A
1.0000	Inf	CDC42
1.0000	Inf	RAB1B
1.0000	Inf	CANX
1.0000	Inf	HLA-A
1.0000	Inf	MYH9
1.0000	Inf	CYB5R3
1.0000	Inf	JUP
1.0000	Inf	CLTA
1.0000	Inf	HNRNPA2B1
1.0000	Inf	JUP
1.0000	Inf	JUP
1.0000	Inf	MYL6
1.0000	Inf	ACTB
1.0000	Inf	ACTB
1.0000	Inf	CDC42
1.0000	Inf	CLTA
1.0000	Inf	CLTA
1.0000	Inf	CLTA
1.0000	Inf	CLTA
1.0000	Inf	CLTA
1.0000	Inf	CLTA
1.0000	Inf	QSOX1
1.0000	Inf	SRI
1.0000	Inf	CLTA
1.0000	Inf	QSOX1

1.0000	Inf	FLNA
1.0000	Inf	MYH9
1.0000	Inf	RAB1B
1.0000	Inf	ITGB1
1.0000	Inf	ITGB1
1.0000	Inf	ITGB1
1.0000	Inf	ITGB1
1.0000	Inf	ITGB1
1.0000	Inf	ITGB1
1.0000	Inf	ITGB1
1.0000	Inf	ITGB1
1.0000	Inf	ITGB1
1.0000	Inf	ACTB
1.0000	Inf	ACTB
1.0000	Inf	ITGB1
1.0000	Inf	CLTA
1.0000	Inf	CLTA
1.0000	Inf	ACTB
1.0000	Inf	HSP90AA1
1.0000	Inf	HSP90AA1
1.0000	Inf	ACTB
1.0000	Inf	CANX
1.0000	Inf	FSCN1
1.0000	Inf	KANSL3
1.0000	Inf	CLTA
1.0000	Inf	CANX
1.0000	Inf	MYH9
1.0000	Inf	ACTB
1.0000	Inf	MYH9
1.0000	Inf	HLA-A
1.0000	Inf	CLTA
1.0000	Inf	ACTB
1.0000	Inf	ACTB
1.0000	Inf	JUP
1.0000	Inf	JUP
1.0000	Inf	HSP90AA1
1.0000	Inf	ITGB1
1.0000	Inf	ACTB
1.0000	Inf	MYH9
1.0000	Inf	HSP90AA1
1.0000	Inf	YWHAZ
1.0000	Inf	RTN4
1.0000	Inf	CLTA
1.0000	Inf	CLTA
1.0000	Inf	CLTA
1.0000	Inf	ACTB
1.0000	Inf	CANX

1.0000	Inf	CANX
1.0000	Inf	CANX
1.0000	Inf	CDC42
1.0000	Inf	CANX
1.0000	Inf	CANX
1.0000	Inf	CANX
1.0000	Inf	ACTB
1.0000	Inf	CLTA
1.0000	Inf	CLTA
1.0000	Inf	ACTB
1.0000	Inf	ACTB
1.0000	Inf	CYB5R3
1.0000	Inf	ACTB
1.0000	Inf	GP1BA
1.0000	Inf	GP1BA
1.0000	Inf	HSP90AA1
1.0000	Inf	HSP90AA1
1.0000	Inf	PPIA
1.0000	Inf	LYZ
1.0000	Inf	CYB5R3
1.0000	Inf	ANPEP
1.0000	Inf	CPN1
1.0000	Inf	CPN1
1.0000	Inf	CTSD
1.0000	Inf	GAPDH
1.0000	Inf	LYZ
1.0000	Inf	ILK
1.0000	Inf	PKM
1.0000	Inf	PKM
1.0000	Inf	GP1BA
1.0000	Inf	CD36
1.0000	Inf	SLC2A3
1.0000	Inf	CD36
1.0000	Inf	YWHAG
1.0000	Inf	ANXA11
1.0000	Inf	GP1BA
1.0000	Inf	GP1BA
1.0000	Inf	CD36
1.0000	Inf	CD36
1.0000	Inf	DSG1
1.0000	Inf	PPIA
1.0000	Inf	AACS
1.0000	Inf	CYB5R3
1.0000	Inf	CYB5R3
1.0000	Inf	CYB5R3
1.0000	Inf	GAPDH
1.0000	Inf	LYZ

1.0000	Inf	PPIA
1.0000	Inf	AACS
1.0000	Inf	AACS
1.0000	Inf	YWHAH
1.0000	Inf	QSOX1
1.0000	Inf	QSOX1
1.0000	Inf	HNRNPF
1.0000	Inf	YWHAH
1.0000	Inf	HSP90AA1
1.0000	Inf	TLN1
1.0000	Inf	HSP90AA1
1.0000	Inf	GAPDH
1.0000	Inf	ACTB
1.0000	Inf	TLN1
1.0000	Inf	YWHAG
1.0000	Inf	AACS
1.0000	Inf	CDC42
1.0000	Inf	HLA-A
1.0000	Inf	HSP90AA1
1.0000	Inf	PKM
1.0000	Inf	YWHAG
1.0000	Inf	PKM
1.0000	Inf	CDC42
1.0000	Inf	MYL12B
1.0000	Inf	CDC42
1.0000	Inf	HSP90AA1
1.0000	Inf	HSP90AA1
1.0000	Inf	HSP90AA1
1.0000	Inf	HSP90AA1
1.0000	Inf	HSP90AA1
1.0000	Inf	CDC42
1.0000	Inf	CDC42
1.0000	Inf	FLNA
1.0000	Inf	FLNA
1.0000	Inf	YWHAH
1.0000	Inf	GAPDH
1.0000	Inf	HLA-A
1.0000	Inf	HLA-A
1.0000	Inf	HNRNPA2B1
1.0000	Inf	GAPDH
1.0000	Inf	PF4V1
1.0000	Inf	CD36
1.0000	Inf	CYB5R3
1.0000	Inf	CDC42
1.0000	Inf	CDC42
1.0000	Inf	LYZ
1.0000	Inf	HLA-A

1.0000	Inf	CTSD
1.0000	Inf	ANPEP
1.0000	Inf	HSP90AA1
1.0000	Inf	GSDMA
1.0000	Inf	LBP
1.0000	Inf	CD36
1.0000	Inf	LBP
1.0000	Inf	CASP14
1.0000	Inf	HNRNPA2B1
1.0000	Inf	CASP14
1.0000	Inf	HNRNPA2B1
1.0000	Inf	ITGB1
1.0000	Inf	ITGB1
1.0000	Inf	CYB5R3
1.0000	Inf	ACTB
1.0000	Inf	ACTB
1.0000	Inf	ACTB
1.0000	Inf	JUP
1.0000	Inf	ILK
1.0000	Inf	YWHAZ
1.0000	Inf	YWHAZ
1.0000	Inf	CD36
1.0000	Inf	CD36
1.0000	Inf	PPIA
1.0000	1.28	CAMP, CCL5, CD226, CD36, CD47, CDC42, CYBB, DCD, DDX60L, DHX9, ENDOD1, F11R, FC
1.0000	1.28	ACTB, ALDOA, ANXA2, ATIC, ATP5F1B, CCT2, CCT4, CCT6A, CCT7, CCT8, CD36, DHX9, EIF5
1.0000	1.28	ACTB, ALDOA, ANXA2, ATIC, ATP5F1B, CCT2, CCT4, CCT6A, CCT7, CCT8, CD36, DHX9, EIF5
1.0000	1.32	ANK1, ANPEP, ANXA5, B2M, BCAP31, CANX, CD226, CD36, CD59, CD9, DSG1, EEF1A1, FC
1.0000	1.46	ACTB, B2M, CD36, F11R, FLG2, HRNR, INPP5D, ITGB1, ITGB3, KRT1, KRT16, LTF, LYZ, PECA
1.0000	1.46	ANPEP, ANXA5, B2M, CD226, CD36, CD59, CD9, FCER1G, FCN1, FLOT1, GP1BA, GSR, ITGA
1.0000	1.46	ACTB, ARPC5, CDC42, CFL1, EEF1A1, FLOT1, FSCN1, ILK, ITGB1, ITGB3, MYADM, MYH9, P
1.0000	1.28	AACS, ACTB, ADAM10, ADH5, ALDOA, ANK1, ANPEP, ANXA2, ANXA5, ANXA7, ARL6IP5, AI
1.0000	1.22	AACS, ACTB, ADAM10, ANK1, ANTXR2, ANXA11, ANXA2, ARL6IP5, ATP2A3, ATP5F1B, B2M
1.0000	1.26	AACS, ACTB, ADAM10, ALDOA, ANK1, ANPEP, ANXA2, ANXA5, ANXA7, ARL6IP5, ARPC5, A
1.0000	1.50	ACTB, CD151, CDC42, FLG2, FLNA, ILK, ITGB1, KRT10, KRT16, KRT17, KRT6A, MSN, PFN1,
1.0000	1.50	ALDOA, CCT2, CCT4, CCT7, CCT8, CD226, CD36, CD9, FCN1, LBP, MSN, PECAM1, RTN4, TC
1.0000	1.50	ACTB, ARL6IP5, CALM3, CLIC1, CYBB, FLNA, G6PD, ITGB1, ITGB3, SRI, STOM, THBS1, TME
1.0000	1.50	ACTB, B2M, CCL5, CD226, CD47, FLNA, GP1BA, GP1BB, HLA-A, HSPD1, INPP5D, LBP, PLEK
1.0000	1.50	ATP2A3, ATP5F1B, BCAP31, CALM3, CCL5, FLNA, GP1BA, GP1BB, ITGB3, RTN4, S100A8, S
1.0000	1.50	ACTB, ALDOA, B2M, F11R, INPP5D, ITGB1, ITGB3, KRT1, LTF, LYZ, PECAM1, PRDX1, SLC2A
1.0000	1.56	ALDOA, CALM3, DSP, ENO1, FLNA, G6PD, HSP90AA1, JUP, MYL6, RHOA, SOD1, SRI, TPM4
1.0000	1.56	BCAP31, HSP90AA1, HSPA8, HSPD1, M6PR, RAC2, RPL11, STOM, UBE2L3, YWHAB, YWHA
1.0000	1.56	ANXA2, B2M, CANX, CD36, CD9, CLTA, FCER1G, FLOT1, ITGB1, ITGB3, LRP1, M6PR, TFRC
1.0000	1.56	ANXA2, B2M, CDC42, FLNA, G6PD, GP1BA, GP1BB, HSPA1B, HSPA9, INPP5D, LTF, MYH9,
1.0000	1.56	ANPEP, ANXA2, CLTA, CTSD, EEF1A1, FLOT1, GNAQ, HSPA8, LRBA, LRP1, M6PR, RAP1B, S
1.0000	1.56	CD36, CFL1, EEF1A1, FSCN1, HSP90AA1, ITGB1, ITGB3, MSN, PLEK, PTPRJ, RHOA, SPTBN1
1.0000	1.56	ANPEP, ANXA2, CLTA, CTSD, EEF1A1, FLOT1, GNAQ, HSPA8, LRBA, LRP1, M6PR, RAP1B, S

1.0000	1.56	ANXA11, ANXA2, ANXA5, ANXA7, CAVIN2, GSDMA, HSPA8, OSBPL2, PFN1, SPTBN1, THBS
1.0000	1.20	AACS, ACTB, ADAM10, ANK1, ANTXR2, ANXA11, ANXA2, ARL6IP5, ATP2A3, ATP5F1B, B2M
1.0000	1.21	ACTB, ADAM10, ADH5, ANXA5, ARL6IP5, ATIC, ATP5F1B, B2M, BCAP31, CALM3, CAMP, C
1.0000	1.33	ADAM10, ANXA2, CCL5, CD226, CD36, CD47, CD9, DHX9, FCN1, FLNA, GP1BA, GSTP1, HL
1.0000	1.33	ACTB, ADAM10, CALM3, CAVIN2, CD226, CDC42, DSP, EEF1A1, EEF2, FLNA, GSTP1, HINT1
1.0000	1.24	ACTB, ANXA2, CCT2, CCT4, CCT6A, CCT7, CCT8, CD36, CSDE1, DAZAP1, DHX9, EIF5A, ENC
1.0000	1.24	ACTB, ALDOA, ANXA2, ATIC, ATP5F1B, CCT2, CCT4, CCT6A, CCT7, CCT8, CD36, CYB5R3, D
1.0000	1.20	ACTB, ANXA2, ARL6IP5, ATP2A3, BCAP31, CALM3, CAMP, CCL5, CCT2, CCT4, CCT6A, CCT7
1.0000	1.66	ATP2A3, BCAP31, CALM3, CCL5, FLNA, GP1BA, GP1BB, ITGB3, SRI, YWHAE
1.0000	1.66	CDC42, CFL1, F11R, FLOT1, G3BP1, ITGB1, RAB33B, RAC2, RAP1B, RHOA
1.0000	1.66	CDC42, CFL1, F11R, FLOT1, G3BP1, ITGB1, RAB33B, RAC2, RAP1B, RHOA
1.0000	1.66	ADAM10, CCL5, FCER1G, LBP, PF4V1, PPIA, RAC2, S100A8, S100A9, THBS1
1.0000	1.66	CD36, FSCN1, HSP90AA1, HSPA1B, MSN, PFN1, PLEK, RAP1B, RHOA, TFRC
1.0000	1.66	ACTB, ADAM10, CDC42, CFL1, FLNA, HSPA8, PFN1, RHOA, TUBB, YWHAZ
1.0000	1.66	ATP2A3, BCAP31, CALM3, CCL5, FLNA, GP1BA, GP1BB, ITGB3, SRI, YWHAE
1.0000	1.66	ADAM10, CCL5, FCER1G, LBP, PF4V1, PPIA, RAC2, S100A8, S100A9, THBS1
1.0000	1.66	ADH5, CD36, GSR, GSTP1, PRDX1, PRDX2, PRDX6, PTGS1, S100A9, SOD1
1.0000	1.66	ADH5, CD36, GSR, GSTP1, PRDX1, PRDX2, PRDX6, PTGS1, S100A9, SOD1
1.0000	1.66	ADAM10, CAMP, CYBB, ITGB1, ITGB3, JUP, KRT1, PGK1, PKM, THBS1
1.0000	1.66	ADH5, CD36, GSR, GSTP1, PRDX1, PRDX2, PRDX6, PTGS1, S100A9, SOD1
1.0000	1.66	ACTB, ARPC5, CFL1, FLOT1, FSCN1, ILK, ITGB1, ITGB3, RAC2, RHOA
1.0000	1.66	ADAM10, CD36, CD47, CD53, CD59, CYBB, PTPRJ, SLC2A3, STOM, TSPAN14
1.0000	1.66	CYBB, H2AC20, H2BC21, ITGB1, KRT1, KRT10, SRI, TPM4, YWHAE, YWHAH
1.0000	1.26	ACTB, ADAM10, ALDOA, CALM3, CAMP, CCL5, CD36, CDC42, EEF1A1, ENO1, FLOT1, GAP
1.0000	1.26	ADAM10, ANPEP, ANTXR2, ANXA2, ANXA5, ATP5F1B, B2M, CD151, CD226, CD36, CD47,
1.0000	1.33	ACTB, ADAM10, B2M, CAMP, CCL5, CD151, CD226, CD47, FCER1G, HLA-A, HSPD1, INPP5
1.0000	1.86	ANXA11, CALM3, CDC42, CFL1, MYH9, RHOA, SPTBN1
1.0000	1.86	CD36, FLOT1, HSPD1, LBP, LTF, RTN4, UBQLN1
1.0000	1.86	F11R, MSN, MYADM, PECAM1, RAP1B, RHOA, VCL
1.0000	1.86	CD36, CYBB, GNAI2, GSTP1, PRDX1, PRDX2, SOD1
1.0000	1.86	FLNA, HNRNPU, HSPA1B, MYH9, RHOA, TUBB, TUBB1
1.0000	1.86	CD36, FLNA, GAPDH, GSTP1, LTF, RHOA, TFRC
1.0000	1.86	ALDOA, F11R, MSN, MYH9, MYL12B, RAC2, RHOA
1.0000	1.86	ADAM10, ITGB1, MATR3, MYH9, NASP, PFN1, RTN4
1.0000	1.86	ARL6IP5, CD36, CD47, ITGB1, PNPLA8, SLC2A1, SLC2A3
1.0000	1.86	CDC42, G3BP1, GNAQ, ILK, JUP, LRP1, RHOA
1.0000	1.86	CCL5, CD36, ITGB3, NCL, PECAM1, PKM, PTPRJ
1.0000	1.86	CCL5, CD36, ITGB3, NCL, PECAM1, PKM, PTPRJ
1.0000	1.86	ACTB, CALM3, CDC42, G6PD, RHOA, YWHAE, YWHAH
1.0000	1.86	ACTB, CALM3, CDC42, G6PD, RHOA, YWHAE, YWHAH
1.0000	1.86	CD47, CDC42, F11R, FLNA, PFN1, PLEK, RHOA
1.0000	1.86	BCAP31, CD9, DAZAP1, DSG1, ITGB1, KRT9, SOD1
1.0000	1.86	CCL5, FERMT3, ITGA2B, ITGA6, ITGB1, ITGB3, MMRN1
1.0000	1.86	ADAM10, ITGB1, MATR3, MYH9, NASP, PFN1, RTN4
1.0000	1.86	CD36, FLNA, GAPDH, GSTP1, LTF, RHOA, TFRC
1.0000	1.86	ATP5F1B, ITGB1, ITGB3, JUP, MYH9, RHOA, THBS1
1.0000	1.86	ANXA2, B2M, CD151, CD36, CDC42, FLOT1, LRP1

1.0000	1.86	ACTB, CALM3, CDC42, G6PD, RHOA, YWHAE, YWHAH
1.0000	1.86	AACS, ANXA2, GNAI2, PPIA, RAB27B, S100A8, SRI
1.0000	1.86	CD53, CD9, FLOT1, G6PD, HNRNPU, ITGB1, MYH9
1.0000	1.86	CALM3, GNAQ, HLA-A, HLA-B, JUP, LBP, PTPRJ
1.0000	1.86	ATP2A3, CALM3, DSP, FLNA, JUP, SRI, YWHAE
1.0000	1.86	CD36, FLOT1, HSPA1B, LBP, LTF, RTN4, UBQLN1
1.0000	1.86	CCL5, CD36, GNAI2, GSTP1, ITGB3, RAP1B, YWHAZ
1.0000	1.86	CCL5, CD36, GNAI2, GSTP1, ITGB3, RAP1B, YWHAZ
1.0000	1.86	CDC42, G3BP1, GNAQ, ILK, JUP, LRP1, RHOA
1.0000	1.86	ACTB, ADAM10, DSP, F11R, PECAM1, RAB27B, VCL
1.0000	1.86	CDC42, G3BP1, GNAQ, ILK, JUP, LRP1, RHOA
1.0000	1.86	DSP, FLOT1, ITGB1, JUP, SLC2A1, VCL, YWHAH
1.0000	1.86	BCAP31, CLTA, FLNA, HLA-A, M6PR, RAB10, RAB27B
1.0000	1.86	CDC42, FSCN1, ICAM2, ITGB1, ITGB3, MSN, SPN
1.0000	1.86	ANTXR2, FCER1G, GP1BA, GP1BB, M6PR, PECAM1, SPN
1.0000	1.86	CAVIN2, DSP, FLNA, HINT1, ITGB3, PLEK, YWHAG
1.0000	2.47	ILK, ITGB3, PECAM1, RHOA
1.0000	2.47	ILK, ITGB3, PECAM1, RHOA
1.0000	2.47	CD36, ITGB3, JUP, PTPRJ
1.0000	2.47	ITGB1, PKM, RHOA, THBS1
1.0000	2.47	CD226, HLA-A, HSPD1, PRDX2
1.0000	2.47	B2M, CD226, CD36, HLA-A
1.0000	2.47	B2M, CTSD, FCER1G, HLA-A
1.0000	2.47	B2M, CD226, CD36, HLA-A
1.0000	2.47	B2M, CD226, CD36, HLA-A
1.0000	2.47	AACS, CCL5, S100A8, SRI
1.0000	2.47	AACS, CCL5, S100A8, SRI
1.0000	2.47	ITGB1, LBP, LGALS3BP, SPN
1.0000	2.47	ACTB, CDC42, HNRNPU, RCC2
1.0000	2.47	HLA-A, HLA-B, LBP, PTPRJ
1.0000	2.47	EEF1A1, GAPDH, QSOX1, UBQLN1
1.0000	2.47	AACS, ADAM10, CCL5, SRI
1.0000	2.47	AACS, CCL5, S100A8, SRI
1.0000	2.47	ADH5, GAPDH, S100A8, S100A9
1.0000	2.47	HSP90AA1, ILK, TFRC, TNKS1BP1
1.0000	2.47	ADH5, GAPDH, S100A8, S100A9
1.0000	2.47	HSP90AA1, ILK, TFRC, TNKS1BP1
1.0000	2.47	CAMP, H2BC21, HLA-A, LTF
1.0000	2.47	B2M, CTSD, FCER1G, HLA-A
1.0000	2.47	CD9, MYADM, PRDX2, SPN
1.0000	2.47	DCD, GAPDH, LTF, LYZ
1.0000	2.47	CD36, DHX9, HSPD1, LBP
1.0000	2.47	ARL6IP5, GSTP1, PPIA, PRDX1
1.0000	2.47	FERMT3, PLEK, RAP1B, TLN1
1.0000	2.47	CCL5, CD9, F11R, MMRN1
1.0000	2.47	FLOT1, LBP, LTF, RTN4
1.0000	2.47	CDC42, G6PD, HNRNPU, ITGB1

1.0000	2.47	ACTB, CALM3, CANX, RAB27B
1.0000	2.47	AACS, CCL5, S100A8, SRI
1.0000	2.47	F11R, PECAM1, RHOA, VCL
1.0000	2.47	GSR, PRDX1, PRDX2, PRDX6
1.0000	2.47	ACTB, PRDX2, RHOA, SOD1
1.0000	2.47	CYBB, LBP, PRDX2, RAC2
1.0000	2.47	ARL6IP5, CD36, ITGB1, PNPLA8
1.0000	2.47	ACTB, CALM3, CANX, RAB27B
1.0000	2.47	CCL5, DHX9, PPIA, STOM
1.0000	2.47	CD9, INPP5D, PRDX2, SPN
1.0000	2.47	GP1BA, ITGB1, SELPLG, SPN
1.0000	2.47	ACTB, ATP2A3, B2M, SRI
1.0000	2.47	ARL6IP5, GSTP1, PPIA, PRDX1
1.0000	2.47	CAMP, DCD, GAPDH, KRT6A
1.0000	2.47	G3BP1, GNAQ, ILK, JUP
1.0000	2.47	G3BP1, GNAQ, ILK, JUP
1.0000	2.47	ARL6IP5, GSTP1, PPIA, PRDX1
1.0000	2.47	ILK, ITGB3, PECAM1, RHOA
1.0000	2.47	AACS, CCL5, S100A8, SRI
1.0000	2.47	ACTB, CDC42, HNRNPU, RCC2
1.0000	2.47	ACTB, CALM3, CANX, RAB27B
1.0000	2.47	ACTB, ITGB1, LRP1, SPTBN1
1.0000	2.47	CALM3, FLNA, G6PD, SRI
1.0000	2.47	ITGB1, LRP1, SPTBN1, STOM
1.0000	2.47	CANX, CYBB, PKM, RPS23
1.0000	2.47	ANPEP, BCAP31, CD59, RAB1B
1.0000	2.47	CLTA, FLNA, M6PR, RAB10
1.0000	2.47	CAVIN2, CD36, FLOT1, SLC2A1
1.0000	2.47	DSC1, DSG1, DSP, JUP
1.0000	2.47	ICAM2, ITGB1, MYH9, RHOA
1.0000	2.47	ICAM2, ITGB1, MYH9, RHOA
1.0000	2.47	ANXA2, CALM3, HSP90AA1, ITGB1
1.0000	2.47	ACTB, H2AC20, H2BC21, XRCC5
1.0000	2.47	CD9, CLTA, M6PR, TFRC
1.0000	2.47	BCAP31, CANX, HLA-A, HLA-B
1.0000	2.47	BCAP31, CANX, HLA-A, HLA-B
1.0000	2.47	ACTB, KANSL3, UBE2L3, XRCC5
1.0000	2.47	CAMP, HSPD1, LBP, LTF
1.0000	2.47	LTF, PF4V1, PPIA, THBS1
1.0000	2.47	ANXA11, ANXA2, ANXA5, ANXA7
1.0000	2.47	GAPDH, RCC2, S100A8, S100A9
1.0000	2.47	CCL5, ILK, PGK1, PKM
1.0000	2.47	CD36, ITGA6, ITGB3, THBS1
1.0000	2.47	CD36, ITGB1, ITGB3, THBS1
1.0000	2.47	HSP90AA1, HSPA1B, YWHAB, YWHAE
1.0000	2.47	PFN1, YWHAB, YWHAE, YWHAZ
1.0000	2.47	PFN1, YWHAB, YWHAE, YWHAZ

1.0000	1.19	ACTB, ANXA2, B2M, BCAP31, CALM3, CAMP, CCL5, CCT2, CCT4, CCT6A, CCT7, CCT8, CD3
1.0000	1.17	ACTB, ADAM10, ANXA2, ANXA7, ARL6IP5, ATP2A3, B2M, BCAP31, CALM3, CAMP, CCL5, (
1.0000	1.17	AACS, ACTB, ADAM10, ANXA2, ARL6IP5, ATP5F1B, B2M, BCAP31, CALM3, CAMP, CCL5, C
1.0000	1.33	CALM3, CAMP, CCL5, CD36, CDC42, ENO1, FLOT1, HSP90AA1, ILK, ITGA6, ITGB3, LTF, NP
1.0000	1.33	ANPEP, CAMP, CCL5, CDC42, CFL1, DCD, EEF1A1, F11R, FCN1, GAPDH, HSPA1B, HSPA8, H
1.0000	1.33	CALM3, CAMP, CCL5, CD36, CDC42, ENO1, FLOT1, HSP90AA1, ILK, ITGA6, ITGB3, LTF, NP
1.0000	1.33	ANXA2, ANXA5, B2M, CD9, G6PD, GNAI2, GP1BA, GSTP1, HSPA9, INPP5D, JUP, KRT1, LBP
1.0000	1.33	ACTB, ALDOA, ARPC5, CFL1, DHX9, EEF1A1, FERMT3, FLNA, FLOT1, FSCN1, HNRNPC, ILK,
1.0000	1.20	ACTB, ANXA2, CCL5, CCT2, CCT4, CCT6A, CCT7, CCT8, CD36, CD47, CSDE1, DHX9, EEF2, E
1.0000	1.17	ACTB, ADAM10, ADH5, ANXA11, ANXA5, ARL6IP5, ATIC, ATP5F1B, B2M, BCAP31, CALM3
1.0000	1.22	ACTB, ALDOA, ATP2A3, ATP5F1B, B2M, BCAP31, CALM3, CCL5, CD36, F11R, FLG2, FLNA,
1.0000	1.22	ADAM10, ADH5, ANPEP, ANTXR2, ANXA11, ANXA2, ANXA5, ANXA7, ATP2A3, CALM3, CA
1.0000	1.34	ANXA2, ATP2A3, B2M, CALM3, CCL5, FLNA, G6PD, GNAI2, GP1BA, GP1BB, ITGB1, ITGB3,
1.0000	1.34	ACTB, B2M, CCL5, CD151, CD47, FCER1G, HLA-A, HSPD1, INPP5D, ITGB1, MSN, MYH9, PR
1.0000	1.34	ADAM10, ATIC, B2M, CCL5, ENO1, F11R, FCER1G, FLNA, G6PD, GALE, HSP90AA1, JUP, M
1.0000	1.15	ACTB, ADAM10, ALDOA, ANPEP, ANXA2, ANXA7, ARPC5, ATIC, ATP5F1B, B2M, BCAP31, (
1.0000	1.25	ACTB, CALM3, CAMP, CCL5, CD36, CDC42, EEF1A1, FLOT1, GNAQ, GSTP1, HNRNPU, HSP
1.0000	1.15	ACTB, ANPEP, ANXA2, ANXA7, ATP5F1B, B2M, CASP14, CD36, CD53, CD9, CDC42, CFL1, (
1.0000	1.15	ACTB, ANPEP, ANXA2, ANXA7, ATP5F1B, B2M, CASP14, CD36, CD53, CD9, CDC42, CFL1, (
1.0000	1.19	ACTB, ANXA2, CD36, CSDE1, DAZAP1, DHX9, EIF5A, ENO1, FLNA, FLOT1, HINT1, HNRNPA
1.0000	1.15	ACTB, ADAM10, ADH5, ALDOA, ANPEP, ANXA2, ANXA5, ARL6IP5, ARPC5, ATIC, ATP2A3, /
1.0000	1.35	ANPEP, CCL5, EEF1A1, F11R, FCN1, HSPA1B, HSPA8, ITGB1, ITGB3, LTF, PCBP1, PPIA, RAB
1.0000	1.35	ACTB, B2M, CCL5, CD47, CD9, F11R, FERMT3, FLOT1, GP1BA, HLA-A, HSPD1, MMRN1, M
1.0000	1.35	AACS, ANK1, ANXA2, CALM3, CANX, CCL5, GNAI2, M6PR, MYH9, PLEK, PPIA, RAB10, RAB
1.0000	1.35	ACTB, CLTA, EEF1A1, ENO1, FLNA, FLOT1, FSCN1, MYADM, MYH9, MYL12B, NCL, PFN1, R
1.0000	1.35	ANXA2, CAVIN2, CD226, CD36, CTSD, FLOT1, GNAI2, INPP5D, ITGB1, LDHB, MYADM, PEC
1.0000	1.35	ANXA2, CAVIN2, CD226, CD36, CTSD, FLOT1, GNAI2, INPP5D, ITGB1, LDHB, MYADM, PEC
1.0000	1.15	AACS, ACTB, ADAM10, ANK1, ANTXR2, ANXA11, ANXA2, ARL6IP5, ARPC5, ATP2A3, ATP5
1.0000	1.25	ACTB, ARL6IP5, CALM3, CCL5, CD36, CPN1, CYBB, DHX9, DSG1, FLNA, FLOT1, G6PD, HNR
1.0000	1.14	ACTB, ADAM10, ANXA2, ANXA5, ARL6IP5, ATP5F1B, B2M, CALM3, CCL5, CD36, CD47, CD
1.0000	1.18	ACTB, ANXA5, ARL6IP5, ATP2A3, BCAP31, CASP14, CCL5, CFL1, CTSD, DHX9, EIF5A, ENO1
1.0000	1.20	CAMP, CCL5, CD226, CD36, CD47, CDC42, CYBB, DCD, DDX60L, DHX9, ENDOD1, FCER1G,
1.0000	1.38	ATP2A3, BCAP31, CALM3, CCL5, FLNA, GP1BA, GP1BB, ITGB3, S100A8, S100A9, SOD1, SR
1.0000	1.38	B2M, CALM3, CCL5, FLNA, G6PD, GNAI2, ITGB1, ITGB3, RHOA, SRI, STOM, UBQLN1, YWH
1.0000	1.38	ARL6IP5, CALM3, CLIC1, CYBB, FLNA, G6PD, ITGB1, ITGB3, SRI, STOM, TMEM109, UBQLN
1.0000	1.38	ACTB, ARL6IP5, BCAP31, CD36, DHX9, ENO1, FLNA, GSTP1, NPM1, PNPLA8, PPIA, PRDX1,
1.0000	1.17	ADAM10, ADH5, ANPEP, ANTXR2, ANXA11, ANXA2, ANXA5, ANXA7, ATP2A3, CALM3, CA
1.0000	1.12	ACTB, ANXA2, ARL6IP5, ATP2A3, BCAP31, CALM3, CAMP, CCL5, CCT2, CCT4, CCT6A, CCT
1.0000	1.41	B2M, CD226, CD59, DCD, GAPDH, HLA-A, HLA-B, LTF, LYZ, PRDX1, TUBB
1.0000	1.41	ADH5, CCL5, CD36, GSR, GSTP1, PRDX1, PRDX2, PRDX6, PTGS1, S100A9, SOD1
1.0000	1.41	ATP5F1B, ITGB1, ITGB3, JUP, KRT16, KRT2, MYH9, PFN1, RHOA, RTN4, THBS1
1.0000	1.41	ANXA2, BCAP31, DHX9, FLNA, HSP90AA1, JUP, MSN, RAC2, STOM, UBE2L3, YWHAE
1.0000	1.41	ARL6IP5, CD36, G6PD, GSR, HSPA1B, PNPLA8, PPIA, PRDX1, PRDX2, SOD1, UBQLN1
1.0000	1.41	ANPEP, F11R, FCN1, HSPA1B, ITGB1, ITGB3, KRT6A, PPIA, RPSA, SELPLG, TFRC
1.0000	1.41	CD36, DHX9, FLOT1, HSPA1B, LTF, NPM1, PPIA, PSMA6, S100A8, S100A9, TFRC
1.0000	1.41	CDC42, CFL1, HNRNPU, ITGB1, MYH9, NPM1, PLEK, RAB10, RAC2, RPS15, YWHAZ
1.0000	1.41	ANXA2, ATP2A3, CALM3, FLNA, G6PD, GP1BA, GP1BB, ITGB3, SRI, UBQLN1, YWHAE

1.0000	1.41	ATP5F1B, ITGB1, ITGB3, JUP, KRT16, KRT2, MYH9, PFN1, RHOA, RTN4, THBS1
1.0000	1.41	ATP5F1B, ITGB1, ITGB3, JUP, KRT16, KRT2, MYH9, PFN1, RHOA, RTN4, THBS1
1.0000	1.41	ACTB, B2M, CCL5, CD47, GP1BA, HLA-A, HSPD1, PRDX2, RHOA, SPN, TFRC
1.0000	1.41	CFL1, EEF1A1, FSCN1, ITGB1, ITGB3, MYADM, MYH9, PLEK, PTPRJ, RHOA, TLN1
1.0000	1.41	ANXA2, CCT2, CCT8, CTSD, CYB5R3, HRNR, HSP90AA1, HSPA8, LYZ, PRDX6, TUBB
1.0000	1.41	ACTB, ADAM10, ANXA2, FLOT1, JUP, MSN, MYH9, RAB10, TLN1, TNKS1BP1, VCL
1.0000	1.41	ANXA11, B2M, CD36, CDC42, CYBB, HLA-A, HLA-B, LTF, RAB10, RAC2, VIM
1.0000	1.41	ACTB, CALM3, CANX, CLTA, FLOT1, HSPA8, RAB10, RAB27B, RAB33B, SLC2A1, YWHAG
1.0000	1.41	ACTB, ADAM10, CANX, FLOT1, HSPA8, ITGB1, ITGB3, PFN1, RHOA, SPTBN1, YWHAZ
1.0000	1.14	ACTB, ANXA5, ARL6IP5, ATP2A3, BCAP31, CASP14, CCL5, CD36, CFL1, CTSD, DHX9, EIF5A,
1.0000	1.14	ARL6IP5, ATP2A3, BCAP31, CALM3, CCL5, CD36, CDC42, CFL1, EIF5A, ENO1, F11R, FLNA,
1.0000	1.24	ANXA2, ARL6IP5, ATP2A3, ATP5F1B, CALM3, CLIC1, CYBB, FLNA, G6PD, GP1BA, GP1BB, IT
1.0000	1.24	ANPEP, ANXA2, BCAP31, CANX, CPN1, CTSD, EEF1A1, FLNA, HINT1, HSP90AA1, HSPA1B, I
1.0000	1.18	ADAM10, ATP5F1B, CCL5, CD151, CD47, CD9, CD99, FERMT3, FLNA, GNAI2, GP1BA, ITGA
1.0000	1.18	ADAM10, ATP5F1B, CCL5, CD151, CD47, CD9, CD99, FERMT3, FLNA, GNAI2, GP1BA, ITGA
1.0000	1.18	ADAM10, ATP5F1B, CCL5, CD151, CD47, CD9, CD99, FERMT3, FLNA, GNAI2, GP1BA, ITGA
1.0000	1.48	ACTB, B2M, KRT1, LTF, LYZ, PRDX1, SLC2A1, SOD1
1.0000	1.48	F11R, KRT2, MSN, MYADM, PECAM1, RAP1B, VCL, VIM
1.0000	1.48	CALM3, DSP, ENO1, HSP90AA1, JUP, RHOA, SOD1, SRI
1.0000	1.48	ADAM10, ANXA2, FLOT1, FSCN1, ITGB1, ITGB3, LRP1, QSOX1
1.0000	1.48	CDC42, FERMT3, FLNA, ILK, ITGB3, MYADM, RCC2, RHOA
1.0000	1.48	ATP2A3, CALM3, DSP, FLNA, JUP, SOD1, SRI, YWHAE
1.0000	1.48	CCL5, CD151, HLA-A, MSN, PRDX2, RAC2, SPN, TFRC
1.0000	1.48	CD53, CD9, FLOT1, G6PD, HNRNPU, ITGB1, MYH9, PTBP1
1.0000	1.48	ADAM10, ANXA2, FLOT1, FSCN1, ITGB1, ITGB3, LRP1, QSOX1
1.0000	1.48	CDC42, DSP, FLOT1, G6PD, HNRNPU, ITGB1, MYL6, RHOA
1.0000	1.48	ACTB, ANXA2, CFL1, HNRNPA2B1, HNRNPU, MATR3, PSMA6, VIM
1.0000	1.48	ACTB, ANXA2, CFL1, HNRNPA2B1, HNRNPU, MATR3, PSMA6, VIM
1.0000	1.48	ANPEP, F11R, HSPA1B, ITGB1, ITGB3, RPSA, SELPLG, TFRC
1.0000	1.48	ADH5, ANPEP, CPN1, MATR3, S100A8, S100A9, SF3A3, SOD1
1.0000	1.48	GSR, GSTP1, PRDX1, PRDX2, PRDX6, PTGS1, S100A9, SOD1
1.0000	1.48	CLTA, ENO1, FLNA, HSP90AA1, PFN1, RCC2, SOD1, SPTBN1
1.0000	1.48	ANPEP, F11R, HSPA1B, ITGB1, ITGB3, RPSA, SELPLG, TFRC
1.0000	1.17	ADH5, CALM3, CAMP, CCL5, CD36, CPN1, CYBB, DSG1, GSTP1, HNRNPU, HSPA1B, HSPA8,
1.0000	2.10	AACS, ACTB, ADAM10, ADH5, ALDOA, ANK1, ANPEP, ANTXR2, ANXA11, ANXA2, ANXA5, A
1.0000	1.23	CALM3, CAMP, CCL5, CD36, FLOT1, HSP90AA1, ILK, ITGB3, LTF, NPM1, PECAM1, PPIA, PT
1.0000	1.23	ANXA2, BCAP31, CANX, CPN1, CTSD, EEF1A1, FLNA, HSP90AA1, HSPA1B, HSPA8, LRP1, M
1.0000	1.23	ADAM10, CD9, CDC42, ENO1, G6PD, HSP90AA1, HSPA1B, ITGB1, KRT17, NPM1, PTPRJ, R
1.0000	1.23	ACTB, CD151, CDC42, DSP, FLG2, FLNA, ILK, ITGB1, ITGB3, KRT10, KRT16, KRT17, KRT6A,
1.0000	1.23	CAMP, CCL5, CD36, GSTP1, HNRNPU, HSPA1B, HSPA8, LBP, LTF, MSN, PF4V1, PRDX2, RH
1.0000	1.23	AACS, ANK1, ANXA2, CALM3, CANX, CCL5, GNAI2, M6PR, MYH9, PLEK, PPIA, RAB10, RAB
1.0000	1.12	ACTB, ANXA5, ARL6IP5, ATP2A3, BCAP31, CASP14, CCL5, CFL1, CTSD, EIF5A, ENO1, FLNA,
1.0000	1.64	CTSD, LRBA, RAB1B, RAB33B, UBQLN1
1.0000	1.64	CALM3, FLOT1, ITGB1, ITGB3, RAP1B
1.0000	1.64	ACTB, CDC42, MSN, RAB10, RHOA
1.0000	1.64	CALM3, DSP, JUP, SRI, YWHAE
1.0000	1.64	CD226, HLA-A, HLA-B, PRDX1, TUBB

1.0000	1.64	B2M, CD226, CD36, HLA-A, TFRC
1.0000	1.64	B2M, CD226, CD36, HLA-A, TFRC
1.0000	1.64	HLA-A, HLA-B, LTF, PRDX2, TTLL12
1.0000	1.64	ENO1, G6PD, GAPDH, PGK1, PKM
1.0000	1.64	CALM3, FLOT1, ITGB1, ITGB3, RAP1B
1.0000	1.64	CYB5R3, G6PD, OSBPL2, SOD1, YWHAH
1.0000	1.64	DCD, GAPDH, LTF, S100A8, S100A9
1.0000	1.64	B2M, LTF, RCC2, RTN4, YWHAH
1.0000	1.64	ILK, ITGB1, ITGB3, RCC2, THBS1
1.0000	1.64	B2M, FLNA, RHOA, RTN4, VIM
1.0000	1.64	CDC42, DSP, G6PD, HNRNPU, ITGB1
1.0000	1.64	ADH5, GAPDH, HMBS, S100A8, S100A9
1.0000	1.64	CD47, CDC42, F11R, PFN1, RHOA
1.0000	1.64	G3BP1, GNAQ, ILK, JUP, LRP1
1.0000	1.64	ANXA2, CD9, GP1BA, KRT1, THBS1
1.0000	1.64	ARL6IP5, GSTP1, PPIA, PRDX1, RHOA
1.0000	1.64	GNAI2, NCL, PKM, RAB10, YWHAG
1.0000	1.64	ACTB, ADAM10, CDC42, DSP, VCL
1.0000	1.64	CD226, HLA-A, HLA-B, PRDX1, TUBB
1.0000	1.64	CYBB, PRDX1, PRDX2, RAC2, SOD1
1.0000	1.64	CD47, CDC42, F11R, PFN1, RHOA
1.0000	1.64	ACTB, CALM3, ITGB1, NASP, RCC2
1.0000	1.64	CDC42, ITGB1, RAB10, RHOA, SPN
1.0000	1.64	ACTB, INPP5D, PRDX2, RHOA, SOD1
1.0000	1.64	S100A8, S100A9, SOD1, SRI, TFRC
1.0000	1.64	B2M, CTSD, FCER1G, HLA-A, HLA-B
1.0000	1.64	MSN, PRDX2, RPLP0, RTN4, SOD1
1.0000	1.64	CDC42, DSP, G6PD, HNRNPU, ITGB1
1.0000	1.64	CDC42, HNRNPU, HSPA9, ITGB1, XRCC5
1.0000	1.64	B2M, CDC42, ITGB3, RTN4, THBS1
1.0000	1.64	CD47, DHX9, LBP, S100A8, S100A9
1.0000	1.64	CCL5, CD36, ITGB3, PECAM1, PTPRJ
1.0000	1.64	DCD, GAPDH, LTF, S100A8, S100A9
1.0000	1.64	CD47, CDC42, F11R, PFN1, RHOA
1.0000	1.64	CALM3, FLOT1, ITGB1, ITGB3, RAP1B
1.0000	1.64	GP1BA, ITGB1, RHOA, SELPLG, SPN
1.0000	1.64	FLOT1, HSPA1B, LBP, LTF, RTN4
1.0000	1.64	CCL5, CD36, GNAI2, ITGB3, RAP1B
1.0000	1.64	CALM3, DSP, FLNA, JUP, YWHAE
1.0000	1.64	CALM3, HSP90AA1, RAB10, SPTBN1, STOM
1.0000	1.64	GSTP1, HSPA1B, ITGA6, PRDX2, THBS1
1.0000	1.64	ACTB, ADAM10, CDC42, CFL1, HSPA8
1.0000	1.64	CD47, CDC42, F11R, PFN1, RHOA
1.0000	1.64	ANXA2, CD9, GP1BA, KRT1, THBS1
1.0000	1.64	CD36, PNPLA8, PPIA, SOD1, UBQLN1
1.0000	1.64	FLNA, HNRNPU, HSPA1B, ITGB1, RHOA
1.0000	1.64	CD36, PNPLA8, PPIA, SOD1, UBQLN1

1.0000	1.64	ARL6IP5, CD36, ITGB1, SLC2A1, THBS1
1.0000	1.64	ACTB, ADAM10, ITGB1, LRP1, SPTBN1
1.0000	1.64	CTSD, LRBA, RAB1B, RAB33B, UBQLN1
1.0000	1.64	ARL6IP5, CD36, ITGB1, SLC2A1, THBS1
1.0000	1.64	GNAI2, GNG5, ILK, ITGB1, RHOA
1.0000	1.64	GSTP1, HSPA1B, ITGA6, PRDX2, THBS1
1.0000	1.64	GSTP1, HSPA1B, ITGA6, PRDX2, THBS1
1.0000	1.64	CD9, CLTA, HSPA8, M6PR, TFRC
1.0000	1.64	CAVIN2, CD36, FLOT1, SELPLG, SLC2A1
1.0000	1.64	CDC42, HNRNPU, RCC2, TUBB, TUBB1
1.0000	1.64	BCAP31, CANX, HLA-A, HLA-B, HSPA8
1.0000	1.64	ACTB, HNRNPA2B1, HNRNPU, RCC2, XRCC5
1.0000	1.64	CANX, CLTA, HNRNPU, RAB27B, SOD1
1.0000	1.64	ANXA2, FLOT1, ITGB1, ITGB3, SRI
1.0000	1.64	GSTP1, PRDX1, PRDX2, PRDX6, PTGS1
1.0000	1.64	CD36, CLIC1, SLC2A1, SLC2A3, VDAC2
1.0000	1.64	GSTP1, PRDX1, PRDX2, PRDX6, PTGS1
1.0000	1.64	CCL5, ILK, NANS, PGK1, PKM
1.0000	1.64	GALE, HSPD1, ITGB3, PPIA, QSOX1
1.0000	1.64	ANK1, HSP90AA1, JUP, SOD1, YWHAE
1.0000	1.64	ANK1, HSP90AA1, JUP, SOD1, YWHAE
1.0000	1.64	ANXA2, GSDMA, OSBPL2, PFN1, TLN1
1.0000	1.64	CYB5R3, CYBB, GSR, PCYOX1, QSOX1
1.0000	1.64	ITGA2B, ITGB1, ITGB3, RPSA, THBS1
1.0000	1.23	CCL5, CD226, CD36, DHX9, FCN1, HLA-A, HLA-B, HSP90AA1, HSPD1, LBP, LTF, MATR3, PR
1.0000	1.23	ATP2A3, ATP5F1B, BCAP31, CALM3, CCL5, FLNA, GP1BA, GP1BB, ITGB3, S100A8, S100A9
1.0000	1.23	ADAM10, CDC42, ENO1, G6PD, HSP90AA1, HSPA1B, ITGB1, KRT17, NPM1, PTPRJ, RHOA,
1.0000	1.23	ATP2A3, ATP5F1B, BCAP31, CALM3, CCL5, FLNA, GP1BA, GP1BB, ITGB3, S100A8, S100A9
1.0000	1.23	ACTB, B2M, G6PD, HNRNPU, HSPA9, INPP5D, ITGB1, ITGB3, LTF, PRDX2, PTBP1, RCC2, R
1.0000	1.23	ACTB, ADAM10, ARPC5, CALM3, CANX, CFL1, FLNA, FSCN1, HSP90AA1, HSPA8, RAB27B,
1.0000	1.23	CANX, CCT2, CCT4, CCT7, CCT8, CDC42, CYBB, FLNA, G3BP1, GNAI2, HSP90AA1, SLC2A3,
1.0000	1.12	ANTXR2, ANXA7, ARL6IP5, ATP2A3, B2M, BCAP31, CANX, CD59, CDC42, CLTA, CYB5R3, C
1.0000	1.12	ACTB, ADAM10, CALM3, CAVIN2, CD226, CDC42, DSP, EEF1A1, EEF2, FLNA, GSTP1, HINT
1.0000	1.15	ACTB, ADAM10, CAMP, CCL5, CD47, CDC42, DHX9, GNAI2, GNG5, HLA-A, HNRNPU, ILK, I
1.0000	1.15	ANPEP, CANX, CCL5, CFL1, CSDE1, DHX9, EEF1A1, F11R, FCN1, HSPA1B, HSPA8, ITGB1, IT
1.0000	1.15	CALM3, CAMP, CCL5, CD36, FLOT1, HSP90AA1, ILK, ITGB3, KANSL3, LTF, NPM1, PECAM1,
1.0000	1.09	ACTB, ANXA2, B2M, CAMP, CD226, CD36, CFL1, CYBB, DHX9, ENO1, FCER1G, FCN1, FLOT
1.0000	1.06	ACTB, ADAM10, ANXA2, ANXA7, B2M, CCL5, CD226, CD36, CD47, CSDE1, CYBB, DAZAP1,
1.0000	1.23	ADAM10, CDC42, CFL1, ITGB1, ITGB3, MATR3, MYADM, MYH9, NASP, PFN1, RTN4, SOD1
1.0000	1.23	ACTB, B2M, CCL5, CD47, F11R, FLOT1, GP1BA, HLA-A, HSPD1, MMRN1, RHOA, TFRC
1.0000	1.23	CAMP, CCL5, CDC42, CFL1, DCD, EEF1A1, GAPDH, HSPA8, HSPD1, KRT6A, LTF, STOM
1.0000	1.23	CCT2, CCT4, CCT6A, CCT7, CCT8, CD36, FLNA, HSP90AA1, JUP, NPM1, TCP1, TFRC
1.0000	1.23	ACTB, CALM3, CDC42, CFL1, FLNA, HNRNPU, HSPA1B, ITGB1, NASP, RCC2, RHOA, SPTBN
1.0000	1.23	ACTB, B2M, FCER1G, HLA-B, INPP5D, ITGB1, MYH9, PRDX2, PTPRJ, RHOA, SOD1, SPN
1.0000	1.23	AACS, ANXA2, CALM3, CCL5, GNAI2, PPIA, RAB10, RAB27B, RAB33B, RAC2, RAP1B, SRI
1.0000	1.23	ANTXR2, ANXA2, B2M, CLTA, CTSD, HLA-A, HLA-B, ITGB1, RAB10, RAB27B, RCC2, TFRC
1.0000	1.23	ADAM10, CANX, CDC42, CYBB, GNAI2, HNRNPU, HSP90AA1, HSPA8, ITGB1, RHOA, RPLP

1.0000	1.23	ADAM10, CANX, CDC42, CYBB, GNAI2, HNRNPU, HSP90AA1, HSPA8, ITGB1, RHOA, RPLP0
1.0000	1.08	AACS, ACTB, ANXA2, ARL6IP5, B2M, BCAP31, CALM3, CCL5, CD151, CD36, CD47, CDC42,
1.0000	1.11	ACTB, ANXA2, ANXA5, ATP2A3, B2M, CALM3, F11R, FLNA, GAPDH, GNAI2, GNAQ, GSTP1
1.0000	1.11	ACTB, ADAM10, CALM3, CANX, CDC42, CLTA, EEF2, FLOT1, GNAI2, GNAQ, HSPA8, ITGB1,
1.0000	1.11	ADAM10, ANXA2, ATP5F1B, B2M, CALM3, CLIC1, CLTA, CYBB, FCER1G, FLNA, FLOT1, GN
1.0000	1.05	ADAM10, ALDOA, ANK1, ANPEP, ANTXR2, ANXA11, ANXA2, ANXA7, ARL6IP5, ARPC5, AT
1.0000	1.06	ACTB, ARL6IP5, ATIC, ATP5F1B, CAMP, CCL5, CD36, CD47, CDC42, CYBB, DHX9, EEF1A1,
1.0000	1.05	ACTB, ADAM10, ANXA2, ANXA5, ANXA7, ARL6IP5, ATP5F1B, B2M, CALM3, CCL5, CD36, C
1.0000	1.08	ACTB, CALM3, CAMP, CCL5, CD36, CDC42, EEF1A1, ENO1, FLOT1, GNAI2, GNAQ, GSTP1,
1.0000	1.08	ACTB, CALM3, CAMP, CCL5, CD36, CDC42, EEF1A1, ENO1, FLOT1, GNAI2, GNAQ, GSTP1,
1.0000	1.05	ACTB, ADAM10, ALDOA, ANXA2, ARPC5, B2M, CCT2, CCT8, CD36, CD47, CD9, CDC42, CL
1.0000	1.13	CALM3, CPN1, CYBB, DSG1, GNAI2, HNRNPU, HSP90AA1, HSPA1B, HSPA8, ITGB3, NCL, P
1.0000	1.13	ACTB, ARL6IP5, B2M, CALM3, CCL5, CLIC1, CYBB, FLNA, G6PD, GNAI2, ITGB1, ITGB3, RH
1.0000	1.05	ADH5, ANPEP, B2M, CAMP, CCL5, CD226, CD36, CD47, CDC42, CFL1, CYBB, DCD, DDX60L
1.0000	2.45	ACTB, TFRC
1.0000	2.45	ACTB, HNRNPU
1.0000	2.45	PPIA, UBE2L3
1.0000	2.45	ACTB, XRCC5
1.0000	2.45	ACTB, HNRNPU
1.0000	2.45	ITGB1, ITGB3
1.0000	2.45	ITGB1, ITGB3
1.0000	2.45	CDC42, RHOA
1.0000	2.45	ANXA2, MYH9
1.0000	2.45	MATR3, RTN4
1.0000	2.45	HLA-A, HLA-B
1.0000	2.45	ARL6IP5, ITGB1
1.0000	2.45	B2M, HLA-A
1.0000	2.45	HLA-A, HSPD1
1.0000	2.45	HLA-A, HLA-B
1.0000	2.45	HLA-A, HLA-B
1.0000	2.45	HLA-A, HLA-B
1.0000	2.45	HLA-A, HLA-B
1.0000	2.45	HLA-A, HLA-B
1.0000	2.45	B2M, HLA-A
1.0000	2.45	B2M, HLA-A
1.0000	2.45	DSP, JUP
1.0000	2.45	ADH5, YWHAZ
1.0000	2.45	ARL6IP5, ITGB1
1.0000	2.45	ACTB, DHX9
1.0000	2.45	FLNA, NCL
1.0000	2.45	FLNA, NCL
1.0000	2.45	ADAM10, MYH9
1.0000	2.45	SLC2A1, SLC2A3
1.0000	2.45	SLC2A1, SLC2A3
1.0000	2.45	ARL6IP5, ITGB1
1.0000	2.45	ARL6IP5, ITGB1
1.0000	2.45	S100A8, S100A9

1.0000	2.45	LBP, TFRC
1.0000	2.45	CDC42, RHOA
1.0000	2.45	BCAP31, CD36
1.0000	2.45	ADAM10, TSPAN14
1.0000	2.45	CD36, THBS1
1.0000	2.45	CALM3, RAP1B
1.0000	2.45	ITGA6, KRT6B
1.0000	2.45	ITGB1, RAB10
1.0000	2.45	ILK, SOD1
1.0000	2.45	ITGB1, ITGB3
1.0000	2.45	HNRNPU, KANSL3
1.0000	2.45	ADH5, YWHAZ
1.0000	2.45	B2M, CD36
1.0000	2.45	FCN1, LBP
1.0000	2.45	ADAM10, TSPAN14
1.0000	2.45	SLC2A1, SLC2A3
1.0000	2.45	SLC2A1, SLC2A3
1.0000	2.45	HNRNPU, KANSL3
1.0000	2.45	HSP90AA1, HSPD1
1.0000	2.45	GNAQ, JUP
1.0000	2.45	GNAQ, JUP
1.0000	2.45	CALM3, LBP
1.0000	2.45	HSP90AA1, XRCC5
1.0000	2.45	RHOA, YWHAH
1.0000	2.45	PRDX2, RAC2
1.0000	2.45	CALM3, SRI
1.0000	2.45	CALM3, SRI
1.0000	2.45	ITGB1, VIM
1.0000	2.45	FSCN1, LRP1
1.0000	2.45	CD36, ITGB3
1.0000	2.45	CD36, ITGB3
1.0000	2.45	CALM3, SRI
1.0000	2.45	CD36, ITGB3
1.0000	2.45	ARL6IP5, ITGB1
1.0000	2.45	ILK, SOD1
1.0000	2.45	ILK, SOD1
1.0000	2.45	CALM3, SRI
1.0000	2.45	CD36, PNPLA8
1.0000	2.45	SLC2A1, SLC2A3
1.0000	2.45	ARL6IP5, ITGB1
1.0000	2.45	ARL6IP5, ITGB1
1.0000	2.45	ARL6IP5, ITGB1
1.0000	2.45	ARL6IP5, ITGB1
1.0000	2.45	ACTB, ITGB3
1.0000	2.45	HLA-A, HLA-B
1.0000	2.45	CALM3, RAP1B
1.0000	2.45	FLNA, MSN

1.0000	2.45	GNAI2, RAP1B
1.0000	2.45	CDC42, SOD1
1.0000	2.45	SLC2A1, SLC2A3
1.0000	2.45	CD36, ITGB3
1.0000	2.45	CD36, THBS1
1.0000	2.45	CD36, THBS1
1.0000	2.45	RHOA, RTN4
1.0000	2.45	RHOA, RTN4
1.0000	2.45	B2M, HSP90AA1
1.0000	2.45	ILK, SOD1
1.0000	2.45	RHOA, RTN4
1.0000	2.45	INPP5D, MYH9
1.0000	2.45	RHOA, YWHAH
1.0000	2.45	F11R, VCL
1.0000	2.45	PLEK, TLN1
1.0000	2.45	RPLP0, RTN4
1.0000	2.45	ATIC, ITGB1
1.0000	2.45	NPM1, PATJ
1.0000	2.45	DHX9, HNRNPA2B1
1.0000	2.45	HLA-A, HLA-B
1.0000	2.45	LTF, PRDX2
1.0000	2.45	S100A8, S100A9
1.0000	2.45	F11R, PFN1
1.0000	2.45	ILK, SOD1
1.0000	2.45	MYH9, RAC2
1.0000	2.45	CDC42, RHOA
1.0000	2.45	DSG1, THBS1
1.0000	2.45	GSTP1, LBP
1.0000	2.45	CD36, GSTP1
1.0000	2.45	CD36, GSTP1
1.0000	2.45	GSTP1, LBP
1.0000	2.45	CD36, GSTP1
1.0000	2.45	CD36, GSTP1
1.0000	2.45	GSTP1, LBP
1.0000	2.45	CD36, HSPD1
1.0000	2.45	ITGB3, PECAM1
1.0000	2.45	GSTP1, PPIA
1.0000	2.45	ITGB1, VIM
1.0000	2.45	ACTB, HNRNPU
1.0000	2.45	ADAM10, MYH9
1.0000	2.45	PLEK, RAP1B
1.0000	2.45	PLEK, RAP1B
1.0000	2.45	LBP, LTF
1.0000	2.45	LBP, LTF
1.0000	2.45	LBP, LTF
1.0000	2.45	RTN3, RTN4
1.0000	2.45	SLC2A1, SLC2A3

1.0000	2.45	F11R, RCC2
1.0000	2.45	ACTB, VCL
1.0000	2.45	ANXA2, CD36
1.0000	2.45	ANXA2, CD36
1.0000	2.45	PFN1, YWHAZ
1.0000	2.45	S100A8, S100A9
1.0000	2.45	CDC42, RHOA
1.0000	2.45	CD47, PECAM1
1.0000	2.45	LTF, S100A9
1.0000	2.45	CCL5, CDC42
1.0000	2.45	HSPA1B, PRDX2
1.0000	2.45	PTPRJ, RTN4
1.0000	2.45	HNRNPU, KANSL3
1.0000	2.45	CTSD, ITGB3
1.0000	2.45	HLA-A, HLA-B
1.0000	2.45	FCER1G, HLA-A
1.0000	2.45	CD36, LRP1
1.0000	2.45	RHOA, YWHAH
1.0000	2.45	ARL6IP5, ITGB1
1.0000	2.45	ANXA2, RAB1B
1.0000	2.45	DHX9, HNRNPA2B1
1.0000	2.45	CD36, LRP1
1.0000	2.45	FLNA, MSN
1.0000	2.45	CCL5, PPIA
1.0000	2.45	ACTB, RHOA
1.0000	2.45	F11R, VCL
1.0000	2.45	ACTB, DHX9
1.0000	2.45	ACTB, TFRC
1.0000	2.45	SOD1, SRI
1.0000	2.45	HLA-A, HLA-B
1.0000	2.45	GNAI2, RAP1B
1.0000	2.45	SLC2A1, SLC2A3
1.0000	2.45	HLA-A, RHOA
1.0000	2.45	HLA-A, RHOA
1.0000	2.45	HSP90AA1, SOD1
1.0000	2.45	CALM3, RAP1B
1.0000	2.45	ITGB1, ITGB3
1.0000	2.45	ITGB1, ITGB3
1.0000	2.45	PRDX2, SOD1
1.0000	2.45	CDC42, SOD1
1.0000	2.45	G6PD, RTN4
1.0000	2.45	FLNA, SOD1
1.0000	2.45	RTN3, RTN4
1.0000	2.45	DHX9, HSP90AA1
1.0000	2.45	DHX9, HSP90AA1
1.0000	2.45	ITGB1, XRCC5
1.0000	2.45	RHOA, YWHAH

1.0000	2.45	GNAI2, RAP1B
1.0000	2.45	PRDX2, SPN
1.0000	2.45	CFL1, MYH9
1.0000	2.45	DHX9, HNRNPA2B1
1.0000	2.45	CALM3, SRI
1.0000	2.45	SLC2A1, SLC2A3
1.0000	2.45	CALM3, SRI
1.0000	2.45	CALM3, SRI
1.0000	2.45	CALM3, SRI
1.0000	2.45	F11R, PFN1
1.0000	2.45	RAP1B, RPLP0
1.0000	2.45	CDC42, RHOA
1.0000	2.45	ARL6IP5, ITGB1
1.0000	2.45	ARL6IP5, ITGB1
1.0000	2.45	ARL6IP5, ITGB1
1.0000	2.45	ITGB1, XRCC5
1.0000	2.45	S100A8, S100A9
1.0000	2.45	PNPLA8, RTN4
1.0000	2.45	CDC42, RHOA
1.0000	2.45	PFN1, YWHAZ
1.0000	2.45	DAZAP1, DSG1
1.0000	2.45	LBP, RAC2
1.0000	2.45	FLNA, YWHAE
1.0000	2.45	CALM3, SRI
1.0000	2.45	CALM3, SRI
1.0000	2.45	CDC42, CFL1
1.0000	2.45	CDC42, CFL1
1.0000	2.45	ANXA2, CD9
1.0000	2.45	ITGB3, PECAM1
1.0000	2.45	ITGB3, PECAM1
1.0000	2.45	CCL5, PF4V1
1.0000	2.45	DHX9, GSDMA
1.0000	2.45	CALM3, SRI
1.0000	2.45	GSTP1, PPIA
1.0000	2.45	S100A8, S100A9
1.0000	2.45	GP1BA, PLEK
1.0000	2.45	CD36, XRCC5
1.0000	2.45	CAMP, SELPLG
1.0000	2.45	SLC2A1, SLC2A3
1.0000	2.45	RAP1B, RPLP0
1.0000	2.45	CAMP, SELPLG
1.0000	2.45	LBP, RAC2
1.0000	2.45	RTN3, RTN4
1.0000	2.45	ACTB, VCL
1.0000	2.45	ITGB3, PECAM1
1.0000	2.45	CD99, PECAM1
1.0000	2.45	CD99, F11R

1.0000	2.45	DSP, JUP
1.0000	2.45	FLNA, YWHAE
1.0000	2.45	FLNA, YWHAE
1.0000	2.45	FLNA, YWHAE
1.0000	2.45	DSP, JUP
1.0000	2.45	CALM3, SRI
1.0000	2.45	DSP, JUP
1.0000	2.45	DSP, JUP
1.0000	2.45	CDC42, GNAI2
1.0000	2.45	ARL6IP5, ITGB1
1.0000	2.45	LBP, RAC2
1.0000	2.45	LBP, RAC2
1.0000	2.45	SOD1, SRI
1.0000	2.45	CD36, ITGB3
1.0000	2.45	ILK, THBS1
1.0000	2.45	CALM3, HSP90AA1
1.0000	2.45	CDC42, RHOA
1.0000	2.45	F11R, VDAC2
1.0000	2.45	ANXA2, CD36
1.0000	2.45	CDC42, CFL1
1.0000	2.45	HSPA1B, PRDX2
1.0000	2.45	CD36, LRP1
1.0000	2.45	HLA-A, HLA-B
1.0000	2.45	SLC2A1, SLC2A3
1.0000	2.45	SLC2A1, SLC2A3
1.0000	2.45	ARL6IP5, ITGB1
1.0000	2.45	ITGB1, ITGB3
1.0000	2.45	DSP, JUP
1.0000	2.45	FLNA, MSN
1.0000	2.45	FLNA, YWHAE
1.0000	2.45	CALM3, RAP1B
1.0000	2.45	CDC42, CFL1
1.0000	2.45	SLC2A1, SLC2A3
1.0000	2.45	ITGB1, LRP1
1.0000	2.45	CD36, LRP1
1.0000	2.45	PECAM1, VCL
1.0000	2.45	ITGB1, YWHAE
1.0000	2.45	HSPA1B, PRDX2
1.0000	2.45	PTPRJ, RTN4
1.0000	2.45	ILK, RHOA
1.0000	2.45	CD47, LBP
1.0000	2.45	SLC2A1, SLC2A3
1.0000	2.45	CALM3, SRI
1.0000	2.45	F11R, VCL
1.0000	2.45	F11R, MMRN1
1.0000	2.45	CALM3, SRI
1.0000	2.45	CALM3, SRI

1.0000	2.45	ACTB, RCC2
1.0000	2.45	RTN3, RTN4
1.0000	2.45	RTN3, RTN4
1.0000	2.45	ARL6IP5, ITGB1
1.0000	2.45	RTN3, RTN4
1.0000	2.45	RTN3, RTN4
1.0000	2.45	PRDX2, SPN
1.0000	2.45	F11R, VCL
1.0000	2.45	GNAI2, RAP1B
1.0000	2.45	CD36, RHOA
1.0000	2.45	CALM3, SRI
1.0000	2.45	PKM, THBS1
1.0000	2.45	ANXA2, MYH9
1.0000	2.45	ARL6IP5, ITGB1
1.0000	2.45	FCN1, PPIA
1.0000	2.45	ARL6IP5, ITGB1
1.0000	2.45	CD36, LRP1
1.0000	2.45	CD36, LRP1
1.0000	2.45	SLC2A1, SLC2A3
1.0000	2.45	GP1BA, RHOA
1.0000	2.45	GP1BA, RHOA
1.0000	2.45	ANXA2, MYH9
1.0000	2.45	B2M, CD36
1.0000	2.45	CD36, HNRNPU
1.0000	2.45	MYH9, RAC2
1.0000	2.45	CDC42, RHOA
1.0000	2.45	MYH9, SOD1
1.0000	2.45	CALM3, RAP1B
1.0000	2.45	ACTB, DHX9
1.0000	2.45	HSPA1B, PRDX2
1.0000	2.45	HSPA1B, PRDX2
1.0000	2.45	ACTB, HNRNPU
1.0000	2.45	ACTB, HNRNPU
1.0000	2.45	HNRNPA2B1, XRCC5
1.0000	2.45	ACTB, HNRNPU
1.0000	2.45	CSDE1, PSMA6
1.0000	2.45	ANXA2, CD151
1.0000	2.45	PSMA5, PSMA6
1.0000	2.45	PSMA5, PSMA6
1.0000	2.45	CLTA, RAB27B
1.0000	2.45	CD59, GP1BA
1.0000	2.45	CD59, GP1BA
1.0000	2.45	BCAP31, HLA-A
1.0000	2.45	CLTA, M6PR
1.0000	2.45	B2M, HLA-A
1.0000	2.45	ACTB, HSPA8
1.0000	2.45	ACTB, HSPA8

1.0000	2.45	CD59, GP1BA
1.0000	2.45	CANX, FLOT1
1.0000	2.45	HSP90AA1, LTF
1.0000	2.45	RAB10, RTN4
1.0000	2.45	ADAM10, TSPAN14
1.0000	2.45	GAPDH, SYNCRIP
1.0000	2.45	B2M, HLA-A
1.0000	2.45	CD36, LRP1
1.0000	2.45	ITGB3, QSOX1
1.0000	2.45	CDC42, UBE2L3
1.0000	2.45	CD36, LRP1
1.0000	2.45	CCL5, PF4V1
1.0000	2.45	SRI, YWHAE
1.0000	2.45	CD36, SLC2A1
1.0000	2.45	SLC2A1, SLC2A3
1.0000	2.45	ITGB1, THBS1
1.0000	2.45	ITGA6, ITGB3
1.0000	2.45	G6PD, SLC2A3
1.0000	2.45	CCL5, PF4V1
1.0000	2.45	CD36, HSPD1
1.0000	2.45	ANK1, JUP
1.0000	2.45	MYL6, TPM4
1.0000	2.45	ITGB1, ITGB3
1.0000	2.45	SLC2A1, SLC2A3
1.0000	2.45	SLC2A1, SLC2A3
1.0000	2.45	SLC2A1, SLC2A3
1.0000	2.45	FLNA, YWHAE
1.0000	2.45	ADH5, GAPDH
1.0000	2.45	CYB5R3, CYBB
1.0000	2.45	PCYOX1, QSOX1
1.0000	2.45	ITGB3, QSOX1
1.0000	2.45	ITGB3, QSOX1
1.0000	2.45	AACS, ATP5F1B
1.0000	2.45	ADH5, GAPDH
1.0000	2.45	CDC42, UBE2L3
1.0000	2.45	ITGB1, ITGB3
1.0000	2.45	ITGB1, ITGB3
1.0000	2.45	F11R, GNG5
1.0000	2.45	CD36, THBS1
1.0000	2.45	CD36, LRP1
1.0000	2.45	CALM3, HSP90AA1
1.0000	2.45	CLTA, LRP1
1.0000	2.45	H2AC20, H2BC21
1.0000	2.45	CCL5, PF4V1
1.0000	2.45	CCL5, PF4V1
1.0000	2.45	ACTB, NPM1
1.0000	2.45	ACTB, HNRNPC

1.0000	2.45	ACTB, HNRNPC
1.0000	2.45	ITGA6, ITGB3
1.0000	2.45	CLTA, LRP1
1.0000	2.45	SLC2A1, SLC2A3
1.0000	2.45	HNRNPA2B1, MATR3
1.0000	2.45	CANX, FLOT1
1.0000	2.45	S100A8, S100A9
1.0000	2.45	S100A8, S100A9
1.0000	2.45	ITGA6, ITGB3
1.0000	2.45	CD36, FCN1
1.0000	2.45	CCL5, PF4V1
1.0000	2.45	HLA-A, HLA-B
1.0000	2.45	S100A8, S100A9
1.0000	2.45	MYH9, PGK1
1.0000	2.45	ANXA11, ANXA2
1.0000	2.45	JUP, VCL
1.0000	2.45	DSG1, PTPRJ
1.0000	2.45	ANXA2, PPIA
1.0000	2.45	HLA-A, HLA-B
1.0000	2.45	CCL5, PF4V1
1.0000	2.45	CD36, THBS1
1.0000	2.45	S100A8, S100A9
1.0000	2.45	S100A8, S100A9
1.0000	2.45	S100A8, S100A9
1.0000	2.45	S100A8, S100A9
1.0000	2.45	ACTB, CALM3
1.0000	2.45	HSP90AA1, RHOA
1.0000	2.45	HSP90AA1, RHOA
1.0000	2.45	NPM1, PSMA6
1.0000	2.45	SLC2A1, SLC2A3
1.0000	2.45	GAPDH, LDHB
1.0000	2.45	SLC2A1, SLC2A3
1.0000	2.45	CD36, CD47
1.0000	2.45	DSP, JUP
1.0000	2.45	SLC2A1, SLC2A3
1.0000	2.45	HNRNPA2B1, PCBP1
1.0000	2.45	HNRNPF, HNRNPU
1.0000	2.45	HNRNPA2B1, NPM1
1.0000	2.45	HNRNPA2B1, HNRNPC
1.0000	1.06	ACTB, ADAM10, B2M, CAMP, CCL5, CD151, CD47, CD9, CDC42, DAZAP1, DHX9, GNAI2, G
1.0000	1.09	ACTB, CALM3, CAMP, CCL5, CD36, FLOT1, GNAQ, GSTP1, HSP90AA1, ILK, ITGB3, LRP1, LT
1.0000	1.09	ACTB, ANXA2, B2M, CDC42, EEF2, FCER1G, FLNA, G6PD, GP1BA, GP1BB, HLA-B, HSPA1B,
1.0000	1.09	ACTB, ANXA2, B2M, CDC42, EEF2, FCER1G, FLNA, G6PD, GP1BA, GP1BB, HLA-B, HSPA1B,
1.0000	1.09	ACTB, ADAM10, ANK1, ARPC5, CALM3, CANX, CDC42, CFL1, CYBB, FLNA, FSCN1, GNAI2, (
1.0000	1.05	ACTB, ADAM10, ANK1, ARPC5, CALM3, CAMP, CANX, CCT4, CCT8, CD36, CDC42, CFL1, CY
1.0000	1.06	ACTB, ANXA2, ARL6IP5, ATP2A3, ATP5F1B, B2M, CALM3, CCL5, CD36, CD47, CLIC1, CYBB
1.0000	1.06	ANXA2, B2M, CCT2, CCT4, CCT6A, CCT7, CCT8, CD151, CD36, CD47, CD53, CDC42, EIF5A,

1.0000	1.22	CD36, ITGB3, JUP, PTPRJ, RCC2, RHOA, THBS1, TLN1, VCL
1.0000	1.22	CD36, FCN1, FLOT1, HSPA1B, HSPD1, LBP, LTF, RTN4, UBQLN1
1.0000	1.22	ALDOA, ENO1, G6PD, GALE, GAPDH, PGK1, PKM, SLC2A1, SLC2A3
1.0000	1.22	CDC42, HSP90AA1, HSPA1B, NPM1, PPIA, RPL11, RPS15, UBE2L3, UBQLN1
1.0000	1.22	ANXA2, B2M, CALM3, CD151, CD36, CDC42, FLOT1, ITGB3, LRP1
1.0000	1.22	ANXA2, CD36, CD9, FCER1G, FLOT1, ITGB1, ITGB3, LRP1, TFRC
1.0000	1.22	CDC42, HSP90AA1, HSPA1B, NPM1, PPIA, RPL11, RPS15, UBE2L3, UBQLN1
1.0000	1.22	ADAM10, ANXA2, FLOT1, FSCN1, HRNR, ITGB1, ITGB3, LRP1, QSOX1
1.0000	1.22	ANPEP, F11R, FCN1, HSPA1B, ITGB1, ITGB3, RPSA, SELPLG, TFRC
1.0000	1.22	CALM3, DSP, ENO1, G6PD, HSP90AA1, JUP, RHOA, SOD1, SRI
1.0000	1.22	CD36, GNAI2, ITGB3, LRP1, NCL, PKM, RAB10, VIM, YWHAG
1.0000	1.22	F11R, FLOT1, PTPRJ, RAP1B, RCC2, RHOA, THBS1, TLN1, VCL
1.0000	1.22	ACTB, B2M, CCL5, CD47, GP1BA, HLA-A, HSPD1, RHOA, TFRC
1.0000	1.22	ENO1, GNAI2, GSTP1, HSPA1B, ITGA6, PPIA, PRDX2, THBS1, VDAC2
1.0000	1.22	B2M, CD59, CD9, CLTA, HLA-A, HLA-B, HSPA8, M6PR, TFRC
1.0000	1.22	ADAM10, CD47, CD53, CD59, CYBB, FCER1G, SLC2A3, STOM, TSPAN14
1.0000	1.22	ANXA2, ANXA5, GAPDH, LTF, NPM1, RPL11, RPS15, YWHAB, YWHAG
1.0000	1.03	ACTB, ADAM10, ANXA2, ANXA7, ARL6IP5, ATP2A3, B2M, BCAP31, CALM3, CAMP, CCL5,
1.0000	1.03	AACS, ACTB, ADAM10, ANXA2, ARL6IP5, B2M, BCAP31, CALM3, CCL5, CCT2, CCT4,
1.0000	1.03	ACTB, ADAM10, ANXA2, ARPC5, B2M, CALM3, CCT2, CCT4, CCT6A, CCT7, CCT8, CD151,
1.0000	1.11	ACTB, ANXA2, B2M, CDC42, FCER1G, HLA-B, INPP5D, ITGB1, LTF, MYH9, PRDX2, PTPRJ,
1.0000	1.11	ACTB, ARPC5, ATIC, B2M, CALM3, CDC42, G6PD, ITGB1, RHOA, RTN3, RTN4, SLC2A1,
1.0000	1.11	CD36, CSDE1, DHX9, ENO1, FLNA, GAPDH, GSTP1, HNRNPA2B1, HNRNPC, HNRNPU,
1.0000	1.11	CD36, CSDE1, DHX9, ENO1, FLNA, GAPDH, HNRNPA2B1, HNRNPC, HNRNPU, HSPA8, NCL,
1.0000	1.11	ACTB, B2M, CCL5, CD151, CD47, FCER1G, HLA-A, HSPD1, MSN, MYH9, PRDX2, RAC2,
1.0000	1.11	ARPC5, CD36, CDC42, FSCN1, HSP90AA1, HSPA1B, HSPA8, MSN, MYADM, PFN1, PLEK,
1.0000	1.11	CD36, CD47, CDC42, FLOT1, FSCN1, G3BP1, HSP90AA1, HSPA1B, MSN, PFN1, PLEK,
1.0000	1.11	ARL6IP5, ATP2A3, BCAP31, CALM3, CCL5, CTSD, F11R, GNAQ, HSPD1, ITGA6, ITGB1,
1.0000	1.11	ACTB, ARPC5, ATIC, B2M, CALM3, CDC42, G6PD, ITGB1, RHOA, RTN3, RTN4, SLC2A1,
1.0000	1.07	ACTB, ARPC5, ATIC, B2M, CALM3, CDC42, G6PD, GSTP1, HSP90AA1, ITGB1, LRP1, RHOA,
1.0000	1.07	ALDOA, ARPC5, CCT8, CTSD, DSC1, DSG1, DSP, EEF1A1, EEF2, FCER1G, FCN1, GSTP1,
1.0000	1.02	ACTB, ADAM10, ALDOA, ANXA2, ARPC5, B2M, CCT2, CCT8, CD36, CD47, CD9, CDC42,
1.0000	1.03	ACTB, ADAM10, ADH5, CALM3, CAMP, CCL5, CD36, CDC42, DSP, FLNA, FLOT1, G6PD,
1.0000	1.03	ACTB, ADAM10, ADH5, CALM3, CAMP, CCL5, CD36, CDC42, DSP, FLNA, FLOT1, G6PD,
1.0000	1.02	AACS, ACTB, ADAM10, ADH5, ALDOA, ANPEP, ANXA2, ANXA5, ARL6IP5, ARPC5, ATP2A3,
1.0000	1.05	ACTB, ADAM10, ANXA2, B2M, CAMP, CFL1, CYBB, F11R, G6PD, HLA-B, HSPA1B, HSPA9,
1.0000	1.03	ACTB, ANXA5, ARL6IP5, BCAP31, CCL5, CFL1, CTSD, EIF5A, ENO1, FLNA, G6PD, GNAI2,
1.0000	1.03	ACTB, ANXA5, ARL6IP5, BCAP31, CCL5, CFL1, CTSD, EIF5A, ENO1, FLNA, G6PD, GNAI2,
1.0000	1.01	ADAM10, ADH5, ANXA2, B2M, CAMP, CCL5, CD226, CD36, CD47, CD9, CDC42, CFL1,
1.0000	1.01	AACS, ACTB, ADAM10, ADH5, ANPEP, ANTXR2, ANXA11, ANXA2, ANXA5, ANXA7,
1.0000	1.04	ADAM10, ATP5F1B, CCL5, CD151, CD47, CD99, FERMT3, GNAI2, ITGA6, ITGB1, ITGB3,
1.0000	1.04	ADAM10, ATP5F1B, CCL5, CD151, CD47, CD99, FERMT3, GNAI2, ITGA6, ITGB1, ITGB3,
1.0000	1.04	AACS, ANK1, ANXA2, CALM3, CANX, CCL5, GNAI2, M6PR, MYH9, PLEK, PNPLA8, PPIA,
1.0000	1.04	ACTB, ANXA2, CSDE1, DAZAP1, DHX9, EIF5A, HNRNPU, HSPA8, ILK, ITGA6, JUP, KANSL3,
1.0000	1.04	ARPC5, CD47, CDC42, F11R, FLNA, FSCN1, HNRNPU, HSPA1B, ITGB3, LRP1, MYADM,
1.0000	1.04	ADAM10, ATP5F1B, CCL5, CD151, CD47, CD99, FERMT3, GNAI2, ITGA6, ITGB1, ITGB3,
1.0000	1.04	ACTB, ANXA2, EIF5A, FLNA, HINT1, HNRNPA2B1, HNRNPA3, HNRNPC, HNRNPF, HNRNPH

1.0000	1.08	CALM3, CCL5, CDC42, FCN1, FLNA, GNAI2, GNAQ, GNG5, GP1BA, ITGB3, PLEK, RAC2, YW
1.0000	1.08	ANXA2, CD9, GP1BA, GSTP1, HLA-A, HLA-B, KRT1, LTF, MMRN1, PRDX2, SOD1, THBS1, TT
1.0000	1.08	ATP2A3, CALM3, DSP, ENO1, FLNA, G6PD, GNAI2, HSP90AA1, JUP, RHOA, SOD1, SRI, YWI
1.0000	1.08	AACS, ANXA2, CALM3, CCL5, GNAI2, PPIA, RAB10, RAB27B, RAB33B, RAC2, RAP1B, S100/
1.0000	1.08	ANPEP, F11R, FCN1, HSPA1B, ITGB1, ITGB3, KRT6A, LTF, PPIA, RPSA, SELPLG, TCP1, TFRC
1.0000	1.08	ARL6IP5, CD36, G6PD, GSR, HSPA1B, PNPLA8, PPIA, PRDX1, PRDX2, SLC2A1, SOD1, UBQL
1.0000	1.08	ATIC, CCL5, CDC42, GAPDH, GSTP1, HMBS, ILK, NANS, PGK1, PKM, PRDX6, TKT, UBE2L3
1.0000	1.02	ACTB, ANXA2, B2M, CDC42, EEF2, FCER1G, FLNA, G6PD, GP1BA, GP1BB, HLA-B, HSPA1B,
1.0000	1.02	ACTB, ANXA11, CALM3, CDC42, CFL1, CLIC1, CLTA, FLNA, GNAI2, HNRNPU, HSPA1B, HSP/
1.0000	1.00	ACTB, ADAM10, ANXA2, ANXA7, B2M, CCL5, CD226, CD36, CD47, CPN1, CSDE1, CYBB, D/
1.0000	1.01	ARL6IP5, ATP2A3, BCAP31, CALM3, CCL5, CCT2, CCT4, CDC42, CTSD, DHX9, F11R, GNAI2,
1.0000	1.01	ALDOA, ANXA2, ARPC5, B2M, CAMP, CCT2, CCT8, CTSD, CYB5R3, EEF1A1, EEF2, FCN1, FE
1.0000	1.01	ALDOA, ANXA2, ARPC5, B2M, CAMP, CCT2, CCT8, CTSD, CYB5R3, EEF1A1, EEF2, FCN1, FE
1.0000	1.01	ALDOA, ANXA2, ARPC5, B2M, CAMP, CCT2, CCT8, CTSD, CYB5R3, EEF1A1, EEF2, FCN1, FE
1.0000	1.00	ACTB, ANXA5, ARL6IP5, BCAP31, CCL5, CD36, CFL1, CTSD, EIF5A, ENO1, FLNA, G6PD, GN/
1.0000	1.00	ADAM10, ANK1, ANTXR2, ANXA7, ARL6IP5, ATP2A3, B2M, BCAP31, CANX, CD59, CDC42,
1.0000	1.22	B2M, CD226, HLA-A, HLA-B, PRDX1, TUBB
1.0000	1.22	B2M, CD226, CD36, HLA-A, HSPD1, TFRC
1.0000	1.22	ANXA2, CDC42, INPP5D, LTF, MYH9, TFRC
1.0000	1.22	ALDOA, ENO1, GAPDH, LDHA, PGK1, PKM
1.0000	1.22	ALDOA, ENO1, GAPDH, LDHA, PGK1, PKM
1.0000	1.22	GNAQ, GSTP1, NPM1, PPIA, PTPRJ, YWHAG
1.0000	1.22	AACS, ADH5, CD36, GSTP1, PNPLA8, PTGS1
1.0000	1.22	ALDOA, ENO1, GAPDH, LDHA, PGK1, PKM
1.0000	1.22	ANXA2, CTSD, LRBA, RAB1B, RAB33B, UBQLN1
1.0000	1.22	ACTB, CDC42, HNRNPU, RCC2, TTLL12, YWHAE
1.0000	1.22	HSP90AA1, MSN, NPM1, PLEK, RHOA, RTN4
1.0000	1.22	ALDOA, ENO1, GAPDH, LDHA, PGK1, PKM
1.0000	1.22	ALDOA, ENO1, GAPDH, LDHA, PGK1, PKM
1.0000	1.22	ALDOA, ENO1, GAPDH, LDHA, PGK1, PKM
1.0000	1.22	ALDOA, ENO1, GAPDH, LDHA, PGK1, PKM
1.0000	1.22	ATP2A3, CD36, LRP1, SLC2A1, SLC2A3, TFRC
1.0000	1.22	ARL6IP5, CD36, ITGB1, PNPLA8, SLC2A1, THBS1
1.0000	1.22	ACTB, ANXA2, CD36, ITGB3, LRP1, OSBPL2
1.0000	1.22	ILK, LRP1, S100A8, S100A9, SOD1, VIM
1.0000	1.22	ADAM10, CDC42, KRT17, RHOA, S100A8, S100A9
1.0000	1.22	DHX9, FLOT1, G3BP1, GAPDH, HSP90AA1, HSPD1
1.0000	1.22	DHX9, FLOT1, G3BP1, GAPDH, HSP90AA1, HSPD1
1.0000	1.22	DHX9, FLOT1, G3BP1, GAPDH, HSP90AA1, HSPD1
1.0000	1.22	GNAI2, NCL, PKM, RAB10, SLC2A1, YWHAG
1.0000	1.22	CCL5, CD47, CDC42, GAPDH, SYNCRIP, VIM
1.0000	1.22	CALM3, GNAI2, PPIA, PTBP1, YWHAB, YWHAE
1.0000	1.22	CDC42, CFL1, EEF1A1, HSPA8, LTF, STOM
1.0000	1.22	ADAM10, CDC42, KRT17, RHOA, S100A8, S100A9
1.0000	1.22	ALDOA, ENO1, GAPDH, LDHA, PGK1, PKM
1.0000	1.22	ALDOA, ENO1, GAPDH, LDHA, PGK1, PKM
1.0000	1.22	BCAP31, DAZAP1, DSG1, ITGB1, KRT9, SOD1

1.0000	1.22	B2M, CDC42, ITGB3, KRT2, RTN4, THBS1
1.0000	1.22	CCL5, CD36, ITGB3, NCL, PECAM1, PTPRJ
1.0000	1.22	CCL5, DHX9, FCN1, LTF, PPIA, STOM
1.0000	1.22	ANXA2, ANXA5, CD9, GP1BA, KRT1, THBS1
1.0000	1.22	FLNA, HNRNPU, HSPA1B, RHOA, TUBB, TUBB1
1.0000	1.22	CCT2, CCT4, HNRNPA2B1, HSP90AA1, TCP1, XRCC5
1.0000	1.22	ANXA2, CD9, GP1BA, KRT1, MMRN1, THBS1
1.0000	1.22	GNAI2, GNG5, ILK, ITGB1, RAB10, RHOA
1.0000	1.22	CCL5, CD47, CDC42, GAPDH, SYNCRIP, VIM
1.0000	1.22	GNAI2, ITGB3, NCL, PKM, RAB10, YWHAG
1.0000	1.22	ATP2A3, CD36, LRP1, SLC2A1, SLC2A3, TFRC
1.0000	1.22	ANXA2, CD9, GP1BA, KRT1, MMRN1, THBS1
1.0000	1.22	ANXA2, FLOT1, ITGB1, SLC2A1, SRI, VCL
1.0000	1.22	DSC1, DSG1, DSP, FCER1G, RHOA, SLC2A3
1.0000	1.22	ALDOA, GAPDH, RCC2, S100A8, S100A9, TTLL12
1.0000	1.22	ALDOA, CANX, FCN1, G6PD, KRT1, SLC2A3
1.0000	1.22	DSP, HSP90AA1, KRT15, KRT5, VIM, YWHAE
1.0000	0.99	ACTB, ANXA2, CCL5, CCT2, CCT4, CCT6A, CCT7, CCT8, CD36, CSDE1, DHX9, EEF1A1, EEF2,
1.0000	0.99	ACTB, ADAM10, ANXA2, ANXA5, ATP2A3, ATP5F1B, B2M, CALM3, CAMP, CD226, CD36, (
1.0000	0.99	ACTB, ADH5, ANXA2, ANXA5, ARL6IP5, ATP2A3, B2M, BCAP31, CAMP, CANX, CCL5, CD15
1.0000	0.99	ARPC5, CD36, CD47, CDC42, F11R, FLNA, FLOT1, FSCN1, G3BP1, HNRNPU, HSP90AA1, HS
1.0000	0.98	ANXA2, ARL6IP5, ATP2A3, B2M, BCAP31, CALM3, CCL5, CCT2, CCT4, CD36, CDC42, CTSD,
1.0000	1.01	AACS, ADH5, ALDOA, ATIC, CD36, ENO1, GAPDH, GSTP1, LDHA, LDHB, OSBPL2, PGK1, PKI
1.0000	1.01	ARL6IP5, BCAP31, CCL5, CD36, CTSD, EIF5A, HSPD1, INPP5D, ITGA6, ITGB1, LRP1, RHOA,
1.0000	1.01	AACS, ADH5, ALDOA, ATIC, CD36, ENO1, GAPDH, GSTP1, LDHA, LDHB, OSBPL2, PGK1, PKI
1.0000	1.01	AACS, ADH5, ALDOA, ATIC, CD36, ENO1, GAPDH, GSTP1, LDHA, LDHB, OSBPL2, PGK1, PKI
1.0000	1.01	ATP2A3, B2M, BCAP31, CALM3, CCL5, FLNA, GNAI2, GP1BA, GP1BB, ITGB3, LTF, S100A8,
1.0000	1.01	ALDOA, ARPC5, CCT8, CTSD, EEF1A1, EEF2, FCN1, GSTP1, HSP90AA1, HSPA1B, HSPA8, JU
1.0000	1.01	CDC42, EEF1A1, EEF2, GNAI2, GNAQ, HSP90AA1, RAB10, RAB1B, RAB27B, RAB33B, RAC2
1.0000	1.01	CDC42, EEF1A1, EEF2, GNAI2, GNAQ, HSP90AA1, RAB10, RAB1B, RAB27B, RAB33B, RAC2
1.0000	1.01	CDC42, EEF1A1, EEF2, GNAI2, GNAQ, HSP90AA1, RAB10, RAB1B, RAB27B, RAB33B, RAC2
1.0000	0.98	AACS, ACTB, ADH5, ALDOA, ANK1, ANXA11, ANXA2, ANXA5, ARPC5, ATIC, B2M, BCAP31,
1.0000	0.97	ACTB, ADAM10, ADH5, ANPEP, ANXA2, ARL6IP5, ATP2A3, B2M, BCAP31, CALM3, CAMP,
1.0000	0.97	AACS, ACTB, ADAM10, ANXA2, ARL6IP5, ATP2A3, ATP5F1B, B2M, BCAP31, CALM3, CAMI
1.0000	1.04	ARL6IP5, CD36, CD47, CLIC1, ITGB1, PCYOX1, PNPLA8, SLC2A1, SLC2A3, VDAC2
1.0000	1.04	ACTB, B2M, FCER1G, INPP5D, ITGB1, PRDX2, PTPRJ, RHOA, SOD1, SPN
1.0000	1.04	CCL5, CD151, HLA-A, HSPD1, INPP5D, MSN, PRDX2, RAC2, SPN, TFRC
1.0000	1.04	ANPEP, F11R, FCN1, HSPA1B, ITGB1, ITGB3, KRT6A, RPSA, SELPLG, TFRC
1.0000	1.04	ANXA2, DHX9, EIF5A, HNRNPU, ITGA6, JUP, KANSL3, NCL, NPM1, PCBP1
1.0000	1.04	CCL5, CD151, HLA-A, HSPD1, INPP5D, MSN, PRDX2, RAC2, SPN, TFRC
1.0000	1.04	CCL5, CD47, DHX9, GSTP1, KRT1, LBP, PSMA6, S100A8, S100A9, SOD1
1.0000	1.04	CDC42, HINT1, HSP90AA1, HSPA1B, NPM1, PPIA, RPL11, RPS15, UBE2L3, UBQLN1
1.0000	1.04	ARPC5, CD47, CDC42, F11R, FLNA, MYADM, PFN1, PLEK, RHOA, SPTBN1
1.0000	1.04	ANK1, DSG1, G6PD, GNAI2, GNAQ, GNG5, JUP, MYH9, OSBPL2, RHOA
1.0000	1.04	B2M, CALM3, CD59, CLTA, HLA-A, HLA-B, M6PR, RAB10, RAB1B, RAB27B
1.0000	1.04	CDC42, CFL1, EEF1A1, ITGB1, ITGB3, PLEK, PTPRJ, RHOA, SPTBN1, TLN1
1.0000	0.97	ACTB, ADAM10, ALDOA, ANK1, ANPEP, ANTXR2, ANXA11, ANXA2, ANXA5, ANXA7, ARL6I

1.0000	0.97	ANTXR2, ANXA7, ARL6IP5, ATP2A3, B2M, BCAP31, CANX, CD59, CDC42, CYB5R3, CYBB, E
1.0000	0.97	ANTXR2, ANXA7, ARL6IP5, ATP2A3, B2M, BCAP31, CANX, CD59, CDC42, CYB5R3, CYBB, E
1.0000	0.96	ACTB, ADAM10, ADH5, ANPEP, ANXA2, ANXA7, ARL6IP5, ATP2A3, B2M, BCAP31, CALM3
1.0000	0.96	ACTB, ANXA2, ARL6IP5, ATP2A3, BCAP31, CALM3, CAMP, CCL5, CD36, CSDE1, CTSD, DHX
1.0000	0.97	ANXA2, ATP2A3, CALM3, CCL5, FLNA, G6PD, GNAI2, GP1BA, GP1BB, ITGB3, RHOA, SRI, U
1.0000	0.97	ADAM10, CCL5, FCER1G, ITGB3, LBP, PF4V1, PPIA, PTPRJ, RAC2, RHOA, S100A8, S100A9,
1.0000	0.97	ANXA11, ATIC, B2M, CALM3, CD36, CYBB, G6PD, PRDX1, PRDX2, RPLP0, S100A8, SOD1, T
1.0000	0.97	AACS, ADH5, ALDOA, CD36, ENO1, GAPDH, GSTP1, LDHA, LDHB, OSBPL2, PGK1, PKM, PN
1.0000	0.97	ARPC5, CD47, CDC42, F11R, FLNA, FSCN1, ITGB3, LRP1, MYADM, PFN1, PLEK, RAC2, RHO
1.0000	0.97	ADAM10, CCL5, FCER1G, ITGB3, LBP, PF4V1, PPIA, PTPRJ, RAC2, RHOA, S100A8, S100A9,
1.0000	0.97	CD36, CSDE1, DHX9, ENO1, FLNA, HNRNPA2B1, HNRNPC, HNRNPU, HSPA8, NPM1, PTBP
1.0000	0.97	CD36, CSDE1, DHX9, ENO1, FLNA, HNRNPA2B1, HNRNPC, HNRNPU, HSPA8, NPM1, PTBP
1.0000	0.97	ANXA11, CALM3, CDC42, CFL1, CLTA, GNAI2, HNRNPU, MYH9, RAB10, RCC2, RHOA, SPTE
1.0000	0.97	ADAM10, B2M, BCAP31, CD59, CDC42, HLA-A, HLA-B, QSOX1, RAB10, RAB1B, RAB33B, R
1.0000	0.97	ACTB, ANXA5, CLIC1, HSPA1B, HSPA8, ITGA2B, KRT1, LGALS3BP, MSN, PFN1, SLC2A1, ST
1.0000	0.94	ADH5, B2M, CAMP, CCL5, CD226, CD36, CD47, CDC42, CFL1, CYBB, DCD, DDX60L, DHX9,
1.0000	0.94	ACTB, ANXA2, ANXA5, ARL6IP5, ATP2A3, BCAP31, CALM3, CCL5, CCT2, CCT4, CDC42, CTS
1.0000	0.94	ADH5, B2M, CAMP, CCL5, CD226, CD36, CD47, CDC42, CFL1, CYBB, DCD, DDX60L, DHX9,
1.0000	0.94	ACTB, ADAM10, ANK1, ARPC5, CALM3, CANX, CCT8, CD36, CDC42, CFL1, CYBB, EEF1A1, F
1.0000	0.94	ADAM10, ANPEP, ANXA2, ARL6IP5, ATP2A3, BCAP31, CANX, CASP14, CPN1, CTSD, DCD, E
1.0000	1.22	FLOT1, ITGB1, ITGB3
1.0000	1.22	HSP90AA1, RHOA, YWHAE
1.0000	1.22	MATR3, NASP, RTN4
1.0000	1.22	B2M, HLA-A, HLA-B
1.0000	1.22	B2M, HLA-A, HLA-B
1.0000	1.22	B2M, HLA-A, HLA-B
1.0000	1.22	ITGB1, RHOA, THBS1
1.0000	1.22	CAMP, H2BC21, LTF
1.0000	1.22	CAMP, H2BC21, LTF
1.0000	1.22	FCER1G, LBP, RAC2
1.0000	1.22	CAMP, H2BC21, LTF
1.0000	1.22	CD226, HLA-A, HSPD1
1.0000	1.22	B2M, HLA-A, HLA-B
1.0000	1.22	B2M, CTSD, FCER1G
1.0000	1.22	CD47, CD99, GP1BA
1.0000	1.22	INPP5D, PRDX2, SPN
1.0000	1.22	CD226, HLA-A, HLA-B
1.0000	1.22	ENO1, PGK1, PKM
1.0000	1.22	ACTB, TNKS1BP1, XRCC5
1.0000	1.22	DHX9, HNRNPA2B1, NPM1
1.0000	1.22	CYB5R3, G6PD, SOD1
1.0000	1.22	ENO1, PGK1, PKM
1.0000	1.22	ENO1, PGK1, PKM
1.0000	1.22	SOD1, SRI, TFRC
1.0000	1.22	ANK1, BCAP31, RAB1B
1.0000	1.22	HSP90AA1, SLC2A1, XRCC5
1.0000	1.22	RAB10, RTN3, RTN4

1.0000	1.22	FLNA, GNAI2, ITGB3
1.0000	1.22	ITGB1, ITGB3, MYADM
1.0000	1.22	FLOT1, MYL6, RHOA
1.0000	1.22	B2M, CD36, SOD1
1.0000	1.22	ARL6IP5, B2M, ITGB1
1.0000	1.22	CYB5R3, G6PD, SOD1
1.0000	1.22	CDC42, HNRNPU, RCC2
1.0000	1.22	GNAQ, ITGB1, NPM1
1.0000	1.22	RHOA, SRI, THBS1
1.0000	1.22	RHOA, SRI, THBS1
1.0000	1.22	RHOA, SRI, THBS1
1.0000	1.22	JUP, RHOA, THBS1
1.0000	1.22	JUP, RHOA, THBS1
1.0000	1.22	ITGB1, ITGB3, THBS1
1.0000	1.22	ANXA2, ENO1, MYH9
1.0000	1.22	RAP1B, RPLP0, SOD1
1.0000	1.22	CD36, SLC2A1, THBS1
1.0000	1.22	PNPLA8, PRDX6, YWHAH
1.0000	1.22	CYB5R3, G6PD, SOD1
1.0000	1.22	CYB5R3, G6PD, SOD1
1.0000	1.22	B2M, HLA-A, HLA-B
1.0000	1.22	B2M, HLA-A, HLA-B
1.0000	1.22	B2M, CTSD, FCER1G
1.0000	1.22	INPP5D, ITGB1, PTPRJ
1.0000	1.22	BCAP31, CANX, UBQLN1
1.0000	1.22	HSPA1B, RHOA, YWHAH
1.0000	1.22	ACTB, ATP2A3, B2M
1.0000	1.22	CCL5, ITGB3, PPIA
1.0000	1.22	CALM3, CDC42, RHOA
1.0000	1.22	DHX9, FLOT1, HSP90AA1
1.0000	1.22	FCN1, HSPA1B, LBP
1.0000	1.22	DHX9, FLOT1, HSP90AA1
1.0000	1.22	FCN1, HSPA1B, LBP
1.0000	1.22	DHX9, FLOT1, HSP90AA1
1.0000	1.22	CD226, HLA-A, HSPD1
1.0000	1.22	FCN1, HSPA1B, LBP
1.0000	1.22	ARL6IP5, ITGB1, THBS1
1.0000	1.22	CCL5, HLA-A, TFRC
1.0000	1.22	HNRNPA2B1, NPM1, PTBP1
1.0000	1.22	HSP90AA1, TFRC, TNKS1BP1
1.0000	1.22	HSP90AA1, TFRC, TNKS1BP1
1.0000	1.22	CCL5, FERMT3, ITGB1
1.0000	1.22	CCL5, F11R, MMRN1
1.0000	1.22	RHOA, SRI, THBS1
1.0000	1.22	FCN1, FLNA, VCL
1.0000	1.22	GNAI2, YWHAB, YWHAE
1.0000	1.22	CALM3, PPIA, PTBP1

1.0000	1.22	GNAI2, YWHAB, YWHAE
1.0000	1.22	CAMP, CCL5, FCER1G
1.0000	1.22	BCAP31, CANX, UBQLN1
1.0000	1.22	CCL5, HLA-A, TFRC
1.0000	1.22	HSPD1, LBP, THBS1
1.0000	1.22	CAMP, CCL5, FCER1G
1.0000	1.22	ANXA2, FLNA, RPL11
1.0000	1.22	CD226, HLA-A, HLA-B
1.0000	1.22	ADAM10, RTN3, RTN4
1.0000	1.22	ADAM10, RTN3, RTN4
1.0000	1.22	HSPD1, LBP, THBS1
1.0000	1.22	HSPD1, LBP, THBS1
1.0000	1.22	GSTP1, PPIA, PTPRJ
1.0000	1.22	JUP, RHOA, THBS1
1.0000	1.22	CD36, SLC2A1, THBS1
1.0000	1.22	EEF1A1, HSPA8, STOM
1.0000	1.22	EEF1A1, HSPA8, STOM
1.0000	1.22	CCL5, LTF, PPIA
1.0000	1.22	CDC42, MSN, RHOA
1.0000	1.22	ACTB, INPP5D, RHOA
1.0000	1.22	B2M, ITGB1, RHOA
1.0000	1.22	CLIC1, ILK, LTF
1.0000	1.22	CLIC1, ILK, LTF
1.0000	1.22	HLA-A, HLA-B, TTLL12
1.0000	1.22	GNAI2, RAB33B, RAP1B
1.0000	1.22	ENO1, HSP90AA1, RHOA
1.0000	1.22	HLA-A, RHOA, SPN
1.0000	1.22	RAP1B, RPLP0, SOD1
1.0000	1.22	ADAM10, CCT8, RTN4
1.0000	1.22	HNRNPA2B1, NPM1, PTBP1
1.0000	1.22	G6PD, RTN4, SOD1
1.0000	1.22	CCL5, HLA-A, TFRC
1.0000	1.22	HNRNPA2B1, NPM1, PTBP1
1.0000	1.22	ARL6IP5, B2M, ITGB1
1.0000	1.22	GNAI2, RAB33B, RAP1B
1.0000	1.22	CCT2, HSP90AA1, HSPD1
1.0000	1.22	INPP5D, PRDX2, SPN
1.0000	1.22	CALM3, ITGB3, SRI
1.0000	1.22	CALM3, CDC42, RHOA
1.0000	1.22	CD47, CDC42, RHOA
1.0000	1.22	FLOT1, ITGB1, ITGB3
1.0000	1.22	ANXA11, CALM3, THBS1
1.0000	1.22	ACTB, HNRNPU, RCC2
1.0000	1.22	CDC42, HNRNPU, RCC2
1.0000	1.22	G6PD, HNRNPU, ITGB1
1.0000	1.22	G6PD, HNRNPU, ITGB1
1.0000	1.22	FLOT1, MYL6, RHOA

1.0000	1.22	ILK, PFN1, RHOA
1.0000	1.22	HSPA8, M6PR, RTN4
1.0000	1.22	ENO1, PGK1, PKM
1.0000	1.22	ENO1, PGK1, PKM
1.0000	1.22	ENO1, PGK1, PKM
1.0000	1.22	EEF1A1, HSP90AA1, HSPA8
1.0000	1.22	ENO1, PGK1, PKM
1.0000	1.22	CAMP, CCL5, CD47
1.0000	1.22	BCAP31, RAB10, RTN4
1.0000	1.22	F11R, ITGB3, SLC2A1
1.0000	1.22	CAMP, CCL5, CD47
1.0000	1.22	RAB10, RTN3, RTN4
1.0000	1.22	FSCN1, MSN, RHOA
1.0000	1.22	FSCN1, MSN, RHOA
1.0000	1.22	FSCN1, MSN, RHOA
1.0000	1.22	HSPA8, M6PR, RTN4
1.0000	1.22	DSP, JUP, YWHAE
1.0000	1.22	ILK, LRP1, THBS1
1.0000	1.22	DSP, JUP, VCL
1.0000	1.22	CDC42, MSN, RHOA
1.0000	1.22	ILK, ITGB3, THBS1
1.0000	1.22	CYBB, JUP, RPLP0
1.0000	1.22	CCL5, EIF5A, HSP90AA1
1.0000	1.22	CALM3, DSP, JUP
1.0000	1.22	CDC42, PFN1, RHOA
1.0000	1.22	CD36, SLC2A1, THBS1
1.0000	1.22	ACTB, NPM1, RCC2
1.0000	1.22	CD36, SLC2A1, THBS1
1.0000	1.22	INPP5D, LTF, PRDX2
1.0000	1.22	ACTB, INPP5D, RHOA
1.0000	1.22	PPIA, SOD1, UBQLN1
1.0000	1.22	CD99, LBP, RAC2
1.0000	1.22	CD99, LBP, RAC2
1.0000	1.22	CYB5R3, G6PD, SOD1
1.0000	1.22	CYB5R3, G6PD, SOD1
1.0000	1.22	FSCN1, ITGB3, LRP1
1.0000	1.22	ITGB1, LRP1, SPTBN1
1.0000	1.22	PPIA, SOD1, UBQLN1
1.0000	1.22	ANXA2, ENO1, MYH9
1.0000	1.22	GNAI2, RAB33B, RAP1B
1.0000	1.22	RAC2, STOM, UBE2L3
1.0000	1.22	GSTP1, LBP, LTF
1.0000	1.22	ACTB, INPP5D, RHOA
1.0000	1.22	ITGB1, LRP1, SPTBN1
1.0000	1.22	CALM3, G6PD, SRI
1.0000	1.22	CCL5, CD9, THBS1
1.0000	1.22	CCL5, CD9, THBS1

1.0000	1.22	ANXA2, CD36, LRP1
1.0000	1.22	CCL5, PF4V1, RHOA
1.0000	1.22	CCL5, PF4V1, RHOA
1.0000	1.22	GNAI2, GNG5, ITGB1
1.0000	1.22	ITGB3, MYH9, SOD1
1.0000	1.22	ACTB, DHX9, NPM1
1.0000	1.22	ENO1, PPIA, VDAC2
1.0000	1.22	CALM3, SRI, UBQLN1
1.0000	1.22	PSMA5, PSMA6, UBQLN1
1.0000	1.22	ACTB, HNRNPU, RCC2
1.0000	1.22	CD53, MYH9, PTPRJ
1.0000	1.22	BCAP31, HLA-A, RAB27B
1.0000	1.22	ADAM10, CLTA, RAB27B
1.0000	1.22	ACTB, RAC2, TPM4
1.0000	1.22	DSP, JUP, VCL
1.0000	1.22	CD9, M6PR, TFRC
1.0000	1.22	CALM3, CLTA, RAB27B
1.0000	1.22	CD59, GP1BA, RAB27B
1.0000	1.22	CANX, HNRNPU, SOD1
1.0000	1.22	ATP2A3, SRI, TMEM109
1.0000	1.22	BCAP31, CD59, RAB1B
1.0000	1.22	FLOT1, G6PD, PATJ
1.0000	1.22	B2M, HLA-A, HLA-B
1.0000	1.22	B2M, HLA-A, HLA-B
1.0000	1.22	BCAP31, CANX, TMX2
1.0000	1.22	CANX, HSPA8, ITGB3
1.0000	1.22	CDC42, HNRNPU, RCC2
1.0000	1.22	CALM3, CLTA, RAB27B
1.0000	1.22	ENO1, PKM, TKT
1.0000	1.22	ITGB1, ITGB3, THBS1
1.0000	1.22	FCN1, HLA-A, HLA-B
1.0000	1.22	ANXA2, GAPDH, LTF
1.0000	1.22	CD36, LGALS3BP, LRP1
1.0000	1.22	CCL5, ITGB3, PF4V1
1.0000	1.22	CD36, OSBPL2, SLC2A1
1.0000	1.22	EEF1A1, MYH9, SPTBN1
1.0000	1.22	LTF, PF4V1, THBS1
1.0000	1.22	GSR, PGK1, QSOX1
1.0000	1.22	ANXA2, GAPDH, LTF
1.0000	1.22	CANX, HSPD1, LRP1
1.0000	1.22	CALM3, CANX, FLOT1
1.0000	1.22	HNRNPA2B1, HNRNPU, PTBP1
1.0000	1.22	HNRNPA2B1, NCL, XRCC5
1.0000	1.22	ITGB1, RPSA, THBS1
1.0000	1.22	ALDOA, G6PD, SLC2A3
1.0000	1.22	G6PD, GAPDH, GSR
1.0000	1.22	ANXA2, GAPDH, LTF

1.0000	1.22	DHX9, HNRNPA2B1, MATR3
1.0000	1.22	DHX9, HNRNPU, STOM
1.0000	1.22	CD36, HSPD1, THBS1
1.0000	1.22	CD36, HSPD1, THBS1
1.0000	1.22	CD47, DSP, JUP
1.0000	0.93	ACTB, CALM3, CDC42, CFL1, FLNA, HNRNPU, HSPA1B, ITGB1, NASP, RCC2, RHOA, SPTBN1
1.0000	0.93	AACS, ADH5, ATP5F1B, CD36, CDC42, CYB5R3, G6PD, GSTP1, INPP5D, LRP1, OSBPL2, PLE
1.0000	0.93	CCL5, CD226, CD47, DHX9, FCN1, GSTP1, HLA-A, HLA-B, HSP90AA1, KRT1, LBP, MATR3, P
1.0000	0.93	ATP2A3, ATP5F1B, B2M, BCAP31, CALM3, CCL5, FLNA, GNAI2, GP1BA, GP1BB, ITGB3, LTF
1.0000	0.93	CCT2, CCT4, CCT6A, CCT7, CCT8, DSG1, FLNA, FLOT1, GAPDH, GNAQ, HSP90AA1, HSPA1B
1.0000	0.93	ATP2A3, ATP5F1B, B2M, BCAP31, CALM3, CCL5, FLNA, GNAI2, GP1BA, GP1BB, ITGB3, LTF
1.0000	0.93	ACTB, ARL6IP5, CCL5, DHX9, FLNA, FLOT1, HNRNPU, HSPA1B, HSPA8, JUP, MSN, NPM1, F
1.0000	0.93	ATP2A3, ATP5F1B, B2M, BCAP31, CALM3, CCL5, FLNA, GNAI2, GP1BA, GP1BB, ITGB3, LTF
1.0000	0.93	ADH5, CYB5R3, CYBB, G6PD, GAPDH, GSR, GSTP1, LDHA, LDHB, PCYOX1, PGK1, PRDX1, P
1.0000	0.94	ACTB, ADAM10, ADH5, ALDOA, ANK1, ANPEP, ANTXR2, ANXA11, ANXA2, ANXA5, ANXA7
1.0000	0.91	CAMP, CCL5, CD226, CD47, CDC42, CYBB, DHX9, ENDOD1, FCER1G, FCN1, G3BP1, GAPDH
1.0000	0.91	ANTXR2, ANXA7, ARL6IP5, ATP2A3, B2M, BCAP31, CANX, CD59, CDC42, CYB5R3, CYBB, E
1.0000	0.97	ALDOA, ENO1, GAPDH, LDHA, LDHB, PGK1, PKM
1.0000	0.97	ACTB, H2BC21, HNRNPC, HNRNPU, KANSL3, NASP, NPM1
1.0000	0.97	CALM3, GNAI2, PPIA, PTBP1, PTPRJ, YWHAB, YWHAE
1.0000	0.97	F11R, ITGB3, JUP, KRT5, RHOA, SLC2A1, THBS1
1.0000	0.97	ILK, LRP1, RHOA, S100A8, S100A9, SOD1, VIM
1.0000	0.97	BCAP31, CANX, HSPA1B, PSMA5, PSMA6, RPL11, UBQLN1
1.0000	0.97	ATP5F1B, ITGB3, JUP, PFN1, RHOA, RTN4, THBS1
1.0000	0.97	ALDOA, ENO1, GALE, GAPDH, LDHA, PGK1, PKM
1.0000	0.97	CCL5, EEF1A1, HSPA8, LTF, PCBP1, PPIA, STOM
1.0000	0.97	ALDOA, ENO1, G6PD, GALE, GAPDH, PGK1, PKM
1.0000	0.97	B2M, CTSD, FCER1G, HLA-A, HLA-B, RAB10, THBS1
1.0000	0.97	ACTB, B2M, FCER1G, PRDX2, RHOA, SOD1, SPN
1.0000	0.97	CD36, CD47, DHX9, HLA-B, HSPD1, INPP5D, LBP
1.0000	0.97	CD36, CD47, DHX9, HLA-B, HSPD1, INPP5D, LBP
1.0000	0.97	CD36, CYBB, DHX9, LBP, PTPRJ, SPN, THBS1
1.0000	0.97	GNAQ, GSTP1, HNRNPU, NPM1, PPIA, PTPRJ, YWHAG
1.0000	0.97	CD47, DSP, FLOT1, ITGB1, ITGB3, JUP, MYADM
1.0000	0.97	CALM3, GNAI2, PLEK, PPIA, PTBP1, YWHAB, YWHAE
1.0000	0.97	BCAP31, CANX, HSPA1B, PSMA5, PSMA6, RPL11, UBQLN1
1.0000	0.97	ACTB, CALM3, ITGB1, NASP, NPM1, RCC2, UBE2L3
1.0000	0.97	CALM3, GNAI2, MYH9, PLEK, RAB10, RAC2, RAP1B
1.0000	0.97	CCL5, CDC42, CFL1, EEF1A1, HSPA8, LTF, STOM
1.0000	0.97	B2M, LTF, S100A8, S100A9, SOD1, SRI, TFRC
1.0000	0.97	ACTB, HLA-B, INPP5D, LTF, PRDX2, RHOA, SOD1
1.0000	0.97	CD36, CYBB, DHX9, LBP, PTPRJ, SPN, THBS1
1.0000	0.97	B2M, CALM3, CD59, CLTA, HLA-A, HLA-B, RAB27B
1.0000	0.97	B2M, HLA-A, HLA-B, ITGB1, LTF, RAB10, TFRC
1.0000	0.97	ADAM10, RPL8, RPLP0, RTN3, RTN4, SPTBN1, YWHAZ
1.0000	0.97	CALM3, CCL5, LTF, NPM1, RAC2, YWHAB, YWHAG
1.0000	0.97	ANXA2, CD47, DSP, ITGB1, JUP, RAB10, RPSA

1.0000	0.97	GSTP1, LRP1, LTF, PF4V1, PPIA, THBS1, TKT
1.0000	0.91	ACTB, ANXA2, ARL6IP5, ATP2A3, BCAP31, CALM3, CAMP, CCL5, CCT2, CCT4, CCT6A, CCT7
1.0000	0.91	ACTB, ANXA2, CCT2, CCT4, CCT6A, CCT7, CCT8, CD36, CSDE1, DAZAP1, DHX9, EIF5A, END
1.0000	0.92	ACTB, ALDOA, ANXA2, ATIC, ATP5F1B, CCL5, CCT2, CCT4, CCT6A, CCT7, CCT8, CD36, CD4
1.0000	0.90	B2M, CD226, CD36, FCN1, HLA-A, HLA-B, HSPD1, LBP, PTPRJ, RAC2, TFRC
1.0000	0.90	CCL5, CD226, CD47, DHX9, FCN1, HSP90AA1, LBP, MATR3, S100A8, S100A9, XRCC5
1.0000	0.90	CALM3, CCL5, CDC42, HSP90AA1, ITGB3, LTF, NPM1, PPIA, RHOA, THBS1, XRCC5
1.0000	0.90	CCL5, CD226, DHX9, FCN1, HLA-A, HLA-B, HSP90AA1, LBP, MATR3, TTLL12, XRCC5
1.0000	0.90	CALM3, CPN1, CYBB, DSG1, HNRNPU, HSPA1B, HSPA8, RHOA, THBS1, UBE2L3, YWHAH
1.0000	0.90	ACTB, ANXA2, ATP2A3, B2M, CALM3, CDC42, DHX9, FLOT1, LRP1, RPL11, SRI
1.0000	0.90	CCL5, CD151, GSTP1, HLA-A, HSPD1, INPP5D, MSN, PRDX2, RAC2, SPN, TFRC
1.0000	0.90	CD36, CYBB, G6PD, GNAI2, GSTP1, PRDX1, PRDX2, RAC2, RHOA, SOD1, THBS1
1.0000	0.90	CCT2, CCT4, CCT6A, CCT7, CCT8, FLNA, HSP90AA1, JUP, NPM1, TCP1, TFRC
1.0000	0.90	CD36, CYBB, GNAI2, ITGB3, LRP1, NCL, PKM, RAB10, SLC2A1, VIM, YWHAG
1.0000	0.90	B2M, CD36, CD9, CYBB, HLA-A, HLA-B, LRP1, M6PR, RAB10, RAC2, TFRC
1.0000	0.90	ACTB, CDC42, CSDE1, DHX9, G3BP1, HNRNPU, NCL, PCBP1, PSMA6, RPLP0, TUBB
1.0000	0.90	ACTB, CDC42, CSDE1, DHX9, G3BP1, HNRNPU, NCL, PCBP1, PSMA6, RPLP0, TUBB
1.0000	0.90	ANK1, DSG1, EEF1A1, G6PD, GNAI2, GNAQ, GNG5, JUP, MYH9, OSBPL2, RHOA
1.0000	0.90	ADH5, ANPEP, CPN1, FLG2, HRNR, LTF, MATR3, S100A8, S100A9, SF3A3, SOD1
1.0000	0.91	ACTB, ADAM10, ALDOA, ANK1, ANXA2, ARPC5, B2M, BCAP31, CALM3, CAVIN2, CCT2, CC
1.0000	0.89	ACTB, ANXA2, CCL5, CCT2, CCT4, CCT6A, CCT7, CCT8, CD36, CSDE1, DHX9, EEF2, EIF5A, E
1.0000	0.89	ACTB, ANXA2, CCL5, CCT2, CCT4, CCT6A, CCT7, CCT8, CD36, CSDE1, DHX9, EEF2, EIF5A, E
1.0000	0.89	ACTB, ADAM10, B2M, CAMP, CCL5, CD47, CD9, CDC42, DHX9, GNAI2, GNG5, GSTP1, HLA
1.0000	0.90	ACTB, ANXA11, CASP14, CAVIN2, CCT4, CCT8, DAZAP1, DHX9, GNAI2, H2BC21, HINT1, HN
1.0000	0.90	AACS, ACTB, ATP2A3, ATP5F1B, CAMP, CCT2, CCT4, CCT6A, CCT7, CCT8, CDC42, DDX60L,
1.0000	0.87	CD226, CD36, CD47, FCER1G, FCN1, FLOT1, HLA-A, HSPA1B, HSPD1, INPP5D, LBP, LTF, PT
1.0000	0.87	B2M, CANX, CCT2, CCT4, CCT6A, CCT7, CCT8, HSP90AA1, HSPA1B, HSPA8, HSPA9, HSPD1
1.0000	0.87	ARL6IP5, CD36, G6PD, GSR, GSTP1, HSPA1B, KRT1, PNPLA8, PPIA, PRDX1, PRDX2, PRDX6,
1.0000	0.87	ALDOA, ARPC5, CD47, CDC42, CFL1, F11R, FLNA, FSCN1, MYADM, PFN1, PLEK, RAC2, RH
1.0000	0.87	ADAM10, CCL5, CD226, CD47, DHX9, FCN1, FLNA, HSP90AA1, LBP, MATR3, RAC2, S100A
1.0000	0.87	CCT2, CCT4, CCT6A, CCT7, CCT8, CD36, FLNA, HNRNPU, HSP90AA1, JUP, NPM1, RPL11, T
1.0000	0.87	ARL6IP5, BCAP31, CCL5, CTSD, EIF5A, HSPD1, INPP5D, ITGA6, ITGB1, RHOA, RPL11, S100
1.0000	0.87	ARL6IP5, BCAP31, CCL5, CTSD, EIF5A, HSPD1, INPP5D, ITGA6, ITGB1, RHOA, RPL11, S100
1.0000	0.87	CDC42, EEF1A1, EEF2, GNAI2, GNAQ, GNG5, RAB10, RAB1B, RAB27B, RAB33B, RAC2, RA
1.0000	0.90	AACS, ACTB, ADAM10, ADH5, ALDOA, ANPEP, ANXA2, ANXA7, ARL6IP5, ATIC, ATP2A3, A
1.0000	0.88	ANXA2, CALM3, CD36, CSDE1, DHX9, ENO1, FLNA, G6PD, GAPDH, GNAI2, GNAQ, GSTP1,
1.0000	0.88	ACTB, CALM3, CAMP, CCL5, CD36, FLOT1, G6PD, GNAI2, GNAQ, GSTP1, HSP90AA1, HSPA
1.0000	0.85	CD36, CSDE1, DHX9, ENO1, FLNA, GAPDH, GSTP1, HNRNPA2B1, HNRNPC, HNRNPU, HSP
1.0000	0.85	CCT2, CCT4, CCT6A, CCT7, CCT8, DSG1, FLNA, FLOT1, GAPDH, GNAQ, HSP90AA1, HSPA1B
1.0000	0.85	ANXA2, ANXA5, F11R, GAPDH, GNAI2, GNAQ, GSTP1, HNRNPU, LRP1, LTF, NPM1, PPIA, P
1.0000	0.85	ACTB, CALM3, CCL5, CDC42, EEF1A1, GNAQ, GSTP1, HNRNPU, HSP90AA1, ITGB3, LTF, NP
1.0000	0.88	ACTB, ALDOA, ANK1, ARPC5, CD47, CDC42, CFL1, DSP, F11R, FLNA, FSCN1, GAPDH, HNR
1.0000	0.88	ADH5, B2M, CAMP, CCL5, CD226, CD36, CD47, CDC42, CFL1, CYBB, DCD, DDX60L, DHX9,
1.0000	0.89	ACTB, ADAM10, ALDOA, ANK1, ANXA2, ARPC5, B2M, BCAP31, CALM3, CAVIN2, CCT2, CC
1.0000	Inf	AACS, ACTB, ADAM10, ADH5, ALDOA, ANK1, ANPEP, ANTXR2, ANXA11, ANXA2, ANXA5,
1.0000	Inf	AACS, ACTB, ADAM10, ADH5, ALDOA, ANK1, ANPEP, ANTXR2, ANXA11, ANXA2, ANXA5,
1.0000	0.84	ANXA5, CCL5, CFL1, ENO1, FLNA, GNAI2, GSTP1, HSPA1B, HSPA9, HSPD1, ITGA6, ITGB1,

1.0000	0.84	ANXA5, CCL5, CFL1, ENO1, FLNA, GNAI2, GSTP1, HSPA1B, HSPA9, HSPD1, ITGA6, ITGB1, L
1.0000	0.84	CSDE1, CTSD, FLNA, FSCN1, G3BP1, HNRNPU, HSPA1B, ITGB1, LRBA, MSN, NPM1, RAB1B
1.0000	0.86	ANXA2, ARL6IP5, ATP2A3, BCAP31, CALM3, CAMP, CCL5, CD36, CSDE1, CTSD, DHX9, EEF
1.0000	0.83	ADAM10, B2M, BCAP31, CD36, CD59, CDC42, CLTA, CSDE1, CYBB, FLNA, GNAQ, HLA-A, H
1.0000	0.86	ACTB, ADAM10, ANK1, ANXA2, CALM3, CAVIN2, CCT2, CD226, CDC42, CLTA, DHX9, DSP,
1.0000	0.80	BCAP31, CANX, HSPA1B, PSMA5, PSMA6, RPL11, UBE2L3, UBQLN1
1.0000	0.80	ITGA6, ITGB3, PTPRJ, RCC2, RHOA, THBS1, TLN1, VCL
1.0000	0.80	ACTB, CALM3, CDC42, HNRNPU, HSPA1B, NPM1, RCC2, RHOA
1.0000	0.80	ARL6IP5, ATP2A3, BCAP31, CTSD, HSPD1, RHOA, S100A8, S100A9
1.0000	0.80	ARL6IP5, ATP2A3, BCAP31, CTSD, HSPD1, RHOA, S100A8, S100A9
1.0000	0.80	ADAM10, ANXA2, CPN1, ENO1, MYH9, PGK1, SRGN, THBS1
1.0000	0.80	HSP90AA1, HSPA1B, NPM1, PPIA, RPL11, RPS15, UBE2L3, UBQLN1
1.0000	0.80	ARL6IP5, ATP2A3, BCAP31, CTSD, HSPD1, RHOA, S100A8, S100A9
1.0000	0.80	ACTB, ANXA2, ATP2A3, B2M, CDC42, FLOT1, LRP1, RPL11
1.0000	0.80	CYBB, GNAI2, ITGB3, NCL, PKM, RAB10, SLC2A1, YWHAG
1.0000	0.80	CCL5, CD226, DHX9, FCN1, HSP90AA1, LBP, MATR3, XRCC5
1.0000	0.80	B2M, G6PD, HSPA1B, HSPA9, INPP5D, PRDX1, PRDX2, SOD1
1.0000	0.80	ACTB, B2M, CCL5, CD47, HLA-A, HSPD1, RHOA, TFRC
1.0000	0.80	ANXA2, F11R, GAPDH, GNAI2, LRP1, LTF, RCC2, THBS1
1.0000	0.80	CAMP, CCL5, DHX9, EIF5A, GSTP1, HSPA1B, ILK, THBS1
1.0000	0.80	ITGA6, ITGB3, PTPRJ, RCC2, RHOA, THBS1, TLN1, VCL
1.0000	0.80	FSCN1, G3BP1, HNRNPU, HSPA1B, MSN, NPM1, RAB1B, RHOA
1.0000	0.80	HSP90AA1, HSPA1B, NPM1, PPIA, RPL11, RPS15, UBE2L3, UBQLN1
1.0000	0.80	ARL6IP5, ATP2A3, BCAP31, CTSD, HSPD1, RHOA, S100A8, S100A9
1.0000	0.80	ACTB, DHX9, H2AC20, H2BC21, HNRNPC, NASP, TCP1, TNKS1BP1
1.0000	0.80	ADAM10, BCAP31, CD9, CLTA, HSPA8, M6PR, RAB27B, TFRC
1.0000	0.80	ACTB, EEF1A1, FLOT1, MYADM, MYH9, SLC2A1, SPTBN1, TPM4
1.0000	0.80	CFL1, EEF1A1, ITGB1, ITGB3, PLEK, PTPRJ, RHOA, TLN1
1.0000	0.80	ANXA2, CCT2, CCT8, CYB5R3, HRNR, LYZ, PRDX6, TUBB
1.0000	0.80	HNRNPA2B1, HNRNPA3, HNRNPC, HNRNPF, HNRNPH1, HNRNPU, SF3A3, SYNCRIP
1.0000	0.80	CALM3, CCL5, GSTP1, LTF, NPM1, RAC2, YWHAB, YWHAG
1.0000	0.80	ADAM10, CDC42, ENO1, G6PD, HSPA1B, KRT17, NPM1, PTPRJ, RHOA, RTN4, S100A8, S10
1.0000	0.80	ACTB, B2M, CCL5, CD226, CD47, HLA-A, HSPD1, INPP5D, LBP, RHOA, TFRC, THBS1
1.0000	0.80	ALDOA, ENO1, G6PD, GALE, GAPDH, LDHA, NANS, PGK1, PKM, PLEK, SLC2A1, SLC2A3
1.0000	0.80	GNAI2, HNRNPU, HSPA1B, HSPA8, ITGB3, NCL, PKM, RAB10, RHOA, UBE2L3, YWHAG, YW
1.0000	0.80	ACTB, B2M, CCL5, CD47, HLA-A, HSPD1, PRDX2, RAC2, RHOA, SOD1, SPN, TFRC
1.0000	0.80	ACTB, CCT2, CCT4, CCT6A, CCT7, CCT8, DHX9, HNRNPA2B1, HSP90AA1, TCP1, TFRC, XRC
1.0000	0.80	ANXA2, ARL6IP5, ATP2A3, BCAP31, CTSD, GAPDH, HSPD1, LTF, RHOA, S100A8, S100A9, T
1.0000	0.80	ADAM10, CCL5, CD47, CD99, CDC42, F11R, ITGB3, MSN, PECAM1, RHOA, SPN, THBS1
1.0000	0.80	ARPC5, CD47, CDC42, F11R, FLNA, HSPA1B, HSPA8, MYADM, PFN1, PLEK, RHOA, SPTBN1
1.0000	0.80	ACTB, B2M, CCL5, CD226, CD47, HLA-A, HSPD1, INPP5D, LBP, PRDX2, RAC2, RHOA, SOD1
1.0000	0.80	ACTB, CALM3, CCL5, GNAQ, GSTP1, HSP90AA1, ITGB3, LTF, NPM1, PPIA, PTPRJ, RHOA, S
1.0000	0.80	CALM3, CCL5, CCT2, CCT4, CDC42, HNRNPA2B1, HSP90AA1, ITGB3, LTF, NPM1, PPIA, RH
1.0000	0.80	BCAP31, EIF5A, ENO1, GNAI2, GSTP1, HSPA1B, ITGA6, PPIA, PRDX2, RPL11, S100A8, S10
1.0000	0.82	ACTB, ANXA2, CCL5, CCT2, CCT4, CCT6A, CCT7, CCT8, CSDE1, DHX9, EEF2, EIF5A, HNRNP
1.0000	0.82	ANXA7, CASP14, CDC42, DSP, F11R, HNRNPH3, HRNR, KRT1, KRT10, KRT14, KRT15, KRT1
1.0000	0.82	ACTB, ANXA2, CCT2, CCT4, CCT6A, CCT7, CCT8, CSDE1, DAZAP1, DHX9, EIF5A, ENO1, HN

1.0000	0.79	B2M, CD226, CD36, CYBB, DHX9, FCER1G, FCN1, FLOT1, G3BP1, GAPDH, HLA-A, HSP90A/
1.0000	0.79	ACTB, B2M, G6PD, HNRNPU, HSPA9, INPP5D, ITGB1, ITGB3, LTF, PGK1, PRDX2, PTBP1, R(
1.0000	0.81	AACS, ACTB, ADAM10, ADH5, ALDOA, ANK1, ANTXR2, ANXA11, ANXA2, ANXA5, ANXA7, /
1.0000	0.81	B2M, CD226, HLA-A, HLA-B
1.0000	0.81	B2M, CD226, HLA-A, HLA-B
1.0000	0.81	FCN1, HSP90AA1, MATR3, XRCC5
1.0000	0.81	B2M, HLA-A, HLA-B, HSPD1
1.0000	0.81	B2M, FCER1G, HLA-A, HLA-B
1.0000	0.81	B2M, CTSD, FCER1G, THBS1
1.0000	0.81	GSTP1, LBP, S100A8, TFRC
1.0000	0.81	CCL5, S100A8, S100A9, THBS1
1.0000	0.81	CD47, CD59, HLA-A, HLA-B
1.0000	0.81	B2M, HLA-A, HLA-B, HSPD1
1.0000	0.81	B2M, HLA-A, HLA-B, HSPD1
1.0000	0.81	ACTB, HSPD1, TFRC, XRCC5
1.0000	0.81	CYB5R3, G6PD, OSBPL2, SOD1
1.0000	0.81	ATP2A3, HSPD1, S100A8, S100A9
1.0000	0.81	CALM3, HSP90AA1, HSPA9, VDAC2
1.0000	0.81	FLNA, HNRNPU, HSPA1B, RHOA
1.0000	0.81	FLNA, GNAI2, GNAQ, ITGB3
1.0000	0.81	ARL6IP5, PPIA, SOD1, UBQLN1
1.0000	0.81	ITGB1, ITGB3, RCC2, THBS1
1.0000	0.81	ANXA2, GAPDH, LTF, THBS1
1.0000	0.81	LRP1, S100A8, S100A9, VIM
1.0000	0.81	CD36, PNPLA8, SLC2A1, THBS1
1.0000	0.81	CD36, PNPLA8, SLC2A1, THBS1
1.0000	0.81	ANXA2, CD36, LRP1, OSBPL2
1.0000	0.81	ENO1, GALE, PGK1, PKM
1.0000	0.81	ADAM10, FLOT1, FSCN1, LRP1
1.0000	0.81	ANXA2, CD36, LRP1, OSBPL2
1.0000	0.81	CDC42, MSN, RAB10, RHOA
1.0000	0.81	PLEK, RAC2, RHOA, TLN1
1.0000	0.81	B2M, CD226, HLA-A, HLA-B
1.0000	0.81	HSPA1B, PPIA, RPL11, RPS15
1.0000	0.81	HSP90AA1, HSPA1B, PFN1, RHOA
1.0000	0.81	DHX9, G3BP1, HNRNPA2B1, XRCC5
1.0000	0.81	CALM3, SRI, UBQLN1, YWHAE
1.0000	0.81	CALM3, SRI, UBQLN1, YWHAE
1.0000	0.81	CD226, CD47, HLA-A, HSPD1
1.0000	0.81	CD226, CD47, HLA-A, HSPD1
1.0000	0.81	EIF5A, GSTP1, HSPA1B, ILK
1.0000	0.81	HNRNPA2B1, RPL11, RPS15, WBP11
1.0000	0.81	HSP90AA1, HSPA1B, THBS1, YWHAE
1.0000	0.81	CALM3, PLEK, PPIA, PTBP1
1.0000	0.81	ARL6IP5, PPIA, SOD1, UBQLN1
1.0000	0.81	ANXA2, GP1BA, KRT1, THBS1
1.0000	0.81	ATP5F1B, JUP, RHOA, THBS1

1.0000	0.81	CFL1, EEF1A1, HSPA8, STOM
1.0000	0.81	CCL5, GNAI2, PLEK, YWHAB
1.0000	0.81	CYB5R3, G6PD, PLEK, SOD1
1.0000	0.81	ENO1, GALE, PGK1, PKM
1.0000	0.81	CCL5, FCN1, LTF, PPIA
1.0000	0.81	LRP1, S100A8, S100A9, VIM
1.0000	0.81	FLNA, INPP5D, ITGB3, TFRC
1.0000	0.81	DHX9, FLOT1, HNRNPA2B1, NPM1
1.0000	0.81	DHX9, FLOT1, HNRNPA2B1, NPM1
1.0000	0.81	CD226, HLA-A, INPP5D, PTPRJ
1.0000	0.81	CD226, HLA-A, INPP5D, PTPRJ
1.0000	0.81	DHX9, FLOT1, HNRNPA2B1, NPM1
1.0000	0.81	G6PD, GAPDH, RHOA, SOD1
1.0000	0.81	DDX60L, DHX9, G3BP1, HSP90AA1
1.0000	0.81	CALM3, SRI, UBQLN1, YWHAE
1.0000	0.81	CALM3, CCL5, G6PD, SRI
1.0000	0.81	MSN, RAC2, S100A8, S100A9
1.0000	0.81	HNRNPU, HSPA1B, PATJ, RHOA
1.0000	0.81	ANXA2, ENO1, MYH9, THBS1
1.0000	0.81	CCL5, HLA-A, RAC2, TFRC
1.0000	0.81	DHX9, G3BP1, HNRNPA2B1, XRCC5
1.0000	0.81	F11R, HSPA8, ITGB3, SLC2A1
1.0000	0.81	CCL5, LBP, RAC2, THBS1
1.0000	0.81	FLNA, HNRNPU, HSPA1B, RHOA
1.0000	0.81	CD36, GNAI2, GSTP1, SOD1
1.0000	0.81	DDX60L, DHX9, G3BP1, HSP90AA1
1.0000	0.81	CALM3, SRI, UBQLN1, YWHAE
1.0000	0.81	CALM3, SRI, UBQLN1, YWHAE
1.0000	0.81	FSCN1, G3BP1, MSN, RHOA
1.0000	0.81	CALM3, SRI, UBQLN1, YWHAE
1.0000	0.81	CALM3, GNAI2, RAC2, RAP1B
1.0000	0.81	ANXA2, ENO1, MYH9, THBS1
1.0000	0.81	HSPA1B, PPIA, RPL11, RPS15
1.0000	0.81	CD36, CYBB, RHOA, SOD1
1.0000	0.81	HSPA9, INPP5D, LTF, PRDX2
1.0000	0.81	CCL5, FCN1, LTF, PPIA
1.0000	0.81	CALM3, SRI, UBQLN1, YWHAE
1.0000	0.81	FERMT3, FSCN1, TPM4, VCL
1.0000	0.81	CYB5R3, HSPA9, RAC2, VDAC2
1.0000	0.81	CLTA, HSPD1, LRP1, TFRC
1.0000	0.81	CALM3, CLTA, RAB10, RAB27B
1.0000	0.81	B2M, CD59, HLA-A, HLA-B
1.0000	0.81	B2M, CD59, HLA-A, HLA-B
1.0000	0.81	CALM3, HSP90AA1, HSPD1, VDAC2
1.0000	0.81	B2M, HLA-A, HLA-B, RCC2
1.0000	0.81	CANX, HNRNPU, RAB27B, SOD1
1.0000	0.81	CALM3, HSP90AA1, HSPD1, VDAC2

1.0000	0.81	BCAP31, CANX, SACM1L, TMX2
1.0000	0.81	CALM3, CLTA, RAB10, RAB27B
1.0000	0.81	CALM3, HSP90AA1, HSPD1, VDAC2
1.0000	0.81	CALM3, HSP90AA1, HSPD1, VDAC2
1.0000	0.81	CANX, HNRNPU, RAB27B, SOD1
1.0000	0.81	FCN1, PSMA5, PSMA6, UBQLN1
1.0000	0.81	FCN1, PSMA5, PSMA6, UBQLN1
1.0000	0.81	DHX9, JUP, NPM1, UBE2L3
1.0000	0.81	ADH5, GSTP1, S100A8, S100A9
1.0000	0.81	ANXA2, GSDMA, OSBPL2, PFN1
1.0000	0.81	GSR, PGK1, QSOX1, TMX2
1.0000	0.81	MYL12B, RAB10, RAB27B, RHOA
1.0000	0.81	RAB10, RAB27B, RAP1B, RHOA
1.0000	0.81	FLNA, PFN1, RCC2, SOD1
1.0000	0.81	ACTB, DHX9, HNRNPC, HNRNPU
1.0000	0.81	ADH5, GSTP1, S100A8, S100A9
1.0000	0.81	CD36, LGALS3BP, LRP1, TFRC
1.0000	0.81	ANXA2, ATP2A3, GAPDH, LTF
1.0000	0.81	ANXA2, GSDMA, OSBPL2, PFN1
1.0000	0.81	ANXA2, GSDMA, OSBPL2, PFN1
1.0000	0.84	ACTB, ALDOA, ANXA2, ATIC, ATP5F1B, CCT2, CCT4, CCT6A, CCT7, CCT8, CD36, CSDE1, CYI
1.0000	0.84	ACTB, ADAM10, ADH5, ARL6IP5, ATIC, ATP5F1B, B2M, CAMP, CCL5, CD36, CD47, CDC42,
1.0000	0.79	ANXA2, ARL6IP5, ATP2A3, BCAP31, CALM3, CCL5, CTSD, F11R, GAPDH, GNAI2, GNAQ, HS
1.0000	0.81	ADH5, ALDOA, ANPEP, ANXA2, BCAP31, CANX, CPN1, CSDE1, CTSD, DHX9, EEF1A1, ENO1
1.0000	0.79	ANXA11, CALM3, CCT2, CCT4, CCT6A, CCT7, CCT8, CDC42, CLTA, DHX9, FLOT1, G6PD, GA
1.0000	0.83	ACTB, ANXA2, ARL6IP5, ATP2A3, B2M, BCAP31, CALM3, CAMP, CCL5, CCT2, CCT4, CCT6A
1.0000	0.83	ACTB, ALDOA, ANXA2, ATIC, ATP5F1B, CCT2, CCT4, CCT6A, CCT7, CCT8, CD36, CSDE1, DA
1.0000	0.82	ACTB, ANXA2, ARL6IP5, ATP2A3, B2M, BCAP31, CALM3, CAMP, CCL5, CCT2, CCT4, CCT6A
1.0000	0.82	ACTB, ALDOA, ANXA2, ATIC, ATP5F1B, CCT2, CCT4, CCT6A, CCT7, CCT8, CD36, CSDE1, DA
1.0000	0.79	B2M, CAMP, CCL5, CD226, CD36, CD47, CD59, CDC42, CYBB, DHX9, ENDOD1, FCER1G, FC
1.0000	0.78	ACTB, ANXA2, ARPC5, CCT2, CCT4, CCT6A, CCT7, CCT8, CD47, CDC42, F11R, FLNA, FSCN1
1.0000	0.71	ADAM10, CCL5, CD47, CD9, CD99, GP1BA, ITGB3, LBP, MSN, RAC2, RHOA, SPN, THBS1
1.0000	0.71	B2M, CD226, CD36, CD47, CD59, FCN1, HLA-A, HLA-B, HSPD1, LBP, PTPRJ, RAC2, TFRC
1.0000	0.71	ANXA2, ARL6IP5, ATP2A3, BCAP31, CTSD, ENO1, HSPA1B, HSPD1, MYH9, RHOA, S100A8,
1.0000	0.71	ANXA2, ARL6IP5, CALM3, CD36, CD47, GNAI2, ITGB3, RAB33B, RAP1B, SRI, THBS1, UBQL
1.0000	0.71	ACTB, B2M, CCL5, CD47, HLA-A, HSPD1, INPP5D, PRDX2, RAC2, RHOA, SOD1, SPN, TFRC
1.0000	0.71	ANXA2, ARL6IP5, ATP2A3, BCAP31, CTSD, GAPDH, HSPD1, LRP1, LTF, RHOA, S100A8, S10
1.0000	0.71	ANXA11, ATP2A3, CLIC1, CYBB, EIF5A, ENO1, GAPDH, GNAQ, MATR3, RAC2, RTN4, TMEN
1.0000	0.71	ADAM10, B2M, BCAP31, CD59, CD9, CLTA, HLA-A, HLA-B, HSPA8, HSPD1, M6PR, RAB27B
1.0000	0.74	B2M, CD226, CD36, CD47, CD59, FCER1G, FCN1, HLA-A, HLA-B, HSPD1, INPP5D, KRT1, LB
1.0000	0.74	ANXA2, BCAP31, CSDE1, DHX9, EEF1A1, FLNA, GAPDH, HNRNPC, HNRNPU, HSP90AA1, H
1.0000	0.80	ACTB, ANXA2, B2M, BCAP31, CALM3, CAMP, CCL5, CCT2, CCT4, CCT6A, CCT7, CCT8, CD3
1.0000	0.68	CALM3, GNAQ, GSTP1, LRP1, MYADM, NPM1, PPIA, PTPRJ, YWHAG
1.0000	0.68	ADAM10, CAMP, CCL5, CD226, FCER1G, HSPD1, LBP, RAC2, THBS1
1.0000	0.68	CCL5, CD226, DHX9, FCN1, HSP90AA1, HSPD1, LBP, MATR3, XRCC5
1.0000	0.68	ATP2A3, CD36, LRP1, PTPRJ, RHOA, SLC2A1, SLC2A3, SOD1, TFRC
1.0000	0.68	ANXA2, CD36, ITGB3, LBP, LRP1, OSBPL2, PNPLA8, SLC2A1, THBS1

1.0000	0.68	ADAM10, ATP5F1B, CD9, MYADM, PRDX2, RCC2, RHOA, SPN, THBS1
1.0000	0.68	ALDOA, CCT2, CCT4, CCT7, CCT8, CD9, MYH9, TCP1, VDAC2
1.0000	0.68	ALDOA, CCT2, CCT4, CCT7, CCT8, CD9, MYH9, TCP1, VDAC2
1.0000	0.68	ANXA2, CSDE1, DHX9, FLNA, HNRNPC, HNRNPU, QSOX1, RPL11, SYNCRIP
1.0000	0.68	ANXA2, CD36, ITGB3, LBP, LRP1, OSBPL2, PNPLA8, SLC2A1, THBS1
1.0000	0.68	CCL5, EIF5A, FCER1G, GSTP1, HSPA1B, ILK, PF4V1, PTPRJ, TTLL12
1.0000	0.68	BCAP31, CANX, HSPA1B, PCYOX1, PSMA5, PSMA6, RPL11, UBE2L3, UBQLN1
1.0000	0.68	CSDE1, DHX9, GAPDH, HNRNPU, NCL, RTN3, RTN4, SYNCRIP, TNKS1BP1
1.0000	0.68	ADAM10, CAMP, CCL5, DHX9, EIF5A, GSTP1, HSPA1B, ILK, THBS1
1.0000	0.68	ARL6IP5, ATP2A3, BCAP31, CTSD, HSPD1, RHOA, S100A8, S100A9, THBS1
1.0000	0.68	BCAP31, CANX, HSPA1B, PCYOX1, PSMA5, PSMA6, RPL11, UBE2L3, UBQLN1
1.0000	0.68	ALDOA, ATP5F1B, ENO1, GAPDH, HSPA1B, HSPA8, LDHA, PGK1, PKM
1.0000	0.68	ACTB, B2M, CCL5, CD47, HLA-A, HSPD1, INPP5D, RHOA, TFRC
1.0000	0.68	GNAQ, GSTP1, HNRNPU, NPM1, PPIA, PTPRJ, RPL11, RPS15, YWHAG
1.0000	0.68	BCAP31, CANX, HSPA1B, PCYOX1, PSMA5, PSMA6, RPL11, UBE2L3, UBQLN1
1.0000	0.68	CD36, G6PD, GNAI2, GSTP1, PRDX2, RAC2, RHOA, SOD1, THBS1
1.0000	0.68	CALM3, CCT8, GNAQ, HSP90AA1, HSPD1, PKM, PTGS1, RAB10, VDAC2
1.0000	0.68	ANXA11, ANXA2, CDC42, GNAI2, HNRNPU, RCC2, RHOA, SLC2A1, TTLL12
1.0000	0.68	CALM3, CCL5, FCN1, FLNA, GNAI2, GNAQ, HSPA1B, HSPA8, PF4V1
1.0000	0.82	AACS, ACTB, ADAM10, ADH5, ALDOA, ANK1, ANPEP, ANTXR2, ANXA11, ANXA2, ANXA5, A
1.0000	0.74	CCL5, CD47, ENO1, G3BP1, GNAI2, GSTP1, HSPA1B, INPP5D, ITGA6, ITGB1, LRP1, LTF, MY
1.0000	0.74	CCL5, CD47, ENO1, G3BP1, GNAI2, GSTP1, HSPA1B, INPP5D, ITGA6, ITGB1, LRP1, LTF, MY
1.0000	0.74	ATP2A3, ATP5F1B, B2M, BCAP31, CALM3, CCL5, FLG2, FLNA, GNAI2, GP1BA, GP1BB, GST
1.0000	0.74	ANXA5, CCL5, CFL1, ENO1, FLNA, GNAI2, GSTP1, HSPA1B, HSPA9, HSPD1, ITGA6, ITGB1, I
1.0000	0.81	ACTB, ALDOA, ANK1, ANXA11, ARL6IP5, ARPC5, CALM3, CASP14, CCT2, CCT4, CCT6A, CC
1.0000	0.77	ADAM10, ANXA2, ANXA7, CALM3, CD36, CSDE1, DHX9, ENO1, FLNA, G6PD, GAPDH, GNA
1.0000	0.77	ACTB, ALDOA, ANK1, CALM3, CASP14, CCT2, CCT4, CCT6A, CCT7, CCT8, DSP, ENO1, FLNA
1.0000	0.77	ACTB, ALDOA, ANK1, CALM3, CASP14, CCT2, CCT4, CCT6A, CCT7, CCT8, DSP, ENO1, FLNA
1.0000	0.77	AACS, ACTB, ATP2A3, ATP5F1B, CCT2, CCT4, CCT6A, CCT7, CCT8, CDC42, DDX60L, DHX9,
1.0000	0.78	AACS, ACTB, ATP2A3, ATP5F1B, CCT2, CCT4, CCT6A, CCT7, CCT8, CDC42, CYB5R3, CYBB, D
1.0000	0.79	ACTB, ALDOA, ANXA2, ATIC, ATP5F1B, CCT2, CCT4, CCT6A, CCT7, CCT8, CD36, CSDE1, DA
1.0000	0.79	ACTB, ALDOA, ANXA11, ANXA2, ANXA7, CANX, CCT4, CCT6A, CSDE1, DAZAP1, DCD, DDX6
1.0000	0.74	ANXA2, CCL5, CD47, CD59, CD9, ENO1, G3BP1, GNAI2, GP1BA, GSTP1, HLA-A, HLA-B, HSP
1.0000	0.77	ADAM10, ANXA2, ANXA7, CALM3, CD36, CSDE1, DHX9, ENO1, FLNA, G6PD, GAPDH, GNA
1.0000	0.79	ACTB, ADAM10, ADH5, ALDOA, ANPEP, ANXA2, ARL6IP5, ATIC, ATP2A3, ATP5F1B, B2M, I
1.0000	0.78	ACTB, ADAM10, ANXA2, ARPC5, ATIC, B2M, CALM3, CASP14, CDC42, CSDE1, DAZAP1, DS
1.0000	0.60	ADAM10, CCL5, LBP, RAC2, THBS1
1.0000	0.60	ADAM10, CCL5, LBP, RAC2, THBS1
1.0000	0.60	ADH5, CYB5R3, G6PD, PLEK, SOD1
1.0000	0.60	ACTB, DHX9, NPM1, TNKS1BP1, XRCC5
1.0000	0.60	ACTB, H2BC21, HNRNPC, NASP, NPM1
1.0000	0.60	BCAP31, HSP90AA1, HSPD1, RAC2, UBE2L3
1.0000	0.60	CDC42, HNRNPU, HSPA8, RAB27B, SOD1
1.0000	0.60	ILK, LRP1, SPTBN1, THBS1, VIM
1.0000	0.60	BCAP31, DAZAP1, ITGB1, KRT9, SOD1
1.0000	0.60	DAZAP1, DSG1, ITGB3, SLC2A1, SOD1
1.0000	0.60	CANX, KRT14, KRT16, SOD1, TFRC

1.0000	0.60	ARL6IP5, B2M, ITGB1, SOD1, THBS1
1.0000	0.60	ADH5, ANPEP, PTGS1, RHOA, SOD1
1.0000	0.60	CCL5, GNAI2, ITGB3, PLEK, YWHAB
1.0000	0.60	HSP90AA1, HSPA1B, SOD1, THBS1, YWHAE
1.0000	0.60	ANXA2, GAPDH, LRP1, LTF, THBS1
1.0000	0.60	ATP5F1B, ITGB3, JUP, RHOA, THBS1
1.0000	0.60	ATP5F1B, ITGB3, PFN1, RTN4, THBS1
1.0000	0.60	CDC42, HNRNPU, HSPA8, RAB27B, SOD1
1.0000	0.60	CD47, DHX9, FLOT1, HNRNPA2B1, NPM1
1.0000	0.60	CANX, CCL5, CSDE1, DHX9, PTBP1
1.0000	0.60	ATIC, RHOA, RTN4, SLC2A1, YWHAE
1.0000	0.60	ATIC, RHOA, RTN4, SLC2A1, YWHAE
1.0000	0.60	G6PD, LTF, PFN1, RHOA, YWHAZ
1.0000	0.60	ATIC, RHOA, RTN4, SLC2A1, YWHAE
1.0000	0.60	ENO1, G6PD, HSPA1B, PTPRJ, RTN4
1.0000	0.60	CDC42, MYADM, PFN1, SPTBN1, VDAC2
1.0000	0.60	B2M, CD226, CD59, HLA-A, HLA-B
1.0000	0.60	CD47, CDC42, PFN1, PLEK, RHOA
1.0000	0.60	CD36, CD47, HLA-B, HSPD1, THBS1
1.0000	0.60	CD36, CD47, HLA-B, HSPD1, THBS1
1.0000	0.60	HNRNPU, HSPA1B, NPM1, PATJ, RHOA
1.0000	0.60	HNRNPA2B1, NCL, RPL11, RPS15, WBP11
1.0000	0.60	ARL6IP5, CALM3, SRI, UBQLN1, YWHAE
1.0000	0.60	HSPD1, INPP5D, ITGB1, PTPRJ, TFRC
1.0000	0.60	CCL5, HLA-A, RAC2, SPN, TFRC
1.0000	0.60	ARL6IP5, CALM3, SRI, UBQLN1, YWHAE
1.0000	0.60	ADH5, ENO1, GALE, PGK1, PKM
1.0000	0.60	DAZAP1, DSG1, ITGB3, SLC2A1, SOD1
1.0000	0.60	DAZAP1, DSG1, ITGB3, SLC2A1, SOD1
1.0000	0.60	ENO1, G6PD, HSPA1B, PTPRJ, RTN4
1.0000	0.60	DAZAP1, DHX9, GSTP1, ITGB3, PRDX1
1.0000	0.60	ITGB1, ITGB3, MYADM, PFN1, SOD1
1.0000	0.60	CD59, HLA-A, HLA-B, INPP5D, TTLL12
1.0000	0.60	CFL1, HSPA8, RHOA, TUBB, YWHAZ
1.0000	0.60	CFL1, HSPA8, RHOA, TUBB, YWHAZ
1.0000	0.60	ADAM10, CCL5, LBP, RAC2, THBS1
1.0000	0.60	ADAM10, CCL5, LBP, RAC2, THBS1
1.0000	0.60	CALM3, CPN1, HNRNPU, RHOA, UBE2L3
1.0000	0.60	F11R, MYADM, NPM1, PFN1, SPTBN1
1.0000	0.60	CCT2, CCT4, HSP90AA1, TCP1, XRCC5
1.0000	0.60	B2M, LTF, SOD1, SRI, TFRC
1.0000	0.60	CDC42, HNRNPU, NPM1, RCC2, RHOA
1.0000	0.60	ARL6IP5, CLIC1, ITGB1, PCYOX1, VDAC2
1.0000	0.60	CDC42, HNRNPU, HSPA8, RAB27B, SOD1
1.0000	0.60	CYB5R3, G6PD, OSBPL2, PLEK, SOD1
1.0000	0.60	F11R, HSPA8, MYADM, PFN1, SPTBN1
1.0000	0.60	ANXA2, CD36, ITGB3, LRP1, THBS1

1.0000	0.60	CD36, GNAI2, GSTP1, SOD1, THBS1
1.0000	0.60	CSDE1, DHX9, HNRNPU, PKM, SYNCRIP
1.0000	0.60	CSDE1, DHX9, HNRNPU, PKM, SYNCRIP
1.0000	0.60	FSCN1, ICAM2, ITGB3, MSN, SPN
1.0000	0.60	ANK1, ATP2A3, SRI, THBS1, TMEM109
1.0000	0.60	ANK1, ATP2A3, SRI, THBS1, TMEM109
1.0000	0.60	B2M, HLA-A, HLA-B, RAB10, TFRC
1.0000	0.60	CYB5R3, CYBB, LDHA, LDHB, RAC2
1.0000	0.60	ACTB, DHX9, HNRNPC, HNRNPU, NPM1
1.0000	0.60	DAZAP1, HNRNPA2B1, HNRNPA3, HNRNPC, HNRNPU
1.0000	0.60	GSR, PCYOX1, PGK1, QSOX1, TMX2
1.0000	0.60	ADH5, FCN1, GSTP1, S100A8, S100A9
1.0000	0.60	ANXA2, CD47, DSP, JUP, RAB10
1.0000	0.67	ANXA2, ARL6IP5, ATP2A3, BCAP31, CTSD, ENO1, GAPDH, HSPA1B, HSPD1, LRP1, LTF, MY
1.0000	0.67	BCAP31, CALM3, CCT2, CCT4, CCT6A, CCT7, CCT8, CD36, FLNA, HSP90AA1, HSPA8, HSPD
1.0000	0.67	CSDE1, FLNA, FSCN1, G3BP1, HNRNPU, HSPA1B, ITGB1, MSN, NPM1, RHOA, RPL11, RPLP
1.0000	0.70	ANXA2, BCAP31, CANX, CPN1, CSDE1, CTSD, DHX9, EEF1A1, FLNA, HNRNPC, HNRNPU, HS
1.0000	0.70	ACTB, CALM3, CCL5, CCT2, CCT4, CDC42, EEF1A1, GNAQ, GSTP1, HNRNPA2B1, HNRNPU,
1.0000	0.65	AACS, ADH5, ALDOA, ATP5F1B, CYBB, ENO1, G6PD, GAPDH, GSR, LDHA, PGK1, PKM, RHC
1.0000	0.65	CALM3, GNAI2, GNAQ, GSTP1, HNRNPU, LRP1, MYADM, NPM1, PLEK, PPIA, PTPRJ, YWH
1.0000	0.65	ALDOA, BCAP31, CCT2, CCT4, CCT7, CCT8, CD9, DAZAP1, ITGB1, KRT9, MYH9, SOD1, TCP
1.0000	0.65	ADAM10, CDC42, ENO1, G6PD, HSPA1B, KRT17, NPM1, PTPRJ, RHOA, RTN4, S100A8, S10
1.0000	0.65	CALM3, GNAI2, GNAQ, GSTP1, HNRNPU, LRP1, MYADM, NPM1, PLEK, PPIA, PTPRJ, YWH
1.0000	0.65	ACTB, CALM3, CDC42, CLIC1, HNRNPU, HSPA1B, HSPA8, ITGB1, NPM1, RCC2, RHOA, RPS
1.0000	0.65	ANXA2, CASP14, DSC1, DSG1, DSP, FLG2, HRNR, JUP, KRT1, KRT10, KRT16, KRT17, KRT2,
1.0000	0.65	ACTB, ARPC5, DHX9, H2AC20, H2BC21, HNRNPA2B1, HNRNPC, HNRNPU, NASP, NCL, RCC
1.0000	0.65	DAZAP1, DHX9, G3BP1, HNRNPA2B1, HNRNPA3, HNRNPC, HNRNPU, HSP90AA1, NCL, PC
1.0000	0.65	CANX, CCT2, CCT4, CCT6A, CCT7, CCT8, HSP90AA1, HSPA1B, HSPA8, HSPA9, HSPD1, NPM
1.0000	0.76	ACTB, ADAM10, ALDOA, ATIC, ATP5F1B, CALM3, CAMP, CCL5, CD36, CDC42, EEF1A1, EN
1.0000	0.77	AACS, ACTB, ADAM10, ADH5, ALDOA, ANPEP, ANXA2, ANXA7, ARL6IP5, ATIC, ATP2A3, A
1.0000	0.75	ACTB, ANXA11, ANXA2, CASP14, CAVIN2, CCT4, CCT8, CFL1, DAZAP1, DHX9, EEF1A1, FLN
1.0000	0.75	AACS, ACTB, ALDOA, ATP2A3, ATP5F1B, CCT2, CCT4, CCT6A, CCT7, CCT8, CDC42, CYB5R3
1.0000	0.75	AACS, ACTB, ADH5, ANXA2, ATP2A3, ATP5F1B, CCT2, CCT4, CCT6A, CCT7, CCT8, CDC42, C
1.0000	0.59	B2M, CD226, FCER1G, HLA-A, HLA-B, HSPD1, INPP5D, PRDX1, TFRC, TUBB
1.0000	0.59	GNAI2, ILK, ITGB3, LRP1, NCL, PTPRJ, RTN4, SPTBN1, THBS1, VIM
1.0000	0.59	AACS, CYB5R3, G6PD, GSTP1, INPP5D, OSBPL2, PNPLA8, PTGS1, SACM1L, SOD1
1.0000	0.59	ALDOA, ATP5F1B, ENO1, GAPDH, HSPA1B, HSPA8, LDHA, PGK1, PKM, RHOA
1.0000	0.59	ALDOA, ATP5F1B, ENO1, GAPDH, HSPA1B, HSPA8, LDHA, PGK1, PKM, RHOA
1.0000	0.59	ALDOA, ATP5F1B, ENO1, GAPDH, HSPA1B, HSPA8, LDHA, PGK1, PKM, RHOA
1.0000	0.59	ALDOA, ATP5F1B, ENO1, GAPDH, HSPA1B, HSPA8, LDHA, PGK1, PKM, RHOA
1.0000	0.59	CALM3, GNAI2, INPP5D, PLEK, PPIA, PTBP1, PTPRJ, SACM1L, YWHAB, YWHAE
1.0000	0.59	ARPC5, HSP90AA1, MSN, MYADM, NPM1, PFN1, PLEK, RHOA, RTN4, SPTBN1
1.0000	0.59	CD36, CD47, CYBB, DHX9, GSTP1, LBP, LTF, PTPRJ, SPN, THBS1
1.0000	0.59	CD36, CD47, CYBB, DHX9, GSTP1, LBP, LTF, PTPRJ, SPN, THBS1
1.0000	0.59	ANXA2, BCAP31, EEF1A1, FLNA, HSP90AA1, HSPA1B, LRP1, MSN, RPL11, UBQLN1
1.0000	0.59	CALM3, GNAQ, GSTP1, HNRNPU, LRP1, MYADM, NPM1, PPIA, PTPRJ, YWHAG
1.0000	0.59	AACS, CYB5R3, G6PD, GSTP1, OSBPL2, PGK1, PLEK, PNPLA8, PTGS1, SOD1

1.0000	0.59	CALM3, CCL5, HSP90AA1, ITGB3, LTF, NPM1, PPIA, RHOA, THBS1, XRCC5
1.0000	0.59	CD36, CD47, CYBB, DHX9, GSTP1, LBP, LTF, PTPRJ, SPN, THBS1
1.0000	0.59	ENO1, GSTP1, ITGB1, MYADM, PLEK, PPIA, PTPRJ, RHOA, THBS1, VDAC2
1.0000	0.59	CD36, CD47, CYBB, DHX9, GSTP1, LBP, LTF, PTPRJ, SPN, THBS1
1.0000	0.59	ACTB, B2M, HLA-B, HSPA1B, HSPA9, INPP5D, LTF, PRDX2, RHOA, SOD1
1.0000	0.59	ARL6IP5, ATP2A3, BCAP31, CTSD, HSPD1, LTF, RHOA, S100A8, S100A9, THBS1
1.0000	0.59	BCAP31, EIF5A, ENO1, PPIA, RPL11, S100A8, S100A9, SOD1, UBQLN1, VDAC2
1.0000	0.59	ANXA2, ATP2A3, ATP5F1B, CD36, CLIC1, CYBB, SLC2A1, SLC2A3, TMEM109, VDAC2
1.0000	0.59	ANXA2, ATP2A3, ATP5F1B, CD36, CLIC1, CYBB, SLC2A1, SLC2A3, TMEM109, VDAC2
1.0000	0.71	ANXA2, CALM3, CD36, CSDE1, DHX9, ENO1, FLNA, G6PD, GAPDH, GNAI2, GNAQ, GSTP1,
1.0000	0.70	AACS, ACTB, ADAM10, ADH5, ALDOA, ANK1, ANPEP, ANTXR2, ANXA11, ANXA2, ANXA5, A
1.0000	0.76	ACTB, ADAM10, ALDOA, ANXA11, ANXA2, ARPC5, ATP5F1B, B2M, CAMP, CANX, CASP14,
1.0000	0.76	ACTB, ADAM10, ALDOA, ANXA11, ANXA2, ARPC5, ATP5F1B, B2M, CAMP, CANX, CASP14,
1.0000	0.76	ACTB, ADAM10, ALDOA, ANXA11, ANXA2, ARPC5, ATP5F1B, B2M, CAMP, CANX, CASP14,
1.0000	0.73	ACTB, ADAM10, ALDOA, ATIC, ATP5F1B, CALM3, CAMP, CCL5, CD36, CDC42, EEF1A1, EN
1.0000	0.74	ALDOA, ANXA11, ANXA2, ANXA7, CANX, CCT4, CCT6A, CSDE1, DAZAP1, DCD, DDX60L, DH
1.0000	0.65	ANXA2, B2M, CDC42, F11R, FLNA, HNRNPC, HNRNPU, HSPA1B, HSPA8, ITGB3, MYADM, I
1.0000	0.65	ADH5, ATP5F1B, BCAP31, CALM3, CASP14, CLIC1, CYB5R3, GSR, GSTP1, HSP90AA1, HSPA
1.0000	0.66	B2M, CD226, CD36, CD47, CYBB, DHX9, F11R, FCER1G, FCN1, FLOT1, G3BP1, GAPDH, GS
1.0000	0.66	B2M, CD226, CD36, CD47, CYBB, DHX9, F11R, FCER1G, FCN1, FLOT1, G3BP1, GAPDH, GS
1.0000	0.66	AACS, ACTB, ATP2A3, ATP5F1B, CCT2, CCT4, CCT6A, CCT7, CCT8, DDX60L, DHX9, G3BP1,
1.0000	0.66	AACS, ACTB, ATP2A3, ATP5F1B, CCT2, CCT4, CCT6A, CCT7, CCT8, DDX60L, DHX9, G3BP1,
1.0000	0.76	ACTB, ADAM10, ADH5, ALDOA, ANPEP, ANXA2, ARL6IP5, ATIC, ATP2A3, ATP5F1B, B2M, I
1.0000	0.62	ALDOA, BCAP31, CCT2, CCT4, CCT7, CCT8, CD9, CSDE1, DAZAP1, DSG1, ITGB1, ITGB3, KR
1.0000	0.62	ALDOA, BCAP31, CCT2, CCT4, CCT7, CCT8, CD9, CSDE1, DAZAP1, DSG1, ITGB1, ITGB3, KR
1.0000	0.62	ANXA2, ANXA5, ATP2A3, CALM3, CCL5, GAPDH, GNAQ, GSTP1, HSP90AA1, LTF, NPM1, R
1.0000	0.74	AACS, ACTB, ALDOA, ANXA2, ATIC, ATP5F1B, CCL5, CCT2, CCT4, CCT6A, CCT7, CCT8, CD3
1.0000	0.74	AACS, ACTB, ALDOA, ANXA2, ATIC, ATP5F1B, CCL5, CCT2, CCT4, CCT6A, CCT7, CCT8, CD3
1.0000	0.74	AACS, ADAM10, ADH5, ALDOA, ANPEP, ATIC, ATP2A3, ATP5F1B, CASP14, CCL5, CCT2, CC
1.0000	0.76	ACTB, ADAM10, ANXA2, ARPC5, ATP5F1B, B2M, CALM3, CANX, CCT2, CCT4, CCT6A, CCT
1.0000	0.68	ACTB, ARL6IP5, ATP2A3, B2M, BCAP31, CANX, CD36, CYBB, DHX9, ENO1, FLNA, FLOT1, G
1.0000	0.75	AACS, ACTB, ADAM10, ADH5, ALDOA, ANPEP, ANXA2, ARL6IP5, ATIC, ATP2A3, ATP5F1B,
1.0000	0.59	ACTB, CCT2, CCT4, CCT6A, CCT7, CCT8, DHX9, HNRNPA2B1, HNRNPC, HNRNPU, HSP90AA
1.0000	0.71	AACS, ACTB, ATP2A3, ATP5F1B, CCT2, CCT4, CCT6A, CCT7, CCT8, CDC42, CYB5R3, CYBB, I
1.0000	0.69	AACS, ACTB, ATP2A3, ATP5F1B, CCT2, CCT4, CCT6A, CCT7, CCT8, CDC42, DDX60L, DHX9,
1.0000	0.69	AACS, ACTB, ATP2A3, ATP5F1B, CCT2, CCT4, CCT6A, CCT7, CCT8, CDC42, DDX60L, DHX9,
1.0000	0.48	GSTP1, INPP5D, LBP, LTF, SRGN, THBS1
1.0000	0.48	FCER1G, HSPD1, LBP, RAC2, SPN, TFRC
1.0000	0.48	B2M, CD226, HLA-A, HLA-B, HSPD1, TFRC
1.0000	0.48	B2M, CD226, HLA-A, HLA-B, HSPD1, TFRC
1.0000	0.48	B2M, CD226, HLA-A, HLA-B, HSPD1, TFRC
1.0000	0.48	B2M, CD226, HLA-A, HLA-B, HSPD1, TFRC
1.0000	0.48	B2M, CD226, HLA-A, HLA-B, HSPD1, TFRC
1.0000	0.48	B2M, CD226, HLA-A, HLA-B, HSPD1, TFRC
1.0000	0.48	B2M, CD226, HLA-A, HLA-B, HSPD1, TFRC
1.0000	0.48	BCAP31, HSP90AA1, HSPD1, RAC2, UBE2L3, VDAC2
1.0000	0.48	ACTB, DHX9, NPM1, TMEM109, TNKS1BP1, XRCC5

1.0000	0.48	HSP90AA1, HSPA1B, HSPA8, HSPA9, HSPD1, THBS1
1.0000	0.48	HSP90AA1, HSPA1B, HSPD1, SOD1, THBS1, YWHAE
1.0000	0.48	GSTP1, HLA-A, HLA-B, KRT1, SOD1, TTLL12
1.0000	0.48	CALM3, CPN1, CYBB, HNRNPU, RHOA, UBE2L3
1.0000	0.48	CCL5, HLA-A, INPP5D, RAC2, SPN, TFRC
1.0000	0.48	B2M, G6PD, HSPA1B, HSPA9, INPP5D, PRDX1
1.0000	0.48	ARL6IP5, CALM3, SRI, THBS1, UBQLN1, YWHAE
1.0000	0.48	ATP2A3, BCAP31, CANX, FLOT1, THBS1, UBQLN1
1.0000	0.48	HSP90AA1, HSPA1B, HSPA8, HSPA9, HSPD1, THBS1
1.0000	0.48	B2M, HSP90AA1, HSPA1B, HSPA8, HSPA9, HSPD1
1.0000	0.48	CD36, FCN1, ITGB3, LRP1, RAC2, THBS1
1.0000	0.48	BCAP31, HSP90AA1, HSPA1B, LRP1, MSN, UBQLN1
1.0000	0.48	CDC42, HNRNPU, NPM1, RCC2, RHOA, RPS15A
1.0000	0.48	ANXA2, GAPDH, LRP1, LTF, RPL11, THBS1
1.0000	0.48	PTPRJ, RCC2, RHOA, THBS1, TLN1, VCL
1.0000	0.48	ANK1, BCAP31, RAB10, RAB1B, RAB33B, SPTBN1
1.0000	0.48	CCL5, HLA-A, INPP5D, RAC2, SPN, TFRC
1.0000	0.48	CD36, CD47, FCER1G, FCN1, PTPRJ, SOD1
1.0000	0.48	CD36, CD47, FCER1G, FCN1, PTPRJ, SOD1
1.0000	0.48	DHX9, HNRNPA2B1, HSPA9, NPM1, RPS15, YWHAE
1.0000	0.48	CD47, CDC42, HSPA1B, PFN1, PLEK, RHOA
1.0000	0.48	PTPRJ, RCC2, RHOA, THBS1, TLN1, VCL
1.0000	0.48	BCAP31, CALM3, HSP90AA1, HSPD1, RAC2, UBE2L3
1.0000	0.48	CCL5, EEF1A1, ILK, ITGB3, NCL, THBS1
1.0000	0.48	ARL6IP5, CCL5, G6PD, GAPDH, RHOA, SOD1
1.0000	0.48	CCL5, EEF1A1, ILK, ITGB3, NCL, THBS1
1.0000	0.48	HNRNPU, HSPA1B, HSPA8, RHOA, UBE2L3, YWHAH
1.0000	0.48	BCAP31, CALM3, HSP90AA1, HSPD1, RAC2, UBE2L3
1.0000	0.48	PTPRJ, RCC2, RHOA, THBS1, TLN1, VCL
1.0000	0.48	PTPRJ, RCC2, RHOA, THBS1, TLN1, VCL
1.0000	0.48	CD47, CDC42, HSPA1B, PFN1, PLEK, RHOA
1.0000	0.48	CALM3, G6PD, ITGB3, SRI, UBQLN1, YWHAE
1.0000	0.48	BCAP31, EIF5A, RPL11, S100A8, S100A9, SOD1
1.0000	0.48	FLOT1, ICAM2, MSN, MYH9, SELPLG, SPN
1.0000	0.48	ADAM10, RPL8, RPLP0, RTN3, RTN4, SPTBN1
1.0000	0.48	CYB5R3, ENO1, HSPA9, RAC2, TMEM109, VDAC2
1.0000	0.48	B2M, CYBB, HLA-A, HLA-B, RAB10, RAC2
1.0000	0.48	EEF1A1, FLOT1, MYADM, MYH9, SLC2A1, SPTBN1
1.0000	0.48	ALDOA, FERMT3, MMRN1, QSOX1, SRGN, THBS1
1.0000	0.48	FLOT1, ICAM2, MSN, MYH9, SELPLG, SPN
1.0000	0.48	CYB5R3, ENO1, HSPA9, RAC2, TMEM109, VDAC2
1.0000	0.48	ADAM10, RPL8, RPLP0, RTN3, RTN4, SPTBN1
1.0000	0.48	DHX9, HNRNPA2B1, HNRNPU, HSPD1, PCBP1, WBP11
1.0000	0.48	DHX9, ENO1, HNRNPU, JUP, NPM1, UBE2L3
1.0000	0.74	AACS, ACTB, ADAM10, ADH5, ALDOA, ANPEP, ANXA2, ANXA7, ARL6IP5, ATIC, ATP5F1B, E
1.0000	0.52	B2M, CD226, FCER1G, HLA-A, HLA-B, HSPD1, INPP5D, PRDX1, RAC2, TFRC, TUBB
1.0000	0.52	B2M, CD9, GSTP1, HSPA1B, ILK, INPP5D, NPM1, PTPRJ, RHOA, SPN, THBS1

1.0000	0.52	ACTB, CCT2, CCT4, CCT6A, CCT7, CCT8, HNRNPA2B1, HNRNPC, HNRNPU, TCP1, XRCC5
1.0000	0.52	DAZAP1, HNRNPA2B1, HNRNPF, HNRNPH1, HNRNPH3, HNRNPU, HSPA8, NCL, NPM1, PR
1.0000	0.52	AACS, ADH5, CD36, GSTP1, INPP5D, LRP1, PLEK, PNPLA8, PRDX6, PTGS1, SACM1L
1.0000	0.52	ARPC5, HSP90AA1, MSN, MYADM, NPM1, PFN1, PLEK, RHOA, RTN4, SOD1, SPTBN1
1.0000	0.52	CCT2, CCT4, CCT6A, CCT7, CCT8, HNRNPA2B1, HNRNPC, HNRNPU, HSP90AA1, TCP1, XRC
1.0000	0.52	DHX9, HNRNPA2B1, HNRNPU, HSPA1B, MYH9, NPM1, PCBP1, SF3A3, SH3BGRL3, SPN, TK
1.0000	0.52	ANXA2, ATP2A3, ATP5F1B, CD36, CLIC1, CYBB, OSBPL2, SLC2A1, SLC2A3, TMEM109, VDA
1.0000	0.69	ACTB, ALDOA, ANK1, CALM3, CASP14, CCT2, CCT4, CCT6A, CCT7, CCT8, CDC42, CSDE1, D
1.0000	0.63	ACTB, CALM3, CYB5R3, CYBB, DHX9, ENO1, FCN1, GNAI2, GNAQ, GNG5, HINT1, HNRNPA
1.0000	0.63	AACS, ACTB, ATP2A3, ATP5F1B, CCT2, CCT4, CCT6A, CCT7, CCT8, DDX60L, DHX9, G3BP1,
1.0000	0.62	ACTB, ADAM10, ADH5, ALDOA, ANK1, ANPEP, ANTXR2, ANXA11, ANXA2, ANXA5, ANXA7
1.0000	0.61	CCL5, CD47, ENO1, G3BP1, GNAI2, GSTP1, HSPA1B, INPP5D, ITGA6, ITGB1, LRP1, LTF, MY
1.0000	0.61	ADAM10, ANXA7, CD36, CSDE1, DHX9, GAPDH, GSTP1, HNRNPA2B1, HNRNPC, HNRNPU,
1.0000	0.58	ARL6IP5, ATP2A3, BCAP31, EIF5A, ENO1, GNAI2, GSTP1, HINT1, HSPA1B, ITGA6, PPIA, PR
1.0000	0.58	ACTB, ANK1, ARPC5, DSP, KRT14, KRT15, KRT16, KRT2, KRT5, KRT6A, KRT6B, KRT9, MSN,
1.0000	0.66	ACTB, ADAM10, B2M, CCL5, CD226, CD36, CD47, CD59, CD9, CD99, DHX9, FCER1G, FCN1
1.0000	0.66	AACS, ACTB, ATP2A3, ATP5F1B, CCT2, CCT4, CCT6A, CCT7, CCT8, CDC42, DDX60L, DHX9,
1.0000	0.54	CALM3, G6PD, GNAI2, GNAQ, GSTP1, HSPA1B, LRP1, MYADM, NPM1, PPIA, PTPRJ, RPL11
1.0000	0.54	CASP14, DSP, FLG2, HRNR, ITGA6, JUP, KRT1, KRT10, KRT16, KRT17, KRT2, KRT5, KRT6A,
1.0000	0.54	ACTB, CCT2, CCT4, CCT6A, CCT7, CCT8, DHX9, G3BP1, HNRNPA2B1, HNRNPC, HNRNPU, H
1.0000	0.54	ALDOA, ATIC, ATP5F1B, ENO1, G6PD, GAPDH, HINT1, HSPA1B, HSPA8, LDHA, NANS, PGK
1.0000	0.54	CASP14, DSP, JUP, KRT1, KRT10, KRT14, KRT15, KRT16, KRT17, KRT2, KRT5, KRT6A, KRT6I
1.0000	0.54	CCT2, HSP90AA1, HSPA1B, HSPA8, HSPA9, HSPD1, PRDX6, RPL11, RTN4, TCP1, TUBB, UB
1.0000	0.54	CCT2, HSP90AA1, HSPA1B, HSPA8, HSPA9, HSPD1, PRDX6, RPL11, RTN4, TCP1, TUBB, UB
1.0000	0.72	AACS, ACTB, ALDOA, ANXA11, ANXA2, ANXA7, ATP2A3, ATP5F1B, CANX, CCT2, CCT4, CCT
1.0000	0.71	AACS, ACTB, ALDOA, ANXA2, ATIC, ATP5F1B, CCL5, CCT2, CCT4, CCT6A, CCT7, CCT8, CD3(
1.0000	0.60	AACS, ADH5, ALDOA, ATIC, ATP5F1B, CD36, CYB5R3, ENO1, G6PD, GALE, GAPDH, GSTP1,
1.0000	0.47	CCT2, CCT4, CCT6A, CCT7, CCT8, HNRNPA2B1, HNRNPC, HNRNPU, HSP90AA1, TCP1, TNK
1.0000	0.47	CD226, CD47, CD59, FCER1G, FCN1, HLA-A, HSP90AA1, INPP5D, KRT1, MATR3, PTPRJ, XR
1.0000	0.47	CD47, CD59, HLA-A, HLA-B, HSPA9, INPP5D, LTF, PRDX2, PTPRJ, SPN, THBS1, TTLL12
1.0000	0.47	ALDOA, ATIC, ATP5F1B, ENO1, GAPDH, HINT1, HSPA1B, HSPA8, LDHA, PGK1, PKM, RHOA
1.0000	0.47	ANK1, ANXA2, CALM3, CCL5, GNAI2, MYH9, PLEK, RAB10, RAB27B, RAB33B, RAC2, RAP1I
1.0000	0.47	ALDOA, ATIC, ATP5F1B, ENO1, GAPDH, HINT1, HSPA1B, HSPA8, LDHA, PGK1, PKM, RHOA
1.0000	0.47	ALDOA, ATIC, ATP5F1B, ENO1, GAPDH, HINT1, HSPA1B, HSPA8, LDHA, PGK1, PKM, RHOA
1.0000	0.47	CCT2, CCT4, CCT6A, CCT7, CCT8, HNRNPA2B1, HNRNPC, HNRNPU, HSP90AA1, TCP1, TNK
1.0000	0.47	CCT2, CCT4, CCT6A, CCT7, CCT8, HNRNPA2B1, HNRNPC, HNRNPU, HSP90AA1, TCP1, TNK
1.0000	0.47	CCT2, CCT4, CCT6A, CCT7, CCT8, HNRNPA2B1, HNRNPC, HNRNPU, HSP90AA1, TCP1, TNK
1.0000	0.47	ALDOA, ATIC, ATP5F1B, ENO1, GAPDH, HINT1, HSPA1B, HSPA8, LDHA, PGK1, PKM, RHOA
1.0000	0.47	HNRNPA2B1, HNRNPA3, HNRNPC, HNRNPF, HNRNPH1, HNRNPH3, HNRNPU, HSPA8, NCL
1.0000	0.70	AACS, ACTB, ALDOA, ANXA11, ANXA2, ANXA7, ATP2A3, ATP5F1B, CANX, CCT2, CCT4, CCT
1.0000	0.54	ANXA2, B2M, CALM3, CD151, CD36, CD47, CDC42, FCER1G, FCN1, FLOT1, GNAI2, ITGB3,
1.0000	0.54	ANTXR2, ANXA2, ARPC5, B2M, CLTA, CTSD, FLOT1, HLA-A, HLA-B, HSPA8, HSPD1, ITGB1,
1.0000	0.64	ADAM10, ANPEP, ATIC, ATP2A3, CASP14, CCL5, CCT2, CCT4, CCT6A, CCT7, CCT8, CDC42,
1.0000	0.39	B2M, G6PD, HSPA1B, HSPA9, INPP5D, PRDX1, SOD1
1.0000	0.39	FCER1G, HSPD1, LBP, LRP1, RAC2, SPN, TFRC
1.0000	0.39	CD226, CD47, FCER1G, FCN1, HLA-A, INPP5D, PTPRJ
1.0000	0.39	B2M, CD226, HLA-A, HLA-B, HSPD1, RAC2, TFRC

1.0000	0.39	CD226, CD47, FCER1G, FCN1, HLA-A, INPP5D, PTPRJ
1.0000	0.39	CD226, CD47, FCER1G, FCN1, HLA-A, INPP5D, PTPRJ
1.0000	0.39	GNAI2, ITGB3, LRP1, NCL, PTPRJ, RTN4, THBS1
1.0000	0.39	GNAQ, ITGB1, ITGB3, NPM1, TMEM109, TNKS1BP1, XRCC5
1.0000	0.39	CSDE1, DHX9, GAPDH, HNRNPU, NCL, SYNCRIP, TNKS1BP1
1.0000	0.39	JUP, KRT16, LRP1, PTPRJ, RHOA, THBS1, VCL
1.0000	0.39	CDC42, HNRNPU, HSPA8, RAB27B, SOD1, TUBA1C, TUBB
1.0000	0.39	ARPC5, ATIC, B2M, RHOA, RTN4, SLC2A1, YWHAE
1.0000	0.39	JUP, KRT16, LRP1, PTPRJ, RHOA, THBS1, VCL
1.0000	0.39	CCL5, GSTP1, HLA-A, INPP5D, RAC2, SPN, TFRC
1.0000	0.39	F11R, ITGB3, NPM1, SLC2A1, TMEM109, TNKS1BP1, XRCC5
1.0000	0.39	ACTB, CALM3, GSTP1, LTF, PTPRJ, RHOA, THBS1
1.0000	0.39	F11R, ITGB3, NPM1, SLC2A1, TMEM109, TNKS1BP1, XRCC5
1.0000	0.39	CALM3, CYBB, DSG1, MSN, NASP, RPLP0, THBS1
1.0000	0.39	CSDE1, DHX9, GAPDH, HNRNPU, NCL, SYNCRIP, TNKS1BP1
1.0000	0.39	JUP, KRT16, LRP1, PTPRJ, RHOA, THBS1, VCL
1.0000	0.39	BCAP31, EIF5A, RPL11, S100A8, S100A9, SOD1, THBS1
1.0000	0.39	DHX9, EEF2, PSMA6, RPL11, RPL8, RPS23, VIM
1.0000	0.39	ATP2A3, CLIC1, ENO1, GAPDH, GNAQ, MATR3, TMEM109
1.0000	0.39	ADAM10, HSPA8, RPL8, RPLP0, RTN3, RTN4, SPTBN1
1.0000	0.39	DHX9, ENO1, HNRNPU, JUP, NPM1, PCBP1, UBE2L3
1.0000	0.69	ACTB, ALDOA, ANK1, ANXA11, ANXA2, ARL6IP5, ARPC5, ATP5F1B, BCAP31, CALM3, CAN
1.0000	0.69	ACTB, ALDOA, ANK1, ANXA11, ANXA2, ARL6IP5, ARPC5, ATP5F1B, BCAP31, CALM3, CAN
1.0000	0.58	ALDOA, ANXA2, ARPC5, B2M, CD36, CD47, CDC42, CFL1, DSP, F11R, FLNA, FSCN1, HSPA1
1.0000	0.57	ANXA2, CALM3, CSDE1, DHX9, FLNA, G6PD, GAPDH, GNAI2, GNAQ, GSTP1, HNRNPU, HSP
1.0000	0.54	ATIC, ATP5F1B, CAMP, CCL5, CD47, CDC42, DHX9, EIF5A, FCER1G, GAPDH, GSTP1, HNRN
1.0000	0.46	ACTB, CCT2, CCT4, CCT6A, CCT7, CCT8, DHX9, HNRNPA2B1, HNRNPC, HNRNPU, HSP90AA
1.0000	0.46	ANXA2, CCT2, CCT4, CCT6A, CCT7, CCT8, CD47, CDC42, FSCN1, G3BP1, HNRNPA2B1, HSP
1.0000	0.46	CCL5, CSDE1, DHX9, EEF2, EIF5A, GAPDH, HNRNPU, KRT17, NCL, NPM1, PKM, RHOA, RTN
1.0000	0.46	CASP14, CCT8, DSP, JUP, KRT1, KRT10, KRT14, KRT15, KRT16, KRT17, KRT2, KRT5, KRT6A,
1.0000	0.46	ACTB, DHX9, ENO1, G3BP1, H2AC20, H2BC21, HNRNPA2B1, HNRNPC, HNRNPU, HSPD1, L
1.0000	0.34	F11R, HNRNPC, HNRNPU, MYADM, NPM1, PFN1, SPTBN1, TFRC
1.0000	0.34	ANXA2, CALM3, GNAI2, RAB10, RAB27B, RAB33B, RAC2, RAP1B
1.0000	0.34	BCAP31, CALM3, CD36, HINT1, PLEK, PTBP1, PTPRJ, THBS1
1.0000	0.34	DHX9, HSP90AA1, RPL11, RPLP0, RPS15, RPSA, SF3A3, XRCC5
1.0000	0.34	ANXA2, CSDE1, DHX9, HNRNPC, HNRNPU, QSOX1, RPL11, SYNCRIP
1.0000	0.34	CCT2, CCT4, CCT6A, CCT7, CCT8, HNRNPA2B1, TCP1, XRCC5
1.0000	0.34	ARPC5, HSP90AA1, HSPA1B, MYADM, PFN1, RHOA, SPTBN1, VDAC2
1.0000	0.34	ALDOA, CCT2, CCT4, CCT7, CCT8, CD9, TCP1, VDAC2
1.0000	0.34	DAZAP1, HNRNPA2B1, HNRNPU, HSPA8, NCL, NPM1, PRDX6, PTBP1
1.0000	0.34	DHX9, HSP90AA1, RPL11, RPLP0, RPS15, RPSA, SF3A3, XRCC5
1.0000	0.34	ADAM10, CCL5, CD99, F11R, ITGB3, MSN, RHOA, SPN
1.0000	0.34	ADAM10, CCL5, CD99, F11R, ITGB3, MSN, RHOA, SPN
1.0000	0.34	CALM3, CYBB, G6PD, JUP, RHOA, RPLP0, S100A8, SOD1
1.0000	0.34	ADH5, CYB5R3, G6PD, LDHA, LDHB, OSBPL2, PLEK, SOD1
1.0000	0.34	CSDE1, DHX9, HNRNPA2B1, HNRNPC, HNRNPU, NPM1, PTBP1, SYNCRIP
1.0000	0.34	CCT2, CCT4, CCT6A, CCT7, CCT8, HNRNPA2B1, TCP1, XRCC5

1.0000	0.34	CCT2, CCT4, CCT6A, CCT7, CCT8, HSP90AA1, TCP1, XRCC5
1.0000	0.34	CCT2, CCT4, CCT6A, CCT7, CCT8, HNRNPA2B1, TCP1, XRCC5
1.0000	0.34	ANXA2, FCN1, GNAI2, GNAQ, GNG5, JUP, OSBPL2, RHOA
1.0000	0.34	ANXA2, FCN1, GNAI2, GNAQ, GNG5, JUP, OSBPL2, RHOA
1.0000	0.65	ACTB, ADAM10, ALDOA, ANPEP, ANXA2, ARL6IP5, ATIC, ATP5F1B, CCL5, CCT2, CCT4, CCT
1.0000	0.51	ADAM10, ATIC, ATP5F1B, CAMP, CCL5, CD47, CDC42, DHX9, EIF5A, FCER1G, GAPDH, GST
1.0000	0.50	AACS, ACTB, ADAM10, ADH5, ALDOA, ANK1, ANPEP, ANTXR2, ANXA11, ANXA2, ANXA5,
1.0000	0.58	ACTB, ADAM10, ADH5, ALDOA, ANK1, ANPEP, ANTXR2, ANXA11, ANXA2, ANXA7, ARL6IP
1.0000	0.48	CYBB, ENO1, F11R, GNAQ, HSP90AA1, HSPA1B, HSPD1, ITGB1, ITGB3, JUP, KRT5, NPM1,
1.0000	0.48	ANXA11, ATP2A3, ATP5F1B, CALM3, CLIC1, CYB5R3, CYBB, EIF5A, ENO1, GAPDH, GNAQ,
1.0000	0.48	ANXA11, ATP2A3, ATP5F1B, CALM3, CLIC1, CYB5R3, CYBB, EIF5A, ENO1, GAPDH, GNAQ,
1.0000	0.39	ALDOA, ATIC, ATP5F1B, ENO1, G6PD, GAPDH, HINT1, HSPA1B, HSPA8, LDHA, PGK1, PKM
1.0000	0.39	ARL6IP5, ATP2A3, BCAP31, EIF5A, ENO1, HINT1, PPIA, RPL11, S100A8, S100A9, SOD1, T
1.0000	0.39	CALM3, CCT4, CCT8, CDC42, DHX9, FLOT1, G6PD, GNAI2, HNRNPU, HSPA1B, MYH16, NP
1.0000	0.43	B2M, CCL5, CD226, CD47, CD59, DHX9, FCER1G, FCN1, HLA-A, HLA-B, HSP90AA1, HSPD1,
1.0000	0.43	ACTB, CCT2, CCT4, CCT6A, CCT7, CCT8, CSDE1, DHX9, HNRNPU, HSPA8, KANSL3, PSMA5,
1.0000	0.43	ATP2A3, ATP5F1B, CCT2, CCT4, CCT6A, CCT7, CCT8, DDX60L, DHX9, G3BP1, HSP90AA1,
1.0000	0.49	ACTB, ADAM10, B2M, CCL5, CD226, CD36, CD47, CD59, CD99, DHX9, FCER1G, FCN1, HLA
1.0000	0.49	B2M, CCL5, CD226, CD36, CD47, CD59, DHX9, FCER1G, FCN1, FLOT1, HLA-A, HLA-B, HSP
1.0000	0.29	B2M, CD226, FCER1G, HLA-A, HLA-B, HSPD1, INPP5D, SPN, TFRC
1.0000	0.29	B2M, CD226, FCER1G, HLA-A, HLA-B, HSPD1, INPP5D, SPN, TFRC
1.0000	0.29	CYBB, GSTP1, HSP90AA1, ITGB3, RHOA, SOD1, TFRC, THBS1, XRCC5
1.0000	0.29	CCL5, CFL1, DDX60L, DHX9, EIF5A, ENO1, G3BP1, HSP90AA1, RPS15A
1.0000	0.29	ALDOA, CCT2, CCT4, CCT7, CCT8, CD9, MSN, TCP1, VDAC2
1.0000	0.29	ANXA11, B2M, CALM3, CYBB, G6PD, S100A8, SOD1, TFRC, THBS1
1.0000	0.29	DAZAP1, DHX9, HNRNPA2B1, HNRNPU, HSPA8, NCL, NPM1, PRDX6, PTBP1
1.0000	0.29	ARPC5, HSP90AA1, HSPA1B, KRT5, MYADM, PFN1, RHOA, SPTBN1, VDAC2
1.0000	0.29	CALM3, CCL5, G6PD, GNAI2, ITGB3, RHOA, SRI, UBQLN1, YWHAE
1.0000	0.29	ANK1, ANXA2, DSP, FLOT1, HSP90AA1, LRP1, MSN, SLC2A1, TFRC
1.0000	0.29	ANK1, ANXA2, DSP, FLOT1, HSP90AA1, LRP1, MSN, SLC2A1, TFRC
1.0000	0.29	ANK1, ANXA2, DSP, FLOT1, HSP90AA1, LRP1, MSN, SLC2A1, TFRC
1.0000	0.29	DAZAP1, DDX60L, DHX9, HNRNPC, HNRNPF, HNRNPH1, HNRNPU, PTBP1, SYNCRIP
1.0000	0.29	DHX9, ENO1, HNRNPA2B1, HNRNPU, HSPD1, NCL, NPM1, PCBP1, XRCC5
1.0000	0.60	ACTB, ALDOA, ANK1, ANXA2, ARPC5, BCAP31, CALM3, CCT2, CCT4, CCT6A, CCT7, CCT8,
1.0000	0.35	ALDOA, ATIC, ATP5F1B, ENO1, G6PD, GAPDH, HINT1, HSPA1B, HSPA8, LDHA, LDHB, PGK
1.0000	0.35	ALDOA, ATIC, ATP5F1B, ENO1, G6PD, GAPDH, HINT1, HSPA1B, HSPA8, LDHA, LDHB, PGK
1.0000	0.51	ADAM10, ALDOA, ARPC5, B2M, CCT2, CCT8, CD36, CDC42, CLTA, DHX9, FERMT3, FSCN1,
1.0000	0.26	ATP5F1B, CLIC1, DHX9, HNRNPC, HNRNPU, ILK, LTF, RPS15, SYNCRIP, TPM4
1.0000	0.26	ADAM10, CCL5, CD47, CD99, ITGB3, LBP, RAC2, RHOA, SPN, THBS1
1.0000	0.26	BCAP31, CALM3, HSP90AA1, HSPA9, HSPD1, LRBA, RAC2, TFRC, UBE2L3, VDAC2
1.0000	0.26	DHX9, HSP90AA1, NPM1, RPL11, RPLP0, RPS15, RPSA, SF3A3, WBP11, XRCC5
1.0000	0.26	CASP14, HRNR, KRT1, KRT16, KRT17, KRT2, KRT5, KRT6A, KRT6B, KRT78
1.0000	0.26	CCT2, CCT4, CCT6A, CCT7, CCT8, HNRNPA2B1, HNRNPC, HNRNPU, TCP1, XRCC5
1.0000	0.26	CCT2, CCT4, CCT6A, CCT7, CCT8, HNRNPA2B1, HNRNPC, HNRNPU, TCP1, XRCC5
1.0000	0.26	ANXA2, B2M, FLOT1, HLA-A, HLA-B, HSPD1, LRP1, LTF, RCC2, TFRC
1.0000	0.26	ATP5F1B, CALM3, CYB5R3, HSPA9, HSPD1, LDHB, PNPLA8, RAC2, TMX2, VDAC2
1.0000	0.44	ATP2A3, CCT2, CCT4, CCT6A, CCT7, CCT8, CDC42, DDX60L, DHX9, EEF1A1, EEF2, G3BP1,

1.0000	0.44	ATP2A3, CCT2, CCT4, CCT6A, CCT7, CCT8, CDC42, DDX60L, DHX9, EEF1A1, EEF2, G3BP1, (
1.0000	0.44	ATP2A3, CCT2, CCT4, CCT6A, CCT7, CCT8, CDC42, DDX60L, DHX9, EEF1A1, EEF2, G3BP1, (
1.0000	0.44	ATP2A3, CCT2, CCT4, CCT6A, CCT7, CCT8, CDC42, DDX60L, DHX9, EEF1A1, EEF2, G3BP1, (
1.0000	0.55	ACTB, ADAM10, ADH5, ALDOA, ANK1, ANPEP, ANTXR2, ANXA11, ANXA2, ANXA7, ARL6IP
1.0000	0.49	ACTB, ANK1, ARPC5, CLTA, DSP, FLG2, H2AC20, H2BC21, JUP, KRT1, KRT10, KRT14, KRT1!
1.0000	0.33	CCL5, CSDE1, DHX9, EEF2, EIF5A, GAPDH, HNRNPU, KRT17, NCL, NPM1, PKM, RHOA, SYN
1.0000	0.33	ANXA2, BCAP31, CSDE1, DHX9, EEF1A1, GAPDH, HNRNPC, HNRNPU, HSPA1B, NPM1, QS(
1.0000	0.33	ALDOA, ATIC, ATP5F1B, ENO1, G6PD, GAPDH, HINT1, HSPA1B, HSPA8, LDHA, LDHB, NAN
1.0000	0.33	CCL5, CSDE1, DHX9, EEF2, EIF5A, GAPDH, HNRNPU, KRT17, NCL, NPM1, PKM, RHOA, SYN
1.0000	0.33	CALM3, CCT4, CCT8, CDC42, DHX9, FLOT1, G6PD, GNAI2, HNRNPU, HSPA1B, MYH16, NPI
1.0000	0.42	ADAM10, BCAP31, CALM3, CCT2, CCT4, CCT6A, CCT7, CCT8, CD36, DHX9, FLNA, HNRNPU
1.0000	0.23	CCT2, CCT4, CCT6A, CCT7, CCT8, HNRNPC, HNRNPU, HSP90AA1, TCP1, TNKS1BP1, XRCC5
1.0000	0.23	CCT2, CCT4, CCT6A, CCT7, CCT8, DHX9, FLOT1, HNRNPA2B1, HNRNPU, NPM1, TCP1
1.0000	0.23	CCT2, CCT4, CCT6A, CCT7, CCT8, HNRNPC, HNRNPU, HSP90AA1, TCP1, TNKS1BP1, XRCC5
1.0000	0.23	BCAP31, CSDE1, DHX9, HNRNPU, HSP90AA1, HSPA1B, LRP1, MSN, SYNCRIP, TNKS1BP1, U
1.0000	0.23	ADAM10, CFL1, EIF5A, FLOT1, FSCN1, HSPA8, LRBA, LRP1, PLEK, SPTBN1, UBQLN1
1.0000	0.23	CSDE1, DHX9, HINT1, HNRNPC, HNRNPU, HSPA1B, NPM1, SYNCRIP, TNKS1BP1, VIM, YWI
1.0000	0.23	ATP5F1B, CALM3, CYB5R3, HSPA9, HSPD1, LDHB, PNPLA8, RAC2, SOD1, TMX2, VDAC2
1.0000	0.23	CANX, DHX9, NPM1, RPL11, RPL8, RPLP0, RPS15, RPS15A, RPS23, RPS3A, RPSA
1.0000	0.23	CASP14, KRT1, KRT10, KRT14, KRT17, KRT2, KRT5, KRT6A, KRT6B, KRT78, KRT9
1.0000	0.23	CCT2, CCT4, CCT6A, CCT7, CCT8, HSP90AA1, HSPA1B, HSPA8, HSPA9, HSPD1, TCP1
1.0000	0.23	CCT2, CCT4, CCT6A, CCT7, CCT8, HSP90AA1, HSPA1B, HSPA8, HSPA9, HSPD1, TCP1
1.0000	0.30	DAZAP1, DHX9, HNRNPA2B1, HNRNPA3, HNRNPC, HNRNPF, HNRNPH1, HNRNPH3, HNRN
1.0000	0.30	DAZAP1, DHX9, HNRNPA2B1, HNRNPA3, HNRNPC, HNRNPF, HNRNPH1, HNRNPH3, HNRN
1.0000	0.30	DAZAP1, DHX9, HNRNPA2B1, HNRNPA3, HNRNPC, HNRNPF, HNRNPH1, HNRNPH3, HNRN
1.0000	0.30	DAZAP1, DHX9, HNRNPA2B1, HNRNPA3, HNRNPC, HNRNPF, HNRNPH1, HNRNPH3, HNRN
1.0000	0.30	DAZAP1, DHX9, HNRNPA2B1, HNRNPA3, HNRNPC, HNRNPF, HNRNPH1, HNRNPH3, HNRN
1.0000	0.30	CASP14, DSP, FLG2, HRNR, KRT1, KRT10, KRT14, KRT15, KRT16, KRT17, KRT2, KRT5, KRT6
1.0000	0.30	BCAP31, CANX, CSDE1, DHX9, HNRNPC, HNRNPU, HSPA1B, NPM1, PCYOX1, PSMA5, PSM
1.0000	0.40	ACTB, CALM3, CASP14, CCT2, CCT4, CCT6A, CCT7, CCT8, DSP, HNRNPU, JUP, KRT1, KRT1(
1.0000	0.40	ACTB, DAZAP1, DHX9, EEF2, G3BP1, GAPDH, HNRNPA2B1, HNRNPA3, HNRNPC, HNRNPF,
1.0000	0.46	ADAM10, ALDOA, ARPC5, B2M, CCT2, CCT8, CD36, CDC42, CFL1, CLTA, DHX9, EIF5A, FER
1.0000	0.21	ATP5F1B, CLIC1, DHX9, HNRNPC, HNRNPU, ILK, LTF, RHOA, RPS15, SRGN, SYNCRIP, TPM4
1.0000	0.21	CASP14, DSP, HRNR, KRT1, KRT10, KRT16, KRT17, KRT2, KRT5, KRT6A, KRT6B, KRT78
1.0000	0.28	CCL5, CSDE1, DHX9, EEF2, EIF5A, GAPDH, HNRNPC, HNRNPU, KRT17, MATR3, NCL, NPM1
1.0000	0.46	ACTB, ANXA2, BCAP31, CANX, CCL5, CCT2, CCT4, CCT6A, CCT7, CCT8, CSDE1, DHX9, EEF1
1.0000	0.26	DAZAP1, DHX9, HNRNPA2B1, HNRNPA3, HNRNPC, HNRNPF, HNRNPH1, HNRNPH3, HNRN
1.0000	0.19	CSDE1, DHX9, HNRNPU, PKM, RPL11, RPL8, RPLP0, RPS15, RPS15A, RPS23, RPS3A, RPSA,
1.0000	0.19	CASP14, DSP, HRNR, KRT1, KRT10, KRT16, KRT17, KRT2, KRT5, KRT6A, KRT6B, KRT78, SOI
1.0000	0.19	CCL5, CSDE1, DHX9, EEF2, EIF5A, HNRNPU, KRT17, NPM1, PKM, RHOA, SYNCRIP, THBS1,
1.0000	0.19	DSP, KRT1, KRT14, KRT15, KRT16, KRT17, KRT2, KRT5, KRT6A, KRT6B, KRT78, KRT9, VIM
1.0000	0.19	CCL5, CSDE1, DHX9, EEF2, EIF5A, HNRNPU, KRT17, NPM1, PKM, RHOA, SYNCRIP, THBS1,
1.0000	0.19	DHX9, EEF1A1, FLNA, HSPA8, HSPA9, NCL, NPM1, PTBP1, RCC2, RPL11, RPS3A, SPTBN1, X
1.0000	0.19	CALM3, CCT2, CCT4, CCT6A, CCT7, CCT8, HNRNPU, RCC2, TCP1, TUBA1C, TUBA4A, TUBB,
1.0000	0.41	ANXA2, B2M, CCL5, CD226, CD36, CSDE1, CYBB, DAZAP1, DHX9, EEF2, EIF5A, ENO1, FCEF
1.0000	0.36	ADAM10, ANPEP, ARL6IP5, CCL5, CPN1, CSDE1, DHX9, EEF1A1, EEF2, EIF5A, G6PD, GAPD
1.0000	0.36	ADAM10, ANPEP, ARL6IP5, CCL5, CPN1, CSDE1, DHX9, EEF1A1, EEF2, EIF5A, G6PD, GAPD

1.0000	0.25	ALDOA, ATIC, ATP5F1B, ENO1, G6PD, GAPDH, HINT1, HSPA1B, HSPA8, INPP5D, LDHA, LD
1.0000	0.17	DSP, KRT1, KRT14, KRT15, KRT16, KRT17, KRT2, KRT5, KRT6A, KRT6B, KRT78, KRT9, SOD1
1.0000	0.17	DSP, KRT1, KRT14, KRT15, KRT16, KRT17, KRT2, KRT5, KRT6A, KRT6B, KRT78, KRT9, SOD1
1.0000	0.17	CSDE1, DAZAP1, DHX9, HNRNPA2B1, HNRNPC, HNRNPU, HSPA8, NCL, NPM1, PRDX6, PTI
1.0000	0.16	ATP2A3, CCT2, CCT4, CCT6A, CCT7, CCT8, DDX60L, DHX9, G3BP1, HSP90AA1, HSPA1B, HS
1.0000	0.22	ANXA2, CSDE1, DAZAP1, DHX9, HNRNPA2B1, HNRNPA3, HNRNPC, HNRNPF, HNRNPH1, H
1.0000	0.44	ACTB, ADAM10, ALDOA, ANXA11, ANXA2, ANXA7, ARPC5, ATP2A3, ATP5F1B, CALM3, CA
1.0000	0.19	CCL5, CSDE1, DHX9, EEF1A1, EEF2, EIF5A, GAPDH, HNRNPU, KRT17, NCL, NPM1, PKM, RH
1.0000	0.19	CCL5, CSDE1, DHX9, EEF1A1, EEF2, EIF5A, GAPDH, HNRNPU, KRT17, NCL, NPM1, PKM, RH
1.0000	0.19	CCL5, CSDE1, DHX9, EEF1A1, EEF2, EIF5A, GAPDH, HNRNPU, KRT17, NCL, NPM1, PKM, RH
1.0000	0.19	CCL5, CSDE1, DHX9, EEF1A1, EEF2, EIF5A, GAPDH, HNRNPU, KRT17, NCL, NPM1, PKM, RH
1.0000	0.14	ALDOA, ATIC, ATP5F1B, CCL5, CSDE1, DHX9, EEF1A1, EEF2, EIF5A, ENO1, GAPDH, GSTP1,

)C42, F11R, FCN1, FERMT3, FLNA, FLOT1, GNAI2, GNAQ, HLA-A, HLA-B, HSP90AA1, HSPA1B, HSPA8, ICAI

;, ITGB3, JUP, PATJ, PECAM1, PFN1, PTPRJ, RAP1B, RCC2, RHOA, THBS1, TLN1, TUBB, VCL, YWHAZ
GNAI2, HINT1, HSP90AA1, HSPA1B, ILK, ITGB1, ITGB3, JUP, LBP, LTF, PPIA, PRDX2, PTBP1, PTPRJ, RAP1B,

GAPDH, GNAI2, HINT1, HSP90AA1, HSPA1B, ILK, ITGB1, ITGB3, JUP, LBP, LTF, PPIA, PRDX2, PTBP1, PTPRJ

'1BA, GP1BB, ILK, ITGB3, KRT1, MMRN1, MYH9, PF4V1, PLEK, PPIA, PRDX2, THBS1, TLN1, VCL
'1BA, GP1BB, ILK, ITGB3, KRT1, MMRN1, MYH9, PF4V1, PLEK, PPIA, PRDX2, THBS1, TLN1, VCL
'1BA, GP1BB, ILK, ITGB3, KRT1, MMRN1, MYH9, PF4V1, PLEK, PPIA, PRDX2, THBS1, TLN1, VCL

, PPIA, RAB10, RAC2, RHOA, RTN3, RTN4, S100A8, S100A9, SOD1, VCL, VIM, XRCC5, YWHAE, YWHAG, Y\
B1, ITGB3, JUP, MSN, MYADM, MYH9, PATJ, PECAM1, PTPRJ, RAB10, RAP1B, RHOA, SLC2A1, TLN1, TNKS

IA, GNAQ, GP1BA, GP1BB, ILK, ITGB1, ITGB3, KRT1, KRT6A, MMRN1, MYH9, PF4V1, PLEK, PPIA, PRDX2, I

, FERMT3, FLNA, FSCN1, GNAI2, GP1BA, HSP90AA1, ILK, ITGA6, ITGB1, ITGB3, JUP, KRT16, KRT2, KRT5, L

NPU, ILK, ITGA2B, ITGB1, ITGB3, JUP, KRT1, LRP1, MATR3, MYH9, NCL, PECAM1, PGK1, PKM, RHOA, RTN

NA, GNAQ, GP1BA, GP1BB, ILK, ITGB1, ITGB3, KRT1, KRT6A, MMRN1, MYH9, PF4V1, PLEK, PPIA, PRDX2, F

, HINT1, HSP90AA1, HSPA1B, ILK, ITGB1, ITGB3, JUP, LBP, LTF, PPIA, PRDX2, PTBP1, PTPRJ, RAP1B, RHOA
NRNPU, HSP90AA1, ILK, ITGA6, ITGB1, ITGB3, KRT2, LRP1, LTF, MSN, MYADM, MYH9, PECAM1, RAB10, F

NPA2B1, HSP90AA1, HSPA8, HSPA9, HSPD1, ITGB1, ITGB3, JUP, LRP1, M6PR, MYH9, NASP, NPM1, PLEK,
ITGB3, MSN, MYADM, MYH9, MYL12B, RAB10, RAC2, RCC2, RHOA, RTN4, SOD1, VCL, YWHAH

D1, F11R, FLNA, FLOT1, G3BP1, GAPDH, GNAI2, GNAQ, GSTP1, HINT1, HSP90AA1, HSPA1B, ILK, INPP5D,
FL1, DSC1, DSG1, DSP, F11R, FERMT3, FLNA, FLOT1, FSCN1, G3BP1, HSPA1B, HSPA8, HSPA9, ILK, ITGA2B
TP5F1B, B2M, CAMP, CANX, CCL5, CCT2, CCT4, CCT6A, CCT7, CCT8, CD36, CD47, CD53, CD59, CD9, CDC

SPA1B, HSPA8, ILK, ITGA6, JUP, KANSL3, LTF, NCL, NPM1, PCBP1, PFN1, PPIA, PRDX2, PSMA6, RHOA, S10

SPA1B, HSPA8, ILK, ITGA6, JUP, KANSL3, LTF, NCL, NPM1, PCBP1, PFN1, PPIA, PRDX2, PSMA6, RHOA, S10
SPA1B, HSPA8, ILK, ITGA6, JUP, KANSL3, LTF, NCL, NPM1, PCBP1, PFN1, PPIA, PRDX2, PSMA6, RHOA, S10
SPA1B, HSPA8, ILK, ITGA6, JUP, KANSL3, LTF, NCL, NPM1, PCBP1, PFN1, PPIA, PRDX2, PSMA6, RHOA, S10
SPA1B, HSPA8, ILK, ITGA6, JUP, KANSL3, LTF, NCL, NPM1, PCBP1, PFN1, PPIA, PRDX2, PSMA6, RHOA, S10
SPA1B, HSPA8, ILK, ITGA6, JUP, KANSL3, LTF, NCL, NPM1, PCBP1, PFN1, PPIA, PRDX2, PSMA6, RHOA, S10
LNA, GP1BA, GP1BB, HLA-A, HLA-B, ICAM2, ITGA2B, ITGA6, ITGB1, ITGB3, LRP1, M6PR, PECAM1, PTPRJ, I
T1, HNRNPA2B1, HSP90AA1, HSPA8, HSPA9, HSPD1, ITGB1, ITGB3, JUP, LBP, LRP1, M6PR, MYH9, NASP,
D1, JUP, LRP1, M6PR, MYH9, NASP, NPM1, PLEK, PPIA, RAB10, RAB1B, RAB27B, RAB33B, RAC2, RPL11, S
MT3, FLNA, FSCN1, GNAI2, GP1BA, HSP90AA1, ILK, ITGA6, ITGB1, ITGB3, JUP, KRT16, KRT2, KRT5, LBP, LR
NA, FLOT1, GP1BA, HLA-A, HSPD1, ICAM2, ILK, ITGA2B, ITGA6, ITGB1, ITGB3, JUP, MMRN1, MSN, MYADM

G, HLA-B, ITGA2B, ITGB3, PECAM1, PTPRJ, RAB10, RAB27B, RAP1B, RHOA, SLC2A3, SRI, STOM, TSPAN14
CDC42, CLIC1, DSC1, DSG1, DSP, F11R, FERMT3, FLG2, FLNA, FLOT1, GP1BA, GP1BB, HLA-A, HSPD1, ICAM
L, JUP, LDHA, MYH9, PCBP1, PFN1, PKM, PRDX1, PRDX6, PTPRJ, RAB10, RTN4, SPTBN1, TAGLN2, TLN1, TI

, CD226, CD36, CD47, CD53, CD59, CDC42, CFL1, CLIC1, CYBB, DSP, EIF5A, ENO1, F11R, FCER1G, FCN1, FI
I2, HNRNPU, HSP90AA1, HSPA1B, HSPA8, ILK, ITGB1, ITGB3, JUP, LRP1, NCL, PKM, RAB10, RAP1B, RHOA,
R1G, FLNA, GP1BA, GP1BB, HLA-A, HLA-B, ICAM2, ITGA2B, ITGA6, ITGB1, ITGB3, LRP1, M6PR, PECAM1, P
, G3BP1, GNAI2, GNAQ, GP1BA, GP1BB, GSTP1, HLA-A, HSPA1B, ILK, INPP5D, ITGA2B, ITGA6, ITGB1, ITGI
F2, ENO1, F11R, FERMT3, FLNA, FSCN1, HSPA8, ICAM2, ITGA2B, ITGA6, ITGB1, ITGB3, JUP, LDHA, MSN, I
RMT3, FLNA, GNAQ, GP1BA, GP1BB, HLA-A, HSPD1, ILK, INPP5D, ITGB1, ITGB3, KRT2, LBP, LRP1, MMRN1
, CD47, CD53, CD59, CDC42, CFL1, CLIC1, CYBB, EIF5A, ENO1, F11R, FCER1G, FCN1, FERMT3, FLNA, FLOT

CDC42, EIF5A, ENO1, F11R, FLNA, FLOT1, G3BP1, GAPDH, GNAI2, GNAQ, GSTP1, HINT1, HSP90AA1, HSPA
5, ATIC, ATP5F1B, B2M, CAMP, CANX, CCL5, CCT2, CCT4, CCT6A, CCT7, CCT8, CD36, CD47, CD53, CD59, C

CYBB, DSP, F11R, FERMT3, FLG2, FLNA, FLOT1, GNG5, GP1BA, HSP90AA1, ILK, ITGA2B, ITGA6, ITGB1, ITG

, CD36, CD47, CD53, CD59, CDC42, CFL1, CLIC1, CYBB, DSP, EIF5A, ENO1, F11R, FCER1G, FCN1, FERMT3,
CD99, CDC42, CFL1, CLTA, DSC1, DSG1, DSP, EEF2, F11R, FERMT3, FLNA, FLOT1, FSCN1, G3BP1, GNAI2, G
, ATP5F1B, B2M, BCAP31, CALM3, CANX, CASP14, CAVIN2, CD151, CD226, CD36, CD47, CD53, CD59, CD
1, ITGB1, ITGB3, JUP, LRP1, NCL, PKM, RAB10, RAP1B, RHOA, RPLP0, SLC2A1, SOD1, UBQLN1, VIM, YWH
TGB1, ITGB3, JUP, KRT1, MATR3, MYH9, NCL, PFN1, PGK1, PKM, RHOA, RTN4, SPTBN1, THBS1, YWHAZ
P1, LTF, MSN, NCL, PF4V1, PKM, PRDX1, PRDX2, RAB10, RAP1B, RHOA, RPLP0, SOD1, SRI, VIM, XRCC5, Y
FLNA, HNRNPA2B1, HNRNPU, HSP90AA1, HSPA8, HSPA9, HSPD1, ITGB1, ITGB3, JUP, LRP1, M6PR, MSN, I
M3, CAMP, CANX, CCT2, CCT4, CCT8, CD36, CD47, CD53, CD59, CD9, CDC42, CLTA, CTSD, CYB5R3, CYBB,
M3, CAMP, CANX, CCT2, CCT4, CCT8, CD36, CD47, CD53, CD59, CD9, CDC42, CLTA, CTSD, CYB5R3, CYBB,

9, CFL1, CLTA, DHX9, EEF2, EIF5A, F11R, FCER1G, FERMT3, FLNA, FSCN1, GNAI2, GNAQ, HLA-A, HNRNPC
TP5F1B, B2M, CAMP, CANX, CCT2, CCT4, CCT6A, CCT7, CCT8, CD47, CD53, CD59, CD9, CDC42, CFL1, CLI
TP5F1B, B2M, CAMP, CANX, CCT2, CCT4, CCT6A, CCT7, CCT8, CD47, CD53, CD59, CD9, CDC42, CFL1, CLI
TP5F1B, B2M, CAMP, CANX, CCT2, CCT4, CCT6A, CCT7, CCT8, CD47, CD53, CD59, CD9, CDC42, CFL1, CLI
TP5F1B, B2M, CAMP, CANX, CCT2, CCT4, CCT6A, CCT7, CCT8, CD47, CD53, CD59, CD9, CDC42, CFL1, CLI

1, HSP90AA1, ITGB3, JUP, LBP, LRP1, LTF, MSN, NASP, NCL, PF4V1, PKM, PRDX1, PRDX2, RAB10, RAP1B,
, CLTA, CTSD, CYBB, DSC1, DSG1, DSP, FCER1G, HLA-A, HLA-B, HSPA8, ITGA2B, ITGB1, ITGB3, LRP1, M6PF
, CLTA, CTSD, CYBB, DSC1, DSG1, DSP, FCER1G, HLA-A, HLA-B, HSPA8, ITGA2B, ITGB1, ITGB3, LRP1, M6PF
AI2, GNAQ, GP1BA, GP1BB, HRNR, ILK, ITGB3, KRT1, KRT16, MMRN1, MYH9, PF4V1, PLEK, PPIA, PRDX2,
AA1, HSPA1B, ILK, ITGB1, ITGB3, LTF, PPIA, PRDX2, PTBP1, PTPRJ, RAP1B, RHOA, RPL11, RPS15, RTN4, S1

CT2, CCT4, CCT6A, CCT7, CCT8, CD36, CDC42, CFL1, CLTA, CTSD, DHX9, DSP, F11R, FCN1, FLNA, FLOT1, G

ı1, F11R, FLNA, G6PD, GNAI2, HSP90AA1, ITGB1, JUP, LRP1, MYL6, PTGS1, PTPRJ, RHOA, SLC2A1, SLC2A3

., CCT7, CCT8, CD36, CDC42, CLTA, CTSD, DHX9, DSP, F11R, FCN1, FLNA, FLOT1, HNRNPU, HSP90AA1, HS
., CCT7, CCT8, CD36, CDC42, CLTA, CTSD, DHX9, DSP, F11R, FCN1, FLNA, FLOT1, HNRNPU, HSP90AA1, HS

TA, CTSD, FLNA, HSP90AA1, HSPA8, HSPA9, HSPD1, JUP, LRP1, M6PR, MYH9, NASP, NPM1, PLEK, PPIA, R

)9, CDC42, CTSD, CYBB, DHX9, EEF2, ENDOD1, F11R, FCER1G, FCN1, FLNA, FLOT1, G3BP1, G6PD, GAPDH,
l, CCT6A, CCT7, CCT8, CD36, CDC42, CLTA, CTSD, DHX9, DSP, F11R, FCN1, FLNA, FLOT1, HNRNPA2B1, HN
1R, FLNA, FLOT1, G3BP1, GAPDH, GNAI2, GNAQ, GSTP1, HINT1, HSP90AA1, HSPA1B, ILK, INPP5D, ITGA6,
NA, GP1BA, GSTP1, HLA-A, HLA-B, HSP90AA1, ITGB1, KRT1, LBP, MATR3, MMRN1, NPM1, PNPLA8, PPIA,

CCT8, CD36, CD47, CD53, CD59, CD9, CLTA, CTSD, CYB5R3, CYBB, DSC1, DSG1, DSP, EEF1A1, EEF2, FCER1

5, ATIC, ATP5F1B, B2M, BCAP31, CALM3, CAMP, CANX, CCT2, CCT4, CCT6A, CCT7, CCT8, CD36, CD47, CD

)151, CD226, CD36, CD47, CD53, CD9, CD99, CLIC1, CYBB, DCD, DSC1, DSG1, F11R, FCER1G, FLNA, GP1B

36, CD47, CD53, CD59, CD9, CTSD, CYB5R3, CYBB, DSC1, DSG1, DSP, EEF1A1, EEF2, FCER1G, FCN1, FERN
A, HSP90AA1, HSPA8, ITGB1, ITGB3, JUP, KRT5, LRP1, MSN, NPM1, PECAM1, PPIA, RAC2, RTN4, SPTBN1,

LOT1, GP1BA, HLA-A, HSPD1, ILK, ITGB3, JUP, MMRN1, MYADM, PRDX2, PTPRJ, RAC2, RCC2, RHOA, SPN,
IAI2, GNAQ, HSP90AA1, ITGB1, ITGB3, JUP, LRP1, NCL, NPM1, PKM, RAB10, RAP1B, RHOA, RPLP0, SLC2A

Q, HRNR, HSP90AA1, HSPA8, LRBA, LRP1, LYZ, M6PR, PCYOX1, PRDX6, RAP1B, SOD1, SRGN, STOM, TUBE

EEF1A1, ENO1, FLNA, GALE, GAPDH, HINT1, HNRNPC, HNRNPU, HSP90AA1, HSPA1B, HSPA8, ITGB1, LDH
CD53, CD59, CD9, CDC42, CLTA, CTSD, CYB5R3, CYBB, DSC1, DSG1, DSP, EEF1A1, ENO1, FCER1G, FLOT1,
T1, G3BP1, HSPA1B, HSPA8, HSPA9, ILK, ITGA2B, ITGA6, ITGB1, ITGB3, JUP, LRP1, MSN, MYH9, NPM1, P
, HSP90AA1, ILK, ITGA6, ITGB1, LRP1, PFN1, PPIA, RAB10, RAC2, RHOA, RTN3, RTN4, S100A8, S100A9, SL
ANXA7, ARL6IP5, ARPC5, ATIC, ATP2A3, ATP5F1B, B2M, BCAP31, CALM3, CAMP, CANX, CASP14, CAVIN2,

ISPA9, ILK, INPP5D, ITGB1, ITGB3, LTF, MYADM, PRDX2, PTBP1, RCC2, RHOA, RTN4, SOD1, VCL, XRCC5, Y
A1B, ILK, INPP5D, ITGB1, ITGB3, JUP, KRT10, KRT17, KRT2, LTF, MYADM, PKM, RHOA, RTN4, TFRC, THBS1
3C21, HSP90AA1, ITGB1, JUP, KRT1, KRT10, MYH9, NPM1, PECAM1, PLEK, SRI, STOM, TFRC, TKT, TPM4, Y
3CAP31, CALM3, CANX, CASP14, CAVIN2, CD151, CD226, CD36, CD47, CD53, CD59, CD9, CD99, CDC42, C
, CDC42, CTSD, DHX9, EEF1A1, F11R, FLNA, FLOT1, GAPDH, GNAI2, GNAQ, GSTP1, HNRNPA2B1, HNRNPU
D151, CD226, CD36, CD47, CD53, CD59, CD9, CD99, CLIC1, CYBB, DCD, DSC1, DSG1, F11R, FCER1G, FLNA,

2B1, HNRNPC, HNRNPF, HNRNPH1, HNRNPH3, HNRNPU, HSPA1B, HSPA8, ILK, ITGA6, JUP, KANSL3, LTF,
FLNA, FLOT1, G3BP1, HSPA1B, HSPA8, HSPA9, ILK, ITGA2B, ITGA6, ITGB1, ITGB3, JUP, LRP1, MSN, MYH9,

, GNAI2, GSTP1, HINT1, HSP90AA1, HSPA1B, ILK, ITGB1, ITGB3, LTF, MYADM, NPM1, PLEK, PPIA, PRDX1,
-2, CFL1, CSDE1, CYBB, DSP, EEF2, FCER1G, FLNA, FLOT1, G6PD, GNG5, GP1BA, GP1BB, GSTP1, HLA-B, HN

ᵢB1, KRT1, KRT10, KRT14, KRT15, KRT16, KRT17, KRT2, KRT5, KRT6A, KRT6B, KRT78, KRT9, MSN, MYADM
AMP, CASP14, CD151, CD36, CD53, CD9, CDC42, CFL1, CSDE1, CYBB, DAZAP1, DSP, EEF2, F11R, FCER1G,
L1R, FCER1G, FLNA, G6PD, GALE, GAPDH, GP1BB, HNRNPC, HNRNPH1, HNRNPU, HSP90AA1, ITGA2B, ITG
LBA, ITGA6, ITGB1, ITGB3, JUP, KRT16, KRT5, LBP, LRP1, MSN, MYADM, PECAM1, PF4V1, PFN1, PPIA, PTI
SD, DHX9, FLNA, HSP90AA1, HSPA8, ITGB1, ITGB3, JUP, KRT5, LRP1, MSN, NPM1, PECAM1, PPIA, RAC2, I
RL6IP5, ARPC5, ATIC, ATP2A3, ATP5F1B, B2M, BCAP31, CALM3, CAMP, CANX, CASP14, CAVIN2, CCL5, CC
ER1G, FCN1, FLNA, FLOT1, G6PD, GNAI2, HSP90AA1, ITGB1, JUP, LRP1, MSN, PPIA, PTPRJ, RAB27B, RAC
SCN1, HSP90AA1, HSPA8, HSPD1, ICAM2, ITGB1, ITGB3, JUP, LRP1, MSN, MYH9, PATJ, PLEK, PTPRJ, RAB
2, CFL1, CSDE1, CYBB, DSP, EEF2, F11R, FCER1G, FLNA, FLOT1, G6PD, GNG5, GP1BA, GP1BB, GSTP1, HLA
90AA1, ITGB1, KRT2, MSN, MYH9, MYL12B, PFN1, RAB10, RAB27B, RCC2, RHOA, S100A8, S100A9, SPTBN
9, CD9, CDC42, DHX9, EIF5A, ENO1, F11R, FCER1G, FCN1, FLNA, FLOT1, G3BP1, G6PD, GAPDH, GNAI2, G

BA, GP1BB, HSPD1, ITGB1, ITGB3, PCYOX1, SLC2A1, SLC2A3, SRI, STOM, THBS1, TMEM109, UBQLN1, VD
GB3, JUP, LRP1, NCL, PKM, RAB10, RAP1B, RHOA, RPLP0, SOD1, THBS1, UBE2L3, VIM, YWHAG, YWHAH
P5D, M6PR, MYH9, NCL, RAB27B, RCC2, RHOA, TLN1, TNKS1BP1, TUBB, VIM, WBP11, YWHAB, YWHAE, Y

, GAPDH, HINT1, HNRNPC, HNRNPU, HSP90AA1, HSPA1B, HSPA8, LRBA, LRP1, NPM1, PCYOX1, PNPLA8,
NO1, FLNA, FLOT1, GAPDH, GSTP1, HINT1, HNRNPA2B1, HNRNPC, HNRNPU, HSP90AA1, HSPA1B, HSPA8
EIF5A, F11R, FCER1G, FCN1, FLNA, FLOT1, GAPDH, GNAI2, HINT1, HLA-A, HLA-B, HSP90AA1, HSPA1B, HS

3, B2M, BCAP31, CALM3, CAMP, CCL5, CCT2, CCT4, CCT6A, CCT7, CCT8, CD151, CD226, CD36, CD47, CD5

ALM3, CANX, CD36, CD47, CD53, CD59, CD9, CDC42, CLIC1, CLTA, CTSD, CYB5R3, CYBB, DSC1, DSG1, DSI

ATP5F1B, B2M, BCAP31, CALM3, CAMP, CANX, CCL5, CD151, CD226, CD36, CD47, CD53, CD59, CD9, CDC
HNRNPU, HRNR, ILK, ITGA6, ITGB1, ITGB3, KRT1, KRT10, KRT14, KRT15, KRT16, KRT17, KRT2, KRT5, KRT6
5A, ENO1, FLNA, FLOT1, HINT1, HNRNPA2B1, HNRNPC, HNRNPU, HSP90AA1, HSPA1B, HSPA8, ILK, ITGA6
CAP31, CALM3, CAMP, CANX, CCL5, CD226, CD36, CD47, CD53, CD59, CD9, CDC42, CFL1, CLIC1, CYBB, DI
K, ITGB3, LRP1, LTF, MYADM, NCL, NPM1, PECAM1, PKM, PPIA, PTPRJ, RHOA, SOD1, SPTBN1, TFRC, THE
LTF, NPM1, PF4V1, PFN1, RAB10, RAB1B, RAB27B, RAC2, RAP1B, RCC2, RHOA, RPL11, RPS15, SRI, UBE2
CD9, CDC42, CLTA, FCER1G, FCN1, FLNA, FLOT1, GNAI2, HSPA1B, HSPA8, ITGB1, ITGB3, LBP, LGALS3BP, L
IF5A, ENO1, FLNA, FLOT1, GAPDH, HINT1, HNRNPA2B1, HNRNPC, HNRNPU, HSP90AA1, HSPA1B, HSPA8,

, HSP90AA1, HSPA8, LRBA, LRP1, LYZ, M6PR, PCYOX1, PRDX6, RAP1B, SOD1, SRGN, STOM, TUBB
, HSP90AA1, HSPA8, LRBA, LRP1, LYZ, M6PR, PCYOX1, PRDX6, RAP1B, SOD1, SRGN, STOM, TUBB
1R, FLNA, FLOT1, G6PD, HLA-B, HNRNPU, HSPA1B, HSPA9, ILK, INPP5D, ITGB1, ITGB3, JUP, KRT1, KRT10,

J, HSP90AA1, HSPA8, HSPA9, HSPD1, JUP, LRP1, M6PR, MSN, NPM1, OSBPL2, RAB10, RAB1B, RAB27B, R

ER1G, FCN1, G3BP1, GAPDH, GSDMA, GSTP1, H2BC21, HLA-A, HLA-B, HSP90AA1, ITGB1, KRT1, KRT16, K
5A, ENO1, FLNA, FLOT1, HINT1, HMBS, HNRNPA2B1, HNRNPC, HNRNPU, HSP90AA1, HSPA1B, HSPA8, ILK
5A, ENO1, FLNA, FLOT1, HINT1, HMBS, HNRNPA2B1, HNRNPC, HNRNPU, HSP90AA1, HSPA1B, HSPA8, ILK
ER1G, FCN1, FLOT1, G6PD, GNAI2, GNAQ, GNG5, GP1BA, GSR, HLA-A, HLA-B, HSPA8, ITGA2B, ITGA6, ITG

RPC5, ATP2A3, ATP5F1B, B2M, BCAP31, CALM3, CAMP, CCL5, CCT2, CCT4, CCT6A, CCT7, CCT8, CD151, C
M, BCAP31, CALM3, CANX, CCL5, CCT2, CCT4, CCT7, CCT8, CD151, CD36, CD47, CD9, CDC42, CLIC1, CLTA
ATP2A3, ATP5F1B, B2M, BCAP31, CALM3, CAMP, CCL5, CCT2, CCT4, CCT6A, CCT7, CCT8, CD151, CD226, (

M, BCAP31, CALM3, CANX, CCL5, CCT2, CCT4, CCT6A, CCT7, CCT8, CD151, CD36, CD47, CD9, CDC42, CFL1
CANX, CCL5, CD36, CD47, CD9, CDC42, CPN1, CYBB, DHX9, DSG1, EEF1A1, EIF5A, FCER1G, FLNA, FLOT1, G
A-A, HLA-B, HSP90AA1, KRT1, LBP, LTF, MATR3, MMRN1, PRDX2, PSMA6, RAC2, S100A8, S100A9, SOD1,
1, HSP90AA1, ILK, ITGB1, ITGB3, JUP, LRBA, MSN, NPM1, PLEK, PTPRJ, RAC2, RCC2, RHOA, TFRC, TUBA4A
01, FLNA, FLOT1, HINT1, HNRNPA2B1, HNRNPC, HNRNPF, HNRNPH1, HNRNPH3, HNRNPU, HSP90AA1, HS
HX9, EIF5A, ENO1, FLNA, FLOT1, G6PD, HINT1, HMBS, HNRNPA2B1, HNRNPC, HNRNPU, HSP90AA1, HSP
7, CCT8, CD36, CDC42, CSDE1, CTSD, DAZAP1, DHX9, EEF1A1, EEF2, EIF5A, ENO1, FLNA, FLOT1, G6PD, GA

DH, GNAQ, GSTP1, HNRNPU, HSP90AA1, ILK, ITGA6, ITGB3, LDHA, LRP1, LTF, MYADM, NCL, NPM1, PECA
CD53, CD59, CD9, ENO1, FCER1G, FCN1, FLOT1, GP1BA, GSR, HLA-A, HLA-B, HNRNPU, HSP90AA1, HSPD1
D, ITGB1, LBP, MSN, MYH9, PRDX1, PRDX2, PTPRJ, RAC2, RHOA, SELPLG, SOD1, SPN, TFRC, THBS1

6, CD47, CDC42, CSDE1, DAZAP1, DHX9, EEF1A1, EEF2, EIF5A, ENO1, FLNA, FLOT1, G6PD, GAPDH, GNAI2
CCT2, CCT4, CCT6A, CCT7, CCT8, CD226, CD36, CD47, CDC42, CSDE1, CTSD, CYBB, DAZAP1, DHX9, EEF1A
CT2, CCT4, CCT6A, CCT7, CCT8, CD151, CD226, CD36, CD47, CD53, CD99, CDC42, CLIC1, CSDE1, CTSD, D

IF5A, ENO1, FLNA, FLOT1, GAPDH, GSTP1, HINT1, HNRNPA2B1, HNRNPC, HNRNPU, HSP90AA1, HSPA1B,
, CAMP, CANX, CCL5, CD36, CD47, CD9, CDC42, CPN1, CYBB, DHX9, DSG1, EEF1A1, EIF5A, ENO1, FCER1G
G6PD, GNAI2, GP1BA, GP1BB, GSR, GSTP1, HRNR, HSPA1B, HSPA9, INPP5D, ITGB1, ITGB3, KRT1, KRT16,
NX, CPN1, CYBB, DCD, DHX9, DSC1, DSG1, ENDOD1, ENO1, FCN1, FLG2, GNAI2, GNAQ, HRNR, ITGA2B, IT

ALM3, CAMP, CANX, CASP14, CD151, CD36, CD53, CD9, CDC42, CFL1, CLIC1, CSDE1, CYBB, DAZAP1, DH
0AA1, ILK, ITGA6, ITGB3, LRP1, LTF, MYADM, NCL, NPM1, PECAM1, PPIA, PTPRJ, RHOA, SOD1, TFRC, TH
LIC1, DAZAP1, DHX9, DSP, EEF2, F11R, FCER1G, FERMT3, FLNA, FLOT1, G6PD, GP1BA, GP1BB, HLA-B, H
LIC1, DAZAP1, DHX9, DSP, EEF2, F11R, FCER1G, FERMT3, FLNA, FLOT1, G6PD, GP1BA, GP1BB, HLA-B, H
2B1, HNRNPA3, HNRNPC, HNRNPF, HNRNPH1, HNRNPH3, HNRNPU, HSPA1B, HSPA8, ILK, ITGA6, JUP, KA
ATP5F1B, B2M, BCAP31, CALM3, CAMP, CASP14, CD226, CD36, CD47, CD59, CD9, CDC42, CFL1, CLIC1, C

F1B, B2M, BCAP31, CALM3, CANX, CCL5, CCT2, CCT4, CCT6A, CCT7, CCT8, CD151, CD36, CD47, CD9, CDC
NPU, HSP90AA1, HSPA1B, HSPA8, JUP, MSN, NASP, NPM1, RAP1B, RHOA, RPLP0, SOD1, THBS1, UBE2L3,
9, CDC42, CFL1, CSDE1, DHX9, ENO1, F11R, FLNA, G3BP1, G6PD, GAPDH, GNAI2, GNAQ, GP1BA, GP1BB,
, FLNA, G6PD, GAPDH, GNAI2, GSDMA, GSTP1, HINT1, HSP90AA1, HSPA1B, HSPA9, HSPD1, INPP5D, ITG
FCN1, G3BP1, GAPDH, GSDMA, H2BC21, HLA-A, HLA-B, HSP90AA1, KRT1, KRT16, KRT6A, LBP, LTF, LYZ,

NX, CPN1, CYBB, DCD, DHX9, DSC1, DSG1, ENDOD1, ENO1, FCN1, FLG2, GNAI2, GNAQ, HRNR, ITGA2B, IT
7, CCT8, CD36, CD47, CSDE1, CTSD, DAZAP1, DHX9, EEF1A1, EEF2, EIF5A, ENO1, FLNA, FLOT1, G6PD, GA

, ENO1, FLNA, G6PD, GAPDH, GNAI2, GSDMA, GSTP1, HINT1, HSP90AA1, HSPA1B, HSPA9, HSPD1, INPP5
FLOT1, G3BP1, GAPDH, GNAI2, GSTP1, HINT1, HSP90AA1, HSPA1B, ILK, INPP5D, ITGB1, ITGB3, LTF, MYA

6, ITGB1, ITGB3, JUP, KRT16, KRT5, LBP, LRP1, MSN, MYADM, PECAM1, PFN1, PTPRJ, RAC2, RCC2, RHO
6, ITGB1, ITGB3, JUP, KRT16, KRT5, LBP, LRP1, MSN, MYADM, PECAM1, PFN1, PTPRJ, RAC2, RCC2, RHO
6, ITGB1, ITGB3, JUP, KRT16, KRT5, LBP, LRP1, MSN, MYADM, PECAM1, PFN1, PTPRJ, RAC2, RCC2, RHO

, LBP, LTF, MSN, NASP, PF4V1, PRDX2, RHOA, RPLP0, S100A8, S100A9, TFRC, THBS1, UBE2L3, VIM, XRCC
NXA7, ARL6IP5, ARPC5, ATIC, ATP2A3, ATP5F1B, B2M, BCAP31, C16orf54, CALM3, CAMP, CANX, CASP1

, G6PD, GAPDH, GNAI2, GSDMA, GSTP1, HINT1, HSP90AA1, HSPA1B, HSPA9, HSPD1, INPP5D, ITGA6, ITG

YBB, EIF5A, FLNA, HLA-A, HLA-B, LRBA, M6PR, PNPLA8, PTGS1, RAB10, RAB1B, RAB27B, RAC2, RHOA, RT
1, HSP90AA1, ILK, ITGB1, ITGB3, JUP, LDHB, LRBA, MSN, NPM1, PLEK, PTPRJ, RAC2, RCC2, RHOA, SLC2A1

1, G3BP1, GAPDH, GNAI2, HLA-A, HSP90AA1, HSPA1B, HSPD1, INPP5D, ITGB1, ITGB3, JUP, KRT17, LBP, L
DHX9, EEF2, EIF5A, ENO1, F11R, FCER1G, FCN1, FLNA, FLOT1, G3BP1, GAPDH, GSTP1, HINT1, HLA-A, HL

CLIC1, CYBB, DHX9, FCER1G, FCN1, FLNA, FLOT1, G6PD, GNAI2, HSP90AA1, HSPA8, ITGB1, ITGB3, JUP, LI
, HNRNPU, ITGB3, LRP1, LTF, NPM1, PPIA, PRDX2, PTPRJ, RCC2, RPL11, RPS15, SRI, THBS1, UBQLN1, YWI
ITGB3, MYH9, PFN1, RAB10, RAB27B, RAB33B, RHOA, RPL8, RPLP0, RPS23, RPS3A, RTN3, RTN4, SLC2A1
AI2, GNAQ, GNG5, GP1BA, GP1BB, HLA-A, HLA-B, HSPA9, HSPD1, ITGA2B, ITGA6, ITGB1, ITGB3, JUP, LRP1
P2A3, B2M, BCAP31, CALM3, CAMP, CANX, CCT2, CCT8, CD36, CD47, CD53, CD59, CD9, CDC42, CLIC1, CL
EIF5A, FCER1G, FLNA, FLOT1, GAPDH, GNAI2, GSTP1, HNRNPU, HSPA1B, HSPA8, HSPA9, HSPD1, ILK, ITGE
CD47, CD59, CD9, CDC42, CFL1, CSDE1, DHX9, ENO1, F11R, FCN1, FLNA, G3BP1, G6PD, GAPDH, GNAI2, G
HNRNPU, HSP90AA1, ILK, ITGA6, ITGB3, LRP1, LTF, MYADM, NCL, NPM1, PECAM1, PLEK, PPIA, PTBP1, P
HNRNPU, HSP90AA1, ILK, ITGA6, ITGB3, LRP1, LTF, MYADM, NCL, NPM1, PECAM1, PLEK, PPIA, PTBP1, P
TA, CSDE1, CTSD, DHX9, DSG1, F11R, FERMT3, FLNA, FLOT1, FSCN1, G3BP1, H2BC21, HNRNPU, HSP90AA

., DHX9, EEF1A1, EIF5A, ENDOD1, ENO1, F11R, FCER1G, FCN1, G3BP1, GAPDH, GSDMA, GSTP1, H2BC21,

NG5, GSTP1, HLA-A, HNRNPU, HSPA1B, HSPD1, ILK, INPP5D, ITGB1, ITGB3, JUP, KRT2, KRT6A, LTF, MSN,
F, MYADM, NCL, NPM1, PECAM1, PPIA, PTPRJ, RHOA, SOD1, TFRC, THBS1, TNKS1BP1, XRCC5, YWHAG
HSPA9, INPP5D, ITGB1, LTF, MYH9, PLEK, PRDX2, PTPRJ, RHOA, SELPLG, SOD1, SPN, TFRC, XRCC5
HSPA9, INPP5D, ITGB1, LTF, MYH9, PLEK, PRDX2, PTPRJ, RHOA, SELPLG, SOD1, SPN, TFRC, XRCC5
GNAQ, HNRNPU, HSP90AA1, HSPA8, ITGB1, PTGS1, RAB27B, RHOA, RPLP0, RTN3, RTN4, SOD1, SPTBN1,
YBB, EEF1A1, FERMT3, FLNA, FLOT1, FSCN1, GNAI2, GNAQ, HNRNPU, HSP90AA1, HSPA8, HSPD1, ICAM2,
, FLNA, G6PD, GNAI2, GP1BA, GP1BB, ITGB1, ITGB3, LTF, PCYOX1, PNPLA8, RHOA, SLC2A1, SLC2A3, SRI,
FLNA, FLOT1, FSCN1, G3BP1, HNRNPA2B1, HSP90AA1, HSPA1B, ITGA6, LRP1, MSN, PFN1, PLEK, PTPRJ,

CCT2, CCT4, CCT6A, CCT7, CCT8, CD226, CD36, CD47, CSDE1, CTSD, CYBB, DAZAP1, DHX9, EEF1A1, EEF2,
A, CCT7, CCT8, CD151, CD36, CD47, CDC42, CLIC1, CTSD, CYBB, DHX9, FCER1G, FCN1, FLNA, FLOT1, G6PD
CD36, CD47, CD53, CDC42, CFL1, EIF5A, ENO1, F11R, FLNA, FLOT1, FSCN1, G3BP1, G6PD, HNRNPA2B1, H

TA, CSDE1, CTSD, DHX9, DSG1, F11R, FERMT3, FLNA, FLOT1, FSCN1, G3BP1, H2BC21, HNRNPU, HSP90AA
PDH, GNAI2, GNAQ, GSTP1, HINT1, HMBS, HSP90AA1, HSPA1B, ILK, ITGB3, KANSL3, KRT1, KRT10, KRT2,
PDH, GNAI2, GNAQ, GSTP1, HINT1, HMBS, HSP90AA1, HSPA1B, ILK, ITGB3, KANSL3, KRT1, KRT10, KRT2,
ATP5F1B, B2M, BCAP31, CALM3, CCL5, CCT2, CCT4, CCT6A, CCT7, CCT8, CD36, CD59, CD9, CFL1, CLIC1,
NPP5D, ITGB1, ITGB3, JUP, KRT1, KRT17, LTF, PGK1, PKM, PRDX2, RHOA, RTN4, SOD1, SRGN, THBS1, VCL
TP1, HSP90AA1, HSPA1B, HSPA9, HSPD1, INPP5D, ITGA6, ITGB1, LTF, NPM1, PPIA, PRDX2, RHOA, RPL11
TP1, HSP90AA1, HSPA1B, HSPA9, HSPD1, INPP5D, ITGA6, ITGB1, LTF, NPM1, PPIA, PRDX2, RHOA, RPL11
B, DCD, DDX60L, DHX9, EIF5A, ENDOD1, ENO1, F11R, FCER1G, FCN1, FLNA, G3BP1, G6PD, GAPDH, GNAI
3, ATP5F1B, CALM3, CANX, CCT2, CCT4, CCT6A, CCT7, CCT8, CDC42, CPN1, CYB5R3, CYBB, DCD, DDX60L,

HSPA9, HSPD1, INPP5D, ITGB1, LTF, MYH9, PLEK, PRDX2, PTPRJ, RHOA, SELPLG, SOD1, SPN, TFRC, XRCC
A8, ITGB1, MYH9, NASP, NPM1, RCC2, RHOA, RPS15A, SPTBN1, TTLL12, TUBA1C, TUBA4A, TUBB, TUBB1,
AZAP1, DHX9, EEF1A1, EEF2, EIF5A, ENO1, F11R, FCER1G, FCN1, FLNA, FLOT1, G3BP1, GAPDH, GP1BA, G
GNAQ, HNRNPA2B1, HNRNPU, HSP90AA1, HSPD1, ITGA6, ITGB1, ITGB3, LTF, NPM1, PLEK, PPIA, RCC2,
RMT3, GSTP1, HRNR, HSP90AA1, HSPA8, JUP, LGALS3BP, LTF, LYZ, MMRN1, PKM, PPIA, PRDX6, PSMA5,
RMT3, GSTP1, HRNR, HSP90AA1, HSPA8, JUP, LGALS3BP, LTF, LYZ, MMRN1, PKM, PPIA, PRDX6, PSMA5,
RMT3, GSTP1, HRNR, HSP90AA1, HSPA8, JUP, LGALS3BP, LTF, LYZ, MMRN1, PKM, PPIA, PRDX6, PSMA5,
AI2, GSTP1, HSP90AA1, HSPA1B, HSPA9, HSPD1, INPP5D, ITGA6, ITGB1, ITGB3, LRP1, LTF, NPM1, PPIA, P
CYB5R3, CYBB, EIF5A, HLA-A, HLA-B, HSPA1B, LRBA, PKM, PNPLA8, PTGS1, QSOX1, RAB10, RAB1B, RAC2

EIF5A, ENO1, FLNA, FLOT1, GAPDH, GSTP1, HINT1, HNRNPA2B1, HNRNPC, HNRNPU, HSP90AA1, HSPA1
CD47, CD9, CFL1, CYBB, DHX9, DSP, ENO1, F11R, FCER1G, FCN1, FLNA, FLOT1, G3BP1, G6PD, GAPDH, GN
1, CD226, CD36, CD47, CD59, CD9, CDC42, CLIC1, CYBB, DCD, DDX60L, DHX9, DSP, ENDOD1, ENO1, F11I
PA1B, HSPA8, MSN, MYADM, NPM1, PFN1, PLEK, PTPRJ, RAB1B, RAC2, RAP1B, RCC2, RHOA, SPTBN1, TF
, DHX9, F11R, FLOT1, GNAI2, GNAQ, HNRNPA2B1, HNRNPU, HSP90AA1, HSPA1B, HSPD1, ITGA6, ITGB1, I

CASP14, CAVIN2, CCT2, CCT4, CCT6A, CCT7, CCT8, CD151, CDC42, CFL1, CLTA, CSDE1, CYB5R3, DAZAP1,
CANX, CASP14, CCL5, CD36, CDC42, CPN1, CSDE1, CTSD, DCD, DHX9, DSP, EEF1A1, EEF2, EIF5A, ENO1, F
P, CCL5, CCT2, CCT4, CCT6A, CCT7, CCT8, CD151, CD226, CD36, CD47, CD53, CD59, CD99, CDC42, CFL1, C

P5, ATIC, ATP2A3, ATP5F1B, B2M, BCAP31, C16orf54, CALM3, CANX, CASP14, CAVIN2, CD151, CD226, C

IF5A, HLA-A, HLA-B, LRBA, PNPLA8, PTGS1, RAB10, RAB1B, RAC2, RHOA, RTN3, RTN4, SACM1L, SRI, TKT,
IF5A, HLA-A, HLA-B, LRBA, PNPLA8, PTGS1, RAB10, RAB1B, RAC2, RHOA, RTN3, RTN4, SACM1L, SRI, TKT,
, CAMP, CANX, CASP14, CCL5, CCT2, CCT4, CCT6A, CCT7, CCT8, CD226, CD36, CD47, CDC42, CPN1, CSDE
X9, EEF1A1, EEF2, EIF5A, ENO1, FLNA, FLOT1, G6PD, GAPDH, GNAI2, GNAQ, GSTP1, HNRNPU, HSP90AA1,

EIF5A, ENDOD1, ENO1, FCER1G, FCN1, G3BP1, GAPDH, GSDMA, GSTP1, H2BC21, HLA-A, HLA-B, HSP90A
D, DHX9, EEF1A1, F11R, GAPDH, GNAI2, GNAQ, GSTP1, HNRNPA2B1, HNRNPU, HSP90AA1, HSPD1, ITGA
EIF5A, ENDOD1, ENO1, FCER1G, FCN1, G3BP1, GAPDH, GSDMA, GSTP1, H2BC21, HLA-A, HLA-B, HSP90A
FLNA, FLOT1, FSCN1, GNAI2, GNAQ, HNRNPU, HSP90AA1, HSPA8, HSPD1, ICAM2, ILK, ITGB1, ITGB3, MSN
ENO1, FCN1, GAPDH, HINT1, HSPA1B, HSPD1, LRP1, LTF, MYH9, PCYOX1, PGK1, PSMA5, PSMA6, RHOA,

, ARL6IP5, ARPC5, ATIC, ATP2A3, ATP5F1B, B2M, BCAP31, CALM3, CAMP, CANX, CASP14, CAVIN2, CCT2,
H, H2BC21, HLA-A, HLA-B, HSP90AA1, KRT1, KRT16, LBP, LTF, MATR3, PRDX1, S100A8, S100A9, SYNCRIP,
IF5A, ENO1, HLA-A, HLA-B, LRBA, PNPLA8, PTGS1, RAB10, RAB1B, RAC2, RHOA, RTN3, RTN4, SACM1L, SI

7, CCT8, CD36, CSDE1, CTSD, DAZAP1, DHX9, EEF2, EIF5A, ENO1, FLOT1, HNRNPA2B1, HNRNPU, HSP90A
OD1, ENO1, FLNA, FLOT1, G3BP1, HINT1, HNRNPA2B1, HNRNPA3, HNRNPC, HNRNPF, HNRNPH1, HNRN
7, CSDE1, CYB5R3, DHX9, EEF1A1, EEF2, EIF5A, ENO1, FLNA, FLOT1, GAPDH, HINT1, HMBS, HNRNPA2B1,

T4, CCT6A, CCT7, CCT8, CD151, CD36, CD47, CD53, CD9, CDC42, CFL1, CLTA, CSDE1, CTSD, DHX9, DSG1,
NO1, HNRNPU, HSP90AA1, ILK, ITGA6, JUP, KANSL3, KRT17, NCL, NPM1, PCBP1, PKM, RHOA, SYNCRIP, T
NO1, HNRNPU, HSP90AA1, ILK, ITGA6, JUP, KANSL3, KRT17, NCL, NPM1, PCBP1, PKM, RHOA, SYNCRIP, T
-A, HNRNPU, HSPA1B, ILK, INPP5D, ITGB1, ITGB3, JUP, KRT6A, LTF, NPM1, PTGS1, PTPRJ, RAC2, RHOA, R
RNPA2B1, HNRNPA3, HNRNPC, HNRNPF, HNRNPH1, HNRNPH3, HNRNPU, HSP90AA1, HSPA1B, HSPA8,
DHX9, EEF1A1, EEF2, FCN1, G3BP1, GNAI2, GNAQ, HNRNPU, HSP90AA1, HSPA1B, HSPA8, HSPA9, HSPD

TP5F1B, B2M, BCAP31, CALM3, CAMP, CANX, CASP14, CCL5, CCT2, CCT4, CCT6A, CCT7, CCT8, CD226, CD
HNRNPA2B1, HNRNPC, HNRNPU, HSPA8, ITGB3, LRP1, MYADM, NCL, NPM1, PLEK, PPIA, PTBP1, PTPRJ,
1B, ILK, ITGB3, KANSL3, LRP1, LTF, MYADM, NCL, NPM1, PECAM1, PPIA, PTBP1, PTPRJ, RHOA, RPL11, R

NPU, HSPA1B, ITGB1, ITGB3, KRT1, KRT14, KRT15, KRT16, KRT17, KRT2, KRT5, KRT6A, KRT6B, KRT78, KRT
EIF5A, ENDOD1, ENO1, FCER1G, FCN1, G3BP1, GAPDH, GSDMA, GSTP1, H2BC21, HLA-A, HLA-B, HSP90A
T4, CCT6A, CCT7, CCT8, CD151, CD36, CD47, CD53, CD9, CDC42, CFL1, CLTA, CSDE1, CTSD, DHX9, DSG1,
ANXA7, ARL6IP5, ARPC5, ATIC, ATP2A3, ATP5F1B, B2M, BCAP31, C16orf54, CALM3, CAMP, CANX, CASP1
ANXA7, ARL6IP5, ARPC5, ATIC, ATP2A3, ATP5F1B, B2M, BCAP31, C16orf54, CALM3, CAMP, CANX, CASP1

2, EIF5A, ENO1, FLOT1, HNRNPU, HSP90AA1, HSPA1B, HSPD1, ILK, ITGB3, KANSL3, KRT17, LRP1, LTF, MS
ILA-B, LRBA, LRP1, M6PR, PTGS1, QSOX1, RAB10, RAB1B, RAB27B, RAB33B, RTN3, SACM1L, SLC2A1, SRG
EEF1A1, EEF2, ENO1, FLNA, FLOT1, GSTP1, HINT1, HNRNPU, HSP90AA1, HSPA1B, HSPA8, HSPA9, HSPD1

J, HSP90AA1, ILK, ITGA6, JUP, KANSL3, KRT17, NCL, NPM1, PCBP1, PKM, RHOA, SYNCRIP, TCP1, THBS1, I
6, KRT17, KRT2, KRT5, KRT6A, KRT6B, KRT78, KRT9, MSN, MYADM, PECAM1, PGK1, RAB10, RAP1B, RHO
RNPA2B1, HNRNPU, HSP90AA1, HSPA8, ILK, ITGA6, JUP, KANSL3, NCL, NPM1, PCBP1, PRDX6, SYNCRIP, T

ARL6IP5, ARPC5, ATIC, ATP2A3, ATP5F1B, B2M, BCAP31, C16orf54, CALM3, CANX, CASP14, CAVIN2, CCL

B5R3, DAZAP1, DHX9, EIF5A, ENDOD1, ENO1, FLNA, FLOT1, G3BP1, G6PD, GAPDH, HINT1, HMBS, HNRNI
CYBB, DHX9, EEF1A1, EIF5A, ENO1, FCER1G, FLNA, FLOT1, G6PD, GAPDH, GNAI2, GNAQ, GSR, GSTP1, HI

., FLNA, GALE, GAPDH, HINT1, HNRNPC, HNRNPU, HSP90AA1, HSPA1B, HSPA8, LDHA, LRP1, MSN, NPM1
PDH, GNAI2, HNRNPU, HSPA1B, MYH16, MYH9, NPM1, PATJ, RCC2, TCP1, TTLL12, TUBA1C, TUBA4A, TU
, CCT7, CCT8, CD226, CD36, CSDE1, CTSD, CYBB, DAZAP1, DHX9, EEF2, EIF5A, ENO1, FCER1G, FCN1, FLO
ZAP1, DHX9, EIF5A, ENDOD1, ENO1, FLNA, FLOT1, G3BP1, G6PD, GAPDH, HINT1, HMBS, HNRNPA2B1, H
, CCT7, CCT8, CD226, CD36, CDC42, CSDE1, CTSD, CYBB, DAZAP1, DHX9, EEF2, EIF5A, ENO1, FCER1G, FC
ZAP1, DHX9, EIF5A, ENDOD1, ENO1, FLNA, FLOT1, G3BP1, G6PD, GAPDH, HINT1, HNRNPA2B1, HNRNPA
CN1, FLOT1, G3BP1, GAPDH, H2BC21, HLA-A, HLA-B, HSP90AA1, HSPA1B, HSPD1, INPP5D, KRT1, KRT16,
, G3BP1, HNRNPA2B1, HNRNPC, HNRNPU, HSPA1B, ITGB3, LRP1, MSN, MYADM, NPM1, PATJ, PFN1, PLE

6, CDC42, CSDE1, DAZAP1, DHX9, EEF2, EIF5A, ENO1, FLOT1, GNAI2, GSTP1, HNRNPA2B1, HNRNPU, HSP

ANXA7, ARL6IP5, ARPC5, ATIC, ATP2A3, ATP5F1B, B2M, BCAP31, C16orf54, CALM3, CAMP, CANX, CASP1
'ADM, NCL, PLEK, PPIA, PRDX2, PTPRJ, RAP1B, RHOA, THBS1, TTLL12, UBQLN1, VDAC2, YWHAB
'ADM, NCL, PLEK, PPIA, PRDX2, PTPRJ, RAP1B, RHOA, THBS1, TTLL12, UBQLN1, VDAC2, YWHAB
P1, HRNR, ITGB3, KRT1, KRT16, LTF, PNPLA8, RTN4, S100A8, S100A9, SOD1, SRI, TFRC, YWHAE
TGB3, LTF, NPM1, PPIA, PRDX2, RHOA, RPS3A, SOD1, TFRC, THBS1, TMEM109, VDAC2, YWHAZ
T7, CCT8, CDC42, CFL1, CLTA, DHX9, DSP, EEF1A1, FERMT3, FLNA, FLOT1, FSCN1, G6PD, GAPDH, GNAI2,
I2, GNAQ, GSTP1, HNRNPA2B1, HNRNPC, HNRNPU, HSPA1B, HSPA8, INPP5D, ITGB3, LBP, LRP1, LTF, MY
, HNRNPU, ILK, JUP, KRT1, KRT10, KRT14, KRT15, KRT16, KRT17, KRT2, KRT5, KRT6A, KRT6B, KRT78, KRT
, HNRNPU, ILK, JUP, KRT1, KRT10, KRT14, KRT15, KRT16, KRT17, KRT2, KRT5, KRT6A, KRT6B, KRT78, KRT
EEF1A1, EEF2, G3BP1, GNAI2, GNAQ, HNRNPU, HSP90AA1, HSPA1B, HSPA8, HSPA9, HSPD1, ILK, MYH9,
DDX60L, DHX9, EEF1A1, EEF2, G3BP1, G6PD, GAPDH, GNAI2, GNAQ, GSR, HINT1, HNRNPU, HSP90AA1, H
ZAP1, DHX9, EIF5A, ENDOD1, ENO1, FLNA, FLOT1, G3BP1, G6PD, GAPDH, HINT1, HMBS, HNRNPA2B1, H
50L, DHX9, DSP, EEF1A1, EEF2, EIF5A, ENDOD1, ENO1, FLNA, FSCN1, G3BP1, H2AC20, H2BC21, HLA-A, HI
PA1B, INPP5D, ITGA6, ITGB1, KRT1, LRP1, LTF, MMRN1, MYADM, NCL, PLEK, PPIA, PRDX2, PTPRJ, RHOA,
I2, GNAQ, GSTP1, HNRNPA2B1, HNRNPC, HNRNPU, HSPA1B, HSPA8, INPP5D, ITGB3, LBP, LRP1, LTF, MY
BCAP31, CALM3, CAMP, CANX, CASP14, CCL5, CD36, CDC42, CPN1, CSDE1, CTSD, DCD, DHX9, DSP, EEF1
P, EEF2, FCER1G, FLG2, FLNA, FLOT1, G6PD, GNG5, GP1BA, GP1BB, HLA-B, HNRNPU, HRNR, HSPA1B, HS

;P90AA1, HSPA1B, HSPA8, LRP1, MSN, NPM1, PCYOX1, PSMA5, PSMA6, RPL11, SYNCRIP, TNKS1BP1, UBI
HSP90AA1, ITGB3, LTF, NPM1, PPIA, PTPRJ, RHOA, RPL11, RPS15, SOD1, TCP1, THBS1, UBE2L3, XRCC5, Y

O1, FLOT1, G6PD, GAPDH, GNAI2, GNAQ, GSTP1, HINT1, HNRNPU, HSP90AA1, HSPA1B, HSPA8, ILK, INPF
TP5F1B, B2M, BCAP31, CALM3, CAMP, CANX, CASP14, CCL5, CCT2, CCT4, CCT6A, CCT7, CCT8, CD226, CD
A, GNAI2, H2BC21, HINT1, HNRNPA2B1, HNRNPA3, HNRNPC, HNRNPF, HNRNPH1, HNRNPH3, HNRNPU,
, CYBB, DDX60L, DHX9, EEF1A1, EEF2, G3BP1, G6PD, GAPDH, GNAI2, GNAQ, GSR, HINT1, HNRNPU, HSP9
CYB5R3, CYBB, DDX60L, DHX9, EEF1A1, EEF2, FCN1, G3BP1, GNAI2, GNAQ, GSDMA, GSR, GSTP1, HNRNPU

HNRNPA2B1, HNRNPC, HNRNPU, HSPA1B, HSPA8, ITGB3, LRP1, LTF, MYADM, NCL, NPM1, PPIA, PTBP1,
ANXA7, ARL6IP5, ARPC5, ATIC, ATP2A3, ATP5F1B, B2M, BCAP31, CALM3, CAMP, CANX, CASP14, CAVIN2,
CAVIN2, CCT2, CCT4, CCT8, CFL1, CTSD, CYB5R3, DAZAP1, DHX9, EEF1A1, EEF2, FCN1, FERMT3, FLG2, FI
CAVIN2, CCT2, CCT4, CCT8, CFL1, CTSD, CYB5R3, DAZAP1, DHX9, EEF1A1, EEF2, FCN1, FERMT3, FLG2, FI
CAVIN2, CCT2, CCT4, CCT8, CFL1, CTSD, CYB5R3, DAZAP1, DHX9, EEF1A1, EEF2, FCN1, FERMT3, FLG2, FI
O1, FLOT1, G6PD, GAPDH, GNAI2, GNAQ, GSTP1, HINT1, HNRNPU, HSP90AA1, HSPA1B, HSPA8, ILK, INPF
HX9, DSP, EEF1A1, EEF2, EIF5A, ENO1, FLNA, FSCN1, G3BP1, HLA-A, HNRNPA2B1, HNRNPA3, HNRNPC, HI

TP1, HLA-A, HLA-B, HSP90AA1, HSPA1B, HSPD1, INPP5D, LBP, LTF, PTPRJ, SOD1, SPN, SPTBN1, SRGN, TH
TP1, HLA-A, HLA-B, HSP90AA1, HSPA1B, HSPD1, INPP5D, LBP, LTF, PTPRJ, SOD1, SPN, SPTBN1, SRGN, TH
HNRNPU, HSP90AA1, HSPA1B, HSPA8, HSPA9, HSPD1, ILK, MYH9, PGK1, PKM, PNPLA8, TCP1, TTLL12, UI
HNRNPU, HSP90AA1, HSPA1B, HSPA8, HSPA9, HSPD1, ILK, MYH9, PGK1, PKM, PNPLA8, TCP1, TTLL12, UI
BCAP31, CALM3, CAMP, CANX, CASP14, CCL5, CCT2, CCT4, CCT6A, CCT7, CCT8, CD36, CD47, CDC42, CPN

6, CD47, CSDE1, CYB5R3, CYBB, DHX9, EEF1A1, EEF2, EIF5A, ENO1, FLNA, FLOT1, GAPDH, GSTP1, HINT1,
6, CSDE1, CYB5R3, DHX9, EEF1A1, EEF2, EIF5A, ENO1, FLNA, FLOT1, G6PD, GAPDH, GSTP1, HINT1, HMBS
T4, CCT6A, CCT7, CCT8, CDC42, CPN1, CTSD, CYB5R3, CYBB, DCD, DDX60L, DHX9, EEF1A1, EEF2, ENDOD
7, CCT8, CD36, CD9, CDC42, CLIC1, CLTA, CSDE1, CYB5R3, CYBB, DAZAP1, DHX9, EEF1A1, EEF2, EIF5A, EN
6PD, GSR, GSTP1, HSP90AA1, HSPA1B, HSPA8, HSPA9, HSPD1, NPM1, PGK1, PNPLA8, PPIA, PRDX1, PRD
B2M, BCAP31, CALM3, CAMP, CANX, CASP14, CCL5, CCT2, CCT4, CCT6A, CCT7, CCT8, CD36, CDC42, CPN

DDX60L, DHX9, EEF1A1, EEF2, G3BP1, G6PD, GAPDH, GNAI2, GNAQ, GSR, HINT1, HNRNPU, HSP90AA1, H
EEF1A1, EEF2, G3BP1, GNAI2, GNAQ, HNRNPU, HSP90AA1, HSPA1B, HSPA8, HSPA9, HSPD1, ILK, MYH9, I
EEF1A1, EEF2, G3BP1, GNAI2, GNAQ, HNRNPU, HSP90AA1, HSPA1B, HSPA8, HSPA9, HSPD1, ILK, MYH9, I

32M, BCAP31, CALM3, CAMP, CANX, CCL5, CCT2, CCT4, CCT6A, CCT7, CCT8, CD36, CD47, CDC42, CPN1, C

HX9, DSP, ENO1, FLNA, G3BP1, HNRNPU, ILK, JUP, KRT1, KRT10, KRT14, KRT15, KRT16, KRT17, KRT2, KR
2B1, HNRNPA3, HNRNPC, HNRNPF, HNRNPH1, HNRNPU, KANSL3, LDHA, LDHB, PSMA5, PSMA6, RAC2, S
HNRNPU, HSP90AA1, HSPA1B, HSPA8, HSPA9, HSPD1, ILK, MYH9, PFN1, PGK1, PKM, PNPLA8, TCP1, TTL
, ARL6IP5, ARPC5, ATIC, ATP2A3, ATP5F1B, B2M, BCAP31, CALM3, CAMP, CANX, CASP14, CAVIN2, CCT2,

HSPA1B, INPP5D, LBP, LRP1, LTF, MYADM, NCL, NPM1, PTBP1, SRGN, SYNCRIP, THBS1, TNKS1BP1, VIM

, FLOT1, GP1BA, HLA-A, HLA-B, HSP90AA1, HSPA1B, HSPA9, HSPD1, INPP5D, ITGB3, KRT1, LBP, LTF, MA
EEF1A1, EEF2, G3BP1, GNAI2, GNAQ, HNRNPU, HSP90AA1, HSPA1B, HSPA8, HSPA9, HSPD1, ILK, MYH9,

T6A, CCT7, CCT8, CDC42, CSDE1, CYB5R3, CYBB, DAZAP1, DCD, DDX60L, DHX9, DSP, EEF1A1, EEF2, EIF5A
5, CD47, CSDE1, CYB5R3, CYBB, DHX9, EEF1A1, EEF2, EIF5A, ENO1, FLNA, FLOT1, G6PD, GAPDH, GSTP1,
HINT1, HSPA1B, HSPA8, LDHA, LDHB, NANS, OSBPL2, PGK1, PKM, PLEK, PNPLA8, PTGS1, RHOA, SLC2A1,

T6A, CCT7, CCT8, CDC42, CSDE1, CYB5R3, CYBB, DAZAP1, DCD, DDX60L, DHX9, DSP, EEF1A1, EEF2, EIF5A

CPN1, CTSD, DCD, DDX60L, DHX9, EEF1A1, EEF2, ENDOD1, G3BP1, GNAI2, GNAQ, GNG5, HINT1, HSP90A

K, CASP14, CCT2, CCT4, CCT6A, CCT7, CCT8, CDC42, CFL1, CLTA, CSDE1, CYB5R3, DHX9, DSP, EEF1A1, EN

K, CASP14, CCT2, CCT4, CCT6A, CCT7, CCT8, CDC42, CFL1, CLTA, CSDE1, CYB5R3, DHX9, DSP, EEF1A1, EN

.B, HSPA8, ITGB1, KRT1, KRT14, KRT15, KRT16, KRT17, KRT2, KRT5, KRT6A, KRT6B, KRT78, KRT9, MYADM

PA1B, ITGB3, LRP1, LTF, MYADM, NCL, NPM1, PPIA, PTPRJ, RPL11, RPS15, RTN3, RTN4, SYNCRIP, THBS1,

PU, HSPA1B, ILK, NCL, PF4V1, PTPRJ, RHOA, RPLP0, SELPLG, SYNCRIP, TFRC, THBS1, TTLL12, VIM, XRCC5

'6A, CCT7, CCT8, CD36, CD47, CPN1, CSDE1, CYB5R3, DAZAP1, DHX9, EEF1A1, EEF2, EIF5A, ENDOD1, EN(
'P1, HNRNPU, HSPA1B, ILK, NCL, PF4V1, PTPRJ, RHOA, RPLP0, SELPLG, SYNCRIP, TFRC, THBS1, TTLL12, VI
ANXA7, ARL6IP5, ARPC5, ATIC, ATP2A3, ATP5F1B, B2M, BCAP31, CALM3, CAMP, CANX, CASP14, CAVIN2,
5, ARPC5, ATP2A3, ATP5F1B, B2M, BCAP31, CALM3, CAMP, CANX, CASP14, CAVIN2, CCT2, CCT4, CCT6A,

-A, HLA-B, HSP90AA1, HSPD1, INPP5D, ITGB3, KRT1, LBP, MATR3, PTPRJ, RAC2, RHOA, SPN, TFRC, THBS
)0AA1, HSPA1B, HSPD1, INPP5D, KRT1, LBP, LTF, MATR3, PTPRJ, RAC2, RTN4, SPN, TFRC, TTLL12, UBQLN

CD47, CDC42, CFL1, CSDE1, CTSD, DHX9, DSP, F11R, FLNA, FSCN1, G3BP1, GAPDH, HNRNPA2B1, HNRNP(

H2BC21, HSP90AA1, HSPA1B, HSPA8, HSPD1, KRT1, KRT10, KRT5, MSN, MYADM, NASP, NPM1, OSBPL2

GNAI2, GNAQ, GNG5, HSP90AA1, HSPA1B, HSPA8, HSPA9, HSPD1, RAB10, RAB1B, RAB27B, RAB33B, RA(

GNAI2, GNAQ, GNG5, HSP90AA1, HSPA1B, HSPA8, HSPA9, HSPD1, RAB10, RAB1B, RAB27B, RAB33B, RAC
GNAI2, GNAQ, GNG5, HSP90AA1, HSPA1B, HSPA8, HSPA9, HSPD1, RAB10, RAB1B, RAB27B, RAB33B, RAC
GNAI2, GNAQ, GNG5, HSP90AA1, HSPA1B, HSPA8, HSPA9, HSPD1, RAB10, RAB1B, RAB27B, RAB33B, RAC
5, ARPC5, ATP2A3, ATP5F1B, B2M, BCAP31, CALM3, CAMP, CANX, CASP14, CAVIN2, CCT2, CCT4, CCT8,
, KRT16, KRT17, KRT2, KRT5, KRT6A, KRT6B, KRT78, KRT9, MATR3, MMRN1, MSN, MYL6, RPL11, RPL8,

, HSP90AA1, HSPA8, HSPD1, JUP, M6PR, MSN, NPM1, RAB10, RAB33B, RAC2, RCC2, RPL11, RTN4, SRGN

, KRT14, KRT15, KRT16, KRT17, KRT2, KRT5, KRT6A, KRT6B, KRT78, KRT9, RAC2, RCC2, TCP1, TPM4, TUB
HNRNPH1, HNRNPH3, HNRNPU, HSPA1B, HSPA8, NCL, NPM1, PCBP1, PSMA6, RPL11, RPL8, RPLP0, RPS
MT3, FSCN1, H2BC21, HSP90AA1, HSPA1B, HSPA8, HSPD1, KRT1, KRT10, KRT5, MSN, MYADM, NASP, NP

A1, EEF2, EIF5A, GAPDH, HNRNPA2B1, HNRNPC, HNRNPU, HSP90AA1, HSPA1B, HSPD1, KANSL3, KRT17,

1G, FCN1, FLOT1, G3BP1, GAPDH, HLA-A, HNRNPC, HNRNPU, HSP90AA1, HSPA1B, HSPA8, HSPD1, ITGB
H, GSR, GSTP1, HNRNPU, KRT17, NCL, NPM1, PKM, RHOA, RPL11, RPL8, RPLP0, RPS15, RPS15A, RPS23,
H, GSR, GSTP1, HNRNPU, KRT17, NCL, NPM1, PKM, RHOA, RPL11, RPL8, RPLP0, RPS15, RPS15A, RPS23,

HNRNPH3, HNRNPU, HSPA1B, HSPA8, NCL, NPM1, PRDX6, PTBP1, SF3A3, SYNCRIP, TNKS1BP1, VIM, WBP
SP14, CAVIN2, CCT4, CCT8, CFL1, CLIC1, CYBB, DAZAP1, DHX9, DSP, EEF1A1, EEF2, EIF5A, ENO1, FLG2, FI
HOA, RPL11, RPL8, RPLP0, RPS15, RPS15A, RPS23, RPS3A, RPSA, SYNCRIP, THBS1, TNKS1BP1, VIM
HOA, RPL11, RPL8, RPLP0, RPS15, RPS15A, RPS23, RPS3A, RPSA, SYNCRIP, THBS1, TNKS1BP1, VIM
HOA, RPL11, RPL8, RPLP0, RPS15, RPS15A, RPS23, RPS3A, RPSA, SYNCRIP, THBS1, TNKS1BP1, VIM
HOA, RPL11, RPL8, RPLP0, RPS15, RPS15A, RPS23, RPS3A, RPSA, SYNCRIP, THBS1, TNKS1BP1, VIM
HMBS, HNRNPU, KRT17, NCL, NPM1, PKM, RHOA, RPL11, RPL8, RPLP0, RPS15, RPS15A, RPS23, RPS3A, F

M2, ITGA2B, ITGA6, ITGB1, ITGB3, LBP, LRP1, MSN, MYH9, PF4V1, PPIA, PTPRJ, S100A8, S100A9, SELPLG,

BP, LRP1, MSN, MYADM, MYH9, PECAM1, PF4V1, PFN1, PPIA, PTPRJ, RAC2, RCC2, RHOA, RTN4, S100A8,

RAC2, RAP1B, RCC2, RHOA, RTN4, S100A8, S100A9, SOD1, VCL, VIM, XRCC5, YWHAH, YWHAZ

PPIA, RAB10, RAB1B, RAB27B, RAB33B, RAC2, RPL11, S100A8, SPTBN1, SRI, STOM, TCP1, TFRC, UBE2L3

ITGA6, ITGB1, ITGB3, JUP, LBP, LRP1, LTF, MYADM, NCL, NPM1, PLEK, PPIA, PRDX1, PRDX2, PTBP1, PTPR
, ITGA6, ITGB1, ITGB3, JUP, LRP1, MSN, MYADM, MYH9, NPM1, PATJ, PECAM1, PFN1, PPIA, PTPRJ, RAB1
42, CFL1, CLIC1, CPN1, CTSD, DCD, DSC1, DSP, EEF1A1, EEF2, ENDOD1, ENO1, F11R, FCN1, FERMT3, FLN

NPM1, OSBPL2, PLEK, PNPLA8, PPIA, RAB10, RAB1B, RAB27B, RAB33B, RAC2, RPL11, S100A8, SLC2A1, S

P1, MSN, MYADM, MYH9, PECAM1, PF4V1, PFN1, PPIA, PTPRJ, RAC2, RCC2, RHOA, RTN4, S100A8, S100/
M, MYH9, PECAM1, PLEK, PPIA, PRDX2, RAB10, RAC2, RHOA, S100A8, S100A9, SELPLG, SPN, TFRC, TLN1,

I2, ILK, ITGA2B, ITGA6, ITGB1, ITGB3, JUP, LGALS3BP, MMRN1, MSN, MYADM, MYH9, PECAM1, PLEK, PP

ERMT3, FLNA, FLOT1, G3BP1, GAPDH, GNAI2, GNAQ, GNG5, GP1BA, GP1BB, GSTP1, HINT1, HLA-A, HSP9

B3, JUP, LBP, LRP1, LTF, MYH9, NCL, PECAM1, PF4V1, PLEK, PRDX2, PTPRJ, RCC2, RHOA, RTN4, SPN, SPTI
MYH9, PCBP1, PFN1, PKM, PPIA, PRDX1, PRDX6, PTPRJ, RAB10, RPSA, RTN4, SPTBN1, TAGLN2, THBS1, TI
., MSN, MYH9, PF4V1, PLEK, PPIA, PRDX1, PRDX2, PTPRJ, RAC2, RHOA, SELPLG, SOD1, SPN, TFRC, THBS1,
1, G3BP1, GAPDH, GNAI2, GNAQ, GNG5, GP1BA, GP1BB, GSTP1, HINT1, HLA-A, HSP90AA1, HSPA1B, HSP

1B, ILK, INPP5D, ITGA6, ITGB1, ITGB3, JUP, LBP, LRP1, LTF, MYADM, NCL, NPM1, PLEK, PPIA, PRDX1, PRI
CD9, CDC42, CFL1, CLIC1, CPN1, CTSD, CYB5R3, DCD, DSC1, DSP, EEF1A1, EEF2, ENDOD1, ENO1, F11R, FC

GB3, JUP, KRT1, KRT10, KRT16, KRT17, KRT6A, LRP1, LTF, MATR3, MSN, MYADM, MYH9, MYL12B, NCL, PI

FLNA, FLOT1, G3BP1, GAPDH, GNAI2, GNAQ, GNG5, GP1BA, GP1BB, GSTP1, HINT1, HLA-A, HSP90AA1, H
NAQ, HSPA1B, HSPA8, HSPA9, ILK, ITGA2B, ITGA6, ITGB1, ITGB3, JUP, LRP1, MSN, MYADM, MYH9, NPM
9, CD99, CDC42, CFL1, CLIC1, CLTA, CSDE1, CTSD, CYBB, DSC1, DSG1, DSP, EEF1A1, EEF2, ENO1, F11R, FC

MYH9, NPM1, OSBPL2, PLEK, RAB10, RAB1B, RAB27B, RAC2, RAP1B, RPL11, RPS15, SOD1, SRI, STOM, TU
DSC1, DSG1, DSP, EEF1A1, EEF2, FCER1G, FCN1, FERMT3, FLG2, FLOT1, GSTP1, HLA-A, HLA-B, HRNR, HS
DSC1, DSG1, DSP, EEF1A1, EEF2, FCER1G, FCN1, FERMT3, FLG2, FLOT1, GSTP1, HLA-A, HLA-B, HRNR, HS

, HNRNPU, HSP90AA1, HSPA8, HSPD1, ICAM2, ITGA2B, ITGA6, ITGB1, ITGB3, JUP, KRT14, LRP1, M6PR,
C1, CTSD, DCD, DSC1, DSP, EEF1A1, EEF2, ENDOD1, ENO1, F11R, FERMT3, FLNA, FLOT1, FSCN1, G6PD, G
C1, CTSD, DCD, DSC1, DSP, EEF1A1, EEF2, ENDOD1, ENO1, F11R, FERMT3, FLNA, FLOT1, FSCN1, G6PD, G
C1, CTSD, DCD, DSC1, DSP, EEF1A1, EEF2, ENDOD1, ENO1, F11R, FERMT3, FLNA, FLOT1, FSCN1, G6PD, G
C1, CTSD, DCD, DSC1, DSP, EEF1A1, EEF2, ENDOD1, ENO1, F11R, FERMT3, FLNA, FLOT1, FSCN1, G6PD, G

R, PECAM1, PTPRJ, RAB10, RAB27B, RAC2, RAP1B, RCC2, RHOA, SLC2A3, SRI, STOM, TFRC, TSPAN14
R, PECAM1, PTPRJ, RAB10, RAB27B, RAC2, RAP1B, RCC2, RHOA, SLC2A3, SRI, STOM, TFRC, TSPAN14

P1BA, GP1BB, HNRNPA2B1, HNRNPU, HSP90AA1, HSPA8, HSPA9, HSPD1, ITGB1, ITGB3, JUP, KRT5, LRBA

PA8, HSPA9, HSPD1, ITGB1, ITGB3, JUP, KRT5, LRBA, LRP1, M6PR, MSN, MYADM, MYH9, NASP, NPM1, P
PA8, HSPA9, HSPD1, ITGB1, ITGB3, JUP, KRT5, LRBA, LRP1, M6PR, MSN, MYADM, MYH9, NASP, NPM1, P

AB10, RAB1B, RAB27B, RAB33B, RAC2, RCC2, RPL11, SPTBN1, SRI, STOM, TCP1, TFRC, UBE2L3, YWHAB,

, GP1BA, GP1BB, H2BC21, HLA-A, HLA-B, HSP90AA1, HSPA1B, HSPA9, HSPD1, INPP5D, ITGA6, ITGB1, ITG
IRNPU, HSP90AA1, HSPA8, HSPA9, HSPD1, ITGB1, ITGB3, JUP, KRT5, LBP, LRBA, LRP1, M6PR, MSN, MYAI
ITGB1, ITGB3, JUP, LBP, LRP1, LTF, MYADM, NCL, NPM1, PLEK, PPIA, PRDX1, PRDX2, PTBP1, PTPRJ, RAP

G, FCN1, FERMT3, FLG2, GSTP1, HLA-B, HRNR, HSP90AA1, HSPA1B, HSPA8, HSPD1, ITGA2B, ITGB3, JUP,

)53, CD59, CD9, CDC42, CFL1, CLIC1, CLTA, CTSD, CYB5R3, CYBB, DCD, DSC1, DSG1, DSP, EEF1A1, EEF2, E

A, GP1BB, GSDMA, HLA-A, HLA-B, HSPA9, HSPD1, ICAM2, ITGA2B, ITGA6, ITGB1, ITGB3, LRBA, LRP1, M6

1T3, FLG2, GSTP1, HLA-B, HRNR, HSP90AA1, HSPA1B, HSPA8, HSPD1, ITGA2B, ITGB3, JUP, KRT1, LGALS3E

HA, LRBA, LRP1, MSN, NPM1, PCYOX1, PGK1, PKM, PNPLA8, PRDX1, PRDX2, PRDX6, PSMA5, PSMA6, QS
, GNAQ, HLA-A, HLA-B, HSPA8, HSPA9, ITGA2B, ITGB1, ITGB3, LRBA, LRP1, M6PR, PECAM1, PNPLA8, PTP
FN1, PPIA, RAB10, RAC2, RHOA, RPL8, RPLP0, RPS15, RPS3A, TLN1, TPM4, VCL, VIM, YWHAB, YWHAE, Y

, CCL5, CCT2, CCT4, CCT6A, CCT7, CCT8, CD151, CD226, CD36, CD47, CD53, CD59, CD9, CD99, CDC42, CF

:FL1, CLIC1, CLTA, CSDE1, CYBB, DSC1, DSG1, DSP, EEF1A1, EEF2, ENO1, F11R, FCER1G, FCN1, FLG2, FLN/
J, HSP90AA1, HSPA1B, HSPD1, ITGA6, ITGB1, ITGB3, JUP, LRP1, LTF, NPM1, PFN1, PLEK, PPIA, PRDX2, PS
, GP1BA, GP1BB, GSDMA, HLA-A, HLA-B, HSPA9, HSPD1, ICAM2, ITGA2B, ITGA6, ITGB1, ITGB3, LRBA, LRI

NCL, NPM1, PCBP1, PFN1, PPIA, PRDX2, PRDX6, PSMA6, PTBP1, RHOA, S100A8, S100A9, SRI, SYNCRIP, T
NPM1, PFN1, PPIA, RAB10, RAC2, RHOA, RPL8, RPLP0, RPS15, RPS3A, TLN1, TPM4, VCL, VIM, YWHAB, Y

PRDX2, PTBP1, PTPRJ, RAP1B, RHOA, RPL11, RPS15, RTN4, S100A8, S100A9, SOD1, TFRC, THBS1, UBQLN
NRNPU, HSP90AA1, HSPA1B, HSPA9, HSPD1, ILK, INPP5D, ITGA2B, ITGA6, ITGB1, ITGB3, JUP, KRT1, LRP1,

FERMT3, FLG2, FLNA, FLOT1, G6PD, GNG5, GP1BA, GP1BB, GSTP1, HLA-B, HNRNPH3, HNRNPU, HRNR, H
GB3, JUP, LDHA, LDHB, LYZ, MATR3, MYH9, NCL, NPM1, PECAM1, PLEK, PRDX1, PRDX6, SLC2A1, SOD1, S

CT2, CCT4, CCT6A, CCT7, CCT8, CD151, CD226, CD36, CD47, CD53, CD59, CD9, CD99, CDC42, CFL1, CLIC1,

-B, HNRNPU, HSP90AA1, HSPA1B, HSPA9, HSPD1, ILK, INPP5D, ITGA2B, ITGA6, ITGB1, ITGB3, JUP, KRT1,

NAQ, GP1BA, GSTP1, HINT1, HLA-A, HLA-B, HSP90AA1, HSPA1B, HSPD1, ILK, INPP5D, ITGA6, ITGB1, ITGE

PRDX1, PRDX2, PRDX6, PSMA5, PSMA6, QSOX1, RAB1B, RAB33B, RPL11, S100A8, S100A9, SYNCRIP, TNK
3, ILK, ITGA6, JUP, KANSL3, KRT17, LTF, NCL, NPM1, PCBP1, PFN1, PKM, PPIA, PRDX2, PSMA6, RHOA, S10
SPD1, ILK, INPP5D, ITGB1, ITGB3, JUP, KRT1, LBP, LTF, MATR3, PPIA, PRDX2, PTBP1, PTPRJ, RAC2, RAP1B

53, CD59, CD9, CD99, CDC42, CFL1, CLIC1, CSDE1, CTSD, CYBB, DAZAP1, DHX9, DSP, EEF1A1, EEF2, EIF5A,

P, EEF1A1, EIF5A, ENO1, FCER1G, FLOT1, GAPDH, GNAQ, HLA-A, HLA-B, HSPA8, HSPA9, HSPD1, ITGA2B, I

C42, CFL1, CLIC1, CPN1, CYBB, DCD, DDX60L, DHX9, DSG1, DSP, EEF1A1, EIF5A, ENDOD1, ENO1, F11R, FC
5A, KRT6B, KRT78, KRT9, LTF, MSN, MYADM, MYL6, PECAM1, PFN1, PGK1, RAB10, RAP1B, RHOA, RTN4, S
, JUP, KANSL3, LTF, NANS, NCL, NPM1, PCBP1, PFN1, PPIA, PRDX2, PSMA6, RHOA, S100A8, S100A9, SRI,
HX9, EEF1A1, EIF5A, ENO1, F11R, FCER1G, FCN1, FERMT3, FLNA, FLOT1, G3BP1, G6PD, GAPDH, GNAI2, G

LRP1, M6PR, MSN, MYH9, PECAM1, PLEK, PTPRJ, RAB10, RAB1B, RAB27B, RAB33B, RAC2, RAP1B, RTN3, S
ILK, ITGA6, JUP, KANSL3, KRT17, LTF, NCL, NPM1, PCBP1, PFN1, PKM, PLEK, PPIA, PRDX2, PSMA6, RHOA

, KRT17, KRT2, LTF, MSN, MYADM, MYH9, MYL12B, PGK1, PKM, PRDX2, PTBP1, RAC2, RCC2, RHOA, RTN4

AC2, RPL11, RPS15, SOD1, STOM, TUBA1C, TUBB, UBE2L3, VDAC2, YWHAB, YWHAE, YWHAG, YWHAH, Y

RT6A, LBP, LGALS3BP, LRP1, LTF, LYZ, MATR3, PF4V1, PRDX1, PRDX2, PSMA6, PTGS1, S100A8, S100A9, S
, ITGA6, JUP, KANSL3, LTF, NANS, NCL, NPM1, PCBP1, PFN1, PPIA, PRDX2, PSMA6, RHOA, S100A8, S100A
, ITGA6, JUP, KANSL3, LTF, NANS, NCL, NPM1, PCBP1, PFN1, PPIA, PRDX2, PSMA6, RHOA, S100A8, S100A

D226, CD36, CD47, CD53, CD59, CD9, CD99, CDC42, CFL1, CLIC1, CSDE1, CTSD, CYBB, DAZAP1, DHX9, DS
, CYBB, DHX9, F11R, FCER1G, FCN1, FLNA, FLOT1, G6PD, GNAI2, GP1BA, GP1BB, HNRNPA2B1, HNRNPU,
CD36, CD47, CD53, CD59, CD9, CD99, CDC42, CFL1, CLIC1, CSDE1, CTSD, CYBB, DAZAP1, DHX9, DSP, EEF1

l, CLIC1, CLTA, CTSD, CYBB, DHX9, F11R, FCER1G, FCN1, FLNA, FLOT1, G6PD, GNAI2, GP1BA, GP1BB, HNI
G6PD, GAPDH, GNAI2, GNAQ, GSTP1, HNRNPU, HSP90AA1, HSPA1B, HSPA8, HSPA9, HSPD1, ILK, ITGB1, I

SPA1B, HSPA8, ILK, ITGA6, JUP, KANSL3, LTF, NCL, NPM1, PCBP1, PFN1, PPIA, PRDX2, PRDX6, PSMA6, PT
A1B, HSPA8, ILK, ITGA6, JUP, KANSL3, LTF, NANS, NCL, NPM1, OSBPL2, PCBP1, PFN1, PPIA, PRDX2, PSM
APDH, GNAI2, GNAQ, GSTP1, HINT1, HNRNPA2B1, HNRNPC, HNRNPF, HNRNPH1, HNRNPH3, HNRNPU, H

AM1, PGK1, PKM, PPIA, PTPRJ, RHOA, SOD1, SPTBN1, TFRC, THBS1, TNKS1BP1, XRCC5, YWHAG, YWHAZ
l, ITGA2B, ITGA6, ITGB1, ITGB3, KRT10, LBP, LTF, MSN, PTPRJ, SPN, TFRC, THBS1, TLN1, TSPAN14

2, GNAQ, GSTP1, HINT1, HNRNPA2B1, HNRNPC, HNRNPF, HNRNPH1, HNRNPH3, HNRNPU, HSP90AA1, HS
1, EEF2, EIF5A, ENO1, F11R, FCER1G, FCN1, FLNA, FLOT1, G3BP1, G6PD, GAPDH, GNAI2, GNAQ, GSTP1, I
AZAP1, DHX9, EEF2, EIF5A, ENO1, F11R, FCN1, FERMT3, FLNA, FLOT1, FSCN1, G3BP1, G6PD, GAPDH, GN

HSPA8, ILK, ITGA6, JUP, KANSL3, KRT17, LTF, NCL, NPM1, PCBP1, PFN1, PKM, PLEK, PPIA, PRDX2, PSMA(
G, FLNA, FLOT1, G6PD, GAPDH, GNAI2, GNAQ, GSR, GSTP1, HNRNPU, HSP90AA1, HSPA1B, HSPA8, HSPA9
LTF, LYZ, NPM1, PECAM1, PNPLA8, PRDX1, PRDX2, PRDX6, RTN4, S100A8, S100A9, SLC2A1, SOD1, SRI, T
TGA6, ITGB1, LRP1, LTF, MATR3, MMRN1, MYL12B, MYL6, PKM, PTGS1, S100A8, S100A9, SF3A3, SOD1, S

X9, DSP, EEF2, F11R, FCER1G, FERMT3, FLG2, FLNA, FLOT1, G6PD, GNG5, GP1BA, GP1BB, GSTP1, HLA-B,

NRNPC, HNRNPH3, HNRNPU, HRNR, HSP90AA1, HSPA1B, HSPA9, ILK, INPP5D, ITGA6, ITGB1, ITGB3, KRT1
NRNPC, HNRNPH3, HNRNPU, HRNR, HSP90AA1, HSPA1B, HSPA9, ILK, INPP5D, ITGA6, ITGB1, ITGB3, KRT1
ANSL3, LTF, NCL, NPM1, PCBP1, PFN1, PPIA, PRDX2, PRDX6, PSMA6, PTBP1, RHOA, RPL11, RPS15, S100A8
SDE1, CYB5R3, CYBB, DAZAP1, DHX9, DSG1, DSP, EEF2, ENO1, F11R, FCER1G, FCN1, FERMT3, FLG2, FLNA

42, CFL1, CLIC1, CLTA, CTSD, CYBB, DHX9, DSP, F11R, FCER1G, FCN1, FLNA, FLOT1, G6PD, GNAI2, GP1BA

GSTP1, HLA-A, HLA-B, HNRNPA2B1, HNRNPC, HNRNPU, HSPA1B, HSPA8, HSPA9, HSPD1, ILK, INPP5D, IT
A6, ITGB1, LTF, NPM1, PKM, PPIA, PRDX2, RHOA, RPL11, RPS3A, RTN3, RTN4, S100A8, S100A9, SOD1, SR

TGA6, ITGB1, LRP1, LTF, MATR3, MMRN1, MYL12B, MYL6, PKM, PTGS1, S100A8, S100A9, SF3A3, SOD1, S
PDH, GNAI2, GNAQ, GSTP1, HINT1, HNRNPA2B1, HNRNPC, HNRNPF, HNRNPH1, HNRNPH3, HNRNPU, HSI

D, ITGA6, ITGB1, ITGB3, LRP1, LTF, NPM1, PKM, PNPLA8, PPIA, PRDX2, RHOA, RPL11, RPS3A, RTN3, RTN
DM, NPM1, PATJ, PLEK, PPIA, PRDX1, PRDX2, PTBP1, PTPRJ, RAB33B, RAC2, RAP1B, RHOA, RPL11, RPS1

4, CAVIN2, CCL5, CCT2, CCT4, CCT6A, CCT7, CCT8, CD151, CD226, CD36, CD47, CD53, CD59, CD9, CD99,

B1, LTF, NPM1, PPIA, PRDX2, RHOA, RPL11, RPS3A, RTN3, RTN4, S100A8, S100A9, SOD1, SRGN, TCP1, TI

A-B, HNRNPA2B1, HNRNPC, HNRNPF, HNRNPH1, HNRNPH3, HNRNPU, HSP90AA1, HSPA1B, HSPA8, HSPI

RP1, MSN, PPIA, PTPRJ, RAB10, RAB27B, RAB33B, RAC2, RAP1B, RHOA, S100A8, SOD1, SRI, STOM, THBS:

.TA, CSDE1, CTSD, CYB5R3, CYBB, DSC1, DSG1, DSP, EEF1A1, EEF2, EIF5A, ENO1, FCER1G, FCN1, FERMT3,
33, JUP, LBP, LRP1, LTF, MSN, NCL, NPM1, PF4V1, PKM, PRDX2, PTPRJ, RAB10, RAP1B, RHOA, RPLP0, SEL
NAQ, GP1BA, GP1BB, GSTP1, HLA-A, HLA-B, HNRNPA2B1, HNRNPC, HNRNPU, HSPA1B, HSPA8, HSPA9, H

.1, HSPA1B, HSPA8, HSPA9, HSPD1, ITGA6, ITGB1, ITGB3, JUP, KRT1, KRT10, KRT14, KRT5, LRBA, MSN, M

HLA-A, HLA-B, HSP90AA1, HSPA1B, HSPA8, HSPD1, ITGB1, ITGB3, KRT1, KRT16, KRT6A, LBP, LTF, LYZ, M.

NPM1, PRDX1, PRDX2, PTGS1, PTPRJ, RAB10, RAC2, RAP1B, RHOA, RPS15A, RTN4, SPN, TFRC, THBS1, U

ILK, ITGB1, ITGB3, MSN, MYADM, MYH9, PKM, PLEK, PTGS1, PTPRJ, RAB10, RAB27B, RAC2, RHOA, RPLP

EIF5A, ENO1, F11R, FCER1G, FCN1, FLNA, FLOT1, G3BP1, G6PD, GAPDH, GNAI2, GNAQ, GSTP1, HINT1,
, GNAI2, GP1BA, GP1BB, HNRNPU, HSP90AA1, HSPA8, ITGB1, ITGB3, JUP, KRT5, LRP1, MSN, NPM1, PEC
NRNPC, HNRNPU, HSP90AA1, HSPA1B, HSPA8, ITGA6, ITGB3, KRT17, LRP1, MSN, MYADM, MYH9, NPM1

1, HSPA1B, HSPA8, HSPA9, HSPD1, ITGA6, ITGB1, ITGB3, JUP, KRT1, KRT10, KRT14, KRT5, LRBA, MSN, M
LRP1, LTF, MYADM, NCL, NPM1, PECAM1, PKM, PPIA, PTBP1, PTPRJ, RHOA, RPL11, RPS15, S100A8, S10
LRP1, LTF, MYADM, NCL, NPM1, PECAM1, PKM, PPIA, PTBP1, PTPRJ, RHOA, RPL11, RPS15, S100A8, S10
CSDE1, DHX9, DSG1, DSP, F11R, FCER1G, FERMT3, FLG2, FLNA, FLOT1, G6PD, GAPDH, GNAI2, GNAQ, GP

2, GNAQ, GP1BA, GSDMA, GSTP1, H2BC21, HLA-A, HLA-B, HSP90AA1, HSPA8, ITGB3, JUP, KRT1, KRT16,
DHX9, DSC1, DSG1, EEF1A1, EEF2, ENDOD1, ENO1, FCN1, FLG2, G3BP1, GNAI2, GNAQ, GSDMA, GSR, GS

P1BB, GSTP1, HINT1, HLA-A, HLA-B, HNRNPA2B1, HNRNPA3, HNRNPC, HNRNPF, HNRNPH1, HNRNPH3, H

RDX2, RHOA, RPL11, RPS3A, RTN4, S100A8, S100A9, SOD1, TCP1, TFRC, THBS1, TMEM109, UBQLN1, VD
2, RHOA, RPLP0, RPS23, RPS3A, RTN3, RTN4, SACM1L, SRI, STOM, SYNCRIP, THBS1, TKT, TMEM109, TMX

B, HSPA8, ILK, ITGA6, JUP, KANSL3, KRT17, LTF, NCL, NPM1, PCBP1, PFN1, PKM, PPIA, PRDX2, PSMA6, RI
IAI2, GP1BA, GSTP1, HLA-A, HLA-B, HSP90AA1, HSPA1B, HSPA9, HSPD1, INPP5D, ITGB1, ITGB3, JUP, KRT:
R, FCER1G, FCN1, FERMT3, FLNA, FLOT1, G3BP1, G6PD, GAPDH, GNAQ, GP1BA, GP1BB, GSDMA, GSR, GS

TGB3, JUP, LRP1, LTF, NPM1, PFN1, PLEK, PPIA, PSMA6, RCC2, RHOA, RPL11, S100A8, S100A9, SOD1, TC

DHX9, DSG1, EEF1A1, EEF2, EIF5A, ENDOD1, ENO1, FLNA, FSCN1, G3BP1, G6PD, GALE, GAPDH, GNAI2, (
CN1, FLNA, FLOT1, G6PD, GAPDH, GNAI2, GNAQ, GP1BA, GP1BB, GSTP1, HINT1, HMBS, HNRNPU, HSP9(
CLIC1, CSDE1, CTSD, CYBB, DAZAP1, DHX9, EEF2, EIF5A, ENO1, F11R, FCER1G, FCN1, FERMT3, FLNA, FLOT

D36, CD47, CD53, CD59, CD9, CD99, CDC42, CFL1, CLIC1, CLTA, CSDE1, CTSD, CYB5R3, CYBB, DCD, DHX9,

1, CTSD, CYBB, DAZAP1, DCD, DHX9, DSP, EEF1A1, EEF2, EIF5A, ENDOD1, ENO1, F11R, FCER1G, FCN1, FL
, HSPA1B, HSPD1, ILK, ITGB3, KANSL3, KRT17, LRP1, LTF, MSN, MYADM, MYH9, NCL, NPM1, PECAM1, PK

A1, KRT1, KRT16, KRT6A, LBP, LTF, LYZ, MATR3, PF4V1, PRDX1, PRDX2, RHOA, RPS15A, S100A8, S100A9,
6, ITGB1, ITGB3, LRP1, LTF, NPM1, PLEK, PPIA, PTPRJ, RAC2, RCC2, RHOA, RPL11, RPS15, S100A8, S100A
A1, KRT1, KRT16, KRT6A, LBP, LTF, LYZ, MATR3, PF4V1, PRDX1, PRDX2, RHOA, RPS15A, S100A8, S100A9,
N, MYADM, MYH9, PKM, PLEK, PTGS1, PTPRJ, RAB10, RAB27B, RAC2, RHOA, RPLP0, RTN3, RTN4, SELPLG

, CCT4, CCT6A, CCT7, CCT8, CD36, CD47, CD53, CD59, CD9, CDC42, CFL1, CLIC1, CLTA, CSDE1, CTSD, CYB5

A1, HSPA1B, HSPA8, HSPD1, ILK, ITGA6, ITGB3, JUP, KANSL3, KRT17, LRP1, LTF, MSN, MYH9, NCL, NPM1,
PH3, HNRNPU, HSP90AA1, HSPA1B, HSPA8, HSPD1, ILK, ITGA6, JUP, KANSL3, LTF, NASP, NCL, NPM1, PCE
, HNRNPC, HNRNPU, HSP90AA1, HSPA1B, HSPA8, ILK, ITGA6, JUP, KANSL3, KRT17, LTF, NANS, NCL, NPM

DSP, EIF5A, ENO1, F11R, FERMT3, FLNA, FLOT1, FSCN1, G3BP1, G6PD, GAPDH, H2BC21, HNRNPA2B1, H

ILK, ITGB3, KANSL3, MYH9, NASP, NCL, NPM1, PCBP1, PSMA5, PSMA6, PTBP1, RPL11, RPS15, RPS15A, RI
1, ILK, LBP, LRP1, LTF, MYH9, PF4V1, PGK1, PKM, PNPLA8, PPIA, RAB10, RAB1B, RAB27B, RAB33B, RAC2,

36, CD47, CDC42, CPN1, CSDE1, CTSD, CYB5R3, CYBB, DAZAP1, DCD, DHX9, DSP, EEF1A1, EEF2, EIF5A, E
QSOX1, RHOA, RPL11, RTN3, RTN4, SYNCRIP, TNKS1BP1, XRCC5, YWHAB, YWHAE, YWHAG, YWHAZ

9, LRP1, MSN, MYADM, MYH9, NPM1, PATJ, PFN1, PLEK, RAC2, RHOA, S100A8, S100A9, SOD1, SPTBN1,
A1, HSPD1, KRT1, KRT16, KRT6A, LBP, LTF, LYZ, MATR3, PF4V1, PRDX1, PRDX2, PTPRJ, RHOA, RPS15A, S
DSP, EIF5A, ENO1, F11R, FERMT3, FLNA, FLOT1, FSCN1, G3BP1, G6PD, GAPDH, H2BC21, HNRNPA2B1, H
4, CAVIN2, CCL5, CCT2, CCT4, CCT6A, CCT7, CCT8, CD151, CD226, CD36, CD47, CD53, CD59, CD9, CD99,
4, CAVIN2, CCL5, CCT2, CCT4, CCT6A, CCT7, CCT8, CD151, CD226, CD36, CD47, CD53, CD59, CD9, CD99,

N, MYH9, NPM1, PECAM1, PKM, PPIA, PTBP1, PTPRJ, RAB1B, RHOA, S100A8, S100A9, SYNCRIP, TFRC, TH

, ILK, ITGB1, ITGB3, JUP, LDHB, LRBA, MSN, NCL, NPM1, PFN1, PLEK, PRDX6, PTPRJ, RAC2, RCC2, RHOA, I

5, CCT2, CCT4, CCT6A, CCT7, CCT8, CD151, CD226, CD36, CD47, CD53, CD59, CD9, CD99, CDC42, CFL1, C

PA2B1, HNRNPA3, HNRNPC, HNRNPF, HNRNPH1, HNRNPH3, HNRNPU, HSP90AA1, HSPA1B, HSPA8, HSPI
NRNPU, HSPA1B, HSPA8, HSPA9, HSPD1, ILK, ITGB3, JUP, LBP, LRP1, LTF, MSN, NCL, NPM1, PF4V1, PGK1

, PCYOX1, PGK1, PKM, PNPLA8, PRDX6, PSMA5, PSMA6, RPL11, SYNCRIP, TNKS1BP1, UBE2L3, UBQLN1,

T1, G3BP1, GAPDH, HLA-A, HNRNPA2B1, HNRNPC, HNRNPU, HSP90AA1, HSPA1B, HSPA8, HSPD1, ILK, IT
NRNPA3, HNRNPC, HNRNPF, HNRNPH1, HNRNPH3, HNRNPU, HSP90AA1, HSPA1B, HSPA8, HSPD1, ILK, IT
N1, FLOT1, G3BP1, GAPDH, GNAI2, GSTP1, HLA-A, HNRNPA2B1, HNRNPC, HNRNPU, HSP90AA1, HSPA1B
3, HNRNPC, HNRNPF, HNRNPH1, HNRNPH3, HNRNPU, HSP90AA1, HSPA1B, HSPA8, HSPD1, ILK, ITGA6, JU
KRT6A, LBP, LRP1, LTF, LYZ, MATR3, PF4V1, PRDX1, PTPRJ, RAC2, RTN4, S100A8, S100A9, SPN, SYNCRIP,

90AA1, HSPA1B, HSPA8, ILK, ITGA6, ITGB3, JUP, KANSL3, KRT17, LTF, NCL, NPM1, PCBP1, PECAM1, PKM

.4, CAVIN2, CCL5, CCT2, CCT4, CCT6A, CCT7, CCT8, CD151, CD226, CD36, CD47, CD53, CD59, CD9, CD99,

HINT1, HNRNPC, HNRNPU, HSPA1B, ILK, INPP5D, JUP, KRT1, KRT10, KRT14, KRT15, KRT16, KRT17, KRT2,
ADM, NCL, NPM1, PPIA, PTBP1, PTPRJ, RPL11, RPS15, RTN3, RTN4, SRGN, SYNCRIP, THBS1, TNKS1BP1, V
9, MYH9, MYL12B, PSMA6, RAC2, RCC2, SLC2A1, SPTBN1, SRI, TCP1, TPM4, TUBA1C, TUBA4A, TUBB, TU
9, MYH9, MYL12B, PSMA6, RAC2, RCC2, SLC2A1, SPTBN1, SRI, TCP1, TPM4, TUBA1C, TUBA4A, TUBB, TU
PGK1, PKM, PNPLA8, PSMA6, RAB10, RAB1B, RAB27B, RAB33B, RAC2, RAP1B, RHOA, TCP1, TTLL12, TUB
ISPA1B, HSPA8, HSPA9, HSPD1, ILK, LDHB, MYH9, PCYOX1, PFN1, PGK1, PKM, PNPLA8, PSMA6, QSOX1, I
NRNPA3, HNRNPC, HNRNPF, HNRNPH1, HNRNPH3, HNRNPU, HSP90AA1, HSPA1B, HSPA8, HSPD1, ILK, IT
NRNPA2B1, HNRNPA3, HNRNPC, HNRNPF, HNRNPH1, HNRNPH3, HNRNPU, HSP90AA1, HSPA1B, HSPA8,

ADM, NCL, NPM1, PLEK, PPIA, PTBP1, PTPRJ, QSOX1, RHOA, RPL11, RPS15, RTN3, RTN4, SOD1, SRGN, S
A1, EEF2, EIF5A, ENO1, FCN1, FLNA, FLOT1, G6PD, GAPDH, GNAI2, GNAQ, GP1BA, GP1BB, GSR, GSTP1, H
PA9, ILK, INPP5D, ITGA6, ITGB1, ITGB3, JUP, KRT1, KRT10, KRT16, KRT17, KRT2, KRT5, KRT6A, KRT6B, KR

P5D, ITGA6, ITGB3, LDHA, LDHB, LRP1, LTF, MYADM, NCL, NPM1, PECAM1, PGK1, PKM, PLEK, PNPLA8, P
D36, CD47, CDC42, CPN1, CSDE1, CTSD, CYB5R3, CYBB, DAZAP1, DCD, DHX9, DSP, EEF1A1, EEF2, EIF5A, E
HSP90AA1, HSPA1B, HSPA8, HSPA9, ILK, ITGB3, KANSL3, MATR3, MYH9, NASP, NCL, NPM1, PCBP1, PSM
90AA1, HSPA1B, HSPA8, HSPA9, HSPD1, ILK, LDHB, MYH9, OSBPL2, PCYOX1, PFN1, PGK1, PKM, PNPLA8,
U, HSP90AA1, HSPA1B, HSPA8, HSPA9, HSPD1, ILK, MYH9, OSBPL2, PCYOX1, PFN1, PGK1, PKM, PLEK, PN

PTPRJ, RPL11, RPS15, RTN3, RTN4, SYNCRIP, THBS1, TNKS1BP1, XRCC5, YWHAB, YWHAE, YWHAG, YWH
, CCT2, CCT4, CCT6A, CCT7, CCT8, CD151, CD36, CD47, CD53, CD59, CD9, CD99, CDC42, CFL1, CLIC1, CLT
LNA, GNAI2, GSR, GSTP1, H2BC21, HINT1, HNRNPA2B1, HNRNPA3, HNRNPC, HNRNPF, HNRNPH1, HNRN
LNA, GNAI2, GSR, GSTP1, H2BC21, HINT1, HNRNPA2B1, HNRNPA3, HNRNPC, HNRNPF, HNRNPH1, HNRN
LNA, GNAI2, GSR, GSTP1, H2BC21, HINT1, HNRNPA2B1, HNRNPA3, HNRNPC, HNRNPF, HNRNPH1, HNRN
P5D, ITGA6, ITGB3, LDHA, LDHB, LRP1, LTF, MYADM, NANS, NCL, NPM1, PECAM1, PGK1, PKM, PLEK, PNF
NRNPF, HNRNPH1, HNRNPH3, HNRNPU, HSP90AA1, HSPA1B, HSPA8, HSPA9, HSPD1, LRP1, MATR3, MSN

1, CSDE1, CTSD, CYB5R3, DAZAP1, DCD, DHX9, DSP, EEF1A1, EEF2, EIF5A, ENDOD1, ENO1, FCN1, FLNA,

HMBS, HNRNPA2B1, HNRNPC, HNRNPU, HSP90AA1, HSPA1B, HSPA8, ILK, INPP5D, ITGA6, JUP, KANSL3,
, HNRNPA2B1, HNRNPC, HNRNPU, HSP90AA1, HSPA1B, HSPA8, ILK, INPP5D, ITGA6, JUP, KANSL3, KRT17
1, ENO1, G3BP1, G6PD, GALE, GAPDH, GNAI2, GNAQ, GNG5, GSR, GSTP1, HINT1, HMBS, HSP90AA1, HSP
O1, F11R, FCER1G, FCN1, FLNA, FLOT1, G3BP1, GAPDH, GNAI2, GNAQ, GNG5, GP1BA, GP1BB, GSTP1, H2

1, CSDE1, CTSD, CYB5R3, DAZAP1, DCD, DHX9, DSP, EEF1A1, EEF2, EIF5A, ENDOD1, ENO1, FCN1, FLNA,

ISPA1B, HSPA8, HSPA9, HSPD1, ILK, LDHB, MYH9, PCYOX1, PFN1, PGK1, PKM, PNPLA8, QSOX1, RAB10, R
PGK1, PKM, PNPLA8, RAB10, RAB1B, RAB27B, RAB33B, RAC2, RAP1B, RHOA, TCP1, TTLL12, TUBA1C, TU
PGK1, PKM, PNPLA8, RAB10, RAB1B, RAB27B, RAB33B, RAC2, RAP1B, RHOA, TCP1, TTLL12, TUBA1C, TU

:SDE1, CTSD, CYB5R3, CYBB, DAZAP1, DHX9, EEF1A1, EEF2, EIF5A, ENDOD1, ENO1, FLNA, FLOT1, G3BP1,

T5, KRT6A, KRT6B, KRT78, KRT9, MYH9, MYL12B, NCL, PCBP1, PSMA6, RAC2, RCC2, RPLP0, SLC2A1, SPTB

, CCT4, CCT6A, CCT7, CCT8, CD36, CD47, CD53, CD59, CD9, CDC42, CFL1, CLIC1, CLTA, CSDE1, CTSD, CYB5

TR3, MSN, PRDX2, PTPRJ, RAC2, RHOA, RTN4, SOD1, SPN, TFRC, THBS1, TTLL12, UBQLN1, XRCC5
PFN1, PGK1, PKM, PNPLA8, RAB10, RAB1B, RAB27B, RAB33B, RAC2, RAP1B, RHOA, TCP1, TTLL12, TUBA1

, ENDOD1, ENO1, FLNA, FSCN1, G3BP1, G6PD, GAPDH, GNAI2, GNAQ, GSR, H2AC20, H2BC21, HINT1, HL
HINT1, HMBS, HNRNPA2B1, HNRNPC, HNRNPU, HSP90AA1, HSPA1B, HSPA8, ILK, INPP5D, ITGA6, JUP, KA

, ENDOD1, ENO1, FLNA, FSCN1, G3BP1, G6PD, GAPDH, GNAI2, GNAQ, GSR, H2AC20, H2BC21, HINT1, HL

AA1, HSPA1B, HSPA8, HSPA9, HSPD1, INPP5D, LTF, LYZ, PNPLA8, PRDX6, PSMA6, PTPRJ, RAB10, RAB1B, F

O1, FERMT3, FLNA, FLOT1, FSCN1, G3BP1, G6PD, GAPDH, GNAI2, H2AC20, H2BC21, HINT1, HNRNPA2B1
O1, FERMT3, FLNA, FLOT1, FSCN1, G3BP1, G6PD, GAPDH, GNAI2, H2AC20, H2BC21, HINT1, HNRNPA2B1

D1, FLNA, FLOT1, G3BP1, G6PD, GAPDH, GSR, GSTP1, HINT1, HMBS, HNRNPA2B1, HNRNPA3, HNRNPC, H

, CCT2, CCT4, CCT6A, CCT7, CCT8, CD151, CD36, CD47, CD53, CD59, CD9, CD99, CDC42, CFL1, CLIC1, CLT

, CCT7, CCT8, CD36, CD47, CD53, CD59, CD9, CDC42, CFL1, CLIC1, CLTA, CSDE1, CTSD, CYB5R3, CYBB, DA

C, HNRNPU, HSP90AA1, HSPA1B, HSPA9, HSPD1, ITGB1, ITGB3, KRT1, KRT14, KRT15, KRT16, KRT17, KRT2

, PFN1, PLEK, RAP1B, RHOA, RPL11, RPLP0, RPS15, RPSA, RTN4, SF3A3, SLC2A1, SPTBN1, TCP1, TFRC, TL

CD36, CD47, CD53, CD59, CD9, CDC42, CFL1, CLIC1, CLTA, CSDE1, CTSD, CYB5R3, CYBB, DAZAP1, DHX9, D
RPLP0, RPS15, RPS15A, RPS23, RPS3A, RPSA, SPTBN1, THBS1, TLN1, TPM4, TUBA1C, TUBA4A, TUBB, TUB

PM1, OSBPL2, PFN1, PLEK, RAP1B, RHOA, RPL11, RPLP0, RPS15, RPSA, RTN4, SF3A3, SLC2A1, SPTBN1, TC

NASP, NCL, NPM1, PCYOX1, PKM, PSMA5, PSMA6, RAB1B, RHOA, RPL11, RPL8, RPLP0, RPS15, RPS15A,

3, KRT17, LBP, MSN, MYH9, NCL, NPM1, PKM, PRDX6, PTPRJ, RHOA, RPL11, SOD1, SPN, SPTBN1, SYNCRI

LNA, G3BP1, G6PD, GAPDH, GNAI2, GNAQ, GSTP1, H2AC20, H2BC21, HINT1, HNRNPA2B1, HNRNPA3, HN

ꞯJ, RAP1B, RHOA, RPL11, RPS15, RTN4, S100A8, S100A9, SOD1, SPTBN1, SRI, TFRC, THBS1, TSPAN14, TTL
10, RAC2, RAP1B, RHOA, RPL8, RPLP0, RPS15, RPS3A, RTN4, SLC2A1, TLN1, TNKS1BP1, TPM4, VCL, VIM, '
A, FLOT1, FSCN1, G6PD, GAPDH, GNAI2, GNAQ, GNG5, GP1BA, GSR, GSTP1, H2AC20, H2BC21, HINT1, HL

LC2A3, SPTBN1, SRI, STOM, TCP1, TFRC, THBS1, UBE2L3, YWHAB, YWHAE, YWHAG, YWHAH, YWHAZ

IA, PRDX2, PTPRJ, RAB10, RAC2, RCC2, RHOA, RPSA, S100A8, S100A9, SELPLG, SPN, TFRC, THBS1, TLN1,

0AA1, HSPA1B, HSPD1, ILK, INPP5D, ITGA2B, ITGA6, ITGB1, ITGB3, JUP, LBP, LGALS3BP, LRP1, LTF, MYAD

D1, ILK, INPP5D, ITGA2B, ITGA6, ITGB1, ITGB3, JUP, LBP, LGALS3BP, LRP1, LTF, MYADM, MYH9, NCL, NP

DX2, PTBP1, PTPRJ, RAP1B, RHOA, RPL11, RPS15, RTN4, S100A8, S100A9, SOD1, SPTBN1, SRI, TFRC, THB
N1, FERMT3, FLG2, FLNA, FLOT1, FSCN1, G6PD, GAPDH, GNAI2, GNAQ, GNG5, GP1BA, GSR, GSTP1, H2A

FN1, PGK1, PKM, RAB10, RAB33B, RAC2, RCC2, RHOA, RTN4, SOD1, SPTBN1, TFRC, THBS1, VCL, YWHAH,

SPA1B, HSPA8, HSPD1, ILK, INPP5D, ITGA2B, ITGA6, ITGB1, ITGB3, JUP, LBP, LGALS3BP, LRP1, LTF, MYAD
I1, PATJ, PECAM1, PFN1, PPIA, PTPRJ, RAB10, RAB27B, RAB33B, RAC2, RAP1B, RHOA, RPL8, RPLP0, RPS1
ER1G, FCN1, FLG2, FLNA, FLOT1, FSCN1, G6PD, GAPDH, GNAI2, GNAQ, GNG5, GP1BA, GP1BB, GSDMA,

P90AA1, HSPA1B, HSPA8, HSPD1, ITGA2B, ITGB1, ITGB3, JUP, KRT1, LGALS3BP, LRP1, LTF, LYZ, M6PR, MI
P90AA1, HSPA1B, HSPA8, HSPD1, ITGA2B, ITGB1, ITGB3, JUP, KRT1, LGALS3BP, LRP1, LTF, LYZ, M6PR, MI

YH9, NPM1, PKM, PPIA, RAP1B, RPSA, SPTBN1, TFRC, THBS1, TLN1, TNKS1BP1, TPM4, TUBB, VIM, XRCC
APDH, GNAI2, GNAQ, GNG5, GP1BA, GSR, GSTP1, H2AC20, H2BC21, HINT1, HLA-A, HLA-B, HNRNPA2B1,
APDH, GNAI2, GNAQ, GNG5, GP1BA, GSR, GSTP1, H2AC20, H2BC21, HINT1, HLA-A, HLA-B, HNRNPA2B1,
APDH, GNAI2, GNAQ, GNG5, GP1BA, GSR, GSTP1, H2AC20, H2BC21, HINT1, HLA-A, HLA-B, HNRNPA2B1,
APDH, GNAI2, GNAQ, GNG5, GP1BA, GSR, GSTP1, H2AC20, H2BC21, HINT1, HLA-A, HLA-B, HNRNPA2B1,

, LRP1, M6PR, MSN, MYADM, MYH9, NASP, NPM1, OSBPL2, PECAM1, PLEK, PPIA, RAB10, RAB1B, RAB27

'ECAM1, PLEK, PPIA, RAB10, RAB1B, RAB27B, RAB33B, RAC2, RCC2, RPL11, RTN4, SPTBN1, SRGN, SRI, ST
'ECAM1, PLEK, PPIA, RAB10, RAB1B, RAB27B, RAB33B, RAC2, RCC2, RPL11, RTN4, SPTBN1, SRGN, SRI, ST

B3, KRT1, KRT16, KRT6A, LBP, LRP1, LTF, LYZ, MATR3, MSN, MYH9, PECAM1, PF4V1, PLEK, PPIA, PRDX1,
DM, MYH9, NASP, NPM1, OSBPL2, PECAM1, PLEK, PNPLA8, PPIA, RAB10, RAB1B, RAB27B, RAB33B, RAC2
1B, RHOA, RPL11, RPS15, RTN4, S100A8, S100A9, SOD1, SPTBN1, TFRC, THBS1, TSPAN14, TTLL12, UBQLI

KRT1, LGALS3BP, LTF, LYZ, MMRN1, MYH9, PECAM1, PKM, PPIA, PRDX6, PSMA5, PTPRJ, QSOX1, RAB10,

NDOD1, ENO1, F11R, FCER1G, FCN1, FERMT3, FLG2, FLNA, FLOT1, FSCN1, G6PD, GAPDH, GNAI2, GNAQ,

PR, MYADM, PECAM1, PNPLA8, PTPRJ, QSOX1, RAC2, RTN3, RTN4, SACM1L, SELPLG, SLC2A1, SLC2A3, SI

3P, LTF, LYZ, MMRN1, MYH9, PECAM1, PKM, PPIA, PRDX6, PSMA5, PTPRJ, QSOX1, RAB10, RAB27B, RAP1

OX1, RAB1B, RAB33B, RPL11, S100A8, S100A9, SYNCRIP, TNKS1BP1, UBE2L3, UBQLN1, VIM, YWHAH
RJ, QSOX1, RAB10, RAB1B, RAB27B, RAB33B, RAC2, RAP1B, RCC2, RHOA, RTN3, SACM1L, SLC2A1, SLC2A

L1, CLIC1, CLTA, CPN1, CSDE1, CTSD, CYB5R3, CYBB, DAZAP1, DCD, DDX60L, DHX9, DSC1, DSG1, DSP, EE

A, FLOT1, FSCN1, G6PD, GAPDH, GNAI2, GNAQ, GNG5, GP1BA, GP1BB, GSDMA, GSR, HINT1, HLA-A, HLA-
MA6, PTPRJ, RAC2, RCC2, RHOA, RPL11, RPS15, S100A8, S100A9, SOD1, SRI, STOM, TCP1, TFRC, THBS1,
1, M6PR, MYADM, PECAM1, PNPLA8, PTPRJ, QSOX1, RAB27B, RAC2, RTN3, RTN4, SACM1L, SELPLG, SLC

LTF, MATR3, MYH9, NASP, NCL, PECAM1, PFN1, PGK1, PKM, PLEK, PPIA, PRDX1, PRDX2, PTPRJ, RAB10,

HSP90AA1, HSPA1B, HSPA9, HSPD1, ILK, INPP5D, ITGA2B, ITGA6, ITGB1, ITGB3, JUP, KRT1, KRT10, KRT14
TOM, TFRC, THBS1, TKT, TMX2, TPM4, UBQLN1, VIM, YWHAB, YWHAE, YWHAG, YWHAH, YWHAZ

, CLTA, CPN1, CSDE1, CTSD, CYB5R3, CYBB, DAZAP1, DCD, DHX9, DSC1, DSG1, DSP, EEF1A1, EEF2, EIF5A,

KRT17, LRP1, LTF, MATR3, MYADM, MYH9, NASP, NCL, PECAM1, PFN1, PGK1, PKM, PLEK, PPIA, PRDX1,

33, JUP, KRT1, LBP, LRP1, LTF, MATR3, MMRN1, MYADM, NCL, NPM1, PLEK, PNPLA8, PPIA, PRDX1, PRDX

)0A8, S100A9, SRI, SYNCRIP, TCP1, TFRC, THBS1, TNKS1BP1, UBE2L3, VIM, XRCC5, YWHAH, YWHAZ
, RHOA, RPL11, RPS15, RTN4, S100A8, S100A9, SOD1, TFRC, THBS1, TSPAN14, UBQLN1, XRCC5

, ENO1, F11R, FCER1G, FCN1, FERMT3, FLNA, FLOT1, FSCN1, G3BP1, G6PD, GAPDH, GNAI2, GNAQ, GNG5

ITGB1, ITGB3, LDHB, LRBA, LRP1, M6PR, MATR3, PECAM1, PNPLA8, PTGS1, PTPRJ, QSOX1, RAB10, RAB1

CER1G, FCN1, FERMT3, FLNA, FLOT1, G3BP1, G6PD, GAPDH, GNAI2, GNAQ, GNG5, GP1BA, GP1BB, GSDM

GNAQ, GNG5, GP1BA, GP1BB, GSR, GSTP1, HINT1, HLA-A, HNRNPU, HSP90AA1, HSPA1B, HSPA8, HSPA9,

, S100A8, S100A9, SRI, SYNCRIP, TCP1, TFRC, THBS1, TNKS1BP1, UBE2L3, VIM, XRCC5, YWHAH, YWHAZ

;G1, DSP, EEF1A1, EEF2, EIF5A, ENO1, F11R, FCER1G, FCN1, FERMT3, FLG2, FLNA, FLOT1, FSCN1, G3BP1,
HSP90AA1, HSPA1B, HSPA8, HSPA9, HSPD1, ITGB1, ITGB3, JUP, LBP, LGALS3BP, LRP1, LTF, M6PR, MSN,
1A1, EEF2, EIF5A, ENO1, F11R, FCER1G, FCN1, FERMT3, FLNA, FLOT1, FSCN1, G3BP1, G6PD, GAPDH, GN/

RNPA2B1, HNRNPU, HSP90AA1, HSPA1B, HSPA8, HSPA9, HSPD1, ITGB1, ITGB3, JUP, LBP, LGALS3BP, LRP
TGB3, JUP, LBP, LRP1, LTF, MSN, NASP, NCL, NPM1, PF4V1, PKM, PRDX2, PTPRJ, RAB10, RAP1B, RHOA, R

BP1, RHOA, S100A8, S100A9, SRI, SYNCRIP, TCP1, TFRC, TNKS1BP1, UBE2L3, VIM, XRCC5, YWHAH, YWHA
A6, RHOA, S100A8, S100A9, SOD1, SRI, TCP1, TFRC, TNKS1BP1, UBE2L3, XRCC5, YWHAH, YWHAZ
SP90AA1, HSPA1B, HSPA8, HSPD1, ILK, ITGA6, ITGB3, JUP, KANSL3, KRT17, LRP1, LTF, MSN, MYADM, MY

SPA1B, HSPA8, ILK, ITGA6, ITGB3, JUP, KANSL3, KRT17, LRP1, LTF, MYADM, NCL, NPM1, PCBP1, PECAM1,
HINT1, HLA-A, HLA-B, HNRNPA2B1, HNRNPC, HNRNPF, HNRNPH1, HNRNPH3, HNRNPU, HSP90AA1, HSPA
AI2, GNG5, GP1BA, GP1BB, GSTP1, HINT1, HLA-A, HLA-B, HNRNPA2B1, HNRNPU, HSP90AA1, HSPA1B, HS

6, RHOA, S100A8, S100A9, SOD1, SRI, SYNCRIP, TCP1, TFRC, THBS1, TNKS1BP1, UBE2L3, VIM, XRCC5, YW
, HSPD1, ILK, ITGB1, ITGB3, JUP, LBP, LRP1, LTF, MSN, NASP, NCL, NPM1, PF4V1, PGK1, PKM, PNPLA8, PI

HNRNPC, HNRNPH3, HNRNPU, HRNR, HSP90AA1, HSPA1B, HSPA9, HSPD1, ILK, INPP5D, ITGA2B, ITGA6,

, KRT10, KRT14, KRT15, KRT16, KRT17, KRT2, KRT5, KRT6A, KRT6B, KRT78, KRT9, LRP1, LTF, MSN, MYAD
, KRT10, KRT14, KRT15, KRT16, KRT17, KRT2, KRT5, KRT6A, KRT6B, KRT78, KRT9, LRP1, LTF, MSN, MYAD
8, S100A9, SF3A3, SRI, SYNCRIP, TFRC, TNKS1BP1, UBE2L3, VIM, WBP11, XRCC5, YWHAH, YWHAZ
A, FLOT1, G3BP1, G6PD, GAPDH, GNAI2, GNAQ, GNG5, GP1BA, GP1BB, GSTP1, HLA-A, HLA-B, HNRNPC, H

, GP1BB, GSTP1, HNRNPA2B1, HNRNPU, HSP90AA1, HSPA1B, HSPA8, HSPA9, HSPD1, ITGB1, ITGB3, JUP,

GA6, ITGB1, ITGB3, JUP, KRT16, LRP1, LTF, MYADM, MYH9, NCL, NPM1, PFN1, PLEK, PPIA, PRDX2, PTBP

P90AA1, HSPA1B, HSPA8, HSPD1, ILK, ITGA6, ITGB3, JUP, KANSL3, KRT17, LRP1, LTF, MSN, MYADM, MYH

5, RTN4, S100A8, S100A9, SOD1, TFRC, THBS1, TMEM109, UBQLN1, VDAC2, YWHAE, YWHAZ

CDC42, CFL1, CLIC1, CLTA, CPN1, CSDE1, CTSD, CYB5R3, CYBB, DAZAP1, DCD, DDX60L, DHX9, DSC1, DSG

D1, ILK, INPP5D, ITGA6, ITGB3, JUP, KANSL3, KRT17, LBP, LRP1, LTF, MATR3, MSN, MYADM, MYH9, NCL,

, FLG2, FLNA, FLOT1, GAPDH, GNAQ, GSTP1, HLA-A, HLA-B, HRNR, HSP90AA1, HSPA1B, HSPA8, HSPD1, I

ISPD1, ILK, INPP5D, ITGA6, ITGB1, ITGB3, JUP, KRT1, KRT16, KRT6A, LBP, LRP1, LTF, MMRN1, MYADM, M

YADM, NASP, NPM1, OSBPL2, PATJ, PECAM1, PFN1, PLEK, PTPRJ, QSOX1, RAB1B, RAB33B, RAC2, RAP1B

ATR3, PF4V1, PPIA, PRDX1, PRDX2, RHOA, RPS15A, RPSA, S100A8, S100A9, SELPLG, SPN, STOM, SYNCRI

ˈHLA-A, HLA-B, HNRNPA2B1, HNRNPC, HNRNPF, HNRNPH1, HNRNPH3, HNRNPU, HSP90AA1, HSPA1B, HSI
AM1, PPIA, PTPRJ, RAB10, RAB27B, RAB33B, RAC2, RAP1B, RHOA, RTN4, S100A8, SOD1, SPTBN1, SRI, ST
., PATJ, PFN1, PLEK, PTPRJ, RAB1B, RAB33B, RAC2, RAP1B, RCC2, RHOA, RTN4, S100A8, S100A9, SPTBN1,

YADM, NASP, NPM1, OSBPL2, PATJ, PECAM1, PFN1, PLEK, PTPRJ, QSOX1, RAB1B, RAB33B, RAC2, RAP1B
0A9, SOD1, SPTBN1, TFRC, THBS1, TNKS1BP1, TTLL12, UBE2L3, UBQLN1, XRCC5, YWHAB, YWHAE, YWHA
0A9, SOD1, SPTBN1, TFRC, THBS1, TNKS1BP1, TTLL12, UBE2L3, UBQLN1, XRCC5, YWHAB, YWHAE, YWHA
1BA, GP1BB, GSR, GSTP1, HNRNPC, HNRNPU, HRNR, HSP90AA1, HSPA1B, HSPA8, HSPA9, HSPD1, ILK, INI

KRT5, KRT6A, LBP, LTF, LYZ, MATR3, MMRN1, PF4V1, PPIA, PRDX1, PRDX2, PSMA6, PTPRJ, RAC2, RHOA,
TP1, HNRNPU, HRNR, HSP90AA1, HSPA1B, HSPA8, HSPA9, HSPD1, ILK, ITGA2B, ITGA6, ITGB1, LRP1, LTF,

HNRNPU, HSP90AA1, HSPA1B, HSPA8, HSPD1, ILK, INPP5D, ITGA6, ITGB3, JUP, KANSL3, KRT17, LBP, LRP1

HOA, RPL11, RPL8, RPLP0, RPS15, RPS15A, RPS23, RPS3A, RPSA, S100A8, S100A9, SRI, SYNCRIP, TCP1, TF
1, KRT17, LBP, LRP1, LTF, PFN1, PGK1, PKM, PRDX2, PTPRJ, RHOA, RTN4, SOD1, SPN, SPTBN1, SRGN, SRI,
STP1, H2BC21, HLA-A, HLA-B, HSP90AA1, HSPA1B, HSPA8, HSPA9, HSPD1, ILK, ITGB1, ITGB3, KRT1, KRT16

GSDMA, GSR, GSTP1, H2BC21, HINT1, HMBS, HNRNPC, HNRNPF, HNRNPH1, HNRNPU, HSP90AA1, HSPA1
0AA1, HSPA1B, HSPA8, HSPD1, ILK, ITGB3, KANSL3, KRT1, KRT10, KRT17, KRT2, LRP1, LTF, MSN, MYADM
T1, FSCN1, G3BP1, G6PD, GAPDH, GNAI2, GNG5, GP1BA, GP1BB, GSTP1, HINT1, HLA-A, HLA-B, HNRNPA2

, DSC1, DSG1, DSP, EEF1A1, EEF2, EIF5A, ENDOD1, ENO1, F11R, FCER1G, FCN1, FERMT3, FLG2, FLNA, FL

NA, FLOT1, G3BP1, G6PD, GAPDH, GNAI2, GNAQ, GP1BA, GP1BB, GSTP1, HINT1, HLA-A, HLA-B, HMBS, H
:M, PPIA, PTBP1, PTPRJ, RAB1B, RHOA, RPL11, RPS15, RTN3, RTN4, S100A8, S100A9, SOD1, SYNCRIP, TFI

5R3, CYBB, DAZAP1, DCD, DHX9, DSC1, DSG1, DSP, EEF1A1, EEF2, EIF5A, ENDOD1, ENO1, F11R, FCER1G,

, PCBP1, PECAM1, PKM, PPIA, PRDX6, PTBP1, PTPRJ, RAB1B, RHOA, S100A8, S100A9, SYNCRIP, TCP1, TF
3P1, PFN1, PPIA, PRDX2, PRDX6, PSMA6, PTBP1, RHOA, RPL11, RPS15, S100A8, S100A9, SF3A3, SRI, SYN
1, PCBP1, PFN1, PKM, PPIA, PRDX2, PSMA6, RHOA, RPL11, RPL8, RPLP0, RPS15, RPS15A, RPS23, RPS3A,

NRNPC, HNRNPU, HRNR, HSP90AA1, HSPA1B, HSPA8, HSPA9, HSPD1, ILK, ITGA6, ITGB1, ITGB3, JUP, KAN

PS23, RPS3A, RPSA, S100A9, SF3A3, SH3BGRL3, SOD1, SPN, SRI, SYNCRIP, TKT, UBE2L3, UBQLN1, WBP11

NDOD1, ENO1, F11R, FCER1G, FCN1, FLNA, FLOT1, G3BP1, G6PD, GALE, GAPDH, GNAI2, GNAQ, GP1BA,

NRNPC, HNRNPU, HRNR, HSP90AA1, HSPA1B, HSPA8, HSPA9, HSPD1, ILK, ITGA6, ITGB1, ITGB3, JUP, KAN
CDC42, CFL1, CLIC1, CLTA, CPN1, CSDE1, CTSD, CYB5R3, CYBB, DAZAP1, DCD, DDX60L, DHX9, DSC1, DSG
CDC42, CFL1, CLIC1, CLTA, CPN1, CSDE1, CTSD, CYB5R3, CYBB, DAZAP1, DCD, DDX60L, DHX9, DSC1, DSG

RPL11, RTN4, SLC2A1, SOD1, SPTBN1, SRI, STOM, TCP1, TFRC, TNKS1BP1, TSPAN14, TUBA4A, TUBB, UBE

LIC1, CLTA, CPN1, CSDE1, CTSD, CYB5R3, CYBB, DAZAP1, DCD, DHX9, DSC1, DSG1, DSP, EEF1A1, EEF2, EI

D1, ILK, ITGA6, JUP, KANSL3, LDHA, LDHB, LTF, NANS, NASP, NCL, NPM1, OSBPL2, PCBP1, PFN1, PGK1, PI
., PKM, PNPLA8, PPIA, PRDX1, PRDX2, PRDX6, PTGS1, PTPRJ, RAB10, RAC2, RAP1B, RHOA, RPLP0, S100A

GA6, ITGB3, JUP, KANSL3, KRT17, LBP, LRP1, LTF, MSN, MYH9, NCL, NPM1, PCBP1, PECAM1, PKM, PPIA,
TGA6, JUP, KANSL3, LDHA, LDHB, LTF, NANS, NASP, NCL, NPM1, PCBP1, PFN1, PGK1, PKM, PPIA, PRDX2,
, HSPA8, HSPD1, ILK, ITGA6, ITGB3, JUP, KANSL3, KRT17, LBP, LRP1, LTF, MSN, MYH9, NCL, NPM1, PCBP
JP, KANSL3, LDHA, LDHB, LTF, NANS, NASP, NCL, NPM1, PCBP1, PFN1, PGK1, PKM, PPIA, PRDX2, PRDX6,

, PLEK, PPIA, PRDX6, PTBP1, PTPRJ, RAB1B, RHOA, SOD1, SYNCRIP, TCP1, TFRC, THBS1, TNKS1BP1, UBE2

CDC42, CFL1, CLIC1, CLTA, CPN1, CSDE1, CTSD, CYB5R3, CYBB, DAZAP1, DCD, DDX60L, DHX9, DSC1, DSG

KRT5, KRT6A, KRT6B, KRT78, KRT9, LRP1, MSN, MYADM, MYH16, MYH9, MYL12B, MYL6, NPM1, PATJ, F

RAB10, RAB1B, RAB27B, RAB33B, RAC2, RAP1B, RHOA, TCP1, TTLL12, TUBA1C, TUBA4A, TUBB, TUBB1, U
TGA6, JUP, KANSL3, LDHA, LDHB, LTF, NANS, NASP, NCL, NPM1, PCBP1, PFN1, PGK1, PKM, PPIA, PRDX2,
HSPA9, HSPD1, LRP1, LTF, MATR3, MSN, MYH9, NCL, NPM1, PCBP1, PFN1, PKM, PPIA, PRDX1, PSMA6, F

INT1, HMBS, HNRNPU, HSP90AA1, HSPA1B, HSPA8, HSPD1, ILK, ITGB3, KANSL3, KRT1, KRT10, KRT17, KF
T78, KRT9, LTF, MATR3, MSN, MYH9, MYL6, NASP, PECAM1, PLEK, PRDX2, PTPRJ, RAB33B, RHOA, RPLP0

PIA, PRDX6, PTBP1, PTPRJ, RHOA, SACM1L, SOD1, SPTBN1, TFRC, THBS1, TKT, TNKS1BP1, XRCC5, YWHA
NDOD1, ENO1, F11R, FCER1G, FCN1, FLNA, FLOT1, G3BP1, G6PD, GALE, GAPDH, GNAI2, GNAQ, GP1BA,
A5, PSMA6, PTBP1, RCC2, RPL11, RPS15, RPS15A, RPS23, RPS3A, RPSA, S100A9, SF3A3, SH3BGRL3, SOD
QSOX1, RAB10, RAB1B, RAB27B, RAB33B, RAC2, RAP1B, RHOA, S100A8, S100A9, SLC2A3, TCP1, TKT, TT
IPLA8, PSMA6, QSOX1, RAB10, RAB1B, RAB27B, RAB33B, RAC2, RAP1B, RHOA, S100A8, S100A9, TCP1, T

A, CSDE1, CTSD, CYB5R3, CYBB, DAZAP1, DDX60L, DHX9, DSC1, DSG1, DSP, EEF1A1, EEF2, EIF5A, ENDOD

PH3, HNRNPU, HRNR, HSP90AA1, HSPA1B, HSPA8, HSPA9, HSPD1, ILK, ITGB3, JUP, KANSL3, KRT1, LGALS

PH3, HNRNPU, HRNR, HSP90AA1, HSPA1B, HSPA8, HSPA9, HSPD1, ILK, ITGB3, JUP, KANSL3, KRT1, LGALS

PH3, HNRNPU, HRNR, HSP90AA1, HSPA1B, HSPA8, HSPA9, HSPD1, ILK, ITGB3, JUP, KANSL3, KRT1, LGALS

LA8, PPIA, PRDX6, PTBP1, PTPRJ, RHOA, SACM1L, SOD1, SPTBN1, TFRC, THBS1, TKT, TNKS1BP1, XRCC5,

, MYH9, NCL, NPM1, PCBP1, PFN1, PKM, PPIA, PRDX1, PSMA6, PTBP1, RCC2, RPL11, RPL8, RPLP0, RPS1

LOT1, G3BP1, G6PD, GAPDH, GNAI2, GNAQ, GP1BA, GP1BB, GSR, GSTP1, HINT1, HMBS, HNRNPA2B1, H

KRT17, LTF, NANS, NCL, NPM1, OSBPL2, PCBP1, PFN1, PKM, PLEK, PNPLA8, PPIA, PRDX2, PSMA6, PTGS1

, LTF, NANS, NCL, NPM1, OSBPL2, PCBP1, PFN1, PGK1, PKM, PLEK, PNPLA8, PPIA, PRDX2, PSMA6, PTGS1

A1B, HSPA8, HSPA9, HSPD1, ILK, INPP5D, ITGB3, LDHA, LDHB, LTF, LYZ, NANS, PCYOX1, PGK1, PKM, PNF

AC20, H2BC21, HINT1, HLA-A, HLA-B, HNRNPA2B1, HNRNPA3, HNRNPC, HNRNPF, HNRNPH1, HNRNPH3

LOT1, G3BP1, G6PD, GALE, GAPDH, GNAI2, GNAQ, GP1BA, GP1BB, GSTP1, HINT1, HMBS, HNRNPA2B1,

AB1B, RAB27B, RAB33B, RAC2, RAP1B, RHOA, TCP1, TTLL12, TUBA1C, TUBA4A, TUBB, TUBB1, UBE2L3, \

, G6PD, GAPDH, GNAI2, GNAQ, GSR, GSTP1, HINT1, HMBS, HNRNPA2B1, HNRNPA3, HNRNPC, HNRNPF, H

5R3, CYBB, DAZAP1, DCD, DHX9, DSC1, DSG1, DSP, EEF1A1, EEF2, EIF5A, ENDOD1, ENO1, F11R, FCER1G,

A-A, HNRNPA2B1, HNRNPA3, HNRNPC, HNRNPF, HNRNPH1, HNRNPH3, HNRNPU, HSP90AA1, HSPA1B, H
ANSL3, KRT17, LTF, NANS, NCL, NPM1, OSBPL2, PCBP1, PFN1, PGK1, PKM, PLEK, PNPLA8, PPIA, PRDX2, P

A-A, HNRNPA2B1, HNRNPA3, HNRNPC, HNRNPF, HNRNPH1, HNRNPH3, HNRNPU, HSP90AA1, HSPA1B, H

, HNRNPC, HNRNPU, HSPA1B, HSPA8, HSPA9, ILK, INPP5D, JUP, KRT1, KRT10, KRT14, KRT15, KRT16, KRT
, HNRNPC, HNRNPU, HSPA1B, HSPA8, HSPA9, ILK, INPP5D, JUP, KRT1, KRT10, KRT14, KRT15, KRT16, KRT

INRNPF, HNRNPH1, HNRNPH3, HNRNPU, HSP90AA1, HSPA1B, HSPA8, HSPD1, ILK, ITGA6, JUP, KANSL3, K

A, CSDE1, CTSD, CYB5R3, CYBB, DAZAP1, DDX60L, DHX9, DSC1, DSG1, DSP, EEF1A1, EEF2, EIF5A, ENDOD
ZAP1, DHX9, DSC1, DSG1, DSP, EEF1A1, EEF2, EIF5A, ENO1, FCER1G, FCN1, FERMT3, FLG2, FLNA, FLOT1,

!, KRT5, KRT6A, KRT6B, KRT78, KRT9, LRBA, LRP1, MSN, MYADM, MYH9, NASP, NPM1, PATJ, PFN1, PLEK,

)SC1, DSG1, DSP, EEF1A1, EEF2, EIF5A, ENO1, FCER1G, FCN1, FERMT3, FLG2, FLNA, FLOT1, G3BP1, G6PD

RPS23, RPS3A, RPSA, SYNCRIP, TCP1, TFRC, THBS1, TNKS1BP1, UBE2L3, UBQLN1, VIM, XRCC5

NRNPC, HNRNPF, HNRNPH1, HNRNPH3, HNRNPU, HRNR, HSP90AA1, HSPA1B, HSPA8, HSPA9, ILK, ITGB3,

A-A, HLA-B, HNRNPA2B1, HNRNPC, HRNR, HSP90AA1, HSPA1B, HSPA8, HSPA9, HSPD1, ITGA2B, ITGB1, I

OM, MYH9, NCL, NPM1, PATJ, PECAM1, PF4V1, PLEK, PPIA, PRDX1, PRDX2, PTBP1, PTPRJ, RAB33B, RAC2

M1, PATJ, PECAM1, PF4V1, PLEK, PPIA, PRDX1, PRDX2, PTBP1, PTPRJ, RAB33B, RAC2, RAP1B, RCC2, RHO

C20, H2BC21, HINT1, HLA-A, HLA-B, HNRNPA2B1, HNRNPC, HRNR, HSP90AA1, HSPA1B, HSPA8, HSPA9, I

M, MYH9, NCL, NPM1, PATJ, PECAM1, PF4V1, PLEK, PPIA, PRDX1, PRDX2, PTBP1, PTPRJ, RAB33B, RAC2,
5, RPS23, RPS3A, RTN3, RTN4, S100A9, SLC2A1, SPTBN1, TLN1, TNKS1BP1, TPM4, VCL, VIM, YWHAB, YW
GSR, HINT1, HLA-A, HLA-B, HNRNPF, HRNR, HSP90AA1, HSPA1B, HSPA8, HSPD1, ICAM2, ILK, INPP5D, ITG

MRN1, MYH9, PECAM1, PKM, PPIA, PRDX1, PRDX6, PSMA5, PTPRJ, QSOX1, RAB10, RAB1B, RAB27B, RAE
MRN1, MYH9, PECAM1, PKM, PPIA, PRDX1, PRDX6, PSMA5, PTPRJ, QSOX1, RAB10, RAB1B, RAB27B, RAE

HNRNPC, HRNR, HSP90AA1, HSPA1B, HSPA8, HSPA9, HSPD1, ITGA2B, ITGB1, ITGB3, JUP, KPRP, KRT1, KF
HNRNPC, HRNR, HSP90AA1, HSPA1B, HSPA8, HSPA9, HSPD1, ITGA2B, ITGB1, ITGB3, JUP, KPRP, KRT1, KF
HNRNPC, HRNR, HSP90AA1, HSPA1B, HSPA8, HSPA9, HSPD1, ITGA2B, ITGB1, ITGB3, JUP, KPRP, KRT1, KF
HNRNPC, HRNR, HSP90AA1, HSPA1B, HSPA8, HSPA9, HSPD1, ITGA2B, ITGB1, ITGB3, JUP, KPRP, KRT1, KF

7B, RAB33B, RAC2, RAP1B, RCC2, RPL11, RPS15, RTN4, S100A8, S100A9, SOD1, SPTBN1, SRGN, SRI, STON

OM, TCP1, TFRC, TSPAN14, UBE2L3, VCL, XRCC5, YWHAB, YWHAE, YWHAG, YWHAH, YWHAZ
OM, TCP1, TFRC, TSPAN14, UBE2L3, VCL, XRCC5, YWHAB, YWHAE, YWHAG, YWHAH, YWHAZ

PRDX2, PTPRJ, RAB10, RAC2, RHOA, RTN4, S100A8, S100A9, SELPLG, SOD1, SPN, SYNCRIP, TFRC, THBS1,
2, RCC2, RPL11, RTN4, SLC2A1, SPTBN1, SRGN, SRI, STOM, TCP1, TFRC, THBS1, TSPAN14, UBE2L3, VCL, X

RAB27B, RAP1B, RHOA, S100A8, S100A9, SLC2A3, SOD1, SRGN, SRI, STOM, TCP1, THBS1, TSPAN14, TUE

, GNG5, GP1BA, GSR, GSTP1, H2AC20, H2BC21, HINT1, HLA-A, HLA-B, HNRNPA2B1, HNRNPC, HRNR, HSP

1B, RHOA, S100A8, S100A9, SLC2A3, SOD1, SRGN, SRI, STOM, TCP1, THBS1, TSPAN14, TUBB, VCL, VDAC2

F1A1, EEF2, EIF5A, ENDOD1, ENO1, F11R, FCER1G, FCN1, FERMT3, FLG2, FLNA, FLOT1, FSCN1, G3BP1, G

-B, HNRNPF, HRNR, HSP90AA1, HSPA1B, HSPA8, HSPD1, ICAM2, ILK, INPP5D, ITGA2B, ITGA6, ITGB1, ITGE

RAB33B, RAC2, RHOA, RTN3, RTN4, S100A8, S100A9, SELPLG, SLC2A1, SOD1, SPN, SPTBN1, SRI, TFRC, TH

, KRT15, KRT16, KRT17, KRT2, KRT5, KRT6A, KRT6B, KRT78, KRT9, LRP1, LTF, MATR3, MSN, MYADM, MY

ENDOD1, ENO1, F11R, FCER1G, FCN1, FERMT3, FLG2, FLNA, FLOT1, FSCN1, G3BP1, G6PD, GAPDH, GNA

PRDX2, PTPRJ, RAB10, RAB33B, RAC2, RHOA, RTN3, RTN4, S100A8, S100A9, SELPLG, SLC2A1, SOD1, SP

(2, PSMA6, PTBP1, PTPRJ, RAC2, RAP1B, RHOA, RPL11, RPS15, RTN4, S100A8, S100A9, SOD1, SPN, SPTB

5, GP1BA, GP1BB, GSR, GSTP1, HINT1, HLA-A, HLA-B, HNRNPA2B1, HNRNPC, HNRNPF, HNRNPH1, HNRNI

B, RAB27B, RAB33B, RAC2, RAP1B, RCC2, RHOA, RTN3, RTN4, SACM1L, SLC2A1, SLC2A3, SRI, STOM, TFR

1A, GSR, GSTP1, H2BC21, HINT1, HLA-A, HLA-B, HNRNPU, HSP90AA1, HSPA1B, HSPA8, HSPA9, HSPD1, ILI

HSPD1, ILK, INPP5D, ITGA2B, ITGA6, ITGB1, ITGB3, JUP, LBP, LGALS3BP, LRP1, LTF, MSN, MYADM, MYH

G6PD, GAPDH, GNAI2, GNAQ, GNG5, GP1BA, GP1BB, GSR, GSTP1, HINT1, HLA-A, HLA-B, HNRNPA2B1, H
MYH9, NASP, NPM1, OSBPL2, PCYOX1, PECAM1, PLEK, PNPLA8, PPIA, PTPRJ, RAB10, RAB1B, RAB27B, RA
AI2, GNAQ, GNG5, GP1BA, GP1BB, GSR, GSTP1, HINT1, HLA-A, HLA-B, HNRNPA2B1, HNRNPC, HNRNPF, H

1, LTF, M6PR, MSN, MYH9, NASP, NPM1, OSBPL2, PCYOX1, PECAM1, PLEK, PNPLA8, PPIA, PTPRJ, RAB10
RPLP0, S100A8, S100A9, SELPLG, SLC2A1, SOD1, SRI, SYNCRIP, TFRC, THBS1, TTLL12, UBE2L3, UBQLN1, V

YH9, NCL, NPM1, PCBP1, PECAM1, PFN1, PKM, PLEK, PPIA, PRDX2, PRDX6, PSMA6, PTBP1, PTPRJ, RAB1B

, PFN1, PKM, PLEK, PPIA, PRDX2, PRDX6, PSMA6, PTBP1, PTPRJ, QSOX1, RAB1B, RAC2, RHOA, RPL11, RT
A1B, HSPA8, HSPD1, ILK, INPP5D, ITGA6, ITGB1, ITGB3, JUP, KANSL3, KRT17, LBP, LRP1, LTF, MATR3, MS
SPA8, HSPD1, ILK, INPP5D, ITGA6, ITGB1, ITGB3, JUP, KANSL3, KRT17, KRT6A, LBP, LRP1, LTF, MMRN1, M

PIA, PRDX1, PRDX2, PRDX6, PTGS1, PTPRJ, RAB10, RAC2, RAP1B, RHOA, RPLP0, S100A8, S100A9, SELPLG

ITGB1, ITGB3, JUP, KRT1, KRT10, KRT14, KRT15, KRT16, KRT17, KRT2, KRT5, KRT6A, KRT6B, KRT78, KRT9,

M, MYH9, PECAM1, PGK1, PLEK, PPIA, PRDX2, PTBP1, PTPRJ, RAB10, RAC2, RAP1B, RCC2, RHOA, RPS15,
M, MYH9, PECAM1, PGK1, PLEK, PPIA, PRDX2, PTBP1, PTPRJ, RAB10, RAC2, RAP1B, RCC2, RHOA, RPS15,

HNRNPU, HRNR, HSP90AA1, HSPA1B, HSPA9, HSPD1, ILK, INPP5D, ITGA2B, ITGA6, ITGB1, ITGB3, JUP, KRT

KRT5, LBP, LGALS3BP, LRBA, LRP1, LTF, M6PR, MSN, MYADM, MYH9, NASP, NPM1, OSBPL2, PCYOX1, PI

1, PTPRJ, QSOX1, RAB33B, RAP1B, RCC2, RHOA, RPL11, RPS3A, RTN3, RTN4, SOD1, SPN, SPTBN1, SRI, SY

H9, NCL, NPM1, PCBP1, PECAM1, PFN1, PKM, PPIA, PRDX2, PRDX6, PSMA6, PTBP1, PTPRJ, RAB1B, RHOA

1, DSP, EEF1A1, EEF2, EIF5A, ENDOD1, ENO1, F11R, FCER1G, FCN1, FERMT3, FLG2, FLNA, FLOT1, FSCN1,

NPM1, PCBP1, PFN1, PKM, PPIA, PRDX2, PRDX6, PSMA6, PTBP1, PTPRJ, RHOA, RPL11, S100A8, S100A9,

TGA2B, ITGB1, ITGB3, JUP, KRT1, LGALS3BP, LRBA, LRP1, LTF, LYZ, M6PR, MATR3, MMRN1, MYH9, PECA

IYH9, NCL, NPM1, PFN1, PGK1, PLEK, PPIA, PRDX2, PTBP1, PTPRJ, QSOX1, RAB33B, RAP1B, RCC2, RHOA,

, RCC2, RHOA, RPL11, RPLP0, RPS15, RPS23, RPSA, RTN3, RTN4, SF3A3, SLC2A1, SPTBN1, TCP1, TFRC, TH

ᐳA8, HSPD1, ILK, INPP5D, ITGA6, ITGB3, JUP, KANSL3, KRT17, LBP, LRP1, LTF, MATR3, MSN, MYADM, MY

, RCC2, RHOA, RPL11, RPLP0, RPS15, RPS23, RPSA, RTN3, RTN4, SF3A3, SLC2A1, SPTBN1, TCP1, TFRC, TH

PP5D, ITGB1, ITGB3, JUP, KRT1, KRT16, LTF, LYZ, MMRN1, MSN, MYADM, MYH9, NPM1, PECAM1, PF4V1

RPS15A, S100A8, S100A9, SLC2A1, SOD1, SPN, SYNCRIP, TFRC, THBS1, TTLL12, TUBB, VIM, XRCC5
MATR3, MMRN1, MYH9, MYL12B, MYL6, OSBPL2, PCYOX1, PFN1, PGK1, PKM, PLEK, PNPLA8, PSMA6, P

., LTF, MATR3, MSN, MYADM, MYH9, NCL, NPM1, PCBP1, PFN1, PGK1, PKM, PPIA, PRDX2, PRDX6, PSMA

5, KRT6A, LBP, LGALS3BP, LRP1, LTF, LYZ, MATR3, MMRN1, MYH9, NPM1, PF4V1, PGK1, PLEK, PNPLA8, F

1B, HSPA8, HSPD1, ILK, INPP5D, JUP, KRT1, KRT10, KRT14, KRT15, KRT16, KRT17, KRT2, KRT5, KRT6A, KR
, MYH9, NCL, NPM1, PCYOX1, PECAM1, PGK1, PKM, PPIA, PSMA5, PSMA6, PTBP1, PTPRJ, RAB1B, RHOA,
2B1, HNRNPC, HNRNPU, HSP90AA1, HSPA1B, HSPA8, HSPD1, ILK, INPP5D, ITGA6, ITGB1, ITGB3, JUP, KAN

DT1, FSCN1, G6PD, GAPDH, GNAI2, GNAQ, GNG5, GP1BA, GP1BB, GSDMA, GSR, HINT1, HLA-A, HLA-B, HI

INRNPA2B1, HNRNPA3, HNRNPC, HNRNPF, HNRNPH1, HNRNPH3, HNRNPU, HSP90AA1, HSPA1B, HSPA8,

FCN1, FERMT3, FLG2, FLNA, FLOT1, FSCN1, G3BP1, G6PD, GAPDH, GNAI2, GNAQ, GNG5, GP1BA, GSR, G

RPSA, S100A8, S100A9, SRI, SYNCRIP, TCP1, TFRC, THBS1, TNKS1BP1, UBE2L3, VIM, XRCC5, YWHAH, YW

SL3, KRT1, KRT10, KRT14, KRT15, KRT16, KRT17, KRT2, KRT5, KRT6A, KRT6B, KRT78, KRT9, LRBA, LRP1,

GP1BB, GSR, GSTP1, HINT1, HLA-A, HLA-B, HMBS, HNRNPA2B1, HNRNPA3, HNRNPC, HNRNPF, HNRNPH1

SL3, KRT1, KRT10, KRT14, KRT15, KRT16, KRT17, KRT2, KRT5, KRT6A, KRT6B, KRT78, KRT9, LRBA, LRP1,
1, DSP, EEF1A1, EEF2, EIF5A, ENDOD1, ENO1, F11R, FCER1G, FCN1, FERMT3, FLG2, FLNA, FLOT1, FSCN1,
1, DSP, EEF1A1, EEF2, EIF5A, ENDOD1, ENO1, F11R, FCER1G, FCN1, FERMT3, FLG2, FLNA, FLOT1, FSCN1,

F5A, ENDOD1, ENO1, F11R, FCER1G, FCN1, FERMT3, FLNA, FLOT1, FSCN1, G3BP1, G6PD, GALE, GAPDH,

‹M, PPIA, PRDX2, PRDX6, PSMA6, PTBP1, RHOA, RPL11, RPS15, S100A8, S100A9, SF3A3, SLC2A1, SLC2A3
8, S100A9, SELPLG, SLC2A1, SOD1, SRI, SYNCRIP, TFRC, THBS1, TTLL12, UBE2L3, UBQLN1, VIM, XRCC5, Y

PRDX6, PTBP1, PTPRJ, RAB1B, RHOA, RPL11, S100A8, S100A9, SOD1, SPN, SPTBN1, SYNCRIP, TCP1, TFR
PRDX6, PSMA6, PTBP1, RHOA, RPL11, RPS15, S100A8, S100A9, SF3A3, SLC2A1, SLC2A3, SRI, SYNCRIP, TC
1, PECAM1, PKM, PLEK, PPIA, PRDX6, PTBP1, PTPRJ, RAB1B, RHOA, RPL11, S100A8, S100A9, SOD1, SPN,
PSMA6, PTBP1, RHOA, RPL11, RPS15, S100A8, S100A9, SF3A3, SRI, SYNCRIP, TCP1, TFRC, TKT, TNKS1BP

ı1, DSP, EEF1A1, EEF2, EIF5A, ENDOD1, ENO1, F11R, FCER1G, FCN1, FERMT3, FLG2, FLNA, FLOT1, FSCN1,

ʔFN1, RAB10, RAC2, RCC2, RHOA, S100A8, S100A9, SLC2A1, SPTBN1, STOM, TAGLN2, TCP1, TLN1, TNKS1

PRDX6, PSMA6, PTBP1, RHOA, RPL11, RPS15, S100A8, S100A9, SF3A3, SRI, SYNCRIP, TCP1, TFRC, TKT, TI
ʔTBP1, RCC2, RPL11, RPL8, RPLP0, RPS15, RPS15A, RPS23, RPS3A, RPSA, RTN4, SF3A3, SPTBN1, SYNCRIP,

ʀT2, LDHA, LDHB, LRP1, LTF, MSN, MYADM, MYH9, NCL, NPM1, PCYOX1, PECAM1, PGK1, PKM, PNPLA8,
), RTN3, RTN4, SELPLG, SLC2A1, SOD1, SPN, SRGN, SRI, TFRC, TMX2, VIM, XRCC5, YWHAE, YWHAH, YWH

GP1BB, GSR, GSTP1, HINT1, HLA-A, HLA-B, HMBS, HNRNPA2B1, HNRNPA3, HNRNPC, HNRNPF, HNRNPH1

1, ENO1, FCER1G, FCN1, FERMT3, FLG2, FLNA, FLOT1, FSCN1, G3BP1, G6PD, GALE, GAPDH, GNAI2, GNA
3BP, LTF, LYZ, MATR3, MMRN1, MYH9, NASP, NCL, NPM1, PCBP1, PKM, PPIA, PRDX6, PSMA5, PSMA6, P
3BP, LTF, LYZ, MATR3, MMRN1, MYH9, NASP, NCL, NPM1, PCBP1, PKM, PPIA, PRDX6, PSMA5, PSMA6, P
3BP, LTF, LYZ, MATR3, MMRN1, MYH9, NASP, NCL, NPM1, PCBP1, PKM, PPIA, PRDX6, PSMA5, PSMA6, P

5, RPS15A, RPS23, RPS3A, RPSA, RTN4, SF3A3, SPTBN1, SYNCRIP, TCP1, TFRC, UBE2L3, VIM, WBP11, XRC

NRNPA3, HNRNPC, HNRNPF, HNRNPH1, HNRNPH3, HNRNPU, HSP90AA1, HSPA1B, HSPA8, HSPD1, ILK, I

, RHOA, RPL11, RPL8, RPLP0, RPS15, RPS15A, RPS23, RPS3A, RPSA, S100A8, S100A9, SACM1L, SOD1, SRI
L, RHOA, RPL11, RPL8, RPLP0, RPS15, RPS15A, RPS23, RPS3A, RPSA, S100A8, S100A9, SACM1L, SOD1, SR
LA8, PPIA, PRDX1, PRDX2, PRDX6, PSMA6, PTGS1, PTPRJ, QSOX1, RAB10, RAB1B, RAB27B, RAB33B, RAC
, HNRNPU, HSP90AA1, HSPA1B, HSPA8, HSPA9, HSPD1, ITGA2B, ITGA6, ITGB1, ITGB3, JUP, KANSL3, LDH

HNRNPA3, HNRNPC, HNRNPF, HNRNPH1, HNRNPH3, HNRNPU, HSP90AA1, HSPA1B, HSPA8, HSPD1, ILK,

HNRNPH1, HNRNPH3, HNRNPU, HSP90AA1, HSPA1B, HSPA8, HSPA9, HSPD1, ILK, INPP5D, ITGA6, ITGB3, .

FCN1, FERMT3, FLG2, FLNA, FLOT1, FSCN1, G3BP1, G6PD, GAPDH, GNAI2, GNAQ, GNG5, GP1BA, GSR, G

ISPA8, HSPA9, HSPD1, ILK, LDHB, LRP1, LTF, MATR3, MSN, MYH9, NCL, NPM1, PCBP1, PCYOX1, PFN1, PG
SMA6, PTGS1, RHOA, RPL11, RPL8, RPLP0, RPS15, RPS15A, RPS23, RPS3A, RPSA, S100A8, S100A9, SACM

ISPA8, HSPA9, HSPD1, ILK, LDHB, LRP1, LTF, MATR3, MSN, MYH9, NCL, NPM1, OSBPL2, PCBP1, PCYOX1,

17, KRT2, KRT5, KRT6A, KRT6B, KRT78, KRT9, LRP1, MSN, MYADM, MYH16, MYH9, MYL12B, MYL6, NASI
17, KRT2, KRT5, KRT6A, KRT6B, KRT78, KRT9, LRP1, MSN, MYADM, MYH16, MYH9, MYL12B, MYL6, NASI

KRT17, LDHA, LDHB, LTF, NANS, NASP, NCL, NPM1, PCBP1, PFN1, PGK1, PKM, PPIA, PRDX2, PRDX6, PSMA

1, ENO1, FCER1G, FCN1, FERMT3, FLG2, FLNA, FLOT1, FSCN1, G3BP1, G6PD, GALE, GAPDH, GNAI2, GNA
FSCN1, G3BP1, G6PD, GAPDH, GNAI2, GNAQ, GSR, GSTP1, H2AC20, H2BC21, HINT1, HLA-A, HLA-B, HNR

PPIA, RAB10, RAB1B, RAB33B, RAC2, RHOA, RPL11, RPLP0, RPS15, RPS23, RPSA, RTN3, RTN4, S100A8, S

, GAPDH, GNAI2, GNAQ, GSR, GSTP1, H2AC20, H2BC21, HINT1, HLA-A, HLA-B, HNRNPA2B1, HNRNPA3, H

JUP, KANSL3, KRT1, KRT10, KRT14, KRT15, KRT16, KRT2, KRT5, KRT6A, KRT9, LDHA, LRP1, LTF, MATR3, M

ΓGB3, JUP, KPRP, KRT1, KRT10, KRT14, KRT15, KRT16, KRT2, KRT5, KRT6A, KRT6B, KRT78, KRT9, LBP, LDH

, RAP1B, RCC2, RHOA, RPL11, RPS15, RTN4, S100A8, S100A9, SOD1, SPN, SPTBN1, SRGN, SRI, TFRC, THB

)A, RPL11, RPS15, RTN4, S100A8, S100A9, SOD1, SPN, SPTBN1, SRGN, SRI, TFRC, THBS1, TLN1, TMEM109

HSPD1, ITGA2B, ITGB1, ITGB3, JUP, KPRP, KRT1, KRT10, KRT14, KRT15, KRT16, KRT2, KRT5, KRT6A, KRT6I

, RAP1B, RCC2, RHOA, RPL11, RPS15, RTN4, S100A8, S100A9, SLC2A1, SOD1, SPN, SPTBN1, SRGN, SRI, T

A2B, ITGA6, ITGB1, ITGB3, JUP, KRT1, KRT10, KRT16, KRT17, KRT2, LGALS3BP, LRBA, LRP1, LTF, M6PR,

33B, RAC2, RAP1B, RCC2, RHOA, S100A8, S100A9, SLC2A1, SLC2A3, SOD1, SRGN, SRI, STOM, TCP1, TFR
33B, RAC2, RAP1B, RCC2, RHOA, S100A8, S100A9, SLC2A1, SLC2A3, SOD1, SRGN, SRI, STOM, TCP1, TFR

RT10, KRT14, KRT15, KRT16, KRT2, KRT5, KRT6A, KRT6B, KRT78, KRT9, LBP, LDHA, LDHB, LGALS3BP, LTF,
RT10, KRT14, KRT15, KRT16, KRT2, KRT5, KRT6A, KRT6B, KRT78, KRT9, LBP, LDHA, LDHB, LGALS3BP, LTF,
RT10, KRT14, KRT15, KRT16, KRT2, KRT5, KRT6A, KRT6B, KRT78, KRT9, LBP, LDHA, LDHB, LGALS3BP, LTF,
RT10, KRT14, KRT15, KRT16, KRT2, KRT5, KRT6A, KRT6B, KRT78, KRT9, LBP, LDHA, LDHB, LGALS3BP, LTF,

M, TCP1, TFRC, TSPAN14, TUBA1C, TUBB, UBE2L3, VCL, VDAC2, XRCC5, YWHAB, YWHAE, YWHAG, YWHA

90AA1, HSPA1B, HSPA8, HSPA9, HSPD1, ITGA2B, ITGB1, ITGB3, JUP, KPRP, KRT1, KRT10, KRT14, KRT15, I

6PD, GALE, GAPDH, GNAI2, GNAQ, GNG5, GP1BA, GP1BB, GSDMA, GSR, GSTP1, H2AC20, H2BC21, HINT1

33, JUP, KRT1, KRT10, KRT16, KRT17, KRT2, LRBA, LRP1, LTF, M6PR, MSN, MYADM, MYH9, NCL, OSBPL2,

H9, MYL12B, MYL6, NASP, NCL, PECAM1, PFN1, PGK1, PKM, PLEK, PPIA, PRDX1, PRDX2, PTBP1, PTPRJ, R

I2, GNAQ, GNG5, GP1BA, GP1BB, GSDMA, GSR, GSTP1, H2BC21, HINT1, HLA-A, HLA-B, HMBS, HNRNPA2

N, SPTBN1, SRGN, SRI, TFRC, THBS1, TMX2, VCL, VIM, XRCC5, YWHAE, YWHAG, YWHAH, YWHAZ

PH3, HNRNPU, HSP90AA1, HSPA1B, HSPA8, HSPA9, HSPD1, ILK, INPP5D, ITGA2B, ITGA6, ITGB1, ITGB3, JU

K, INPP5D, ITGA2B, ITGA6, ITGB1, ITGB3, JUP, KRT1, KRT16, KRT5, KRT6A, LBP, LGALS3BP, LRP1, LTF, LYZ,

9, NCL, NPM1, PATJ, PECAM1, PF4V1, PGK1, PKM, PLEK, PNPLA8, PPIA, PRDX1, PRDX2, PRDX6, PTBP1, PT

HNRNPC, HNRNPF, HNRNPH1, HNRNPH3, HNRNPU, HRNR, HSP90AA1, HSPA1B, HSPA8, HSPA9, HSPD1, IL
AB33B, RAC2, RAP1B, RHOA, RPL11, RPLP0, RPS15, RTN3, S100A8, SLC2A1, SLC2A3, SOD1, SPTBN1, SRI, S
NRNPH1, HNRNPH3, HNRNPU, HSP90AA1, HSPA1B, HSPA8, HSPA9, HSPD1, ILK, INPP5D, ITGA2B, ITGA6,

, RAB1B, RAB27B, RAB33B, RAC2, RAP1B, RCC2, RHOA, RPL11, RPLP0, RPS15, RTN3, S100A8, SLC2A1, SL

3, RHOA, RPL11, RPS15, RTN3, RTN4, S100A8, S100A9, SOD1, SRI, SYNCRIP, TCP1, TFRC, THBS1, TNKS1BP

N3, RTN4, S100A8, S100A9, SOD1, SRI, SYNCRIP, TCP1, TFRC, THBS1, TNKS1BP1, UBE2L3, UBQLN1, VIM,

N, MYADM, MYH9, NCL, NPM1, PCBP1, PECAM1, PFN1, PKM, PLEK, PPIA, PRDX2, PRDX6, PSMA6, PTBP1

MSN, MYADM, NCL, NPM1, PCBP1, PECAM1, PFN1, PKM, PLEK, PPIA, PRDX2, PRDX6, PTBP1, PTPRJ, RAB1

, SLC2A1, SOD1, SPN, SRI, SYNCRIP, TFRC, THBS1, TTLL12, UBE2L3, UBQLN1, VIM, XRCC5, YWHAG, YWHA

, LRP1, LTF, MATR3, MSN, MYADM, MYH9, MYL12B, MYL6, NASP, NCL, PECAM1, PFN1, PGK1, PKM, PLEK

RPS3A, RTN3, RTN4, S100A8, S100A9, SOD1, SPN, SYNCRIP, TAGLN2, TFRC, TPM4, TUBB, VCL, VIM, XRC

RPS3A, RTN3, RTN4, S100A8, S100A9, SOD1, SPN, SYNCRIP, TAGLN2, TFRC, TPM4, TUBB, VCL, VIM, XRC

T1, KRT14, KRT16, KRT17, KRT2, KRT5, KRT6A, KRT6B, KRT78, KRT9, LBP, LRP1, LTF, LYZ, MATR3, MMRN1

ECAM1, PLEK, PNPLA8, PPIA, PTPRJ, RAB10, RAB1B, RAB27B, RAB33B, RAC2, RAP1B, RCC2, RHOA, RPL11

NCRIP, TFRC, THBS1, TMEM109, TNKS1BP1, TTLL12, UBQLN1, VCL, VDAC2, VIM, XRCC5, YWHAB, YWHA

, RPL11, RPS15, RTN3, RTN4, S100A8, S100A9, SOD1, SRI, SYNCRIP, TCP1, TFRC, THBS1, TNKS1BP1, UBE2

, G3BP1, G6PD, GALE, GAPDH, GNAI2, GNAQ, GNG5, GP1BA, GP1BB, GSDMA, GSR, GSTP1, H2AC20, H2B

SOD1, SPN, SPTBN1, SRGN, SRI, SYNCRIP, TFRC, THBS1, TNKS1BP1, UBE2L3, VIM, XRCC5, YWHAH, YWH

M1, PKM, PNPLA8, PPIA, PRDX6, PSMA5, PTGS1, PTPRJ, QSOX1, RAB10, RAB1B, RAB27B, RAB33B, RAC2,

RPL11, RPS15, RPS3A, RTN3, RTN4, SOD1, SPN, SPTBN1, SRGN, SRI, SYNCRIP, TFRC, THBS1, TMEM109, T

H9, NCL, NPM1, PCBP1, PECAM1, PFN1, PKM, PPIA, PRDX2, PRDX6, PSMA6, PTBP1, PTPRJ, RAB1B, RHO

, PFN1, PLEK, PNPLA8, PPIA, PRDX1, PRDX2, PRDX6, PTGS1, PTPRJ, RAP1B, RHOA, RPL11, RPLP0, RTN4,

TGS1, QSOX1, RAB10, RAB1B, RAB27B, RAB33B, RAC2, RAP1B, RHOA, S100A8, S100A9, SF3A3, SOD1, SR

\6, PTBP1, PTPRJ, RHOA, RPL11, RPL8, RPLP0, RPS15, RPS15A, RPS23, RPS3A, RPSA, S100A8, S100A9, SF3

ᴾPIA, PRDX1, PRDX2, PRDX6, PSMA6, PTGS1, RHOA, S100A8, S100A9, SLC2A1, SOD1, SPN, SYNCRIP, TFR(

T6B, KRT78, KRT9, LDHA, LDHB, LRBA, MSN, MYH9, MYL12B, MYL6, NANS, NPM1, OSBPL2, PATJ, PCBP1,
, RPL11, RPL8, RPLP0, RPS15, RPS15A, RPS23, RPS3A, RPSA, RTN3, RTN4, S100A8, S100A9, SOD1, SPTBN1
ISL3, KRT1, KRT10, KRT17, KRT2, KRT6A, LBP, LRP1, LTF, MATR3, MMRN1, MSN, MYADM, MYH9, NCL, N

NRNPA2B1, HNRNPC, HNRNPF, HNRNPH1, HNRNPU, HRNR, HSP90AA1, HSPA1B, HSPA8, HSPA9, HSPD1,

, HSPD1, ILK, INPP5D, ITGA6, ITGB3, JUP, KANSL3, KRT1, KRT10, KRT17, KRT2, LBP, LRP1, LTF, MATR3, MS

STP1, H2AC20, H2BC21, HINT1, HLA-A, HLA-B, HNRNPA2B1, HNRNPA3, HNRNPC, HNRNPF, HNRNPH1, H

MSN, MYADM, MYH9, NASP, NPM1, OSBPL2, PATJ, PECAM1, PFN1, PLEK, PPIA, PTPRJ, QSOX1, RAB10, R

L, HNRNPH3, HNRNPU, HSP90AA1, HSPA1B, HSPA8, HSPA9, HSPD1, ILK, INPP5D, ITGA6, ITGB1, ITGB3, JU

MSN, MYADM, MYH9, NASP, NPM1, OSBPL2, PATJ, PECAM1, PFN1, PLEK, PPIA, PTPRJ, QSOX1, RAB10, R
, G3BP1, G6PD, GALE, GAPDH, GNAI2, GNAQ, GNG5, GP1BA, GP1BB, GSDMA, GSR, GSTP1, H2AC20, H2B
, G3BP1, G6PD, GALE, GAPDH, GNAI2, GNAQ, GNG5, GP1BA, GP1BB, GSDMA, GSR, GSTP1, H2AC20, H2B

GNAI2, GNAQ, GNG5, GP1BA, GP1BB, GSDMA, GSTP1, H2AC20, H2BC21, HINT1, HLA-A, HLA-B, HMBS, H

3, SOD1, SRI, SYNCRIP, TCP1, TFRC, TKT, TNKS1BP1, UBE2L3, VIM, WBP11, XRCC5, YWHAH, YWHAZ

, G3BP1, G6PD, GALE, GAPDH, GNAI2, GNAQ, GNG5, GP1BA, GP1BB, GSDMA, GSR, GSTP1, H2AC20, H2B

PPIA, PSMA5, PSMA6, PTBP1, PTPRJ, RAB1B, RHOA, RPL11, RPL8, RPLP0, RPS15, RPS15A, RPS23, RPS3A,

L, HNRNPH3, HNRNPU, HSP90AA1, HSPA1B, HSPA8, HSPD1, ILK, INPP5D, ITGA6, ITGB3, JUP, KANSL3, KR

Q, GSDMA, GSR, GSTP1, H2BC21, HINT1, HLA-A, HLA-B, HMBS, HNRNPA2B1, HNRNPC, HNRNPF, HNRNP
TBP1, QSOX1, RAB33B, RCC2, RPL11, RPS15, RPS15A, RPS23, RPS3A, RPSA, S100A8, S100A9, SF3A3, SH
TBP1, QSOX1, RAB33B, RCC2, RPL11, RPS15, RPS15A, RPS23, RPS3A, RPSA, S100A8, S100A9, SF3A3, SH
TBP1, QSOX1, RAB33B, RCC2, RPL11, RPS15, RPS15A, RPS23, RPS3A, RPSA, S100A8, S100A9, SF3A3, SH

TGA6, ITGB3, JUP, KANSL3, KRT1, KRT10, KRT17, KRT2, LDHA, LDHB, LRP1, LTF, MSN, MYADM, MYH9, N

A, LDHB, LRP1, LTF, MMRN1, MYH16, MYH9, MYL12B, MYL6, NASP, NCL, NPM1, PCBP1, PCYOX1, PECA

INPP5D, ITGA6, ITGB3, JUP, KANSL3, KRT1, KRT10, KRT17, KRT2, LDHA, LDHB, LRP1, LTF, MSN, MYADM,

JUP, KANSL3, KRT17, LDHA, LDHB, LRBA, LRP1, LTF, MYADM, NANS, NASP, NCL, NPM1, OSBPL2, PCBP1,

STP1, H2AC20, H2BC21, HINT1, HLA-A, HLA-B, HNRNPA2B1, HNRNPA3, HNRNPC, HNRNPF, HNRNPH1, H

GK1, PKM, PNPLA8, PPIA, PRDX1, PSMA6, PTBP1, PTGS1, QSOX1, RAB10, RAB1B, RAB27B, RAB33B, RAC2
1L, SOD1, SRI, SYNCRIP, TCP1, TFRC, THBS1, TKT, TNKS1BP1, UBE2L3, VIM, XRCC5, YWHAH, YWHAZ

PFN1, PGK1, PKM, PNPLA8, PPIA, PRDX1, PSMA6, PTBP1, PTGS1, QSOX1, RAB10, RAB1B, RAB27B, RAB3

P, NCL, NPM1, OSBPL2, PATJ, PCBP1, PFN1, PSMA6, PTBP1, RAB10, RAC2, RAP1B, RCC2, RHOA, RPL11, R
P, NCL, NPM1, OSBPL2, PATJ, PCBP1, PFN1, PSMA6, PTBP1, RAB10, RAC2, RAP1B, RCC2, RHOA, RPL11, R

A6, PTBP1, RHOA, RPL11, RPL8, RPLP0, RPS15, RPS15A, RPS23, RPS3A, RPSA, RTN3, RTN4, S100A8, S100/

Q, GSDMA, GSR, GSTP1, H2AC20, H2BC21, HINT1, HLA-A, HLA-B, HMBS, HNRNPA2B1, HNRNPA3, HNRNI
NPA2B1, HNRNPA3, HNRNPC, HNRNPF, HNRNPH1, HNRNPH3, HNRNPU, HRNR, HSP90AA1, HSPA1B, HSI

S100A9, SOD1, SPTBN1, SRGN, TCP1, TFRC, TLN1, TNKS1BP1, TPM4, TUBA1C, TUBA4A, TUBB, TUBB1, UB

HNRNPC, HNRNPF, HNRNPH1, HNRNPH3, HNRNPU, HRNR, HSP90AA1, HSPA1B, HSPA8, HSPA9, HSPD1, II

MSN, MYH9, NASP, NCL, NPM1, PCBP1, PFN1, PKM, PPIA, PRDX1, PRDX6, PSMA5, PSMA6, PTBP1, RAC2,

ıA, LDHB, LGALS3BP, LTF, LYZ, MSN, MYH9, MYL12B, MYL6, NANS, NCL, PATJ, PCBP1, PCYOX1, PECAM1,

S1, TLN1, TMEM109, TSPAN14, TTLL12, UBQLN1, VDAC2, VIM, YWHAB, YWHAE, YWHAG, YWHAH, YWH

3, KRT78, KRT9, LBP, LDHA, LDHB, LGALS3BP, LTF, LYZ, MMRN1, MSN, MYH9, MYL12B, MYL6, NANS, NCI

FRC, THBS1, TLN1, TMEM109, TSPAN14, TTLL12, UBQLN1, VDAC2, VIM, YWHAB, YWHAE, YWHAG, YWHA

MMRN1, MSN, MYADM, MYH9, MYL12B, NCL, OSBPL2, PATJ, PCYOX1, PECAM1, PFN1, PKM, PLEK, PTPRJ,

LYZ, MSN, MYH9, MYL12B, MYL6, NANS, NCL, PATJ, PCBP1, PCYOX1, PECAM1, PFN1, PGK1, PKM, PPIA, P
LYZ, MSN, MYH9, MYL12B, MYL6, NANS, NCL, PATJ, PCBP1, PCYOX1, PECAM1, PFN1, PGK1, PKM, PPIA, P
LYZ, MSN, MYH9, MYL12B, MYL6, NANS, NCL, PATJ, PCBP1, PCYOX1, PECAM1, PFN1, PGK1, PKM, PPIA, P
LYZ, MSN, MYH9, MYL12B, MYL6, NANS, NCL, PATJ, PCBP1, PCYOX1, PECAM1, PFN1, PGK1, PKM, PPIA, P

‹RT16, KRT2, KRT5, KRT6A, KRT6B, KRT78, KRT9, LBP, LDHA, LDHB, LGALS3BP, LRP1, LTF, LYZ, M6PR, MN

l, HLA-A, HLA-B, HMBS, HNRNPA2B1, HNRNPA3, HNRNPC, HNRNPF, HNRNPH1, HNRNPH3, HNRNPU, HR

PATJ, PCYOX1, PECAM1, PLEK, PTPRJ, RAB10, RAB27B, RAC2, RAP1B, RCC2, RHOA, RPSA, RTN3, RTN4, S

.AB10, RAB33B, RAC2, RAP1B, RCC2, RHOA, RPLP0, RTN3, RTN4, S100A8, S100A9, SELPLG, SLC2A1, SOD1

B1, HNRNPA3, HNRNPC, HNRNPF, HNRNPH1, HNRNPH3, HNRNPU, HRNR, HSP90AA1, HSPA1B, HSPA8, H

JP, KANSL3, KRT16, KRT17, KRT5, KRT6A, LBP, LGALS3BP, LRP1, LTF, MMRN1, MSN, MYADM, MYH9, NCI

, MATR3, MMRN1, MSN, MYADM, MYH9, NASP, NCL, NPM1, PATJ, PECAM1, PF4V1, PGK1, PKM, PLEK, P

TGS1, PTPRJ, RAB10, RAB33B, RAC2, RAP1B, RCC2, RHOA, RPL11, RPLP0, RPS15, RTN4, S100A8, S100A9,

.K, INPP5D, ITGA2B, ITGA6, ITGB1, ITGB3, JUP, KANSL3, KRT1, KRT10, KRT16, KRT17, KRT2, KRT5, KRT6A,
STOM, TCP1, TFRC, THBS1, TMEM109, TUBA1C, TUBB, UBE2L3, UBQLN1, VDAC2, YWHAB, YWHAE, YWH
ITGB1, ITGB3, JUP, KANSL3, KRT1, KRT10, KRT16, KRT17, KRT2, KRT5, KRT6A, LBP, LGALS3BP, LRP1, LTF,

C2A3, SOD1, SPTBN1, SRI, STOM, TCP1, TFRC, THBS1, TMEM109, TUBA1C, TUBB, UBE2L3, UBQLN1, VDA

, PTPRJ, QSOX1, RAB1B, RAC2, RHOA, RPL11, RPS15, RTN3, RTN4, S100A8, S100A9, SOD1, SPN, SPTBN1,
.B, RAB27B, RAC2, RAP1B, RCC2, RHOA, RPL11, RPS15, RPS15A, RTN4, S100A8, S100A9, SOD1, SPN, SPTI

K, PPIA, PRDX1, PRDX2, PTBP1, PTPRJ, RAB10, RAB33B, RAC2, RAP1B, RCC2, RHOA, RPLP0, RPS15, RPS3A

L, MYADM, MYH9, MYL6, NASP, NCL, PECAM1, PF4V1, PFN1, PGK1, PKM, PLEK, PPIA, PRDX1, PRDX2, PT

., RPLP0, RPS15, RTN3, RTN4, S100A8, S100A9, SLC2A1, SLC2A3, SOD1, SPTBN1, SRGN, SRI, STOM, TCP1,

C21, HINT1, HLA-A, HLA-B, HMBS, HNRNPA2B1, HNRNPA3, HNRNPC, HNRNPF, HNRNPH1, HNRNPH3, HN

, RAP1B, RCC2, RHOA, RPLP0, RPS23, RPS3A, RTN3, RTN4, S100A8, S100A9, SACM1L, SLC2A1, SLC2A3, S(

TNKS1BP1, TTLL12, UBQLN1, VCL, VDAC2, VIM, XRCC5, YWHAB, YWHAE, YWHAG, YWHAH, YWHAZ

A, RPL11, RPS15, RTN3, RTN4, S100A8, S100A9, SOD1, SPN, SPTBN1, SRGN, SRI, SYNCRIP, TCP1, TFRC, TH

S100A8, S100A9, SLC2A1, SOD1, SPTBN1, SRI, SYNCRIP, TCP1, TFRC, THBS1, TLN1, TNKS1BP1, TUBB, VCL

RI, TCP1, THBS1, TKT, TPM4, TTLL12, TUBA1C, TUBA4A, TUBB, TUBB1, UBE2L3, VDAC2, XRCC5

3A3, SOD1, SPN, SPTBN1, SRGN, SRI, SYNCRIP, TFRC, THBS1, TNKS1BP1, TSPAN14, UBE2L3, VIM, WBP11,

PFN1, PGK1, PKM, PLEK, PPIA, PRDX1, PRDX2, PRDX6, PSMA5, PSMA6, RAB10, RAB1B, RAC2, RAP1B, R(
1, SRGN, SYNCRIP, TFRC, THBS1, TNKS1BP1, TSPAN14, TTLL12, UBE2L3, UBQLN1, VIM, XRCC5, YWHAB, Y
PM1, PCBP1, PECAM1, PFN1, PKM, PLEK, PPIA, PRDX2, PRDX6, PTBP1, PTPRJ, RAB1B, RAB27B, RAC2, RA

ICAM2, ILK, INPP5D, ITGA2B, ITGA6, ITGB1, ITGB3, JUP, KRT1, KRT10, KRT16, KRT17, KRT2, KRT5, KRT6A

SN, MYADM, MYH9, NASP, NCL, NPM1, PCBP1, PCYOX1, PECAM1, PFN1, PGK1, PKM, PPIA, PRDX2, PRDX

NRNPH3, HNRNPU, HRNR, HSP90AA1, HSPA1B, HSPA8, HSPA9, HSPD1, ILK, ITGA2B, ITGB1, ITGB3, JUP,

AB1B, RAB33B, RAC2, RAP1B, RCC2, RHOA, RPL11, RPLP0, RPS15, RPS23, RPSA, RTN3, RTN4, S100A8, S1

JP, KANSL3, KRT1, KRT10, KRT17, KRT2, LBP, LDHA, LDHB, LRBA, LRP1, LTF, LYZ, MATR3, MSN, MYADM,

AB1B, RAB33B, RAC2, RAP1B, RCC2, RHOA, RPL11, RPLP0, RPS15, RPS23, RPSA, RTN3, RTN4, S100A8, S1
C21, HINT1, HLA-A, HLA-B, HMBS, HNRNPA2B1, HNRNPA3, HNRNPC, HNRNPF, HNRNPH1, HNRNPH3, HN
C21, HINT1, HLA-A, HLA-B, HMBS, HNRNPA2B1, HNRNPA3, HNRNPC, HNRNPF, HNRNPH1, HNRNPH3, HN

NRNPA2B1, HNRNPA3, HNRNPC, HNRNPF, HNRNPH1, HNRNPH3, HNRNPU, HSP90AA1, HSPA1B, HSPA8,

C21, HINT1, HLA-A, HLA-B, HMBS, HNRNPA2B1, HNRNPA3, HNRNPC, HNRNPF, HNRNPH1, HNRNPH3, HN

, RPSA, RTN3, RTN4, S100A8, S100A9, SOD1, SPTBN1, SRGN, SYNCRIP, TFRC, THBS1, TKT, TNKS1BP1, TSP

T1, KRT10, KRT17, KRT2, LBP, LDHA, LDHB, LRP1, LTF, MATR3, MSN, MYADM, MYH9, NANS, NASP, NCL,

H1, HNRNPU, HRNR, HSP90AA1, HSPA1B, HSPA8, HSPA9, HSPD1, ILK, INPP5D, ITGA2B, ITGB1, ITGB3, JU

BGRL3, SOD1, SPN, SPTBN1, SRGN, SRI, SYNCRIP, THBS1, TKT, TSPAN14, TUBB, UBE2L3, UBQLN1, VCL,

BGRL3, SOD1, SPN, SPTBN1, SRGN, SRI, SYNCRIP, THBS1, TKT, TSPAN14, TUBB, UBE2L3, UBQLN1, VCL,

BGRL3, SOD1, SPN, SPTBN1, SRGN, SRI, SYNCRIP, THBS1, TKT, TSPAN14, TUBB, UBE2L3, UBQLN1, VCL,

NS, NASP, NCL, NPM1, PCBP1, PCYOX1, PECAM1, PFN1, PGK1, PKM, PNPLA8, PPIA, PRDX2, PRDX6, PS

1, PPIA, PSMA5, PSMA6, RAC2, RPL11, RPL8, RPLP0, RPS15, RPS15A, RPS23, RPS3A, RPSA, SF3A3, SOD

MYH9, NANS, NASP, NCL, NPM1, OSBPL2, PCBP1, PCYOX1, PECAM1, PFN1, PGK1, PKM, PLEK, PNPLA8,

PCYOX1, PECAM1, PFN1, PGK1, PKM, PLEK, PNPLA8, PPIA, PRDX1, PRDX2, PRDX6, PSMA5, PSMA6, PTBP

NRNPH3, HNRNPU, HRNR, HSP90AA1, HSPA1B, HSPA8, HSPA9, HSPD1, ILK, INPP5D, ITGA2B, ITGB1, ITGE

:, RAP1B, RCC2, RHOA, RPL11, RPL8, RPLP0, RPS15, RPS15A, RPS23, RPS3A, RPSA, RTN4, SF3A3, SPTBN1,

3B, RAC2, RAP1B, RCC2, RHOA, RPL11, RPL8, RPLP0, RPS15, RPS15A, RPS23, RPS3A, RPSA, RTN4, SF3A3,

PL8, RPLP0, RPS15, RPS15A, RPS23, RPS3A, RPSA, S100A8, S100A9, SLC2A1, SPTBN1, SRI, STOM, TAGLN2
PL8, RPLP0, RPS15, RPS15A, RPS23, RPS3A, RPSA, S100A8, S100A9, SLC2A1, SPTBN1, SRI, STOM, TAGLN2

A9, SF3A3, SOD1, SRI, SYNCRIP, TCP1, TFRC, THBS1, TKT, TNKS1BP1, UBE2L3, VIM, WBP11, XRCC5, YWH/

PC, HNRNPF, HNRNPH1, HNRNPH3, HNRNPU, HRNR, HSP90AA1, HSPA1B, HSPA8, HSPA9, HSPD1, ILK, INI
PA8, HSPA9, HSPD1, ILK, INPP5D, ITGA2B, ITGB1, ITGB3, JUP, KANSL3, KRT1, KRT10, KRT14, KRT15, KRT1

_K, ITGA2B, ITGB1, ITGB3, JUP, KANSL3, KRT1, KRT10, KRT14, KRT15, KRT16, KRT2, KRT5, KRT6A, KRT9, L

RCC2, RHOA, RPL11, RPLP0, RPS15, RPS15A, RPS23, RPS3A, RPSA, RTN4, S100A8, S100A9, SF3A3, SH3B(

PF4V1, PFN1, PGK1, PKM, PPIA, PRDX1, PRDX2, PRDX6, PSMA5, PSMA6, PTBP1, PTGS1, PTPRJ, QSOX1,

L, PATJ, PCBP1, PCYOX1, PECAM1, PF4V1, PFN1, PGK1, PKM, PLEK, PPIA, PRDX1, PRDX2, PRDX6, PSMA5,

, RAB10, RAB27B, RAC2, RAP1B, RCC2, RHOA, RPSA, RTN3, RTN4, S100A8, S100A9, SELPLG, SLC2A1, SLC2

PRDX1, PRDX2, PRDX6, PSMA5, PSMA6, PTBP1, PTGS1, PTPRJ, QSOX1, RAB10, RAB1B, RAB27B, RAC2, RA
PRDX1, PRDX2, PRDX6, PSMA5, PSMA6, PTBP1, PTGS1, PTPRJ, QSOX1, RAB10, RAB1B, RAB27B, RAC2, RA
PRDX1, PRDX2, PRDX6, PSMA5, PSMA6, PTBP1, PTGS1, PTPRJ, QSOX1, RAB10, RAB1B, RAB27B, RAC2, RA
PRDX1, PRDX2, PRDX6, PSMA5, PSMA6, PTBP1, PTGS1, PTPRJ, QSOX1, RAB10, RAB1B, RAB27B, RAC2, RA

MRN1, MSN, MYH9, MYL12B, MYL6, NANS, NCL, PATJ, PCBP1, PCYOX1, PECAM1, PFN1, PGK1, PKM, PPIA

NR, HSP90AA1, HSPA1B, HSPA8, HSPA9, HSPD1, ICAM2, ILK, INPP5D, ITGA2B, ITGA6, ITGB1, ITGB3, JUP,

100A8, S100A9, SELPLG, SLC2A1, SLC2A3, SOD1, SPN, SPTBN1, SRI, STOM, TFRC, THBS1, TLN1, TNKS1BP

L, SPN, SPTBN1, SRGN, SRI, TAGLN2, TFRC, THBS1, TMX2, TUBB, VCL, VIM, XRCC5, YWHAE, YWHAG, YWH

HSPA9, HSPD1, ICAM2, ILK, INPP5D, ITGA2B, ITGA6, ITGB1, ITGB3, JUP, KANSL3, KRT1, KRT10, KRT14, KRT

L, NPM1, PATJ, PCBP1, PECAM1, PF4V1, PFN1, PKM, PLEK, PNPLA8, PPIA, PRDX1, PRDX2, PRDX6, PSMA6

'NPLA8, PPIA, PRDX1, PRDX2, PRDX6, PSMA6, PTBP1, PTGS1, PTPRJ, RAB10, RAB33B, RAC2, RAP1B, RCC

SELPLG, SLC2A1, SOD1, SPN, SPTBN1, SRGN, SRI, SYNCRIP, TFRC, THBS1, TLN1, TMEM109, TNKS1BP1, TS

LBP, LGALS3BP, LRP1, LTF, LYZ, MATR3, MMRN1, MSN, MYADM, MYH9, MYL12B, NCL, NPM1, PATJ, PC

MATR3, MMRN1, MSN, MYADM, MYH9, MYL12B, NCL, NPM1, PATJ, PCBP1, PECAM1, PF4V1, PFN1, PG

SRGN, SRI, SYNCRIP, TCP1, TFRC, THBS1, TNKS1BP1, UBE2L3, UBQLN1, VIM, XRCC5, YWHAB, YWHAE, Y\
3N1, SRI, STOM, SYNCRIP, TCP1, TFRC, THBS1, TNKS1BP1, TSPAN14, UBE2L3, UBQLN1, VIM, XRCC5, YWH

\, RTN3, RTN4, S100A8, S100A9, SELPLG, SLC2A1, SOD1, SPN, SPTBN1, SRGN, SRI, SYNCRIP, TAGLN2, TFR

GS1, PTPRJ, RAB10, RAB33B, RAC2, RHOA, RPS15, RTN3, RTN4, S100A8, S100A9, SELPLG, SLC2A1, SLC2A

TFRC, THBS1, TMEM109, TSPAN14, TUBA1C, TUBB, UBE2L3, UBQLN1, VCL, VDAC2, XRCC5, YWHAB, YW

NRNPU, HRNR, HSP90AA1, HSPA1B, HSPA8, HSPA9, HSPD1, ICAM2, ILK, INPP5D, ITGA2B, ITGA6, ITGB1, IT

OD1, SRGN, SRI, STOM, SYNCRIP, TCP1, TFRC, THBS1, TKT, TMEM109, TMX2, TSPAN14, TUBB, UBQLN1, \

:C2, RHOA, RPL11, RPL8, RPLP0, RPS15, RPS15A, RPS23, RPS3A, RPSA, S100A8, S100A9, SLC2A1, SOD1, S

.P1B, RCC2, RHOA, RPL11, RPS15, RPS15A, RTN4, S100A8, S100A9, SOD1, SPN, SPTBN1, SRI, STOM, SYNC

, KRT9, LBP, LDHA, LDHB, LGALS3BP, LRBA, LRP1, LTF, M6PR, MATR3, MSN, MYADM, MYH9, MYL6, NCL,

‹6, PSMA5, PSMA6, PTBP1, PTPRJ, RAB1B, RHOA, RPL11, RPL8, RPLP0, RPS15, RPS15A, RPS23, RPS3A, RF

‹ANSL3, KPRP, KRT1, KRT10, KRT14, KRT15, KRT16, KRT2, KRT5, KRT6A, KRT6B, KRT78, KRT9, LBP, LDHA,

00A9, SF3A3, SLC2A1, SOD1, SPTBN1, SRGN, TCP1, TFRC, THBS1, TLN1, TNKS1BP1, TPM4, TUBA1C, TUBA

MYH9, NANS, NASP, NCL, NPM1, OSBPL2, PCBP1, PCYOX1, PECAM1, PFN1, PGK1, PKM, PLEK, PNPLA8, P

00A9, SF3A3, SLC2A1, SOD1, SPTBN1, SRGN, TCP1, TFRC, THBS1, TLN1, TNKS1BP1, TPM4, TUBA1C, TUBA
IRNPU, HRNR, HSP90AA1, HSPA1B, HSPA8, HSPA9, HSPD1, ICAM2, ILK, INPP5D, ITGA2B, ITGA6, ITGB1, IT
IRNPU, HRNR, HSP90AA1, HSPA1B, HSPA8, HSPA9, HSPD1, ICAM2, ILK, INPP5D, ITGA2B, ITGA6, ITGB1, IT

HSPA9, HSPD1, ICAM2, ILK, INPP5D, ITGA2B, ITGA6, ITGB1, ITGB3, JUP, KPRP, KRT1, KRT10, KRT14, KRT

RNPU, HRNR, HSP90AA1, HSPA1B, HSPA8, HSPA9, HSPD1, ICAM2, ILK, INPP5D, ITGA2B, ITGA6, ITGB1, I

NPM1, OSBPL2, PCBP1, PCYOX1, PECAM1, PFN1, PGK1, PKM, PLEK, PNPLA8, PPIA, PRDX2, PRDX6, PSMA

P, KPRP, KRT1, KRT10, KRT14, KRT15, KRT16, KRT17, KRT2, KRT5, KRT6A, KRT6B, KRT78, KRT9, LDHA, LDH

MA5, PSMA6, PTBP1, PTPRJ, RAB1B, RHOA, RPL11, RPL8, RPLP0, RPS15, RPS15A, RPS23, RPS3A, RPSA, RT

L, SPTBN1, SYNCRIP, TCP1, TFRC, THBS1, TNKS1BP1, TPM4, TUBB, UBE2L3, UBQLN1, VCL, VDAC2, VIM, W

PPIA, PRDX2, PRDX6, PSMA5, PSMA6, PTBP1, PTGS1, PTPRJ, RAB1B, RHOA, RPL11, RPL8, RPLP0, RPS15, R

'1, PTGS1, PTPRJ, QSOX1, RAB1B, RAB33B, RAC2, RHOA, RPL11, RPL8, RPLP0, RPS15, RPS15A, RPS23, RP

33, JUP, KANSL3, KPRP, KRT1, KRT10, KRT14, KRT15, KRT16, KRT17, KRT2, KRT5, KRT6A, KRT6B, KRT78, K

SYNCRIP, TCP1, TFRC, TKT, TTLL12, TUBA1C, TUBA4A, TUBB, TUBB1, UBE2L3, VDAC2, VIM, WBP11, XRC

SPTBN1, SYNCRIP, TCP1, TFRC, TKT, TTLL12, TUBA1C, TUBA4A, TUBB, TUBB1, UBE2L3, VDAC2, VIM, WB

2, TCP1, TLN1, TNKS1BP1, TPM4, TTLL12, TUBA1C, TUBA4A, TUBB, TUBB1, VCL, VDAC2, VIM, XRCC5
2, TCP1, TLN1, TNKS1BP1, TPM4, TTLL12, TUBA1C, TUBA4A, TUBB, TUBB1, VCL, VDAC2, VIM, XRCC5

PP5D, ITGA2B, ITGB1, ITGB3, JUP, KANSL3, KPRP, KRT1, KRT10, KRT14, KRT15, KRT16, KRT17, KRT2, KRT5
.6, KRT17, KRT2, KRT5, KRT6A, KRT6B, KRT78, KRT9, LDHA, LDHB, LGALS3BP, LRBA, LRP1, LTF, LYZ, M6PR

DHA, LDHB, LGALS3BP, LRBA, LRP1, LTF, LYZ, M6PR, MATR3, MMRN1, MSN, MYH9, NASP, NCL, NPM1, C

GRL3, SLC2A1, SOD1, SPN, SPTBN1, SRI, SYNCRIP, TFRC, TKT, TMEM109, TNKS1BP1, TTLL12, TUBA1C, TU

RAB10, RAB1B, RAB27B, RAC2, RAP1B, RHOA, RPL11, RPLP0, RPS15A, RPS3A, RPSA, RTN3, S100A8, S100

, PSMA6, PTBP1, PTGS1, PTPRJ, QSOX1, RAB10, RAB1B, RAB27B, RAC2, RAP1B, RHOA, RPL11, RPLP0, RPS

2A3, SOD1, SPN, SPTBN1, SRI, STOM, TFRC, THBS1, TLN1, TNKS1BP1, TPM4, TSPAN14, UBQLN1, VCL, VI

P1B, RHOA, RPL11, RPLP0, RPS15A, RPS3A, RPSA, S100A8, S100A9, SBSN, SH3BGRL3, SLC2A1, SLC2A3,
P1B, RHOA, RPL11, RPLP0, RPS15A, RPS3A, RPSA, S100A8, S100A9, SBSN, SH3BGRL3, SLC2A1, SLC2A3,
P1B, RHOA, RPL11, RPLP0, RPS15A, RPS3A, RPSA, S100A8, S100A9, SBSN, SH3BGRL3, SLC2A1, SLC2A3,
P1B, RHOA, RPL11, RPLP0, RPS15A, RPS3A, RPSA, S100A8, S100A9, SBSN, SH3BGRL3, SLC2A1, SLC2A3,

, PRDX1, PRDX2, PRDX6, PSMA5, PSMA6, PTBP1, PTGS1, PTPRJ, QSOX1, RAB10, RAB1B, RAB27B, RAB33

KANSL3, KRT1, KRT10, KRT14, KRT15, KRT16, KRT17, KRT2, KRT5, KRT6A, KRT6B, KRT78, KRT9, LBP, LDH

T15, KRT16, KRT17, KRT2, KRT5, KRT6A, KRT6B, KRT78, KRT9, LBP, LDHA, LDHB, LGALS3BP, LRBA, LRP1, L

;, PTBP1, PTGS1, PTPRJ, QSOX1, RAB10, RAB1B, RAB27B, RAB33B, RAC2, RAP1B, RCC2, RHOA, RPL11, RP

2, RHOA, RPL11, RPLP0, RPS15, RPS15A, RTN4, S100A8, S100A9, SELPLG, SLC2A1, SOD1, SPN, SPTBN1, SI

SPAN14, TTLL12, UBE2L3, UBQLN1, VDAC2, VIM, XRCC5, YWHAB, YWHAE, YWHAG, YWHAH, YWHAZ

BP1, PECAM1, PF4V1, PFN1, PGK1, PKM, PLEK, PNPLA8, PPIA, PRDX1, PRDX2, PRDX6, PSMA6, PTBP1, PT

K1, PKM, PLEK, PNPLA8, PPIA, PRDX1, PRDX2, PRDX6, PSMA6, PTBP1, PTGS1, PTPRJ, QSOX1, RAB10, RA

3, SOD1, SPN, SPTBN1, SRGN, SRI, SYNCRIP, TFRC, THBS1, TLN1, TMX2, TPM4, VCL, VIM, XRCC5, YWHAE

TGB3, JUP, KPRP, KRT1, KRT10, KRT14, KRT15, KRT16, KRT17, KRT2, KRT5, KRT6A, KRT6B, KRT78, LBP, LD

;PTBN1, SRI, SYNCRIP, TAGLN2, TCP1, TKT, TLN1, TNKS1BP1, TPM4, TUBA4A, TUBB, UBE2L3, UBQLN1, V(

:RIP, TCP1, TFRC, THBS1, TNKS1BP1, TSPAN14, UBE2L3, UBQLN1, VIM, XRCC5, YWHAE, YWHAH

, NPM1, OSBPL2, PATJ, PCBP1, PCYOX1, PECAM1, PFN1, PGK1, PLEK, PNPLA8, PPIA, PRDX6, PTBP1, PTGS

PSA, RTN3, RTN4, S100A8, S100A9, SF3A3, SOD1, SPN, SPTBN1, SRGN, SRI, SYNCRIP, TCP1, TFRC, THBS1,

LDHB, LGALS3BP, LRBA, LRP1, LTF, LYZ, M6PR, MATR3, MMRN1, MSN, MYH9, MYL12B, MYL6, NANS, NA

PIA, PRDX1, PRDX2, PRDX6, PSMA5, PSMA6, PTBP1, PTGS1, PTPRJ, QSOX1, RAB1B, RAB33B, RAC2, RHO/

TGB3, JUP, KANSL3, KPRP, KRT1, KRT10, KRT14, KRT15, KRT16, KRT17, KRT2, KRT5, KRT6A, KRT6B, KRT7ε

TGB3, JUP, KANSL3, KPRP, KRT1, KRT10, KRT14, KRT15, KRT16, KRT17, KRT2, KRT5, KRT6A, KRT6B, KRT7ε

15, KRT16, KRT17, KRT2, KRT5, KRT6A, KRT6B, KRT78, LBP, LDHA, LDHB, LGALS3BP, LRBA, LRP1, LTF, LYZ

ΓGB3, JUP, KPRP, KRT1, KRT10, KRT14, KRT15, KRT16, KRT17, KRT2, KRT5, KRT6A, KRT6B, KRT78, KRT9, L

\5, PSMA6, PTBP1, PTGS1, PTPRJ, RAB1B, RHOA, RPL11, RPL8, RPLP0, RPS15, RPS15A, RPS23, RPS3A, RPS

HB, LGALS3BP, LRBA, LRP1, LTF, LYZ, M6PR, MMRN1, MSN, MYADM, MYH9, MYL12B, MYL6, NANS, NASP

N3, RTN4, S100A8, S100A9, SF3A3, SOD1, SPTBN1, SRGN, SRI, SYNCRIP, TCP1, TFRC, THBS1, TKT, TNKS1E

RPS15A, RPS23, RPS3A, RPSA, RTN3, RTN4, S100A8, S100A9, SACM1L, SF3A3, SLC2A1, SLC2A3, SOD1, SP

S3A, RPSA, RTN3, RTN4, S100A8, S100A9, SACM1L, SF3A3, SLC2A1, SLC2A3, SOD1, SPTBN1, SRI, SYNCRIP

:RT9, LBP, LDHA, LDHB, LGALS3BP, LRBA, LRP1, LTF, LYZ, M6PR, MATR3, MMRN1, MSN, MYADM, MYH1(

;, KRT6A, KRT6B, KRT78, KRT9, LDHA, LDHB, LGALS3BP, LRBA, LRP1, LTF, LYZ, M6PR, MATR3, MMRN1, M
:, MATR3, MMRN1, MSN, MYADM, MYH16, MYH9, MYL12B, MYL6, NASP, NCL, NPM1, OSBPL2, PATJ, PC

)SBPL2, PCBP1, PCYOX1, PECAM1, PFN1, PKM, PNPLA8, PPIA, PRDX1, PRDX6, PSMA5, PSMA6, PTBP1, PT

A9, SBSN, SH3BGRL3, SLC2A1, SLC2A3, SOD1, SPN, SPTBN1, SRGN, SRI, STOM, TAGLN2, TCP1, TFRC, THB

;15A, RPS3A, RPSA, RTN3, S100A8, S100A9, SBSN, SH3BGRL3, SLC2A1, SLC2A3, SOD1, SPN, SPTBN1, SRG

SOD1, SPN, SPTBN1, SRI, STOM, TAGLN2, TCP1, TFRC, THBS1, TKT, TLN1, TMEM109, TPM4, TUBA4A, TUB
SOD1, SPN, SPTBN1, SRI, STOM, TAGLN2, TCP1, TFRC, THBS1, TKT, TLN1, TMEM109, TPM4, TUBA4A, TUB
SOD1, SPN, SPTBN1, SRI, STOM, TAGLN2, TCP1, TFRC, THBS1, TKT, TLN1, TMEM109, TPM4, TUBA4A, TUB
SOD1, SPN, SPTBN1, SRI, STOM, TAGLN2, TCP1, TFRC, THBS1, TKT, TLN1, TMEM109, TPM4, TUBA4A, TUB

B, RAC2, RAP1B, RCC2, RHOA, RPL11, RPLP0, RPS15A, RPS3A, RPSA, S100A8, S100A9, SBSN, SH3BGRL3, S

A, LDHB, LGALS3BP, LRBA, LRP1, LTF, LYZ, M6PR, MATR3, MMRN1, MSN, MYADM, MYH9, MYL12B, MYL

.TF, LYZ, M6PR, MMRN1, MSN, MYADM, MYH9, MYL6, NANS, NASP, NCL, NPM1, OSBPL2, PATJ, PCBP1, F

S15, RPS15A, RPS3A, RTN3, RTN4, S100A8, S100A9, SOD1, SPN, SPTBN1, SRGN, SRI, STOM, SYNCRIP, TCI

RGN, SRI, SYNCRIP, TFRC, THBS1, TLN1, TMEM109, TNKS1BP1, TSPAN14, TTLL12, TUBB, UBE2L3, UBQLN

'GS1, PTPRJ, QSOX1, RAB10, RAB1B, RAB27B, RAB33B, RAC2, RAP1B, RCC2, RHOA, RPL11, RPLP0, RPS15,

B1B, RAB27B, RAB33B, RAC2, RAP1B, RCC2, RHOA, RPL11, RPS15, RPS15A, RPS3A, RTN3, RTN4, S100A8,

HA, LDHB, LGALS3BP, LRBA, LRP1, LTF, LYZ, M6PR, MATR3, MMRN1, MSN, MYADM, MYH9, MYL12B, M

;1, PTPRJ, QSOX1, RAB10, RAB1B, RAB27B, RAB33B, RAC2, RAP1B, RCC2, RHOA, RPL11, RPL8, RPLP0, RP

TNKS1BP1, TSPAN14, TTLL12, UBE2L3, UBQLN1, VIM, WBP11, XRCC5, YWHAB, YWHAE, YWHAG, YWHAI

ASP, NCL, NPM1, OSBPL2, PATJ, PCBP1, PCYOX1, PECAM1, PFN1, PGK1, PKM, PNPLA8, PPIA, PRDX1, PRD

A, RPL11, RPL8, RPLP0, RPS15, RPS15A, RPS23, RPS3A, RPSA, RTN3, RTN4, S100A8, S100A9, SACM1L, SF3

3, KRT9, LBP, LDHA, LDHB, LGALS3BP, LRBA, LRP1, LTF, LYZ, M6PR, MATR3, MMRN1, MSN, MYADM, MYH
3, KRT9, LBP, LDHA, LDHB, LGALS3BP, LRBA, LRP1, LTF, LYZ, M6PR, MATR3, MMRN1, MSN, MYADM, MYH

, M6PR, MATR3, MMRN1, MSN, MYADM, MYH9, MYL12B, MYL6, NASP, NCL, NPM1, OSBPL2, PATJ, PCBI

.BP, LDHA, LDHB, LGALS3BP, LRBA, LRP1, LTF, LYZ, M6PR, MATR3, MMRN1, MSN, MYADM, MYH9, MYL1

;A, RTN3, RTN4, S100A8, S100A9, SACM1L, SF3A3, SLC2A1, SLC2A3, SOD1, SPN, SPTBN1, SRGN, SRI, SYN

P, NCL, NPM1, OSBPL2, PATJ, PCBP1, PCYOX1, PECAM1, PFN1, PGK1, PKM, PLEK, PNPLA8, PPIA, PRDX1,

BP1, TSPAN14, TTLL12, UBE2L3, UBQLN1, VIM, WBP11, XRCC5, YWHAB, YWHAE, YWHAG, YWHAH, YWH

TBN1, SRGN, SRI, SYNCRIP, TCP1, TFRC, THBS1, TKT, TNKS1BP1, TSPAN14, TTLL12, UBE2L3, UBQLN1, VIM

›, TCP1, TFRC, THBS1, TKT, TNKS1BP1, UBE2L3, UBQLN1, VIM, WBP11, XRCC5, YWHAB, YWHAE, YWHAG

5, MYH9, MYL12B, MYL6, NANS, NASP, NCL, NPM1, OSBPL2, PATJ, PCBP1, PCYOX1, PECAM1, PFN1, PGK1

ISN, MYADM, MYH16, MYH9, MYL12B, MYL6, NANS, NASP, NCL, NPM1, OSBPL2, PATJ, PCBP1, PCYOX1,
BP1, PCYOX1, PECAM1, PFN1, PKM, PNPLA8, PPIA, PRDX1, PRDX6, PSMA5, PSMA6, PTBP1, PTGS1, PTPR

TGS1, PTPRJ, QSOX1, RAB10, RAB1B, RAB27B, RAB33B, RAC2, RAP1B, RCC2, RHOA, RPL11, RPLP0, RPS15

S1, TKT, TLN1, TMEM109, TPM4, TUBA4A, TUBB, TUBB1, VCL, VIM, YWHAB, YWHAE, YWHAG, YWHAH,

N, SRI, STOM, TAGLN2, TCP1, TFRC, THBS1, TKT, TLN1, TMEM109, TPM4, TUBA4A, TUBB, TUBB1, VCL, V

SLC2A1, SLC2A3, SOD1, SPN, SPTBN1, SRGN, SRI, STOM, TAGLN2, TCP1, TFRC, THBS1, TKT, TLN1, TMEM1

.6, NANS, NASP, NCL, NPM1, OSBPL2, PATJ, PCBP1, PCYOX1, PECAM1, PF4V1, PFN1, PGK1, PKM, PLEK, P

PCYOX1, PECAM1, PF4V1, PFN1, PGK1, PKM, PLEK, PNPLA8, PPIA, PRDX1, PRDX2, PRDX6, PSMA5, PSMA

P1, TFRC, THBS1, TLN1, TMEM109, TNKS1BP1, TSPAN14, TTLL12, TUBB, UBE2L3, UBQLN1, VCL, VDAC2, V

RPS15A, RPS3A, RTN3, RTN4, S100A8, S100A9, SLC2A1, SOD1, SPN, SPTBN1, SRGN, SRI, STOM, SYNCRIP

S100A9, SOD1, SPN, SPTBN1, SRGN, SRI, STOM, SYNCRIP, TCP1, TFRC, THBS1, TKT, TLN1, TMEM109, TN

YL6, NASP, NCL, NPM1, OSBPL2, PATJ, PCBP1, PCYOX1, PECAM1, PF4V1, PFN1, PGK1, PKM, PLEK, PNPLA

S15, RPS15A, RPS23, RPSA, RTN3, RTN4, S100A8, S100A9, SACM1L, SELPLG, SLC2A1, SLC2A3, SOD1, SPN

›X2, PRDX6, PSMA5, PSMA6, PTBP1, PTGS1, PTPRJ, QSOX1, RAB10, RAB1B, RAB27B, RAB33B, RAC2, RAP

3A3, SLC2A1, SLC2A3, SOD1, SPN, SPTBN1, SRGN, SRI, SYNCRIP, TCP1, TFRC, THBS1, TKT, TNKS1BP1, TSP/

H16, MYH9, MYL12B, MYL6, NANS, NASP, NCL, NPM1, OSBPL2, PATJ, PCBP1, PCYOX1, PECAM1, PF4V1, F
H16, MYH9, MYL12B, MYL6, NANS, NASP, NCL, NPM1, OSBPL2, PATJ, PCBP1, PCYOX1, PECAM1, PF4V1, F

P1, PCYOX1, PECAM1, PF4V1, PFN1, PGK1, PKM, PLEK, PPIA, PRDX1, PRDX2, PRDX6, PSMA5, PSMA6, PTI

.2B, MYL6, NANS, NASP, NCL, NPM1, OSBPL2, PATJ, PCBP1, PCYOX1, PECAM1, PF4V1, PFN1, PGK1, PKM,

CRIP, TCP1, TFRC, THBS1, TKT, TNKS1BP1, TSPAN14, TTLL12, UBE2L3, UBQLN1, VIM, WBP11, XRCC5, YW

PRDX2, PRDX6, PSMA5, PSMA6, PTGS1, PTPRJ, QSOX1, RAB10, RAB1B, RAB27B, RAB33B, RAC2, RAP1B, I

1, PKM, PNPLA8, PPIA, PRDX1, PRDX2, PRDX6, PSMA5, PSMA6, PTBP1, PTGS1, PTPRJ, QSOX1, RAB10, RA

PECAM1, PFN1, PGK1, PKM, PLEK, PNPLA8, PPIA, PRDX1, PRDX2, PRDX6, PSMA5, PSMA6, PTBP1, PTGS1
1J, QSOX1, RAB10, RAB1B, RAB27B, RAB33B, RAC2, RAP1B, RCC2, RHOA, RPL11, RPL8, RPLP0, RPS15, RPS

, RPS15A, RPS23, RPS3A, RPSA, RTN3, RTN4, S100A8, S100A9, SACM1L, SF3A3, SH3BGRL3, SLC2A1, SLC2

'NPLA8, PPIA, P

6, PTBP1, PTGS

VIM, XRCC5

8, PPIA, PRDX1

1B, RCC2, RHOA

PFN1, PGK1, PKM
PFN1, PGK1, PKM

BP1, PTGS1, PT

, PLEK, PNPLA8,

/HAB, YWHAE,

RCC2, RHOA, R

\B1B, RAB27B, RA

., PTPRJ, QSOX1, R
;15A, RPS23, RP

A3, SOD1, SPN

Table 8. REACTOME pathways associated with Differentially Expressed Proteins

REACTOME ID	REACTOME pathway	arbitrary category	# all protei
R-HSA-446728	Cell junction organization	other	115
R-HSA-74160	Gene expression (Transcription)	cancer-related	1547
R-HSA-5625740	RHO GTPases activate PKNs	cancer-related	94
R-HSA-1500931	Cell-Cell communication	immune-related	153
R-HSA-446353	Cell-extracellular matrix interactions	cancer-related	18
R-HSA-430116	GP1b-IX-V activation signalling	cancer-related	12
R-HSA-195258	RHO GTPase Effectors	cancer-related	326
R-HSA-168256	Immune System	immune-related	2068
R-HSA-373755	Semaphorin interactions	other	65
R-HSA-76009	Platelet Aggregation (Plug Formation)	cancer-related	39
R-HSA-5627123	RHO GTPases activate PAKs	cancer-related	24
R-HSA-449147	Signaling by Interleukins	immune-related	473
R-HSA-162582	Signal Transduction	cancer-related	2599
R-HSA-1280218	Adaptive Immune System	immune-related	770
R-HSA-212436	Generic Transcription Pathway	other	1253
R-HSA-73857	RNA Polymerase II Transcription	other	1377
R-HSA-5628897	TP53 Regulates Metabolic Genes	cancer-related	87
R-HSA-9006934	Signaling by Receptor Tyrosine Kinases	cancer-related	532
R-HSA-5674135	MAP2K and MAPK activation	cancer-related	40
R-HSA-6802948	Signaling by high-kinase activity BRAF mutants	cancer-related	36
R-HSA-6785807	Interleukin-4 and Interleukin-13 signaling	immune-related	108
R-HSA-421270	Cell-cell junction organization	cancer-related	88
R-HSA-9614085	FOXO-mediated transcription	cancer-related	65
R-HSA-9614399	Regulation of localization of FOXO transcription	cancer-related	11
R-HSA-392517	Rap1 signalling	immune-related	16
R-HSA-140877	Formation of Fibrin Clot (Clotting Cascade)	other	39
R-HSA-1280215	Cytokine Signaling in Immune system	immune-related	792
R-HSA-109606	Intrinsic Pathway for Apoptosis	cancer-related	55
R-HSA-111447	Activation of BAD and translocation to mitochon	cancer-related	15
R-HSA-114452	Activation of BH3-only proteins	cancer-related	30
R-HSA-9755779	SARS-CoV-2 targets host intracellular signalling a	other	12
R-HSA-75035	Chk1/Chk2(Cds1) mediated inactivation of Cyclir	cancer-related	13
R-HSA-9735871	SARS-CoV-1 targets host intracellular signalling a	other	15
R-HSA-194315	Signaling by Rho GTPases	cancer-related	706
R-HSA-9716542	Signaling by Rho GTPases, Miro GTPases and RH	cancer-related	722
R-HSA-1640170	Cell Cycle	cancer-related	691
R-HSA-1474244	Extracellular matrix organization	cancer-related	300
R-HSA-1445148	Translocation of SLC2A4 (GLUT4) to the plasma r	cancer-related	72
R-HSA-109582	Hemostasis	cancer-related	623
R-HSA-2682334	EPH-Ephrin signaling	cancer-related	92
R-HSA-6802952	Signaling by BRAF and RAF1 fusions	cancer-related	67
R-HSA-69481	G2/M Checkpoints	other	167
R-HSA-9656223	Signaling by RAF1 mutants	cancer-related	43

R-HSA-5683057	MAPK family signaling cascades	cancer-related	325
R-HSA-168249	Innate Immune System	immune-related	1076
R-HSA-2029480	Fcgamma receptor (FCGR) dependent phagocytc	cancer-related	86
R-HSA-2029482	Regulation of actin dynamics for phagocytic cup	cancer-related	61
R-HSA-2132295	MHC class II antigen presentation	immune-related	123
R-HSA-69473	G2/M DNA damage checkpoint	cancer-related	94
R-HSA-109581	Apoptosis	cancer-related	182
R-HSA-3700989	Transcriptional Regulation by TP53	cancer-related	362
R-HSA-5357801	Programmed Cell Death	cancer-related	217
R-HSA-9664407	Parasite infection	other	62
R-HSA-9664417	Leishmania phagocytosis	other	62
R-HSA-9664422	FCGR3A-mediated phagocytosis	other	62
R-HSA-166016	Toll Like Receptor 4 (TLR4) Cascade	immune-related	147
R-HSA-168898	Toll-like Receptor Cascades	immune-related	170
R-HSA-212165	Epigenetic regulation of gene expression	other	189
R-HSA-9013148	CDC42 GTPase cycle	cancer-related	155
R-HSA-354192	Integrin signaling	cancer-related	27
R-HSA-3247509	Chromatin modifying enzymes	other	270
R-HSA-4839726	Chromatin organization	other	270
R-HSA-5689603	UCH proteinases	cancer-related	102
R-HSA-9018519	Estrogen-dependent gene expression	other	149
R-HSA-5625900	RHO GTPases activate CIT	cancer-related	20
R-HSA-9013406	RHOQ GTPase cycle	cancer-related	59
R-HSA-9013409	RHOJ GTPase cycle	cancer-related	55
R-HSA-5686938	Regulation of TLR by endogenous ligand	immune-related	21
R-HSA-3928663	EPHA-mediated growth cone collapse	cancer-related	30
R-HSA-400685	Sema4D in semaphorin signaling	cancer-related	25
R-HSA-416572	Sema4D induced cell migration and growth-cone	cancer-related	21
R-HSA-3214847	HATs acetylate histones	cancer-related	141
R-HSA-75892	Platelet Adhesion to exposed collagen	cancer-related	16
R-HSA-210991	Basigin interactions	cancer-related	25
R-HSA-372708	p130Cas linkage to MAPK signaling for integrins	cancer-related	15
R-HSA-354194	GRB2:SOS provides linkage to MAPK signaling fo	cancer-related	15
R-HSA-1643685	Disease	other	2074
R-HSA-418990	Adherens junctions interactions	cancer-related	57
R-HSA-2022377	Metabolism of Angiotensinogen to Angiotensins	other	18
R-HSA-2980736	Peptide hormone metabolism	other	89
R-HSA-3000157	Laminin interactions	cancer-related	30
R-HSA-8875878	MET promotes cell motility	cancer-related	41
R-HSA-1474290	Collagen formation	cancer-related	90
R-HSA-9671793	Diseases of hemostasis	other	19
R-HSA-9823587	Defects of platelet adhesion to exposed collager	other	8
R-HSA-190873	Gap junction degradation	other	12
R-HSA-196025	Formation of annular gap junctions	other	11
R-HSA-2022090	Assembly of collagen fibrils and other multimeri	cancer-related	61
R-HSA-9845620	Enhanced binding of GP1BA variant to VWF mult	other	7
R-HSA-9846298	Defective binding of VWF variant to GPIb:IX:V	other	7

R-HSA-140837	Intrinsic Pathway of Fibrin Clot Formation	other	23
R-HSA-9651496	Defects of contact activation system (CAS) and k	other	16
R-HSA-9668250	Defective factor IX causes hemophilia B	other	9
R-HSA-9673221	Defective F9 activation	other	6
R-HSA-446343	Localization of the PINCH-ILK-PARVIN complex to	cancer-related	4
R-HSA-6802957	Oncogenic MAPK signaling	cancer-related	84
R-HSA-69620	Cell Cycle Checkpoints	cancer-related	292
R-HSA-6802946	Signaling by moderate kinase activity BRAF muta	cancer-related	47
R-HSA-6802949	Signaling by RAS mutants	cancer-related	47
R-HSA-6802955	Paradoxical activation of RAF signaling by kinase	cancer-related	47
R-HSA-9649948	Signaling downstream of RAS mutants	cancer-related	47
R-HSA-216083	Integrin cell surface interactions	cancer-related	85
R-HSA-5673001	RAF/MAP kinase cascade	cancer-related	280
R-HSA-5684996	MAPK1/MAPK3 signaling	cancer-related	286
R-HSA-8878171	Transcriptional regulation by RUNX1	cancer-related	236
R-HSA-9679506	SARS-CoV Infections	other	417
R-HSA-5653656	Vesicle-mediated transport	other	673
R-HSA-69278	Cell Cycle, Mitotic	other	560
R-HSA-68886	M Phase	other	416
R-HSA-447115	Interleukin-12 family signaling	immune-related	57
R-HSA-202733	Cell surface interactions at the vascular wall	immune-related	138
R-HSA-76002	Platelet activation, signaling and aggregation	cancer-related	263
R-HSA-9694516	SARS-CoV-2 Infection	other	300
R-HSA-9645723	Diseases of programmed cell death	cancer-related	108
R-HSA-983231	Factors involved in megakaryocyte development	cancer-related	168
R-HSA-201681	TCF dependent signaling in response to WNT	cancer-related	232
R-HSA-157858	Gap junction trafficking and regulation	other	49
R-HSA-190828	Gap junction trafficking	other	47
R-HSA-8936459	RUNX1 regulates genes involved in megakaryocy	cancer-related	94
R-HSA-5627117	RHO GTPases Activate ROCKs	cancer-related	20
R-HSA-194138	Signaling by VEGF	cancer-related	108
R-HSA-4420097	VEGFA-VEGFR2 Pathway	cancer-related	99
R-HSA-1236974	ER-Phagosome pathway	immune-related	91
R-HSA-9678108	SARS-CoV-1 Infection	other	144
R-HSA-5663202	Diseases of signal transduction by growth factor	cancer-related	458
R-HSA-5218920	VEGFR2 mediated vascular permeability	cancer-related	29
R-HSA-5663213	RHO GTPases Activate WASPs and WAVEs	cancer-related	36
R-HSA-8862803	Deregulated CDK5 triggers multiple neurodegen	other	22
R-HSA-8863678	Neurodegenerative Diseases	other	22
R-HSA-9734009	Defective Intrinsic Pathway for Apoptosis	cancer-related	25
R-HSA-6806834	Signaling by MET	cancer-related	79
R-HSA-68875	Mitotic Prophase	other	141
R-HSA-9679191	Potential therapeutics for SARS	other	99
R-HSA-2672351	Stimuli-sensing channels	other	109
R-HSA-1474165	Reproduction	other	154
R-HSA-166520	Signaling by NTRKs	cancer-related	134
R-HSA-187037	Signaling by NTRK1 (TRKA)	cancer-related	115

R-HSA-196849	Metabolism of water-soluble vitamins and cofac	other	127
R-HSA-5687128	MAPK6/MAPK4 signaling	cancer-related	89
R-HSA-9604323	Negative regulation of NOTCH4 signaling	cancer-related	54
R-HSA-3928665	EPH-ephrin mediated repulsion of cells	cancer-related	51
R-HSA-211000	Gene Silencing by RNA	other	137
R-HSA-5250913	Positive epigenetic regulation of rRNA expressio	other	106
R-HSA-5250924	B-WICH complex positively regulates rRNA expre	other	90
R-HSA-9013407	RHOH GTPase cycle	cancer-related	38
R-HSA-9013408	RHOG GTPase cycle	cancer-related	74
R-HSA-166058	MyD88:MAL(TIRAP) cascade initiated on plasma	immune-related	114
R-HSA-168179	Toll Like Receptor TLR1:TLR2 Cascade	immune-related	117
R-HSA-168188	Toll Like Receptor TLR6:TLR2 Cascade	immune-related	114
R-HSA-181438	Toll Like Receptor 2 (TLR2) Cascade	immune-related	117
R-HSA-5260271	Diseases of Immune System	immune-related	33
R-HSA-5602358	Diseases associated with the TLR signaling casca	immune-related	33
R-HSA-5602498	MyD88 deficiency (TLR2/4)	immune-related	18
R-HSA-5603041	IRAK4 deficiency (TLR2/4)	immune-related	19
R-HSA-2173782	Binding and Uptake of Ligands by Scavenger Rec	cancer-related	42
R-HSA-196836	Vitamin C (ascorbate) metabolism	other	8
R-HSA-3000178	ECM proteoglycans	cancer-related	76
R-HSA-445144	Signal transduction by L1	cancer-related	21
R-HSA-5336415	Uptake and function of diphtheria toxin	other	6
R-HSA-6798695	Neutrophil degranulation	immune-related	480
R-HSA-422475	Axon guidance	other	553
R-HSA-9675108	Nervous system development	other	578
R-HSA-199991	Membrane Trafficking	other	634
R-HSA-6803157	Antimicrobial peptides	other	98
R-HSA-9609646	HCMV Infection	other	290
R-HSA-162909	Host Interactions of HIV factors	other	137
R-HSA-196854	Metabolism of vitamins and cofactors	other	194
R-HSA-9609690	HCMV Early Events	other	235
R-HSA-450531	Regulation of mRNA stability by proteins that bi	other	88
R-HSA-8852276	The role of GTSE1 in G2/M progression after G2	cancer-related	77
R-HSA-437239	Recycling pathway of L1	other	47
R-HSA-5626467	RHO GTPases activate IQGAPs	cancer-related	32
R-HSA-9824446	Viral Infection Pathways	other	985
R-HSA-5663220	RHO GTPases Activate Formins	cancer-related	140
R-HSA-453274	Mitotic G2-G2/M phases	other	199
R-HSA-69275	G2/M Transition	other	197
R-HSA-1236975	Antigen processing-Cross presentation	immune-related	106
R-HSA-9705683	SARS-CoV-2-host interactions	other	204
R-HSA-5663205	Infectious disease	other	1273
R-HSA-373760	L1CAM interactions	cancer-related	119
R-HSA-983169	Class I MHC mediated antigen processing & pres	immune-related	382
R-HSA-8950505	Gene and protein expression by JAK-STAT signali	immune-related	38
R-HSA-9020591	Interleukin-12 signaling	immune-related	47
R-HSA-1266738	Developmental Biology	cancer-related	1271

R-HSA-525793	Myogenesis	other	30
R-HSA-388841	Costimulation by the CD28 family	immune-related	69
R-HSA-389356	CD28 co-stimulation	immune-related	33
R-HSA-8863795	Downregulation of ERBB2 signaling	cancer-related	29
R-HSA-1989781	PPARA activates gene expression	other	117
R-HSA-381340	Transcriptional regulation of white adipocyte dif	other	84
R-HSA-400206	Regulation of lipid metabolism by PPARalpha	other	119
R-HSA-168928	DDX58/IFIH1-mediated induction of interferon-a	immune-related	82
R-HSA-381038	XBP1(S) activates chaperone genes	immune-related	50
R-HSA-381070	IRE1alpha activates chaperones	immune-related	52
R-HSA-381119	Unfolded Protein Response (UPR)	immune-related	95
R-HSA-162588	Budding and maturation of HIV virion	other	36
R-HSA-162599	Late Phase of HIV Life Cycle	other	144
R-HSA-5696394	DNA Damage Recognition in GG-NER	cancer-related	38
R-HSA-5696398	Nucleotide Excision Repair	cancer-related	111
R-HSA-5696399	Global Genome Nucleotide Excision Repair (GG-I	cancer-related	84
R-HSA-9759475	Regulation of CDH11 Expression and Function	other	28
R-HSA-9759476	Regulation of Homotypic Cell-Cell Adhesion	other	31
R-HSA-9762292	Regulation of CDH11 function	other	11
R-HSA-9764260	Regulation of Expression and Function of Type II	other	31
R-HSA-9833576	CDH11 homotypic and heterotypic interactions	other	7
R-HSA-174824	Plasma lipoprotein assembly, remodeling, and cl	other	75
R-HSA-8866427	VLDLR internalisation and degradation	other	16
R-HSA-8964043	Plasma lipoprotein clearance	other	37
R-HSA-177504	Retrograde neurotrophin signalling	other	14
R-HSA-8984722	Interleukin-35 Signalling	immune-related	12
R-HSA-9020956	Interleukin-27 signaling	immune-related	11
R-HSA-416700	Other semaphorin interactions	cancer-related	19
R-HSA-9035034	RHOF GTPase cycle	cancer-related	42
R-HSA-3214858	RMTs methylate histone arginines	cancer-related	78
R-HSA-532668	N-glycan trimming in the ER and Calnexin/Calret	cancer-related	35
R-HSA-901042	Calnexin/calreticulin cycle	cancer-related	26
R-HSA-5689901	Metalloprotease DUBs	cancer-related	37
R-HSA-182971	EGFR downregulation	immune-related	31
R-HSA-74752	Signaling by Insulin receptor	other	82
R-HSA-77387	Insulin receptor recycling	other	30
R-HSA-8874081	MET activates PTK2 signaling	cancer-related	30
R-HSA-165159	MTOR signalling	cancer-related	41
R-HSA-166208	mTORC1-mediated signalling	cancer-related	24
R-HSA-399955	SEMA3A-Plexin repulsion signaling by inhibiting I	cancer-related	14
R-HSA-9830369	Kidney development	other	46
R-HSA-9772755	Formation of WDR5-containing histone-modifyir	other	41
R-HSA-9692916	SARS-CoV-1 activates/modulates innate immune	other	42
R-HSA-3000484	Scavenging by Class F Receptors	immune-related	6
R-HSA-140875	Common Pathway of Fibrin Clot Formation	cancer-related	22
R-HSA-8964038	LDL clearance	other	19
R-HSA-9683701	Translation of Structural Proteins	immune-related	30

R-HSA-9694548	Maturation of spike protein	other	37
R-HSA-9694635	Translation of Structural Proteins	immune-related	58
R-HSA-9772573	Late SARS-CoV-2 Infection Events	other	71
R-HSA-420029	Tight junction interactions	other	29
R-HSA-1227990	Signaling by ERBB2 in Cancer	cancer-related	26
R-HSA-9664565	Signaling by ERBB2 KD Mutants	cancer-related	25
R-HSA-9665686	Signaling by ERBB2 TMD/JMD mutants	cancer-related	22
R-HSA-9830674	Formation of the ureteric bud	other	21
R-HSA-9662834	CD163 mediating an anti-inflammatory response	immune-related	9
R-HSA-169893	Prolonged ERK activation events	cancer-related	14
R-HSA-170968	Frs2-mediated activation	cancer-related	12
R-HSA-187687	Signalling to ERKs	cancer-related	34
R-HSA-1566977	Fibronectin matrix formation	cancer-related	6
R-HSA-5601884	PIWI-interacting RNA (piRNA) biogenesis	other	29
R-HSA-1187000	Fertilization	other	25
R-HSA-1236382	Constitutive Signaling by Ligand-Responsive EGF	cancer-related	19
R-HSA-1643713	Signaling by EGFR in Cancer	cancer-related	25
R-HSA-5637810	Constitutive Signaling by EGFRvIII	cancer-related	15
R-HSA-5637812	Signaling by EGFRvIII in Cancer	cancer-related	15
R-HSA-5637815	Signaling by Ligand-Responsive EGFR Variants in	cancer-related	19
R-HSA-9634285	Constitutive Signaling by Overexpressed ERBB2	cancer-related	11
R-HSA-9652282	Drug-mediated inhibition of ERBB2 signaling	cancer-related	4
R-HSA-9665230	Drug resistance in ERBB2 KD mutants	cancer-related	4
R-HSA-9665233	Resistance of ERBB2 KD mutants to trastuzumab	cancer-related	4
R-HSA-9665244	Resistance of ERBB2 KD mutants to sapitinib	cancer-related	4
R-HSA-9665245	Resistance of ERBB2 KD mutants to tesevatinib	cancer-related	4
R-HSA-9665246	Resistance of ERBB2 KD mutants to neratinib	cancer-related	4
R-HSA-9665247	Resistance of ERBB2 KD mutants to osimertinib	cancer-related	4
R-HSA-9665249	Resistance of ERBB2 KD mutants to afatinib	cancer-related	4
R-HSA-9665250	Resistance of ERBB2 KD mutants to AEE788	cancer-related	4
R-HSA-9665251	Resistance of ERBB2 KD mutants to lapatinib	cancer-related	4
R-HSA-9665348	Signaling by ERBB2 ECD mutants	cancer-related	16
R-HSA-9665737	Drug resistance in ERBB2 TMD/JMD mutants	cancer-related	4
R-HSA-180689	APOBEC3G mediated resistance to HIV-1 infectio	other	10
R-HSA-5675221	Negative regulation of MAPK pathway	cancer-related	43
R-HSA-1300645	Acrosome Reaction and Sperm:Oocyte Membrai	other	5
R-HSA-450385	Butyrate Response Factor 1 (BRF1) binds and de:	cancer-related	17
R-HSA-450513	Tristetraprolin (TTP, ZFP36) binds and destabilize	cancer-related	17
R-HSA-450604	KSRP (KHSRP) binds and destabilizes mRNA	cancer-related	17
R-HSA-9022699	MECP2 regulates neuronal receptors and channe	other	17
R-HSA-373752	Netrin-1 signaling	immune-related	50
R-HSA-5099900	WNT5A-dependent internalization of FZD4	other	15
R-HSA-5140745	WNT5A-dependent internalization of FZD2, FZD5	other	13
R-HSA-168275	Entry of Influenza Virion into Host Cell via Endoc	other	11
R-HSA-173107	Binding and entry of HIV virion	other	11
R-HSA-175474	Assembly Of The HIV Virion	other	23
R-HSA-170984	ARMS-mediated activation	cancer-related	7

R-HSA-8875555	MET activates RAP1 and RAC1	cancer-related	11
R-HSA-432142	Platelet sensitization by LDL	other	17
R-HSA-3769402	Deactivation of the beta-catenin transactivating	cancer-related	42
R-HSA-9764302	Regulation of CDH19 Expression and Function	other	7
R-HSA-162585	Uncoating of the HIV Virion	other	7
R-HSA-162589	Reverse Transcription of HIV RNA	other	6
R-HSA-164516	Minus-strand DNA synthesis	other	6
R-HSA-164525	Plus-strand DNA synthesis	other	6
R-HSA-418885	DCC mediated attractive signaling	immune-related	14
R-HSA-8983711	OAS antiviral response	immune-related	10
R-HSA-434313	Intracellular metabolism of fatty acids regulates	other	3
R-HSA-9660537	Signaling by MRAS-complex mutants	cancer-related	8
R-HSA-9726840	SHOC2 M1731 mutant abolishes MRAS complex	cancer-related	8
R-HSA-9726842	Gain-of-function MRAS complexes activate RAF s	cancer-related	8
R-HSA-9683686	Maturation of spike protein	other	5
R-HSA-428543	Inactivation of CDC42 and RAC1	immune-related	8
R-HSA-389359	CD28 dependent Vav1 pathway	immune-related	12
R-HSA-162658	Golgi Cisternae Pericentriolar Stack Reorganizati	other	14
R-HSA-3000471	Scavenging by Class B Receptors	immune-related	6
R-HSA-8875513	MET interacts with TNS proteins	cancer-related	5
R-HSA-74182	Ketone body metabolism	other	10
R-HSA-77111	Synthesis of Ketone Bodies	other	8
R-HSA-192905	vRNP Assembly	other	6
R-HSA-9820962	Assembly and release of respiratory syncytial vir	other	3
R-HSA-9834752	Respiratory syncytial virus genome replication	other	4
R-HSA-166020	Transfer of LPS from LBP carrier to CD14	immune-related	2
R-HSA-168268	Virus Assembly and Release	other	11
R-HSA-168316	Assembly of Viral Components at the Budding Si	other	7
R-HSA-382551	Transport of small molecules	other	735
R-HSA-9692914	SARS-CoV-1-host interactions	other	98
R-HSA-397014	Muscle contraction	other	205
R-HSA-114608	Platelet degranulation	cancer-related	129
R-HSA-76005	Response to elevated platelet cytosolic Ca2+	cancer-related	134
R-HSA-8939211	ESR-mediated signaling	cancer-related	222
R-HSA-9006931	Signaling by Nuclear Receptors	other	298
R-HSA-9658195	Leishmania infection	other	167
R-HSA-9824443	Parasitic Infection Pathways	other	167
R-HSA-157118	Signaling by NOTCH	cancer-related	235
R-HSA-162906	HIV Infection	other	239
R-HSA-5688426	Deubiquitination	other	297
R-HSA-977225	Amyloid fiber formation	other	110
R-HSA-380320	Recruitment of NuMA to mitotic centrosomes	other	94
R-HSA-445355	Smooth Muscle Contraction	other	45
R-HSA-9013149	RAC1 GTPase cycle	cancer-related	185
R-HSA-9013423	RAC3 GTPase cycle	cancer-related	94
R-HSA-6811434	COPI-dependent Golgi-to-ER retrograde traffic	other	99
R-HSA-8856688	Golgi-to-ER retrograde transport	other	133

R-HSA-73894	DNA Repair	cancer-related	335
R-HSA-9013694	Signaling by NOTCH4	cancer-related	82
R-HSA-983712	Ion channel transport	other	186
R-HSA-9659379	Sensory processing of sound	other	76
R-HSA-9662360	Sensory processing of sound by inner hair cells o	other	69
R-HSA-9662361	Sensory processing of sound by outer hair cells c	other	54
R-HSA-1168372	Downstream signaling events of B Cell Receptor	immune-related	83
R-HSA-983705	Signaling by the B Cell Receptor (BCR)	immune-related	112
R-HSA-69002	DNA Replication Pre-Initiation	other	158
R-HSA-69306	DNA Replication	other	186
R-HSA-68867	Assembly of the pre-replicative complex	other	141
R-HSA-8939236	RUNX1 regulates transcription of genes involved	cancer-related	129
R-HSA-9013026	RHOB GTPase cycle	cancer-related	70
R-HSA-170834	Signaling by TGF-beta Receptor Complex	cancer-related	94
R-HSA-9006936	Signaling by TGFB family members	cancer-related	124
R-HSA-3000171	Non-integrin membrane-ECM interactions	cancer-related	59
R-HSA-425407	SLC-mediated transmembrane transport	other	247
R-HSA-3000170	Syndecan interactions	cancer-related	27
R-HSA-446107	Type I hemidesmosome assembly	cancer-related	11
R-HSA-195721	Signaling by WNT	cancer-related	331
R-HSA-9012999	RHO GTPase cycle	cancer-related	449
R-HSA-68877	Mitotic Prometaphase	other	204
R-HSA-9013404	RAC2 GTPase cycle	cancer-related	88
R-HSA-70171	Glycolysis	other	73
R-HSA-70326	Glucose metabolism	other	93
R-HSA-9646399	Aggrephagy	other	43
R-HSA-5339562	Uptake and actions of bacterial toxins	other	38
R-HSA-5689880	Ub-specific processing proteases	cancer-related	219
R-HSA-3928662	EPHB-mediated forward signaling	immune-related	42
R-HSA-2565942	Regulation of PLK1 Activity at G2/M Transition	other	88
R-HSA-380259	Loss of Nlp from mitotic centrosomes	other	70
R-HSA-380270	Recruitment of mitotic centrosome proteins anc	other	82
R-HSA-380284	Loss of proteins required for interphase microtu	other	70
R-HSA-380287	Centrosome maturation	other	82
R-HSA-5620912	Anchoring of the basal body to the plasma mem	other	98
R-HSA-8854518	AURKA Activation by TPX2	other	73
R-HSA-983170	Antigen Presentation: Folding, assembly and pep	immune-related	29
R-HSA-9705671	SARS-CoV-2 activates/modulates innate and ada	other	127
R-HSA-5358351	Signaling by Hedgehog	cancer-related	149
R-HSA-5610787	Hedgehog 'off' state	cancer-related	113
R-HSA-9013106	RHOC GTPase cycle	cancer-related	74
R-HSA-418346	Platelet homeostasis	cancer-related	88
R-HSA-381426	Regulation of Insulin-like Growth Factor (IGF) tra	other	125
R-HSA-8957275	Post-translational protein phosphorylation	other	108
R-HSA-1227986	Signaling by ERBB2	cancer-related	50
R-HSA-453279	Mitotic G1 phase and G1/S transition	other	149
R-HSA-6807070	PTEN Regulation	other	140

R-HSA-69202	Cyclin E associated events during G1/S transitior	other	83
R-HSA-69206	G1/S Transition	other	131
R-HSA-69242	S Phase	other	162
R-HSA-69656	Cyclin A:Cdk2-associated events at S phase entry	other	85
R-HSA-8878166	Transcriptional regulation by RUNX2	other	121
R-HSA-8948751	Regulation of PTEN stability and activity	other	69
R-HSA-157579	Telomere Maintenance	other	112
R-HSA-162587	HIV Life Cycle	other	157
R-HSA-8878159	Transcriptional regulation by RUNX3	other	97
R-HSA-195253	Degradation of beta-catenin by the destruction (	other	83
R-HSA-977606	Regulation of Complement cascade	immune-related	47
R-HSA-8951664	Neddylation	other	246
R-HSA-73884	Base Excision Repair	other	91
R-HSA-1912408	Pre-NOTCH Transcription and Translation	cancer-related	92
R-HSA-9758941	Gastrulation	other	126
R-HSA-9793380	Formation of paraxial mesoderm	other	69
R-HSA-351906	Apoptotic cleavage of cell adhesion proteins	cancer-related	11
R-HSA-2299718	Condensation of Prophase Chromosomes	other	73
R-HSA-9610379	HCMV Late Events	other	204
R-HSA-164938	Nef-mediates down modulation of cell surface re	other	22
R-HSA-164940	Nef mediated downregulation of MHC class I cor	other	11
R-HSA-164952	The role of Nef in HIV-1 replication and disease p	other	29
R-HSA-2559580	Oxidative Stress Induced Senescence	other	126
R-HSA-2559583	Cellular Senescence	other	197
R-HSA-382556	ABC-family proteins mediated transport	other	103
R-HSA-5619084	ABC transporter disorders	other	78
R-HSA-446652	Interleukin-1 family signaling	other	153
R-HSA-427413	NoRC negatively regulates rRNA expression	other	108
R-HSA-5250941	Negative epigenetic regulation of rRNA expressio	other	111
R-HSA-73864	RNA Polymerase I Transcription	other	113
R-HSA-1474228	Degradation of the extracellular matrix	cancer-related	140
R-HSA-1500620	Meiosis	other	117
R-HSA-2559586	DNA Damage/Telomere Stress Induced Senescer	other	79
R-HSA-912446	Meiotic recombination	other	85
R-HSA-204005	COPII-mediated vesicle transport	other	68
R-HSA-5658442	Regulation of RAS by GAPs	cancer-related	68
R-HSA-174143	APC/C-mediated degradation of cell cycle protei	other	87
R-HSA-176408	Regulation of APC/C activators between G1/S an	other	80
R-HSA-187577	SCF(Skp2)-mediated degradation of p27/p21	other	60
R-HSA-2559582	Senescence-Associated Secretory Phenotype (SA	other	110
R-HSA-453276	Regulation of mitotic cell cycle	other	87
R-HSA-68949	Orc1 removal from chromatin	other	71
R-HSA-69017	CDK-mediated phosphorylation and removal of (	other	72
R-HSA-69052	Switching of origins to a post-replicative state	other	91
R-HSA-69239	Synthesis of DNA	other	120
R-HSA-69563	p53-Dependent G1 DNA Damage Response	cancer-related	66
R-HSA-69580	p53-Dependent G1/S DNA damage checkpoint	cancer-related	66

R-HSA-69615	G1/S DNA Damage Checkpoints	other	68
R-HSA-9616222	Transcriptional regulation of granulopoiesis	immune-related	89
R-HSA-75815	Ubiquitin-dependent degradation of Cyclin D	other	52
R-HSA-1169091	Activation of NF-kappaB in B cells	immune-related	67
R-HSA-1234174	Cellular response to hypoxia	other	75
R-HSA-1234176	Oxygen-dependent proline hydroxylation of Hyp	other	66
R-HSA-1236978	Cross-presentation of soluble exogenous antiger	other	49
R-HSA-169911	Regulation of Apoptosis	other	53
R-HSA-174084	Autodegradation of Cdh1 by Cdh1:APC/C	other	63
R-HSA-174113	SCF-beta-TrCP mediated degradation of Emi1	other	55
R-HSA-174154	APC/C:Cdc20 mediated degradation of Securin	other	67
R-HSA-174178	APC/C:Cdh1 mediated degradation of Cdc20 anc	other	73
R-HSA-174184	Cdc20:Phospho-APC/C mediated degradation of	other	72
R-HSA-176409	APC/C:Cdc20 mediated degradation of mitotic p	other	75
R-HSA-176814	Activation of APC/C and APC/C:Cdc20 mediated	other	76
R-HSA-179419	APC:Cdc20 mediated degradation of cell cycle pı	other	73
R-HSA-180534	Vpu mediated degradation of CD4	other	53
R-HSA-180585	Vif-mediated degradation of APOBEC3G	other	55
R-HSA-211733	Regulation of activated PAK-2p34 by proteasom	other	50
R-HSA-349425	Autodegradation of the E3 ubiquitin ligase COP1	other	52
R-HSA-350562	Regulation of ornithine decarboxylase (ODC)	other	51
R-HSA-351202	Metabolism of polyamines	other	59
R-HSA-4608870	Asymmetric localization of PCP proteins	other	64
R-HSA-4641257	Degradation of AXIN	other	55
R-HSA-4641258	Degradation of DVL	other	57
R-HSA-5358346	Hedgehog ligand biogenesis	other	65
R-HSA-5362768	Hh mutants are degraded by ERAD	other	56
R-HSA-5387390	Hh mutants abrogate ligand secretion	other	59
R-HSA-5607761	Dectin-1 mediated noncanonical NF-kB signaling	immune-related	60
R-HSA-5610780	Degradation of GLI1 by the proteasome	other	60
R-HSA-5610783	Degradation of GLI2 by the proteasome	other	60
R-HSA-5610785	GLI3 is processed to GLI3R by the proteasome	other	60
R-HSA-5632684	Hedgehog 'on' state	other	85
R-HSA-5668541	TNFR2 non-canonical NF-kB pathway	immune-related	102
R-HSA-5676590	NIK-->noncanonical NF-kB signaling	immune-related	59
R-HSA-5678895	Defective CFTR causes cystic fibrosis	other	61
R-HSA-69541	Stabilization of p53	cancer-related	57
R-HSA-69601	Ubiquitin Mediated Degradation of Phosphoryla	other	52
R-HSA-69610	p53-Independent DNA Damage Response	cancer-related	52
R-HSA-69613	p53-Independent G1/S DNA damage checkpoint	cancer-related	52
R-HSA-8854050	FBXL7 down-regulates AURKA during mitotic ent	other	55
R-HSA-8939902	Regulation of RUNX2 expression and activity	cancer-related	73
R-HSA-8941858	Regulation of RUNX3 expression and activity	other	55
R-HSA-9020702	Interleukin-1 signaling	immune-related	115
R-HSA-9762114	GSK3B and BTRC:CUL1-mediated-degradation of	other	52
R-HSA-9824272	Somitogenesis	other	55
R-HSA-1442490	Collagen degradation	cancer-related	64

R-HSA-177929	Signaling by EGFR	cancer-related	53
R-HSA-9013700	NOTCH4 Activation and Transmission of Signal to	cancer-related	11
R-HSA-73772	RNA Polymerase I Promoter Escape	other	90
R-HSA-73854	RNA Polymerase I Promoter Clearance	other	110
R-HSA-8978934	Metabolism of cofactors	other	21
R-HSA-9013420	RHOU GTPase cycle	cancer-related	40
R-HSA-110328	Recognition and association of DNA glycosylase	other	60
R-HSA-110329	Cleavage of the damaged pyrimidine	other	60
R-HSA-110330	Recognition and association of DNA glycosylase	other	55
R-HSA-110331	Cleavage of the damaged purine	other	55
R-HSA-1221632	Meiotic synapsis	other	78
R-HSA-171306	Packaging Of Telomere Ends	other	51
R-HSA-201722	Formation of the beta-catenin:TCF transactivatir	other	90
R-HSA-212300	PRC2 methylates histones and DNA	other	72
R-HSA-427359	SIRT1 negatively regulates rRNA expression	other	67
R-HSA-427389	ERCC6 (CSB) and EHMT2 (G9a) positively regulat	other	76
R-HSA-5334118	DNA methylation	other	64
R-HSA-5578749	Transcriptional regulation by small RNAs	other	105
R-HSA-5617472	Activation of anterior HOX genes in hindbrain de	other	121
R-HSA-5619507	Activation of HOX genes during differentiation	cancer-related	121
R-HSA-5625886	Activated PKN1 stimulates transcription of AR (a	cancer-related	66
R-HSA-68616	Assembly of the ORC complex at the origin of re	cancer-related	68
R-HSA-73728	RNA Polymerase I Promoter Opening	other	62
R-HSA-73927	Depurination	other	55
R-HSA-73928	Depyrimidination	other	60
R-HSA-73929	Base-Excision Repair, AP Site Formation	other	62
R-HSA-9670095	Inhibition of DNA recombination at telomere	other	67
R-HSA-9710421	Defective pyroptosis	other	72
R-HSA-9821002	Chromatin modifications during the maternal to	other	70
R-HSA-2173789	TGF-beta receptor signaling activates SMADs	immune-related	47
R-HSA-3214815	HDACs deacetylate histones	other	94
R-HSA-909733	Interferon alpha/beta signaling	immune-related	77
R-HSA-399954	Sema3A PAK dependent Axon repulsion	other	16
R-HSA-2028269	Signaling by Hippo	cancer-related	20
R-HSA-202131	Metabolism of nitric oxide: NOS3 activation and	other	16
R-HSA-203615	eNOS activation	cancer-related	12
R-HSA-8852135	Protein ubiquitination	other	79
R-HSA-425393	Transport of inorganic cations/anions and aminc	other	108
R-HSA-9696264	RND3 GTPase cycle	cancer-related	42
R-HSA-1566948	Elastic fibre formation	cancer-related	44
R-HSA-2129379	Molecules associated with elastic fibres	cancer-related	37
R-HSA-8866654	E3 ubiquitin ligases ubiquitinate target proteins	other	59
R-HSA-8964616	G beta:gamma signalling through CDC42	cancer-related	20
R-HSA-9696270	RND2 GTPase cycle	cancer-related	43
R-HSA-162592	Integration of provirus	other	14
R-HSA-162594	Early Phase of HIV Life Cycle	other	21
R-HSA-189200	Cellular hexose transport	other	21

R-HSA-8986944	Transcriptional Regulation by MECP2	other	62
R-HSA-425561	Sodium/Calcium exchangers	other	13
R-HSA-9818027	NFE2L2 regulating anti-oxidant/detoxification er	other	19
R-HSA-512988	Interleukin-3, Interleukin-5 and GM-CSF signalin	immune-related	48
R-HSA-9820965	Respiratory syncytial virus (RSV) genome replica	other	19
R-HSA-5625970	RHO GTPases activate KTN1	cancer-related	11
R-HSA-1236973	Cross-presentation of particulate exogenous ant	immune-related	8
R-HSA-1474151	Tetrahydrobiopterin (BH4) synthesis, recycling, s	other	12
R-HSA-400451	Free fatty acids regulate insulin secretion	cancer-related	11
R-HSA-2025928	Calcineurin activates NFAT	immune-related	11
R-HSA-2173791	TGF-beta receptor signaling in EMT (epithelial to	cancer-related	16
R-HSA-193634	Axonal growth inhibition (RHOA activation)	other	9
R-HSA-193697	p75NTR regulates axonogenesis	other	10
R-HSA-446203	Asparagine N-linked glycosylation	cancer-related	304
R-HSA-9755511	KEAP1-NFE2L2 pathway	cancer-related	126
R-HSA-1632852	Macroautophagy	other	136
R-HSA-9663891	Selective autophagy	other	81
R-HSA-6811442	Intra-Golgi and retrograde Golgi-to-ER traffic	other	202
R-HSA-2467813	Separation of Sister Chromatids	other	190
R-HSA-2555396	Mitotic Metaphase and Anaphase	other	236
R-HSA-68882	Mitotic Anaphase	other	235
R-HSA-9709957	Sensory Perception	other	609
R-HSA-9759194	Nuclear events mediated by NFE2L2	cancer-related	97
R-HSA-3371497	HSP90 chaperone cycle for steroid hormone rec	other	55
R-HSA-8980692	RHOA GTPase cycle	cancer-related	147
R-HSA-4086400	PCP/CE pathway	cancer-related	92
R-HSA-1257604	PIP3 activates AKT signaling	cancer-related	268
R-HSA-397795	G-protein beta:gamma signalling	cancer-related	32
R-HSA-5576891	Cardiac conduction	other	132
R-HSA-3371511	HSF1 activation	cancer-related	12
R-HSA-73886	Chromosome Maintenance	other	140
R-HSA-5213460	RIPK1-mediated regulated necrosis	cancer-related	34
R-HSA-5218859	Regulated Necrosis	cancer-related	62
R-HSA-5675482	Regulation of necroptotic cell death	cancer-related	32
R-HSA-193704	p75 NTR receptor-mediated signalling	other	97
R-HSA-9816359	Maternal to zygotic transition (MZT)	other	117
R-HSA-166658	Complement cascade	immune-related	58
R-HSA-983168	Antigen processing: Ubiquitination & Proteasom	immune-related	309
R-HSA-1912422	Pre-NOTCH Expression and Processing	cancer-related	108
R-HSA-190861	Gap junction assembly	other	36
R-HSA-606279	Deposition of new CENPA-containing nucleosom	other	73
R-HSA-774815	Nucleosome assembly	other	73
R-HSA-2995410	Nuclear Envelope (NE) Reassembly	other	75
R-HSA-9668328	Sealing of the nuclear envelope (NE) by ESCRT-II	other	31
R-HSA-432720	Lysosome Vesicle Biogenesis	other	35
R-HSA-432722	Golgi Associated Vesicle Biogenesis	other	56
R-HSA-5619115	Disorders of transmembrane transporters	other	176

R-HSA-5578775	Ion homeostasis	other	56
R-HSA-936837	Ion transport by P-type ATPases	other	57
R-HSA-211945	Phase I - Functionalization of compounds	other	106
R-HSA-877300	Interferon gamma signaling	immune-related	96
R-HSA-983189	Kinesins	other	59
R-HSA-6811436	COPI-independent Golgi-to-ER retrograde traffic	other	51
R-HSA-2871837	FCERI mediated NF-kB activation	immune-related	82
R-HSA-202424	Downstream TCR signaling	immune-related	98
R-HSA-5607764	CLEC7A (Dectin-1) signaling	other	101
R-HSA-418555	G alpha (s) signalling events	cancer-related	158
R-HSA-190236	Signaling by FGFR	cancer-related	89
R-HSA-5654738	Signaling by FGFR2	cancer-related	73
R-HSA-9013424	RHOV GTPase cycle	cancer-related	38
R-HSA-190840	Microtubule-dependent trafficking of connexons	other	18
R-HSA-190872	Transport of connexons to the plasma membran	other	19
R-HSA-389977	Post-chaperonin tubulin folding pathway	other	22
R-HSA-5620924	Intraflagellar transport	other	54
R-HSA-9609736	Assembly and cell surface presentation of NMDA	other	41
R-HSA-9634597	GPER1 signaling	cancer-related	45
R-HSA-3371568	Attenuation phase	cancer-related	14
R-HSA-3371571	HSF1-dependent transactivation	cancer-related	24
R-HSA-447041	CHL1 interactions	other	9
R-HSA-445095	Interaction between L1 and Ankyrins	cancer-related	31
R-HSA-1236977	Endosomal/Vacuolar pathway	other	11
R-HSA-5673000	RAF activation	cancer-related	36
R-HSA-6803529	FGFR2 alternative splicing	cancer-related	26
R-HSA-418360	Platelet calcium homeostasis	other	30
R-HSA-210990	PECAM1 interactions	immune-related	12
R-HSA-6799990	Metal sequestration by antimicrobial proteins	other	6
R-HSA-418359	Reduction of cytosolic Ca++ levels	other	14
R-HSA-9612973	Autophagy	other	151
R-HSA-198933	Immunoregulatory interactions between a Lymp	immune-related	134
R-HSA-199977	ER to Golgi Anterograde Transport	other	154
R-HSA-6807878	COPI-mediated anterograde transport	other	101
R-HSA-948021	Transport to the Golgi and subsequent modificat	other	185
R-HSA-8856828	Clathrin-mediated endocytosis	cancer-related	146
R-HSA-3299685	Detoxification of Reactive Oxygen Species	cancer-related	37
R-HSA-9711123	Cellular response to chemical stress	cancer-related	215
R-HSA-5617833	Cilium Assembly	other	201
R-HSA-913531	Interferon Signaling	immune-related	274
R-HSA-372790	Signaling by GPCR	cancer-related	709
R-HSA-388396	GPCR downstream signalling	cancer-related	634
R-HSA-556833	Metabolism of lipids	other	758
R-HSA-1169410	Antiviral mechanism by IFN-stimulated genes	immune-related	154
R-HSA-3858494	Beta-catenin independent WNT signaling	cancer-related	146
R-HSA-168255	Influenza Infection	other	168
R-HSA-211859	Biological oxidations	other	222

R-HSA-8876198	RAB GEFs exchange GTP for GDP on RABs	cancer-related	90
R-HSA-9006925	Intracellular signaling by second messengers	other	310
R-HSA-9009391	Extra-nuclear estrogen signaling	other	77
R-HSA-9820952	Respiratory Syncytial Virus Infection Pathway	other	123
R-HSA-9613829	Chaperone Mediated Autophagy	other	22
R-HSA-73887	Death Receptor Signaling	cancer-related	156
R-HSA-111465	Apoptotic cleavage of cellular proteins	cancer-related	38
R-HSA-75153	Apoptotic execution phase	cancer-related	52
R-HSA-2500257	Resolution of Sister Chromatid Cohesion	other	126
R-HSA-9648025	EML4 and NUDC in mitotic spindle formation	other	117
R-HSA-199992	trans-Golgi Network Vesicle Budding	other	72
R-HSA-2454202	Fc epsilon receptor (FCERI) signaling	other	134
R-HSA-70263	Gluconeogenesis	other	35
R-HSA-202403	TCR signaling	immune-related	120
R-HSA-450408	AUF1 (hnRNP D0) binds and destabilizes mRNA	cancer-related	56
R-HSA-8856825	Cargo recognition for clathrin-mediated endocyt	cancer-related	106
R-HSA-438064	Post NMDA receptor activation events	other	80
R-HSA-442755	Activation of NMDA receptors and postsynaptic	other	93
R-HSA-8955332	Carboxyterminal post-translational modification	cancer-related	42
R-HSA-9619483	Activation of AMPK downstream of NMDARs	other	29
R-HSA-114604	GPVI-mediated activation cascade	cancer-related	35
R-HSA-9662851	Anti-inflammatory response favouring Leishman	other	82
R-HSA-9664433	Leishmania parasite growth and survival	other	82
R-HSA-5668599	RHO GTPases Activate NADPH Oxidases	cancer-related	24
R-HSA-8873719	RAB geranylgeranylation	cancer-related	65
R-HSA-6805567	Keratinization	cancer-related	215
R-HSA-6809371	Formation of the cornified envelope	cancer-related	129
R-HSA-9824439	Bacterial Infection Pathways	other	166
R-HSA-9007101	Rab regulation of trafficking	cancer-related	124
R-HSA-5621481	C-type lectin receptors (CLRs)	immune-related	144
R-HSA-9013418	RHOBTB2 GTPase cycle	cancer-related	23
R-HSA-422356	Regulation of insulin secretion	other	78
R-HSA-1852241	Organelle biogenesis and maintenance	cancer-related	296
R-HSA-597592	Post-translational protein modification	cancer-related	1462
R-HSA-71387	Metabolism of carbohydrates	other	299
R-HSA-389958	Cooperation of Prefoldin and TriC/CCT in actin a	cancer-related	32
R-HSA-389957	Prefoldin mediated transfer of substrate to CCT,	cancer-related	28
R-HSA-2262752	Cellular responses to stress	other	807
R-HSA-8953897	Cellular responses to stimuli	other	821
R-HSA-3371556	Cellular response to heat stress	cancer-related	89
R-HSA-9706574	RHOBTB GTPase Cycle	cancer-related	35
R-HSA-9700206	Signaling by ALK in cancer	cancer-related	91
R-HSA-9725370	Signaling by ALK fusions and activated point mut	cancer-related	91
R-HSA-112314	Neurotransmitter receptors and postsynaptic sig	other	205
R-HSA-163685	Integration of energy metabolism	other	108
R-HSA-72163	mRNA Splicing - Major Pathway	other	207
R-HSA-72172	mRNA Splicing	other	215

R-HSA-72203	Processing of Capped Intron-Containing Pre-mRI	other	285
R-HSA-390466	Chaperonin-mediated protein folding	other	90
R-HSA-391251	Protein folding	other	96
R-HSA-9833482	PKR-mediated signaling	other	79
R-HSA-390450	Folding of actin by CCT/TriC	cancer-related	10
R-HSA-376176	Signaling by ROBO receptors	cancer-related	219
R-HSA-112315	Transmission across Chemical Synapses	other	270
R-HSA-8953854	Metabolism of RNA	other	729
R-HSA-389960	Formation of tubulin folding intermediates by C(	cancer-related	25
R-HSA-168273	Influenza Viral RNA Transcription and Replicatioı	other	147
R-HSA-392499	Metabolism of proteins	cancer-related	2077
R-HSA-112316	Neuronal System	other	410
R-HSA-9010553	Regulation of expression of SLITs and ROBOs	other	172
R-HSA-1430728	Metabolism	other	2127
R-HSA-71291	Metabolism of amino acids and derivatives	cancer-related	375

# DEPs	p.val	FDR	OR	ProteinName
10	0.0010	0.6769	10.90	ACTB, CD151, F11R, FLNA, ILK, ITGA6, ITGB1, JUP, KRT14, KRT5
24	0.0016	1.0000	4.04	ACTB, CALM3, G6PD, GP1BA, GSR, H2AC20, H2BC21, HSP90AA1, H
11	0.0028	1.0000	7.22	H2AC20, H2BC21, MYH9, MYL12B, MYL6, RHOA, YWHAB, YWHAE,
12	0.0065	1.0000	5.38	ACTB, CD151, CD47, F11R, FLNA, ILK, ITGA6, ITGB1, JUP, KRT14, KR
4	0.0068	1.0000	Inf	ACTB, FLNA, ILK, ITGB1
4	0.0068	1.0000	Inf	FLNA, GP1BA, GP1BB, YWHAZ
27	0.0072	1.0000	3.04	ACTB, ARPC5, CALM3, CDC42, CFL1, CYBB, FLNA, H2AC20, H2BC21,
90	0.0073	1.0000	2.16	ACTB, ADAM10, ALDOA, ANPEP, ANXA2, ARPC5, B2M, BCAP31, CA
8	0.0086	1.0000	7.90	CFL1, HSP90AA1, ITGB1, MYH9, MYL12B, MYL6, RHOA, TLN1
6	0.0088	1.0000	13.03	GP1BA, GP1BB, ITGA2B, ITGB3, RAP1B, TLN1
6	0.0088	1.0000	13.03	CALM3, CDC42, FLNA, MYH9, MYL12B, MYL6
25	0.0095	1.0000	3.03	ANXA2, CANX, CCL5, CD36, CDC42, CFL1, FSCN1, HNRNPA2B1, HNF
66	0.0109	1.0000	2.15	ACTB, ADAM10, ARPC5, ATP2A3, BCAP31, CALM3, CCL5, CCT2, CCT
29	0.0162	1.0000	2.60	B2M, BCAP31, CALM3, CANX, CD226, CD36, CD99, CDC42, CLTA, C
21	0.0166	1.0000	3.00	CALM3, G6PD, GP1BA, GSR, H2AC20, H2BC21, HSPD1, ITGA2B, NPI
21	0.0166	1.0000	3.00	CALM3, G6PD, GP1BA, GSR, H2AC20, H2BC21, HSPD1, ITGA2B, NPI
9	0.0199	1.0000	5.23	G6PD, GSR, PRDX1, PRDX2, YWHAB, YWHAE, YWHAG, YWHAH, YW
19	0.0219	1.0000	3.00	ACTB, ADAM10, CALM3, CDC42, CLTA, CTSD, CYBB, HNRNPF, HNRN
7	0.0237	1.0000	6.48	ACTB, ITGA2B, ITGB3, RAP1B, TLN1, VCL, YWHAB
7	0.0237	1.0000	6.48	ACTB, ITGA2B, ITGB3, RAP1B, TLN1, VCL, YWHAB
7	0.0237	1.0000	6.48	CD36, FSCN1, HSP90AA1, HSPA8, ITGB1, LBP, VIM
3	0.0241	1.0000	Inf	ACTB, F11R, JUP
3	0.0241	1.0000	Inf	YWHAB, YWHAG, YWHAZ
3	0.0241	1.0000	Inf	YWHAB, YWHAG, YWHAZ
3	0.0241	1.0000	Inf	RAP1B, YWHAB, YWHAZ
3	0.0241	1.0000	Inf	GP1BA, GP1BB, PF4V1
36	0.0255	1.0000	2.24	ANXA2, B2M, CANX, CCL5, CD36, CDC42, CFL1, DHX9, FLNA, FSCN1
5	0.0264	1.0000	10.26	YWHAB, YWHAE, YWHAG, YWHAH, YWHAZ
5	0.0264	1.0000	10.26	YWHAB, YWHAE, YWHAG, YWHAH, YWHAZ
5	0.0264	1.0000	10.26	YWHAB, YWHAE, YWHAG, YWHAH, YWHAZ
5	0.0264	1.0000	10.26	YWHAB, YWHAE, YWHAG, YWHAH, YWHAZ
5	0.0264	1.0000	10.26	YWHAB, YWHAE, YWHAG, YWHAH, YWHAZ
5	0.0264	1.0000	10.26	YWHAB, YWHAE, YWHAG, YWHAH, YWHAZ
42	0.0266	1.0000	2.13	ACTB, ARPC5, BCAP31, CALM3, CCT2, CCT6A, CCT7, CDC42, CFL1, C
42	0.0266	1.0000	2.13	ACTB, ARPC5, BCAP31, CALM3, CCT2, CCT6A, CCT7, CDC42, CFL1, C
17	0.0287	1.0000	3.00	H2AC20, H2BC21, HSP90AA1, NPM1, PSMA5, PSMA6, RAB1B, RCC
12	0.0297	1.0000	3.68	ADAM10, CD151, CD47, CTSD, F11R, ICAM2, ITGA2B, ITGA6, ITGB1
12	0.0297	1.0000	3.68	ACTB, CALM3, MYH9, RAB10, TUBA1C, TUBA4A, TUBB1, YWHAB, Y
52	0.0342	1.0000	1.94	ACTB, ALDOA, ANXA2, ANXA5, ATP2A3, CALM3, CD36, CD47, CD9,
10	0.0382	1.0000	3.90	ACTB, ADAM10, ARPC5, CDC42, CFL1, CLTA, MYH9, MYL12B, MYL6
8	0.0488	1.0000	4.29	ACTB, CALM3, ITGA2B, ITGB3, RAP1B, TLN1, VCL, YWHAB
8	0.0488	1.0000	4.29	H2BC21, PSMA5, PSMA6, YWHAB, YWHAE, YWHAG, YWHAH, YWH
8	0.0488	1.0000	4.29	ACTB, CALM3, ITGA2B, ITGB3, RAP1B, TLN1, VCL, YWHAB

13	0.0493	1.0000	3.05	ACTB, CALM3, CDC42, ITGA2B, ITGB3, PHB, PSMA5, PSMA6, RAP1E
64	0.0605	1.0000	1.71	ACTB, ADAM10, ALDOA, ANPEP, ANXA2, ARPC5, B2M, CALM3, CAN
6	0.0618	1.0000	5.10	ACTB, ARPC5, CDC42, CFL1, HSP90AA1, MYH9
6	0.0618	1.0000	5.10	ACTB, ARPC5, CDC42, CFL1, HSP90AA1, MYH9
6	0.0618	1.0000	5.10	CANX, CLTA, CTSD, TUBA1C, TUBA4A, TUBB1
6	0.0618	1.0000	5.10	H2BC21, YWHAB, YWHAE, YWHAG, YWHAH, YWHAZ
11	0.0647	1.0000	3.10	BCAP31, DSG1, DSP, PSMA5, PSMA6, VIM, YWHAB, YWHAE, YWHA
11	0.0647	1.0000	3.10	G6PD, GSR, NPM1, PRDX1, PRDX2, TNKS1BP1, YWHAB, YWHAE, YV
14	0.0756	1.0000	2.59	BCAP31, DSG1, DSP, FLOT1, HSP90AA1, PSMA5, PSMA6, UBE2L3, V
4	0.0760	1.0000	7.57	ACTB, ARPC5, CDC42, MYH9
4	0.0760	1.0000	7.57	ACTB, ARPC5, CDC42, MYH9
4	0.0760	1.0000	7.57	ACTB, ARPC5, CDC42, MYH9
4	0.0760	1.0000	7.57	CD36, LBP, S100A8, S100A9
4	0.0760	1.0000	7.57	CD36, LBP, S100A8, S100A9
4	0.0760	1.0000	7.57	ACTB, H2AC20, H2BC21, KANSL3
4	0.0760	1.0000	7.57	CDC42, JUP, STOM, TFRC
4	0.0760	1.0000	7.57	ITGA2B, ITGB3, RAP1B, TLN1
4	0.0760	1.0000	7.57	ACTB, H2AC20, H2BC21, KANSL3
4	0.0760	1.0000	7.57	ACTB, H2AC20, H2BC21, KANSL3
4	0.0760	1.0000	7.57	ACTB, H2AC20, PSMA5, PSMA6
4	0.0760	1.0000	7.57	CTSD, H2AC20, H2BC21, HSP90AA1
4	0.0760	1.0000	7.57	MYH9, MYL12B, MYL6, RHOA
4	0.0760	1.0000	7.57	CDC42, JUP, STOM, TFRC
4	0.0760	1.0000	7.57	CDC42, JUP, STOM, TFRC
4	0.0760	1.0000	7.57	CD36, LBP, S100A8, S100A9
4	0.0760	1.0000	7.57	MYH9, MYL12B, MYL6, RHOA
4	0.0760	1.0000	7.57	MYH9, MYL12B, MYL6, RHOA
4	0.0760	1.0000	7.57	MYH9, MYL12B, MYL6, RHOA
4	0.0760	1.0000	7.57	ACTB, H2AC20, H2BC21, KANSL3
4	0.0760	1.0000	7.57	FCER1G, GP1BA, GP1BB, ITGB1
4	0.0760	1.0000	7.57	ITGA6, ITGB1, PPIA, SPN
4	0.0760	1.0000	7.57	ITGA2B, ITGB3, RAP1B, TLN1
4	0.0760	1.0000	7.57	ITGA2B, ITGB3, RAP1B, TLN1
72	0.0810	1.0000	1.61	ACTB, ADAM10, ANTXR2, ARPC5, ATIC, B2M, BCAP31, CALM3, CAN
2	0.0844	1.0000	Inf	ACTB, JUP
2	0.0844	1.0000	Inf	ANPEP, CTSD
2	0.0844	1.0000	Inf	ANPEP, CTSD
2	0.0844	1.0000	Inf	ITGA6, ITGB1
2	0.0844	1.0000	Inf	ITGB1, RAP1B
2	0.0844	1.0000	Inf	CD151, ITGA6
2	0.0844	1.0000	Inf	GP1BA, GP1BB
2	0.0844	1.0000	Inf	GP1BA, GP1BB
2	0.0844	1.0000	Inf	ACTB, CLTA
2	0.0844	1.0000	Inf	ACTB, CLTA
2	0.0844	1.0000	Inf	CD151, ITGA6
2	0.0844	1.0000	Inf	GP1BA, GP1BB
2	0.0844	1.0000	Inf	GP1BA, GP1BB

2	0.0844	1.0000	Inf	GP1BA, GP1BB
2	0.0844	1.0000	Inf	GP1BA, GP1BB
2	0.0844	1.0000	Inf	GP1BA, GP1BB
2	0.0844	1.0000	Inf	GP1BA, GP1BB
2	0.0844	1.0000	Inf	ILK, ITGB1
9	0.0850	1.0000	3.20	ACTB, CALM3, ITGA2B, ITGB3, PHB, RAP1B, TLN1, VCL, YWHAB
9	0.0850	1.0000	3.20	H2BC21, PSMA5, PSMA6, RCC2, YWHAB, YWHAE, YWHAG, YWHAH
9	0.0850	1.0000	3.20	ACTB, CALM3, ITGA2B, ITGB3, PHB, RAP1B, TLN1, VCL, YWHAB
9	0.0850	1.0000	3.20	ACTB, CALM3, ITGA2B, ITGB3, PHB, RAP1B, TLN1, VCL, YWHAB
9	0.0850	1.0000	3.20	ACTB, CALM3, ITGA2B, ITGB3, PHB, RAP1B, TLN1, VCL, YWHAB
9	0.0850	1.0000	3.20	ACTB, CALM3, ITGA2B, ITGB3, PHB, RAP1B, TLN1, VCL, YWHAB
9	0.0850	1.0000	3.20	CD47, F11R, ICAM2, ITGA2B, ITGA6, ITGB1, ITGB3, PECAM1, THBS1
12	0.0996	1.0000	2.57	ACTB, CALM3, ITGA2B, ITGB3, PHB, PSMA5, PSMA6, RAP1B, SPTBN
12	0.0996	1.0000	2.57	ACTB, CALM3, ITGA2B, ITGB3, PHB, PSMA5, PSMA6, RAP1B, SPTBN
7	0.1125	1.0000	3.38	GP1BA, H2AC20, H2BC21, ITGA2B, PSMA5, PSMA6, THBS1
21	0.1181	1.0000	1.95	B2M, CANX, EEF1A1, G3BP1, HLA-A, HLA-B, HSP90AA1, ITGB1, NPN
27	0.1204	1.0000	1.80	ACTB, ANK1, ARPC5, CALM3, CD36, CD59, CLTA, HSP90AA1, HSPA8
13	0.1427	1.0000	2.19	H2AC20, H2BC21, HSP90AA1, PSMA5, PSMA6, RAB1B, RCC2, TUBA
13	0.1427	1.0000	2.19	H2AC20, H2BC21, HSP90AA1, PSMA5, PSMA6, RAB1B, RCC2, TUBA
13	0.1427	1.0000	2.19	ANXA2, CANX, CDC42, CFL1, HNRNPA2B1, HNRNPF, HSPA9, MSN, F
13	0.1427	1.0000	2.19	CD47, CD99, F11R, FCER1G, INPP5D, ITGA6, ITGB1, ITGB3, PECAM1
37	0.1437	1.0000	1.60	ALDOA, ANXA5, CALM3, CD36, CD9, CDC42, CFL1, ENDOD1, FCER1
16	0.1488	1.0000	1.99	B2M, CANX, G3BP1, HLA-A, HLA-B, HSP90AA1, RPS15, RPS15A, RPS
5	0.1503	1.0000	3.76	H2AC20, H2BC21, PRDX1, PRDX2, YWHAE
5	0.1503	1.0000	3.76	ACTB, CDC42, TUBA1C, TUBA4A, TUBB1
5	0.1503	1.0000	3.76	H2AC20, H2BC21, PSMA5, PSMA6, YWHAZ
5	0.1503	1.0000	3.76	ACTB, CLTA, TUBA1C, TUBA4A, TUBB1
5	0.1503	1.0000	3.76	ACTB, CLTA, TUBA1C, TUBA4A, TUBB1
5	0.1503	1.0000	3.76	GP1BA, H2AC20, H2BC21, ITGA2B, THBS1
5	0.1503	1.0000	3.76	CFL1, MYH9, MYL12B, MYL6, RHOA
8	0.1761	1.0000	2.52	ACTB, CALM3, CDC42, CYBB, HSP90AA1, ITGB3, JUP, RHOA
8	0.1761	1.0000	2.52	ACTB, CALM3, CDC42, CYBB, HSP90AA1, ITGB3, JUP, RHOA
8	0.1761	1.0000	2.52	B2M, CD36, HLA-A, HLA-B, PSMA5, PSMA6, S100A8, S100A9
14	0.1931	1.0000	1.90	CANX, EEF1A1, NPM1, PPIA, RPS15, RPS15A, RPS23, RPS3A, RPSA,
20	0.1942	1.0000	1.70	ACTB, ADAM10, ATIC, CALM3, HSP90AA1, ITGA2B, ITGB3, MSN, M
3	0.2049	1.0000	4.97	CALM3, HSP90AA1, JUP
3	0.2049	1.0000	4.97	ACTB, ARPC5, CDC42
3	0.2049	1.0000	4.97	PRDX1, PRDX2, YWHAE
3	0.2049	1.0000	4.97	PRDX1, PRDX2, YWHAE
3	0.2049	1.0000	4.97	PRDX1, PRDX2, YWHAE
3	0.2049	1.0000	4.97	ITGB1, PTPRJ, RAP1B
3	0.2049	1.0000	4.97	H2AC20, H2BC21, RAB1B
3	0.2049	1.0000	4.97	HSP90AA1, ITGB1, TUBB
3	0.2049	1.0000	4.97	CALM3, SRI, STOM
3	0.2049	1.0000	4.97	CD9, H2AC20, H2BC21
3	0.2049	1.0000	4.97	CLTA, RHOA, YWHAB
3	0.2049	1.0000	4.97	CLTA, RHOA, YWHAB

3	0.2049	1.0000	4.97	CYB5R3, SLC2A1, SLC2A3
3	0.2049	1.0000	4.97	CDC42, PSMA5, PSMA6
3	0.2049	1.0000	4.97	PSMA5, PSMA6, YWHAZ
3	0.2049	1.0000	4.97	ACTB, ADAM10, CLTA
3	0.2049	1.0000	4.97	H2AC20, H2BC21, HSP90AA1
3	0.2049	1.0000	4.97	ACTB, H2AC20, H2BC21
3	0.2049	1.0000	4.97	ACTB, H2AC20, H2BC21
3	0.2049	1.0000	4.97	JUP, STOM, TFRC
3	0.2049	1.0000	4.97	CDC42, ITGB1, TFRC
3	0.2049	1.0000	4.97	CD36, S100A8, S100A9
3	0.2049	1.0000	4.97	CD36, S100A8, S100A9
3	0.2049	1.0000	4.97	CD36, S100A8, S100A9
3	0.2049	1.0000	4.97	CD36, S100A8, S100A9
3	0.2049	1.0000	4.97	CD36, S100A8, S100A9
3	0.2049	1.0000	4.97	CD36, S100A8, S100A9
3	0.2049	1.0000	4.97	CD36, S100A8, S100A9
3	0.2049	1.0000	4.97	CD36, S100A8, S100A9
3	0.2049	1.0000	4.97	CD36, HSP90AA1, LRP1
3	0.2049	1.0000	4.97	CYB5R3, SLC2A1, SLC2A3
3	0.2049	1.0000	4.97	ITGA2B, ITGB1, ITGB3
3	0.2049	1.0000	4.97	ITGA2B, ITGB1, ITGB3
3	0.2049	1.0000	4.97	CD9, EEF2, HSP90AA1
52	0.2093	1.0000	1.39	ADAM10, ALDOA, ANPEP, ANXA2, ARPC5, B2M, CAMP, CCT2, CCT8
33	0.2178	1.0000	1.47	ACTB, ADAM10, ANK1, ARPC5, CDC42, CFL1, CLTA, HSP90AA1, HSP
33	0.2178	1.0000	1.47	ACTB, ADAM10, ANK1, ARPC5, CDC42, CFL1, CLTA, HSP90AA1, HSP
24	0.2356	1.0000	1.53	ACTB, ANK1, ARPC5, CALM3, CD59, CLTA, HSPA8, M6PR, MYH9, RA
6	0.2389	1.0000	2.49	CAMP, DCD, LTF, LYZ, S100A8, S100A9
6	0.2389	1.0000	2.49	H2AC20, H2BC21, ITGB1, TUBA1C, TUBA4A, TUBB1
6	0.2389	1.0000	2.49	B2M, HLA-A, NPM1, PPIA, PSMA5, PSMA6
6	0.2389	1.0000	2.49	CALM3, CYB5R3, HSP90AA1, LRP1, SLC2A1, SLC2A3
6	0.2389	1.0000	2.49	H2AC20, H2BC21, ITGB1, TUBA1C, TUBA4A, TUBB1
6	0.2389	1.0000	2.49	HSPA1B, HSPA8, PSMA5, PSMA6, YWHAB, YWHAZ
6	0.2389	1.0000	2.49	HSP90AA1, PSMA5, PSMA6, TUBA1C, TUBA4A, TUBB1
6	0.2389	1.0000	2.49	ACTB, CLTA, MSN, TUBA1C, TUBA4A, TUBB1
6	0.2389	1.0000	2.49	ACTB, CALM3, CDC42, TUBA1C, TUBA4A, TUBB1
37	0.2483	1.0000	1.39	B2M, BCAP31, CANX, CLTA, EEF1A1, G3BP1, H2AC20, H2BC21, HLA
9	0.2490	1.0000	2.00	ACTB, CDC42, ITGB1, PFN1, RCC2, RHOA, TUBA1C, TUBA4A, TUBB1
9	0.2490	1.0000	2.00	HSP90AA1, PSMA5, PSMA6, TUBA1C, TUBA4A, TUBB, TUBB1, YWH
9	0.2490	1.0000	2.00	HSP90AA1, PSMA5, PSMA6, TUBA1C, TUBA4A, TUBB, TUBB1, YWH
9	0.2490	1.0000	2.00	B2M, CD36, CYBB, HLA-A, HLA-B, PSMA5, PSMA6, S100A8, S100A9
15	0.2492	1.0000	1.68	B2M, G3BP1, HLA-A, HLA-B, HSP90AA1, RPS15, RPS15A, RPS23, RP
50	0.2495	1.0000	1.34	ACTB, ANTXR2, ARPC5, B2M, BCAP31, CALM3, CANX, CD9, CDC42,
12	0.2509	1.0000	1.79	ACTB, ANK1, CLTA, HSPA8, ITGA2B, ITGB1, ITGB3, MSN, SPTBN1, T
12	0.2509	1.0000	1.79	B2M, BCAP31, CANX, CD36, CYBB, HLA-A, HLA-B, PSMA5, PSMA6, S
12	0.2509	1.0000	1.79	ANXA2, CDC42, CFL1, HNRNPA2B1, HNRNPF, HSPA9, MSN, PPIA, R
12	0.2509	1.0000	1.79	ANXA2, CDC42, CFL1, HNRNPA2B1, HNRNPF, HSPA9, MSN, PPIA, R
54	0.2739	1.0000	1.29	ACTB, ADAM10, ANK1, ARPC5, CASP14, CD36, CDC42, CFL1, CLTA,

1	0.2920	1.0000	Inf	CDC42
1	0.2920	1.0000	Inf	CDC42
1	0.2920	1.0000	Inf	CDC42
1	0.2920	1.0000	Inf	HSP90AA1
1	0.2920	1.0000	Inf	CD36
1	0.2920	1.0000	Inf	CD36
1	0.2920	1.0000	Inf	CD36
1	0.2920	1.0000	Inf	HSP90AA1
1	0.2920	1.0000	Inf	TLN1
1	0.2920	1.0000	Inf	TLN1
1	0.2920	1.0000	Inf	TLN1
1	0.2920	1.0000	Inf	PPIA
1	0.2920	1.0000	Inf	PPIA
1	0.2920	1.0000	Inf	ACTB
1	0.2920	1.0000	Inf	ACTB
1	0.2920	1.0000	Inf	ACTB
1	0.2920	1.0000	Inf	JUP
1	0.2920	1.0000	Inf	JUP
1	0.2920	1.0000	Inf	JUP
1	0.2920	1.0000	Inf	JUP
1	0.2920	1.0000	Inf	JUP
1	0.2920	1.0000	Inf	CLTA
1	0.2920	1.0000	Inf	CLTA
1	0.2920	1.0000	Inf	CLTA
1	0.2920	1.0000	Inf	CLTA
1	0.2920	1.0000	Inf	CANX
1	0.2920	1.0000	Inf	CANX
1	0.2920	1.0000	Inf	ITGB1
1	0.2920	1.0000	Inf	ACTB
1	0.2920	1.0000	Inf	H2AC20
1	0.2920	1.0000	Inf	CANX
1	0.2920	1.0000	Inf	CANX
1	0.2920	1.0000	Inf	H2AC20
1	0.2920	1.0000	Inf	CDC42
1	0.2920	1.0000	Inf	CTSD
1	0.2920	1.0000	Inf	CTSD
1	0.2920	1.0000	Inf	ITGB1
1	0.2920	1.0000	Inf	YWHAB
1	0.2920	1.0000	Inf	YWHAB
1	0.2920	1.0000	Inf	TLN1
1	0.2920	1.0000	Inf	ITGB1
1	0.2920	1.0000	Inf	KANSL3
1	0.2920	1.0000	Inf	PPIA
1	0.2920	1.0000	Inf	HSP90AA1
1	0.2920	1.0000	Inf	PF4V1
1	0.2920	1.0000	Inf	CLTA
1	0.2920	1.0000	Inf	CANX

1	0.2920	1.0000	Inf	CANX
1	0.2920	1.0000	Inf	CANX
1	0.2920	1.0000	Inf	CANX
1	0.2920	1.0000	Inf	F11R
1	0.2920	1.0000	Inf	HSP90AA1
1	0.2920	1.0000	Inf	HSP90AA1
1	0.2920	1.0000	Inf	HSP90AA1
1	0.2920	1.0000	Inf	ITGB1
1	0.2920	1.0000	Inf	MYH9
1	0.2920	1.0000	Inf	YWHAB
1	0.2920	1.0000	Inf	YWHAB
1	0.2920	1.0000	Inf	YWHAB
1	0.2920	1.0000	Inf	ITGB1
1	0.2920	1.0000	Inf	HSP90AA1
1	0.2920	1.0000	Inf	CD9
1	0.2920	1.0000	Inf	HSP90AA1
1	0.2920	1.0000	Inf	HSP90AA1
1	0.2920	1.0000	Inf	HSP90AA1
1	0.2920	1.0000	Inf	HSP90AA1
1	0.2920	1.0000	Inf	HSP90AA1
1	0.2920	1.0000	Inf	HSP90AA1
1	0.2920	1.0000	Inf	HSP90AA1
1	0.2920	1.0000	Inf	HSP90AA1
1	0.2920	1.0000	Inf	HSP90AA1
1	0.2920	1.0000	Inf	HSP90AA1
1	0.2920	1.0000	Inf	HSP90AA1
1	0.2920	1.0000	Inf	HSP90AA1
1	0.2920	1.0000	Inf	HSP90AA1
1	0.2920	1.0000	Inf	HSP90AA1
1	0.2920	1.0000	Inf	HSP90AA1
1	0.2920	1.0000	Inf	HSP90AA1
1	0.2920	1.0000	Inf	HSP90AA1
1	0.2920	1.0000	Inf	HSP90AA1
1	0.2920	1.0000	Inf	PPIA
1	0.2920	1.0000	Inf	YWHAB
1	0.2920	1.0000	Inf	CD9
1	0.2920	1.0000	Inf	YWHAB
1	0.2920	1.0000	Inf	YWHAB
1	0.2920	1.0000	Inf	YWHAZ
1	0.2920	1.0000	Inf	SLC2A3
1	0.2920	1.0000	Inf	CDC42
1	0.2920	1.0000	Inf	CLTA
1	0.2920	1.0000	Inf	CLTA
1	0.2920	1.0000	Inf	CLTA
1	0.2920	1.0000	Inf	PPIA
1	0.2920	1.0000	Inf	PPIA
1	0.2920	1.0000	Inf	YWHAB

1	0.2920	1.0000	Inf	RAP1B
1	0.2920	1.0000	Inf	PECAM1
1	0.2920	1.0000	Inf	YWHAZ
1	0.2920	1.0000	Inf	JUP
1	0.2920	1.0000	Inf	PPIA
1	0.2920	1.0000	Inf	PPIA
1	0.2920	1.0000	Inf	PPIA
1	0.2920	1.0000	Inf	PPIA
1	0.2920	1.0000	Inf	CDC42
1	0.2920	1.0000	Inf	FLNA
1	0.2920	1.0000	Inf	CD36
1	0.2920	1.0000	Inf	YWHAB
1	0.2920	1.0000	Inf	YWHAB
1	0.2920	1.0000	Inf	YWHAB
1	0.2920	1.0000	Inf	CANX
1	0.2920	1.0000	Inf	CDC42
1	0.2920	1.0000	Inf	CDC42
1	0.2920	1.0000	Inf	RAB1B
1	0.2920	1.0000	Inf	CD36
1	0.2920	1.0000	Inf	ITGB1
1	0.2920	1.0000	Inf	AACS
1	0.2920	1.0000	Inf	AACS
1	0.2920	1.0000	Inf	HSP90AA1
1	0.2920	1.0000	Inf	HSP90AA1
1	0.2920	1.0000	Inf	HSP90AA1
1	0.2920	1.0000	Inf	LBP
1	0.2920	1.0000	Inf	CANX
1	0.2920	1.0000	Inf	CANX
13	0.3185	1.0000	1.56	ATP2A3, CALM3, CLTA, GNG5, PHB, PSMA5, PSMA6, SLC2A1, SLC2/
13	0.3185	1.0000	1.56	EEF1A1, NPM1, PPIA, RPS15, RPS15A, RPS23, RPS3A, RPSA, YWHAI
10	0.3270	1.0000	1.66	ANXA2, ATP2A3, CALM3, MYL12B, MYL6, SRI, TLN1, TPM4, VCL, VI
26	0.3316	1.0000	1.33	ALDOA, ANXA5, CALM3, CD36, CD9, CFL1, ENDOD1, FERMT3, FLNA
26	0.3316	1.0000	1.33	ALDOA, ANXA5, CALM3, CD36, CD9, CFL1, ENDOD1, FERMT3, FLNA
7	0.3337	1.0000	1.86	CALM3, CTSD, GNAI2, GNG5, H2AC20, H2BC21, HSP90AA1
7	0.3337	1.0000	1.86	CALM3, CTSD, GNAI2, GNG5, H2AC20, H2BC21, HSP90AA1
7	0.3337	1.0000	1.86	ACTB, ARPC5, CALM3, CDC42, GNAI2, GNG5, MYH9
7	0.3337	1.0000	1.86	ACTB, ARPC5, CALM3, CDC42, GNAI2, GNG5, MYH9
7	0.3337	1.0000	1.86	ADAM10, ATP2A3, H2AC20, H2BC21, PSMA5, PSMA6, YWHAZ
7	0.3337	1.0000	1.86	B2M, HLA-A, NPM1, PPIA, PSMA5, PSMA6, XRCC5
7	0.3337	1.0000	1.86	ACTB, H2AC20, H2BC21, PSMA5, PSMA6, RHOA, VDAC2
7	0.3337	1.0000	1.86	ADAM10, B2M, H2AC20, H2BC21, LTF, LYZ, TSPAN14
7	0.3337	1.0000	1.86	HSP90AA1, TUBA1C, TUBA4A, TUBB, TUBB1, YWHAE, YWHAG
7	0.3337	1.0000	1.86	ANXA2, CALM3, MYL12B, MYL6, TLN1, TPM4, VCL
4	0.3338	1.0000	2.47	CDC42, CYBB, ITGB1, TFRC
4	0.3338	1.0000	2.47	CDC42, CYBB, ITGB1, TFRC
4	0.3338	1.0000	2.47	RAB1B, TUBA1C, TUBA4A, TUBB1
4	0.3338	1.0000	2.47	RAB1B, TUBA1C, TUBA4A, TUBB1

4	0.3338	1.0000	2.47	ACTB, H2AC20, H2BC21, XRCC5
4	0.3338	1.0000	2.47	ADAM10, PSMA5, PSMA6, YWHAZ
4	0.3338	1.0000	2.47	ATP2A3, CALM3, SRI, STOM
4	0.3338	1.0000	2.47	ACTB, MSN, MYH9, SPTBN1
4	0.3338	1.0000	2.47	ACTB, MSN, MYH9, SPTBN1
4	0.3338	1.0000	2.47	ACTB, MSN, MYH9, SPTBN1
4	0.3338	1.0000	2.47	CALM3, PPIA, PSMA5, PSMA6
4	0.3338	1.0000	2.47	CALM3, PPIA, PSMA5, PSMA6
4	0.3338	1.0000	2.47	H2AC20, H2BC21, PSMA5, PSMA6
4	0.3338	1.0000	2.47	H2AC20, H2BC21, PSMA5, PSMA6
4	0.3338	1.0000	2.47	H2AC20, H2BC21, PSMA5, PSMA6
4	0.3338	1.0000	2.47	H2AC20, H2BC21, PSMA5, PSMA6
4	0.3338	1.0000	2.47	FLOT1, JUP, STOM, TFRC
4	0.3338	1.0000	2.47	F11R, ITGB1, ITGB3, RHOA
4	0.3338	1.0000	2.47	F11R, ITGB1, ITGB3, RHOA
4	0.3338	1.0000	2.47	ITGA6, ITGB1, ITGB3, THBS1
4	0.3338	1.0000	2.47	CALM3, SLC2A1, SLC2A3, SRI
4	0.3338	1.0000	2.47	ITGA6, ITGB1, ITGB3, THBS1
4	0.3338	1.0000	2.47	CD151, ITGA6, KRT14, KRT5
11	0.4065	1.0000	1.41	CALM3, CLTA, GNG5, H2AC20, H2BC21, PFN1, PSMA5, PSMA6, RA(
21	0.4159	1.0000	1.24	ACTB, BCAP31, CCT2, CCT6A, CCT7, CDC42, CYBB, DSG1, DSP, FLOT
8	0.4282	1.0000	1.48	HSP90AA1, RCC2, TUBA1C, TUBA4A, TUBB, TUBB1, YWHAE, YWHA
5	0.4561	1.0000	1.64	CDC42, CYBB, ITGB1, RAC2, TFRC
5	0.4561	1.0000	1.64	ALDOA, ENO1, GAPDH, PGK1, PKM
5	0.4561	1.0000	1.64	ALDOA, ENO1, GAPDH, PGK1, PKM
5	0.4561	1.0000	1.64	HSP90AA1, TUBA1C, TUBA4A, TUBB1, VIM
5	0.4561	1.0000	1.64	ANTXR2, CALM3, CD9, EEF2, HSP90AA1
5	0.4561	1.0000	1.64	H2AC20, H2BC21, PSMA5, PSMA6, VDAC2
5	0.4561	1.0000	1.64	ACTB, ARPC5, CDC42, CFL1, RHOA
5	0.4561	1.0000	1.64	HSP90AA1, TUBA4A, TUBB, YWHAE, YWHAG
5	0.4561	1.0000	1.64	HSP90AA1, TUBA4A, TUBB, YWHAE, YWHAG
5	0.4561	1.0000	1.64	HSP90AA1, TUBA4A, TUBB, YWHAE, YWHAG
5	0.4561	1.0000	1.64	HSP90AA1, TUBA4A, TUBB, YWHAE, YWHAG
5	0.4561	1.0000	1.64	HSP90AA1, TUBA4A, TUBB, YWHAE, YWHAG
5	0.4561	1.0000	1.64	HSP90AA1, TUBA4A, TUBB, YWHAE, YWHAG
5	0.4561	1.0000	1.64	HSP90AA1, TUBA4A, TUBB, YWHAE, YWHAG
5	0.4561	1.0000	1.64	B2M, BCAP31, CANX, HLA-A, HLA-B
5	0.4561	1.0000	1.64	B2M, G3BP1, HLA-A, HLA-B, HSP90AA1
5	0.4561	1.0000	1.64	PSMA5, PSMA6, TUBA1C, TUBA4A, TUBB1
5	0.4561	1.0000	1.64	PSMA5, PSMA6, TUBA1C, TUBA4A, TUBB1
5	0.4561	1.0000	1.64	FLOT1, JUP, RHOA, STOM, TFRC
5	0.4561	1.0000	1.64	ATP2A3, CALM3, GNG5, PECAM1, SRI
2	0.4997	1.0000	2.45	ADAM10, QSOX1
2	0.4997	1.0000	2.45	ADAM10, QSOX1
2	0.4997	1.0000	2.45	HSP90AA1, RHOA
2	0.4997	1.0000	2.45	PSMA5, PSMA6
2	0.4997	1.0000	2.45	PSMA5, PSMA6

2	0.4997	1.0000	2.45	PSMA5, PSMA6
2	0.4997	1.0000	2.45	PSMA5, PSMA6
2	0.4997	1.0000	2.45	PSMA5, PSMA6
2	0.4997	1.0000	2.45	PSMA5, PSMA6
2	0.4997	1.0000	2.45	PSMA5, PSMA6
2	0.4997	1.0000	2.45	PSMA5, PSMA6
2	0.4997	1.0000	2.45	H2AC20, H2BC21
2	0.4997	1.0000	2.45	PPIA, XRCC5
2	0.4997	1.0000	2.45	PSMA5, PSMA6
2	0.4997	1.0000	2.45	PSMA5, PSMA6
2	0.4997	1.0000	2.45	CD59, CPN1
2	0.4997	1.0000	2.45	PSMA5, PSMA6
2	0.4997	1.0000	2.45	H2AC20, H2BC21
2	0.4997	1.0000	2.45	H2AC20, H2BC21
2	0.4997	1.0000	2.45	PSMA5, PSMA6
2	0.4997	1.0000	2.45	PSMA5, PSMA6
2	0.4997	1.0000	2.45	DSG1, DSP
2	0.4997	1.0000	2.45	H2AC20, H2BC21
2	0.4997	1.0000	2.45	H2AC20, H2BC21
2	0.4997	1.0000	2.45	B2M, HLA-A
2	0.4997	1.0000	2.45	B2M, HLA-A
2	0.4997	1.0000	2.45	B2M, HLA-A
2	0.4997	1.0000	2.45	H2AC20, H2BC21
2	0.4997	1.0000	2.45	H2AC20, H2BC21
2	0.4997	1.0000	2.45	PSMA5, PSMA6
2	0.4997	1.0000	2.45	PSMA5, PSMA6
2	0.4997	1.0000	2.45	PSMA5, PSMA6
2	0.4997	1.0000	2.45	H2AC20, H2BC21
2	0.4997	1.0000	2.45	H2AC20, H2BC21
2	0.4997	1.0000	2.45	H2AC20, H2BC21
2	0.4997	1.0000	2.45	ADAM10, CTSD
2	0.4997	1.0000	2.45	H2AC20, H2BC21
2	0.4997	1.0000	2.45	H2AC20, H2BC21
2	0.4997	1.0000	2.45	H2AC20, H2BC21
2	0.4997	1.0000	2.45	CD59, RAB1B
2	0.4997	1.0000	2.45	PSMA5, PSMA6
2	0.4997	1.0000	2.45	PSMA5, PSMA6
2	0.4997	1.0000	2.45	PSMA5, PSMA6
2	0.4997	1.0000	2.45	PSMA5, PSMA6
2	0.4997	1.0000	2.45	H2AC20, H2BC21
2	0.4997	1.0000	2.45	PSMA5, PSMA6
2	0.4997	1.0000	2.45	PSMA5, PSMA6
2	0.4997	1.0000	2.45	PSMA5, PSMA6
2	0.4997	1.0000	2.45	PSMA5, PSMA6
2	0.4997	1.0000	2.45	PSMA5, PSMA6
2	0.4997	1.0000	2.45	PSMA5, PSMA6
2	0.4997	1.0000	2.45	PSMA5, PSMA6

2	0.4997	1.0000	2.45	PSMA5, PSMA6
2	0.4997	1.0000	2.45	H2AC20, H2BC21
2	0.4997	1.0000	2.45	PSMA5, PSMA6
2	0.4997	1.0000	2.45	PSMA5, PSMA6
2	0.4997	1.0000	2.45	PSMA5, PSMA6
2	0.4997	1.0000	2.45	PSMA5, PSMA6
2	0.4997	1.0000	2.45	PSMA5, PSMA6
2	0.4997	1.0000	2.45	PSMA5, PSMA6
2	0.4997	1.0000	2.45	PSMA5, PSMA6
2	0.4997	1.0000	2.45	PSMA5, PSMA6
2	0.4997	1.0000	2.45	PSMA5, PSMA6
2	0.4997	1.0000	2.45	PSMA5, PSMA6
2	0.4997	1.0000	2.45	PSMA5, PSMA6
2	0.4997	1.0000	2.45	PSMA5, PSMA6
2	0.4997	1.0000	2.45	PSMA5, PSMA6
2	0.4997	1.0000	2.45	PSMA5, PSMA6
2	0.4997	1.0000	2.45	PSMA5, PSMA6
2	0.4997	1.0000	2.45	PSMA5, PSMA6
2	0.4997	1.0000	2.45	PSMA5, PSMA6
2	0.4997	1.0000	2.45	PSMA5, PSMA6
2	0.4997	1.0000	2.45	PSMA5, PSMA6
2	0.4997	1.0000	2.45	PSMA5, PSMA6
2	0.4997	1.0000	2.45	PSMA5, PSMA6
2	0.4997	1.0000	2.45	PSMA5, PSMA6
2	0.4997	1.0000	2.45	PSMA5, PSMA6
2	0.4997	1.0000	2.45	PSMA5, PSMA6
2	0.4997	1.0000	2.45	PSMA5, PSMA6
2	0.4997	1.0000	2.45	PSMA5, PSMA6
2	0.4997	1.0000	2.45	PSMA5, PSMA6
2	0.4997	1.0000	2.45	PSMA5, PSMA6
2	0.4997	1.0000	2.45	PSMA5, PSMA6
2	0.4997	1.0000	2.45	PSMA5, PSMA6
2	0.4997	1.0000	2.45	PSMA5, PSMA6
2	0.4997	1.0000	2.45	PSMA5, PSMA6
2	0.4997	1.0000	2.45	PSMA5, PSMA6
2	0.4997	1.0000	2.45	PSMA5, PSMA6
2	0.4997	1.0000	2.45	PSMA5, PSMA6
2	0.4997	1.0000	2.45	PSMA5, PSMA6
2	0.4997	1.0000	2.45	PSMA5, PSMA6
2	0.4997	1.0000	2.45	PSMA5, PSMA6
2	0.4997	1.0000	2.45	PSMA5, PSMA6
2	0.4997	1.0000	2.45	PSMA5, PSMA6
2	0.4997	1.0000	2.45	PSMA5, PSMA6
2	0.4997	1.0000	2.45	PSMA5, PSMA6
2	0.4997	1.0000	2.45	PSMA5, PSMA6
2	0.4997	1.0000	2.45	PSMA5, PSMA6
2	0.4997	1.0000	2.45	ADAM10, CTSD

2	0.4997	1.0000	2.45	ADAM10, CDC42
2	0.4997	1.0000	2.45	ADAM10, YWHAZ
2	0.4997	1.0000	2.45	H2AC20, H2BC21
2	0.4997	1.0000	2.45	H2AC20, H2BC21
2	0.4997	1.0000	2.45	CALM3, HSP90AA1
2	0.4997	1.0000	2.45	CDC42, SPTBN1
2	0.4997	1.0000	2.45	H2AC20, H2BC21
2	0.4997	1.0000	2.45	H2AC20, H2BC21
2	0.4997	1.0000	2.45	H2AC20, H2BC21
2	0.4997	1.0000	2.45	H2AC20, H2BC21
2	0.4997	1.0000	2.45	H2AC20, H2BC21
2	0.4997	1.0000	2.45	H2AC20, H2BC21
2	0.4997	1.0000	2.45	H2AC20, H2BC21
2	0.4997	1.0000	2.45	H2AC20, H2BC21
2	0.4997	1.0000	2.45	H2AC20, H2BC21
2	0.4997	1.0000	2.45	H2AC20, H2BC21
2	0.4997	1.0000	2.45	H2AC20, H2BC21
2	0.4997	1.0000	2.45	H2AC20, H2BC21
2	0.4997	1.0000	2.45	H2AC20, H2BC21
2	0.4997	1.0000	2.45	H2AC20, H2BC21
2	0.4997	1.0000	2.45	H2AC20, H2BC21
2	0.4997	1.0000	2.45	H2AC20, H2BC21
2	0.4997	1.0000	2.45	H2AC20, H2BC21
2	0.4997	1.0000	2.45	H2AC20, H2BC21
2	0.4997	1.0000	2.45	H2AC20, H2BC21
2	0.4997	1.0000	2.45	H2AC20, H2BC21
2	0.4997	1.0000	2.45	H2AC20, H2BC21
2	0.4997	1.0000	2.45	H2AC20, H2BC21
2	0.4997	1.0000	2.45	H2AC20, H2BC21
2	0.4997	1.0000	2.45	ITGB1, ITGB3
2	0.4997	1.0000	2.45	H2AC20, H2BC21
2	0.4997	1.0000	2.45	HLA-A, HLA-B
2	0.4997	1.0000	2.45	CFL1, HSP90AA1
2	0.4997	1.0000	2.45	YWHAB, YWHAE
2	0.4997	1.0000	2.45	CALM3, HSP90AA1
2	0.4997	1.0000	2.45	CALM3, HSP90AA1
2	0.4997	1.0000	2.45	HLA-A, UBE2L3
2	0.4997	1.0000	2.45	CALM3, SRI
2	0.4997	1.0000	2.45	DSG1, DSP
2	0.4997	1.0000	2.45	ITGB1, ITGB3
2	0.4997	1.0000	2.45	ITGB1, ITGB3
2	0.4997	1.0000	2.45	HLA-A, UBE2L3
2	0.4997	1.0000	2.45	CDC42, GNG5
2	0.4997	1.0000	2.45	DSG1, TFRC
2	0.4997	1.0000	2.45	PPIA, XRCC5
2	0.4997	1.0000	2.45	PPIA, XRCC5
2	0.4997	1.0000	2.45	SLC2A1, SLC2A3

2	0.4997	1.0000	2.45	CALM3, SLC2A3
2	0.4997	1.0000	2.45	CALM3, SRI
2	0.4997	1.0000	2.45	GSR, PRDX1
2	0.4997	1.0000	2.45	INPP5D, YWHAZ
2	0.4997	1.0000	2.45	HSP90AA1, HSPA8
2	0.4997	1.0000	2.45	CDC42, RHOA
2	0.4997	1.0000	2.45	CD36, CYBB
2	0.4997	1.0000	2.45	CALM3, HSP90AA1
2	0.4997	1.0000	2.45	CD36, GNAQ
2	0.4997	1.0000	2.45	CALM3, PPIA
2	0.4997	1.0000	2.45	F11R, RHOA
2	0.4997	1.0000	2.45	RHOA, RTN4
2	0.4997	1.0000	2.45	RHOA, RTN4
9	0.5178	1.0000	1.22	ANK1, CANX, CD59, NANS, RAB1B, SPTBN1, TUBA1C, TUBA4A, TUB
6	0.5647	1.0000	1.22	G6PD, GSR, PRDX1, PSMA5, PSMA6, TKT
6	0.5647	1.0000	1.22	HSP90AA1, HSPA8, TUBA1C, TUBA4A, TUBB1, VIM
6	0.5647	1.0000	1.22	HSP90AA1, HSPA8, TUBA1C, TUBA4A, TUBB1, VIM
6	0.5647	1.0000	1.22	M6PR, RAB1B, RAB33B, TUBA1C, TUBA4A, TUBB1
6	0.5647	1.0000	1.22	PSMA5, PSMA6, RCC2, TUBA1C, TUBA4A, TUBB1
6	0.5647	1.0000	1.22	PSMA5, PSMA6, RCC2, TUBA1C, TUBA4A, TUBB1
6	0.5647	1.0000	1.22	PSMA5, PSMA6, RCC2, TUBA1C, TUBA4A, TUBB1
6	0.5647	1.0000	1.22	ACTB, CALM3, LRP1, MSN, MYH9, SPTBN1
6	0.5647	1.0000	1.22	G6PD, GSR, PRDX1, PSMA5, PSMA6, TKT
6	0.5647	1.0000	1.22	HSP90AA1, HSPA1B, HSPA8, TUBA1C, TUBA4A, TUBB1
6	0.5647	1.0000	1.22	BCAP31, FLOT1, JUP, RHOA, STOM, TFRC
6	0.5647	1.0000	1.22	CLTA, PFN1, PSMA5, PSMA6, RAC2, RHOA
3	0.6471	1.0000	1.22	PSMA5, PSMA6, RAC2
3	0.6471	1.0000	1.22	CDC42, GNG5, RHOA
3	0.6471	1.0000	1.22	ATP2A3, CALM3, SRI
3	0.6471	1.0000	1.22	EEF1A1, HSP90AA1, YWHAE
3	0.6471	1.0000	1.22	H2AC20, H2BC21, NPM1
3	0.6471	1.0000	1.22	FLOT1, HSP90AA1, UBE2L3
3	0.6471	1.0000	1.22	FLOT1, HSP90AA1, UBE2L3
3	0.6471	1.0000	1.22	FLOT1, HSP90AA1, UBE2L3
3	0.6471	1.0000	1.22	RHOA, RTN4, YWHAE
3	0.6471	1.0000	1.22	H2AC20, H2BC21, TNKS1BP1
3	0.6471	1.0000	1.22	CD59, CPN1, FCN1
3	0.6471	1.0000	1.22	PSMA5, PSMA6, UBE2L3
3	0.6471	1.0000	1.22	ATP2A3, H2AC20, H2BC21
3	0.6471	1.0000	1.22	TUBA1C, TUBA4A, TUBB1
3	0.6471	1.0000	1.22	H2AC20, H2BC21, NPM1
3	0.6471	1.0000	1.22	H2AC20, H2BC21, NPM1
3	0.6471	1.0000	1.22	TUBA1C, TUBA4A, TUBB1
3	0.6471	1.0000	1.22	TUBA1C, TUBA4A, TUBB1
3	0.6471	1.0000	1.22	CLTA, HSPA8, M6PR
3	0.6471	1.0000	1.22	CLTA, HSPA8, TFRC
3	0.6471	1.0000	1.22	PSMA5, PSMA6, SLC2A1

3	0.6471	1.0000	1.22	ATP2A3, CALM3, SRI
3	0.6471	1.0000	1.22	ATP2A3, CALM3, SRI
3	0.6471	1.0000	1.22	ADH5, CYB5R3, PTGS1
3	0.6471	1.0000	1.22	B2M, HLA-A, HLA-B
3	0.6471	1.0000	1.22	TUBA1C, TUBA4A, TUBB1
3	0.6471	1.0000	1.22	TUBA1C, TUBA4A, TUBB1
3	0.6471	1.0000	1.22	FCER1G, PSMA5, PSMA6
3	0.6471	1.0000	1.22	INPP5D, PSMA5, PSMA6
3	0.6471	1.0000	1.22	CALM3, PSMA5, PSMA6
3	0.6471	1.0000	1.22	GNAI2, GNG5, ITGB1
3	0.6471	1.0000	1.22	HNRNPF, HNRNPH1, PTBP1
3	0.6471	1.0000	1.22	HNRNPF, HNRNPH1, PTBP1
3	0.6471	1.0000	1.22	CDC42, SPTBN1, TPM4
3	0.6471	1.0000	1.22	TUBA1C, TUBA4A, TUBB1
3	0.6471	1.0000	1.22	TUBA1C, TUBA4A, TUBB1
3	0.6471	1.0000	1.22	TUBA1C, TUBA4A, TUBB1
3	0.6471	1.0000	1.22	TUBA1C, TUBA4A, TUBB1
3	0.6471	1.0000	1.22	TUBA1C, TUBA4A, TUBB1
3	0.6471	1.0000	1.22	GNAI2, GNG5, ITGB1
3	0.6471	1.0000	1.22	HSP90AA1, HSPA1B, HSPA8
3	0.6471	1.0000	1.22	HSP90AA1, HSPA1B, HSPA8
3	0.6471	1.0000	1.22	ANK1, HSPA8, ITGB1
3	0.6471	1.0000	1.22	ACTB, ANK1, SPTBN1
3	0.6471	1.0000	1.22	B2M, HLA-A, HLA-B
3	0.6471	1.0000	1.22	CALM3, PHB, YWHAB
3	0.6471	1.0000	1.22	HNRNPF, HNRNPH1, PTBP1
3	0.6471	1.0000	1.22	ATP2A3, CALM3, SRI
3	0.6471	1.0000	1.22	INPP5D, ITGB3, PECAM1
3	0.6471	1.0000	1.22	LTF, S100A8, S100A9
3	0.6471	1.0000	1.22	ATP2A3, CALM3, SRI
7	0.6572	1.0000	0.97	EEF1A1, HSP90AA1, HSPA8, TUBA1C, TUBA4A, TUBB1, VIM
7	0.6572	1.0000	0.97	B2M, CD226, CD99, HLA-A, HLA-B, ICAM2, ITGB1
7	0.6572	1.0000	0.97	ANK1, CD59, RAB1B, SPTBN1, TUBA1C, TUBA4A, TUBB1
7	0.6572	1.0000	0.97	ANK1, CD59, RAB1B, SPTBN1, TUBA1C, TUBA4A, TUBB1
7	0.6572	1.0000	0.97	ANK1, CD59, RAB1B, SPTBN1, TUBA1C, TUBA4A, TUBB1
7	0.6572	1.0000	0.97	ACTB, ARPC5, CLTA, HSPA8, M6PR, TFRC, UBQLN1
7	0.6572	1.0000	0.97	CYBB, GSR, GSTP1, PRDX1, PRDX2, PRDX6, SOD1
11	0.6720	1.0000	0.90	CYBB, G6PD, GSR, GSTP1, PRDX1, PRDX2, PRDX6, PSMA5, PSMA6,
11	0.6720	1.0000	0.90	CCT2, CCT4, CCT8, HSP90AA1, TCP1, TUBA1C, TUBA4A, TUBB, TUB
11	0.6720	1.0000	0.90	B2M, DHX9, FLNA, HLA-A, HLA-B, HSPA1B, HSPA8, NPM1, TUBA1C,
8	0.7335	1.0000	0.80	CALM3, CCL5, CDC42, GNAI2, GNAQ, GNG5, ITGB1, RHOA
8	0.7335	1.0000	0.80	CALM3, CCL5, CDC42, GNAI2, GNAQ, GNG5, ITGB1, RHOA
8	0.7335	1.0000	0.80	AACS, CD36, INPP5D, M6PR, OSBPL2, PNPLA8, PTGS1, SACM1L
8	0.7335	1.0000	0.80	DHX9, FLNA, HSPA1B, HSPA8, NPM1, TUBA1C, TUBA4A, TUBB1
8	0.7335	1.0000	0.80	CALM3, CLTA, GNG5, PFN1, PSMA5, PSMA6, RAC2, RHOA
12	0.7346	1.0000	0.80	CANX, CLTA, HSP90AA1, HSPA1B, RPL11, RPL8, RPLP0, RPS15, RPS1
4	0.7516	1.0000	0.81	ADH5, CYB5R3, GSTP1, PTGS1

4	0.7516	1.0000	0.81	RAB10, RAB1B, RAB27B, YWHAE
4	0.7516	1.0000	0.81	CALM3, PSMA5, PSMA6, RAC2
4	0.7516	1.0000	0.81	CALM3, GNAI2, GNG5, HSP90AA1
4	0.7516	1.0000	0.81	BCAP31, HSP90AA1, HSPA8, NCL
4	0.7516	1.0000	0.81	EEF1A1, HSP90AA1, HSPA8, VIM
4	0.7516	1.0000	0.81	RHOA, RTN4, UBE2L3, YWHAE
4	0.7516	1.0000	0.81	BCAP31, DSG1, DSP, VIM
4	0.7516	1.0000	0.81	BCAP31, DSG1, DSP, VIM
4	0.7516	1.0000	0.81	RCC2, TUBA1C, TUBA4A, TUBB1
4	0.7516	1.0000	0.81	RCC2, TUBA1C, TUBA4A, TUBB1
4	0.7516	1.0000	0.81	CLTA, HSPA8, M6PR, TFRC
4	0.7516	1.0000	0.81	CALM3, FCER1G, PSMA5, PSMA6
4	0.7516	1.0000	0.81	ALDOA, ENO1, GAPDH, PGK1
4	0.7516	1.0000	0.81	INPP5D, PSMA5, PSMA6, PTPRJ
4	0.7516	1.0000	0.81	HSPA1B, HSPA8, PSMA5, PSMA6
4	0.7516	1.0000	0.81	CLTA, M6PR, TFRC, UBQLN1
4	0.7516	1.0000	0.81	CALM3, TUBA1C, TUBA4A, TUBB1
4	0.7516	1.0000	0.81	CALM3, TUBA1C, TUBA4A, TUBB1
4	0.7516	1.0000	0.81	TTLL12, TUBA1C, TUBA4A, TUBB1
4	0.7516	1.0000	0.81	CALM3, TUBA1C, TUBA4A, TUBB1
4	0.7516	1.0000	0.81	CDC42, FCER1G, RAC2, RHOA
4	0.7516	1.0000	0.81	CALM3, GNAI2, GNG5, MYH9
4	0.7516	1.0000	0.81	CALM3, GNAI2, GNG5, MYH9
4	0.7516	1.0000	0.81	CYBB, RAC2, S100A8, S100A9
4	0.7516	1.0000	0.81	RAB10, RAB1B, RAB27B, RAB33B
17	0.7878	1.0000	0.73	CASP14, DSC1, DSG1, DSP, JUP, KRT1, KRT10, KRT14, KRT15, KRT16
17	0.7878	1.0000	0.73	CASP14, DSC1, DSG1, DSP, JUP, KRT1, KRT10, KRT14, KRT15, KRT16
9	0.7951	1.0000	0.68	ANTXR2, B2M, CALM3, CD9, EEF2, ENO1, HSP90AA1, LTF, PGK1
5	0.8254	1.0000	0.60	RAB10, RAB1B, RAB27B, RAB33B, YWHAE
5	0.8254	1.0000	0.60	CALM3, FCER1G, ICAM2, PSMA5, PSMA6
5	0.8254	1.0000	0.60	CCT2, CCT6A, CCT7, HNRNPC, HSP90AA1
5	0.8254	1.0000	0.60	CD36, GNAI2, GNAQ, GNG5, SLC2A1
14	0.8316	1.0000	0.65	ATP5F1B, CALM3, CCT2, CCT4, CCT8, HSP90AA1, HSPA9, TCP1, TUE
34	0.8399	1.0000	0.71	ACTB, ADAM10, ANK1, CALM3, CANX, CD59, EEF1A1, EEF2, EIF5A,
10	0.8440	1.0000	0.59	ALDOA, CALM3, ENO1, G6PD, GALE, GAPDH, PGK1, PKM, SLC2A1,
10	0.8440	1.0000	0.59	ACTB, CCT2, CCT4, CCT6A, CCT7, CCT8, TCP1, TUBA1C, TUBA4A, TL
10	0.8440	1.0000	0.59	ACTB, CCT2, CCT4, CCT6A, CCT7, CCT8, TCP1, TUBA1C, TUBA4A, TL
31	0.8623	1.0000	0.67	CYBB, EEF1A1, G6PD, GSR, GSTP1, H2AC20, H2BC21, HSP90AA1, H
31	0.8623	1.0000	0.67	CYBB, EEF1A1, G6PD, GSR, GSTP1, H2AC20, H2BC21, HSP90AA1, H
6	0.8776	1.0000	0.48	EEF1A1, HSP90AA1, HSPA1B, HSPA8, HSPA9, YWHAE
6	0.8776	1.0000	0.48	CCT2, CCT6A, CCT7, HNRNPC, HSP90AA1, VIM
6	0.8776	1.0000	0.48	ATIC, MSN, MYH9, NPM1, TPM4, VCL
6	0.8776	1.0000	0.48	ATIC, MSN, MYH9, NPM1, TPM4, VCL
6	0.8776	1.0000	0.48	CALM3, GNAI2, GNG5, TUBA1C, TUBA4A, TUBB1
6	0.8776	1.0000	0.48	CD36, GNAI2, GNAQ, GNG5, SLC2A1, TKT
12	0.9117	1.0000	0.47	DHX9, HNRNPA2B1, HNRNPA3, HNRNPC, HNRNPF, HNRNPH1, HNR
12	0.9117	1.0000	0.47	DHX9, HNRNPA2B1, HNRNPA3, HNRNPC, HNRNPF, HNRNPH1, HNR

12	0.9117	1.0000	0.47	DHX9, HNRNPA2B1, HNRNPA3, HNRNPC, HNRNPF, HNRNPH1, HNR
12	0.9117	1.0000	0.47	ACTB, CCT2, CCT4, CCT6A, CCT7, CCT8, GNAQ, GNG5, TCP1, TUBA1
12	0.9117	1.0000	0.47	ACTB, CCT2, CCT4, CCT6A, CCT7, CCT8, GNAQ, GNG5, TCP1, TUBA1
7	0.9143	1.0000	0.39	DHX9, HSPA1B, HSPA8, NPM1, TUBA1C, TUBA4A, TUBB1
7	0.9143	1.0000	0.39	ACTB, CCT2, CCT4, CCT6A, CCT7, CCT8, TCP1
13	0.9343	1.0000	0.42	CDC42, PFN1, PSMA5, PSMA6, RHOA, RPL11, RPL8, RPLP0, RPS15,
8	0.9401	1.0000	0.34	ARL6IP5, CALM3, GNAI2, GNG5, HSPA8, TUBA1C, TUBA4A, TUBB1
27	0.9418	1.0000	0.51	DHX9, HNRNPA2B1, HNRNPA3, HNRNPC, HNRNPF, HNRNPH1, HNR
9	0.9582	1.0000	0.29	CCT2, CCT4, CCT6A, CCT7, CCT8, TCP1, TUBA1C, TUBA4A, TUBB1
9	0.9582	1.0000	0.29	HSP90AA1, RPL11, RPL8, RPLP0, RPS15, RPS15A, RPS23, RPS3A, RP
57	0.9611	1.0000	0.57	ACTB, ADAM10, ANK1, ANPEP, ATP5F1B, B2M, CALM3, CANX, CCT
10	0.9709	1.0000	0.26	ARL6IP5, CALM3, FLOT1, GNAI2, GNG5, HSPA8, RTN3, TUBA1C, TU
10	0.9709	1.0000	0.26	PSMA5, PSMA6, RPL11, RPL8, RPLP0, RPS15, RPS15A, RPS23, RPS3
43	0.9738	1.0000	0.49	AACS, ADH5, ALDOA, ATIC, ATP5F1B, CALM3, CD36, CYB5R3, ENO1
11	0.9798	1.0000	0.23	GSR, PSMA5, PSMA6, RPL11, RPL8, RPLP0, RPS15, RPS15A, RPS23,

SPD1, ITGA2B, KANSL3, NPM1, PRDX1, PRDX2, PSMA5, PSMA6, SLC2A3, THBS1, TNKS1BP1, YWHAB, YW

, ITGB1, MYH9, MYL12B, MYL6, PFN1, RAC2, RCC2, RHOA, S100A8, S100A9, TUBA1C, TUBA4A, TUBB1, Y'
LM3, CAMP, CANX, CCL5, CCT2, CCT8, CD226, CD36, CD47, CD53, CD59, CD99, CDC42, CFL1, CLTA, CPN1

RNPF, HSP90AA1, HSPA8, HSPA9, INPP5D, ITGB1, LBP, MSN, PPIA, PSMA5, PSMA6, RAP1B, RPLP0, SOD1,
T6A, CCT7, CDC42, CFL1, CLTA, CTSD, CYBB, DSG1, DSP, F11R, FLNA, FLOT1, GNAI2, GNAQ, GNG5, H2AC2
TSD, CYBB, HLA-A, HLA-B, ICAM2, INPP5D, ITGB1, PPIA, PSMA5, PSMA6, PTPRJ, RAP1B, S100A8, S100A9,
M1, PRDX1, PRDX2, PSMA5, PSMA6, SLC2A3, THBS1, TNKS1BP1, YWHAB, YWHAE, YWHAG, YWHAH, YW
M1, PRDX1, PRDX2, PSMA5, PSMA6, SLC2A3, THBS1, TNKS1BP1, YWHAB, YWHAE, YWHAG, YWHAH, YW

., HLA-A, HLA-B, HNRNPA2B1, HNRNPF, HSP90AA1, HSPA1B, HSPA8, HSPA9, INPP5D, ITGB1, LBP, MSN, N

CYBB, DSG1, DSP, FLNA, FLOT1, H2AC20, H2BC21, HNRNPC, HSP90AA1, ITGB1, JUP, MYH9, MYL12B, MYL
CYBB, DSG1, DSP, FLNA, FLOT1, H2AC20, H2BC21, HNRNPC, HSP90AA1, ITGB1, JUP, MYH9, MYL12B, MYL

CD99, CDC42, CFL1, ENDOD1, F11R, FCER1G, FERMT3, FLNA, GNAI2, GNAQ, GNG5, GP1BA, GP1BB, INPF

MP, CCT2, CCT8, CD36, CD47, CD53, CD59, CDC42, CFL1, CPN1, CTSD, CYB5R3, CYBB, DCD, DHX9, DSC1, I

NX, CD36, CD9, CDC42, CLTA, EEF1A1, EEF2, ENO1, G3BP1, GALE, GNAI2, GNG5, GP1BA, GP1BB, H2AC20,

M1, PPIA, RPS15, RPS15A, RPS23, RPS3A, RPSA, TUBB, YWHAB, YWHAE, YWHAG, YWHAH, YWHAZ
3, LRP1, M6PR, MYH9, RAB10, RAB1B, RAB27B, RAB33B, SPTBN1, TFRC, TUBA1C, TUBA4A, TUBB1, UBQL

G, FERMT3, FLNA, GNAI2, GNAQ, GNG5, GP1BA, GP1BB, ITGA2B, ITGB3, LGALS3BP, MMRN1, PECAM1, F

3, CD36, CD47, CD53, CD59, CTSD, CYB5R3, CYBB, DSC1, DSG1, DSP, EEF1A1, EEF2, FCER1G, FCN1, FLG2,
PA8, ITGA2B, ITGB1, ITGB3, MSN, MYH9, MYL12B, MYL6, PFN1, PSMA5, PSMA6, RHOA, RPL11, RPL8, RPL
PA8, ITGA2B, ITGB1, ITGB3, MSN, MYH9, MYL12B, MYL6, PFN1, PSMA5, PSMA6, RHOA, RPL11, RPL8, RPL
AB10, RAB1B, RAB27B, RAB33B, SPTBN1, TFRC, TUBA1C, TUBA4A, TUBB1, UBQLN1, YWHAB, YWHAE, YW

A-A, HLA-B, HSP90AA1, HSPA1B, HSPA8, ITGB1, NCL, NPM1, PPIA, PSMA5, PSMA6, RPL11, RPL8, RPLP0, R

CLTA, EEF1A1, EEF2, ENO1, G3BP1, GNAI2, GNG5, H2AC20, H2BC21, HLA-A, HLA-B, HSP90AA1, HSPA1B,

DSC1, DSG1, DSP, H2AC20, H2BC21, HSP90AA1, HSPA8, ITGA2B, ITGB1, ITGB3, JUP, KRT1, KRT10, KRT14

A, ITGA2B, ITGB3, LGALS3BP, MMRN1, PECAM1, PFN1, PLEK, PPIA, QSOX1, RAB27B, SOD1, SRGN, TAGLN
A, ITGA2B, ITGB3, LGALS3BP, MMRN1, PECAM1, PFN1, PLEK, PPIA, QSOX1, RAB27B, SOD1, SRGN, TAGLN

H2AC20, H2BC21, HLA-A, HNRNPC, HSPA8, NANS, NPM1, PSMA5, PSMA6, QSOX1, RAB10, RAB1B, RAB2

SPA1B, HSPA8, HSPA9, PRDX1, PRDX2, PRDX6, PSMA5, PSMA6, RPL11, RPL8, RPLP0, RPS15, RPS15A, RPS

SPA1B, HSPA8, HSPA9, PRDX1, PRDX2, PRDX6, PSMA5, PSMA6, RPL11, RPL8, RPLP0, RPS15, RPS15A, RPS

ꞦNPU, HSPA1B, HSPA8, NCL, PCBP1, PSMA5, PSMA6, PTBP1, RPL11, RPL8, RPLP0, RPS15, RPS15A, RPS23,

2, CCT4, CCT6A, CCT7, CCT8, CD59, CTSD, EEF1A1, EEF2, EIF5A, GNAQ, GNG5, H2AC20, H2BC21, HLA-A, I

., G6PD, GALE, GAPDH, GNAI2, GNAQ, GNG5, GSR, GSTP1, HMBS, HSP90AA1, INPP5D, LDHA, LDHB, LRP1

1, CTSD, CYB5R3, CYBB, DCD, DHX9, DSC1, DSG1, DSP, EEF1A1, EEF2, FCER1G, FCN1, FLG2, FLNA, FSCN1,

20, H2BC21, HNRNPC, HNRNPF, HNRNPH1, HSP90AA1, ITGA2B, ITGB1, ITGB3, JUP, MYH9, MYL12B, MYL6

NPM1, PPIA, PSMA5, PSMA6, PTPRJ, RAP1B, RPLP0, SOD1, TCP1, TUBA1C, TUBA4A, TUBB1, VIM, YWHAZ

.6, PFN1, RAC2, RCC2, RHOA, S100A8, S100A9, SPTBN1, STOM, TFRC, TPM4, TUBA1C, TUBA4A, TUBB1, V

.6, PFN1, RAC2, RCC2, RHOA, S100A8, S100A9, SPTBN1, STOM, TFRC, TPM4, TUBA1C, TUBA4A, TUBB1, V

P5D, ITGA2B, ITGA6, ITGB1, ITGB3, LGALS3BP, MMRN1, PECAM1, PF4V1, PFN1, PLEK, PPIA, QSOX1, RAB2

DSG1, DSP, EEF1A1, EEF2, FCER1G, FCN1, FLG2, GSTP1, HLA-B, HRNR, HSP90AA1, HSPA1B, HSPA8, ICAM

, H2BC21, HLA-A, HLA-B, HSP90AA1, HSPA1B, HSPA8, ITGA2B, ITGB1, ITGB3, LTF, MSN, MYH9, NCL, NPM

PFN1, PLEK, PPIA, QSOX1, RAB27B, RAC2, RAP1B, RHOA, SOD1, SRGN, TAGLN2, THBS1, TLN1, TUBA4A, V

GSTP1, HLA-B, HRNR, HSP90AA1, HSPA1B, HSPA8, JUP, KRT1, LTF, LYZ, PECAM1, PKM, PPIA, PRDX6, PSM

RPS15, RPS15A, RPS23, RPS3A, RPSA, TUBA1C, TUBA4A, TUBB, TUBB1, XRCC5, YWHAB, YWHAE, YWHAG,

HSPA8, ITGB1, LTF, MYH9, NCL, NPM1, PGK1, PPIA, PSMA5, PSMA6, RPL11, RPL8, RPLP0, RPS15, RPS15

, KRT15, KRT16, KRT17, KRT2, KRT5, KRT6A, KRT6B, KRT78, KRT9, MSN, MYH9, MYL12B, MYL6, PFN1, PS

7B, RAB33B, RHOA, RPL8, RPS23, SPTBN1, THBS1, TTLL12, TUBA1C, TUBA4A, TUBB1, UBE2L3, VDAC2

HNRNPC, HSPA8, HSPA9, HSPD1, LTF, LYZ, NANS, NPM1, PSMA5, PSMA6, QSOX1, RAB10, RAB1B, RAB27

, M6PR, OSBPL2, PGK1, PKM, PNPLA8, PSMA5, PSMA6, PTGS1, RPL11, RPL8, RPLP0, RPS15, RPS15A, RP

GSTP1, HLA-A, HLA-B, HNRNPA2B1, HNRNPF, HRNR, HSP90AA1, HSPA1B, HSPA8, HSPA9, ICAM2, INPP5I

5, PFN1, PHB, PSMA5, PSMA6, PTBP1, PTPRJ, RAC2, RAP1B, RCC2, RHOA, RTN4, S100A8, S100A9, SPTBN

27B, RAC2, RAP1B, RHOA, SELPLG, SOD1, SPN, SRGN, SRI, TAGLN2, THBS1, TLN1, TUBA1C, TUBA4A, TUBI

2, JUP, KRT1, LBP, LTF, LYZ, MYH9, PECAM1, PKM, PPIA, PRDX6, PSMA5, PSMA6, PTPRJ, QSOX1, RAB10,

I1, PGK1, PHB, PPIA, PRDX1, PRDX2, PSMA5, PSMA6, RAC2, RAP1B, RPL11, RPL8, RPLP0, RPS15, RPS15A,

MA5, PTPRJ, QSOX1, RAB10, RAP1B, RHOA, S100A8, S100A9, SLC2A3, STOM, TSPAN14, TUBB, VCL, XRCC

A, RPS23, RPS3A, RPSA, TUBA1C, TUBA4A, TUBB, TUBB1, XRCC5, YWHAB, YWHAE, YWHAG, YWHAH, YW

MA5, PSMA6, RHOA, RPL11, RPL8, RPLP0, RPS15, RPS15A, RPS23, RPS3A, RPSA, SPTBN1, TLN1, TNKS1B

B, RAB33B, RHOA, RPL11, RPL8, RPLP0, RPS15, RPS15A, RPS23, RPS3A, RPSA, SPTBN1, TCP1, THBS1, TSP

D, ITGB1, JUP, KRT1, LBP, LTF, LYZ, MSN, MYH9, NPM1, PECAM1, PKM, PPIA, PRDX6, PSMA5, PSMA6, PT

1, STOM, TFRC, THBS1, TLN1, TPM4, TUBA1C, TUBA4A, TUBB1, UBE2L3, VCL, VIM, YWHAB, YWHAE, YWI

, RPS23, RPS3A, RPSA, S100A8, S100A9, SLC2A1, SPTBN1, THBS1, TLN1, TPM4, TUBA1C, TUBA4A, TUBB,

PRJ, QSOX1, RAB10, RAC2, RAP1B, RHOA, RPLP0, S100A8, S100A9, SLC2A3, SOD1, STOM, TCP1, TSPAN1

4, TUBA1C, TUBA4A, TUBB, TUBB1, UBE2L3, VCL, VIM, XRCC5, YWHAB, YWHAZ

Table 9. REACTOME pathways for which the Pathway Activation Score (PAS) values were (

REACTOME ID	REACTOME pathway	arbitrary category
R-HSA-5627123	RHO GTPases activate PAKs	cancer-related
R-HSA-76009	Platelet Aggregation (Plug Formation)	cancer-related
R-HSA-5674135	MAP2K and MAPK activation	cancer-related
R-HSA-6802948	Signaling by high-kinase activity BRAF mutants	cancer-related
R-HSA-5625740	RHO GTPases activate PKNs	cancer-related
R-HSA-6802952	Signaling by BRAF and RAF1 fusions	cancer-related
R-HSA-9656223	Signaling by RAF1 mutants	cancer-related
R-HSA-373755	Semaphorin interactions	other
R-HSA-5339562	Uptake and actions of bacterial toxins	immune-related
R-HSA-445355	Smooth Muscle Contraction	other
R-HSA-5626467	RHO GTPases activate IQGAPs	cancer-related
R-HSA-157858	Gap junction trafficking and regulation	other
R-HSA-190828	Gap junction trafficking	other
R-HSA-2682334	EPH-Ephrin signaling	cancer-related
R-HSA-5627117	RHO GTPases Activate ROCKs	cancer-related
R-HSA-1445148	Translocation of SLC2A4 (GLUT4) to the plasma membrar	other
R-HSA-6802957	Oncogenic MAPK signaling	cancer-related
R-HSA-6802946	Signaling by moderate kinase activity BRAF mutants	cancer-related
R-HSA-6802949	Signaling by RAS mutants	cancer-related
R-HSA-6802955	Paradoxical activation of RAF signaling by kinase inactive	cancer-related
R-HSA-9649948	Signaling downstream of RAS mutants	cancer-related
R-HSA-983170	Antigen Presentation: Folding, assembly and peptide loac	immune-related
R-HSA-9646399	Aggrephagy	other
R-HSA-216083	Integrin cell surface interactions	cancer-related
R-HSA-437239	Recycling pathway of L1	other
R-HSA-76002	Platelet activation, signaling and aggregation	cancer-related
R-HSA-111447	Activation of BAD and translocation to mitochondria	cancer-related
R-HSA-114452	Activation of BH3-only proteins	cancer-related
R-HSA-9755779	SARS-CoV-2 targets host intracellular signalling and regul	immune-related
R-HSA-2029482	Regulation of actin dynamics for phagocytic cup formatic	cancer-related
R-HSA-75035	Chk1/Chk2(Cds1) mediated inactivation of Cyclin B:Cdk1	cancer-related
R-HSA-9735871	SARS-CoV-1 targets host intracellular signalling and regul	immune-related
R-HSA-3371497	HSP90 chaperone cycle for steroid hormone receptors (S	other
R-HSA-6809371	Formation of the cornified envelope	cancer-related
R-HSA-373760	L1CAM interactions	cancer-related
R-HSA-390466	Chaperonin-mediated protein folding	other
R-HSA-391251	Protein folding	other
R-HSA-6798695	Neutrophil degranulation	immune-related
R-HSA-114608	Platelet degranulation	cancer-related
R-HSA-76005	Response to elevated platelet cytosolic Ca2+	cancer-related
R-HSA-9692914	SARS-CoV-1-host interactions	immune-related
R-HSA-389960	Formation of tubulin folding intermediates by CCT/TriC	cancer-related
R-HSA-9678108	SARS-CoV-1 Infection	immune-related
R-HSA-389958	Cooperation of Prefoldin and TriC/CCT in actin and tubul	cancer-related
R-HSA-389957	Prefoldin mediated transfer of substrate to CCT/TriC	cancer-related
R-HSA-3928662	EPHB-mediated forward signaling	immune-related
R-HSA-8950505	Gene and protein expression by JAK-STAT signaling after	immune-related

R-HSA-9020591	Interleukin-12 signaling	immune-related
R-HSA-447115	Interleukin-12 family signaling	immune-related
R-HSA-5628897	TP53 Regulates Metabolic Genes	cancer-related

different in between the MP and HD groups.

p.value	FDR	effect size	MP vs. HD	# all protei	# detected prote
0.0002	0.0103	very large	UP	24	6
0.0005	0.0239	very large	UP	39	6
0.0015	0.0722	very large	UP	40	7
0.0015	0.0722	very large	UP	36	7
0.0039	0.1788	very large	UP	94	11
0.0052	0.2338	very large	UP	67	8
0.0052	0.2338	very large	UP	43	8
0.0147	0.6317	large	UP	65	8
0.0149	0.6317	large	DOWN	38	5
0.0171	0.7007	large	UP	45	7
0.0172	0.7007	large	UP	32	6
0.0255	0.9955	large	UP	49	5
0.0255	0.9955	large	UP	47	5
0.0255	0.9955	large	UP	92	10
0.0336	1.0000	large	UP	20	5
0.0433	1.0000	large	UP	72	12
0.0524	1.0000	large	UP	84	9
0.0524	1.0000	large	UP	47	9
0.0524	1.0000	large	UP	47	9
0.0524	1.0000	large	UP	47	9
0.0524	1.0000	large	UP	47	9
0.0585	1.0000	large	UP	29	5
0.0660	1.0000	medium	UP	43	5
0.0892	1.0000	medium	UP	85	9
0.1230	1.0000	medium	UP	47	6
0.1431	1.0000	medium	UP	263	37
0.1431	1.0000	medium	UP	15	5
0.1431	1.0000	medium	UP	30	5
0.1431	1.0000	medium	UP	12	5
0.1431	1.0000	medium	UP	61	6
0.1431	1.0000	medium	UP	13	5
0.1431	1.0000	medium	UP	15	5
0.1720	1.0000	medium	UP	55	6
0.2116	1.0000	medium	DOWN	129	17
0.2176	1.0000	medium	UP	119	12
0.3849	1.0000	small	UP	90	12
0.3849	1.0000	small	UP	96	12
0.3935	1.0000	small	DOWN	480	52
0.4813	1.0000	small	UP	129	26
0.4813	1.0000	small	UP	134	26
0.5036	1.0000	small	DOWN	98	13
0.5184	1.0000	small	DOWN	25	9
0.6082	1.0000	small	UP	144	14
0.6569	1.0000	small	UP	32	10
0.6569	1.0000	small	UP	28	10
0.7318	1.0000	small	UP	42	5
0.8311	1.0000	no effect	DOWN	38	12

0.8311	1.0000	no effect	DOWN	47	12
0.8521	1.0000	no effect	DOWN	57	13
0.9054	1.0000	no effect	UP	87	9

Protein Name

CALM3, CDC42, FLNA, MYH9, MYL12B, MYL6
GP1BA, GP1BB, ITGA2B, ITGB3, RAP1B, TLN1
ACTB, ITGA2B, ITGB3, RAP1B, TLN1, VCL, YWHAB
ACTB, ITGA2B, ITGB3, RAP1B, TLN1, VCL, YWHAB
H2AC20, H2BC21, MYH9, MYL12B, MYL6, RHOA, YWHAB, YWHAE, YWHAG, YWHAH, YWHAZ
ACTB, CALM3, ITGA2B, ITGB3, RAP1B, TLN1, VCL, YWHAB
ACTB, CALM3, ITGA2B, ITGB3, RAP1B, TLN1, VCL, YWHAB
CFL1, HSP90AA1, ITGB1, MYH9, MYL12B, MYL6, RHOA, TLN1
ANTXR2, CALM3, CD9, EEF2, HSP90AA1
ANXA2, CALM3, MYL12B, MYL6, TLN1, TPM4, VCL
ACTB, CALM3, CDC42, TUBA1C, TUBA4A, TUBB1
ACTB, CLTA, TUBA1C, TUBA4A, TUBB1
ACTB, CLTA, TUBA1C, TUBA4A, TUBB1
ACTB, ADAM10, ARPC5, CDC42, CFL1, CLTA, MYH9, MYL12B, MYL6, RHOA
CFL1, MYH9, MYL12B, MYL6, RHOA
ACTB, CALM3, MYH9, RAB10, TUBA1C, TUBA4A, TUBB1, YWHAB, YWHAE, YWHAG, YWHAH, YWHAZ
ACTB, CALM3, ITGA2B, ITGB3, PHB, RAP1B, TLN1, VCL, YWHAB
ACTB, CALM3, ITGA2B, ITGB3, PHB, RAP1B, TLN1, VCL, YWHAB
ACTB, CALM3, ITGA2B, ITGB3, PHB, RAP1B, TLN1, VCL, YWHAB
ACTB, CALM3, ITGA2B, ITGB3, PHB, RAP1B, TLN1, VCL, YWHAB
ACTB, CALM3, ITGA2B, ITGB3, PHB, RAP1B, TLN1, VCL, YWHAB
B2M, BCAP31, CANX, HLA-A, HLA-B
HSP90AA1, TUBA1C, TUBA4A, TUBB1, VIM
CD47, F11R, ICAM2, ITGA2B, ITGA6, ITGB1, ITGB3, PECAM1, THBS1
ACTB, CLTA, MSN, TUBA1C, TUBA4A, TUBB1
ALDOA, ANXA5, CALM3, CD36, CD9, CDC42, CFL1, ENDOD1, FCER1G, FERMT3, FLNA, GNAI2, GNAQ, GNG5, (
YWHAB, YWHAE, YWHAG, YWHAH, YWHAZ
YWHAB, YWHAE, YWHAG, YWHAH, YWHAZ
YWHAB, YWHAE, YWHAG, YWHAH, YWHAZ
ACTB, ARPC5, CDC42, CFL1, HSP90AA1, MYH9
YWHAB, YWHAE, YWHAG, YWHAH, YWHAZ
YWHAB, YWHAE, YWHAG, YWHAH, YWHAZ
HSP90AA1, HSPA1B, HSPA8, TUBA1C, TUBA4A, TUBB1
CASP14, DSC1, DSG1, DSP, JUP, KRT1, KRT10, KRT14, KRT15, KRT16, KRT17, KRT2, KRT5, KRT6A, KRT6B, KRT
ACTB, ANK1, CLTA, HSPA8, ITGA2B, ITGB1, ITGB3, MSN, SPTBN1, TUBA1C, TUBA4A, TUBB1
ACTB, CCT2, CCT4, CCT6A, CCT7, CCT8, GNAQ, GNG5, TCP1, TUBA1C, TUBA4A, TUBB1
ACTB, CCT2, CCT4, CCT6A, CCT7, CCT8, GNAQ, GNG5, TCP1, TUBA1C, TUBA4A, TUBB1
ADAM10, ALDOA, ANPEP, ANXA2, ARPC5, B2M, CAMP, CCT2, CCT8, CD36, CD47, CD53, CD59, CTSD, CYB5R3
ALDOA, ANXA5, CALM3, CD36, CD9, CFL1, ENDOD1, FERMT3, FLNA, ITGA2B, ITGB3, LGALS3BP, MMRN1, PE(
ALDOA, ANXA5, CALM3, CD36, CD9, CFL1, ENDOD1, FERMT3, FLNA, ITGA2B, ITGB3, LGALS3BP, MMRN1, PE(
EEF1A1, NPM1, PPIA, RPS15, RPS15A, RPS23, RPS3A, RPSA, YWHAB, YWHAE, YWHAG, YWHAH, YWHAZ
CCT2, CCT4, CCT6A, CCT7, CCT8, TCP1, TUBA1C, TUBA4A, TUBB1
CANX, EEF1A1, NPM1, PPIA, RPS15, RPS15A, RPS23, RPS3A, RPSA, YWHAB, YWHAE, YWHAG, YWHAH, YWH/
ACTB, CCT2, CCT4, CCT6A, CCT7, CCT8, TCP1, TUBA1C, TUBA4A, TUBB1
ACTB, CCT2, CCT4, CCT6A, CCT7, CCT8, TCP1, TUBA1C, TUBA4A, TUBB1
ACTB, ARPC5, CDC42, CFL1, RHOA
ANXA2, CDC42, CFL1, HNRNPA2B1, HNRNPF, HSPA9, MSN, PPIA, RAP1B, RPLP0, SOD1, TCP1

ANXA2, CDC42, CFL1, HNRNPA2B1, HNRNPF, HSPA9, MSN, PPIA, RAP1B, RPLP0, SOD1, TCP1
ANXA2, CANX, CDC42, CFL1, HNRNPA2B1, HNRNPF, HSPA9, MSN, PPIA, RAP1B, RPLP0, SOD1, TCP1
G6PD, GSR, PRDX1, PRDX2, YWHAB, YWHAE, YWHAG, YWHAH, YWHAZ

GP1BA, GP1BB, ITGA2B, ITGB3, LGALS3BP, MMRN1, PECAM1, PFN1, PLEK, PPIA, QSOX1, RAB27B, RAC2, F

3, CYBB, DSC1, DSG1, DSP, EEF1A1, EEF2, FCER1G, FCN1, FLG2, GSTP1, HLA-B, HRNR, HSP90AA1, HSPA1B,

, HSPA8, JUP, KRT1, LTF, LYZ, PECAM1, PKM, PPIA, PRDX6, PSMA5, PTPRJ, QSOX1, RAB10, RAP1B, RHOA, S

S100A8, S100A9, SLC2A3, STOM, TSPAN14, TUBB, VCL, XRCC5

Table 10. Proteins identified in CD3(-) fraction of plasma sEV

ProteinName	Protein Description	HD (median)	HD number of observations	MP (median)
**AACS**	Acetoacetyl-CoA synthetase	1.65E+06	7	2.68E+06
**ABI3BP**	Target of Nesh-SH3	1.19E+06	10	2.38E+06
**ACAN**	Aggrecan core protein	5.98E+05	3	2.29E+06
**ACTB**	Actin, cytoplasmic 1	7.48E+07	10	2.19E+08
**ACTG2**	Actin, gamma-enteric smooth muscle	1.23E+06	10	1.75E+06
**ACTN1**	Alpha-actinin-1	2.79E+04	2	8.43E+03
**ADAM10**	Disintegrin and metalloproteinase domain-co	5.03E+05	7	1.11E+06
**ADIPOQ**	Adiponectin	1.16E+07	10	9.73E+06
**ALDOA**	Fructose-bisphosphate aldolase A	1.37E+06	10	3.74E+06
**ANK1**	Ankyrin-1	5.86E+05	9	4.01E+05
**ANPEP**	Aminopeptidase N	2.32E+06	10	1.80E+06
**ANTXR2**	Anthrax toxin receptor 2	5.39E+05	4	
**ANXA11**	Annexin A11	1.30E+05	4	8.01E+05
**ANXA2**	Annexin A2	3.65E+05	5	5.06E+05
**ANXA5**	Annexin A5	2.36E+06	6	2.71E+06
**ANXA7**	Annexin A7	2.51E+06	7	3.03E+06
**ARL6IP5**	PRA1 family protein 3	4.21E+04	2	1.21E+05
**ARPC5**	Actin-related protein 2/3 complex subunit 5	8.15E+05	5	1.16E+06
**ATP2A3**	Sarcoplasmic/endoplasmic reticulum calcium	1.58E+06	10	5.89E+06
**ATP5F1B**	ATP synthase subunit beta, mitochondrial	6.32E+04	3	2.14E+04
**B2M**	Beta-2-microglobulin	2.32E+05	1	1.58E+07
**BCAP31**	B-cell receptor-associated protein 31	3.56E+05	2	7.22E+05
**C16orf54**	Transmembrane protein C16orf54	3.39E+05	10	6.93E+05
**C1orf56**	Protein MENT	1.99E+06	10	2.03E+06
**CAMP**	Cathelicidin antimicrobial peptide	3.98E+07	10	2.12E+07
**CANX**	Calnexin	1.11E+06	9	1.67E+06
**CASP14**	Caspase-14	5.32E+05	1	6.77E+05
**CAVIN2**	Caveolae-associated protein 2	3.20E+06	10	2.54E+07
**CCL5**	C-C motif chemokine 5	4.98E+04	3	1.50E+05
**CCT2**	T-complex protein 1 subunit beta	8.58E+04	4	1.03E+05
**CCT4**	T-complex protein 1 subunit delta		0	2.53E+04
**CCT6A**	T-complex protein 1 subunit zeta	8.84E+04	2	
**CCT7**	T-complex protein 1 subunit eta	4.42E+04	4	1.68E+04
**CCT8**	T-complex protein 1 subunit theta		0	1.91E+05
**CD151**	CD151 antigen	1.33E+06	10	2.59E+06
**CD226**	CD226 antigen		0	5.19E+05
**CD36**	Platelet glycoprotein 4	6.42E+06	10	2.04E+07
**CD47**	Leukocyte surface antigen CD47	1.62E+05	3	2.65E+06
**CD53**	Leukocyte surface antigen CD53	8.39E+05	9	1.11E+06
**CD59**	CD59 glycoprotein	1.79E+06	10	2.62E+06

**CD9**	CD9 antigen	1.22E+07	10	3.11E+07
**CD99**	CD99 antigen	1.68E+06	8	1.14E+06
**CDC42**	Cell division control protein 42 homolog	2.51E+06	10	8.02E+06
**CDCA2**	Cell division cycle-associated protein 2	3.11E+06	10	3.05E+06
**CELSR2**	Cadherin EGF LAG seven-pass G-type recepto	3.01E+07	10	2.19E+07
**CFL1**	Cofilin-1	2.19E+05	8	7.53E+05
**CLDN5**	Claudin-5	9.58E+04	7	2.78E+05
**CLIC1**	Chloride intracellular channel protein 1	3.86E+05	1	4.00E+05
**CLTA**	Clathrin light chain A	7.22E+05	2	4.54E+05
**CLTCL1**	Clathrin heavy chain 2	8.84E+06	5	4.35E+05
**CMTM5**	CKLF-like MARVEL transmembrane domain-containing pr		0	7.87E+05
**CMTM6**	CKLF-like MARVEL transmembrane domain-containing pr		0	1.14E+05
**COL1A1**	Collagen alpha-1(I) chain	4.58E+07	10	4.52E+07
**COL1A2**	Collagen alpha-2(I) chain	3.43E+06	10	5.99E+06
**COL2A1**	Collagen alpha-1(II) chain	1.60E+05	8	1.29E+06
**COL6A1**	Collagen alpha-1(VI) chain	1.02E+06	9	7.87E+05
**COL6A3**	Collagen alpha-3(VI) chain	1.70E+06	10	7.00E+05
**COL7A1**	Collagen alpha-1(VII) chain	3.55E+05	4	2.04E+05
**COLEC11**	Collectin-11	1.47E+05	10	1.31E+05
**CPN1**	Carboxypeptidase N catalytic chain	8.04E+06	10	4.97E+06
**CSDE1**	Cold shock domain-containing protein E1	1.28E+04	1	6.28E+04
**CTSD**	Cathepsin D		0	5.61E+05
**CYB5R3**	NADH-cytochrome b5 reductase 3	8.11E+06	6	2.92E+06
**CYBB**	Cytochrome b-245 heavy chain	1.91E+05	6	1.20E+05
**DBH**	Dopamine beta-hydroxylase	2.60E+05	1	1.12E+06
**DCD**	Dermcidin	4.09E+05	7	3.97E+05
**DCDC1**	Doublecortin domain-containing protein 1	2.76E+07	6	6.03E+06
**DDX60L**	Probable ATP-dependent RNA helicase DDX6	7.69E+05	5	3.40E+05
**DHX9**	ATP-dependent RNA helicase A	2.51E+04	5	7.10E+03
**DPP6**	Dipeptidyl aminopeptidase-like protein 6	6.73E+05	3	4.04E+05
**DSC1**	Desmocollin-1	1.45E+06	5	1.42E+06
**DSG1**	Desmoglein-1	3.94E+05	4	2.94E+05
**DSP**	Desmoplakin	6.65E+05	1	
**EEF1A1**	Elongation factor 1-alpha 1	2.71E+05	4	3.73E+05
**EFEMP1**	EGF-containing fibulin-like extracellular matr	4.03E+05	9	5.68E+05
**EIF5A**	Eukaryotic translation initiation factor 5A-1	4.24E+05	1	4.64E+04
**ENDOD1**	Endonuclease domain-containing 1 protein	2.97E+05	4	3.78E+05
**ENO1**	Alpha-enolase	3.63E+05	8	4.02E+05
**EPB42**	Protein 4.2	7.27E+05	7	4.81E+05
**F11R**	Junctional adhesion molecule A	5.90E+05	9	2.45E+06
**FBN1**	Fibrillin-1	7.49E+05	5	1.15E+06
**FCER1G**	1 affinity immunoglobulin epsilon receptor s	3.77E+06	10	4.16E+06
**FCN1**	Ficolin-1	1.37E+06	10	4.47E+05
**FERMT3**	Fermitin family homolog 3	5.18E+06	10	5.47E+06
**FLG2**	Filaggrin-2	1.22E+06	2	1.35E+05
**FLNA**	Filamin-A	5.35E+07	10	1.43E+08
**FLOT1**	Flotillin-1	7.03E+04	3	7.21E+05

**FLOT2**	Flotillin-2		0	1.66E+06
**FSCN1**	Fascin	7.77E+04	3	4.02E+04
**GALE**	UDP-glucose 4-epimerase	1.60E+07	10	8.35E+06
**GAPDH**	Glyceraldehyde-3-phosphate dehydrogenase	2.65E+06	10	7.65E+06
**GNAI2**	Guanine nucleotide-binding protein G(i) subu	1.73E+06	10	2.70E+06
**GNAQ**	Guanine nucleotide-binding protein G(q) sub	3.58E+05	9	1.13E+06
**GNB4**	Guanine nucleotide-binding protein subunit	3.65E+06	5	1.88E+06
**GNG5**	Guanine nucleotide-binding protein G(I)/G(S)	9.68E+05	5	2.55E+06
**GP1BA**	Platelet glycoprotein Ib alpha chain	3.86E+06	10	1.70E+07
**GP1BB**	Platelet glycoprotein Ib beta chain	1.71E+06	10	1.14E+07
**GP9**	Platelet glycoprotein IX	1.62E+06	10	1.03E+07
**GSDMA**	Gasdermin-A	1.75E+05	2	
**GSR**	Glutathione reductase, mitochondrial	1.70E+05	1	3.26E+05
**GYPA**	Glycophorin-A	1.80E+06	2	3.92E+05
**H2AC20**	Histone H2A type 2-C	9.72E+05	10	4.77E+05
**H2BC21**	Histone H2B type 2-E	2.16E+06	8	8.00E+05
**H3-3A**	Histone H3.3	1.29E+06	5	6.52E+05
**H4C1**	Histone H4	1.91E+06	9	2.94E+05
**HGFAC**	Hepatocyte growth factor activator	9.05E+05	5	3.31E+05
**HINT1**	Histidine triad nucleotide-binding protein 1	2.05E+05	2	
**HLA-A**	HLA class I histocompatibility antigen, A alph	6.94E+06	10	1.20E+07
**HLA-B**	HLA class I histocompatibility antigen, B alph	5.01E+06	10	9.99E+06
**HMBS**	Porphobilinogen deaminase		0	5.03E+05
**HNRNPA1L2**	Heterogeneous nuclear ribonucleoprotein A1	4.21E+05	2	
**HNRNPA2B1**	Heterogeneous nuclear ribonucleoproteins A	7.17E+05	1	
**HNRNPC**	Heterogeneous nuclear ribonucleoproteins C	1.25E+05	1	
**HNRNPH3**	Heterogeneous nuclear ribonucleoprotein H3	4.50E+05	2	2.58E+05
**HNRNPU**	Heterogeneous nuclear ribonucleoprotein U	4.66E+05	2	
**HRNR**	Hornerin	5.57E+05	10	1.68E+06
**HSP90AA1**	Heat shock protein HSP 90-alpha	3.00E+05	8	1.59E+05
**HSPA1B**	Heat shock 70 kDa protein 1B	5.60E+04	3	1.17E+05
**HSPA8**	Heat shock cognate 71 kDa protein	1.54E+06	10	1.57E+06
**HSPA9**	Stress-70 protein, mitochondrial	3.32E+05	1	1.32E+06
**HSPD1**	60 kDa heat shock protein, mitochondrial	9.17E+05	2	8.17E+03
**ICAM2**	Intercellular adhesion molecule 2	3.21E+05	4	2.08E+06
**IFITM2**	Interferon-induced transmembrane protein 2	8.41E+05	9	9.45E+05
**ILK**	Integrin-linked protein kinase	1.72E+06	10	1.28E+06
**INPP5D**	Phosphatidylinositol 3,4,5-trisphosphate 5-p	1.22E+06	5	4.02E+06
**ITGA2**	Integrin alpha-2	2.85E+05	6	2.06E+05
**ITGA2B**	Integrin alpha-IIb	5.00E+07	10	1.07E+08
**ITGA6**	Integrin alpha-6	1.92E+06	10	4.84E+06
**ITGB1**	Integrin beta-1	3.73E+06	10	7.21E+06
**ITGB3**	Integrin beta-3	3.22E+07	10	7.67E+07
**JUP**	Junction plakoglobin	4.91E+05	1	
**KANSL3**	KAT8 regulatory NSL complex subunit 3	2.48E+05	1	2.85E+05
**KIAA2026**	Uncharacterized protein KIAA2026	1.01E+07	10	6.75E+06
**KPRP**	Keratinocyte proline-rich protein	4.30E+05	2	5.15E+05

**KRT1**	Keratin, type II cytoskeletal 1	1.63E+08	10	1.84E+08
**KRT10**	Keratin, type I cytoskeletal 10	4.53E+07	10	6.47E+07
**KRT14**	Keratin, type I cytoskeletal 14	1.54E+07	10	3.15E+06
**KRT15**	Keratin, type I cytoskeletal 15	4.73E+05	1	
**KRT16**	Keratin, type I cytoskeletal 16	1.17E+07	5	5.84E+06
**KRT17**	Keratin, type I cytoskeletal 17	4.90E+05	2	5.21E+05
**KRT2**	Keratin, type II cytoskeletal 2 epidermal	2.55E+07	10	4.62E+07
**KRT24**	Keratin, type I cytoskeletal 24	4.21E+06	9	1.20E+06
**KRT32**	Keratin, type I cuticular Ha2	2.34E+06	2	1.02E+06
**KRT5**	Keratin, type II cytoskeletal 5	4.64E+07	10	3.43E+07
**KRT6A**	Keratin, type II cytoskeletal 6A	8.30E+05	9	6.42E+05
**KRT6B**	Keratin, type II cytoskeletal 6B	3.08E+05	4	2.11E+05
**KRT78**	Keratin, type II cytoskeletal 78	2.95E+05	4	3.18E+05
**KRT85**	Keratin, type II cuticular Hb5	9.47E+05	9	7.45E+05
**KRT86**	Keratin, type II cuticular Hb6	2.04E+05	3	3.23E+05
**KRT9**	Keratin, type I cytoskeletal 9	2.15E+07	10	4.49E+07
**LAMA2**	Laminin subunit alpha-2	5.64E+04	1	1.11E+05
**LAMP2**	Lysosome-associated membrane glycoprotein 2		0	3.95E+05
**LBP**	Lipopolysaccharide-binding protein	2.07E+07	10	6.10E+05
**LDHA**	L-lactate dehydrogenase A chain	4.07E+05	3	1.06E+06
**LDHB**	L-lactate dehydrogenase B chain	3.86E+07	9	2.09E+06
**LGALS3BP**	Galectin-3-binding protein	6.56E+08	10	4.66E+08
**LRBA**	Lipopolysaccharide-responsive and beige-like	2.07E+05	3	
**LRP1**	Prolow-density lipoprotein receptor-related	2.65E+06	10	3.78E+06
**LTBP1**	Latent-transforming growth factor beta-bind	2.04E+05	9	2.13E+05
**LTF**	Lactotransferrin	5.49E+05	4	2.25E+06
**LYZ**	Lysozyme C	1.47E+05	5	
**M6PR**	Cation-dependent mannose-6-phosphate rec	2.15E+05	5	3.15E+05
**MARCO**	Macrophage receptor MARCO	1.88E+06	10	1.06E+06
**MASP1**	Mannan-binding lectin serine protease 1	2.90E+07	10	3.47E+07
**MASP2**	Mannan-binding lectin serine protease 2	3.96E+06	10	2.89E+06
**MATR3**	Matrin-3	1.85E+05	1	
**MBL2**	Mannose-binding protein C	9.73E+06	10	1.22E+07
**MMRN1**	Multimerin-1	6.59E+06	10	3.51E+06
**MSN**	Moesin	7.21E+06	10	6.05E+06
**MUC1**	Mucin-1	9.73E+04	10	1.40E+05
**MUC5B**	Mucin-5B	4.73E+05	3	1.74E+06
**MXRA5**	Matrix-remodeling-associated protein 5	2.45E+06	10	1.64E+06
**MYADM**	Myeloid-associated differentiation marker		0	1.78E+05
**MYH16**	Putative uncharacterized protein MYH16	2.51E+06	10	3.87E+06
**MYH9**	Myosin-9	4.29E+07	10	2.09E+08
**MYL12B**	Myosin regulatory light chain 12B	5.96E+05	10	2.00E+06
**MYL6**	Myosin light polypeptide 6	2.11E+06	10	5.29E+06
**NASP**	Nuclear autoantigenic sperm protein	1.74E+05	2	
**NCL**	Nucleolin	4.13E+05	1	2.98E+04
**NR0B1**	Nuclear receptor subfamily 0 group B memb	7.08E+07	10	5.79E+07
**OSBPL2**	Oxysterol-binding protein-related protein 2	5.46E+06	10	3.60E+06

**PATJ**	InaD-like protein		0	1.30E+06
**PCBP1**	Poly(rC)-binding protein 1	1.82E+05	1	
**PCYOX1**	Prenylcysteine oxidase 1	1.36E+07	10	1.21E+07
**PECAM1**	Platelet endothelial cell adhesion molecule	3.63E+06	10	6.77E+06
**PF4V1**	Platelet factor 4 variant	1.64E+06	9	1.77E+07
**PFN1**	Profilin-1	2.49E+06	10	6.68E+06
**PGK1**	Phosphoglycerate kinase 1		0	4.38E+04
**PHB**	Prohibitin	1.62E+05	1	
**PHLDB2**	Pleckstrin homology-like domain family B me	5.35E+07	10	5.30E+07
**PIGR**	Polymeric immunoglobulin receptor	2.59E+07	10	3.53E+07
**PKD2L1**	Polycystic kidney disease 2-like 1 protein	3.70E+07	5	2.11E+07
**PKM**	Pyruvate kinase PKM	3.14E+05	3	5.67E+05
**PLA2G7**	Platelet-activating factor acetylhydrolase	1.79E+06	5	1.36E+06
**PLEK**	Pleckstrin	5.64E+05	10	6.43E+05
**PNPLA8**	Calcium-independent phospholipase A2-gam	4.93E+06	10	2.60E+06
**PPARG**	Peroxisome proliferator-activated receptor gamma		0	1.79E+05
**PPIA**	Peptidyl-prolyl cis-trans isomerase A	3.23E+06	10	1.21E+07
**PRDX1**	Peroxiredoxin-1	4.53E+05	5	1.38E+05
**PRDX6**	Peroxiredoxin-6	1.40E+05	2	
**PSMA3**	Proteasome subunit alpha type-3	1.06E+05	6	2.35E+05
**PTBP1**	Polypyrimidine tract-binding protein 1	5.49E+05	1	
**PTGS1**	Prostaglandin G/H synthase 1	1.85E+05	2	7.65E+05
**PTPRJ**	Receptor-type tyrosine-protein phosphatase	3.95E+05	5	3.23E+06
**QSOX1**	Sulfhydryl oxidase 1	2.46E+06	10	3.45E+05
**RAB10**	Ras-related protein Rab-10	9.87E+05	10	2.99E+06
**RAB1B**	Ras-related protein Rab-1B	1.04E+06	10	3.31E+06
**RAB27B**	Ras-related protein Rab-27B		0	5.86E+05
**RAB33B**	Ras-related protein Rab-33B	7.05E+05	7	7.75E+05
**RAC2**	Ras-related C3 botulinum toxin substrate 2	8.55E+05	8	3.56E+06
**RAP1B**	Ras-related protein Rap-1b	1.26E+07	10	2.40E+07
**RELN**	Reelin	1.83E+06	8	3.29E+06
**RHOA**	Transforming protein RhoA	2.17E+05	3	8.41E+05
**RPL9**	60S ribosomal protein L9	1.02E+05	2	
**RPS15**	40S ribosomal protein S15	1.28E+05	1	
**RPS15A**	40S ribosomal protein S15a	9.51E+04	4	9.02E+04
**RPS23**	40S ribosomal protein S23	5.04E+05	1	
**RPSA**	40S ribosomal protein SA		0	5.97E+04
**RSU1**	Ras suppressor protein 1		0	6.22E+05
**RTN2**	Reticulon-2	3.21E+05	1	1.05E+06
**RTN3**	Reticulon-3	7.67E+05	10	2.24E+06
**RTN4**	Reticulon-4	6.62E+05	4	1.80E+06
**S100A8**	Protein S100-A8	1.19E+06	6	3.29E+05
**S100A9**	Protein S100-A9	2.25E+06	10	2.26E+06
**SACM1L**	Phosphatidylinositol-3-phosphatase SAC1	2.43E+05	2	7.84E+05
**SBSN**	Suprabasin	3.75E+05	5	5.77E+05
**SELPLG**	P-selectin glycoprotein ligand 1	1.15E+06	10	1.35E+06
**SF3A3**	Splicing factor 3A subunit 3	1.88E+05	1	

**SH3BGRL3**	SH3 domain-binding glutamic acid-rich-like p	3.10E+05	3	6.05E+06
**SIGLEC16**	Sialic acid-binding Ig-like lectin 16	5.70E+08	8	7.50E+05
**SLC2A1**	Solute carrier family 2, facilitated glucose tra	1.83E+06	10	1.29E+06
**SLC2A3**	Solute carrier family 2, facilitated glucose tra	2.22E+06	10	4.43E+06
**SLC4A1**	Band 3 anion transport protein	7.20E+06	10	2.92E+06
**SNX14**	Sorting nexin-14	1.96E+05	1	
**SPATA48**	Spermatogenesis-associated protein 48	9.21E+05	10	1.07E+06
**SPN**	Leukosialin	1.10E+06	5	9.95E+05
**SPTA1**	Spectrin alpha chain, erythrocytic 1	1.24E+06	5	1.74E+06
**SPTB**	Spectrin beta chain, erythrocytic	1.36E+06	10	2.18E+06
**SPTBN1**	Spectrin beta chain, non-erythrocytic 1	5.90E+05	5	
**SPTLC3**	Serine palmitoyltransferase 3	1.20E+09	10	1.20E+09
**SRGN**	Serglycin	1.41E+06	10	1.41E+06
**SRI**	Sorcin	1.23E+06	9	1.89E+06
**SSC5D**	Soluble scavenger receptor cysteine-rich dor	5.48E+07	10	7.77E+07
**STEAP3**	Metalloreductase STEAP3	9.47E+05	5	8.48E+05
**STOM**	Stomatin	2.72E+07	10	4.01E+07
**STX11**	Syntaxin-11	4.17E+05	7	7.82E+05
**SVEP1**	Sushi, von Willebrand factor type A, EGF and	1.56E+06	9	1.97E+06
**SYNCRIP**	Heterogeneous nuclear ribonucleoprotein Q	1.49E+05	3	
**TAGLN2**	Transgelin-2	5.99E+05	6	1.02E+06
**TCP1**	T-complex protein 1 subunit alpha	2.88E+05	2	
**TFRC**	Transferrin receptor protein 1	6.00E+06	10	8.76E+06
**TGFBI**	Transforming growth factor-beta-induced pr	9.37E+05	8	8.58E+05
**TGM3**	Protein-glutamine gamma-glutamyltransfera	7.93E+05	8	3.50E+05
**THBS1**	Thrombospondin-1	5.98E+07	10	3.10E+07
**THBS4**	Thrombospondin-4	8.22E+05	5	3.30E+05
**TKT**	Transketolase	1.03E+05	3	
**TLN1**	Talin-1	6.24E+07	10	8.31E+07
**TM4SF1**	Transmembrane 4 L6 family member 1	7.16E+05	6	3.18E+05
**TMEM109**	Transmembrane protein 109	1.40E+05	1	8.31E+05
**TMEM198**	Transmembrane protein 198	1.03E+06	10	1.54E+06
**TMX2**	Thioredoxin-related transmembrane protein	2.73E+06	8	1.66E+06
**TNC**	Tenascin	3.29E+07	10	3.77E+07
**TNKS1BP1**	182 kDa tankyrase-1-binding protein		0	6.96E+05
**TPM4**	Tropomyosin alpha-4 chain	4.14E+05	4	1.31E+06
**TSPAN14**	Tetraspanin-14		0	1.19E+04
**TTLL12**	Tubulin--tyrosine ligase-like protein 12	4.38E+06	4	3.90E+05
**TUBA1C**	Tubulin alpha-1C chain	2.29E+06	10	9.60E+06
**TUBA4A**	Tubulin alpha-4A chain	5.25E+05	6	2.81E+06
**TUBB**	Tubulin beta chain	1.60E+06	5	1.53E+06
**TUBB1**	Tubulin beta-1 chain	9.48E+05	9	5.44E+06
**UBA52**	Ubiquitin-60S ribosomal protein L40	1.91E+06	10	1.06E+06
**UBE2L3**	Ubiquitin-conjugating enzyme E2 L3	2.07E+05	2	1.02E+04
**VCAN**	Versican core protein	2.15E+06	6	1.76E+06
**VCL**	Vinculin	7.54E+06	10	1.89E+07
**VIM**	Vimentin	4.29E+05	2	

**WBP11**	WW domain-binding protein 11	3.50E+06	9	7.84E+06
**YWHAB**	14-3-3 protein beta/alpha	7.73E+05	9	1.79E+06
**YWHAE**	14-3-3 protein epsilon	1.05E+06	10	4.17E+06
**YWHAG**	14-3-3 protein gamma	5.97E+06	10	5.74E+06
**YWHAH**	14-3-3 protein eta	3.21E+06	10	1.14E+07
**YWHAZ**	14-3-3 protein zeta/delta	2.81E+07	10	1.52E+08
**ZNF275**	Zinc finger protein 275	3.54E+05	4	6.63E+05

MP number of observations	analysis type	p-value (U-test)	FDR (U-test)	Effect size (RBCC)	p-value (Fisher test)	FDR (Fisher test)	Effect size (Crammer V)
8	C	0.9090	0.9535	-0.040	NA	NA	NA
10	C	0.0524	0.1553	-0.520	NA	NA	NA
3	D	NA	NA	NA	1.0000	1.0000	0.000
10	C	0.0355	0.1379	-0.560	NA	NA	NA
10	C	0.6842	0.8231	-0.120	NA	NA	NA
4	D	NA	NA	NA	0.6285	1.0000	0.218
9	C	0.0187	0.0933	-0.630	NA	NA	NA
10	C	0.5787	0.7349	0.160	NA	NA	NA
10	C	0.0068	0.0513	-0.700	NA	NA	NA
9	C	0.4055	0.6007	0.230	NA	NA	NA
9	C	0.8534	0.9164	0.060	NA	NA	NA
0	D	NA	NA	NA	0.0867	1.0000	0.500
8	C	0.0071	0.0513	-0.700	NA	NA	NA
4	D	NA	NA	NA	1.0000	1.0000	0.101
10	C	0.0443	0.1464	-0.540	NA	NA	NA
8	C	0.2088	0.3713	-0.340	NA	NA	NA
5	D	NA	NA	NA	0.3498	1.0000	0.315
4	D	NA	NA	NA	1.0000	1.0000	0.101
10	C	0.0147	0.0870	-0.640	NA	NA	NA
1	D	NA	NA	NA	0.5820	1.0000	0.250
3	D	NA	NA	NA	0.5820	1.0000	0.250
5	D	NA	NA	NA	0.3498	1.0000	0.315
10	C	0.0524	0.1553	-0.520	NA	NA	NA
8	C	0.8501	0.9164	0.060	NA	NA	NA
10	C	0.4359	0.6283	0.220	NA	NA	NA
10	C	0.1431	0.3013	-0.400	NA	NA	NA
2	D	NA	NA	NA	1.0000	1.0000	0.140
10	C	0.0007	0.0129	-0.840	NA	NA	NA
5	D	NA	NA	NA	0.6499	1.0000	0.204
5	D	NA	NA	NA	1.0000	1.0000	0.101
1	D	NA	NA	NA	1.0000	1.0000	0.229
0	D	NA	NA	NA	0.4737	1.0000	0.333
2	D	NA	NA	NA	0.6285	1.0000	0.218
4	D	NA	NA	NA	0.0867	1.0000	0.500
10	C	0.0089	0.0600	-0.680	NA	NA	NA
6	D	NA	NA	NA	0.0108	0.6111	0.655
10	C	0.0892	0.2302	-0.460	NA	NA	NA
9	C	0.0030	0.0320	-0.770	NA	NA	NA
10	C	0.2475	0.4124	-0.320	NA	NA	NA
10	C	0.5288	0.6936	-0.180	NA	NA	NA

10	C	0.0185	0.0933	-0.620	NA	NA	NA
9	C	0.7333	0.8698	0.100	NA	NA	NA
10	C	0.0185	0.0933	-0.620	NA	NA	NA
10	C	0.6842	0.8231	0.120	NA	NA	NA
9	C	0.1431	0.3013	0.400	NA	NA	NA
9	C	0.0090	0.0600	-0.700	NA	NA	NA
7	C	0.2835	0.4491	-0.290	NA	NA	NA
8	D	NA	NA	NA	0.0055	0.6111	0.704
7	D	NA	NA	NA	0.0698	1.0000	0.503
6	D	NA	NA	NA	1.0000	1.0000	0.101
7	D	NA	NA	NA	0.0031	0.6111	0.734
4	D	NA	NA	NA	0.0867	1.0000	0.500
10	C	0.5787	0.7349	-0.160	NA	NA	NA
10	C	0.1230	0.2733	-0.420	NA	NA	NA
3	D	NA	NA	NA	0.0698	1.0000	0.503
8	C	0.1613	0.3229	0.380	NA	NA	NA
10	C	0.0115	0.0707	0.660	NA	NA	NA
3	D	NA	NA	NA	1.0000	1.0000	0.105
7	C	0.2116	0.3721	0.340	NA	NA	NA
10	C	0.0892	0.2302	0.460	NA	NA	NA
2	D	NA	NA	NA	1.0000	1.0000	0.140
5	D	NA	NA	NA	0.0325	0.9167	0.577
10	C	0.5189	0.6936	-0.180	NA	NA	NA
6	C	0.6675	0.8153	0.120	NA	NA	NA
1	D	NA	NA	NA	1.0000	1.0000	0.000
6	C	0.9078	0.9535	0.040	NA	NA	NA
10	C	0.5189	0.6936	-0.180	NA	NA	NA
5	D	NA	NA	NA	1.0000	1.0000	0.000
2	D	NA	NA	NA	0.3498	1.0000	0.315
3	D	NA	NA	NA	1.0000	1.0000	0.000
8	C	0.2971	0.4660	-0.280	NA	NA	NA
3	D	NA	NA	NA	1.0000	1.0000	0.105
0	D	NA	NA	NA	1.0000	1.0000	0.229
2	D	NA	NA	NA	0.6285	1.0000	0.218
3	C	0.0509	0.1553	0.510	NA	NA	NA
1	D	NA	NA	NA	1.0000	1.0000	0.000
3	D	NA	NA	NA	1.0000	1.0000	0.105
9	C	0.7910	0.9096	-0.080	NA	NA	NA
5	C	0.2742	0.4478	0.290	NA	NA	NA
9	C	0.0342	0.1379	-0.570	NA	NA	NA
3	D	NA	NA	NA	0.6499	1.0000	0.204
10	C	0.3930	0.5877	-0.240	NA	NA	NA
10	C	0.5288	0.6936	0.180	NA	NA	NA
10	C	0.4813	0.6650	0.200	NA	NA	NA
3	D	NA	NA	NA	1.0000	1.0000	0.116
10	C	0.0039	0.0366	-0.740	NA	NA	NA
3	D	NA	NA	NA	1.0000	1.0000	0.000

3	D	NA	NA	NA	0.2105	1.0000	0.420
3	D	NA	NA	NA	1.0000	1.0000	0.000
10	C	0.1230	0.2733	0.420	NA	NA	NA
10	C	0.0029	0.0320	-0.760	NA	NA	NA
9	C	0.2799	0.4478	-0.300	NA	NA	NA
9	C	0.0962	0.2404	-0.450	NA	NA	NA
9	C	0.3579	0.5403	-0.250	NA	NA	NA
3	D	NA	NA	NA	0.6499	1.0000	0.204
10	C	0.0115	0.0707	-0.660	NA	NA	NA
10	C	0.0015	0.0201	-0.800	NA	NA	NA
10	C	0.0005	0.0097	-0.860	NA	NA	NA
0	D	NA	NA	NA	0.4737	1.0000	0.333
1	D	NA	NA	NA	1.0000	1.0000	0.000
1	D	NA	NA	NA	1.0000	1.0000	0.140
10	C	0.1903	0.3421	0.360	NA	NA	NA
10	C	0.1040	0.2548	0.440	NA	NA	NA
3	D	NA	NA	NA	0.6499	1.0000	0.204
8	C	0.0448	0.1464	0.540	NA	NA	NA
7	C	0.5846	0.7365	0.150	NA	NA	NA
0	D	NA	NA	NA	0.4737	1.0000	0.333
10	C	0.1655	0.3229	-0.380	NA	NA	NA
10	C	0.1903	0.3421	-0.360	NA	NA	NA
1	D	NA	NA	NA	1.0000	1.0000	0.229
0	D	NA	NA	NA	0.4737	1.0000	0.333
0	D	NA	NA	NA	1.0000	1.0000	0.229
0	D	NA	NA	NA	1.0000	1.0000	0.229
1	D	NA	NA	NA	1.0000	1.0000	0.140
0	D	NA	NA	NA	0.4737	1.0000	0.333
10	C	0.0355	0.1379	-0.560	NA	NA	NA
9	C	1.0000	1.0000	0.000	NA	NA	NA
4	D	NA	NA	NA	1.0000	1.0000	0.105
10	C	0.8534	0.9164	-0.060	NA	NA	NA
2	D	NA	NA	NA	1.0000	1.0000	0.140
2	D	NA	NA	NA	1.0000	1.0000	0.000
5	D	NA	NA	NA	1.0000	1.0000	0.101
8	C	0.8499	0.9164	-0.060	NA	NA	NA
10	C	0.8534	0.9164	-0.060	NA	NA	NA
2	D	NA	NA	NA	0.3498	1.0000	0.315
6	C	0.9689	0.9891	-0.020	NA	NA	NA
10	C	0.1431	0.3013	-0.400	NA	NA	NA
10	C	0.0355	0.1379	-0.560	NA	NA	NA
10	C	0.3150	0.4893	-0.280	NA	NA	NA
10	C	0.0753	0.2084	-0.480	NA	NA	NA
0	D	NA	NA	NA	1.0000	1.0000	0.229
1	D	NA	NA	NA	1.0000	1.0000	0.000
10	C	0.4359	0.6283	0.220	NA	NA	NA
1	D	NA	NA	NA	1.0000	1.0000	0.140

10	C	0.1655	0.3229	-0.380	NA	NA	NA
10	C	0.0433	0.1464	-0.540	NA	NA	NA
10	C	0.2799	0.4478	0.300	NA	NA	NA
0	D	NA	NA	NA	1.0000	1.0000	0.229
5	D	NA	NA	NA	1.0000	1.0000	0.000
5	D	NA	NA	NA	0.3498	1.0000	0.315
10	C	0.0288	0.1280	-0.580	NA	NA	NA
6	C	0.0866	0.2302	0.460	NA	NA	NA
6	D	NA	NA	NA	0.1698	1.0000	0.408
10	C	0.1230	0.2733	0.420	NA	NA	NA
7	C	0.1291	0.2830	0.410	NA	NA	NA
2	D	NA	NA	NA	0.6285	1.0000	0.218
3	D	NA	NA	NA	1.0000	1.0000	0.105
4	C	0.0185	0.0933	0.620	NA	NA	NA
3	D	NA	NA	NA	1.0000	1.0000	0.000
10	C	0.1655	0.3229	-0.380	NA	NA	NA
3	D	NA	NA	NA	0.5820	1.0000	0.250
2	D	NA	NA	NA	0.4737	1.0000	0.333
10	C	0.0001	0.0040	0.940	NA	NA	NA
4	D	NA	NA	NA	1.0000	1.0000	0.105
10	C	1.0000	1.0000	0.000	NA	NA	NA
10	C	0.3527	0.5374	0.260	NA	NA	NA
0	D	NA	NA	NA	0.2105	1.0000	0.420
10	C	0.0232	0.1062	-0.600	NA	NA	NA
8	C	0.5702	0.7349	0.160	NA	NA	NA
3	D	NA	NA	NA	1.0000	1.0000	0.105
0	D	NA	NA	NA	0.0325	0.9167	0.577
8	C	0.0407	0.1464	-0.540	NA	NA	NA
9	C	0.0039	0.0366	0.740	NA	NA	NA
10	C	0.7959	0.9096	-0.080	NA	NA	NA
10	C	0.8534	0.9164	0.060	NA	NA	NA
0	D	NA	NA	NA	1.0000	1.0000	0.229
10	C	0.7394	0.8698	-0.100	NA	NA	NA
10	C	0.1230	0.2733	0.420	NA	NA	NA
10	C	0.6305	0.7760	-0.140	NA	NA	NA
7	C	0.6227	0.7760	0.140	NA	NA	NA
1	D	NA	NA	NA	0.5820	1.0000	0.250
10	C	0.0015	0.0201	0.800	NA	NA	NA
3	D	NA	NA	NA	0.2105	1.0000	0.420
10	C	0.4813	0.6650	-0.200	NA	NA	NA
10	C	0.0001	0.0040	-0.940	NA	NA	NA
10	C	0.0892	0.2302	-0.460	NA	NA	NA
9	C	0.1903	0.3421	-0.360	NA	NA	NA
0	D	NA	NA	NA	0.4737	1.0000	0.333
2	D	NA	NA	NA	1.0000	1.0000	0.140
10	C	0.7959	0.9096	0.080	NA	NA	NA
7	C	0.0209	0.1015	0.620	NA	NA	NA

4	D	NA	NA	NA	0.0867	1.0000	0.500
0	D	NA	NA	NA	1.0000	1.0000	0.229
10	C	1.0000	1.0000	0.000	NA	NA	NA
10	C	0.0232	0.1062	-0.600	NA	NA	NA
10	C	0.0001	0.0047	-0.920	NA	NA	NA
10	C	0.0185	0.0933	-0.620	NA	NA	NA
1	D	NA	NA	NA	1.0000	1.0000	0.229
0	D	NA	NA	NA	1.0000	1.0000	0.229
10	C	0.6305	0.7760	0.140	NA	NA	NA
10	C	0.1903	0.3421	-0.360	NA	NA	NA
5	D	NA	NA	NA	1.0000	1.0000	0.000
4	D	NA	NA	NA	1.0000	1.0000	0.105
7	C	0.9377	0.9743	0.030	NA	NA	NA
10	C	0.8534	0.9164	-0.060	NA	NA	NA
8	C	0.0756	0.2084	0.480	NA	NA	NA
1	D	NA	NA	NA	1.0000	1.0000	0.229
10	C	0.0000	0.0017	-1.000	NA	NA	NA
3	D	NA	NA	NA	0.6499	1.0000	0.204
0	D	NA	NA	NA	0.4737	1.0000	0.333
3	D	NA	NA	NA	0.3698	1.0000	0.302
0	D	NA	NA	NA	1.0000	1.0000	0.229
6	D	NA	NA	NA	0.1698	1.0000	0.408
3	D	NA	NA	NA	0.6499	1.0000	0.204
4	C	0.0002	0.0047	0.980	NA	NA	NA
10	C	0.0433	0.1464	-0.540	NA	NA	NA
10	C	0.2799	0.4478	-0.300	NA	NA	NA
1	D	NA	NA	NA	1.0000	1.0000	0.229
6	C	0.9692	0.9891	0.020	NA	NA	NA
10	C	0.0046	0.0385	-0.760	NA	NA	NA
10	C	0.1903	0.3421	-0.360	NA	NA	NA
10	C	0.1211	0.2733	-0.420	NA	NA	NA
6	D	NA	NA	NA	0.3698	1.0000	0.302
0	D	NA	NA	NA	0.4737	1.0000	0.333
0	D	NA	NA	NA	1.0000	1.0000	0.229
4	D	NA	NA	NA	1.0000	1.0000	0.000
0	D	NA	NA	NA	1.0000	1.0000	0.229
1	D	NA	NA	NA	1.0000	1.0000	0.229
3	D	NA	NA	NA	0.2105	1.0000	0.420
1	D	NA	NA	NA	1.0000	1.0000	0.000
8	C	0.5706	0.7349	-0.160	NA	NA	NA
10	C	0.0019	0.0236	-0.820	NA	NA	NA
3	D	NA	NA	NA	0.3698	1.0000	0.302
9	C	0.8534	0.9164	0.060	NA	NA	NA
4	D	NA	NA	NA	0.6285	1.0000	0.218
5	D	NA	NA	NA	1.0000	1.0000	0.000
9	C	0.5288	0.6936	-0.180	NA	NA	NA
0	D	NA	NA	NA	1.0000	1.0000	0.229

1	D	NA	NA	NA	0.5820	1.0000	0.250
10	C	0.9097	0.9535	0.040	NA	NA	NA
10	C	0.4813	0.6650	0.200	NA	NA	NA
10	C	0.0753	0.2084	-0.480	NA	NA	NA
10	C	0.1230	0.2733	0.420	NA	NA	NA
0	D	NA	NA	NA	1.0000	1.0000	0.229
9	C	0.7394	0.8698	0.100	NA	NA	NA
5	D	NA	NA	NA	1.0000	1.0000	0.000
9	C	0.1455	0.3024	-0.390	NA	NA	NA
10	C	0.2475	0.4124	-0.320	NA	NA	NA
0	D	NA	NA	NA	0.0325	0.9167	0.577
10	C	0.9118	0.9535	-0.040	NA	NA	NA
10	C	0.7959	0.9096	0.080	NA	NA	NA
9	C	0.0962	0.2404	-0.450	NA	NA	NA
10	C	0.0524	0.1553	-0.520	NA	NA	NA
7	C	0.4821	0.6650	-0.190	NA	NA	NA
10	C	0.2176	0.3784	-0.340	NA	NA	NA
4	D	NA	NA	NA	0.3698	1.0000	0.302
9	C	0.2263	0.3893	-0.330	NA	NA	NA
0	D	NA	NA	NA	0.2105	1.0000	0.420
9	C	0.0362	0.1379	-0.560	NA	NA	NA
0	D	NA	NA	NA	0.4737	1.0000	0.333
10	C	0.5288	0.6936	-0.180	NA	NA	NA
7	C	0.4238	0.6222	0.220	NA	NA	NA
6	C	0.1800	0.3421	0.360	NA	NA	NA
10	C	0.1051	0.2548	0.440	NA	NA	NA
3	D	NA	NA	NA	0.6499	1.0000	0.204
0	D	NA	NA	NA	0.2105	1.0000	0.420
10	C	0.3527	0.5374	-0.260	NA	NA	NA
4	D	NA	NA	NA	0.6563	1.0000	0.200
2	D	NA	NA	NA	1.0000	1.0000	0.140
9	C	0.9705	0.9891	-0.020	NA	NA	NA
7	C	0.1588	0.3229	0.380	NA	NA	NA
10	C	0.2475	0.4124	-0.320	NA	NA	NA
1	D	NA	NA	NA	1.0000	1.0000	0.229
8	C	0.0385	0.1432	-0.540	NA	NA	NA
3	D	NA	NA	NA	0.2105	1.0000	0.420
7	D	NA	NA	NA	0.3698	1.0000	0.302
10	C	0.0355	0.1379	-0.560	NA	NA	NA
10	C	0.0044	0.0385	-0.760	NA	NA	NA
3	D	NA	NA	NA	0.6499	1.0000	0.204
10	C	0.0068	0.0513	-0.700	NA	NA	NA
10	C	0.1903	0.3421	0.360	NA	NA	NA
5	D	NA	NA	NA	0.3498	1.0000	0.315
7	C	0.4631	0.6616	-0.200	NA	NA	NA
10	C	0.0524	0.1553	-0.520	NA	NA	NA
0	D	NA	NA	NA	0.4737	1.0000	0.333

10	C	0.0433	0.1464	-0.540	NA	NA	NA
9	C	0.0587	0.1707	-0.510	NA	NA	NA
10	C	0.0002	0.0047	-0.900	NA	NA	NA
10	C	0.8534	0.9164	-0.060	NA	NA	NA
10	C	0.0015	0.0201	-0.800	NA	NA	NA
10	C	0.0002	0.0047	-0.900	NA	NA	NA
7	D	NA	NA	NA	0.3698	1.0000	0.302

Size of effect	MP vs HD	ProteinID	Entrez
no effect	UP	AACS	65985
large	UP	ABI3BP	25890
no effect	DOWN	ACAN	176
large	UP	ACTB	60
small	UP	ACTG2	72
small	UP	ACTN1	87
large	UP	ADAM10	102
small	DOWN	ADIPOQ	9370
very large	UP	ALDOA	226
small	DOWN	ANK1	286
no effect	DOWN	ANPEP	290
large	DOWN	ANTXR2	118429
very large	UP	ANXA11	311
small	DOWN	ANXA2	302
large	UP	ANXA5	308
medium	UP	ANXA7	310
medium	UP	ARL6IP5	10550
small	DOWN	ARPC5	10092
large	UP	ATP2A3	489
small	DOWN	ATP5F1B	506
small	UP	B2M	567
medium	UP	BCAP31	10134
large	UP	C16orf54	283897
no effect	DOWN	MENT	54964
small	DOWN	CAMP	820
medium	UP	CANX	821
small	UP	CASP14	23581
very large	UP	CAVIN2	8436
small	UP	CCL5	6352
small	UP	CCT2	10576
small	UP	CCT4	10575
medium	DOWN	CCT6A	908
small	DOWN	CCT7	10574
large	UP	CCT8	10694
large	UP	CD151	977
large	UP	CD226	10666
medium	UP	CD36	948
very large	UP	CD47	961
medium	UP	CD53	963
small	UP	CD59	966

large	UP	CD9	928
small	DOWN	CD99	4267
large	UP	CDC42	998
small	DOWN	CDCA2	157313
medium	DOWN	CELSR2	1952
very large	UP	CFL1	1072
small	UP	CLDN5	7122
very large	UP	CLIC1	1192
large	UP	CLTA	1211
small	UP	CLTCL1	8218
very large	UP	CMTM5	116173
large	UP	CMTM6	54918
small	UP	COL1A1	1277
medium	UP	COL1A2	1278
large	DOWN	COL2A1	1280
medium	DOWN	COL6A1	1291
large	DOWN	COL6A3	1293
small	DOWN	COL7A1	1294
medium	DOWN	COLEC11	78989
medium	DOWN	CPN1	1369
small	UP	CSDE1	7812
large	UP	CTSD	1509
small	UP	CYB5R3	1727
small	DOWN	CYBB	1536
no effect	DOWN	DBH	1621
no effect	DOWN	DCD	117159
small	UP	DCDC1	341019
no effect	DOWN	DDX60L	91351
medium	DOWN	DHX9	1660
no effect	DOWN	DPP6	1804
small	UP	DSC1	1823
small	DOWN	DSG1	1828
small	DOWN	DSP	1832
small	DOWN	EEF1A1	1915
large	DOWN	EFEMP1	2202
no effect	DOWN	EIF5A	1984
small	DOWN	ENDOD1	23052
no effect	UP	ENO1	2023
small	DOWN	EPB42	2038
large	UP	F11R	50848
small	DOWN	FBN1	2200
small	UP	FCER1G	2207
small	DOWN	FCN1	2219
small	DOWN	FERMT3	83706
small	UP	FLG2	388698
very large	UP	FLNA	2316
no effect	DOWN	FLOT1	10211

medium	UP	FLOT2	2319
no effect	DOWN	FSCN1	6624
medium	DOWN	GALE	2582
very large	UP	GAPDH	2597
medium	UP	GNAI2	2771
medium	UP	GNAQ	2776
small	UP	GNB4	59345
small	DOWN	GNG5	2787
large	UP	GP1BA	2811
very large	UP	GP1BB	2812
very large	UP	GP9	2815
medium	DOWN	GSDMA	284110
no effect	DOWN	GSR	2936
small	DOWN	GYPA	2993
medium	DOWN	H2AC20	8338
medium	DOWN	H2BC21	8349
small	DOWN	H3-3A	3020
large	DOWN	H4C1	8359
small	DOWN	HGFAC	3083
medium	DOWN	HINT1	3094
medium	UP	HLA-A	3105
medium	UP	HLA-B	3106
small	UP	HMBS	3145
medium	DOWN	HNRNPA1I	144983
small	DOWN	HNRNPA2I	3181
small	DOWN	HNRNPC	3183
small	DOWN	HNRNPH3	3189
medium	DOWN	HNRNPU	3192
large	UP	HRNR	388697
no effect	UP	HSP90AA1	3320
small	UP	HSPA1B	3304
no effect	UP	HSPA8	3312
small	UP	HSPA9	3313
no effect	DOWN	HSPD1	3329
small	UP	ICAM2	3384
no effect	UP	IFITM2	10581
no effect	UP	ILK	3611
medium	DOWN	INPP5D	3635
no effect	UP	ITGA2	3673
medium	UP	ITGA2B	3674
large	UP	ITGA6	3655
small	UP	ITGB1	3688
medium	UP	ITGB3	3690
small	DOWN	JUP	3728
no effect	DOWN	KANSL3	55683
small	DOWN	KIAA2026	158358
small	DOWN	KPRP	448834

medium	UP	KRT1	3848
large	UP	KRT10	3858
medium	DOWN	KRT14	3861
small	DOWN	KRT15	3866
no effect	DOWN	KRT16	3868
medium	UP	KRT17	3872
large	UP	KRT2	3849
medium	DOWN	KRT24	192666
medium	UP	KRT32	3882
medium	DOWN	KRT5	3852
medium	DOWN	KRT6A	3853
small	DOWN	KRT6B	3854
small	DOWN	KRT78	196374
large	DOWN	KRT85	3891
no effect	DOWN	KRT86	3892
medium	UP	KRT9	3857
small	UP	LAMA2	3908
medium	UP	LAMP2	3920
very large	DOWN	LBP	3929
small	UP	LDHA	3939
no effect	UP	LDHB	3945
small	DOWN	LGALS3BP	3959
medium	DOWN	LRBA	987
large	UP	LRP1	4035
small	DOWN	LTBP1	4052
small	DOWN	LTF	4057
large	DOWN	LYZ	4069
large	UP	M6PR	4074
very large	DOWN	MARCO	8685
no effect	UP	MASP1	5648
no effect	DOWN	MASP2	10747
small	DOWN	MATR3	9782
small	UP	MBL2	4153
medium	DOWN	MMRN1	22915
small	UP	MSN	4478
small	DOWN	MUC1	4582
small	DOWN	MUC5B	727897
very large	DOWN	MXRA5	25878
medium	UP	MYADM	91663
small	UP	MYH16	84176
very large	UP	MYH9	4627
medium	UP	MYL12B	103910
medium	UP	MYL6	4637
medium	DOWN	NASP	4678
small	UP	NCL	4691
no effect	DOWN	NR0B1	190
large	DOWN	OSBPL2	9885

large	UP	PATJ	10207
small	DOWN	PCBP1	5093
no effect	UP	PCYOX1	51449
large	UP	PECAM1	5175
very large	UP	PF4V1	5197
large	UP	PFN1	5216
small	UP	PGK1	5230
small	DOWN	PHB	5245
small	DOWN	PHLDB2	90102
medium	UP	PIGR	5284
no effect	DOWN	PKD2L1	9033
small	UP	PKM	5315
no effect	DOWN	PLA2G7	7941
no effect	UP	PLEK	5341
medium	DOWN	PNPLA8	50640
small	UP	PPARG	5468
very large	UP	PPIA	5478
small	DOWN	PRDX1	5052
medium	DOWN	PRDX6	9588
medium	DOWN	PSMA3	5684
small	DOWN	PTBP1	5725
medium	UP	PTGS1	5742
small	DOWN	PTPRJ	5795
very large	DOWN	QSOX1	5768
large	UP	RAB10	10890
medium	UP	RAB1B	81876
small	UP	RAB27B	5874
no effect	DOWN	RAB33B	83452
very large	UP	RAC2	5880
medium	UP	RAP1B	5908
medium	UP	RELN	5649
medium	UP	RHOA	387
medium	DOWN	RPL9	6133
small	DOWN	RPS15	6209
no effect	DOWN	RPS15A	6210
small	DOWN	RPS23	6228
small	UP	RPSA	3921
medium	UP	RSU1	6251
no effect	DOWN	RTN2	6253
small	UP	RTN3	10313
very large	UP	RTN4	57142
medium	DOWN	S100A8	6279
no effect	DOWN	S100A9	6280
small	UP	SACM1L	22908
no effect	DOWN	SBSN	374897
small	UP	SELPLG	6404
small	DOWN	SF3A3	10946

small	DOWN	SH3BGRL3	83442
no effect	DOWN	SIGLEC16	400709
small	DOWN	SLC2A1	6513
medium	UP	SLC2A3	6515
medium	DOWN	SLC4A1	6521
small	DOWN	SNX14	57231
small	DOWN	SPATA48	1E+08
no effect	DOWN	SPN	6693
medium	UP	SPTA1	6708
medium	UP	SPTB	6710
large	DOWN	SPTBN1	6711
no effect	UP	SPTLC3	55304
no effect	DOWN	SRGN	5552
medium	UP	SRI	6717
large	UP	SSC5D	284297
small	UP	STEAP3	55240
medium	UP	STOM	2040
medium	DOWN	STX11	8676
medium	UP	SVEP1	79987
medium	DOWN	SYNCRIP	10492
large	UP	TAGLN2	8407
medium	DOWN	TCP1	6950
small	UP	TFRC	7037
small	DOWN	TGFBI	7045
medium	DOWN	TGM3	7053
medium	DOWN	THBS1	7057
small	DOWN	THBS4	7060
medium	DOWN	TKT	7086
small	UP	TLN1	7094
small	DOWN	TM4SF1	4071
small	UP	TMEM109	79073
no effect	UP	TMEM198	130612
medium	DOWN	TMX2	51075
medium	UP	TNC	3371
small	UP	TNKS1BP1	85456
large	UP	TPM4	7171
medium	UP	TSPAN14	81619
medium	UP	TTLL12	23170
large	UP	TUBA1C	84790
very large	UP	TUBA4A	7277
small	DOWN	TUBB	203068
very large	UP	TUBB1	81027
medium	DOWN	UBA52	7311
medium	UP	UBE2L3	7332
small	UP	VCAN	1462
large	UP	VCL	7414
medium	DOWN	VIM	7431

large	UP	WBP11	51729
large	UP	YWHAB	7529
very large	UP	YWHAE	7531
no effect	UP	YWHAG	7532
very large	UP	YWHAH	7533
very large	UP	YWHAZ	7534
medium	UP	ZNF275	10838

Table 11. Antibodies used for Western blots

Target Pathway	Proteins	Producer	Catalogue #
sEV	CD9	Santa Cruz	SC-13118
	TSG101	Invitrogen	MA1-23296
	Alix	Invitrogen	MA5-32773
	Grp94	Cell Signaling	2104
	14-3-3	Santa Cruz	SC-1657
	TGF-beta	Abcam	AB31013
	CD3	Santa Cruz	SC-1239
	CD41 (ITGB2A	Proteintech	24552-1-AP
MAPK SIGNALING	P-38	Cell Signaling	4511
	P-ERK	Cell Signaling	4370
	P-JNK	Cell Signaling	4668
	P-RAF1	Cell Signaling	9427
RHO GTPASE PATHWAY	Rac 1/2/3	Santa Cruz	SC-514583
	PAK4	Santa Cruz	SC-393367
	Rock1	Santa Cruz	SC-17794
	ITGB1	Santa Cruz	SC-374429
LOADING CONTROLS	Beta- actin	Santa Cruz	SC-47778
